# Chiral Triphenylacetic Acid Esters: Residual Stereoisomerism and Solid-State Variability of Molecular Architectures

**DOI:** 10.1021/acs.joc.1c00279

**Published:** 2021-04-28

**Authors:** Natalia Prusinowska, Agnieszka Czapik, Marcin Kwit

**Affiliations:** † Faculty of Chemistry, Adam Mickiewicz University, Uniwersytetu Poznańskiego 8, 61 614 Poznań, Poland; ‡ Centre for Advanced Technologies, Adam Mickiewicz University, Uniwersytetu Poznańskiego 10, 61 614 Poznań, Poland

## Abstract

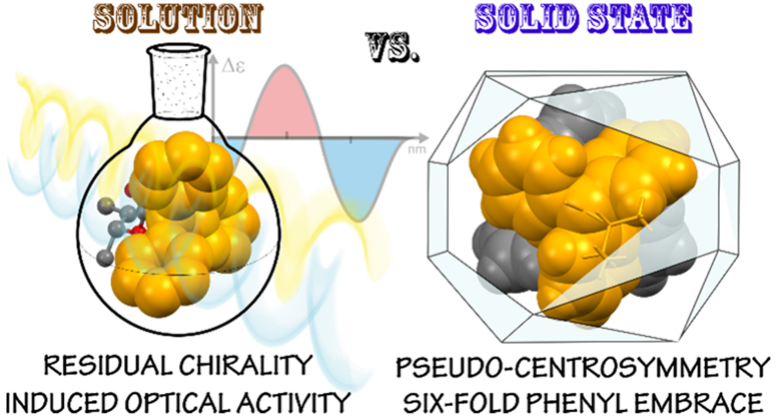

We have proven the usability and versatility of chiral triphenylacetic acid esters, compounds of high structural diversity, as chirality-sensing stereodynamic probes and as molecular tectons in crystal engineering. The low energy barrier to stereoisomer interconversion has been exploited to sense the chirality of an alkyl substituent in the esters. The structural information are cascaded from the permanently chiral alcohol (inducer) to the stereodynamic chromophoric probe through cooperative interactions. The ECD spectra of triphenylacetic acid esters are highly sensitive to very small structural differences in the inducer core. The tendencies to maximize the C–H···O hydrogen bonds, van der Waals interactions, and London dispersion forces determine the way of packing molecules in the crystal lattice. The phenyl embraces of trityl groups allowed, to some extent, the control of molecular organization in the crystal. However, the spectrum of possible molecular arrangements is very broad and depends on the type of substituent, the optical purity of the sample, and the presence of a second trityl group in the proximity. Racemates crystallize as the solid solution of enantiomers, where the trityl group acts as a protecting group for the stereogenic center. Therefore, the absolute configuration of the inducer is irrelevant to the packing mode of molecules in the crystal.

## Introduction

The dynamic stereochemistry and residual stereoisomerism of molecular propellers were the subjects of intense studies initiated by Mislow in the early 1970s.^1−3^ Then, after a nearly 20 year period of freezing activity in the field, there was renewed interest in molecular propellers associated with their increasing number of applications. One of the simplest entities showing propensity to residual stereoisomerism, namely, the triphenylmethyl group (CPh_3_, Tr, trityl), is currently used in organic synthesis as protecting devices, catalysts,^4,5^ construction of molecular machines,^6^ medical chemistry,^7^ and bioimaging.^8^

**Figure 1:**
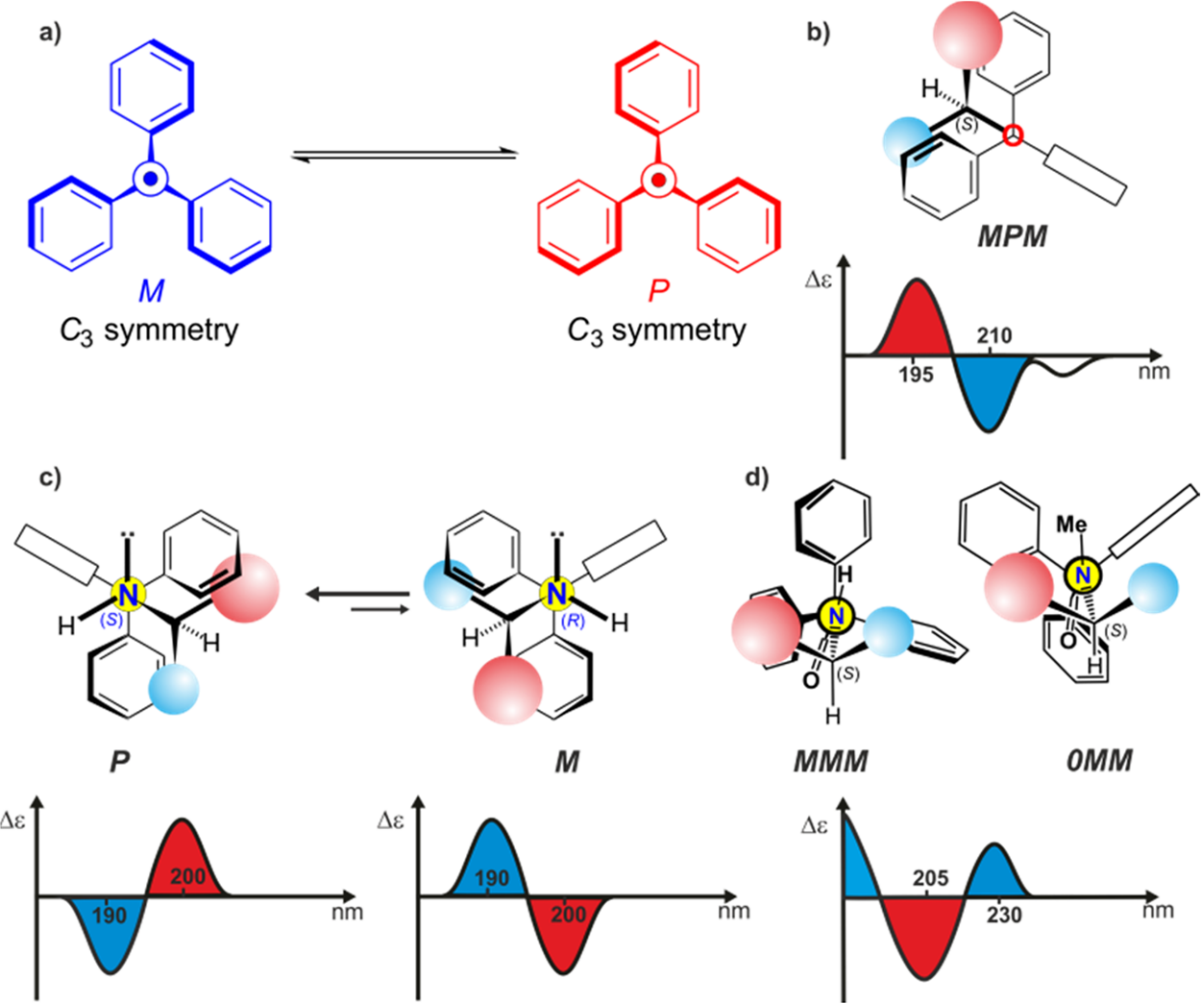
(a) Extreme *C*_3_-symmetric conformers of triphenylmethane propeller. The “bevel gear” mechanism of chirality induction for (b) *O*-trityl ethers and (c) *N*-trityl amines, and correlation of the shape of ECD spectrum with dominant conformation of *O*-trityl ethers and *N*-trityl amines, respectively (projections dawn the O–CPh_3_ or N–CPh_3_ bond). (d) Mechanism of chirality induction and correlation between the shape of ECD spectrum and the dominant conformation of chiral secondary and tertiary triphenylacetamides (projection down the N–C(=O) bond).

From the stereochemical point of view, the trityl moiety represents an unique example of stereodynamic system that resembles a macroscopic rotor with variable blades geometry. Due to a low enantiomerization barrier, the parent triphenylmethane and related systems exist as a mixture of quickly interconverting enantiomers, characterized by the same sense of blade’s twist, either *P* and *M*, as well as by the highest available *C*_3_ symmetry (Figure 1a).

Not until recently has Gawronski demonstrated usefulness of the trityl as a stereodynamic chirality sensor for alcohols and amines.^9−13^ The mutual matching between the permanently chiral part of the molecule (usually called “the inducer”) and the stereodynamic probe has resulted in the appearance of nonzero Cotton effects (CEs) in electronic circular dichroism (ECD).^14,15^ It should be emphasized that the similarities in the patterns of the ECD spectra of trityl derivatives do not directly translate into the similarities in the mechanisms of the optical activity induction. The initially established “bevel-gear” mechanism of chirality induction is dominant for *O*-trityl ethers,^9^*N*-trityl amines,^10^*O*-triphenylsilyl ethers,^16^ and 3,3,3-triphenylpropionic acid derivatives^17^ (see Figure 1b,c). For other derivatives studied so far, the established mechanisms of chirality induction are rather case-sensitive and generally proceed through a set of cooperative interactions.^18,19^ The involvement of the triarylmethyl moiety into the rigid triptycene skeleton eliminates any conformational changes of the propeller. In such cases, the chirality of the whole triptycene system is achieved by proper functionalization of benzene rings.^20^

The intensively studied triphenylacetamides can be considered the counterpart of *N*-trityl amines. A rigid amide spacer linking the inductor and the chromophore part of the molecule does not significantly disturb the chirality induction process^19^ (Figure 1d). However, the dynamic stereochemistry of triphenylacetic acid esters being chiral congeners of *O*-trityl ethers has not been a subject of interest. As early as in 1912, Chugayev reported the optical rotation of menthyl triphenylacetate, the first chiral ester derivative of triphenylacetic acid.^21^ However, neither this nor the later works contributed much to understanding the mechanism of inducing the optical activity in such compounds.^22^ Thus, being for ages isolated curiosities of chemistry, the chiral mono- and diesters of triphenylacetic acid constitute the missing pieces of the jigsaw puzzle showing dynamic stereochemistry of trityl-containing compounds.

In addition to synthetic and stereochemical applications, trityl and related groups are widely utilized in crystal engineering to construct inclusion crystals.^23^ Akazome has a proven propensity of *N*-tritylamino acids to the solid-state enantiodiscrimination of chiral guests.^24,25^ The presence of trityl groups in the amino acid core has prevented the formation of inherent hydrogen bonds, and the loss of hydrogen bonds was compensated for by an inclusion of guest molecules. In the secondary amides of triphenylacetic acid, the trityl group has played the role of supramolecular protecting group for the amide N–H hydrogen bond functionality.^26^ However, the presence of additional supramolecular synthons within the *N*-triphenylacetylamino acid skeleton allowed for the back-activation of the N–H group, then for the formation of associates of the wheel-and-axle structure and/or various multicomponent crystals.^26,27^

The presence of π-electron fragments enables various intra- and intermolecular interactions. Therefore, the trityl group itself and its analogues are considered useful building blocks (tectons) in molecular tectonics.^28,29^ In principle, the multiple phenyl embraces are engaged in simultaneous attractive (mostly the dispersive) interactions^30^ which would allow for the control over the molecular organization in the materials.^29,31,32^ After analyzing the available data and based on his own research, Wuest very recently outlined the key requirements for the use of the triarylmethyl groups to control molecular organization.^29^ One of the most important conclusions of Wuest’s study, showing the difficulties in predicting material structure, was as follows: “strong directional intermolecular interactions will be most effective when closely related alternatives of similar energy are absent”.^29^ Due to the multiple and concerted edge-to-face interactions between interdigitating trityl (and related) groups of opposite helicity, the 6-fold phenyl embraces are considered to be an attractive supramolecular motif. However, even for predisposed trityl-containing compounds the *a priori* prediction of the occurrence of this form is burdened with considerable risk.^29,31^

In contrast, the presence of the highly polar groups in salts of triphenylacetic acid and primary amines allowed for the formation of multicoordinated polyhedrons exhibiting a novel supramolecular chirality in the solid state.^33^ In such crystals, the phenyl–phenyl interactions gave a small or even negligible impact to have control over molecular organization. Despite the hundreds of crystal structures of trityl-containing derivatives reported so far, it is worth emphasizing that no crystal structure of a triphenylacetic acid ester had been deposited in The Crystal Structure Database until this manuscript was written.^34^

Feeling that there are still some unresolved issues in the field of dynamic stereochemistry and molecular tectonics of trityl-containing compounds, which might be properly addressed, we have decided to direct our attention to chiral triphenylacetic acid esters. The structural dynamics of these compounds has never been the subject of an in-depth study. Additionally, little to nothing is known about a possibility of and, thus mechanism of, chirogenesis in chiral esters of triphenylacetic acid. Demonstration of similarities and differences in chirogenesis occurring in triphenylacetic acid amides, esters, and respective *O*-trityl ethers would constitute an outcome of this part of the study.

The chosen research objects are characterized by the absence of supramolecular synthons in the skeleton and by the presence of a chiral side chain (the inducer part) in the molecule. These two factors can affect not only the structural dynamics of the isolated molecule but the association mode and thus the material structure. The possible competition between intra- and intermolecular interactions make the compounds in which more than one trityl groups exist in proximity especially interesting in the context of self-organization of molecules in the crystal. Thus, in the absence of supramolecular synthons that allowed for the long-range order of molecules in the crystal lattice (e.g., formation of the hydrogen bonds cascades), we expect the dominant impact of the phenyl–phenyl interactions on the organization mode of the entities.

## Results and Discussion

### Chirogenesis and Molecular Dynamics of Esters **1**–**22**

**Chart 1:**
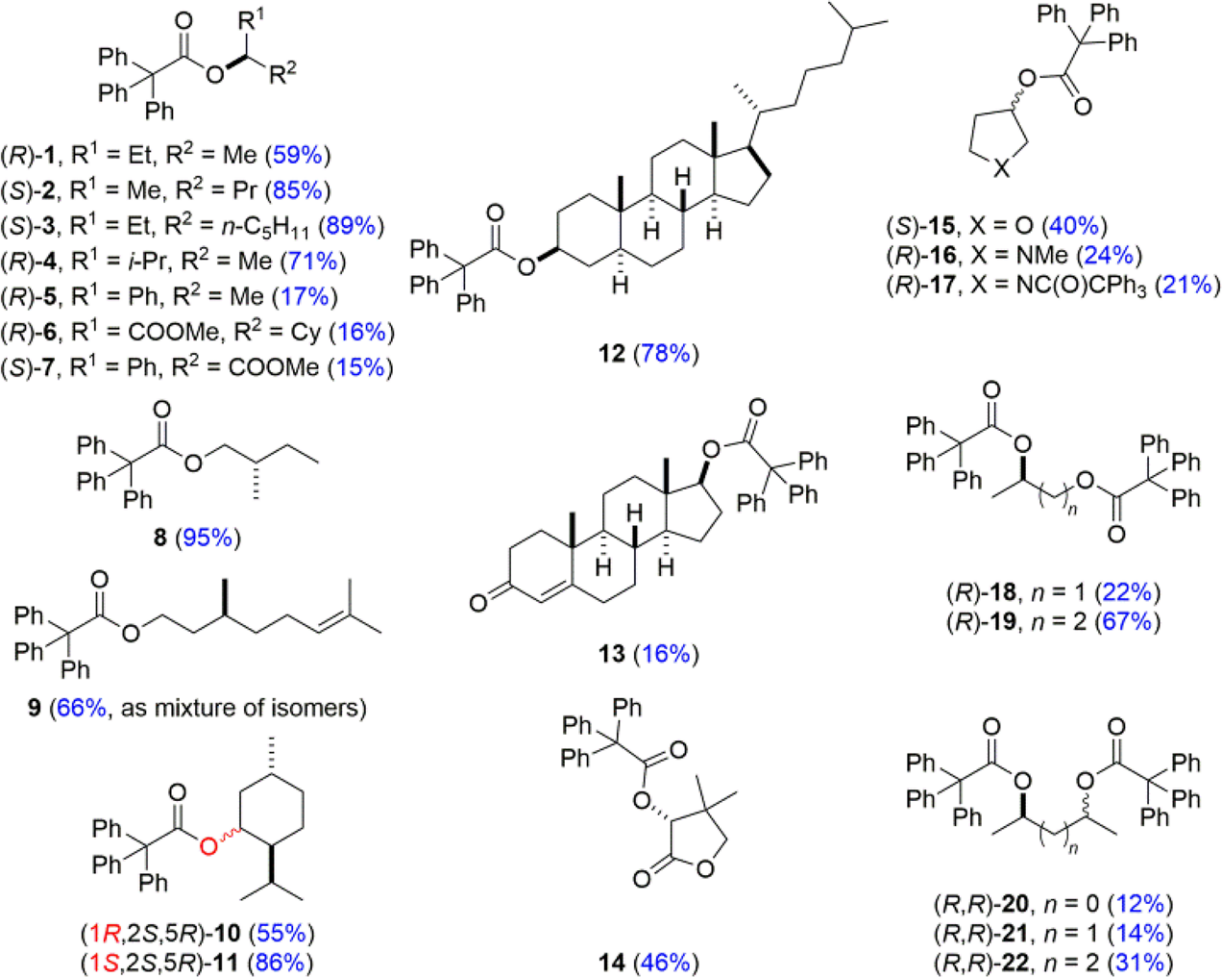
Structures of Compounds under Studya

Although initial attempts to synthesize chiral esters in the reaction of an alcohol with the acid chloride were unsuccessful, the fusion of a triphenylacetyl chloride with an excess of respective alcohol provided compounds **1**–**22** (Chart 1) with yields ranging from 12 to 95%. The reactions in solution that are a natural choice for this type of synthesis either led to the formation of only small quantities of the esters, or in our hands, the reactions were inefficient. We have not observed any racemization nor epimerization in the products.^22^ However, in the particular case of citronellol, during the course of the esterification reaction, migration of a double bond took place. This is most likely due to the reversible addition–elimination of hydrochloride and is not hampered by the addition of the base. Unfortunately, these compounds are not separable from each other, and the isolated yield of **9** refers to the sum of isomers differing in the position of a double bond.

a Values in parentheses refer to the isolated yield of the given product.

The choice of the chiral inducer has been dictated by the availability and the structural variability of a given alcohol. Apart from **5** and **7**, the compounds under study do not contain any other aromatic chromophore, which may, although not necessarily, disturb the chiroptical phenomena. The inducers can be considered as ECD-silent in the region of the trityl group absorption. Therefore, the possibly observed phenomena will result from the generation of optical activity in the chromophore and will have their source in the chirality transfer from the inducer to the probe. Additionally, diesters **20**–**22** give us a chance to study the competitive or cooperative effects on chirality inductions and association.

**Figure 2:**
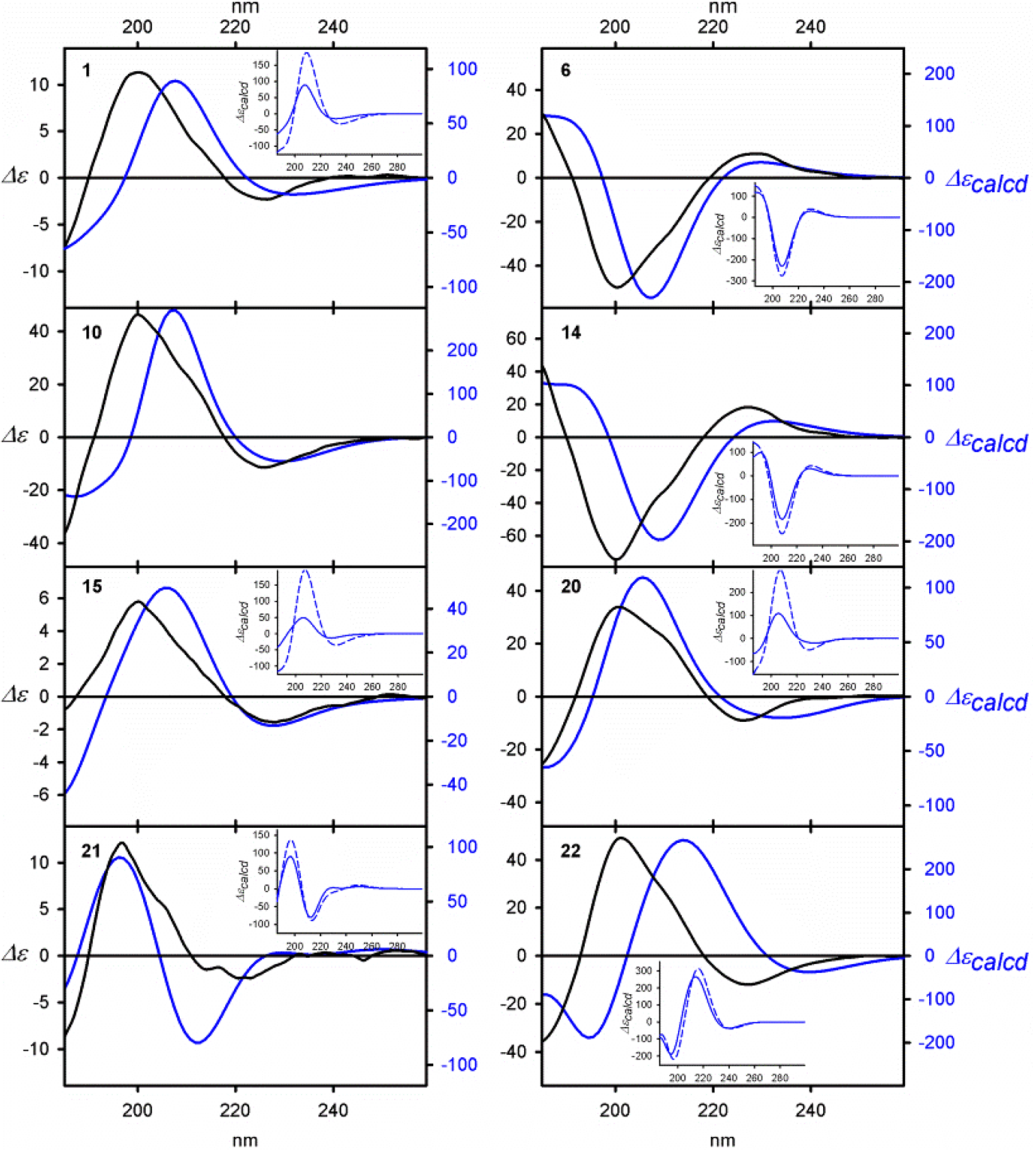
Examples of the ECD spectra of **1**, **6**, **10**, **14**, **15**, and **20**–**22** measured in cyclohexane (solid black lines) and calculated at the TD-M06-2*X*/6-311++G(2d,2p) level (solid blue lines). The calculated ECD spectra were Boltzmann-averaged based on ΔΔ*G* values. Wavelengths were corrected to match the experimental UV maxima. With an exception of **10**, for which only one low-energy conformer has been found, inserts show the comparison between the ECD spectra calculated for the lowest energy conformer of a given compound (dashed blue lines) and the ΔΔ*G*-based and Boltzmann averaged (solid blue lines).

Since they are deceptively simple, the compounds under study are characterized by rather complex conformational dynamics. In each molecule, there are at least five torsion angles for which rotation barriers are expected to be low. The ^1^H NMR spectra of **1**–**22** show only sharp signals, which suggest rather high structural dynamics, associated with a number of easily interconverted conformations. This, in turn, might lead to mutually canceling contributions of conformational diasteoisomers to the overall ECD spectra; therefore, no rise or very weak CEs will be observed. However, the ECD spectra measured in cyclohexane solution of **1**–**22** with no exceptions show CEs in the region of trityl UV absorption, thus confirming the ongoing chirogenesis in a way that leaves no doubt (Table 1 and see Figure 2 for examples of measured ECD spectra). Lowering the concentration of the sample (up to 10^–6^ mol L^–1^) has no effect on the observed phenomena. In fact, with the exception of increasing the noise level, we did not see any changes in the shape of respective ECD spectra. Therefore, a possible aggregation has not played a role at the concentration range between 10^–4^ and 10^–6^ mol L^–1^.

**Table 1:** UV (ε, in dm^3^·mol^–1^·cm^–1^) and ECD (Δε, in dm^3^·mol^–1^·cm^–1^) Data for **1**–**22** Measured in Cyclohexane Solution^a^

compd.	Δε (nm)	ε (nm)^b^
**1**	–2.30 (226); 11.31 (200); –7.46 (185)^c^	75400 (197)
**2**	4.16 (226); −13.90 (199); 12.79 (185)^c^	75800 (197)
**3**	1.19 (224); −2.76 (202); 3.86 (185)^c^	73400 (197)
**4**	–3.24 (225); 14.16 (200); –9.08 (185)^c^	74300 (197)
**5**	–1.00 (225); 2.55 (211); 2.87 (205); 8.14 (191)	121000 (189)
**6**	11.03 (229); −49.85 (200); 29.32 (185)^c^	74900 (197)
**7**	18.53 (222); 13.88 (200); –45.59 (189)	116200 (192)
**8**	–0.39 (216); −1.04 (205); –0.60 (194)	74300 (197)
**9**	0.50 (225); −1.43 (196); 1.61 (185)^c^	66400 (196)
**10**	–11.37 (225); 46.38 (200); –36.50 (185)^c^	72400 (197)
**11**	–2.90 (221); −3.41 (210); −2.31 (200); 4.12 (191)	72800 (196)
**12**	–0.67 (227); 4.82 (200)	74100 (196)
**13**	–0.96 (340); 8.89 (228); 7.09 (191)	65700 (197)^d^
**14**	18.40 (227); −74.45 (200); 43.76 (185)^c^	72600 (196)
**15**	–1.57 (228); 5.80 (200); –0.8 (185)^c^	66100 (197)^d^
**16**	0.63 (224); −5.14 (195); 1.17 (185)^c^	69800 (197)
**17**	11.32 (231); −37.30 (210); −36.62 (198); 29.60 (185)^c^	147700 (190)
**18**	2.55 (231); −7.23 (208); 3.02 (196); –1.65 (185)^c^	141200 (196)
**19**	–13.35 (219); 47.84 (197); –30.06 (185)^c^	140800 (195)
**20**	–9.02 (226); 33.91 (201); –25.59 (185)^c^	142700 (196)
**21**	–1.40 (221); 13.11 (196); –7.69 (185)^c^	143900 (194)
**22**	–11.89 (227); 49.16 (201); –35.73 (185)^c^	141200 (196)

a The concentration of analytes ranged from 1.52 to 2.91 × 10^–4^ mol L^–1^. The spectra were recorded in pure cyclohexane, from 400 to 185 nm, with a scan speed of 50 nm min^–1^ and 8 accumulations (see the Experimental Section).

b Only well-established absorption bands of ε > 2000 were reported.

c End of measuring range.

d Partially insoluble in cyclohexane.

We add that the change of the solvent to more polar acetonitrile has resulted in small or negligible changes in the observed chiroptical phenomena. In fact, only for **5** and **18** could the overall shape of the ECD spectra measured in polar solvent be considered different from those measured in a nonpolar environment, whereas for **2**-**7**, **12**, **14**, **15**, and **19**, an increase of the solvent polarity has caused a decrease of CEs amplitudes while retaining the same shape of the ECD spectrum (see Table SI_2, and see copies of ECD spectra posted in Supporting Information).

The low-energy CEs associated with ^1^L_b_ electron transition appear at around 225 ± 6 nm, whereas the second more intense and opposite-sign ECD bands of ^1^B type are found at around 200 ± 5 nm. The third, higher-energy CEs reach their maxima usually bellow 185 nm. Those associated the UV spectra are dominated by strong absorption maximum at around 195 ± 5 nm. Other transitions do not form any well-distinguished shoulder peaks apart from the lowest energy ones appearing at around 260–280 nm. Strictly speaking, for the majority of cases this particular band is hardly visible, and the extinction coefficient (ε) does not exceed 2000. The presence of an additional chromophore of the ketone, enone, and COOR type does not disturb this pattern, although additional CEs are visible in the region of the n−π* electron transition. Replacing of an aliphatic substituent at stereogenic center by the aromatic phenyl group makes the ECD spectra of **5** and **7** more complex. As one might expect, the weakest induced CEs are found for derivatives **8** and **9**, in which the ester group is spaced from the stereogenic center. The existence of **9** as the mixture of isomers has not affected the observed phenomena.

This simple relationship (the higher structural difference between the substituents flanking stereogenic center, the more intense the CEs) can be assigned with a great deal of caution for acyclic derivatives **1**–**4** and **6**. For these compounds, the steric power of aliphatic substituents in the dynamic chirality induction rises as follows: Me < Et < *n*-C_5_H_11_ < *n*-Pr < *i*-Pr < Cy. For cyclic monoesters, such a simple relationship is not seen. In menthol derivatives **10** and **11**, the change of the absolute configuration at the C1 carbon atom (from *R* to *S*), associated with the change of position of the ester group from equatorial to axial, led to the significant decrease of the CEs amplitudes. The highest amplitude of CEs has been found for **14**, which can make an impression that the impact of *gem*-dimethyl group on the ECD spectrum overwhelms that of the carbonyl group.

In the context of efficiency of the dynamic induction of optical activity, the derivatives **12** and **15**, where there is no significant difference between the substituents’ flanking chirality element, deserve special attention. In the former case, the probe stereodifferentiates the CH_2_ and the CH_2_C*H(C) groups in the β position. In the case of the latter, tetrahydrofuran derivative **15**, the structural difference between the −O– and −CH_2_– groups is even more subtle. However, the efficiency of the chirogenesis, as estimated on the basis of CEs amplitudes, is higher than that observed for more structurally diversified **12**. Quantitatively, for derivative **15**, the low-energy CE appearing at around 227 nm is over 2-fold higher in intensity than that measured for **12** (Δε = −1.57 vs Δε = −0.67). However, the higher-energy CD band, located at around 200 nm, is only 1.2 times higher for **15** (Δε = 5.80) than the respective CE that has been found experimentally for **12** (Δε = 4.80).

As the structure and the energy relationships between conformers of the given compound cannot be determined experimentally, we have conducted calculations at the appropriate DFT level for the representative examples **1**, **4**, **6**, **10**, **11**, **14**, **15**, **18**, and **20**–**22**.^35−38^ Eventually, this could shed light on the mechanism of chirality induction (for details regarding the calculation methodology, see the Supporting Information). Since the detailed elaboration of each structure might obscure the basic problem, we will discuss here some generalities. The best combination of methods for structure/spectra prediction has been chosen by comparison of experimental and Boltzmann averaged CD spectra calculated for the structures optimized with the use of different density functionals. In the cases discussed here and for the same method used for geometry optimization, the results of ECD calculations with the use of the M06-2X hybrid functional only slightly outperforms results obtained with the use of CAM-B3LYP hybrid functional. Therefore, the method of geometry optimization seemed to be crucial for the correctness of the final results.^14,36^ While for esters **1**, **4**, **10**, **11**, and **14** the “classical” B3LYP hybrid functional gave the best results,^37^ the conformational dynamic and structure of individual conformers of **6** and **15**, affected by CH···O interactions between inductor and acceptor, were better reproduced by the newer M06-2X hybrid functional. The empirical correction for dispersion that was added to the B3LYP functional was only relevant in the cases of **18**, **20**, and **21** having the ester groups in the close proximity.^39^ However, for the remaining diester, **22**, the London dispersive interactions did not much affect the structure of the compound. For a given compound, the wavelengths in UV and ECD calculated spectra (overlapped by Gaussian function) were multiplied by the same scaling factors obtained from a simple equation: UV λ_max(exp)_/UV λ_max(calcd)_. In the cases discussed here, the scaling factors were ca. 1.05, which means that the calculated spectra were blue-shifted with respect to the experimental ones.

The number of thermally available conformers that need to be taken into account during further analysis varies, depending on the structure of the ester. For a highly structurally diversified ester, such as **10**, there was only one low-energy conformer found. In contrast are the highly flexible diester molecules. In the example case of **22**, we found 12 conformers (at the B3LYP/6-311G(d,p) level) that ranged in relative energies by less than 2 kcal mol^–1^. Among these individuals, the lowest-energy conformer No. 3 was found almost 2-fold more abundant than the second lowest energy conformer No. 1. The estimated ΔΔ*G*-based populations for these two species were 15 and 28%, respectively, for conformers Nos. 1 and 3 of **22**.

**Figure 3:**
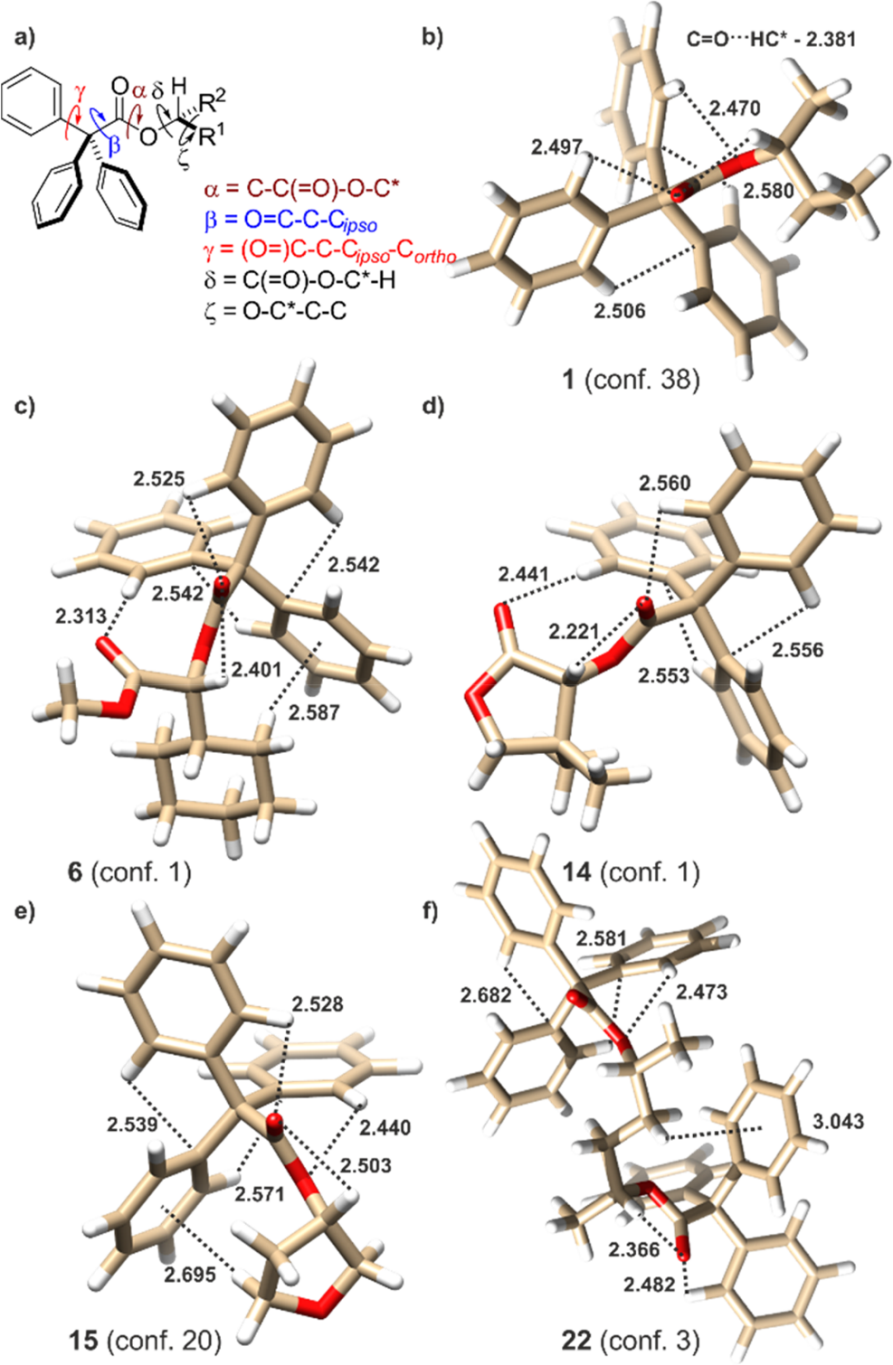
(a) Definition of torsion angles α, β_1_-β_3_, γ_1_-γ_3_, δ, and ζ. Example structures of calculated, ΔΔ*G*-based, lowest-energy conformers of esters (b) **1**; (c) **6**; (d) **14**; and (e) **15** and the (f) *C*_2_-symmetrical conformer no. 3 of **22**. Dashed lines indicate possible attractive interactions. Distances are in Angstroms.

The selected structural, energetic, and spectral data found for the lowest energy conformers of **1**, **4**, **6**, **10**, **11**, **14**, **15**, **18**, and **20**–**22** have been juxtaposed in Table 2; Figure 3 shows the example structures. All remaining theoretical results are in the Supporting Information.

**Table 2:** ΔΔ*G*-Based Percentage Populations (pop), Values of the γ_1_–γ_3_ Angles (deg), Helicities of Trityl Chromophore, and Sequences of Cotton Effects (CEs) and Similarity Index (*∑*) Calculated for the Lowest Energy Conformers of **1**, **4**, **6**, **10**, **11**, **14**, **15**, **18**, and **20**–**22**

compd.^a^	pop.	γ_1_^b^	γ_2_^b^	γ_3_^b^	helicity^c^	CEs^d^	*∑*^e^
**1** (38)^f^	37	67.69	–13.15	47.65	*PMP*	∓/–	0.97
**4** (46)^f^	27	–41.80	–66.71	–34.96	*MMM*	±	0.92
**6** (1)^g^	63	4.70	–63.15	–52.11	*0MM*	±/+	0.95
**10** (1)^f^	100	66.50	–11.31	49.05	*PMP*	∓/–	0.98
**11** (56)^f^	59	66.14	42.20	37.34	*PPP*	∓/–	0.91
**14** (1)^f^	78	6.55	–63.59	–51.80	*PMM*	±/+	0.98
**15** (20)^g^	31	64.95	–12.48	49.42	*PMP*	∓/–	0.95
**18** (32)^h^	30	42.22	84.16	–25.96	*PPM*	±/+	0.95
**20** (22)^h^	30	43.58	55.81	52.07	*PPP*	∓/–	0.96
		78.94	–28.17	37.85	*PMP*		
**21** (1)^h^	75	50.87	51.46	47.58	*PPP*	∓/–	0.85
		–86.04	39.98	26.47	*MPP*		
**22** (3)^i^^,^^j^	28	11.74	69.89	–16.19	*PPM*	∓/–	0.97

a The number of the lowest energy conformer is given in parentheses (conformers are numbered according to their appearance during conformational search).

b γ = (O=)C–C_Tr_–C_*ipso*_–C_*ortho*_ (of the two possibilities the absolute values ≤ 90° has been chosen).

c Helicity of the phenyl rings is defined as *M* (−90° < γ < 0°), *P* (0° < γ < 90°) or 0 (for γ angles deviating from zero by less than |5°|).

d Calculated at the TD-M06-2*X*/6-311++G(2d,2p) level for the given the lowest energy conformer sequence of Cotton effects.

e Similarity between ECD spectra: experimental and the calculated for the given lowest energy conformer.

f Optimized at the B3LYP/6-311++G(d,p) level.

g Optimized at the M06-2*X*/6-311++G(d,p) level.

h Optimized at the B3LYP-GD3BJ/6-311G(d,p) level;

i Optimized at the B3LYP/6-311G(d,p) level.

j *C*_2_ symmetry.

The structure of each individual conformer might be described by a set of torsion angles α, β_1_–β_3_, γ_1_–γ_3_, δ, and ζ. The angles α = C_Tr_–C(=O)–O–C*and δ = C(=O)–O–C*–H are, to some extent, correlated and describe the conformation of TrCOOC*H(R_1_,R_2_) fragment. Without exception, the α angles adapt an *antiperiplanar* (*ap*) conformation. The position of the C=O group and the C*–H bond are the best described by the δ angle. Only for a few higher-energy structures did the conformation of the δ angle deviate from either *synperiplanar* (*sp*) or *synclinal* (*sc*). The favored *syn* position of the C=O and C*–H bonds is caused by the interaction of the opposite polarized dipoles; hence, any deformation of the δ angle toward the parallel arrangement of the dipoles will increase the energy of the entire molecular system.

The ζ torsion angle (ζ = O–C*–C–C or O–C*–C–C*) describes the conformation of the chiral backbone. The values of the ζ angle vary for these compounds, where the carbon chain has the possibility of free rotation or for those in which the ring undergoes pseudorotation.^40^ For example, for **1** the ζ angle adapts either a ±*sc* or an ±*ap* conformation, but for the even more flexible **15**, no specific range of values can be distinguished. In contrast, there are rigid **10** and **11**, where the ζ torsion angle adapts only a −*sc* or +*sc* conformation, respectively. Conformation of the backbone affects energy of the whole molecule and, to a lesser extent, the chiroptical properties.

Conformation of the trityl group, described by the sets of β_1_–β_3_ and γ_1_–γ_3_ angles, are of the key importance for the observed induced ECD. The β_1_–β_3_ torsion angles (β = O=C–C–C_*ipso*_) determine the spatial orientation of each phenyl blade relative to the carbonyl group. In the majority of cases, one of the phenyl groups lies parallel to the (O=)C–C(−C_*ipso*_) bond (the associated β angle is in an *ac* conformation), whereas the second phenyl is almost parallel to the C=O bond (the β angle adapts an *ac* conformation, but of the opposite sign to the previously mentioned one). The *sp* conformation of the third β angle resulted from possible C=O···H–C_*ortho*_ interactions (the calculated C=O···H–C_*ortho*_ distance ranging from 2.11 to 2.60 Å). These interactions constitute the main factor affecting the structure (helicity) of the trityl group. Furthermore, the conformation of this particular ring, as defined precisely by the γ angle (γ = (O=)C–C–C_*ipso*_–C_*ortho*_, of the two possibilities the absolute values ≤90° has been chosen) is ±*sc*. The orientation of the second phenyl, parallel to the (O=)C–C(−C_*ipso*_) bond, is ±*sp* and is controlled by the attractive (O=)C–O···H–C_*ortho*_ interactions. The remaining phenyl ring, whose protons are not involved in any CH···O interactions, adjusted the conformation to other phenyl rings present in the chromophore and to the substituents flanking chirality element. This particular phenyl ring orientates itself in such a way as to maximize the probability of both intratrityl CH···π and π···C_sp3_H interactions with the protons from the chiral backbone (if possible). In other words, this particular conformation of the blade appears at the more crowded side of the molecule and the plane of the phenyl group is facing the bulkier substituent at the stereogenic center.

In the particular cases of the lowest-energy conformers No. 1 of **6** and No. 1 of **14**, the C=O···H–C_*ortho*_ interactions involving the second ketone or carbomethoxy carbonyl group compete and prevail over the attractive (O=)C–O···H–C_*ortho*_ interactions. The possibility for the double C=O···H–C_*ortho*_ structure-stabilizing interactions limits the number of thermally available conformers. Hence, not only the steric hindrance (*gem*-dimethyl group) but, most of all, the strong electrostatic interactions determine the structure and properties. This is the reason why these compound show the unexpectedly high degree of induction of optical activity among all items under study.

As an effect of the cascade process, a nonequal population of optically active conformational diasteroisomers is formed. Such a residual diasteroisomerism cannot be directly observed experimentally; however, a nearly perfect similarity between the calculated and the experimental ECD spectra strongly supports this conclusion (Figures 2 and SI_79–SI_130).

The performed theoretical analysis led to the conclusion that even for the conformationally labile esters discussed here the dominant impact on the overall ECD spectrum can be attributed to the lowest-energy conformer of the given compound. At this point of the discussion, we took the liberty to make a digression. “The lowest-energy conformer of a given compound is considered to dominate over the overall ECD spectrum.” However, this is a kind of generalization that is not supported by any strict rule. The relation ‘the more the abundant conformer, the more effect on the chiroptical response’ is rather an expectation that has now become the prevailing rule. One should bear in mind that the overall ECD spectrum is a function of population of conformers as well as rotatory strengths generated by them. Moreover, for a given structure, there is no direct correlation between rotatory strengths and conformer population.

However, in the cases discussed here, the expected correlations between the abundance of the given lowest energy conformer and its impact on the overall ECD spectrum has been fulfilled. To be as strict as possible, for each of compounds **1**, **4**, **6**, **10**, **11**, **14**, **15**, **18**, and **20**–**22**, we have estimated the similarity factors (*∑*) between the experimental ECD spectrum and the one calculated for the lowest energy conformer of a given ester (see Table 2).^41^ With the exception of **21**, the similarity factors *∑* have ranged from 0.91 to 0.98, which quantitatively indicated a very good match between the experimental and theoretical results. The lower *∑* value estimated for **21** (0.85) resulted from deficiencies of experimental data rather than poor reproduction of material reality by theoretical methods.^42^

Referring again to the lowest energy conformers as the representative examples, we have correlated the helicity of the trityl chromophore, defined either as *M* (−90° < γ < 0°), *P* (0° < γ < 90°), or 0 (−5° ≤ γ ≤ 5°) to the sequence of the CE appearing in the spectral region of the trityl UV absorption. The negative/positive/negative (∓/−) sequence of CEs correlates with *PPP* or *PPM* chromophore helicity, whereas in a *MMM* or *MM0*-helical chromophore, CEs of the opposite sequence, namely, ±/+ are generated.

**Figure 4:**
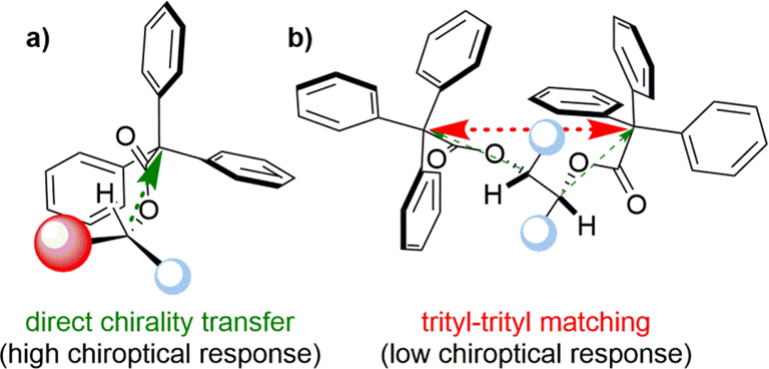
(a) Direct chirality transfer from stereogenic center to the trityl chromophore. (b) Trityl–trityl matching interrupting the chirality transfer from stereogenic centers to the chromophores.

One can expect that for the compounds having more than one noninteracting ester groups, the CEs magnitude should be the linear combination of the contributions from individual chromophores. However, the presence of an additional trityl group in the proximity does not automatically increase the observed CEs amplitudes. This is particularly seen for symmetrical derivative **21**. Significantly, the separation of the two stereogenic centers by one −CH_2_– group results in a reduction in the intensity of the CEs. In compounds **18**, **20**, and **21** the trityl–trityl matching interrupts the cascade chirality induction from stereogenic center to the chromophore (see Figure 4). Thus, the chiroptical response is much smaller than that observed for **19** and **22**, where the TrCOO fragments are separated by two methylene groups. In **19** and **22**, again the stereogenic center(s) play the key role in chirality induction, which in these cases takes place accordingly to the above-described mechanism.

### Control over the Solid-State Molecular Organization in the Crystals of Esters

**Figure 5:**
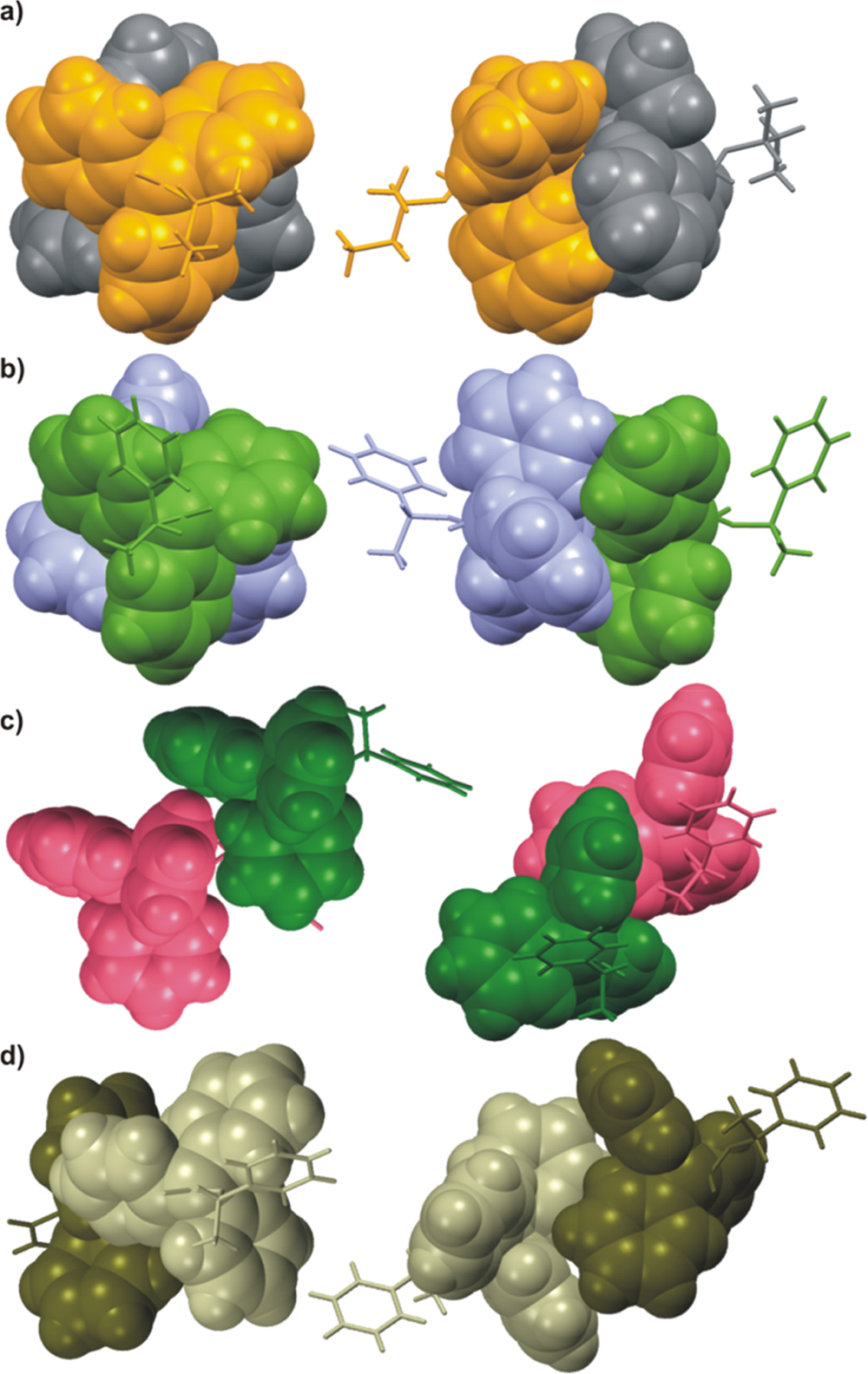
Examples of the 6-fold phenyl embrace supramolecular synthons (shown as space-fill models) found in the crystal structures of (a) **1** and (b) **5-**α. (c) Interaction of trityl groups without formation of phenyl embrace synthon in **5-**β, and (d) offset 6-fold phenyl embrace in (*rac*)-**5**.

As the intentionally designed ester molecules do not contain functional groups commonly regarded as hydrogen bond donors, the classical hydrogen bonds cannot be observed in the crystal structure. However, the presence of the trityl group in the molecules favors the occurrence of π-electron system interactions. We have decided to use such a nonclassical supramolecular tool (the trityl group) allowing to predict the organization of molecules in the crystal. In the particular cases, we have expected to observe a characteristic supramolecular motif, which is the 6-fold phenyl embrace (Figure 5a,b).^29^ In the great majority, the analyzed compounds were chiral, and we expected to observe the offset 6-fold phenyl embrace formation (Figure 5d). However, our previous experience with trityl-containing derivatives has shown that they can crystallize with an increased number of molecules in an asymmetric unit (*Z*′ > 1). Thereby, in an asymmetric unit we often have observed two (or more) molecules of the opposite helicity, where the trityl groups formed *pseudocentrosymmetric* dimers.^27^ The benefits of creating this supramolecular synthon are comparable to those of the formation of a classical hydrogen bond. The percentage share of individual intermolecular interactions in the Hishfeld surface for molecules in the crystal structure was calculated and is shown in the Figure SI_131. The summary of crystallographic data for analyzed compounds is presented in Table SI_55.

**Figure 6:**
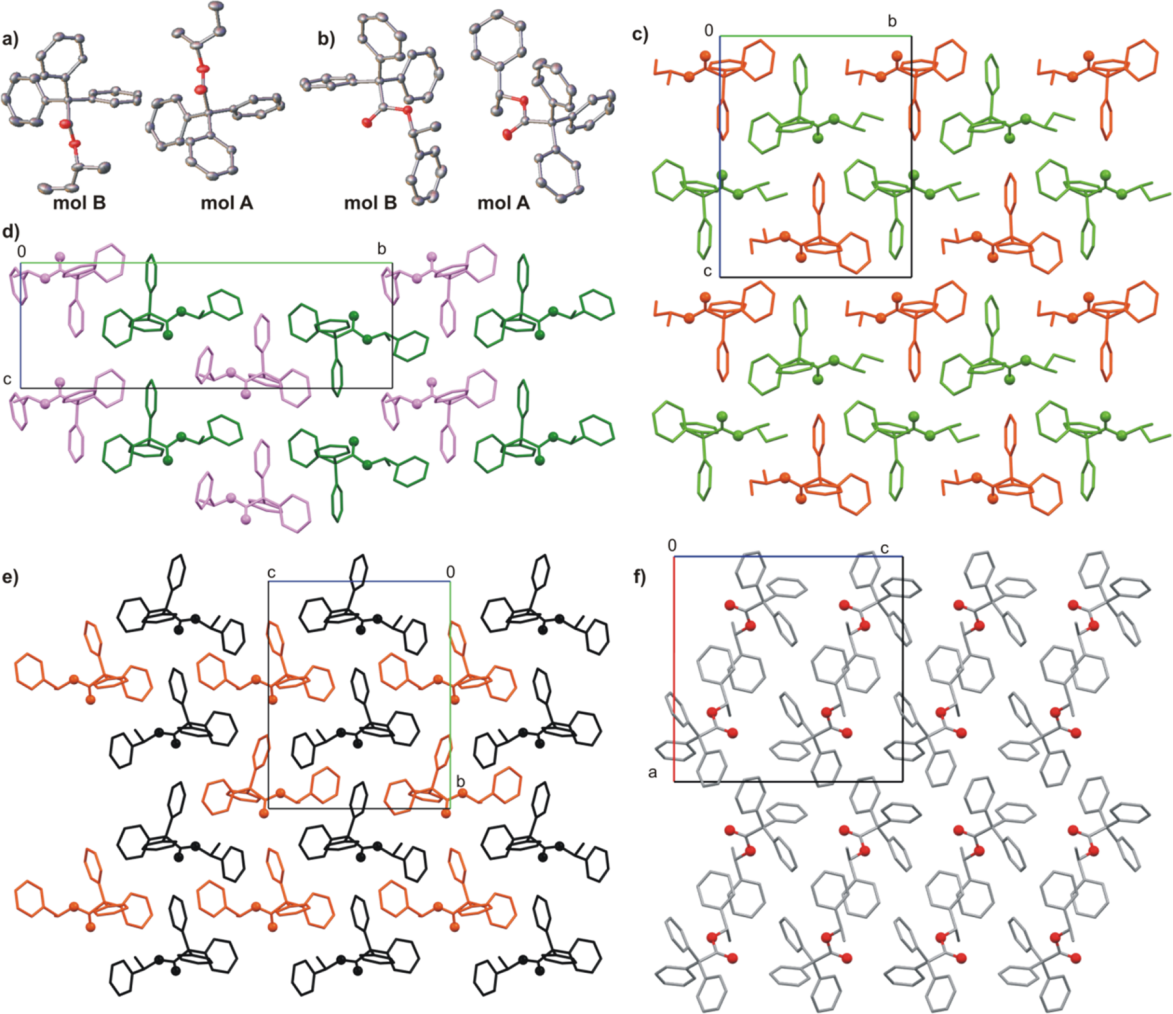
(a) Molecular structure of asymmetric unit of **1**. (b) Molecular structure of asymmetric unit of compound **5**. (c) Molecular packing in the crystal of **1** (mol **A**, green and mol **B**, orange) and comparing of molecular packing in the crystal structures of (d) **5**-α (mol **A**, green and mol **B**, violet), (e) **5**-β (mol **A**, orange and mol **B**, black), and (f) (*rac*)-**5**. Hydrogen atoms are omitted for clarity. The oxygen atoms are shown as balls.

We have chosen compound **1** as a model molecule in the structural study. The bulky trityl part of the molecule is expected to dominate the crystal packing mode in **1**. In the crystal of **1**, the asymmetric unit consists of two symmetrically independent molecules **A** and **B**, which differ in the conformation of the 2-butyl chain. In molecule **A** the aliphatic part adapts bent conformation, while in molecule **B** it is is extended. In the crystal structure, the trityl groups arrange themselves as propellers of opposite helicities and mutually interact to form the supramolecular six-ring motif, stabilized by edge-to-face interactions. The three-dimensional crystal structure is stabilized by numerous C–H···π interactions involving also aliphatic 2-butyl substituents as donors (Figure 6a,**c**).

The 6-fold phenyl embrace supramolecular synthon is almost centrosymmetric, so we have decided to check the effect of the stereogenic center and enantiomeric purity on the molecular packing in the crystals of (*R*)-**1** and (*rac*)-**1**. Comparison of the lattice parameters and analysis of the packing mode of molecules in crystals of (*rac*)-**1** and (*R*)-**1** have shown that the crystals are isostructural. Furthermore, (*rac*)-**1** crystallizes as a solid solution of the enantiomers (the refined ratio of the occupancy factors is 77.5:22.5). The disorder in the crystal concerns not only the configuration on the stereogenic center but also the conformation of the 2-butyl chain of the molecule: the alkyl group adapts either an extended or bent conformation. The (*rac*)-**1** is an example of the specific protective effect of the trityl group on the stereogenic center. In other words, the packing of the mode of the molecules in the crystal is indifferent to both the absolute configuration at the stereogenic center and conformation of the aliphatic chain.^27^

Replacing the aliphatic substituent with the 2-phenylethyl group resulted in the emergence of competition for the interactions of trityl systems. Compound **5** crystallizes in two polymorphic forms, designated here as **5**-α and **5**-β. Interestingly, both forms are monoclinic, belonging to the *P*2_1_ symmetry group, and the crystals of both forms were obtained in one crystallization. Surprisingly, the X-ray powder pattern, measured for the ground sample, shows no diffraction picks that would indicate the presence of a detectable amount of **5**-α (Figure SI_135). It can be assumed that under the conditions of crystallization, **5**-α is formed in a very small amount, while **5**-β is more preferred. However, the transformation of **5**-α formed during the primary crystallization into the **5**-β polymorph, by grinding the sample in a mortar, cannot be excluded.

For both polymorphs of **5**, the asymmetric unit contains of two molecules, **A** and **B**, that differ in geometry. In the case of the **5**-α polymorph, the trityl groups of **A** and **B** molecules form propellers characterized by opposite helicities and arranged in the 6-fold phenyl embrace supramolecular synthon stabilized by edge-to-face interactions. In the crystal, molecules **A** and **B** form alternately arranged layers which penetrate each other (see Figure 6b,d). In the crystal of **5**-β, the trityl groups also form propellers, but of the same *MMM* helicity, so it is impossible to create expected supramolecular motif. In this particular case, the trityl groups from **A** and **B** interact with each other, but the acceptor of the C–H···π interaction is the outer side of the trityl group (Figure 5c). As predicted, the phenylethyl substituent of molecule **A** interacts with the inner side of the trityl system of **B** through both edge-to-face and C–H···π interactions (the CH_3_ group is the donor). Similar to the α form, one can note the formation of alternating layers of molecules **A** and **B** in the crystal structure of **5**-β (see Figure 6e).

In principle, introducing centrosymmetry into the system should result in increased possibilities of forming the desired (centrosymmetric) supramolecular synthon. In the crystal structure of (*rac*)-**5**, the trityl groups interact with each other; however, the 6-fold phenyl embrace motif is not observed. The acceptor for the edge-to-face interactions is the outer side of the trityl group (see Figure 6f). Also the structure of the molecule itself differs from that found in the crystals of the α and β polymorphs, which proves high conformational liability of this compound.

The introduction of a relatively large menthol (or neomenthol) substituent to the ester molecule resulted in a reduction of the trityl groups interactions. In the crystal structures of **10** and **11**, the interactions of trityl groups are very limited and are replaced by interaction with a menthyl substituent. In both cases, the crystals are made from layers of molecules. The mutual alignment of molecules, the structure of layers, and intermolecular interactions are closely related to the geometry of the substituent (see Figure SI_136).

**Figure 7:**
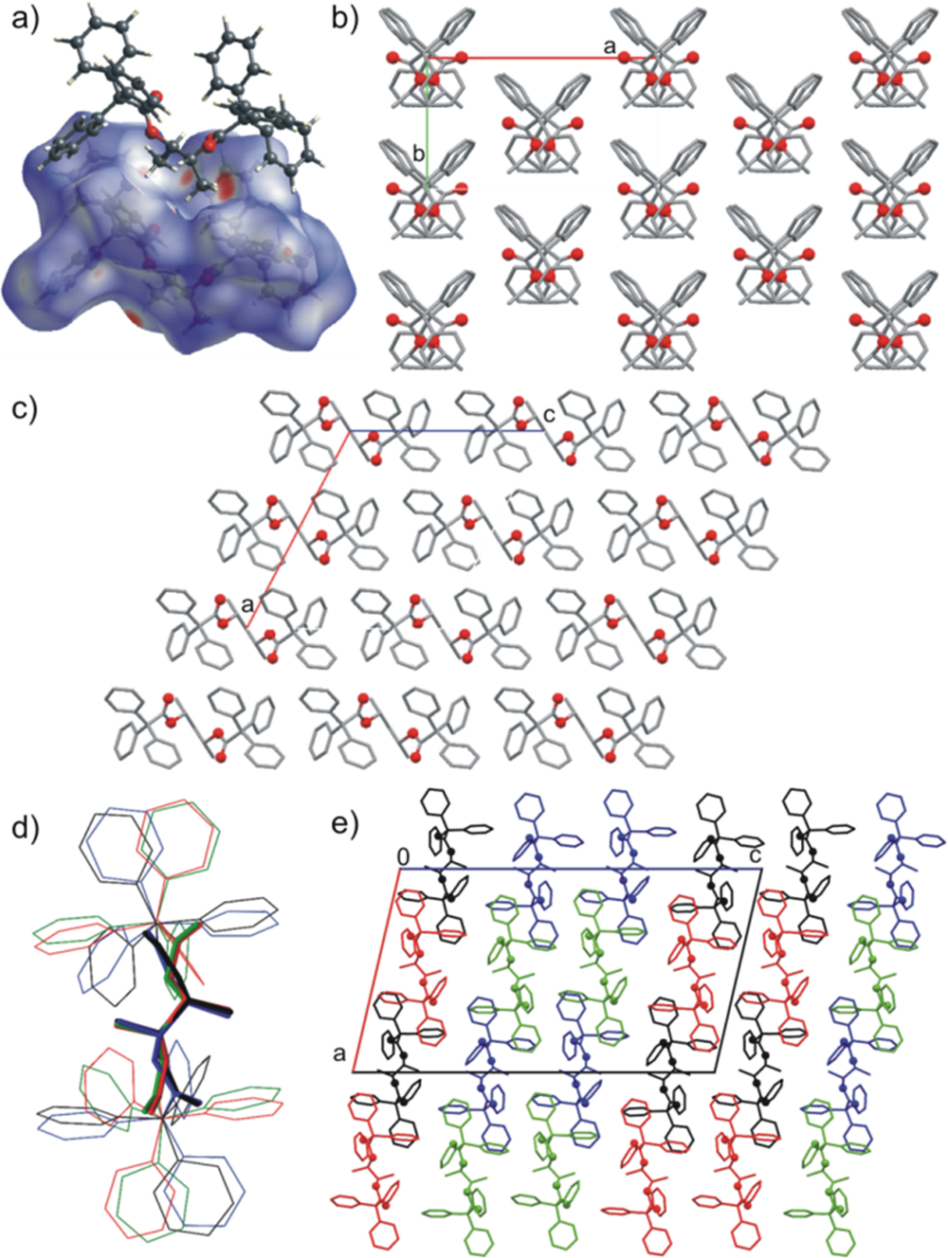
(a) Intermolecular C–H···π interaction in crystal structure of **18**. (b) Molecular columns in crystal of **21** (view along *c*-axis). (c) Molecular packing in crystal of **21** (view along *b*-axis). (d) Comparison of the geometry of symmetrically independent molecules in crystal structure of **20**. (e) Molecular packing in the crystal of **20** (alternating layers of **A** + **B** and **C** + **D** molecules). Hydrogen atoms are omitted for clarity. The oxygen atoms are shown as balls.

A separate group is formed by derivatives containing two trityl substituents at the opposite ends of the aliphatic chain. In terms of the geometry of the molecule and the arrangement of the molecules in the crystal, compounds **18**, **19**, and **21** turned out to be very similar. It is worth noting that for all these compounds the helicity of all trityl groups is *MMM*. This could lead us to a simple supposition that formation of the desired 6-fold supramolecular motif would not be possible. In the crystal of **18**, the molecule lies on the 2-fold axis passing through the C_sp2_–C_sp2_ bond. Such an arrangement of the molecule causes substitutional disorder: The position of the methyl group in the molecule cannot be determined unambiguously, and in the adopted model, it is equally likely attached to the C2 or C2′ atom (the atom numbering scheme is shown in Figure SI_137). The molecule is folded in such a way that a kind of cavity is formed between the trityl groups, into which another molecule fits, and the whole system is stabilized by the C–H···π interactions (Figure 7a). This type of association of the molecules is observed in the crystals of compounds **18**, **19**, and **21**. The crystal structure of **19** is disordered in a manner similar to that found for crystal of **18**. The methyl group in the crystal structure of **19** cannot be located; therefore, in the adopted model it is equally likely to be attached to the C2 and C4 atom (the atom numbering scheme is shown in Figure SI_138). This is, however, not the only disorder. As mentioned, the molecules are arranged in columns; however, in the crystal of **19** some of them are shifted by half the length of the molecule relative to the adjacent columns. This is the case for about 15% of the columns in the crystal (disorder model is presented in Figure SI_138). In general, in the crystal structures of **18**, **19**, and **21**, the molecules are arranged in columns stabilized as a whole by compensation of C–H···π interactions.

The structure of the column is stabilized by the mutual interactions of the trityl groups, but the 6-fold phenyl embrace synthon has not been found in the crystals of **18**, **19**, and **21**. The characteristic motifs of the arrangement of molecules are shown in Figure 7b,c. Is worth emphasizing that in the case of the structure of compound **19**, in order to maintain the characteristic arrangement of the molecules, it should be assumed that the shifting of the columns by half the length of the molecules occurs much more often (it concerns half of them). Due to the fact that the molecular arrangement motif seems to be repetitive in this group of compounds, it can be assumed that this is the reason for the column disorder (as a tendency toward a more favorable arrangement of the molecules).

The crystal structure of **20** is an exception. The compound crystallizes with four molecules in the asymmetric unit, and the geometry of the molecules (in pairs **A** and **C** as well as **B** and **D**) remains very similar. Similar to compounds **18**, **19**, and **21**, the helicity of both trityl groups in one molecule is always the same: *PPP* for molecules **A** and **C** and *MMM* for molecules **B** and **D**. Specific sorting of the molecules in the crystal structure is observed. Molecules **A** and **B** as well as **C** and **D** form alternating layers (001), as shown in the Figure 7d,e. In the crystal structure, the interacting trityl groups (in the pairs **A**–**B** and **C**–**D**) led to the formation of a pseudocentrosymmetric 6-fold phenyl embrace supramolecular synthon.

## Conclusions

In this work, some attempts have been made at a comprehensive approach to triphenylacetic acid esters, and the compounds are characterized by high structural diversity. The usability and versatility of such specific trityl derivatives as chirality-sensing stereodynamical probes has been proven. At the molecular level, the mechanism of action of these compounds is based on some fundamental processes, namely, chirality induction and chirality transfer through a set of weak but complementary noncovalent interactions. Thus, the formation of sets of conformational diasteroisomers, characterized by a specific propeller’s twist, is responsible for the generation of optical activity.

The electronic circular dichroism spectroscopy in conjunction with theoretical calculations enables the determination of such a residual stereoisomerism in the chiral triphenylacetic acid ester. The tendency to maximize the attractive CH···O and CH···π interactions is considered to have control over the conformation of the molecule and the trityl fragment in particular. In the first approximation, the ability to generate nonzero Cotton effects depends on the structure of the inducer. A greater structural differentiation is expected to reflect in higher power in dynamic chirality induction. However, the situation is not as simple as it might seem at first glance, and there are some additional factors that should be taken into account. Definitely, more effective chirogenesis is observed for derivatives in which the TrCOO group is attached directly to the stereogenic center. For esters **8** and **9**, having the stereogenic center spaced from the oxygen atom and thus, from the “hub” of the propeller, the observed amplitudes of Cotton effects are the weakest within the whole series. However, the contingency to additional C=O···H–C_*ortho*_ interactions with substituent’s carbonyl group seems to be an equally important or even more important structural factor than steric hindrances.

**Figure 8:**
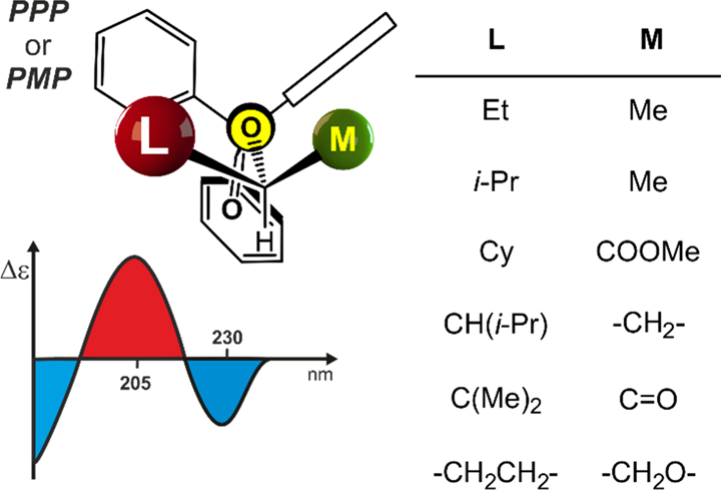
Correlation model of ECD spectrum with the dominant conformation for chiral esters of triphenylacetic acid (projection down the O–C(=O) bond).

Looking more broadly, the transmission of information from the chiral inducer to the structurally adaptable chromophoric probes is not only an interesting phenomenon but also has found practical applications in stereochemical assignments.^12^ A simple model of the optical activity of chiral esters of triphenylacetic acid, as proposed by us (shown in Figure 8), allows the sequence of CEs to be correlated within the substitution pattern at the stereogenic center (not necessarily with the absolute configuration).

Comparison of triphenylacetic acid esters with previously studied triphenylacetamides leads to the conclusion that in both cases the mechanism of generating optical activity is slightly different. For both types of compounds, the C=O···H–C_*ortho*_ interactions most affect the structure. However, for secondary amides, the amide group acts as a specific hydrogen bond donor that additionally stabilizes the conformation through attractive (N)H···C_*ipso*_ interaction.^19^ In the case of esters, the linking oxygen atom serves as hydrogen bond acceptor. The tertiary amides have no counterpart among esters and control their conformation by sterical repulsions between trityl group and substituent at the nitrogen atom. The direct comparison of ECD data found for **1** with that of the respective triphenylacetamide having 2-butane substituent at the nitrogen atom clearly indicates the greater efficiency of the chirality transmission process in the ester.

The bent structure of chiral *O*-trityl ethers, possibly by directing sterical interactions between the inducer and the chromophore, led to the generation of strong chiroptical response.^9^ For example, the consequence of the bevel gear mechanism of chirality transmission taking place in the trityl ether of menthol was the appearance of intense CEs in the higher-energy region of ECD spectrum (Δε = +25.6 at 208 nm and Δε = −80.4 at 194 nm). On the contrary, CEs measured for the trityl ether of (*S*)-2-butanol (Δε = −3.8 at 208 nm, Δε = 16.7 at 194 nm) are comparable to those measured for **1**.^9^ However, the chirogenesis efficiency in the dynamically chiral trityl derivatives rises as follows: triphenylacetamides < triphenylacetic acid esters < *O*-trityl ethers.

In general, the process of dynamic induction of an optical activity in any probe is easily observed for inducers of significant structural variability. The inducers, characterized by small or even negligible differences in substituents flanking stereogenic center, are studied rather unwillingly. The unprecedently high sensitivity of the triphenylacetic acid to molecular chirality is clearly illustrated with the derivative of tetrahydrofuran **15**. The probe can distinguish the difference between oxygen atom and methylene group.

Unexpectedly, the process of optical activity generation is interrupted by the presence of the second trityl group in close proximity. In these compounds, the trityl–trityl interactions could be more important for chirogenesis that the direct chirality induction from the permanent stereogenic center to the chromophore.

The field of supramolecular chemistry needs new synthons that will allowed predictable interactions, which will make it possible to control or at least predict the distribution of molecules in the crystal.^43^ The trityl group is a substituent with great potential to participate in intermolecular interactions, and our intention was to use this group as a supramolecular tool. In the studied materials, phenyl rings take part in interactions, acting as the donor and acceptor of edge-to-face interactions. It should be emphasized that both the inner and outer side of the trityl group take part in the interactions. Unfortunately, the expected (and desired) 6-fold phenyl embrace is a supramolecular synthon with high unreliability and low predictability. The conclusion from the research conducted by us and others seems not very optimistic, namely, the prediction of a material structure that is based on the structure of the trityl-containing molecule seems to be largely random. This is obviously due to the various alternative possibilities to phenyl groups interactions and, therefore, to formation of diverse aggregates that remain similar in energy.

Our study can be presented in a broader context. Despite some recent experimental findings, the cascade chirality induction, understood as a sequential induction of helicity in molecular propeller and then in a prochiral substrate, has never been a subject of in-depth studies.^5^ Demonstrating the relationship between asymmetric synthesis and dynamic induction of optical activity will confirm the universal nature of the observed phenomena.

## Experimental Section

### General Information

^1^H and ^13^C NMR spectra were recorded on Bruker Ultrashield 300 MHz or Varian VNMR-S 400 MHz instruments. Chemical shifts (δ) are reported in ppm relative to SiMe_4_. HR-MS spectra were obtained with a Bruker Impact HD, QTOF MS spectrometer. UV and ECD spectra were recorded in spectroscopic grade cyclohexane or acetonitrile using a JASCO J-810 instrument. The UV and ECD measurements were performed in quartz cell (0.5 mm path length), at a scanning speed of −50 nm min^–1^ and a resolution of 0.5 nm. The concentrations of the samples are collected in Table SI_1. FT-IR spectra were measured on a Nicolet iS 50 spectrometer using ATR module. A JASCO P-2000 polarimeter was used for optical rotation ([α]_D_) measurements (carried out at ca. 20 °C). Column chromatography was performed on J. T. Baker Silica Gel 40 μm (chromatography grade). Merck Kieselgel type 60F_254_ analytical plates were used for TLC analyses. Melting points were measured on Büchi Melting Point B-545 and uncorrected. All reagents were used as purchased from commercial suppliers. All solvents were provided by local suppliers and were purified by conventional methods prior to use.

(*R*)-Methyl 2-cyclohexyl-2-hydroxyacetate was prepared according to the literature procedure.^22h^

### General Procedure for Synthesis of the Esters of Triphenylacetic Acid

To a suspension of triphenylacetic acid (1.24 g, 4.3 mmol) in 15 mL of dry toluene containing three drops of DMF was added dropwise thionyl chloride (1.5 mL). The mixture was gently refluxed at 80 °C for 3 h using the heating mantle as the heat source. After cooling and evaporation of all volatiles under reduced pressure, the crude triphenylacetic acid chloride was used without further purification.

The esters were prepared by fusing triphenylacetic acid chloride (1 equiv) with an excess of an anhydrous alcohol (*x* equiv) at 125 °C (oil bath) by 18 h. The mixture was cooled and dissolved in CH_2_Cl_2_. To the mixture was added silica gel (ca. 100 mg), and all volatiles were removed by evaporation under reduced pressure. The crude products were purified by column chromatography on silica gel (eluent *n*-hexane/CH_2_Cl_2_).

During all experimental work, no unexpected or unusually high safety hazards were encountered.

#### (*R*)-*sec*-Butyl 2,2,2-triphenylacetate (**1**)

Scale 0.82 mmol, *x* = 3; eluent *n*-hexane/CH_2_Cl_2_ 4:1 to 2:1. Yield 168 mg (59%), white crystalline solid. Mp 96–97 °C. [α]_D_^20^ −2 (*c* 1.01, CHCl_3_). ^1^H NMR (300 MHz, CDCl_3_) δ 7.35–7.20 (m, 15H), 4.97 (h, *J* = 6.2 Hz, 1H), 1.53–1.43 (m, 2H), 1.17 (d, *J* = 6.3 Hz, 3H), 0.71 (t, *J* = 7.5 Hz, 3H). ^13^C{^1^H} NMR (75 MHz, CDCl3) δ 173.1, 143.1, 130.3, 127.6, 126.7, 74.1, 67.6, 28.5, 18.9, 9.5. ATR-IR 3055, 3028, 2968, 2928, 2879, 1716, 1489, 1443, 1211, 1186, 761, 741, 699 cm^–1^. HRMS (ESI) *m*/*z*: [M + Na]^+^ Calcd for C_24_H_24_O_2_Na 367.1669. Found 367.1675.

#### (*rac*)-*sec*-Butyl-2,2,2-triphenylacetate ((*rac*)-**1**)^22b^

The title compound was obtained from (*rac*)-2-butanol and triphenylacetyl chloride under the above-mentioned reaction conditions. Scale 1.6 mmol. Yield 372 mg (70%), white crystalline solid. The NMR spectra of product (*rac*)-**1** were the same as those described for (*R*)-**1**. HRMS (ESI) *m/z:* [M + K]^+^ Calcd for C_24_H_24_O_2_K 383.1408. Found 383.1416.

#### (*S*)-Pentan-2-yl 2,2,2-triphenylacetate (**2**)

Scale 0.62 mmol, *x* = 2; eluent *n*-hexane/CH_2_Cl_2_ 4:1 to 2:1. Yield 189 mg (85%), colorless oil. [α]_D_^20^ +5 (*c* 2.32, CHCl_3_). ^1^H NMR (300 MHz, CDCl_3_) δ 7.30–7.20 (m, 15H), 5.04 (h, *J* = 6.3 Hz, 1H), 1.51–1.26 (m, 2H), 1.18–1.04 (m, 2H), 1.17 (d, *J* = 6.3 Hz, 3H), 0.79 (t, *J* = 7.3 Hz, 3H). ^13^C{^1^H} NMR (75 MHz, CDCl_3_) δ 173.1, 143.1, 130.3, 127.6, 126.7, 72.6, 67.6, 37.7, 19.4, 18.3, 13.8. ATR-IR 3059, 3033, 2959, 2932, 2873, 1724, 1493, 1446, 1216, 1118, 741, 727, 695 cm^–1^. HRMS (ESI) *m*/*z*: [M + Na]^+^ Calcd for C_25_H_26_O_2_Na 381.1825. Found 381.1825.

#### (*S*)-Octan-3-yl 2,2,2-triphenylacetate (**3**)

Scale 1.09 mmol, *x* = 3; eluent *n*-hexane/CH_2_Cl_2_ 4:1 to 2:1. Yield 390 mg (89%), colorless oil. [α]_D_^20^ +5.5 (*c* 3.32, CHCl_3_). ^1^H NMR (300 MHz, CDCl_3_) δ 7.29–7.22 (m, 15H), 4.92 (quintet, *J* = 6.1 Hz, 1H), 1.57–1.41 (m, 4H), 1.20–1.03 (m, 6H), 0.83 (t, *J* = 6.9 Hz, 3H), 0.71 (t, *J* = 7.4 Hz, 3H).^13^C{^1^H} NMR (75 MHz, CDCl_3_) δ 173.2, 143.2, 130.4, 127.6, 126.7, 67.7, 32.8, 31.7, 26.3, 24.7, 22.5, 14.0, 9.4. ATR-IR 3059, 3033, 2955, 2931, 2859, 1723, 1493, 1447, 1217, 1186, 740, 726, 696 cm^–1^. HRMS (ESI) *m*/*z*: [M + Na]^+^ Calcd for C_28_H_32_O_2_Na 423.2295. Found 423.2309.

#### (*R*)-3-Methylbutan-2-yl-2,2,2-triphenylacetate (**4**)

Scale 0.57 mmol, *x* = 3.3; eluent *n*-hexane/CH_2_Cl_2_ 4:1 to 2:1. Yield 144 mg (71%), white amorphous solid. Mp 92–95 °C. [α]_D_^20^ +3.6 (*c* 0.84, CHCl_3_). ^1^H NMR (400 MHz, CDCl_3_) δ 7.29–7.21 (m, 15H), 4.90–4.84 (m, 1H), 1.74–1.63 (m, 1H), 1.12 (d, *J* = 6.4 Hz, 2H), 0.69 (dd, *J* = 6.8, 3.3 Hz, 6H). ^13^C{^1^H} NMR (100 MHz, CDCl_3_) δ 173.0, 143.1, 130.3, 127.6, 126.7, 67.6, 32.4, 18.0, 17.5, 16.0. ATR-IR 3057, 3020, 2961, 2934, 2873, 1715, 1489, 1443, 1209, 1185, 760, 743, 699 cm^–1^. HRMS (ESI) *m*/*z*: [M + Na]^+^ Calcd for C_25_H_26_O_2_Na 381.1825. Found 381.1830.

#### (*R*)-1-Phenylethyl-2,2,2-triphenylacetate (**5**)

Scale 1.0 mmol, *x* = 3; eluent *n*-hexane/CH_2_Cl_2_ 4:1 to 2:1; crystallized by *n*-hexane. Yield 168 mg (17%), white crystalline solid. Mp 120–122 °C. [α]_D_^20^ +14 (*c* 0.5, CHCl_3_). ^1^H NMR (400 MHz, CDCl_3_) δ 7.28–7.15 (m, 18H), 7.08–7.06 (m, 2H), 6.02 (q, *J* = 6.6 Hz, 1H), 1.46 (d, *J* = 6.6 Hz, 3H). ^13^C{^1^H} NMR (100 MHz, CDCl_3_) δ 172.5, 142.9, 141.0, 130.3, 128.2, 127.7, 127.6, 126.7, 126.2, 73.9, 67.4, 22.0. ATR-IR 3088, 3055, 3032, 2978, 2930, 1723, 1490, 1445, 1216, 1197, 760, 696 cm^–1^. HRMS (ESI) *m*/*z*: [M + Na]^+^ Calcd for C_28_H_24_O_2_Na 415.1669. Found 415.1679.

#### (*rac*)-1-Phenylethyl-2,2,2-triphenylacetate ((*rac*)-**5**)

The title compound was obtained from (*rac*)-1-phenylethanol and triphenylacetyl chloride under above-mentioned reaction conditions. Yield 116 mg (30%), white crystalline solid. The NMR spectra of the product (*rac*)-**5** were the same as described for (*R*)-**5**. HRMS (ESI) *m*/*z*: [M + K]^+^ Calcd for C_28_H_24_O_2_K 431.1408. Found 431.1422.

#### (*R*)-1-Cyclohexyl-2-methoxy-2-oxoethyl-2,2,2-triphenylacetate (**6**)

Scale 0.97 mmol, *x* = 1.3; eluent *n*-hexane/CH_2_Cl_2_ 4:1 to 2:1; separated from triphenylmethanol by column chromatography on alumina (eluent *n*-hexane/CH_2_Cl_2_ 1:1). Yield 48 mg (16%), colorless oil. [α]_D_^20^ −27 (*c* 0.95, CHCl_3_). ^1^H NMR (300 MHz, CDCl_3_) δ 7.32–7.21 (m, 15H), 4.80 (d, *J* = 4.4 Hz, 1H), 3.73 (s, 3H), 1.80–1.71 (m, 1H), 1.38–0.65 (m, 10H). ^13^C{^1^H} NMR (100 MHz, CDCl_3_) δ 173.4, 170.0, 142.7, 130.3, 127.7, 126.7, 77.9, 67.4, 52.0, 39.4, 28.7, 27.0, 25.9, 25.7. ATR-IR 3059, 3033, 2928, 2854, 1735, 1493, 1447, 1173, 743, 696 cm^–1^. HRMS (ESI) *m*/*z*: [M + Na]^+^ Calcd for C_29_H_30_O_4_Na 465.2036. Found 465.2030.

#### (*S*)-2-Methoxy-2-oxo-1-phenylethyl 2,2,2-triphenylacetate (**7**)

Scale 1 mmol, *x* = 3; eluent *n*-hexane/CH_2_Cl_2_ 4:1 to 2:1; separated from triphenylmethanol by column chromatography on alumina (eluent *n*-hexane/CH_2_Cl_2_ 1:1). Yield 66 mg (15%), colorless oil. [α]_D_^20^ +72 (*c* 1.04, CHCl_3_). ^1^H NMR (300 MHz, CDCl_3_) δ 7.31–7.17 (m, 20H), 6.01 (s, 1H), 3.71 (s, 3H). ^13^C{^1^H} NMR (100 MHz, CDCl_3_) δ 173.1, 169.1, 142.5, 133.3, 130.4, 128.9, 128.5, 127.7, 127.2, 126.9, 75.5, 67.4, 52.6. ATR-IR 3057, 3028, 2957, 1752, 1729, 1492, 1449, 1218, 1170, 740, 694 cm^–1^. HRMS (ESI) *m*/*z*: [M + Na]^+^ Calcd for C_29_H_24_O_4_Na 459.1567. Found 459.1566.

#### (*S*)-2-Methylbutyl-2,2,2-triphenylacetate (**8**)

Scale 1 mmol, *x* = 3; eluent *n*-hexane/CH_2_Cl_2_ 4:1 to 2:1. Yield 371 mg (95%), white amorphous solid. Mp 64–66 °C. [α]_D_^20^ +3.3 (*c* 1.12, CHCl_3_). ^1^H NMR (300 MHz, CDCl_3_) δ 7.31–7.17 (m, 15H), 4.04 (dq, *J* = 12.9, 6.0 Hz, 2H), 1.66–1.55 (m, 1H), 1.23–1.11 (m, 1H), 1.08–0.94 (m, 1H), 0.78 (t, *J* = 7.4 Hz, 3H), 0.72 (d, *J* = 6.8 Hz, 3H). ^13^C{^1^H} NMR (100 MHz, CDCl_3_) δ 173.7, 143.0, 130.3, 127.6, 126.8, 70.1, 67.6, 34.0, 25.8, 16.4, 11.1. ATR-IR 3063, 3034, 2955, 2928, 2875, 1727, 1493, 1444, 1223, 1198, 697 cm^–1^. HRMS (ESI) *m*/*z*: [M + Na]^+^ Calcd for C_25_H_26_O_2_Na 381.1825. Found 381.1830.

#### (3*S*)-3,7-Dimethyloct-6-en-1-yl-2,2,2-triphenylacetate (**9**)

Scale 1 mmol, *x* = 3; eluent *n*-hexane/CH_2_Cl_2_ 4:1 to 2:1. Yield 286 mg (66%) as nonseparable mixture of isomers, differing in the position of the double bond in the skeleton of the molecule; the title compound consists of the major fraction (over 90%). Colorless oil. [α]_D_^20^ −2.3 (*c* 1.79, CHCl_3_). ^1^H NMR (300 MHz, CDCl_3_) δ 7.31–7.16 (m, 15H), 5.01 (t, *J* = 6.9, 1H), 1.92–1.81 (m, 1H), 1.66 (s, 3H), 1.57 (s, 3H), 1.38–1.00 (m, 6H), 0.78 (d, *J* = 6.1 Hz, 3H). ^13^C{^1^H} NMR (101 MHz, CDCl_3_) δ 173.6, 143.0, 131.2, 130.3, 127.6, 126.8, 109.7, 67.5, 64.0, 36.8, 35.2, 29.2, 25.7, 25.3, 19.1, 17.6. ATR-IR 3059, 3022, 2961, 2925, 1729, 1493, 1446, 1212, 1184, 740, 697 cm^–1^. HRMS (ESI) *m*/*z*: [M + Na]^+^ Calcd for C_30_H_34_O_2_Na 449.2451. Found 449.2464.

#### (1*R*,2*S*,5*R*)-2-Isopropyl-5-methylcyclohexyl-2,2,2-triphenylacetate (**10**)

Scale 1.1 mmol, *x* = 1; eluent *n*-hexane/CH_2_Cl_2_ 4:1 to 1:1. Yield 262 mg (55%), white crystalline solid. Mp 101–102 °C (lit. 100–101 °C).^20^ [α]_D_^20^ −5 (*c* 1.26, CHCl_3_). ^1^H NMR (300 MHz, CDCl_3_) δ 7.30–7.19 (m, 15H), 4.74 (td, *J* = 10.9, 4.3 Hz, 1H), 2.18–2.11 (m, 1H), 1.67–1.56 (m, 2H), 1.52–1.39 (m, 1H), 1.22–1.10 (m, 2H), 1.02–0.95 (m, 2H), 0.90 (d, *J* = 6.9 Hz, 3H), 0.88–0.74 (m, 1H), 0.62 (d, *J* = 6.9 Hz, 3H), 0.53 (d, *J* = 6.9 Hz, 3H). ^13^C{^1^H} NMR (75 MHz, CDCl_3_) δ 172.9, 143.1, 130.3, 127.6, 126.6, 76.1, 67.6, 46.9, 40.3, 34.2, 31.5, 25.1, 22.7, 22.1, 15.6. ATR-IR 3091, 3064, 3036, 2946, 2932, 2869, 2850, 1725, 1494, 1447, 1214, 1194, 743, 698 cm^–1^. HRMS (ESI) *m*/*z*: [M + Na]^+^ Calcd for C_30_H_34_O_2_Na 449.2451. Found 449.2455.

#### (1*S*,2*S*,5*R*)-2-Isopropyl-5-methylcyclohexyl-2,2,2-triphenylacetate (**11**)

Scale 1.1 mmol, *x* = 1; eluent *n*-hexane/CH_2_Cl_2_ 4:1 to 2:1. Yield 411 mg (86%), white crystalline solid. Mp 103–105 °C. [α]_D_^20^ +11 (*c* 1.02, CHCl_3_). ^1^H NMR (300 MHz, CDCl_3_) δ 7.28–7.19 (m, 15H), 5.32 (bs, 1H), 1.97–1.92 (m, 1H), 1.52–1.51 (m, 1H), 1.02–0.82 (m, 5H), 0.77 (d, *J* = 5.7 Hz, 3H), 0.71 (t, *J* = 5.5 Hz, 6H). ^13^C{^1^H} NMR (75 MHz, CDCl_3_) δ 172.8, 143.1, 130.3, 127.6, 126.6, 72.8, 67.9, 47.2, 38.7, 34.7, 28.5, 26.4, 25.1, 22.1, 21.0, 20.9. ATR-IR 3059, 3023, 2965, 2954, 2914, 2882, 2849, 1714, 1494, 1443, 1226, 1215, 740, 698 cm^–1^. HRMS (ESI) *m*/*z*: [M + Na]^+^ Calcd for C_30_H_34_O_2_Na 449.2451. Found 449.2467.

#### Cholestan-3β-yl triphenylacetate (**12**)

Scale 0.74 mmol, *x* = 1; eluent *n*-hexane/CH_2_Cl_2_ 4:1 to 2:1. Yield 364 mg (78%), white amorphous solid. Mp 125–127 °C (lit. 127–129 °C).^22d^ [α]_D_^20^ +19 (*c* 0.94, CHCl_3_). ^1^H NMR (300 MHz, CDCl_3_) δ 7.35–7.17 (m, 15H), 4.86 (tt, *J* = 11.1, 5.0 Hz, 1H), 1.96–1.92 (m, 1H), 1.83–1.57 (m, 5H), 1.51–0.95 (m, 25H), 0.89 (d, *J* = 4.2 Hz, 3H), 0.87 (d, *J* = 1.3 Hz, 3H), 0.85 (d, *J* = 1.3 Hz, 3H), 0.74 (s, 3H), 0.63 (s, 3H). ^13^C{^1^H} NMR (75 MHz, CDCl_3_) δ 173.0, 143.2, 130.4, 127.6, 126.7, 75.4, 67.4, 56.4, 56.3, 54.2, 44.7, 42.6, 40.0, 39.5, 36.7, 36.2, 35.8, 35.5, 35.46, 33.6, 32.0, 28.6, 28.3, 28.0, 27.1, 24.2, 23.9, 22.6, 21.2, 12.0. ATR-IR 3059, 3023, 2930, 2865, 1727, 1493, 1467, 1445, 1214, 741, 697 cm^–1^. HRMS (ESI) *m*/*z*: [M + Na]^+^ Calcd for C_47_H_62_O_2_Na 681.4642. Found 681.4631.

#### Testosterone triphenylacetate (**13**)

Reaction temp. 165 °C, scale 0.95 mmol, *x* = 1; eluent *n*-hexane/CH_2_Cl_2_ 4:1 to 0:1. Yield 83 mg (16%), white amorphous solid. Mp 73–83 °C. [α]_D_^20^ +56 (*c* 1.6, CHCl_3_). ^1^H NMR (300 MHz, CDCl_3_) δ 7.30–7.19 (m, 15H), 5.72 (s, 1H), 4.69 (t, *J* = 8.9, 7.9 Hz, 1H), 2.41–2.17 (m, 5H), 2.03–1.96 (m, 1H), 1.85–1.38 (m, 9H), 1.14 (s, 3H), 1.07–0.83 (m, 5H), 0.43 (s, 3H). ^13^C{^1^H} NMR (100 MHz, CDCl_3_) δ 199.6, 173.5, 171.0, 143.0, 142.5, 130.3, 127.6, 126.8, 123.9, 84.0, 67.5, 53.6, 49.9, 42.5, 38.6, 36.5, 35.3, 33.9, 32.8, 31.4, 29.7, 27.0, 23.6, 20.5, 17.3. ATR-IR 3058, 3023, 2923, 2852, 1726, 1672, 1492, 1446, 1215, 1187, 744, 697 cm^–1^. HRMS (ESI) *m*/*z*: [M + Na]^+^ Calcd for C_39_H_42_O_3_Na 581.3026. Found 581.3036.

#### (*R*)-4,4-Dimethyl-2-oxotetrahydrofuran-3-yl-2,2,2-triphenylacetate (**14**)

Scale 1.1 mmol, *x* = 2.2; eluent *n*-hexane/CH_2_Cl_2_ 4:1 to 1:1. Yield 196 mg (46%), white amorphous solid. Mp 133–142 °C. [α]_D_^20^ +11 (*c* 1.02, CHCl_3_). ^1^H NMR (400 MHz, CDCl_3_) δ 7.34–7.22 (m, 15H), 5.51 (s, 1H), 3.94 (q, *J* = 9.1 Hz, 2H), 0.96 (s, 3H), 0.59 (s, 3H). ^13^C{^1^H} NMR (100 MHz, CDCl_3_) δ 172.3, 171.9, 142.4, 130.2, 127.9, 127.0, 76.2, 75.9, 67.3, 40.1, 22.6, 19.3. ATR-IR 3049, 2998, 2973, 2959, 2927, 1786, 1742, 1496, 1476, 1464, 1448, 1175, 1149, 750, 701 cm^–1^. HRMS (ESI) *m*/*z*: [M + Na]^+^ Calcd for C_26_H_24_O_4_Na 423.1567. Found 423.1549.

#### (*S*)-Tetrahydrofuran-3-yl-2,2,2-triphenylacetate (**15**)

Scale 1 mmol, *x* = 3; eluent *n*-hexane/CH_2_Cl_2_ 4:1 to 0:1. Yield 145 mg (40%), colorless oil. [α]_D_^20^ −3.1 (*c* 1.12, CHCl_3_). ^1^H NMR (300 MHz, CDCl_3_) δ 7.32–7.18 (m, 15H), 5.45–5.41 (m, 1H), 3.94–3.89 (m, 1H), 3.78–3.70 (m, 2H), 3.61–3.55 (m, 1H), 2.13–2.01 (m, 1H), 1.89–1.81 (m, 1H). ^13^C{^1^H} NMR (100 MHz, CDCl_3_) δ 173.2, 142.7, 130.2, 127.7, 126.9, 76.1, 72.6, 66.9, 32.5. ATR-IR 3054, 3023, 2924, 2870, 1723, 1489, 1445, 1205, 742, 699 cm^–1^. HRMS (ESI) *m*/*z*: [M + Na]^+^ Calcd for C_24_H_22_O_3_Na 381.1461. Found 381.1469.

#### (*R*)-1-Methylpyrrolidin-3-yl-2,2,2-triphenylacetate (**16**)

Scale 1.1 mmol, *x* = 2; eluent *n*-hexane/CH_2_Cl_2_ 4:1 to CH_2_Cl_2_/MeOH 98:2. Yield 95 mg (24%), light brown oil. [α]_D_^20^ −6 (*c* 0.83, CHCl_3_). ^1^H NMR (300 MHz, CDCl_3_) δ 7.31–7.16 (m, 15H), 5.37–5.30 (m, 1H), 2.99–2.93 (m, 1H), 2.51–2.35 (m, 3H), 2.26 (s, 1H), 2.21–2.12 (m, 1H), 1.77–1.68 (m, 1H). ^13^C{^1^H} NMR (100 MHz, CDCl_3_) δ 173.2, 142.8, 130.2, 127.7, 126.8, 75.8, 67.3, 61.3, 54.7, 42.0, 32.1. ATR-IR 3058, 3032, 2940, 2839, 2781, 1725, 1493, 1446, 1216, 1177, 742, 696 cm^–1^. HRMS (ESI) *m*/*z*: [M + Na]^+^ Calcd for C_25_H_26_NO_2_ [M + H]^+^: 372.1958. Found 372.1953.

#### (*R*)-1-(2,2,2-Triphenylacetyl)pyrrolidin-3-yl-2,2,2-triphenylacetate (**17**)

Scale 2 mmol, *x* = 0.5; eluent *n*-hexane/CH_2_Cl_2_ 4:1 to CH_2_Cl_2_/MeOH 99:1. Yield 95 mg (21%), white amorphous solid. Mp 93–103 °C. [α]_D_^20^ −81 (*c* 0.66, CHCl_3_). ^1^H NMR (400 MHz, CDCl_3_) δ 7.29–7.07 (m, 60H), 5.43–5.41 (m, 1H), 5.09 (t, *J* = 3.7 Hz, 1H), 3.89 (dd, *J* = 14.4, 5.0 Hz,H), 3.72–3.67 (m, 1H), 3.22 (dd, *J* = 11.8, 4.4 Hz, 1H), 2.93 (d, *J* = 12.9 Hz, 1H), 2.81–2.78 (m, 1H), 2.22 (dd, *J* = 12.8, 4.3 Hz, 1H), 1.98 (td, *J* = 11.1, 6.0 Hz, 1H), 1.82–1.67 (m, 3H), 1.50–1.48 (m, 1H). ^13^C{^1^H} NMR (100 MHz, CDCl_3_) δ 172.9, 172.7, 171.4, 171.2, 143.1, 142.52, 142.49, 142.4, 142.2, 130.34, 130.30, 130.2, 130.14, 130.09, 127.9, 127.73, 127.72, 127.69, 127.6, 127.1, 126.9, 126.7, 126.6, 126.5, 76.1, 72.4, 67.5, 53.3, 52.4, 45.9, 45.8, 32.0, 29.0. ATR-IR 3057, 3022, 2928, 1728, 1636, 1491, 1445, 1385, 1212, 1181, 741, 696 cm^–1^. HRMS (ESI) *m*/*z*: [M + Na]^+^ Calcd for C_44_H_37_NO_3_Na 650.2666. Found 650.2664.

#### (*R*)-Propane-1,2-diyl-bis(2,2,2-triphenylacetate) (**18**)

Scale 2 mmol, *x* = 1; eluent *n*-hexane/CH_2_Cl_2_ 4:1 to 0:1. Yield 136 mg (22%), white crystalline solid. Mp 154–156 °C. [α]_D_^20^ −5 (*c* 0.83, CHCl_3_). ^1^H NMR (400 MHz, CDCl_3_) δ 7.27–7.21 (m, 2H), 7.21–7.07 (m, 28H), 5.24–5.17 (m, 1H), 4.32 (dd, *J* = 11.9, 3.5 Hz, 1H), 4.10 (dd, *J* = 11.9, 5.2 Hz, 1H), 0.91 (d, *J* = 6.5 Hz, 3H). ^13^C{^1^H} NMR (100 MHz, CDCl_3_) δ 173.3, 172.6, 142.7, 142.6, 130.3, 130.22, 130.20, 127.8, 127.7, 127.6, 127.1, 126.9, 126.8, 69.7, 67.5, 67.4, 66.7, 15.7. ATR-IR 3059, 3022, 2999, 2924, 1735, 1724, 1488, 1444, 1209, 1181, 744, 696 cm^–1^. HRMS (ESI) *m*/*z*: [M + Na]^+^ Calcd for C_43_H_36_O_4_Na 639.2506. Found 639.2514.

#### (*R*)-Butane-1,3-diyl bis(2,2,2-triphenylacetate) (**19**)

Scale 1 mmol, *x* = 0.5; eluent *n*-hexane/CH_2_Cl_2_ 4:1 to 0:1. Yield 211 mg (67%), white crystalline solid. Mp 192–197 °C. [α]_D_^20^ −14 (*c* 1.42, CHCl_3_). ^1^H NMR (300 MHz, CDCl_3_) δ 7.30–7.14 (m, 30H), 4.83–4.72 (m, 1H), 4.01 (dt, *J* = 11.0, 5.5 Hz, 1H), 3.76 (dt, *J* = 11.3, 6.8 Hz, 1H), 1.66 (q, *J* = 5.9 Hz, 2H), 1.07 (d, *J* = 6.3 Hz, 3H). ^13^C{^1^H} NMR (100 MHz, CDCl_3_) δ 173.3, 172.6, 142.9, 130.2, 127.9, 127.72, 127.7, 127.2, 126.84, 126.75, 69.4, 67.4, 61.6, 34.3, 19.4. ATR-IR 3087, 3060, 3024, 2991, 2924, 2854, 1722, 1488, 1443, 1206, 1183, 745, 698 cm^–1^. HRMS (ESI) *m*/*z*: [M + Na]^+^ Calcd for C_44_H_38_O_4_Na 653.2662. Found 653.2657.

#### (2*R*,3*R*)-Butane-2,3-diyl-bis(2,2,2-triphenylacetate) (**20**)

Scale 1 mmol, *x* = 0.5; eluent *n*-hexane/CH_2_Cl_2_ 4:1 to 0:1. Yield 39 mg (12%), white crystalline solid. Mp 146–150 °C. [α]_D_^20^ +16 (*c* 1.1, CHCl_3_). ^1^H NMR (600 MHz, CDCl_3_) δ 7.22–7.13 (m, 15H), 5.01–4.97 (m, 1H), 0.83 (d, *J* = 6.4 Hz, 3H). ^13^C{^1^H} NMR (150 MHz, CDCl_3_) δ 172.7, 142.7, 130.3, 127.7, 126.8, 72.6, 67.5, 14.9. ATR-IR 3055, 3024, 2991, 2938, 2851, 1732, 1493, 1443, 1209, 1176, 740, 696 cm^–1^. HRMS (ESI) *m*/*z*: [M + Na]^+^ Calcd for C_44_H_38_O_4_Na 653.2662. Found 653.2652.

#### (2*R*,4*R*)-Pentane-2,4-diyl-bis(2,2,2-triphenylacetate) (**21**)

Scale 1 mmol, *x* = 0.5; eluent *n*-hexane/CH_2_Cl_2_ 4:1 to 0:1. Yield 48 mg (14%), white crystalline solid. Mp 267–270 °C. [α]_D_^20^ +12 (*c* 0.66, CHCl_3_). ^1^H NMR (400 MHz, CDCl_3_) δ 7.29–7.19 (m, 15H), 4.81–4.72 (m, 1H), 1.52–1.49 (dd, *J* = 7.3, 5.8 Hz, 1H), 1.01 (d, *J* = 6.2 Hz, 3H). ^13^C{^1^H} NMR (100 MHz, CDCl_3_) δ 172.7, 142.9, 130.2, 127.7, 126.7, 69.4, 67.5, 42.1, 19.6. ATR-IR 3088, 3059, 3027, 2983, 2924, 2853, 1720, 1492, 1444, 1207, 1185, 758, 744, 698 cm^–1^. HRMS (ESI) *m*/*z*: [M + Na]^+^ Calcd for C_45_H_40_O_4_Na 667.2819. Found 667.2819.

#### (2*R*,5*R*)-Hexane-2,5-diyl-bis(2,2,2-triphenylacetate) (**22**)

Scale 1 mmol, *x* = 0.5; eluent *n*-hexane/CH_2_Cl_2_ 4:1 to 0:1. Yield 96 mg (31%), white amorphous solid. Mp 147–149 °C. [α]_D_^20^ +11 (*c* 0.86, CHCl_3_). ^1^H NMR (300 MHz, CDCl_3_) δ 7.23–7.16 (m, 15H), 4.84 (dt, *J* = 11.4, 5.5 Hz, 1H), 1.06 (d, *J* = 6.2 Hz, 5H). ^13^C{^1^H} NMR (100 MHz, CDCl_3_) δ 172.9, 143.0, 130.2, 127.7, 126.7, 72.1, 67.4, 31.0, 19.5. ATR-IR 3059, 3025, 2969, 2926, 2855, 1720, 1489, 1444, 1212, 1199, 738, 699 cm^–1^. HRMS (ESI) *m*/*z*: [M + Na]^+^ Calcd for C_46_H_42_O_4_Na 681.2975. Found 681.2972.

## Supporting Information Available

Experimental details, calculation details, X-ray crystallography details, total energies, percentage populations and structures of all calculated low-energy conformers, calculated UV and ECD spectra, copies of UV and ECD spectra of **1**–**22** measured in cyclohexane and acetonitrile, ^1^H and ^13^C{H} NMR spectra of all synthesized new compounds, and Cartesian coordinates for all calculated structures (PDF)
FAIR data, including the primary NMR FID files, for compounds **1**–**22** (ZIP)

## Accession Codes

CCDC 2039790–2039800 contain the supplementary crystallographic data for this paper. These data can be obtained free of charge via www.ccdc.cam.ac.uk/data_request/cif, or by emailing data_request@ccdc.cam.ac.uk, or by contacting The Cambridge Crystallographic Data Centre, 12 Union Road, Cambridge CB2 1EZ, UK; fax: +44 1223 336033.

## Dedication

Dedicated to Prof. Janusz Jurczak on the occasion of his 80th birthday anniversary.

## References

[ref1] aOki M. The Chemistry of Rotational Isomers; Springer, Berlin, 1993;.

[ref2] aFinocchiaro P.; Gust D.; Mislow K. Separation of conformational stereoisomers in a triarylmethane. A Novel Type of Stereoisomerism. J. Am. Chem. Soc. 1973, 95, 8172–8173. 10.1021/ja00805a039.

[ref3] aKawada Y.; Iwamura H. Phase isomerism in gear-shaped molecules. Tetrahedron Lett. 1981, 22, 1533–1536. 10.1016/S0040-4039(01)90370-3.

[ref4] aGreene T. W.; Wats P. G. M. Protective Groups in Organic Synthesis; Wiley, New York, 2006.

[ref5] aNaidu V. R.; Ni S.; Franzén J. The Carbocation: A Forgotten Lewis Acid Catalyst. ChemCatChem 2015, 7, 1896–1905. 10.1002/cctc.201500225.

[ref6] aDattler D.; Fuks G.; Heiser J.; Moulin E.; Perrot A.; Yao X.; Giuseppone N. Design of Collective Motions from Synthetic Molecular Switches, Rotors, and Motors. Chem. Rev. 2020, 120, 310–433. 10.1021/acs.chemrev.9b00288. 31869214

[ref7] aRadwan M. O.; Ciftci H. I.; Ali T. F. S.; Koga R.; Tateishi H.; Nakata A.; Ito A.; Yoshida M.; Fujita M.; Otsuka M. Structure activity study of *S*-trityl-cysteamine dimethylaminopyridine derivatives as SIRT2 inhibitors: Improvement of SIRT2 binding and inhibition. Bioorg. Med. Chem. Lett. 2020, 30, 127458 10.1016/j.bmcl.2020.127458. 32755678

[ref8] aFleck N.; Heubach C. A.; Hett T.; Haege F. R.; Bawol P. P.; Baltruschat H.; Schiemann O. SLIM: A Short-Linked, Highly Redox-Stable Trityl Label for High-Sensitivity In-Cell EPR Distance Measurements. Angew. Chem., Int. Ed. 2020, 59, 9767–9772. 10.1002/anie.202004452. PMC7318235 32329172

[ref9] Ściebura J.; Skowronek P.; Gawroński J. Trityl Ethers: Molecular Bevel Gears Reporting Chirality through Circular Dichroism Spectra. Angew. Chem., Int. Ed. 2009, 48, 7069–7072. 10.1002/anie.200902167. 19688796

[ref10] Ściebura J.; Gawroński J. Double Chirality Transmission in Trityl Amines: Sensing Molecular Dynamic Stereochemistry by Circular Dichroism and DFT Calculations. Chem. - Eur. J. 2011, 17, 13138–13141. 10.1002/chem.201101699. 22006675

[ref11] Gawroński J.; Koput J.; Wierzbicki A. On the Induced Optical Activity of Triphenylmethane Dyes. Z. Naturforsch., A: Phys. Sci. 1986, 41a, 1245–1249. 10.1515/zna-1986-1012.

[ref12] aOzcelik A.; Pereira-Cameselle R.; Poklar Ulrih N.; Petrovic A. G.; Alonso-Gómez J. L. Chiroptical Sensing: A Conceptual Introduction. Sensors 2020, 20, 974 10.3390/s20040974. PMC7071115 32059394

[ref13] Borovkov V. V.; Hembury G. A.; Inoue Y. Origin, Control, and Application of Supramolecular Chirogenesis in Bisporphyrin-Based Systems. Acc. Chem. Res. 2004, 37, 449–459. 10.1021/ar0302437. 15260507

[ref14] aLightner D. A.; Gurst J. E. Organic Conformational Analysis and Stereochemistry from Circular Dichroism Spectroscopy; Wiley-VCH: New York, 2000.

[ref15] aGrauso L.; Teta R.; Esposito G.; Menna M.; Mangoni A. Computational prediction of chiroptical properties in structure elucidation of natural products. Nat. Prod. Rep. 2019, 36, 1005–1030. 10.1039/C9NP00018F. 31166350

[ref16] Wang L.; Zhang T.; Redden B. K.; Sheppard C. I.; Clark R. W.; Smith M. D.; Wiskur S. L. Understanding Internal Chirality Induction of Triarylsilyl Ethers Formed from Enantiopure Alcohols. J. Org. Chem. 2016, 81, 8187–8193. 10.1021/acs.joc.6b01137. 27501133

[ref17] Skowronek P.; Czapik A.; Rajska Z.; Kwit M. Molecular and supramolecular helicity induction in trityl group-containing compunds: the case of chiral 3,3,3-triphenylpropionic acid derivatives. Tetrahedron 2019, 75, 4497–4505. 10.1016/j.tet.2019.06.040.

[ref18] aBendzińska-Berus W.; Jelecki M.; Kwit M.; Rychlewska U. Transfer of chirality in *N*-triphenylacetylamino acids and chiral derivatives of *N*-triphenylacetyl Gly–Gly dipeptide and control of their assembly with steric constraints. CrystEngComm 2019, 21, 3420–3430. 10.1039/C9CE00429G.

[ref19] aPrusinowska N.; Bendzińska-Berus W.; Jelecki M.; Rychlewska U.; Kwit M. Triphenylacetic Acid Amides: Molecular Propellers with Induced Chirality. Eur. J. Org. Chem. 2015, 2015, 738–749. 10.1002/ejoc.201403182.

[ref20] Preda G.; Nitti A.; Pasini D. Chiral Triptycenes in Supramolecular and Materials Chemistry. ChemistryOpen 2020, 9, 719–727. 10.1002/open.202000077. 32547902 PMC7290281

[ref21] Tschugaeff L.; Glinin G. Über das optische Drehungsvermögen einiger aktiver Triphenyl-essigester. Ber. Dtsch. Chem. Ges. 1912, 45, 2759–2764. 10.1002/cber.191204502187.

[ref22] aRule H. G.; Bain J. CCXLVI.—Optical activity and the polarity of substituent groups. Part XV. Phenyl-substituted esters and ethers of l-menthol and β-octyl alcohol. J. Chem. Soc. 1930, 0, 1894–1903. 10.1039/JR9300001894.

[ref23] Weber E., Ed. Molecular Inclusion and Molecular Recognition: Clathrates I; Topics in Current Chemistry Series; Springer: Berlin, 1987; Vol. 140.

[ref24] Akazome M. Chiral Recognition by Inclusion Crystals of Amino-Acid Derivatives Having Trityl Groups. In Advances in Organic Crystal Chemistry; Tamura R., Miyata M., Eds.; Springer: Berlin, 2015; pp 463.

[ref25] aMegumi K.; Nadiah Binti Mohd Arif F.; Matsumoto S.; Akazome M. Design and Evaluation of Salts between *N*-Trityl Amino Acid and *tert*-Butylamine as Inclusion Crystals of Alcohols. Cryst. Growth Des. 2012, 12, 5680–5685. 10.1021/cg301172g.

[ref26] Bendzińska-Berus W.; Warżajtis B.; Gajewy J.; Kwit M.; Rychlewska U. Trityl Group as an Crystal Engineering Tool for Construction of Inclusion Compounds and for Suppression of Amide NH···O=C Hydrogen Bonds. Cryst. Growth Des. 2017, 17, 2560–2568. 10.1021/acs.cgd.7b00105.

[ref27] Czapik A.; Jelecki M.; Kwit M. Chiral Cocrystal Solid Solutions, Molecular Complexes, and Salts of *N*-Triphenylacetyl-l-Tyrosine and Diamines. Int. J. Mol. Sci. 2019, 20, 5004 10.3390/ijms20205004. PMC6829379 31658607

[ref28] aHosseini M. W. Molecular Tectonics: From Simple Tectons to Complex Molecular Networks. Acc. Chem. Res. 2005, 38, 313–323. 10.1021/ar0401799. 15835878

[ref29] Duong A.; Lévesque A.; Homand C.; Maris T.; Wuest J. D. Controlling Molecular Organization by Using Phenyl Embraces of Multiple Trityl Groups. J. Org. Chem. 2020, 85, 4026–4035. 10.1021/acs.joc.9b02974. 32070093

[ref30] aLiptrot D. J.; Power P. P. London dispersion forces in sterically crowded inorganic and organometallic molecules. Nat. Chem. Rev. 2017, 1, 0004 10.1038/s41570-016-0004.

[ref31] aLacour J.; Bernardinelli G.; Russell V.; Dance I. Crystal Packing Interpretation of the Association of Chiral Threefold Propeller Ions: TRISPHAT Anion with a Triarylcarbenium Cation. CrystEngComm 2002, 4, 165–170. 10.1039/b202524h.

[ref32] aMaddox J. Crystals from First Principles. Nature 1988, 335, 201 10.1038/335201a0.

[ref33] Sasaki T.; Ida Y.; Yuge T.; Yamamoto A.; Hisaki I.; Tohnai N.; Miyata M. Chirality Generation in Supramolecular Clusters: Analogues of Octacoordinated Polyhedrons. Cryst. Growth Des. 2015, 15, 658–665. 10.1021/cg5013445.

[ref34] Groom C. R.; Bruno I. J.; Lightfoot M. P.; Ward S. C. The Cambridge Structural Database. Acta Crystallogr., Sect. B: Struct. Sci., Cryst. Eng. Mater. 2016, 72, 171–179. 10.1107/S2052520616003954. PMC4822653 27048719

[ref35] Frisch M. J.; Trucks G. W.; Schlegel H. B.; Scuseria G. E.; Robb M. A.; Cheeseman J. R.; Scalmani G.; Barone V.; Mennucci B.; Petersson G. A.; Nakatsuji H.; Caricato M.; Li X.; Hratchian H. P.; Izmaylov A. F.; Bloino J.; Zheng G.; Sonnenberg J. L.; Hada M.; Ehara M.; Toyota K.; Fukuda R.; Hasegawa J.; Ishida M.; Nakajima T.; Honda Y.; Kitao O.; Nakai H.; Vreven T.; Montgomery J. A. Jr.; Peralta J. E.; Ogliaro F.; Bearpark M.; Heyd J. J.; Brothers E.; Kudin K. N.; Staroverov V. N.; Kobayashi R.; Normand J.; Raghavachari K.; Rendell A.; Burant J. C.; Iyengar S. S.; Tomasi J.; Cossi M.; Rega N.; Millam J. M.; Klene M.; Knox J. E.; Cross J. B.; Bakken V.; Adamo C.; Jaramillo J.; Gomperts R.; Stratmann R. E.; Yazyev O.; Austin A. J.; Cammi R.; Pomelli C.; Ochterski J. W.; Martin R. L.; Morokuma K.; Zakrzewski V. G.; Voth G. A.; Salvador P.; Dannenberg J. J.; Dapprich S.; Daniels A. D.; Farkas O.; Foresman J. B.; Ortiz J. V.; Cioslowski J.; Fox D. J. Gaussian 09, revision D.01; Gaussian, Inc.: Wallingford, CT, 2009.

[ref36] Kwit M.; Rozwadowska M. D.; Gawroński J.; Grajewska A. Density Functional Theory Calculations of the Optical Rotation and Electronic Circular Dichroism: The Absolute Configuration of the Highly Flexible *trans*-Isocytoxazone Revised. J. Org. Chem. 2009, 74, 8051–8063. 10.1021/jo901175s. 19817355

[ref37] aBecke A. D. Density-functional thermochemistry. III. The role of exact exchange. J. Chem. Phys. 1993, 98, 5648–5652. 10.1063/1.464913.

[ref38] aZhao Y.; Truhlar D. G. The M06 suite of density functionals for main group thermochemistry, thermochemical kinetics, noncovalent interactions, excited states, and transition elements: two new functionals and systematic testing of four M06-class functionals and 12 other functionals. Theor. Chem. Acc. 2008, 120, 215–241. 10.1007/s00214-007-0310-x.

[ref39] aGrimme S.; Ehrlich S.; Goerigk L. Effect of the damping function in dispersion corrected density functional theory. J. Comput. Chem. 2011, 32, 1456–1465. 10.1002/jcc.21759. 21370243

[ref40] aEliel E. L.; Wilen S. H. Stereochemistry of Organic Compounds; John Wiley and Sons Inc.: New York, 1994.

[ref41] Bruhn T.; Schaumlöffel A.; Hemberger Y.; Pescitelli G. SpecDis version 1.71; Berlin, Germany, 2017. https:/specdis-software.jimdo.com.

[ref42] The ECD spectrum measured for **21** exhibited a very low signal-to-noise ratio. Unfortunately, despite many attempts, we were not able to register an ECD spectrum of a better quality.

[ref43] Atwood J. L., Gokel G. W., Barbour L. J., Eds. Comprehensive Supramolecular Chemistry II; Elsevier: Amsterdam, 2017.

