# Supplementary material for: Chiral Triphenylacetic Acid Esters: Residual Stereoisomerism and Solid-State Variability of Molecular Architectures
Source: J Org Chem. 2021 Apr 28;86(9):6433–48. doi: 10.1021/acs.joc.1c00279 (PMC8279475; doi:10.1021/acs.joc.1c00279)
Supplement: Supplementary file 1 — jo1c00279_si_001.pdf [file jo1c00279_si_001.pdf]

Supporting Information for:

Chiral triphenylacetic acid esters – residual stereoisomerism and solid-state variability of molecular architectures

Natalia Prusinowska[a,b], Agnieszka Czapik[a] and Marcin Kwit[a,b]*

a) Faculty of Chemistry, Adam Mickiewicz University, Uniwersytetu Poznańskiego 8, 61 614 Poznań, Poland. E-mail: marcin.kwit@amu.edu.pl

b) Centre for Advanced Technologies, Adam Mickiewicz University, Uniwersytetu Poznańskiego 10, 61 614 Poznań

Table of contents:

Calculations details.. S19

Table SI_1. Concentrations (c, in mol L^{-1}) of the samples used for UV and ECD measurements. S21

Table SI_2. ECD ($\Delta\varepsilon$, in $dm^3{\cdot}mol^{-1}{\cdot}cm^{-1}$) and UV ($\varepsilon$, in $dm^3{\cdot}mol^{-1}{\cdot}cm^{-1}$) data for derivatives **1**-**22** measured in acetonitrile solution. S22

Table SI_3. Total and free energies (E, ΔG, in Hartree), relative energies (ΔE, $\Delta\Delta G$ in kcal mol^{-1}), percentage populations and number of imaginary frequencies (#ImFreq) calculated at the B3LYP/6-311++G(d,p) level for individual conformers of **1**. S23

Table SI_4. Total and free energies (E, ΔG, in Hartree), relative energies (ΔE, $\Delta\Delta G$ in kcal mol^{-1}), percentage populations and number of imaginary frequencies (#ImFreq) calculated at the M06-2X/6-311++G(d,p) level for individual conformers of **1**. S24

Table SI_5. Total and free energies (E, ΔG, in Hartree), relative energies (ΔE, $\Delta\Delta G$ in kcal mol^{-1}), percentage populations and number of imaginary frequencies (#ImFreq) calculated at the B3LYP/6-311++G(d,p) level for individual conformers of **4**. S25

Table SI_6. Total and free energies (E, ΔG, in Hartree), relative energies (ΔE, $\Delta\Delta G$ in kcal mol^{-1}), percentage populations and number of imaginary frequencies (#ImFreq) calculated at the M06-2X/6-311++G(d,p) level for individual conformers of **4**. S25

Table SI_7. Total and free energies (E, ΔG, in Hartree), relative energies (ΔE, $\Delta\Delta G$ in kcal mol^{-1}), percentage populations and number of imaginary frequencies (#ImFreq) calculated at the B3LYP/6-311++G(d,p) level for individual conformers of **6**. S26

Table SI_8. Total and free energies (E, ΔG, in Hartree), relative energies (ΔE, $\Delta\Delta G$ in kcal mol^{-1}), percentage populations and number of imaginary frequencies (#ImFreq) calculated at the M06-2X/6-311++G(d,p) level for individual conformers of **6**. S26

Table SI_9. Total and free energies (E, ΔG, in Hartree), relative energies (ΔE, $\Delta\Delta G$ in kcal mol^{-1}), percentage populations and number of imaginary frequencies (#ImFreq) calculated at the B3LYP/6-311++G(d,p) level for individual conformers of **10**. S27

Table SI_10. Total and free energies (E, ΔG, in Hartree), relative energies (ΔE, $\Delta\Delta G$ in kcal mol^{-1}), percentage populations and number of imaginary frequencies (#ImFreq) calculated at the M06-2X/6-311++G(d,p) level for individual conformers of **10**. S27

Table SI_11. Total and free energies (E, ΔG, in Hartree), relative energies (ΔE, $\Delta\Delta G$ in kcal mol^{-1}), percentage populations and number of imaginary frequencies (#ImFreq) calculated at the B3LYP/6-311++G(d,p) level for individual conformers of **11**. S27

Table SI_12. Total and free energies (E, ΔG, in Hartree), relative energies (ΔE, $\Delta\Delta G$ in kcal mol^{-1}), percentage populations and number of imaginary frequencies (#ImFreq) calculated at the M06-2X/6-311++G(d,p) level for individual conformers of **11**. S28

Table SI_13. Total and free energies (E, ΔG, in Hartree), relative energies (ΔE, $\Delta\Delta G$ in kcal mol^{-1}), percentage populations and number of imaginary frequencies (#ImFreq) calculated at the B3LYP/6-311++G(d,p) level for individual conformers of **14**. S28

Table SI_14. Total and free energies (E, ΔG, in Hartree), relative energies (ΔE, $\Delta\Delta G$ in kcal mol^{-1}), percentage populations and number of imaginary frequencies (#ImFreq) calculated at the M06-2X/6-311++G(d,p) level for individual conformers of **14**. S28

Table SI_15. Total and free energies (E, ΔG, in Hartree), relative energies (ΔE, $\Delta\Delta G$ in kcal mol^{-1}), percentage populations and number of imaginary frequencies (#ImFreq) calculated at the B3LYP/6-311++G(d,p) level for individual conformers of **15**. S29

Table SI_16. Total and free energies (E, ΔG, in Hartree), relative energies (ΔE, $\Delta\Delta G$ in kcal mol^{-1}), percentage populations and number of imaginary frequencies (#ImFreq) calculated at the M06-2X/6-311++G(d,p) level for individual conformers of **15**. S30

Table SI_17. Total and free energies (E, ΔG, in Hartree), relative energies (ΔE, $\Delta\Delta G$ in kcal mol^{-1}), percentage populations and number of imaginary frequencies (#ImFreq) calculated at the B3LYP/6-311G(d,p) level for individual conformers of **18**. S31

Table SI_18. Total and free energies (E, ΔG, in Hartree), relative energies (ΔE, $\Delta\Delta G$ in kcal mol^{-1}), percentage populations and number of imaginary frequencies (#ImFreq) calculated at the B3LYP-GD3BJ/6-311G(d,p) level for individual conformers of **18**. S32

Table SI_19. Total and free energies (E, ΔG, in Hartree), relative energies (ΔE, $\Delta\Delta G$ in kcal mol^{-1}), percentage populations and number of imaginary frequencies (#ImFreq) calculated at the M06-2X/6-311G(d,p) level for individual conformers of **18**. S32

Table SI_20. Total and free energies (E, ΔG, in Hartree), relative energies (ΔE, $\Delta\Delta G$ in kcal mol^{-1}), percentage populations and number of imaginary frequencies (#ImFreq) calculated at the B3LYP/6-311G(d,p) level for individual conformers of **20**. S33

Table SI_21. Total and free energies (E, ΔG, in Hartree), relative energies (ΔE, $\Delta\Delta G$ in kcal mol^{-1}), percentage populations and number of imaginary frequencies (#ImFreq) calculated at the B3LYP-GD3BJ/6-311G(d,p) level for individual conformers of **20**. S33

Table SI_22. Total and free energies (E, ΔG, in Hartree), relative energies (ΔE, $\Delta\Delta G$ in kcal mol^{-1}), percentage populations and number of imaginary frequencies (#ImFreq) calculated at the M06-2X/6-311G(d,p) level for individual conformers of **20**. S34

Table SI_23. Total and free energies (E, ΔG, in Hartree), relative energies (ΔE, $\Delta\Delta G$ in kcal mol^{-1}), percentage populations and number of imaginary frequencies (#ImFreq) calculated at the B3LYP/6-311G(d,p) level for individual conformers of **21**. S34

Table SI_24. Total and free energies (E, ΔG, in Hartree), relative energies (ΔE, $\Delta\Delta G$ in kcal mol^{-1}), percentage populations and number of imaginary frequencies (#ImFreq) calculated at the B3LYP-GD3BJ/6-311G(d,p) level for individual conformers of **21**. S34

Table SI_25. Total and free energies (E, ΔG, in Hartree), relative energies (ΔE, $\Delta\Delta G$ in kcal mol^{-1}), percentage populations and number of imaginary frequencies (#ImFreq) calculated at the M06-2X/6-311G(d,p) level for individual conformers of **21**. S35

Table SI_26. Total and free energies (E, ΔG, in Hartree), relative energies (ΔE, $\Delta\Delta G$ in kcal mol^{-1}), percentage populations and number of imaginary frequencies (#ImFreq) calculated at the B3LYP/6-311G(d,p) level for individual conformers of **22**. S35

Table SI_27. Total and free energies (E, ΔG, in Hartree), relative energies (ΔE, $\Delta\Delta G$ in kcal mol^{-1}), percentage populations and number of imaginary frequencies (#ImFreq) calculated at the B3LYP-GD3BJ/6-311G(d,p) level for individual conformers of **22**. S36

Table SI_28. Total and free energies (E, ΔG, in Hartree), relative energies (ΔE, $\Delta\Delta G$ in kcal mol^{-1}), percentage populations and number of imaginary frequencies (#ImFreq) calculated at the M06-2X/6-311G(d,p) level for individual conformers of **22**. S36

Table SI_29. Dihedral angles α, β, γ, δ and ζ (in degrees) and selected interatomic distances l_1, l_2 (in Å) calculated at the B3LYP/6-311++G(d,p) level for individual low-energy conformers of **1**......................... S37

Table SI_30. Dihedral angles α, β, γ, δ and ζ (in degrees) and selected interatomic distances l_1, l_2 (in Å) calculated at the M06-2X/6-311++G(d,p) level for individual low-energy conformers of **1**...................... S38

Table SI_31. Dihedral angles α, β, γ, δ and ζ (in degrees) and selected interatomic distances l_1, l_2 (in Å) calculated at the B3LYP/6-311++G(d,p) level for individual low-energy conformers of **4**......................... S39

Table SI_32. Dihedral angles α, β, γ, δ and ζ (in degrees) and selected interatomic distances l_1, l_2 (in Å) calculated at the M06-2X/6-311++G(d,p) level for individual low-energy conformers of **4**...................... S39

Table SI_33. Dihedral angles α, β, γ, δ and ζ (in degrees) and selected interatomic distances l_1, l_2 (in Å) calculated at the B3LYP/6-311++G(d,p) level for individual low-energy conformers of **6**......................... S40

Table SI_34. Dihedral angles α, β, γ, δ and ζ (in degrees) and selected interatomic distances $l_1$and l_2 (in Å) calculated at the M06-2X/6-311++G(d,p) level for individual low-energy conformers of **6**...................... S41

Table SI_35. Dihedral angles α, β, γ, δ and ζ (in degrees) and selected interatomic distances l_1, l_2 (in Å) calculated at the DFT/6-311++G(d,p) level for individual low-energy conformers of **10**. S41

Table SI_36. Dihedral angles α, β, γ, δ and ζ (in degrees) and selected interatomic distances l_1, l_2 (in Å) calculated at the B3LYP/6-311++G(d,p) level for individual low-energy conformers of **11**....................... S42

Table SI_37. Dihedral angles α, β, γ, δ and ζ (in degrees) and selected interatomic distances l_1, l_2 (in Å) calculated at the M06-2X/6-311++G(d,p) level for individual low-energy conformers of **11**.................... S43

Table SI_38. Dihedral angles α, β, γ, δ, ζ and φ (in degrees) and selected interatomic distances l_1, l_2 and l_3 (in Å) calculated at the B3LYP/6-311++G(d,p) level for individual low-energy conformers of **14**. S44

Table SI_39. Dihedral angles α, β, γ, δ, ζ and φ (in degrees) and selected interatomic distances l_1, l_2 and l_3 (in Å) calculated at the M06-2X/6-311++G(d,p) level for individual low-energy conformers of **14**. S44

Table SI_40. Dihedral angles α, β, γ, δ, ζ and φ (in degrees) and selected interatomic distances l_1, l_2 and l_3 (in Å) calculated at the B3LYP/6-311++G(d,p) level for individual low-energy conformers of **15**. S45

Table SI_41. Dihedral angles α, β, γ, δ, ζ and φ (in degrees) and selected interatomic distances l_1, l_2 and l_3 (in Å) calculated at the M06-2X/6-311++G(d,p) level for individual low-energy conformers of **15**. S46

Table SI_42. Dihedral angles α, β, γ, δ, ζ and φ (in degrees) and selected interatomic distances l_1, l_2 and l_3 (in Å) calculated at the B3LYP/6-311G(d,p) level for individual low-energy conformers of **18**. S47

Table SI_43. Dihedral angles α, β, γ, δ, ζ and φ (in degrees) and selected interatomic distances l_1, l_2 and l_3 (in Å) calculated at the B3LYP-GD3Bj/6-311G(d,p) level for individual low-energy conformers of **18**....... S49

Table SI_44. Dihedral angles α, β, γ, δ, ζ and φ (in degrees) and selected interatomic distances l_1, l_2 and l_3 (in Å) calculated at the M06-2X/6-311G(d,p) level for individual low-energy conformers of **18**. S50

Table SI_45. Dihedral angles α, β, γ, δ, ζ and φ (in degrees) and selected interatomic distances l_1, l_2 and l_3 (in Å) calculated at the B3LYP/6-311G(d,p) level for individual low-energy conformers of **20**. S51

Table SI_46. Dihedral angles α, β, γ, δ, ζ and φ (in degrees) and selected interatomic distances l_1, l_2 and l_3 (in Å) calculated at the B3LYP-GD3BJ/6-311G(d,p) level for individual low-energy conformers of **20**. S53

Table SI_47. Dihedral angles α, β, γ, δ, ζ and φ (in degrees) and selected interatomic distances l_1, l_2 and l_3 (in Å) calculated at the M06-2X/6-311G(d,p) level for individual low-energy conformers of **20**. S54

Table SI_48. Dihedral angles α, β, γ, δ, ζ and φ (in degrees) and selected interatomic distances l_1, l_2 and l_3 (in Å) calculated at the B3LYP/6-311G(d,p) level for individual low-energy conformers of **21**. S55

Table SI_49. Dihedral angles α, β, γ, δ, ζ and φ (in degrees) and selected interatomic distances l_1, l_2 and l_3 (in Å) calculated at the B3LYP-GD3BJ/6-311G(d,p) level for individual low-energy conformers of **21**. S56

Table SI_50. Dihedral angles α, β, γ, δ, ζ and φ (in degrees) and selected interatomic distances l_1, l_2 and l_3 (in Å) calculated at the M06/6-311G(d,p) level for individual low-energy conformers of **21**.................... S57

Table SI_51. Dihedral angles α, β, γ, δ, ζ and φ (in degrees) and selected interatomic distances l_1, l_2 and l_3 (in Å) calculated at the B3LYP/6-311G(d,p) level for individual low-energy conformers of **22**. S58

Table SI_52. Dihedral angles α, β, γ, δ, ζ and φ (in degrees) and selected interatomic distances l_1, l_2 and l_3 (in Å) calculated at the B3LYP-GD3BJ/6-311G(d,p) level for individual low-energy conformers of **22**. S60

Table SI_53. Dihedral angles α, β, γ, δ, ζ and φ (in degrees) and selected interatomic distances l_1, l_2 and l_3 (in Å) calculated at the M06-2X/6-311G(d,p) level for individual low-energy conformers of **22**. S61

Figure SI_1. Structures of individual, low-energy conformers of **1**, calculated at the B3LYP/6-311++G(d,p) level of theory. S62

Figure SI_2. Structures of individual, low-energy conformers of **1**, calculated at the M06-2X/6-311++G(d,p) level of theory. S63

Figure SI_3. Structures of individual, low-energy conformers of **4**, calculated at the B3LYP/6-311++G(d,p) level of theory. S64

Figure SI_4. Structures of individual, low-energy conformers of **4**, calculated at the M06-2X/6-311++G(d,p) level of theory. S64

Figure SI_5. Structures of individual, low-energy conformers of **6**, calculated at the B3LYP/6-311++G(d,p) level of theory. S65

Figure SI_6. Structures of individual, low-energy conformers of **6**, calculated at the M06-2X/6-311++G(d,p) level of theory. S66

Figure SI_7. Structure of individual, low-energy conformer of **10**, calculated at the B3LYP/6-311++G(d,p) level of theory. S67

Figure SI_8. Structure of individual, low-energy conformer of **10**, calculated at the M06-2X/6-311++G(d,p) level of theory. S67

Figure SI_9. Structures of individual, low-energy conformers of **11**, calculated at the B3LYP/6-311++G(d,p) level of theory. S68

Figure SI_10. Structures of individual, low-energy conformers of **11**, calculated at the M06-2X/6-311++G(d,p) level of theory. S69

Figure SI_11. Structures of individual, low-energy conformers of **14**, calculated at the B3LYP/6-311++G(d,p) level of theory. S70

Figure SI_12. Structures of individual, low-energy conformers of **14**, calculated at the M06-2X/6-311++G(d,p) level of theory. S70

Figure SI_13. Structures of individual, low-energy conformers of **15**, calculated at the B3LYP/6-311++G(d,p) level of theory. S71

Figure SI_14. Structures of individual, low-energy conformers of **15**, calculated at the M06-2X/6-311++G(d,p) level of theory. S72

Figure SI_15a. Structures of individual, low-energy conformers of **18**, calculated at the B3LYP/6-311G(d,p) level of theory, part I. S73

Figure SI_15b. Structures of individual, low-energy conformers of **18**, calculated at the B3LYP/6-311G(d,p) level of theory, part II. S74

Figure SI_16. Structures of individual, low-energy conformers of **11**, calculated at the B3LYP-GD3BJ/6-311G(d,p) level of theory. S75

Figure SI_17. Structures of individual, low-energy conformers of **18**, calculated at the M06-2X/6-311G(d,p) level of theory. S76

Figure SI_18. Structures of individual, low-energy conformers of **20**, calculated at the B3LYP/6-311G(d,p) level of theory. S77

Figure SI_19. Structures of individual, low-energy conformers of **20**, calculated at the B3LYP-GD3BJ/6-311G(d,p) level of theory. S78

Figure SI_20. Structures of individual, low-energy conformers of **20**, calculated at the M06-2X/6-311G(d,p) level of theory. S79

Figure SI_21. Structures of individual, low-energy conformers of **21**, calculated at the B3LYP/6-311G(d,p) level of theory. S80

Figure SI_22. Structures of individual, low-energy conformers of **21**, calculated at the B3LYP-GD3BJ/6-311G(d,p) level of theory. S80

Figure SI_23. Structures of individual, low-energy conformers of **21**, calculated at the M06-2X/6-311G(d,p) level of theory. S81

Figure SI_24a. Structures of individual, low-energy conformers of **22**, calculated at the B3LYP/6-311G(d,p) level of theory, part I. S82

Figure SI_24b. Structures of individual, low-energy conformers of **22**, calculated at the B3LYP/6-311G(d,p) level of theory, part II. S83

Figure SI_25. Structures of individual, low-energy conformers of **22**, calculated at the B3LYP-GD3BJ/6-311G(d,p) level of theory. S83

Figure SI_26. Structures of individual, low-energy conformers of **22**, calculated at the M06-2X/6-311G(d,p) level of theory. S84

Figure SI_27. UV and ECD spectra of the low-energy conformers of compound **1** calculated at TD-CAM-B3LYP/6-311++G(2d,2p) level for structures optimized at B3LYP/6-311++G(d,p) level. Wavelengths were not corrected. S85

Figure SI_28. UV and ECD spectra of the low-energy conformers of compound **1** calculated at TD-M06-2X/6-311++G(2d,2p) level for structures optimized at B3LYP/6-311++G(d,p) level. Wavelengths were not corrected. S86

Figure SI_29. UV and ECD spectra of the low-energy conformers of compound **1** calculated at TD-CAM-B3LYP/6-311++G(2d,2p) level for structures optimized at M06-2X/6-311++G(d,p) level. Wavelengths were not corrected. S87

Figure SI_30. UV and ECD spectra of the low-energy conformers of compound **1** calculated at TD-M06-2X/6-311++G(2d,2p) level for structures optimized at M06-2X/6-311++G(d,p) level. Wavelengths were not corrected. S88

Figure SI_31. UV and ECD spectra of the low-energy conformers of compound **4** calculated at TD-CAM-B3LYP/6-311++G(2d,2p) level for structures optimized at B3LYP/6-311++G(d,p) level. Wavelengths were not corrected. S89

Figure SI_32. UV and ECD spectra of the low-energy conformers of compound **4** calculated at TD-M06-2X/6-311++G(2d,2p) level for structures optimized at B3LYP/6-311++G(d,p) level. Wavelengths were not corrected. S89

Figure SI_33. UV and ECD spectra of the low-energy conformers of compound **4** calculated at TD-CAM-B3LYP/6-311++G(2d,2p) level for structures optimized at M06-2X/6-311++G(d,p) level. Wavelengths were not corrected. S90

Figure SI_34. UV and ECD spectra of the low-energy conformers of compound **4** calculated at TD-M06-2X/6-311++G(2d,2p) level for structures optimized at M06-2X/6-311++G(d,p) level. Wavelengths were not corrected. S90

Figure SI_35. UV and ECD spectra of the low-energy conformers of compound **6** calculated at TD-CAM-B3LYP/6-311++G(2d,2p) level for structures optimized at B3LYP/6-311++G(d,p) level. Wavelengths were not corrected. S91

Figure SI_36. UV and ECD spectra of the low-energy conformers of compound **6** calculated at TD-M06-2X/6-311++G(2d,2p) level for structures optimized at B3LYP/6-311++G(d,p) level. Wavelengths were not corrected. S92

Figure SI_37. UV and ECD spectra of the low-energy conformers of compound **6** calculated at TD-CAM-B3LYP/6-311++G(2d,2p) level for structures optimized at M06-2X/6-311++G(d,p) level. Wavelengths were not corrected. S93

Figure SI_38. UV and ECD spectra of the low-energy conformers of compound **6** calculated at TD-M06-2X/6-311++G(2d,2p) level for structures optimized at M06-2X/6-311++G(d,p) level. Wavelengths were not corrected. S93

Figure SI_39. UV and ECD spectra of the low-energy conformers of compound **10** calculated at TD-CAM-B3LYP/6-311++G(2d,2p) level for structures optimized at B3LYP/6-311++G(d,p) level. Wavelengths were not corrected. S94

Figure SI_40. UV and ECD spectra of the low-energy conformers of compound **10** calculated at TD-M06-2X/6-311++G(2d,2p) level for structures optimized at B3LYP/6-311++G(d,p) level. Wavelengths were not corrected. S94

Figure SI_41. UV and ECD spectra of the low-energy conformers of compound **10** calculated at TD-CAM-B3LYP/6-311++G(2d,2p) level for structures optimized at M06-2X/6-311++G(d,p) level. Wavelengths were not corrected. S95

Figure SI_42. UV and ECD spectra of the low-energy conformers of compound **10** calculated at TD-M06-2X/6-311++G(2d,2p) level for structures optimized at M06-2X/6-311++G(d,p) level. Wavelengths were not corrected. S95

Figure SI_43. UV and ECD spectra of the low-energy conformers of compound **11** calculated at TD-CAM-B3LYP/6-311++G(2d,2p) level for structures optimized at B3LYP/6-311++G(d,p) level. Wavelengths were not corrected. S96

Figure SI_44. UV and ECD spectra of the low-energy conformers of compound **11** calculated at TD-M06-2X/6-311++G(2d,2p) level for structures optimized at B3LYP/6-311++G(d,p) level. Wavelengths were not corrected. S96

Figure SI_45. UV and ECD spectra of the low-energy conformers of compound **11** calculated at TD-CAM-B3LYP/6-311++G(2d,2p) level for structures optimized at M06-2X/6-311++G(d,p) level. Wavelengths were not corrected. S97

Figure SI_46. UV and ECD spectra of the low-energy conformers of compound **11** calculated at TD-M06-2X/6-311++G(2d,2p) level for structures optimized at M06-2X/6-311++G(d,p) level. Wavelengths were not corrected. S97

Figure SI_47. UV and ECD spectra of the low-energy conformers of compound **14** calculated at TD-CAM-B3LYP/6-311++G(2d,2p) level for structures optimized at B3LYP/6-311++G(d,p) level. Wavelengths were not corrected. S98

Figure SI_48. UV and ECD spectra of the low-energy conformers of compound **14** calculated at TD-M06-2X/6-311++G(2d,2p) level for structures optimized at B3LYP/6-311++G(d,p) level. Wavelengths were not corrected. S98

Figure SI_49. UV and ECD spectra of the low-energy conformers of compound **14** calculated at TD-CAM-B3LYP/6-311++G(2d,2p) level for structures optimized at M06-2X/6-311++G(d,p) level. Wavelengths were not corrected. S99

Figure SI_50. UV and ECD spectra of the low-energy conformers of compound **14** calculated at TD-M06-2X/6-311++G(2d,2p) level for structures optimized at M06-2X/6-311++G(d,p) level. Wavelengths were not corrected. S99

Figure SI_51. UV and ECD spectra of the low-energy conformers of compound **15** calculated at TD-CAM-B3LYP/6-311++G(2d,2p) level for structures optimized at B3LYP/6-311++G(d,p) level. Wavelengths were not corrected. S100

Figure SI_52. UV and ECD spectra of the low-energy conformers of compound **15** calculated at TD-M06-2X/6-311++G(2d,2p) level for structures optimized at B3LYP/6-311++G(d,p) level. Wavelengths were not corrected. S100

Figure SI_53. UV and ECD spectra of the low-energy conformers of compound **15** calculated at TD-CAM-B3LYP/6-311++G(2d,2p) level for structures optimized at M06-2X/6-311++G(d,p) level. Wavelengths were not corrected. S101

Figure SI_54. UV and ECD spectra of the low-energy conformers of compound **15** calculated at TD-M06-2X/6-311++G(2d,2p) level for structures optimized at M06-2X/6-311++G(d,p) level. Wavelengths were not corrected. S101

Figure SI_55. UV and ECD spectra of the low-energy conformers of compound **18** calculated at TD-CAM-B3LYP/6-311++G(d,p) level for structures optimized at B3LYP/6-311G(d,p) level. Wavelengths were not corrected. S103

Figure SI_56. UV and ECD spectra of the low-energy conformers of compound **18** calculated at TD-M06-2X/6-311++G(d,p) level for structures optimized at B3LYP/6-311G(d,p) level. Wavelengths were not corrected. S105

Figure SI_57. UV and ECD spectra of the low-energy conformers of compound **18** calculated at TD-CAM-B3LYP/6-311++G(d,p) level for structures optimized at B3LYP-GD3BJ/6-311G(d,p) level. Wavelengths were not corrected. S106

Figure SI_58. UV and ECD spectra of the low-energy conformers of compound **18** calculated at TD-M06-2X/6-311++G(d,p) level for structures optimized at B3LYP-GD3BJ/6-311G(d,p) level. Wavelengths were not corrected. S107

Figure SI_59. UV and ECD spectra of the low-energy conformers of compound **18** calculated at TD-CAM-B3LYP/6-311++G(d,p) level for structures optimized at M06-2X/6-311G(d,p) level. Wavelengths were not corrected. S108

Figure SI_60. UV and ECD spectra of the low-energy conformers of compound **18** calculated at TD-M06-2X/6-311++G(d,p) level for structures optimized at M06-2X/6-311G(d,p) level. Wavelengths were not corrected. S109

Figure SI_61. UV and ECD spectra of the low-energy conformers of compound **20** calculated at TD-CAM-B3LYP/6-311++G(d,p) level for structures optimized at B3LYP/6-311G(d,p) level. Wavelengths were not corrected. S110

Figure SI_62. UV and ECD spectra of the low-energy conformers of compound **20** calculated at TD-M06-2X/6-311++G(d,p) level for structures optimized at B3LYP/6-311G(d,p) level. Wavelengths were not corrected. S111

Figure SI_63. UV and ECD spectra of the low-energy conformers of compound **20** calculated at TD-CAM-B3LYP/6-311++G(d,p) level for structures optimized at B3LYP-GD3BJ/6-311G(d,p) level. Wavelengths were not corrected. S112

Figure SI_64. UV and ECD spectra of the low-energy conformers of compound **20** calculated at TD-M06-2X/6-311++G(d,p) level for structures optimized at B3LYP-GD3BJ/6-311G(d,p) level. Wavelengths were not corrected. S112

Figure SI_65. UV and ECD spectra of the low-energy conformers of compound **20** calculated at TD-CAM-B3LYP/6-311++G(d,p) level for structures optimized at M06-2X/6-311G(d,p) level. Wavelengths were not corrected. S113

Figure SI_66. UV and ECD spectra of the low-energy conformers of compound **20** calculated at TD-M06-2X/6-311++G(d,p) level for structures optimized at M06-2X/6-311G(d,p) level. Wavelengths were not corrected. S113

Figure SI_67. UV and ECD spectra of the low-energy conformers of compound **21** calculated at TD-CAM-B3LYP/6-311++G(d,p) level for structures optimized at B3LYP/6-311G(d,p) level. Wavelengths were not corrected. S114

Figure SI_68. UV and ECD spectra of the low-energy conformers of compound **21** calculated at TD-M06-2X/6-311++G(d,p) level for structures optimized at B3LYP/6-311G(d,p) level. Wavelengths were not corrected. S114

Figure SI_69. UV and ECD spectra of the low-energy conformers of compound **21** calculated at TD-CAM-B3LYP/6-311++G(d,p) level for structures optimized at B3LYP-GD3BJ/6-311G(d,p) level. Wavelengths were not corrected. S115

Figure SI_70. UV and ECD spectra of the low-energy conformers of compound **21** calculated at TD-M06-2X/6-311++G(d,p) level for structures optimized at B3LYP-GD3BJ/6-311G(d,p) level. Wavelengths were not corrected. S115

Figure SI_71. UV and ECD spectra of the low-energy conformers of compound **21** calculated at TD-CAM-B3LYP/6-311++G(d,p) level for structures optimized at M06-2X/6-311G(d,p) level. Wavelengths were not corrected. S116

Figure SI_72. UV and ECD spectra of the low-energy conformers of compound **21** calculated at TD-M06-2X/6-311++G(d,p) level for structures optimized at M06-2X/6-311G(d,p) level. Wavelengths were not corrected. S116

Figure SI_73. UV and ECD spectra of the low-energy conformers of compound **22** calculated at TD-CAM-B3LYP/6-311++G(d,p) level for structures optimized at B3LYP/6-311G(d,p) level. Wavelengths were not corrected. S117

Figure SI_74. UV and ECD spectra of the low-energy conformers of compound **22** calculated at TD-M06-2X/6-311++G(d,p) level for structures optimized at B3LYP/6-311G(d,p) level. Wavelengths were not corrected. S118

Figure SI_75. UV and ECD spectra of the low-energy conformers of compound **22** calculated at TD-CAM-B3LYP/6-311++G(d,p) level for structures optimized at B3LYP-GD3BJ/6-311G(d,p) level. Wavelengths were not corrected. S119

Figure SI_76. UV and ECD spectra of the low-energy conformers of compound **22** calculated at TD-M06-2X/6-311++G(d,p) level for structures optimized at B3LYP-GD3BJ/6-311G(d,p) level. Wavelengths were not corrected. S119

Figure SI_77. UV and ECD spectra of the low-energy conformers of compound **22** calculated at TD-CAM-B3LYP/6-311++G(d,p) level for structures optimized at M06-2X/6-311G(d,p) level. Wavelengths were not corrected. S120

Figure SI_78. UV and ECD spectra of the low-energy conformers of compound **22** calculated at TD-M06-2X/6-311++G(d,p) level for structures optimized at M06-2X/6-311G(d,p) level. Wavelengths were not corrected. S120

Figure SI_79. UV (upper panel) and ECD (lower panel) spectra of **1**, experimental, measured in cyclohexane (solid black lines) and calculated at the TD-CAM-B3LYP/6-311++G(2d,2p) level for structures optimized at the B3LYP/6-311++G(d,p) level, ΔE-based Boltzmann (red lines) and ΔΔG-based Boltzmann averaged (blue solid lines). Insert shows the comparison between Boltzmann averaged ECD spectrum and that calculated for ΔΔG-based on the lowest energy conformer of a given compound (dashed blue line). All calculated spectra were wavelength corrected to match experimental UV maxima. S121

Figure SI_80. UV (upper panel) and ECD (lower panel) spectra of **1**, experimental, measured in cyclohexane (solid black lines) and calculated at the TD-M06-2X/6-311++G(2d,2p) level for structures optimized at the B3LYP/6-311++G(d,p) level, ΔE-based Boltzmann (red lines) and ΔΔG-based Boltzmann averaged (blue solid lines). Insert shows the comparison between Boltzmann averaged ECD spectrum and that calculated for ΔΔG-based on the lowest energy conformer of a given compound (dashed blue line). All calculated spectra were wavelength corrected to match experimental UV maxima. S122

Figure SI_81. UV (upper panel) and ECD (lower panel) spectra of **1**, experimental, measured in cyclohexane (solid black lines) and calculated at the TD-CAM-B3LYP/6-311++G(2d,2p) level for structures optimized at the M06-2X/6-311++G(d,p) level, ΔE-based Boltzmann (red lines) and ΔΔG-based Boltzmann averaged (blue solid lines). Insert shows the comparison between Boltzmann averaged ECD spectrum and that calculated for ΔΔG-based on the lowest energy conformer of a given compound (dashed blue line). All calculated spectra were wavelength corrected to match experimental UV maxima. S123

Figure SI_82. UV (upper panel) and ECD (lower panel) spectra of **1**, experimental, measured in cyclohexane (solid black lines) and calculated at the TD-M06-2X/6-311++G(2d,2p) level for structures optimized at the M06-2X/6-311++G(d,p) level, ΔE-based Boltzmann (red lines) and ΔΔG-based Boltzmann averaged (blue solid lines). Insert shows the comparison between Boltzmann averaged ECD spectrum and that calculated for ΔΔG-based on the lowest energy conformer of a given compound (dashed blue line). All calculated spectra were wavelength corrected to match experimental UV maxima. S124

Figure SI_83. UV (upper panel) and ECD (lower panel) spectra of **4**, experimental, measured in cyclohexane (solid black lines) and calculated at the TD-CAM-B3LYP/6-311++G(2d,2p) level for structures optimized at the B3LYP/6-311++G(d,p) level, ΔE-based Boltzmann (red lines) and ΔΔG-based Boltzmann averaged (blue solid lines). Insert shows the comparison between Boltzmann averaged ECD spectrum and

that calculated for ΔΔG-based on the lowest energy conformer of a given compound (dashed blue line). All calculated spectra were wavelength corrected to match experimental UV maxima. S125

Figure SI_84. UV (upper panel) and ECD (lower panel) spectra of **4**, experimental, measured in cyclohexane (solid black lines) and calculated at the TD-M06-2X/6-311++G(2d,2p) level for structures optimized at the B3LYP/6-311++G(d,p) level, ΔE-based Boltzmann (red lines) and ΔΔG-based Boltzmann averaged (blue solid lines). Insert shows the comparison between Boltzmann averaged ECD spectrum and that calculated for ΔΔG-based on the lowest energy conformer of a given compound (dashed blue line). All calculated spectra were wavelength corrected to match experimental UV maxima. S126

Figure SI_85. UV (upper panel) and ECD (lower panel) spectra of **4**, experimental, measured in cyclohexane (solid black lines) and calculated at the TD-CAM-B3LYP/6-311++G(2d,2p) level for structures optimized at the M06-2X/6-311++G(d,p) level, ΔE-based Boltzmann (red lines) and ΔΔG-based Boltzmann averaged (blue solid lines). Insert shows the comparison between Boltzmann averaged ECD spectrum and that calculated for ΔΔG-based on the lowest energy conformer of a given compound (dashed blue line). All calculated spectra were wavelength corrected to match experimental UV maxima. S127

Figure SI_86. UV (upper panel) and ECD (lower panel) spectra of **4**, experimental, measured in cyclohexane (solid black lines) and calculated at the TD-M06-2X/6-311++G(2d,2p) level for structures optimized at the M06-2X/6-311++G(d,p) level, ΔE-based Boltzmann (red lines) and ΔΔG-based Boltzmann averaged (blue solid lines). Insert shows the comparison between Boltzmann averaged ECD spectrum and that calculated for ΔΔG-based on the lowest energy conformer of a given compound (dashed blue line). All calculated spectra were wavelength corrected to match experimental UV maxima. S128

Figure SI_87. UV (upper panel) and ECD (lower panel) spectra of **6**, experimental, measured in cyclohexane (solid black lines) and calculated at the TD-CAM-B3LYP/6-311++G(2d,2p) level for structures optimized at the B3LYP/6-311++G(d,p) level, ΔE-based Boltzmann (red lines) and ΔΔG-based Boltzmann averaged (blue solid lines). Insert shows the comparison between Boltzmann averaged ECD spectrum and that calculated for ΔΔG-based on the lowest energy conformer of a given compound (dashed blue line). All calculated spectra were wavelength corrected to match experimental UV maxima. S129

Figure SI_88. UV (upper panel) and ECD (lower panel) spectra of **6**, experimental, measured in cyclohexane (solid black lines) and calculated at the TD-M06-2X/6-311++G(2d,2p) level for structures optimized at the B3LYP/6-311++G(d,p) level, ΔE-based Boltzmann (red lines) and ΔΔG-based Boltzmann averaged (blue solid lines). Insert shows the comparison between Boltzmann averaged ECD spectrum and that calculated for ΔΔG-based on the lowest energy conformer of a given compound (dashed blue line). All calculated spectra were wavelength corrected to match experimental UV maxima. S130

Figure SI_89. UV (upper panel) and ECD (lower panel) spectra of **6**, experimental, measured in cyclohexane (solid black lines) and calculated at the TD-CAM-B3LYP/6-311++G(2d,2p) level for structures optimized at the M06-2X/6-311++G(d,p) level, ΔE-based Boltzmann (red lines) and ΔΔG-based Boltzmann averaged (blue solid lines). Insert shows the comparison between Boltzmann averaged ECD spectrum and that calculated for ΔΔG-based on the lowest energy conformer of a given compound (dashed blue line). All calculated spectra were wavelength corrected to match experimental UV maxima. S131

Figure SI_90. UV (upper panel) and ECD (lower panel) spectra of **6**, experimental, measured in cyclohexane (solid black lines) and calculated at the TD-M06-2X/6-311++G(2d,2p) level for structures optimized at the M06-2X/6-311++G(d,p) level, ΔE-based Boltzmann (red lines) and ΔΔG-based Boltzmann averaged (blue solid lines). Insert shows the comparison between Boltzmann averaged ECD spectrum and that calculated for ΔΔG-based on the lowest energy conformer of a given compound (dashed blue line). All calculated spectra were wavelength corrected to match experimental UV maxima. S132

Figure SI_91. UV (upper panel) and ECD (lower panel) spectra of **10**, experimental, measured in cyclohexane (solid black lines) and calculated at the TD-CAM-B3LYP/6-311++G(2d,2p) level for structures optimized at the B3LYP/6-311++G(d,p) level, ΔE-based Boltzmann (red lines) and ΔΔG-based Boltzmann averaged (blue solid lines). Insert shows the comparison between Boltzmann averaged ECD spectrum and that calculated for ΔΔG-based on the lowest energy conformer of a given compound (dashed blue line). All calculated spectra were wavelength corrected to match experimental UV maxima. S133

Figure SI_92. UV (upper panel) and ECD (lower panel) spectra of **10**, experimental, measured in cyclohexane (solid black lines) and calculated at the TD-M06-2X/6-311++G(2d,2p) level for structures optimized at the B3LYP/6-311++G(d,p) level, ΔE-based Boltzmann (red lines) and ΔΔG-based Boltzmann averaged (blue solid lines). Insert shows the comparison between Boltzmann averaged ECD spectrum and that calculated for ΔΔG-based on the lowest energy conformer of a given compound (dashed blue line). All calculated spectra were wavelength corrected to match experimental UV maxima. S134

Figure SI_93. UV (upper panel) and ECD (lower panel) spectra of **10**, experimental, measured in cyclohexane (solid black lines) and calculated at the TD-CAM-B3LYP/6-311++G(2d,2p) level for structures optimized at the M06-2X/6-311++G(d,p) level, ΔE-based Boltzmann (red lines) and ΔΔG-based Boltzmann averaged (blue solid lines). Insert shows the comparison between Boltzmann averaged ECD spectrum and that calculated for ΔΔG-based on the lowest energy conformer of a given compound (dashed blue line). All calculated spectra were wavelength corrected to match experimental UV maxima. S135

Figure SI_94. UV (upper panel) and ECD (lower panel) spectra of **10**, experimental, measured in cyclohexane (solid black lines) and calculated at the TD-M06-2X/6-311++G(2d,2p) level for structures optimized at the M06-2X/6-311++G(d,p) level, ΔE-based Boltzmann (red lines) and ΔΔG-based Boltzmann averaged (blue solid lines). Insert shows the comparison between Boltzmann averaged ECD spectrum and that calculated for ΔΔG-based on the lowest energy conformer of a given compound (dashed blue line). All calculated spectra were wavelength corrected to match experimental UV maxima. S136

Figure SI_95. UV (upper panel) and ECD (lower panel) spectra of **11**, experimental, measured in cyclohexane (solid black lines) and calculated at the TD-CAM-B3LYP/6-311++G(2d,2p) level for structures optimized at the B3LYP/6-311++G(d,p) level, ΔE-based Boltzmann (red lines) and ΔΔG-based Boltzmann averaged (blue solid lines). Insert shows the comparison between Boltzmann averaged ECD spectrum and that calculated for ΔΔG-based on the lowest energy conformer of a given compound (dashed blue line). All calculated spectra were wavelength corrected to match experimental UV maxima. S137

Figure SI_96. UV (upper panel) and ECD (lower panel) spectra of **11**, experimental, measured in cyclohexane (solid black lines) and calculated at the TD-M06-2X/6-311++G(2d,2p) level for structures optimized at the B3LYP/6-311++G(d,p) level, ΔE-based Boltzmann (red lines) and ΔΔG-based Boltzmann averaged (blue solid lines). Insert shows the comparison between Boltzmann averaged ECD spectrum and that calculated for ΔΔG-based on the lowest energy conformer of a given compound (dashed blue line). All calculated spectra were wavelength corrected to match experimental UV maxima. S138

Figure SI_97. UV (upper panel) and ECD (lower panel) spectra of **11**, experimental, measured in cyclohexane (solid black lines) and calculated at the TD-CAM-B3LYP/6-311++G(2d,2p) level for structures optimized at the M06-2X/6-311++G(d,p) level, ΔE-based Boltzmann (red lines) and ΔΔG-based Boltzmann averaged (blue solid lines). Insert shows the comparison between Boltzmann averaged ECD spectrum and that calculated for ΔΔG-based on the lowest energy conformer of a given compound (dashed blue line). All calculated spectra were wavelength corrected to match experimental UV maxima. S139

Figure SI_98. UV (upper panel) and ECD (lower panel) spectra of **11**, experimental, measured in cyclohexane (solid black lines) and calculated at the TD-M06-2X/6-311++G(2d,2p) level for structures optimized at the M06-2X/6-311++G(d,p) level, ΔE-based Boltzmann (red lines) and ΔΔG-based Boltzmann

averaged (blue solid lines). Insert shows the comparison between Boltzmann averaged ECD spectrum and that calculated for ΔΔG-based on the lowest energy conformer of a given compound (dashed blue line). All calculated spectra were wavelength corrected to match experimental UV maxima. S140

Figure SI_99. UV (upper panel) and ECD (lower panel) spectra of **14**, experimental, measured in cyclohexane (solid black lines) and calculated at the TD-CAM-B3LYP/6-311++G(2d,2p) level for structures optimized at the B3LYP/6-311++G(d,p) level, ΔE-based Boltzmann (red lines) and ΔΔG-based Boltzmann averaged (blue solid lines). Insert shows the comparison between Boltzmann averaged ECD spectrum and that calculated for ΔΔG-based on the lowest energy conformer of a given compound (dashed blue line). All calculated spectra were wavelength corrected to match experimental UV maxima. S141

Figure SI_100. UV (upper panel) and ECD (lower panel) spectra of **14**, experimental, measured in cyclohexane (solid black lines) and calculated at the TD-M06-2X/6-311++G(2d,2p) level for structures optimized at the B3LYP/6-311++G(d,p) level, ΔE-based Boltzmann (red lines) and ΔΔG-based Boltzmann averaged (blue solid lines). Insert shows the comparison between Boltzmann averaged ECD spectrum and that calculated for ΔΔG-based on the lowest energy conformer of a given compound (dashed blue line). All calculated spectra were wavelength corrected to match experimental UV maxima. S142

Figure SI_101. UV (upper panel) and ECD (lower panel) spectra of **14**, experimental, measured in cyclohexane (solid black lines) and calculated at the TD-CAM-B3LYP/6-311++G(2d,2p) level for structures optimized at the M06-2X/6-311++G(d,p) level, ΔE-based Boltzmann (red lines) and ΔΔG-based Boltzmann averaged (blue solid lines). Insert shows the comparison between Boltzmann averaged ECD spectrum and that calculated for ΔΔG-based on the lowest energy conformer of a given compound (dashed blue line). All calculated spectra were wavelength corrected to match experimental UV maxima. S143

Figure SI_102. UV (upper panel) and ECD (lower panel) spectra of **14**, experimental, measured in cyclohexane (solid black lines) and calculated at the TD-M06-2X/6-311++G(2d,2p) level for structures optimized at the M06-2X/6-311++G(d,p) level, ΔE-based Boltzmann (red lines) and ΔΔG-based Boltzmann averaged (blue solid lines). Insert shows the comparison between Boltzmann averaged ECD spectrum and that calculated for ΔΔG-based on the lowest energy conformer of a given compound (dashed blue line). All calculated spectra were wavelength corrected to match experimental UV maxima. S144

Figure SI_103. UV (upper panel) and ECD (lower panel) spectra of **15**, experimental, measured in cyclohexane (solid black lines) and calculated at the TD-CAM-B3LYP/6-311++G(2d,2p) level for structures optimized at the B3LYP/6-311++G(d,p) level, ΔE-based Boltzmann (red lines) and ΔΔG-based Boltzmann averaged (blue solid lines). Insert shows the comparison between Boltzmann averaged ECD spectrum and that calculated for ΔΔG-based on the lowest energy conformer of a given compound (dashed blue line). All calculated spectra were wavelength corrected to match experimental UV maxima. S145

Figure SI_104. UV (upper panel) and ECD (lower panel) spectra of **15**, experimental, measured in cyclohexane (solid black lines) and calculated at the TD-M06-2X/6-311++G(2d,2p) level for structures optimized at the B3LYP/6-311++G(d,p) level, ΔE-based Boltzmann (red lines) and ΔΔG-based Boltzmann averaged (blue solid lines). Insert shows the comparison between Boltzmann averaged ECD spectrum and that calculated for ΔΔG-based on the lowest energy conformer of a given compound (dashed blue line). All calculated spectra were wavelength corrected to match experimental UV maxima. S146

Figure SI_105. UV (upper panel) and ECD (lower panel) spectra of **15**, experimental, measured in cyclohexane (solid black lines) and calculated at the TD-CAM-B3LYP/6-311++G(2d,2p) level for structures optimized at the M06-2X/6-311++G(d,p) level, ΔE-based Boltzmann (red lines) and ΔΔG-based Boltzmann averaged (blue solid lines). Insert shows the comparison between Boltzmann averaged ECD spectrum and that calculated for ΔΔG-based on the lowest energy conformer of a given compound (dashed blue line). All calculated spectra were wavelength corrected to match experimental UV maxima. S147

Figure SI_106. UV (upper panel) and ECD (lower panel) spectra of **15**, experimental, measured in cyclohexane (solid black lines) and calculated at the TD-M06-2X/6-311++G(2d,2p) level for structures optimized at the M06-2X/6-311++G(d,p) level, ΔE-based Boltzmann (red lines) and ΔΔG-based Boltzmann averaged (blue solid lines). Insert shows the comparison between Boltzmann averaged ECD spectrum and that calculated for ΔΔG-based on the lowest energy conformer of a given compound (dashed blue line). All calculated spectra were wavelength corrected to match experimental UV maxima. S148

Figure SI_107. UV (upper panel) and ECD (lower panel) spectra of **18**, experimental, measured in cyclohexane (solid black lines) and calculated at the TD-CAM-B3LYP/6-311++G(d,p) level for structures optimized at the B3LYP/6-311G(d,p) level, ΔE-based Boltzmann (red lines) and ΔΔG-based Boltzmann averaged (blue solid lines). Insert shows the comparison between Boltzmann averaged ECD spectrum and that calculated for ΔΔG-based on the lowest energy conformer of a given compound (dashed blue line). All calculated spectra were wavelength corrected to match experimental UV maxima. S149

Figure SI_108. UV (upper panel) and ECD (lower panel) spectra of **18**, experimental, measured in cyclohexane (solid black lines) and calculated at the TD-M06-2X/6-311++G(d,p) level for structures optimized at the B3LYP/6-311G(d,p) level, ΔE-based Boltzmann (red lines) and ΔΔG-based Boltzmann averaged (blue solid lines). Insert shows the comparison between Boltzmann averaged ECD spectrum and that calculated for ΔΔG-based on the lowest energy conformer of a given compound (dashed blue line). All calculated spectra were wavelength corrected to match experimental UV maxima. S150

Figure SI_109. UV (upper panel) and ECD (lower panel) spectra of **18**, experimental, measured in cyclohexane (solid black lines) and calculated at the TD-CAM-B3LYP/6-311++G(d,p) level for structures optimized at the B3LYP-GD3BJ/6-311G(d,p) level, ΔE-based Boltzmann (red lines) and ΔΔG-based Boltzmann averaged (blue solid lines). Insert shows the comparison between Boltzmann averaged ECD spectrum and that calculated for ΔΔG-based on the lowest energy conformer of a given compound (dashed blue line). All calculated spectra were wavelength corrected to match experimental UV maxima. S151

Figure SI_110. UV (upper panel) and ECD (lower panel) spectra of **18**, experimental, measured in cyclohexane (solid black lines) and calculated at the TD-M06-2X/6-311++G(d,p) level for structures optimized at the B3LYP-GD3BJ/6-311G(d,p) level, ΔE-based Boltzmann (red lines) and ΔΔG-based Boltzmann averaged (blue solid lines). Insert shows the comparison between Boltzmann averaged ECD spectrum and that calculated for ΔΔG-based on the lowest energy conformer of a given compound (dashed blue line). All calculated spectra were wavelength corrected to match experimental UV maxima. S152

Figure SI_111. UV (upper panel) and ECD (lower panel) spectra of **18**, experimental, measured in cyclohexane (solid black lines) and calculated at the TD-CAM-B3LYP/6-311++G(d,p) level for structures optimized at the M06-2X/6-311G(d,p) level, ΔE-based Boltzmann (red lines) and ΔΔG-based Boltzmann averaged (blue solid lines). Insert shows the comparison between Boltzmann averaged ECD spectrum and that calculated for ΔΔG-based on the lowest energy conformer of a given compound (dashed blue line). All calculated spectra were wavelength corrected to match experimental UV maxima. S153

Figure SI_112. UV (upper panel) and ECD (lower panel) spectra of **18**, experimental, measured in cyclohexane (solid black lines) and calculated at the TD-M06-2X/6-311++G(d,p) level for structures optimized at the M06-2X/6-311G(d,p) level, ΔE-based Boltzmann (red lines) and ΔΔG-based Boltzmann averaged (blue solid lines). Insert shows the comparison between Boltzmann averaged ECD spectrum and that calculated for ΔΔG-based on the lowest energy conformer of a given compound (dashed blue line). All calculated spectra were wavelength corrected to match experimental UV maxima. S154

Figure SI_113. UV (upper panel) and ECD (lower panel) spectra of **20**, experimental, measured in cyclohexane (solid black lines) and calculated at the TD-CAM-B3LYP/6-311++G(d,p) level for structures optimized at the B3LYP/6-311G(d,p) level, ΔE-based Boltzmann (red lines) and ΔΔG-based Boltzmann averaged (blue solid lines). Insert shows the comparison between Boltzmann averaged ECD spectrum and that calculated for ΔΔG-based on the lowest energy conformer of a given compound (dashed blue line). All calculated spectra were wavelength corrected to match experimental UV maxima. S155

Figure SI_114. UV (upper panel) and ECD (lower panel) spectra of **20**, experimental, measured in cyclohexane (solid black lines) and calculated at the TD-M06-2X/6-311++G(d,p) level for structures optimized at the B3LYP/6-311G(d,p) level, ΔE-based Boltzmann (red lines) and ΔΔG-based Boltzmann averaged (blue solid lines). Insert shows the comparison between Boltzmann averaged ECD spectrum and that calculated for ΔΔG-based on the lowest energy conformer of a given compound (dashed blue line). All calculated spectra were wavelength corrected to match experimental UV maxima. S156

Figure SI_115. UV (upper panel) and ECD (lower panel) spectra of **20**, experimental, measured in cyclohexane (solid black lines) and calculated at the TD-CAM-B3LYP/6-311++G(d,p) level for structures optimized at the B3LYP-GD3BJ/6-311G(d,p) level, ΔE-based Boltzmann (red lines) and ΔΔG-based Boltzmann averaged (blue solid lines). Insert shows the comparison between Boltzmann averaged ECD spectrum and that calculated for ΔΔG-based on the lowest energy conformer of a given compound (dashed blue line). All calculated spectra were wavelength corrected to match experimental UV maxima. S157

Figure SI_116. UV (upper panel) and ECD (lower panel) spectra of **20**, experimental, measured in cyclohexane (solid black lines) and calculated at the TD-M06-2X/6-311++G(d,p) level for structures optimized at the B3LYP-GD3BJ/6-311G(d,p) level, ΔE-based Boltzmann (red lines) and ΔΔG-based Boltzmann averaged (blue solid lines). Insert shows the comparison between Boltzmann averaged ECD spectrum and that calculated for ΔΔG-based on the lowest energy conformer of a given compound (dashed blue line). All calculated spectra were wavelength corrected to match experimental UV maxima. S158

Figure SI_117. UV (upper panel) and ECD (lower panel) spectra of **20**, experimental, measured in cyclohexane (solid black lines) and calculated at the TD-CAM-B3LYP/6-311++G(d,p) level for structures optimized at the M06-2X/6-311G(d,p) level, ΔE-based Boltzmann (red lines) and ΔΔG-based Boltzmann averaged (blue solid lines). Insert shows the comparison between Boltzmann averaged ECD spectrum and that calculated for ΔΔG-based on the lowest energy conformer of a given compound (dashed blue line). All calculated spectra were wavelength corrected to match experimental UV maxima. S159

Figure SI_118. UV (upper panel) and ECD (lower panel) spectra of **20**, experimental, measured in cyclohexane (solid black lines) and calculated at the TD-M06-2X/6-311++G(d,p) level for structures optimized at the M06-2X/6-311G(d,p) level, ΔE-based Boltzmann (red lines) and ΔΔG-based Boltzmann averaged (blue solid lines). Insert shows the comparison between Boltzmann averaged ECD spectrum and that calculated for ΔΔG-based on the lowest energy conformer of a given compound (dashed blue line). All calculated spectra were wavelength corrected to match experimental UV maxima. S160

Figure SI_119. UV (upper panel) and ECD (lower panel) spectra of **21**, experimental, measured in cyclohexane (solid black lines) and calculated at the TD-CAM-B3LYP/6-311++G(d,p) level for structures optimized at the B3LYP/6-311G(d,p) level, ΔE-based Boltzmann (red lines) and ΔΔG-based Boltzmann averaged (blue solid lines). Insert shows the comparison between Boltzmann averaged ECD spectrum and that calculated for ΔΔG-based on the lowest energy conformer of a given compound (dashed blue line). All calculated spectra were wavelength corrected to match experimental UV maxima. S161

Figure SI_120. UV (upper panel) and ECD (lower panel) spectra of **21**, experimental, measured in cyclohexane (solid black lines) and calculated at the TD-M06-2X/6-311++G(d,p) level for structures optimized at the B3LYP/6-311G(d,p) level, ΔE-based Boltzmann (red lines) and ΔΔG-based Boltzmann averaged (blue solid lines). Insert shows the comparison between Boltzmann averaged ECD spectrum and that calculated for ΔΔG-based on the lowest energy conformer of a given compound (dashed blue line). All calculated spectra were wavelength corrected to match experimental UV maxima. S162

Figure SI_121. UV (upper panel) and ECD (lower panel) spectra of **21**, experimental, measured in cyclohexane (solid black lines) and calculated at the TD-CAM-B3LYP/6-311++G(d,p) level for structures optimized at the B3LYP-GD3BJ/6-311G(d,p) level, ΔE-based Boltzmann (red lines) and ΔΔG-based Boltzmann averaged (blue solid lines). Insert shows the comparison between Boltzmann averaged ECD spectrum and that calculated for ΔΔG-based on the lowest energy conformer of a given compound (dashed blue line). All calculated spectra were wavelength corrected to match experimental UV maxima. S163

Figure SI_122. UV (upper panel) and ECD (lower panel) spectra of **21**, experimental, measured in cyclohexane (solid black lines) and calculated at the TD-M06-2X/6-311++G(d,p) level for structures optimized at the B3LYP-GD3BJ/6-311G(d,p) level, ΔE-based Boltzmann (red lines) and ΔΔG-based Boltzmann averaged (blue solid lines). Insert shows the comparison between Boltzmann averaged ECD spectrum and that calculated for ΔΔG-based on the lowest energy conformer of a given compound (dashed blue line). All calculated spectra were wavelength corrected to match experimental UV maxima. S164

Figure SI_123. UV (upper panel) and ECD (lower panel) spectra of **21**, experimental, measured in cyclohexane (solid black lines) and calculated at the TD-CAM-B3LYP/6-311++G(d,p) level for structures optimized at the M06-2X/6-311G(d,p) level, ΔE-based Boltzmann (red lines) and ΔΔG-based Boltzmann averaged (blue solid lines). Insert shows the comparison between Boltzmann averaged ECD spectrum and that calculated for ΔΔG-based on the lowest energy conformer of a given compound (dashed blue line). All calculated spectra were wavelength corrected to match experimental UV maxima. S165

Figure SI_124. UV (upper panel) and ECD (lower panel) spectra of **21**, experimental, measured in cyclohexane (solid black lines) and calculated at the TD-M06-2X/6-311++G(d,p) level for structures optimized at the M06-2X/6-311G(d,p) level, ΔE-based Boltzmann (red lines) and ΔΔG-based Boltzmann averaged (blue solid lines). Insert shows the comparison between Boltzmann averaged ECD spectrum and that calculated for ΔΔG-based on the lowest energy conformer of a given compound (dashed blue line). All calculated spectra were wavelength corrected to match experimental UV maxima. S166

Figure SI_125. UV (upper panel) and ECD (lower panel) spectra of **22**, experimental, measured in cyclohexane (solid black lines) and calculated at the TD-CAM-B3LYP/6-311++G(d,p) level for structures optimized at the B3LYP/6-311G(d,p) level, ΔE-based Boltzmann (red lines) and ΔΔG-based Boltzmann averaged (blue solid lines). Insert shows the comparison between Boltzmann averaged ECD spectrum and that calculated for ΔΔG-based on the lowest energy conformer of a given compound (dashed blue line). All calculated spectra were wavelength corrected to match experimental UV maxima. S167

Figure SI_126. UV (upper panel) and ECD (lower panel) spectra of **22**, experimental, measured in cyclohexane (solid black lines) and calculated at the TD-M06-2X/6-311++G(d,p) level for structures optimized at the B3LYP/6-311G(d,p) level, ΔE-based Boltzmann (red lines) and ΔΔG-based Boltzmann averaged (blue solid lines). Insert shows the comparison between Boltzmann averaged ECD spectrum and that calculated for ΔΔG-based on the lowest energy conformer of a given compound (dashed blue line). All calculated spectra were wavelength corrected to match experimental UV maxima. S168

Figure SI_127. UV (upper panel) and ECD (lower panel) spectra of **22**, experimental, measured in cyclohexane (solid black lines) and calculated at the TD-CAM-B3LYP/6-311++G(d,p) level for structures optimized at the B3LYP-GD3BJ/6-311G(d,p) level, ΔE-based Boltzmann (red lines) and ΔΔG-based Boltzmann averaged (blue solid lines). Insert shows the comparison between Boltzmann averaged ECD spectrum and that calculated for ΔΔG-based on the lowest energy conformer of a given compound (dashed blue line). All calculated spectra were wavelength corrected to match experimental UV maxima. S169

Figure SI_128. UV (upper panel) and ECD (lower panel) spectra of **22**, experimental, measured in cyclohexane (solid black lines) and calculated at the TD-M06-2X/6-311++G(d,p) level for structures optimized at the B3LYP-GD3BJ/6-311G(d,p) level, ΔE-based Boltzmann (red lines) and ΔΔG-based Boltzmann averaged (blue solid lines). Insert shows the comparison between Boltzmann averaged ECD spectrum and that calculated for ΔΔG-based on the lowest energy conformer of a given compound (dashed blue line). All calculated spectra were wavelength corrected to match experimental UV maxima. S170

Figure SI_129. UV (upper panel) and ECD (lower panel) spectra of **22**, experimental, measured in cyclohexane (solid black lines) and calculated at the TD-CAM-B3LYP/6-311++G(d,p) level for structures optimized at the M06-2X/6-311G(d,p) level, ΔE-based Boltzmann (red lines) and ΔΔG-based Boltzmann averaged (blue solid lines). Insert shows the comparison between Boltzmann averaged ECD spectrum and that calculated for ΔΔG-based on the lowest energy conformer of a given compound (dashed blue line). All calculated spectra were wavelength corrected to match experimental UV maxima. S171

Figure SI_130. UV (upper panel) and ECD (lower panel) spectra of **22**, experimental, measured in cyclohexane (solid black lines) and calculated at the TD-M06-2X/6-311++G(d,p) level for structures optimized at the M06-2X/6-311G(d,p) level, ΔE-based Boltzmann (red lines) and ΔΔG-based Boltzmann averaged (blue solid lines). Insert shows the comparison between Boltzmann averaged ECD spectrum and that calculated for ΔΔG-based on the lowest energy conformer of a given compound (dashed blue line). All calculated spectra were wavelength corrected to match experimental UV maxima. S172

Single crystal X-ray analysis S173

Table SI_54. Dihedral angles *α*, *β*, *γ*, *δ* and ζ (in degrees), selected interatomic distances l_1, l_2 (in Å) and helicities of trityl groups observed in the crystal structures of compounds **1**, **5**, **10**, **11**, **18**-**21**. S174

Table SI_55. Selected crystal data and structure refinement details for **1**, **5**, **10**, **11**, **18**-**21**. S176

Figure SI_131. Percentage contributions of various intermolecular interaction to the Hirshfeld surfaces of molecules in crystal structure. S177

Figure SI_132. Molecular structure of compound a) **1** and b) (*rac*)-**1** (2-butyl substituent is disordered and the minor position showed as thinner lines). Atoms numbering scheme presented for one molecule. Displacement ellipsoids are drawn at 50% probability level. The sixfold phenyl embrace supramolecular synthon in the crystal structure of **1** c) top view and d) side view. Comparison of molecular packing in isostructural crystals of compound e) **1** (symmetrically independent molecules are indicated with different colors) and f) (*rac*)-**1** (minor position of 2-butyl substituent shown as pink). Hydrogen atoms are omitted for clarity and oxygen atoms are shown as balls. S178

Figure SI_133. Molecular structure of asymmetric unit of a) **5**-*α* and b) **5**-*β*. Atoms numbering scheme presented for one molecule. Displacement ellipsoids are drawn at 50% probability level. Comparison of molecular packing in the crystals of c) **5**-*α* and d) **5**-*β*. Symmetrically independent molecules are indicated with different colors. Hydrogen atoms are omitted for clarity and oxygen atoms are shown as balls. ... S179

Figure SI_134. a) Molecular structure with atoms numbering scheme and b) molecular packing in crystal of (*rac*)-**5**. Displacement ellipsoids are drawn at 50% probability level. Hydrogen atoms are omitted for clarity and oxygen atoms are shown as balls. S180

Figure SI_135. The recorded X-ray powder diffraction pattern of compound **5** (red) and predicted pattern based on single-crystal X-ray diffraction indices of polymorphs **5**-*α* and **5**-*β*. S181

Figure SI_136. Molecular structure and atoms numbering scheme of compound a) **10** and b) **11**. Displacement ellipsoids are drawn at 50% probability level. Comparison of molecular packing in crystal structure of c) **10** and d) **11**. Hydrogen atoms are omitted for clarity and oxygen atoms are shown as balls. S182

Figure SI_137. Molecular structure and atoms numbering scheme of compound **18**. Only the asymmetric part has been numbered for clarity. Displacement ellipsoids are drawn at 50% probability level. Molecular packing in crystal structure b) view along b axis and c) view along c axis. Hydrogen atoms are omitted for clarity and oxygen atoms are shown as balls. S183

Figure SI_138. Molecular structure and atoms numbering scheme of compound **19**. Displacement ellipsoids are drawn at 50% probability level. b) Scheme of molecular disorder in columns. c) Molecular packing in crystal structure, view along a axis. Hydrogen atoms are omitted for clarity and oxygen atoms are shown as balls. S184

Figure SI_139. Molecular structure of asymmetric unit in crystal structure of compound **20**. Displacement ellipsoids are drawn at 50% probability level. b) Comparison molecular conformation of symmetrically independent molecules. c) Molecular packing in crystal structure -molecules **A** + **B** and **C** + **D** forms alternating double layers. Hydrogen atoms are omitted for clarity and oxygen atoms are shown as balls. S185

Figure SI_140. a) Molecular structure and atoms numbering scheme of compound **21**. Only the asymmetric part has been numbered for clarity. Displacement ellipsoids are drawn at 50% probability level. Molecular packing in crystal structure b) view along b axis and c) view along c axis. Hydrogen atoms are omitted for clarity and oxygen atoms are shown as balls. S186

Copies of ^{1}H and ^{13}C NMR spectra S187

Copies of ECD spectra S237

Cartesian coordinates S260

References S581

Calculations details

Starting geometries of esters **1**, **4**, **6**, **10**, **11**, **14**, **15**, **18**, **20**-**22** were obtained by a conformational search with the use of Scigress[1] software and by pre-optimization of all conformers at the B3LYP/6-31G(d) level.[2] The conformational searches were done by systematic changes of all rotatable torsion angles with 30° steps. This allowed to identify the minimum energy structures which were further re-optimized with the independent use of a B3LYP[3] and M06-2X[4] hybrid functionals in conjunction with an enhanced 6-311++G(d,p) basis set. In the case of diesters **18**, **20**-**22** smaller 6-311G(d,p) basis set was used for geometry optimization.[2] Expecting the impact of non-covalent (London) interactions on the structure of specific compounds, the diesters **18**, **20**-**22** were additionally optimized with the use of B3LYP hybrid functional with dispersion correction[5] and in conjunction with the 6-311G(d,p) basis set.

The structures thus obtained were the real minimum energy conformers (no imaginary frequencies were found). The total and free energy values were used to obtain the Boltzmann population of conformers at 298.15 K. For density functional theory calculations, only the results for conformers that were different from the most stable one by less than 2 kcal mol^{-1} were taken into account, following a generally accepted protocol.[6] Relative energies(unit kcal mol^{-1}) discussed in the main text refer to Gibbs free energies ($\Delta\Delta G$) computed at the respective DFT level of theory. Energetic and structural data have been juxtaposed in **Tables SI_1**-**SI_53**, whereas in **Figures SI_1**-**SI_26** have shown structures of individual low-energy conformers of **1**, **4**, **6**, **10**, **11**, **14**, **15**, **18**, **20**-**22**, calculated at various DFT levels.

ECD spectra for all structures optimized at the DFT level were calculated employing M06-2X[4] and CAM-B3LYP,[7] hybrid functionals, in conjunction with the 6-311++G(2d,2p) or 6-311++G(d,p) basis sets (see **Figures SI_27**-**SI_78**).[2] The calculated ECD spectra were Boltzmann averaged by taking into account thermally accessible conformers of **1**, **4**, **6**, **10**, **11**, **14**, **15**, **18**, **20**-**22** ranging from 0 to 2.0 kcal mol^{-1} in relative energies.[6]

Rotatory strengths were calculated using both length and velocity representations. In the present study, the differences between the length and velocity calculated values of the rotator strengths were quite small, and for this reason, only the velocity representations were further used.

The ECD spectra were simulated by overlapping Gaussian functions for each transition, according to the procedure previously described by Harada and Stephens.[8]

The solvent effect on the structure and ECD spectra was not taken into account, since all measurements for esters **1**, **4**, **6**, **10**, **11**, **14**, **15**, **18**, **20**-**22** were done in non-polar cyclohexane.

Since there are no significant differences between results obtained with the use of M06-2X and CAM-B3LYP functionals, we limited our discussion to the results obtained with the use of M06-2X functional only.

The best combination of methods for structure/spectra prediction was chosen by comparison of experimental and Boltzmann averaged CD spectra calculated using all possible combinations of methods (see **Figures SI_79**-**SI_130**). The best performed combination for prediction of the structure and the ECD spectra of esters **1**, **4**, **6**, **10**, **11** and **14** consists of B3LYP/6-311++G(d,p) method for

structure optimization and TD-M06-2X/6-311++G(2d,2p) for calculations of rotatory strengths. The ester **15** is an exception, the much better results were obtained for combination of M06-2X/6-311++G(d,p) method for geometry calculations and TD-M06-2X/6-311++G(2d,2p) method for calculations of chiroptical properties. For the particular ester **15** the CH•••O interaction between inductor and acceptor may take place.

The comparison of ECD spectra – the experimental ones and those calculated with the use of various methods, provided some general conclusions. Firstly – the use of the B3LYP hybrid functional that have long-range corrections improved the final results only for diesters **18** and **20**, having the ester groups in the close proximity. In the remaining cases, namely **21** and **22**, the results obtained with the use of B3LYP-GD3BJ method did not improve the results but elongated the CPU time. Therefore, only for **18** and **20,** the London dispersive interactions control the structure of the given compound.
Secondly, the structure of investigated monoesters is controlled by intermolecular interactions satisfactorily reproduced by "classical" B3LYP functional.
Thirdly. In the case of compounds, in which the electrostatic attractive C=O··H-C_{Ar} interactions take place, much better results were obtained with the newer M06-2X hybrid functional than that obtained with "classical" B3LYP hybrid functional. This is especially visible for the derivative **6,** whereas for **14,** the impact of electrostatic interactions is overestimated.

Table SI_1. Concentrations (*c*, in mol L^{-1}) of the samples used for UV and ECD measurements.

Compound	*c* (cyclohexane)	*c* (acetonitrile)
**1**	2.908×10^{-4}	2.972×10^{-4}
**2**	2.839×10^{-4}	2.842×10^{-4}
**3**	2.52×10^{-4}	2.496×10^{-4}
**4**	2.822×10^{-4}	2.811×10^{-4}
**5**	2.596×10^{-4}	2.563×10^{-4}
**6**	2.295×10^{-4}	2.293×10^{-4}
**7**	2.348×10^{-4}	2.355×10^{-4}
**8**	2.833×10^{-4}	2.803×10^{-4}
**9**	2.388×10^{-4}	2.393×10^{-4}
**10**	2.386×10^{-4}	2.348×10^{-4}
**11**	2.376×10^{-4}	2.379×10^{-4}
**12**	1.517×10^{-4}	1.532×10^{-4}
**13**	1.796×10^{-4}	1.8×10^{-4}
**14**	2.546×10^{-4}	2.519×10^{-4}
**15**	2.8×10^{-4}	2.809×10^{-4}
**16**	2.726×10^{-4}	2.702×10^{-4}
**17**	1.607×10^{-4}	1.602×10^{-4}
**18**	1.632×10^{-4}	1.647×10^{-4}
**19**	1.355×10^{-4}	1.599×10^{-4}
**20**	1.588×10^{-4}	1.599×10^{-4}
**21**	1.738×10^{-3}	2.989×10^{-3}
**22**	1.529×10^{-4}	1.524×10^{-4}

Table SI_2. ECD ($\Delta\varepsilon$, in $dm^3 \cdot mol^{-1} \cdot cm^{-1}$) and UV ($\varepsilon$, in $dm^3 \cdot mol^{-1} \cdot cm^{-1}$) data for derivatives **1**-**22** measured in acetonitrile solution.

Compound	$\Delta\varepsilon$ (nm)	ε (nm)
**1**	-2.5 (229); 11.4 (200); -7.1 (185)	73450 (196)
**2**	3.4 (227); -13.2 (201); 10.3 (185)	72000 (197)
**3**	1.1 (225); -2.5 (203); 3.8 (185)	69550 (196)
**4**	-3.2 (229); 15.5 (201); -8.2 (185)	70500 (196)
**5**	-1.5 (232); 4.5 (203); 3.3 (192); -2.2 (185)	120050 (189)
**6**	6.4 (231); -33.0 (200); 21.0 (185)	73100 (196)
**7**	15.6 (222); -26.9 (190)	112600 (191)
**8**	0.5 (222); -1.3 (202); -1.1 (197); 2.4 (185)	71800 (196)
**9**	0.7 (226); -2.2 (203); -2.1 (198); 1.4 (185)	75100 (196)
**10**	-9.3 (229); 41.4 (201); -27.0 (185)	71200 (196)
**11**	-2.9 (214); -3.0(204); 4.5 (185)	70100 (196)
**12**	-0.6 (228); 2.4 (200); 1.4 (185)	66200 (186)
**13**	-1.2 (328); 8.5 (227); 4.4 (185)	73600 (196)
**14**	10.9 (228); -51.5 (200); 31.4 (185)	71100 (196)
**15**	-1.5 (231); 5.1 (199); -0.8 (185)	65950 (196)
**16**	1.2 (230); -5.3 (198)	72750 (196)
**17**	10.6 (231); -41.4 (204); 8.0 (185)	142000 (193)
**18**	-0.5 (262); 3.1 (230); -2.9 (209); 0.5 (199); -3.1 (191); 0.3 (185)	138300 (195)
**19**	-6.8 (223); 30.0 (196); -19.4 (185)	141550 (196)
**20**	-5.6 (229); 34.1 (204); -21.8 (185)	135250 (194)
**21**	-3.7 (225); 14.6 (196); -27.1 (185)	15510 (188)
**22**	-11.0 (230); 45.1 (202); -24.9 (186)	131700 (196)

Table SI_3. Total and free energies (*E*, *ΔG*, in Hartree), relative energies (*ΔE*, *ΔΔG* in kcal mol^{-1}), percentage populations and number of imaginary frequencies (#ImFreq) calculated at the B3LYP/6-311++G(d,p) level for individual conformers of **1**.

Conformer no[a]	*E*	*ΔG*	*ΔE*	Pop.	*ΔΔG*	Pop	#ImFreq
1	-1079.74787	-1079.38658	0.00	14.39	0.98	7.03	0
17	-1079.74654	-1079.38	0.84	3.5	1.99	1.27	0
33	-1079.74678	-1079.38659	0.68	4.55	0.98	7.1	0
38	-1079.7472	-1079.38815	0.42	7.08	0.00	36.97	0
39	-1079.74652	-1079.38554	0.85	3.45	1.64	2.32	0
40	-1079.7472	-1079.38693	0.42	7.03	0.76	10.16	0
41	-1079.74714	-1079.38684	0.46	6.63	0.82	9.2	0
42	-1079.74591	-1079.38496	1.23	1.81	2.01	-	0
43	-1079.74622	-1079.38515	1.04	2.49	1.88	1.54	0
44	-1079.74747	-1079.38635	0.25	9.38	1.13	5.48	0
45	-1079.74777	-1079.38634	0.06	12.98	1.14	5.43	0
49	-1079.74777	-1079.38633	0.06	12.98	1.14	5.39	0
50	-1079.74667	-1079.38524	0.76	4.02	1.83	1.69	0
52	-1079.74725	-1079.3865	0.39	7.48	1.04	6.42	0
53	-1079.74611	-1079.38417	1.10	2.23	2.5	-	0

[a] Conformers are numbered according to their appearance during conformational search.

Table SI_4. Total and free energies (E, ΔG, in Hartree), relative energies (ΔE, $\Delta\Delta G$ in kcal mol^{-1}), percentage populations and number of imaginary frequencies (#ImFreq) calculated at the M06-2X/6-311++G(d,p) level for individual conformers of **1**.

Conformer no[a]	*E*	*ΔG*	*ΔE*	Pop.	*ΔΔG*	Pop	#ImFreq
1	-1079.30025	-1078.93348	0	37.59	0.11	20.95	0
17	-1079.29975	-1078.93227	0.31	22.19	0.88	5.8	0
33	-1079.29767	-1078.93	1.62	2.44	0.90	5.52	0
38	-1079.29764	-1078.93165	1.64	2.37	1.26	3.01	0
39	-1079.29734	-1078.93116	1.82	1.73	1.58	1.78	0
40	-1079.29722	-1078.93297	1.90	1.52	0.44	12.16	0
41	-1079.29707	-1078.9309	1.99	1.29	1.74	1.36	0
44	-1079.29919	-1078.93367	0.66	12.26	0.00	25.43	0
45	-1079.29898	-1078.93324	0.79	9.83	0.26	16.28	0
50	-1079.29752	-1078.93175	1.71	2.1	1.20	3.32	0
52	-1079.29726	-1078.93068	1.87	1.59	1.87	1.07	0
53	-1079.29836	-1078.93174	1.19	5.08	1.21	3.3	0

[a] Conformers are numbered according to their appearance during conformational search.

Table SI_5. Total and free energies (*E*, *ΔG*, in Hartree), relative energies (*ΔE*, *ΔΔG* in kcal mol^{-1}), percentage populations and number of imaginary frequencies (#ImFreq) calculated at the B3LYP/6-311++G(d,p) level for individual conformers of **4**.

Conformer no[a]	*E*	*ΔG*	*ΔE*	Pop.	*ΔΔG*	Pop	#ImFreq
1	-1119.07063	-1118.68223	0	21.34	0.64	9.26	0
20	-1119.07028	-1118.68245	0.22	14.7	0.5	11.68	0
22	-1119.0704	-1118.68254	0.14	16.72	0.44	12.89	0
42	-1119.07051	-1118.68312	0.08	18.79	0.08	23.85	0
44	-1119.06983	-1118.68269	0.5	9.11	0.35	15.09	0
46	-1119.07054	-1118.68325	0.06	19.33	0	27.23	0

[a] Conformers are numbered according to their appearance during conformational search.

Table SI_6. Total and free energies (*E*, *ΔG*, in Hartree), relative energies (*ΔE*, *ΔΔG* in kcal mol^{-1}), percentage populations and number of imaginary frequencies (#ImFreq) calculated at the M06-2X/6-311++G(d,p) level for individual conformers of **4**.

Conformer no[a]	*E*	*ΔG*	*ΔE*	Pop.	*ΔΔG*	Pop	#ImFreq
1	-1118.60547	-1118.213	0.00	42.42	0.00	59.64	0
20	-1118.60451	-1118.21146	0.60	15.28	0.96	11.71	0
22	-1118.60511	-1118.21114	0.22	29.03	1.16	8.37	0
42	-1118.60276	-1118.21138	1.70	2.39	1.01	10.78	0
44	-1118.60271	-1118.21017	1.73	2.29	1.77	3	0
46	-1118.60396	-1118.21091	0.95	8.59	1.31	6.5	0

[a] Conformers are numbered according to their appearance during conformational search.

Table SI_7. Total and free energies (*E*, *ΔG*, in Hartree), relative energies (*ΔE*, *ΔΔG* in kcal mol^{-1}), percentage populations and number of imaginary frequencies (#ImFreq) calculated at the B3LYP/6-311++G(d,p) level for individual conformers of **6**.

Conformer no[a]	*E*	*ΔG*	*ΔE*	Pop.	*ΔΔG*	Pop	#ImFreq
1	-1424.44985	-1423.98764	0.00	47.62	0.00	29.07	0
4	-1424.44827	-1423.99	0.99	8.97	0.90	6.37	0
7	-1424.44834	-1423.99	0.95	9.63	0.70	8.97	0
8	-1424.44875	-1423.99	0.69	14.81	0.59	10.81	0
10	-1424.44707	-1423.99	1.74	2.5	1.21	3.75	0
14	-1424.44789	-1423.99	1.23	5.95	1.12	4.39	0
17	-1424.44584	-1423.98579	2.51	-	1.16	4.09	0
18	-1424.44751	-1423.98528	1.47	4	1.48	2.38	0
20	-1424.44712	-1423.9869	1.71	2.64	0.47	13.16	0
21	-1424.44667	-1423.98533	1.99	1.65	1.46	2.49	0
28	-1424.44696	-1423.98691	1.81	2.24	0.46	13.37	0
46	-1424.44594	-1423.9846	2.45	-	1.91	1.15	0

[a] Conformers are numbered according to their appearance during conformational search.

Table SI_8. Total and free energies (*E*, *ΔG*, in Hartree), relative energies (*ΔE*, *ΔΔG* in kcal mol^{-1}), percentage populations and number of imaginary frequencies (#ImFreq) calculated at the M06-2X/6-311++G(d,p) level for individual conformers of **6**.

Conformer no[a]	*E*	*ΔG*	*ΔE*	Pop.	*ΔΔG*	Pop	#ImFreq
1	-1423.87727	-1423.40816	0.00	67.16	0.00	62.73	0
7	-1423.87478	-1423.40654	1.56	4.82	1.02	11.2	0
11	-1423.87449	-1423.40431	1.75	3.52	2.42	-	0
14	-1423.87576	-1423.40623	0.95	13.56	1.21	8.11	0
17	-1423.87435	-1423.40454	1.83	3.04	2.27	-	0
28	-1423.87391	-1423.40501	2.11	-	1.98	2.21	0
30	-1423.87492	-1423.40686	1.47	5.58	0.82	15.75	0
32	-1423.8741	-1423.4032	1.99	2.33	3.11	-	0

[a] Conformers are numbered according to their appearance during conformational search.

Table SI_9. Total and free energies (*E*, *ΔG*, in Hartree), relative energies (*ΔE*, *ΔΔG* in kcal mol^{-1}), percentage populations and number of imaginary frequencies (#ImFreq) calculated at the B3LYP/6-311++G(d,p) level for individual conformers of **10**.

Conformer no[a]	*E*	*ΔG*	*ΔE*	Pop.	*ΔΔG*	Pop	#ImFreq
1	-1314.48305	-1313.97887	0	100	0	100	0

[a] Conformers are numbered according to their appearance during conformational search.

Table SI_10. Total and free energies (*E*, *ΔG*, in Hartree), relative energies (*ΔE*, *ΔΔG* in kcal mol^{-1}), percentage populations and number of imaginary frequencies (#ImFreq) calculated at the M06-2X/6-311++G(d,p) level for individual conformers of **10**.

Conformer no[a]	*E*	*ΔG*	*ΔE*	Pop.	*ΔΔG*	Pop	#ImFreq
1	-1313.9347	-1313.4237	0	100	0	100	0

[a] Conformers are numbered according to their appearance during conformational search.

Table SI_11. Total and free energies (*E*, *ΔG*, in Hartree), relative energies (*ΔE*, *ΔΔG* in kcal mol^{-1}), percentage populations and number of imaginary frequencies (#ImFreq) calculated at the B3LYP/6-311++G(d,p) level for individual conformers of **11**.

Conformer no[a]	*E*	*ΔG*	*ΔE*	Pop.	*ΔΔG*	Pop	#ImFreq
1	-1314.4815	-1313.97671	0.30	27.31	1.34	6.18	0
22	-1314.48123	-1313.97729	0.47	20.43	0.98	11.37	0
27	-1314.48199	-1313.97795	0.00	45.54	0.56	23.09	0
52	-1314.47884	-1313.97413	1.97	1.62	2.96	-	0
56	-1314.4794	-1313.98	1.62	2.93	0.00	59.35	0
62	-1314.47911	-1313.97387	1.80	2.16	3.12	-	0

[a] Conformers are numbered according to their appearance during conformational search.

Table SI_12. Total and free energies (*E*, *ΔG*, in Hartree), relative energies (*ΔE*, *ΔΔG* in kcal mol^{-1}), percentage populations and number of imaginary frequencies (#ImFreq) calculated at the M06-2X/6-311++G(d,p) level for individual conformers of **11**.

Conformer no[a]	*E*	*ΔG*	*ΔE*	Pop.	*ΔΔG*	Pop	#ImFreq
1	-1313.93677	-1313.4233	0.00	82.88	0.00	65.32	0
27	-1313.93424	-1313.42235	1.58	5.73	0.60	23.69	0
31	-1313.9341	-1313.42102	1.67	4.94	1.43	5.83	0
33	-1313.93381	-1313.41858	1.85	3.62	2.96	-	0
50	-1313.93358	-1313.41987	1.99	2.84	2.16	-	0
52	-1313.93306	-1313.42035	2.33	-	1.85	2.86	0
56	-1313.9327	-1313.42015	2.55	-	1.98	2.3	0

[a] Conformers are numbered according to their appearance during conformational search.

Table SI_13. Total and free energies (*E*, *ΔG*, in Hartree), relative energies (*ΔE*, *ΔΔG* in kcal mol^{-1}), percentage populations and number of imaginary frequencies (#ImFreq) calculated at the B3LYP/6-311++G(d,p) level for individual conformers of **14**.

Conformer no[a]	*E*	*ΔG*	*ΔE*	Pop.	*ΔΔG*	Pop	#ImFreq
1	-1306.47214	-1306.09	0.00	88.55	0.00	77.91	0
6	-1306.46911	-1306.0896	1.90	3.58	1.69	4.49	0
8	-1306.46985	-1306.09067	1.43	7.86	1.02	13.97	0
39	-1306.46791	-1306.0894	2.65	–	1.81	3.64	0

[a] Conformers are numbered according to their appearance during conformational search.

Table SI_14. Total and free energies (*E*, *ΔG*, in Hartree), relative energies (*ΔE*, *ΔΔG* in kcal mol^{-1}), percentage populations and number of imaginary frequencies (#ImFreq) calculated at the M06-2X/6-311++G(d,p) level for individual conformers of **14**.

Conformer no[a]	*E*	*ΔG*	*ΔE*	Pop.	*ΔΔG*	Pop	#ImFreq
1	-1305.95634	-1305.56935	0.00	68.72	0.37	28.35	0
6	-1305.95492	-1305.56849	0.89	15.22	0.91	11.36	0
8	-1305.95337	-1305.56994	1.87	2.93	0.00	53.1	0
27	-1305.95478	-1305.56719	0.98	13.12	1.73	2.87	0
49	-1305.95219	-1305.56757	2.61		1.49	4.32	0

[a] Conformers are numbered according to their appearance during conformational search.

Table SI_15. Total and free energies (E, ΔG, in Hartree), relative energies (ΔE, $\Delta\Delta G$ in kcal mol^{-1}), percentage populations and number of imaginary frequencies (#ImFreq) calculated at the B3LYP/6-311++G(d,p) level for individual conformers of **15**.

Conformer no[a]	*E*	*ΔG*	*ΔE*	Pop.	*ΔΔG*	Pop	#ImFreq
1	-1153.76091	-1153.41	0.00	26.47	0.38	10.1	0
17	-1153.75997	-1153.41	0.59	9.77	0.28	11.94	0
18	-1153.76037	-1153.42	0.34	14.89	0.00	19.11	0
20	-1153.76014	-1153.41469	0.48	11.68	0.24	12.74	0
34	-1153.75955	-1153.41379	0.86	6.23	0.80	4.94	0
47	-1153.75946	-1153.41486	0.91	5.69	0.13	15.22	0
54	-1153.7601	-1153.41365	0.51	11.19	0.89	4.24	0
65	-1153.75978	-1153.41458	0.71	7.97	0.31	11.31	0
70	-1153.75953	-1153.4145	0.87	6.10	0.36	10.39	0

[a] Conformers are numbered according to their appearance during conformational search.

Table SI_16. Total and free energies (E, ΔG, in Hartree), relative energies (ΔE, $\Delta\Delta G$ in kcal mol^{-1}), percentage populations and number of imaginary frequencies (#ImFreq) calculated at the M06-2X/6-311++G(d,p) level for individual conformers of **15**.

Conformer no[a]	*E*	*ΔG*	*ΔE*	Pop.	*ΔΔG*	Pop	#ImFreq
1	-1153.30037	-1152.95	0.02	23.95	0.33	18.11	0
17	-1153.29972	-1152.94792	0.42	12.1	0.56	12.08	0
18	-1153.29966	-1152.95	0.46	11.38	0.89	6.97	0
20	-1153.3004	-1152.94882	0.00	24.74	0.00	31.36	0
34	-1153.29872	-1152.9457	1.06	4.16	1.96	1.15	0
47	-1153.29819	-1152.94664	1.39	2.38	1.37	3.12	0
58	-1153.29923	-1152.9472	0.73	7.19	1.01	5.67	0
65	-1153.29883	-1152.94747	0.98	4.69	0.84	7.56	0
70	-1153.2993	-1152.94769	0.69	7.76	0.71	9.48	0
75	-1153.29785	-1152.94698	1.60	1.66	1.15	4.49	0

[a] Conformers are numbered according to their appearance during conformational search.

Table SI_17. Total and free energies (*E*, *ΔG*, in Hartree), relative energies (*ΔE*, *ΔΔG* in kcal mol^{-1}), percentage populations and number of imaginary frequencies (#ImFreq) calculated at the B3LYP/6-311G(d,p) level for individual conformers of **18**.

Conformer no[a]	*E*	*ΔG*	*ΔE*	Pop.	*ΔΔG*	Pop	#ImFreq
1	-1961.63782	-1961.0419	1.06	1.9	1.72	1.1	0
5	-1961.63951	-1961.0439	0.00	11.3	0.47	9.13	0
9	-1961.63811	-1961.0425	0.88	2.58	1.34	2.07	0
13	-1961.63759	-1961.04178	1.21	1.48	1.80	0.97	0
15	-1961.63885	-1961.04311	0.41	5.67	0.96	3.96	0
17	-1961.63843	-1961.04132	0.68	3.62	2.08	-	0
18	-1961.63925	-1961.0429	0.16	8.66	1.09	3.18	0
26	-1961.63797	-1961.04179	0.97	2.22	1.79	0.98	0
28	-1961.63938	-1961.04366	0.08	9.86	0.62	7.1	0
32	-1961.63863	-1961.04151	0.55	4.47	1.96	0.73	0
34	-1961.63951	-1961.04279	0.00	11.37	1.16	2.83	0
35	-1961.63654	-1961.04097	1.86	0.49	2.30	-	0
36	-1961.63874	-1961.04408	0.48	5.03	0.35	11.1	0
42	-1961.63858	-1961.04286	0.58	4.25	1.12	3.03	0
43	-1961.63834	-1961.04275	0.73	3.3	1.19	2.7	0
45	-1961.63715	-1961.04173	1.48	0.94	1.82	0.92	0
46	-1961.63766	-1961.0429	1.16	1.6	1.09	3.17	0
48	-1961.6374	-1961.04175	1.33	1.21	1.81	0.94	0
58	-1961.63862	-1961.04464	0.56	4.42	0.00	20.09	0
59	-1961.63864	-1961.04116	0.54	4.54	2.18	-	0
60	-1961.63672	-1961.04142	1.75	0.59	2.02	-	0
61	-1961.6379	-1961.04149	1.01	2.06	1.98	0.71	0
69	-1961.63704	-1961.04119	1.55	0.83	2.16	-	0
70	-1961.63763	-1961.04265	1.18	1.55	1.25	2.43	0
71	-1961.63746	-1961.04408	1.29	1.29	0.35	11.16	0
73	-1961.63828	-1961.04358	0.77	3.1	0.66	6.55	0
76	-1961.63702	-1961.04266	1.57	0.81	1.24	2.46	0
77	-1961.63708	-1961.0414	1.53	0.86	2.04	-	0
93	-1961.63614	-1961.04274	2.12	-	1.19	2.67	0

[a] Conformers are numbered according to their appearance during conformational search.

Table SI_18. Total and free energies (*E*, *ΔG*, in Hartree), relative energies (*ΔE*, *ΔΔG* in kcal mol^{-1}), percentage populations and number of imaginary frequencies (#ImFreq) calculated at the B3LYP-GD3BJ/6-311G(d,p) level for individual conformers of **18**.

Conformer no[a]	*E*	*ΔG*	*ΔE*	Pop.	*ΔΔG*	Pop	#ImFreq
13	-1961.8714	-1961.26924	0.08	29.26	0.54	12.19	0
15	-1961.87078	-1961.26993	0.47	15.14	0.10	25.32	0
17	-1961.87087	-1961.26851	0.42	16.68	1.00	5.62	0
26	-1961.86666	-1961.26711	3.06	-	1.88	1.27	0
32	-1961.87154	-1961.2701	0.00	33.68	0.00	30.23	0
35	-1961.86722	-1961.26755	2.71	-	1.60	2.02	0
36	-1961.8682	-1961.26855	2.09	-	0.97	5.87	0
46	-1961.86741	-1961.26882	2.59	-	0.80	7.77	0
60	-1961.86752	-1961.26769	2.52	-	1.51	2.36	0
63	-1961.86723	-1961.26701	2.70	-	1.94	1.14	0
77	-1961.86978	-1961.26861	1.10	5.25	0.94	6.23	0

[a] Conformers are numbered according to their appearance during conformational search.

Table SI_19. Total and free energies (*E*, *ΔG*, in Hartree), relative energies (*ΔE*, *ΔΔG* in kcal mol^{-1}), percentage populations and number of imaginary frequencies (#ImFreq) calculated at the M06-2X/6-311G(d,p) level for individual conformers of **18**.

Conformer no[a]	*E*	*ΔG*	*ΔE*	Pop.	*ΔΔG*	Pop	#ImFreq
1	-1960.87251	-1960.26243	1.82	1.46	2.16	-	0
5	-1960.87167	-1960.26353	2.35	-	1.47	3.05	0
13	-1960.87522	-1960.26485	0.12	25.65	0.64	12.29	0
15	-1960.8728	-1960.26276	1.64	1.98	1.95	1.35	0
17	-1960.87385	-1960.26371	0.98	5.98	1.35	3.71	0
18	-1960.87309	-1960.26465	1.46	2.69	0.77	9.98	0
28	-1960.87541	-1960.26518	0.00	31.43	0.43	17.57	0
32	-1960.87347	-1960.26402	1.22	4.01	1.16	5.1	0
36	-1960.87227	-1960.26456	1.97	1.13	0.83	9.05	0
42	-1960.87522	-1960.26587	0.12	25.68	0.00	36.47	0
73	-1960.86827	-1960.26282	4.48	-	1.92	1.43	0

[a] Conformers are numbered according to their appearance during conformational search.

Table SI_20. Total and free energies (*E*, *ΔG*, in Hartree), relative energies (*ΔE*, *ΔΔG* in kcal mol^{-1}), percentage populations and number of imaginary frequencies (#ImFreq) calculated at the B3LYP/6-311G(d,p) level for individual conformers of **20**.

Conformer no[a]	*E*	*ΔG*	*ΔE*	Pop.	*ΔΔG*	Pop	#ImFreq
1	-2000.96492	-2000.3432	0.51	13.37	0.01	24.72	0
11	-2000.96394	-2000.342	1.13	4.76	0.75	6.92	0
14	-2000.96381	-2000.3419	1.21	4.12	0.82	6.22	0
16	-2000.96475	-2000.34164	0.62	11.2	0.98	4.74	0
17	-2000.96484	-2000.34177	0.56	12.32	0.9	5.45	0
20	-2000.96356	-2000.3401	1.37	3.18	1.94	0.93	0
21	-2000.96342	-2000.34138	1.46	2.73	1.14	3.6	0
22	-2000.96574	-2000.3432	0.00	31.86	0.00	24.77	0
23	-2000.96431	-2000.34196	0.9	6.99	0.78	6.63	0
25	-2000.96355	-2000.34063	1.37	3.13	1.61	1.63	0
43	-2000.96327	-2000.34028	1.55	2.32	1.83	1.12	0
46	-2000.96172	-2000.34005	2.52	-	1.98	0.88	0
47	-2000.96378	-2000.34255	1.23	4.02	0.41	12.39	0

[a] Conformers are numbered according to their appearance during conformational search.

Table SI_21. Total and free energies (*E*, *ΔG*, in Hartree), relative energies (*ΔE*, *ΔΔG* in kcal mol^{-1}), percentage populations and number of imaginary frequencies (#ImFreq) calculated at the B3LYP-GD3BJ/6-311G(d,p) level for individual conformers of **20**.

Conformer no[a]	*E*	*ΔG*	*ΔE*	Pop.	*ΔΔG*	Pop	#ImFreq
1	-2001.2047	-2000.58	0.95	5.48	0.85	7.12	0
11	-2001.20602	-2000.58	0.12	22.11	1.32	3.22	0
14	-2001.20508	-2000.58	0.71	8.21	0.78	7.96	0
17	-2001.20621	-2000.57785	0.00	27.12	0.51	12.54	0
20	-2001.20606	-2000.57805	0.10	23.07	0.39	15.51	0
21	-2001.20419	-2000.57673	1.27	3.19	1.22	3.80	0
22	-2001.20378	-2000.57867	1.53	2.05	0.00	29.76	0
25	-2001.20515	-2000.57813	0.67	8.77	0.34	16.79	0
43	-2001.20275	-2000.57659	2.17	-	1.30	3.30	0

[a] Conformers are numbered according to their appearance during conformational search.

Table SI_22. Total and free energies (E, ΔG, in Hartree), relative energies (ΔE, $\Delta\Delta G$ in kcal mol^{-1}), percentage populations and number of imaginary frequencies (#ImFreq) calculated at the M06-2X/6-311G(d,p) level for individual conformers of **20**.

Conformer no[a]	E	ΔG	ΔE	Pop.	$\Delta\Delta G$	Pop	#ImFreq
1	-2000.18136	-1999.54587	1.52	3.4	0.95	6.89	0
11	-2000.18379	-1999.54475	0.00	44.46	1.66	2.09	0
14	-2000.18141	-1999.54538	1.49	3.59	1.26	4.08	0
17	-2000.18293	-1999.54673	0.54	17.95	0.41	17.03	0
20	-2000.1829	-1999.54611	0.56	17.25	0.80	8.89	0
21	-2000.18175	-1999.54618	1.28	5.11	0.76	9.55	0
22	-2000.1813	-1999.54621	1.56	3.19	0.74	9.79	0
23	-2000.17877	-1999.54595	3.15	-	0.90	7.48	0
25	-2000.18174	-1999.54739	1.29	5.05	0.00	34.2	0

[a] Conformers are numbered according to their appearance during conformational search.

Table SI_23. Total and free energies (E, ΔG, in Hartree), relative energies (ΔE, $\Delta\Delta G$ in kcal mol^{-1}), percentage populations and number of imaginary frequencies (#ImFreq) calculated at the B3LYP/6-311G(d,p) level for individual conformers of **21**.

Conformer no[a]	E	ΔG	ΔE	Pop.	$\Delta\Delta G$	Pop	#ImFreq
1	-2040.29554	-2039.64705	0.00	57.34	0.00	55.91	0
6	-2040.29371	-2039.64619	1.15	8.20	0.54	22.35	0
12	-2040.2943	-2039.64526	0.78	15.32	1.12	8.38	0
15	-2040.29451	-2039.64511	0.65	19.14	1.22	7.12	0
31	-2040.28977	-2039.64442	3.62	-	1.65	3.45	0
54	-2040.28943	-2039.64422	3.84	-	1.77	2.79	0

[a] Conformers are numbered according to their appearance during conformational search.

Table SI_24. Total and free energies (E, ΔG, in Hartree), relative energies (ΔE, $\Delta\Delta G$ in kcal mol^{-1}), percentage populations and number of imaginary frequencies (#ImFreq) calculated at the B3LYP-GD3BJ/6-311G(d,p) level for individual conformers of **21**.

Conformer no[a]	E	ΔG	ΔE	Pop.	$\Delta\Delta G$	Pop	#ImFreq
1	-2040.54056	-2039.88688	0.00	94.76	0.00	75.15	0
6	-2040.53672	-2039.88553	2.41	-	0.85	17.94	0
16	-2040.53783	-2039.88463	1.71	5.24	1.41	6.91	0

[a] Conformers are numbered according to their appearance during conformational search.

Table SI_25. Total and free energies (*E*, *ΔG*, in Hartree), relative energies (*ΔE*, *ΔΔG* in kcal mol^{-1}), percentage populations and number of imaginary frequencies (#ImFreq) calculated at the M06-2X/6-311G(d,p) level for individual conformers of **21**.

Conformer no[a]	*E*	*ΔG*	*ΔE*	Pop.	*ΔΔG*	Pop	#ImFreq
1	-2039.4893	-2038.82929	1.06	11.01	0.00	88.26	0
12	-2039.48803	-2038.8257	1.85	2.89	2.25	-	0
15	-2039.48792	-2038.82515	1.93	2.55	2.60	-	0
16	-2039.48878	-2038.82584	1.39	6.34	2.17	-	0
25	-2039.48836	-2038.82512	1.65	4.06	2.62	-	0
35	-2039.49099	-2038.82739	0.00	66.07	1.19	11.74	0
46	-2039.48831	-2038.82377	1.68	3.89	3.46	-	0
61	-2039.48812	-2038.82366	1.80	3.18	3.53	-	0

[a] Conformers are numbered according to their appearance during conformational search.

Table SI_26. Total and free energies (*E*, *ΔG*, in Hartree), relative energies (*ΔE*, *ΔΔG* in kcal mol^{-1}), percentage populations and number of imaginary frequencies (#ImFreq) calculated at the B3LYP/6-311G(d,p) level for individual conformers of **22**.

Conformer no[a]	*E*	*ΔG*	*ΔE*	Pop.	*ΔΔG*	Pop	#ImFreq
1	-2079.61752	-2078.94311	0.01	16.47	0.34	15.6	0
3	-2079.61753	-2078.94365	0.00	16.52	0.00	27.73	0
5	-2079.61651	-2078.94248	0.64	5.62	0.74	8.01	0
7	-2079.61649	-2078.94288	0.65	5.51	0.49	12.2	0
8	-2079.61645	-2078.942	0.67	5.3	1.04	4.83	0
9	-2079.61682	-2078.94166	0.44	7.8	1.25	3.36	0
10	-2079.61506	-2078.93769	1.55	1.22	3.74	-	0
11	-2079.61753	-2078.94144	0.00	16.52	1.38	2.67	0
12	-2079.61684	-2078.94233	0.43	7.97	0.83	6.86	0
13	-2079.61529	-2078.94021	1.40	1.54	2.16	-	0
15	-2079.61591	-2078.94248	1.02	2.97	0.74	8.01	0
17	-2079.6148	-2078.93923	1.71	0.92	2.77	-	0
26	-2079.6152	-2078.93935	1.46	1.4	2.70	-	0
31	-2079.61327	-2078.94115	2.67	-	1.57	1.96	0
36	-2079.61702	-2078.94244	0.32	9.61	0.76	7.68	0
71	-2079.61446	-2078.94059	1.92	0.64	1.92	1.08	0

[a] Conformers are numbered according to their appearance during conformational search.

Table SI_27. Total and free energies (*E*, *ΔG*, in Hartree), relative energies (*ΔE*, *ΔΔG* in kcal mol^{-1}), percentage populations and number of imaginary frequencies (#ImFreq) calculated at the B3LYP-GD3BJ/6-311G(d,p) level for individual conformers of **22**.

Conformer no[a]	*E*	*ΔG*	*ΔE*	Pop.	*ΔΔG*	Pop	#ImFreq
1	-2079.87057	-2079.18934	0.01	35.17	0.40	24.96	0
3	-2079.87057	-2079.18997	0.00	35.26	0.00	49.07	0
5	-2079.86797	-2079.18712	1.63	2.25	1.79	2.38	0
7	-2079.86976	-2079.18928	0.51	15.0	0.43	23.59	0
17	-2079.86901	-2079.18645	0.98	6.74	2.21	-	0
26	-2079.86883	-2079.18633	1.09	5.57	2.29	-	0

[a] Conformers are numbered according to their appearance during conformational search.

Table SI_28. Total and free energies (*E*, *ΔG*, in Hartree), relative energies (*ΔE*, *ΔΔG* in kcal mol^{-1}), percentage populations and number of imaginary frequencies (#ImFreq) calculated at the M06-2X/6-311G(d,p) level for individual conformers of **22**.

Conformer no[a]	*E*	*ΔG*	*ΔE*	Pop.	*ΔΔG*	Pop	#ImFreq
1	-2078.79565	-2078.10589	0.15	26.94	0.39	29.08	0
3	-2078.79566	-2078.10651	0.14	27.33	0.00	55.92	0
11	-2078.79589	-2078.10509	0.00	34.79	0.89	12.39	0
17	-2078.79342	-2078.10362	1.55	2.52	1.81	2.61	0
26	-2078.79455	-2078.1031	0.84	8.42	2.14	-	0

[a] Conformers are numbered according to their appearance during conformational search.

Table SI_29. Dihedral angles α, β, γ, δ and ζ (in degrees) and selected interatomic distances l_1, l_2 (in Å) calculated at the B3LYP/6-311++G(d,p) level for individual low-energy conformers of **1**.

Conformer no[a]	α[b]	β_1[c]	β_2[c]	β_3[c]	γ_1[d]	γ_2[d]	γ_3[d]	δ[e]	ζ[f]	l_1[g]	l_2[h]
1	-174.74	-110.40	127.18	9.70	66.32	-11.79	49.00	33.69	-97.17	2.542	2.438
17	-176.67	-113.13	124.54	7.28	67.79	-13.16	47.93	-32.09	62.62	2.503	2.468
33	178.87	-110.76	127.30	6.31	-1.76	-60.12	-54.21	34.66	63.22	2.515	2.425
38	-175.72	-113.03	124.65	7.36	67.69	-13.15	47.65	-34.15	-174.10	2.497	2.470
39	-171.37	-111.54	126.17	8.56	65.12	-9.80	50.26	33.46	63.88	2.562	2.424
40	171.97	-125.88	111.94	-8.28	10.50	-66.08	-49.30	-28.97	-175.48	2.537	2.434
41	-172.75	-117.85	120.04	2.43	61.47	-2.62	52.88	32.11	-173.03	2.560	2.399
42	168.75	-132.04	105.56	-14.42	21.32	-71.76	-44.05	-25.30	65.27	2.493	2.538
43	169.74	-156.92	81.48	-38.08	49.51	75.21	-12.92	-0.98	-68.00	2.255	2.775
44	171.02	-145.36	92.54	-27.44	43.57	3.82	-22.22	39.65	-64.11	2.274	2.806
45	178.49	-132.52	106.85	-12.73	48.29	44.08	54.89	39.29	-64.36	2.454	2.221
49	178.49	-132.53	106.83	-12.73	48.31	44.07	54.87	39.27	-64.37	2.453	2.221
50	-177.63	-134.47	104.89	-14.39	47.90	40.26	56.22	35.32	63.45	2.470	2.235
52	-178.22	-135.99	103.47	-15.74	48.30	40.76	56.15	34.20	-172.72	2.459	2.245
53	-177.98	153.59	31.83	-85.35	65.94	42.73	38.89	-31.06	61.05	2.344	2.503

[a] Conformers are numbered according to their appearance during conformational search; [b] – α = C_{Tr}-C(=O)-O-C*; [c] – β =O=C-C-C_{ipso}; [d] – γ = (O=)C-C_{Tr}-C_{ipso}-C_{ortho} (of the two possibilities the absolute values ≤90° has been chosen); [e] – δ = C(=O)-O-C*-H; [f] – ζ = O-C*-C-C(*); [g] – l_1 = C=O···HC_{ortho}; [h] – l_2 = (O=)CO···HC_{ortho}.

Table SI_30. Dihedral angles *α*, *β*, *γ*, *δ* and ζ (in degrees) and selected interatomic distances l_1, l_2 (in Å) calculated at the M06-2X/6-311++G(d,p) level for individual low-energy conformers of **1**.

Conformer no[a]	*α*[b]	β_1[c]	β_2[c]	β_3[c]	γ_1[d]	γ_2[d]	γ_3[d]	*δ*[e]	*ζ*[f]	l_1[g]	l_2[h]
1	-172.82	-121.12	117.14	-0.55	63.62	-6.68	51.45	34.22	-59.23	2.496	2.442
17	-166.73	-110.93	126.91	8.79	64.12	-12.04	49.92	-40.17	62.48	2.535	2.415
33	173.77	-111.60	126.83	5.78	1.05	-59.82	-54.92	44.43	63.91	2.510	2.419
38	-162.70	-103.44	135.00	16.51	72.18	-20.93	45.29	-42.51	-179.59	2.517	2.465
39	-174.62	-113.48	124.78	7.07	67.99	-13.93	47.91	38.67	63.48	2.491	2.449
40	174.90	-120.96	117.14	-3.60	10.27	-65.68	-50.73	-35.47	-177.67	2.502	2.446
41	-174.68	-122.45	115.81	-1.92	62.18	-4.12	52.23	36.53	-176.63	2.497	2.430
44	161.98	-133.56	104.96	-14.90	20.71	-71.49	-45.03	46.18	-58.84	2.506	2.477
45	-179.60	-132.50	107.21	-13.14	48.67	46.84	54.08	42.40	-57.46	2.422	2.205
50	176.92	-149.11	91.57	-27.83	48.72	46.93	54.90	37.52	58.71	2.365	2.294
52	178.58	-144.86	94.91	-23.72	49.23	37.51	58.02	38.72	-177.09	2.422	2.291
53	-164.57	-101.04	138.30	15.74	47.64	61.86	45.29	-39.73	60.40	2.485	2.302

[a] Conformers are numbered according to their appearance during conformational search; [b] – *α* = C_{Tr}-C(=O)-O-C*; [c] – *β* =O=C-C-C_{ipso}; [d] – *γ* = (O=)C-C_{Tr}-C_{ipso}-C_{ortho} (of the two possibilities the absolute values ≤90° has been chosen); [e] – *δ* = C(=O)-O-C*-H; [f] – *ζ* = O-C*-C-C(*); [g] – l_1 = C=O···HC_{ortho}; [h] – l_2 = (O=)CO···HC_{ortho}.

Table SI_31. Dihedral angles *α*, *β*, *γ*, *δ* and ζ (in degrees) and selected interatomic distances l_1, l_2 (in Å) calculated at the B3LYP/6-311++G(d,p) level for individual low-energy conformers of **4**.

Conformer no[a]	*α*[b]	$β_1$[c]	$β_2$[c]	$β_3$[c]	$γ_1$[d]	$γ_2$[d]	$γ_3$[d]	*δ*[e]	*ζ*[f]	$ζ_2$[f]	l_1[g]	l_2[h]
1	-175.15	-114.27	123.32	5.94	63.99	-7.26	50.97	33.12	59.77	-66.42	2.546	2.418
20	171.32	-126.94	110.37	-9.76	19.98	-70.37	-44.76	40.53	60.32	-66.08	2.466	2.565
22	-176.92	-116.13	121.56	4.38	66.62	-10.74	48.68	-31.62	65.02	-168.66	2.490	2.467
42	-171.85	-114.37	123.35	5.82	64.22	-8.39	50.27	30.50	65.05	-168.53	2.535	2.438
44	170.67	-149.80	88.32	-31.50	47.39	77.42	-15.42	-22.56	65.69	-168.07	2.235	2.734
46	178.51	-44.93	-166.19	72.82	-41.80	-66.71	-34.96	28.12	61.73	-64.48	2.222	2.653

[a] Conformers are numbered according to their appearance during conformational search; [b] – *α* = C_{Tr}-C(=O)-O-C*; [c] – *β* =O=C-C-C_{ipso}; [d] – *γ* = (O=)C-C_{Tr}-C_{ipso}-C_{ortho} (of the two possibilities the absolute values ≤90° has been chosen); [e] – *δ* = C(=O)-O-C*-H; [f] – *ζ* = O-C*-C-C(*); [g] – l_1 = C=O···HC_{ortho}; [h] – l_2 = (O=)CO···HC_{ortho}.

Table SI_32. Dihedral angles *α*, *β*, *γ*, *δ* and ζ (in degrees) and selected interatomic distances l_1, l_2 (in Å) calculated at the M06-2X/6-311++G(d,p) level for individual low-energy conformers of **4**.

Conformer no[a]	*α*[b]	$β_1$[c]	$β_2$[c]	$β_3$[c]	$γ_1$[d]	$γ_2$[d]	$γ_3$[d]	*δ*[e]	*ζ*[f]	$ζ_2$[f]	l_1[g]	l_2[h]
1	-173.79	-121.37	117.11	-0.43	64.62	-6.12	51.44	33.75	63.06	-60.80	2.489	2.432
20	179.24	-95.53	144.10	21.76	-11.40	-58.22	-57.44	47.25	63.86	-60.02	2.454	2.445
22	-167.71	-111.86	126.21	8.28	64.19	-11.02	51.02	-39.06	62.88	-171.88	2.540	2.410
42	-175.36	-121.15	117.30	-0.23	64.70	-7.08	50.69	35.92	61.02	-173.58	2.478	2.444
44	176.45	-121.25	116.57	-4.28	17.59	-69.19	-46.61	-30.48	66.04	-168.74	2.438	2.531
46	178.04	-21.49	-143.45	96.39	-44.03	-69.83	-35.84	34.84	64.62	-59.70	2.455	2.474

[a] Conformers are numbered according to their appearance during conformational search; [b] – *α* = C_{Tr}-C(=O)-O-C*; [c] – *β* =O=C-C-C_{ipso}; [d] – *γ* = (O=)C-C_{Tr}-C_{ipso}-C_{ortho} (of the two possibilities the absolute values ≤90° has been chosen); [e] – *δ* = C(=O)-O-C*-H; [f] – *ζ* = O-C*-C-C(*); [g] – l_1 = C=O···HC_{ortho}; [h] – l_2 = (O=)CO···HC_{ortho}.

Table SI_33. Dihedral angles α, β, γ, δ and ζ (in degrees) and selected interatomic distances l_1, l_2 (in Å) calculated at the B3LYP/6-311++G(d,p) level for individual low-energy conformers of **6**.

Conformer no[a]	α[b]	β_1[c]	β_2[c]	β_3[c]	γ_1[d]	γ_2[d]	γ_3[d]	δ[e]	ζ[f]	ζ_2[f]	l_1[g]	l_2[h]
1	173.94	-128.20	109.38	-10.46	10.62	-65.13	-49.76	-42.80	66.26	-59.39	2.569	2.431
4	173.00	-128.90	108.95	-11.14	12.24	-66.98	-48.84	-45.37	165.41	-67.19	2.560	2.441
7	-179.35	-156.54	83.19	-34.74	49.73	36.46	56.62	-49.20	64.53	-61.35	2.383	2.449
8	174.89	-124.42	113.36	-6.82	8.39	-64.58	-50.68	-52.51	63.89	-172.07	2.556	2.430
10	179.20	-148.97	90.74	-27.59	47.29	37.80	57.73	-49.95	167.58	-65.12	2.439	2.347
14	170.48	-129.40	108.34	-11.38	7.54	-63.83	-50.73	-41.80	65.12	-60.68	2.594	2.373
17	-176.61	-99.11	138.23	20.91	84.28	-36.78	29.00	-46.86	65.15	-60.80	2.313	2.721
18	-179.81	-150.96	88.52	-29.39	48.91	33.62	58.40	-57.23	61.21	-174.74	2.443	2.402
20	177.90	-163.74	76.22	-41.75	53.54	38.00	52.40	-47.29	64.68	-61.20	2.294	2.544
21	168.48	-128.94	109.03	-10.86	8.49	-65.12	-50.08	-42.75	162.90	-70.13	2.580	2.385
28	179.03	-177.30	62.39	-55.98	60.44	44.83	37.33	-41.87	64.99	-60.85	2.160	2.699
46	177.82	-156.21	83.71	-34.41	48.97	37.89	56.17	-55.96	59.95	-176.39	2.375	2.444

[a] Conformers are numbered according to their appearance during conformational search; [b] – α = C_{Tr}-C(=O)-O-C*; [c] – β =O=C-C-C_{ipso}; [d] – γ = (O=)C-C_{Tr}-C_{ipso}-C_{ortho} (of the two possibilities the absolute values ≤90° has been chosen); [e] – δ = C(=O)-O-C*-H; [f] – ζ = O-C*-C-C(*); [g] – l_1 = C=O···HC_{ortho}; [h] – l_2 = (O=)CO···HC_{ortho}.

Table SI_34. Dihedral angles *α*, *β*, *γ*, *δ* and ζ (in degrees) and selected interatomic distances $l_1$and l_2 (in Å) calculated at the M06-2X/6-311++G(d,p) level for individual low-energy conformers of **6**.

Conformer no[a]	*α*[b]	$β_1$[c]	$β_2$[c]	$β_3$[c]	$γ_1$[d]	$γ_2$[d]	$γ_3$[d]	*δ*[e]	*ζ*[f]	$ζ_2$[f]	l_1[g]	l_2[h]
1	169.63	-120.34	118.46	-2.03	4.70	-63.15	-52.11	-34.74	61.96	-61.50	2.525	2.407
7	-179.07	-152.49	87.45	-30.64	50.94	30.29	58.66	-51.72	63.05	-60.55	2.436	2.366
11	168.85	-112.22	126.54	5.98	-3.37	-57.86	-55.51	-46.69	60.25	-176.40	2.531	2.408
14	167.22	-117.53	121.65	1.06	-2.90	-60.91	-54.21	-38.77	62.78	-60.74	2.542	2.347
17	-171.85	-98.91	139.64	21.67	-86.95	-35.01	31.95	-51.40	60.44	-63.60	2.358	2.619
28	-172.20	159.14	37.60	-79.93	67.23	39.52	40.96	-49.82	59.71	-64.31	2.277	2.524
30	170.64	-111.92	126.75	5.90	-1.44	-59.22	-55.24	-49.18	61.32	-175.25	2.526	2.424
32	-179.69	-142.30	96.84	-20.18	57.31	10.34	57.32	-49.62	54.33	-72.83	2.466	2.454

[a] Conformers are numbered according to their appearance during conformational search; [b] – *α* = C_{Tr}-C(=O)-O-C*; [c] – *β* =O=C-C-C_{ipso}; [d] – *γ* = (O=)C-C_{Tr}-C_{ipso}-C_{ortho} (of the two possibilities the absolute values ≤90° has been chosen); [e] – *δ* = C(=O)-O-C*-H; [f] – *ζ* = O-C*-C-C(*); [g] – l_1 = C=O···HC_{ortho}; [h] – l_2 = (O=)CO···HC_{ortho}.

Table SI_35. Dihedral angles *α*, *β*, *γ*, *δ* and ζ (in degrees) and selected interatomic distances l_1, l_2 (in Å) calculated at the DFT/6-311++G(d,p) level for individual low-energy conformers of **10**.

Conformer no[a]	*α*[b]	$β_1$[c]	$β_2$[c]	$β_3$[c]	$γ_1$[d]	$γ_2$[d]	$γ_3$[d]	*δ*[e]	*ζ*[f]	l_1[g]	l_2[h]
1[i]	-176.56	-111.57	125.98	8.64	66.50	-11.31	49.05	31.37	-57.37	2.532	2.447
1[j]	-172.12	-119.70	118.93	1.27	64.74	-6.00	52.04	26.52	-55.43	2.506	2.434

[a] Conformers are numbered according to their appearance during conformational search; [b] – *α* = C_{Tr}-C(=O)-O-C*; [c] – *β* =O=C-C-C_{ipso}; [d] – *γ* = (O=)C-C_{Tr}-C_{ipso}-C_{ortho} (of the two possibilities the absolute values ≤90° has been chosen); [e] – *δ* = C(=O)-O-C*-H; [f] – *ζ* = O-C*-C-C(*); [g] – l_1 = C=O···HC_{ortho}; [h] – l_2 = (O=)CO···HC_{ortho}; [i] optimized at the B3LYP/6-311++G(d,p) level; [j] optimized at the M06-2X/6-311++G(d,p) level.

Table SI_36. Dihedral angles α, β, γ, δ and ζ (in degrees) and selected interatomic distances l_1, l_2 (in Å) calculated at the B3LYP/6-311++G(d,p) level for individual low-energy conformers of **11**.

Conformer no[a]	α[b]	β_1[c]	β_2[c]	β_3[c]	γ_1[d]	γ_2[d]	γ_3[d]	δ[e]	ζ[f]	l_1[g]	l_2[h]
1	-174.53	-108.81	128.47	11.23	70.06	-18.94	45.68	-37.13	60.29	2.488	2.540
22	176.67	-123.21	114.10	-6.44	16.26	-69.13	-46.87	-18.18	61.06	2.466	2.549
27	-176.73	153.60	31.83	-85.28	66.17	42.43	39.50	-31.22	58.67	2.339	2.518
52	-174.68	160.66	39.01	-78.29	66.18	42.06	37.76	-30.92	66.68	2.264	2.587
56	-175.32	161.89	40.30	-77.08	66.14	42.20	37.34	-30.74	65.45	2.256	2.597
62	-178.82	-21.33	-142.98	95.81	-44.05	-66.36	-39.72	-33.81	66.99	2.479	2.462

[a] Conformers are numbered according to their appearance during conformational search; [b] – α = C_{Tr}-C(=O)-O-C*; [c] – β =O=C-C-C_{ipso}; [d] – γ = (O=)C-C_{Tr}-C_{ipso}-C_{ortho} (of the two possibilities the absolute values ≤90° has been chosen); [e] – δ = C(=O)-O-C*-H; [f] – ζ = O-C*-C-C(*); [g] – l_1 = C=O···HC_{ortho}; [h] – l_2 = (O=)CO···HC_{ortho}.

Table SI_37. Dihedral angles *α*, *β*, *γ*, *δ* and ζ (in degrees) and selected interatomic distances l_1, l_2 (in Å) calculated at the M06-2X/6-311++G(d,p) level for individual low-energy conformers of **11**.

Conformer no[a]	*α*[b]	$β_1$[c]	$β_2$[c]	$β_3$[c]	$γ_1$[d]	$γ_2$[d]	$γ_3$[d]	*δ*[e]	*ζ*[f]	l_1[g]	l_2[h]
1	-167.25	-113.12	125.31	7.19	67.40	-14.58	48.37	-42.81	57.67	2.499	2.457
27	-166.95	-102.10	137.49	15.03	45.74	64.29	44.20	-38.28	56.14	2.462	2.329
31	-168.82	-116.43	121.67	4.52	72.65	-20.85	44.63	-49.23	61.14	2.413	2.588
33	-168.25	-115.82	122.09	5.01	75.97	-27.82	39.88	-51.04	61.88	2.350	2.723
50	-179.25	-132.46	107.33	-10.43	-67.40	-42.65	-44.02	-41.33	63.29	2.425	2.370
52	-168.41	151.23	29.02	-89.19	70.95	42.35	33.08	-38.36	65.61	2.356	2.544
56	-165.32	149.38	27.02	-90.73	71.20	41.16	36.72	-39.79	67.46	2.356	2.535

[a] Conformers are numbered according to their appearance during conformational search; [b] – *α* = C_{Tr}-C(=O)-O-C*; [c] – *β* =O=C-C-C_{ipso}; [d] – *γ* = (O=)C-C_{Tr}-C_{ipso}-C_{ortho} (of the two possibilities the absolute values ≤90° has been chosen); [e] – *δ* = C(=O)-O-C*-H; [f] – *ζ* = O-C*-C-C(*); [g] – l_1 = C=O···HC_{ortho}; [h] – l_2 = (O=)CO···HC_{ortho}.

Table SI_38. Dihedral angles α, β, γ, δ, ζ and φ (in degrees) and selected interatomic distances l_1, l_2 and l_3 (in Å) calculated at the B3LYP/6-311++G(d,p) level for individual low-energy conformers of **14**.

Conf. no[a]	α[b]	β_1[c]	β_2[c]	β_3[c]	γ_1[d]	γ_2[d]	γ_3[d]	δ[e]	ζ[f]	φ_1[g]	φ_2[g]	φ_3[g]	φ_4[g]	φ_5[g]	l_1[h]	l_2[i]	l_3[j]
1	176.4	-122.0	-4.5	115.7	6.5	-51.8	-63.6	-11.9	-155.3	-21.2	34.0	-34.1	24.4	-2.1	2.560	2.443	2.441
6	-176.4	-7.8	-125.0	112.8	-48.1	11.7	-69.0	13.1	-84.6	21.6	-30.9	28.6	-17.9	-2.2	2.510	2.458	2.721
8	-175.8	-29.4	-150.9	88.5	57.8	49.4	34.6	-23.5	-155.3	-21.2	34.3	-34.4	24.8	-2.4	2.430	2.392	2.661
39	-174.2	26.1	-91.5	148.0	43.8	34.6	68.7	18.9	-83.9	21.5	-31.1	29.1	-18.5	-1.7	2.431	2.509	

[a] Conformers are numbered according to their appearance during conformational search; [b] – α = C_{Tr}-C(=O)-O-C*; [c] – β =O=C-C-C_{ipso}; [d] – γ = (O=)C-C_{Tr}-C_{ipso}-C_{ortho} (of the two possibilities the absolute values ≤90° has been chosen); [e] – δ = C(=O)-O-C*-H; [f] – ζ = O-C*-C-C; [g] φ_1 = (O=)C-O-C-C; φ_2 = O-C-C-C*; φ_3 = C-C-C*-C(=O); φ_4 = C-C*-C(=O)-O; φ_5 = C*-C(=O)-O-C; [h] – l_1 = C=O···HC_{ortho}; [i] – l_2 = (O=)CO···HC_{ortho}; [j] – l_3 = $C{=}O_{lactone}$···HC_{ortho}.

Table SI_39. Dihedral angles α, β, γ, δ, ζ and φ (in degrees) and selected interatomic distances l_1, l_2 and l_3 (in Å) calculated at the M06-2X/6-311++G(d,p) level for individual low-energy conformers of **14**.

Conf. no[a]	α[b]	β_1[c]	β_2[c]	β_3[c]	γ_1[d]	γ_2[d]	γ_3[d]	δ[e]	ζ[f]	φ_1[g]	φ_2[g]	φ_3[g]	φ_4[g]	φ_5[g]	l_1[h]	l_2[i]	l_3[j]
1	171.5	-6.7	-124.9	113.9	-49.9	10.8	-67.4	-18.0	-157.1	-21.7	36.2	-36.9	27.0	-3.4	2.521	2.433	2.522
6	177.7	-16.9	-135.5	103.2	-42.6	21.7	-75.4	26.5	-80.5	23.4	-33.5	30.9	-19.3	-2.4	2.484	2.450	
8	-175.6	-25.0	94.1	-146.1	57.1	44.4	46.7	-32.1	-155.3	-21.3	35.6	-36.1	26.4	-3.3	2.418	2.274	2.491
27	-179.3	17.6	-102.6	135.8	41.0	-24.9	77.2	-163.1	-110.2	25.5	-23.8	14.6	-0.5	-15.6	2.466	2.507	2.737
49	178.6	6.9	126.4	-114.3	-53.7	-34.1	-63.2	-154.3	-109.7	25.4	-24.2	15.3	-1.3	-15.1	2.466	2.297	2.658

[a] Conformers are numbered according to their appearance during conformational search; [b] – α = C_{Tr}-C(=O)-O-C*; [c] – β =O=C-C-C_{ipso}; [d] – γ = (O=)C-C_{Tr}-C_{ipso}-C_{ortho} (of the two possibilities the absolute values ≤90° has been chosen); [e] – δ = C(=O)-O-C*-H; [f] – ζ = O-C*-C-C; [g] φ_1 = (O=)C-O-C-C; φ_2 = O-C-C-C*; φ_3 = C-C-C*-C(=O); φ_4 = C-C*-C(=O)-O; φ_5 = C*-C(=O)-O-C; [h] – l_1 = C=O···HC_{ortho}; [i] – l_2 = (O=)CO···HC_{ortho}; [j] – l_3 = $C{=}O_{lactone}$···HC_{ortho}.

Table SI_40. Dihedral angles *α*, *β*, *γ*, *δ*, *ζ* and *φ* (in degrees) and selected interatomic distances l_1, l_2 and l_3 (in Å) calculated at the B3LYP/6-311++G(d,p) level for individual low-energy conformers of **15**.

Conf. no[a]	α[b]	β_1[c]	β_2[c]	β_3[c]	γ_1[d]	γ_2[d]	γ_3[d]	δ[e]	ζ[f]	φ_1[g]	φ_2[g]	φ_3[g]	φ_4[g]	φ_5[g]	l_1[h]	l_2[i]	l_3[j]
1	-179.92	-137.59	101.73	-16.96	49.82	33.77	57.49	44.26	-81.52	-41.02	-13.04	-10.43	32.10	-41.02	2.494	2.268	2.921
17	174.00	-148.55	89.43	-30.36	41.83	83.11	-24.15	43.04	-109.75	5.15	-25.35	37.58	-35.91	19.23	2.326	2.875	-
18	-176.56	-94.59	144.83	23.35	-29.25	-53.35	-57.02	46.29	-109.39	5.45	-25.77	37.99	-36.12	19.18	2.446	2.342	-
20	-173.68	-109.67	128.05	10.39	67.85	-14.02	47.64	-36.35	-110.51	6.62	-26.43	37.90	-35.30	17.88	2.528	2.453	-
34	176.60	-125.65	112.07	-8.20	11.97	-66.83	-48.43	43.17	-97.13	18.83	4.83	-26.96	40.37	-37.25	2.524	2.447	-
47	170.32	-127.30	110.43	-9.56	10.95	-65.74	-49.57	-37.75	-113.50	4.83	-25.30	37.83	-36.36	19.70	2.561	2.437	-
54	-179.98	-105.52	134.36	14.55	-50.41	-46.28	-52.77	-45.61	-91.29	27.14	-4.79	-18.84	37.18	-40.65	2.415	2.243	3.471
65	-177.47	-102.38	137.18	16.92	-42.51	-48.02	-55.78	-38.38	-115.58	2.81	-23.64	37.07	-36.92	21.31	2.452	2.255	-
70	-177.12	152.23	30.39	-86.76	66.39	42.27	39.11	-35.15	-106.85	10.21	-28.31	37.47	-32.50	13.87	2.361	2.501	-

[a] Conformers are numbered according to their appearance during conformational search; [b] – α = C_{Tr}-C(=O)-O-C*; [c] – β =O=C-C-C_{ipso}; [d] – γ = (O=)C-C_{Tr}-C_{ipso}-C_{ortho} (of the two possibilities the absolute values ≤90° has been chosen); [e] – δ = C(=O)-O-C*-H; [f] – ζ = O-C*-C-X; [g] φ_1 = X-C2-C3*-C4; φ_2 = C2-C3*-C4-C5; φ_3 = C3*-C4-C5-X; φ_4 = C4-C5-X-C2; φ_5 = C5-X-C2-C3*; [h] – l_1 = C=O···HC_{ortho}; [i] – l_2 = (O=)CO···HC_{ortho}; [j] – l_3 = X···HC_{ortho}.

Table SI_41. Dihedral angles α, β, γ, δ, ζ and φ (in degrees) and selected interatomic distances l_1, l_2 and l_3 (in Å) calculated at the M06-2X/6-311++G(d,p) level for individual low-energy conformers of **15**.

Conf. no[a]	α[b]	β_1[c]	β_2[c]	β_3[c]	γ_1[d]	γ_2[d]	γ_3[d]	δ[e]	ζ[f]	φ_1[g]	φ_2[g]	φ_3[g]	φ_4[g]	φ_5[g]	l_1[h]	l_2[i]	l_3[j]
1	172.52	-147.69	92.43	-26.06	50.29	36.49	58.28	49.39	-76.08	35.51	-37.39	27.36	-5.47	-18.99	2.435	2.306	2.498
17	166.12	-136.77	101.97	-18.23	24.20	-75.45	-42.50	55.98	-113.31	0.46	-24.25	40.74	-42.41	26.17	2.482	2.507	-
18	172.57	170.77	146.93	26.04	-38.92	-46.04	-59.44	54.00	-76.80	34.33	-38.59	30.67	-9.70	-15.63	2.464	2.308	2.906
20	-163.94	-111.62	126.49	8.10	64.95	-12.48	49.42	-51.74	-92.27	22.37	-36.08	38.38	-25.72	1.93	2.528	2.440	-
34	163.50	-157.86	81.72	-38.17	51.41	69.42	-10.79	47.45	-78.80	34.35	-14.21	-10.12	32.99	-42.54	2.253	2.548	2.837
47	175.73	-117.11	121.11	0.34	8.05	-64.53	-50.88	-41.34	-110.26	15.71	-33.06	40.09	-31.82	9.98	2.484	2.457	-
58	-165.64	-110.14	140.96	20.14	-56.09	-44.94	-52.00	-55.70	-78.02	37.67	-20.83	-2.19	26.76	-40.84	2.364	2.236	2.754
65	-176.74	-118.03	122.42	3.52	-62.94	-40.30	-50.08	-48.37	-83.69	31.44	-37.24	31.20	-12.27	-12.25	2.424	2.279	3.317
70	-166.35	-100.56	138.94	16.33	46.87	62.46	44.92	-48.91	-83.26	30.69	-38.37	33.82	-15.55	-9.69	2.487<br>2.511	2.306	3.440
75	-170.88	148.07	25.74	-91.22	64.12	43.64	43.98	-49.44	-84.83	31.49	-9.30	-15.49	36.61	-43.04	2.384<br>2.555	2.368	3.499

[a] Conformers are numbered according to their appearance during conformational search; [b] – α = C_{Tr}-C(=O)-O-C*; [c] – β =O=C-C-C_{ipso}; [d] – γ = (O=)C-C_{Tr}-C_{ipso}-C_{ortho} (of the two possibilities the absolute values ≤90° has been chosen); [e] – δ = C(=O)-O-C*-H; [f] – ζ = O-C*-C-X; [g] φ_1 = X-C2-C3*-C4; φ_2 = C2-C3*-C4-C5; φ_3 = C3*-C4-C5-X; φ_4 = C4-C5-X-C2; φ_5 = C5-X-C2-C3*; [h] – l_1 = C=O···HC_{ortho}; [i] – l_2 = (O=)CO···HC_{ortho}; [j] – l_3 = X···HC_{ortho}.

Table SI_42. Dihedral angles *α*, *β*, *γ*, *δ*, ζ and *φ* (in degrees) and selected interatomic distances l_1, l_2 and l_3 (in Å) calculated at the B3LYP/6-311G(d,p) level for individual low-energy conformers of **18**.

Conf. no[a]	*α*[b]	*β1*[c]	*β2*[c]	*β3*[c]	*γ1*[d]	*γ2*[d]	*γ3*[d]	*δ*[e]	*ζ*[f]	*φ*[g]	*l1*[h]	*l2*[i]	*l3*[j]
1	168.80	-139.44	98.45	-21.34	28.06	-78.30	-37.89	-33.80	63.63	-56.93	2.447	2.548	6.926
5	177.31	-141.83	98.31	-21.27	45.64	51.92	52.01	-8.33	-74.71	167.19	2.353	2.291	6.430
9	172.46	-139.87	98.02	-21.94	31.45	-81.61	-34.05	35.88	69.86	-52.39	2.383	2.603	7.000
13	174.98	-138.11	101.80	-17.94	45.79	50.58	52.94	-32.27	65.73	-55.09	2.383	2.264	6.924
15	177.01	-144.47	95.84	-23.67	46.36	51.82	52.10	-9.88	-70.34	171.54	2.344	2.306	6.130
17	178.24	-94.12	145.47	24.31	-35.84	-50.29	-56.24	-31.66	64.74	-55.92	2.411	2.324	6.841
18	165.50	-148.01	89.82	-29.58	42.22	84.16	-25.96	-30.59	72.88	-48.17	2.338	2.753<br>2.880	6.454
26	-169.47	-96.21	141.89	23.63	79.98	-28.96	36.44	33.46	70.98	-51.57	2.438	2.540	6.930
28	177.06	-143.76	96.44	-23.09	46.31	52.69	50.97	0.46	-77.24	164.38	2.328	2.309	6.515
32	173.77	-42.78	-164.58	74.50	-42.78	-67.69	-37.10	-34.84	68.75	-51.76	2.233	2.639	6.147
34	168.38	-138.54	99.34	-20.31	26.15	-75.32	-40.85	-9.34	-68.13	174.00	2.505	2.543	6.193
35	-175.71	-83.61	154.60	35.99	-75.94	-48.00	13.91	-29.92	66.45	-54.37	2.248	2.746<br>2.769	6.948
36	169.79	-137.33	100.59	-19.13	20.86	-72.95	-43.65	34.01	-70.95	169.33	2.527	2.469	6.848
42	171.32	-140.43	99.59	-19.95	46.38	53.02	51.59	-29.84	75.13	-45.87	2.348	2.285	6.553
43	166.99	-141.00	97.03	-22.60	26.24	-75.51	-41.42	-2.17	-71.84	170.00	2.539	2.518	6.512
45	177.77	-96.92	142.89	22.26	-43.83	-49.54	-53.40	-32.83	66.41	-54.64	2.370	2.291	7.053
46	167.73	-138.12	99.67	-19.99	24.73	-75.37	-40.50	-31.77	63.90	-57.25	2.478	2.518	7.050
48	-178.34	-104.11	136.02	16.10	-55.77	-42.67	-52.17	32.13	69.91	-52.46	2.380	2.289	6.970
58	-169.15	-101.51	136.13	18.18	73.13	-21.45	42.64	31.21	72.62	-50.14	2.495	2.488	7.038

59	165.70	-145.43	92.75	-26.82	30.83	-79.08	-38.62	-17.25	-93.32	149.16	2.528	2.561	6.080
60	178.72	151.46	29.72	-87.07	63.09	43.88	41.92	39.01	-79.16	-161.27	2.379<br>2.630	2.419	6.366
61	174.32	-149.93	90.35	-28.36	48.46	46.75	53.98	-24.25	-94.94	147.68	2.356	2.339	6.082
69	174.69	-31.83	-153.83	84.92	-41.20	-65.14	-42.28	31.60	-70.72	169.68	2.334<br>2.600	2.477	6.539
70	-179.12	-133.86	165.63	-14.00	44.94	44.78	55.48	38.20	71.54	-51.70	2.455	2.224	7.088
71	175.80	-136.59	101.13	-18.86	29.60	-78.87	-35.95	37.03	-176.64	63.35	2.390	2.602	8.047
73	169.68	-142.03	96.02	-23.50	28.27	-77.83	-38.19	28.69	-74.36	166.25	2.475	2.538	6.750
76	175.80	-131.67	106.04	-14.04	22.35	-73.32	-41.95	37.50	-173.58	66.38	2.450	2.537	8.094
77	173.20	-30.87	-152.98	85.72	-41.35	-62.98	-43.48	37.37	-72.40	167.88	2.346<br>2.583	2.433	6.801
93	-171.47	-110.74	127.12	9.40	67.41	-12.86	47.64	30.43	-177.33	62.56	2.513	2.455	8.044

[a] Conformers are numbered according to their appearance during conformational search; [b] – α = C_{Tr}-C(=O)-O-C*; [c] – β =O=C-C-C_{ipso}; [d] – γ = (O=)C-C_{Tr}-C_{ipso}-C_{ortho} (of the two possibilities the absolute values ≤90° has been chosen); [e] – δ = C(=O)-O-C*-H; [f] – ζ = O-C*-C-O [g] φ = C3-C2-C1-O; [h] – l_1 = C=O···HC_{ortho}; [i] – l_2 = (O=)CO···HC_{ortho}; [j] – l_3 = C_{Tr}···C_{Tr}

Table SI_43. Dihedral angles α, β, γ, δ, ζ and φ (in degrees) and selected interatomic distances l_1, l_2 and l_3 (in Å) calculated at the B3LYP-GD3Bj/6-311G(d,p) level for individual low-energy conformers of **18**.

Conf. no[a]	α[b]	β_1[c]	β_2[c]	β_3[c]	γ_1[d]	γ_2[d]	γ_3[d]	δ[e]	ζ[f]	φ[g]	l_1[h]	l_2[i]	l_3[j]
13	178.87	-124.44	115.79	-5.43	41.24	55.96	52.18	-31.03	58.27	-60.96	2.449	2.193	6.373
15	-178.91	-160.98	79.26	-40.03	55.75	54.79	31.86	-8.65	-60.05	-177.91	2.108<br>2.184	2.459<br>2.795	5.530
17	-176.49	-88.55	152.19	30.43	-42.43	-43.43	-57.50	-33.15	60.12	-59.23	2.405	2.313	6.402
26	-176.82	-100.93	138.50	20.13	-89.55	-25.65	36.71	42.10	58.71	-61.76	2.415	2.469<br>2.870	6.356
32	173.34	-45.27	-166.80	71.79	-32.61	-69.44	-41.80	-35.66	59.20	-59.40	2.215<br>2.597	2.666	5.484
35	-162.83	-93.51	144.47	25.68	-98.53	-26.44	39.92	-18.76	58.32	-61.08	2.523	2.443	6.367
36	164.14	-134.70	103.66	-16.27	24.78	-74.78	-42.03	39.67	-64.04	177.03	2.478	3.080	6.309
46	177.96	-140.51	97.87	-22.92	44.00	77.44	-15.95	-23.16	59.13	-60.19	2.193	2.597<br>2.801	6.520
60	177.51	169.98	49.43	-67.90	61.82	43.77	42.90	-1.30	-73.62	168.68	2.220<br>2.634	2.546	5.473
63	-173.50	159.71	37.93	-79.15	70.38	34.87	40.17	176.18	-62.93	168.90	2.266<br>2.541	2.605	5.430
77	173.13	-115.82	123.65	4.58	-47.16	-40.86	-55.62	-21.92	-58.47	-176.21	2.509<br>2.389	2.160	5.482

[a] Conformers are numbered according to their appearance during conformational search; [b] – α = C_{Tr}-C(=O)-O-C*; [c] – β =O=C-C-C_{ipso}; [d] – γ = (O=)C-C_{Tr}-C_{ipso}-C_{ortho} (of the two possibilities the absolute values ≤90° has been chosen); [e] – δ = C(=O)-O-C*-H; [f] – ζ = O-C*-C-O [g] φ = C3-C2-C1-O; [h] – l_1 = C=O···HC_{ortho}; [i] – l_2 = (O=)CO···HC_{ortho}; [j] – l_3 = C_{Tr}···C_{Tr}

Table SI_44. Dihedral angles α, β, γ, δ, ζ and φ (in degrees) and selected interatomic distances l_1, l_2 and l_3 (in Å) calculated at the M06-2X/6-311G(d,p) level for individual low-energy conformers of **18**.

Conf. no[a]	α[b]	β_1[c]	β_2[c]	β_3[c]	γ_1[d]	γ_2[d]	γ_3[d]	δ[e]	ζ[f]	φ[g]	l_1[h]	l_2[i]	l_3[j]
1	176.05	-138.44	100.23	-21.27	43.39	77.80	-22.21	-28.71	58.98	-59.40	2.228	2.556<br>2.791	6.356
5	179.11	-130.46	109.77	-11.03	40.51	61.20	51.34	-7.25	-66.20	176.51	2.393<br>2.528	2.270	6.002
13	179.61	-122.66	117.50	-3.43	41.80	55.63	52.95	-34.83	58.68	-59.97	2.459<br>2.891	2.183	6.308
15	175.98	-137.06	103.19	-17.42	45.54	63.40	41.42	-24.92	-62.12	-179.13	2.213<br>2.265	2.330	5.475
17	-174.94	-86.10	154.65	32.70	-41.98	-41.45	-59.48	-35.72	60.95	-58.11	2.421	2.316	6.341
18	163.44	-143.15	95.41	-24.96	49.94	74.94	-17.08	-20.87	69.11	-49.71	2.212	2.512	6.001
28	173.77	-157.57	83.27	-36.09	50.44	55.01	45.82	34.82	-65.21	175.38	2.220	2.380<br>2.669	6.059
32	174.99	-34.56	-156.64	82.10	-33.22	-75.95	-39.40	-42.85	62.67	-56.12	2.277<br>2.479	2.712	5.493
36	158.52	-134.89	103.70	-15.81	21.77	-72.34	-44.33	45.92	-61.71	179.88	2.507<br>2.979	2.497	6.230
42	-175.12	-113.95	125.80	3.81	42.42	55.80	51.09	-33.01	69.16	-48.44	2.483<br>2.709	2.179	6.077
73	161.11	-140.19	98.39	-21.15	29.04	-77.96	-38.50	28.76	-67.40	174.53	2.443	2.543	6.281

[a] Conformers are numbered according to their appearance during conformational search; [b] – α = C_{Tr}-C(=O)-O-C*; [c] – β =O=C-C-C_{ipso}; [d] – γ = (O=)C-C_{Tr}-C_{ipso}-C_{ortho} (of the two possibilities the absolute values ≤90° has been chosen); [e] – δ = C(=O)-O-C*-H; [f] – ζ = O-C*-C-O [g] φ = C3-C2-C1-O; [h] – l_1 = C=O···HC_{ortho}; [i] – l_2 = (O=)CO···HC_{ortho}; [j] – l_3 = C_{Tr}···C_{Tr}

Table SI_45. Dihedral angles *α*, *β*, *γ*, *δ*, ζ and *φ* (in degrees) and selected interatomic distances l_1, l_2 and l_3 (in Å) calculated at the B3LYP/6-311G(d,p) level for individual low-energy conformers of **20**.

Conf. no[a]		*α*[b]	β_1[c]	β_2[c]	β_3[c]	γ_1[d]	γ_2[d]	γ_3[d]	*δ*[e]	*ζ*[f]	*φ*[g]	l_1[h]	l_2[i]	l_3[j]
1	C2	172.66	-139.32	98.37	-21.69	39.12	86.32	-24.84	41.43	67.18	-178.46	2.284	2.772<br>2.872	6.934
	C3	-172.52	-100.32	137.36	19.14	69.89	-17.33	45.32	37.64			2.557	2.407	
11	C2	-178.80	-97.27	140.37	22.65	82.94	-34.29	31.76	-1.66	65.15	-177.47	2.350	2.644	6.955
	C3	-178.80	-97.27	140.37	22.65	82.94	-34.29	31.76	-1.66			2.350	2.644	
14	C2	166.25	-153.58	64.94	-34.55	44.27	79.40	-19.31	-9.89	61.72	179.80	2.293	2.698<br>2.814	6.948
	C3	-178.24	-99.81	138.21	20.47	81.23	-31.33	33.94	0.74			2.366	2.615	
16	C2	169.98	-148.55	89.33	-30.34	46.63	79.11	-19.09	36.03	70.16	-175.73	2.255	2.760<br>2.824	7.108
	C3	169.98	-148.55	89.33	-30.34	46.63	79.11	-19.09	36.03			2.255	2.760<br>2.824	
17	C2	174.76	-107.48	132.16	12.53	-46.90	-45.80	-54.42	-7.85	61.60	179.23	2.441	2.220	6.881
	C3	-177.44	-95.71	141.76	24.14	85.47	-37.76	28.46	-4.04			2.317	2.698	
20	C2	170.41	-152.00	88.83	-30.40	47.71	53.31	50.70	-11.41	62.24	179.73	2.287	2.372	6.957
	C3	-178.35	-97.41	140.49	22.69	84.39	-34.62	30.69	-2.53			2.337	2.651	
21	C2	177.06	-116.52	123.38	4.53	-62.24	-39.51	-50.26	35.99	66.81	-179.59	2.417	2.339	7.012
	C3	174.52	-133.34	104.15	-16.00	32.11	-80.78	-34.10	40.20			2.333	2.707	
22	C2	-173.38	-105.17	132.07	14.44	68.78	-17.59	45.56	32.82	66.43	-178.84	2.515	2.464	7.012
	C3	-178.67	-112.85	126.24	4.71	45.70	58.75	47.28	36.45			2.437<br>2.762	2.264	
23	C2	172.20	-27.29	-149.55	89.12	-38.67	-64.05	-44.78	39.37	-71.03	50.24	2.395<br>2.489	2.424	6.535
	C3	165.09	-142.78	95.18	-24.27	29.53	-78.32	-37.90	34.16			2.472	2.569	
25	C2	171.23	-153.21	87.49	-31.57	49.79	55.71	47.32	25.88	-71.67	49.17	2.240	2.403<br>2.806	6.344

	C3	173.37	-140.53	98.19	-18.32	-62.93	-46.15	-39.89	41.08			2.420<br>2.526	2.358	
43	C2	161.60	-142.83	95.61	-23.76	23.89	-74.70	-42.24	-0.27	-75.86	47.05	2.571	2.466	6.263
	C3	175.78	152.54	30.94	-85.74	62.97	43.10	43.37	39.20			2.369<br>2.629	2.426	
46	C2	166.27	-140.24	97.74	-21.89	26.81	-76.09	-40.65	-2.69	-68.30	46.26	2.513	2.539	6.087
	C3	-171.41	161.65	39.71	-77.16	64.83	39.77	42.54	175.19			2.259<br>2.674	2.534	
47	C2	171.77	-28.88	-152.14	86.80	-43.39	-63.61	-42.08	29.21	-71.18	50.58	2.351<br>2.629	2.429	6.241
	C3	171.77	-28.89	-152.15	86.79	-43.39	-63.61	-42.09	29.21			2.351<br>2.629	2.429	

[a] Conformers are numbered according to their appearance during conformational search; [b] – α = C_{Tr}-C(=O)-O-C*; [c] – β =O=C-C-C_{ipso}; [d] – γ = (O=)C-C_{Tr}-C_{ipso}-C_{ortho} (of the two possibilities the absolute values ≤90° has been chosen); [e] – δ = C(=O)-O-C*-H; [f] – ζ = O-C*-C*-O; [g] φ = C1-C2-C3-C4; [h] – l_1 = C=O···HC_{ortho}; [i] – l_2 = (O=)CO···HC_{ortho}; [j] – l_3 = C_{Tr}···C_{Tr}

Table SI_46. Dihedral angles α, β, γ, δ, ζ and φ (in degrees) and selected interatomic distances l_1, l_2 and l_3 (in Å) calculated at the B3LYP-GD3BJ/6-311G(d,p) level for individual low-energy conformers of **20**.

Conf. no[a]		α[b]	β_1[c]	β_2[c]	β_3[c]	γ_1[d]	γ_2[d]	γ_3[d]	δ[e]	ζ[f]	φ[g]	l_1[h]	l_2[i]	l_3[j]
1	C2	167.77	-131.03	106.50	-13.82	20.51	-69.73	-44.34	41.72	58.94	178.09	2.484	2.504	6.365
	C3	-177.17	-102.28	136.67	18.30	76.83	-22.97	40.18	42.18			2.462	2.447	
11	C2	-174.72	-101.67	136.82	18.80	80.19	-30.21	35.49	-12.38	61.63	-176.83	2.383	2.580	6.503
	C3	-174.72	-101.67	136.82	18.80	80.19	-30.21	35.49	-12.38			2.383	2.580	
14	C2	168.41	-151.17	88.12	-32.22	45.92	74.84	-16.34	-6.42	55.83	177.72	2.243	2.595; 2.731	6.439
	C3	-171.61	-98.17	139.92	21.91	78.12	-27.78	38.24	-8.60			2.451	2.507	
17	C2	-172.80	-101.13	136.73	18.88	78.66	-31.11	35.97	-10.47	58.79	179.74	2.394	2.604	6.489
	C3	-179.49	-103.68	136.98	16.61	-49.48	-38.71	-56.81	-16.16			2.467	2.185; 2.697	
20	C2	170.98	-143.30	97.91	-22.22	43.58	55.81	52.07	-9.17	54.83	175.89	2.349	2.261; 2.750	6.422
	C3	-171.05	-98.52	139.65	21.58	78.94	-28.17	37.85	-10.24			2.441	2.520	
21	C2	174.84	-106.96	134.38	14.18	-63.58	-36.50	-51.28	43.03	58.95	177.08	2.378	2.335; 2.681	6.444
	C3	169.53	-130.21	107.63	-12.68	23.45	-73.59	-42.14	42.53			2.441	2.562	
22	C2	-171.10	-109.69	128.49	10.09	63.31	-8.50	50.52	37.46	63.12	-178.82	2.582	2.356	6.581
	C3	-177.19	-125.47	114.77	-6.57	49.08	52.55	51.03	42.72			2.424	2.190	
25	C2	172.25	-153.09	87.55	-31.83	52.23	56.65	43.49	18.21	-67.77	54.25	2.203	2.365; 2.812	5.962
	C3	174.59	-135.69	103.46	-13.53	-63.76	-45.19	-39.77	38.51			2.360<br>2.580	2.321	
43	C2	163.47	-125.42	113.81	-4.84	-28.36	-51.38	-58.12	-5.82	-68.94	56.19	2.661<br>2.784<br>2.256	2.149	5.427
	C3	177.06	170.74	50.25	-67.14	62.12	43.48	42.32	-2.97			2.213<br>2.571	2.565	

[a] Conformers are numbered according to their appearance during conformational search; [b] – α = C_{Tr}-C(=O)-O-C*; [c] – β =O=C-C-C_{ipso}; [d] – γ = (O=)C-C_{Tr}-C_{ipso}-C_{ortho} (of the two possibilities the absolute values ≤90° has been chosen); [e] – δ = C(=O)-O-C*-H; [f] – ζ = O-C*-C-C(*); [g] φ = C1-C2-C3-C4; [h] – l_1 = C=O···HC_{ortho}; [i] – l_2 = (O=)CO···HC_{ortho}; [j] – l_3 = C_{Tr}···C_{Tr}

Table SI_47. Dihedral angles *α*, *β*, *γ*, *δ*, ζ and *φ* (in degrees) and selected interatomic distances l_1, l_2 and l_3 (in Å) calculated at the M06-2X/6-311G(d,p) level for individual low-energy conformers of **20**.

Conf. no[a]		*α*[b]	$β_1$[c]	$β_2$[c]	$β_3$[c]	$γ_1$[d]	$γ_2$[d]	$γ_3$[d]	*δ*[e]	*ζ*[f]	*φ*[g]	l_1[h]	l_2[i]	l_3[j]
1	C2	164.40	-134.61	102.81	-16.60	23.57	-72.01	-42.50	45.86	61.12	-178.64	2.469	2.488	6.342
	C3	179.84	-102.44	136.56	18.45	82.10	-28.29	35.98	45.92			2.396	2.508	
11	C2	-174.82	-101.63	137.59	19.06	80.13	-28.05	38.08	-15.21	63.24	173.78	2.413	2.525	6.499
	C3	-174.82	-101.63	137.59	19.06	80.13	-28.05	38.08	-15.21			2.413	2.525	
14	C2	167.32	-148.32	90.93	-29.56	45.71	75.15	-18.66	-11.98	55.61	178.88	2.263	2.547; 2.745	6.364
	C3	-171.39	-93.72	144.20	25.88	79.26	-30.31	36.51	-13.37			2.446	2.479	
17	C2	-173.09	-98.59	139.39	21.14	78.50	-31.72	36.28	-15.23	58.98	-178.80	2.407	2.564	6.426
	C3	-178.91	-101.16	139.66	19.08	-49.32	-35.59	-58.40	-19.89			2.474	2.185; 2.569	
20	C2	169.39.	-140.34	100.69	-19.96	44.62	57.84	51.21	-14.86	54.26	176.46	2.349	2.249	6.341
	C3	-170.42	-94.09	143.96	25.64	80.54	-31.07	36.13	-14.05			2.437	2.506	
21	C2	173.57	-109.88	131.50	11.69	-65.50	-35.40	-51.55	48.16	62.25	-178.77	2.419	2.546	6.421
	C3	167.00	-134.16	103.95	-16.31	26.32	-77.11	-39.86	46.44			2.419	2.546	
22	C2	-173.90	-120.41	119.99	-2.12	48.65	54.45	50.55	42.87	63.95	-176.54	2.436	2.181	6.570
	C3	-171.48	-116.81	121.33	3.18	60.69	-4.82	52.17	36.97			2.533	2.369	
23	C2	156.10	-138.32	100.54	-19.08	24.16	-74.25	-44.02	35.86	-66.33	56.45	2.526	2.513	6.208
	C3	178.43	-21.22	-142.85	96.13	-43.86	-60.90	-44.94	34.60			2.452; 2.476	2.294	
25	C2	171.62	-155.62	85.11	-34.32	52.33	56.83	43.77	21.84	-68.11	54.03	2.202	2.376; 2.746	5.972
	C3	174.96	-138.36	101.05	-16.15	-64.82	-44.40	-39.43	42.08			2.363; 2.522	2.334	

[a] Conformers are numbered according to their appearance during conformational search; [b] – *α* = C_{Tr}-C(=O)-O-C*; [c] – *β* =O=C-C-C_{ipso}; [d] – *γ* = (O=)C-C_{Tr}-C_{ipso}-C_{ortho} (of the two possibilities the absolute values ≤90° has been chosen); [e] – *δ* = C(=O)-O-C*-H; [f] – *ζ* = O-C*-C-C(*); [g] *φ* = C1-C2-C3-C4; [h] – l_1 = C=O···HC_{ortho}; [i] – l_2 = (O=)CO···HC_{ortho}; [j] – l_3 = C_{Tr}···C_{Tr}

Table SI_48. Dihedral angles α, β, γ, δ, ζ and φ (in degrees) and selected interatomic distances l_1, l_2 and l_3 (in Å) calculated at the B3LYP/6-311G(d,p) level for individual low-energy conformers of **21**.

Conf. no[a]		α[b]	β_1[c]	β_2[c]	β_3[c]	γ_1[d]	γ_2[d]	γ_3[d]	δ[e]	ζ[f]	φ_1[g]	φ_2[g]	l_1[h]	l_2[i]	l_3[j]	l_4[k]
1	C2	178.46	-43.61	-165.07	73.95	-45.46	-65.44	-36.42	25.79	-63.28	177.29	173.74	2.229	2.599	6.582	2.675
	C4	175.68	-137.54	102.24	-17.23	47.75	49.52	53.20	29.92	-66.28			2.386	2.264		2.681
6	C2	173.60	-133.68	103.87	-16.12	26.50	-75.40	-40.91	43.22	-63.16	176.63	172.26	2.458	2.603	6.971	
	C4	-177.87	-80.84	157.12	38.60	-75.03	-49.47	11.53	36.67	-68.01			2.242	2.740; 2.814		
12	C2	-178.30	-118.33	119.54	2.54	67.73	-11.15	48.17	-7.43	-66.36	175.50	175.07	2.457	2.502	6.521	2.427
	C4	170.11	-148.08	89.83	-29.92	39.90	-86.10	-30.92	33.59	-64.93			2.418	2.706		2.993
15	C2	168.41	-150.25	88.00	-31.68	36.96	-84.64	-32.85	-6.32	-65.41	176.37	175.68	2.469	2.605	6.536	2.416
	C4	168.88	-147.14	90.70	-29.02	38.32	-83.93	-34.13	30.16	-64.40			2.466	2.696		2.744
31	C2	170.96	-137.00	100.91	-18.95	25.20	-75.93	-39.77	-30.37	-178.60	63.80	63.94	2.453	2.535	8.400	
	C4	-172.88	-97.75	140.23	22.13	82.33	-32.88	32.94	-33.98	-178.61			2.371	2.621		
54	C2	-175.12	-109.45	128.41	10.85	71.76	-18.89	43.87	-36.64	-179.27	63.32	63.14	2.453	2.518	8.336	
	C4	176.59	-108.23	131.44	11.90	-48.17	-46.49	-53.52	-34.16	-179.18			2.432	2.226		

[a] Conformers are numbered according to their appearance during conformational search; [b] – α = C_{Tr}-C(=O)-O-C*; [c] – β =O=C-C-C_{ipso}; [d] – γ = (O=)C-C_{Tr}-C_{ipso}-C_{ortho} (of the two possibilities the absolute values ≤90° has been chosen); [e] – δ = C(=O)-O-C*-H; [f] – ζ = O-C*-C-C(*); [g] φ_1 = C1-C2-C3-C4; φ_2 = C2-C3-C4-C5; [h] – l_1 = C=O···HC_{ortho}; [i] – l_2 = (O=)CO···HC_{ortho}; [j] – l_3 = C_{Tr}···C_{Tr}; [k] – l_4 = C=O HC_{ortho} of the second trityl group.

Table SI_49. Dihedral angles *α*, *β*, *γ*, *δ*, ζ and *φ* (in degrees) and selected interatomic distances l_1, l_2 and l_3 (in Å) calculated at the B3LYP-GD3BJ/6-311G(d,p) level for individual low-energy conformers of **21**.

Conf. no[a]		*α*[b]	$β_1$[c]	$β_2$[c]	$β_3$[c]	$γ_1$[d]	$γ_2$[d]	$γ_3$[d]	*δ*[e]	*ζ*[f]	$φ_1$[g]	$φ_2$[g]	l_1[h]	l_2[i]	l_3[j]
1	C2	179.12	-145.64	94.60	-25.02	50.87	51.46	47.58	45.60	-57.55	178.20	-177.81	2.272	2.279	6.248
	C4	-172.62	-84.91	152.76	34.56	-86.04	-39.98	26.47	49.00	-57.42			2.377	2.571	
6	C2	178.53	-83.94	156.31	34.98	-44.62	-53.09	-52.45	43.78	-60.44	179.67	-177.49	2.301	2.425	6.336
	C4	-176.98	-72.10	166.32	47.23	-67.36	-56.53	-3.63	41.16	-57.60			2.181	2.744	
16	C2	-173.26	-133.92	105.29	-11.82	67.68	4.56	51.04	30.44	-52.69	-173.23	-173.58	2.395	2.430	6.123
	C4	-164.30	-85.58	153.09	34.00	-71.13	-54.87	9.05	-179.56	-46.73			2.197	2.553	

[a] Conformers are numbered according to their appearance during conformational search; [b] – $α$ = C_{Tr}-C(=O)-O-C*; [c] – $β$ =O=C-C-C_{ipso}; [d] – $γ$ = (O=)C-C_{Tr}-C_{ipso}-C_{ortho} (of the two possibilities the absolute values ≤90° has been chosen); [e] – $δ$ = C(=O)-O-C*-H; [f] – $ζ$ = O-C*-C-C(*); [g] $φ_1$ = C1-C2-C3-C4; $φ_2$ = C2-C3-C4-C5; [h] – l_1 = C=O···HC_{ortho}; [i] – l_2 = (O=)CO···HC_{ortho}; [j] – l_3 = C_{Tr}···C_{Tr}

Table SI_50. Dihedral angles *α*, *β*, *γ*, *δ*, ζ and *φ* (in degrees) and selected interatomic distances l_1, l_2 and l_3 (in Å) calculated at the M06/6-311G(d,p) level for individual low-energy conformers of **21**.

Conf. no[a]		*α*[b]	*β*$_1$[c]	*β*$_2$[c]	*β*$_3$[c]	*γ*$_1$[d]	*γ*$_2$[d]	*γ*$_3$[d]	*δ*[e]	*ζ*[f]	*φ*$_1$[g]	*φ*$_2$[g]	*l*$_1$[h]	*l*$_2$[i]	*l*$_3$[j]	*l*$_4$[k]
1	C2	-178.71	-68.65	171.59	50.16	28.66	-59.24	-38.68	42.07	-58.53	-177.90	-178.79	2.151	2.642; 2.716	6.237	2.753
	C4	177.33	-146.40	93.39	-24.97	48.26	37.28	61.41	42.28	-58.70			2.500	2.299		2.551
12	C2	-169.25	-98.71	140.16	21.15	79.52	-29.32	38.02	-20.45	-64.67	178.08	179.01	2.431	2.538	6.037	2.477
	C4	170.20	-156.17	82.72	-37.45	43.91	80.16	-28.85	34.35	-61.50			2.431	2.658; 2.808		2.731
15	C2	158.27	-143.33	95.40	-25.00	34.43	-79.03	-43.10	15.13	-56.44	-175.04	-175.04	2.584	2.650	5.972	2.358; 2.412
	C4	158.29	-143.33	95.39	-25.00	34.43	-79.02	-43.10	15.10	-56.45			2.584	2.650		2.357; 2.413
16	C2	-159.19	-91.76	146.28	27.42	-83.23	-45.21	25.35	-179.63	-41.16	-167.19	-172.22	2.323	2.785; 2.795	6.184	
	C4	177.25	-140.53	99.72	-20.10	44.92	51.34	55.45	38.95	-51.93			2.410	2.245; 2.782		2.586
25	C2	-162.13	-118.77	119.59	0.99	57.75	-1.17	54.41	38.92	-59.63	179.94	171.15	2.555	2.364	6.614	
	C4	171.23	-133.38	104.93	-14.97	26.05	-72.61	-41.38	-179.90	-61.56			2.438	2.544		
35	C2	-169.22	-97.80	142.12	20.94	-39.78	-53.92	-55.54	-179.08	-47.16	-174.22	-170.51	2.387	2.239	6063	
	C4	-173.28	-142.14	97.33	-20.57	64.20	11.59	50.42	38.34	-49.86			2.340	2.420		
46	C2	173.24	-137.30	101.63	-18.86	30.31	-79.14	-37.10	-176.18	-47.38	-174.36	-174.36	2.399	2.569	6.426	
	C4	173.24	-137.30	101.63	-18.86	30.31	-79.14	-37.10	-176.18	-47.38			2.399	2.569		
61	C2	-175.82	-138.30	101.98	-18.18	43.07	43.59	58.66	-175.88	-42.43	-169.01	-172.09	2.476	2.204	6.306	
	C4	172.34	-141.32	96.01	-23.34	35.77	-77.86	-34.22	-177.80	-44.71			2.399	2.615		

[a] Conformers are numbered according to their appearance during conformational search; [b] – *α* = C_{Tr}-C(=O)-O-C*; [c] – *β* =O=C-C-C_{ipso}; [d] – *γ* = (O=)C-C_{Tr}-C_{ipso}-C_{ortho} (of the two possibilities the absolute values ≤90° has been chosen); [e] – *δ* = C(=O)-O-C*-H; [f] – *ζ* = O-C*-C-C(*); [g] *φ*$_1$ = C1-C2-C3-C4; *φ*$_2$ = C2-C3-C4-C5; [h] – l_1 = C=O···HC_{ortho}; [i] – l_2 = (O=)CO···HC_{ortho}; [j] – l_3 = C_{Tr}···C_{Tr}; [k] – l_4 = C=O···HC_{ortho} of the second trityl group.

Table SI_51. Dihedral angles α, β, γ, δ, ζ and φ (in degrees) and selected interatomic distances l_1, l_2 and l_3 (in Å) calculated at the B3LYP/6-311G(d,p) level for individual low-energy conformers of **22**.

Conf. no[a]		α[b]	β_1[c]	β_2[c]	β_3[c]	γ_1[d]	γ_2[d]	γ_3[d]	δ[e]	ζ[f]	φ_1[g]	φ_2[g]	φ_3[g]	l_1[h]	l_2[i]	l_3[j]
1	C2	-173.95	-108.33	129.42	11.78	69.88	-16.18	45.32	31.81	-67.14	172.52	-177.78	172.52	2.483	2.472	7.717
	C5	-173.95	-108.33	129.42	11.78	69.88	-16.18	45.32	31.81	-67.14				2.483	2.472	
3	C2	-173.93	-108.37	129.38	11.74	69.89	-16.19	45.30	31.67	-67.09	172.57	-177.77	172.57	2.482	2.473	7.716
	C5	-173.93	-108.37	129.38	11.74	69.90	-16.19	45.29	31.67	-67.09				2.482	2.473	
5	C2	178.20	-109.03	128.83	7.38	8.51	-66.29	-49.07	42.56	-62.38	177.31	179.45	175.59	2.400	2.585	7.744
	C5	-172.65	-101.74	136.06	17.99	71.31	-19.01	44.07	36.15	-64.04				2.520	2.445	
7	C2	169.31	-156.92	81.50	-37.94	49.63	75.11	-13.33	19.01	-69.45	171.21	-176.95	172.27	2.250	2.769; 2.770	7.872
	C5	-174.61	-107.13	130.52	12.96	71.11	-18.85	43.85	32.52	-67.41				2.470	2.498	
8	C2	171.73	-139.53	98.47	-21.32	28.00	-78.23	-37.39	-31.32	-173.60	68.32	170.03	172.12	2.444	2.548	7.208
	C5	169.69	-148.94	89.03	-30.47	44.08	81.46	-21.57	-4.26	-69.68				2.283	2.758; 2.822	
9	C2	-174.83	-104.66	132.98	15.21	72.11	-20.20	42.91	33.72	-66.87	173.03	-73.79	173.03	2.467	2.495	9.051
	C5	-174.83	-104.66	132.98	15.21	72.11	-20.20	42.91	33.72	-66.87				2.467	2.495	
10	C2	164.76	-137.37	100.60	-19.23	28.29	-77.50	-38.58	-28.11	56.82	-62.88	-174.57	-62.88	2.456	2.589	6.717
	C5	164.76	-137.37	100.60	-19.23	28.29	-77.50	-38.58	-28.11	56.82				2.456	2.589	
11	C2	177.57	-122.43	117.00	-1.05	-60.65	-43.41	-47.51	35.50	-68.28	171.69	177.85	173.73	2.409; 2.837	2.297	7.575
	C5	-174.36	-109.20	128.24	10.89	70.29	-18.71	44.40	30.25	-66.13				2.461	2.522	
12	C2	177.07	-178.56	61.37	-56.59	60.22	46.67	38.87	33.49	-68.96	171.15	174.86	65.94	2.153	2.701; 2.803	7.401
	C5	-171.24	-102.57	135.31	17.33	73.37	-22.76	42.36	-30.89	-176.36				2.490	2.503	
13	C2	178.53	-128.29	109.75	-6.75	64.50	-6.28	48.97	-11.67	-74.50	167.71	176.10	62.14	2.408	2.539	7.280
	C5	-171.64	-83.19	155.45	36.57	-73.15	-49.26	13.59	-12.28	179.78				2.251	2.692; 2.771	

15	C2	-174.56	-105.84	131.82	14.05	70.40	-17.55	44.76	34.93	-66.40	173.52	-71.84	176.48	2.495	2.464	9.106
	C5	170.61	-147.18	90.72	-28.99	46.86	78.96	-17.88	40.21	-63.49				2.234	2.750; 2.842	
17	C2	164.27	-133.97	103.81	-15.93	23.65	-73.23	-43.34	-22.10	57.05	-62.71	-178.69	-62.01	2.512	2.555	6.790
	C5	173.24	-139.69	100.28	-19.15	47.65	48.27	53.82	-32.21	57.74				2.399	2.262	
26	C2	170.80	-139.08	98.80	-21.08	28.93	-78.81	-36.82	-32.20	-176.12	67.28	81.67	168.77	2.421; 2.389	2.566	6.953
	C5	174.79	-45.64	-167.29	71.64	-42.34	-65.92	-37.83	36.48	-71.09				2.209	2.638	
31	C2	165.57	-143.89	94.26	-25.33	34.29	-82.86	-32.37	-23.24	61.56	-58.64	174.57	-61.59	2.396	2.630	7.768
	C5	171.09	-129.30	108.35	-11.77	18.10	-70.89	-44.96	40.15	60.20				2.474	2.509	
36	C2	179.87	-115.91	123.43	2.05	43.44	60.67	47.14	37.12	-61.95	177.86	-175.17	177.77	2.412	2.293	8.142
	C5	171.22	-139.46	98.17	-21.80	38.63	-86.07	-27.72	41.99	-61.87				2.294	2.758; 2.878	
71	C2	-177.67	-134.88	104.57	-14.80	49.14	40.59	55.14	29.51	-172.03	67.96	65.22	156.66	2.450	2.231	7.446
	C5	171.45	-42.40	-164.38	74.88	-45.93	-64.38	-38.31	32.64	-84.09				2.231	2.563	

[a] Conformers are numbered according to their appearance during conformational search; [b] – α = C_{Tr}-C(=O)-O-C*; [c] – β =O=C-C-C_{ipso}; [d] – γ = (O=)C-C_{Tr}-C_{ipso}-C_{ortho} (of the two possibilities the absolute values ≤90° has been chosen); [e] – δ = C(=O)-O-C*-H; [f] – ζ = O-C*-C-C(*); [g] φ_1 = C1-C2-C3-C4; φ_2 = C2-C3-C4-C5; φ_3 = C3-C4-C5-C6; [h] – l_1 = C=O···HC_{ortho}; [i] – l_2 = (O=)CO···HC_{ortho}; [j] – l_3 = C=O···HC_{ortho}; distance between C=O and HC_{ortho} of the second trityl group.

Table SI_52. Dihedral angles α, β, γ, δ, ζ and φ (in degrees) and selected interatomic distances l_1, l_2 and l_3 (in Å) calculated at the B3LYP-GD3BJ/6-311G(d,p) level for individual low-energy conformers of **22**.

Conf. no[a]		α[b]	β_1[c]	β_2[c]	β_3[c]	γ_1[d]	γ_2[d]	γ_3[d]	δ[e]	ζ[f]	φ_1[g]	φ_2[g]	φ_3[g]	l_1[h]	l_2[i]	l_3[j]
1	C2	-173.84	-109.63	128.33	10.75	70.07	-19.91	43.73	18.64	-66.52	174.64	175.81	174.64	2.452	2.499	6.983
	C5	-173.84	-109.63	128.33	10.75	70.07	-19.91	43.73	18.64	-66.52				2.452	2.499	
3	C2	-173.83	-109.59	128.37	10.79	70.10	-19.96	43.69	18.63	-66.54	174.63	175.84	174.64	2.452	2.500	6.983
	C5	-173.83	-109.60	128.37	10.79	70.09	-19.96	43.70	18.62	-66.53				2.452	2.500	
5	C2	173.58	-110.80	126.42	6.00	-1.18	-58.00	-54.82	39.21	-67.74	173.16	160.53	-179.30	2.501	2.434	6.580
	C5	-164.91	-108.63	130.64	10.73	59.72	6.89	54.78	39.63	-59.46				2.671	2.199	
7	C2	177.35	-146.76	93.10	-25.20	49.83	33.05	57.25	31.25	-68.52	171.75	174.25	174.54	2.417	2.309	7.098
	C5	-171.89	-107.59	130.34	12.90	73.16	-20.71	43.24	16.82	-65.78				2.456	2.500	
17	C2	167.14	-154.42	85.20	-33.69	50.43	71.04	1.58	-24.44	59.96	-58.77	-169.88	-54.53	2.141	2.575	5.819
	C5	179.07	-154.31	86.31	-32.52	46.30	41.28	62.73	-35.08	64.30				2.512	2.371	
26	C2	175.15	-138.31	99.75	-20.60	30.18	-84.63	-34.45	-43.06	-154.13	88.05	83.79	170.24	2.346	2.567	5.502
	C5	177.67	-42.65	-165.09	73.41	-10.43	-70.90	-43.44	32.80	-70.07				2.235; 2.271	2.676	

[a] Conformers are numbered according to their appearance during conformational search; [b] – α = C_{Tr}-C(=O)-O-C*; [c] – β =O=C-C-C_{ipso}; [d] – γ = (O=)C-C_{Tr}-C_{ipso}-C_{ortho} (of the two possibilities the absolute values ≤90° has been chosen); [e] – δ = C(=O)-O-C*-H; [f] – ζ = O-C*-C-C(*); [g] φ_1 = C1-C2-C3-C4; φ_2 = C2-C3-C4-C5; φ_3 = C3-C4-C5-C6; [h] – l_1 = C=O···HC_{ortho}; [i] – l_2 = (O=)CO···HC_{ortho}; [j] – l_3 = C_{Tr}···C_{Tr}

Table SI_53. Dihedral angles α, β, γ, δ, ζ and φ (in degrees) and selected interatomic distances l_1, l_2 and l_3 (in Å) calculated at the M06-2X/6-311G(d,p) level for individual low-energy conformers of **22**.

Conf. no[a]		α[b]	β_1[c]	β_2[c]	β_3[c]	γ_1[d]	γ_2[d]	γ_3[d]	δ[e]	ζ[f]	φ_1[g]	φ_2[g]	φ_3[g]	l_1[h]	l_2[i]	l_3[j]	l_4[k]
1	C2	-174.18	-116.68	121.87	4.35	69.39	-17.08	45.99	25.93	-67.18	173.93	176.69	173.93	2.433	2.514	7.011	
	C5	-174.18	-116.68	121.87	4.35	69.39	-17.08	45.99	25.93	-67.18				2.433	2.514		
3	C2	-174.17	-116.65	121.90	4.37	69.40	-17.06	45.97	25.87	-67.17	173.95	176.72	173.95	2.433	2.513	7.011	
	C5	-174.19	-116.66	121.90	4.36	69.38	-17.06	45.97	25.87	-67.16				2.433	2.513		
11	C2	178.99	-125.18	115.70	-2.99	-61.41	-36.59	-50.49	35.37	-67.06	174.04	171.84	177.15	2.470; 2.761	2.213	7.016	
	C5	-174.60	-115.08	122.80	5.49	69.95	-22.53	43.62	27.79	-64.03				2.407	2.599		
17	C2	163.40	-136.67	102.34	-17.40	20.65	-75.22	-41.18	-29.51	62.24	-56.27	-169.93	-54.20	2.444	2.468	5.904	2.691
	C5	176.45	-152.39	87.95	-30.86	45.59	38.51	65.19	-37.77	64.17				2.565	2.347; 2.648		2.815
26	C2	178.31	-135.58	102.71	-18.20	30.22	-85.98	-34.18	-45.32	-151.73	90.98	85.21	-167.92	2.320	2.569	5.417	2.339
	C5	177.68	-159.46	79.10	-37.75	-70.73	-43.80	-17.96	32.79	-71.90				2.298	2.613		

[a] Conformers are numbered according to their appearance during conformational search; [b] – α = C_{Tr}-C(=O)-O-C*; [c] – β =O=C-C-C_{ipso}; [d] – γ = (O=)C-C_{Tr}-C_{ipso}-C_{ortho} (of the two possibilities the absolute values ≤90° has been chosen); [e] – δ = C(=O)-O-C*-H; [f] – ζ = O-C*-C-C(*); [g] φ_1 = C1-C2-C3-C4; φ_2 = C2-C3-C4-C5; φ_3 = C3-C4-C5-C6; [h] – l_1 = C=O···HC_{ortho}; [i] – l_2 = (O=)CO···HC_{ortho}; [j] – l_3 = C_{Tr}···C_{Tr}; [k] – l_4 = C=O···HC_{ortho}; distance between C=O and HC_{ortho} of the second trityl group.

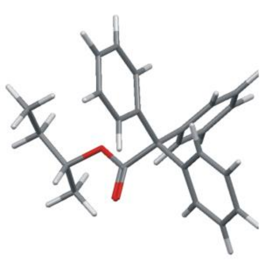

1 (conf 1)

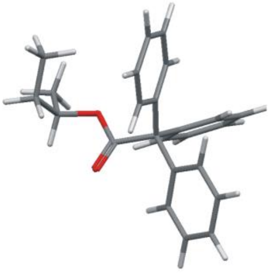

1 (conf 17)

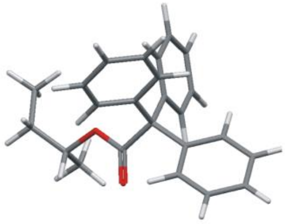

1 (conf 33)

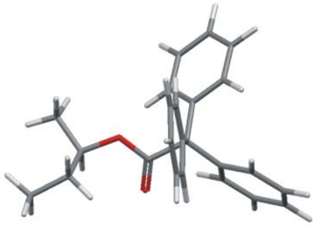

1 (conf 38)

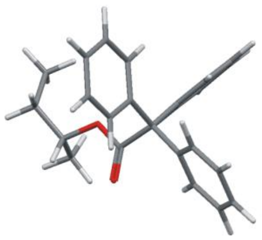

1 (conf 39)

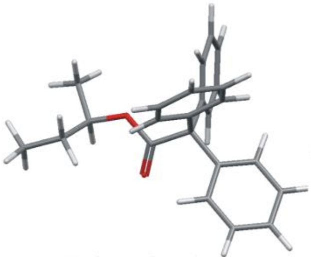

1 (conf 40)

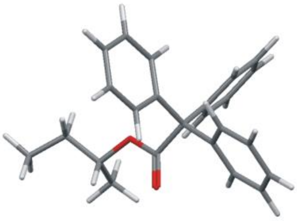

1 (conf 41)

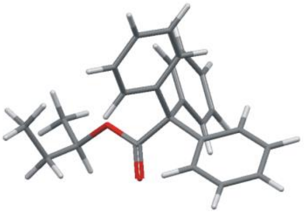

1 (conf 42)

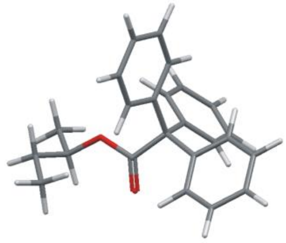

1 (conf 43)

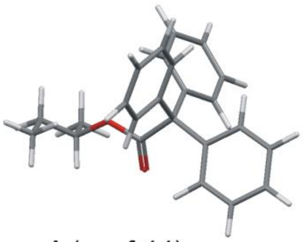

1 (conf 44)

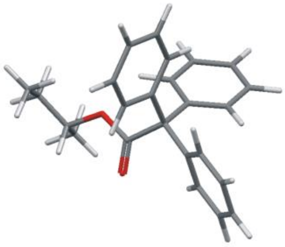

1 (conf 45)

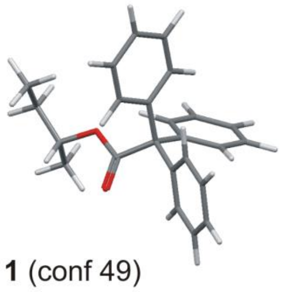

1 (conf 49)

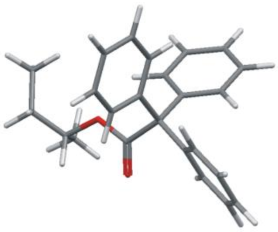

1 (conf 50)

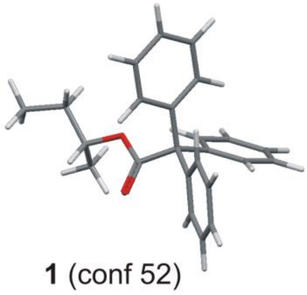

1 (conf 52)

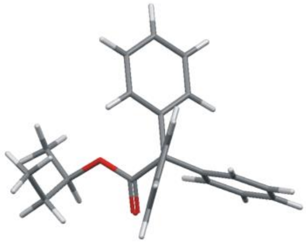

1 (conf 53)

Figure SI_1. Structures of individual, low-energy conformers of **1**, calculated at the B3LYP/6-311++G(d,p) level of theory.

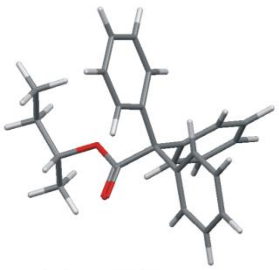

1 (conf 1)

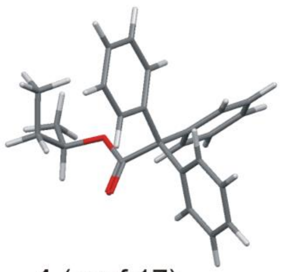

1 (conf 17)

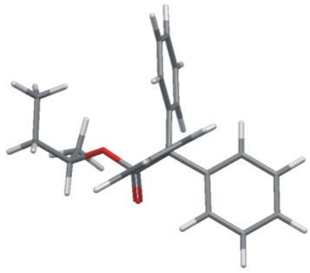

1 (conf 33)

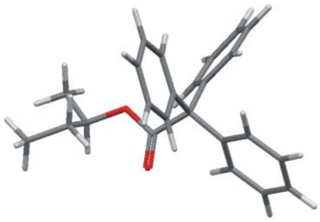

1 (conf 38)

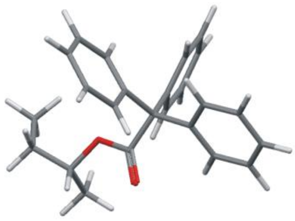

1 (conf 39)

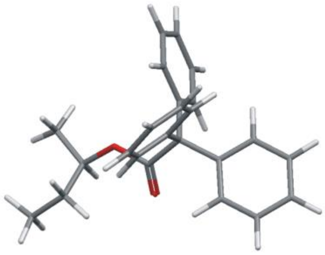

1 (conf 40)

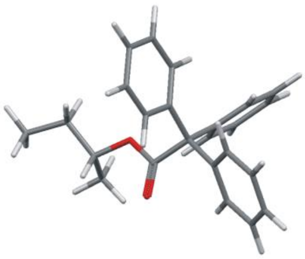

1 (conf 41)

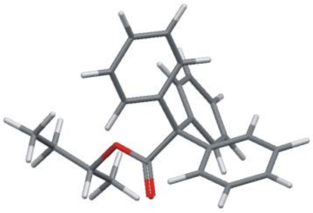

1 (conf 44)

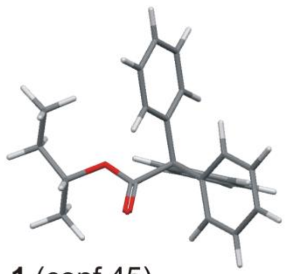

1 (conf 45)

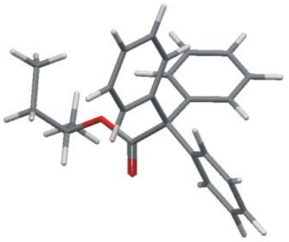

1 (conf 50)

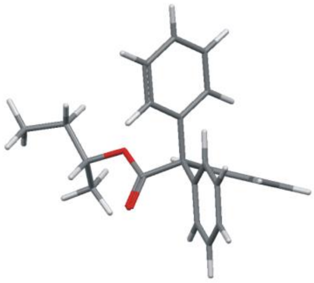

1 (conf 52)

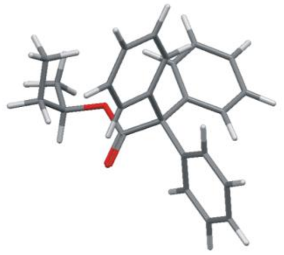

1 (conf 53)

Figure SI_2. Structures of individual, low-energy conformers of **1**, calculated at the M06-2X/6-311++G(d,p) level of theory.

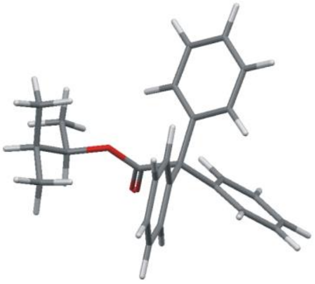

4 (conf 1)

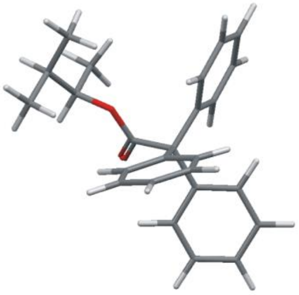

4 (conf 20)

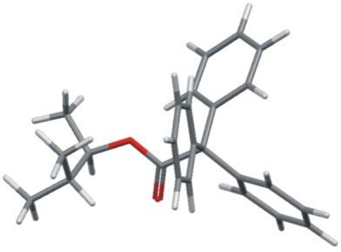

4 (conf 22)

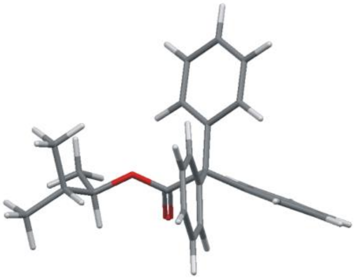

4 (conf 42)

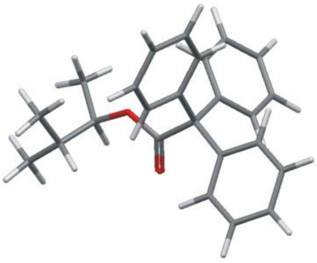

4 (conf 44)

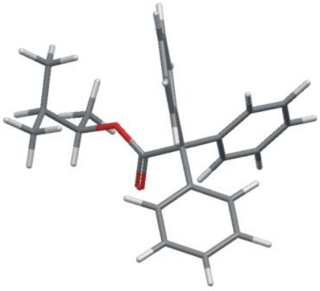

4 (conf 46)

Figure SI_3. Structures of individual, low-energy conformers of **4**, calculated at the B3LYP/6-311++G(d,p) level of theory.

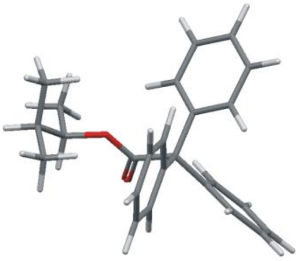

4 (conf 1)

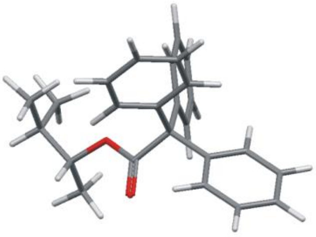

4 (conf 20)

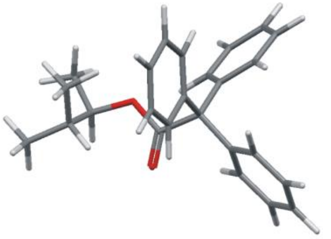

4 (conf 22)

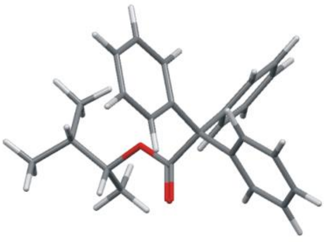

4 (conf 42)

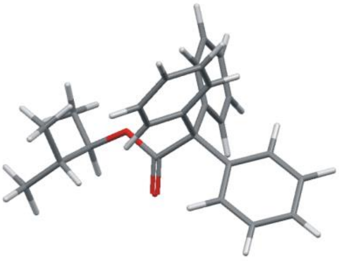

4 (conf 44)

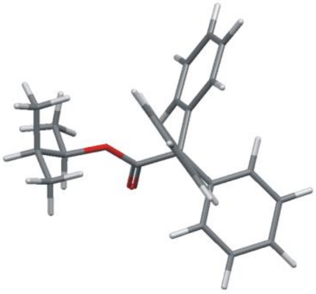

4 (conf 46)

Figure SI_4. Structures of individual, low-energy conformers of **4**, calculated at the M06-2X/6-311++G(d,p) level of theory.

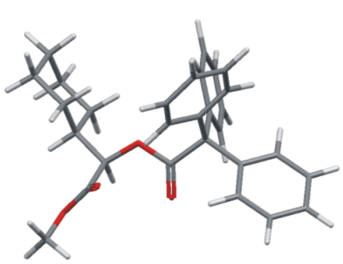

6 (conf 1)

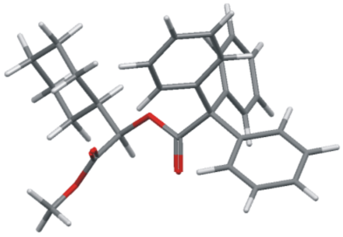

6 (conf 4)

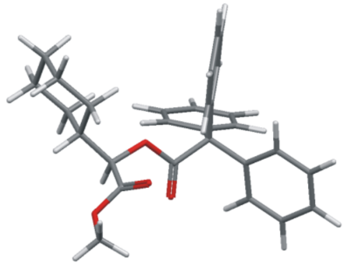

6 (conf 7)

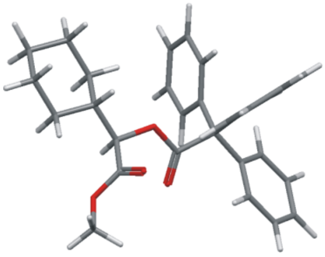

6 (conf 8)

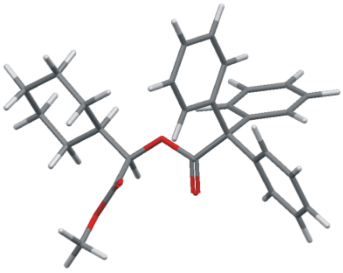

6 (conf 10)

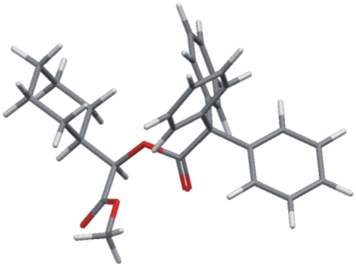

6 (conf 14)

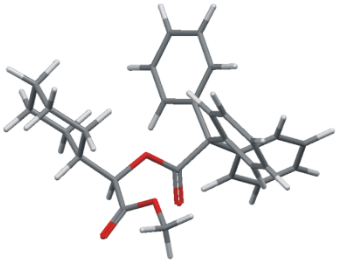

6 (conf 17)

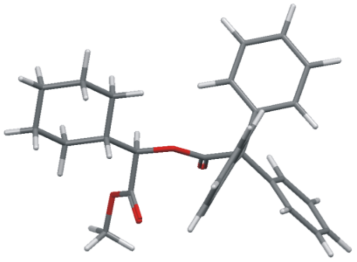

6 (conf 18)

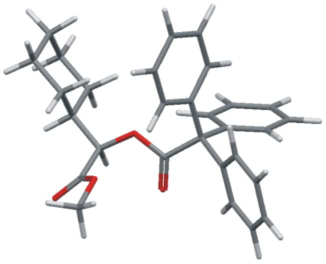

6 (conf 20)

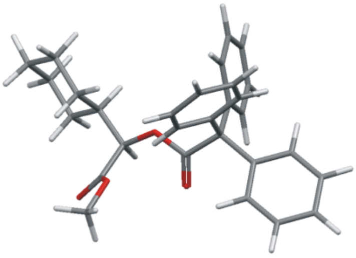

6 (conf 21)

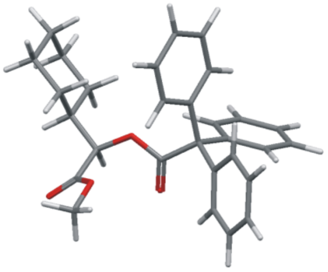

6 (conf 28)

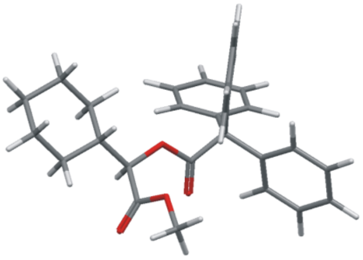

6 (conf 46)

Figure SI_5. Structures of individual, low-energy conformers of **6**, calculated at the B3LYP/6-311++G(d,p) level of theory.

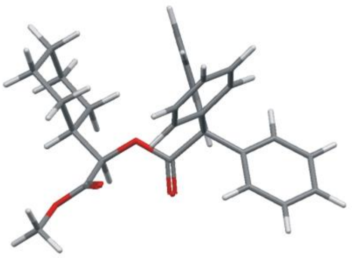

6 (conf 1)

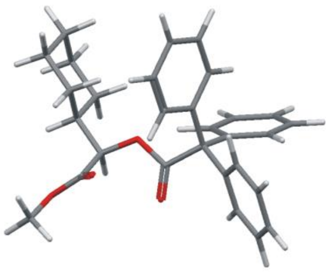

6 (conf 7)

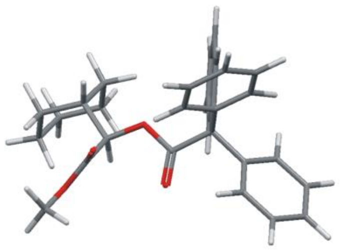

6 (conf 11)

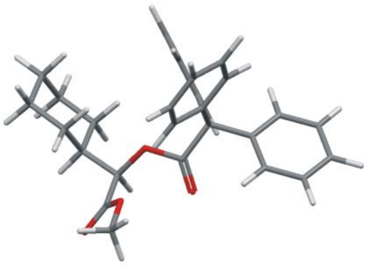

6 (conf 14)

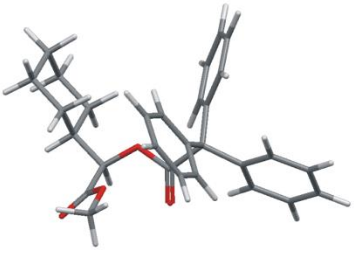

6 (conf 17)

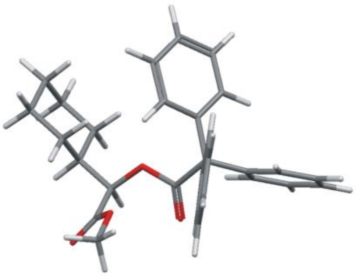

6 (conf 28)

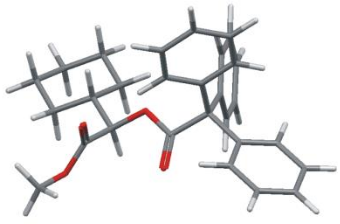

6 (conf 30)

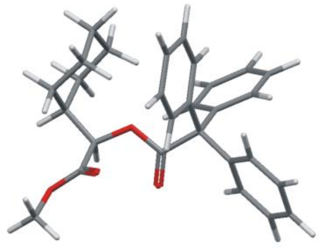

6 (conf 32)

Figure SI_6. Structures of individual, low-energy conformers of **6**, calculated at the M06-2X/6-311++G(d,p) level of theory.

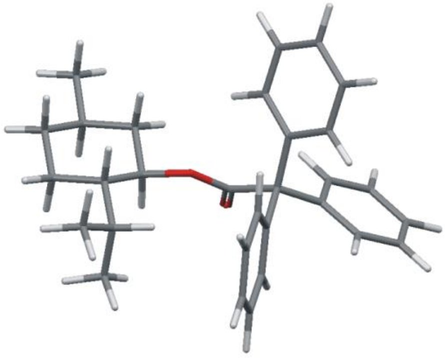

10 (conf 1)

Figure SI_7. Structure of individual, low-energy conformer of **10**, calculated at the B3LYP/6-311++G(d,p) level of theory.

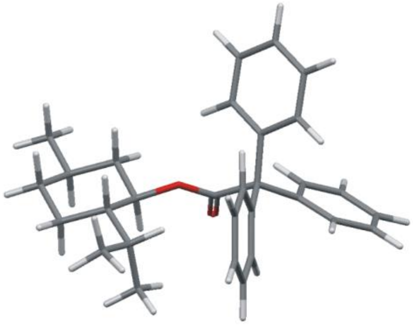

10 (conf 1)

Figure SI_8. Structure of individual, low-energy conformer of **10**, calculated at the M06-2X/6-311++G(d,p) level of theory.

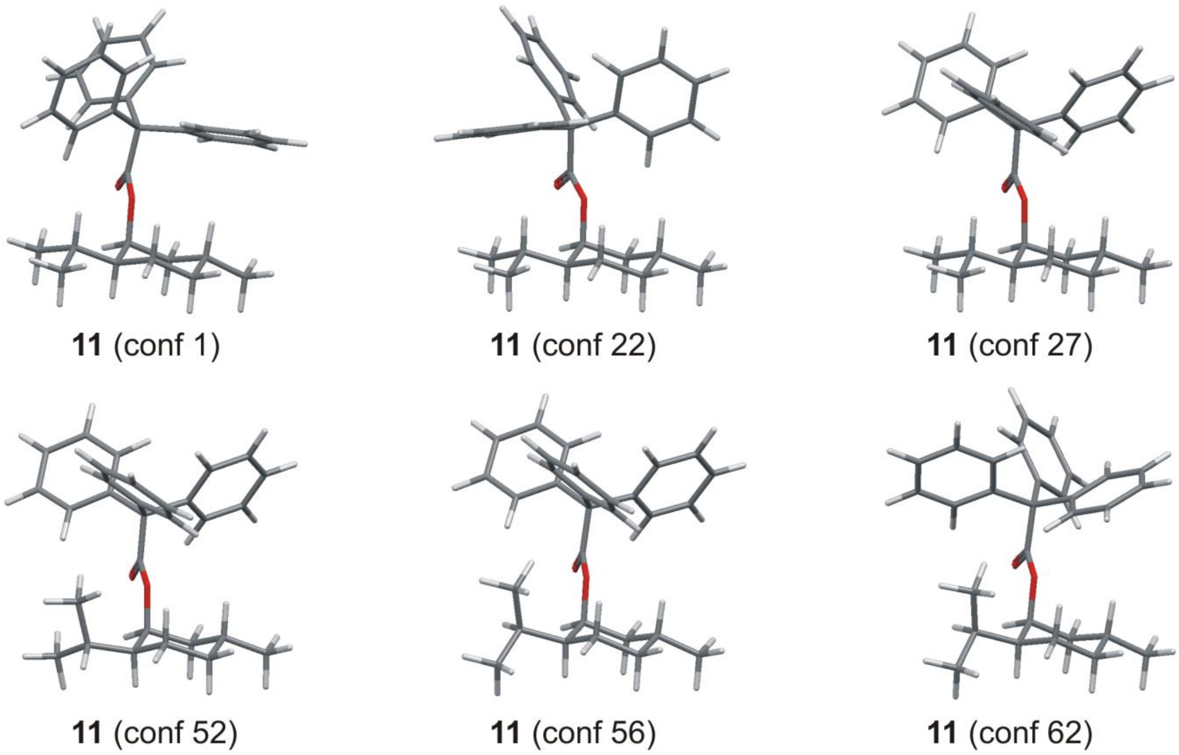

Figure SI_9. Structures of individual, low-energy conformers of **11**, calculated at the B3LYP/6-311++G(d,p) level of theory.

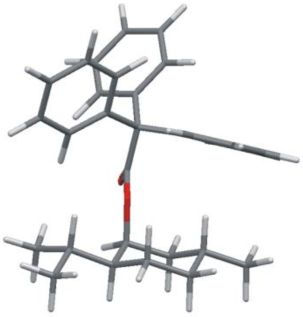

11 (conf 1)

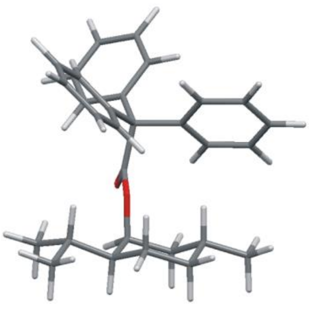

11 (conf 27)

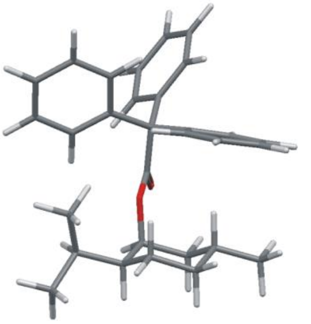

11 (conf 31)

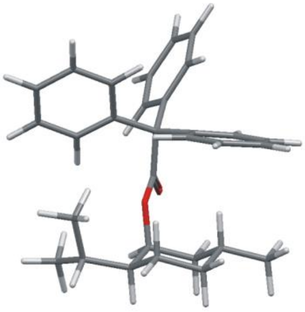

11 (conf 33)

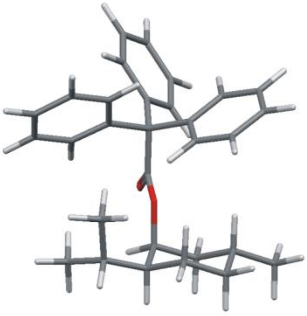

11 (conf 50)

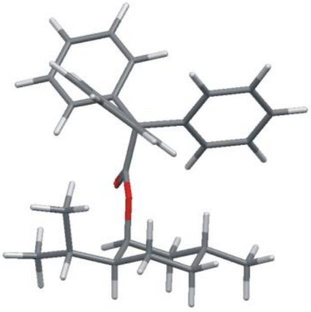

11 (conf 52)

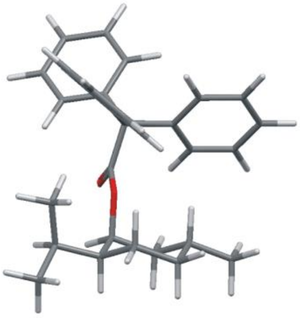

11 (conf 56)

Figure SI_10. Structures of individual, low-energy conformers of **11**, calculated at the M06-2X/6-311++G(d,p) level of theory.

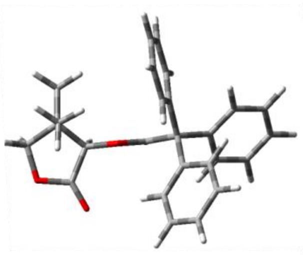

14 (conf. 1)

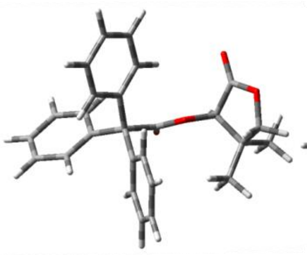

14 (conf. 6)

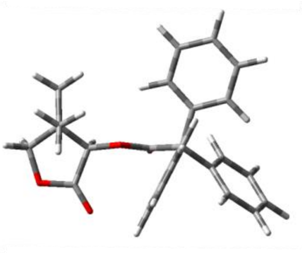

14 (conf. 8)

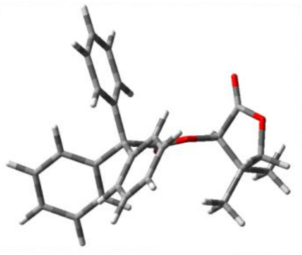

14 (conf. 39)

Figure SI_11. Structures of individual, low-energy conformers of **14**, calculated at the B3LYP/6-311++G(d,p) level of theory.

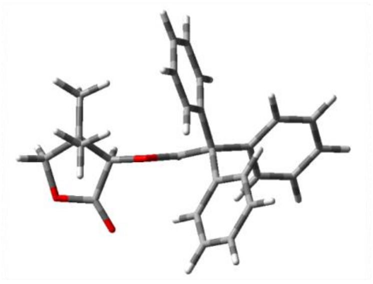

14 (conf. 1)

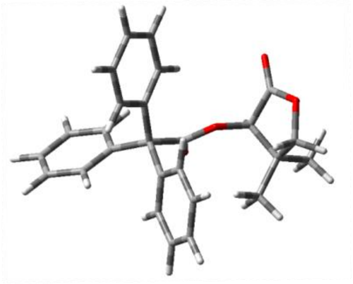

14 (conf. 6)

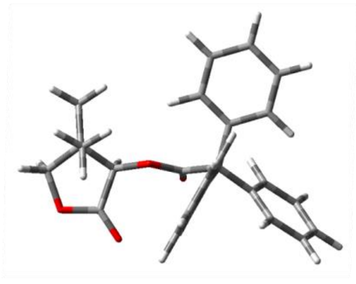

14 (conf. 8)

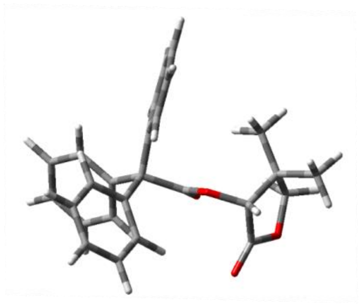

14 (conf. 27)

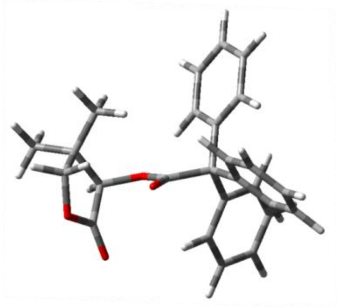

14 (conf. 49)

Figure SI_12. Structures of individual, low-energy conformers of **14**, calculated at the M06-2X/6-311++G(d,p) level of theory.

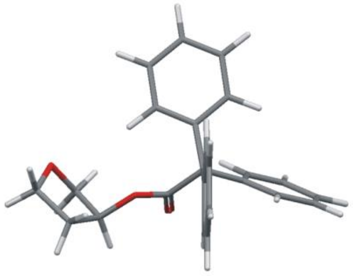

15 (conf 1)

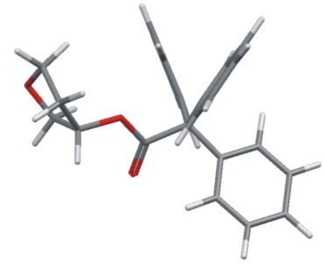

15 (conf 17)

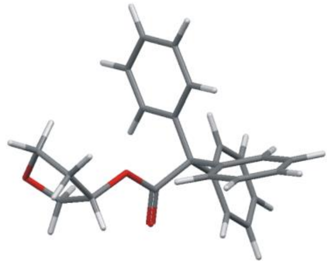

15 (conf 18

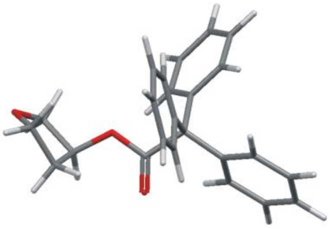

15 (conf 20)

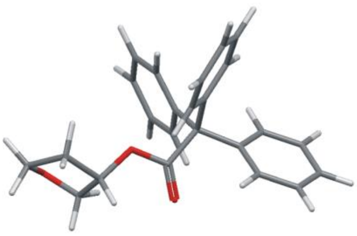

15 (conf 34)

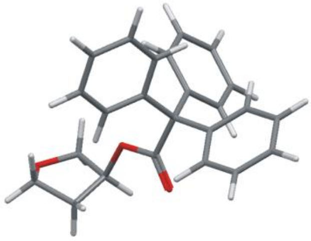

15 (conf 47)

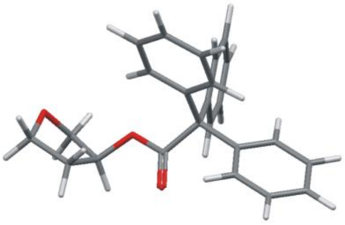

15 (conf 54)

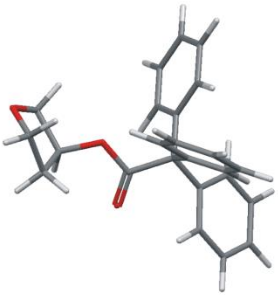

15 (conf 65)

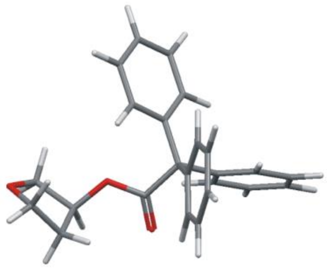

15 (conf 70)

Figure SI_13. Structures of individual, low-energy conformers of **15**, calculated at the B3LYP/6-311++G(d,p) level of theory.

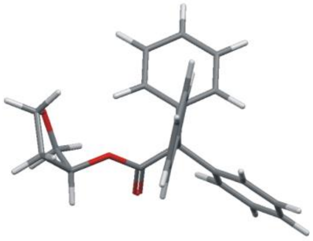

15 (conf 1)

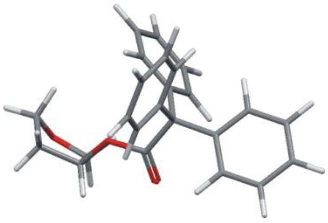

15 (conf 17)

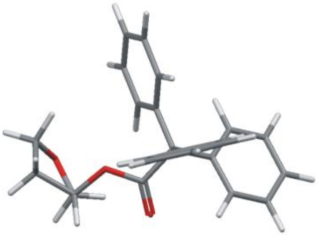

15 (conf 18

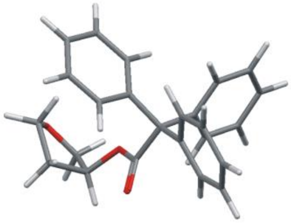

15 (conf 20)

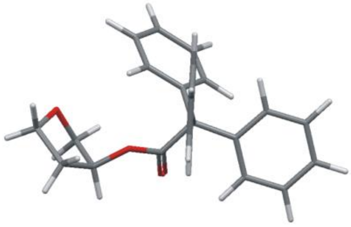

15 (conf 34)

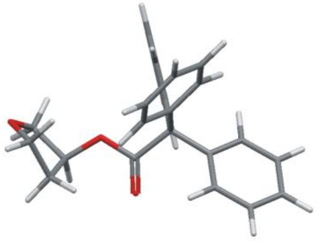

15 (conf 47)

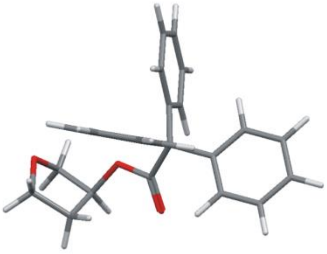

15 (conf 58)

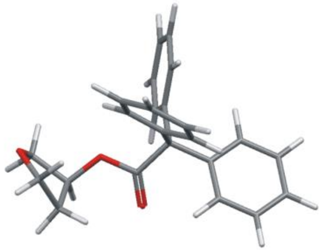

15 (conf 65)

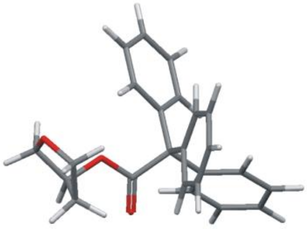

15 (conf 70)

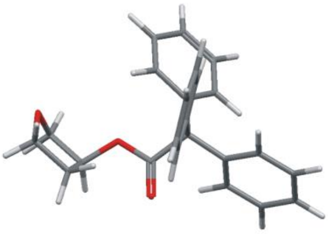

15 (conf 75)

Figure SI_14. Structures of individual, low-energy conformers of **15**, calculated at the M06-2X/6-311++G(d,p) level of theory.

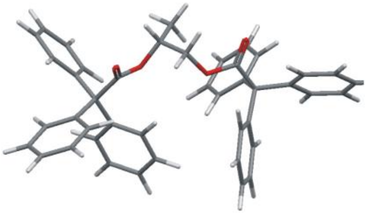

18 (conf 1)

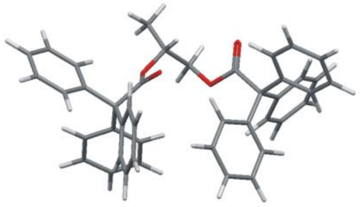

18 (conf 5)

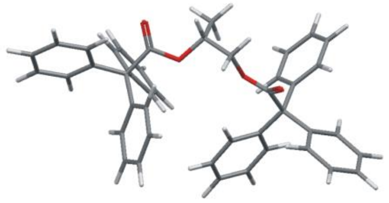

18 (conf 9)

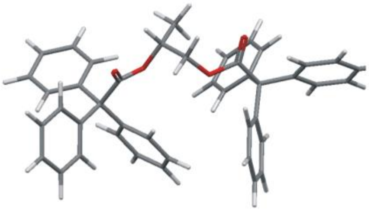

18 (conf 13)

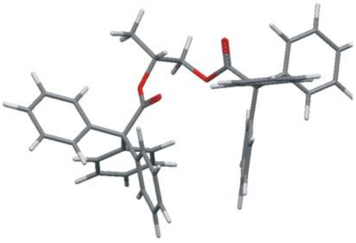

18 (conf 15)

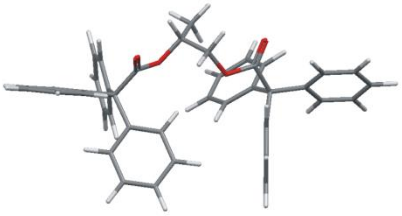

18 (conf 17)

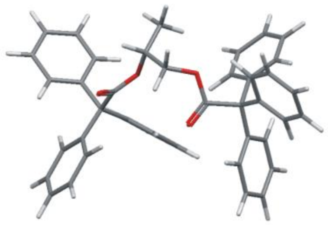

18 (conf 18)

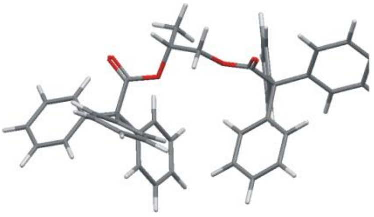

18 (conf 26)

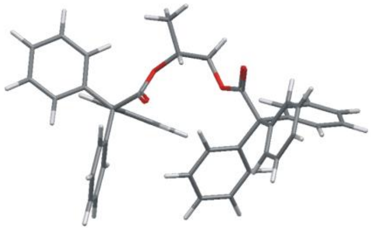

18 (conf 28)

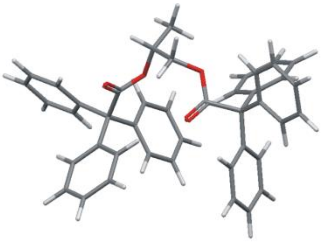

18 (conf 32)

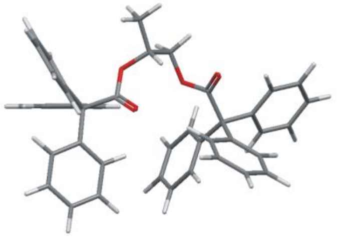

18 (conf 34)

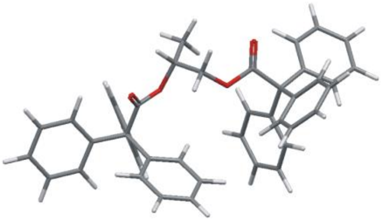

18 (conf 35)

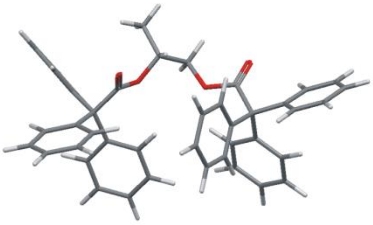

18 (conf 36)

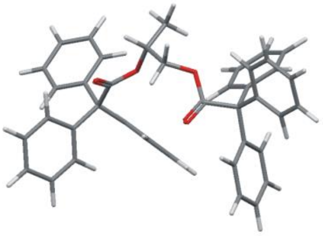

18 (conf 42)

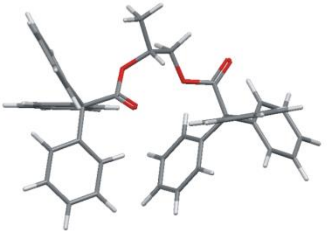

18 (conf 43)

Figure SI_15a. Structures of individual, low-energy conformers of **18**, calculated at the B3LYP/6-311G(d,p) level of theory, part I.

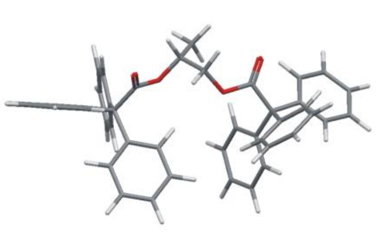

18 (conf 45)

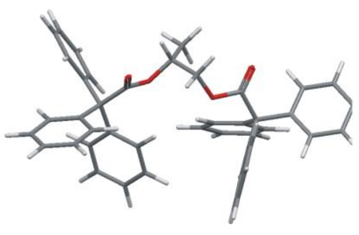

18 (conf 46)

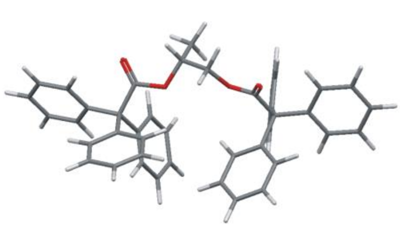

18 (conf 48)

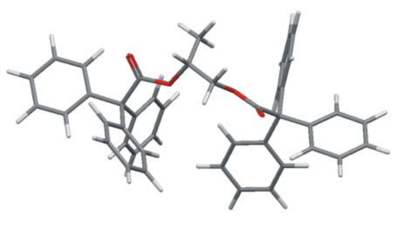

18 (conf 58)

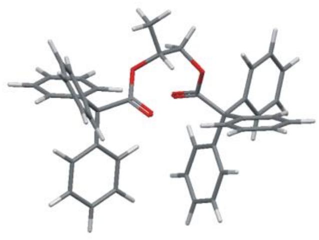

18 (conf 59)

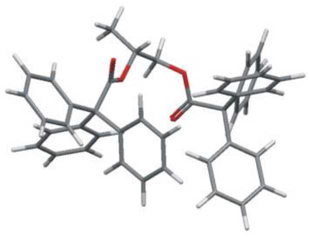

18 (conf 60)

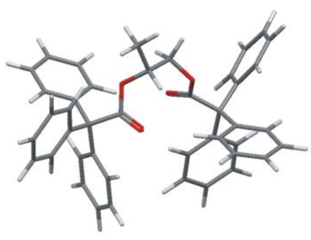

18 (conf 61)

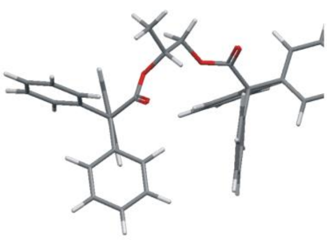

18 (conf 69)

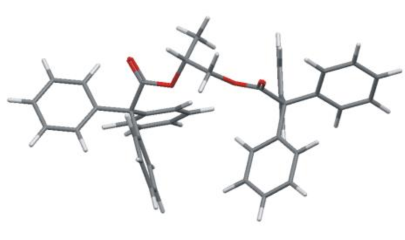

18 (conf 70)

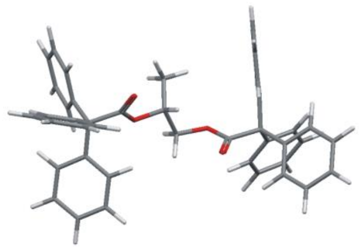

18 (conf 71)

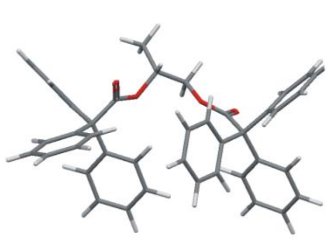

18 (conf 73)

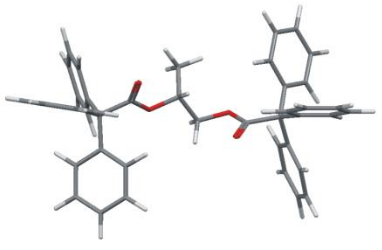

18 (conf 76)

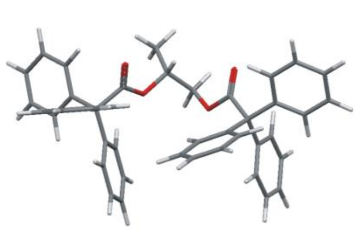

18 (conf 77)

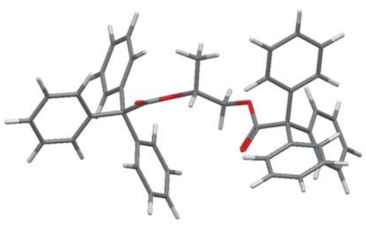

18 (conf 93)

Figure SI_15b. Structures of individual, low-energy conformers of **18**, calculated at the B3LYP/6-311G(d,p) level of theory, part II.

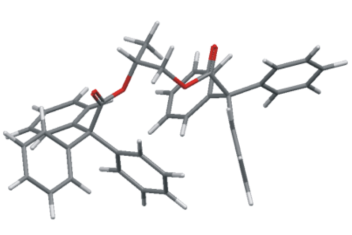

18 (conf 13)

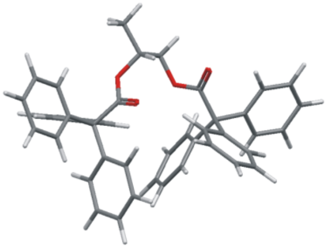

18 (conf 15)

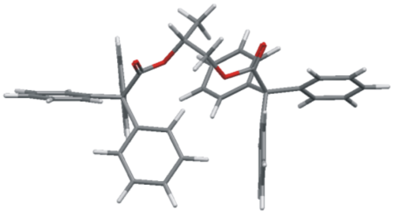

18 (conf 17)

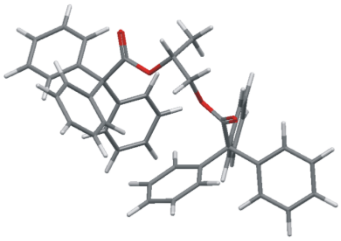

18 (conf 26)

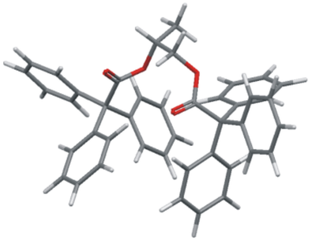

18 (conf 32)

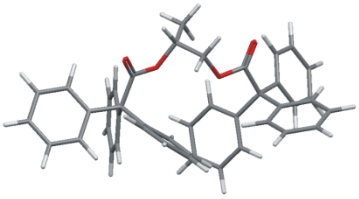

18 (conf 35)

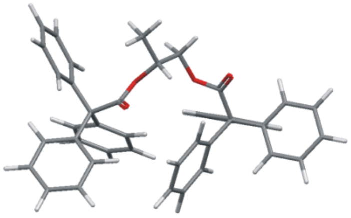

18 (conf 36)

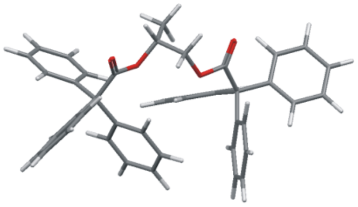

18 (conf 46)

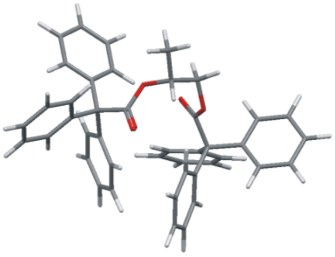

18 (conf 60)

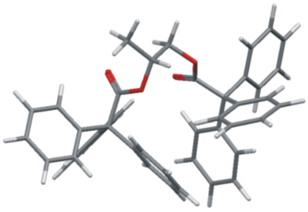

18 (conf 63)

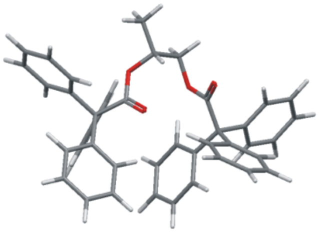

18 (conf 77)

Figure SI_16. Structures of individual, low-energy conformers of **11**, calculated at the B3LYP-GD3BJ/6-311G(d,p) level of theory.

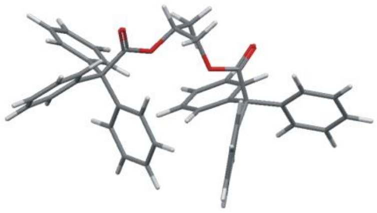

18 (conf 1)

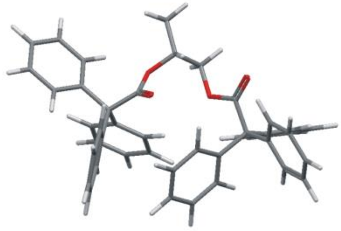

18 (conf 5)

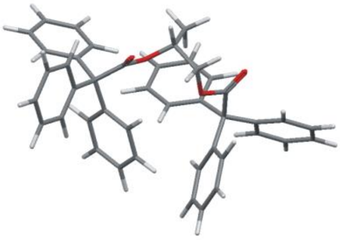

18 (conf 13)

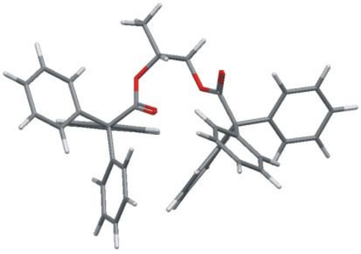

18 (conf 15)

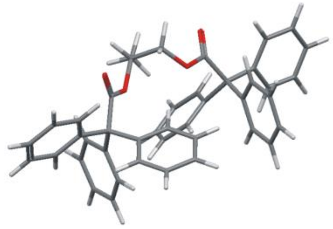

18 (conf 17)

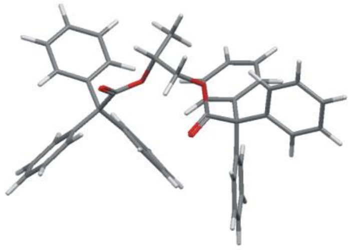

18 (conf 18)

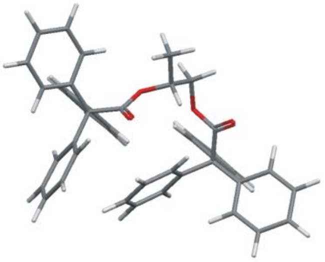

18 (conf 28)

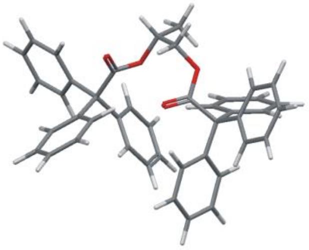

18 (conf 32)

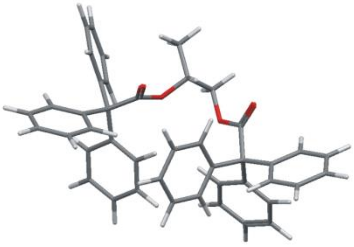

18 (conf 36)

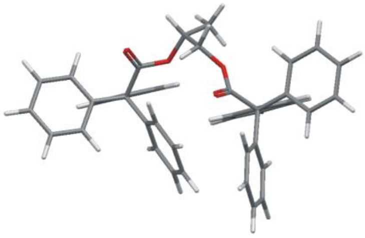

18 (conf 42)

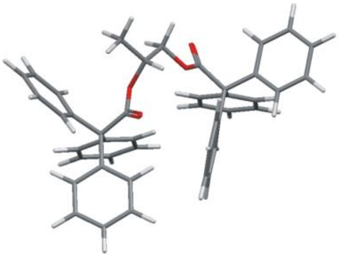

18 (conf 73)

Figure SI_17. Structures of individual, low-energy conformers of **18**, calculated at the M06-2X/6-311G(d,p) level of theory.

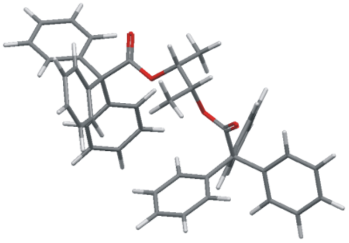

20 (conf 1)

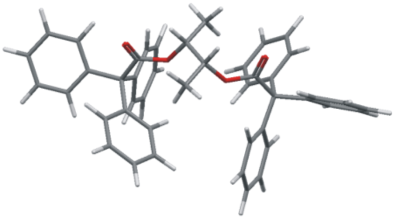

20 (conf 11)

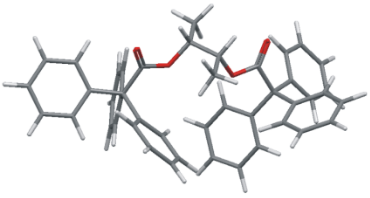

20 (conf 14)

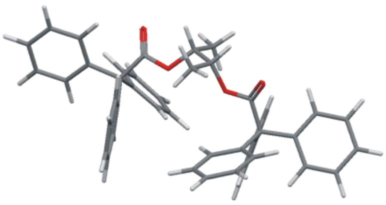

20 (conf 16)

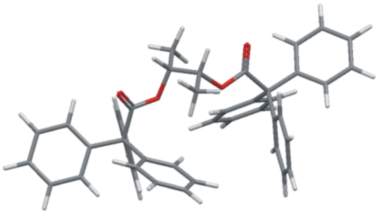

20 (conf 17)

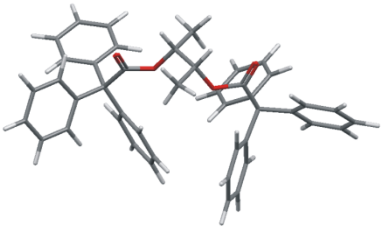

20 (conf 20)

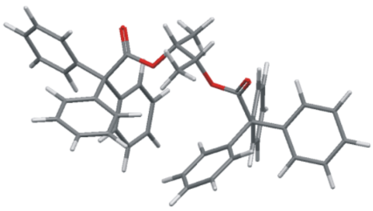

20 (conf 21)

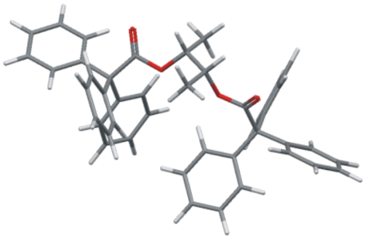

20 (conf 22)

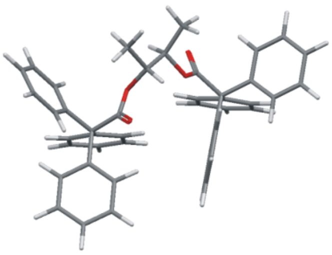

20 (conf 23)

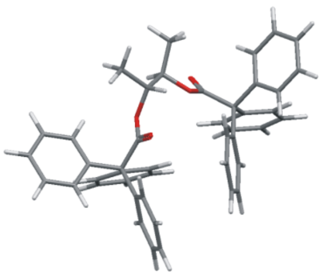

20 (conf 25)

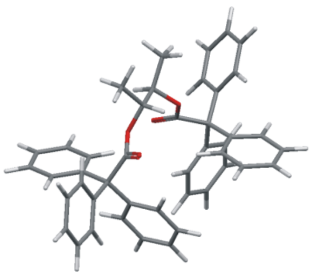

20 (conf 43)

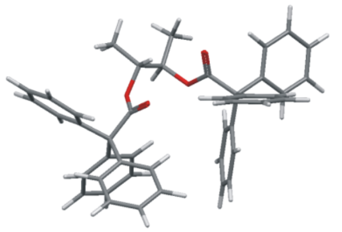

20 (conf 46)

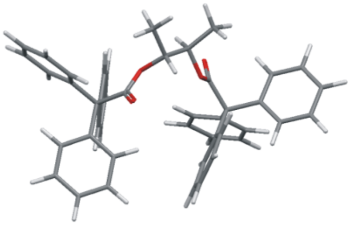

20 (conf 47)

Figure SI_18. Structures of individual, low-energy conformers of **20**, calculated at the B3LYP/6-311G(d,p) level of theory.

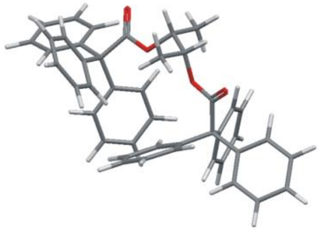

20 (conf 1)

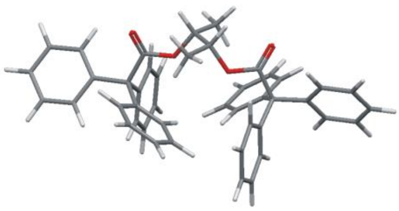

20 (conf 11)

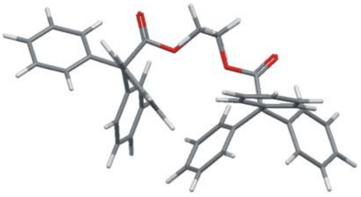

20 (conf 14)

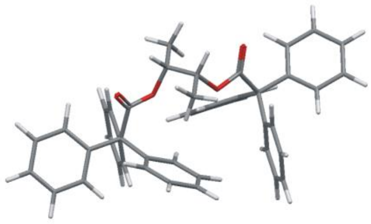

20 (conf 17)

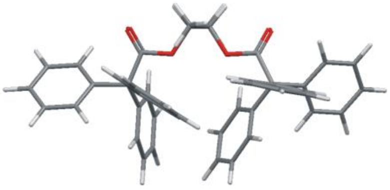

20 (conf 20)

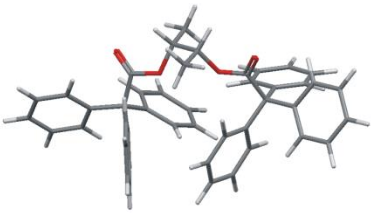

20 (conf 21)

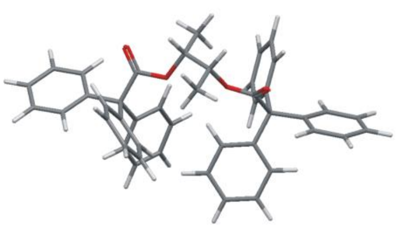

20 (conf 22)

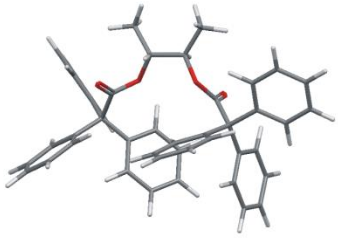

20 (conf 25)

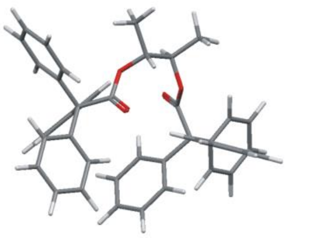

20 (conf 43)

Figure SI_19. Structures of individual, low-energy conformers of **20**, calculated at the B3LYP-GD3BJ/6-311G(d,p) level of theory.

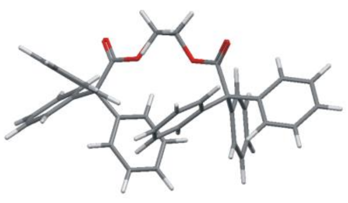

20 (conf 1)

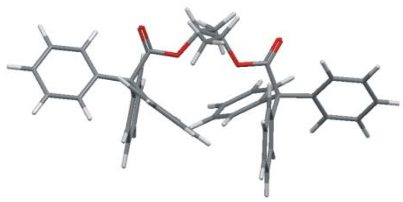

20 (conf 11)

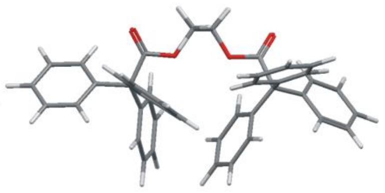

20 (conf 14)

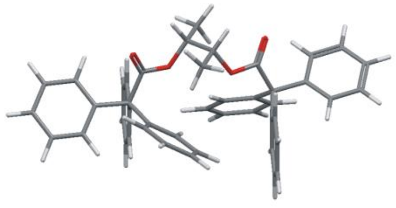

20 (conf 17)

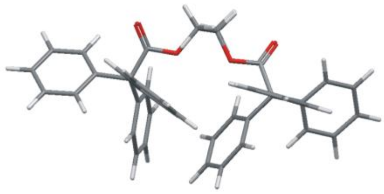

20 (conf 20)

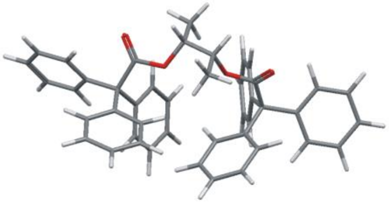

20 (conf 21)

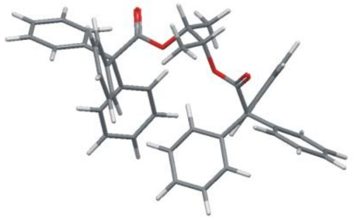

20 (conf 22)

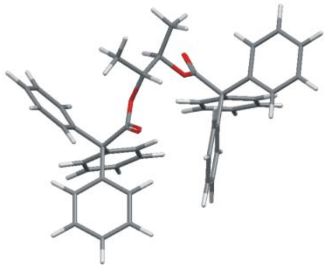

20 (conf 23)

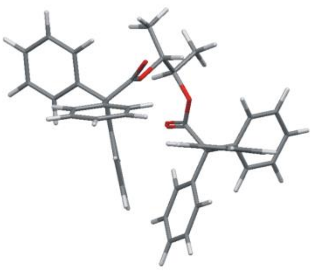

20 (conf 25)

Figure SI_20. Structures of individual, low-energy conformers of **20**, calculated at the M06-2X/6-311G(d,p) level of theory.

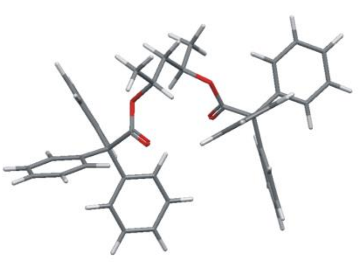

21 (conf 1)

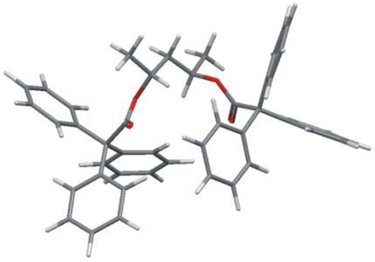

21 (conf 6)

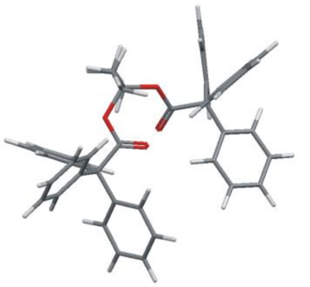

21 (conf 12)

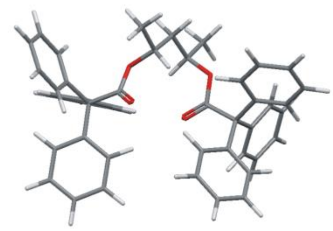

21 (conf 15)

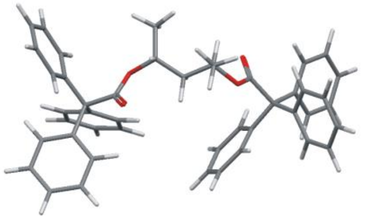

21 (conf 31)

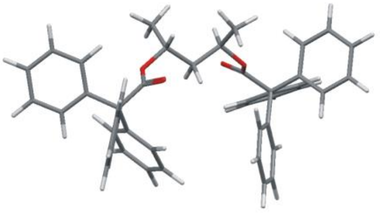

21 (conf 54)

Figure SI_21. Structures of individual, low-energy conformers of **21**, calculated at the B3LYP/6-311G(d,p) level of theory.

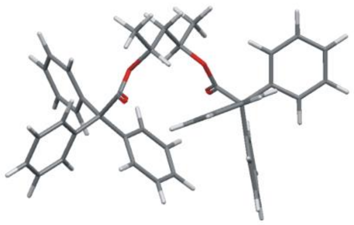

21 (conf 1)

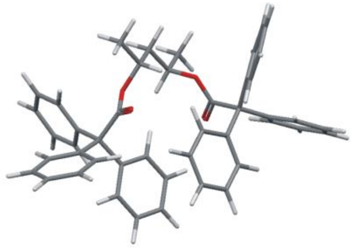

21 (conf 6)

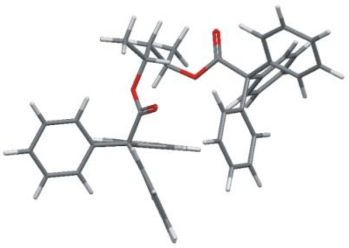

21 (conf 16)

Figure SI_22. Structures of individual, low-energy conformers of **21**, calculated at the B3LYP-GD3BJ/6-311G(d,p) level of theory.

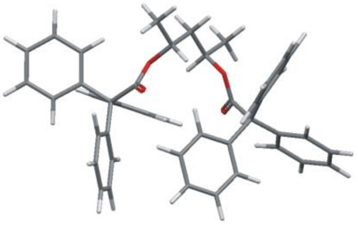

21 (conf 1)

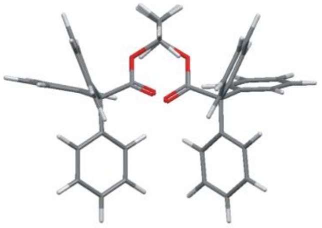

21 (conf 12)

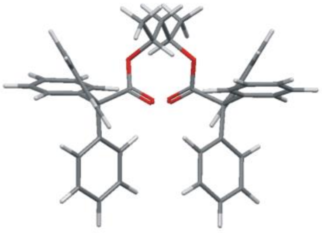

21 (conf 15)

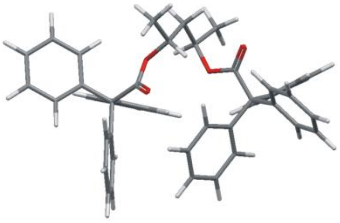

21 (conf 16)

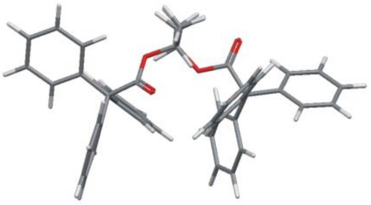

21 (conf 25)

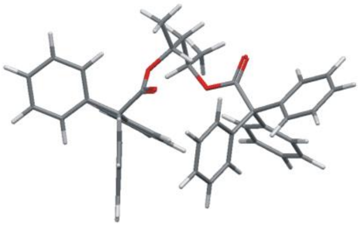

21 (conf 35)

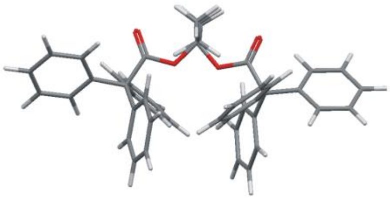

21 (conf 46)

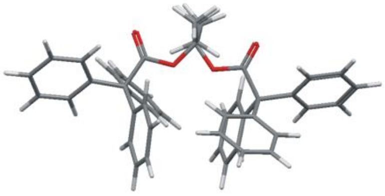

21 (conf 61)

Figure SI_23. Structures of individual, low-energy conformers of **21**, calculated at the M06-2X/6-311G(d,p) level of theory.

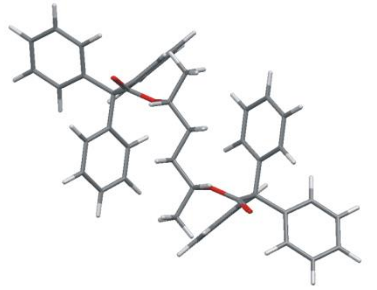

22 (conf 1)

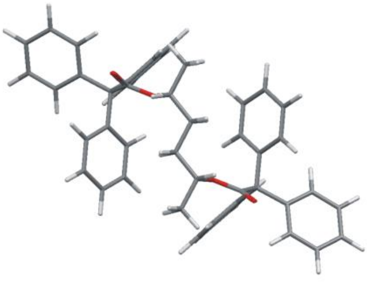

22 (conf 3)

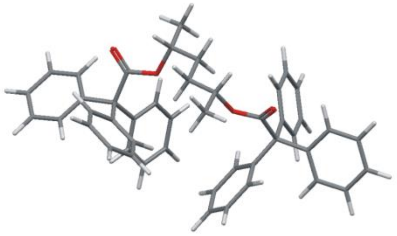

22 (conf 5)

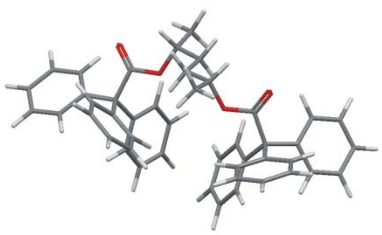

22 (conf 7)

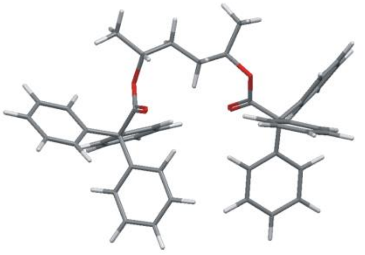

22 (conf 8)

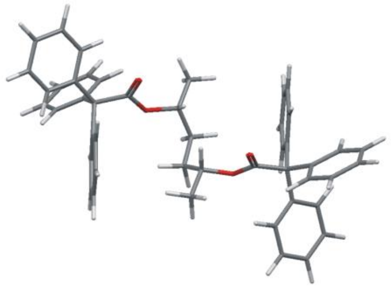

22 (conf 9)

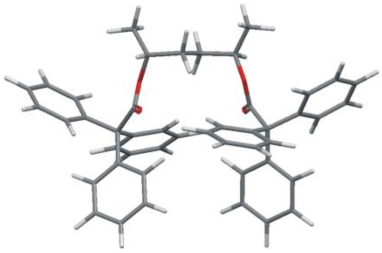

22 (conf 10)

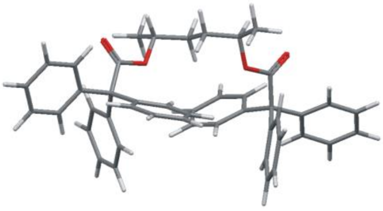

22 (conf 11)

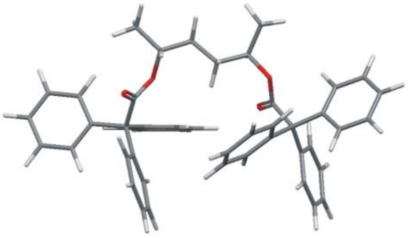

22 (conf 12)

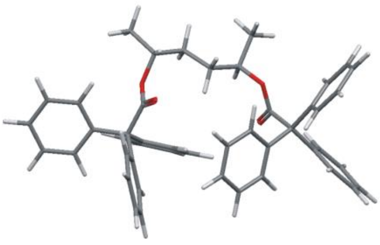

22 (conf 13)

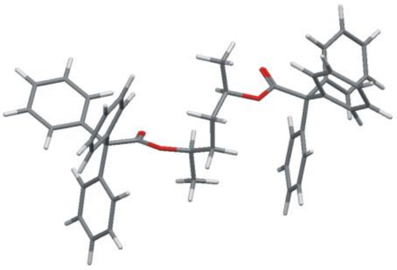

22 (conf 15)

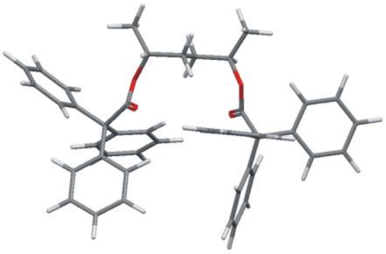

22 (conf 17)

Figure SI_24a. Structures of individual, low-energy conformers of **22**, calculated at the B3LYP/6-311G(d,p) level of theory, part I.

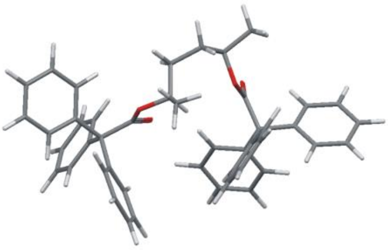

22 (conf 26)

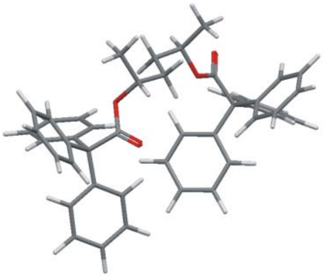

22 (conf 31)

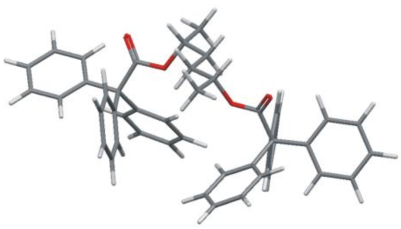

22 (conf 36)

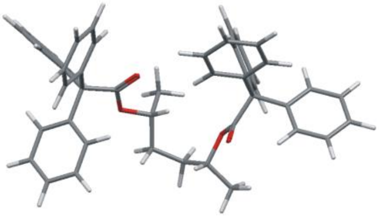

22 (conf 71)

Figure SI_24b. Structures of individual, low-energy conformers of **22**, calculated at the B3LYP/6-311G(d,p) level of theory, part II.

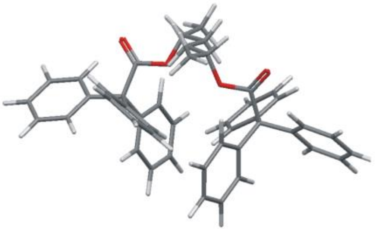

22 (conf 1)

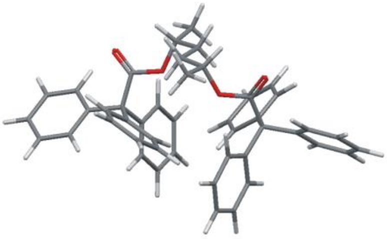

22 (conf 3)

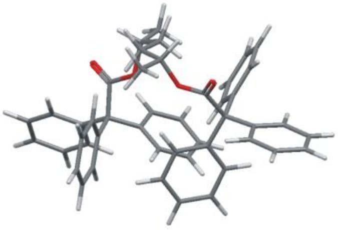

22 (conf 5)

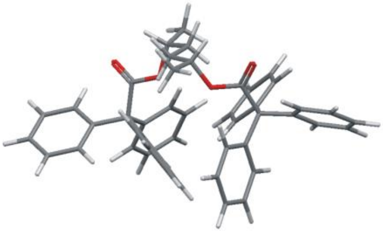

22 (conf 7)

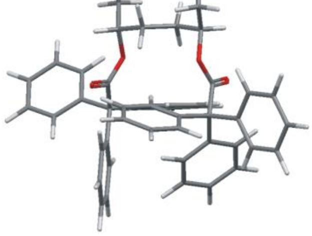

22 (conf 17)

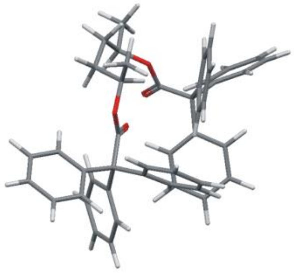

22 (conf 26)

Figure SI_25. Structures of individual, low-energy conformers of **22**, calculated at the B3LYP-GD3BJ/6-311G(d,p) level of theory.

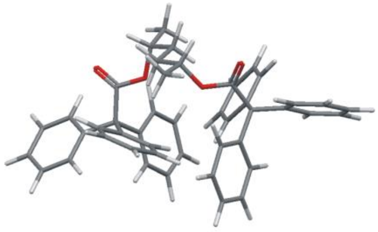

22 (conf 1)

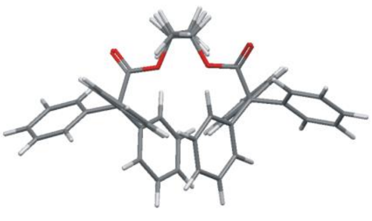

22 (conf 3)

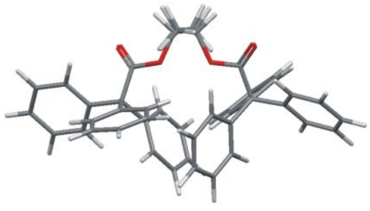

22 (conf 11)

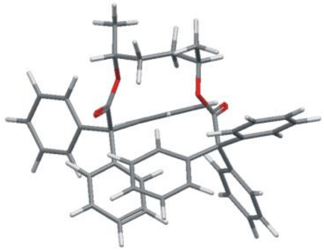

22 (conf 17)

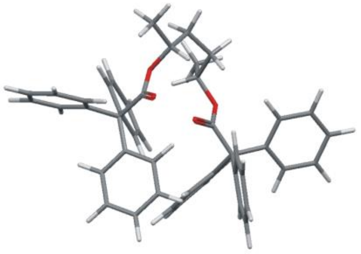

22 (conf 26)

Figure SI_26. Structures of individual, low-energy conformers of **22**, calculated at the M06-2X/6-311G(d,p) level of theory.

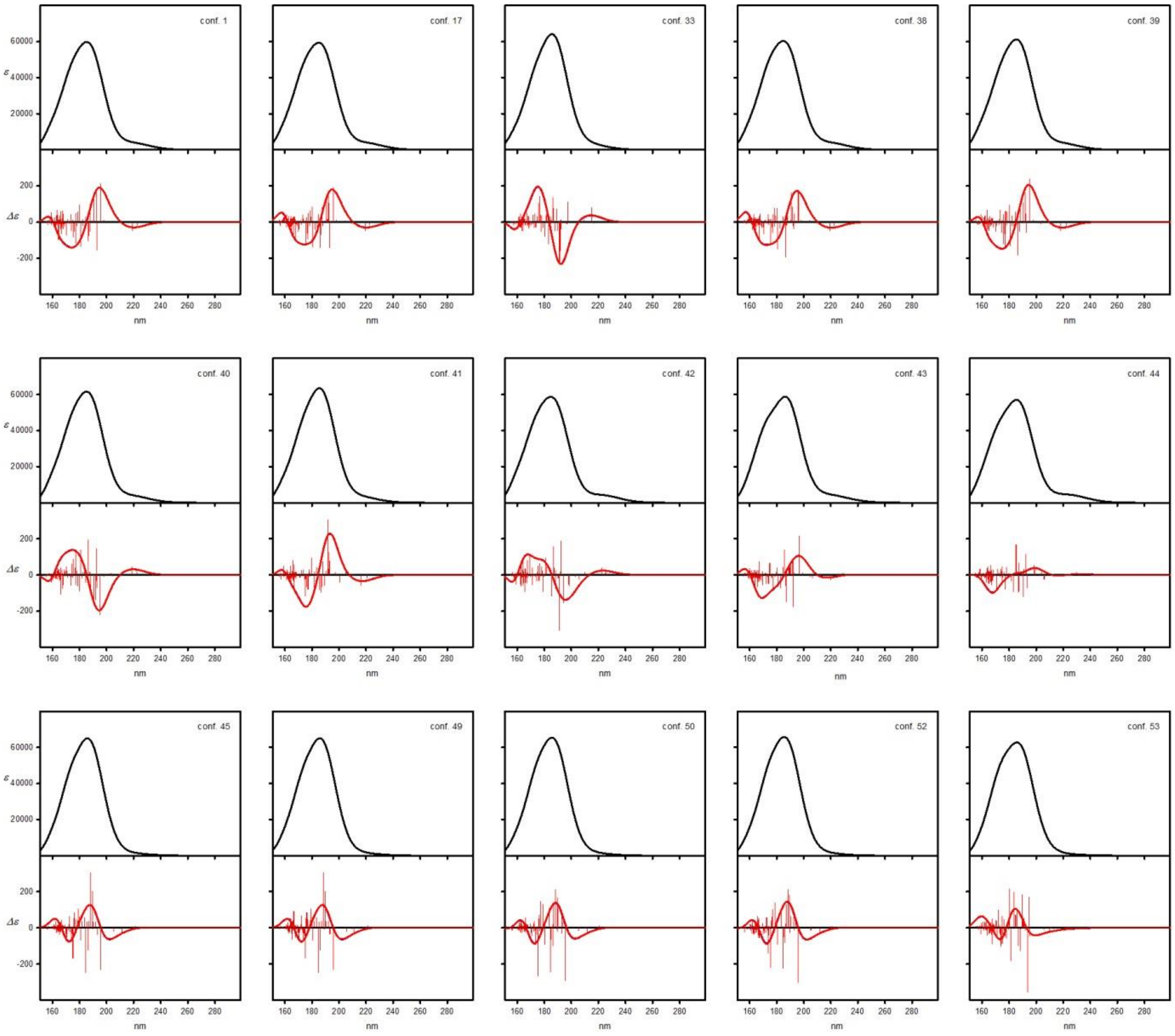

Figure SI_27. UV and ECD spectra of the low-energy conformers of compound **1** calculated at TD-CAM-B3LYP/6-311++G(2d,2p) level for structures optimized at B3LYP/6-311++G(d,p) level. Wavelengths were not corrected.

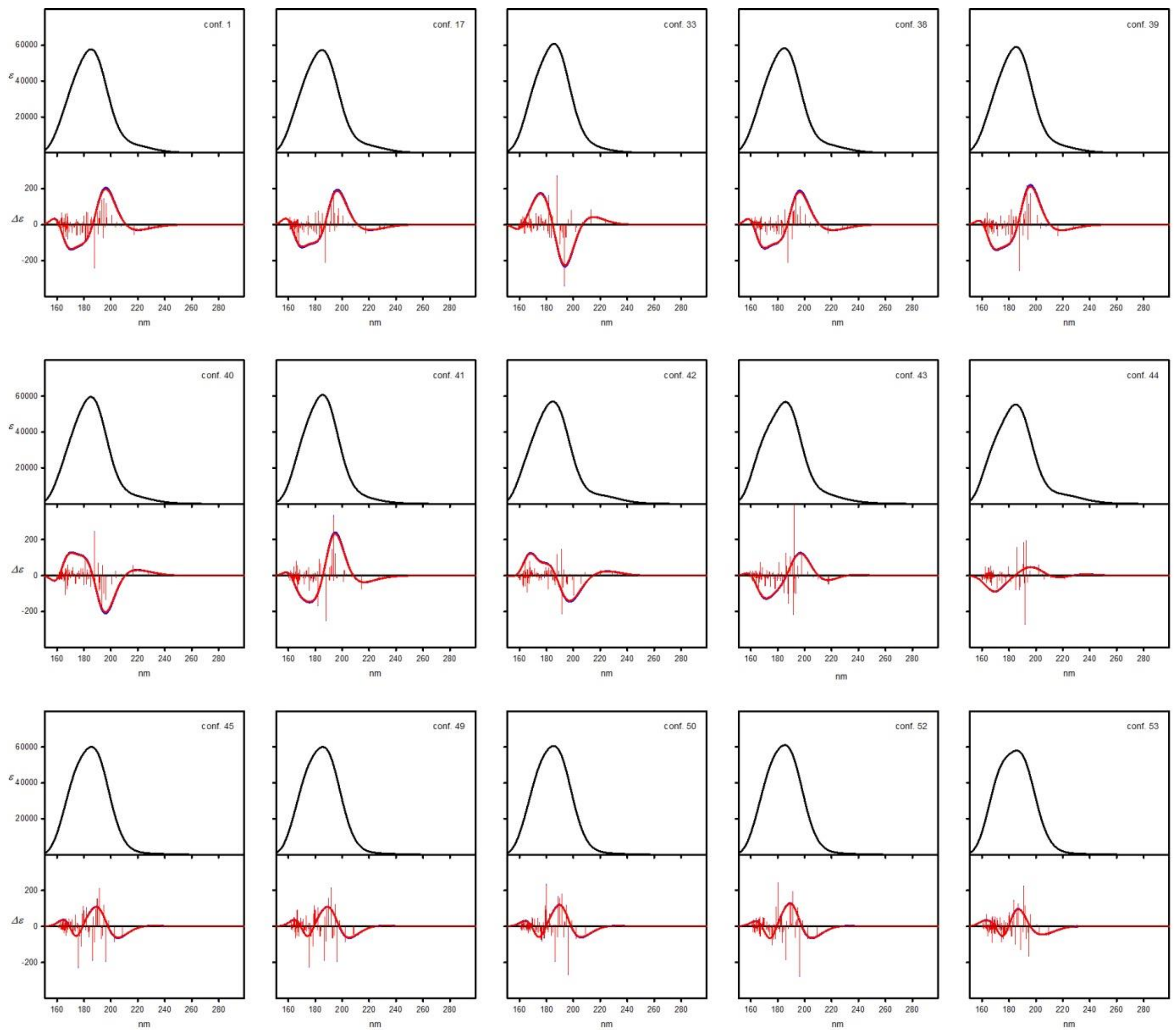

Figure SI_28. UV and ECD spectra of the low-energy conformers of compound **1** calculated at TD-M06-2X/6-311++G(2d,2p) level for structures optimized at B3LYP/6-311++G(d,p) level. Wavelengths were not corrected.

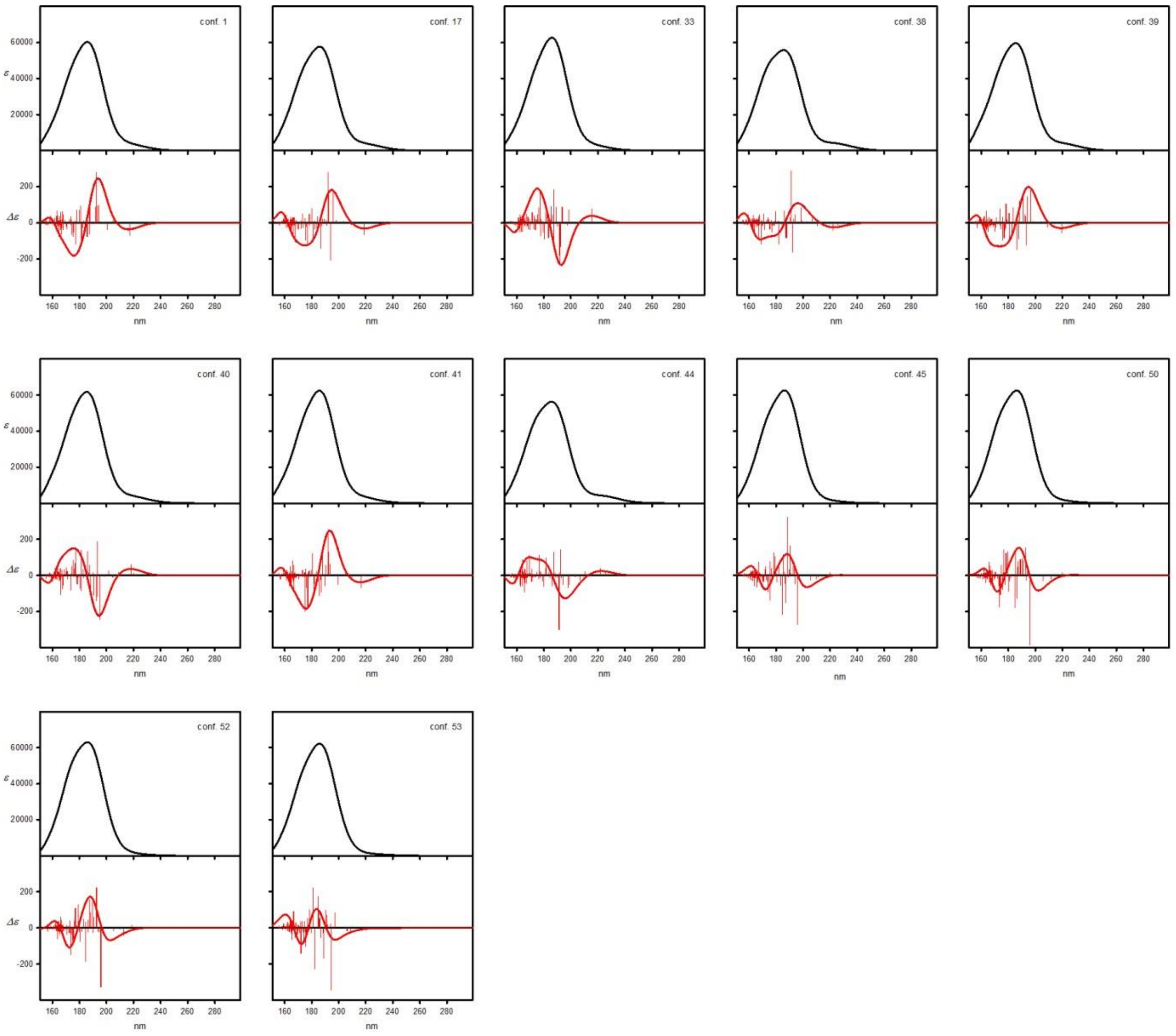

Figure SI_29. UV and ECD spectra of the low-energy conformers of compound **1** calculated at TD-CAM-B3LYP/6-311++G(2d,2p) level for structures optimized at M06-2X/6-311++G(d,p) level. Wavelengths were not corrected.

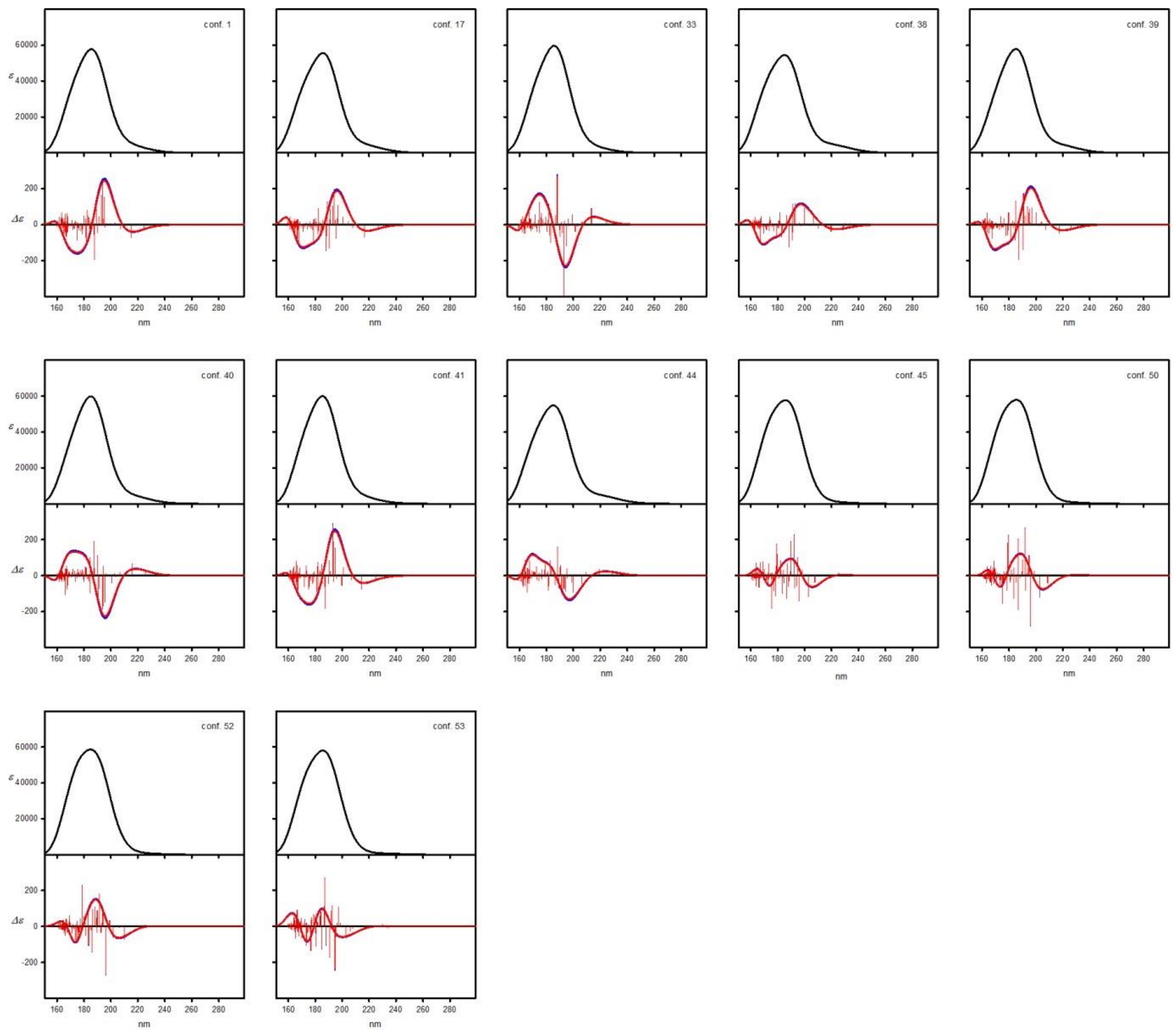

Figure SI_30. UV and ECD spectra of the low-energy conformers of compound **1** calculated at TD-M06-2X/6-311++G(2d,2p) level for structures optimized at M06-2X/6-311++G(d,p) level. Wavelengths were not corrected.

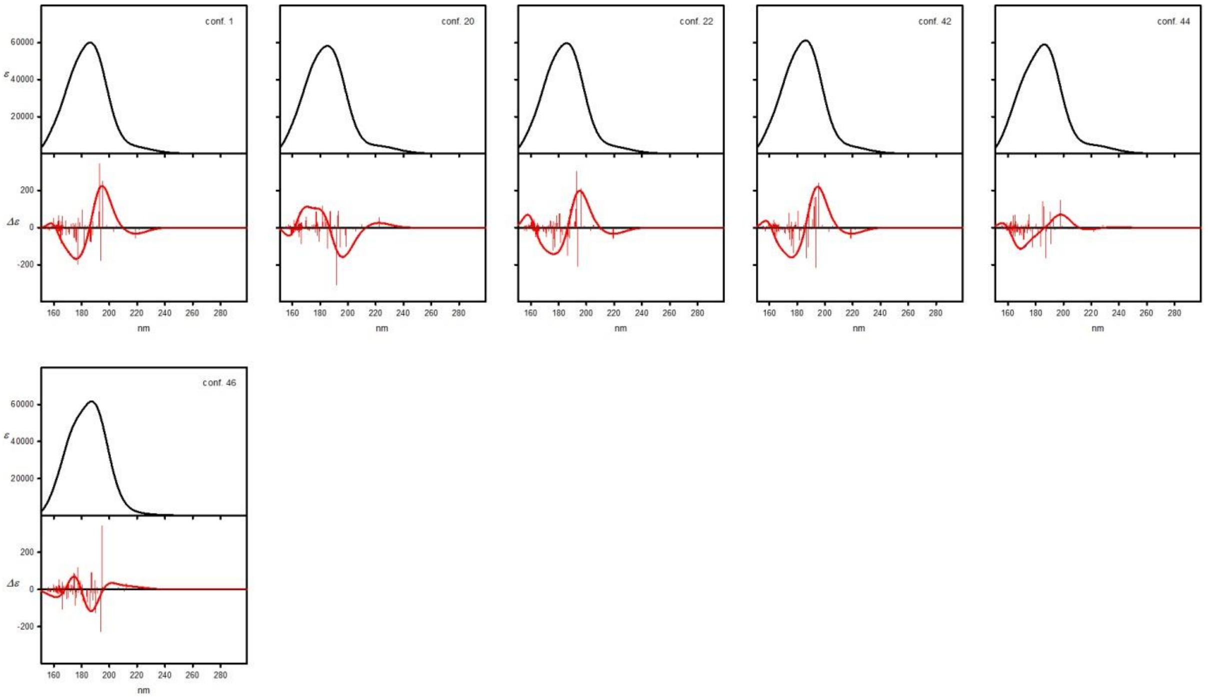

Figure SI_31. UV and ECD spectra of the low-energy conformers of compound **4** calculated at TD-CAM-B3LYP/6-311++G(2d,2p) level for structures optimized at B3LYP/6-311++G(d,p) level. Wavelengths were not corrected.

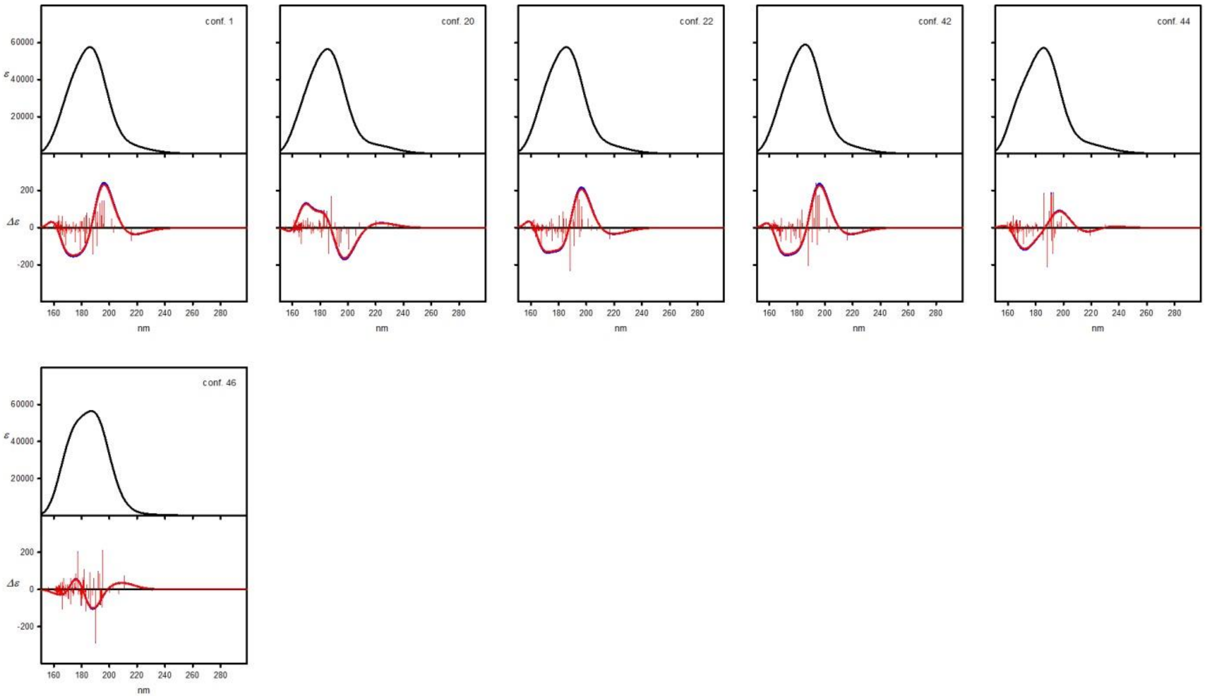

Figure SI_32. UV and ECD spectra of the low-energy conformers of compound **4** calculated at TD-M06-2X/6-311++G(2d,2p) level for structures optimized at B3LYP/6-311++G(d,p) level. Wavelengths were not corrected.

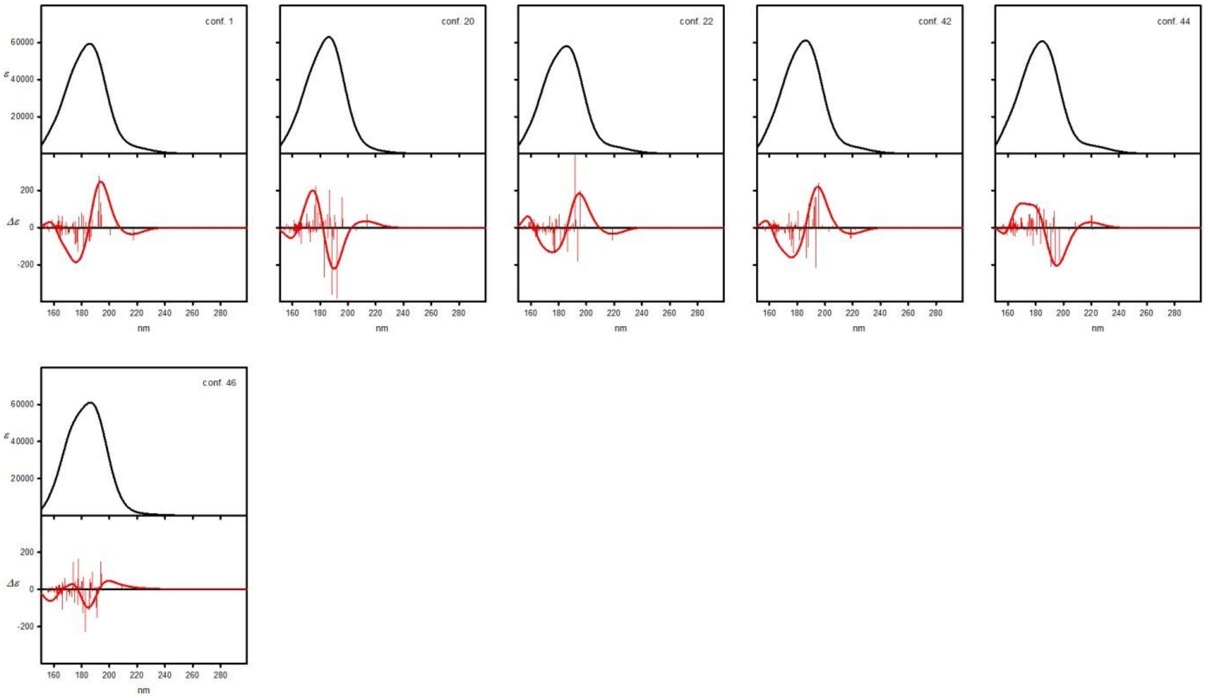

Figure SI_33. UV and ECD spectra of the low-energy conformers of compound **4** calculated at TD-CAM-B3LYP/6-311++G(2d,2p) level for structures optimized at M06-2X/6-311++G(d,p) level. Wavelengths were not corrected.

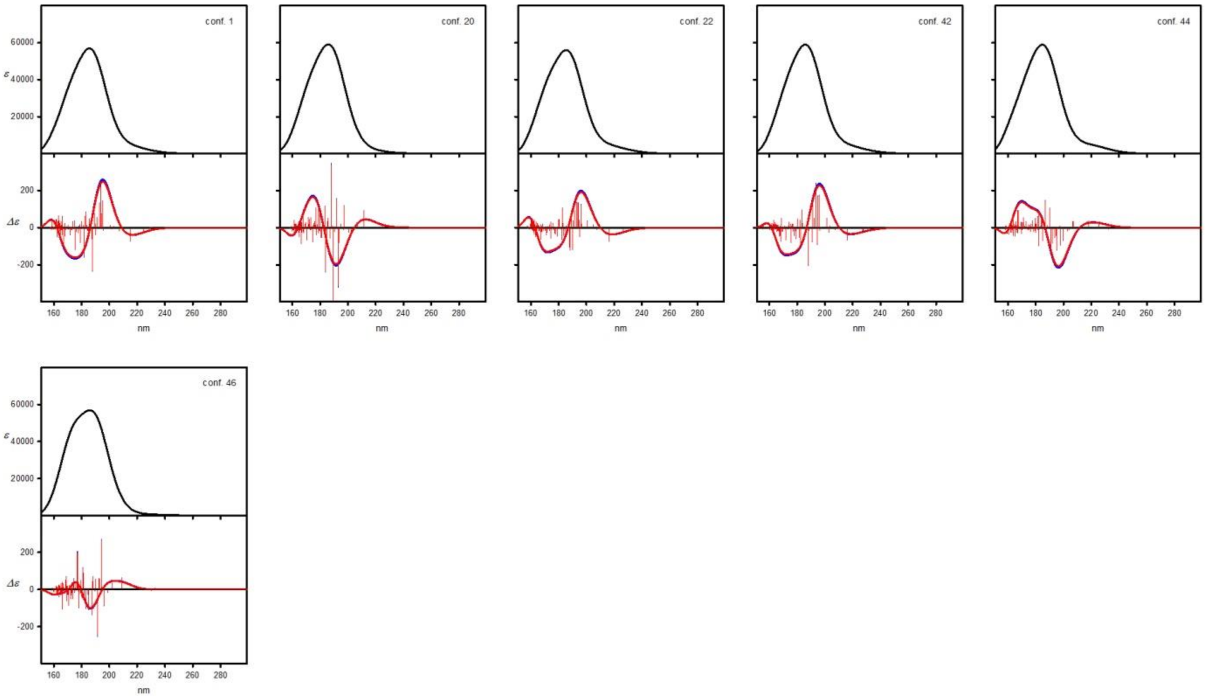

Figure SI_34. UV and ECD spectra of the low-energy conformers of compound **4** calculated at TD-M06-2X/6-311++G(2d,2p) level for structures optimized at M06-2X/6-311++G(d,p) level. Wavelengths were not corrected.

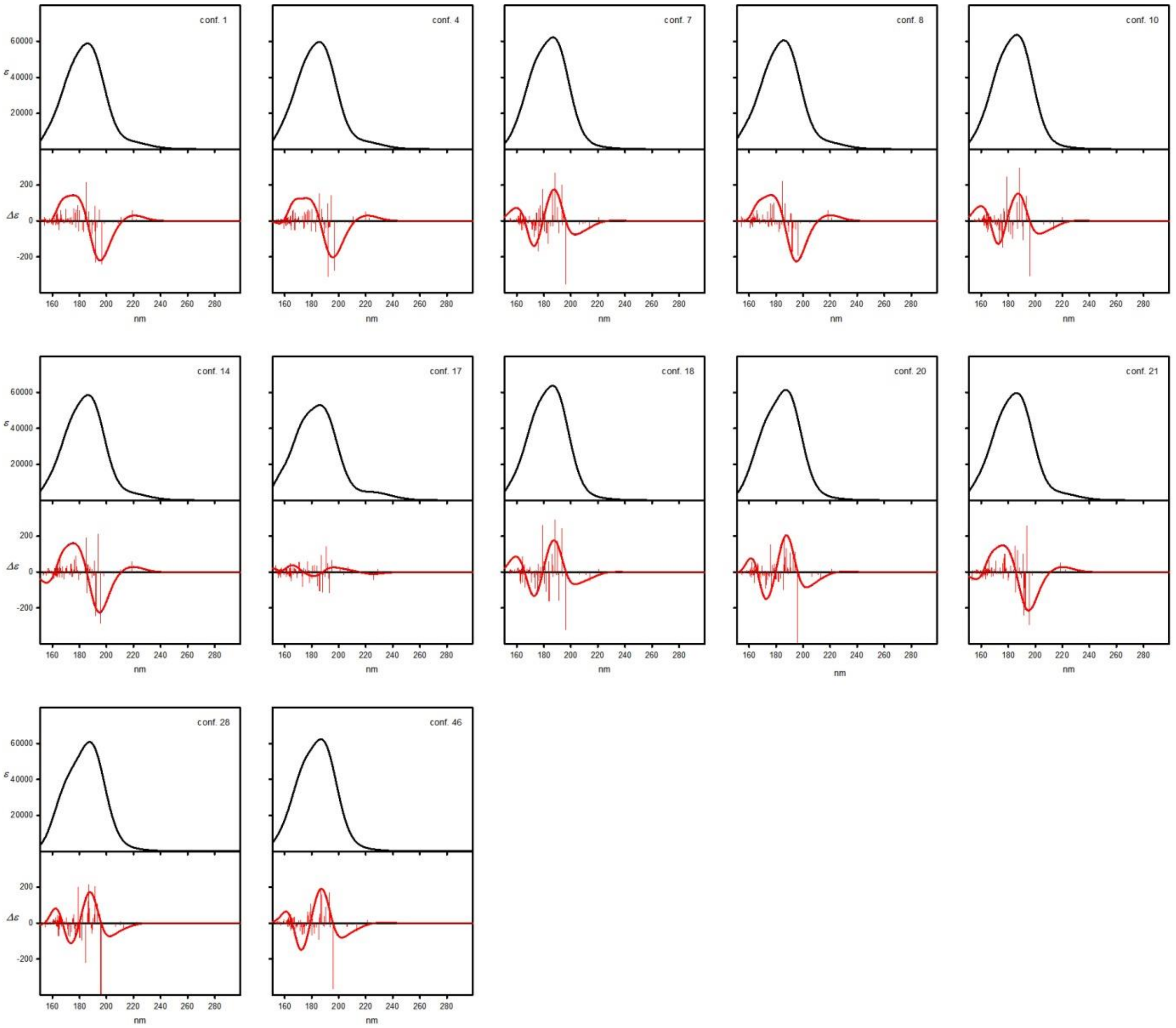

Figure SI_35. UV and ECD spectra of the low-energy conformers of compound **6** calculated at TD-CAM-B3LYP/6-311++G(2d,2p) level for structures optimized at B3LYP/6-311++G(d,p) level. Wavelengths were not corrected.

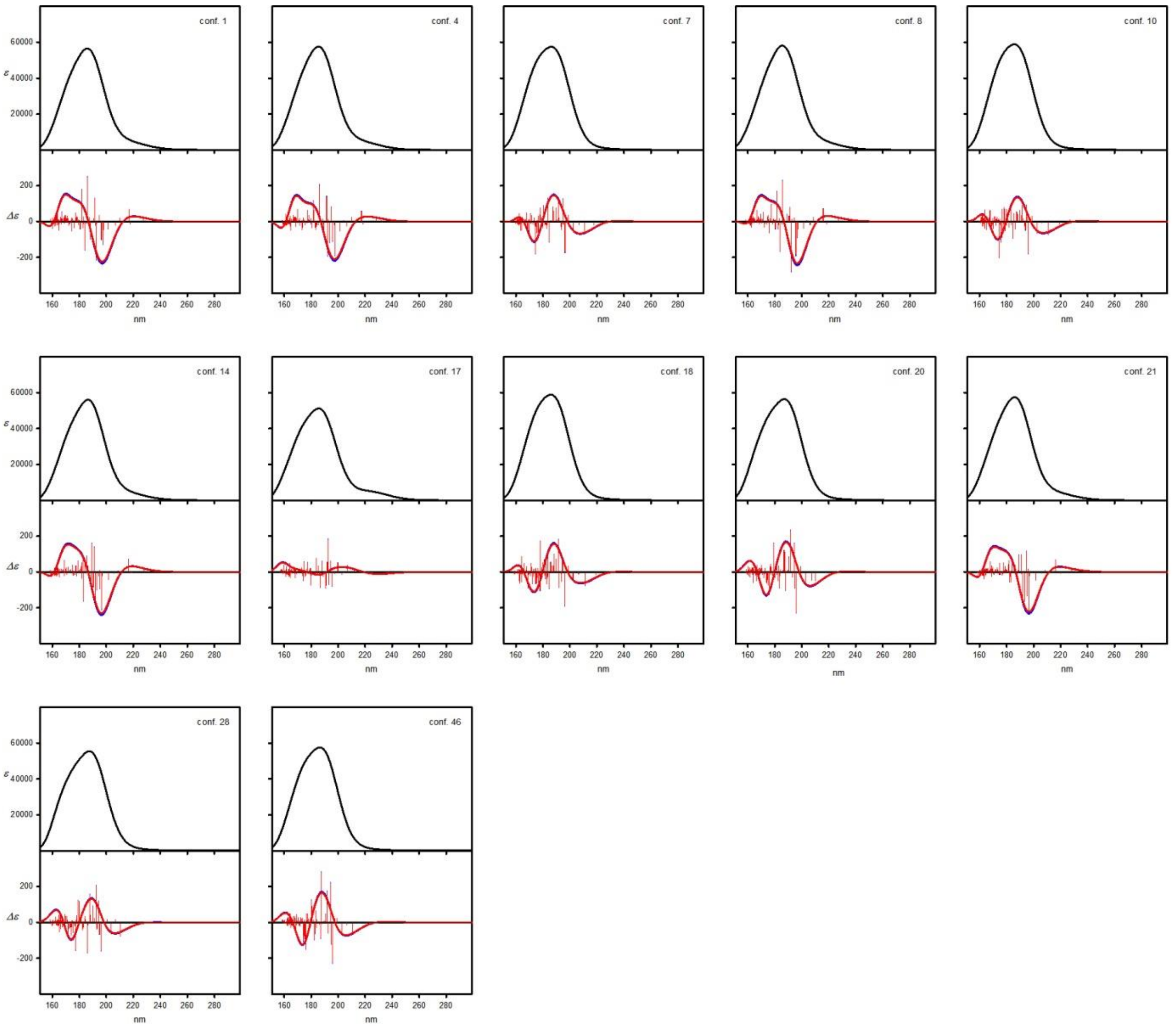

Figure SI_36. UV and ECD spectra of the low-energy conformers of compound **6** calculated at TD-M06-2X/6-311++G(2d,2p) level for structures optimized at B3LYP/6-311++G(d,p) level. Wavelengths were not corrected.

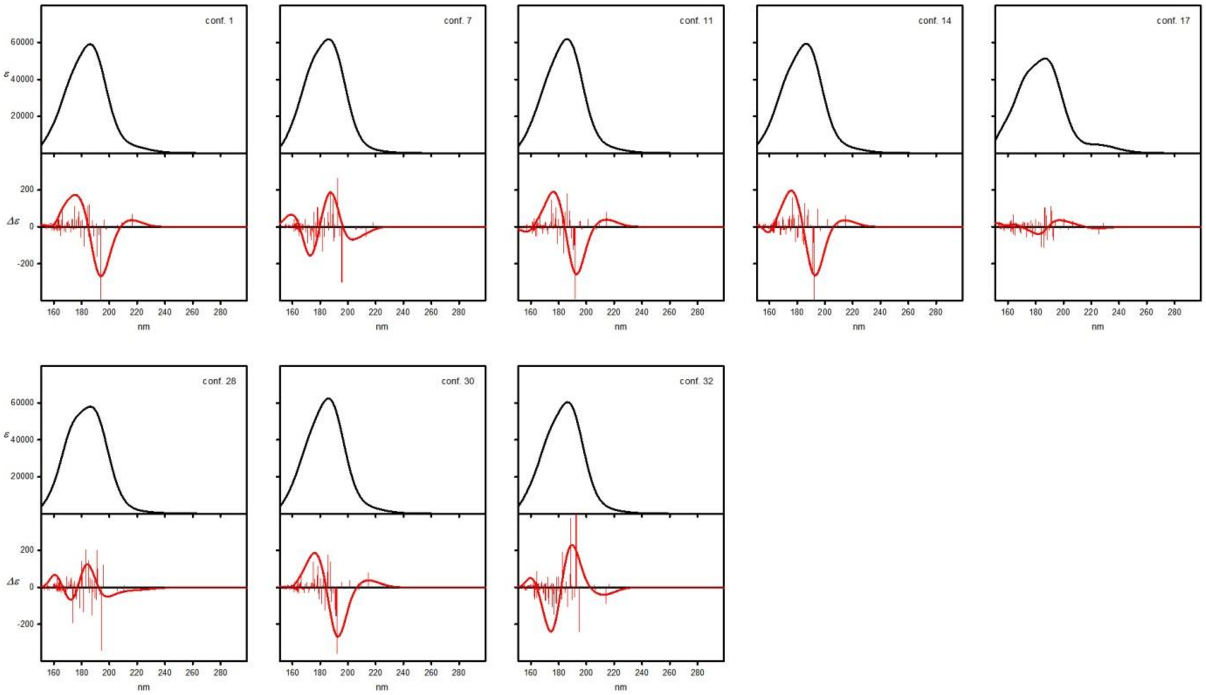

Figure SI_37. UV and ECD spectra of the low-energy conformers of compound **6** calculated at TD-CAM-B3LYP/6-311++G(2d,2p) level for structures optimized at M06-2X/6-311++G(d,p) level. Wavelengths were not corrected.

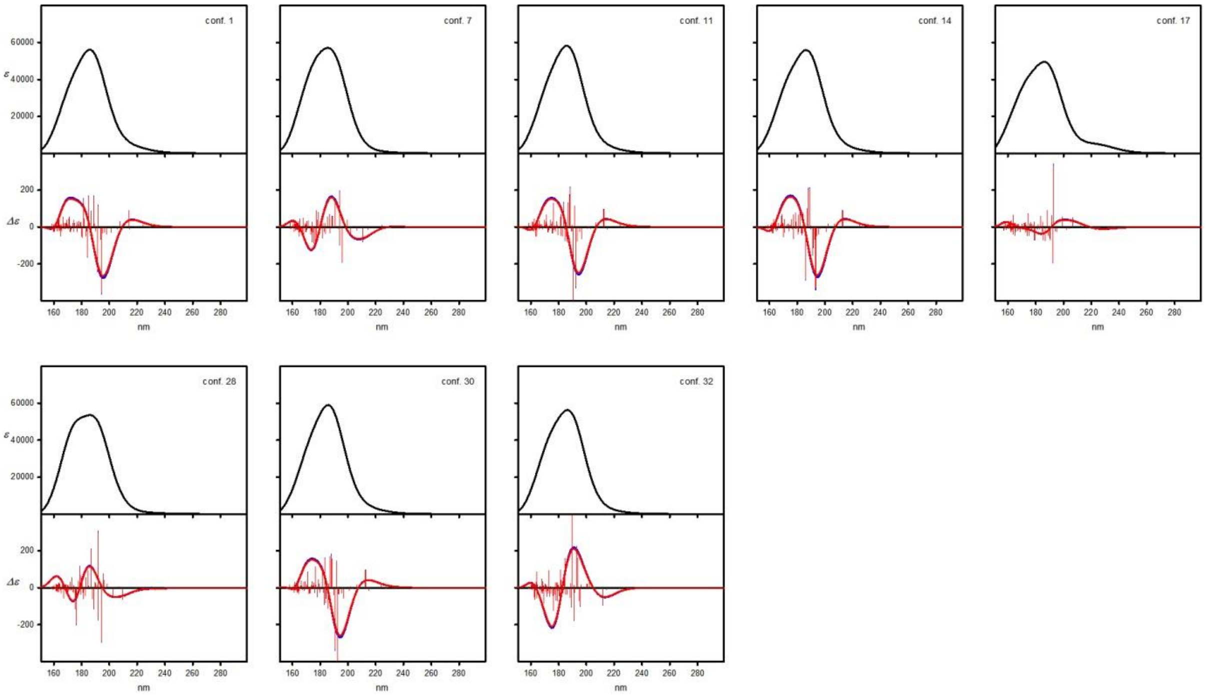

Figure SI_38. UV and ECD spectra of the low-energy conformers of compound **6** calculated at TD-M06-2X/6-311++G(2d,2p) level for structures optimized at M06-2X/6-311++G(d,p) level. Wavelengths were not corrected.

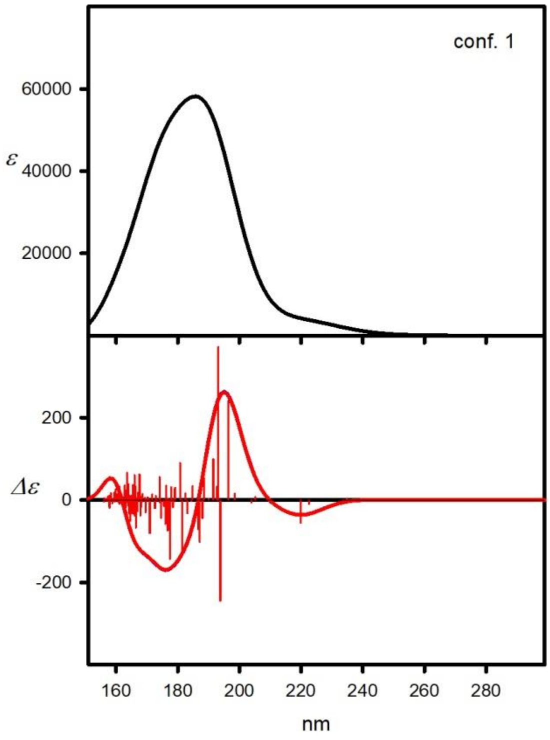

Figure SI_39. UV and ECD spectra of the low-energy conformers of compound **10** calculated at TD-CAM-B3LYP/6-311++G(2d,2p) level for structures optimized at B3LYP/6-311++G(d,p) level. Wavelengths were not corrected.

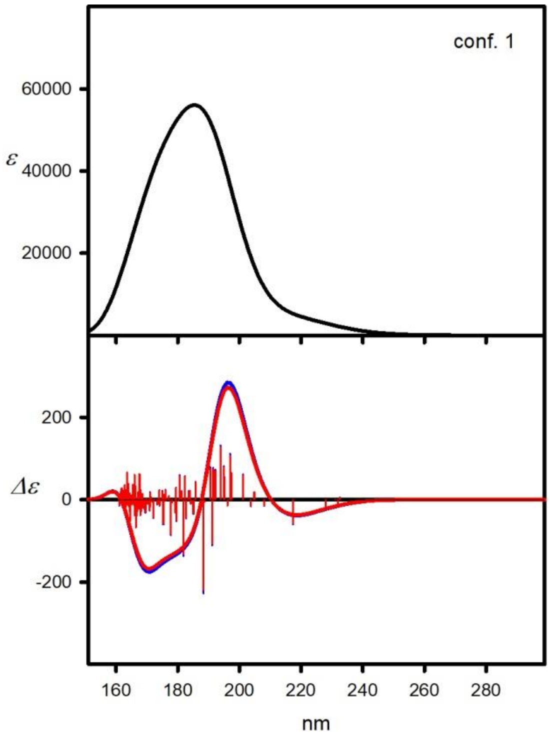

Figure SI_40. UV and ECD spectra of the low-energy conformers of compound **10** calculated at TD-M06-2X/6-311++G(2d,2p) level for structures optimized at B3LYP/6-311++G(d,p) level. Wavelengths were not corrected.

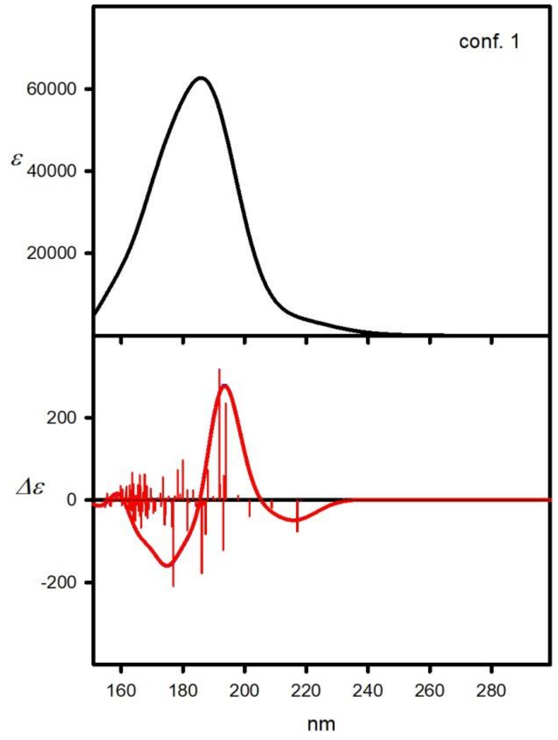

Figure SI_41. UV and ECD spectra of the low-energy conformers of compound **10** calculated at TD-CAM-B3LYP/6-311++G(2d,2p) level for structures optimized at M06-2X/6-311++G(d,p) level. Wavelengths were not corrected.

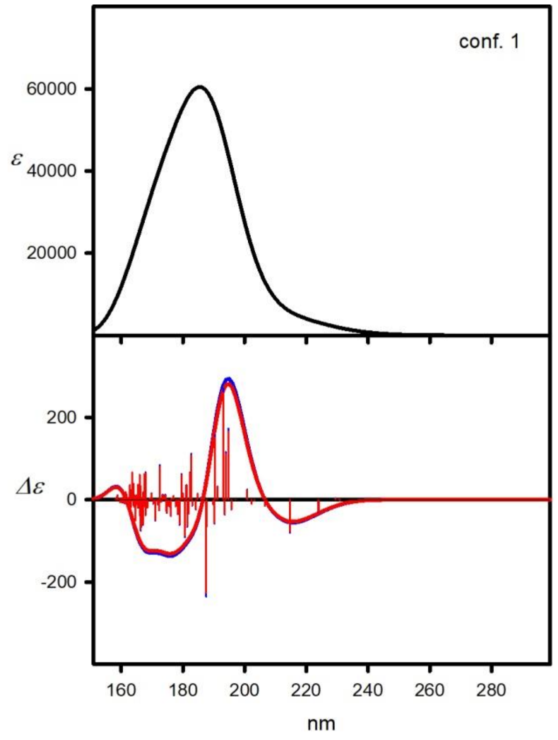

Figure SI_42. UV and ECD spectra of the low-energy conformers of compound **10** calculated at TD-M06-2X/6-311++G(2d,2p) level for structures optimized at M06-2X/6-311++G(d,p) level. Wavelengths were not corrected.

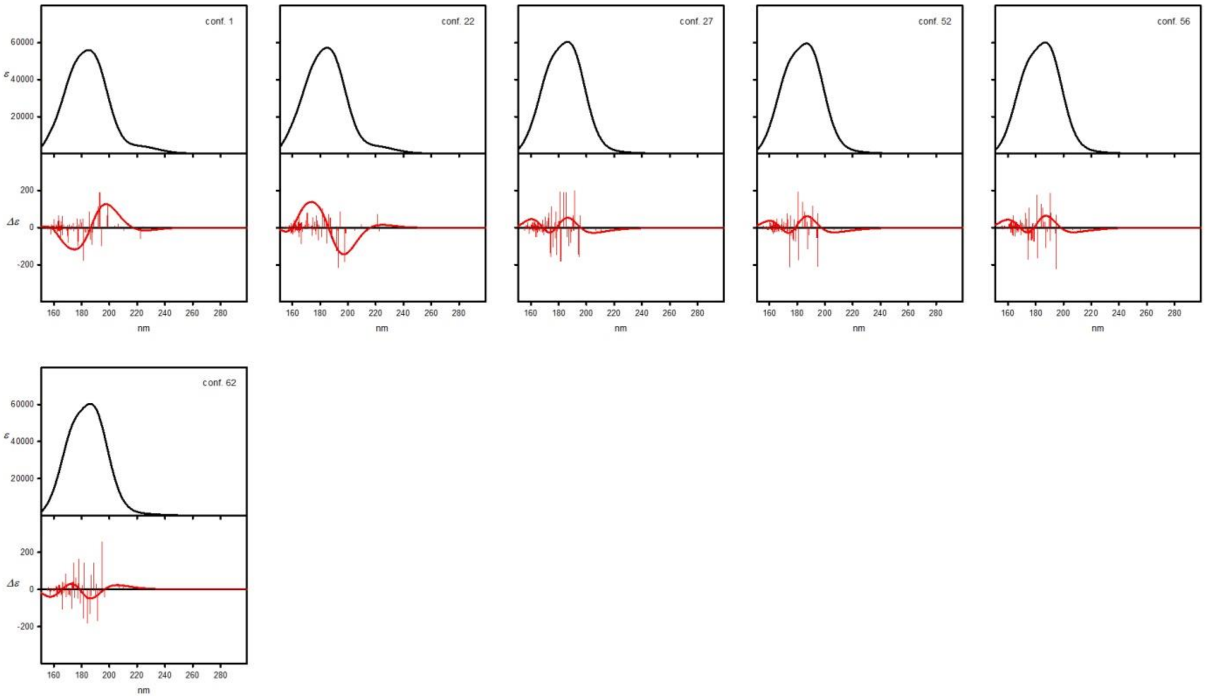

Figure SI_43. UV and ECD spectra of the low-energy conformers of compound **11** calculated at TD-CAM-B3LYP/6-311++G(2d,2p) level for structures optimized at B3LYP/6-311++G(d,p) level. Wavelengths were not corrected.

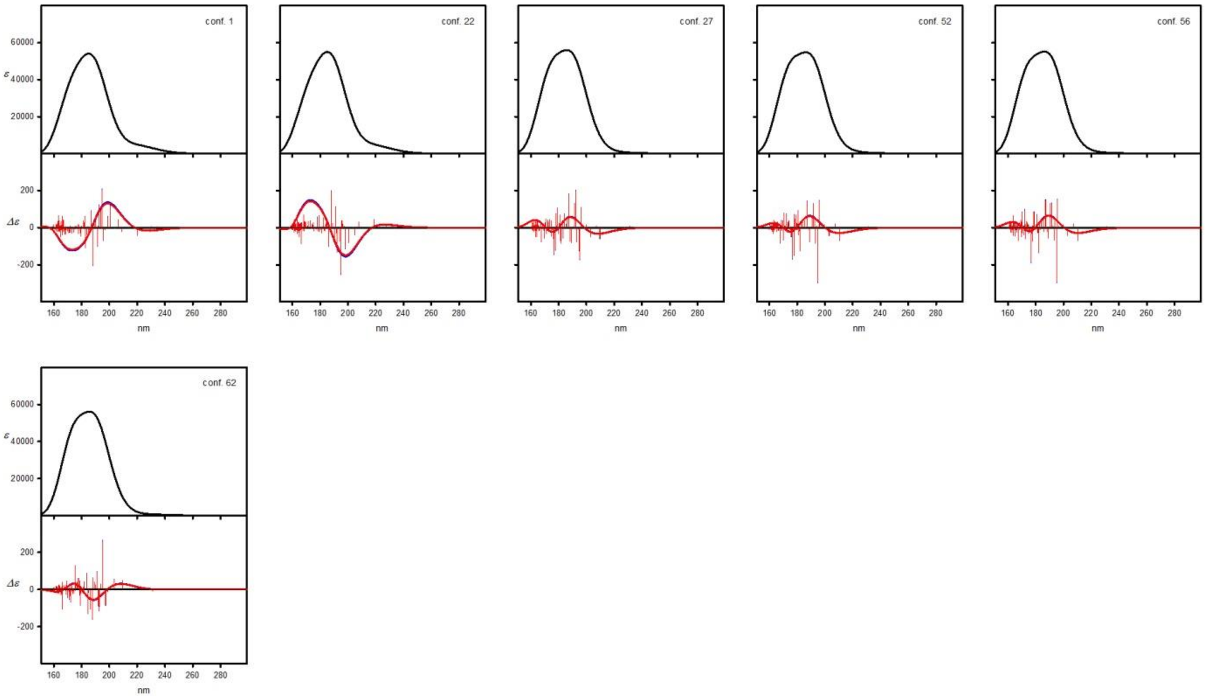

Figure SI_44. UV and ECD spectra of the low-energy conformers of compound **11** calculated at TD-M06-2X/6-311++G(2d,2p) level for structures optimized at B3LYP/6-311++G(d,p) level. Wavelengths were not corrected.

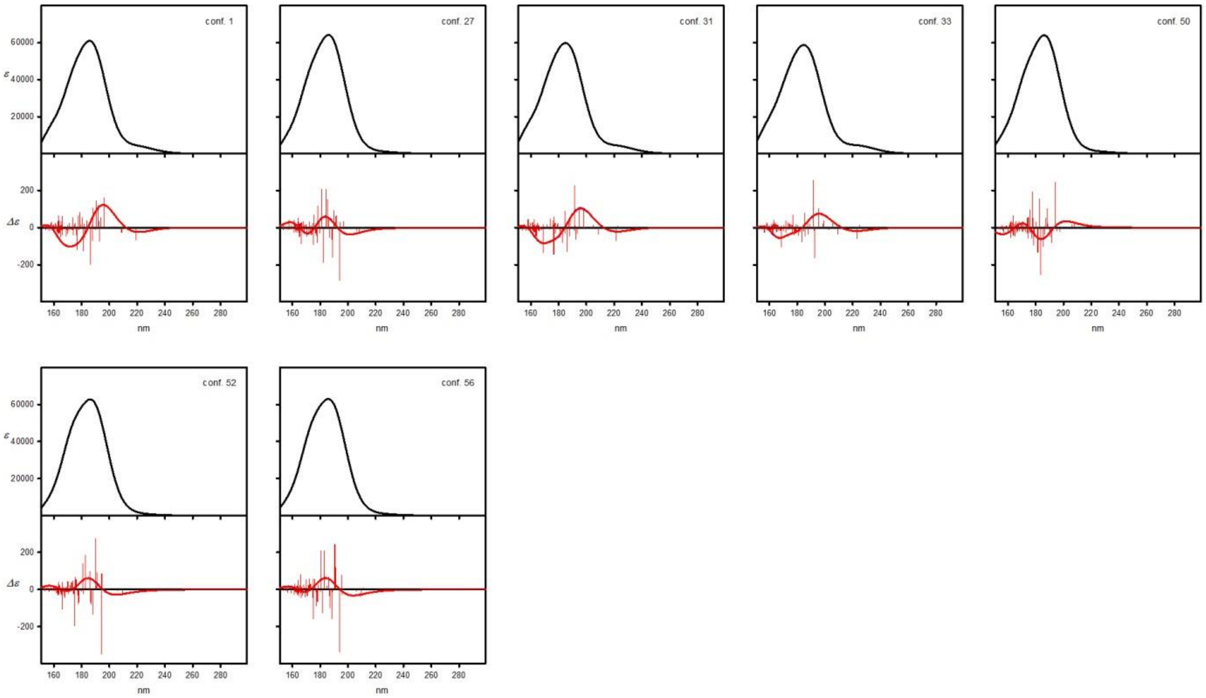

Figure SI_45. UV and ECD spectra of the low-energy conformers of compound **11** calculated at TD-CAM-B3LYP/6-311++G(2d,2p) level for structures optimized at M06-2X/6-311++G(d,p) level. Wavelengths were not corrected.

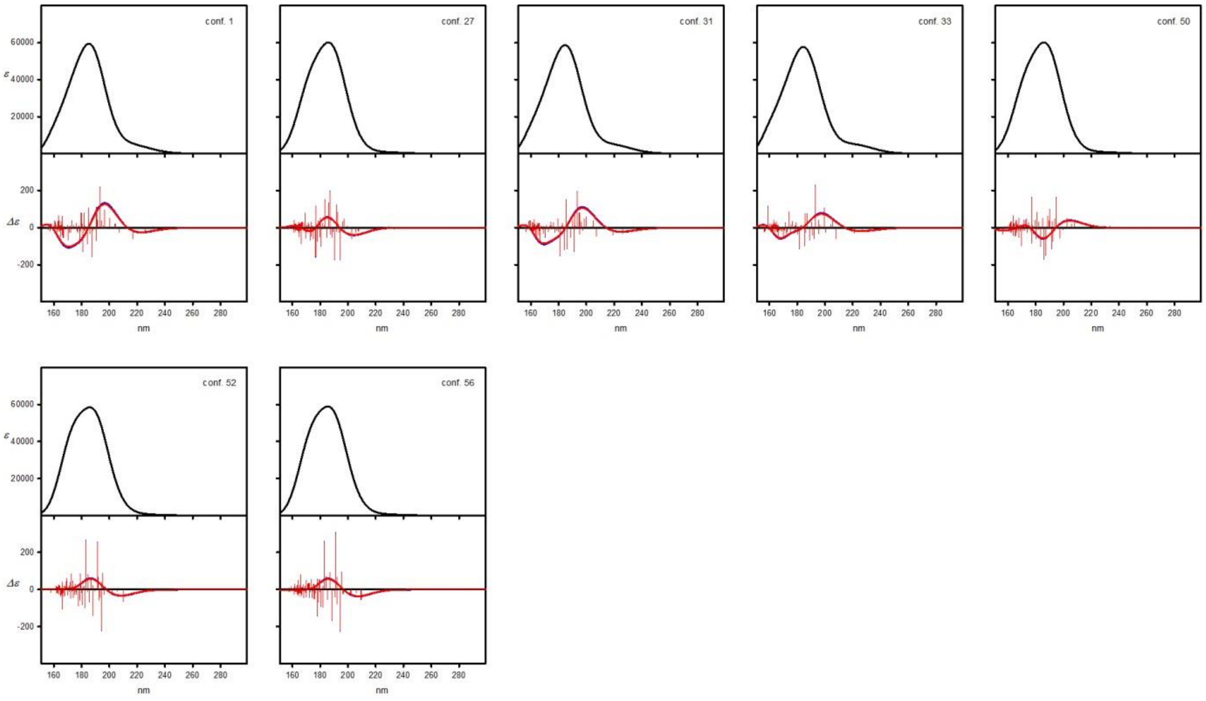

Figure SI_46. UV and ECD spectra of the low-energy conformers of compound **11** calculated at TD-M06-2X/6-311++G(2d,2p) level for structures optimized at M06-2X/6-311++G(d,p) level. Wavelengths were not corrected.

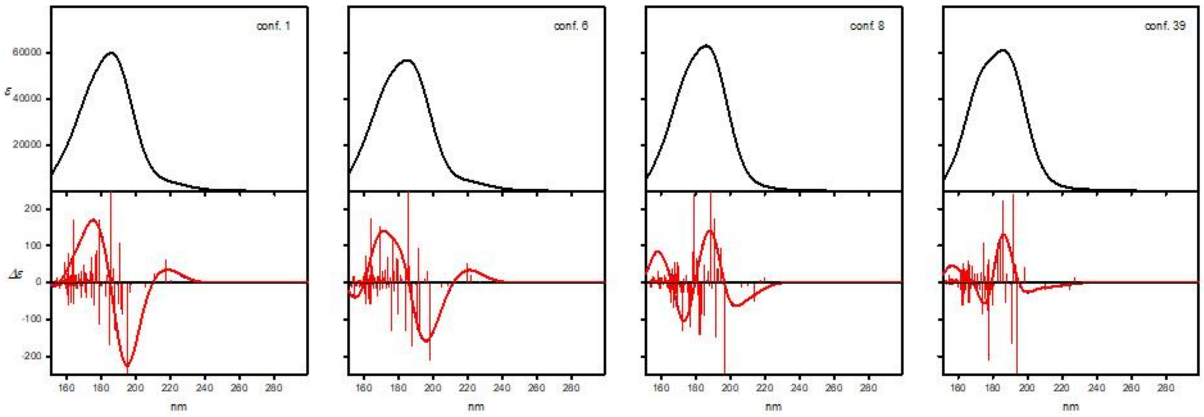

Figure SI_47. UV and ECD spectra of the low-energy conformers of compound **14** calculated at TD-CAM-B3LYP/6-311++G(2d,2p) level for structures optimized at B3LYP/6-311++G(d,p) level. Wavelengths were not corrected.

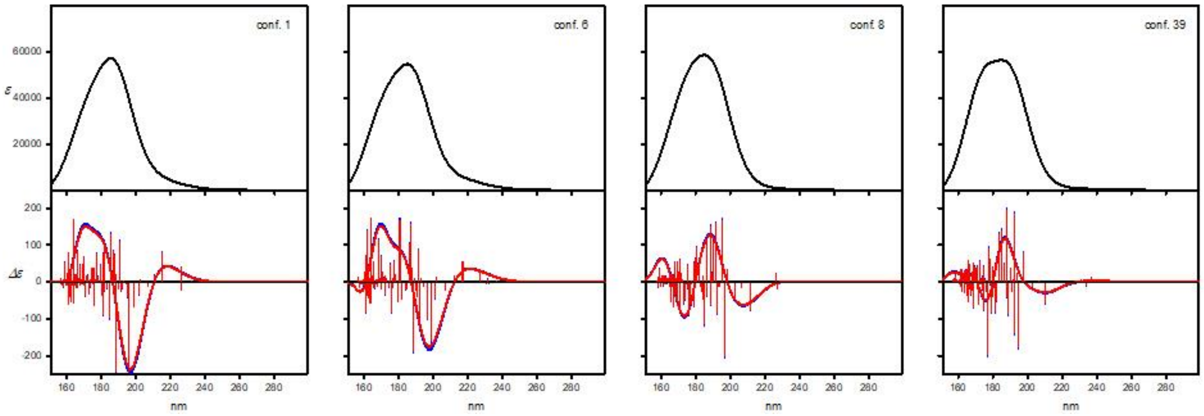

Figure SI_48. UV and ECD spectra of the low-energy conformers of compound **14** calculated at TD-M06-2X/6-311++G(2d,2p) level for structures optimized at B3LYP/6-311++G(d,p) level. Wavelengths were not corrected.

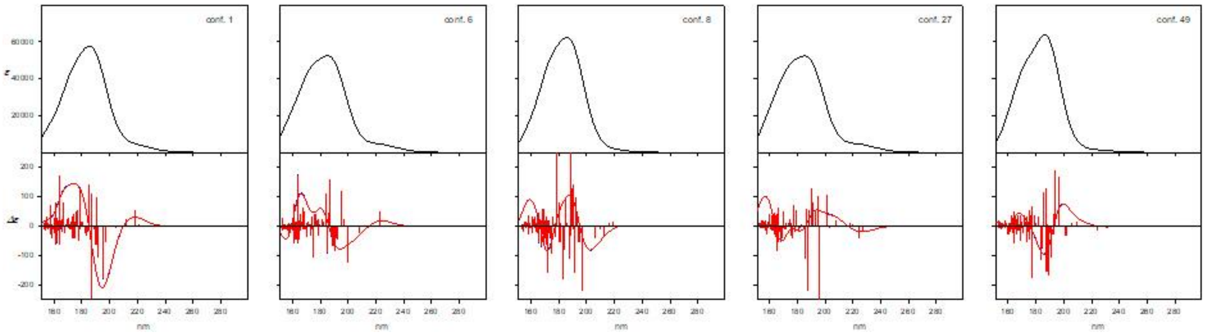

Figure SI_49. UV and ECD spectra of the low-energy conformers of compound **14** calculated at TD-CAM-B3LYP/6-311++G(2d,2p) level for structures optimized at M06-2X/6-311++G(d,p) level. Wavelengths were not corrected.

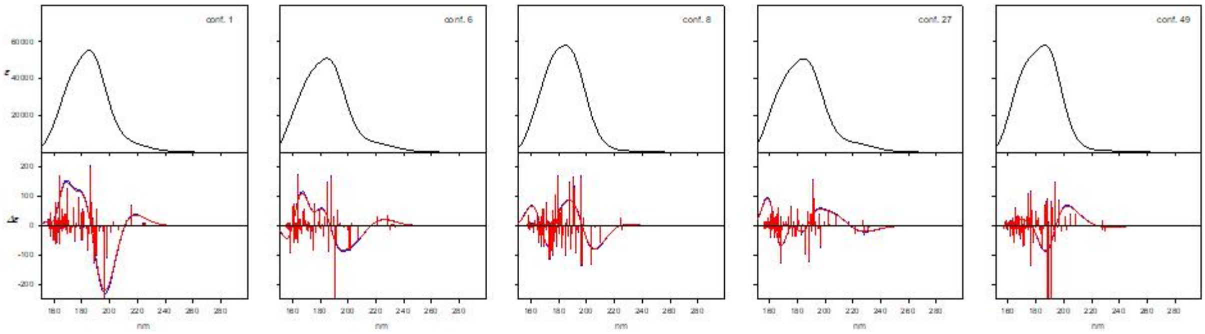

Figure SI_50. UV and ECD spectra of the low-energy conformers of compound **14** calculated at TD-M06-2X/6-311++G(2d,2p) level for structures optimized at M06-2X/6-311++G(d,p) level. Wavelengths were not corrected.

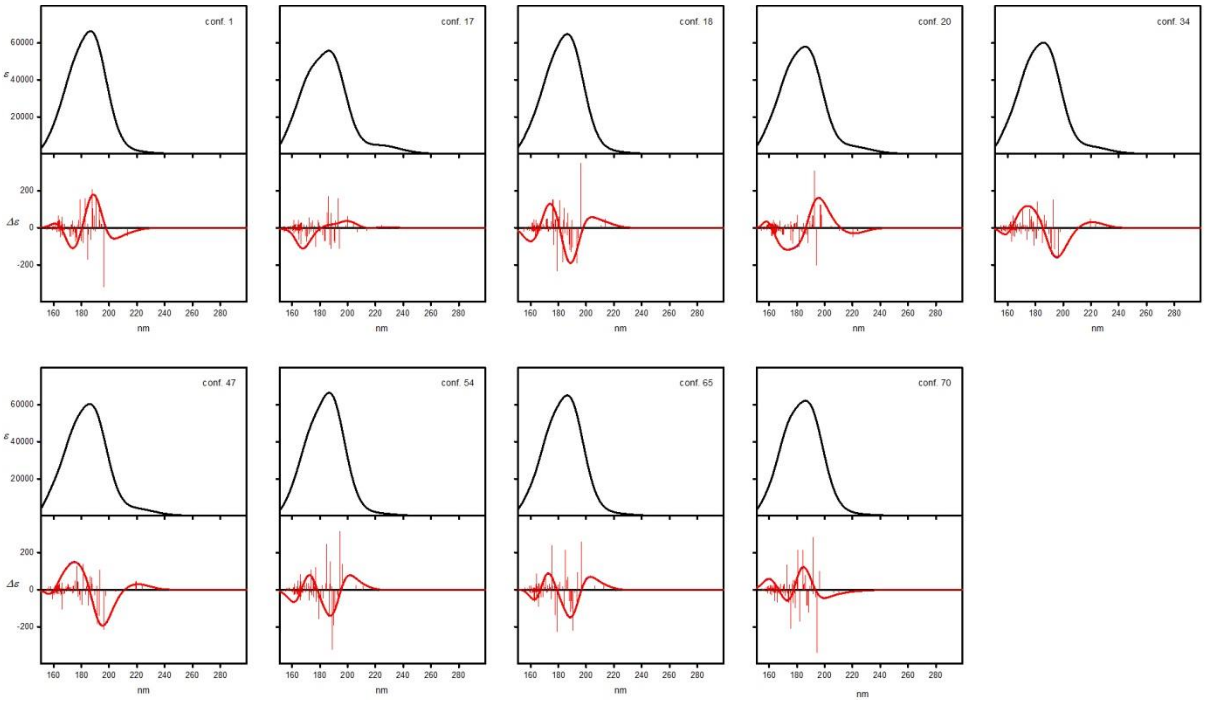

Figure SI_51. UV and ECD spectra of the low-energy conformers of compound **15** calculated at TD-CAM-B3LYP/6-311++G(2d,2p) level for structures optimized at B3LYP/6-311++G(d,p) level. Wavelengths were not corrected.

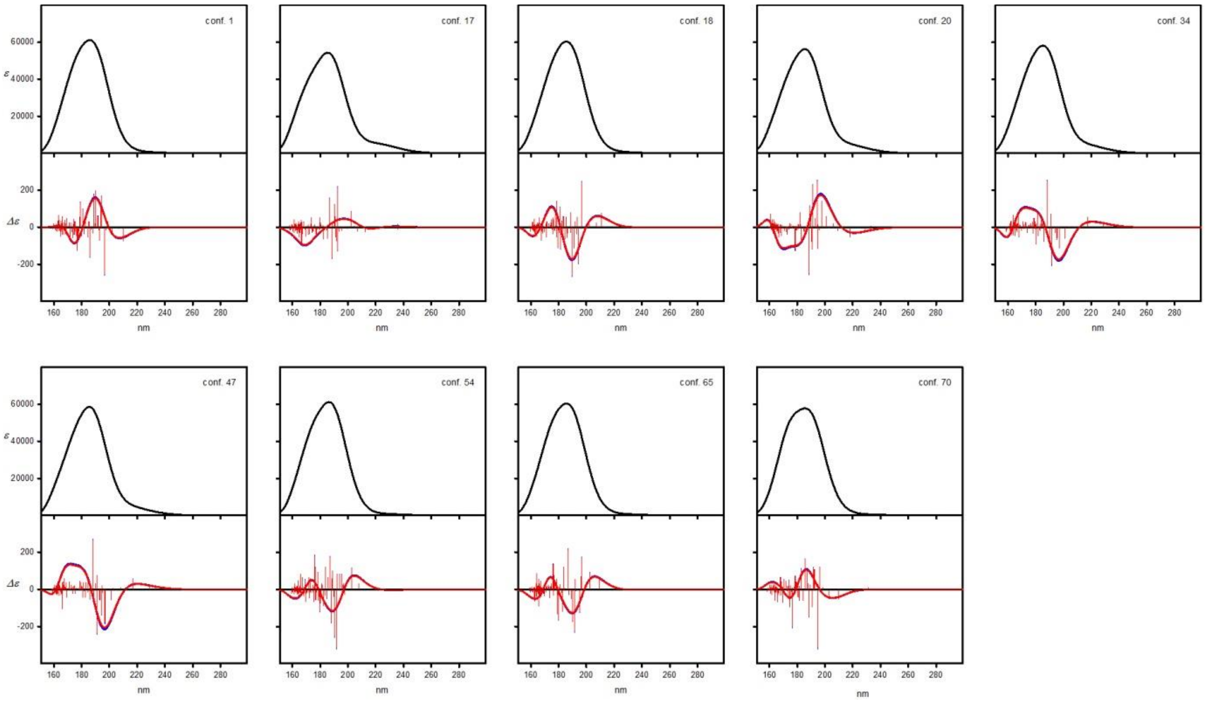

Figure SI_52. UV and ECD spectra of the low-energy conformers of compound **15** calculated at TD-M06-2X/6-311++G(2d,2p) level for structures optimized at B3LYP/6-311++G(d,p) level. Wavelengths were not corrected.

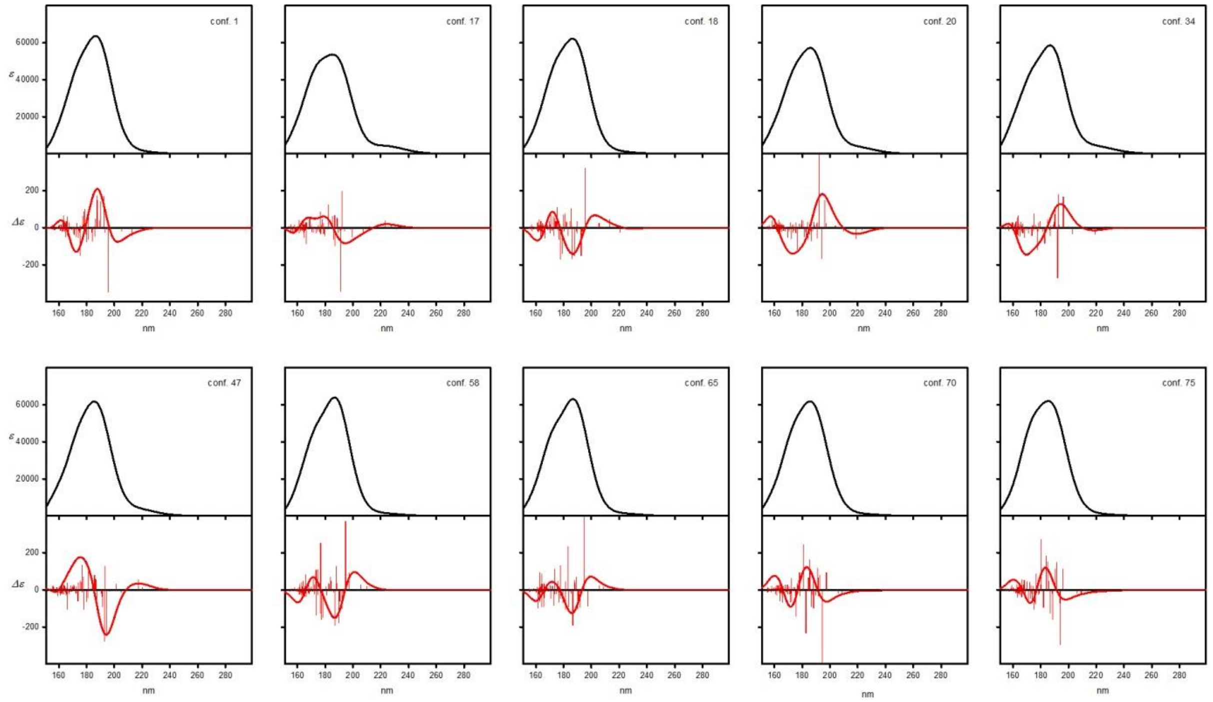

Figure SI_53. UV and ECD spectra of the low-energy conformers of compound **15** calculated at TD-CAM-B3LYP/6-311++G(2d,2p) level for structures optimized at M06-2X/6-311++G(d,p) level. Wavelengths were not corrected.

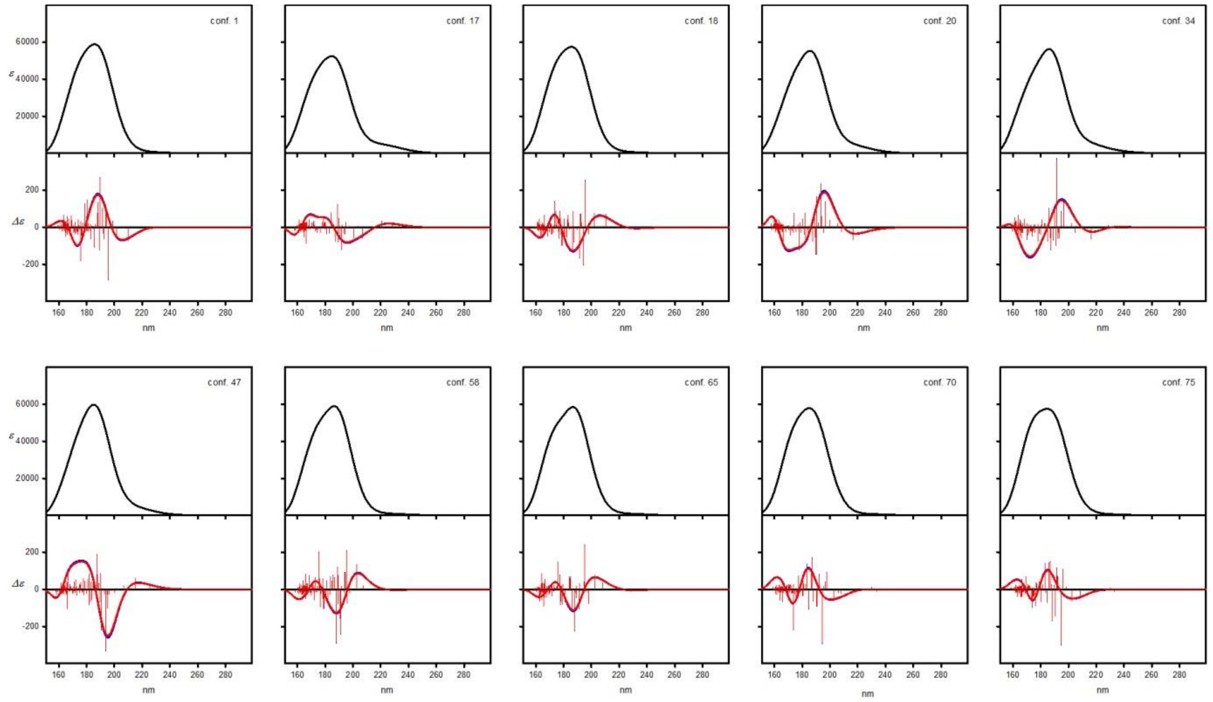

Figure SI_54. UV and ECD spectra of the low-energy conformers of compound **15** calculated at TD-M06-2X/6-311++G(2d,2p) level for structures optimized at M06-2X/6-311++G(d,p) level. Wavelengths were not corrected.

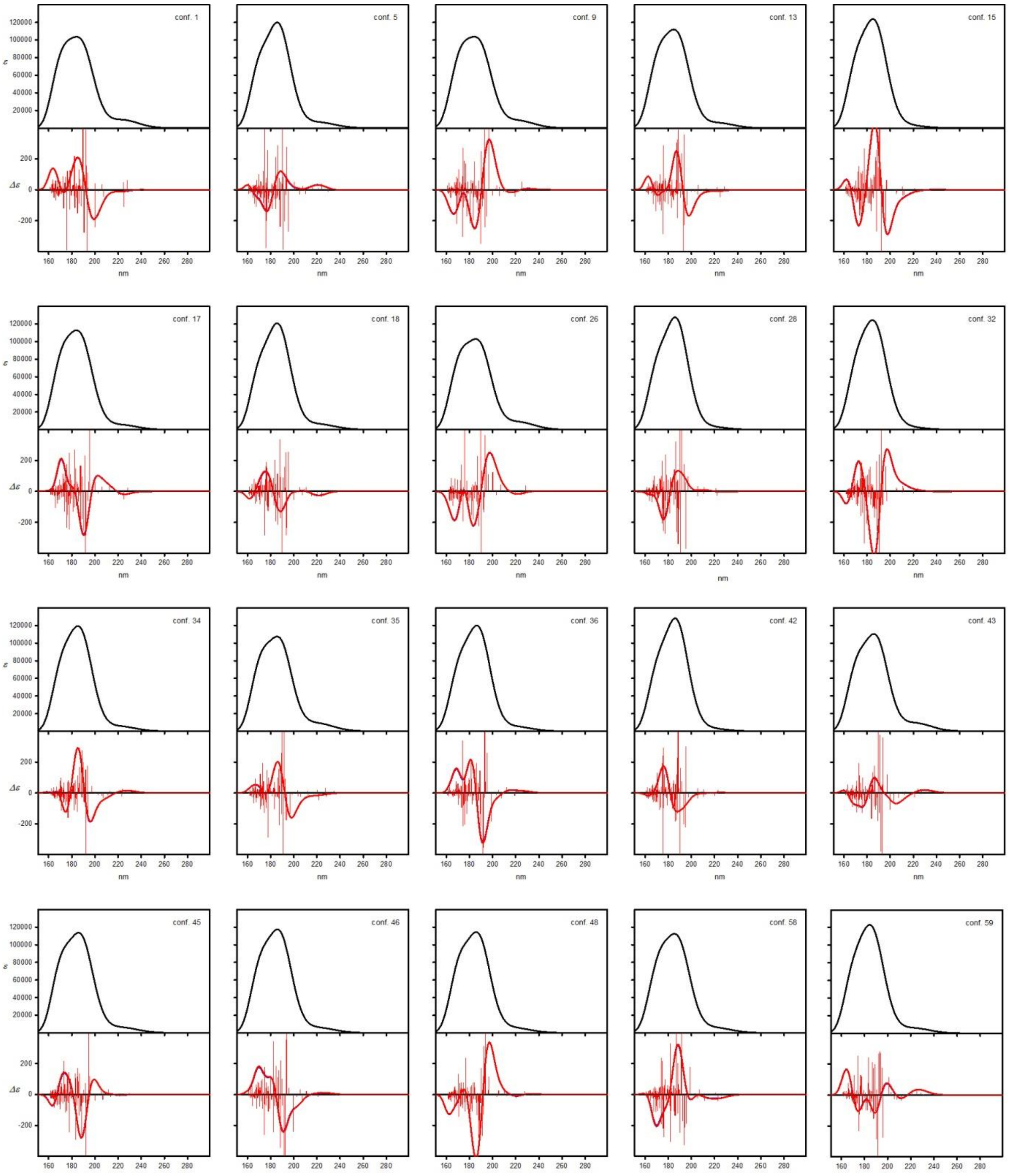
conf. 1
conf. 5
conf. 9
conf. 13
conf. 15
conf. 17
conf. 18
conf. 26
conf. 28
conf. 32
conf. 34
conf. 35
conf. 36
conf. 42
conf. 43
conf. 45
conf. 46
conf. 48
conf. 58
conf. 59
ε
Δε
nm

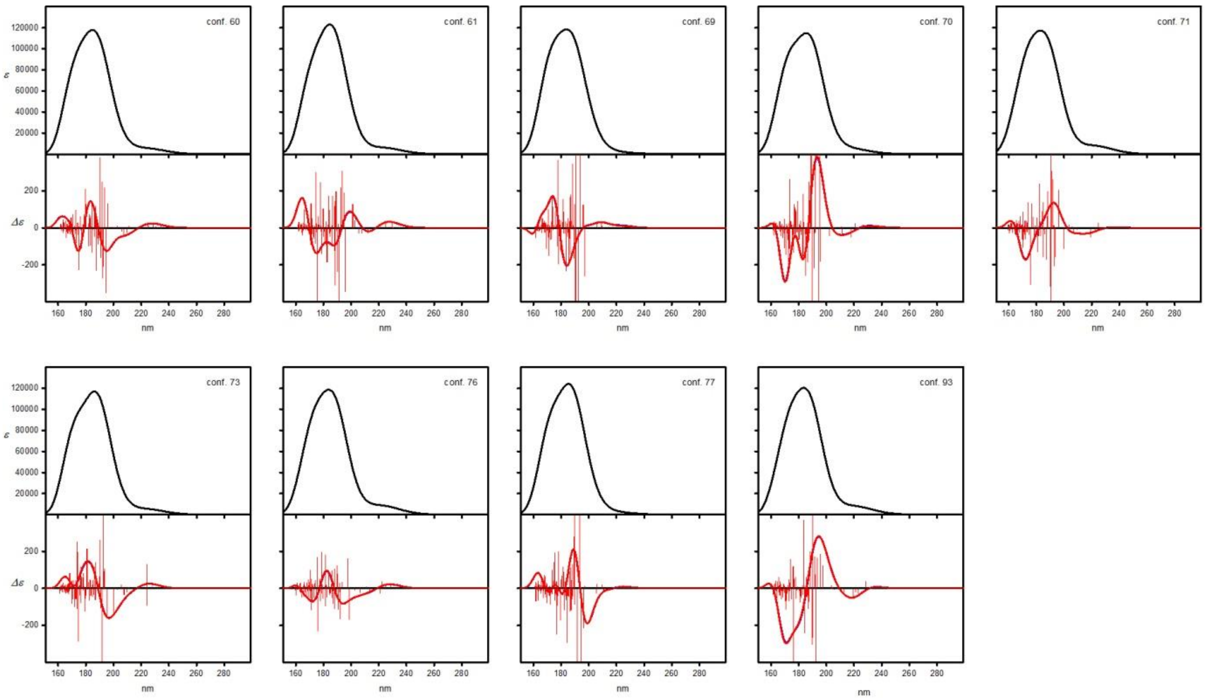

Figure SI_55. UV and ECD spectra of the low-energy conformers of compound **18** calculated at TD-CAM-B3LYP/6-311++G(d,p) level for structures optimized at B3LYP/6-311G(d,p) level. Wavelengths were not corrected.

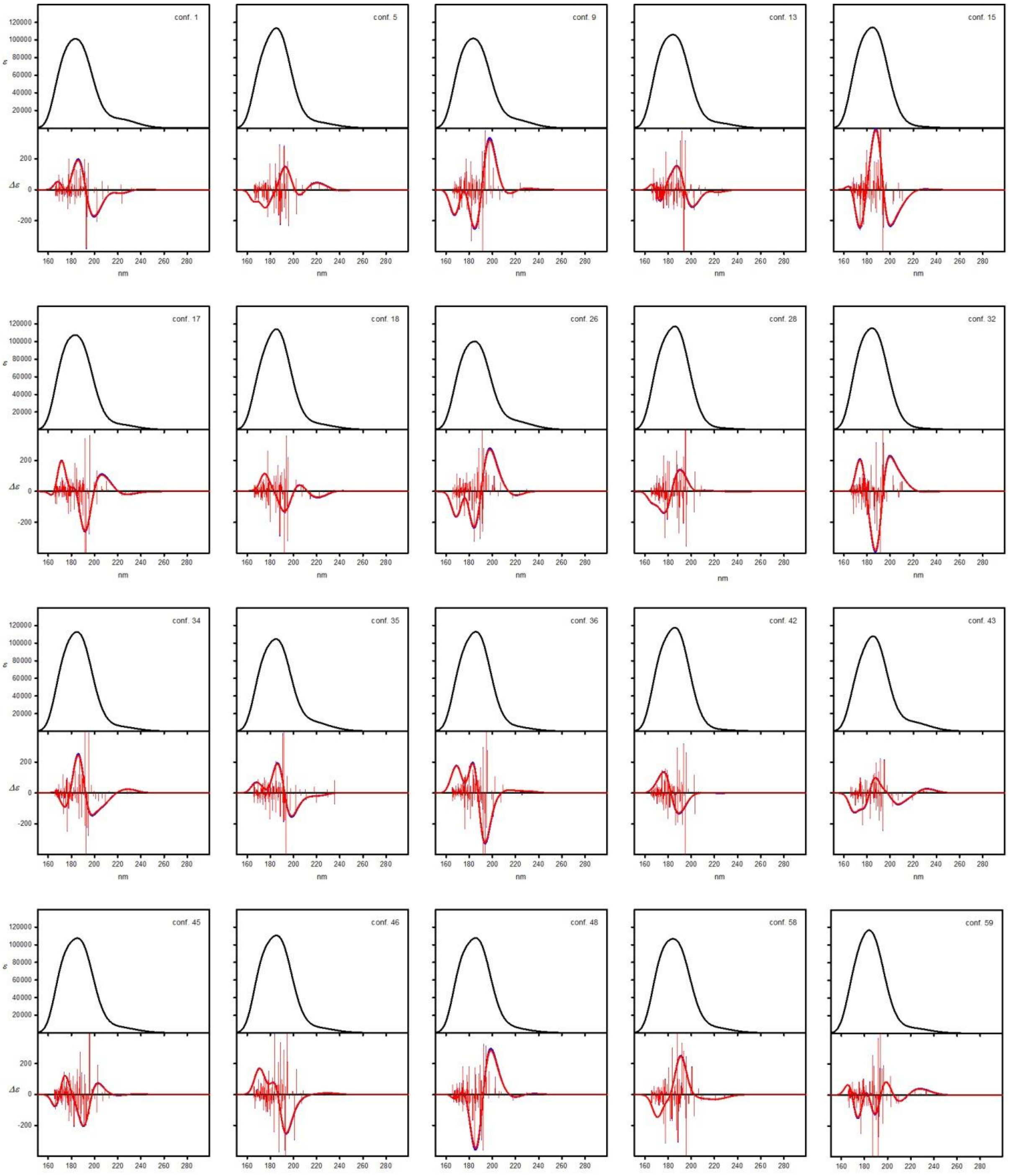
conf. 1
conf. 5
conf. 9
conf. 13
conf. 15
conf. 17
conf. 18
conf. 26
conf. 28
conf. 32
conf. 34
conf. 35
conf. 36
conf. 42
conf. 43
conf. 45
conf. 46
conf. 48
conf. 58
conf. 59
ε
Δε
nm

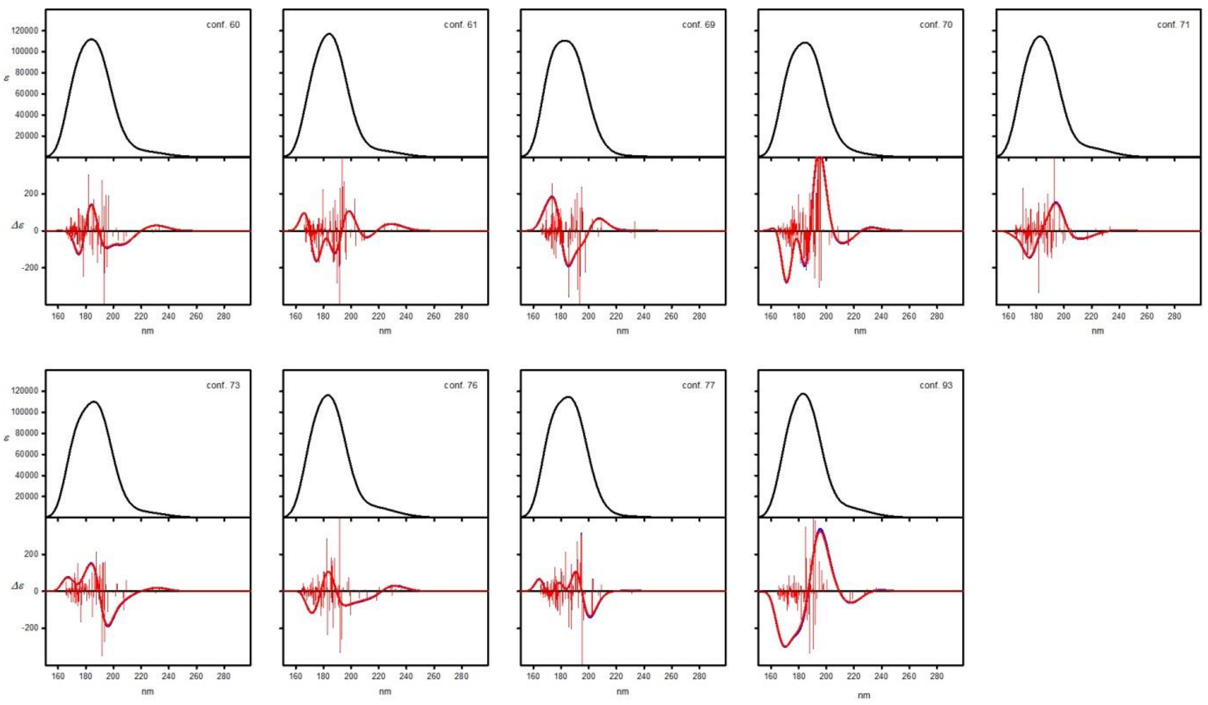

Figure SI_56. UV and ECD spectra of the low-energy conformers of compound **18** calculated at TD-M06-2X/6-311++G(d,p) level for structures optimized at B3LYP/6-311G(d,p) level. Wavelengths were not corrected.

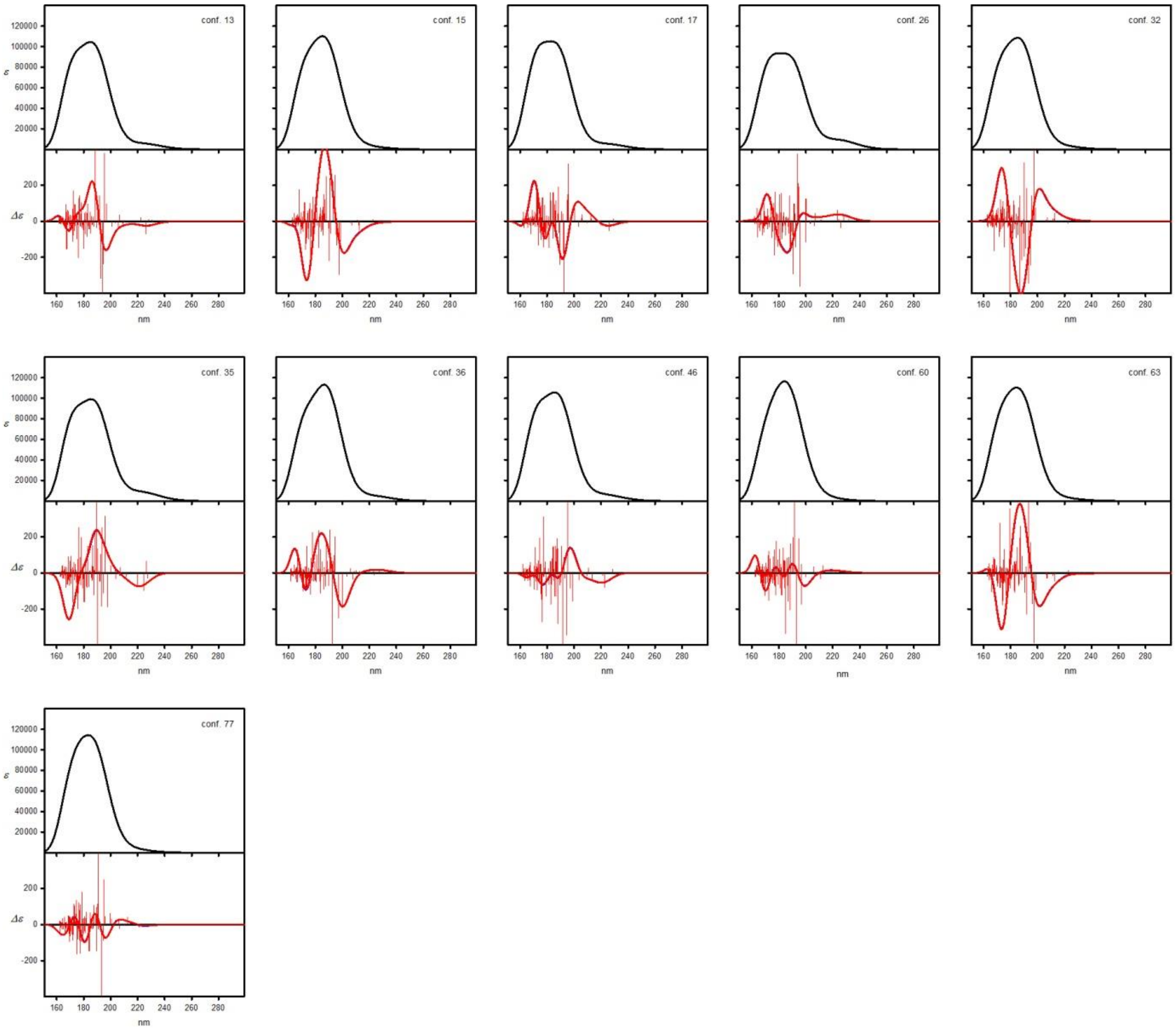

Figure SI_57. UV and ECD spectra of the low-energy conformers of compound **18** calculated at TD-CAM-B3LYP/6-311++G(d,p) level for structures optimized at B3LYP-GD3BJ/6-311G(d,p) level. Wavelengths were not corrected.

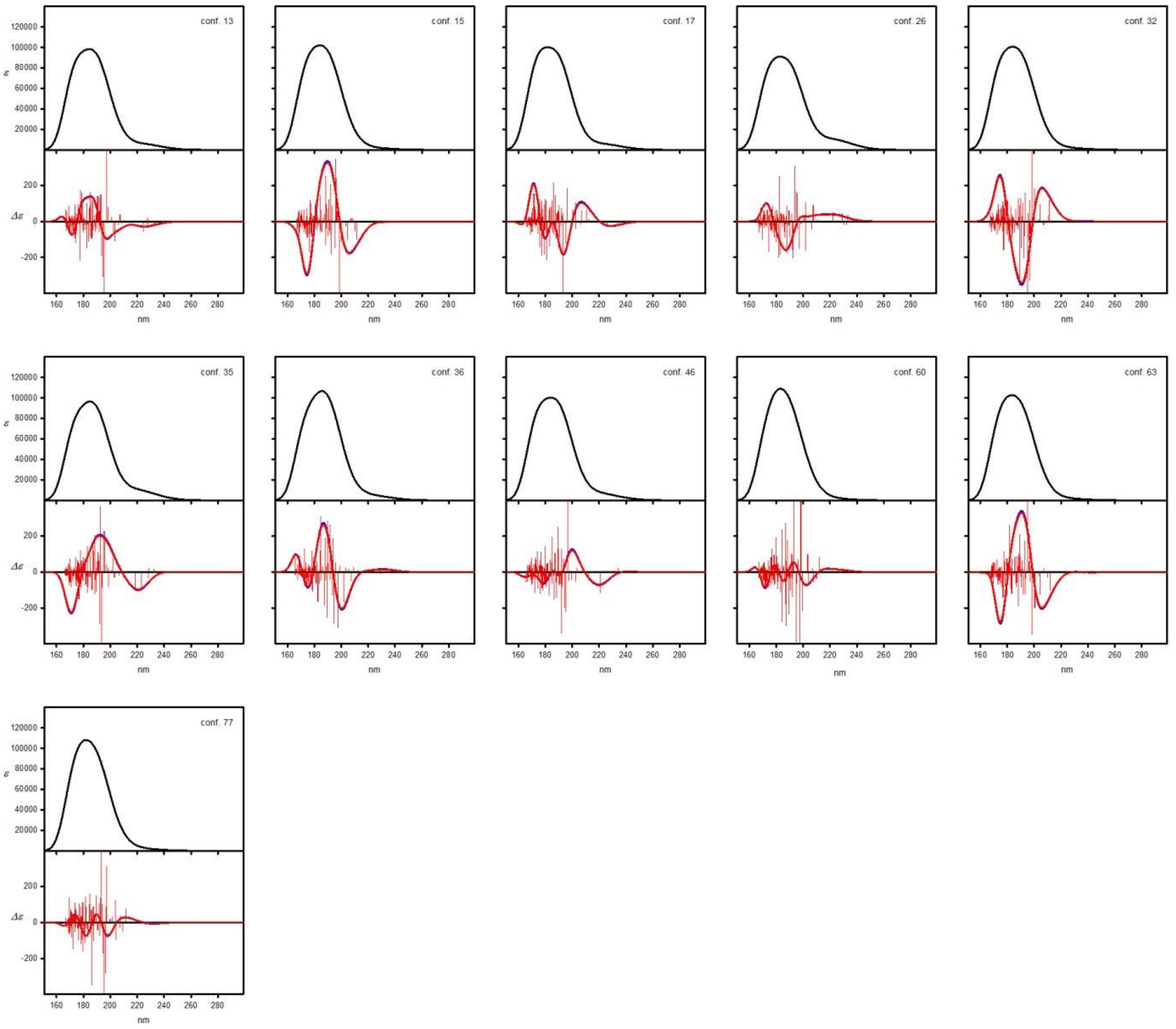

Figure SI_58. UV and ECD spectra of the low-energy conformers of compound **18** calculated at TD-M06-2X/6-311++G(d,p) level for structures optimized at B3LYP-GD3BJ/6-311G(d,p) level. Wavelengths were not corrected.

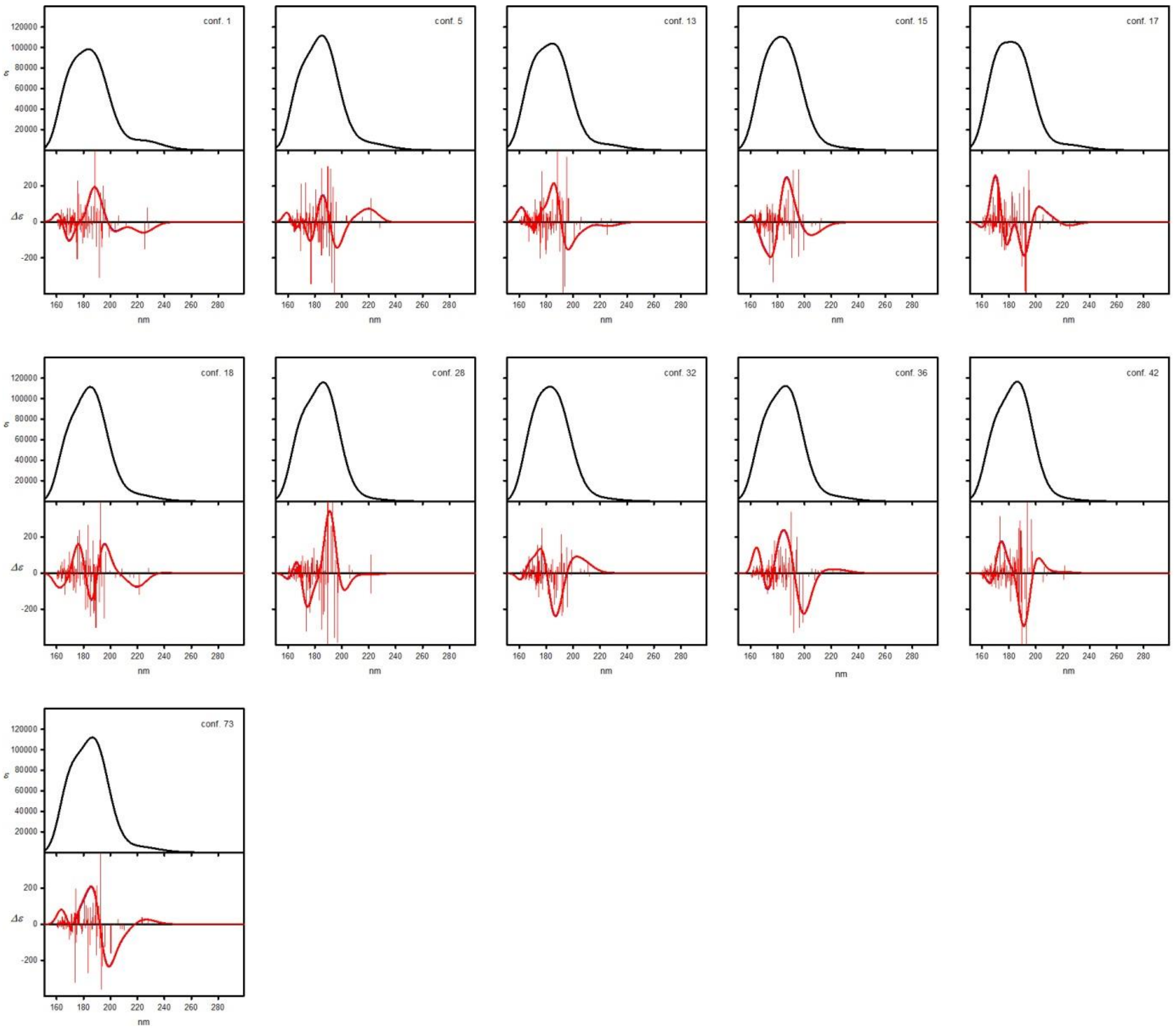

Figure SI_59. UV and ECD spectra of the low-energy conformers of compound **18** calculated at TD-CAM-B3LYP/6-311++G(d,p) level for structures optimized at M06-2X/6-311G(d,p) level. Wavelengths were not corrected.

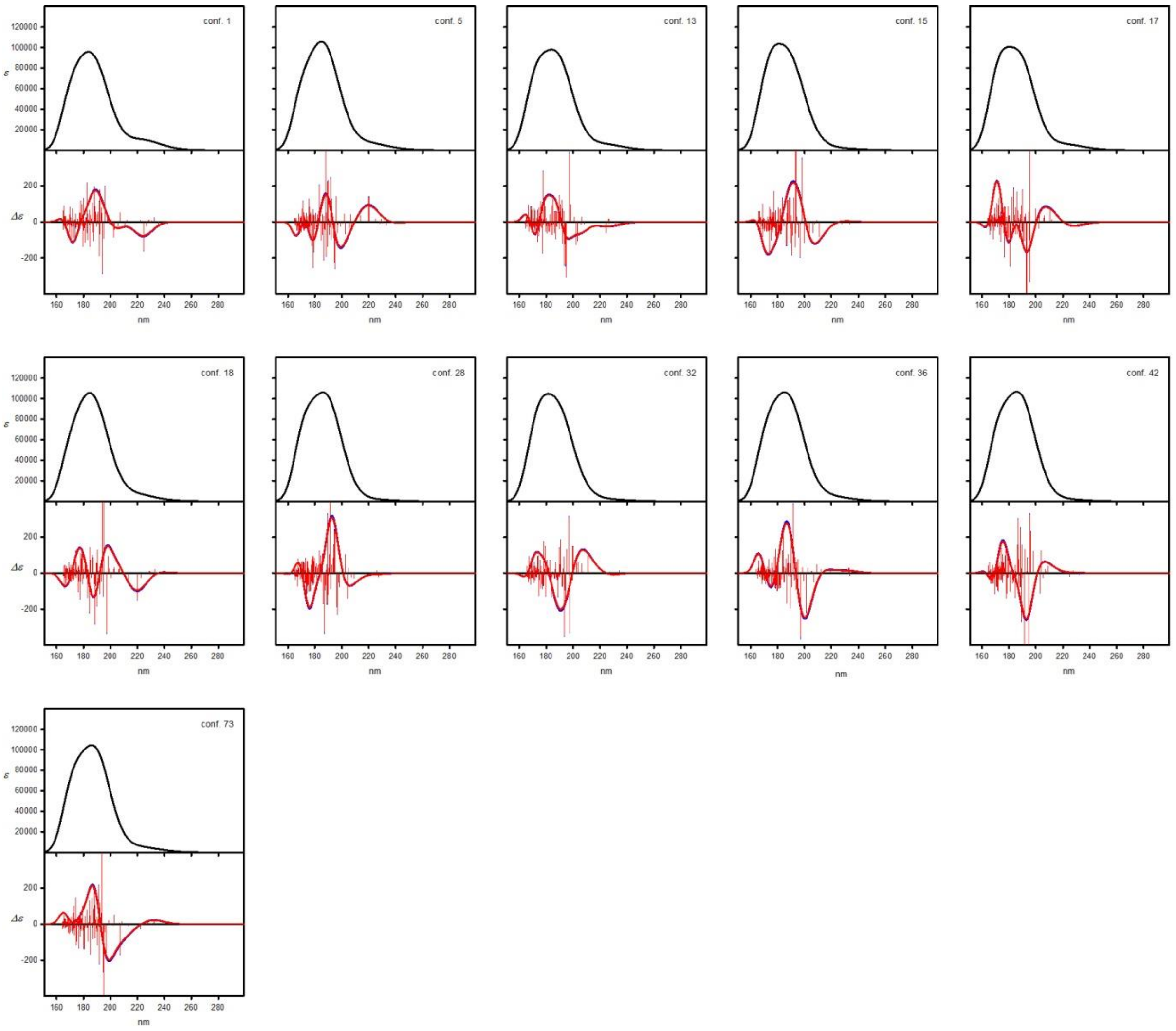

Figure SI_60. UV and ECD spectra of the low-energy conformers of compound **18** calculated at TD-M06-2X/6-311++G(d,p) level for structures optimized at M06-2X/6-311G(d,p) level. Wavelengths were not corrected.

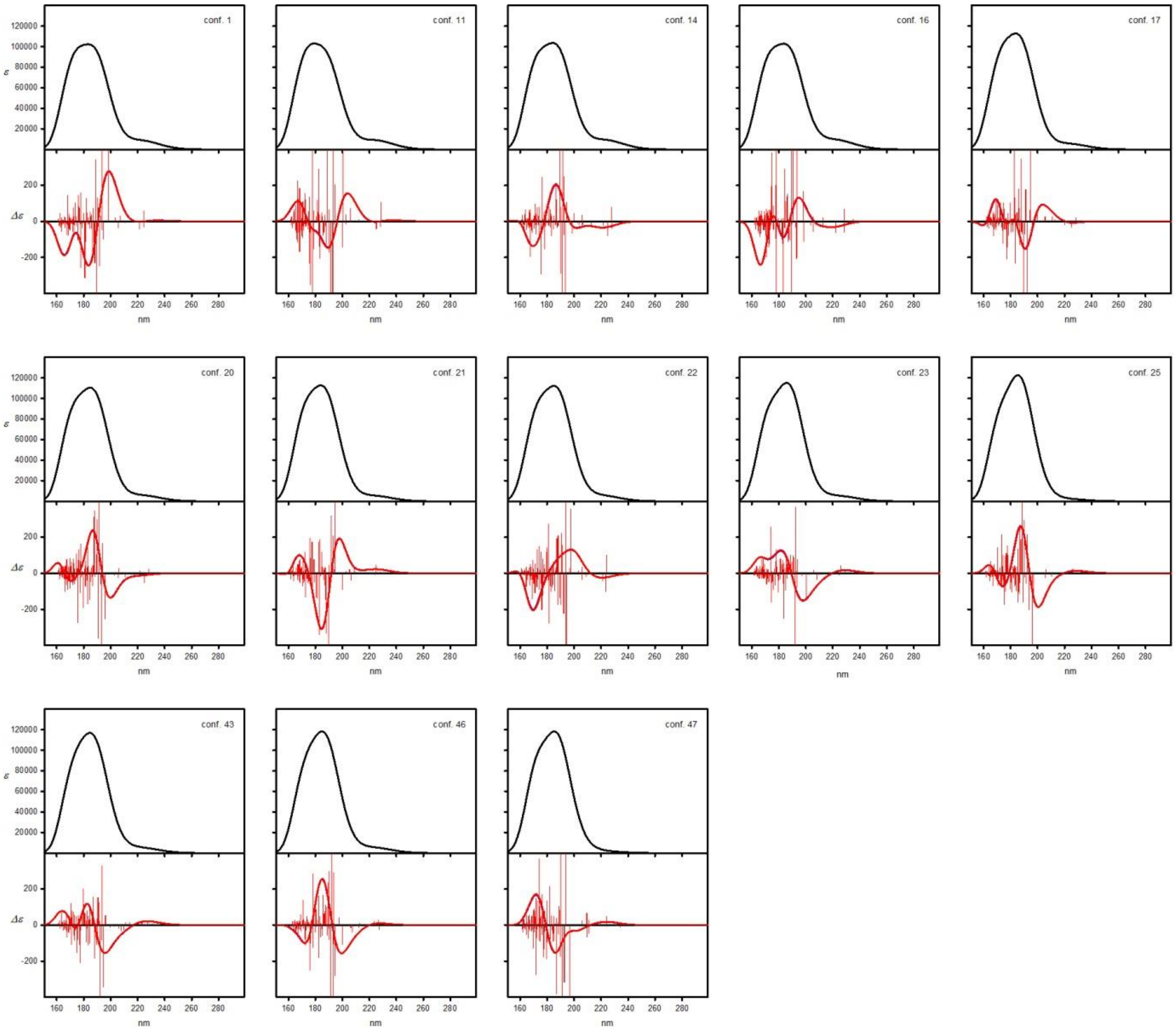

Figure SI_61. UV and ECD spectra of the low-energy conformers of compound **20** calculated at TD-CAM-B3LYP/6-311++G(d,p) level for structures optimized at B3LYP/6-311G(d,p) level. Wavelengths were not corrected.

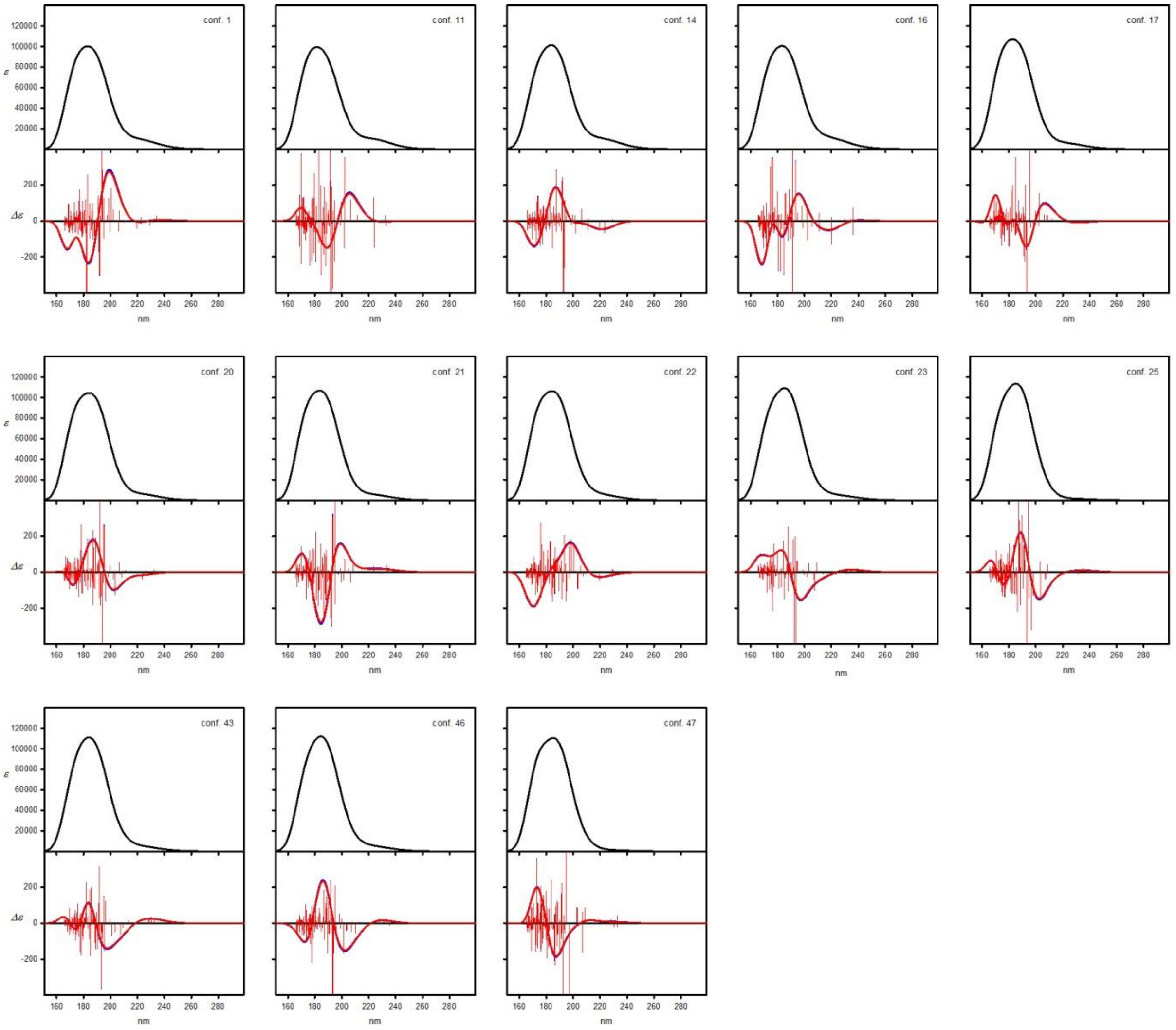

Figure SI_62. UV and ECD spectra of the low-energy conformers of compound **20** calculated at TD-M06-2X/6-311++G(d,p) level for structures optimized at B3LYP/6-311G(d,p) level. Wavelengths were not corrected.

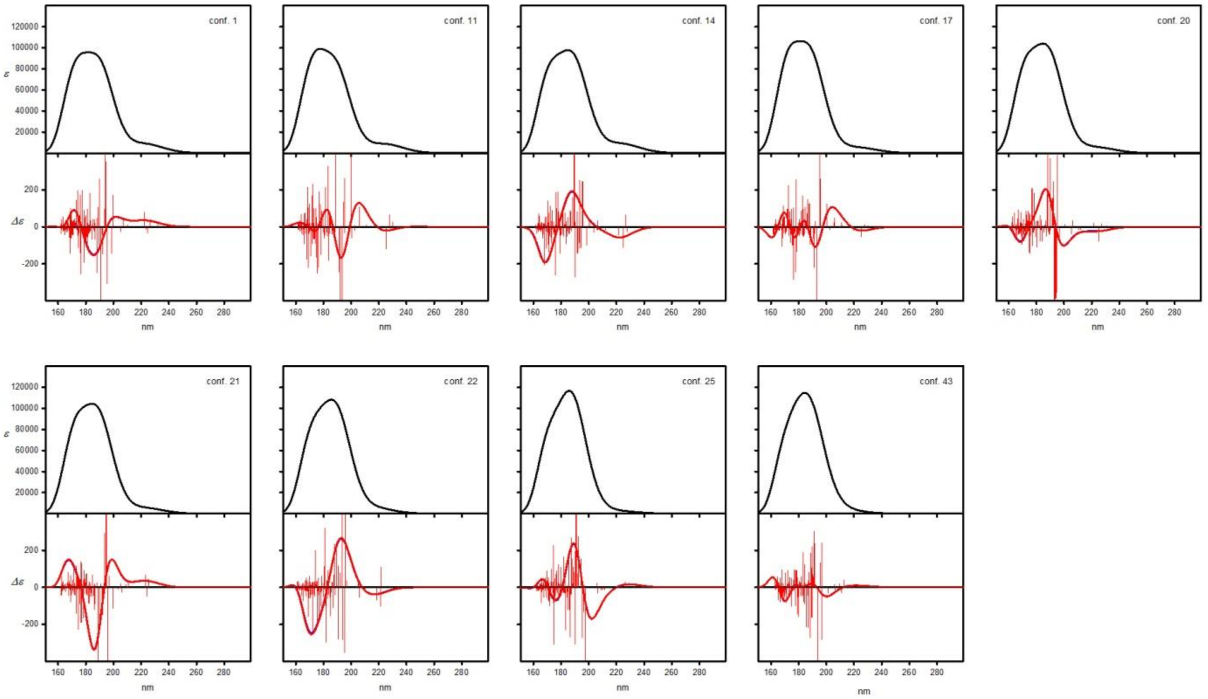

Figure SI_63. UV and ECD spectra of the low-energy conformers of compound **20** calculated at TD-CAM-B3LYP/6-311++G(d,p) level for structures optimized at B3LYP-GD3BJ/6-311G(d,p) level. Wavelengths were not corrected.

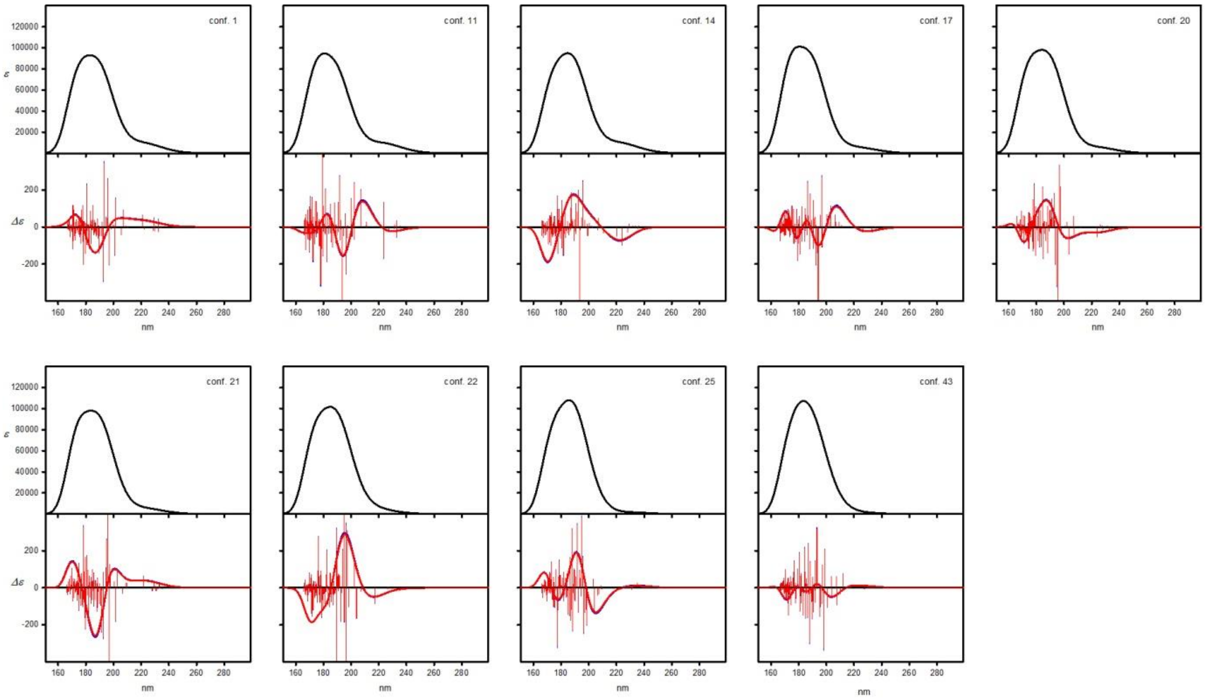

Figure SI_64. UV and ECD spectra of the low-energy conformers of compound **20** calculated at TD-M06-2X/6-311++G(d,p) level for structures optimized at B3LYP-GD3BJ/6-311G(d,p) level. Wavelengths were not corrected.

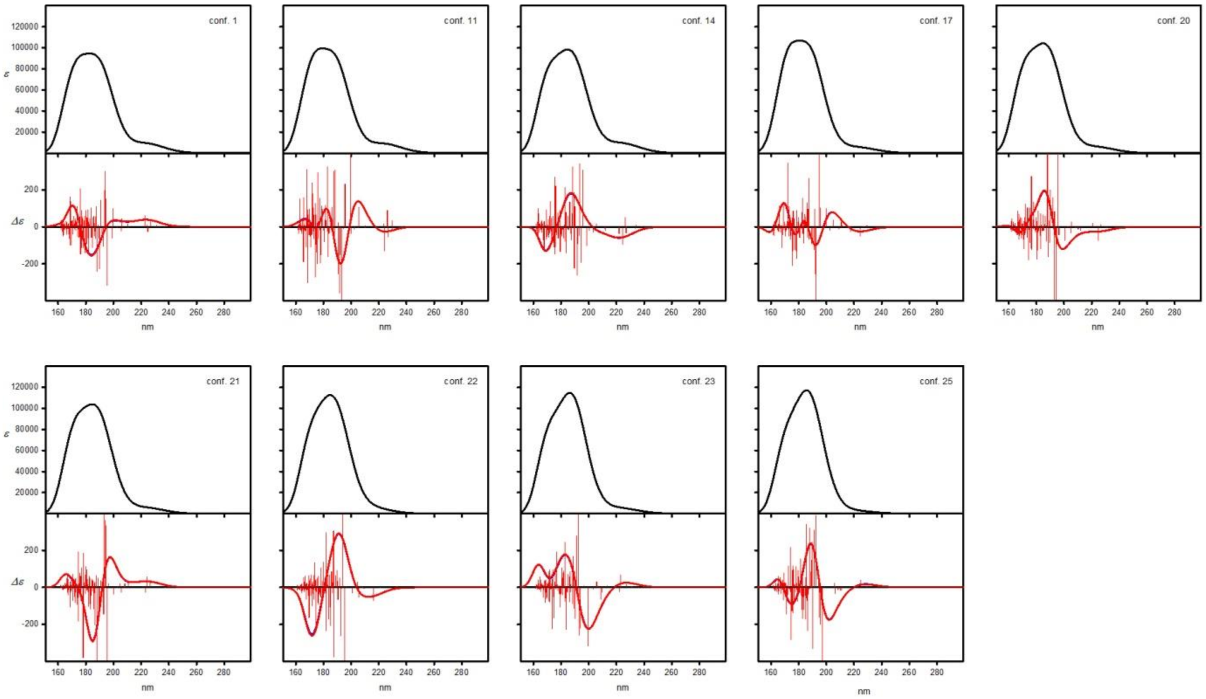

Figure SI_65. UV and ECD spectra of the low-energy conformers of compound **20** calculated at TD-CAM-B3LYP/6-311++G(d,p) level for structures optimized at M06-2X/6-311G(d,p) level. Wavelengths were not corrected.

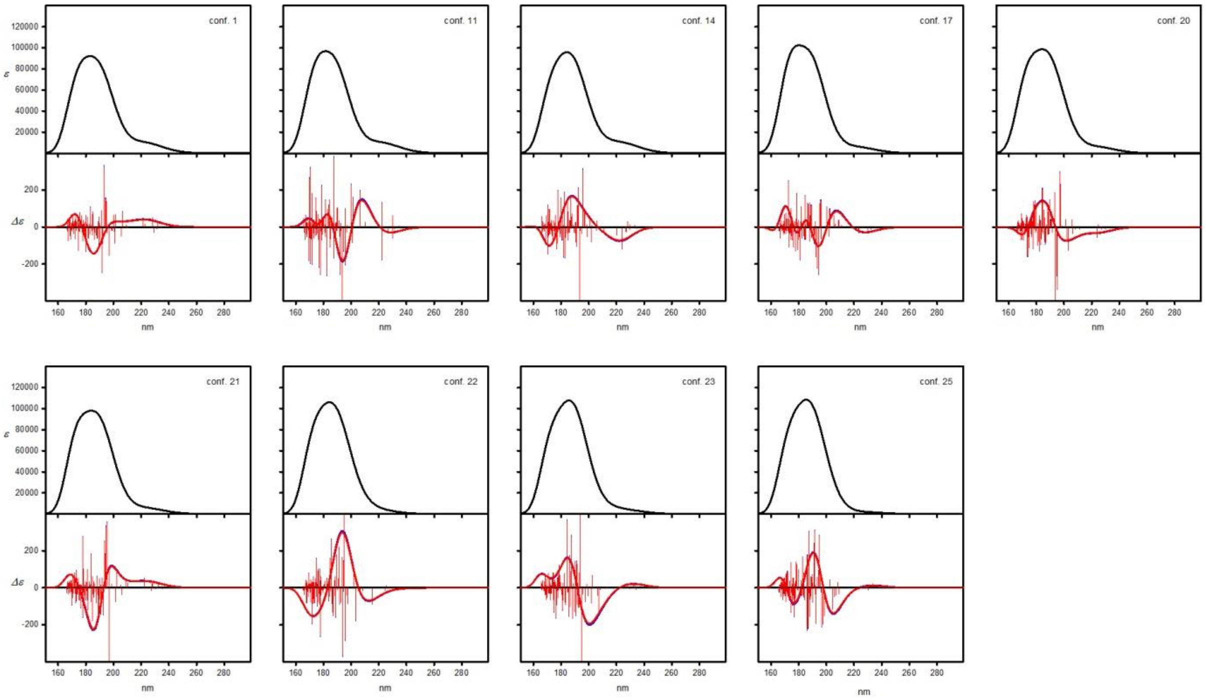

Figure SI_66. UV and ECD spectra of the low-energy conformers of compound **20** calculated at TD-M06-2X/6-311++G(d,p) level for structures optimized at M06-2X/6-311G(d,p) level. Wavelengths were not corrected.

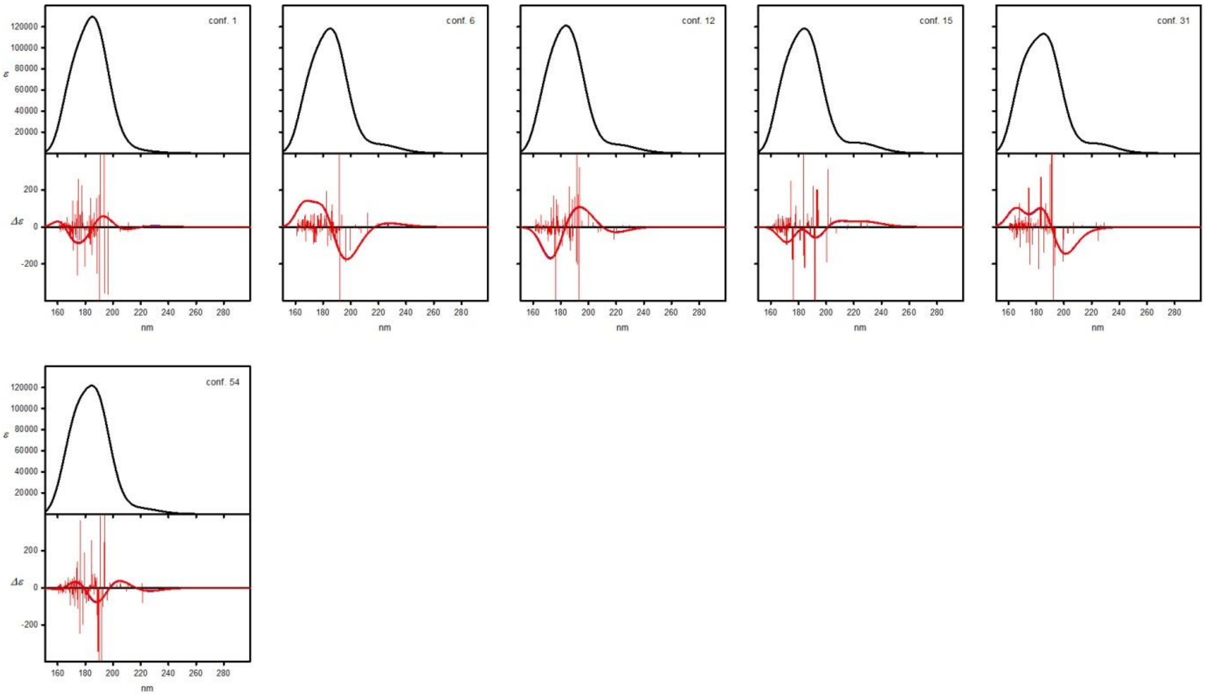

Figure SI_67. UV and ECD spectra of the low-energy conformers of compound **21** calculated at TD-CAM-B3LYP/6-311++G(d,p) level for structures optimized at B3LYP/6-311G(d,p) level. Wavelengths were not corrected.

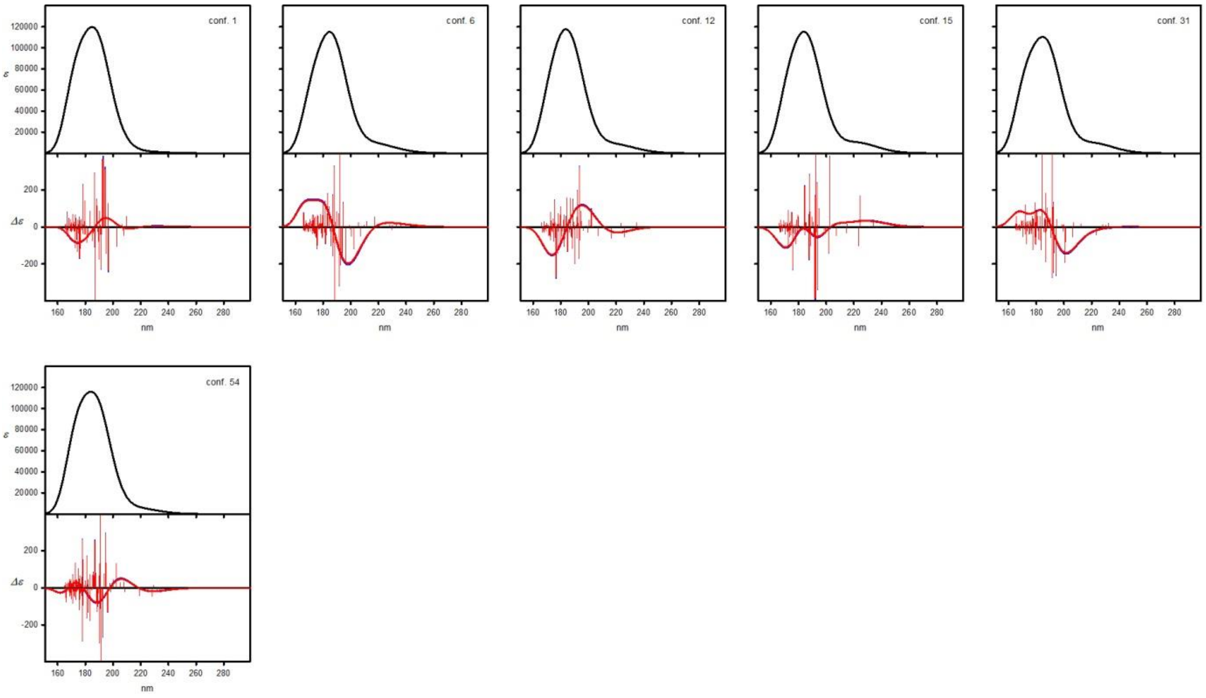

Figure SI_68. UV and ECD spectra of the low-energy conformers of compound **21** calculated at TD-M06-2X/6-311++G(d,p) level for structures optimized at B3LYP/6-311G(d,p) level. Wavelengths were not corrected.

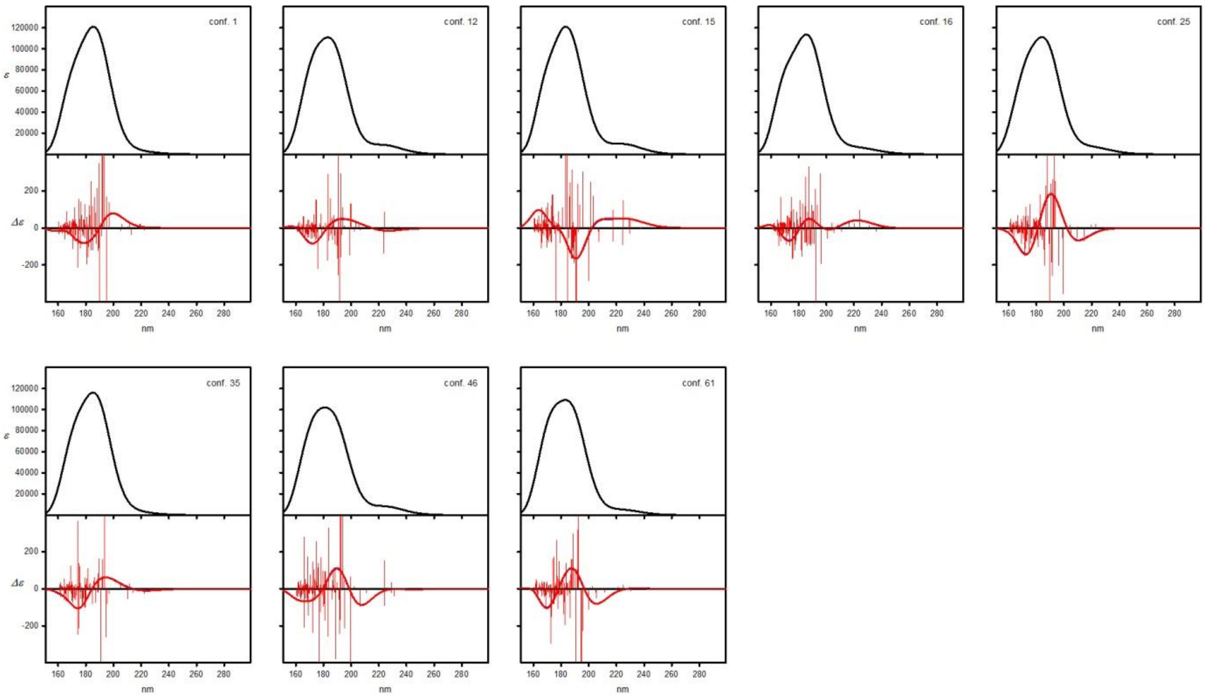

Figure SI_69. UV and ECD spectra of the low-energy conformers of compound **21** calculated at TD-CAM-B3LYP/6-311++G(d,p) level for structures optimized at B3LYP-GD3BJ/6-311G(d,p) level. Wavelengths were not corrected.

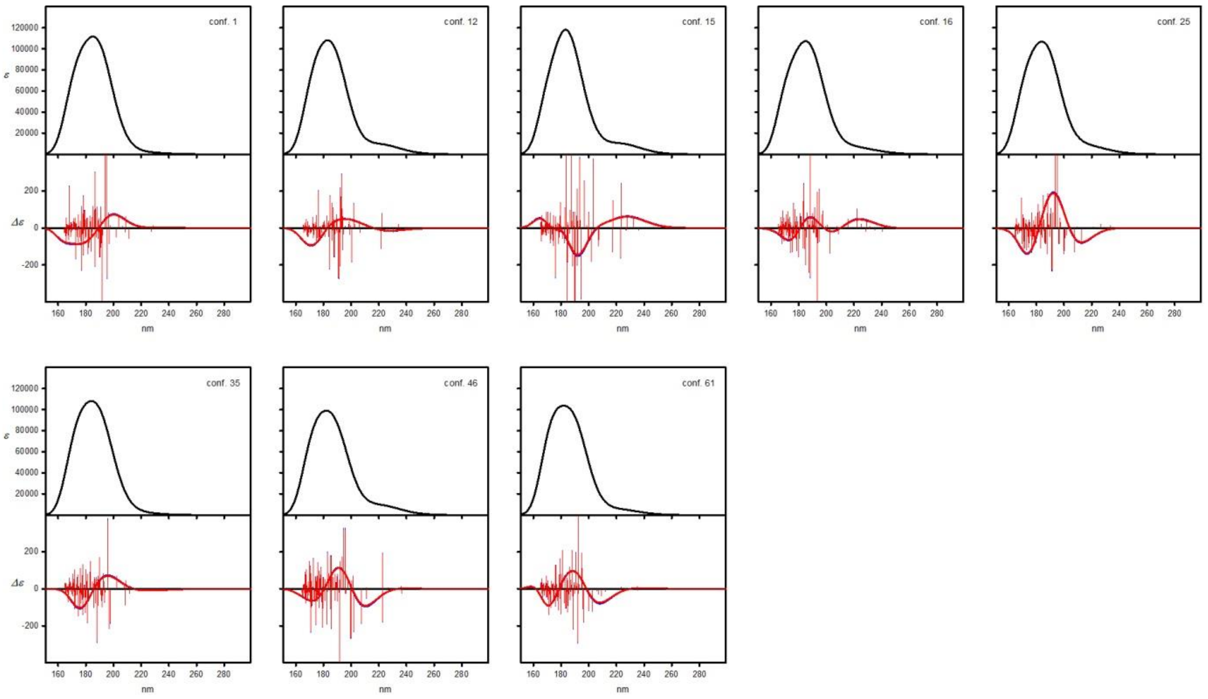

Figure SI_70. UV and ECD spectra of the low-energy conformers of compound **21** calculated at TD-M06-2X/6-311++G(d,p) level for structures optimized at B3LYP-GD3BJ/6-311G(d,p) level. Wavelengths were not corrected.

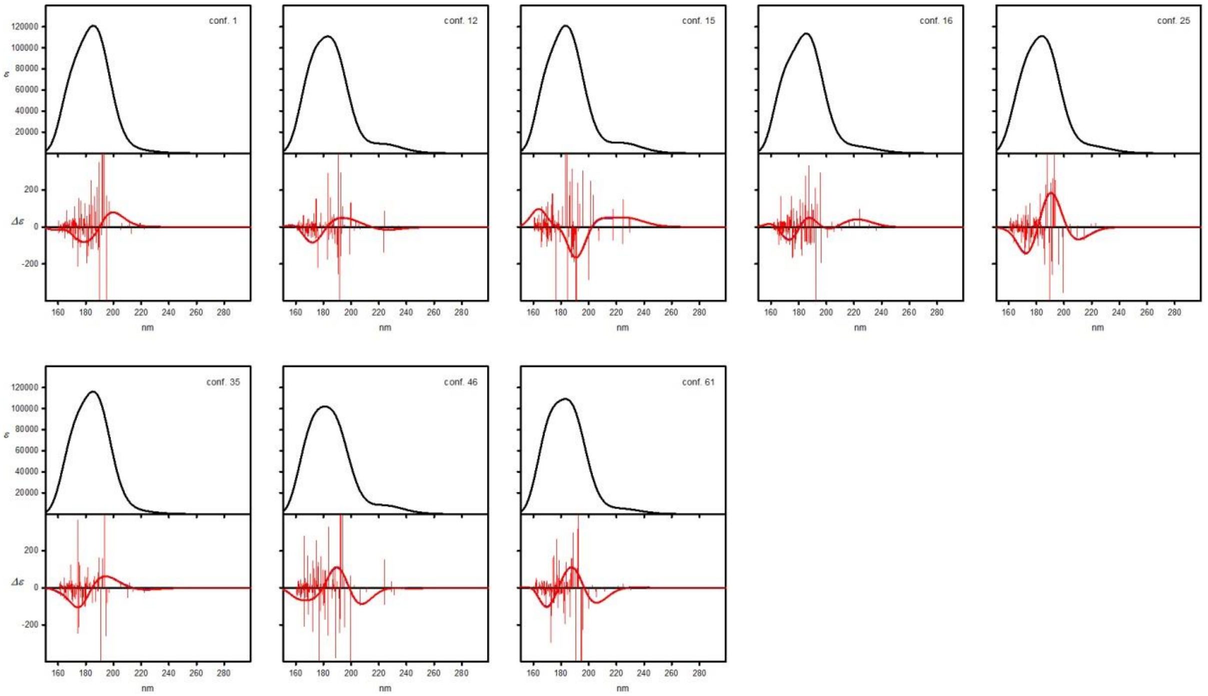

Figure SI_71. UV and ECD spectra of the low-energy conformers of compound **21** calculated at TD-CAM-B3LYP/6-311++G(d,p) level for structures optimized at M06-2X/6-311G(d,p) level. Wavelengths were not corrected.

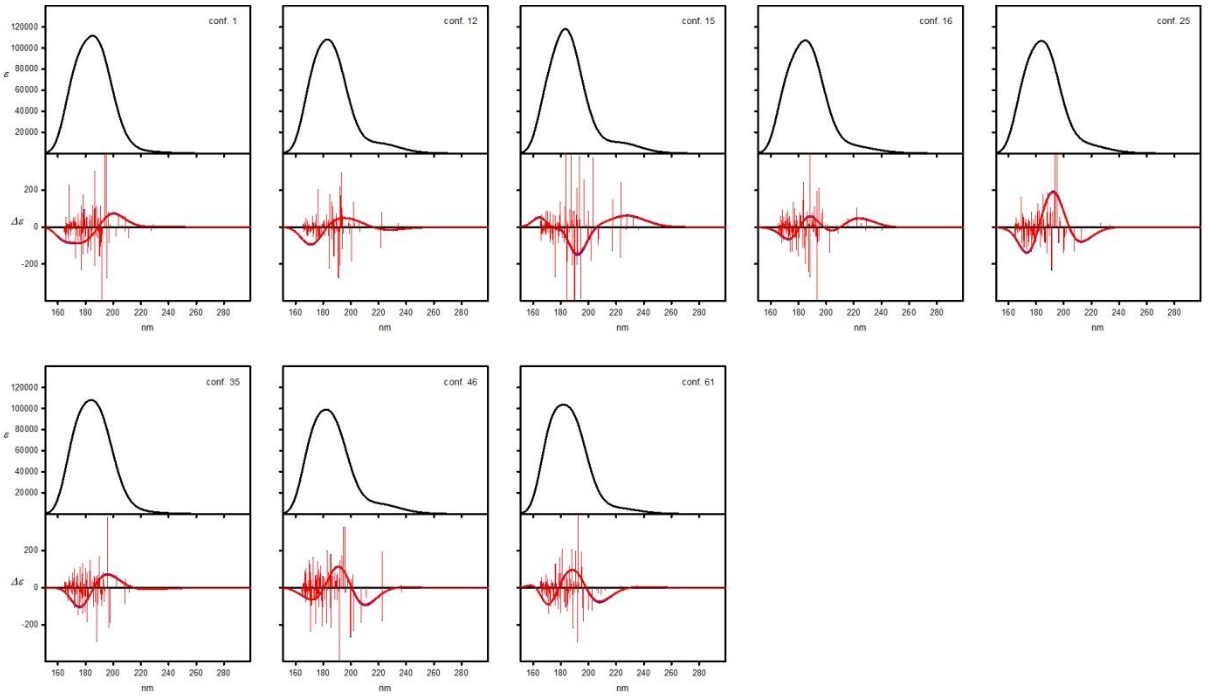

Figure SI_72. UV and ECD spectra of the low-energy conformers of compound **21** calculated at TD-M06-2X/6-311++G(d,p) level for structures optimized at M06-2X/6-311G(d,p) level. Wavelengths were not corrected.

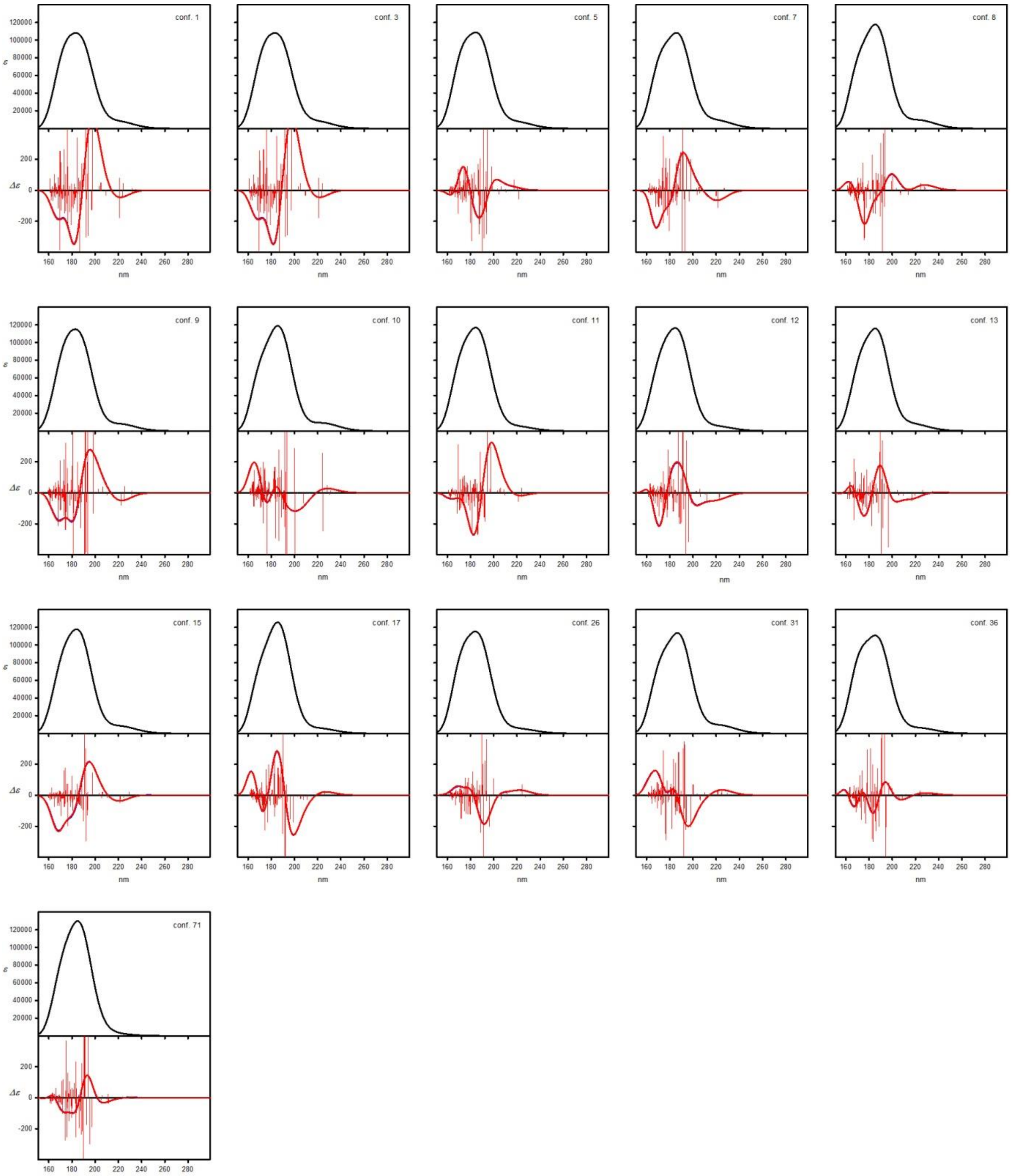

Figure SI_73. UV and ECD spectra of the low-energy conformers of compound **22** calculated at TD-CAM-B3LYP/6-311++G(d,p) level for structures optimized at B3LYP/6-311G(d,p) level. Wavelengths were not corrected.

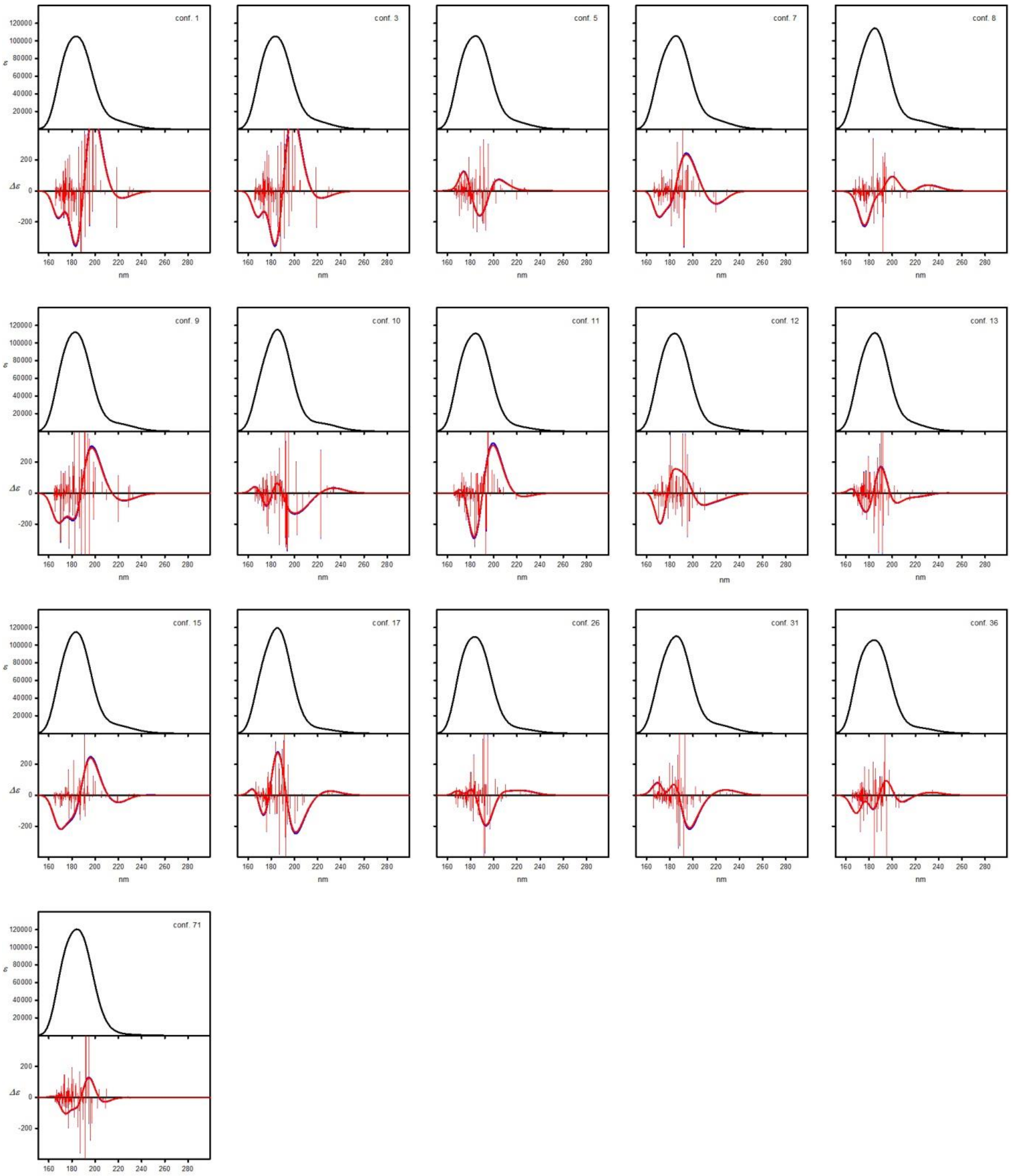

Figure SI_74. UV and ECD spectra of the low-energy conformers of compound **22** calculated at TD-M06-2X/6-311++G(d,p) level for structures optimized at B3LYP/6-311G(d,p) level. Wavelengths were not corrected.

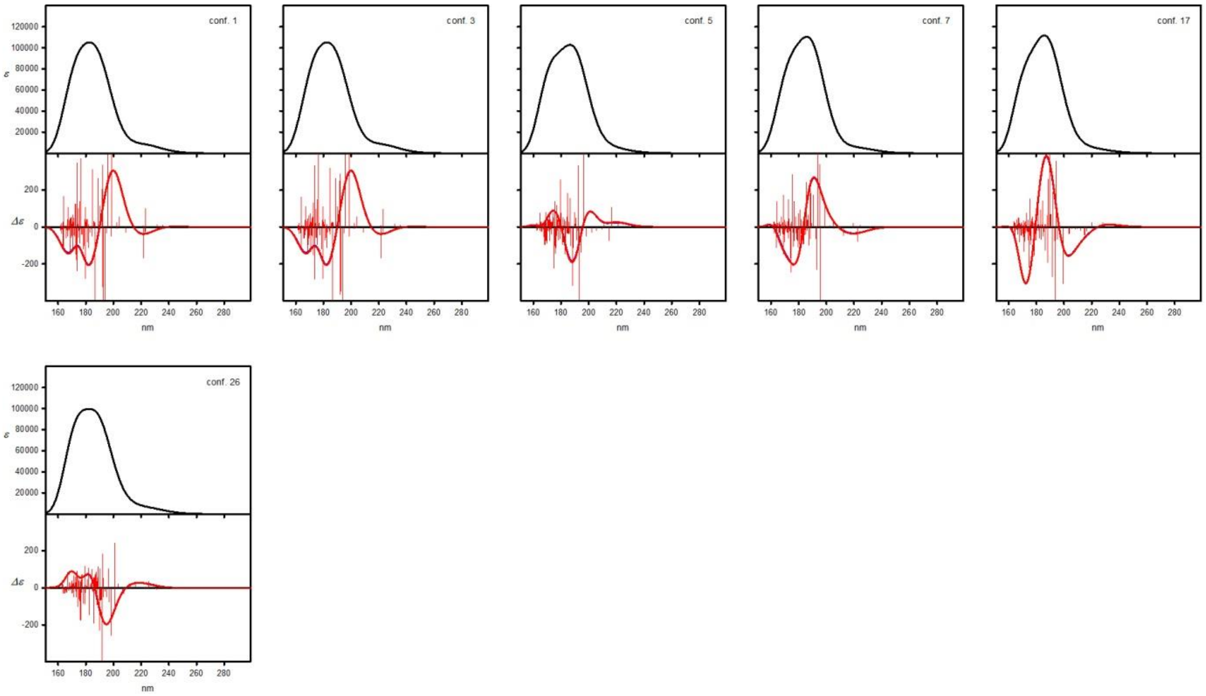

Figure SI_75. UV and ECD spectra of the low-energy conformers of compound **22** calculated at TD-CAM-B3LYP/6-311++G(d,p) level for structures optimized at B3LYP-GD3BJ/6-311G(d,p) level. Wavelengths were not corrected.

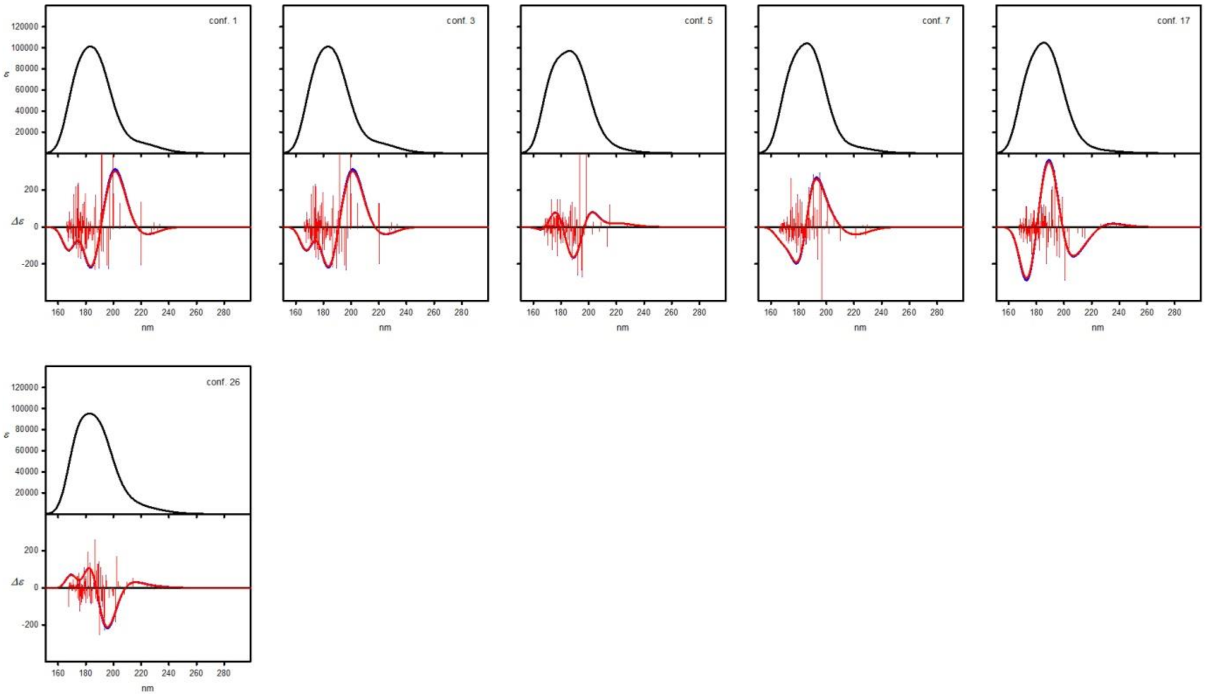

Figure SI_76. UV and ECD spectra of the low-energy conformers of compound **22** calculated at TD-M06-2X/6-311++G(d,p) level for structures optimized at B3LYP-GD3BJ/6-311G(d,p) level. Wavelengths were not corrected.

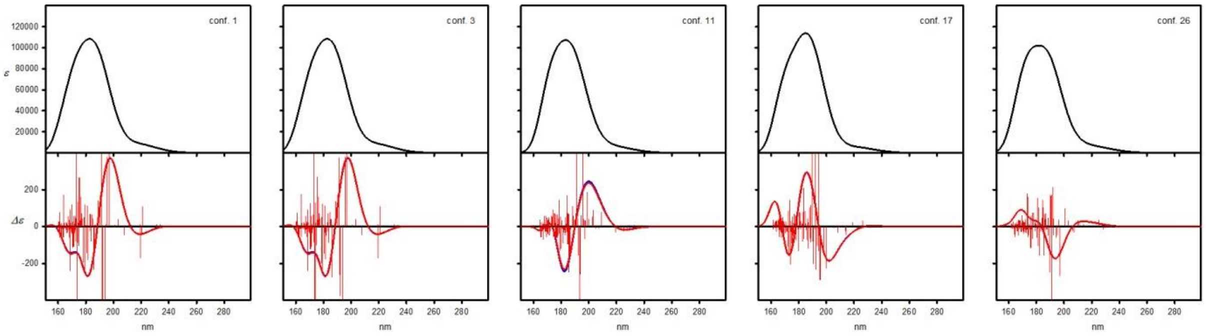

Figure SI_77. UV and ECD spectra of the low-energy conformers of compound **22** calculated at TD-CAM-B3LYP/6-311++G(d,p) level for structures optimized at M06-2X/6-311G(d,p) level. Wavelengths were not corrected.

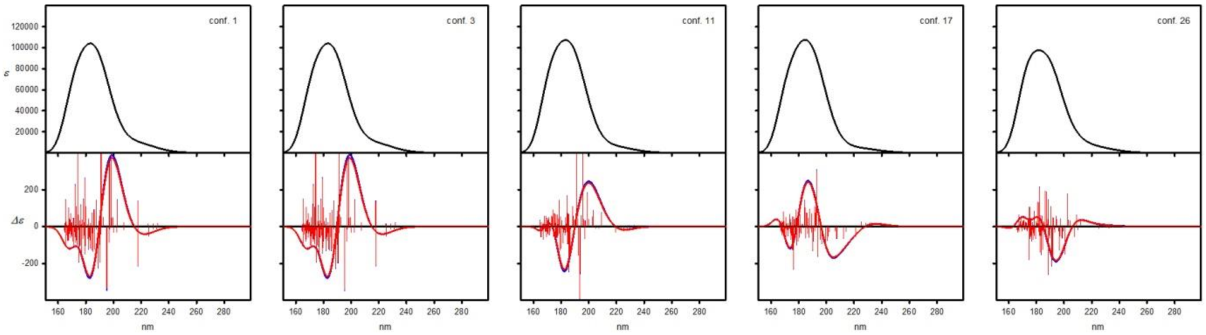

Figure SI_78. UV and ECD spectra of the low-energy conformers of compound **22** calculated at TD-M06-2X/6-311++G(d,p) level for structures optimized at M06-2X/6-311G(d,p) level. Wavelengths were not corrected.

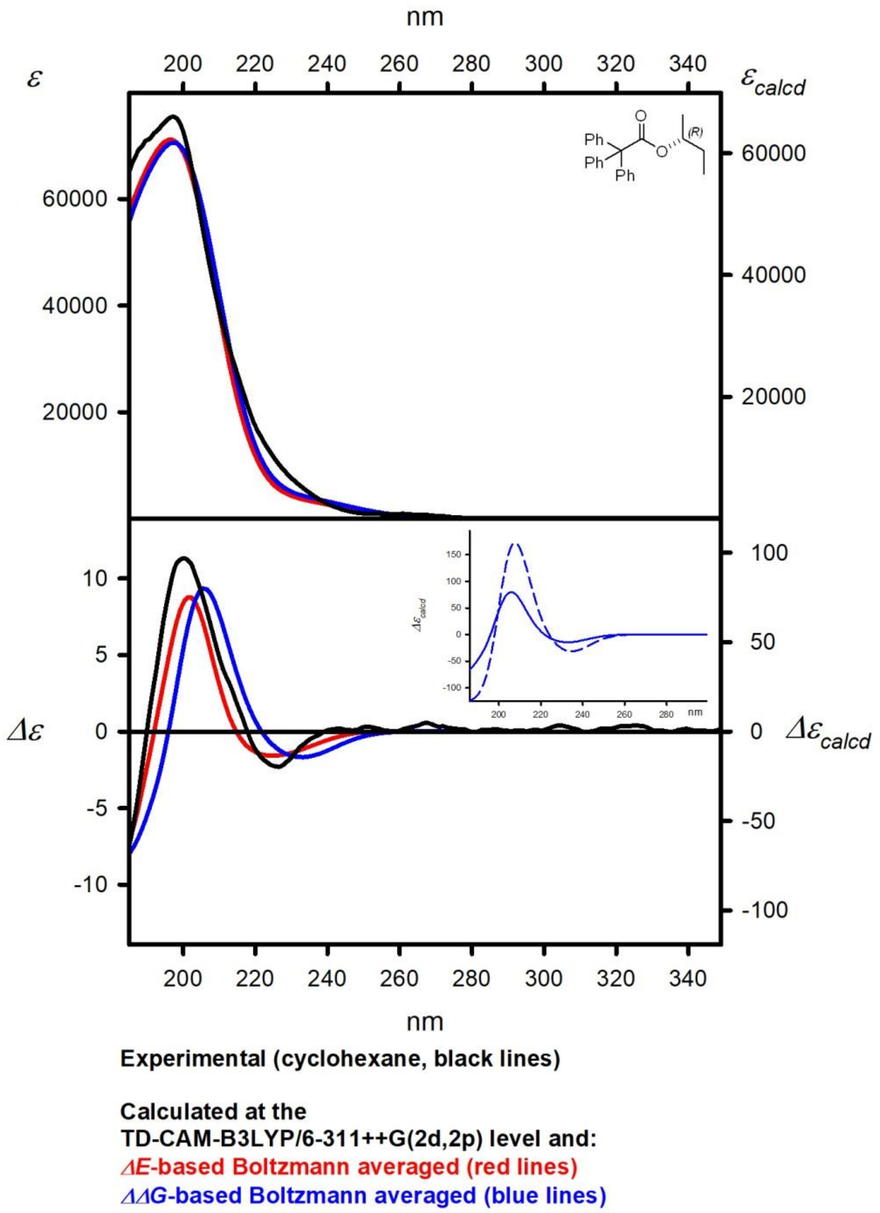

Figure SI_79. UV (upper panel) and ECD (lower panel) spectra of **1**, experimental, measured in cyclohexane (solid black lines) and calculated at the TD-CAM-B3LYP/6-311++G(2d,2p) level for structures optimized at the B3LYP/6-311++G(d,p) level, ΔE-based Boltzmann (red lines) and ΔΔG-based Boltzmann averaged (blue solid lines). Insert shows the comparison between Boltzmann averaged ECD spectrum and that calculated for ΔΔG-based on the lowest energy conformer of a given compound (dashed blue line). All calculated spectra were wavelength corrected to match experimental UV maxima.

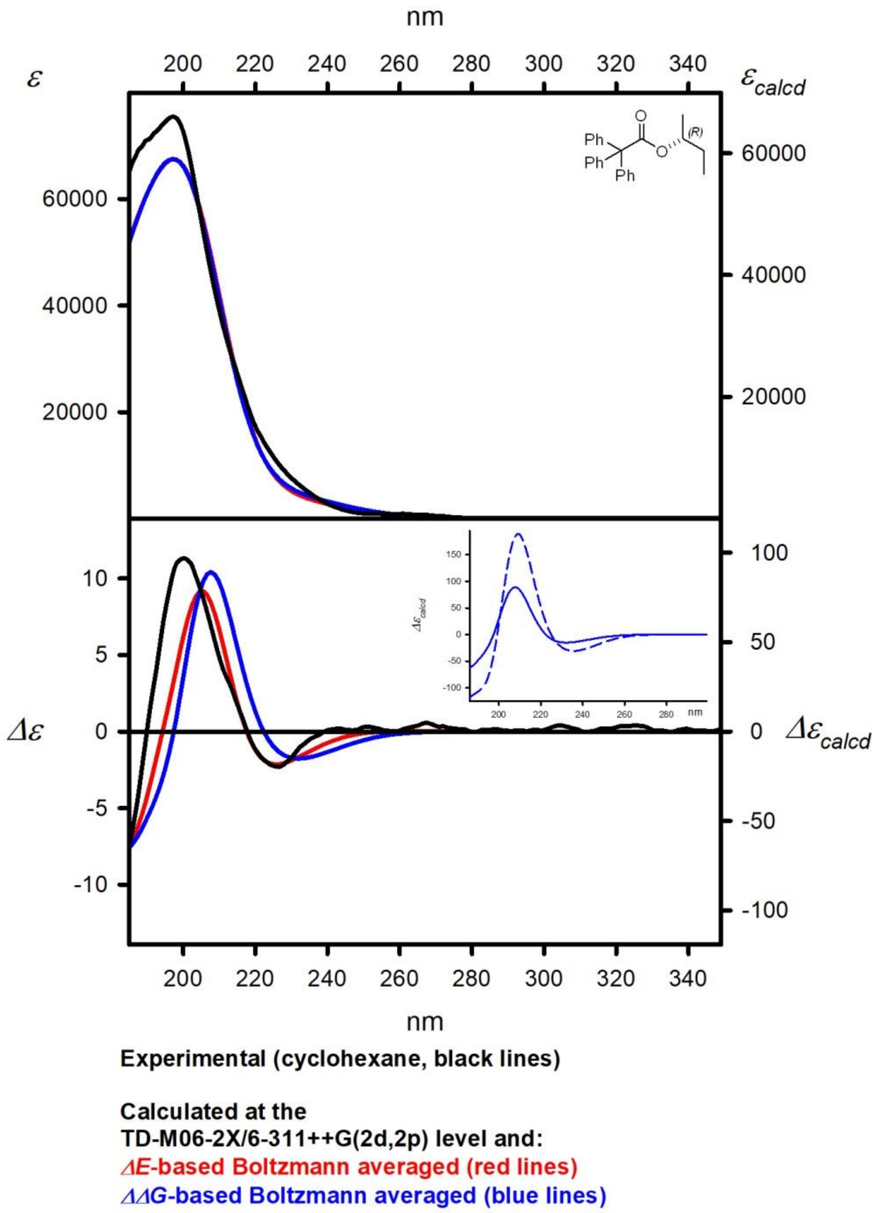

Figure SI_80. UV (upper panel) and ECD (lower panel) spectra of **1**, experimental, measured in cyclohexane (solid black lines) and calculated at the TD-M06-2X/6-311++G(2d,2p) level for structures optimized at the B3LYP/6-311++G(d,p) level, ΔE-based Boltzmann (red lines) and ΔΔG-based Boltzmann averaged (blue solid lines). Insert shows the comparison between Boltzmann averaged ECD spectrum and that calculated for ΔΔG-based on the lowest energy conformer of a given compound (dashed blue line). All calculated spectra were wavelength corrected to match experimental UV maxima.

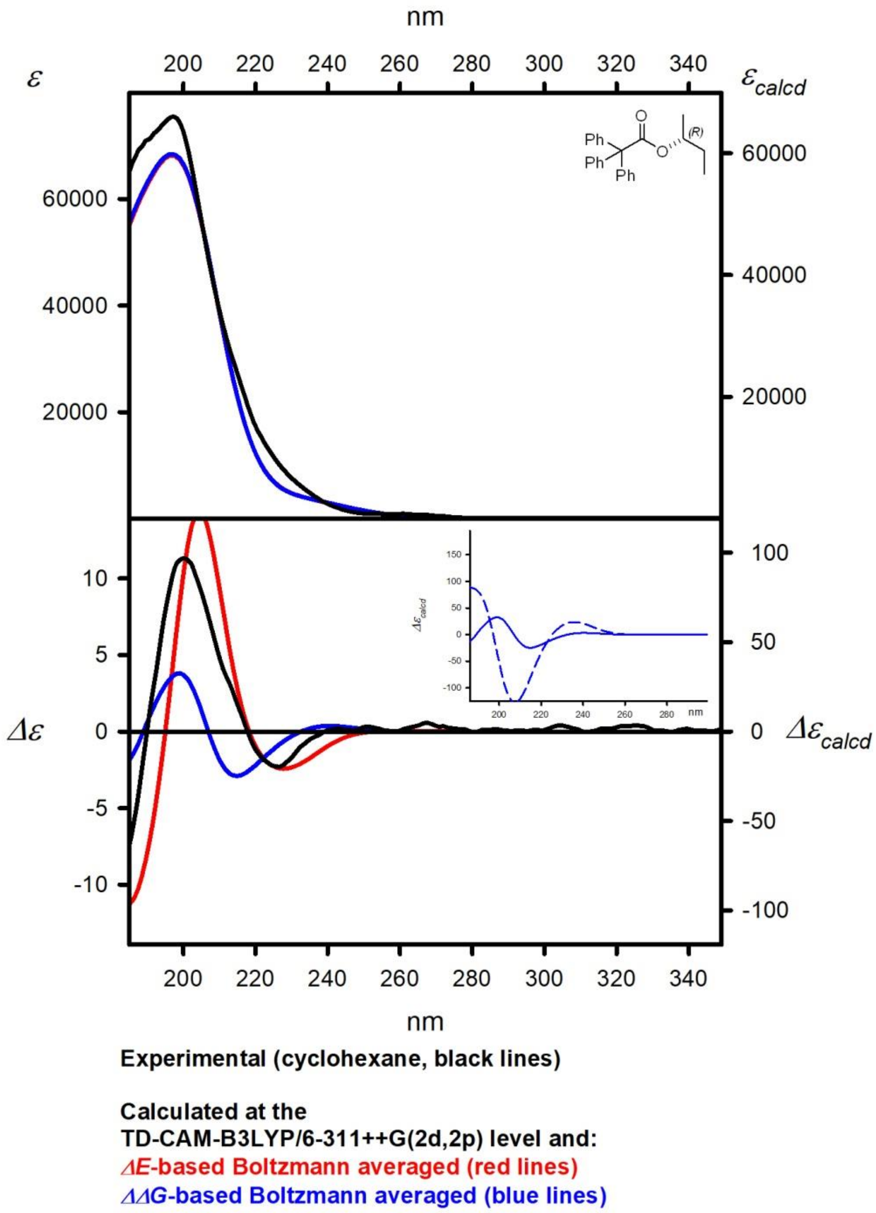

Figure SI_81. UV (upper panel) and ECD (lower panel) spectra of **1**, experimental, measured in cyclohexane (solid black lines) and calculated at the TD-CAM-B3LYP/6-311++G(2d,2p) level for structures optimized at the M06-2X/6-311++G(d,p) level, ΔE-based Boltzmann (red lines) and ΔΔG-based Boltzmann averaged (blue solid lines). Insert shows the comparison between Boltzmann averaged ECD spectrum and that calculated for ΔΔG-based on the lowest energy conformer of a given compound (dashed blue line). All calculated spectra were wavelength corrected to match experimental UV maxima.

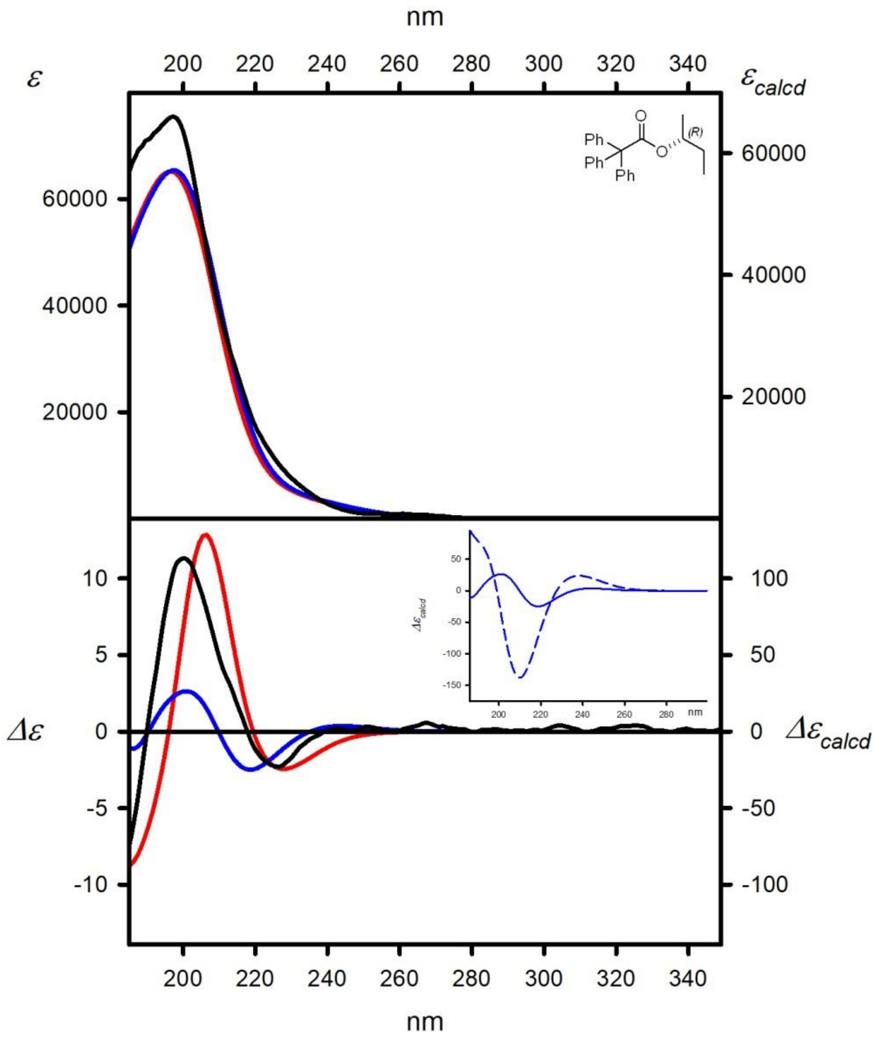

Experimental (cyclohexane, black lines)

Calculated at the
TD-M06-2X/6-311++G(2d,2p) level and:
***ΔE*-based Boltzmann averaged (red lines)**
***ΔΔG*-based Boltzmann averaged (blue lines)**

Figure SI_82. UV (upper panel) and ECD (lower panel) spectra of **1**, experimental, measured in cyclohexane (solid black lines) and calculated at the TD-M06-2X/6-311++G(2d,2p) level for structures optimized at the M06-2X/6-311++G(d,p) level, ΔE-based Boltzmann (red lines) and ΔΔG-based Boltzmann averaged (blue solid lines). Insert shows the comparison between Boltzmann averaged ECD spectrum and that calculated for ΔΔG-based on the lowest energy conformer of a given compound (dashed blue line). All calculated spectra were wavelength corrected to match experimental UV maxima.

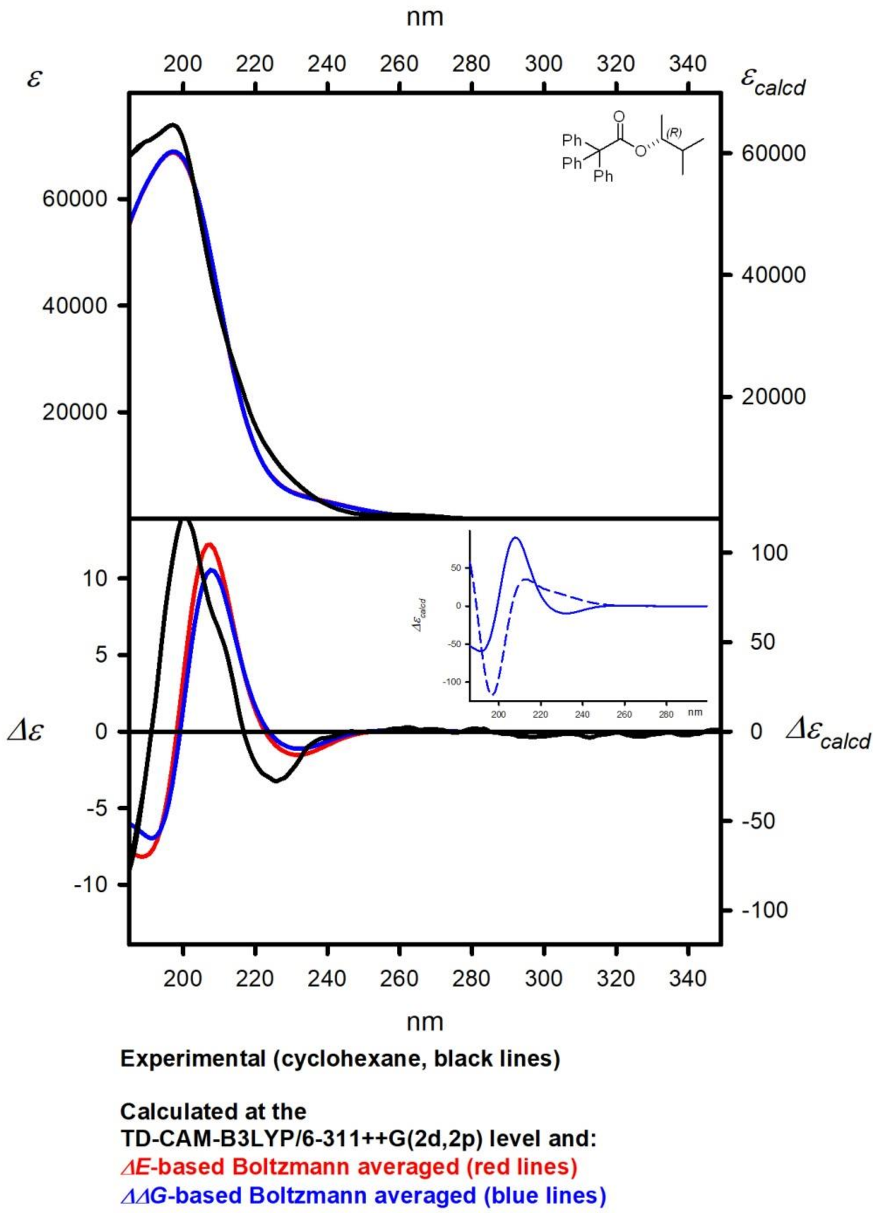

Figure SI_83. UV (upper panel) and ECD (lower panel) spectra of **4**, experimental, measured in cyclohexane (solid black lines) and calculated at the TD-CAM-B3LYP/6-311++G(2d,2p) level for structures optimized at the B3LYP/6-311++G(d,p) level, ΔE-based Boltzmann (red lines) and ΔΔG-based Boltzmann averaged (blue solid lines). Insert shows the comparison between Boltzmann averaged ECD spectrum and that calculated for ΔΔG-based on the lowest energy conformer of a given compound (dashed blue line). All calculated spectra were wavelength corrected to match experimental UV maxima.

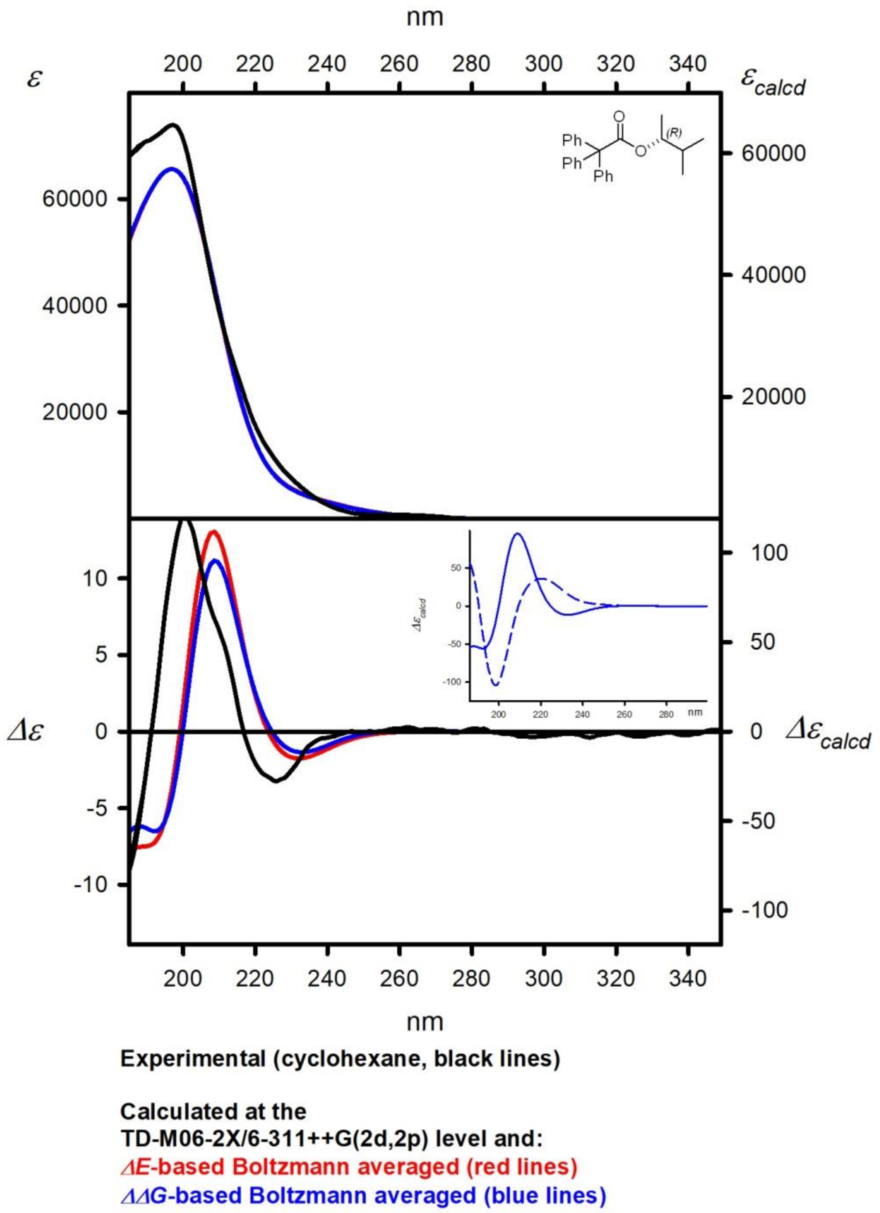

Figure SI_84. UV (upper panel) and ECD (lower panel) spectra of **4**, experimental, measured in cyclohexane (solid black lines) and calculated at the TD-M06-2X/6-311++G(2d,2p) level for structures optimized at the B3LYP/6-311++G(d,p) level, ΔE-based Boltzmann (red lines) and ΔΔG-based Boltzmann averaged (blue solid lines). Insert shows the comparison between Boltzmann averaged ECD spectrum and that calculated for ΔΔG-based on the lowest energy conformer of a given compound (dashed blue line). All calculated spectra were wavelength corrected to match experimental UV maxima.

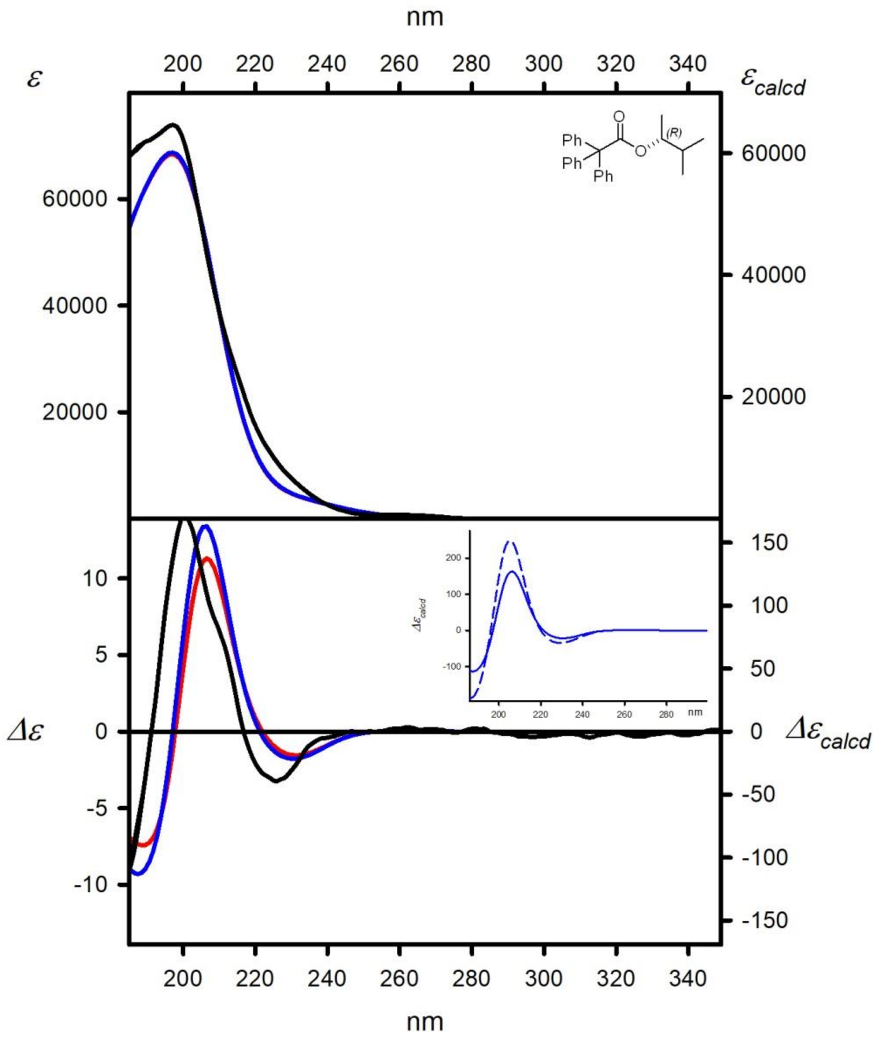

Experimental (cyclohexane, black lines)

Calculated at the
TD-CAM-B3LYP/6-311++G(2d,2p) level and:
***ΔE*-based Boltzmann averaged (red lines)**
***ΔΔG*-based Boltzmann averaged (blue lines)**

Figure SI_85. UV (upper panel) and ECD (lower panel) spectra of **4**, experimental, measured in cyclohexane (solid black lines) and calculated at the TD-CAM-B3LYP/6-311++G(2d,2p) level for structures optimized at the M06-2X/6-311++G(d,p) level, ΔE-based Boltzmann (red lines) and ΔΔG-based Boltzmann averaged (blue solid lines). Insert shows the comparison between Boltzmann averaged ECD spectrum and that calculated for ΔΔG-based on the lowest energy conformer of a given compound (dashed blue line). All calculated spectra were wavelength corrected to match experimental UV maxima.

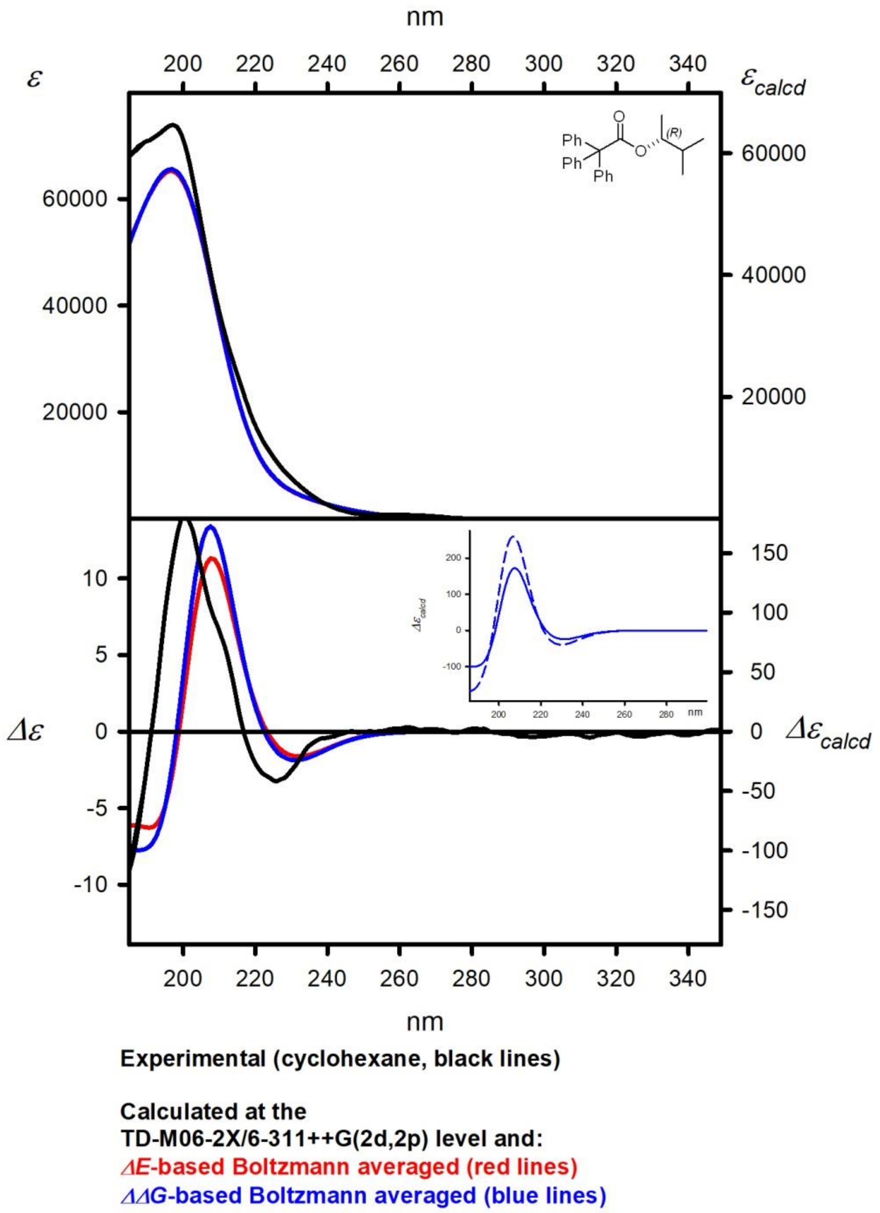

Figure SI_86. UV (upper panel) and ECD (lower panel) spectra of **4**, experimental, measured in cyclohexane (solid black lines) and calculated at the TD-M06-2X/6-311++G(2d,2p) level for structures optimized at the M06-2X/6-311++G(d,p) level, ΔE-based Boltzmann (red lines) and ΔΔG-based Boltzmann averaged (blue solid lines). Insert shows the comparison between Boltzmann averaged ECD spectrum and that calculated for ΔΔG-based on the lowest energy conformer of a given compound (dashed blue line). All calculated spectra were wavelength corrected to match experimental UV maxima.

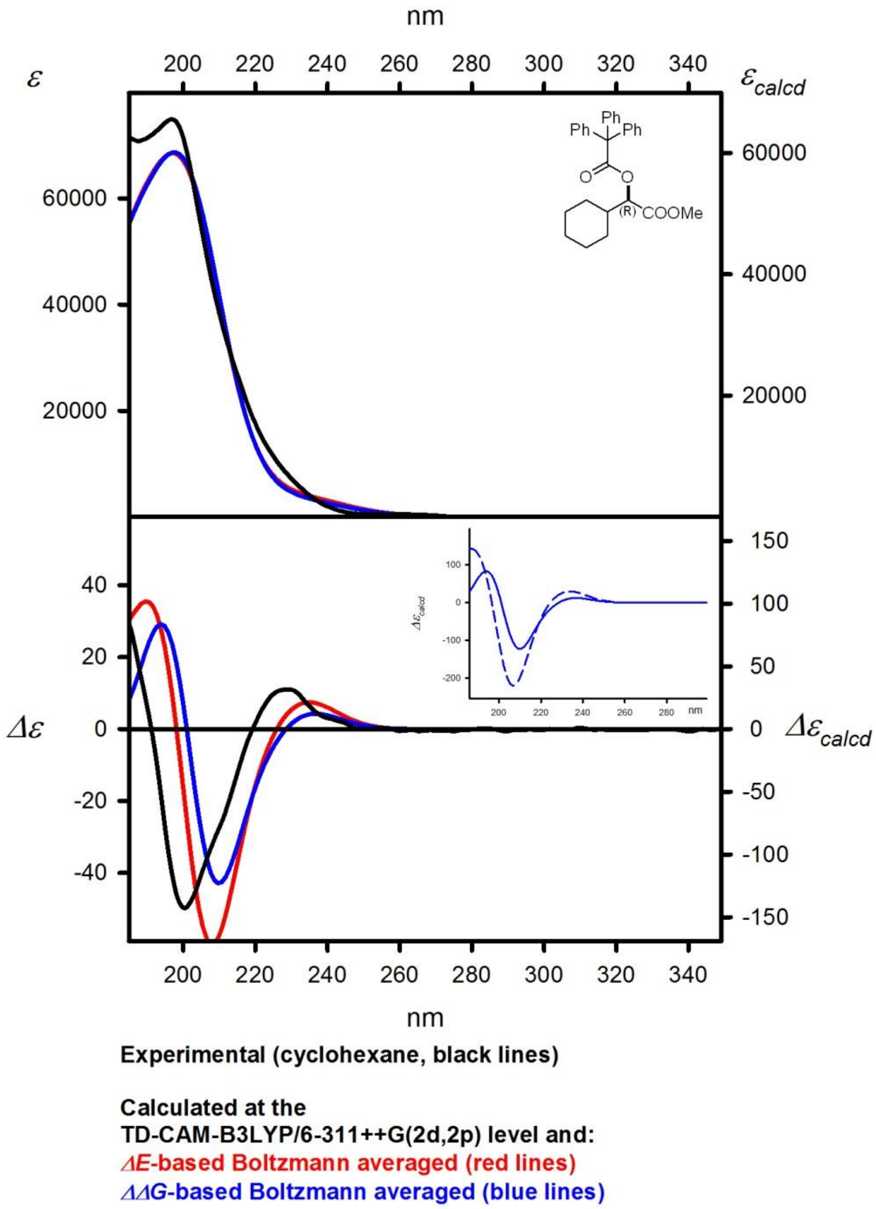

Figure SI_87. UV (upper panel) and ECD (lower panel) spectra of **6**, experimental, measured in cyclohexane (solid black lines) and calculated at the TD-CAM-B3LYP/6-311++G(2d,2p) level for structures optimized at the B3LYP/6-311++G(d,p) level, ΔE-based Boltzmann (red lines) and ΔΔG-based Boltzmann averaged (blue solid lines). Insert shows the comparison between Boltzmann averaged ECD spectrum and that calculated for ΔΔG-based on the lowest energy conformer of a given compound (dashed blue line). All calculated spectra were wavelength corrected to match experimental UV maxima.

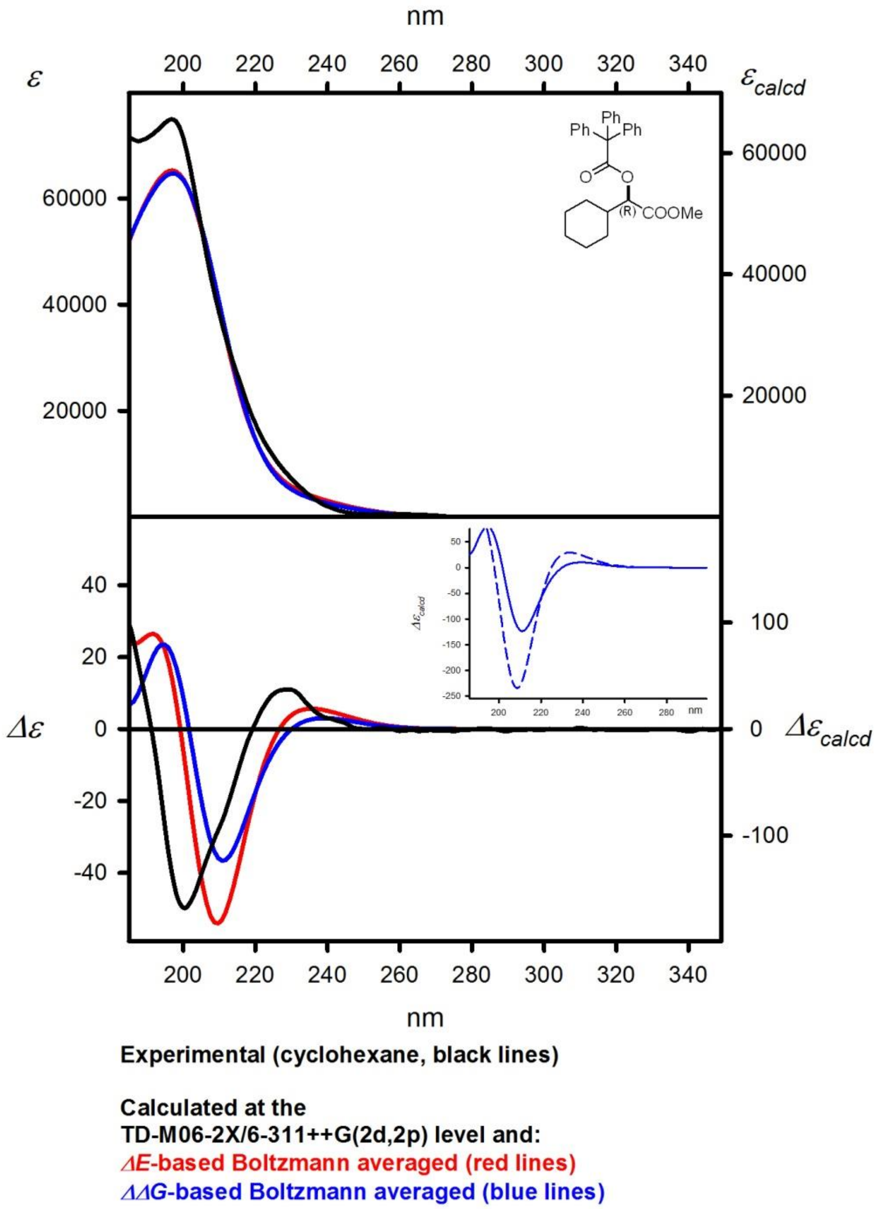

Figure SI_88. UV (upper panel) and ECD (lower panel) spectra of **6**, experimental, measured in cyclohexane (solid black lines) and calculated at the TD-M06-2X/6-311++G(2d,2p) level for structures optimized at the B3LYP/6-311++G(d,p) level, ΔE-based Boltzmann (red lines) and ΔΔG-based Boltzmann averaged (blue solid lines). Insert shows the comparison between Boltzmann averaged ECD spectrum and that calculated for ΔΔG-based on the lowest energy conformer of a given compound (dashed blue line). All calculated spectra were wavelength corrected to match experimental UV maxima.

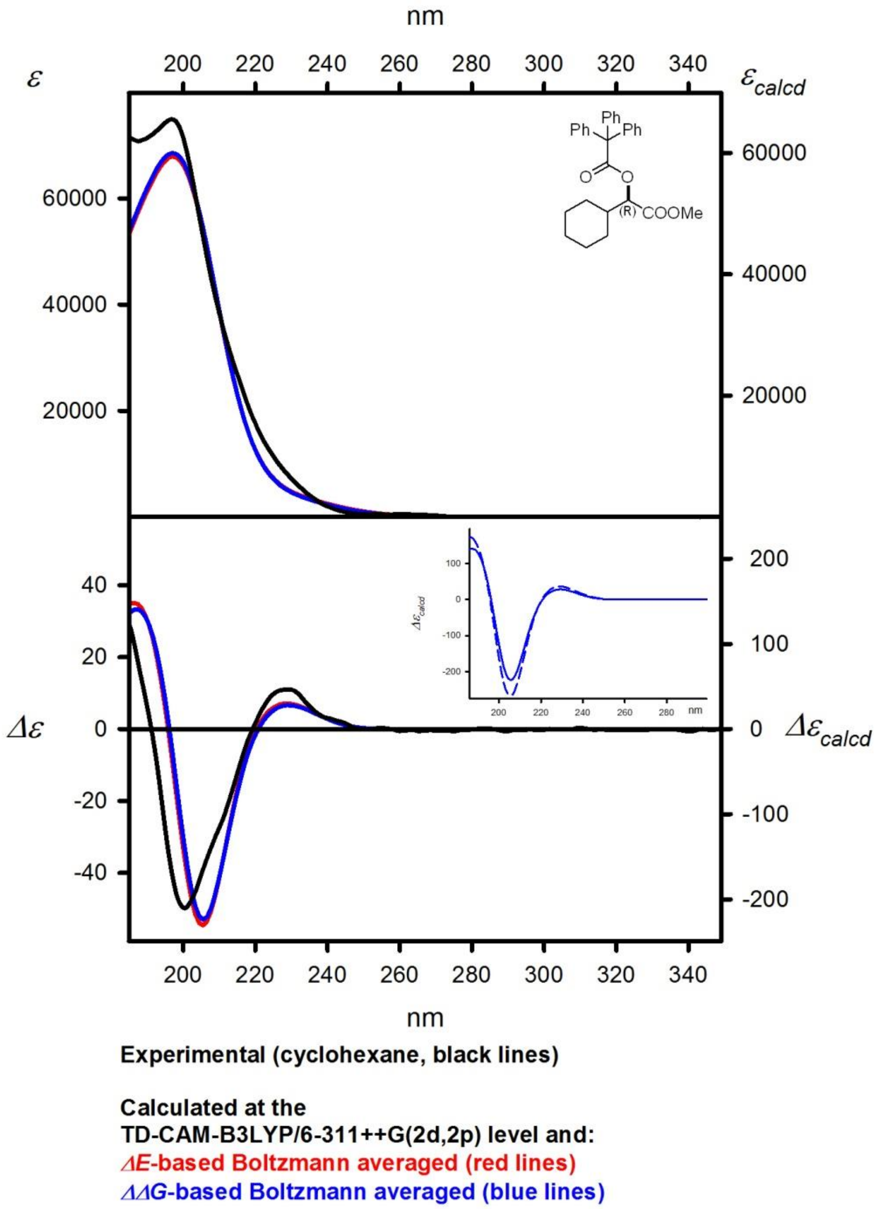

Figure SI_89. UV (upper panel) and ECD (lower panel) spectra of **6**, experimental, measured in cyclohexane (solid black lines) and calculated at the TD-CAM-B3LYP/6-311++G(2d,2p) level for structures optimized at the M06-2X/6-311++G(d,p) level, ΔE-based Boltzmann (red lines) and ΔΔG-based Boltzmann averaged (blue solid lines). Insert shows the comparison between Boltzmann averaged ECD spectrum and that calculated for ΔΔG-based on the lowest energy conformer of a given compound (dashed blue line). All calculated spectra were wavelength corrected to match experimental UV maxima.

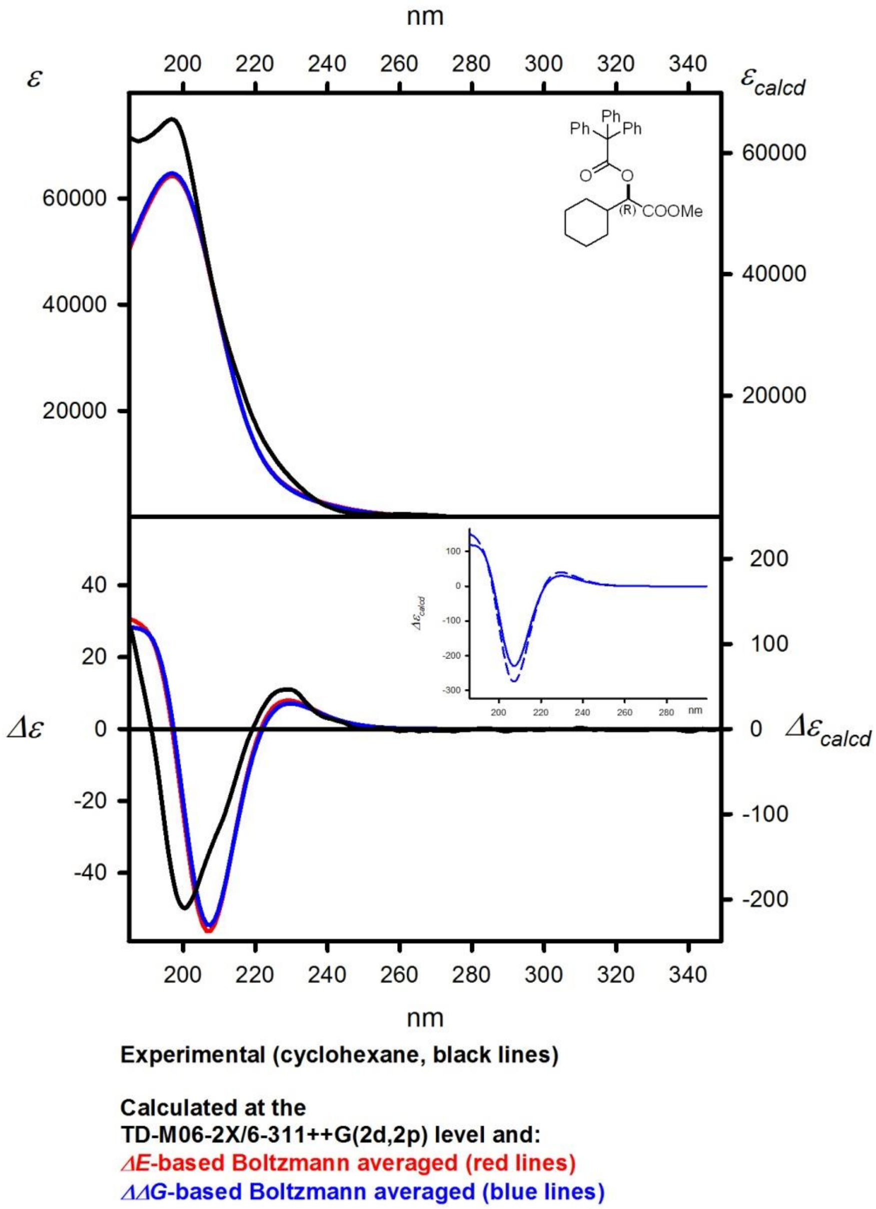

Figure SI_90. UV (upper panel) and ECD (lower panel) spectra of **6**, experimental, measured in cyclohexane (solid black lines) and calculated at the TD-M06-2X/6-311++G(2d,2p) level for structures optimized at the M06-2X/6-311++G(d,p) level, ΔE-based Boltzmann (red lines) and ΔΔG-based Boltzmann averaged (blue solid lines). Insert shows the comparison between Boltzmann averaged ECD spectrum and that calculated for ΔΔG-based on the lowest energy conformer of a given compound (dashed blue line). All calculated spectra were wavelength corrected to match experimental UV maxima.

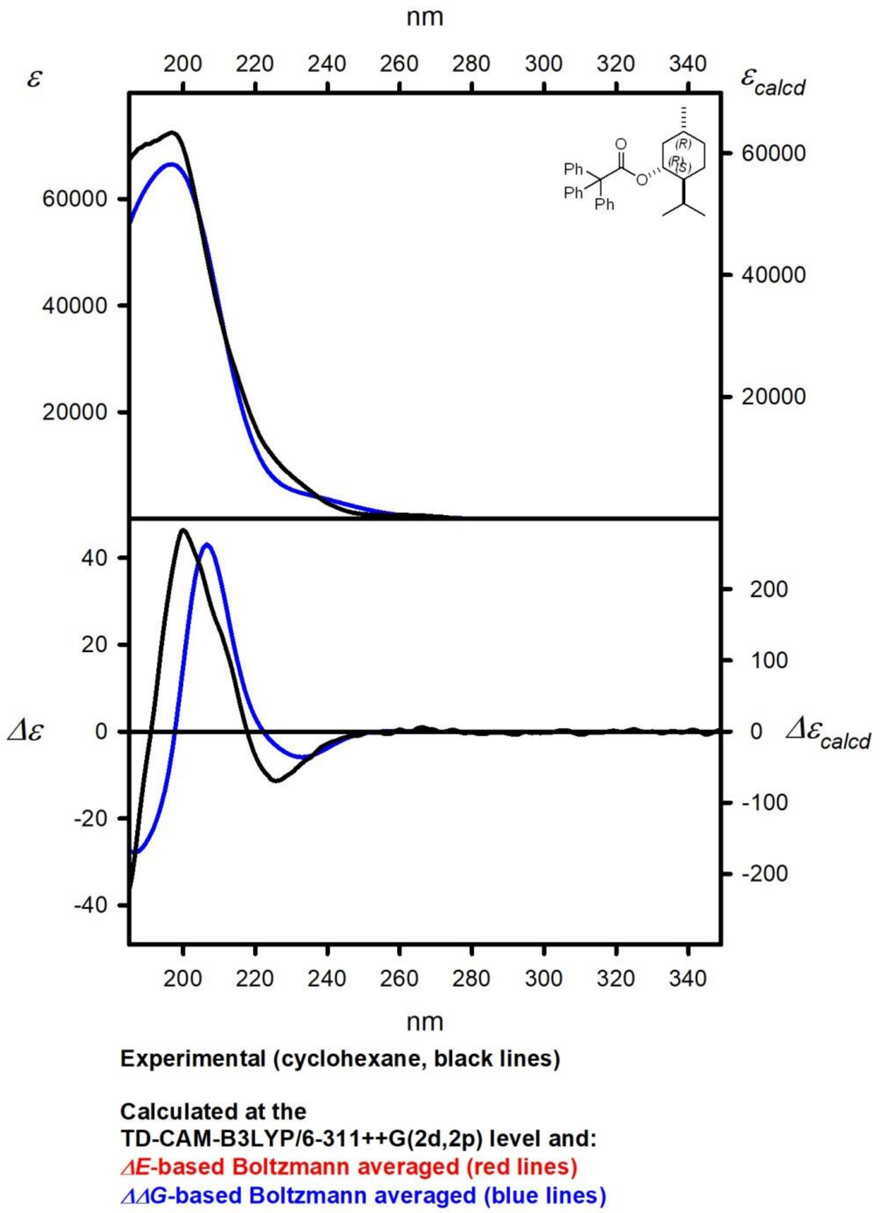

Figure SI_91. UV (upper panel) and ECD (lower panel) spectra of **10**, experimental, measured in cyclohexane (solid black lines) and calculated at the TD-CAM-B3LYP/6-311++G(2d,2p) level for structures optimized at the B3LYP/6-311++G(d,p) level, ΔE-based Boltzmann (red lines) and ΔΔG-based Boltzmann averaged (blue solid lines). Insert shows the comparison between Boltzmann averaged ECD spectrum and that calculated for ΔΔG-based on the lowest energy conformer of a given compound (dashed blue line). All calculated spectra were wavelength corrected to match experimental UV maxima.

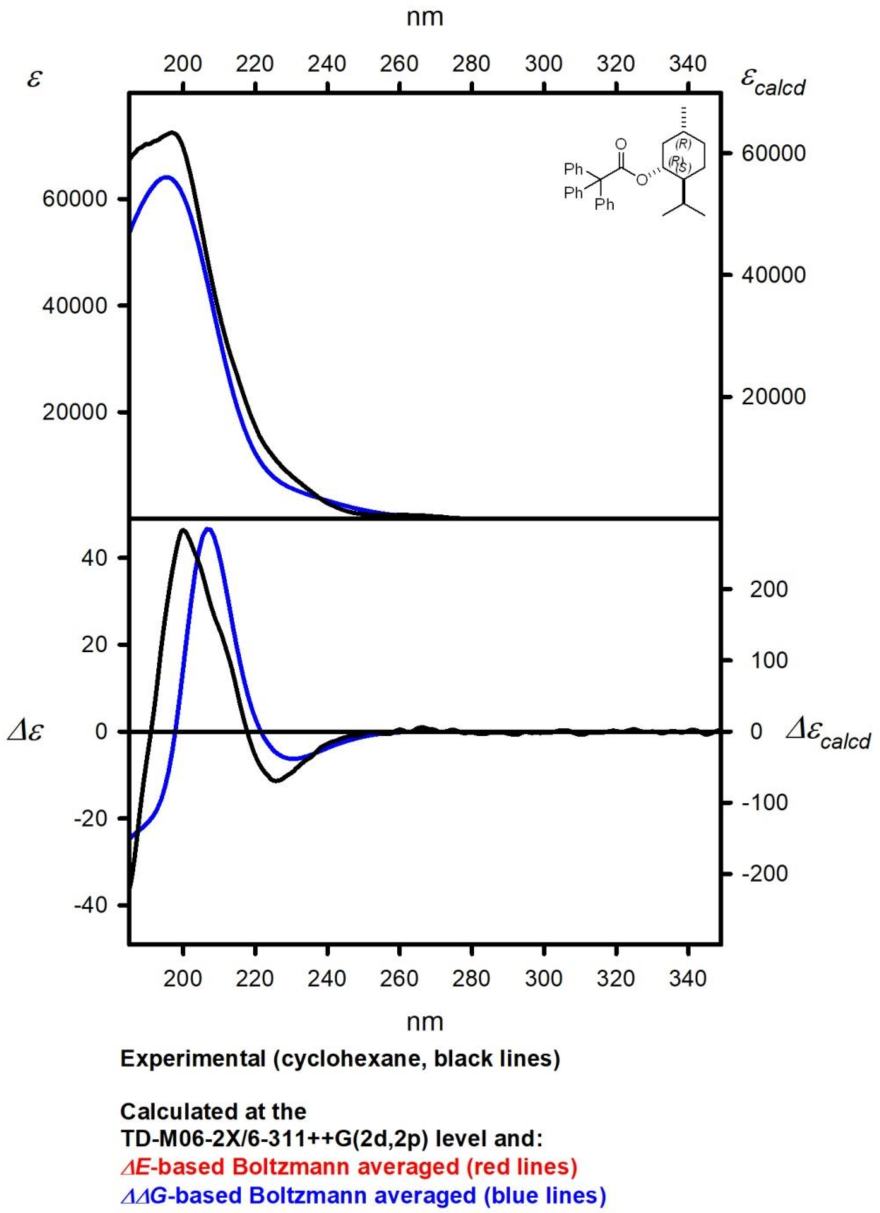

Figure SI_92. UV (upper panel) and ECD (lower panel) spectra of **10**, experimental, measured in cyclohexane (solid black lines) and calculated at the TD-M06-2X/6-311++G(2d,2p) level for structures optimized at the B3LYP/6-311++G(d,p) level, ΔE-based Boltzmann (red lines) and ΔΔG-based Boltzmann averaged (blue solid lines). Insert shows the comparison between Boltzmann averaged ECD spectrum and that calculated for ΔΔG-based on the lowest energy conformer of a given compound (dashed blue line). All calculated spectra were wavelength corrected to match experimental UV maxima.

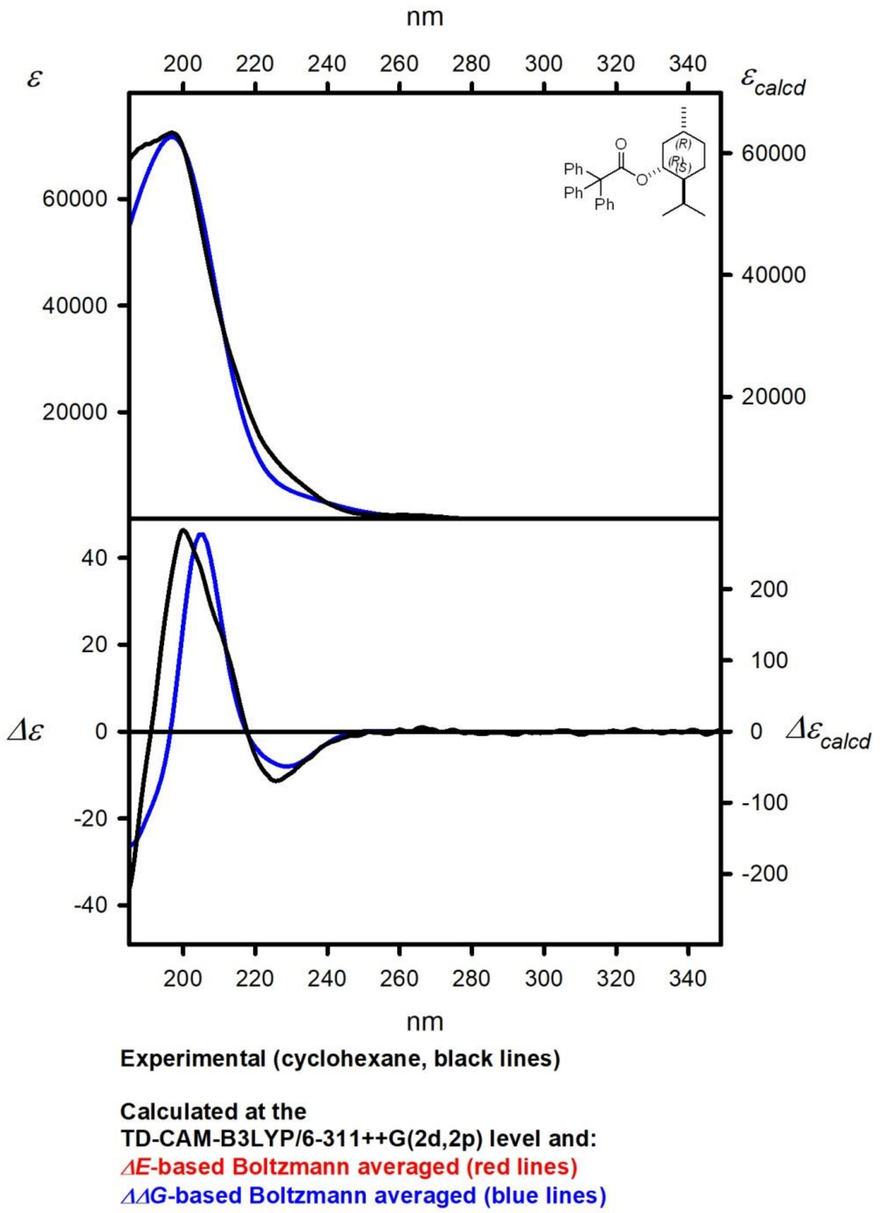

Figure SI_93. UV (upper panel) and ECD (lower panel) spectra of **10**, experimental, measured in cyclohexane (solid black lines) and calculated at the TD-CAM-B3LYP/6-311++G(2d,2p) level for structures optimized at the M06-2X/6-311++G(d,p) level, ΔE-based Boltzmann (red lines) and ΔΔG-based Boltzmann averaged (blue solid lines). Insert shows the comparison between Boltzmann averaged ECD spectrum and that calculated for ΔΔG-based on the lowest energy conformer of a given compound (dashed blue line). All calculated spectra were wavelength corrected to match experimental UV maxima.

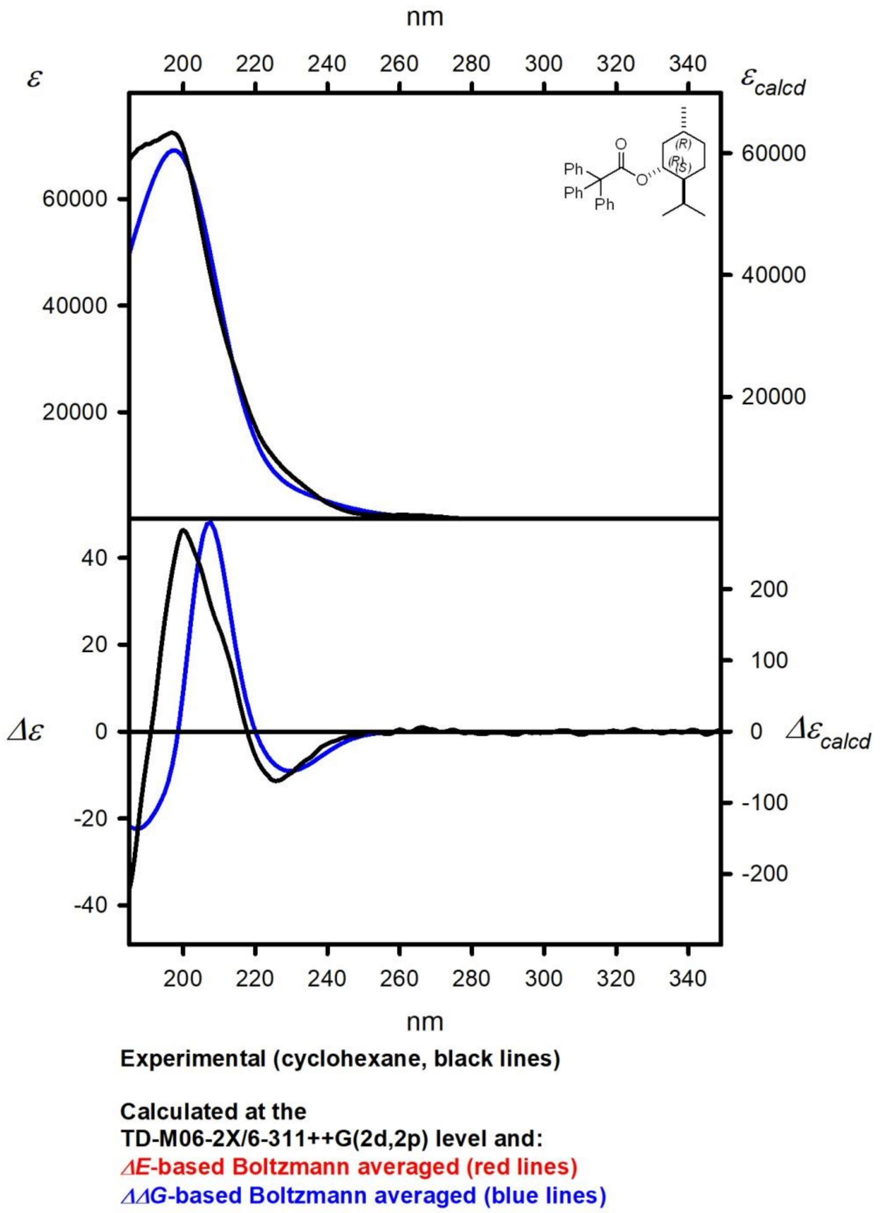

Figure SI_94. UV (upper panel) and ECD (lower panel) spectra of **10**, experimental, measured in cyclohexane (solid black lines) and calculated at the TD-M06-2X/6-311++G(2d,2p) level for structures optimized at the M06-2X/6-311++G(d,p) level, ΔE-based Boltzmann (red lines) and ΔΔG-based Boltzmann averaged (blue solid lines). Insert shows the comparison between Boltzmann averaged ECD spectrum and that calculated for ΔΔG-based on the lowest energy conformer of a given compound (dashed blue line). All calculated spectra were wavelength corrected to match experimental UV maxima.

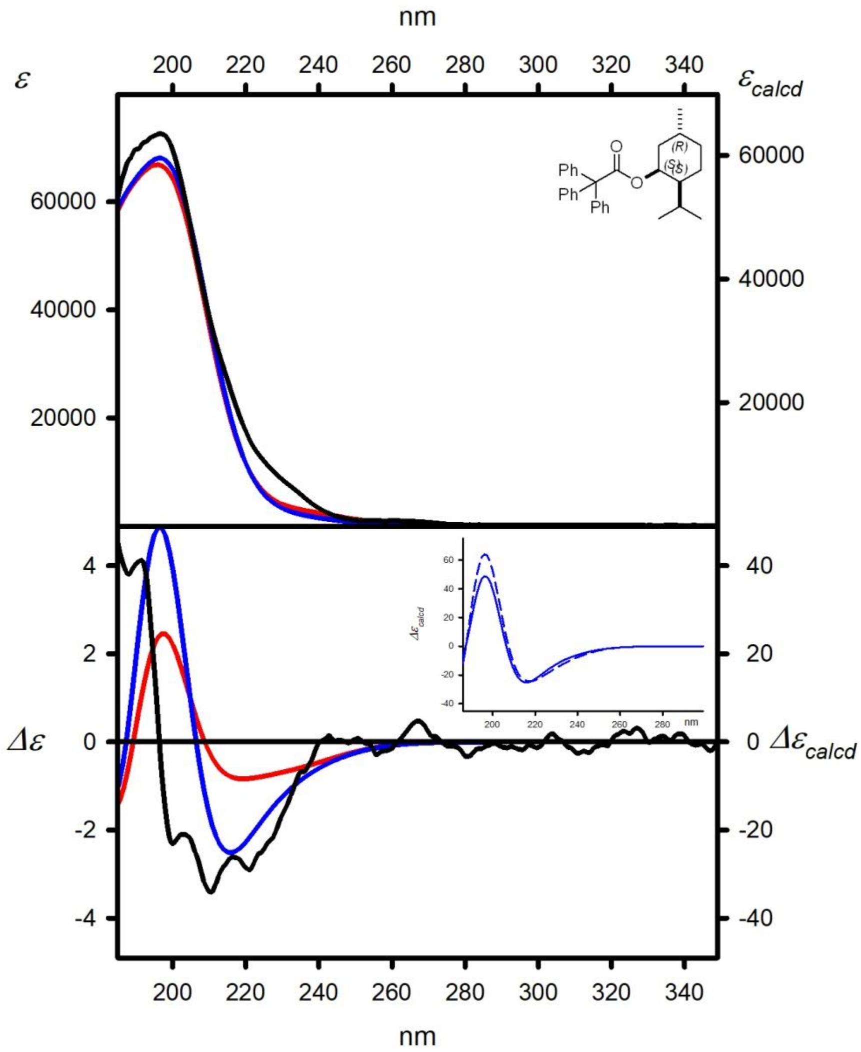

Experimental (cyclohexane, black lines)

Calculated at the
TD-CAM-B3LYP/6-311++G(2d,2p) level and:
***ΔE*-based Boltzmann averaged (red lines)**
***ΔΔG*-based Boltzmann averaged (blue lines)**

Figure SI_95. UV (upper panel) and ECD (lower panel) spectra of **11**, experimental, measured in cyclohexane (solid black lines) and calculated at the TD-CAM-B3LYP/6-311++G(2d,2p) level for structures optimized at the B3LYP/6-311++G(d,p) level, ΔE-based Boltzmann (red lines) and ΔΔG-based Boltzmann averaged (blue solid lines). Insert shows the comparison between Boltzmann averaged ECD spectrum and that calculated for ΔΔG-based on the lowest energy conformer of a given compound (dashed blue line). All calculated spectra were wavelength corrected to match experimental UV maxima.

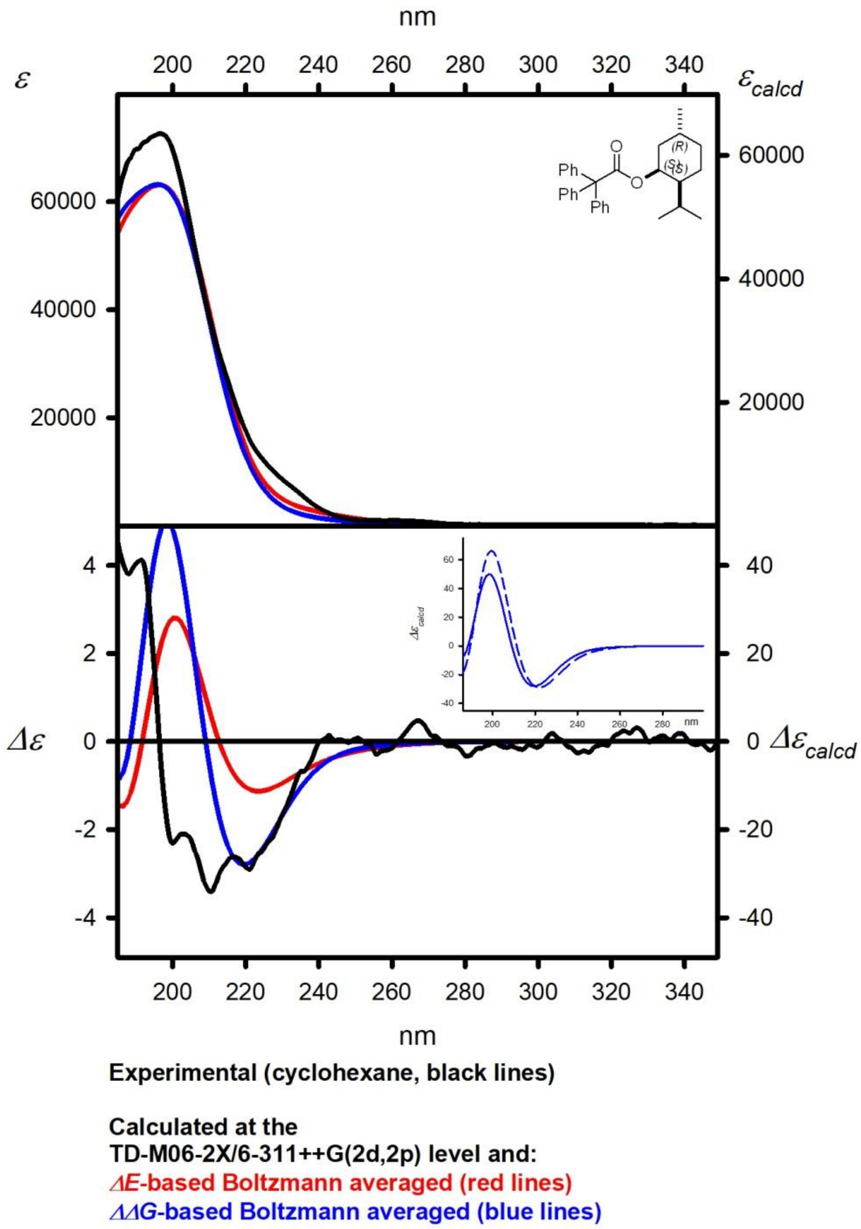

Figure SI_96. UV (upper panel) and ECD (lower panel) spectra of **11**, experimental, measured in cyclohexane (solid black lines) and calculated at the TD-M06-2X/6-311++G(2d,2p) level for structures optimized at the B3LYP/6-311++G(d,p) level, ΔE-based Boltzmann (red lines) and ΔΔG-based Boltzmann averaged (blue solid lines). Insert shows the comparison between Boltzmann averaged ECD spectrum and that calculated for ΔΔG-based on the lowest energy conformer of a given compound (dashed blue line). All calculated spectra were wavelength corrected to match experimental UV maxima.

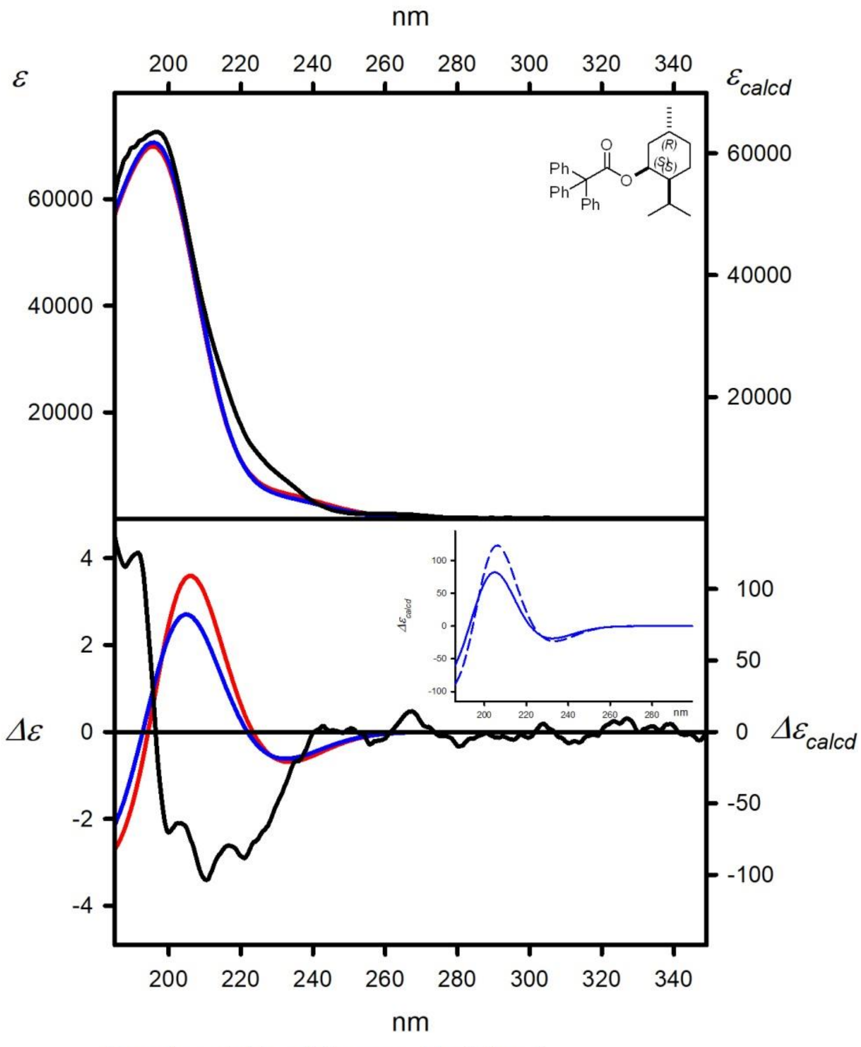

Figure SI_97. UV (upper panel) and ECD (lower panel) spectra of **11**, experimental, measured in cyclohexane (solid black lines) and calculated at the TD-CAM-B3LYP/6-311++G(2d,2p) level for structures optimized at the M06-2X/6-311++G(d,p) level, ΔE-based Boltzmann (red lines) and ΔΔG-based Boltzmann averaged (blue solid lines). Insert shows the comparison between Boltzmann averaged ECD spectrum and that calculated for ΔΔG-based on the lowest energy conformer of a given compound (dashed blue line). All calculated spectra were wavelength corrected to match experimental UV maxima.

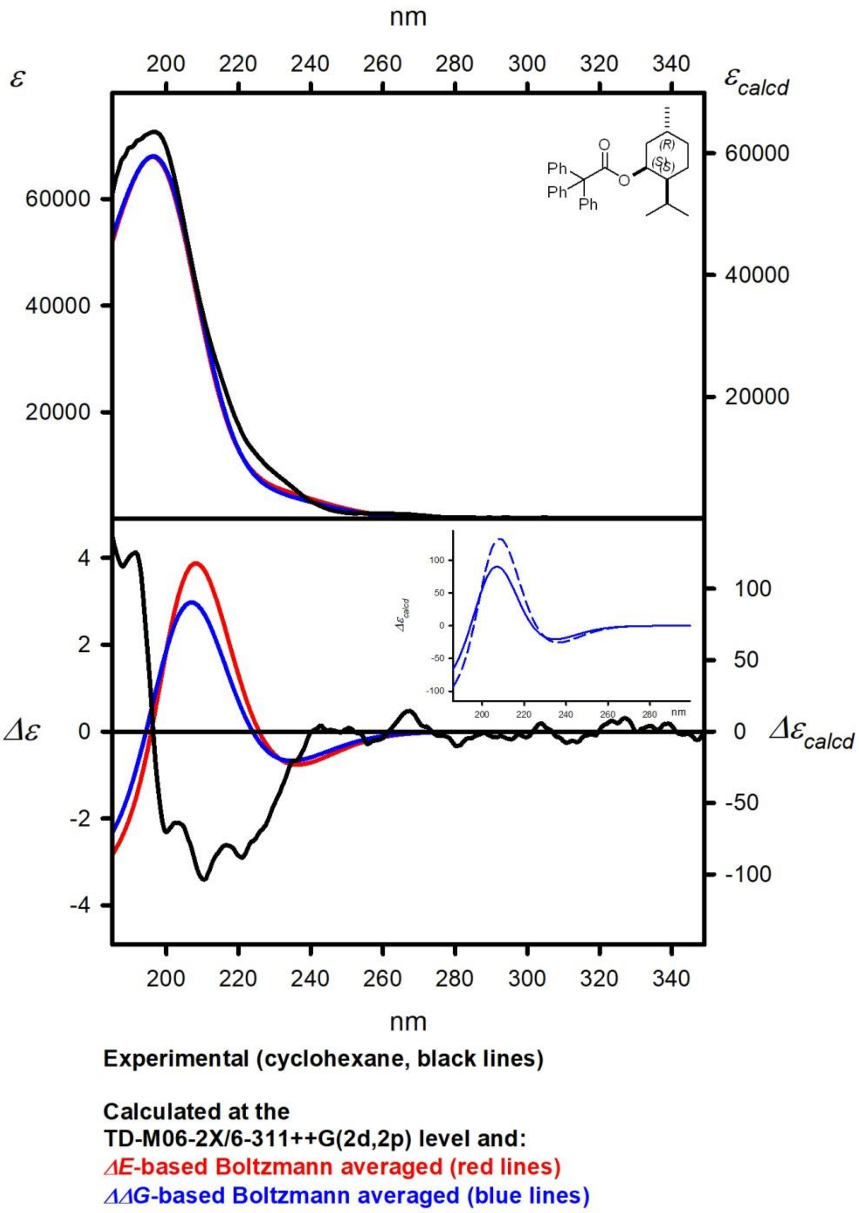

Figure SI_98. UV (upper panel) and ECD (lower panel) spectra of **11**, experimental, measured in cyclohexane (solid black lines) and calculated at the TD-M06-2X/6-311++G(2d,2p) level for structures optimized at the M06-2X/6-311++G(d,p) level, ΔE-based Boltzmann (red lines) and ΔΔG-based Boltzmann averaged (blue solid lines). Insert shows the comparison between Boltzmann averaged ECD spectrum and that calculated for ΔΔG-based on the lowest energy conformer of a given compound (dashed blue line). All calculated spectra were wavelength corrected to match experimental UV maxima.

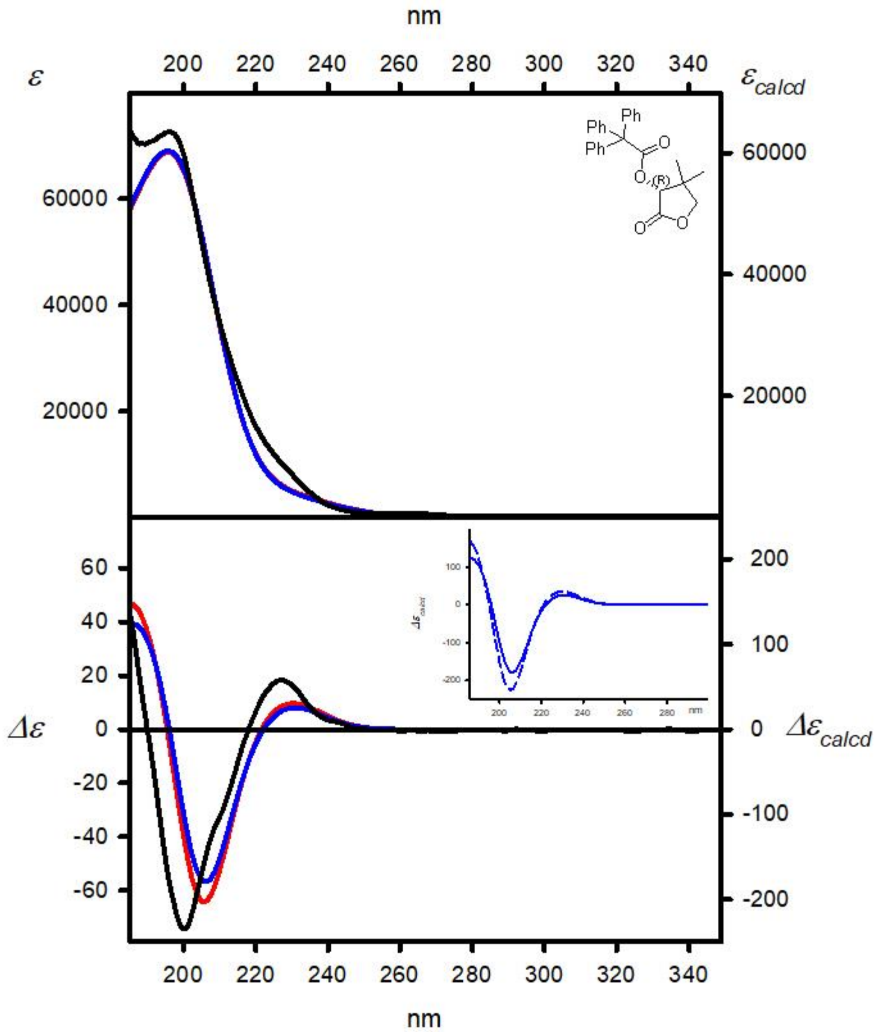

Figure SI_99. UV (upper panel) and ECD (lower panel) spectra of **14**, experimental, measured in cyclohexane (solid black lines) and calculated at the TD-CAM-B3LYP/6-311++G(2d,2p) level for structures optimized at the B3LYP/6-311++G(d,p) level, ΔE-based Boltzmann (red lines) and ΔΔG-based Boltzmann averaged (blue solid lines). Insert shows the comparison between Boltzmann averaged ECD spectrum and that calculated for ΔΔG-based on the lowest energy conformer of a given compound (dashed blue line). All calculated spectra were wavelength corrected to match experimental UV maxima.

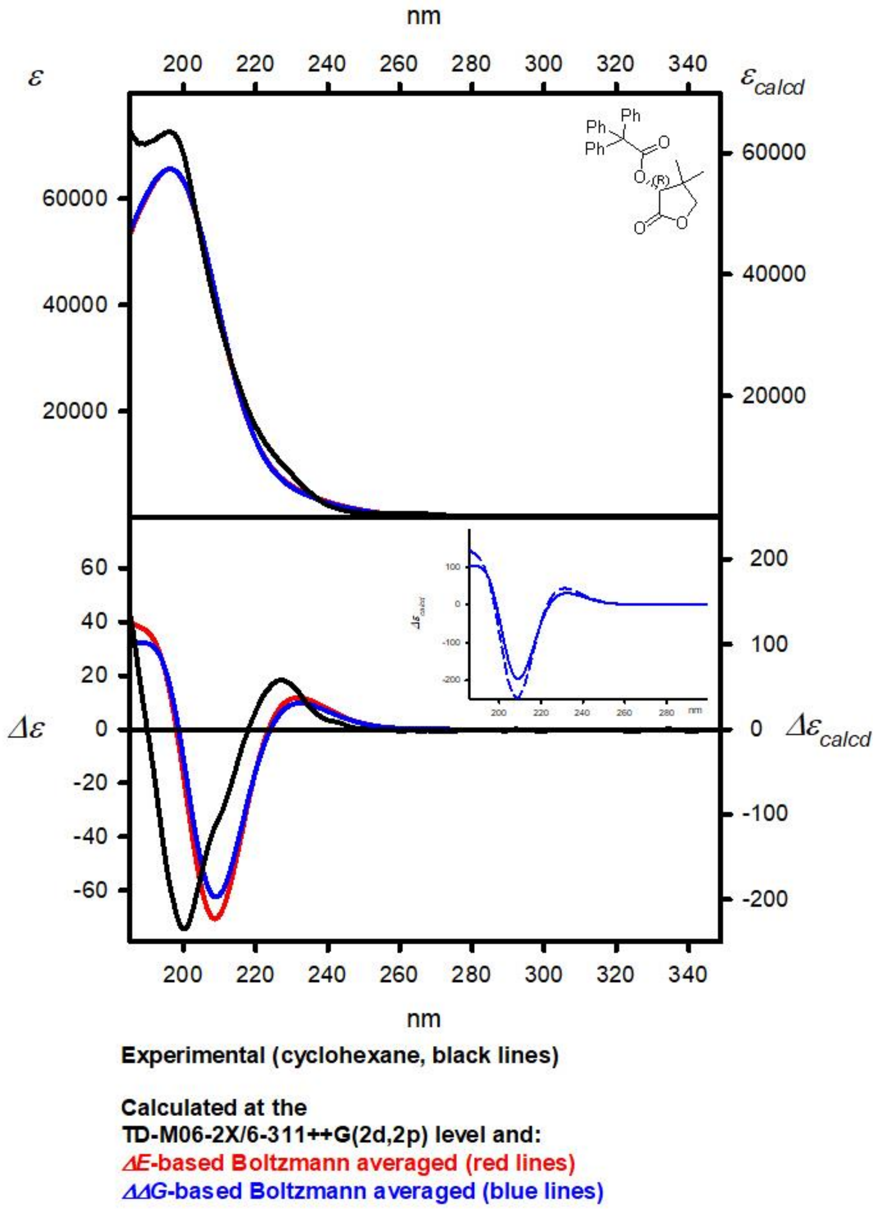

Figure SI_100. UV (upper panel) and ECD (lower panel) spectra of **14**, experimental, measured in cyclohexane (solid black lines) and calculated at the TD-M06-2X/6-311++G(2d,2p) level for structures optimized at the B3LYP/6-311++G(d,p) level, ΔE-based Boltzmann (red lines) and ΔΔG-based Boltzmann averaged (blue solid lines). Insert shows the comparison between Boltzmann averaged ECD spectrum and that calculated for ΔΔG-based on the lowest energy conformer of a given compound (dashed blue line). All calculated spectra were wavelength corrected to match experimental UV maxima.

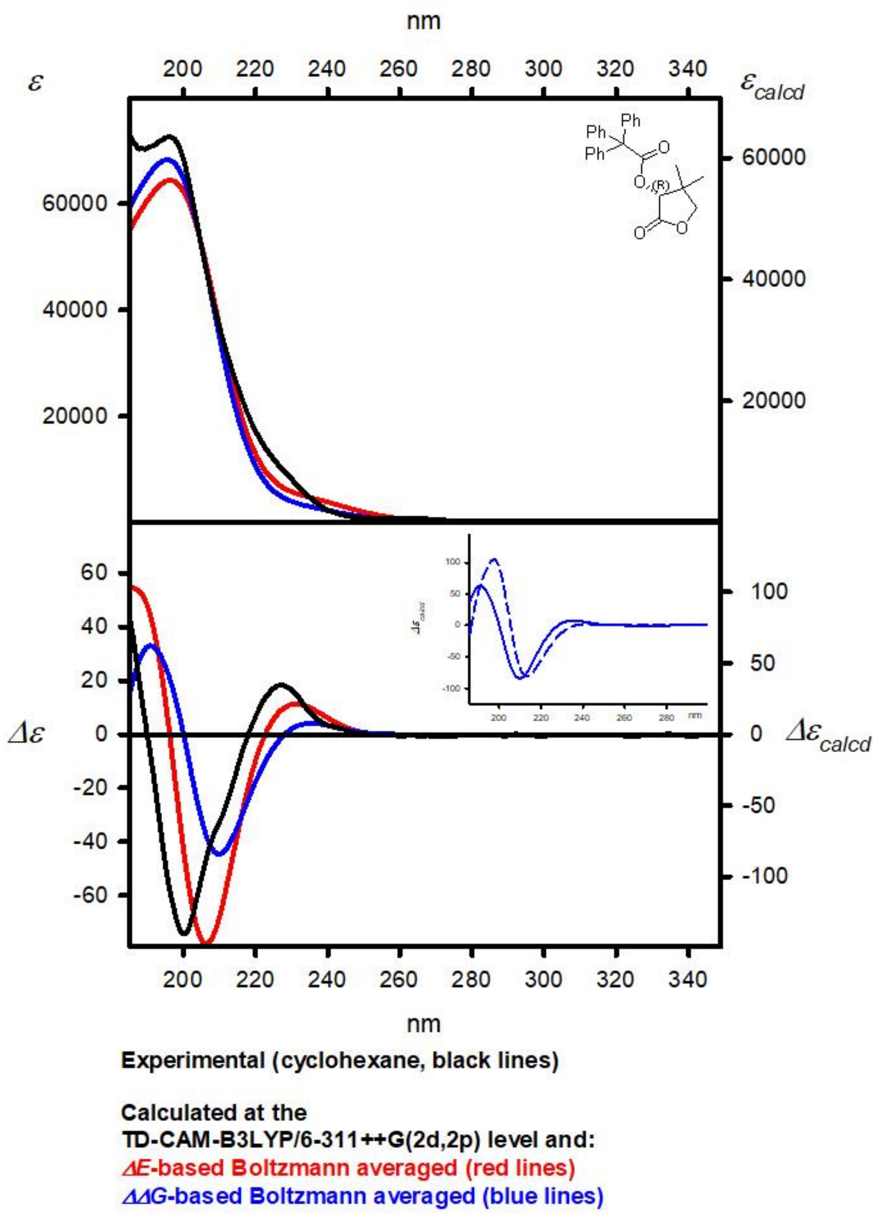

Figure SI_101. UV (upper panel) and ECD (lower panel) spectra of **14**, experimental, measured in cyclohexane (solid black lines) and calculated at the TD-CAM-B3LYP/6-311++G(2d,2p) level for structures optimized at the M06-2X/6-311++G(d,p) level, ΔE-based Boltzmann (red lines) and ΔΔG-based Boltzmann averaged (blue solid lines). Insert shows the comparison between Boltzmann averaged ECD spectrum and that calculated for ΔΔG-based on the lowest energy conformer of a given compound (dashed blue line). All calculated spectra were wavelength corrected to match experimental UV maxima.

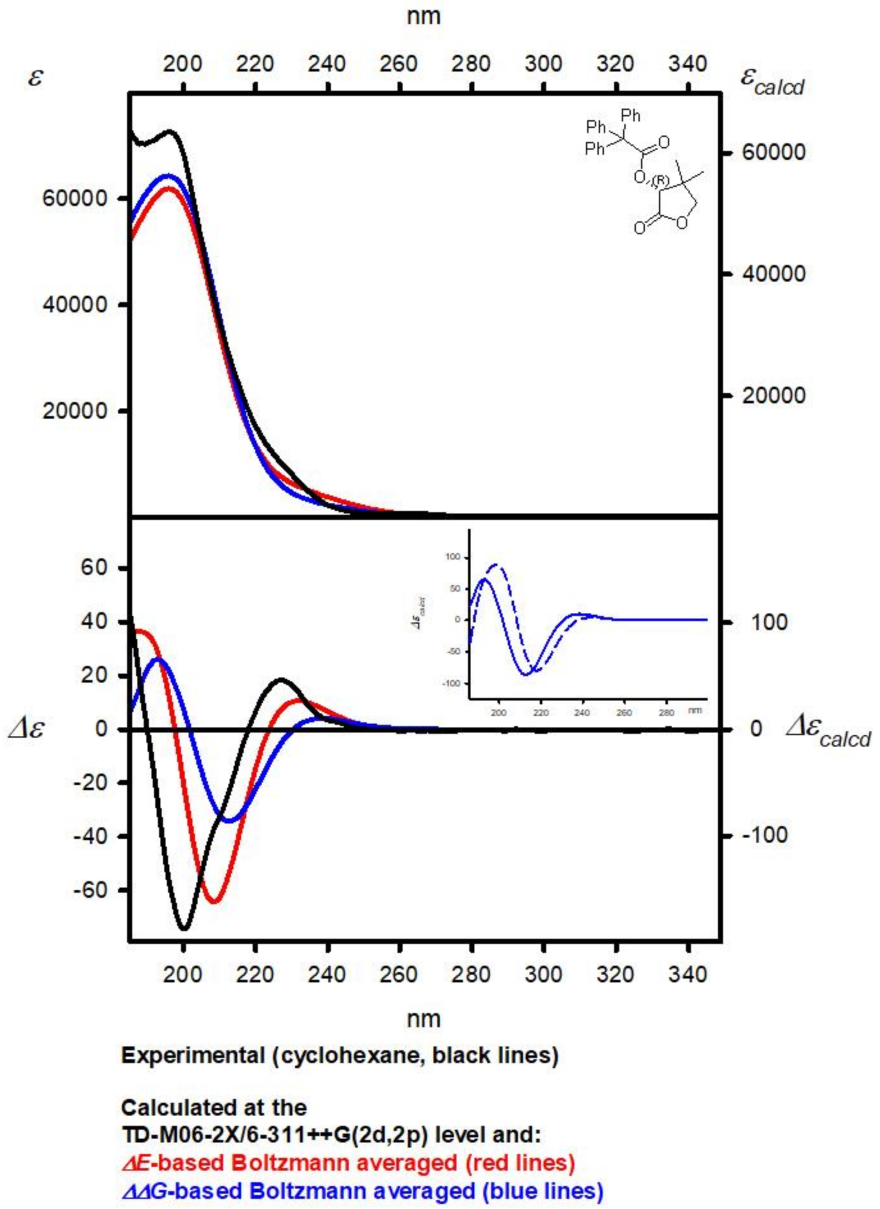

Figure SI_102. UV (upper panel) and ECD (lower panel) spectra of **14**, experimental, measured in cyclohexane (solid black lines) and calculated at the TD-M06-2X/6-311++G(2d,2p) level for structures optimized at the M06-2X/6-311++G(d,p) level, ΔE-based Boltzmann (red lines) and ΔΔG-based Boltzmann averaged (blue solid lines). Insert shows the comparison between Boltzmann averaged ECD spectrum and that calculated for ΔΔG-based on the lowest energy conformer of a given compound (dashed blue line). All calculated spectra were wavelength corrected to match experimental UV maxima.

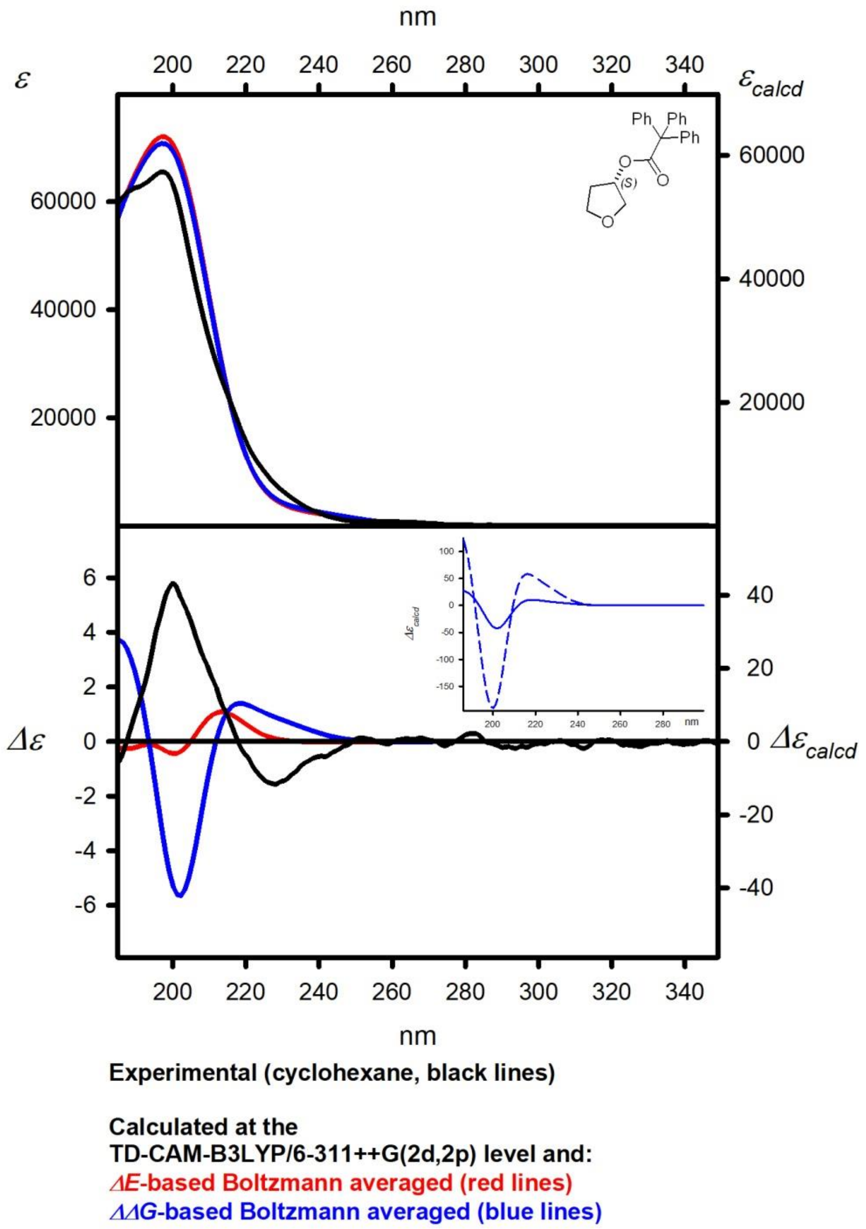

Figure SI_103. UV (upper panel) and ECD (lower panel) spectra of **15**, experimental, measured in cyclohexane (solid black lines) and calculated at the TD-CAM-B3LYP/6-311++G(2d,2p) level for structures optimized at the B3LYP/6-311++G(d,p) level, ΔE-based Boltzmann (red lines) and ΔΔG-based Boltzmann averaged (blue solid lines). Insert shows the comparison between Boltzmann averaged ECD spectrum and that calculated for ΔΔG-based on the lowest energy conformer of a given compound (dashed blue line). All calculated spectra were wavelength corrected to match experimental UV maxima.

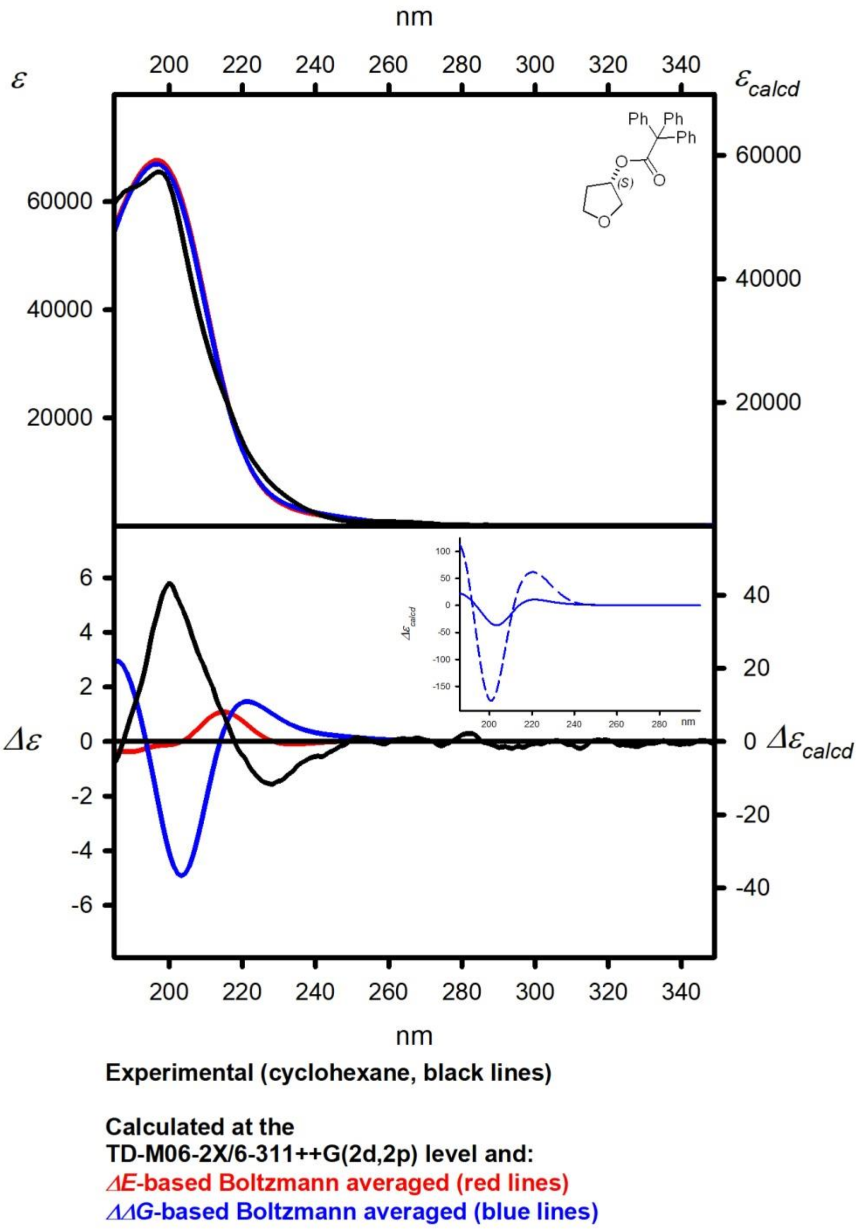

Figure SI_104. UV (upper panel) and ECD (lower panel) spectra of **15**, experimental, measured in cyclohexane (solid black lines) and calculated at the TD-M06-2X/6-311++G(2d,2p) level for structures optimized at the B3LYP/6-311++G(d,p) level, ΔE-based Boltzmann (red lines) and ΔΔG-based Boltzmann averaged (blue solid lines). Insert shows the comparison between Boltzmann averaged ECD spectrum and that calculated for ΔΔG-based on the lowest energy conformer of a given compound (dashed blue line). All calculated spectra were wavelength corrected to match experimental UV maxima.

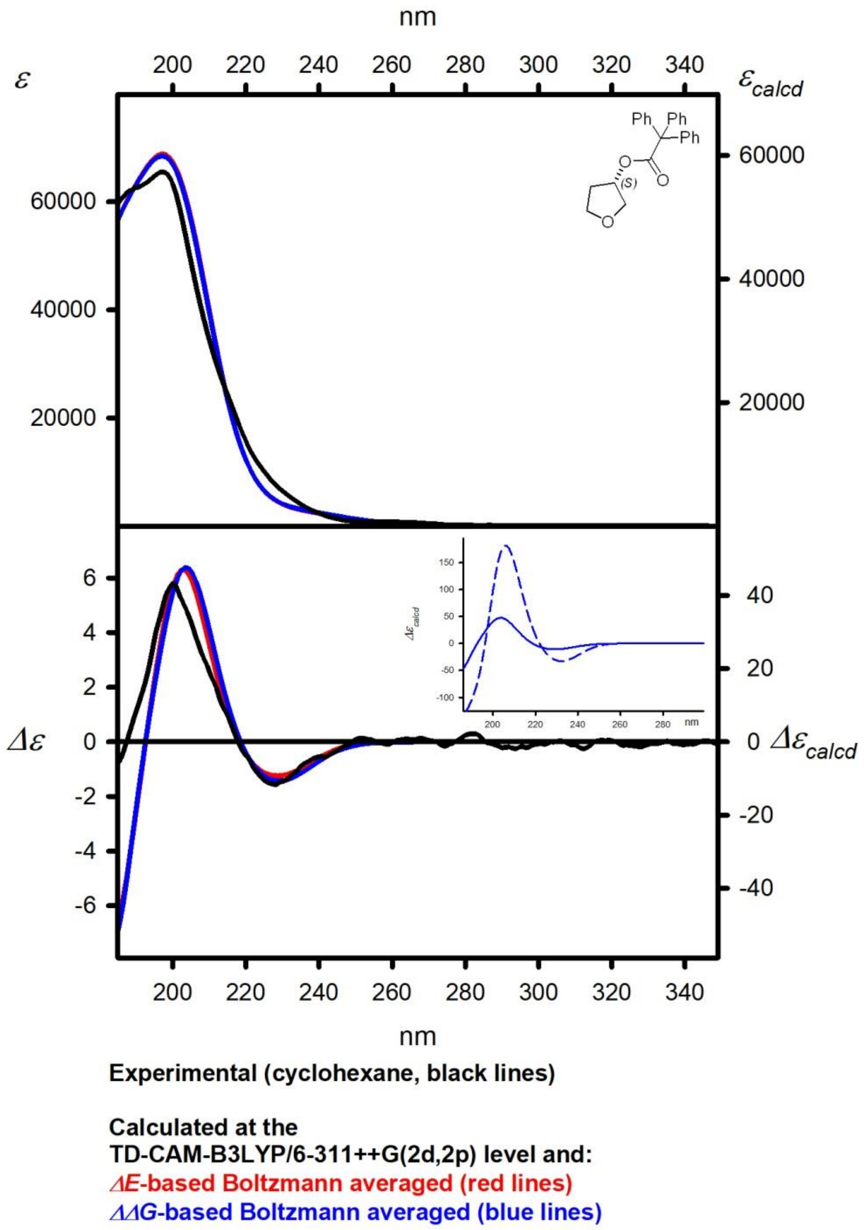

Figure SI_105. UV (upper panel) and ECD (lower panel) spectra of **15**, experimental, measured in cyclohexane (solid black lines) and calculated at the TD-CAM-B3LYP/6-311++G(2d,2p) level for structures optimized at the M06-2X/6-311++G(d,p) level, ΔE-based Boltzmann (red lines) and ΔΔG-based Boltzmann averaged (blue solid lines). Insert shows the comparison between Boltzmann averaged ECD spectrum and that calculated for ΔΔG-based on the lowest energy conformer of a given compound (dashed blue line). All calculated spectra were wavelength corrected to match experimental UV maxima.

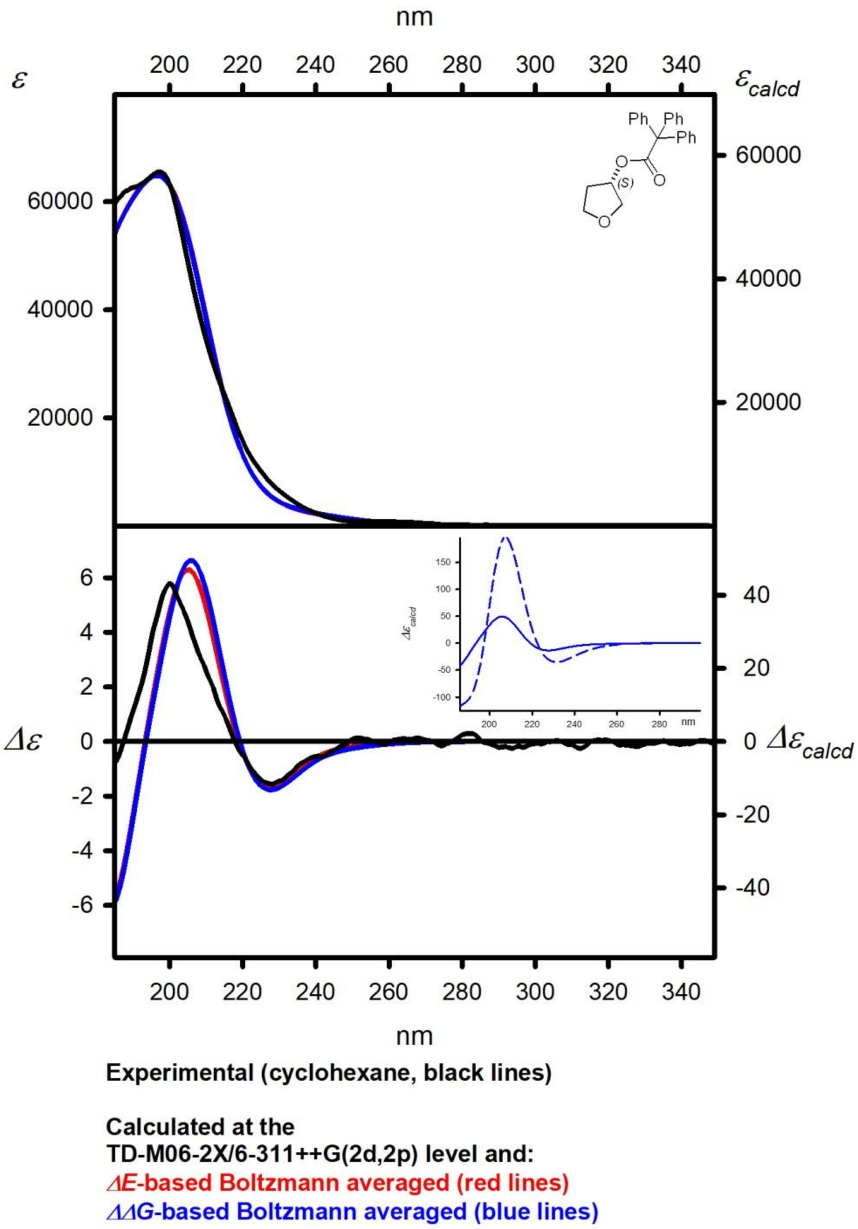

Figure SI_106. UV (upper panel) and ECD (lower panel) spectra of **15**, experimental, measured in cyclohexane (solid black lines) and calculated at the TD-M06-2X/6-311++G(2d,2p) level for structures optimized at the M06-2X/6-311++G(d,p) level, ΔE-based Boltzmann (red lines) and ΔΔG-based Boltzmann averaged (blue solid lines). Insert shows the comparison between Boltzmann averaged ECD spectrum and that calculated for ΔΔG-based on the lowest energy conformer of a given compound (dashed blue line). All calculated spectra were wavelength corrected to match experimental UV maxima.

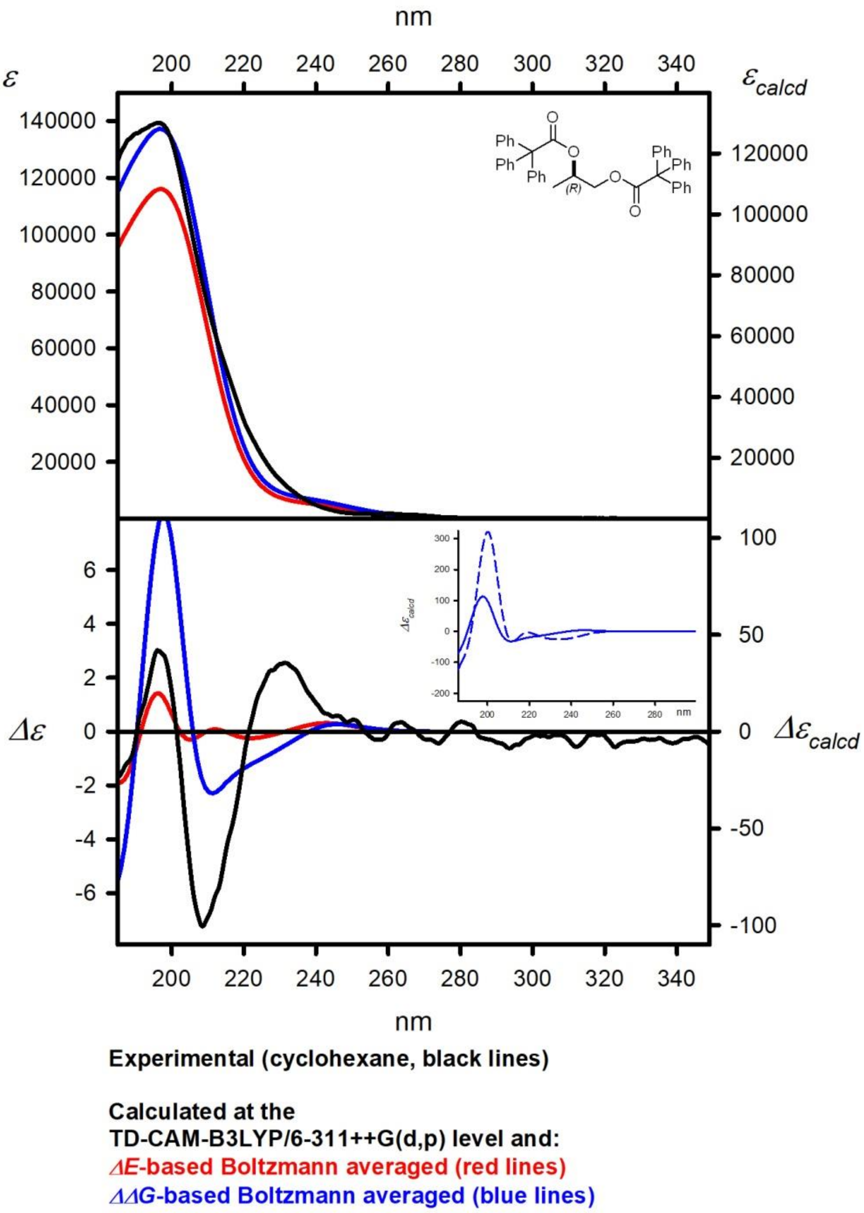

Figure SI_107. UV (upper panel) and ECD (lower panel) spectra of **18**, experimental, measured in cyclohexane (solid black lines) and calculated at the TD-CAM-B3LYP/6-311++G(d,p) level for structures optimized at the B3LYP/6-311G(d,p) level, ΔE-based Boltzmann (red lines) and ΔΔG-based Boltzmann averaged (blue solid lines). Insert shows the comparison between Boltzmann averaged ECD spectrum and that calculated for ΔΔG-based on the lowest energy conformer of a given compound (dashed blue line). All calculated spectra were wavelength corrected to match experimental UV maxima.

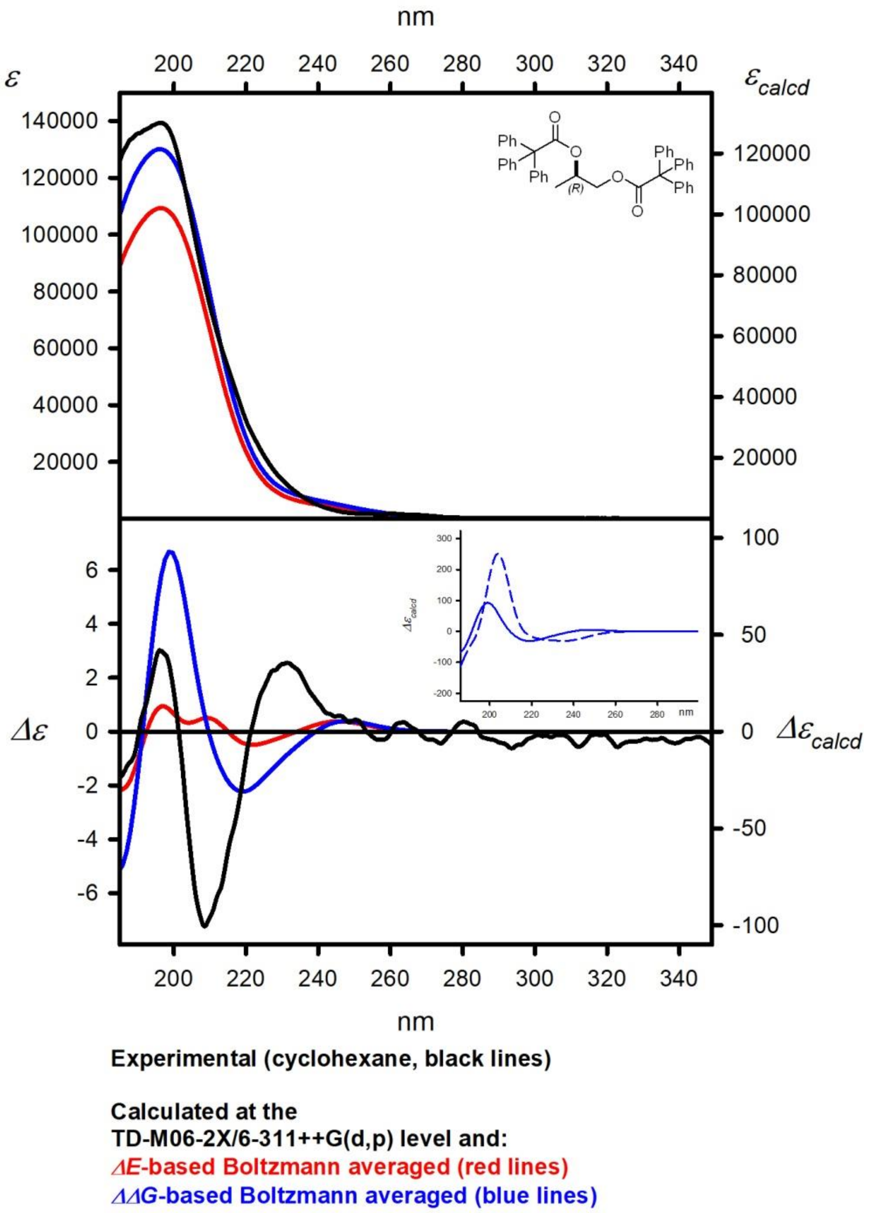

Figure SI_108. UV (upper panel) and ECD (lower panel) spectra of **18**, experimental, measured in cyclohexane (solid black lines) and calculated at the TD-M06-2X/6-311++G(d,p) level for structures optimized at the B3LYP/6-311G(d,p) level, ΔE-based Boltzmann (red lines) and ΔΔG-based Boltzmann averaged (blue solid lines). Insert shows the comparison between Boltzmann averaged ECD spectrum and that calculated for ΔΔG-based on the lowest energy conformer of a given compound (dashed blue line). All calculated spectra were wavelength corrected to match experimental UV maxima.

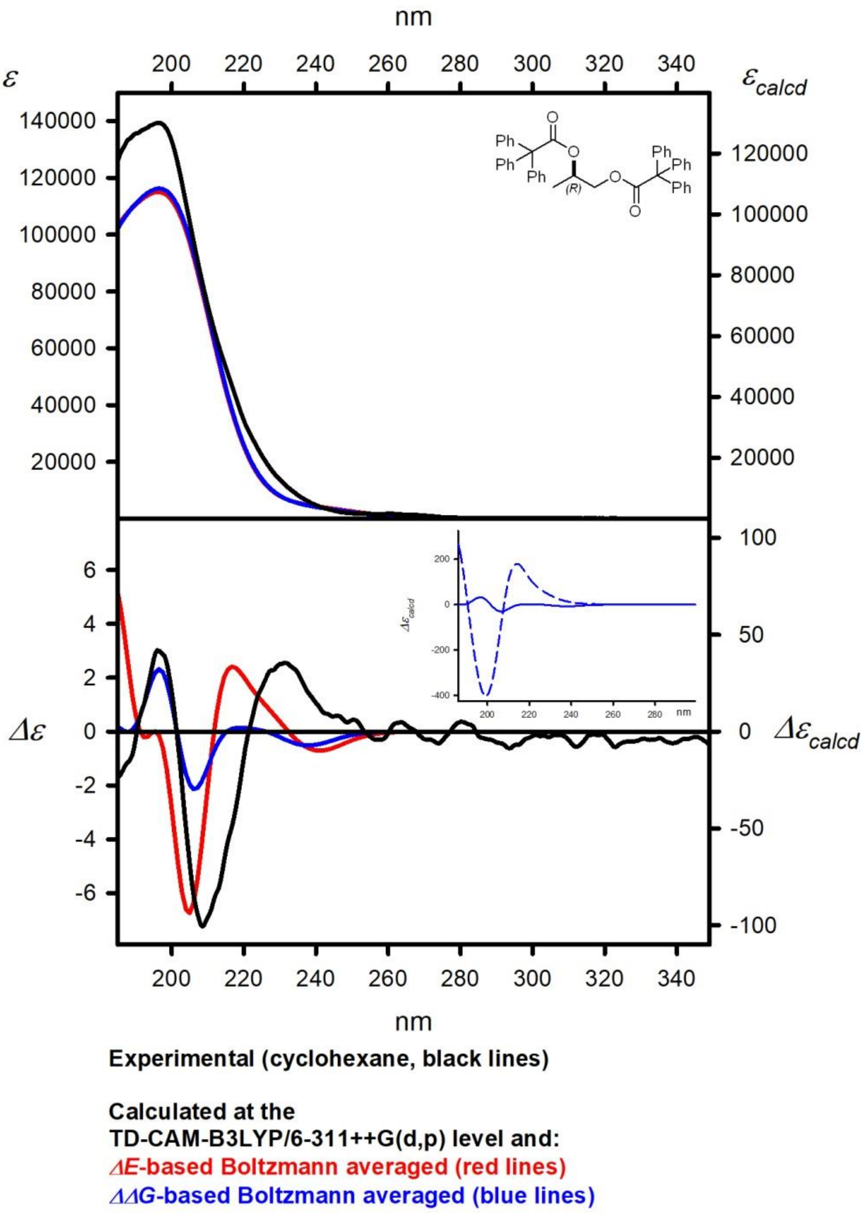

Figure SI_109. UV (upper panel) and ECD (lower panel) spectra of **18**, experimental, measured in cyclohexane (solid black lines) and calculated at the TD-CAM-B3LYP/6-311++G(d,p) level for structures optimized at the B3LYP-GD3BJ/6-311G(d,p) level, ΔE-based Boltzmann (red lines) and ΔΔG-based Boltzmann averaged (blue solid lines). Insert shows the comparison between Boltzmann averaged ECD spectrum and that calculated for ΔΔG-based on the lowest energy conformer of a given compound (dashed blue line). All calculated spectra were wavelength corrected to match experimental UV maxima.

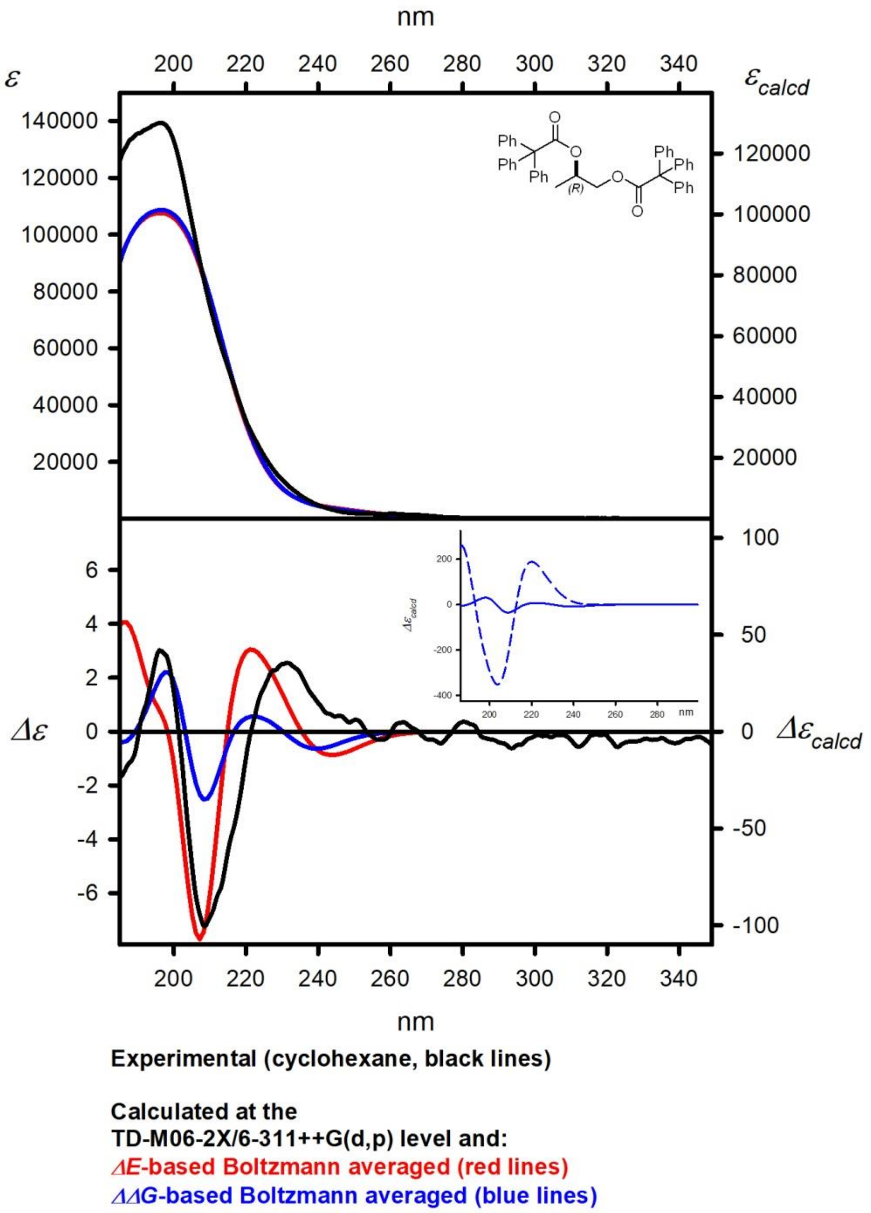

Figure SI_110. UV (upper panel) and ECD (lower panel) spectra of **18**, experimental, measured in cyclohexane (solid black lines) and calculated at the TD-M06-2X/6-311++G(d,p) level for structures optimized at the B3LYP-GD3BJ/6-311G(d,p) level, ΔE-based Boltzmann (red lines) and ΔΔG-based Boltzmann averaged (blue solid lines). Insert shows the comparison between Boltzmann averaged ECD spectrum and that calculated for ΔΔG-based on the lowest energy conformer of a given compound (dashed blue line). All calculated spectra were wavelength corrected to match experimental UV maxima.

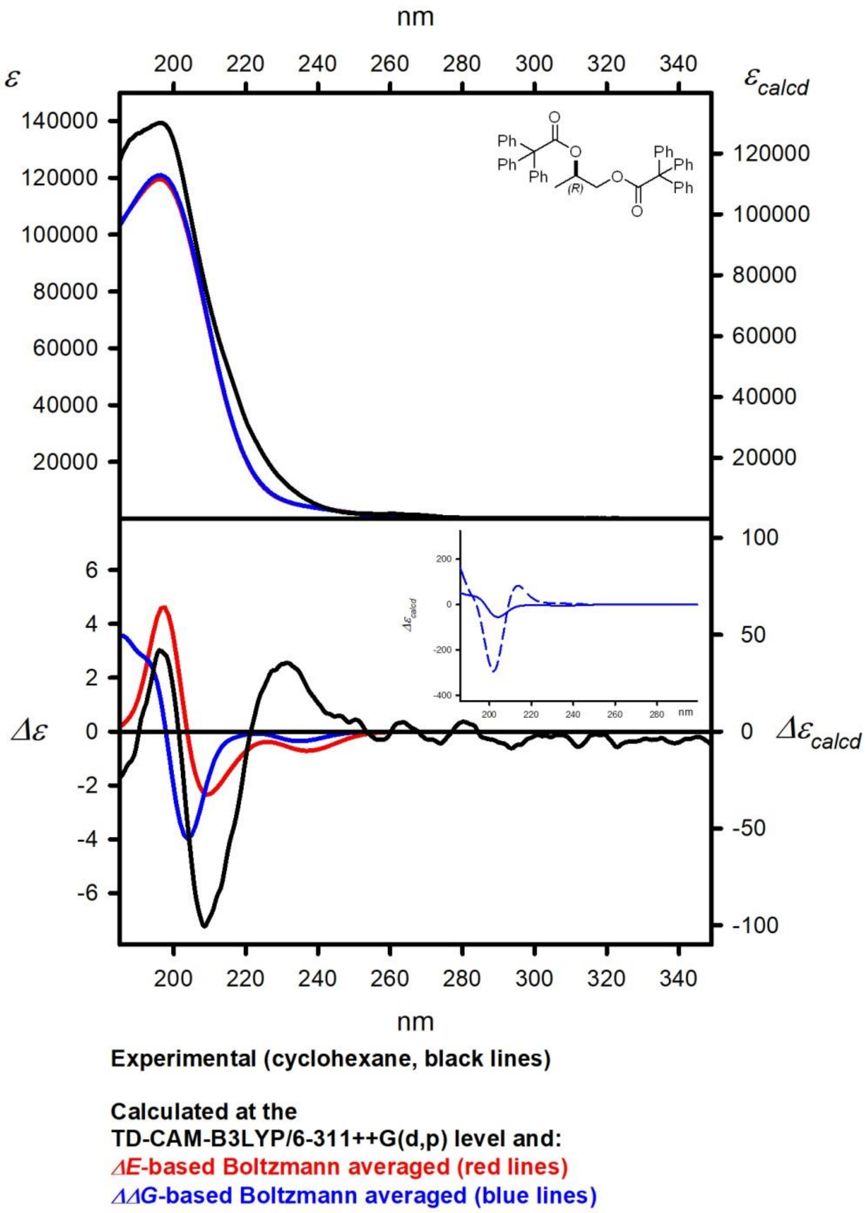

Figure SI_111. UV (upper panel) and ECD (lower panel) spectra of **18**, experimental, measured in cyclohexane (solid black lines) and calculated at the TD-CAM-B3LYP/6-311++G(d,p) level for structures optimized at the M06-2X/6-311G(d,p) level, ΔE-based Boltzmann (red lines) and ΔΔG-based Boltzmann averaged (blue solid lines). Insert shows the comparison between Boltzmann averaged ECD spectrum and that calculated for ΔΔG-based on the lowest energy conformer of a given compound (dashed blue line). All calculated spectra were wavelength corrected to match experimental UV maxima.

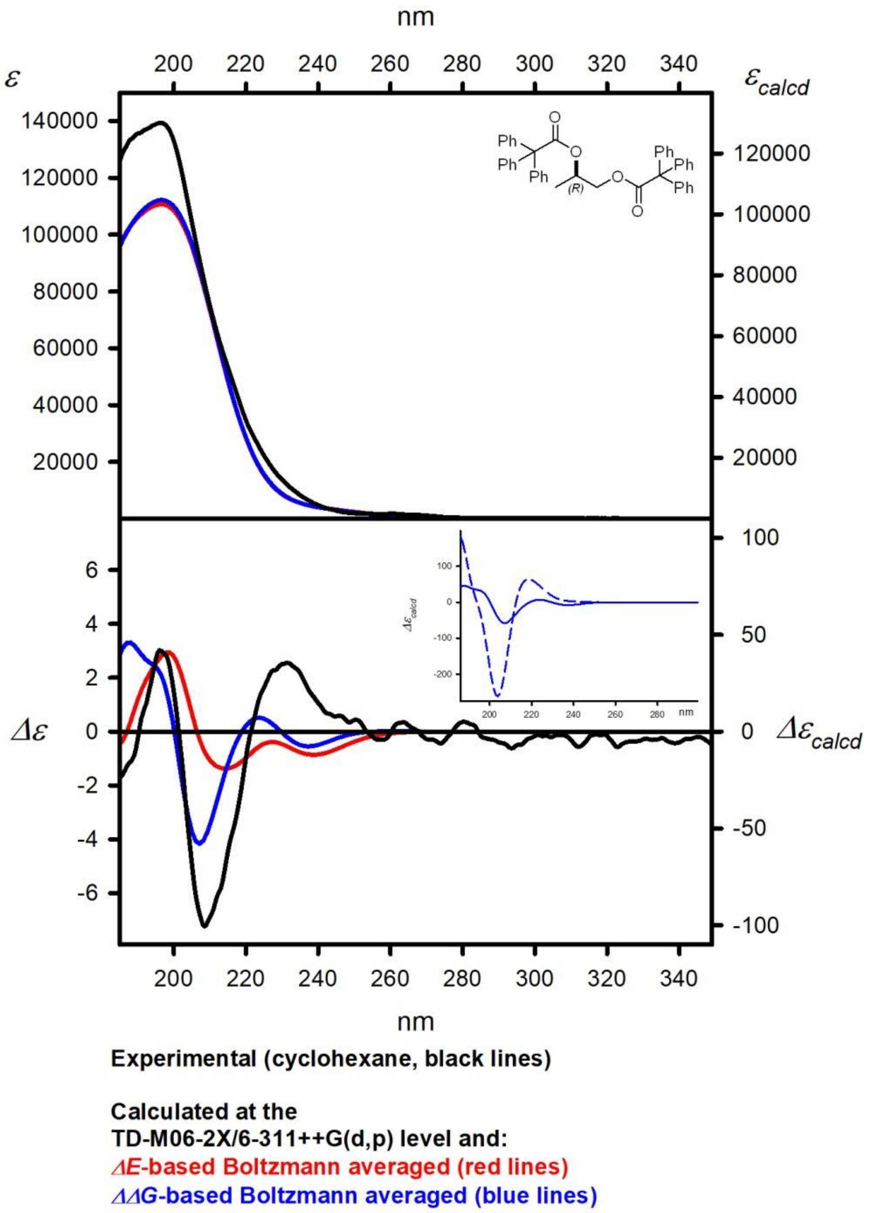

Figure SI_112. UV (upper panel) and ECD (lower panel) spectra of **18**, experimental, measured in cyclohexane (solid black lines) and calculated at the TD-M06-2X/6-311++G(d,p) level for structures optimized at the M06-2X/6-311G(d,p) level, ΔE-based Boltzmann (red lines) and ΔΔG-based Boltzmann averaged (blue solid lines). Insert shows the comparison between Boltzmann averaged ECD spectrum and that calculated for ΔΔG-based on the lowest energy conformer of a given compound (dashed blue line). All calculated spectra were wavelength corrected to match experimental UV maxima.

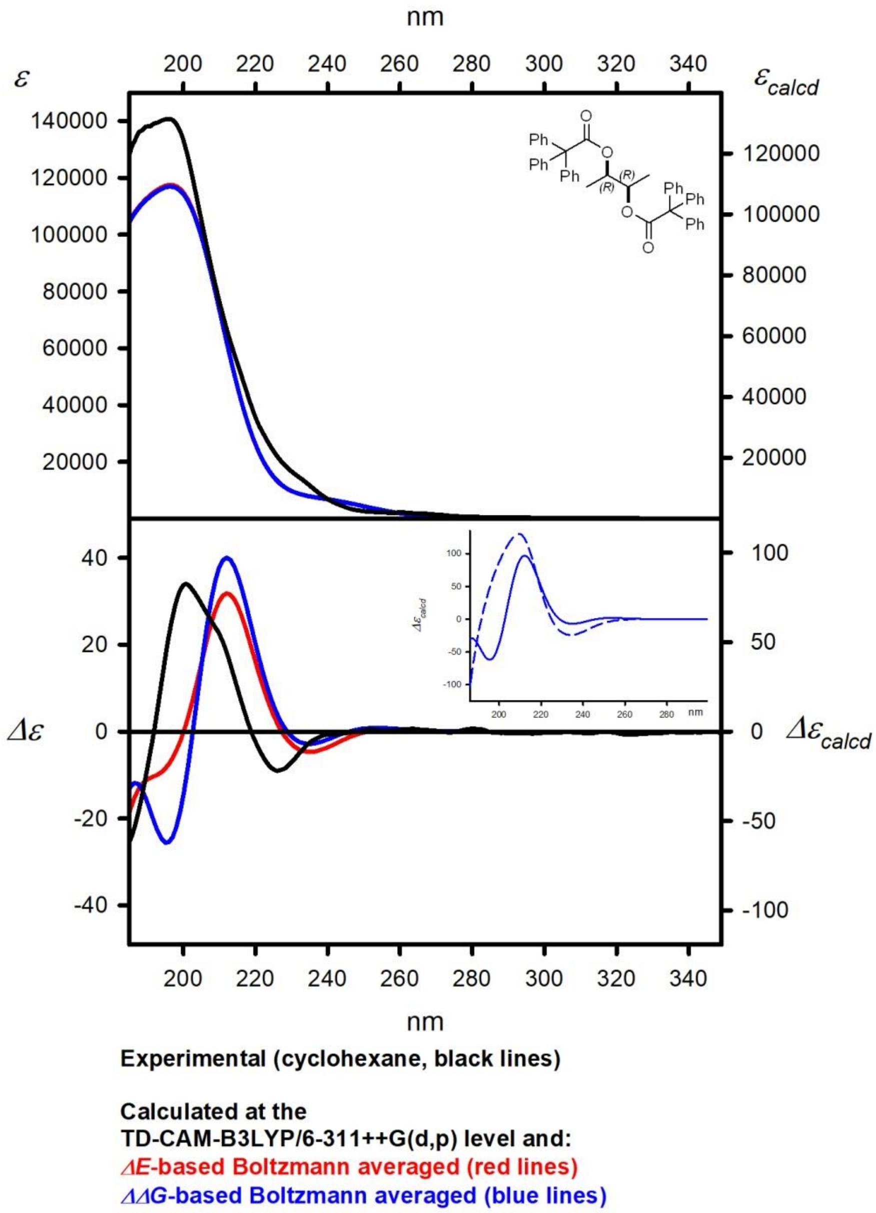

Figure SI_113. UV (upper panel) and ECD (lower panel) spectra of **20**, experimental, measured in cyclohexane (solid black lines) and calculated at the TD-CAM-B3LYP/6-311++G(d,p) level for structures optimized at the B3LYP/6-311G(d,p) level, ΔE-based Boltzmann (red lines) and ΔΔG-based Boltzmann averaged (blue solid lines). Insert shows the comparison between Boltzmann averaged ECD spectrum and that calculated for ΔΔG-based on the lowest energy conformer of a given compound (dashed blue line). All calculated spectra were wavelength corrected to match experimental UV maxima.

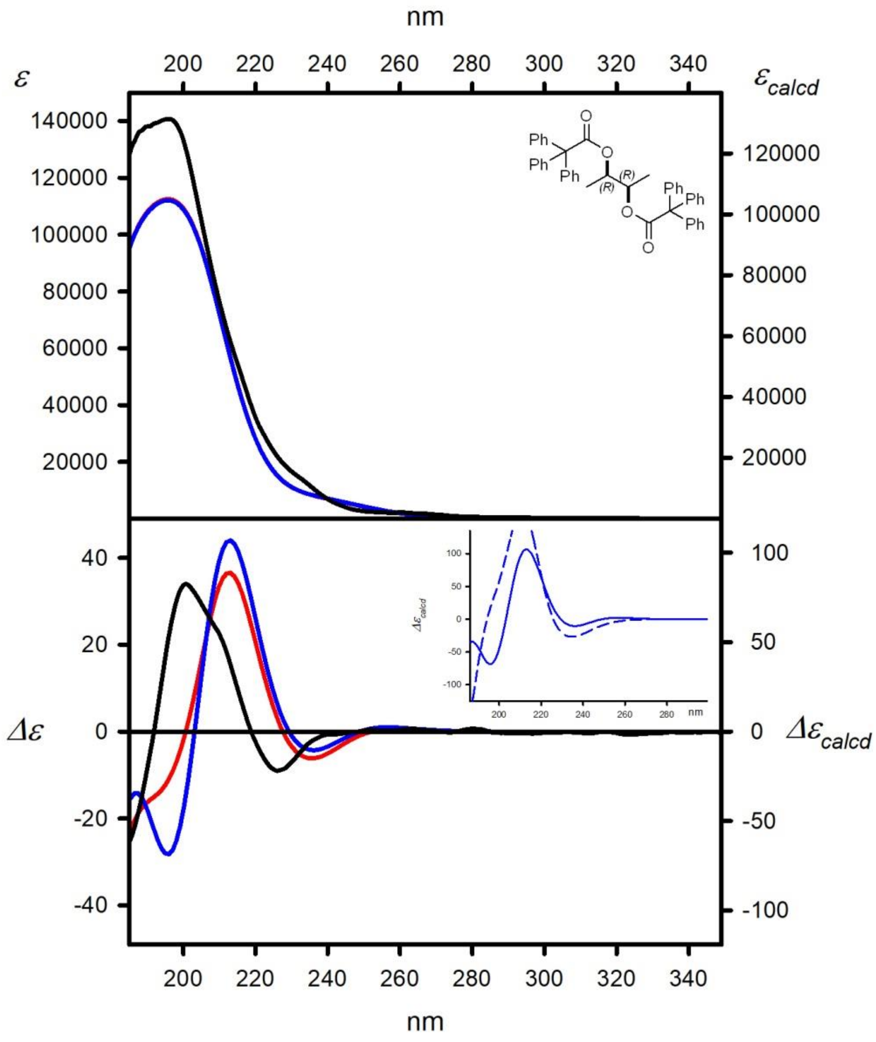

Experimental (cyclohexane, black lines)

Calculated at the
TD-M06-2X/6-311++G(d,p) level and:
***ΔE*-based Boltzmann averaged (red lines)**
***ΔΔG*-based Boltzmann averaged (blue lines)**

Figure SI_114. UV (upper panel) and ECD (lower panel) spectra of **20**, experimental, measured in cyclohexane (solid black lines) and calculated at the TD-M06-2X/6-311++G(d,p) level for structures optimized at the B3LYP/6-311G(d,p) level, ΔE-based Boltzmann (red lines) and ΔΔG-based Boltzmann averaged (blue solid lines). Insert shows the comparison between Boltzmann averaged ECD spectrum and that calculated for ΔΔG-based on the lowest energy conformer of a given compound (dashed blue line). All calculated spectra were wavelength corrected to match experimental UV maxima.

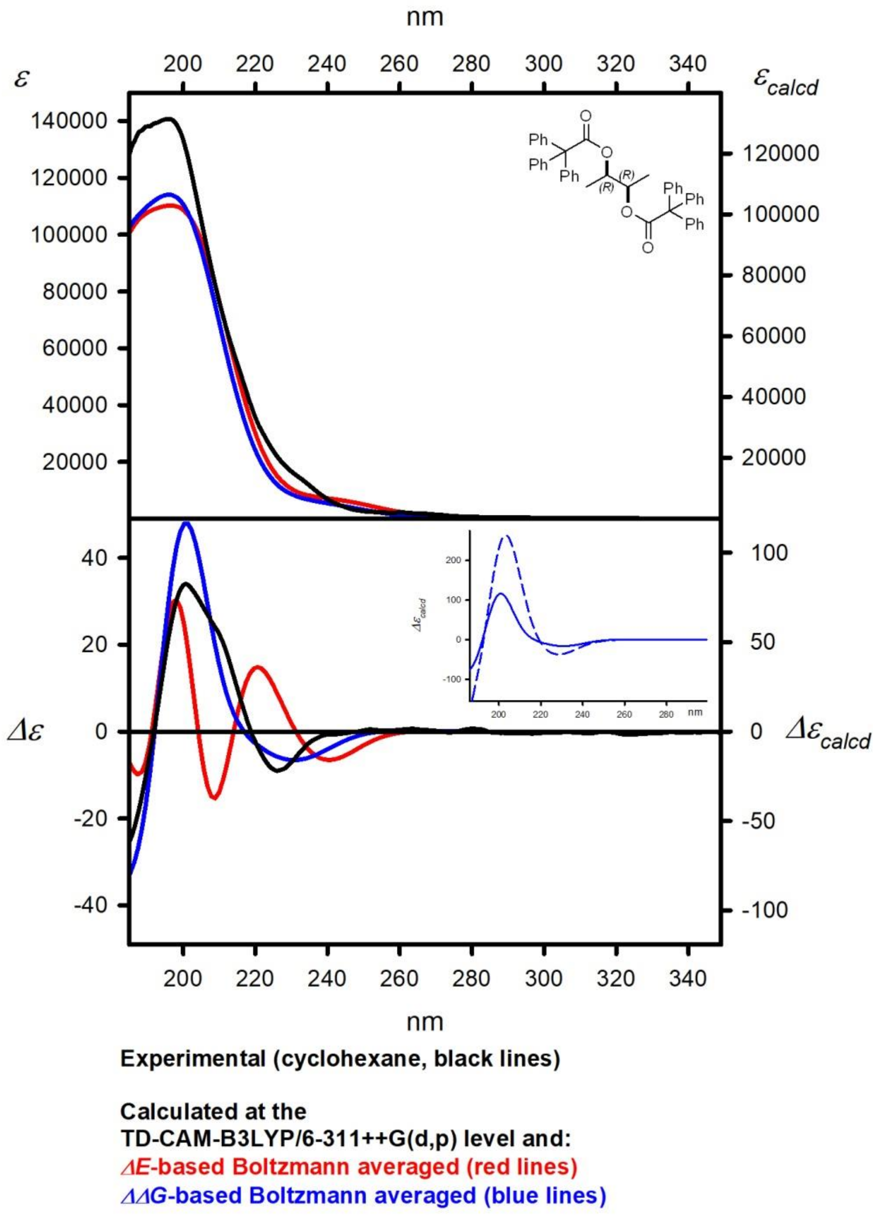

Figure SI_115. UV (upper panel) and ECD (lower panel) spectra of **20**, experimental, measured in cyclohexane (solid black lines) and calculated at the TD-CAM-B3LYP/6-311++G(d,p) level for structures optimized at the B3LYP-GD3BJ/6-311G(d,p) level, ΔE-based Boltzmann (red lines) and ΔΔG-based Boltzmann averaged (blue solid lines). Insert shows the comparison between Boltzmann averaged ECD spectrum and that calculated for ΔΔG-based on the lowest energy conformer of a given compound (dashed blue line). All calculated spectra were wavelength corrected to match experimental UV maxima.

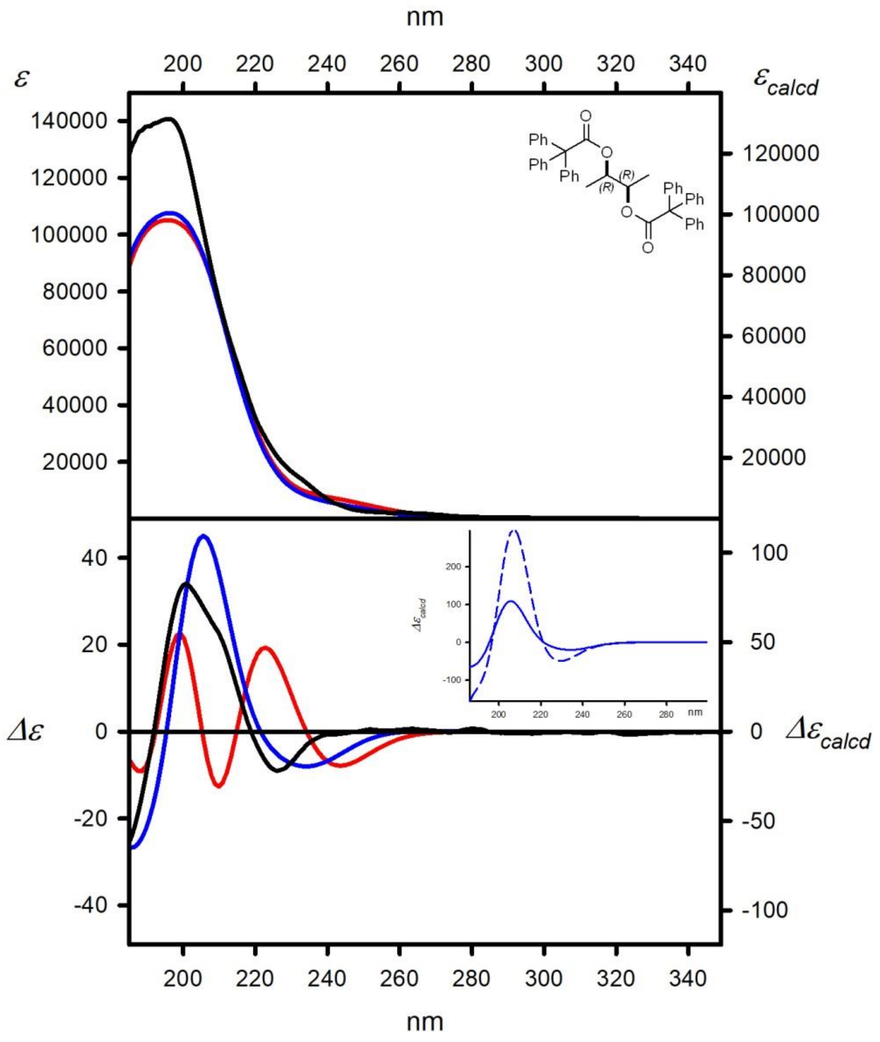

Experimental (cyclohexane, black lines)

Calculated at the
TD-M06-2X/6-311++G(d,p) level and:
***ΔE*-based Boltzmann averaged (red lines)**
***ΔΔG*-based Boltzmann averaged (blue lines)**

Figure SI_116. UV (upper panel) and ECD (lower panel) spectra of **20**, experimental, measured in cyclohexane (solid black lines) and calculated at the TD-M06-2X/6-311++G(d,p) level for structures optimized at the B3LYP-GD3BJ/6-311G(d,p) level, ΔE-based Boltzmann (red lines) and ΔΔG-based Boltzmann averaged (blue solid lines). Insert shows the comparison between Boltzmann averaged ECD spectrum and that calculated for ΔΔG-based on the lowest energy conformer of a given compound (dashed blue line). All calculated spectra were wavelength corrected to match experimental UV maxima.

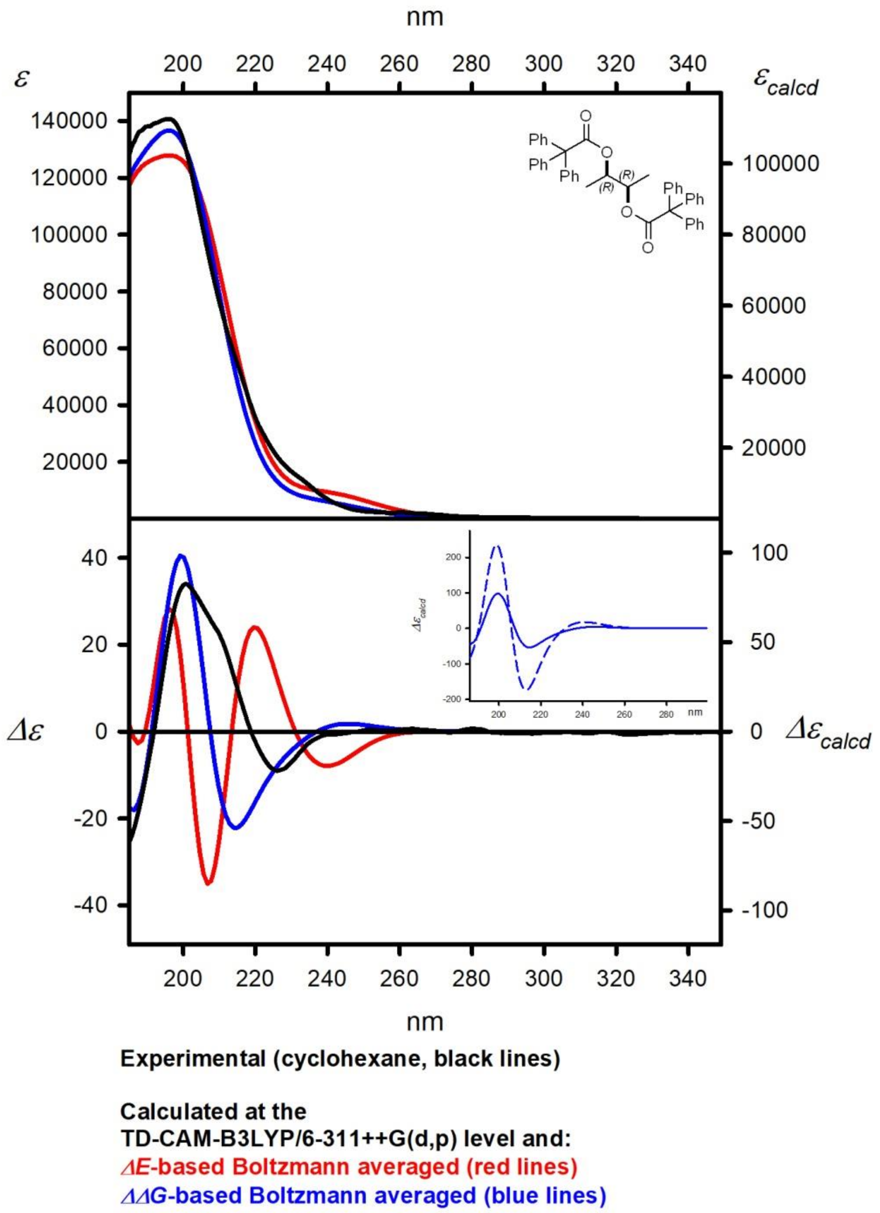

Figure SI_117. UV (upper panel) and ECD (lower panel) spectra of **20**, experimental, measured in cyclohexane (solid black lines) and calculated at the TD-CAM-B3LYP/6-311++G(d,p) level for structures optimized at the M06-2X/6-311G(d,p) level, ΔE-based Boltzmann (red lines) and ΔΔG-based Boltzmann averaged (blue solid lines). Insert shows the comparison between Boltzmann averaged ECD spectrum and that calculated for ΔΔG-based on the lowest energy conformer of a given compound (dashed blue line). All calculated spectra were wavelength corrected to match experimental UV maxima.

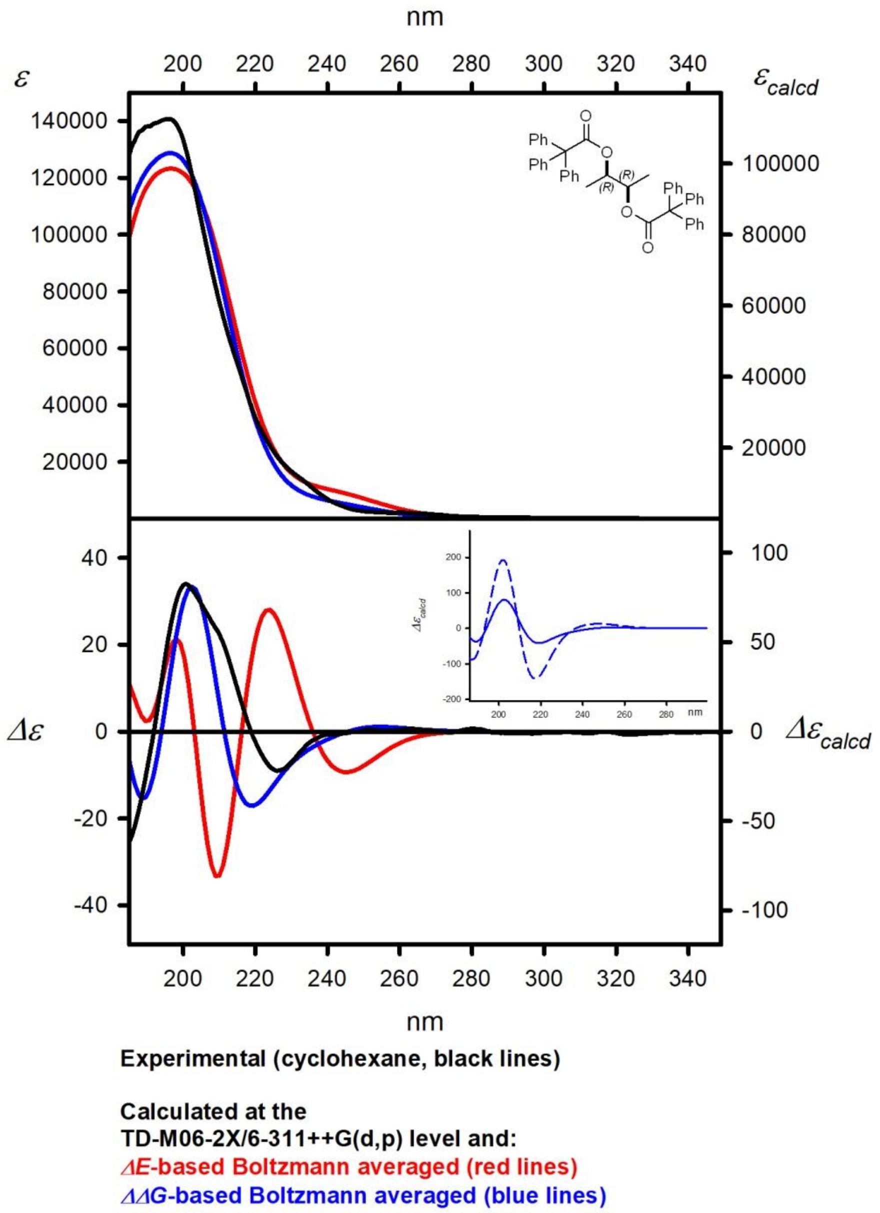

Figure SI_118. UV (upper panel) and ECD (lower panel) spectra of **20**, experimental, measured in cyclohexane (solid black lines) and calculated at the TD-M06-2X/6-311++G(d,p) level for structures optimized at the M06-2X/6-311G(d,p) level, ΔE-based Boltzmann (red lines) and ΔΔG-based Boltzmann averaged (blue solid lines). Insert shows the comparison between Boltzmann averaged ECD spectrum and that calculated for ΔΔG-based on the lowest energy conformer of a given compound (dashed blue line). All calculated spectra were wavelength corrected to match experimental UV maxima.

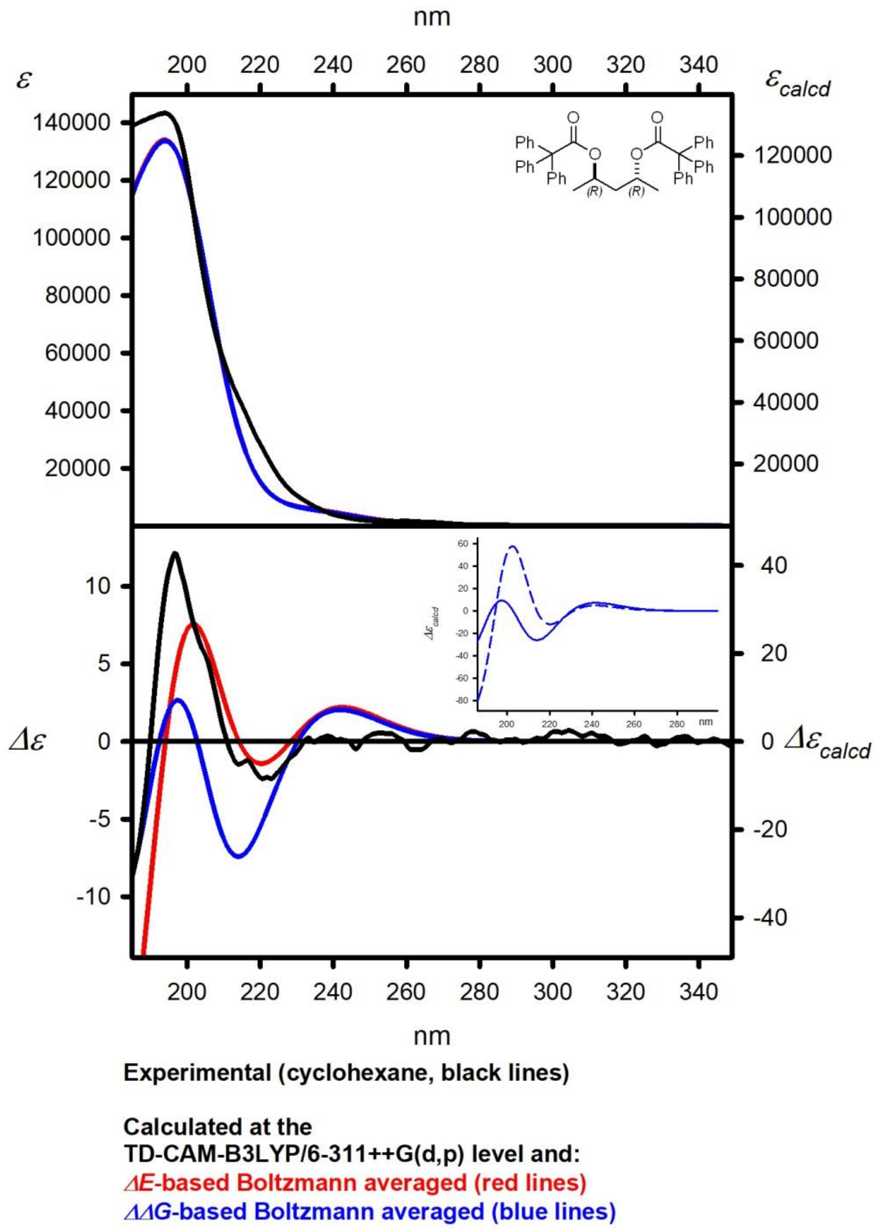

Figure SI_119. UV (upper panel) and ECD (lower panel) spectra of **21**, experimental, measured in cyclohexane (solid black lines) and calculated at the TD-CAM-B3LYP/6-311++G(d,p) level for structures optimized at the B3LYP/6-311G(d,p) level, ΔE-based Boltzmann (red lines) and ΔΔG-based Boltzmann averaged (blue solid lines). Insert shows the comparison between Boltzmann averaged ECD spectrum and that calculated for ΔΔG-based on the lowest energy conformer of a given compound (dashed blue line). All calculated spectra were wavelength corrected to match experimental UV maxima.

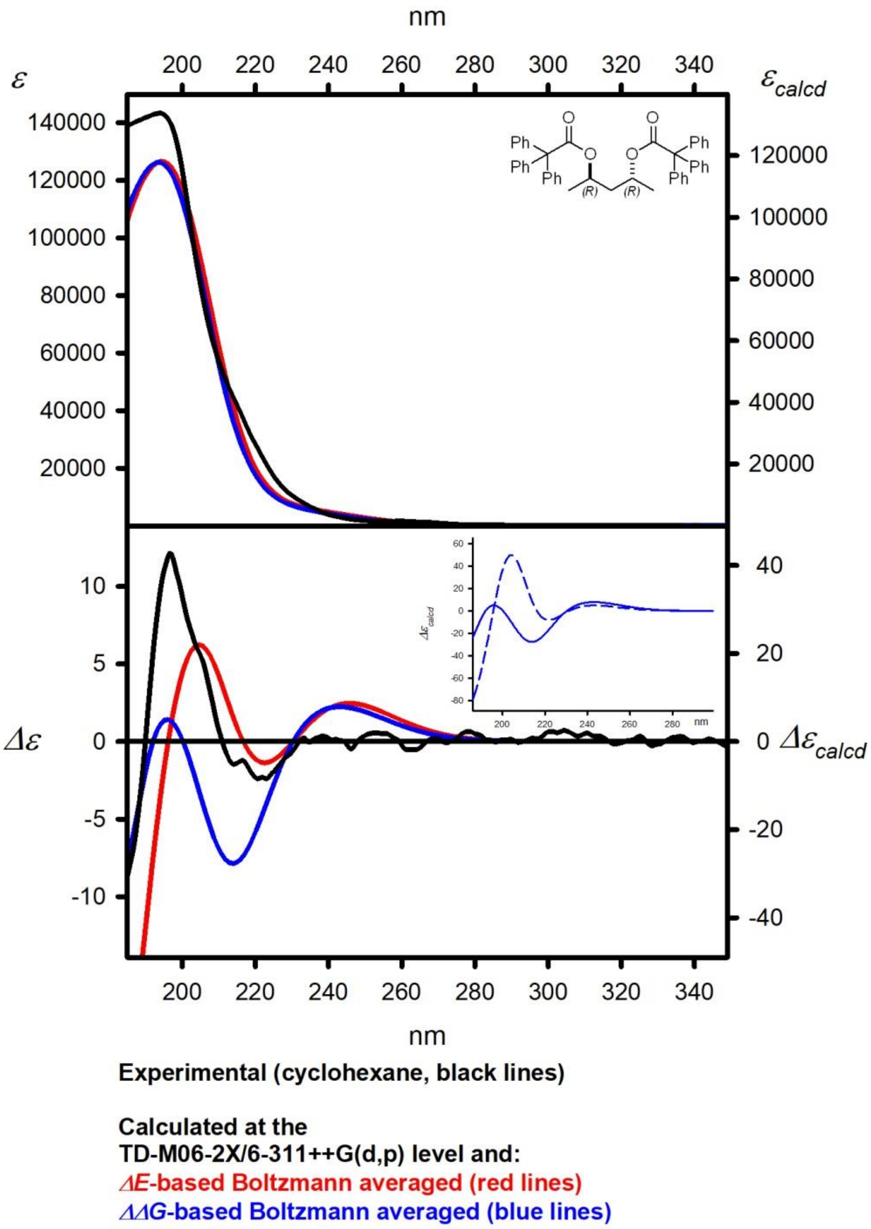

Figure SI_120. UV (upper panel) and ECD (lower panel) spectra of **21**, experimental, measured in cyclohexane (solid black lines) and calculated at the TD-M06-2X/6-311++G(d,p) level for structures optimized at the B3LYP/6-311G(d,p) level, ΔE-based Boltzmann (red lines) and ΔΔG-based Boltzmann averaged (blue solid lines). Insert shows the comparison between Boltzmann averaged ECD spectrum and that calculated for ΔΔG-based on the lowest energy conformer of a given compound (dashed blue line). All calculated spectra were wavelength corrected to match experimental UV maxima.

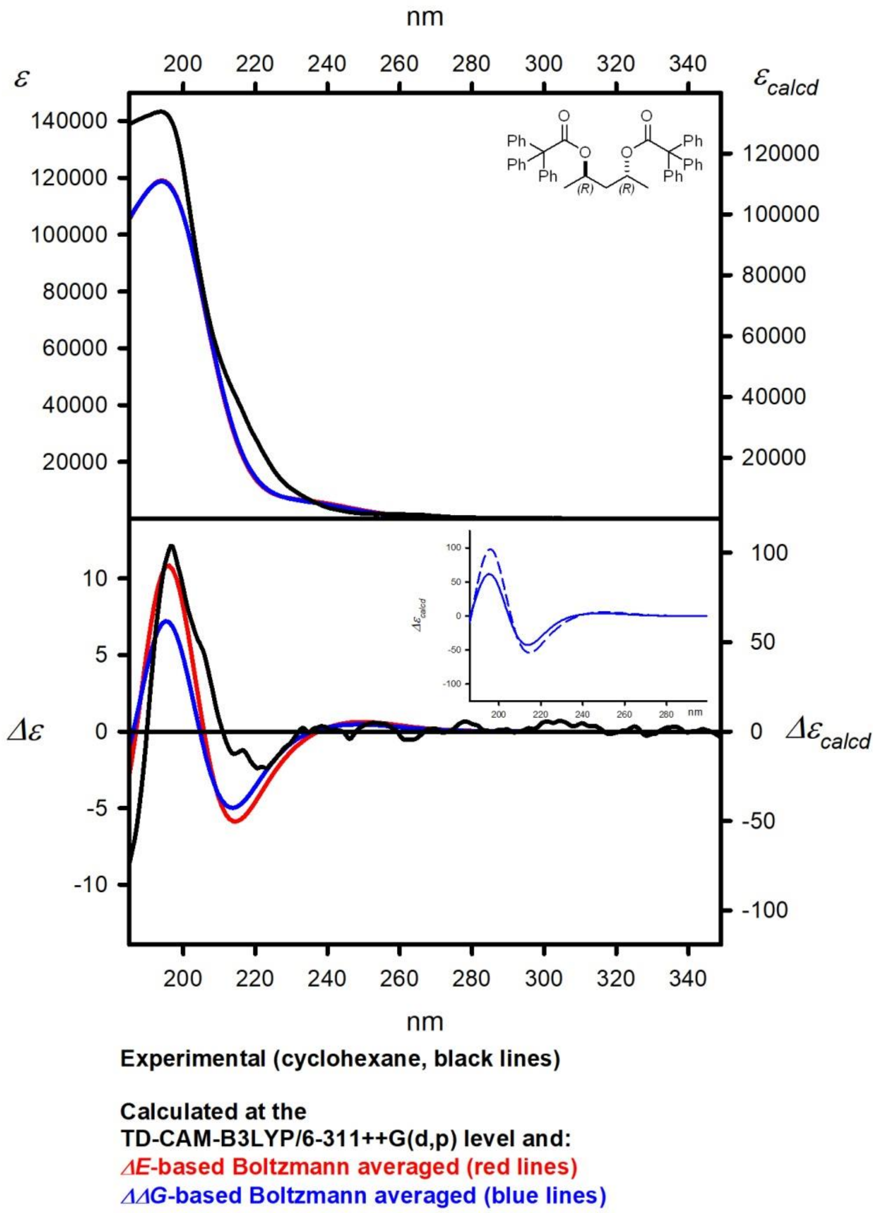

Figure SI_121. UV (upper panel) and ECD (lower panel) spectra of **21**, experimental, measured in cyclohexane (solid black lines) and calculated at the TD-CAM-B3LYP/6-311++G(d,p) level for structures optimized at the B3LYP-GD3BJ/6-311G(d,p) level, ΔE-based Boltzmann (red lines) and ΔΔG-based Boltzmann averaged (blue solid lines). Insert shows the comparison between Boltzmann averaged ECD spectrum and that calculated for ΔΔG-based on the lowest energy conformer of a given compound (dashed blue line). All calculated spectra were wavelength corrected to match experimental UV maxima.

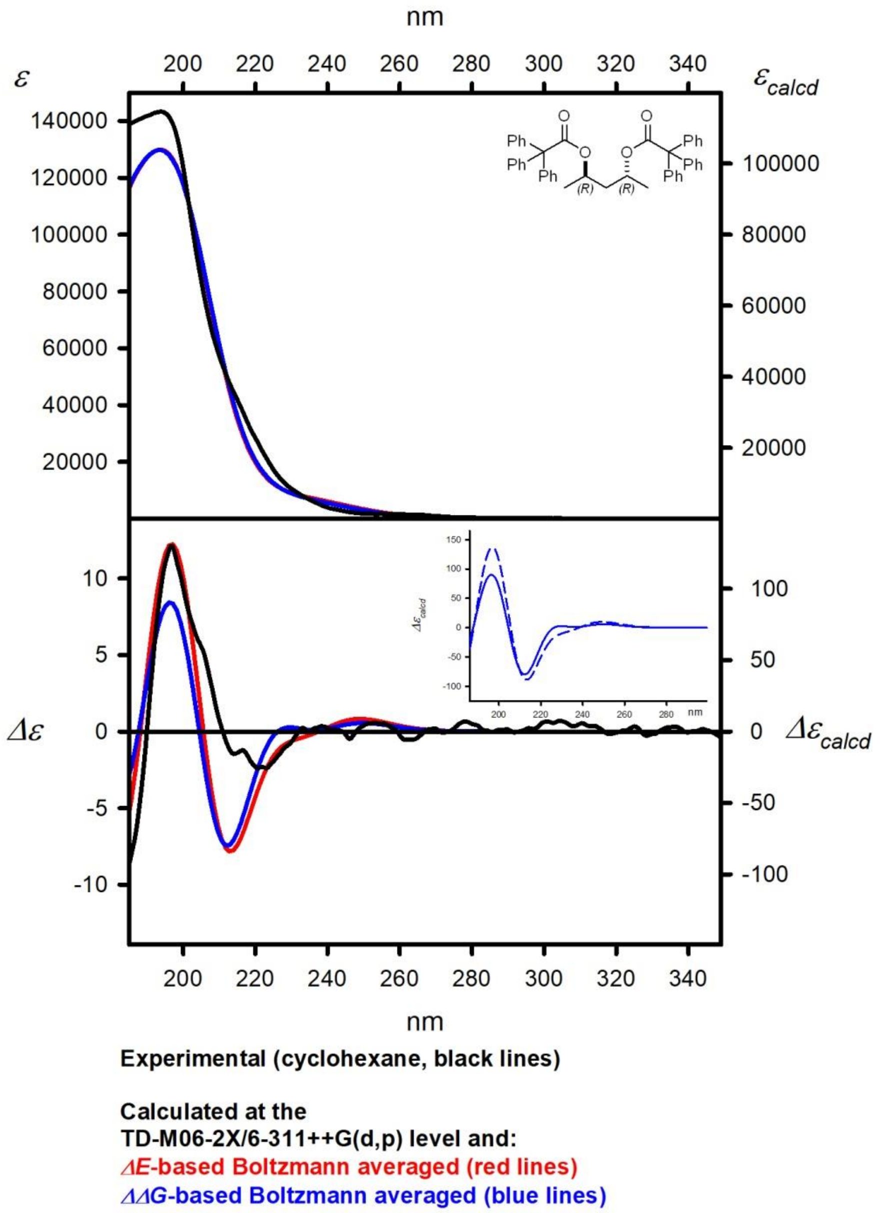

Figure SI_122. UV (upper panel) and ECD (lower panel) spectra of **21**, experimental, measured in cyclohexane (solid black lines) and calculated at the TD-M06-2X/6-311++G(d,p) level for structures optimized at the B3LYP-GD3BJ/6-311G(d,p) level, ΔE-based Boltzmann (red lines) and ΔΔG-based Boltzmann averaged (blue solid lines). Insert shows the comparison between Boltzmann averaged ECD spectrum and that calculated for ΔΔG-based on the lowest energy conformer of a given compound (dashed blue line). All calculated spectra were wavelength corrected to match experimental UV maxima.

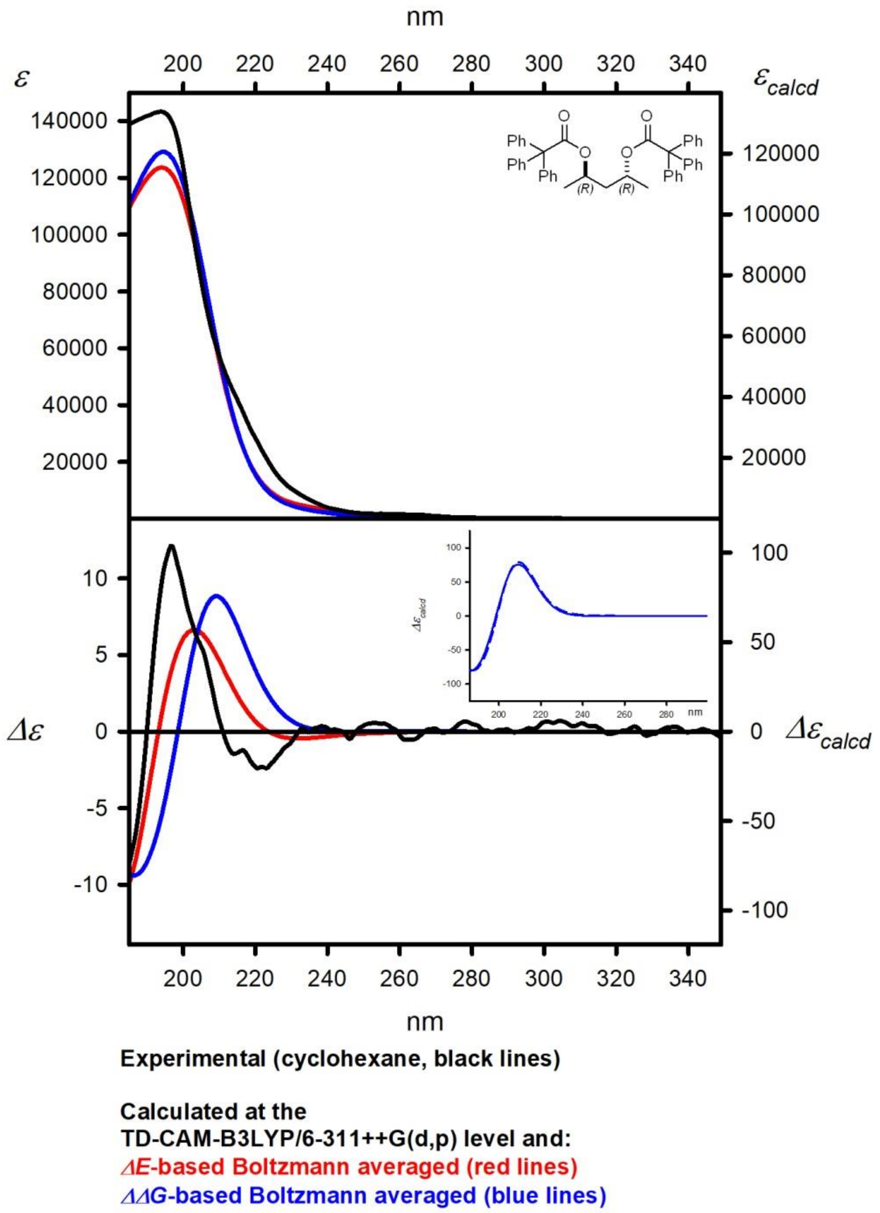

Figure SI_123. UV (upper panel) and ECD (lower panel) spectra of **21**, experimental, measured in cyclohexane (solid black lines) and calculated at the TD-CAM-B3LYP/6-311++G(d,p) level for structures optimized at the M06-2X/6-311G(d,p) level, ΔE-based Boltzmann (red lines) and ΔΔG-based Boltzmann averaged (blue solid lines). Insert shows the comparison between Boltzmann averaged ECD spectrum and that calculated for ΔΔG-based on the lowest energy conformer of a given compound (dashed blue line). All calculated spectra were wavelength corrected to match experimental UV maxima.

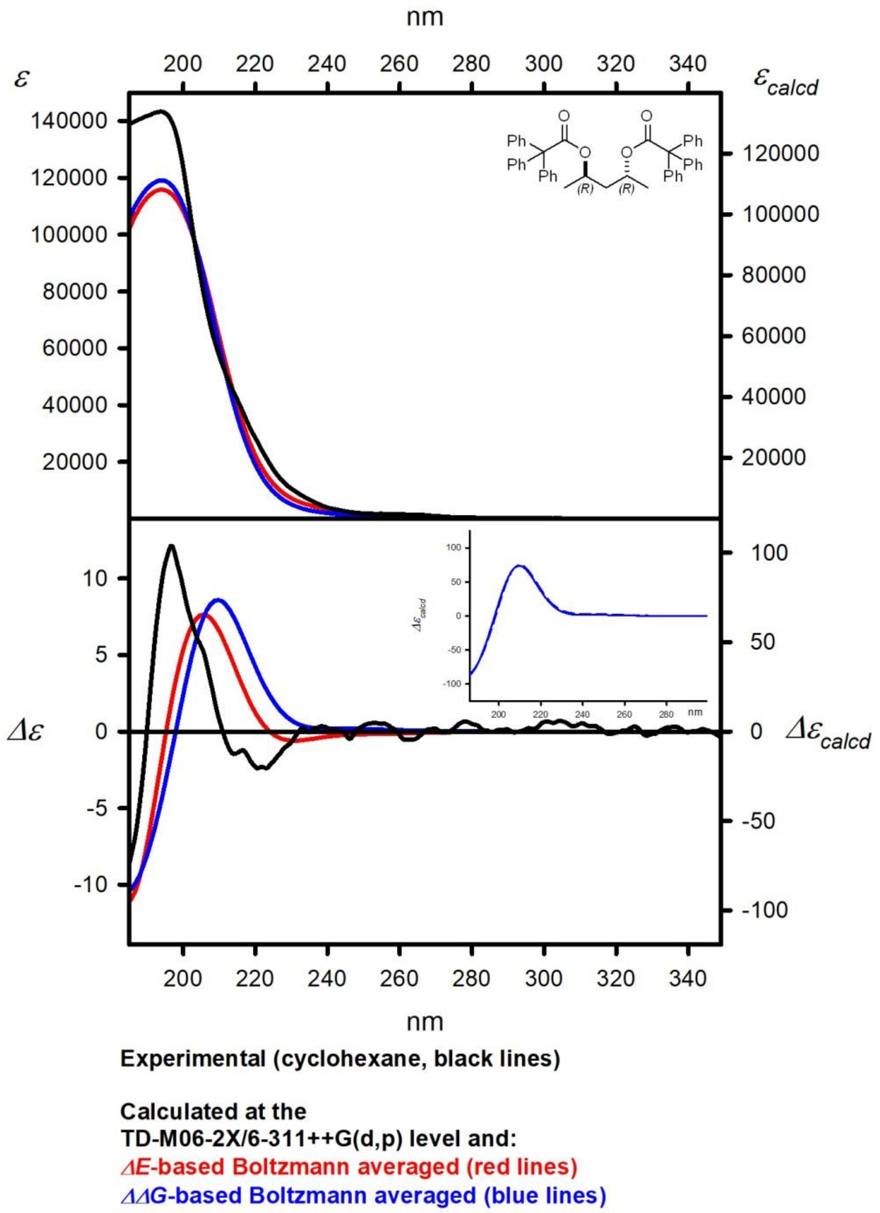

Figure SI_124. UV (upper panel) and ECD (lower panel) spectra of **21**, experimental, measured in cyclohexane (solid black lines) and calculated at the TD-M06-2X/6-311++G(d,p) level for structures optimized at the M06-2X/6-311G(d,p) level, ΔE-based Boltzmann (red lines) and ΔΔG-based Boltzmann averaged (blue solid lines). Insert shows the comparison between Boltzmann averaged ECD spectrum and that calculated for ΔΔG-based on the lowest energy conformer of a given compound (dashed blue line). All calculated spectra were wavelength corrected to match experimental UV maxima.

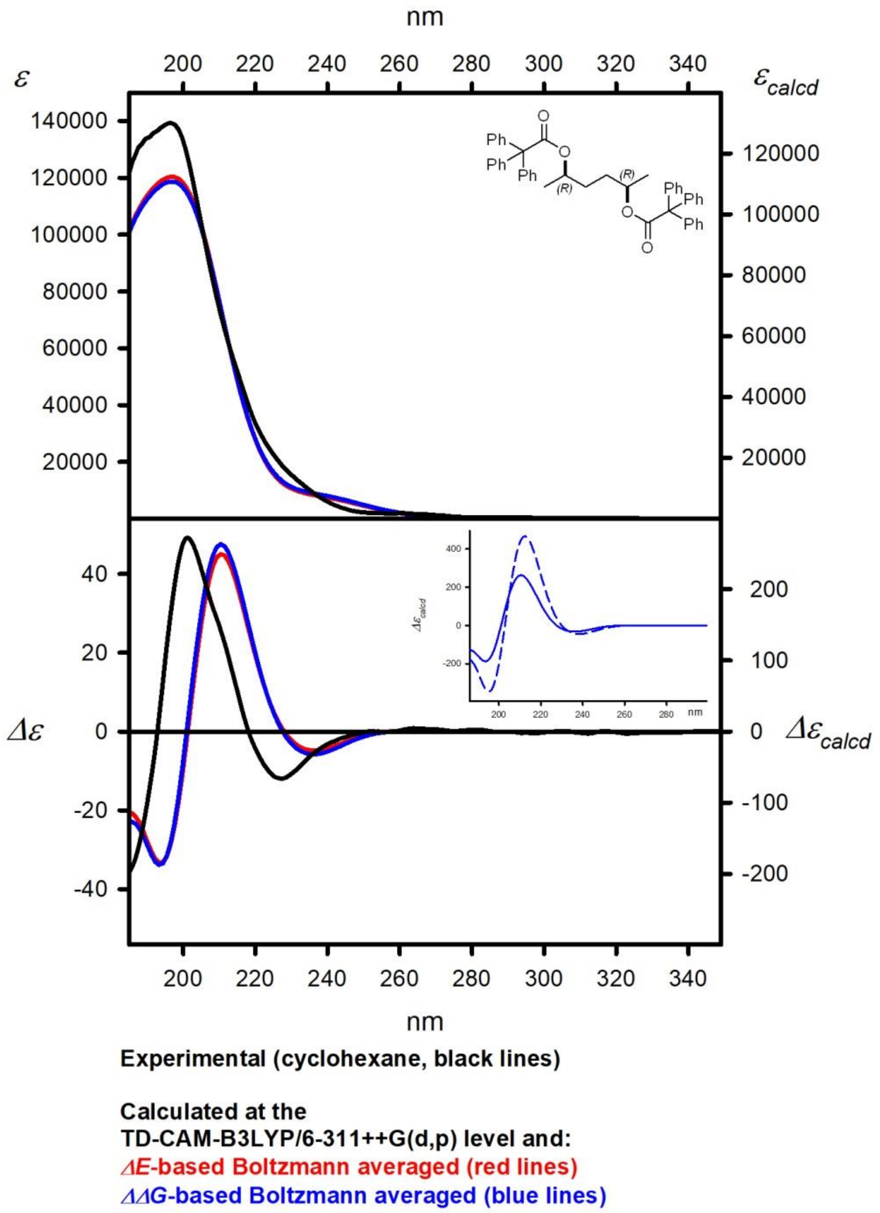

Figure SI_125. UV (upper panel) and ECD (lower panel) spectra of **22**, experimental, measured in cyclohexane (solid black lines) and calculated at the TD-CAM-B3LYP/6-311++G(d,p) level for structures optimized at the B3LYP/6-311G(d,p) level, ΔE-based Boltzmann (red lines) and ΔΔG-based Boltzmann averaged (blue solid lines). Insert shows the comparison between Boltzmann averaged ECD spectrum and that calculated for ΔΔG-based on the lowest energy conformer of a given compound (dashed blue line). All calculated spectra were wavelength corrected to match experimental UV maxima.

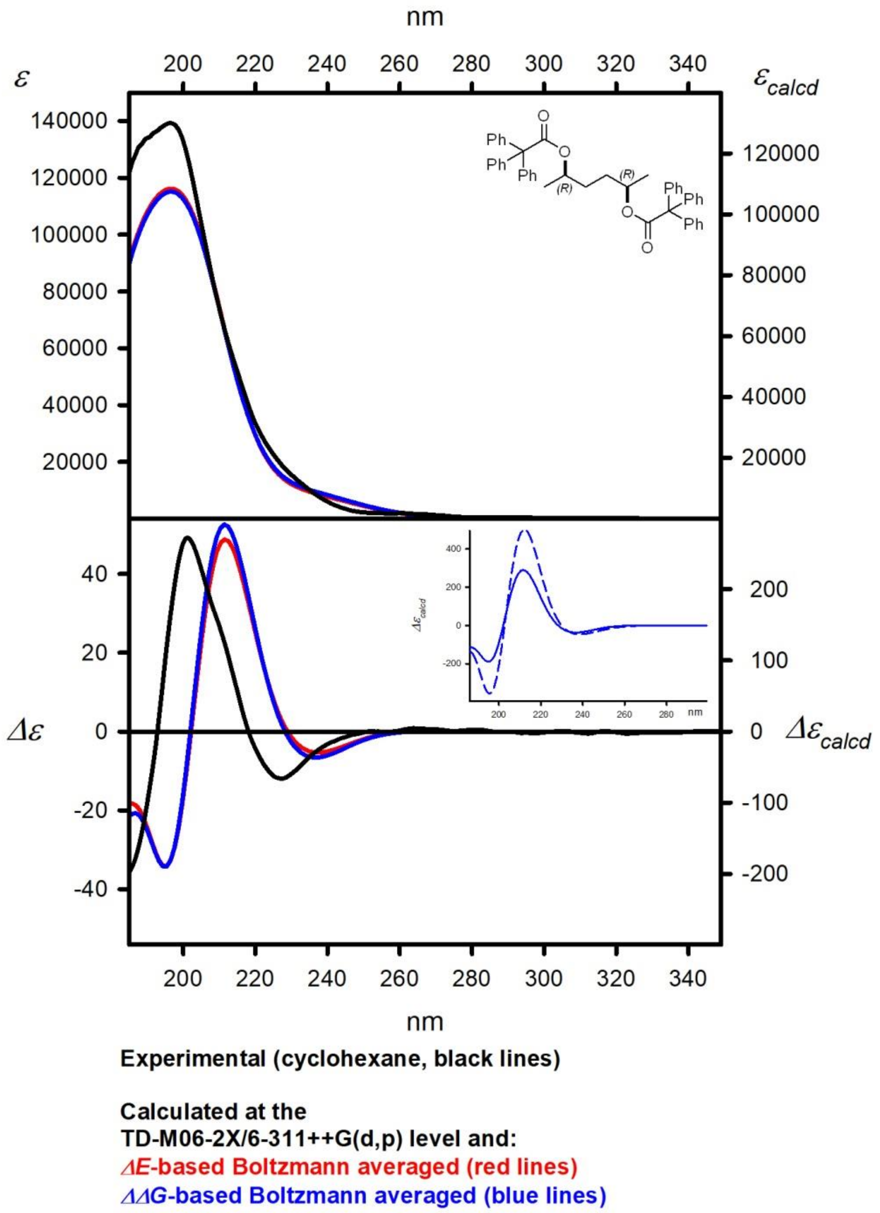

Figure SI_126. UV (upper panel) and ECD (lower panel) spectra of **22**, experimental, measured in cyclohexane (solid black lines) and calculated at the TD-M06-2X/6-311++G(d,p) level for structures optimized at the B3LYP/6-311G(d,p) level, ΔE-based Boltzmann (red lines) and ΔΔG-based Boltzmann averaged (blue solid lines). Insert shows the comparison between Boltzmann averaged ECD spectrum and that calculated for ΔΔG-based on the lowest energy conformer of a given compound (dashed blue line). All calculated spectra were wavelength corrected to match experimental UV maxima.

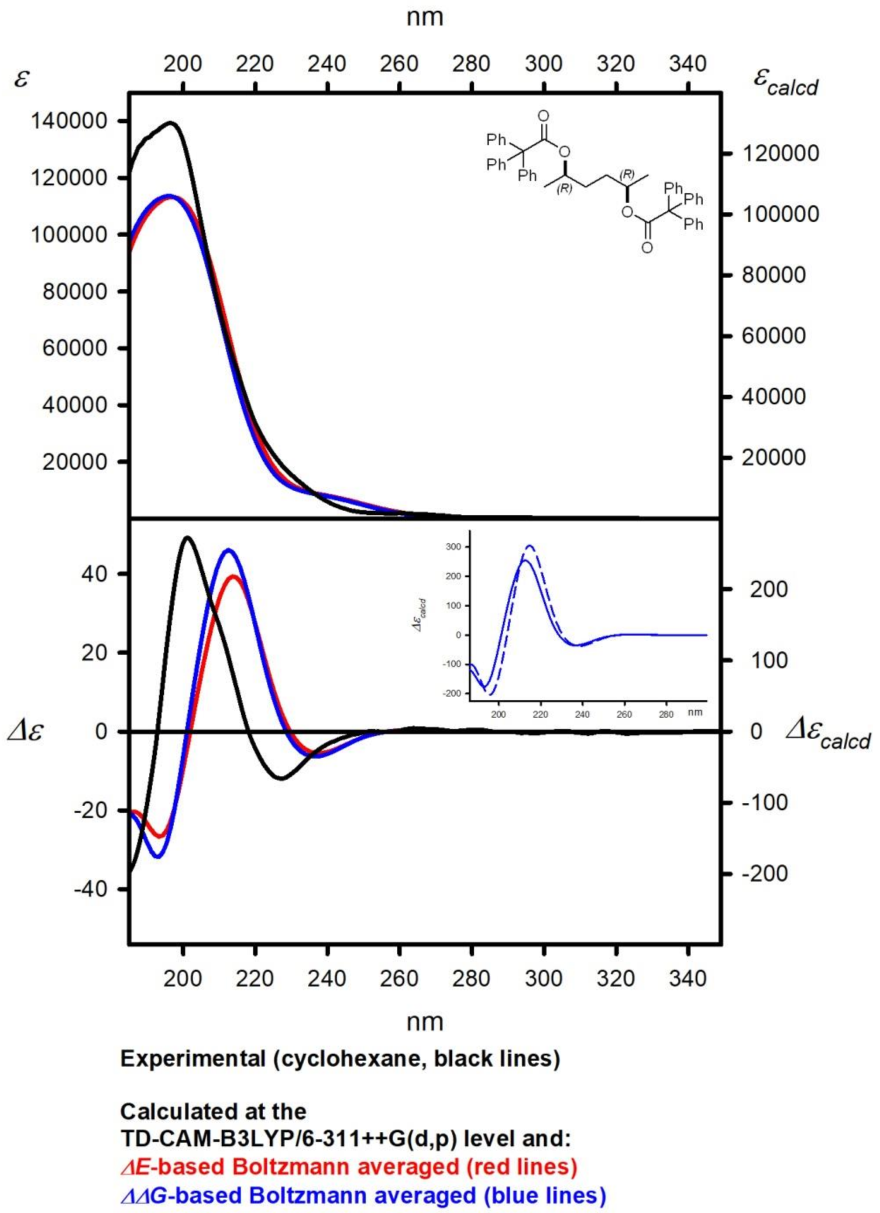

Figure SI_127. UV (upper panel) and ECD (lower panel) spectra of **22**, experimental, measured in cyclohexane (solid black lines) and calculated at the TD-CAM-B3LYP/6-311++G(d,p) level for structures optimized at the B3LYP-GD3BJ/6-311G(d,p) level, ΔE-based Boltzmann (red lines) and ΔΔG-based Boltzmann averaged (blue solid lines). Insert shows the comparison between Boltzmann averaged ECD spectrum and that calculated for ΔΔG-based on the lowest energy conformer of a given compound (dashed blue line). All calculated spectra were wavelength corrected to match experimental UV maxima.

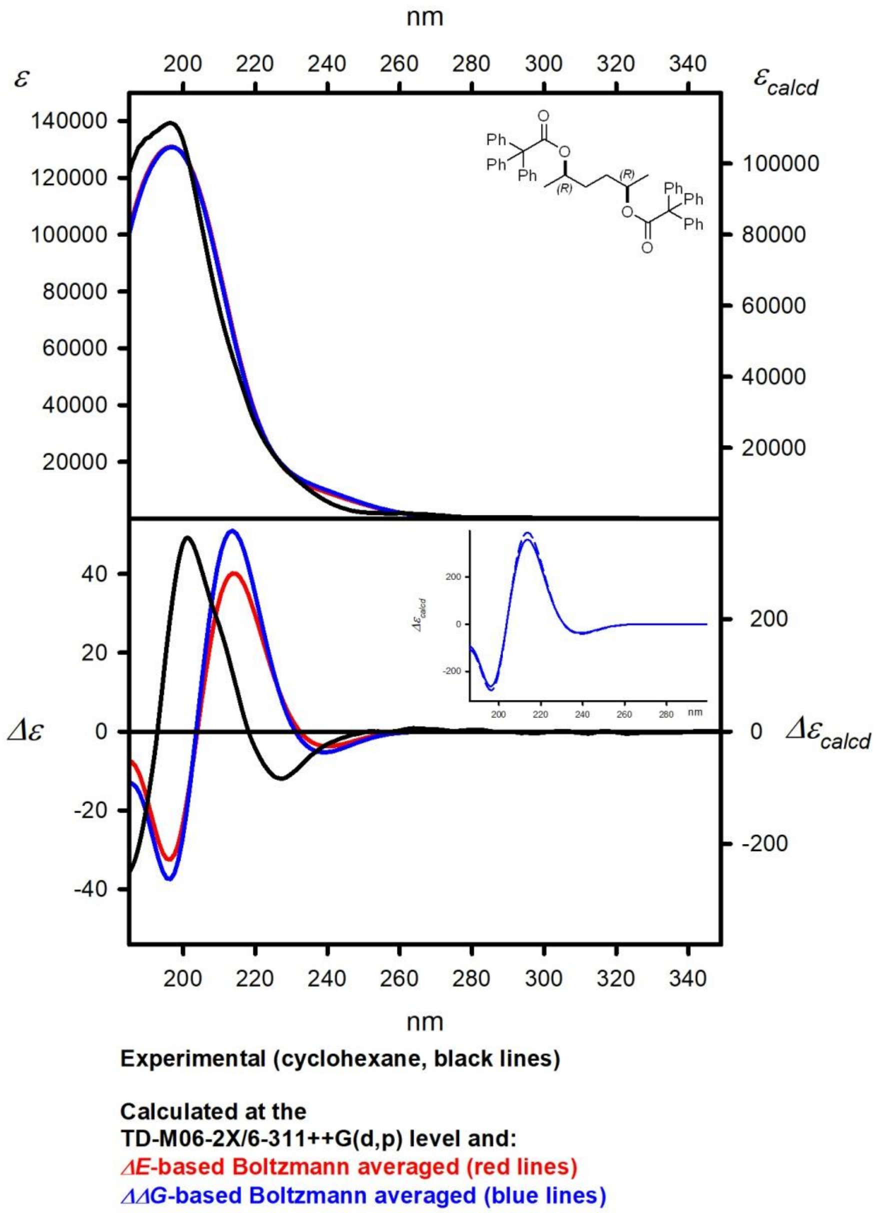

Figure SI_128. UV (upper panel) and ECD (lower panel) spectra of **22**, experimental, measured in cyclohexane (solid black lines) and calculated at the TD-M06-2X/6-311++G(d,p) level for structures optimized at the B3LYP-GD3BJ/6-311G(d,p) level, ΔE-based Boltzmann (red lines) and ΔΔG-based Boltzmann averaged (blue solid lines). Insert shows the comparison between Boltzmann averaged ECD spectrum and that calculated for ΔΔG-based on the lowest energy conformer of a given compound (dashed blue line). All calculated spectra were wavelength corrected to match experimental UV maxima.

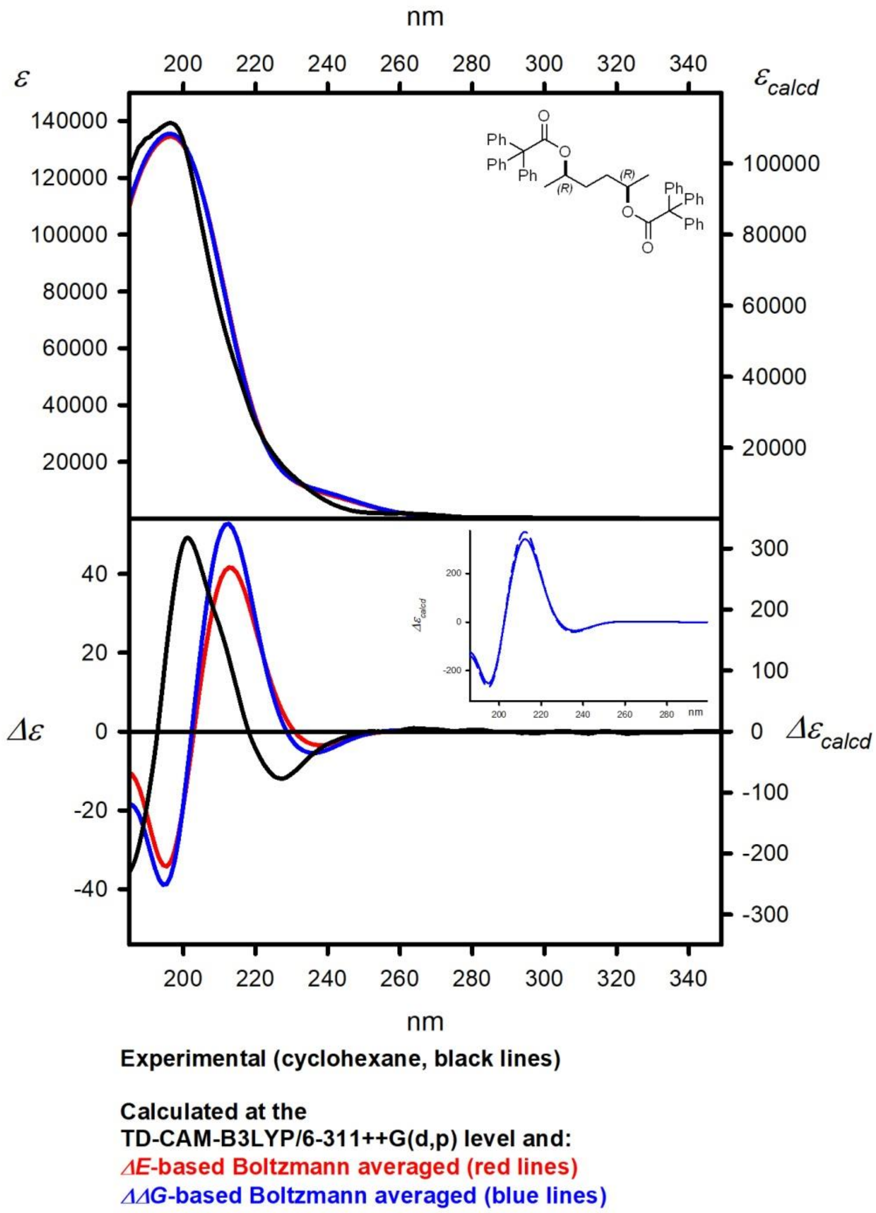

Figure SI_129. UV (upper panel) and ECD (lower panel) spectra of **22**, experimental, measured in cyclohexane (solid black lines) and calculated at the TD-CAM-B3LYP/6-311++G(d,p) level for structures optimized at the M06-2X/6-311G(d,p) level, ΔE-based Boltzmann (red lines) and ΔΔG-based Boltzmann averaged (blue solid lines). Insert shows the comparison between Boltzmann averaged ECD spectrum and that calculated for ΔΔG-based on the lowest energy conformer of a given compound (dashed blue line). All calculated spectra were wavelength corrected to match experimental UV maxima.

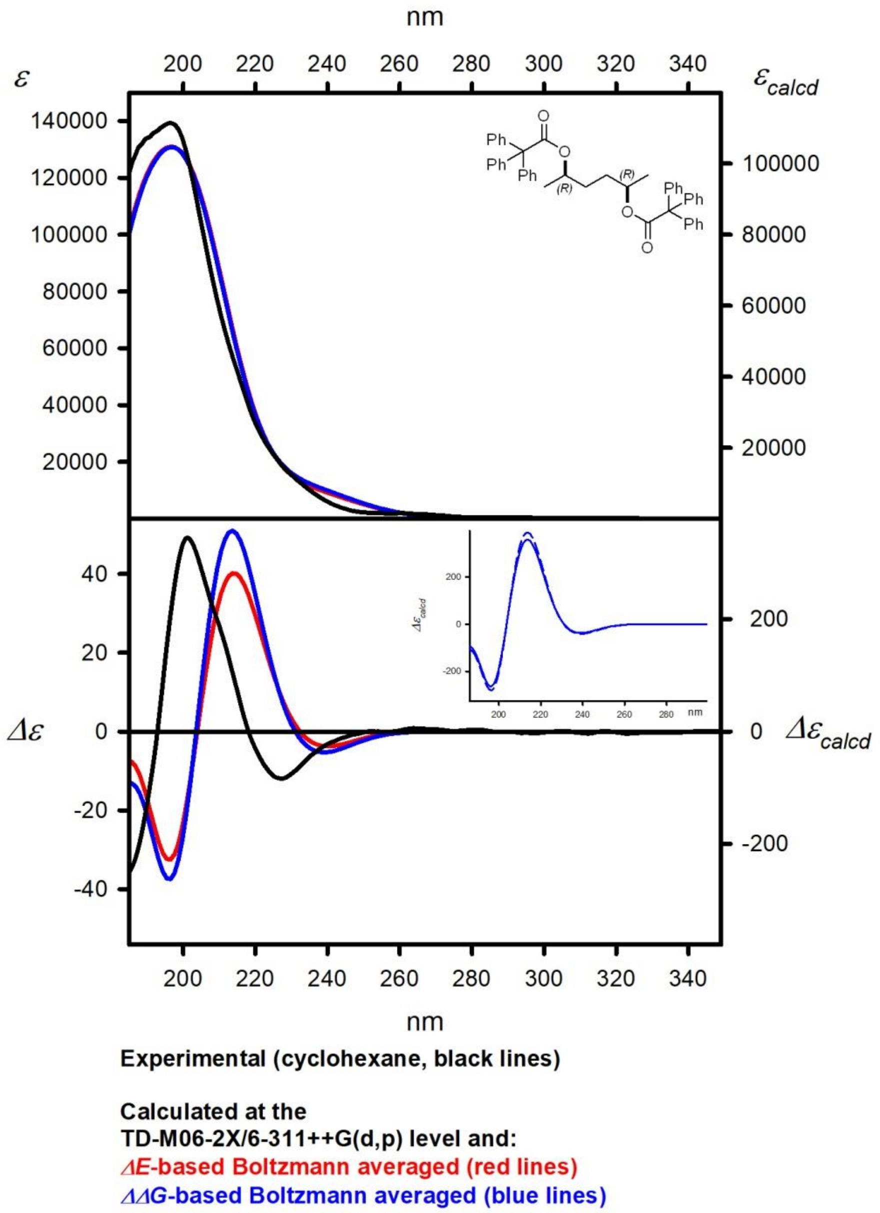

Figure SI_130. UV (upper panel) and ECD (lower panel) spectra of **22**, experimental, measured in cyclohexane (solid black lines) and calculated at the TD-M06-2X/6-311++G(d,p) level for structures optimized at the M06-2X/6-311G(d,p) level, ΔE-based Boltzmann (red lines) and ΔΔG-based Boltzmann averaged (blue solid lines). Insert shows the comparison between Boltzmann averaged ECD spectrum and that calculated for ΔΔG-based on the lowest energy conformer of a given compound (dashed blue line). All calculated spectra were wavelength corrected to match experimental UV maxima.

Single crystal X-ray analysis

The colourless single crystals of compounds **1**, **5**, **10**, **11**, **18**-**21** suitable for X-ray structural analysis were obtained by slow evaporation of solvent (dichloromethane, *n*-hexane or mixture thereof). The diffraction data were collected at 130K with an Oxford Diffraction SuperNova diffractometer (Oxford Diffraction), using Cu Kα radiation (λ = 1.54184 Å) equipped with a mirror monochromator and CCD detector (Atlas). The intensity data were collected and processed using the CrysAlisPro software.[9] The structures were solved by direct methods with the program SHELXT 2018/2 [10] and refined by full-matrix least-squares method on F^2 with SHELXL 2018/3.[11] The carbon-bound hydrogen atoms were refined as riding on their carriers and their displacement parameters were set equal to 1.5Ueq(C) for the methyl groups and 1.2Ueq(C) for the remaining H atoms. Absolute structures of the compounds were specified by the synthetic procedure and confirmed using Flack parameter.[12]

A powder pattern of compound **5** was registered with a four-circle SuperNova diffractometer (Oxford Diffraction) using Cu Kα radiation (λ=1.54184 Å), equipped with a mirror monochromator and CCD detector (Atlas). A 0.3 mm pinhole collimator was used and the detector was set at 70 mm from the sample. The exposure time was fixed to 300 second per scan and images were collected with a 360 degrees phi rotation. The powder patterns of polymorph **5**-α and **5**-*β* for comparison were calculated with Mercury 4.3.0 software.[13]

Selected geometrical data are given in **Table SI_54**. A summary of the crystallographic data is given in **Table SI_55**. Molecular graphics were generated with Olex2[14] and Mercury 4.3.0 software. The analysis of intermolecular interactions was carried out using the Crystal Explorer.[15] **Figure SI_131** presents the contribution to the Hirshfeld surface area for various intermolecular interactions for molecules in the studied crystal structures.

In crystal structure of **1**-*rac* molecule is slightly disordered in 2-butyl part. The site occupancy factors for the alkyl chains are 0.78 and 0.22. (**Figure SI_132b**). For disordered part the following restrains was used: RIGU O1 C2A C3A C4A C5A and RIGU C2 C3 C4 C5.

In refinement process of crystal structure of **11** four reflection have been omitted: 7 -4 4, 8 -2 3, 7 -3 4, 7 -2 4.

The molecule in crystal structure of **18** lies on the two-fold axis passing through the C_{sp2}-C_{sp2} bond. it is impossible to locate the methyl group unambiguously and in the adopted model it is equally likely attached to the C2 or C2' (**Figure SI_137**). It is similar in the crystal of **19**. The methyl group is equally likely attached to the C2 and C4 atom. The molecules in crystal structure are arranged in columns and the additional disorder is the result of the molecule shifting in the column by half its length (**Figure SI_138)**. The site occupancy factors for molecules are 0.85 and 0.15. For disordered part the following restrains was used: DFIX 1.54 0.005 C2B C6B, SIMU 0.04 0.08 1.7 C11B C12B C13B C14B C15B C16B, SIMU 0.04 0.08 1.7 C41B C42B C43B C44B C45B C46B, SIMU 0.04 0.08 1.7 C51B C52B C53B C54B C55B C56B, SIMU 0.04 0.08 1.7 C61B C62B C63B C64B C65B C66B, SIMU 0.04 0.08 1.7 C31B C32B C33B C34B C35B C36B, SIMU 0.04 0.08 1.7 C21B C22B C23B C24B C25B C26B.

Table SI_54. Dihedral angles *α*, *β*, *γ*, *δ* and ζ (in degrees), selected interatomic distances l_1, l_2 (in Å) and helicities of trityl groups observed in the crystal structures of compounds **1**, **5**, **10**, **11**, **18**-**21**.

Compound		*α*[a]	*β1*[b]	*β2*[b]	*β3*[b]	*γ1*[c]	*γ2*[c]	*γ3*[c]	*δ*[d]	*ζ*[e]	*l1*[f]	*l2*[g]	Helicity
**1**	mol A	175.2 (2)	-3.1 (3)	116.9 (2)	-121.3 (2)	61.0 (2)	42.6 (3)	45.2 (3)	-16.6	178.1 (2)	2.68	2.22	*PPP*
	mol B	175.6 (2)	7.9 (3)	127.9 (2)	-111.4 (2)	-63.1 (2)	-47.5 (3)	-33.9 (3)	33.4	-65.6 (3)	2.68	2.28	*MMM*
	mol R	172.8 (2)							-16.9	177.1 (2)			
(*rac*)-**1**			-6.5 (2)	113.6 (1)	-125.7 (1)	61.9 (1)	42.6 (2)	46.4 (1)			2.67	2.24	*PPP*
	mol S	-166.5 (5)							-42.4	55.0 (12)			
**5**-α	mol A	-175.0 (2)	20.0 (3)	141.2 (2)	-97.5 (2)	-59.6 (2)	-49.0 (3)	-26.0 (3)	32.2	20.6 (3)	2.57	2.37	*MMM*
	mol B	177.6 (2)	-12.4 (3)	107.7 (2)	-131.8 (2)	58.1 (2)	48.9 (3)	40.6 (3)	-34.0	41.3 (3)	2.54	2.31	*PPP*
**5**-β	mol A	-178.1 (2)	19.7 (3)	140.8 (2)	-98.8 (3)	-54.1 (2)	-45.2 (3)	-30.5 (3)	53.0	53.4 (3)	2.44	2.30	*MMM*
	mol B	175.6 (2)	-1.9 (3)	116.3 (2)	-122.6 (2)	-59.0 (2)	-40.4 (3)	-54.5 (3)	30.9	69.5 (3)	2.65	2.27	*MMM*
(*rac*)-**5**		-174.0 (1)	2.95 (13)	124.7 (0)	-115.2 (1)	53.3 (1)	57.2 (1)	44.2 (1)	-37.9	-47.6 (1)	2.57	2.28	*PPP*
**10**		-179.4 (1)	-10.2 (2)	108.0 (2)	-129.8 (2)	60.3 (2)	9.3 (2)	49.6 (2)	40.1	-176.5 (1)	2.59	2.42	*PPP*
**11**		-174.6 (2)	-63.7 (3)	55.8 (3)	176.3 (2)	-54.8 (3)	-23.9 (3)	-58.7 (3)	-7.8	63.3 (3)	2.22	2.78	*MMM*
**18**		173.0 (3)	4.3 (5)	122.4 (4)	-115.4 (4)	-58.2 (4)	-42.2 (4)	-26.6 (4)	25.2	136.9 (4)	2.59	2.24	*MMM*
**19**	mol **A**	172.3 (2	127.5 (3)	-110.5 (3)	8.6 (3)	-44.9 (3)	-17.8 (4)	-58.4 (3)	45.3	-58.0 (2)	2.58	2.33	*MMM*
	*	175.9 (2)	138.3 (2)	-99.4 (3)	19.8 (4)	-41.8 (3)	-33.4 (5)	-62.2 (4)	48.7	-58.6 (2)	2.57	2.34	*MMM*
	mol **B**	177 (2)	134 (2)	-90 (3)	15 (4)	-34 (2)	-39 (2)	-51 (3)	45.3	-61.2 (17)	2.46	2.31	*MMM*
		173.2 (15)	132 (2)	-99 (2)	12 (3)	-39.2 (18)	-29 (2)	-54.4 (19)	52.1	-59.6 (17)	2.58	2.43	*MMM*
**20**	mol **A**	-176.3 (3)	-6.1 (5)	112.4 (4)	-126.0 (4)	63.2 (4)	18.5 (4)	48.5 (4)	39.1	-51.3 (4)	3.08	2.32	*PPP*
		176.8 (3)	2.3 (5)	123.0 (4)	-116.4 (4)	52.7 (4)	51.3 (4)	48.1 (4)	41.4	-54.0 (4)	2.50	2.25	*PPP*
	mol **B**	160.1 (3)	-0.6 (4)	118.7 (4)	-118.9 (3)	-65.4 (4)	-50.3 (4)	-1.8 (4)	-13.2	-61.4 (4)	2.90	2.38	*MMM*
		174.8 (3)	1.3 (5)	121.9 (4)	−117.5 (4)	−54.7 (4)	−52.4 (4)	−39.4 (5)	-21.0	-60.3 (4)	2.50	2.21	*MMM*

mol **C**	173.3 (3)	4.6 (5)	123.9 (4)	-115.5 (4)	52.7 (4)	53.1 (4)	46.8 (4)	38.8	-53.3 (4)	2.51	2.23	*PPP*
	-174.9 (3)	-2.1 (4)	115.8 (4)	-122.2 (3)	64.7 (4)	12.2 (4)	49.0 (4)	41.4	-51.3 (4)	2.78	2.34	*PPP*
mol **D**	172.4 (3)	1.2 (5)	120.7 (4)	-118.8 (4)	−53.8 (4)	−53.1 (4)	−38.9 (4)	-19.8	-61.0 (4)	2.52	2.22	*MMM*
	159.4 (3)	0.8 (5)	120.0 (4)	-118.1 (4)	-64.7 (4)	-49.6 (4)	-7.6 (4)	-9.2	-61.2 (4)	2.83	2.35	*MMM*
**21**	174.8 (2)	19.0 (3)	139.3 (2)	-99.7 (2)	-59.9 (2)	-47.1 (2)	-23.1 (3)	46.7	-61.2 (1)	2.56	2.38	*MMM*

[a]– α = C_{Tr}-C(=O)-O-C*; [b] – β =O=C-C-C_{ipso}; [c] – γ = (O=)C-C_{Tr}-C_{ipso}-C_{ortho} (of the two possibilities the absolute values ≤90° has been chosen); [d] – δ = C(=O)-O-C*-H; [e] – ζ = O-C*-C-C(*); [f] – l_1 = C=O···HC_{ortho}; [g] – l_2 = (O=)CO···HC_{ortho}.

Table SI_55. Selected crystal data and structure refinement details for **1**, **5**, **10**, **11**, **18**-**21**.

	**1**	(*rac*)-**1**	**5**-α	**5**-β	(*rac*)-**5**	**10**	**11**	**18**	**19**	**20**	**21**
Chemical formula	$C_{24}H_{24}O_2$	$C_{24}H_{24}O_2$	$C_{28}H_{24}O_2$	$C_{28}H_{24}O_2$	$C_{28}H_{24}O_2$	$C_{30}H_{34}O_2$	$C_{30}H_{34}O_2$	$C_{43}H_{36}O_4$	$C_{44}H_{38}O_4$	$C_{44}H_{38}O_4$	$C_{45}H_{40}O_4$
Mr	344.43	344.43	392.47	392.47	392.47	426.57	426.57	616.72	630.74	630.74	644.77
Crystal system, space group	Monoclinic, $P2_1$	Monoclinic, $P2_1/c$	Monoclinic, $P2_1$	Monoclinic, $P2_1$	Monoclinic, $P2_1/c$	Monoclinic, $P2_1$	Monoclinic, $P2_1$	Monoclinic, $C2$	Monoclinic, $P2_1$	Monoclinic, $P2$	Monoclinic, $C2$
Temperature (K)	130	130	130	130	130	130	130	130	130	130	130
a, *b*, *c* (Å)	8.6848 (1), 13.1859 (1), 16.7979 (1)	8.7143 (2), 13.1260 (3), 16.7238 (3)	8.56847 (8), 27.5418 (3), 9.4209 (1)	8.76286 (12), 17.3973 (3), 14.2707 (2)	15.90336 (5), 8.05841 (3), 16.20465 (6)	8.81491 (6), 15.42044 (11), 9.11196 (8)	8.5973 (2), 16.5110 (4), 9.2226 (3)	15.8708 (8), 7.9327 (2), 14.1241 (7)	14.2775 (1), 8.0436 (1), 14.4025 (2)	20.0936 (2), 9.9377 (1), 34.7742 (3)	16.3482 (4), 8.2085 (1), 14.2443 (4)
β (°)	99.848 (1)	99.740 (2)	100.4459 (10)	101.8322 (12)	90.0241 (3)	93.3367 (7)	113.412 (3)	114.416 (6)	91.020 (1)	103.090 (1)	117.606 (3)
V (Å^3)	1895.30 (3)	1885.36 (7)	2186.40 (4)	2129.34 (5)	2076.72 (1)	1236.49 (2)	1201.37 (6)	1619.17 (14)	1653.76 (3)	6763.42 (12)	1693.89 (8)
Z	4	4	4	4	4	2	2	2	2	8	2
D_x (Mg m^{-3})	1.207	1.213	1.192	1.243	1.255	1.146	1.179	1.265	1.267	1.239	1.264
Radiation type	Cu *Kα*	Cu *Kα*	Cu *Kα*	Cu *Kα*	Cu *Kα*	Cu *Kα*	Cu *Kα*	Cu *Kα*	Cu *Kα*	Cu *Kα*	Cu *Kα*
μ (mm^{-1})	0.59	0.59	0.57	0.59	0.61	0.54	0.55	0.63	0.63	0.62	0.62
Crystal size (mm)	0.24 × 0.10 × 0.05	0.50 × 0.07 × 0.06	0.20 × 0.07 × 0.02	0.16 × 0.10 × 0.03	0.4 × 0.3 × 0.07	0.45 × 0.34 × 0.05	0.23 × 0.20 × 0.02	0.45 × 0.10 × 0.02	0.30 × 0.20 × 0.03	0.55 × 0.35 × 0.15	0.40 × 0.16 × 0.02
No. of measured, independent and observed [$I > 2\sigma(I)$] reflections	28779, 7191, 6962	13757, 3711, 3358	23089, 7327, 6906	21638, 7691, 7164	61948, 4368, 4197	13582, 4521, 4463	17587, 4553, 4296	11856, 3082, 2881	47791, 6267, 6126	112063, 25552, 24266	11151, 3485, 3374
R_{int}	0.027	0.036	0.030	0.036	0.023	0.023	0.052	0.046	0.036	0.057	0.029
$R[F^2 > 2\sigma(F^2)]$, $wR(F^2)$, S	0.036, 0.095, 1.04	0.043, 0.108, 1.07	0.031, 0.075, 1.05	0.034, 0.080, 1.04	0.034, 0.085, 1.05	0.029, 0.076, 1.07	0.040, 0.110, 1.08	0.047, 0.128, 1.10	0.039, 0.110, 1.05	0.082, 0.217, 1.05	0.033, 0.092, 1.09
No. of parameters	473	277	543	543	272	292	292	218	577	1737	223
Δ>$_{max}$, Δ>$_{min}$ (e Å^{-3})	0.24, -0.27	0.29, -0.18	0.14, -0.14	0.16, -0.13	0.27, -0.20	0.18, -0.16	0.18, -0.14	0.16, -0.21	0.24, -0.22	0.87, -0.37	0.20, -0.17
Absolute structure parameter	0.03 (6)	-	-0.05 (9)	-0.06 (11)	-	0.01 (8)	0.24 (19)	0.0 (2)	0.00 (6)	-0.04 (8)	-0.06 (11)

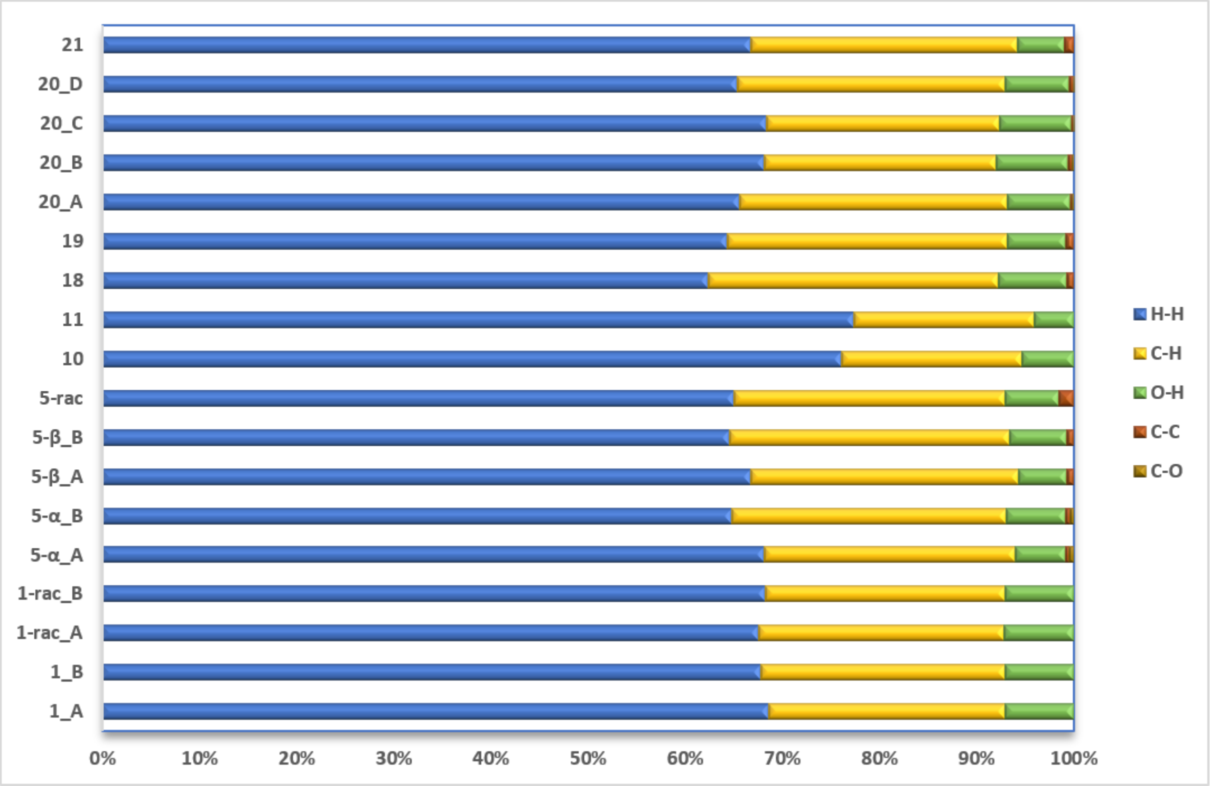

Figure SI_131. Percentage contributions of various intermolecular interaction to the Hirshfeld surfaces of molecules in crystal structure.

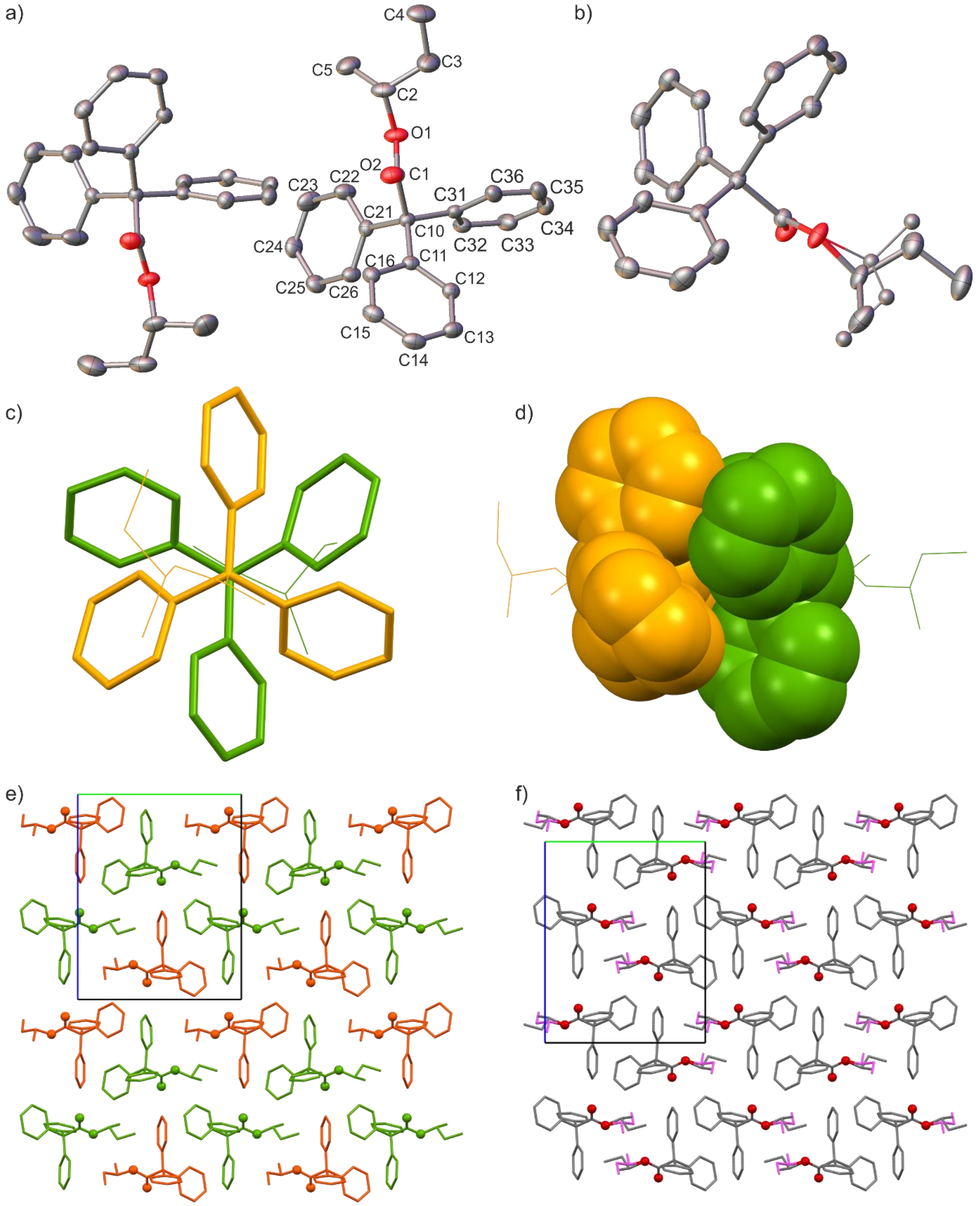

Figure SI_132. Molecular structure of compound a) **1** and b) (*rac*)-**1** (2-butyl substituent is disordered and the minor position showed as thinner lines). Atoms numbering scheme presented for one molecule. Displacement ellipsoids are drawn at 50% probability level. The sixfold phenyl embrace supramolecular synthon in the crystal structure of **1** c) top view and d) side view. Comparison of molecular packing in isostructural crystals of compound e) **1** (symmetrically independent molecules are indicated with different colors) and f) (*rac*)-**1** (minor position of 2-butyl substituent shown as pink). Hydrogen atoms are omitted for clarity and oxygen atoms are shown as balls.

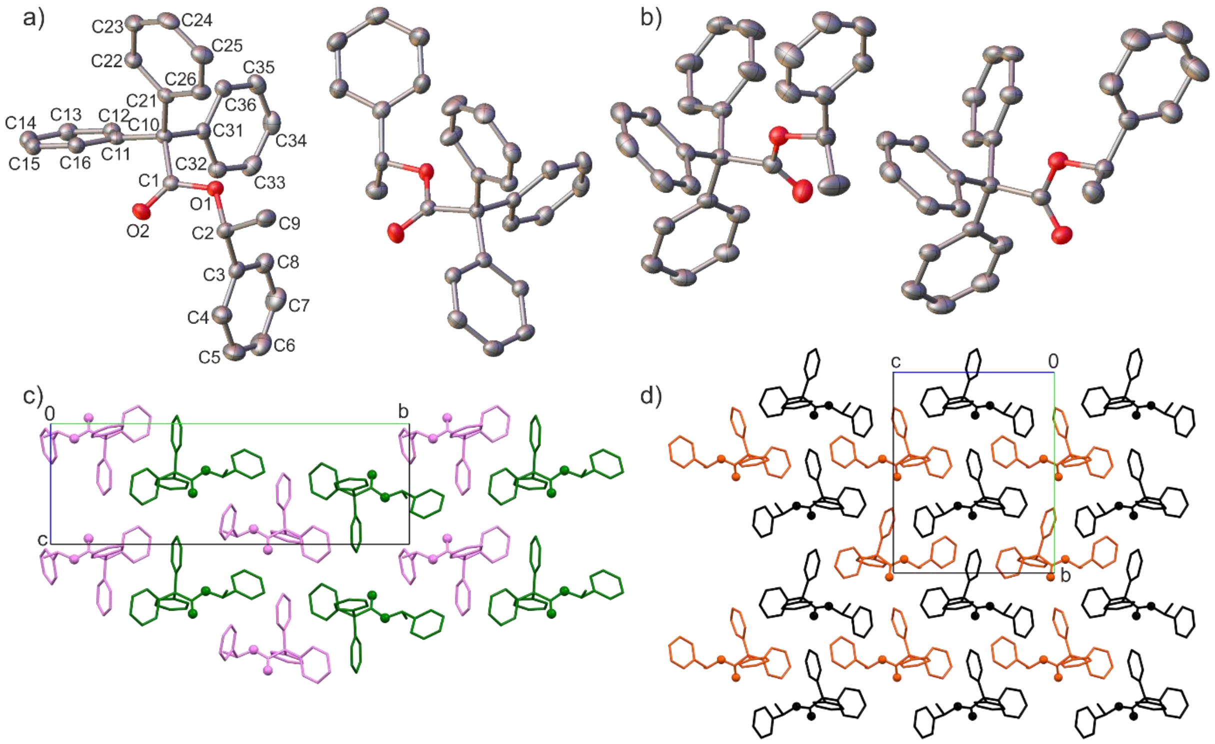

Figure SI_133. Molecular structure of asymmetric unit of a) **5**-*α* and b) **5**-*β*. Atoms numbering scheme presented for one molecule. Displacement ellipsoids are drawn at 50% probability level. Comparison of molecular packing in the crystals of c) **5**-*α* and d) **5**-*β*. Symmetrically independent molecules are indicated with different colors. Hydrogen atoms are omitted for clarity and oxygen atoms are shown as balls.

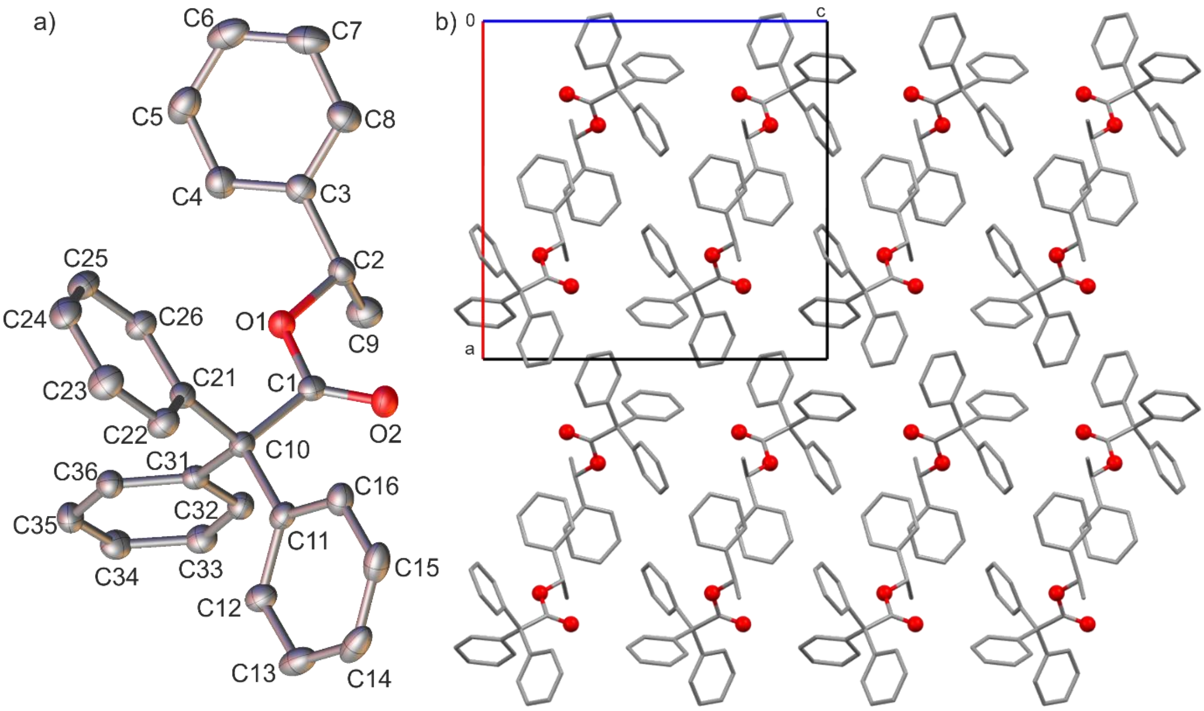

Figure SI_134. a) Molecular structure with atoms numbering scheme and b) molecular packing in crystal of (*rac*)-**5**. Displacement ellipsoids are drawn at 50% probability level. Hydrogen atoms are omitted for clarity and oxygen atoms are shown as balls.

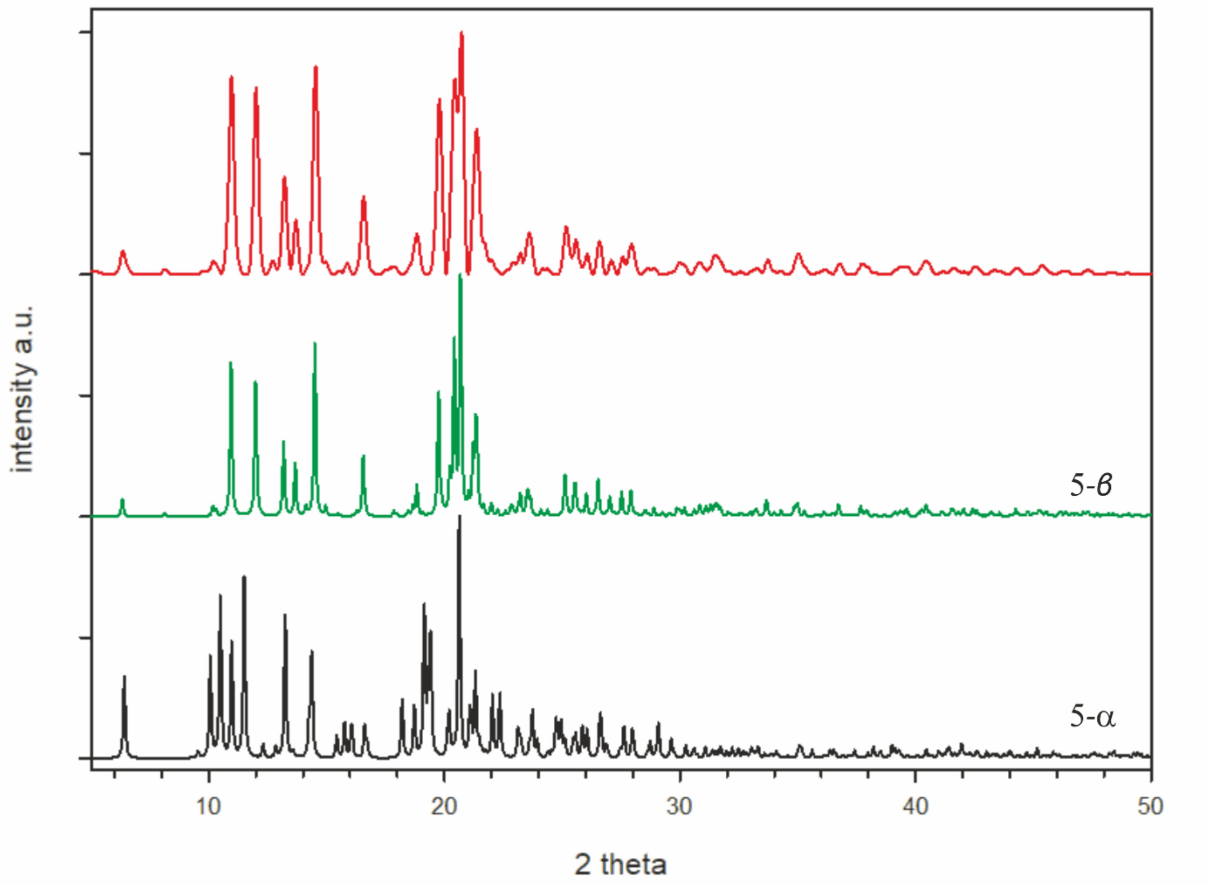

Figure SI_135. The recorded X-ray powder diffraction pattern of compound **5** (red) and predicted pattern based on single-crystal X-ray diffraction indices of polymorphs **5**-*α* and **5**-*β*.

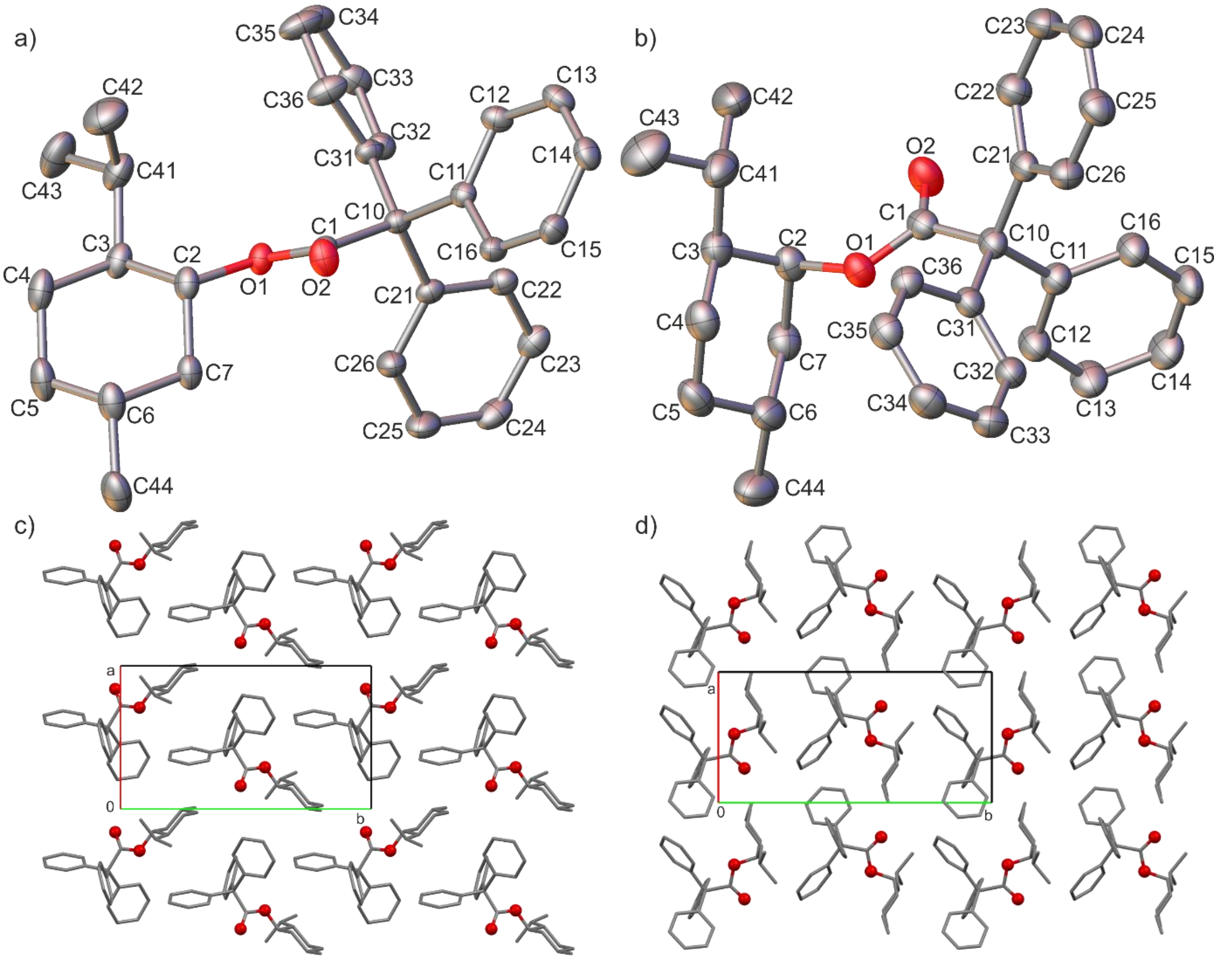

Figure SI_136. Molecular structure and atoms numbering scheme of compound a) **10** and b) **11**. Displacement ellipsoids are drawn at 50% probability level. Comparison of molecular packing in crystal structure of c) **10** and d) **11**. Hydrogen atoms are omitted for clarity and oxygen atoms are shown as balls.

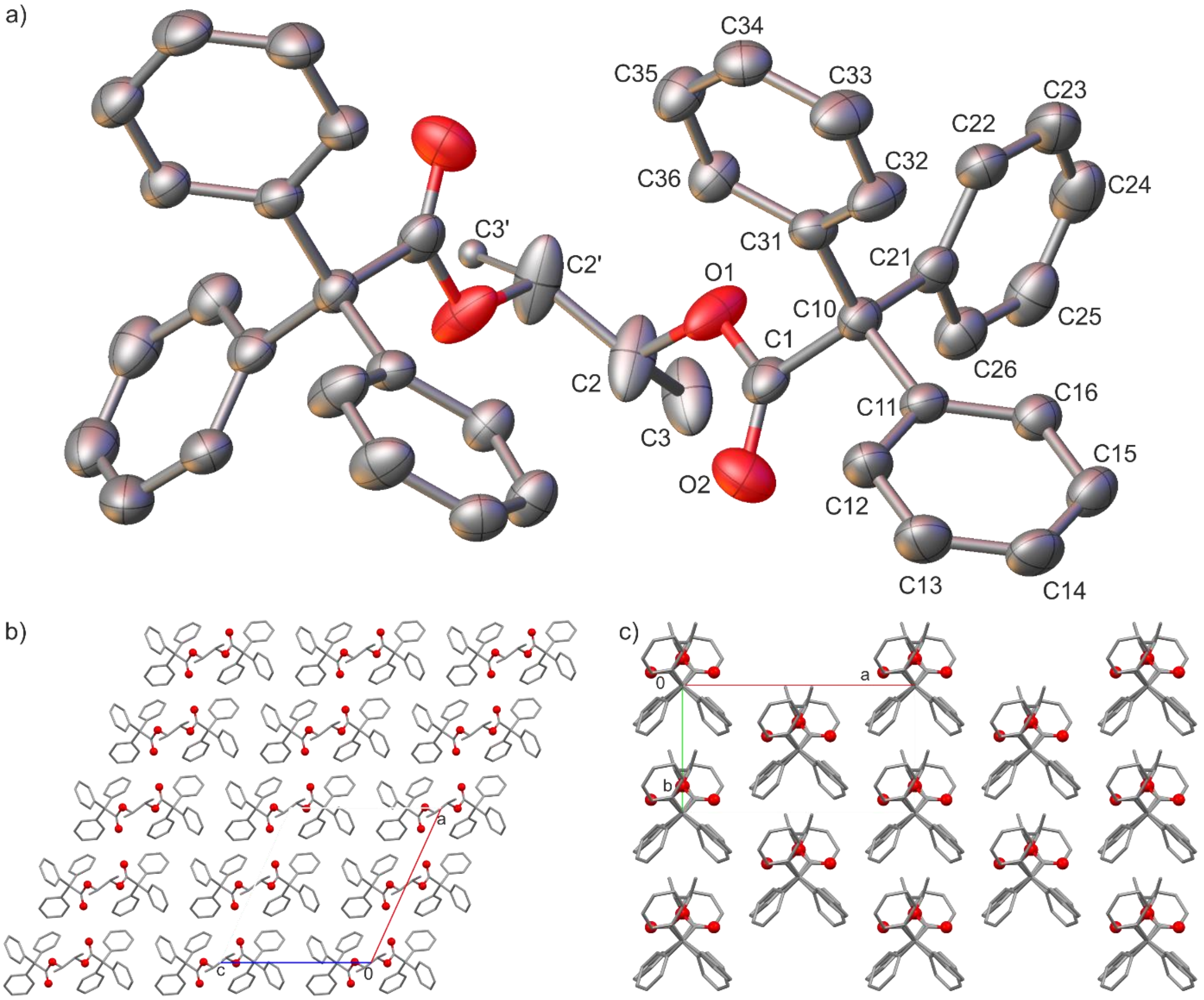

Figure SI_137. Molecular structure and atoms numbering scheme of compound **18**. Only the asymmetric part has been numbered for clarity. Displacement ellipsoids are drawn at 50% probability level. Molecular packing in crystal structure b) view along b axis and c) view along c axis. Hydrogen atoms are omitted for clarity and oxygen atoms are shown as balls.

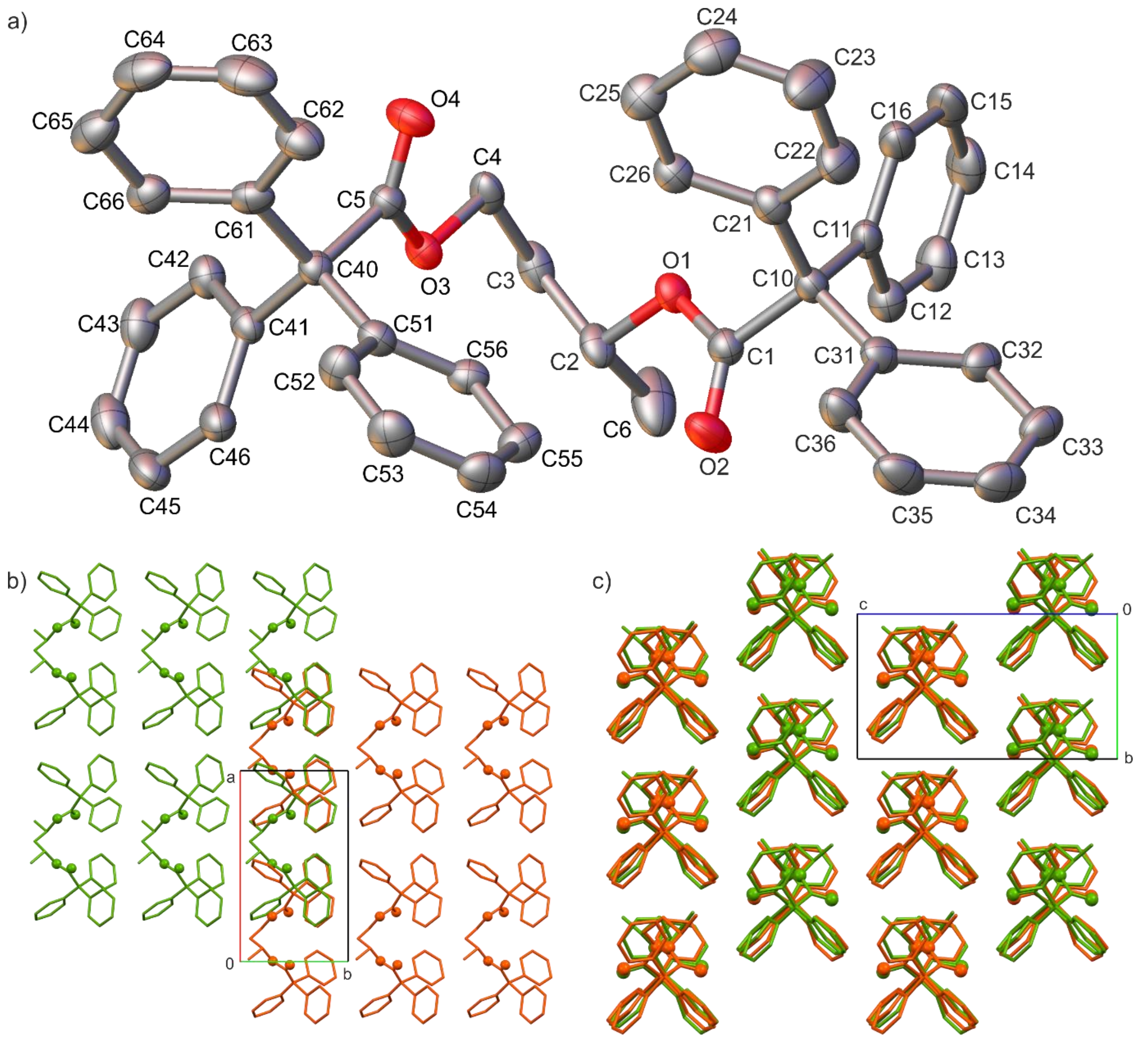

Figure SI_138. Molecular structure and atoms numbering scheme of compound **19**. Displacement ellipsoids are drawn at 50% probability level. b) Scheme of molecular disorder in columns. c) Molecular packing in crystal structure, view along a axis. Hydrogen atoms are omitted for clarity and oxygen atoms are shown as balls.

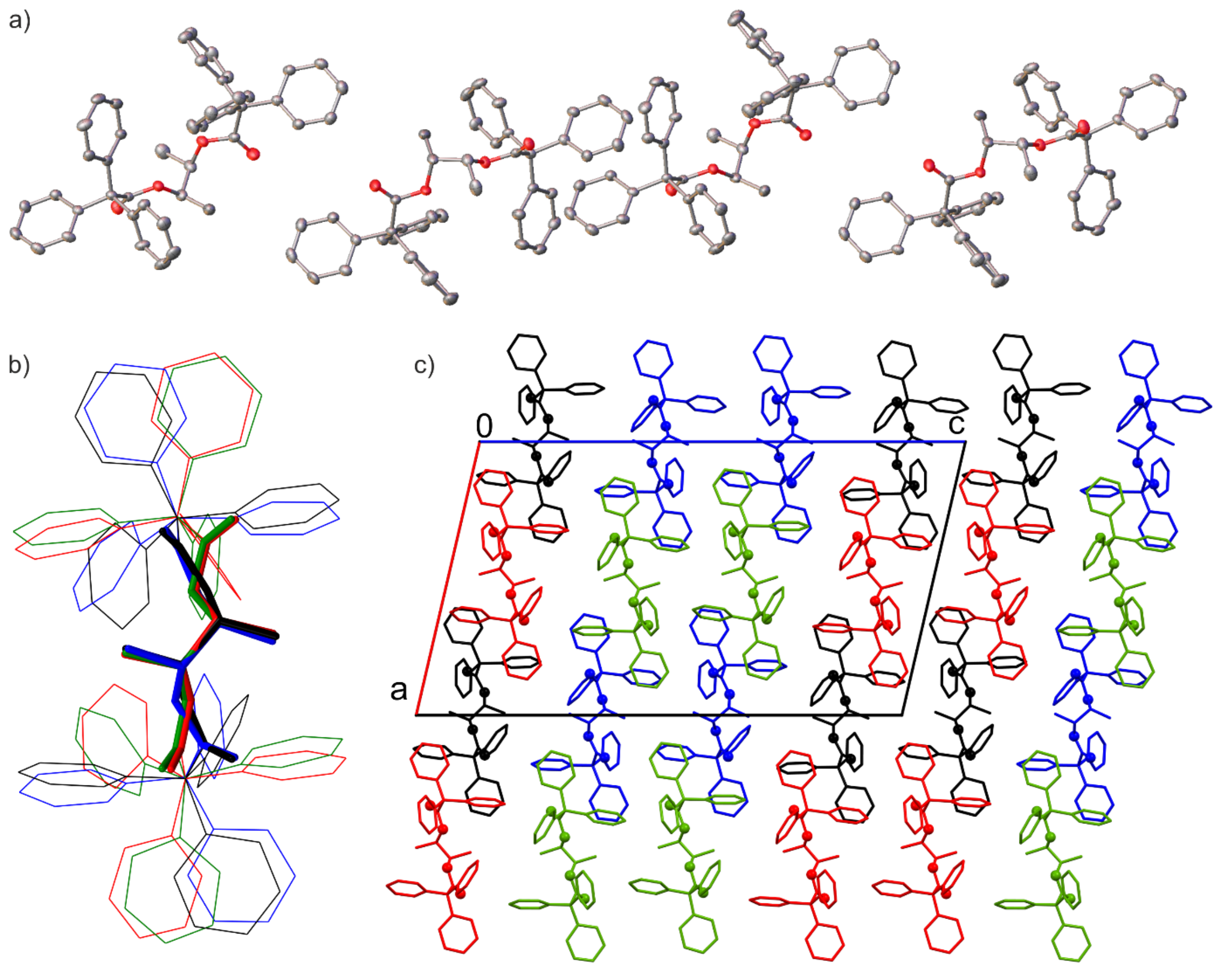

Figure SI_139. Molecular structure of asymmetric unit in crystal structure of compound **20**. Displacement ellipsoids are drawn at 50% probability level. b) Comparison molecular conformation of symmetrically independent molecules. c) Molecular packing in crystal structure -molecules **A** + **B** and **C** + **D** forms alternating double layers. Hydrogen atoms are omitted for clarity and oxygen atoms are shown as balls.

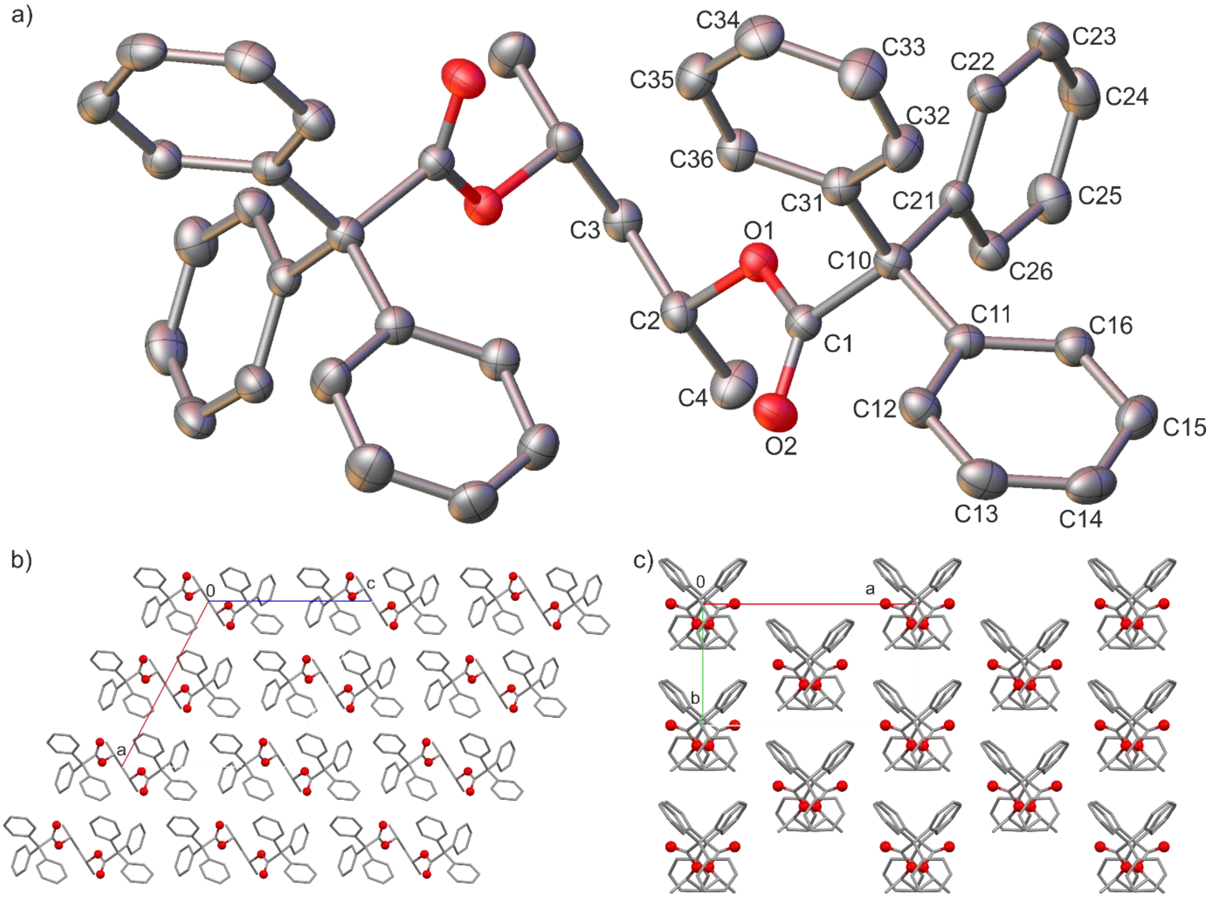

Figure SI_140. a) Molecular structure and atoms numbering scheme of compound **21**. Only the asymmetric part has been numbered for clarity. Displacement ellipsoids are drawn at 50% probability level. Molecular packing in crystal structure b) view along b axis and c) view along c axis. Hydrogen atoms are omitted for clarity and oxygen atoms are shown as balls.

Copies of ^{1}H and ^{13}C NMR spectra

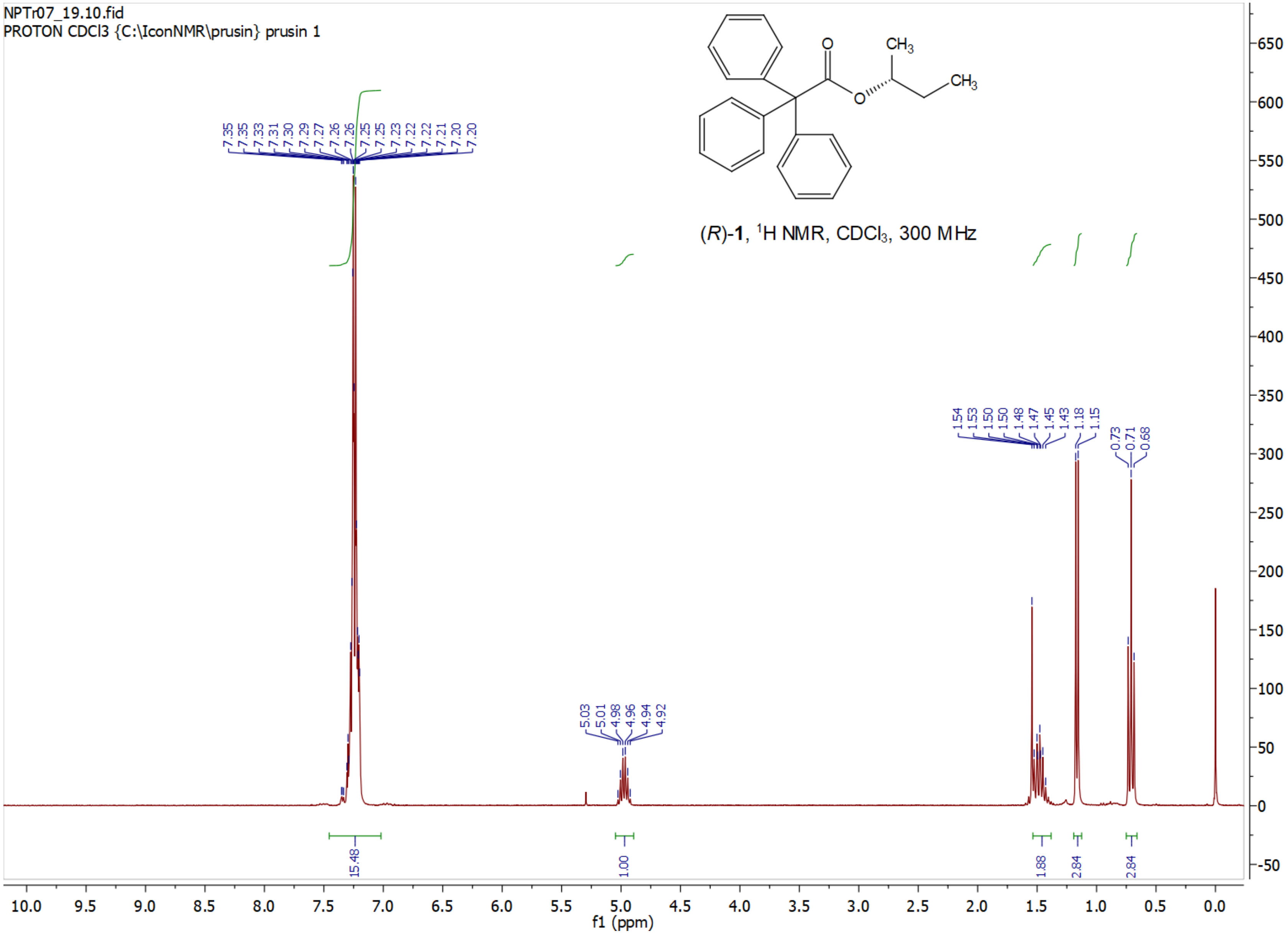

NPTr07_19.10.fid
PROTON CDCl3 {C:\IconNMR\prusin} prusin 1
(R)-1, ¹H NMR, CDCl₃, 300 MHz
O
CH3
CH3
7.35 7.35 7.33 7.31 7.30 7.29 7.27 7.26 7.26 7.25 7.25 7.23 7.22 7.22 7.21 7.20 7.20
5.03 5.01 4.98 4.96 4.94 4.92
1.54 1.53 1.50 1.50 1.48 1.47 1.45 1.43 1.18 1.15
0.73 0.71 0.68
15.48
1.00
1.88
2.84
2.84
f1 (ppm)

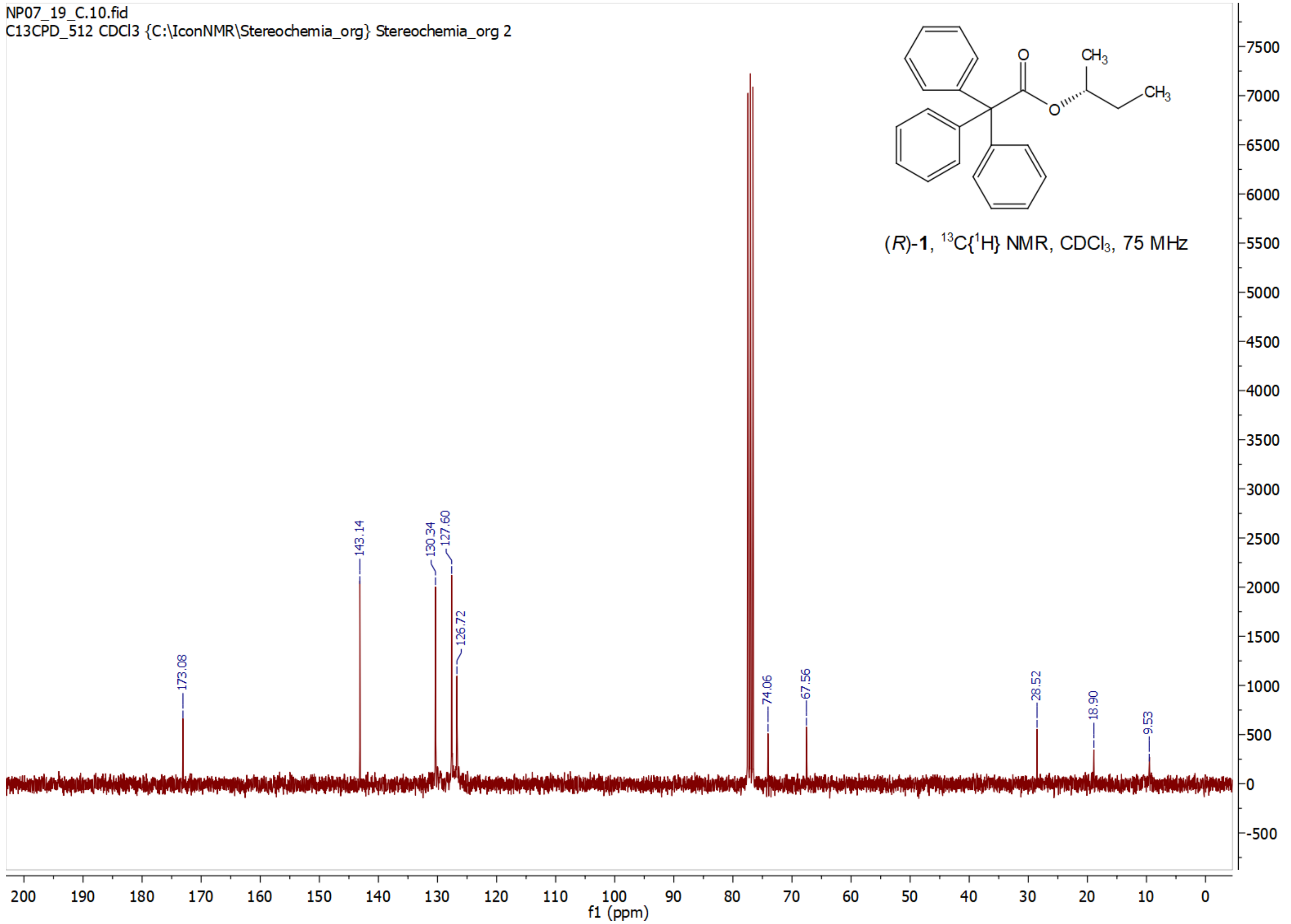
NP07_19_C.10.fid
C13CPD_512 CDCl3 {C:\IconNMR\Stereochemia_org} Stereochemia_org 2
O
O
CH3
CH3
(R)-1, 13C{1H} NMR, CDCl3, 75 MHz
173.08
143.14
130.34
127.60
126.72
74.06
67.56
28.52
18.90
9.53
f1 (ppm)

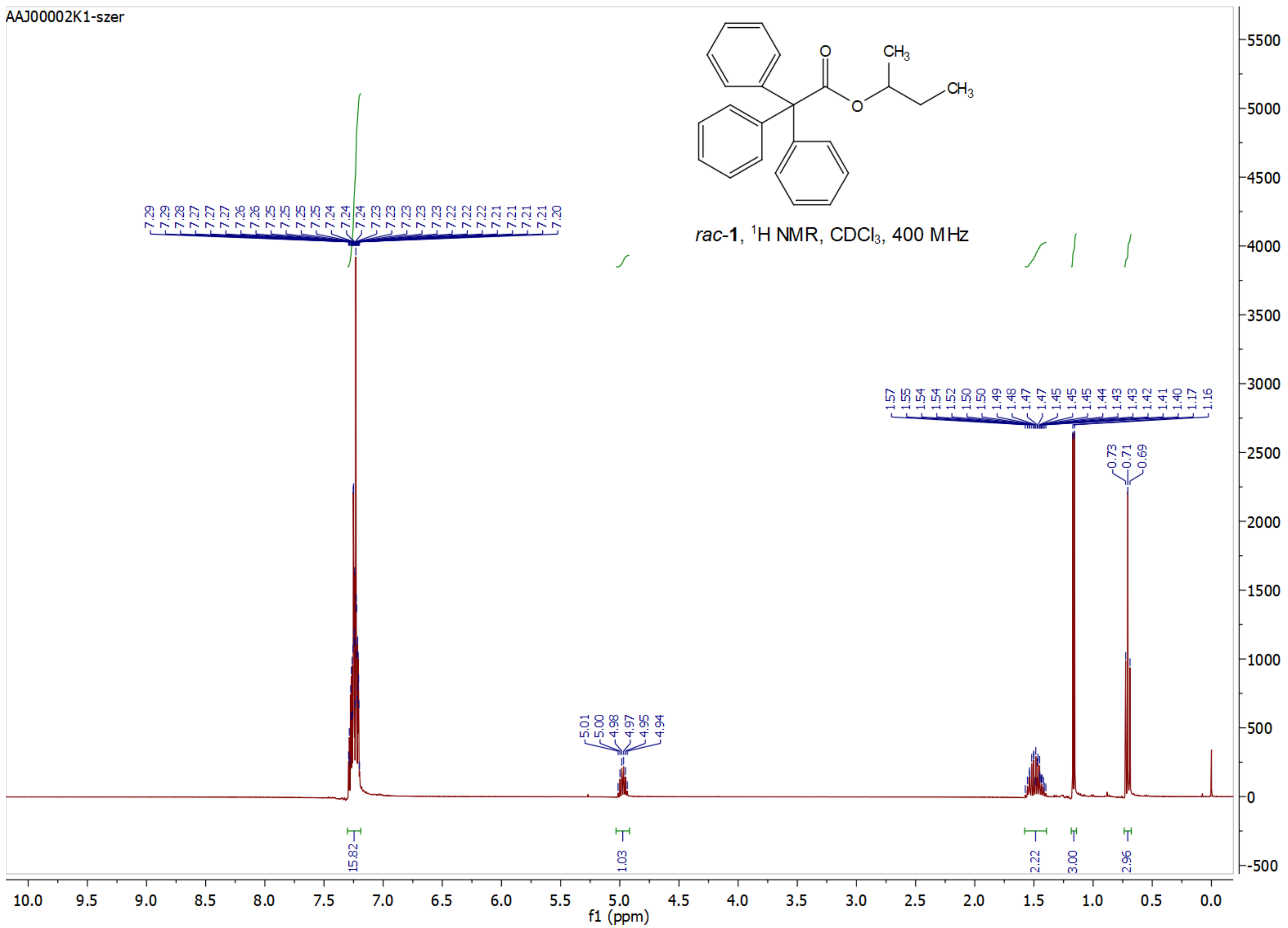

AAJ00002K1-szer
rac-1, 1H NMR, CDCl3, 400 MHz
O
O
CH3
CH3
f1 (ppm)

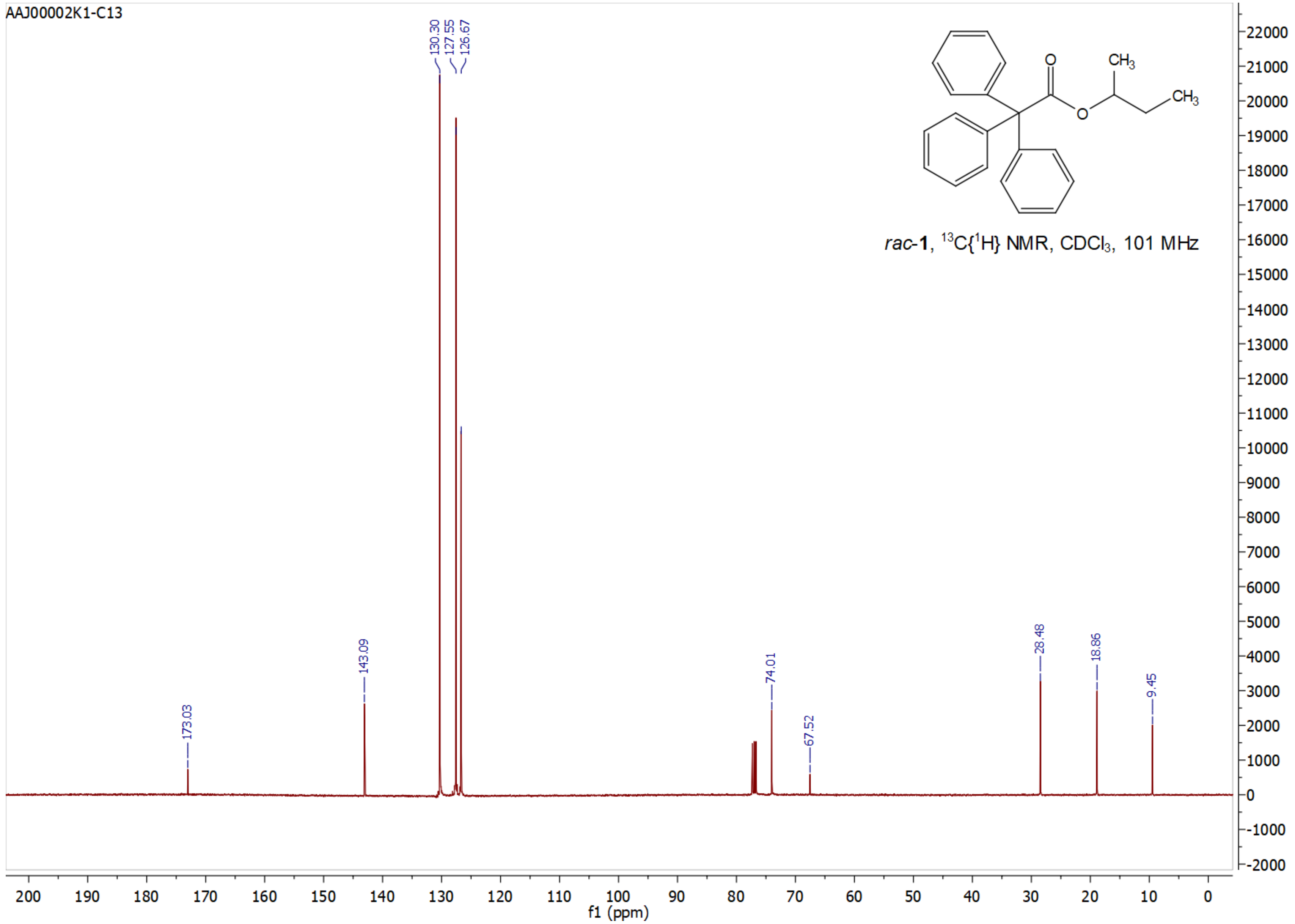

AAJ00002K1-C13
130.30
127.55
126.67
173.03
143.09
74.01
67.52
28.48
18.86
9.45
O
CH3
CH3
O
rac-1, 13C{1H} NMR, CDCl3, 101 MHz
f1 (ppm)

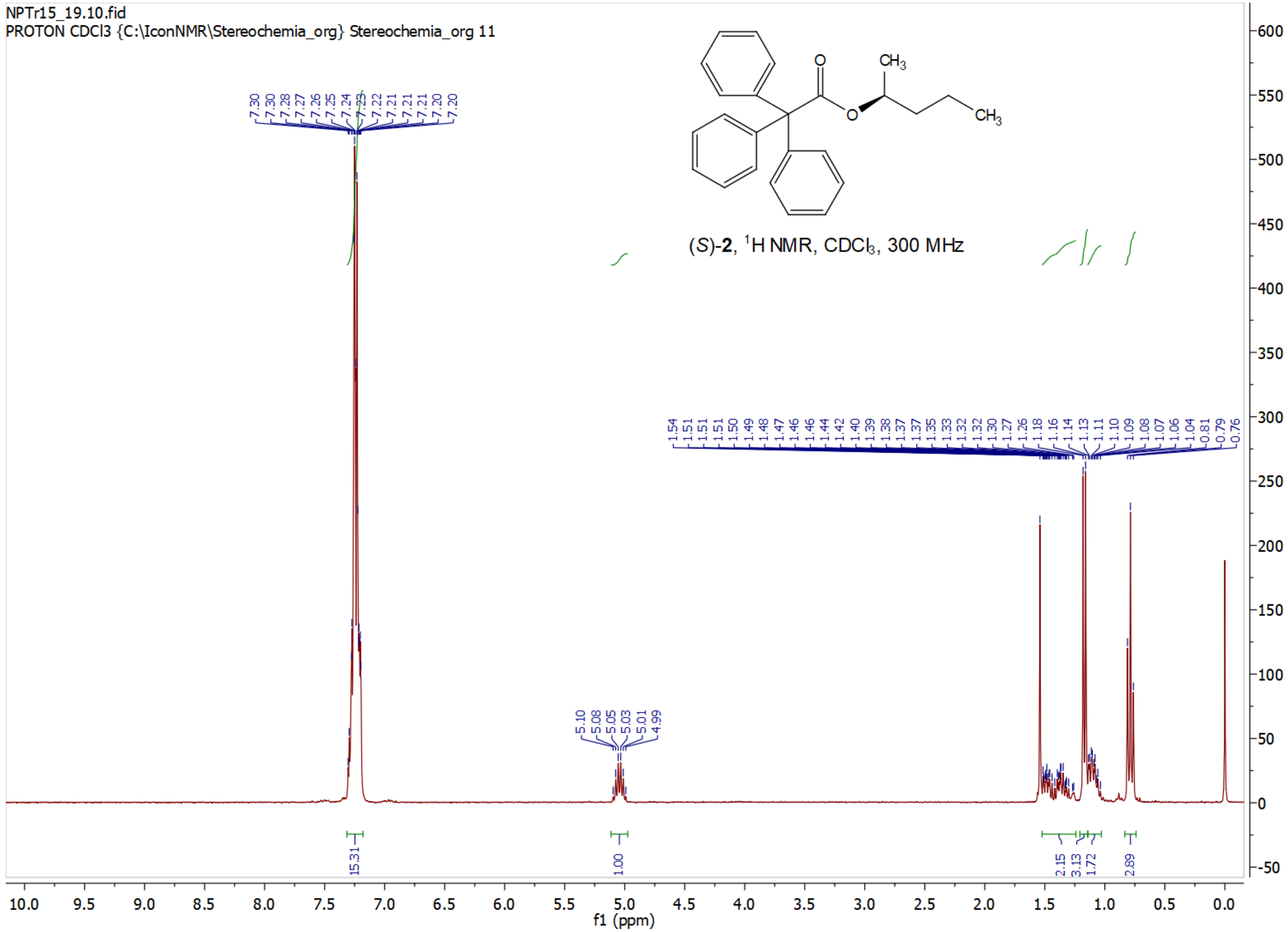
NPTr15_19.10.fid
PROTON CDCl3 {C:\IconNMR\Stereochemia_org} Stereochemia_org 11
(S)-2, 1H NMR, CDCl3, 300 MHz
f1 (ppm)

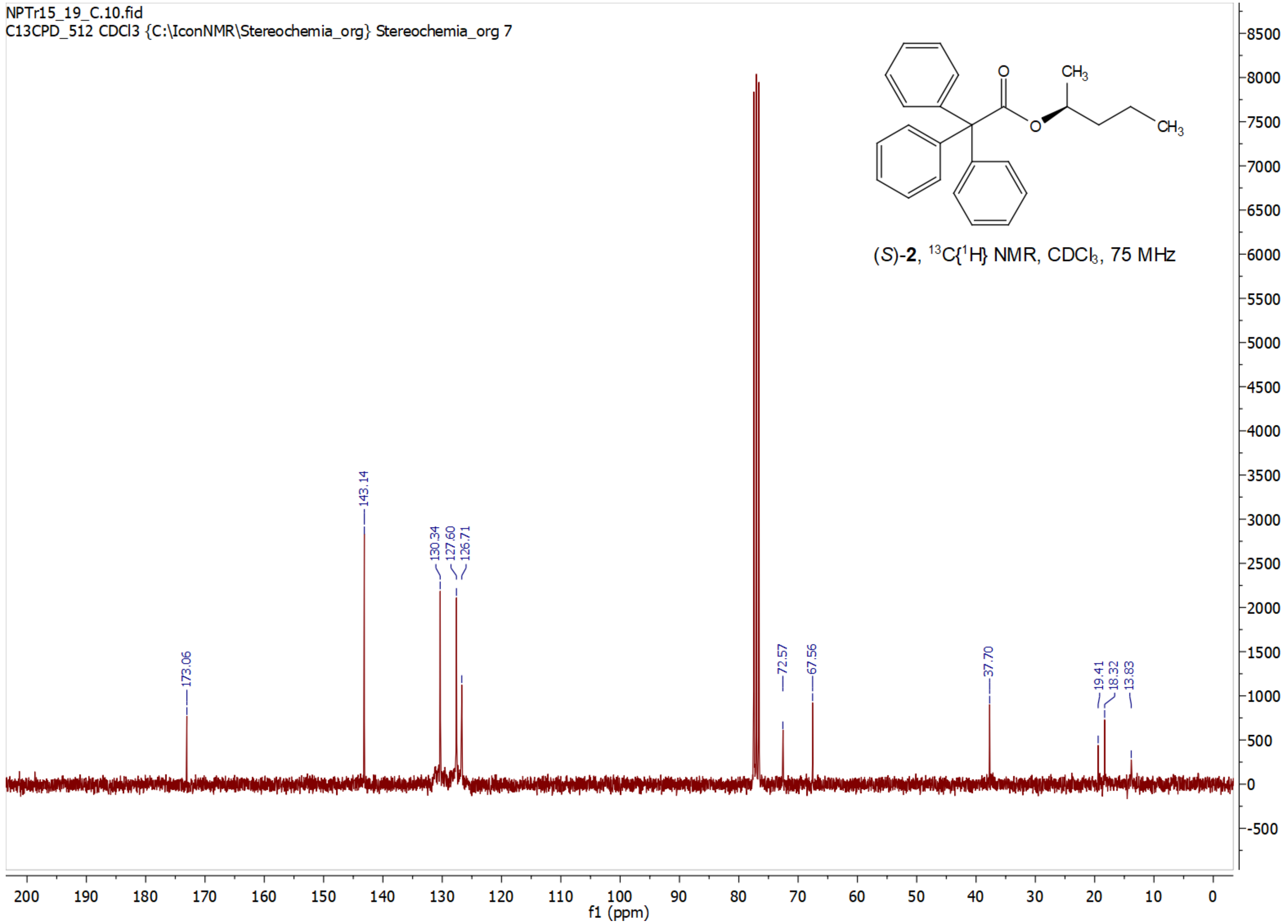
NPTr15_19_C.10.fid
C13CPD_512 CDCl3 {C:\IconNMR\Stereochemia_org} Stereochemia_org 7
(S)-2, 13C{1H} NMR, CDCl3, 75 MHz
CH3
O
O
CH3
173.06
143.14
130.34
127.60
126.71
72.57
67.56
37.70
19.41
18.32
13.83
f1 (ppm)

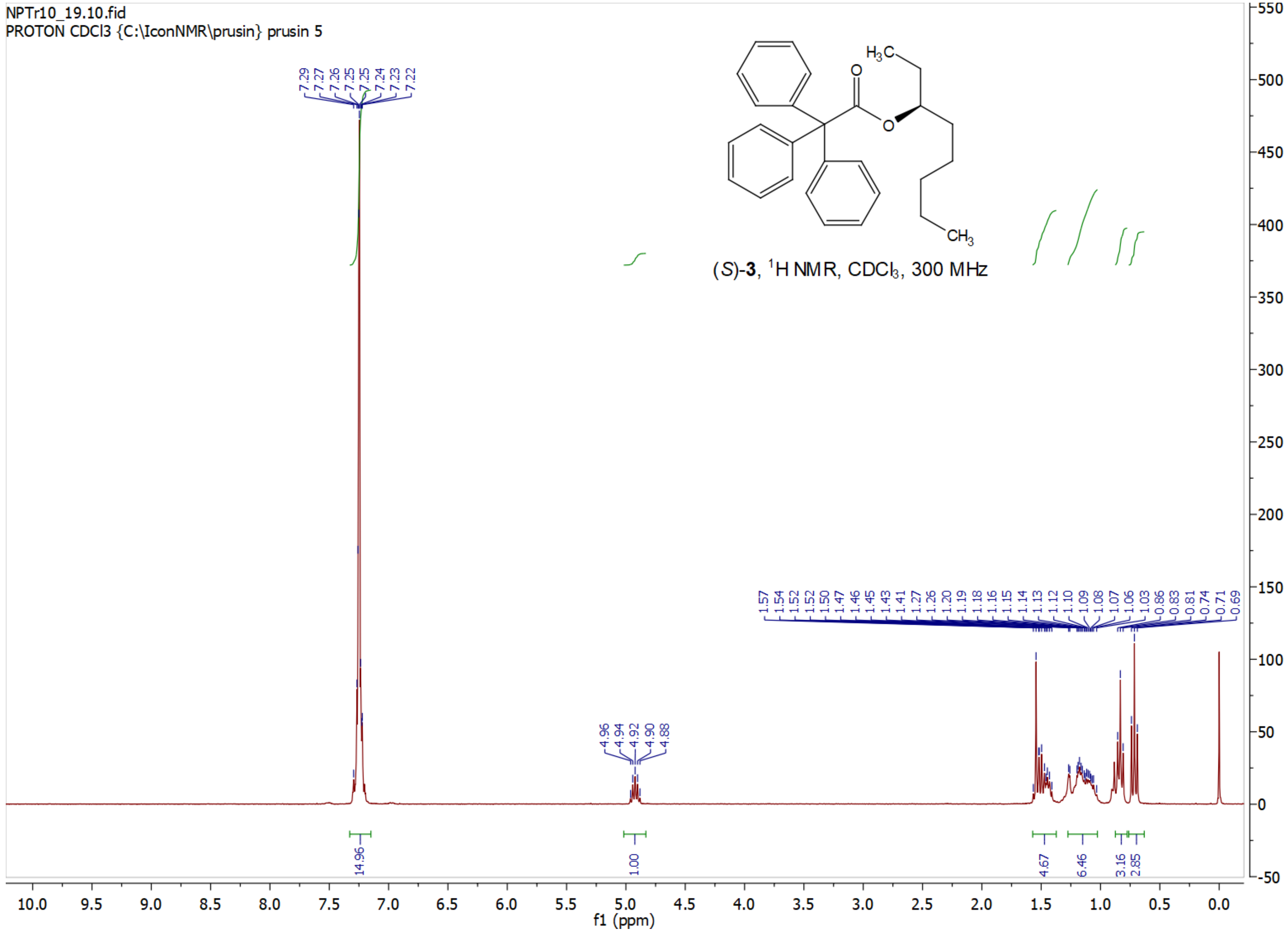
NPTr10_19.10.fid
PROTON CDCl3 {C:\IconNMR\prusin} prusin 5
H3C
O
O
CH3
(S)-3, 1H NMR, CDCl3, 300 MHz
7.29 7.27 7.26 7.25 7.25 7.24 7.23 7.22
4.96 4.94 4.92 4.90 4.88
1.57 1.54 1.52 1.52 1.50 1.47 1.46 1.45 1.43 1.41 1.27 1.26 1.20 1.19 1.18 1.16 1.15 1.14 1.13 1.12 1.10 1.09 1.08 1.07 1.06 1.03 0.86 0.83 0.81 0.74 0.71 0.69
14.96
1.00
4.67
6.46
3.16
2.85
f1 (ppm)

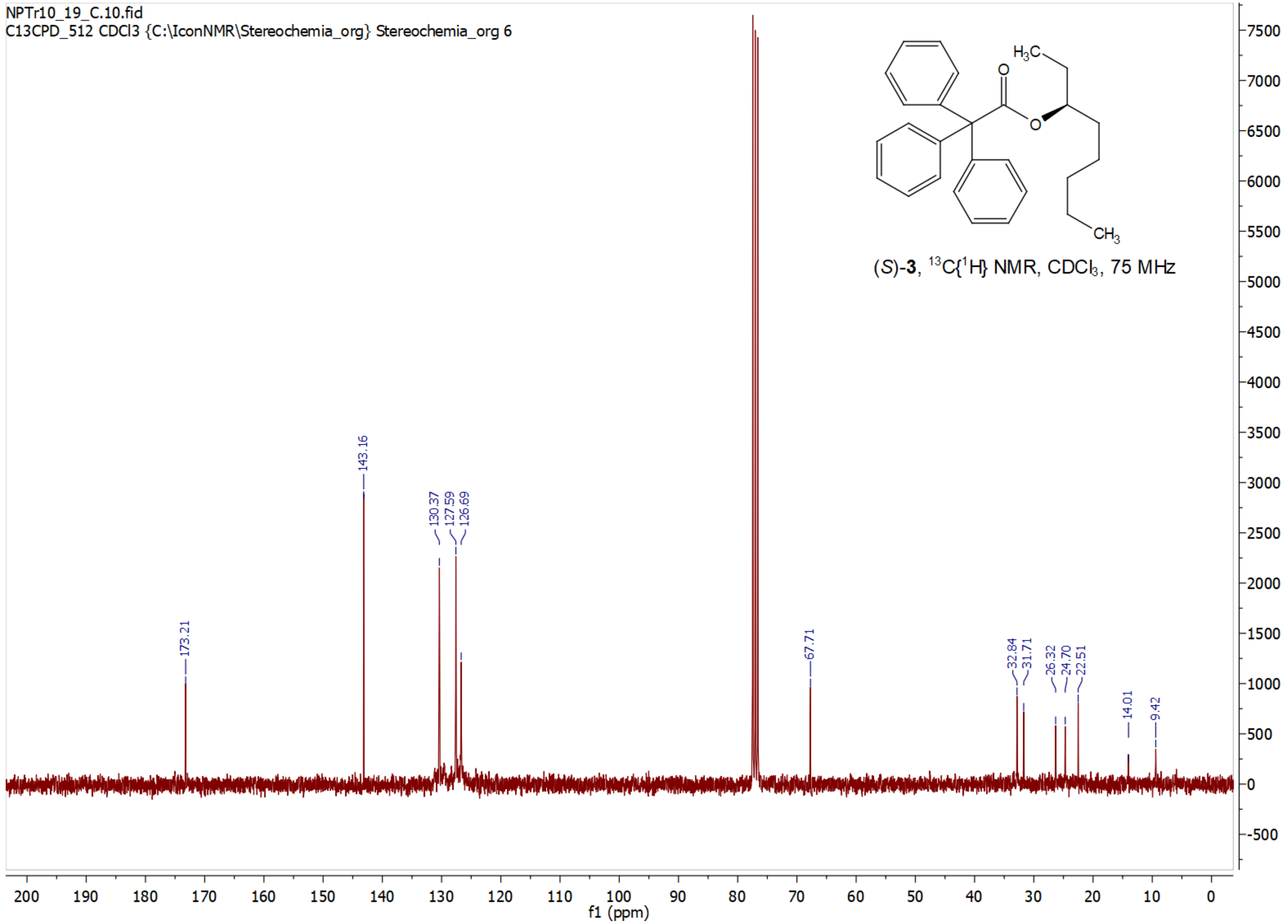

NPTr10_19_C.10.fid
C13CPD_512 CDCl3 {C:\IconNMR\Stereochemia_org} Stereochemia_org 6
(S)-3, 13C{1H} NMR, CDCl3, 75 MHz
173.21
143.16
130.37
127.59
126.69
67.71
32.84
31.71
26.32
24.70
22.51
14.01
9.42
f1 (ppm)

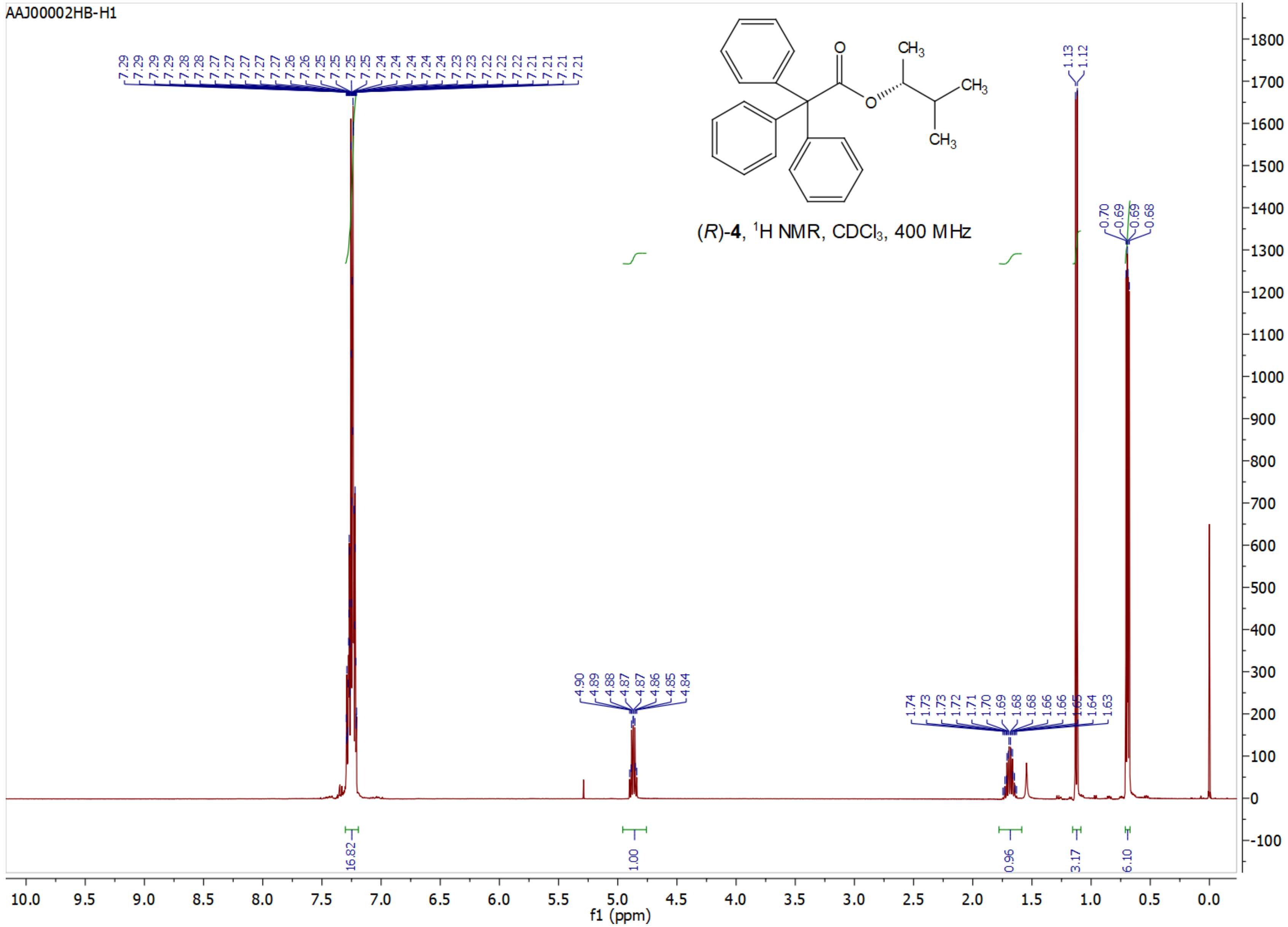

AAJ00002HB-H1
(R)-4, 1H NMR, CDCl3, 400 MHz
f1 (ppm)
16.82
1.00
0.96
3.17
6.10

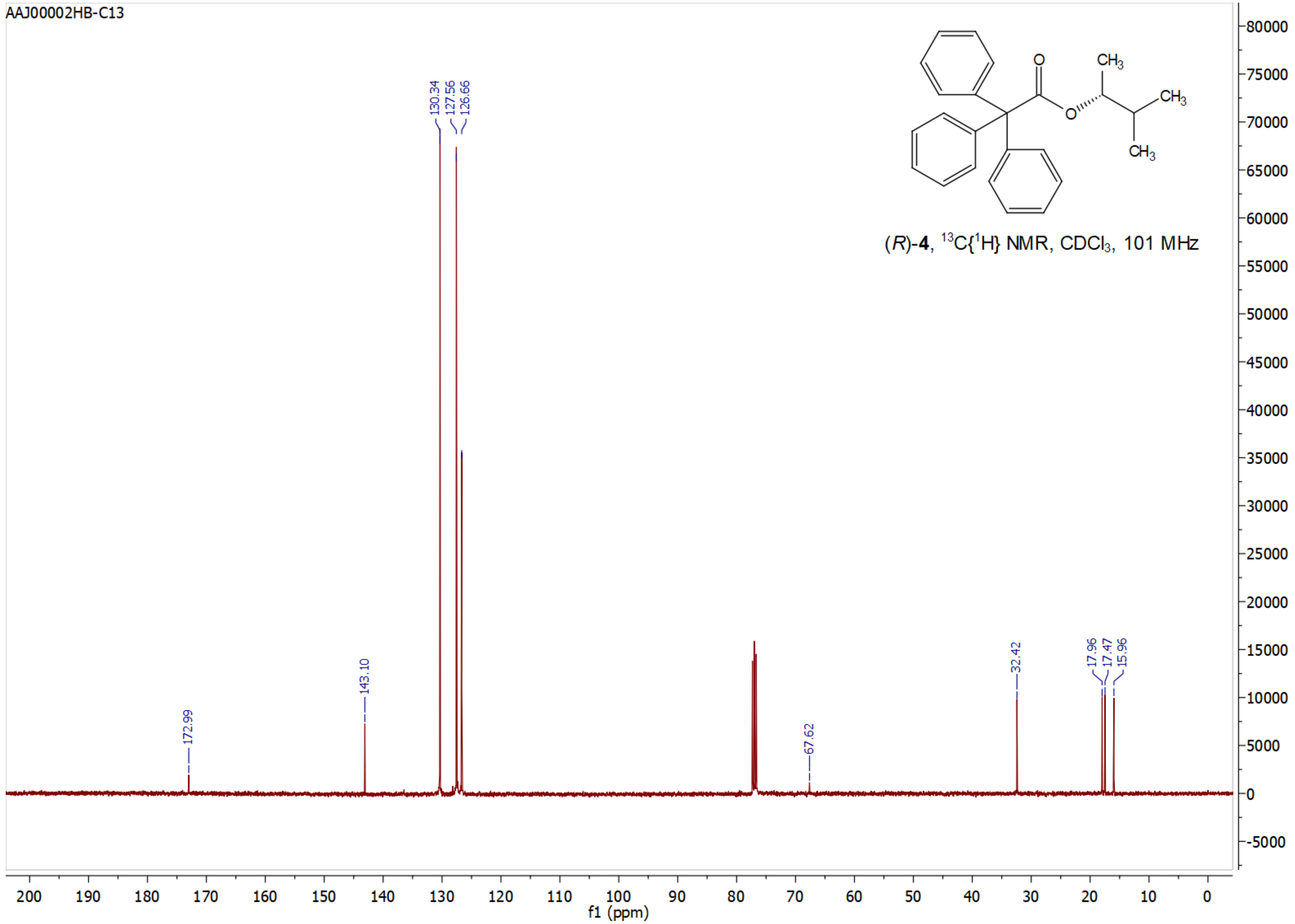

AAJ00002HB-C13
(R)-4, 13C{1H} NMR, CDCl3, 101 MHz
CH3
O
130.34
127.56
126.66
172.99
143.10
67.62
32.42
17.96
17.47
15.96
f1 (ppm)

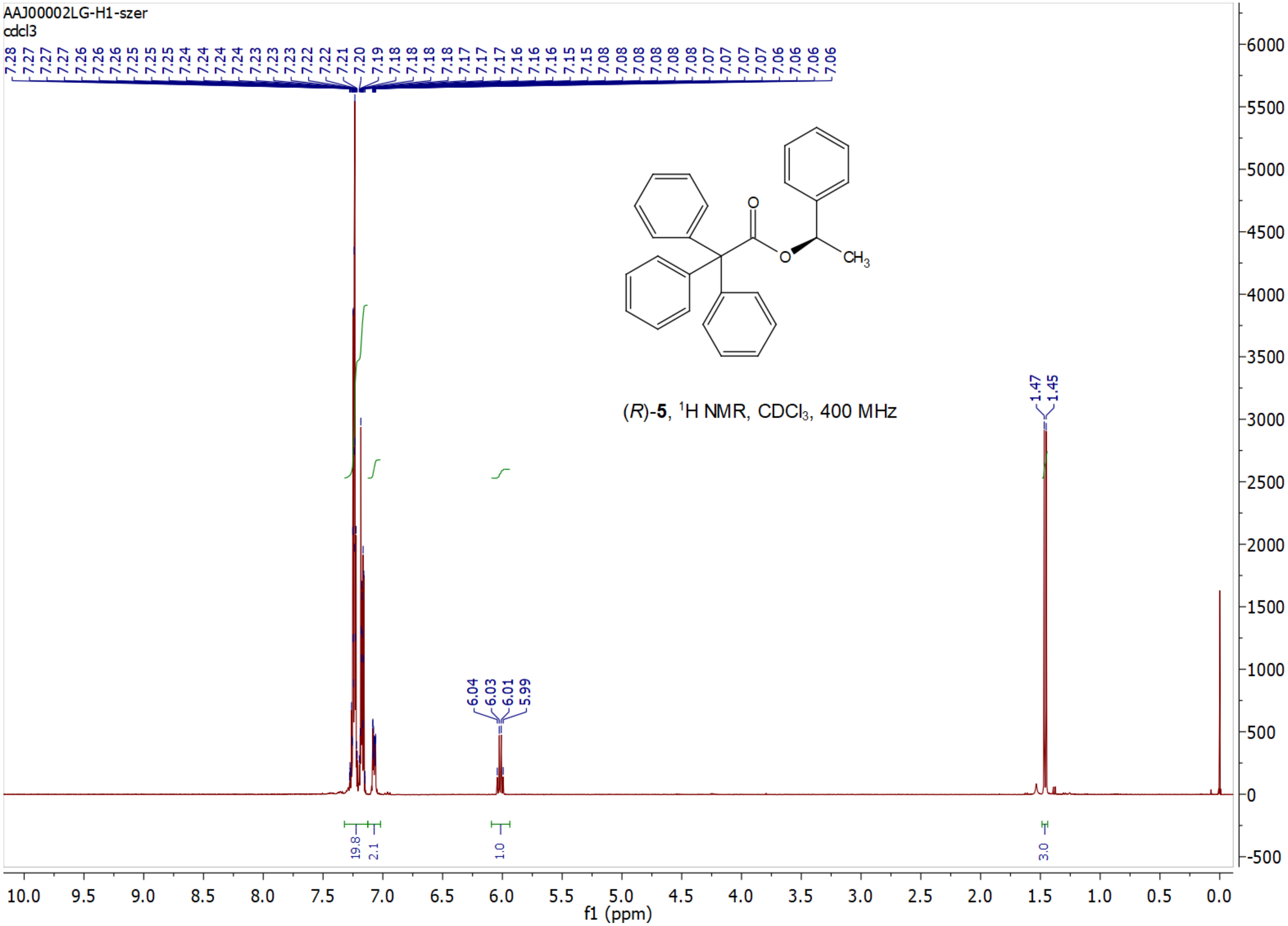
AAJ00002LG-H1-szer
cdcl3
7.28 7.27 7.27 7.27 7.26 7.26 7.26 7.25 7.25 7.25 7.24 7.24 7.24 7.24 7.23 7.23 7.23 7.22 7.22 7.21 7.20 7.19 7.18 7.18 7.18 7.18 7.17 7.17 7.17 7.16 7.16 7.16 7.15 7.15 7.08 7.08 7.08 7.08 7.08 7.08 7.07 7.07 7.07 7.07 7.06 7.06 7.06 7.06
O
O
CH3
(R)-5, 1H NMR, CDCl3, 400 MHz
6.04
6.03
6.01
5.99
1.47
1.45
19.8
2.1
1.0
3.0
f1 (ppm)

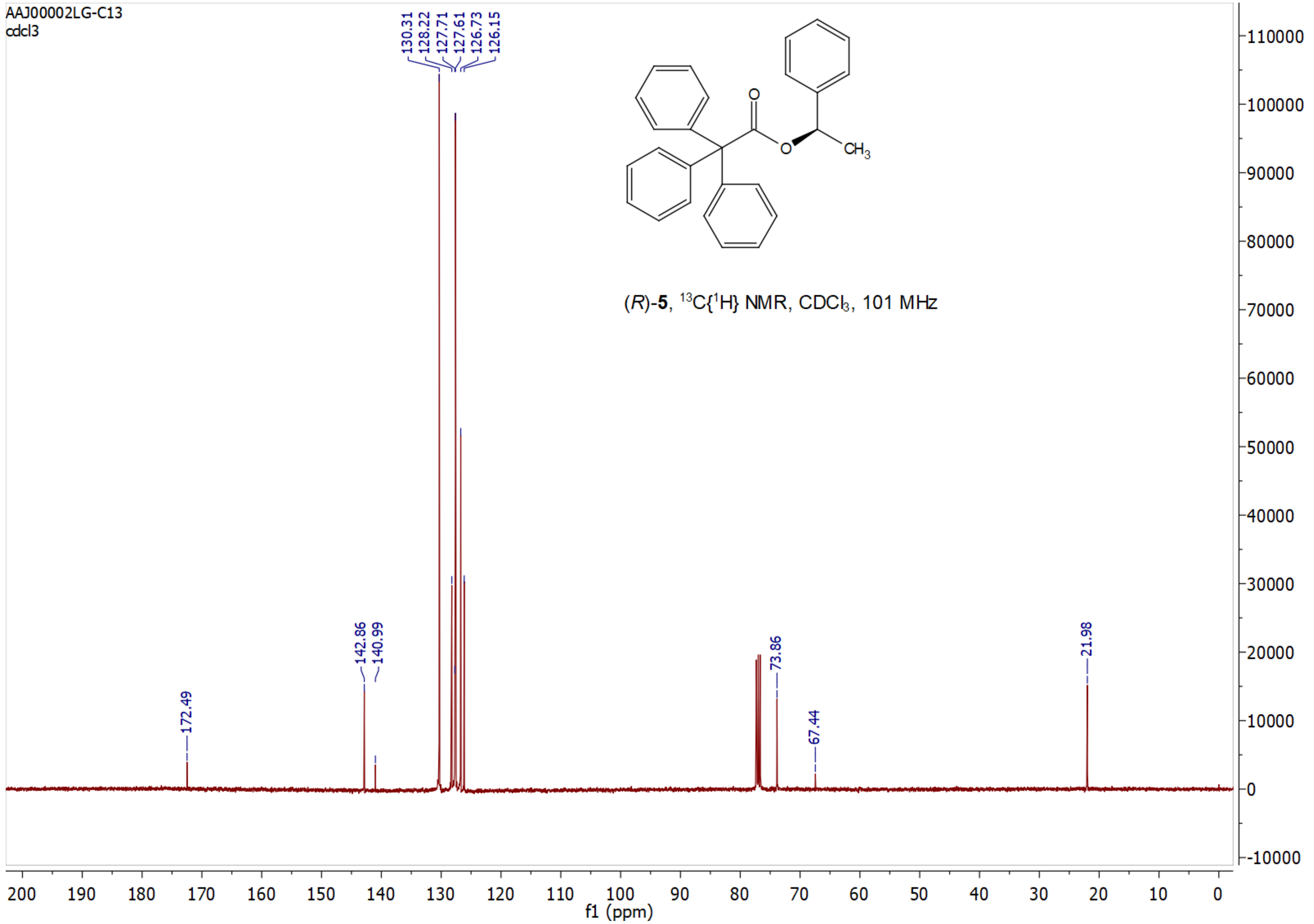

AAJ00002LG-C13
cdcl3
130.31
128.22
127.71
127.61
126.73
126.15
O
O
CH3
(R)-5, 13C{1H} NMR, CDCl3, 101 MHz
172.49
142.86
140.99
73.86
67.44
21.98
110000
100000
90000
80000
70000
60000
50000
40000
30000
20000
10000
0
-10000
200 190 180 170 160 150 140 130 120 110 100 90 80 70 60 50 40 30 20 10 0
f1 (ppm)

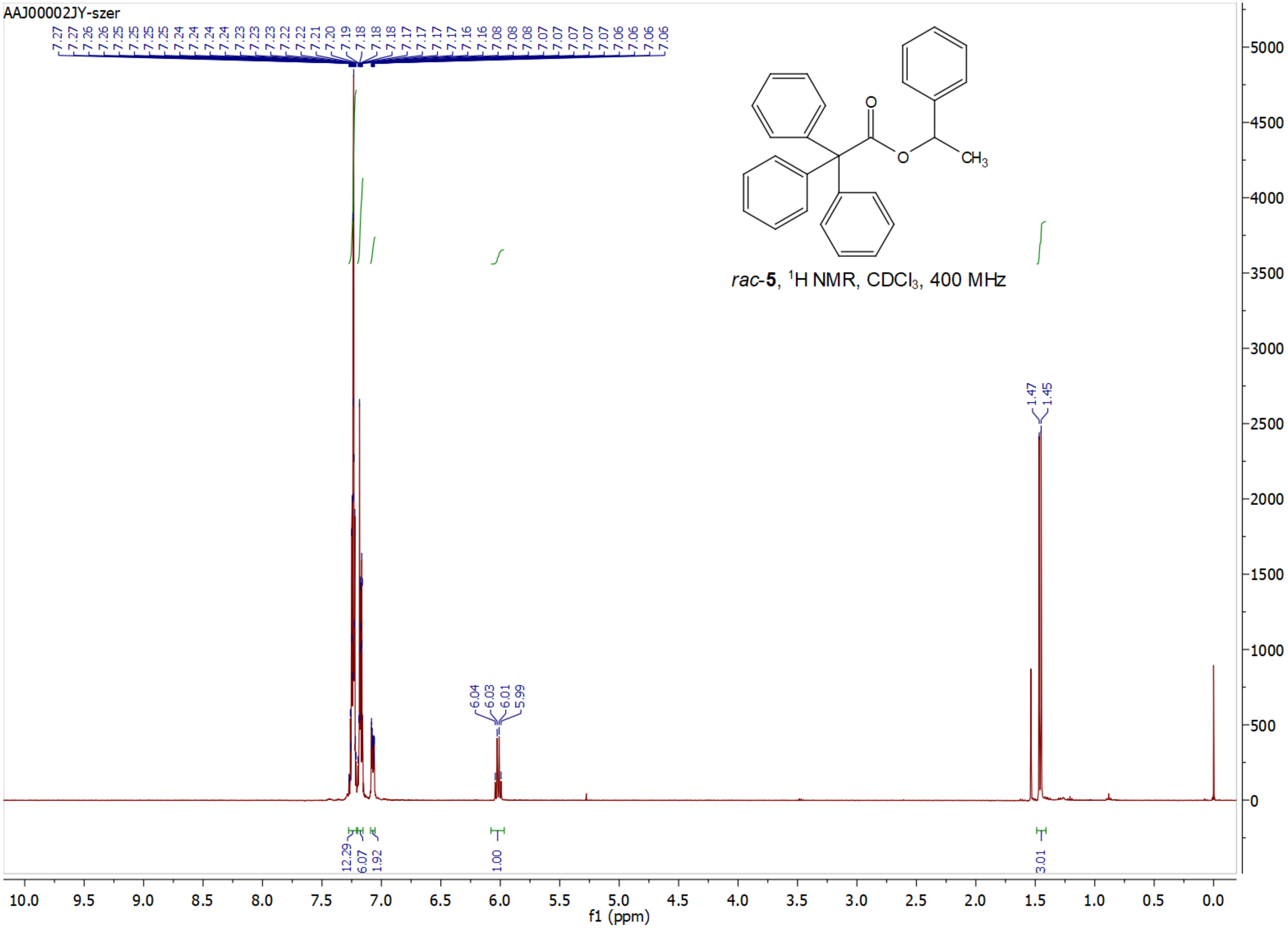
AAJ00002JY-szer
rac-5, ¹H NMR, CDCl₃, 400 MHz
CH3
O
O
f1 (ppm)
12.29
6.07
1.92
1.00
3.01
6.04
6.03
6.01
5.99
1.47
1.45

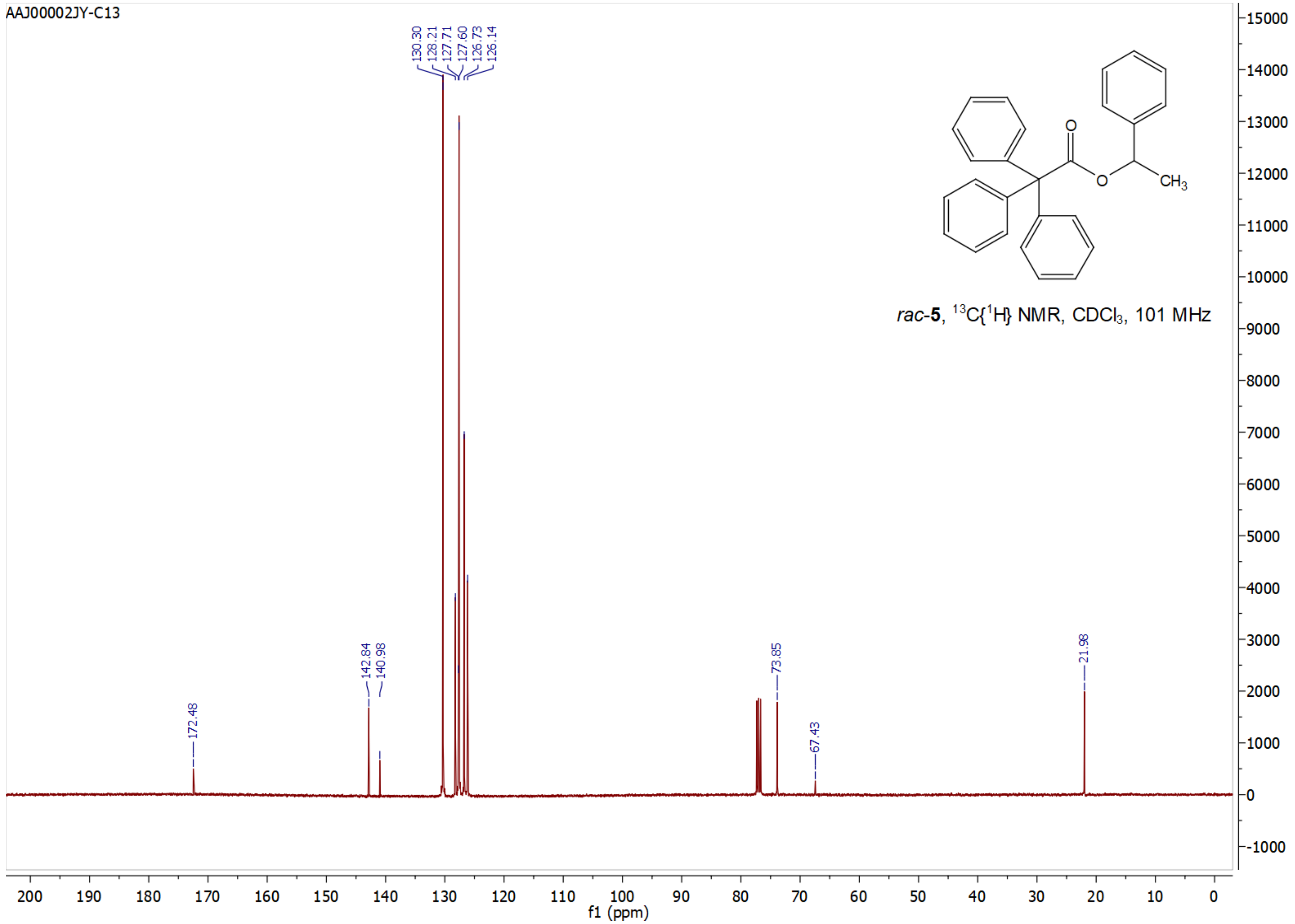
AAJ00002JY-C13
130.30
128.21
127.71
127.60
126.73
126.14
172.48
142.84
140.98
73.85
67.43
21.98
O
O
CH3
rac-5, 13C{1H} NMR, CDCl3, 101 MHz
f1 (ppm)

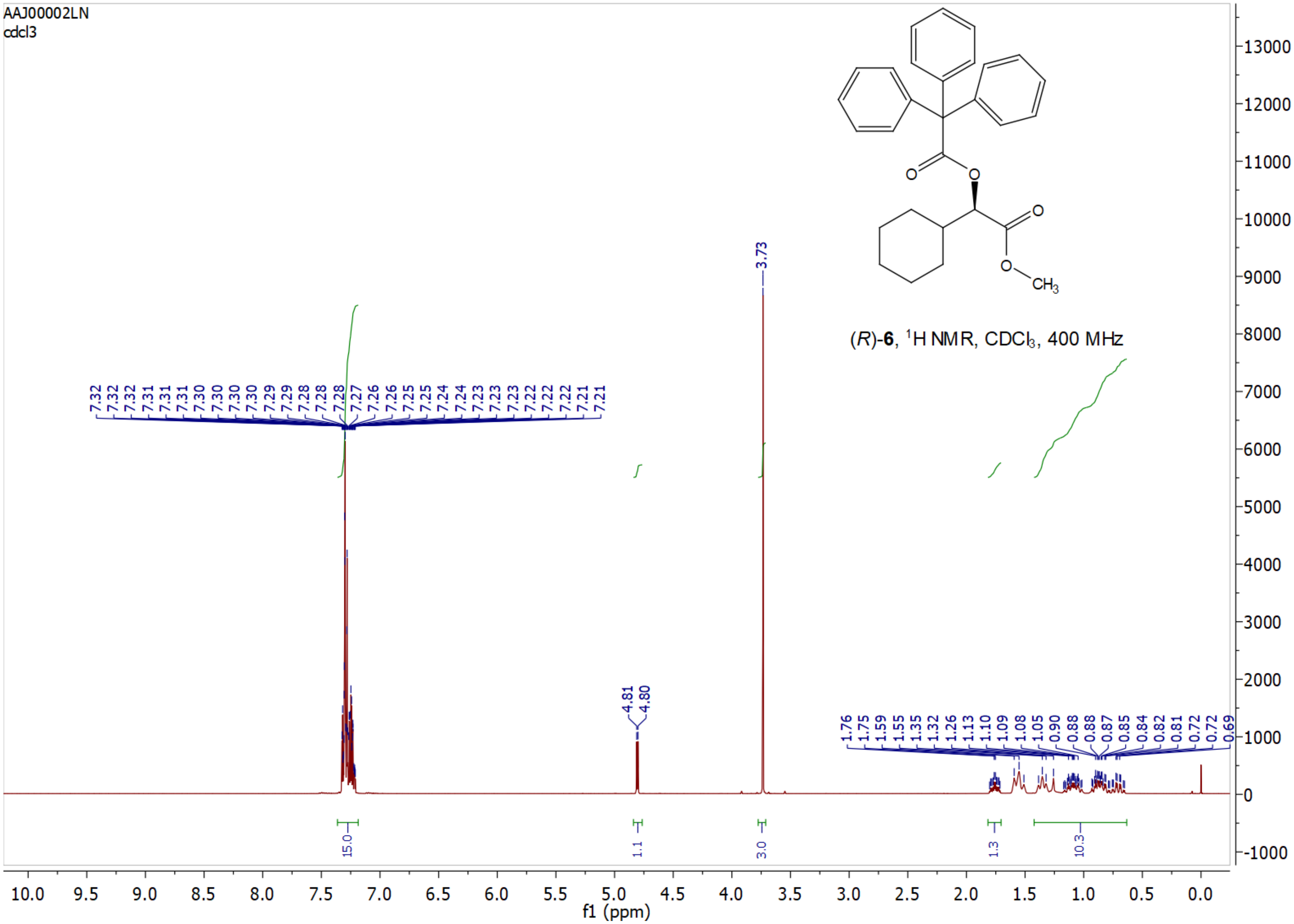

AAJ00002LN
cdcl3
(R)-6, 1H NMR, CDCl3, 400 MHz
7.32 7.32 7.32 7.31 7.31 7.30 7.30 7.30 7.30 7.29 7.29 7.28 7.28 7.28 7.27 7.26 7.26 7.25 7.25 7.24 7.24 7.23 7.23 7.23 7.22 7.22 7.22 7.21 7.21
4.81 4.80
3.73
1.76 1.75 1.59 1.55 1.35 1.32 1.26 1.13 1.10 1.09 1.08 1.05 0.90 0.88 0.88 0.87 0.85 0.84 0.82 0.81 0.72 0.72 0.69
15.0
1.1
3.0
1.3
10.3
f1 (ppm)

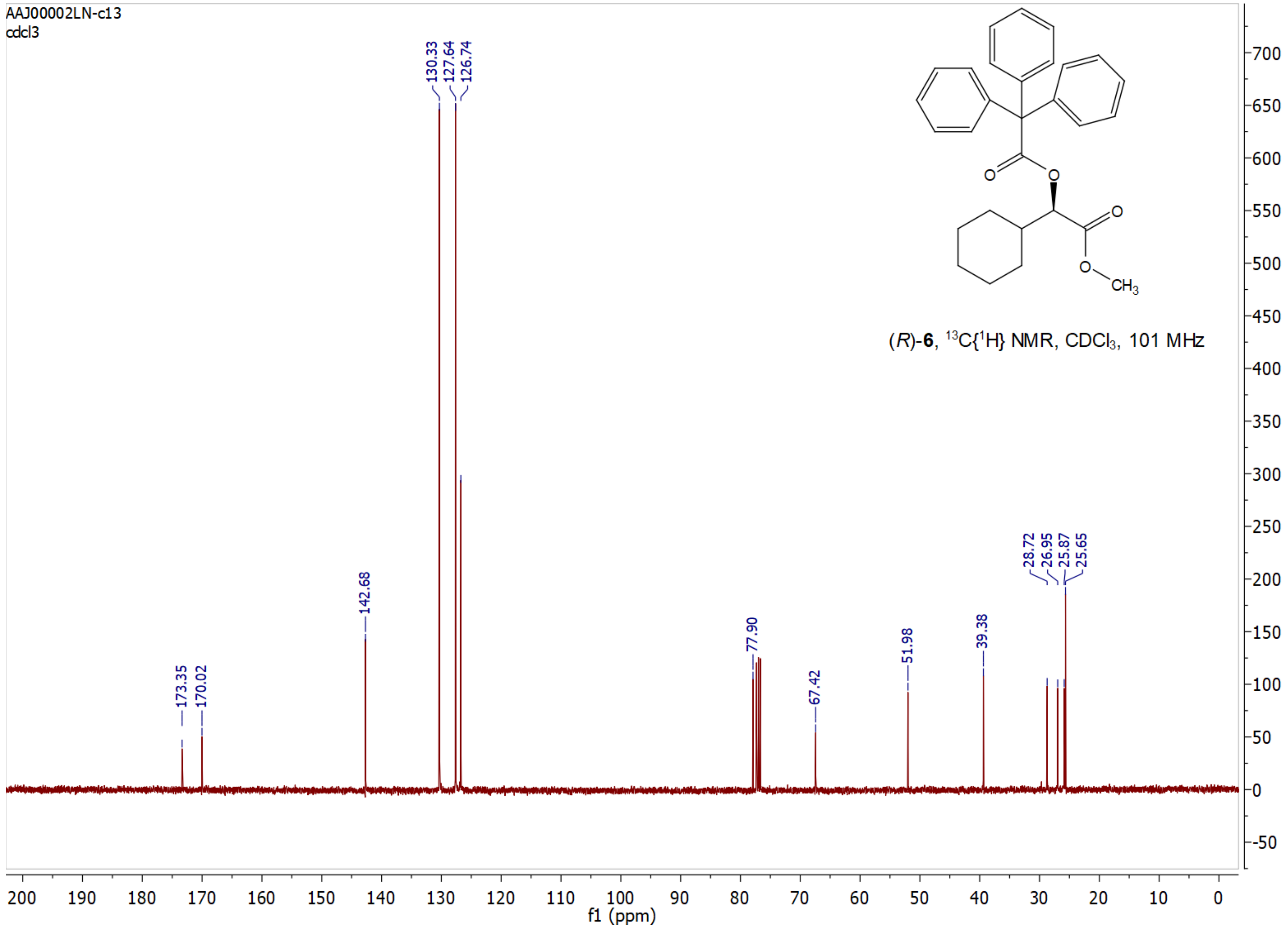
AAJ00002LN-c13
cdcl3
130.33
127.64
126.74
142.68
173.35
170.02
77.90
67.42
51.98
39.38
28.72
26.95
25.87
25.65
(R)-6, 13C{1H} NMR, CDCl3, 101 MHz
f1 (ppm)

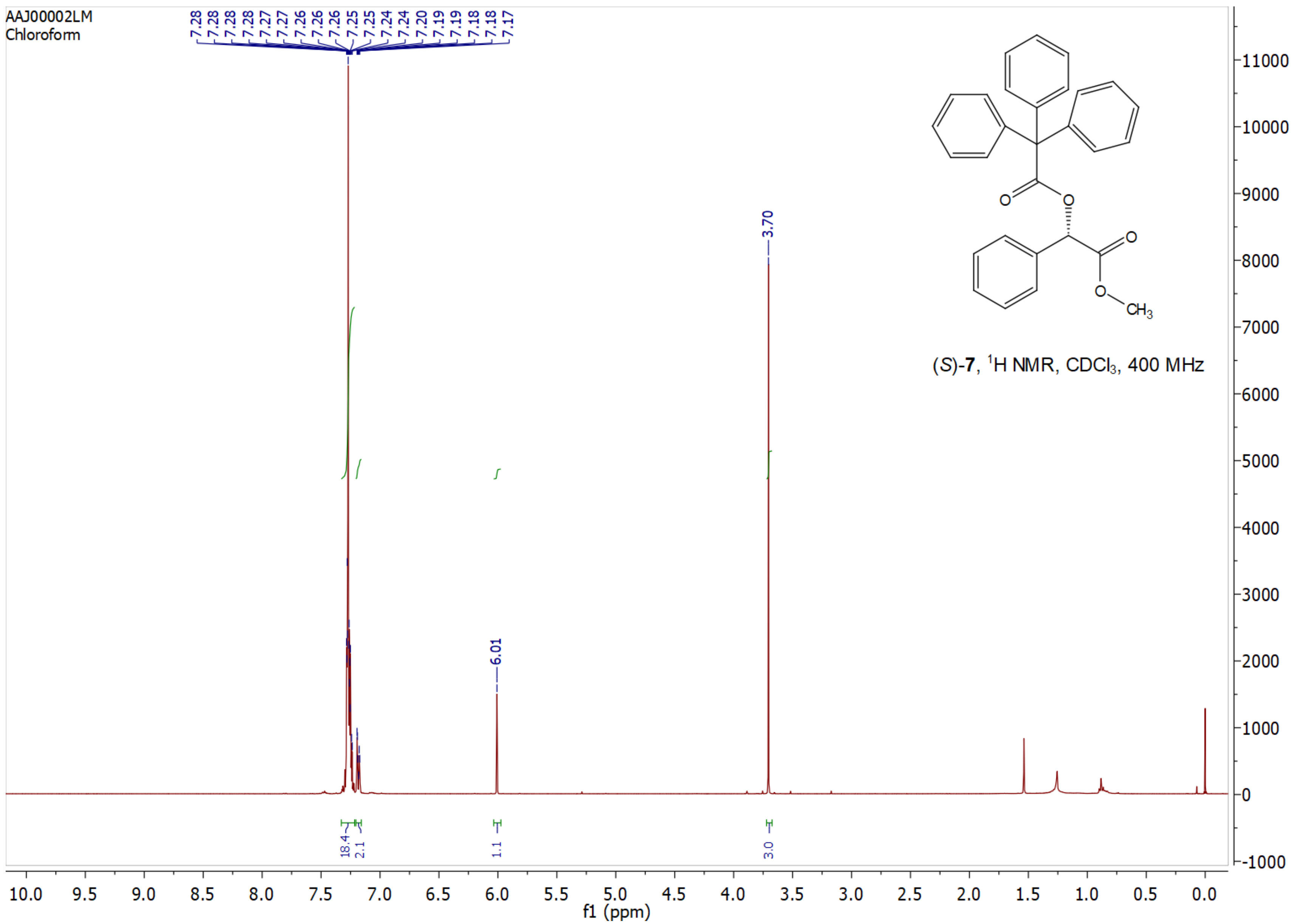
AAJ00002LM
Chloroform
7.28
7.28
7.28
7.28
7.27
7.27
7.26
7.26
7.26
7.25
7.25
7.24
7.24
7.20
7.19
7.19
7.18
7.18
7.17
6.01
3.70
O
O
O
O
CH3
(S)-7, 1H NMR, CDCl3, 400 MHz
18.4
2.1
1.1
3.0
f1 (ppm)

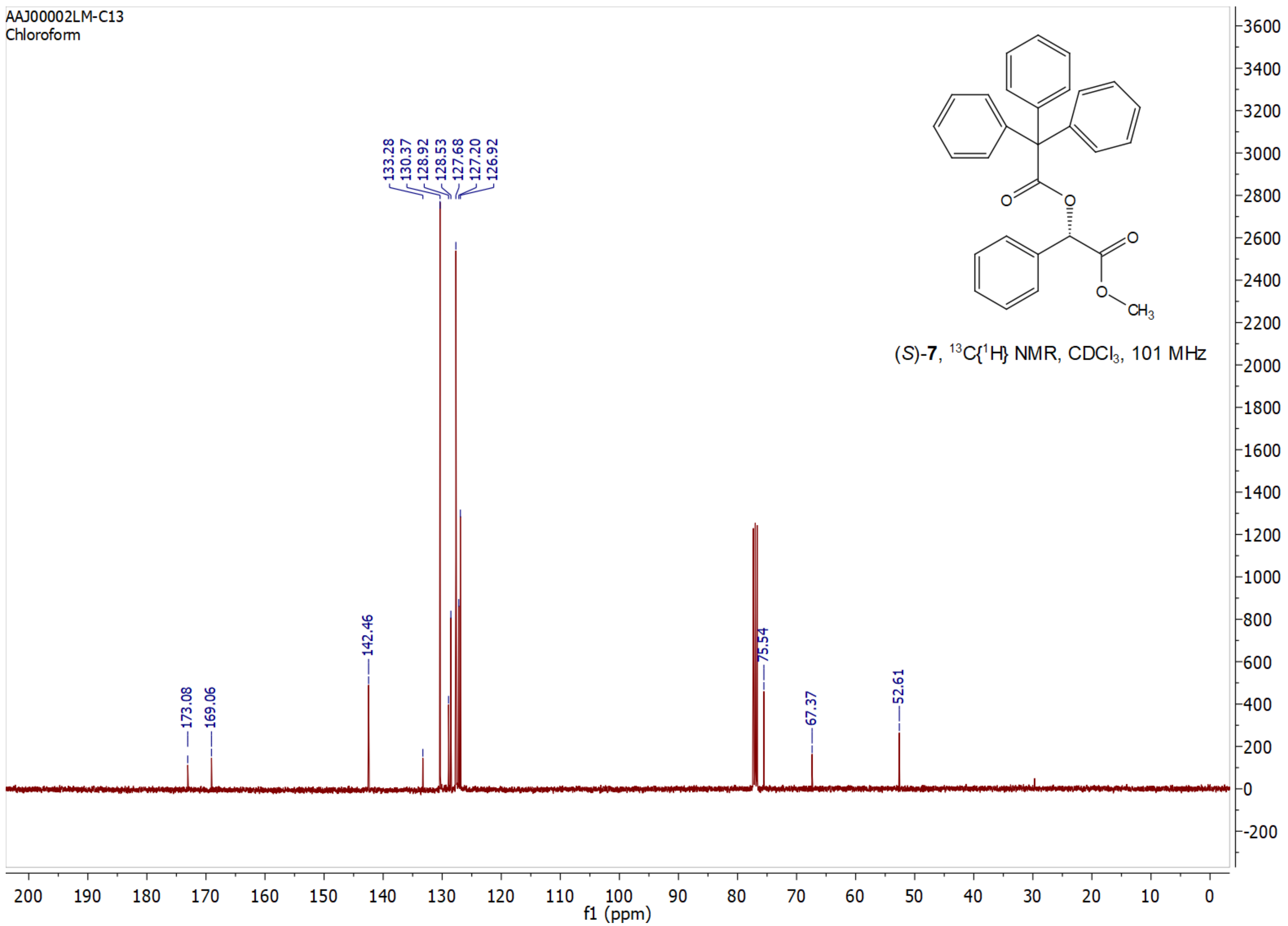
AAJ00002LM-C13
Chloroform
(S)-7, 13C{1H} NMR, CDCl3, 101 MHz
133.28
130.37
128.92
128.53
127.68
127.20
126.92
173.08
169.06
142.46
75.54
67.37
52.61
f1 (ppm)

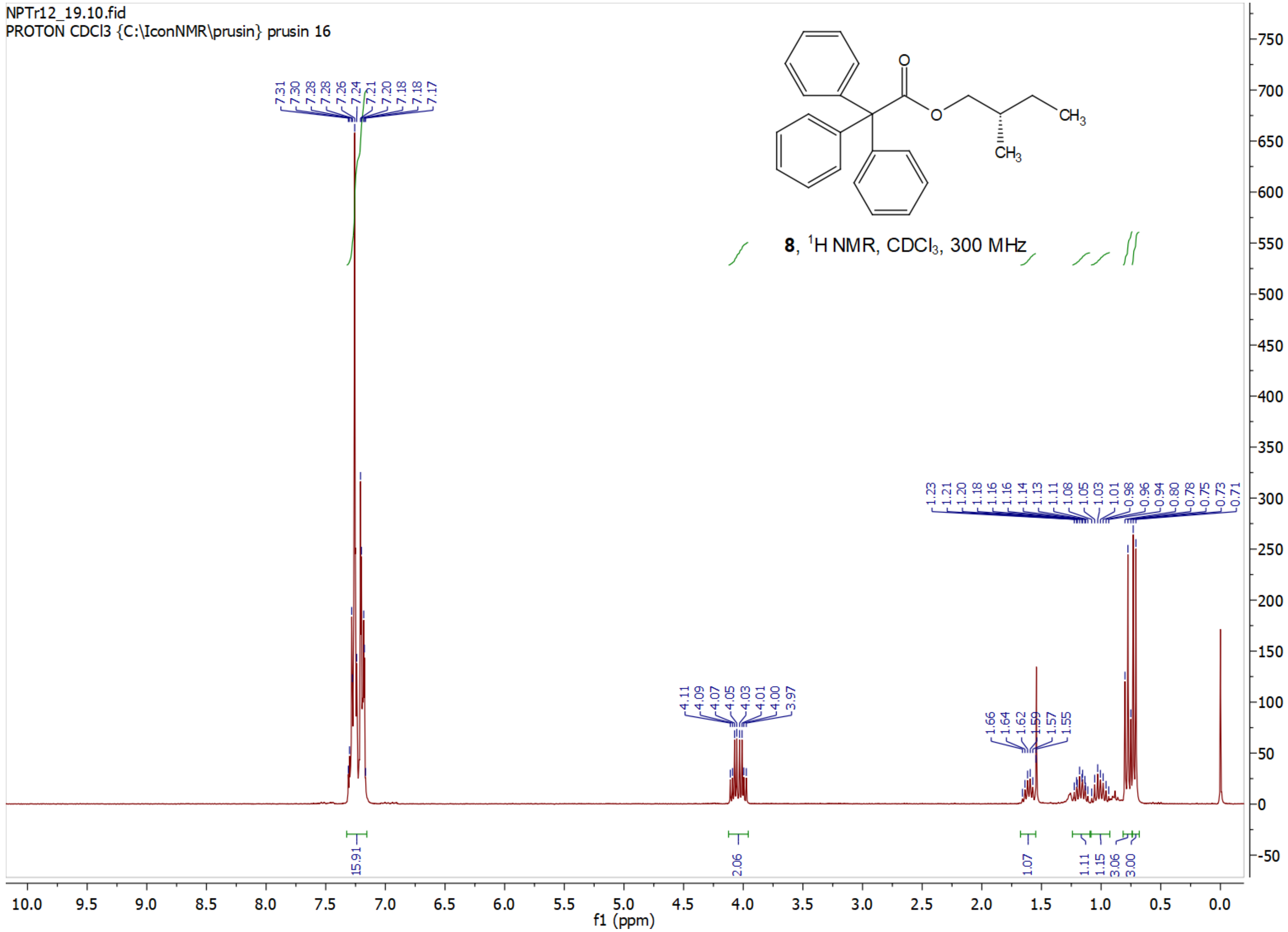
NPTr12_19.10.fid
PROTON CDCl3 {C:\IconNMR\prusin} prusin 16
8, 1H NMR, CDCl3, 300 MHz
O
O
CH3
CH3
f1 (ppm)
15.91
2.06
1.07
1.11
1.15
3.06
3.00

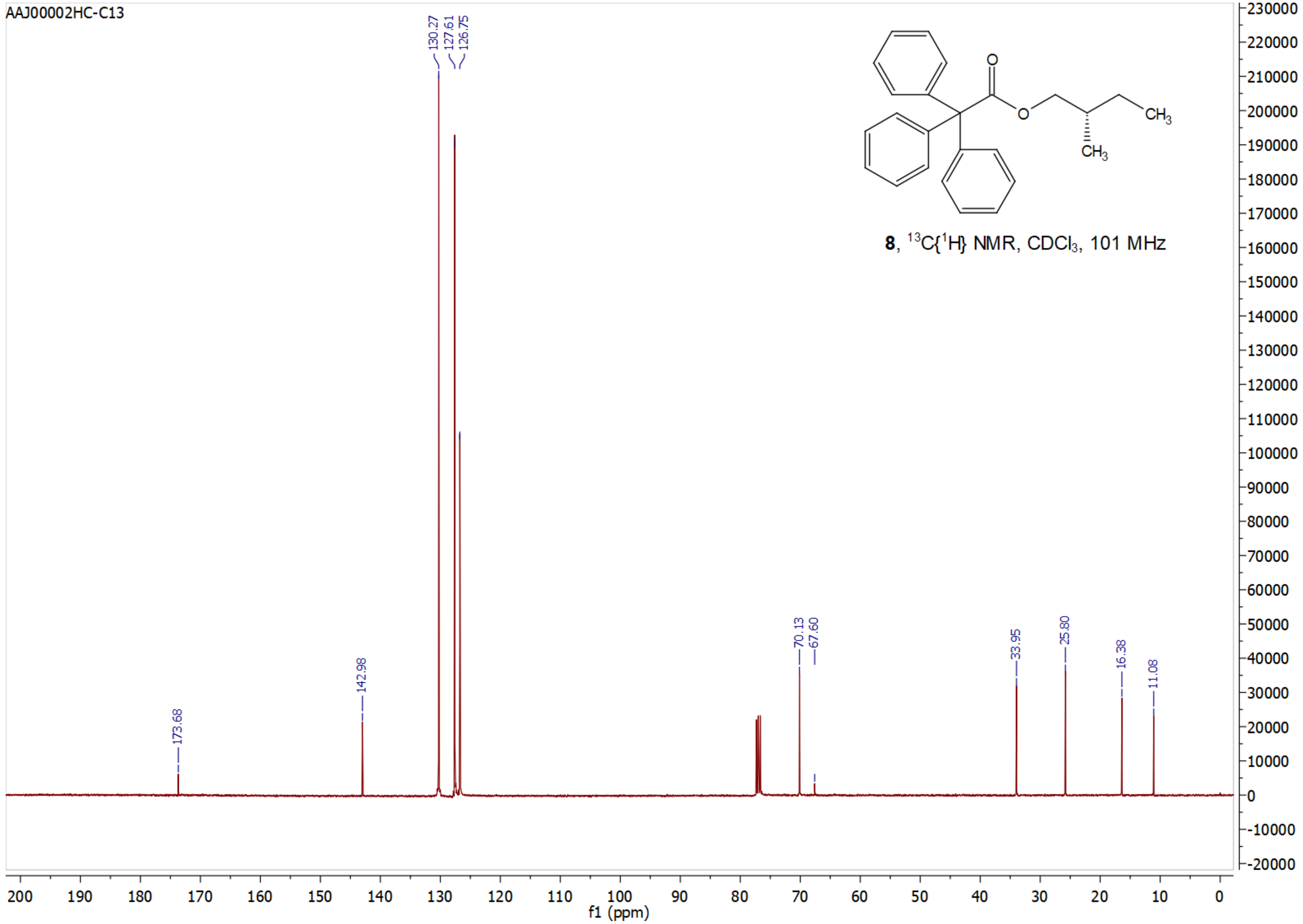
AAJ00002HC-C13
130.27
127.61
126.75
173.68
142.98
70.13
67.60
33.95
25.80
16.38
11.08
O
O
CH3
CH3
8, 13C{1H} NMR, CDCl3, 101 MHz
230000
220000
210000
200000
190000
180000
170000
160000
150000
140000
130000
120000
110000
100000
90000
80000
70000
60000
50000
40000
30000
20000
10000
0
-10000
-20000
200
190
180
170
160
150
140
130
120
110
100
90
80
70
60
50
40
30
20
10
0
f1 (ppm)

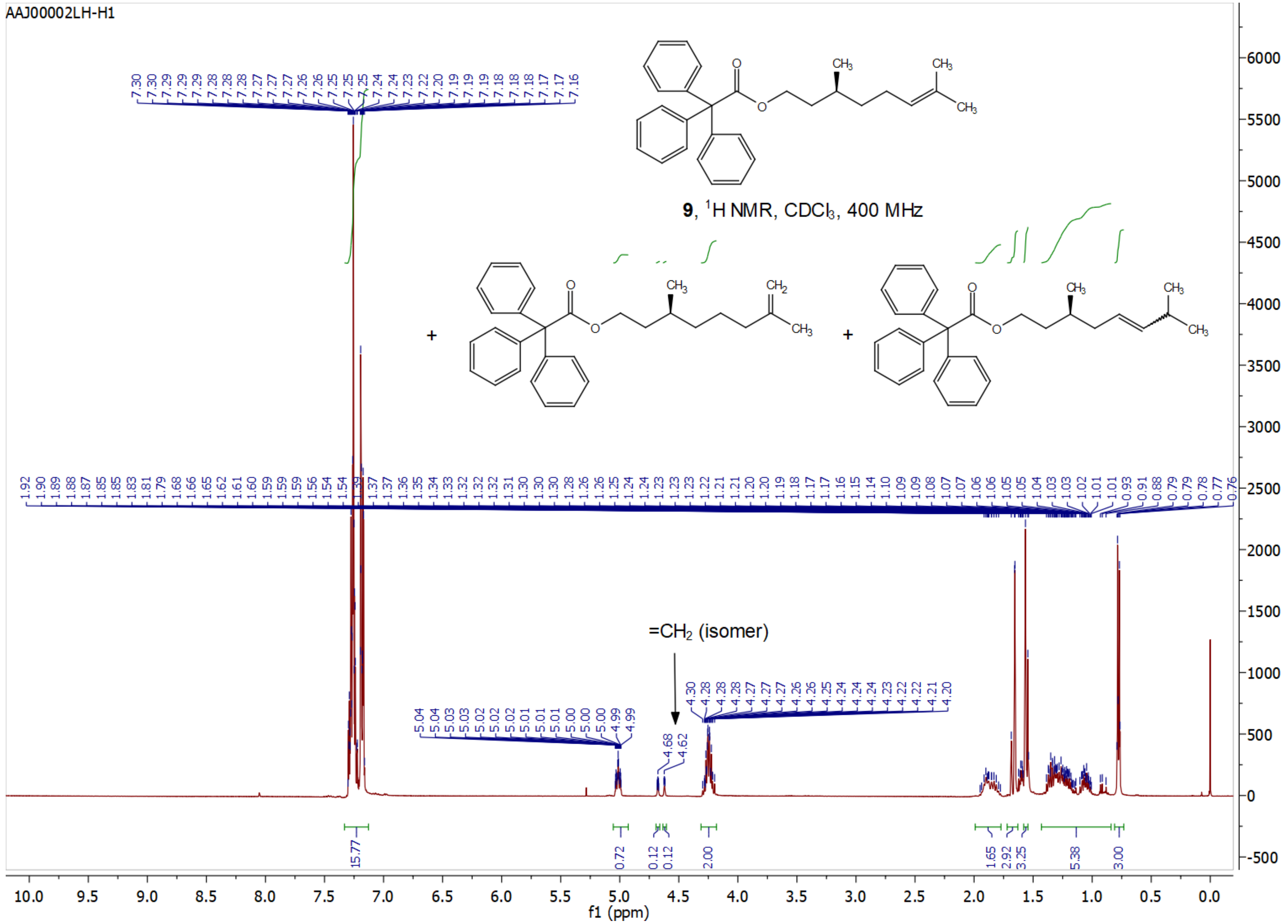
AAJ00002LH-H1
9, 1H NMR, CDCl3, 400 MHz
=CH2 (isomer)
f1 (ppm)

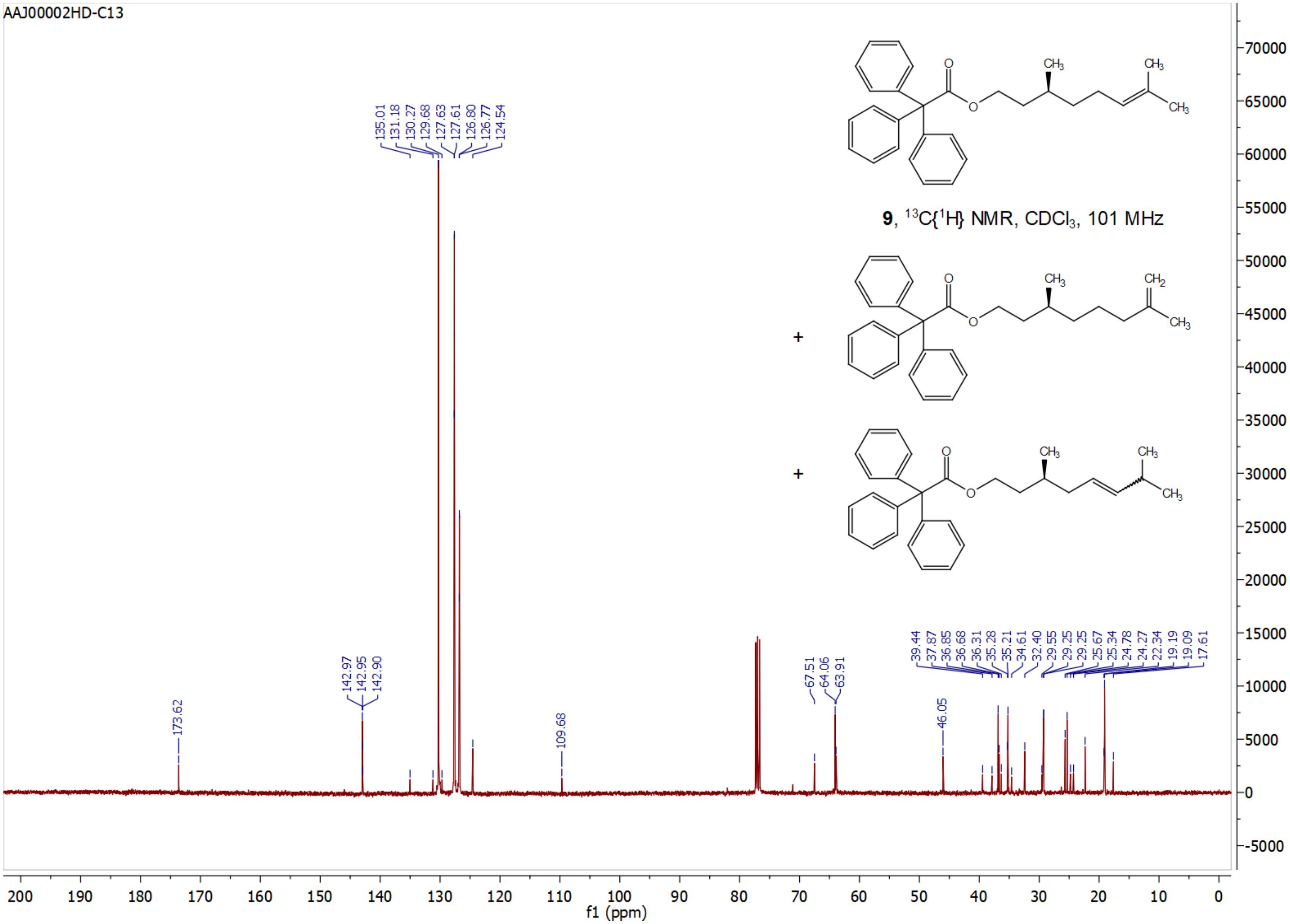
AAJ00002HD-C13
9, $^{13}C\{^{1}H\}$ NMR, $CDCl_3$, 101 MHz
173.62
142.97
142.95
142.90
135.01
131.18
130.27
129.68
127.63
127.61
126.80
126.77
124.54
109.68
67.51
64.06
63.91
46.05
39.44
37.87
36.85
36.68
36.31
35.28
35.21
34.61
32.40
29.55
29.25
29.25
25.67
25.34
24.78
24.27
22.34
19.19
19.09
17.61
f1 (ppm)

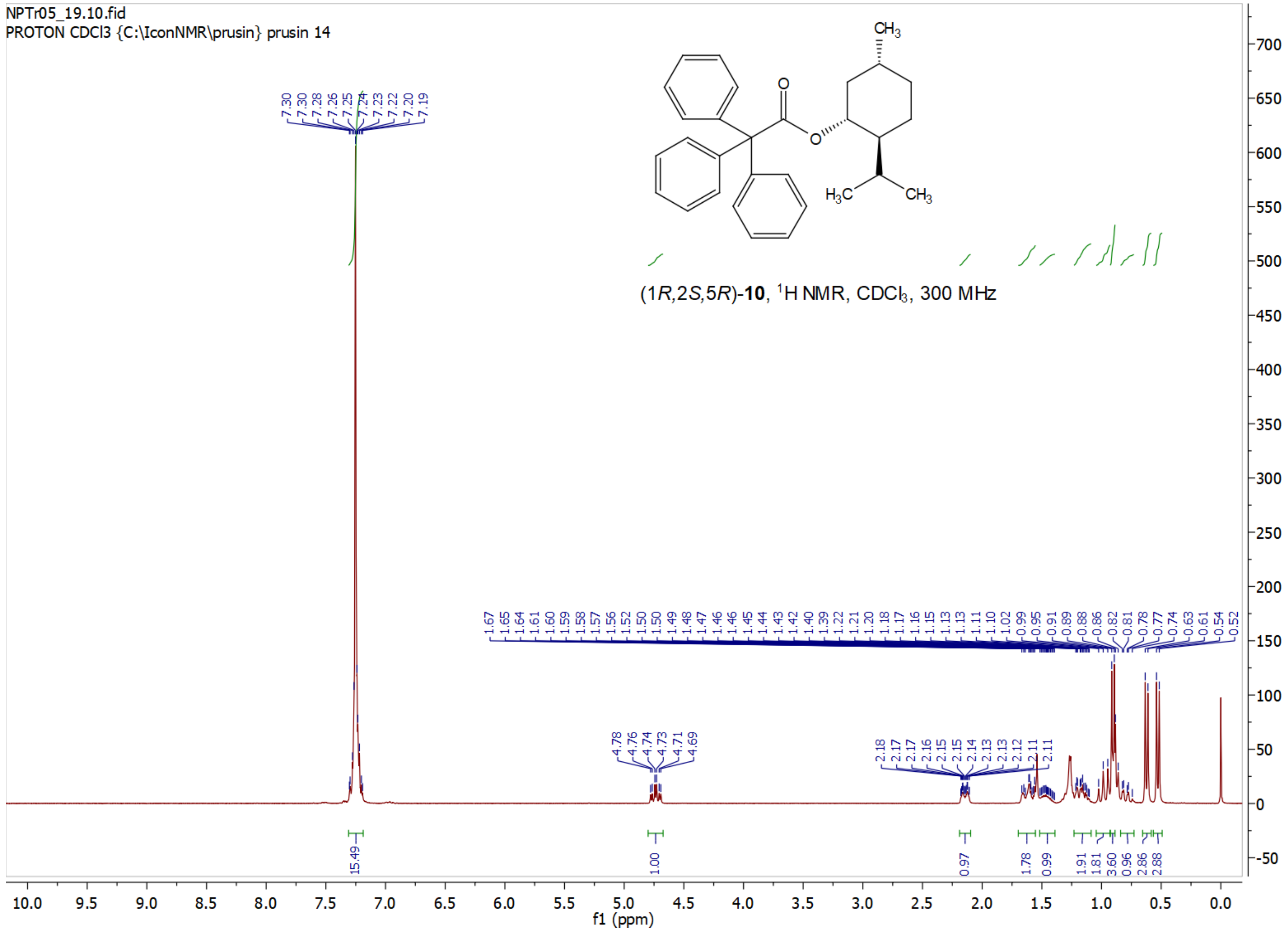

NPTr05_19.10.fid
PROTON CDCl3 {C:\IconNMR\prusin} prusin 14
(1R,2S,5R)-10, 1H NMR, CDCl3, 300 MHz
f1 (ppm)

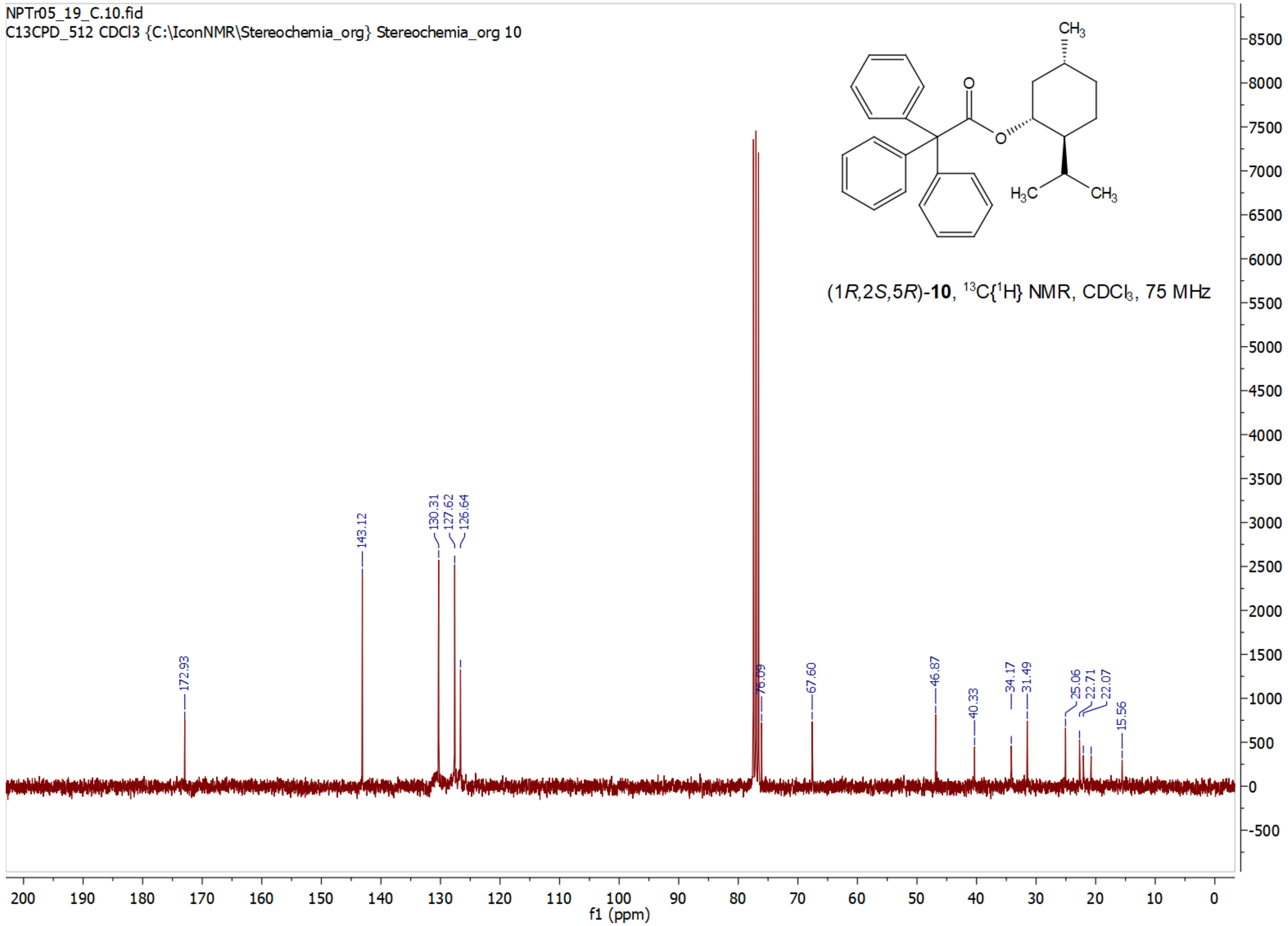

NPTr05_19_C.10.fid
C13CPD_512 CDCl3 {C:\IconNMR\Stereochemia_org} Stereochemia_org 10
(1R,2S,5R)-10, 13C{1H} NMR, CDCl3, 75 MHz
CH3
O
O
H3C
CH3
172.93
143.12
130.31
127.62
126.64
76.09
67.60
46.87
40.33
34.17
31.49
25.06
22.71
22.07
15.56
f1 (ppm)

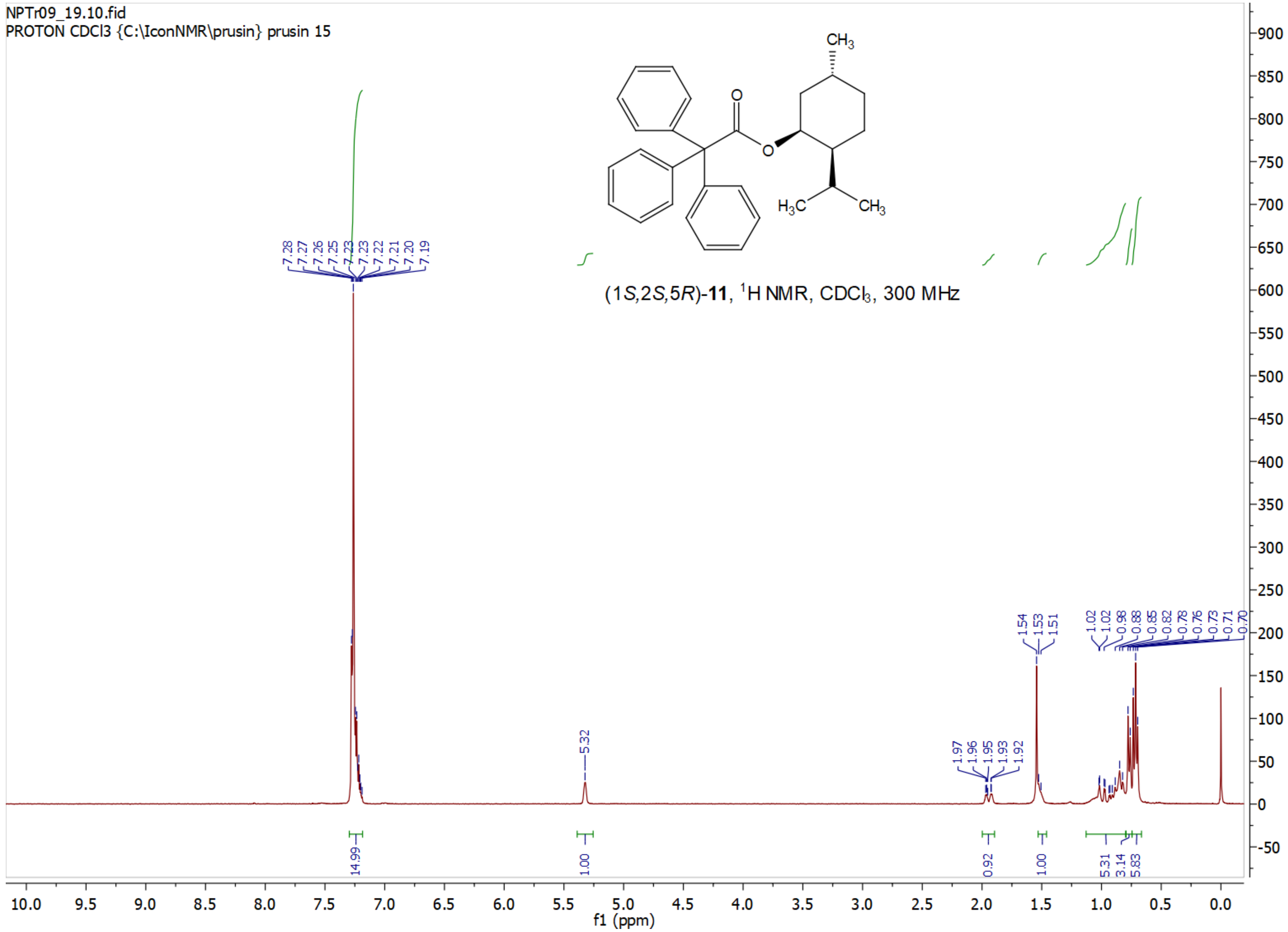
NPTr09_19.10.fid
PROTON CDCl3 {C:\IconNMR\prusin} prusin 15
(1S,2S,5R)-11, 1H NMR, CDCl3, 300 MHz
CH3
O
O
H3C
CH3
7.28 7.27 7.26 7.25 7.23 7.23 7.22 7.21 7.20 7.19
5.32
1.97 1.96 1.95 1.93 1.92
1.54 1.53 1.51
1.02 1.02 0.98 0.88 0.85 0.82 0.78 0.76 0.73 0.71 0.70
14.99
1.00
0.92
1.00
5.31 3.14 5.83
f1 (ppm)

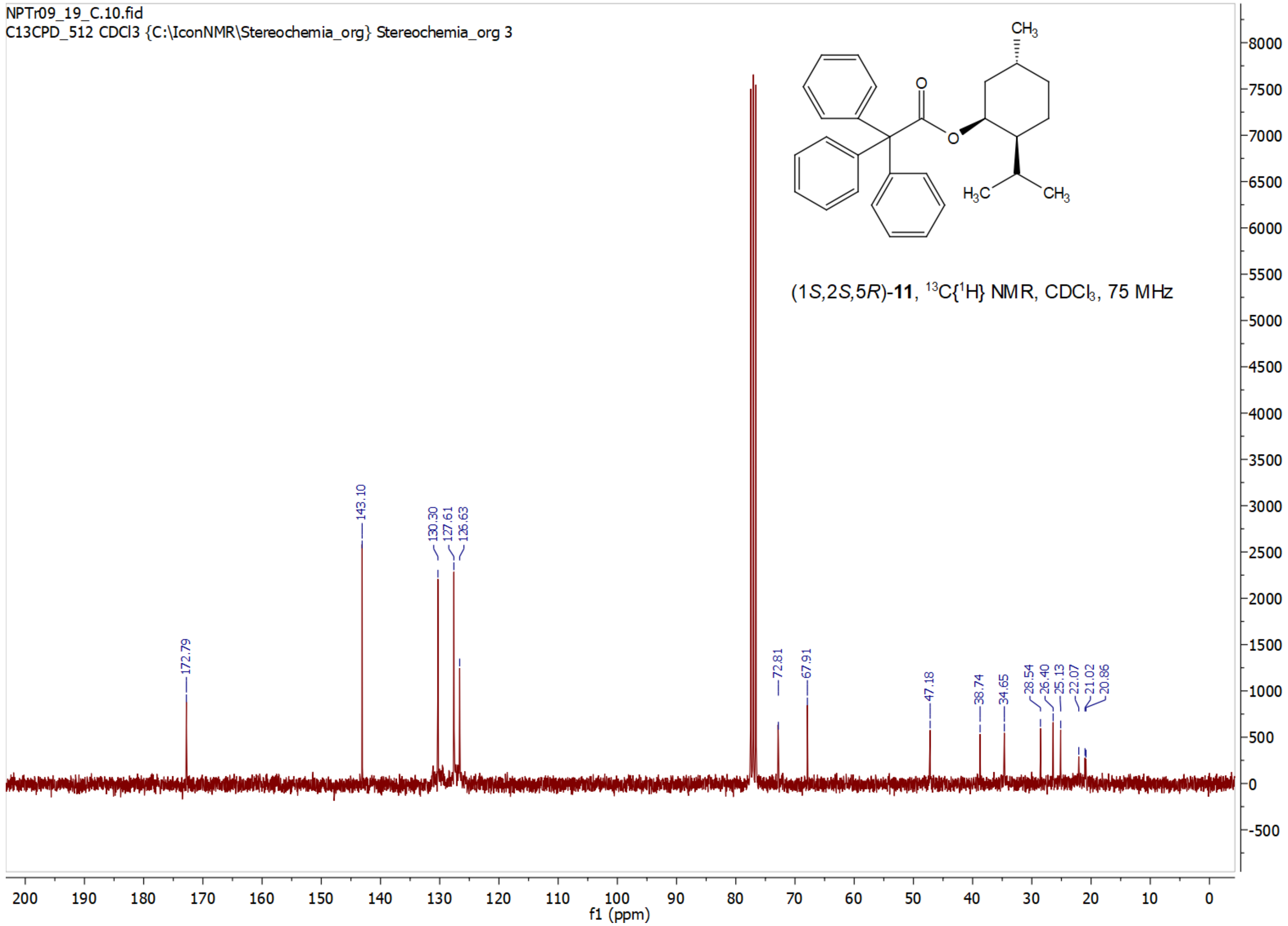

NPTr09_19_C.10.fid
C13CPD_512 CDCl3 {C:\IconNMR\Stereochemia_org} Stereochemia_org 3
(1S,2S,5R)-11, 13C{1H} NMR, CDCl3, 75 MHz
172.79
143.10
130.30
127.61
126.63
72.81
67.91
47.18
38.74
34.65
28.54
26.40
25.13
22.07
21.02
20.86
f1 (ppm)

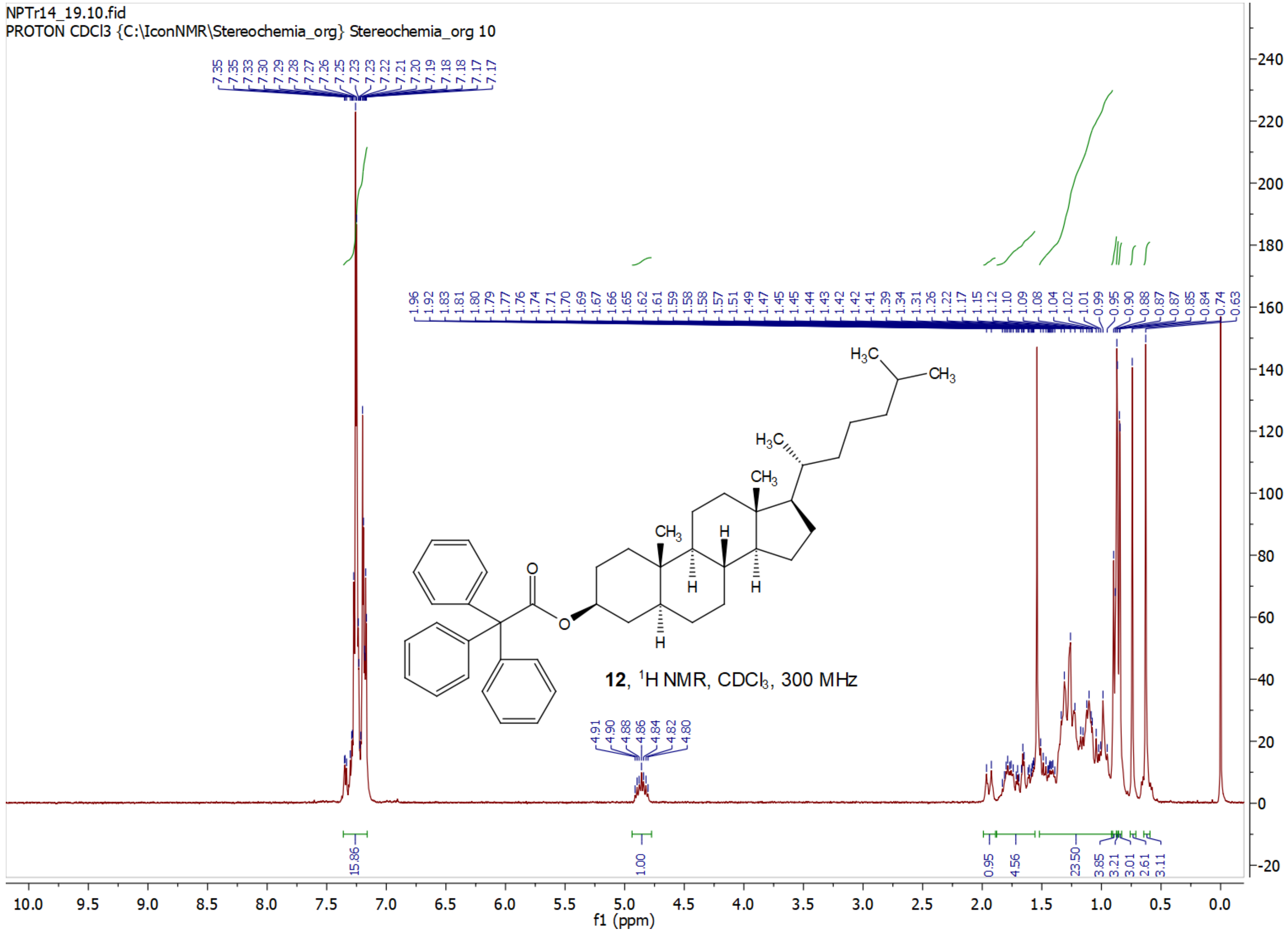

NPTr14_19.10.fid
PROTON CDCl3 {C:\IconNMR\Stereochemia_org} Stereochemia_org 10
12, 1H NMR, CDCl3, 300 MHz
f1 (ppm)

NPTr14_19_C.10.fid
C13CPD_512 CDCl3 {C:\IconNMR\Stereochemia_org} Stereochemia_org 5

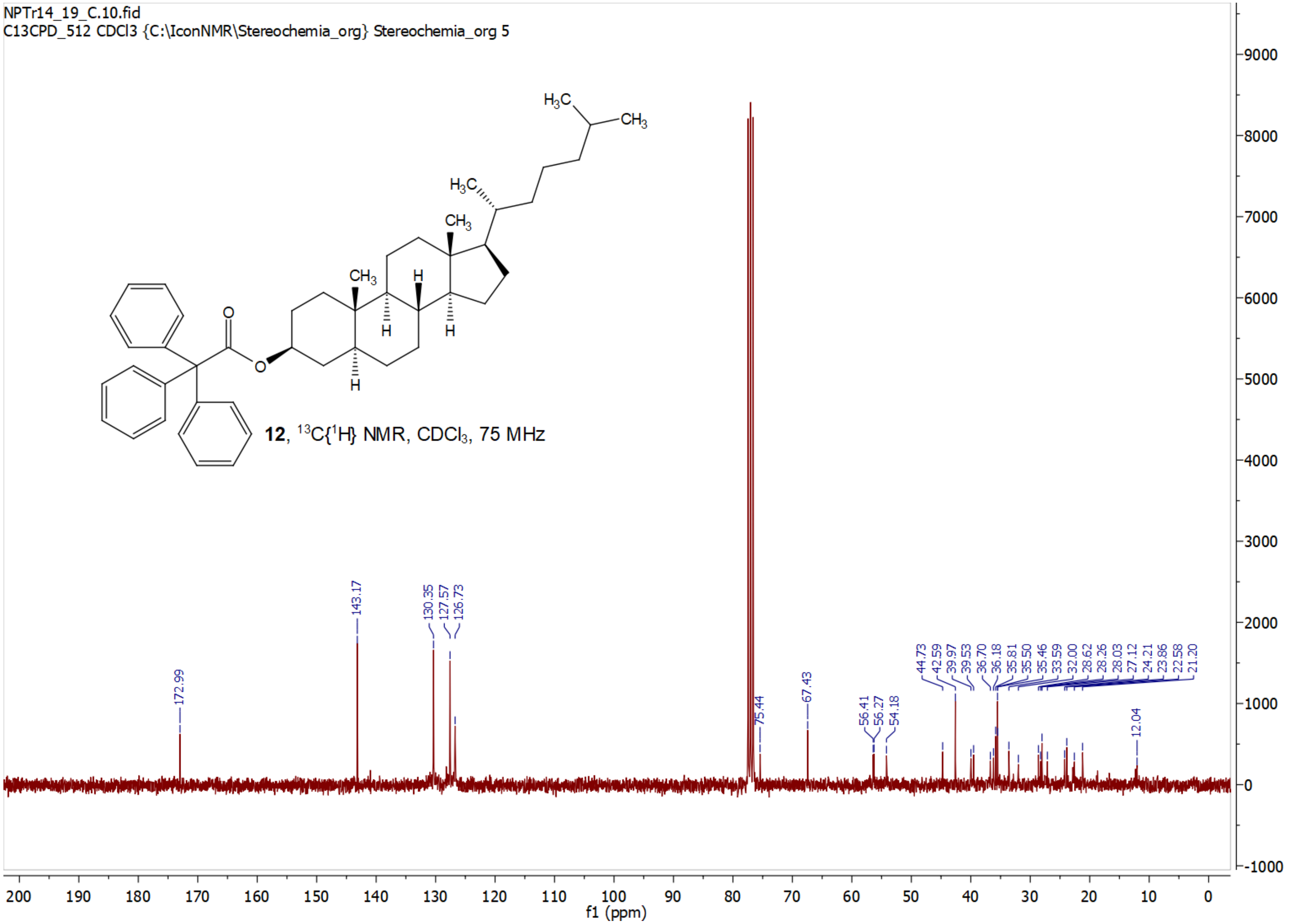

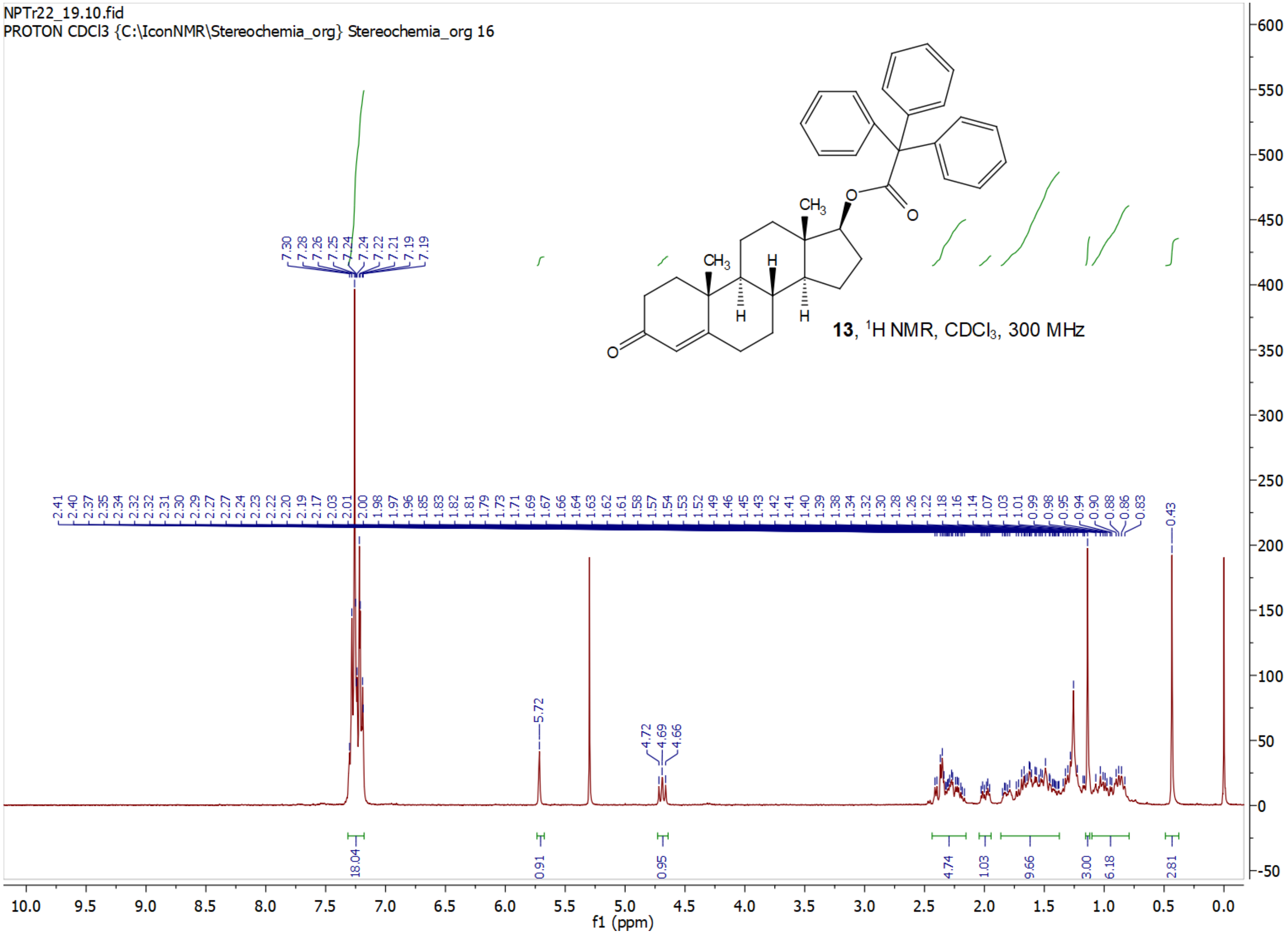

NPTr22_19.10.fid
PROTON CDCl3 {C:\IconNMR\Stereochemia_org} Stereochemia_org 16
13, ^{1}H NMR, CDCl$_3$, 300 MHz
7.30 7.28 7.26 7.25 7.24 7.24 7.22 7.21 7.19 7.19
5.72
4.72 4.69 4.66
2.41 2.40 2.37 2.35 2.34 2.32 2.32 2.31 2.30 2.29 2.27 2.27 2.24 2.23 2.22 2.20 2.19 2.17 2.03 2.01 2.00 1.98 1.97 1.96 1.85 1.83 1.82 1.81 1.79 1.73 1.71 1.69 1.67 1.66 1.64 1.63 1.62 1.61 1.58 1.57 1.54 1.53 1.52 1.49 1.46 1.45 1.43 1.42 1.41 1.40 1.39 1.38 1.34 1.32 1.30 1.28 1.26 1.22 1.18 1.16 1.14 1.07 1.03 1.01 0.99 0.98 0.95 0.94 0.90 0.88 0.86 0.83
0.43
18.04
0.91
0.95
4.74
1.03
9.66
3.00
6.18
2.81
f1 (ppm)

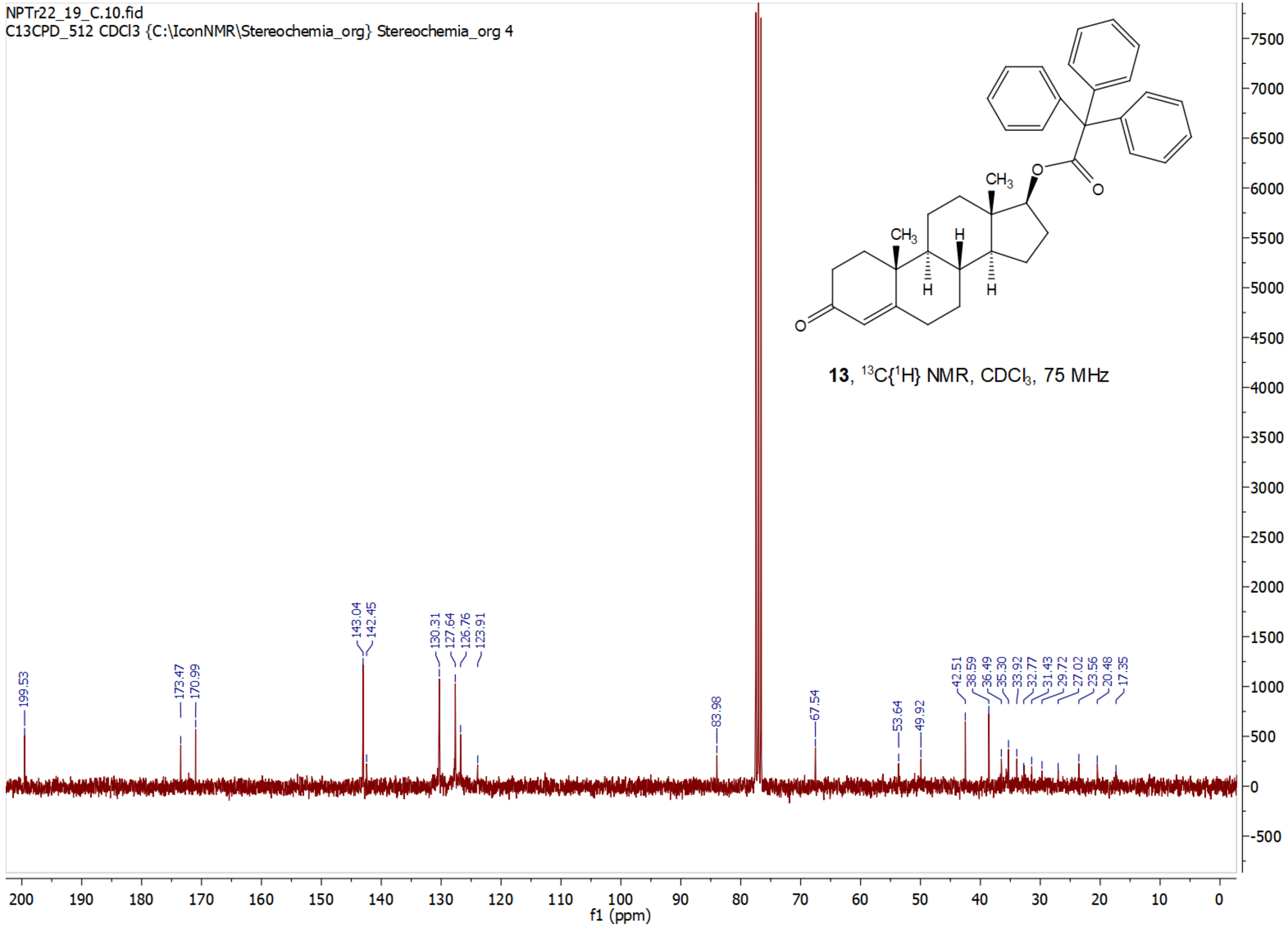

NPTr22_19_C.10.fid
C13CPD_512 CDCl3 {C:\IconNMR\Stereochemia_org} Stereochemia_org 4
CH3
CH3
H
H
H
O
O
O
13, 13C{1H} NMR, CDCl3, 75 MHz
199.53
173.47
170.99
143.04
142.45
130.31
127.64
126.76
123.91
83.98
67.54
53.64
49.92
42.51
38.59
36.49
35.30
33.92
32.77
31.43
29.72
27.02
23.56
20.48
17.35
f1 (ppm)

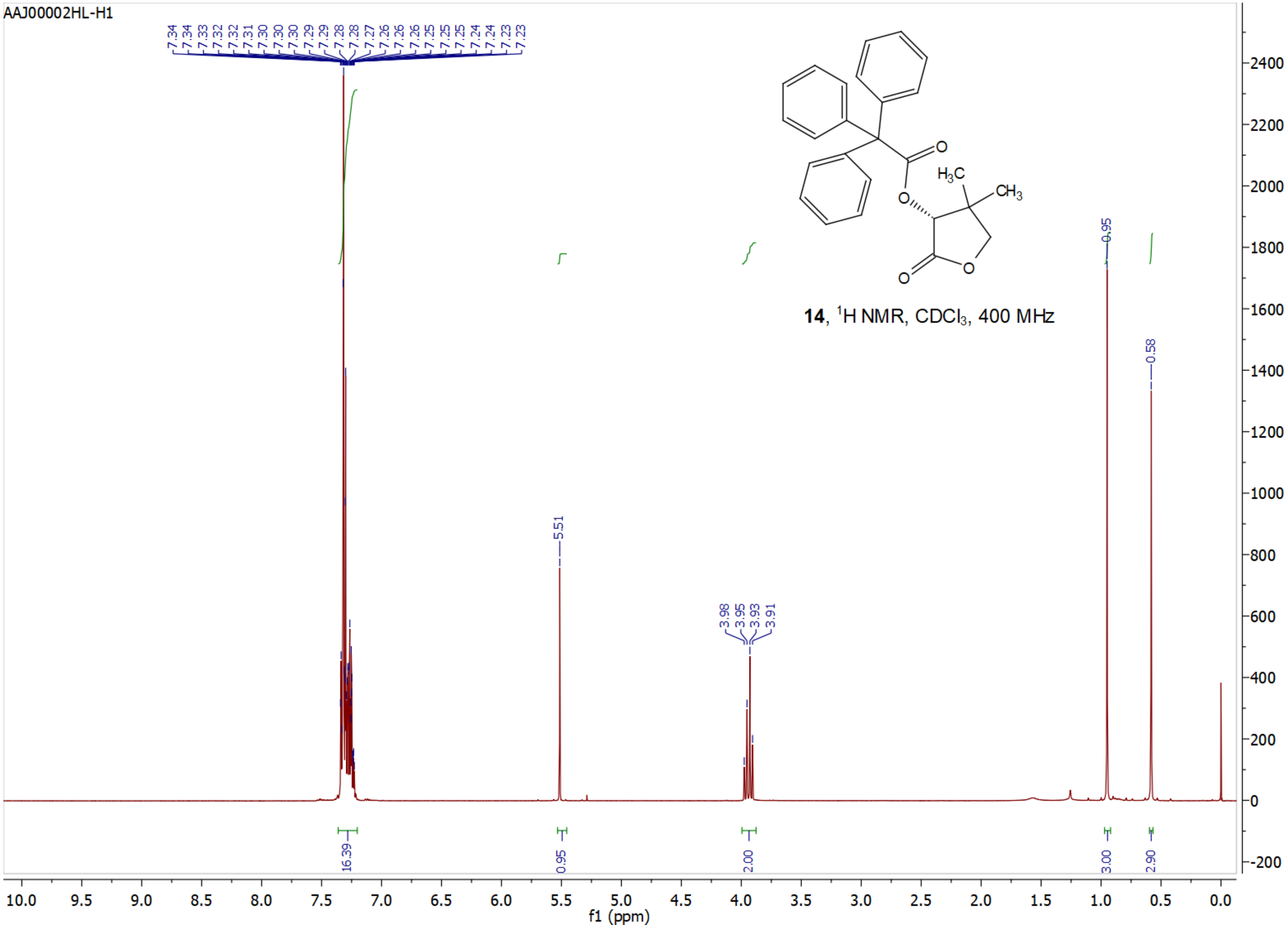

14, ^{1}H NMR, $CDCl_3$, 400 MHz

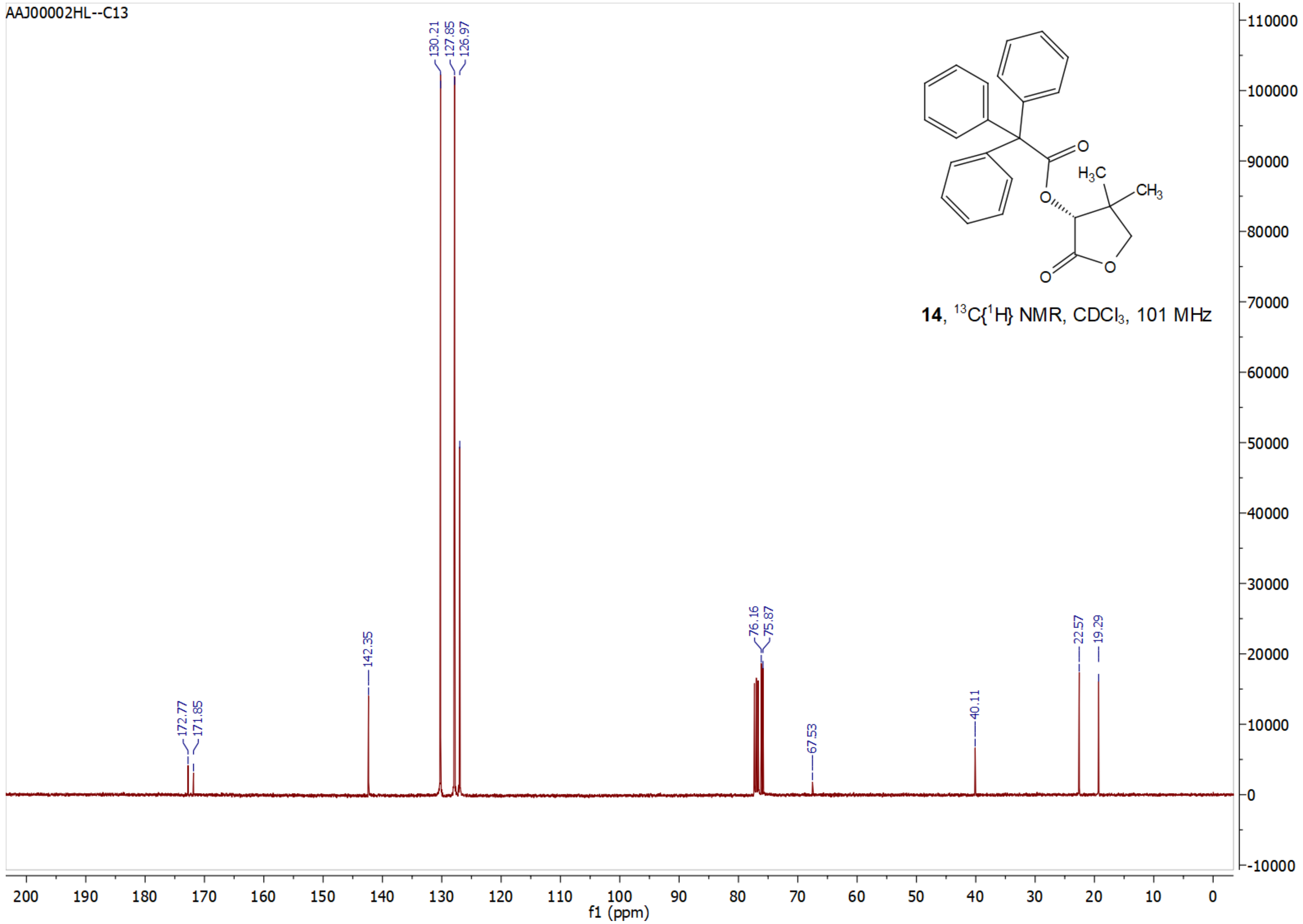

AAJ00002HL--C13
130.21
127.85
126.97
172.77
171.85
142.35
76.16
75.87
67.53
40.11
22.57
19.29
H3C
CH3
O
14, 13C{1H} NMR, CDCl3, 101 MHz
f1 (ppm)

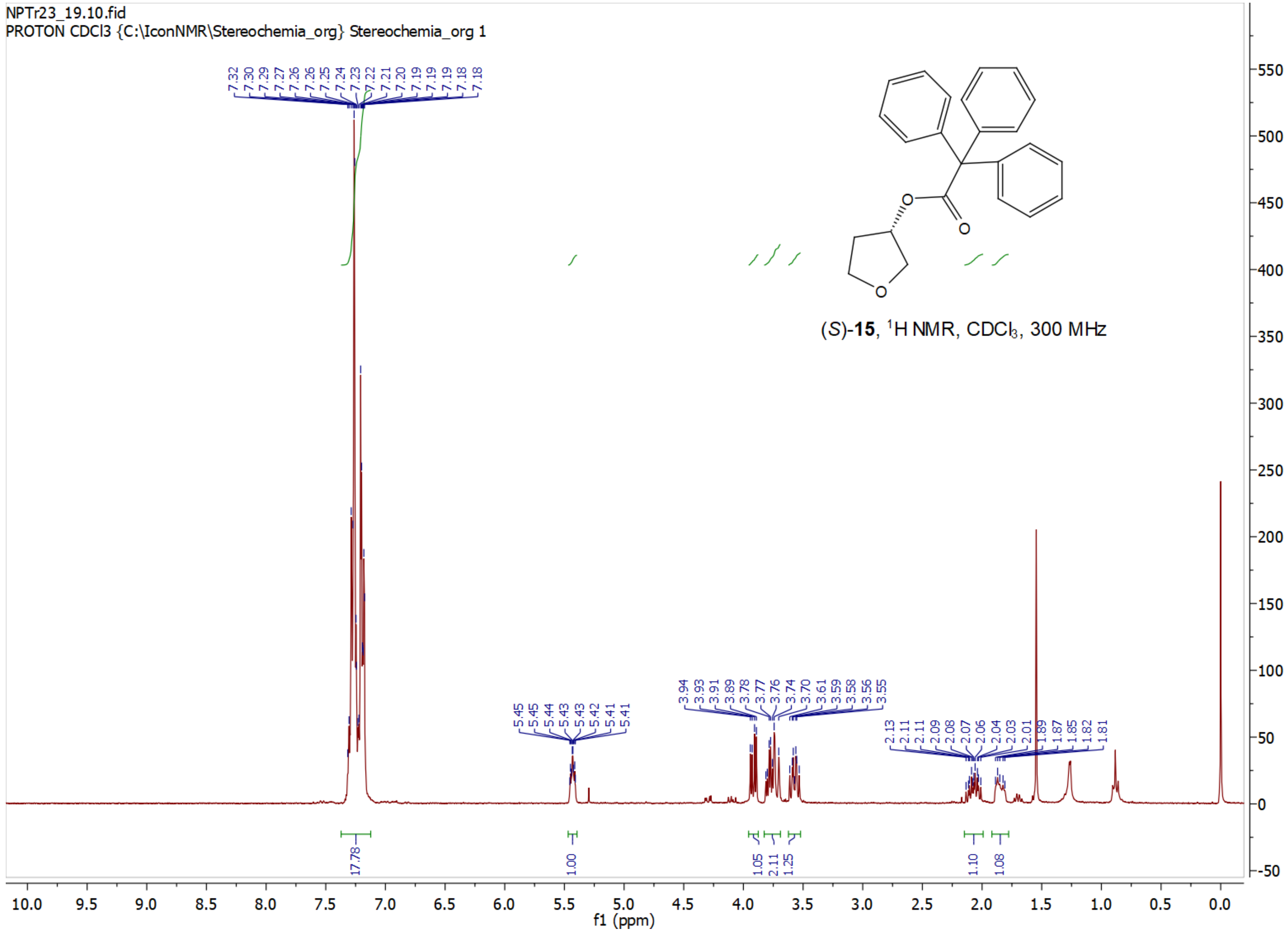

NPTr23_19.10.fid
PROTON CDCl3 {C:\IconNMR\Stereochemia_org} Stereochemia_org 1
(S)-15, 1H NMR, CDCl3, 300 MHz
f1 (ppm)

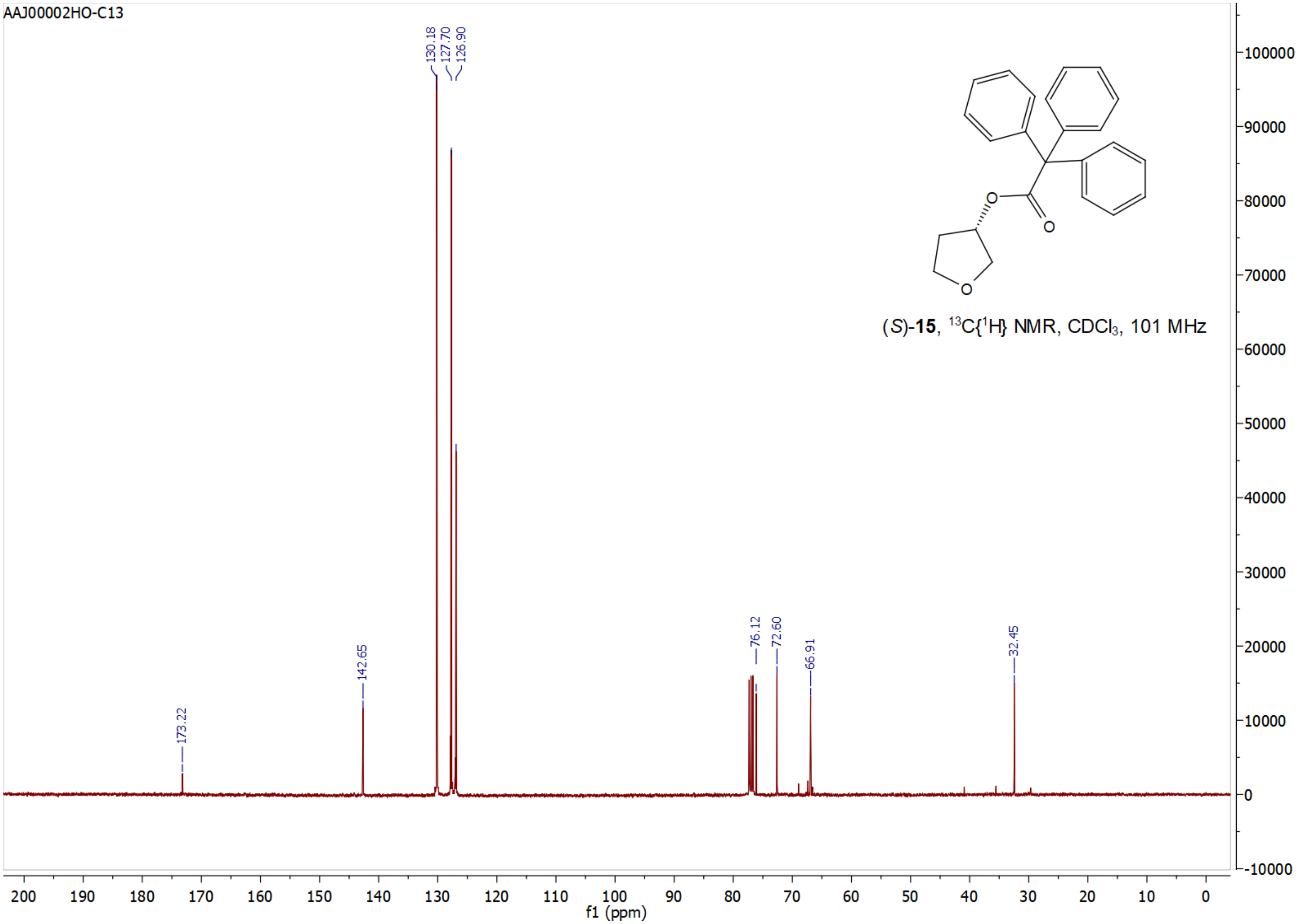
AAJ00002HO-C13
130.18
127.70
126.90
173.22
142.65
76.12
72.60
66.91
32.45
O
O
O
(S)-15, 13C{1H} NMR, CDCl3, 101 MHz
100000
90000
80000
70000
60000
50000
40000
30000
20000
10000
0
-10000
200
190
180
170
160
150
140
130
120
110
100
90
80
70
60
50
40
30
20
10
0
f1 (ppm)

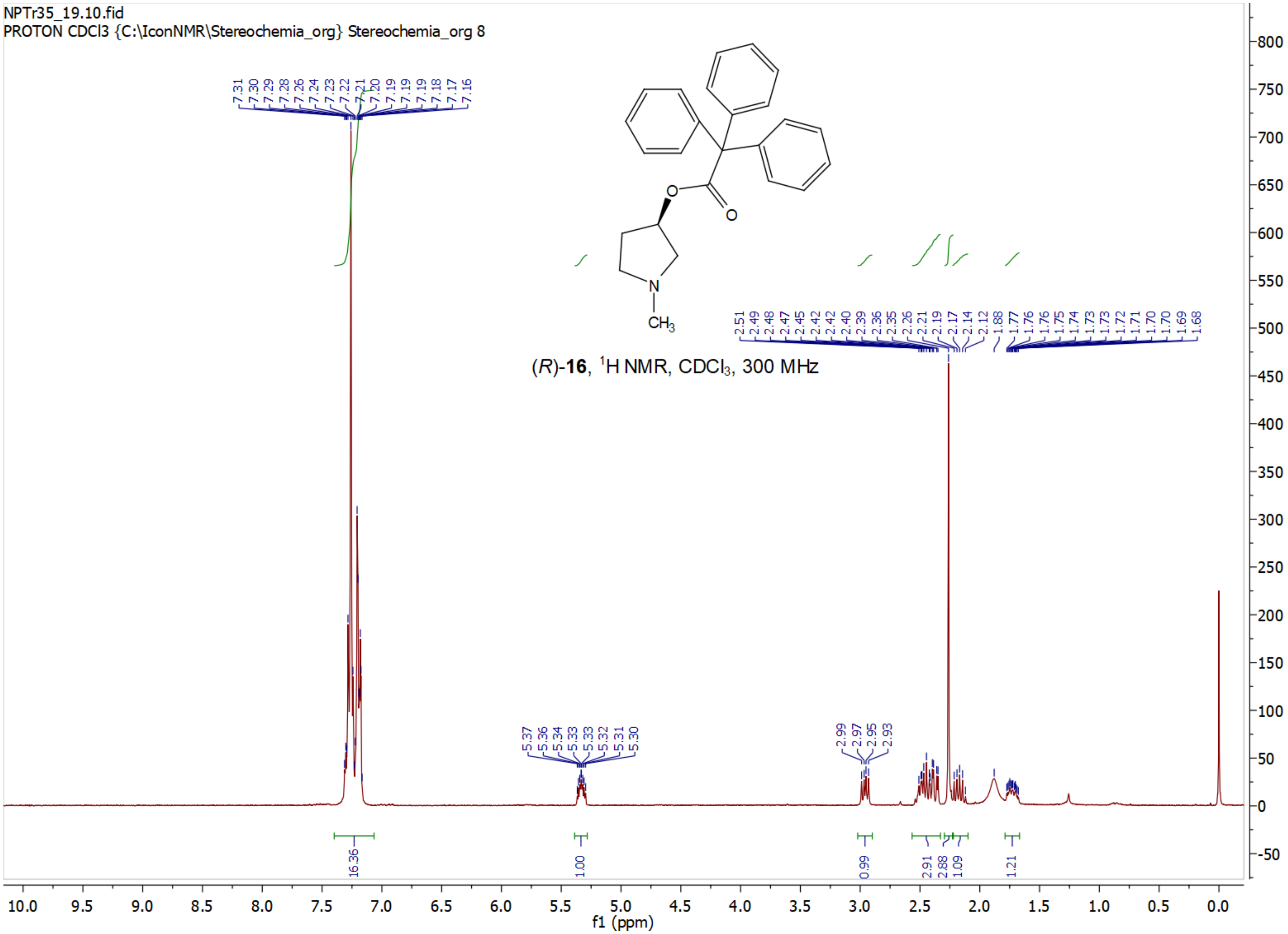
NPTr35_19.10.fid
PROTON CDCl3 {C:\IconNMR\Stereochemia_org} Stereochemia_org 8
(R)-16, 1H NMR, CDCl3, 300 MHz
f1 (ppm)

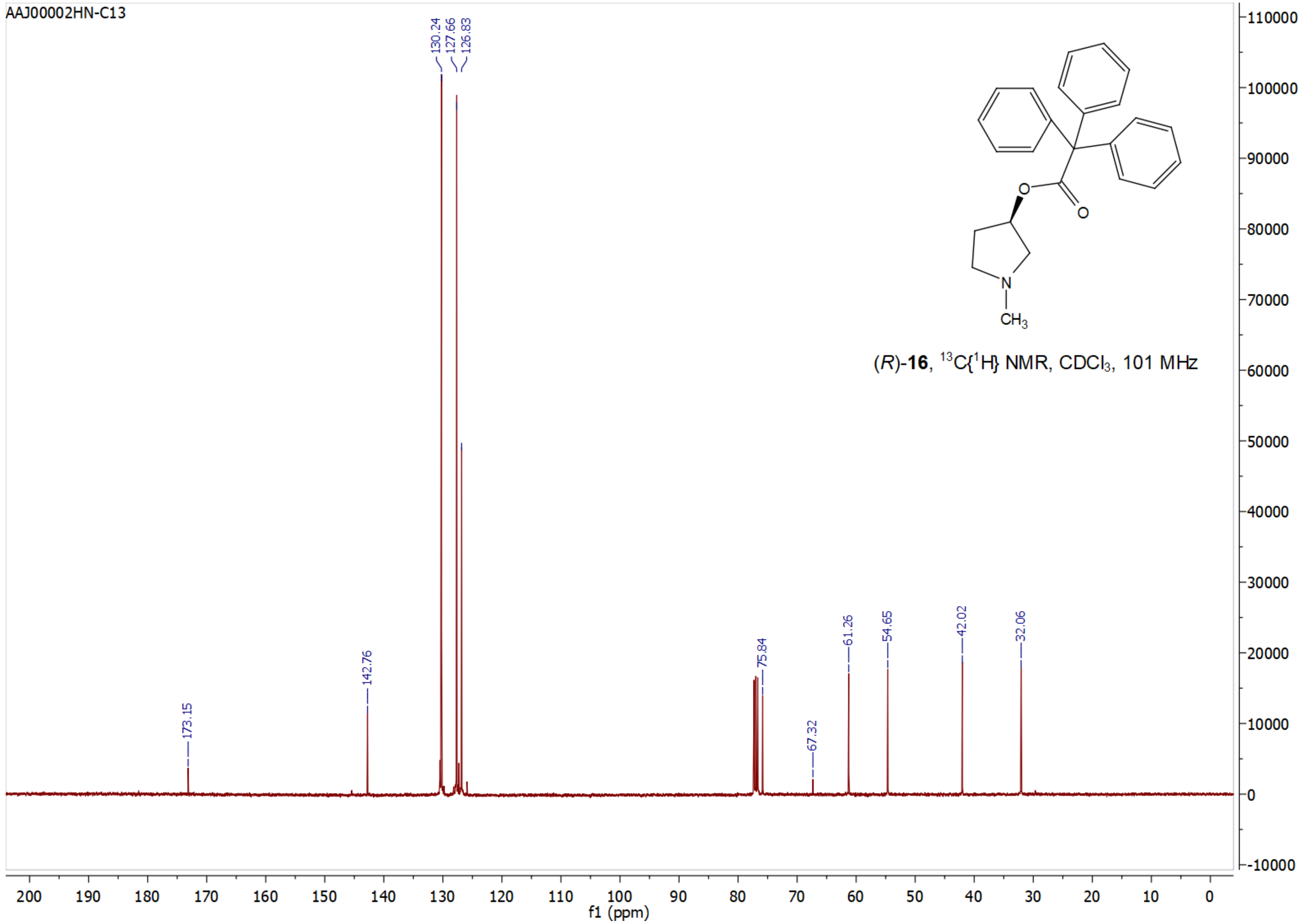
AAJ00002HN-C13
130.24
127.66
126.83
173.15
142.76
75.84
67.32
61.26
54.65
42.02
32.06
O
O
N
CH3
(R)-16, 13C{1H} NMR, CDCl3, 101 MHz
110000
100000
90000
80000
70000
60000
50000
40000
30000
20000
10000
0
-10000
200 190 180 170 160 150 140 130 120 110 100 90 80 70 60 50 40 30 20 10 0
f1 (ppm)

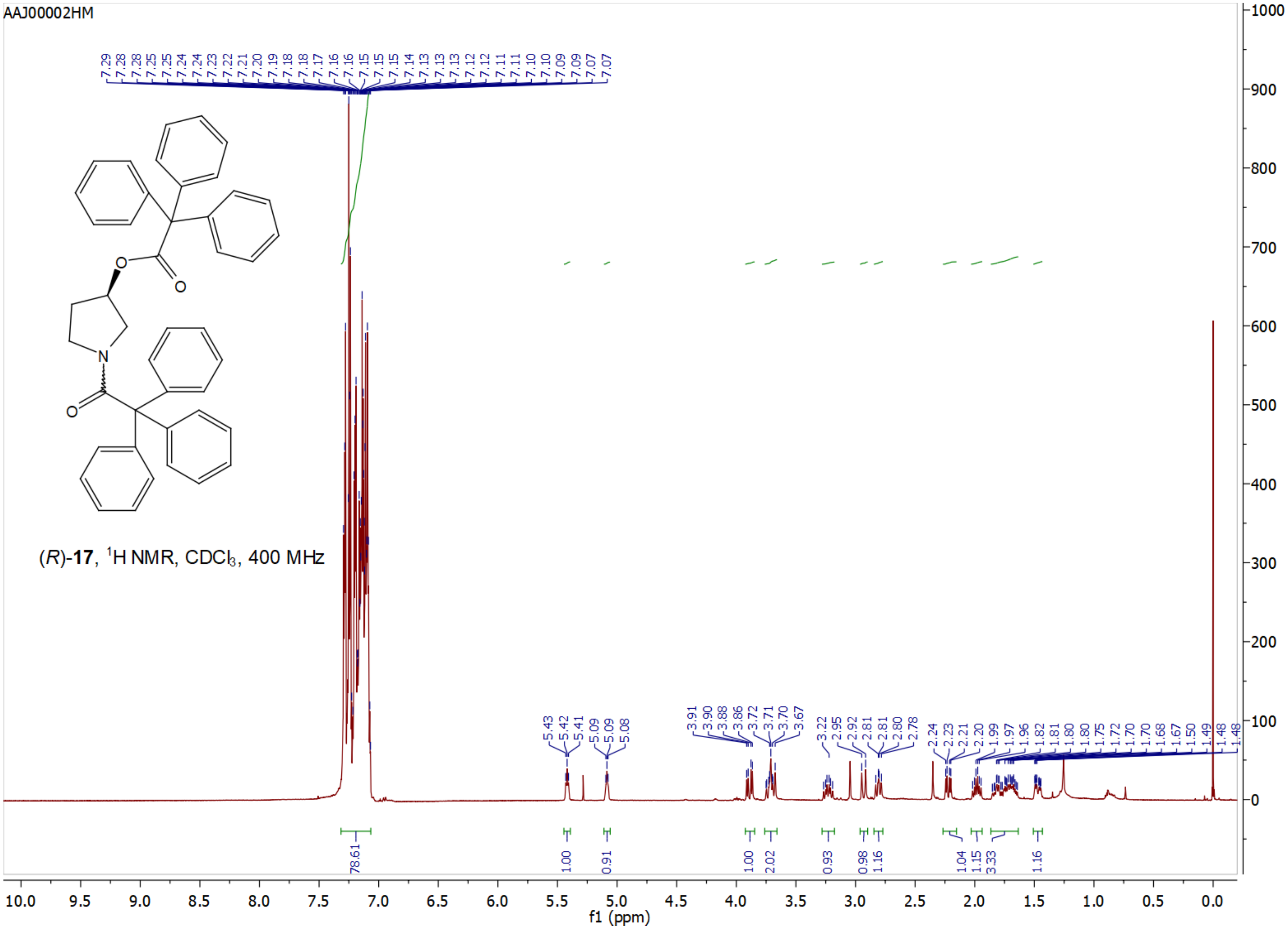
AAJ00002HM
(R)-17, ¹H NMR, CDCl3, 400 MHz
7.29 7.28 7.28 7.25 7.25 7.24 7.24 7.23 7.22 7.21 7.20 7.19 7.18 7.18 7.17 7.16 7.16 7.15 7.15 7.15 7.14 7.13 7.13 7.13 7.12 7.12 7.11 7.11 7.10 7.10 7.09 7.09 7.07 7.07
5.43 5.42 5.41 5.09 5.09 5.08
3.91 3.90 3.88 3.86 3.72 3.71 3.70 3.67
3.22 2.95 2.92 2.81 2.81 2.80 2.78
2.24 2.23 2.21 2.20 1.99 1.97 1.96 1.82 1.81 1.80 1.75 1.72 1.70 1.70 1.68 1.67 1.50 1.49 1.48 1.48
78.61 1.00 0.91 1.00 2.02 0.93 0.98 1.16 1.04 1.15 3.33 1.16
f1 (ppm)

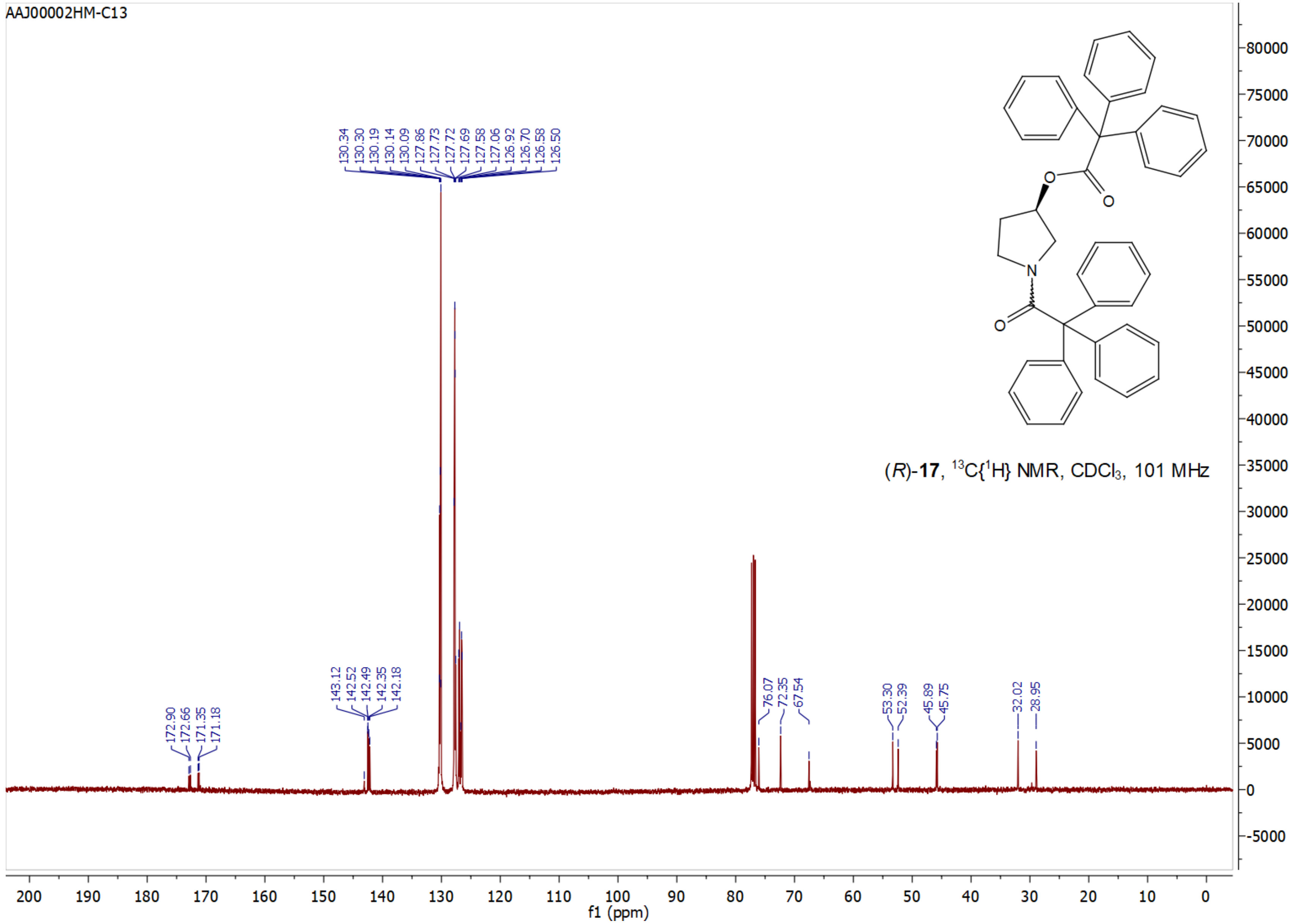

AAJ00002HM-C13
(R)-17, 13C{1H} NMR, CDCl3, 101 MHz
172.90
172.66
171.35
171.18
143.12
142.52
142.49
142.35
142.18
130.34
130.30
130.19
130.14
130.09
127.86
127.73
127.72
127.69
127.58
127.06
126.92
126.70
126.58
126.50
76.07
72.35
67.54
53.30
52.39
45.89
45.75
32.02
28.95
f1 (ppm)

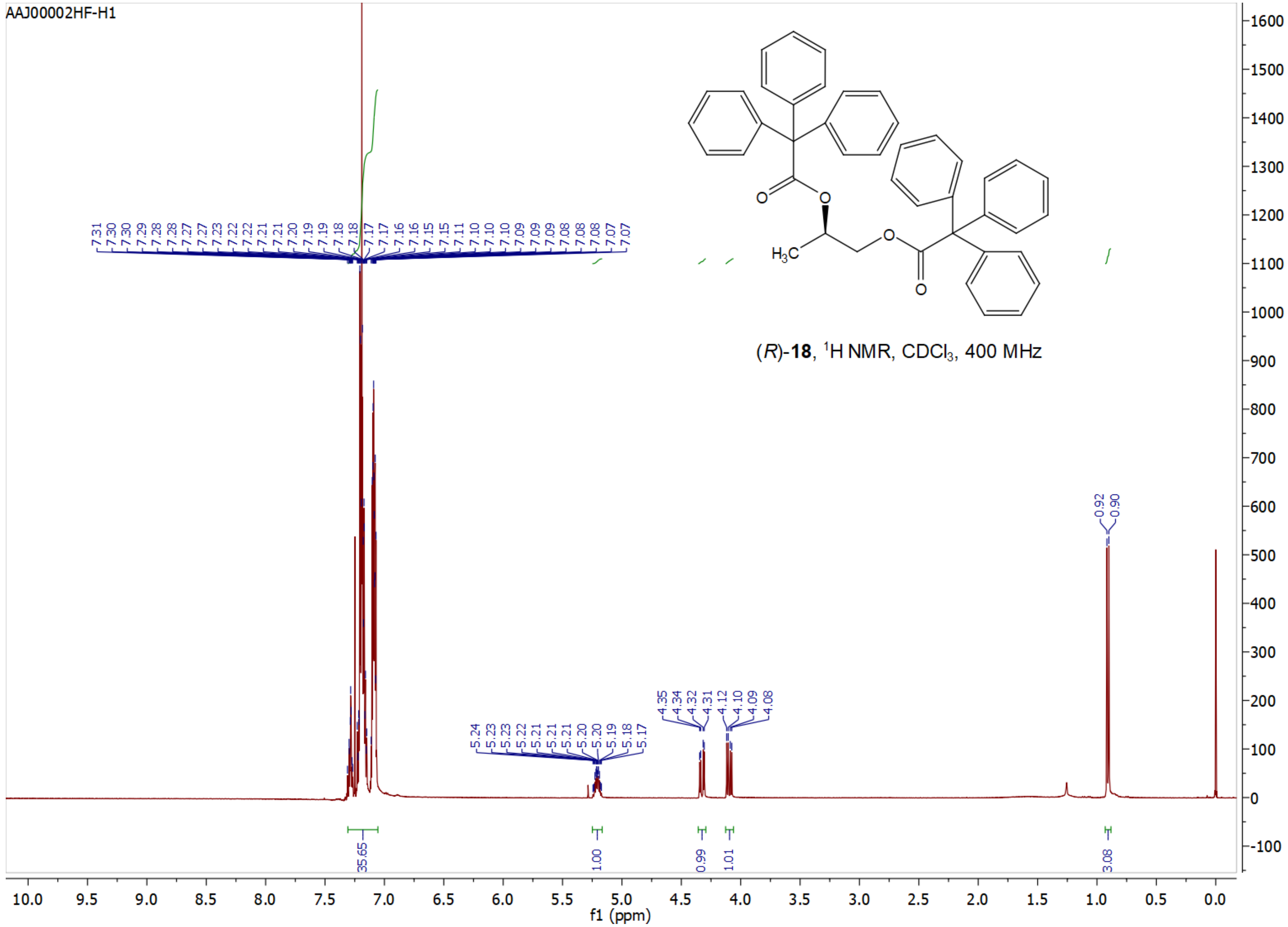

AAJ00002HF-H1
(R)-18, ¹H NMR, CDCl₃, 400 MHz
f1 (ppm)

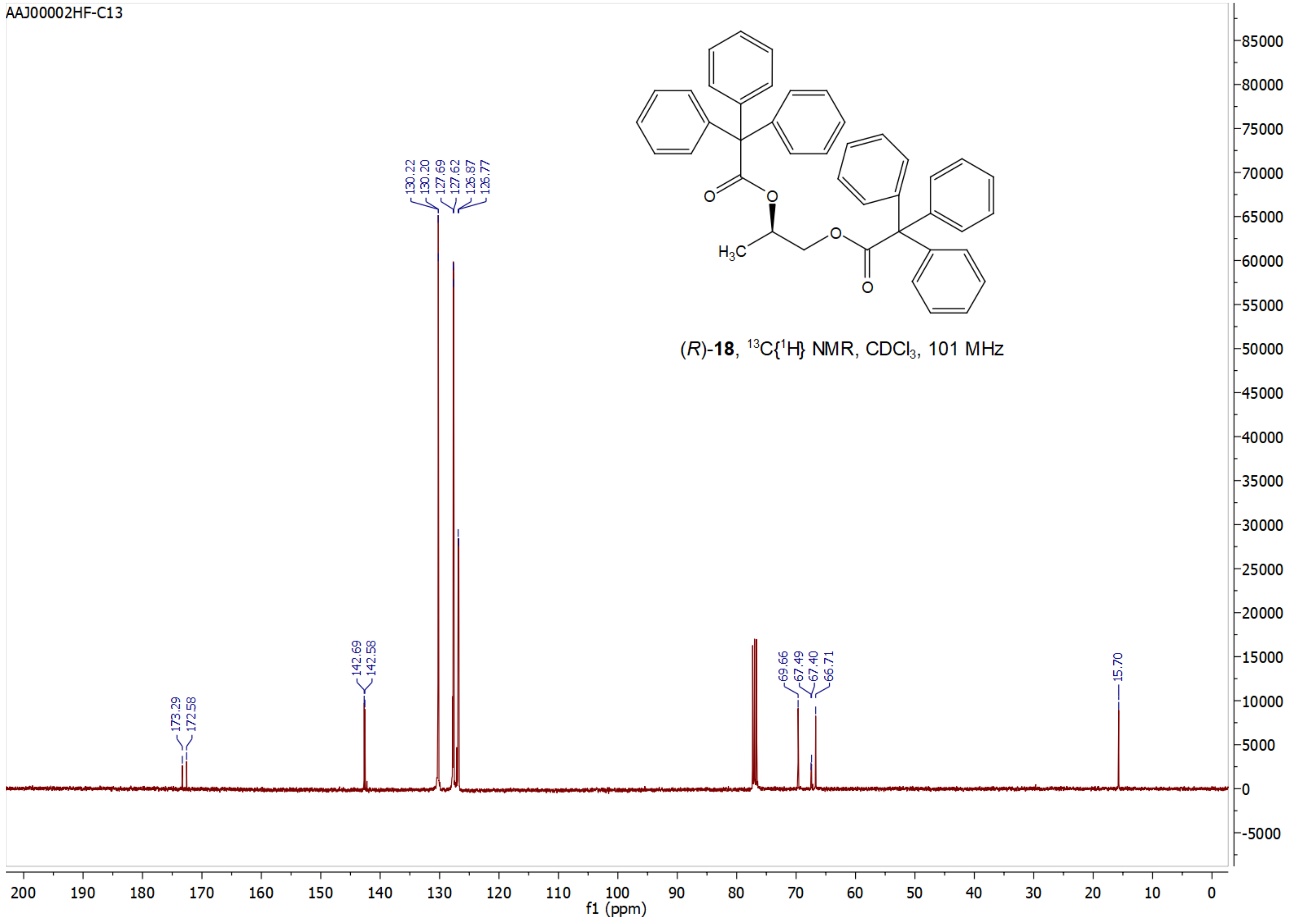
AAJ00002HF-C13
(R)-18, 13C{1H} NMR, CDCl3, 101 MHz
130.22
130.20
127.69
127.62
126.87
126.77
173.29
172.58
142.69
142.58
69.66
67.49
67.40
66.71
15.70
f1 (ppm)

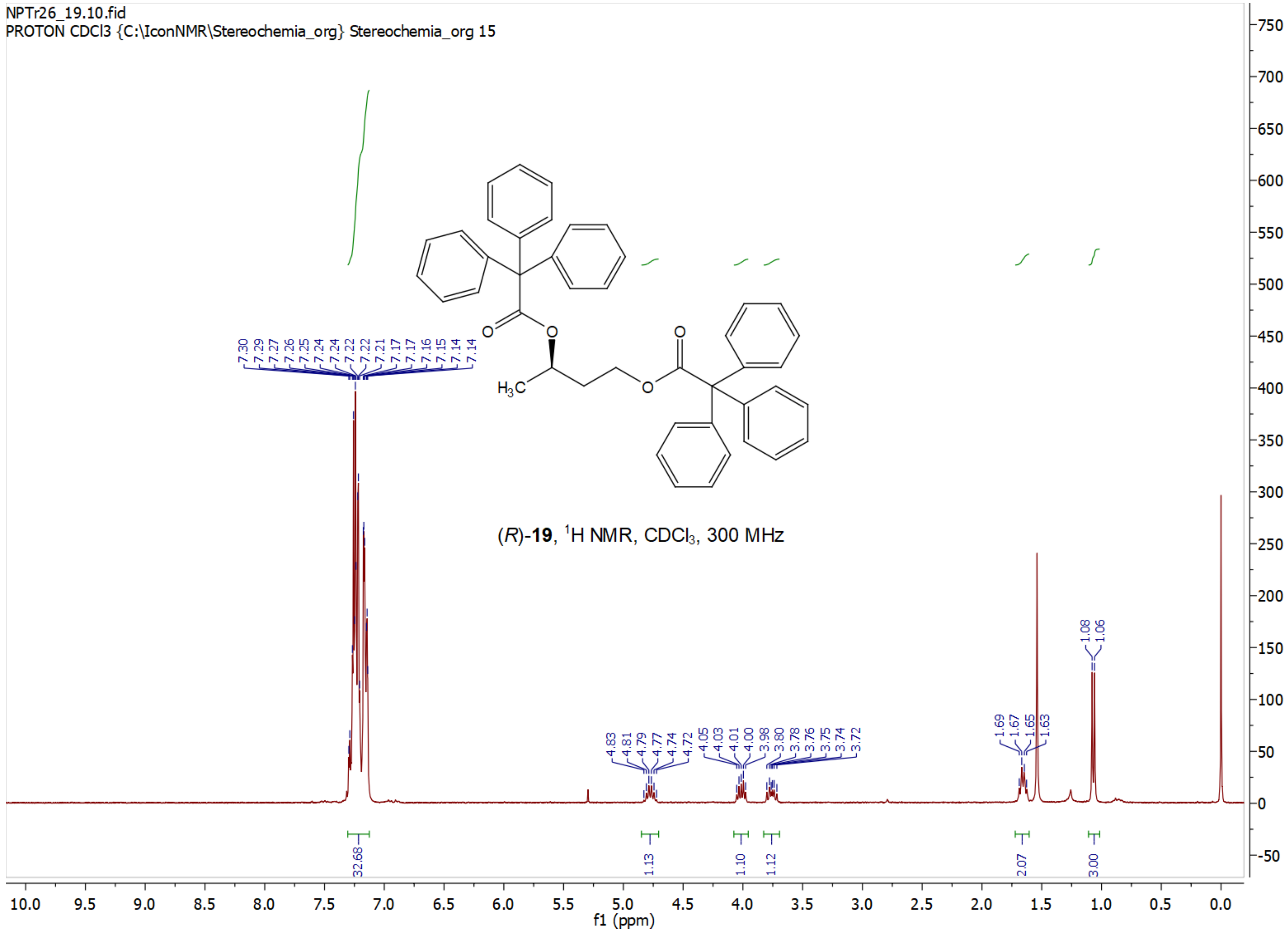

NPTr26_19.10.fid
PROTON CDCl3 {C:\IconNMR\Stereochemia_org} Stereochemia_org 15
(R)-19, 1H NMR, CDCl3, 300 MHz
7.30 7.29 7.27 7.26 7.25 7.24 7.24 7.22 7.22 7.21 7.17 7.17 7.16 7.15 7.14 7.14
4.83 4.81 4.79 4.77 4.74 4.72
4.05 4.03 4.01 4.00 3.98
3.80 3.78 3.76 3.75 3.74 3.72
1.69 1.67 1.65 1.63
1.08 1.06
32.68
1.13
1.10
1.12
2.07
3.00
f1 (ppm)

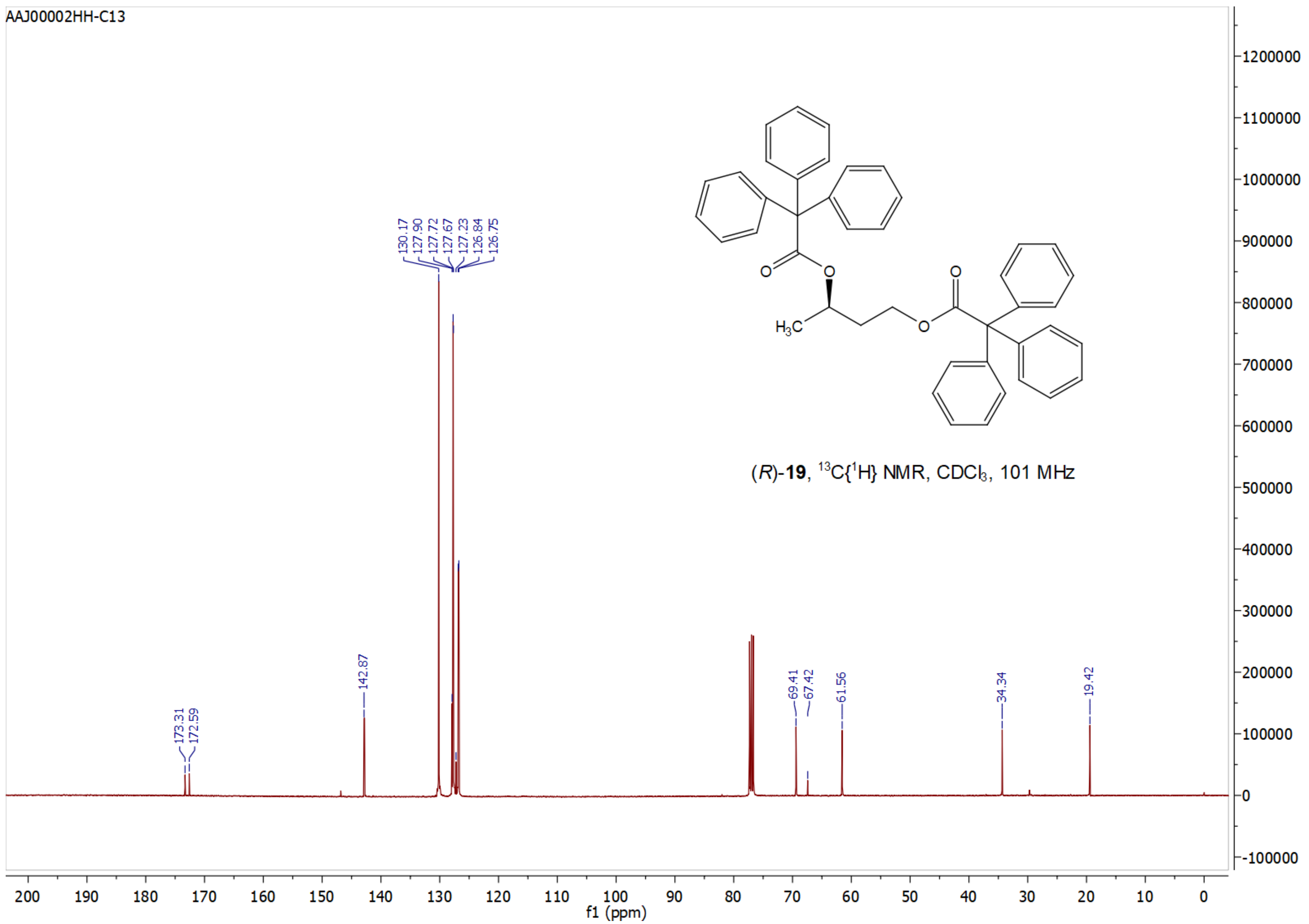

AAJ00002HH-C13
130.17
127.90
127.72
127.67
127.23
126.84
126.75
142.87
173.31
172.59
69.41
67.42
61.56
34.34
19.42
O
O
O
O
H3C
(R)-19, 13C{1H} NMR, CDCl3, 101 MHz
f1 (ppm)

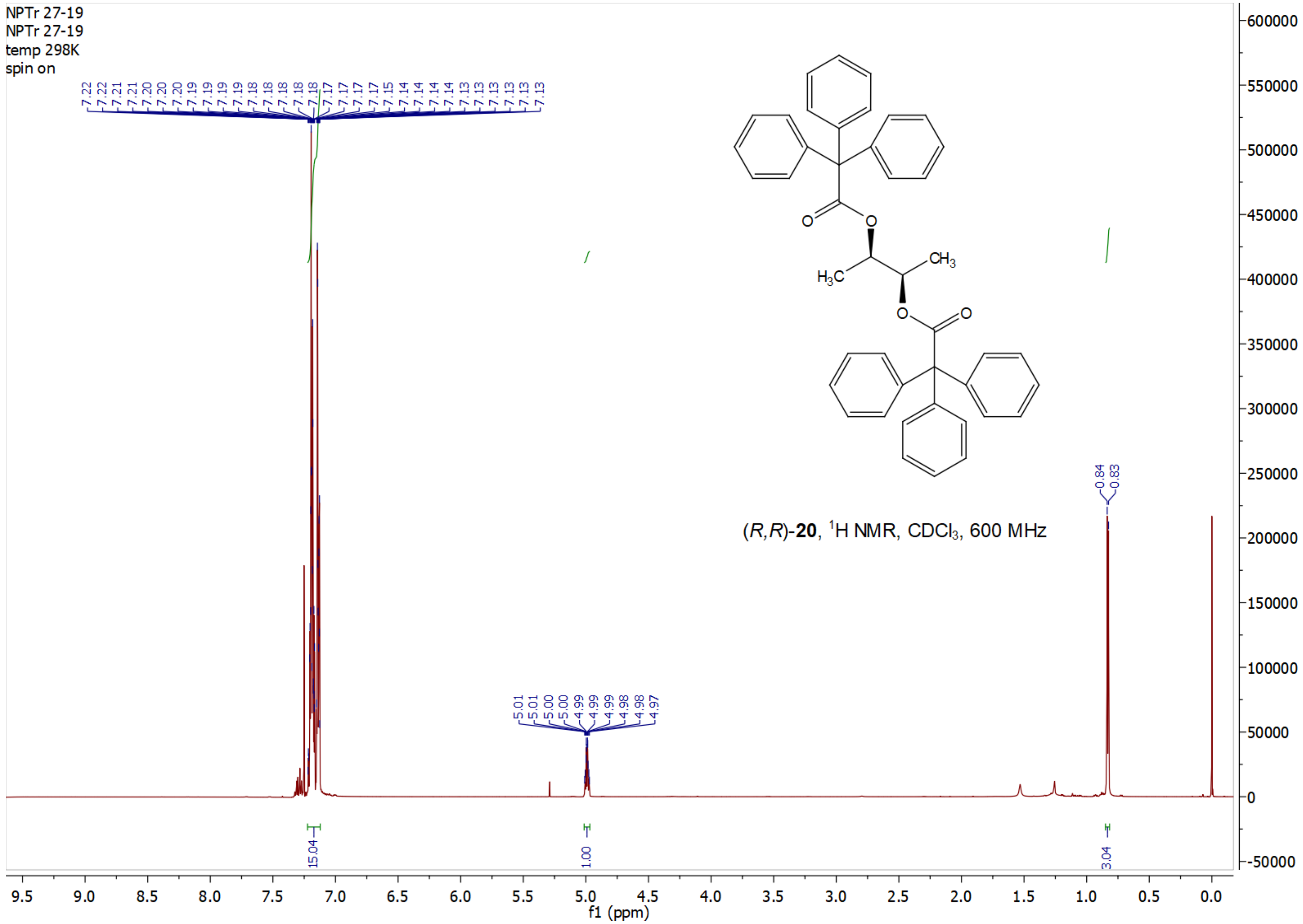
NPTr 27-19
NPTr 27-19
temp 298K
spin on
(R,R)-20, ¹H NMR, CDCl3, 600 MHz
f1 (ppm)
15.04
1.00
3.04

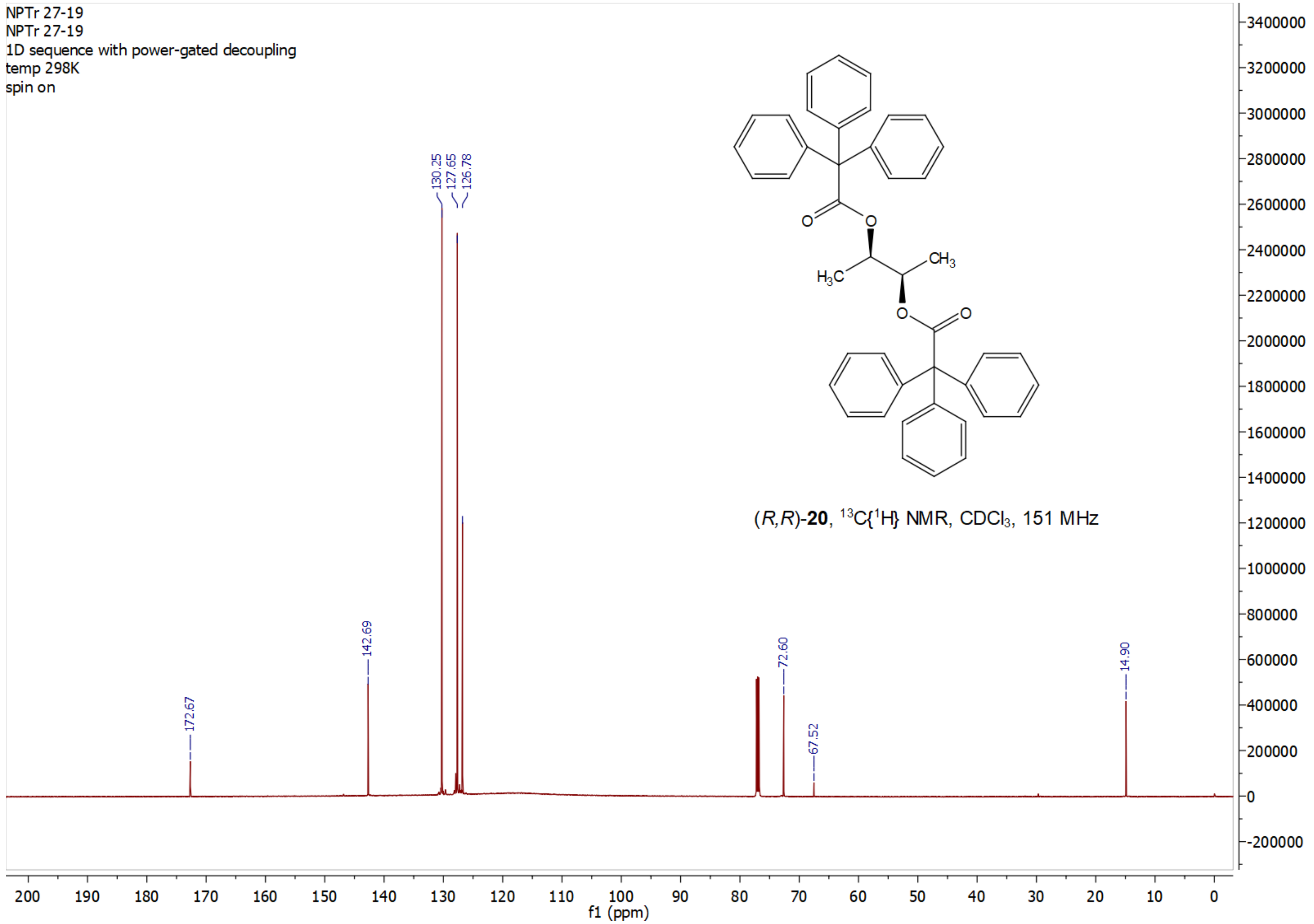

(*R*,*R*)-**20**, $^{13}C\{^1H\}$ NMR, $CDCl_3$, 151 MHz

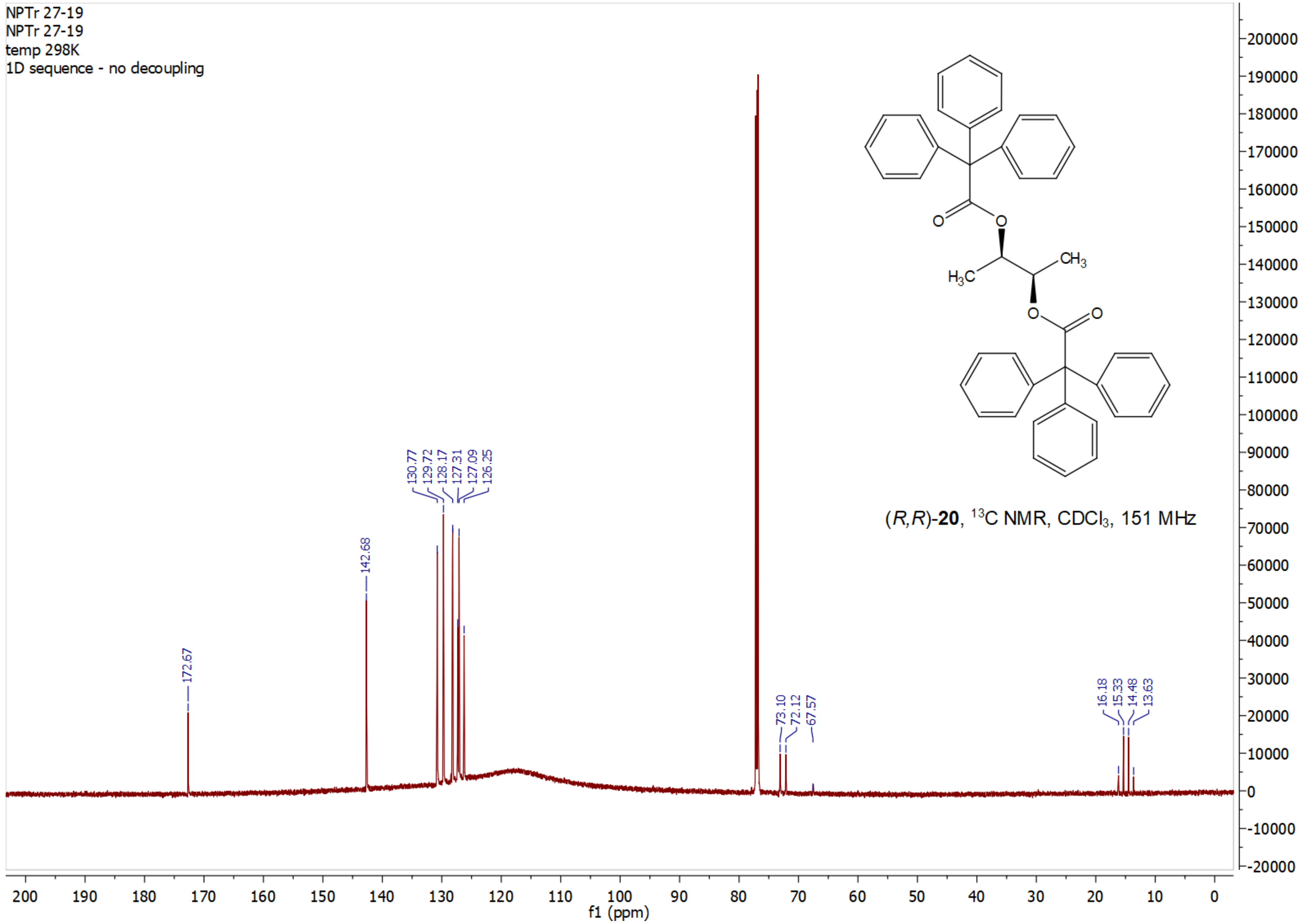
NPTr 27-19
NPTr 27-19
temp 298K
1D sequence - no decoupling
172.67
142.68
130.77
129.72
128.17
127.31
127.09
126.25
73.10
72.12
67.57
16.18
15.33
14.48
13.63
f1 (ppm)
O
O
H3C
CH3
O
O
(R,R)-20, 13C NMR, CDCl3, 151 MHz

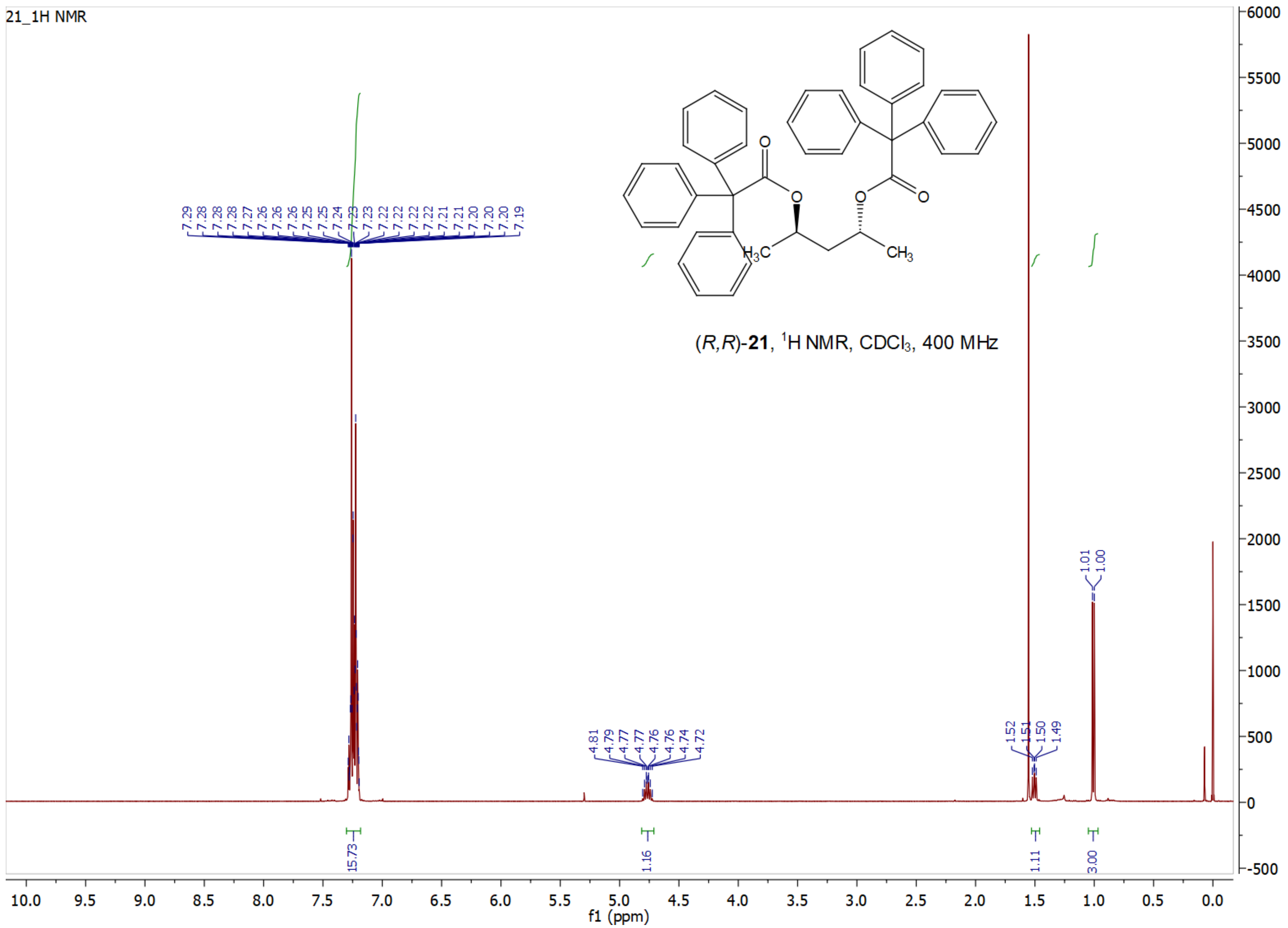
21_1H NMR
(R,R)-21, 1H NMR, CDCl3, 400 MHz
H3C
CH3
O
f1 (ppm)
15.73
1.16
1.11
3.00

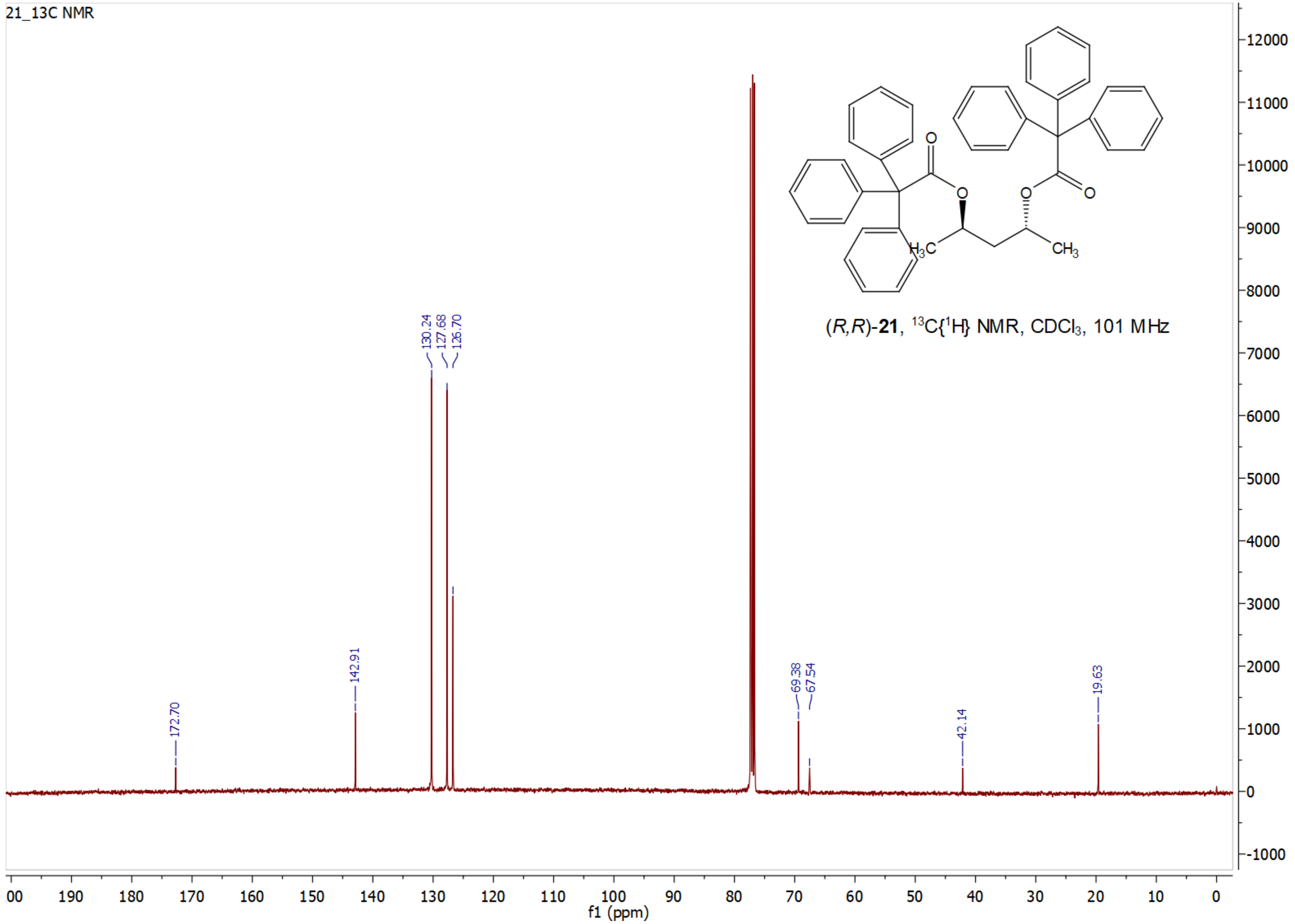
21_13C NMR
(R,R)-21, 13C{1H} NMR, CDCl3, 101 MHz
O
O
O
O
H3C
CH3
130.24
127.68
126.70
142.91
172.70
69.38
67.54
42.14
19.63
f1 (ppm)

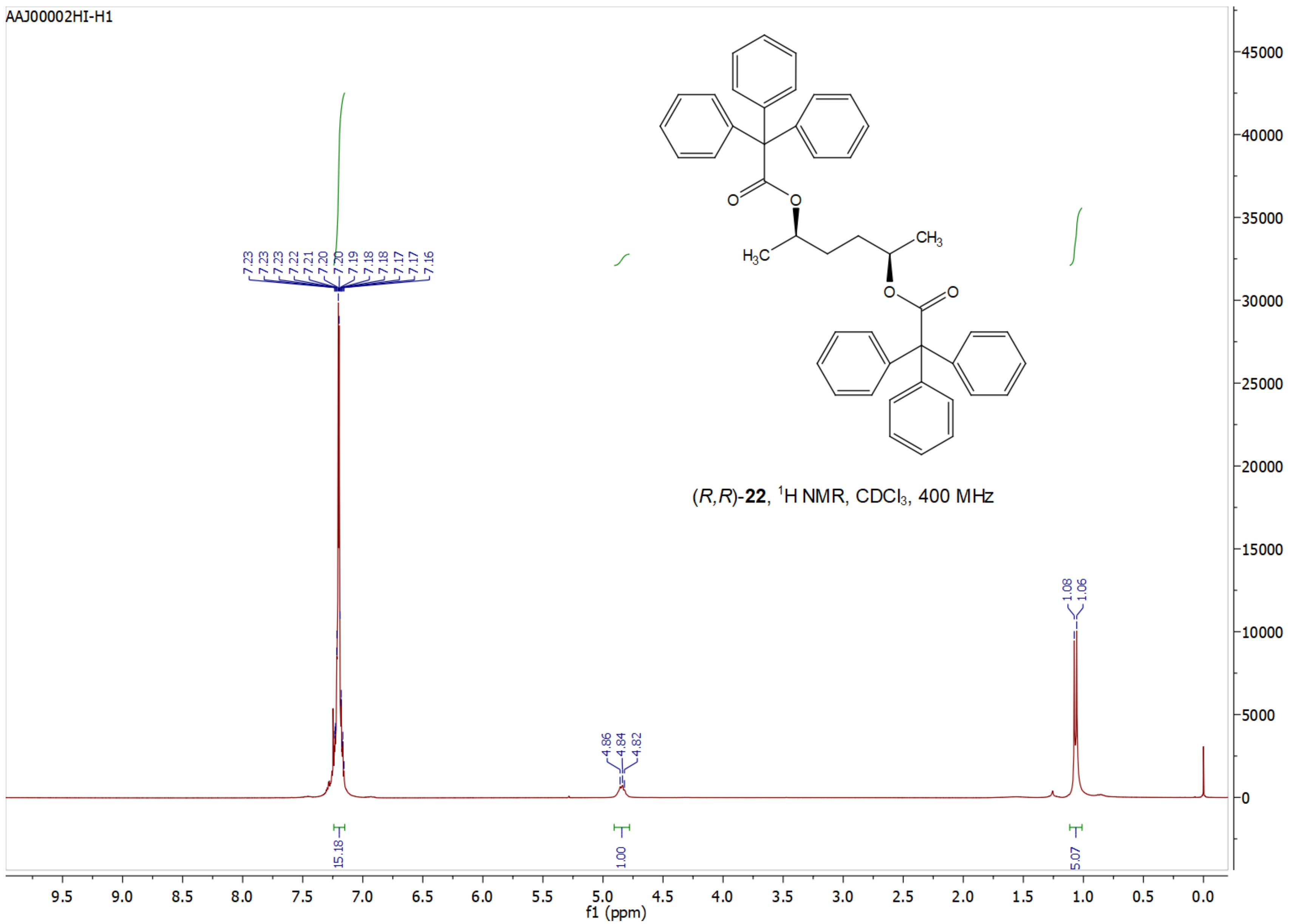

AAJ00002HI-H1
(R,R)-22, ¹H NMR, CDCl3, 400 MHz
7.23 7.23 7.23 7.22 7.21 7.20 7.20 7.19 7.18 7.18 7.17 7.17 7.16
4.86 4.84 4.82
1.08 1.06
15.18
1.00
5.07
f1 (ppm)

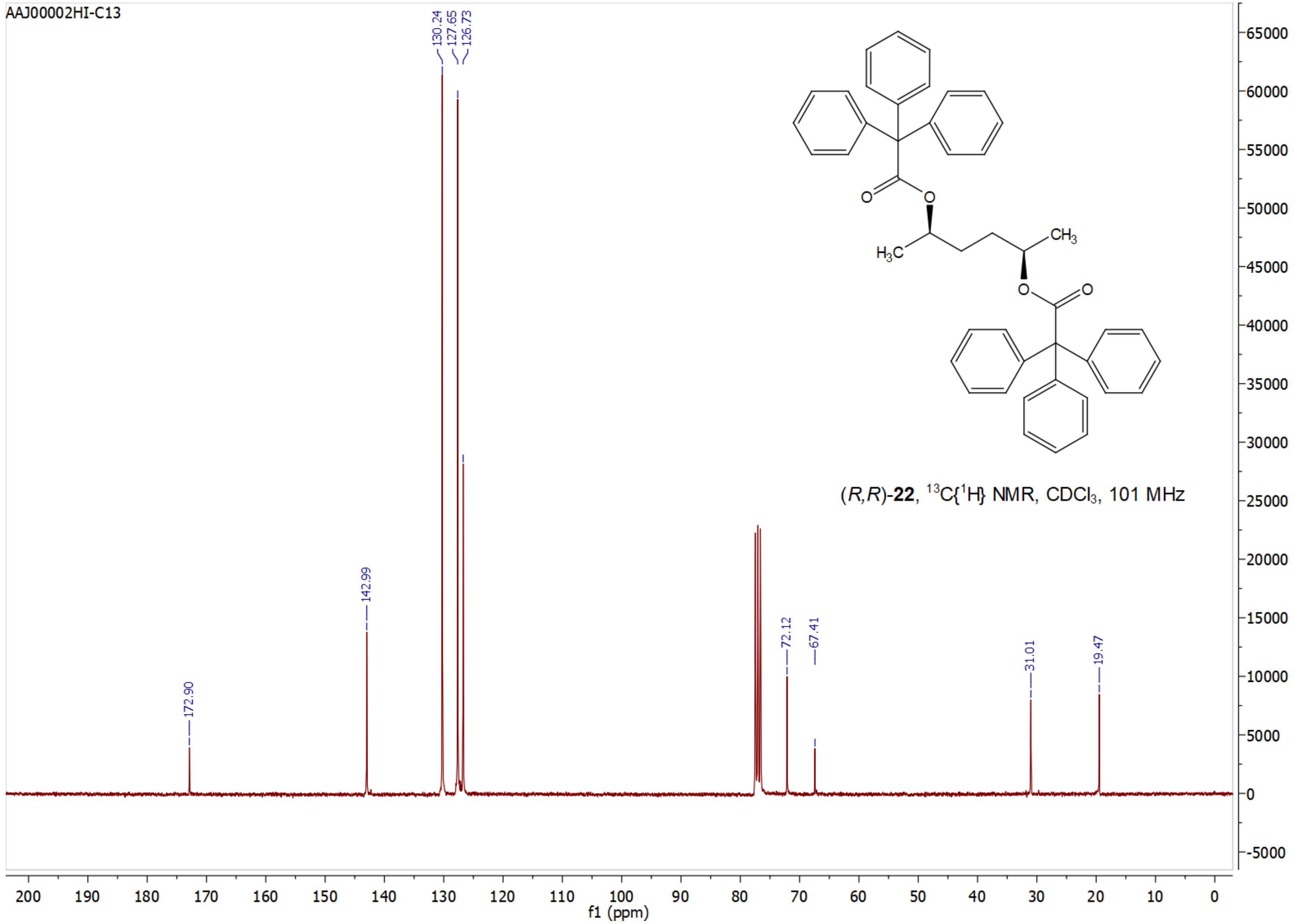
AAJ00002HI-C13
130.24
127.65
126.73
142.99
172.90
72.12
67.41
31.01
19.47
CH3
H3C
O
(R,R)-22, 13C{1H} NMR, CDCl3, 101 MHz
f1 (ppm)

Copies of ECD spectra

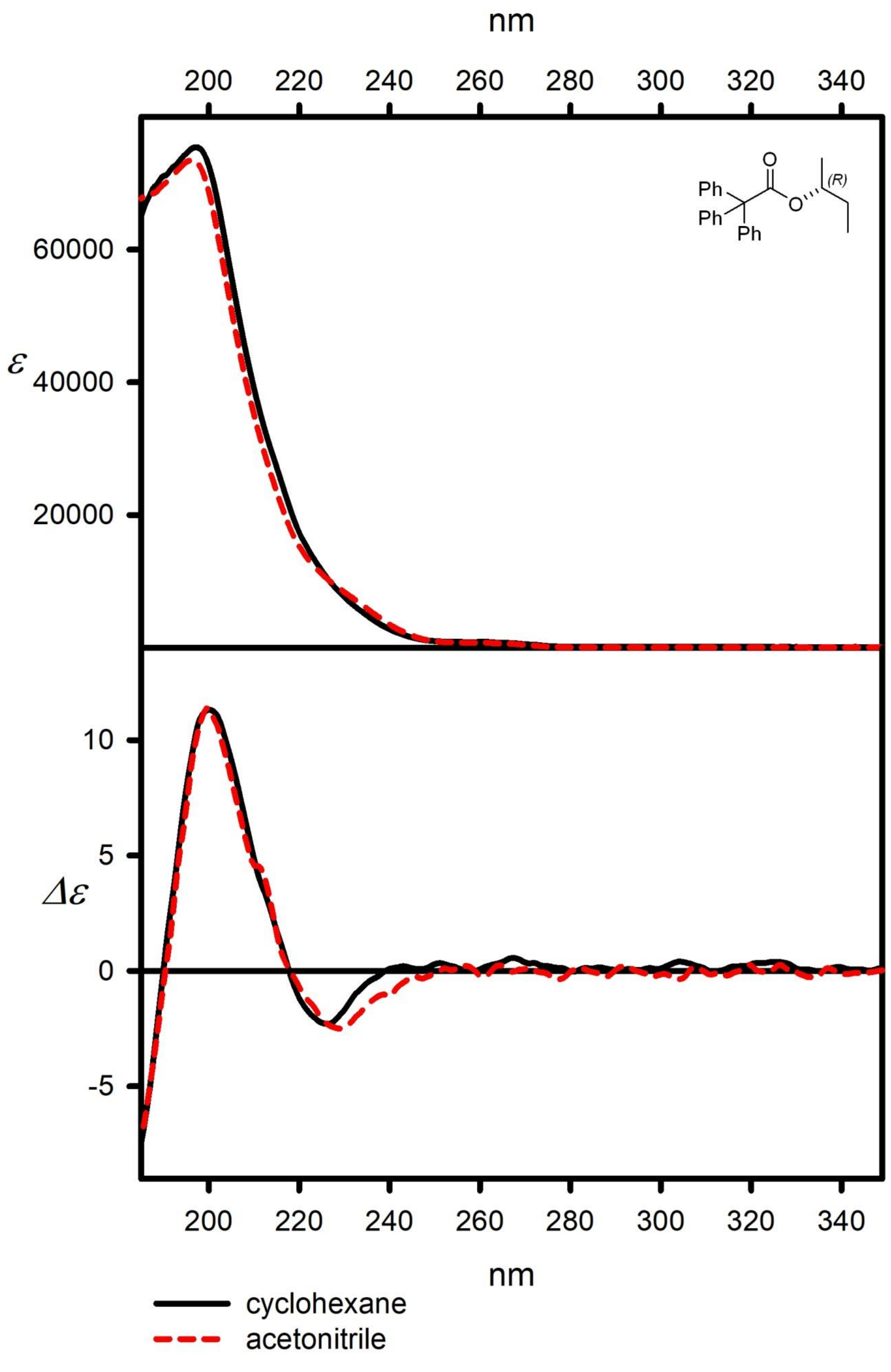

nm
200 220 240 260 280 300 320 340
Ph
Ph
Ph
O
O
(R)
ε
60000
40000
20000
10
5
Δε
0
-5
nm
cyclohexane
acetonitrile

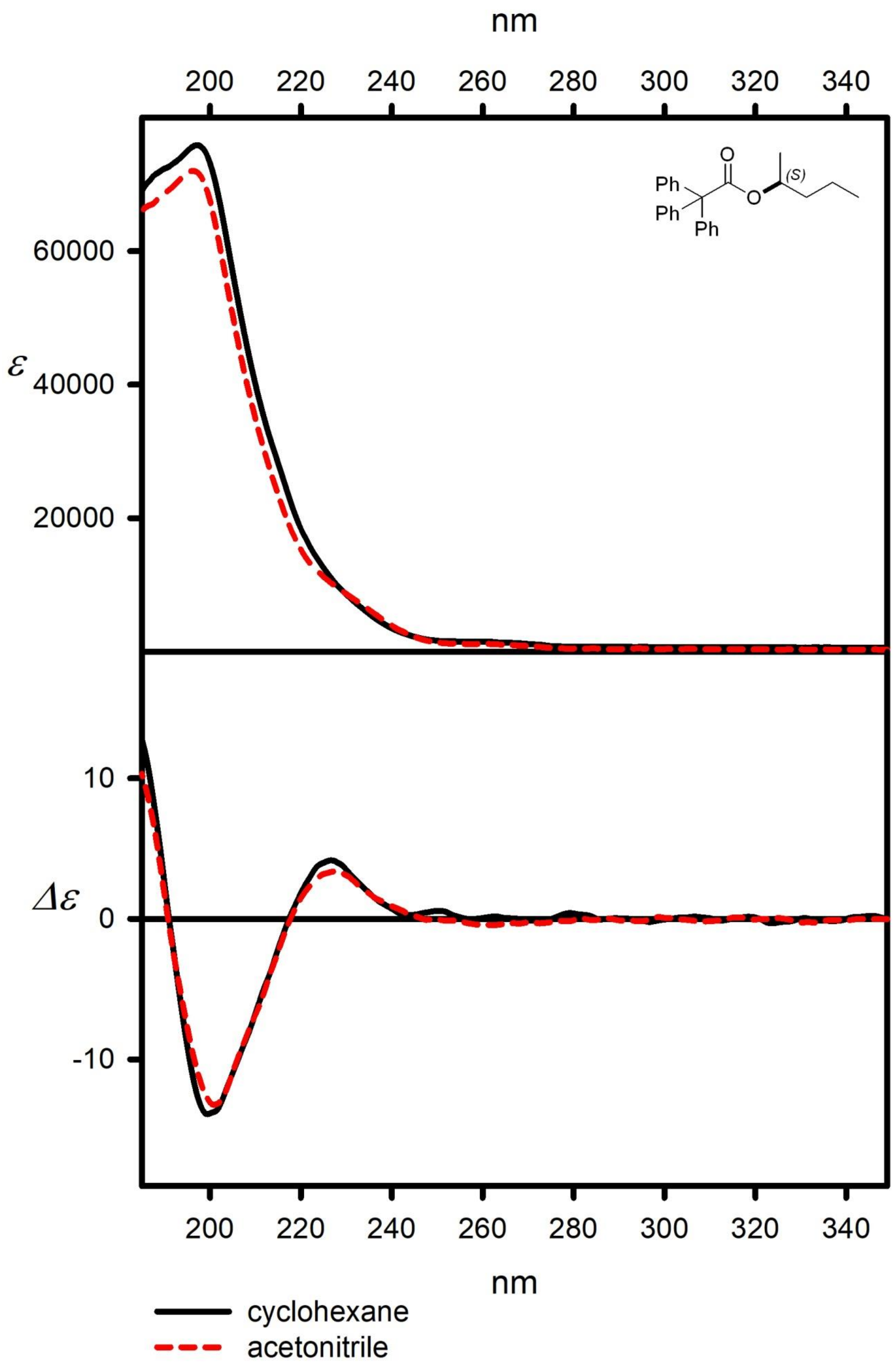

nm
200
220
240
260
280
300
320
340
60000
40000
20000
ε
Ph
Ph
Ph
O
O
(S)
10
0
-10
Δε
nm
cyclohexane
acetonitrile

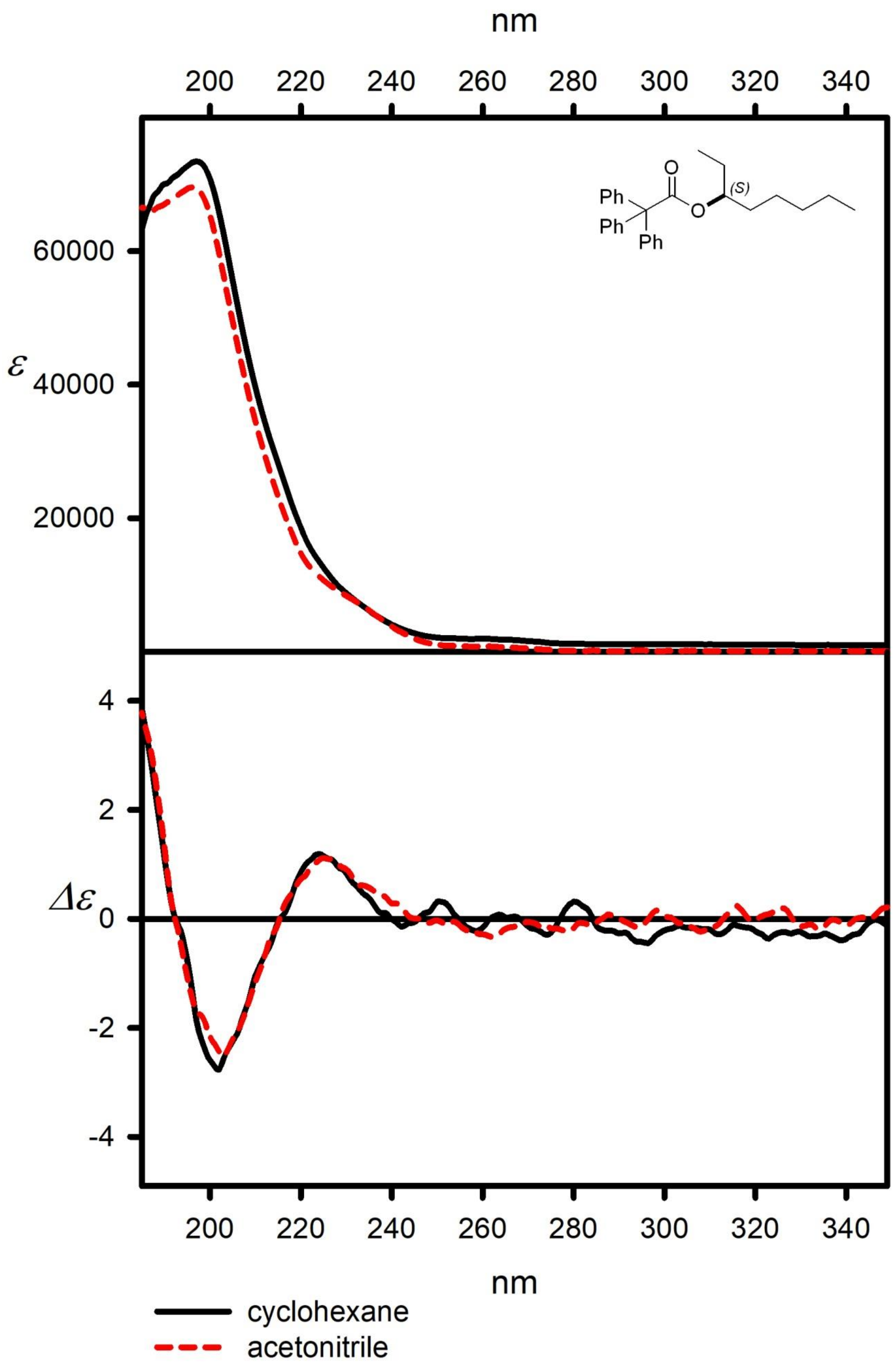

nm
200
220
240
260
280
300
320
340
ε
60000
40000
20000
Ph
Ph
Ph
O
O
(S)
Δε
4
2
0
-2
-4
nm
cyclohexane
acetonitrile

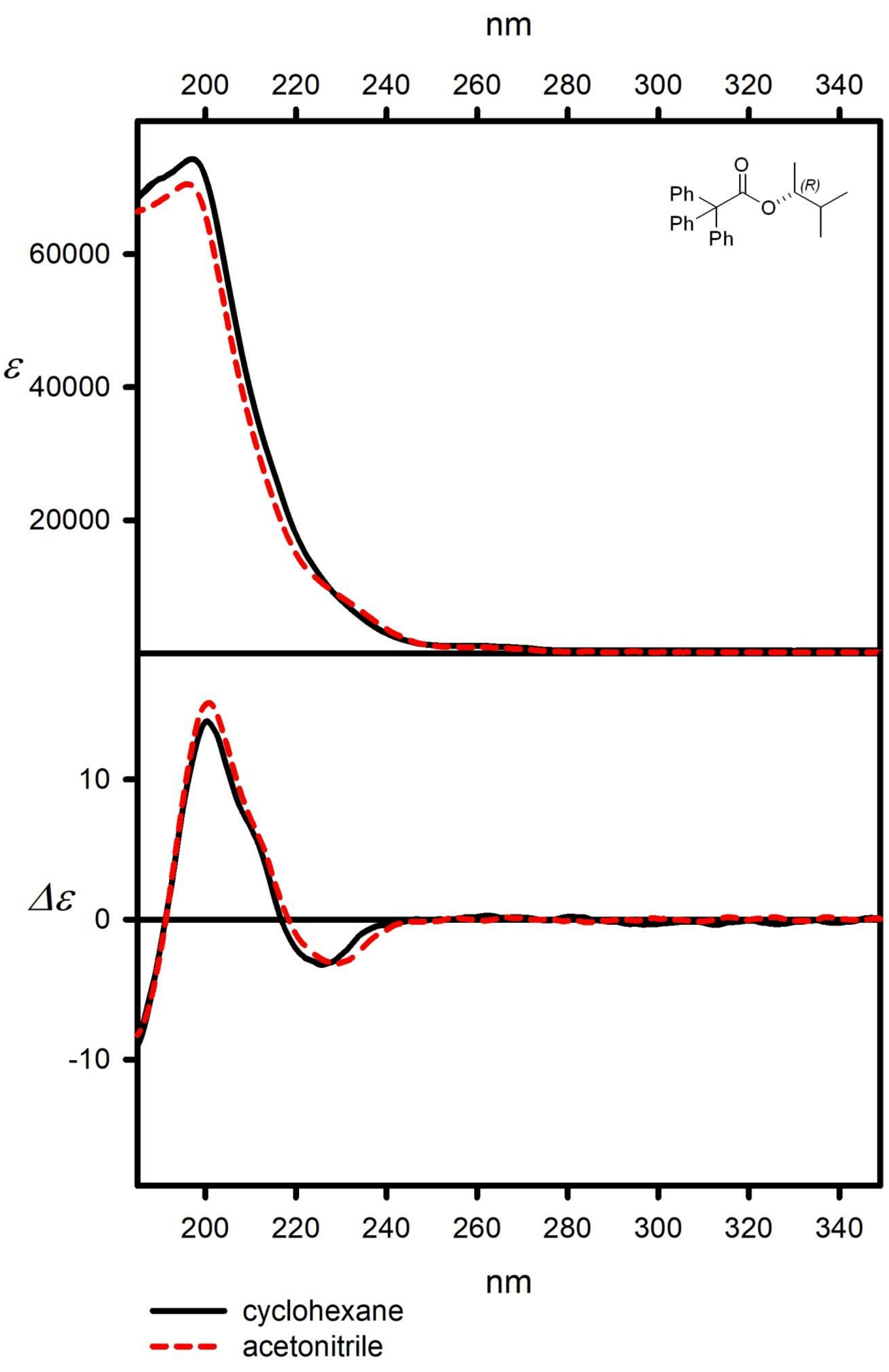

nm
200
220
240
260
280
300
320
340
ε
60000
40000
20000
Δε
10
0
-10
Ph
Ph
Ph
O
O
(R)
cyclohexane
acetonitrile

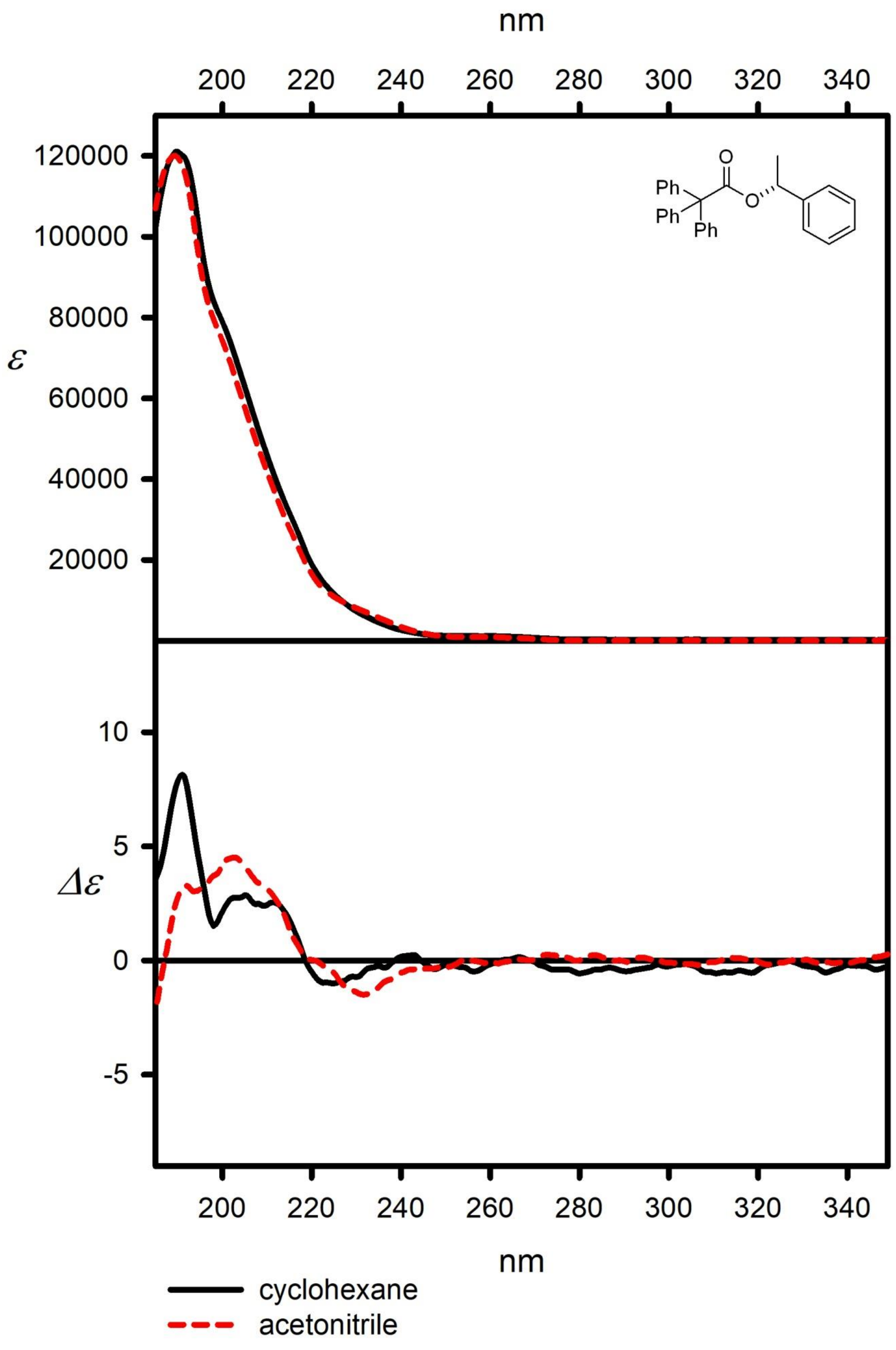
nm
200
220
240
260
280
300
320
340
120000
100000
80000
60000
40000
20000
ε
O
Ph
Ph
Ph
O
10
5
Δε
0
-5
cyclohexane
acetonitrile

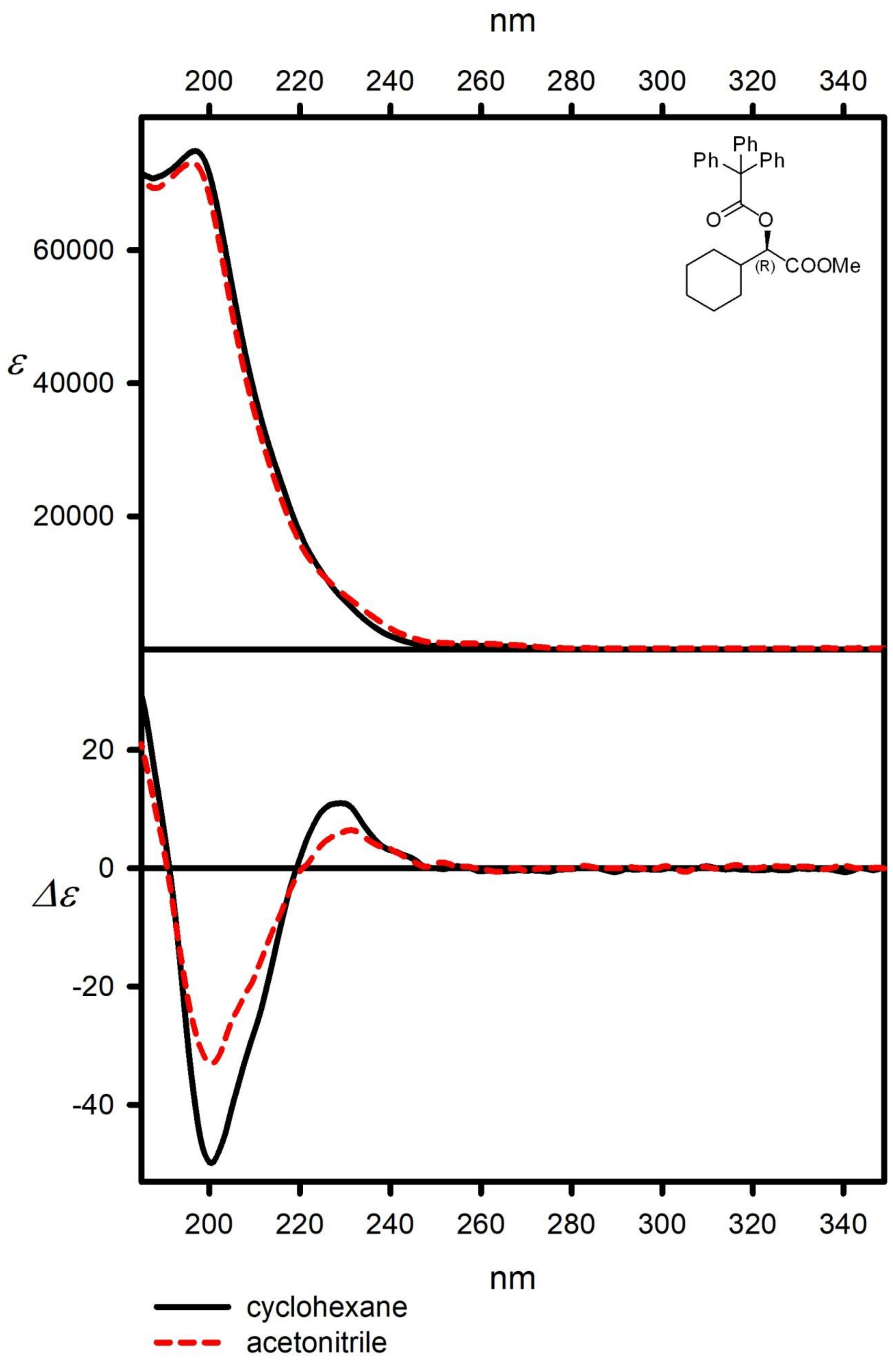

nm
200
220
240
260
280
300
320
340
60000
ε
40000
20000
Ph
Ph
Ph
O
O
(R)
COOMe
20
0
Δε
-20
-40
200
220
240
260
280
300
320
340
nm
cyclohexane
acetonitrile

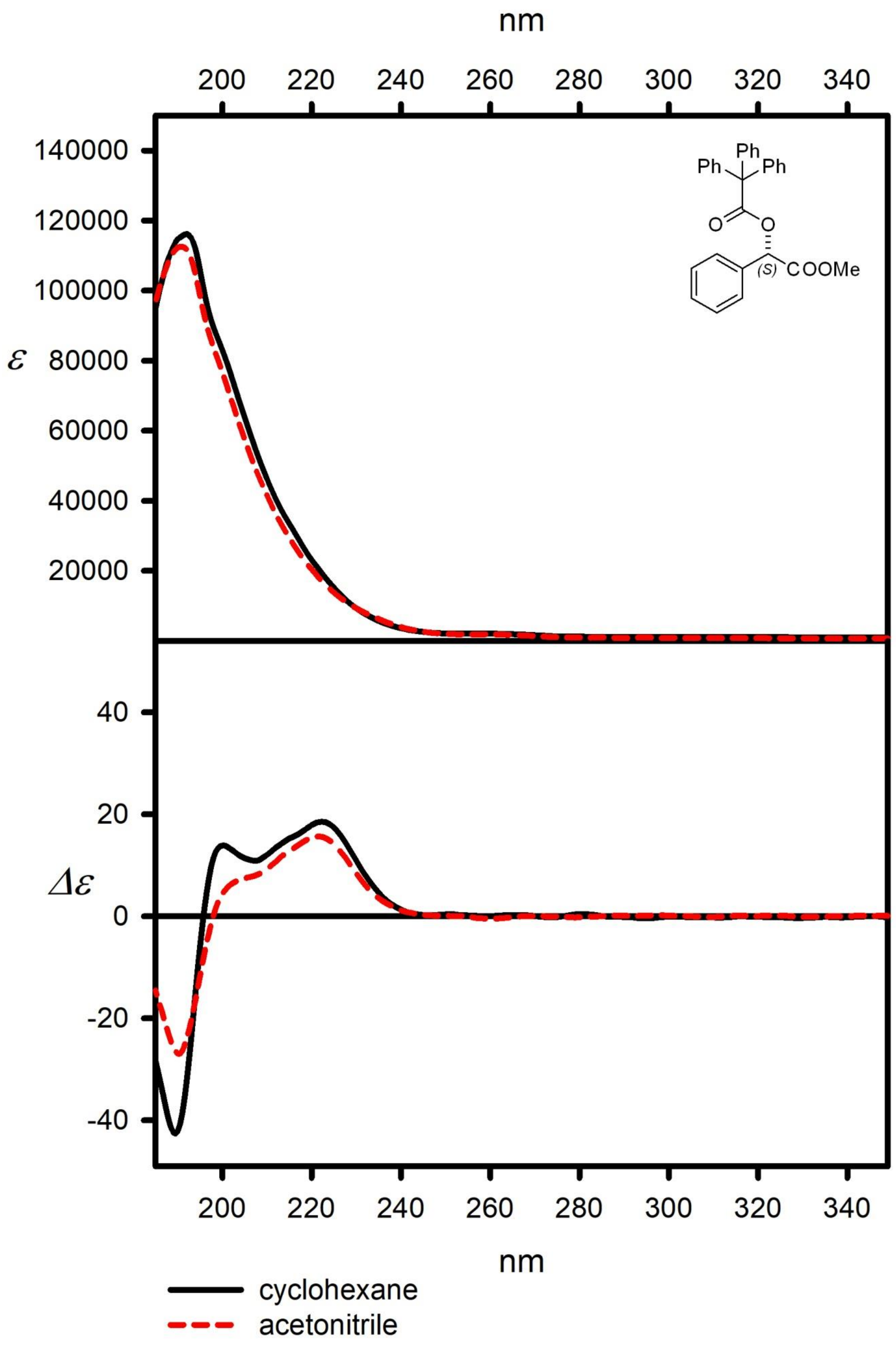

nm
200
220
240
260
280
300
320
340
140000
120000
100000
ε
80000
60000
40000
20000
Ph
Ph
Ph
O
O
(S)
COOMe
40
20
Δε
0
-20
-40
nm
cyclohexane
acetonitrile

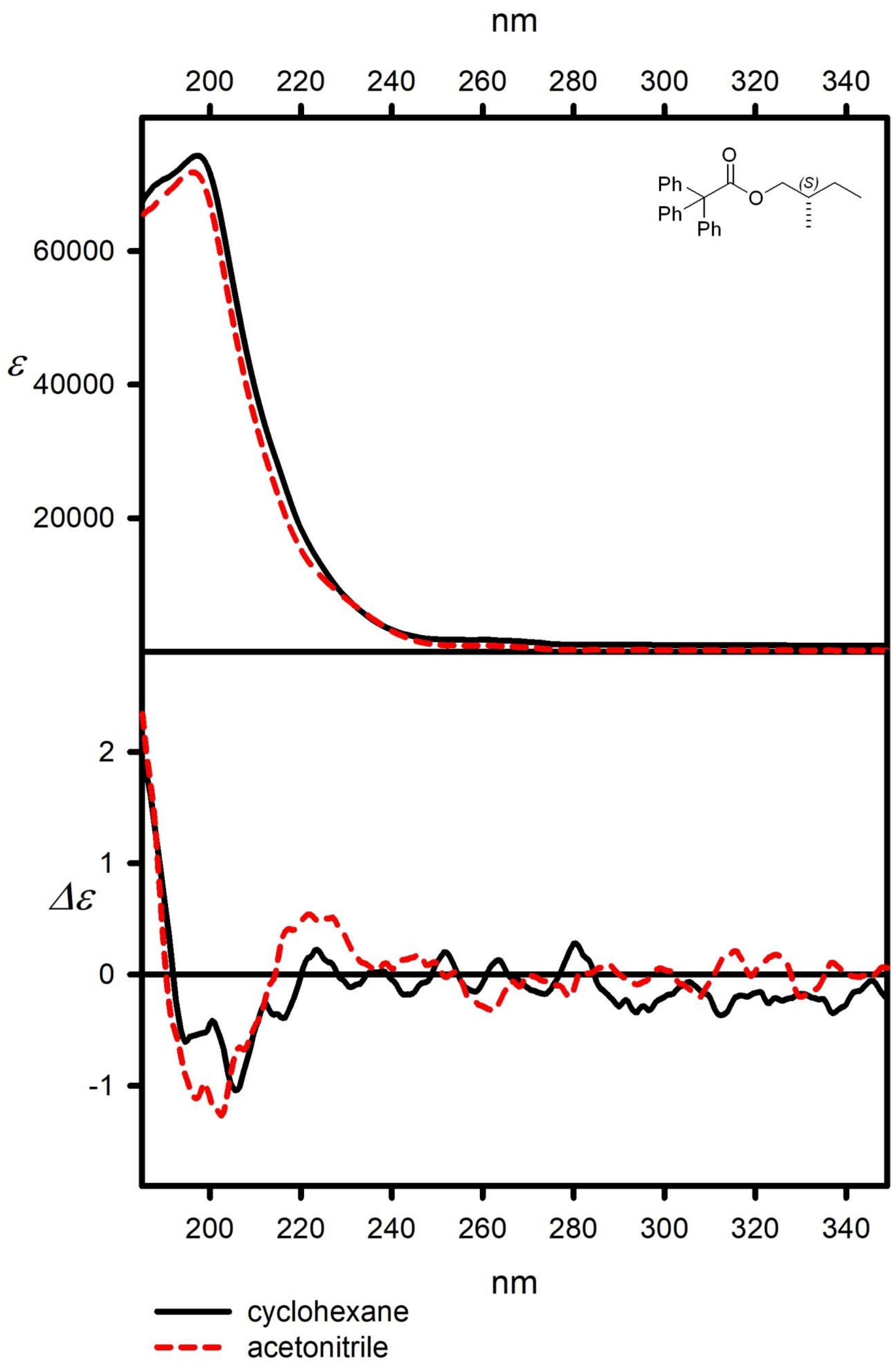

nm
200
220
240
260
280
300
320
340
60000
40000
20000
ε
Ph
Ph
Ph
O
O
(S)
2
1
0
-1
Δε
nm
cyclohexane
acetonitrile

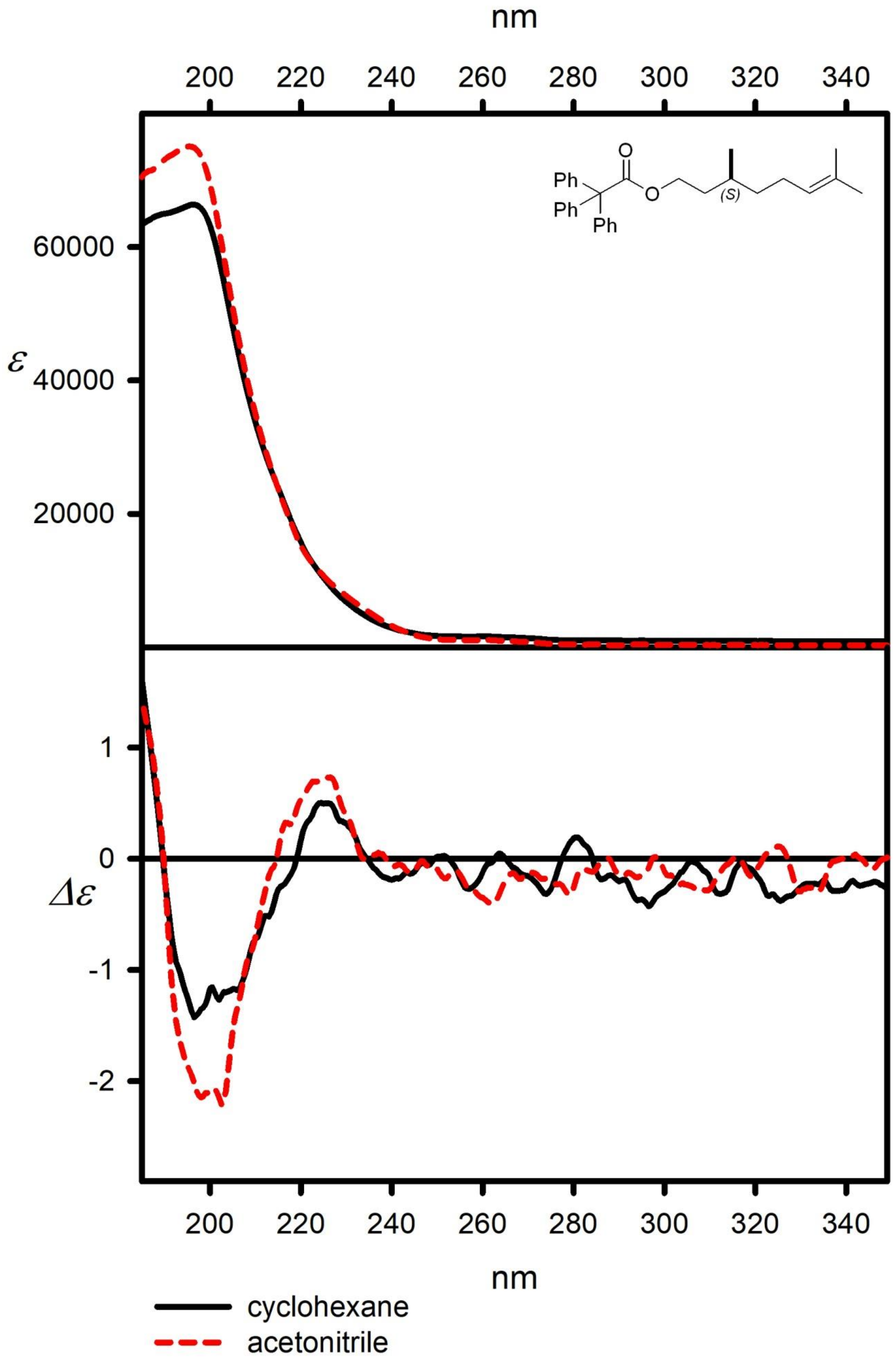
nm
200 220 240 260 280 300 320 340
Ph
Ph
Ph
O
O
(S)
ε
60000
40000
20000
1
0
-1
-2
Δε
nm
cyclohexane
acetonitrile

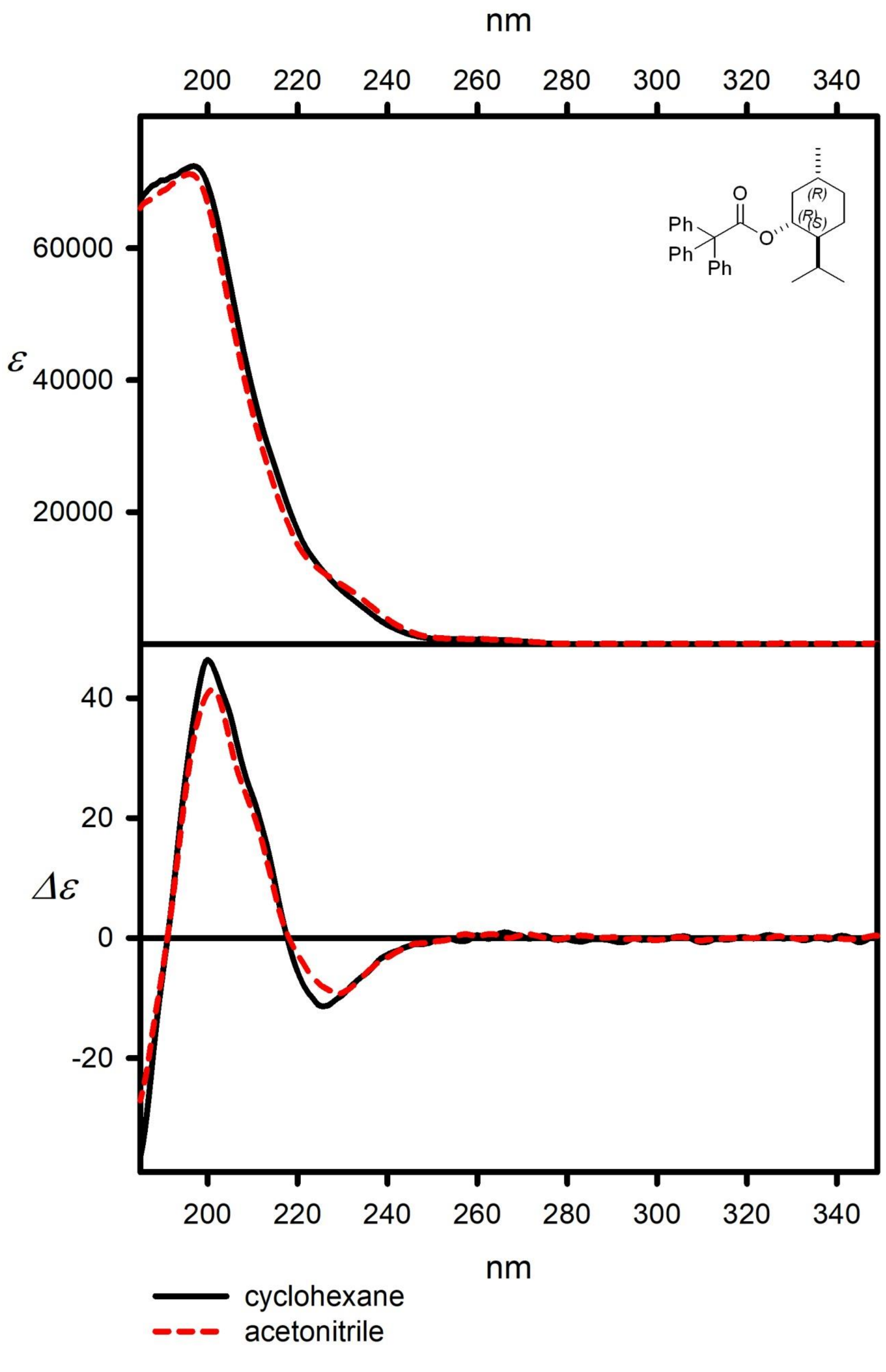
nm
200
220
240
260
280
300
320
340
60000
40000
20000
ε
Δε
40
20
0
-20
O
Ph
Ph
Ph
O
(R)
(R)
(S)
nm
cyclohexane
acetonitrile

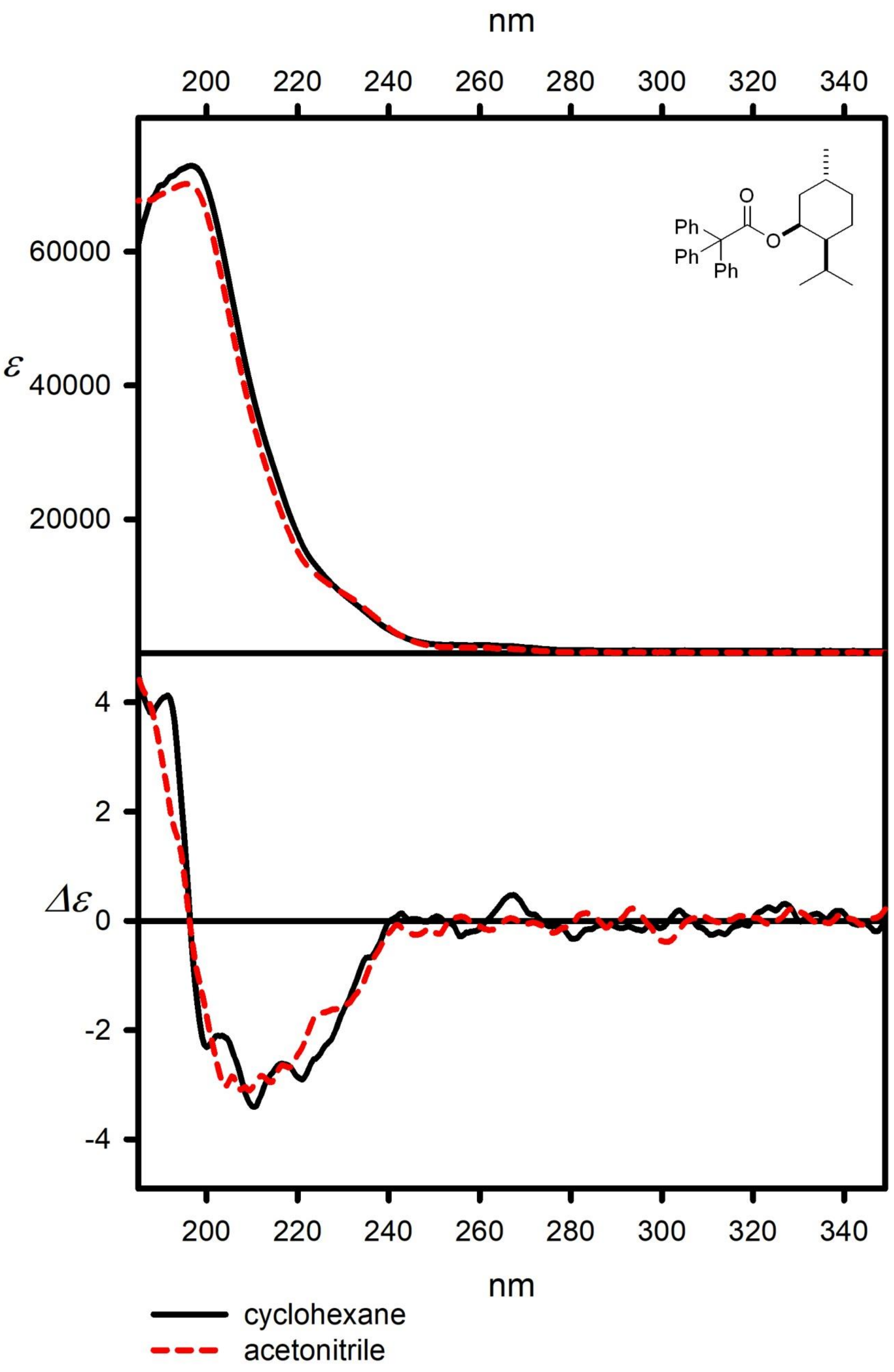
nm
200
220
240
260
280
300
320
340
ε
60000
40000
20000
Ph
Ph
Ph
O
O
4
2
Δε
0
-2
-4
cyclohexane
acetonitrile

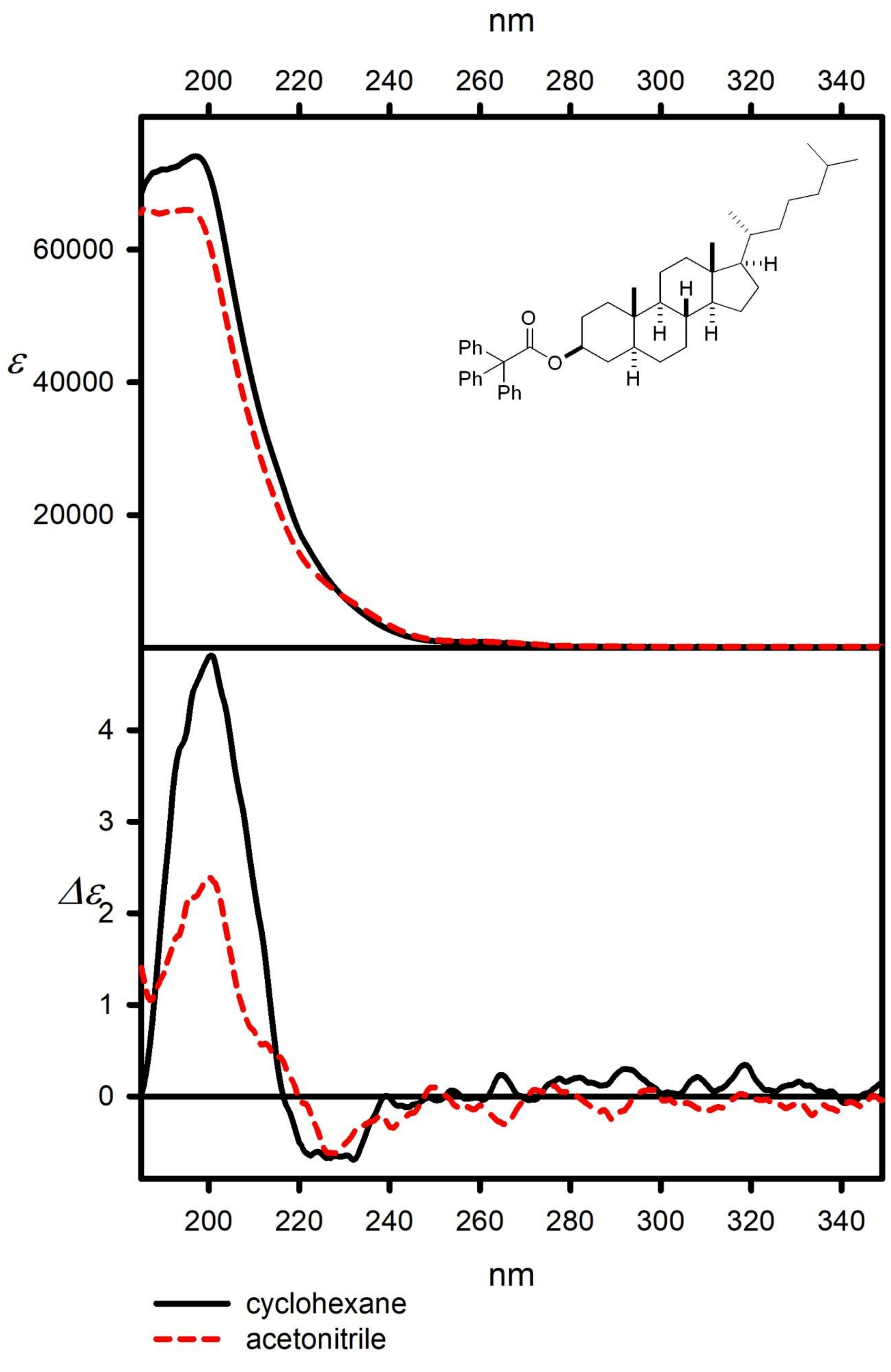
nm
200 220 240 260 280 300 320 340
ε
60000
40000
20000
Ph
Ph
Ph
O
O
H
H
H
H
H
Δε
4
3
2
1
0
200 220 240 260 280 300 320 340
nm
cyclohexane
acetonitrile

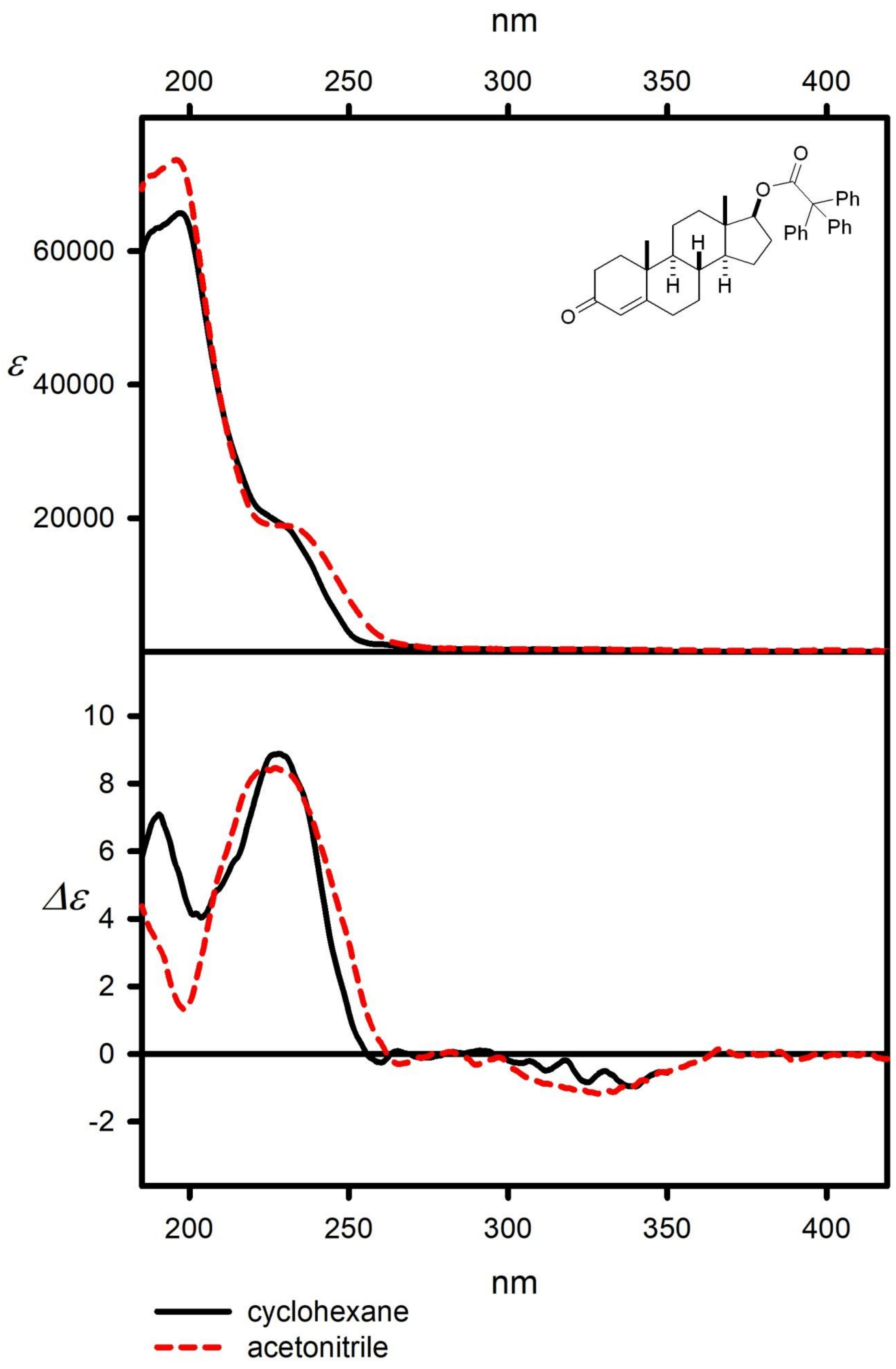
nm
200
250
300
350
400
60000
40000
20000
ε
Ph
Ph
Ph
O
O
H
H
H
O
10
8
6
4
2
0
-2
Δε
nm
cyclohexane
acetonitrile

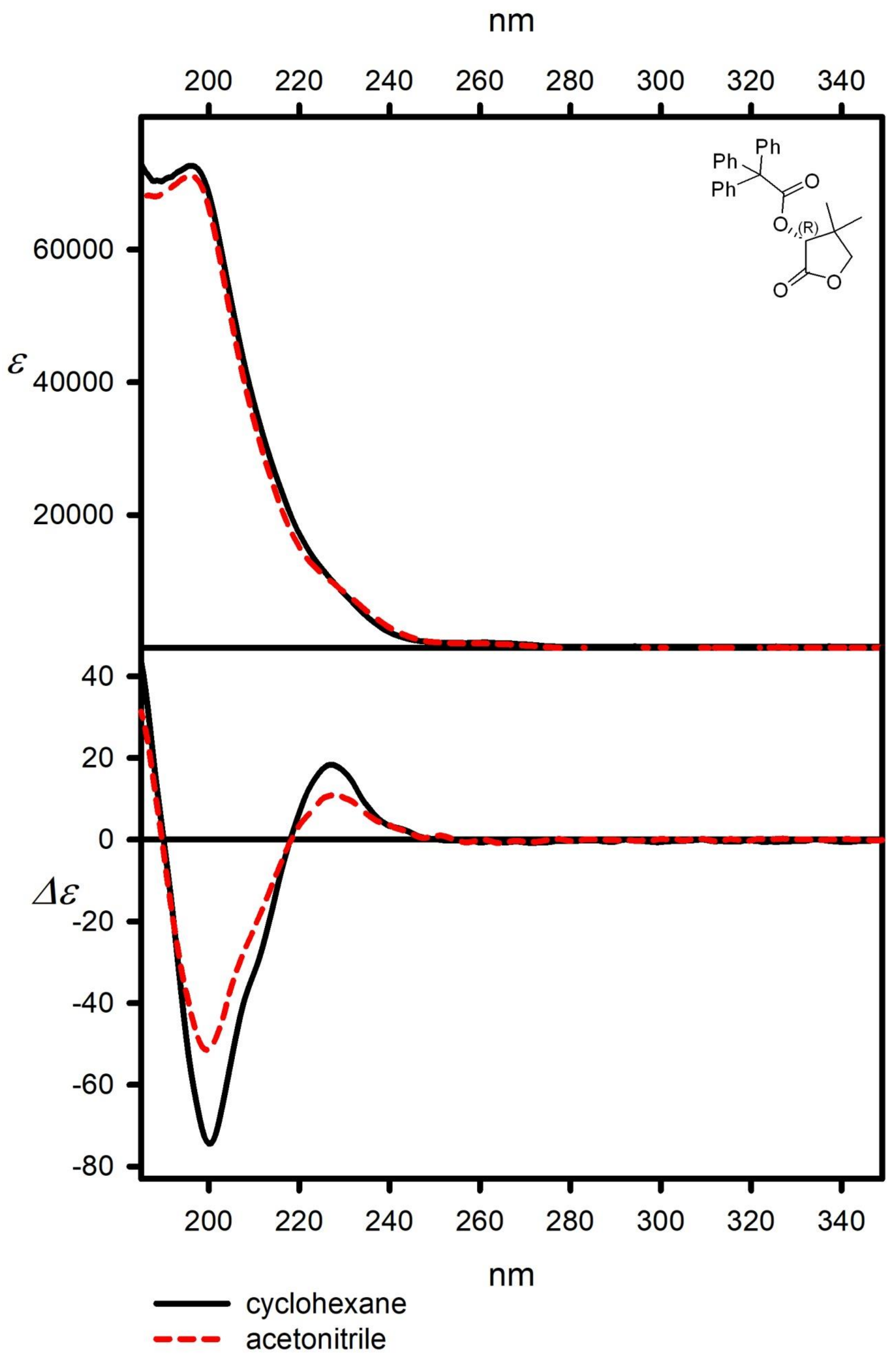
nm
200
220
240
260
280
300
320
340
Ph
Ph
Ph
O
O
(R)
O
O
60000
40000
20000
ε
40
20
0
-20
-40
-60
-80
Δε
nm
cyclohexane
acetonitrile

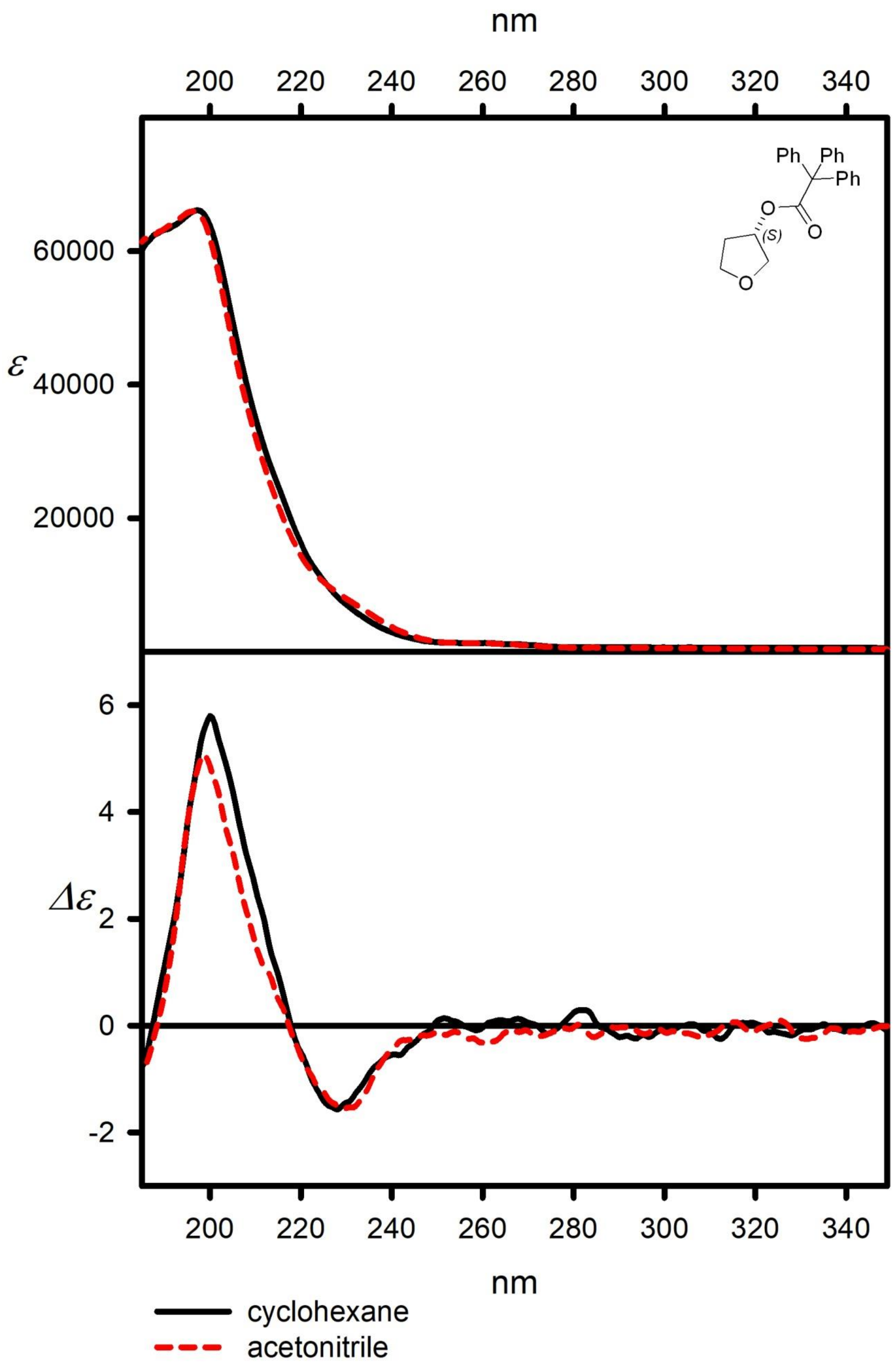
nm
200
220
240
260
280
300
320
340
Ph
Ph
Ph
O
(S)
O
O
ε
60000
40000
20000
6
4
Δε
2
0
-2
nm
cyclohexane
acetonitrile

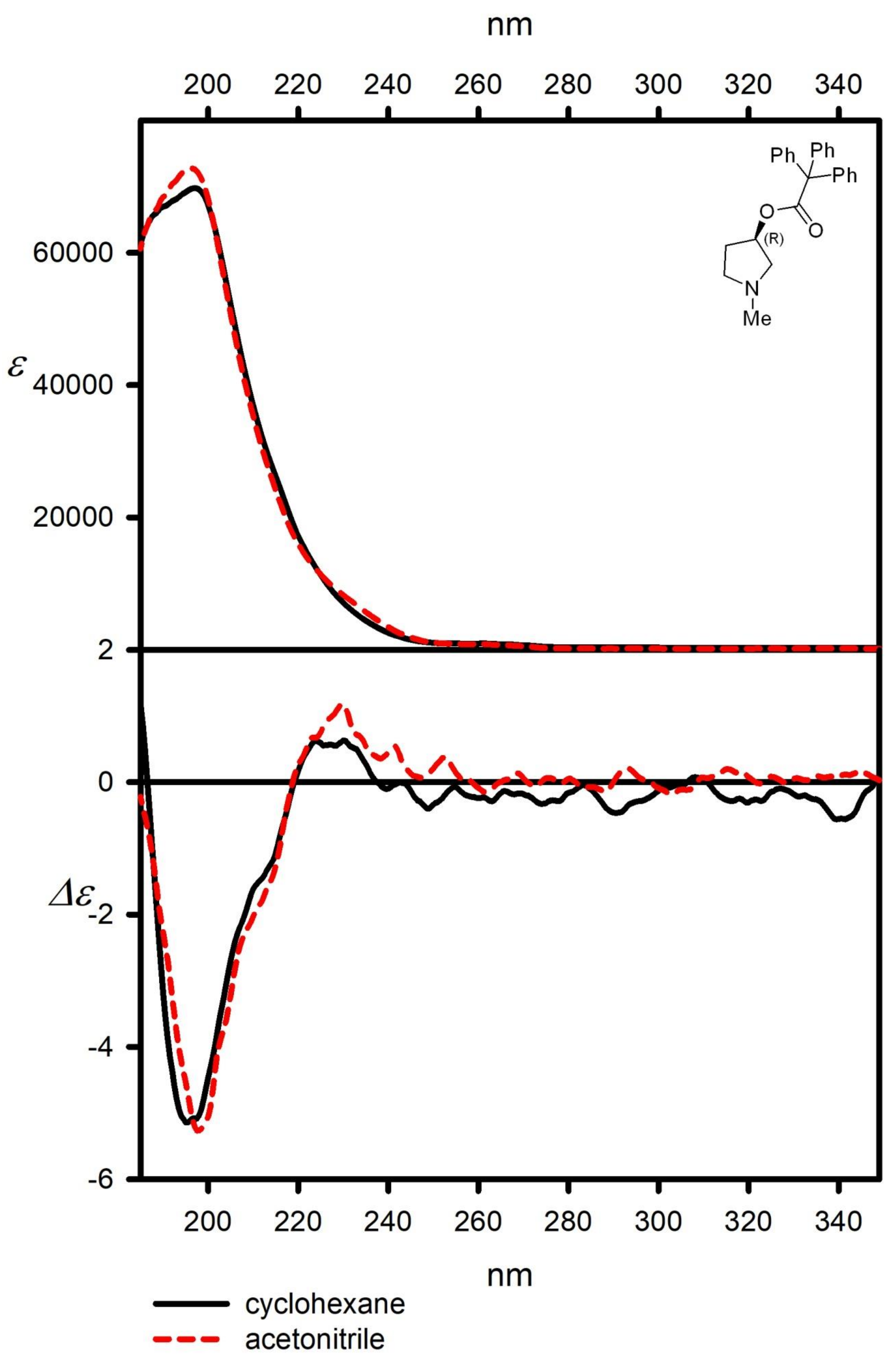
nm
200
220
240
260
280
300
320
340
ε
60000
40000
20000
2
Δε
0
-2
-4
-6
Ph
Ph
Ph
O
O
(R)
N
Me
cyclohexane
acetonitrile

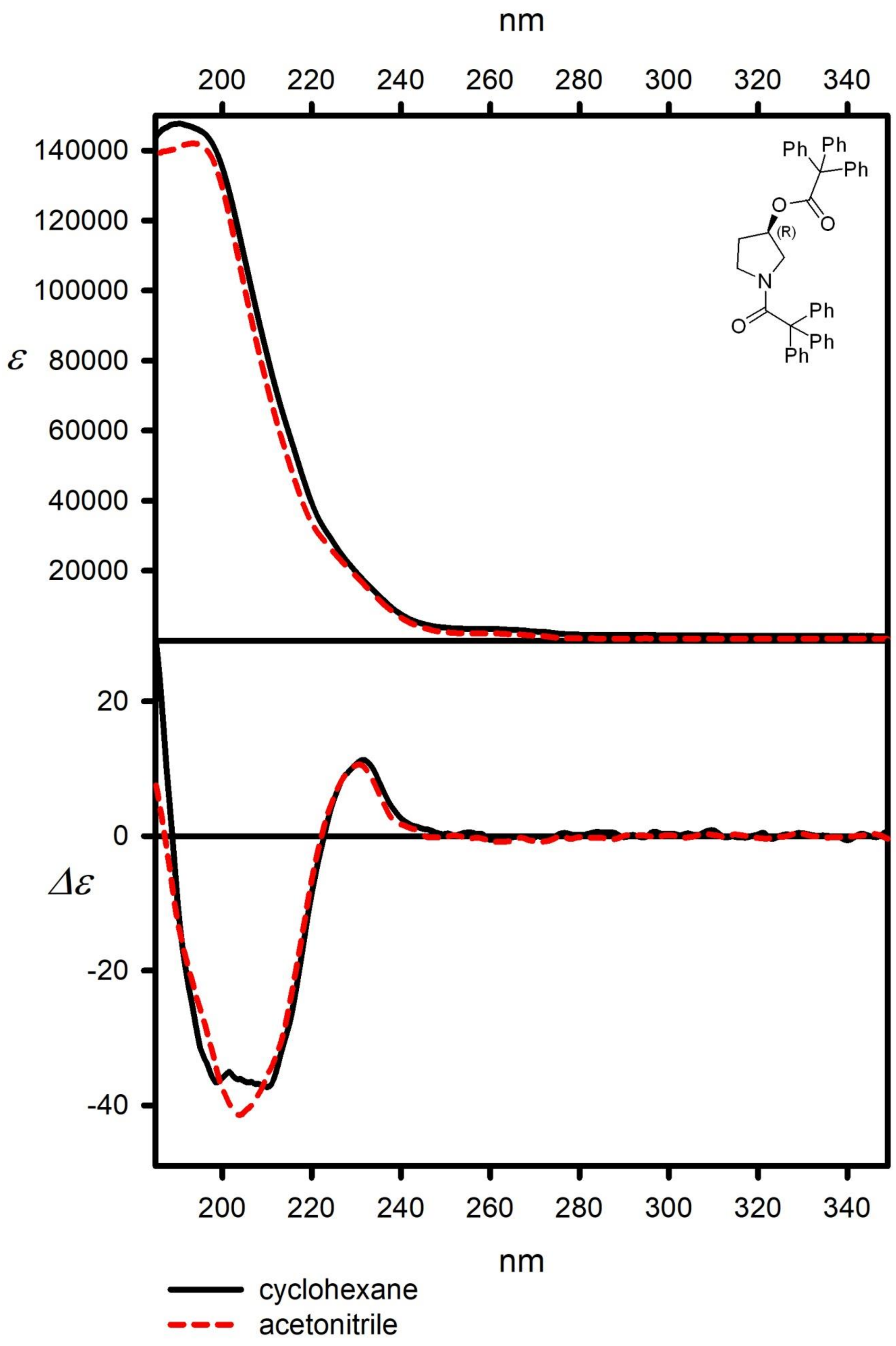

nm
200
220
240
260
280
300
320
340
140000
120000
100000
80000
60000
40000
20000
ε
20
0
-20
-40
Δε
Ph
Ph
Ph
O
O
(R)
N
O
Ph
Ph
Ph
cyclohexane
acetonitrile

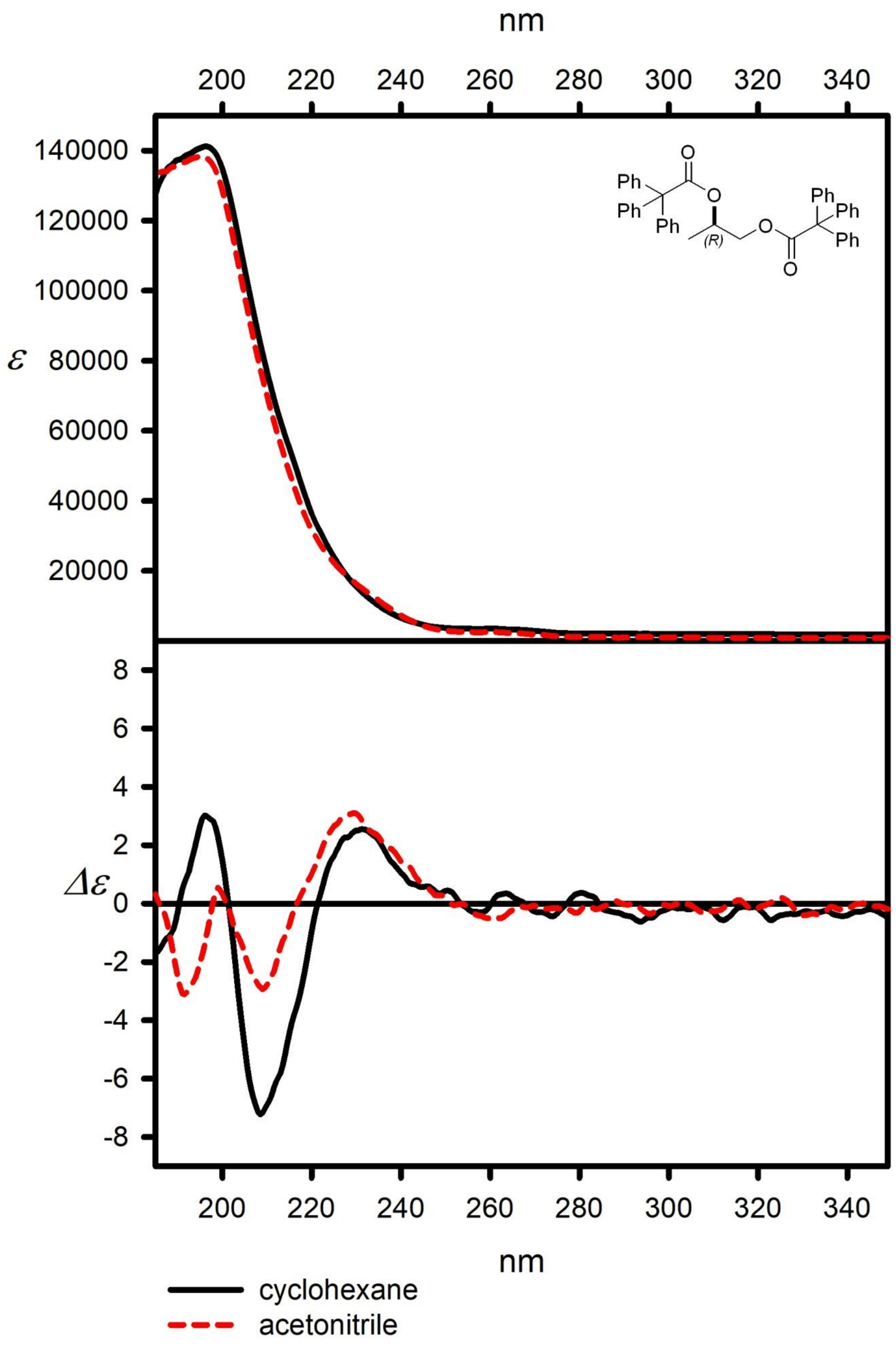
nm
200
220
240
260
280
300
320
340
140000
120000
100000
ε
80000
60000
40000
20000
Ph
Ph
Ph
O
O
(R)
O
O
Ph
Ph
Ph
8
6
4
2
Δε
0
-2
-4
-6
-8
cyclohexane
acetonitrile

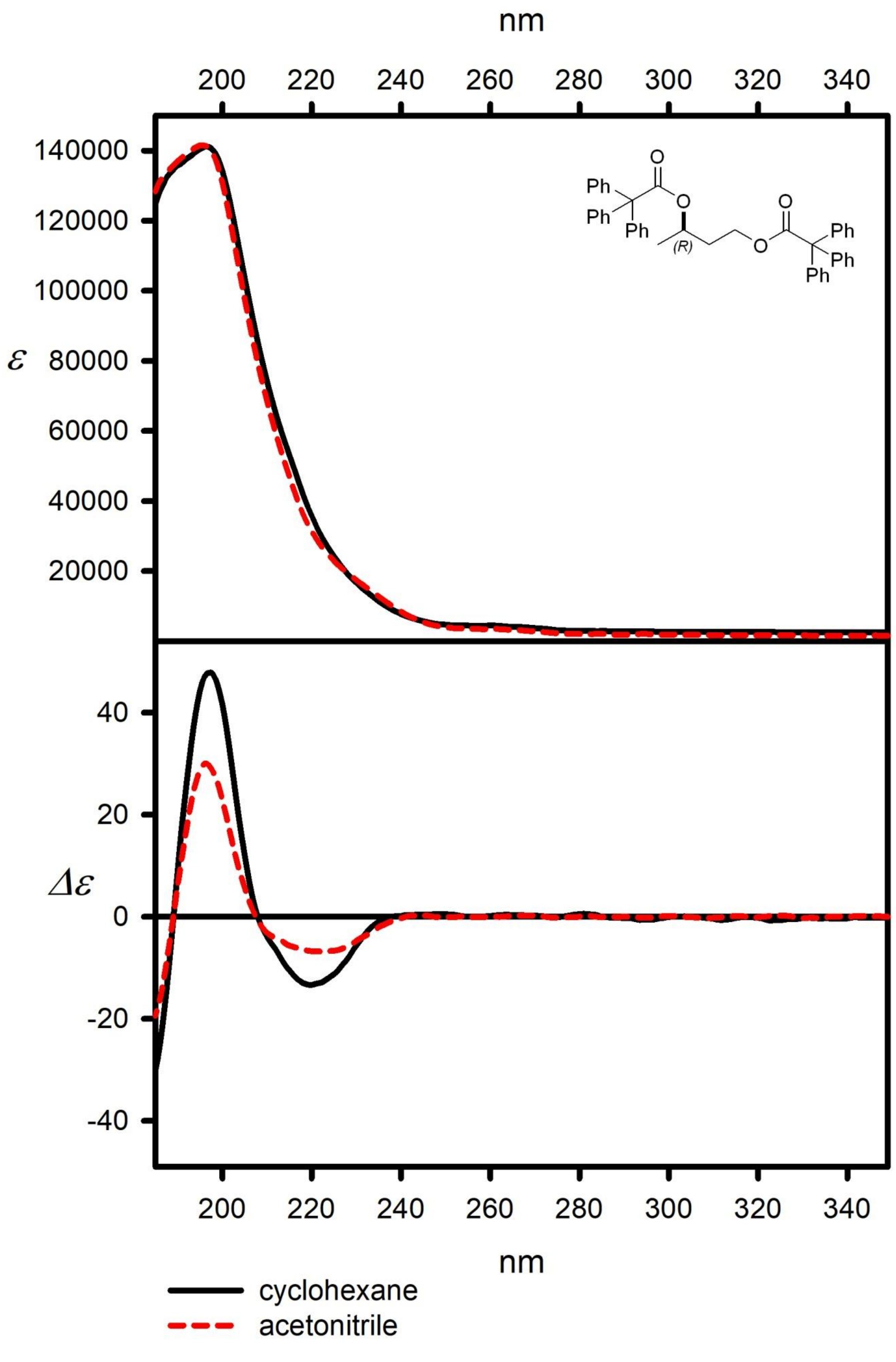
nm
200
220
240
260
280
300
320
340
140000
120000
100000
ε
80000
60000
40000
20000
Ph
O
O
Ph
Ph
(R)
O
O
O
Ph
Ph
Ph
Δε
40
20
0
-20
-40
cyclohexane
acetonitrile

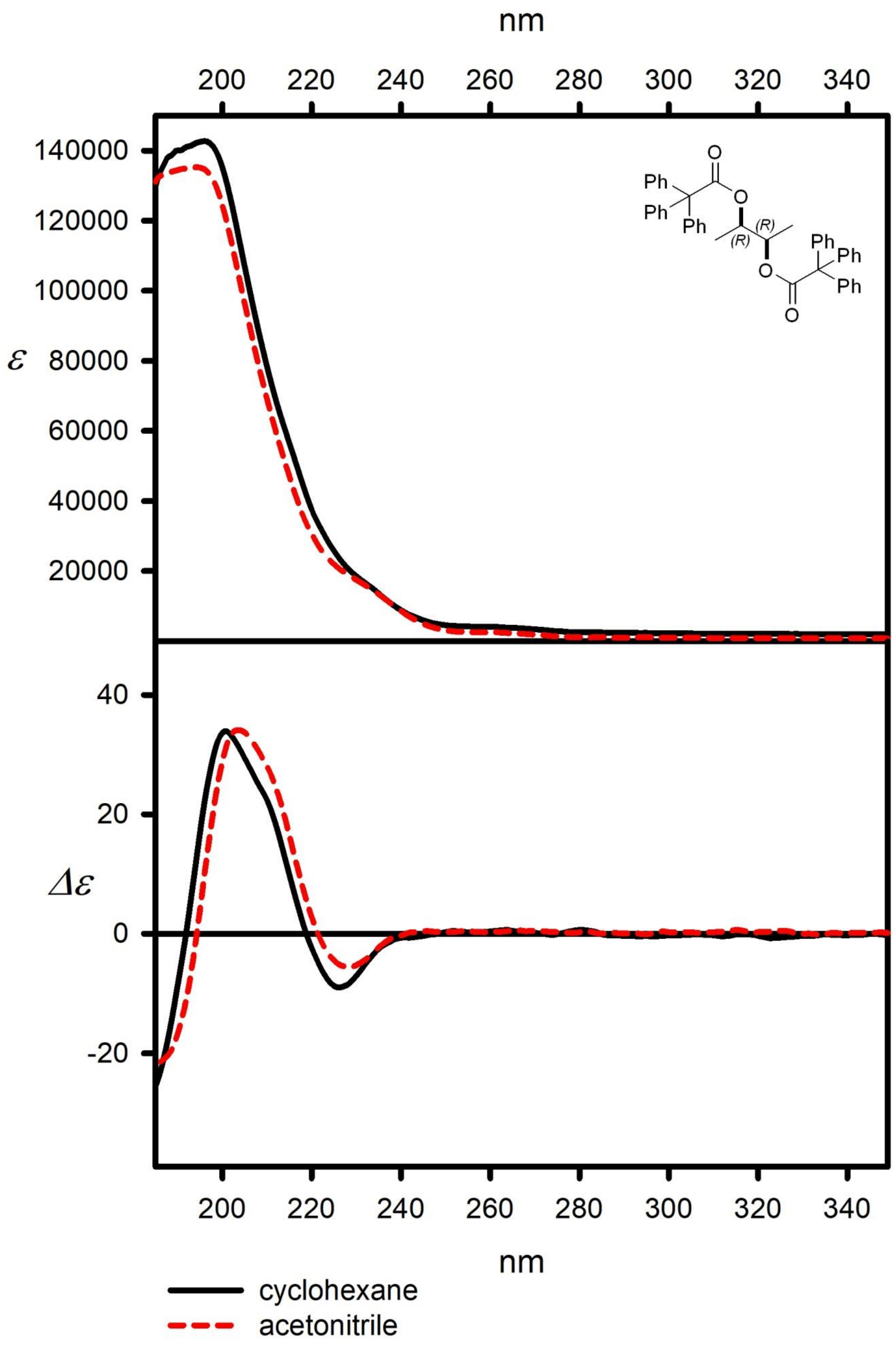

nm
200 220 240 260 280 300 320 340
140000 120000 100000 80000 60000 40000 20000
ε
40 20 0 -20
Δε
Ph Ph Ph O O (R) (R) O O Ph Ph Ph
cyclohexane
acetonitrile

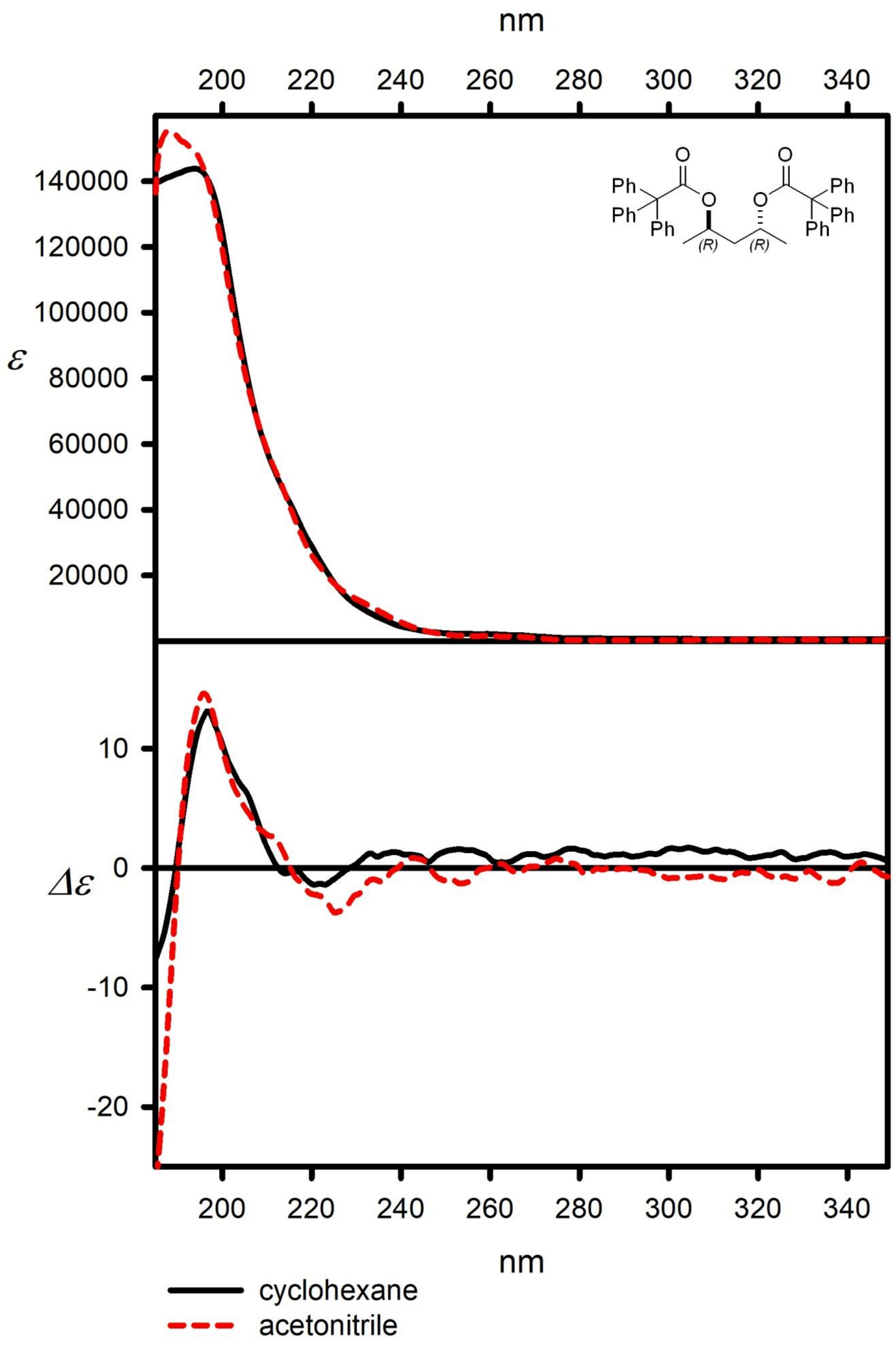
nm
200 220 240 260 280 300 320 340
140000
120000
100000
80000
60000
40000
20000
ε
Ph
Ph
Ph
O
O
(R)
(R)
Ph
Ph
Ph
10
0
-10
-20
Δε
nm
cyclohexane
acetonitrile

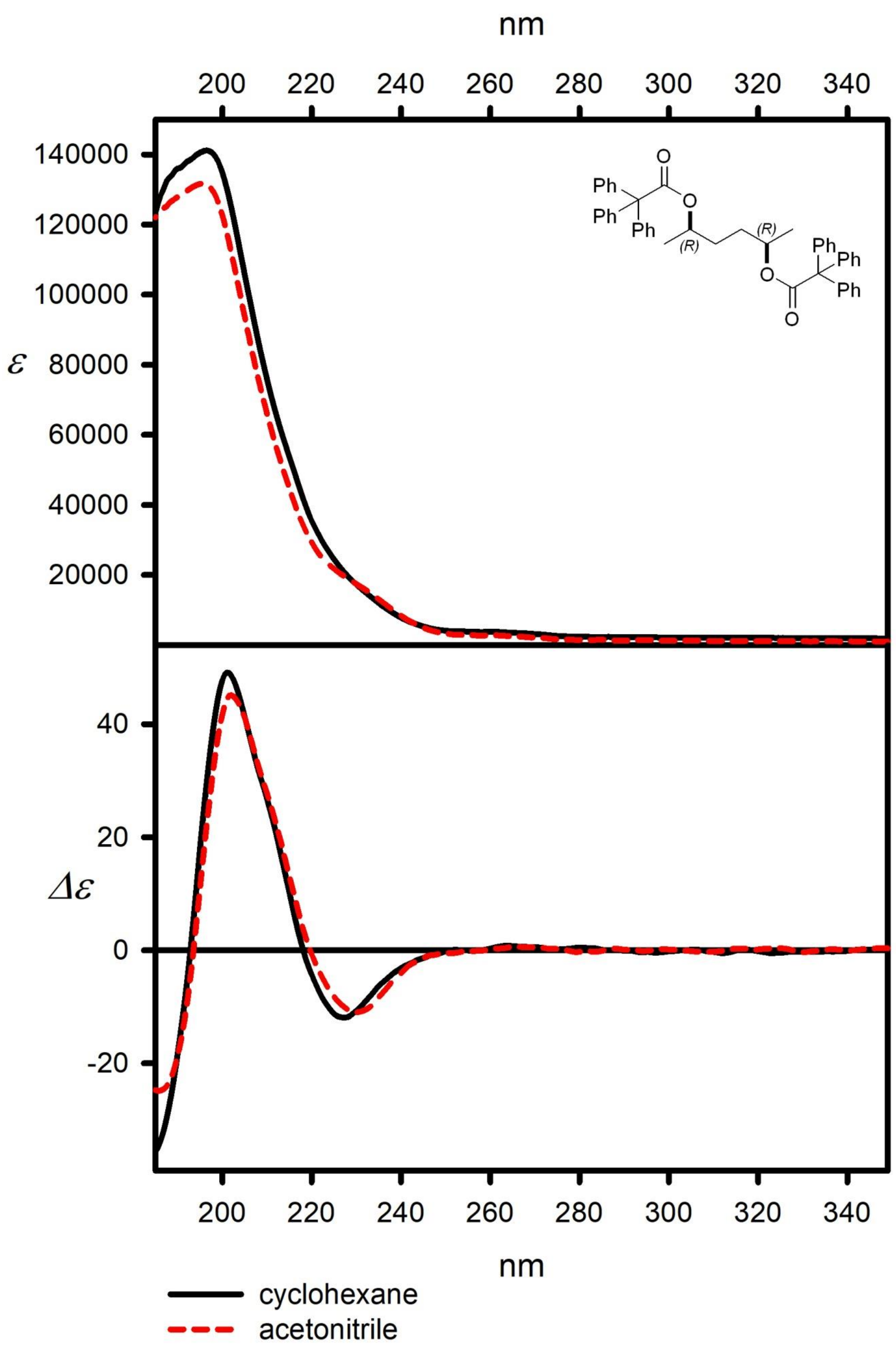
nm
200
220
240
260
280
300
320
340
140000
120000
100000
80000
60000
40000
20000
ε
Ph
Ph
Ph
O
O
(R)
(R)
Ph
Ph
Ph
O
O
40
20
0
-20
Δε
nm
cyclohexane
acetonitrile

Cartesian coordinates

1 (optimized at the B3LYP/6-311++G(d,p) level)

Conformer no	
1	O 1.74300100 -0.78681200 -0.59855500<br>C 0.61085500 -0.33759900 -1.16640700<br>C 2.87194500 -1.11236900 -1.47491800<br>H 2.83814900 -0.41258000 -2.31319700<br>C 4.13583100 -0.89758000 -0.64855200<br>H 4.08648900 -1.54025900 0.23761500<br>C 2.72349500 -2.53916300 -1.98922000<br>H 3.55630200 -2.78049900 -2.65522900<br>H 1.79767400 -2.65650000 -2.55478800<br>C 4.37633800 0.55614000 -0.23573500<br>H 3.55812500 0.93415500 0.37964900<br>O 0.46559100 -0.20848900 -2.35685400<br>C -0.43253000 0.08220600 -0.08528300<br>C -1.77723800 0.46253100 -0.75299200<br>C -2.35126600 -0.39298800 -1.70289900<br>C -3.60334200 -0.12585100 -2.24580100<br>C -4.32170500 0.99931500 -1.84260300<br>C -3.77202500 1.84774100 -0.88751100<br>C -2.51262600 1.58046400 -0.34835900<br>C 0.26170900 1.29288800 0.59189300<br>C 0.46134000 2.46114100 -0.15850600<br>C 1.13561800 3.55331100 0.37956400<br>C 1.63322000 3.49937000 1.68108600<br>C 1.46088900 2.33725000 2.42724000<br>C 0.78824600 1.24133000 1.88488200<br>C -0.77041100 -1.06803700 0.89352800<br>C -0.38941400 -2.39471100 0.67530100<br>C -0.79869100 -3.41339300 1.53758600<br>C -1.59757700 -3.12637600 2.63881500<br>C -1.99577100 -1.80872700 2.86290300<br>C -1.59411000 -0.79785600 1.99647800<br>H -1.81758700 -1.27609900 -2.02774000<br>H -4.01898700 -0.80028500 -2.98636400<br>H -5.29791400 1.20771200 -2.26593700<br>H -4.31881000 2.72366500 -0.55580600<br>H -2.10816100 2.25775200 0.39268600<br>H 0.08473500 2.51563300 -1.17341900<br>H 1.27223100 4.44652900 -0.22000300<br>H 2.15505700 4.35054400 2.10387000<br>H 1.85464100 2.27446000 3.43580300<br>H 0.68448200 0.34148800 2.47566500<br>H 0.23539100 -2.65419200 -0.16769300<br>H -0.48729700 -4.43350300 1.34127400<br>H -1.91339000 -3.91695800 3.31006800<br>H -2.62945200 -1.56725400 3.70918000<br>H -1.93627500 0.21422400 2.17547700

	H	2.73154800	-3.25368100	-1.16137700
	H	4.46720200	1.20515300	-1.11227800
	H	5.30077400	0.64628500	0.34080300
	H	4.97965300	-1.25759900	-1.24794400
17	O	-1.67057300	0.85389000	-0.69030900
	C	-0.59608900	0.23022700	-1.20219100
	C	-2.79162600	1.14832800	-1.58888300
	H	-2.36830500	1.33285700	-2.57797600
	C	-3.72255800	-0.06537800	-1.67971400
	H	-4.48341200	0.17104600	-2.43324900
	C	-3.43902600	2.41415200	-1.04743800
	H	-4.30711800	2.67675900	-1.65826200
	H	-2.73687000	3.24988700	-1.07551500
	C	-4.39708800	-0.48086700	-0.36851400
	H	-5.06844800	0.29446000	0.00985400
	O	-0.49751700	-0.09570800	-2.35927100
	C	0.45113200	-0.07183100	-0.08618300
	C	-0.31452100	-1.05763700	0.83420100
	C	-0.80642900	-0.69486500	2.09057200
	C	-1.55052600	-1.59464300	2.85458000
	C	-1.83016500	-2.86910500	2.36986900
	C	-1.36776100	-3.23392500	1.10608900
	C	-0.62213900	-2.33650400	0.34687200
	C	0.91891800	1.21748800	0.63124100
	C	0.65823800	2.49873700	0.13757500
	C	1.18320000	3.63182100	0.76090400
	C	1.97964000	3.50712700	1.89406500
	C	2.25807400	2.23433500	2.39121300
	C	1.74093000	1.10704800	1.76182300
	C	1.73436700	-0.69350100	-0.69007500
	C	2.39545100	-1.75890200	-0.07079600
	C	3.60848300	-2.24021300	-0.56515000
	C	4.18698500	-1.66248300	-1.69023500
	C	3.54504400	-0.59058100	-2.30898600
	C	2.33880800	-0.10889100	-1.81119300
	H	-0.61857200	0.29686500	2.47879500
	H	-1.91523200	-1.29110800	3.82979200
	H	-2.40727300	-3.56800900	2.96490800
	H	-1.58731800	-4.21856200	0.70825200
	H	-0.27283000	-2.63358900	-0.63523100
	H	0.04027200	2.63232800	-0.74027400
	H	0.96474600	4.61294900	0.35376200
	H	2.38546300	4.38711000	2.38010800
	H	2.88736900	2.11675300	3.26652500
	H	1.98956500	0.12696200	2.15026600
	H	1.96853200	-2.22799000	0.80620400
	H	4.09654900	-3.06944500	-0.06472600
	H	5.12714300	-2.03756900	-2.07891000
	H	3.98477300	-0.12451800	-3.18390100
	H	1.86566600	0.72776200	-2.30692000
	H	-3.76951500	2.28295200	-0.01557900
	H	-3.65916400	-0.69825300	0.40662400

	H	-4.99425500	-1.38343000	-0.52161300
	H	-3.14510200	-0.90129300	-2.08416500
33	O	1.78253100	0.23065700	-0.82087300
	C	0.54257000	-0.01534200	-1.27062000
	C	2.87357700	0.27696900	-1.79938600
	H	2.46340500	0.70564100	-2.71610800
	C	3.92964100	1.22225500	-1.22481900
	H	4.69804900	1.35448300	-1.99528800
	C	3.36175100	-1.13870000	-2.08186700
	H	4.21460500	-1.10471100	-2.76604500
	H	2.57331600	-1.72709200	-2.55320200
	C	4.57721600	0.77376900	0.08929200
	H	3.82360100	0.60217100	0.86062900
	O	0.28378400	-0.19718200	-2.42364100
	C	-0.47940000	-0.05532000	-0.09002800
	C	-0.68929500	1.36501000	0.49495700
	C	-0.01369200	2.49835200	0.03535400
	C	-0.27834900	3.76099200	0.57043300
	C	-1.22031100	3.91481000	1.58113100
	C	-1.90826000	2.79324700	2.04458700
	C	-1.65211600	1.53959800	1.50065800
	C	0.13495300	-1.04416200	0.93160900
	C	0.52866900	-0.67137400	2.21749500
	C	1.10912100	-1.59844700	3.08572600
	C	1.31795100	-2.91201500	2.67901100
	C	0.94707800	-3.29234800	1.38889600
	C	0.36595500	-2.36783300	0.52772800
	C	-1.86445300	-0.52359600	-0.60596900
	C	-2.46629800	0.14856900	-1.67958800
	C	-3.73624700	-0.20163300	-2.12371700
	C	-4.44668300	-1.22526800	-1.49624600
	C	-3.87100100	-1.88611500	-0.41743600
	C	-2.59230700	-1.53745100	0.02301400
	H	0.73156000	2.41509800	-0.74378300
	H	0.25807000	4.62315600	0.18964500
	H	-1.42413700	4.89466000	1.99789400
	H	-2.65356300	2.89525500	2.82568500
	H	-2.21520200	0.68489900	1.85601000
	H	0.39575200	0.34888900	2.55060200
	H	1.40359400	-1.28360900	4.08091600
	H	1.76873100	-3.63088800	3.35397800
	H	1.11050600	-4.31022100	1.05221000
	H	0.08194500	-2.68096700	-0.47132300
	H	-1.93908400	0.95185100	-2.17595000
	H	-4.17348100	0.32962000	-2.96193900
	H	-5.43803100	-1.49656500	-1.84174500
	H	-4.41226100	-2.67626400	0.09147300
	H	-2.17266600	-2.06402100	0.86958900
	H	3.67449600	-1.64116300	-1.16471100
	H	5.26359200	1.54227000	0.45428600
	H	5.15295900	-0.14709200	-0.03259800
	H	3.46332000	2.20299600	-1.08538700

38	O	-1.74869400	0.76533400	-0.32238700
	C	-0.75226600	0.15646400	-0.98723200
	C	-3.01786500	0.97528000	-1.02251900
	H	-2.78176400	1.16454600	-2.07250100
	C	-3.86389800	-0.29531000	-0.91214800
	H	-3.25817900	-1.13689100	-1.25730300
	C	-3.64088500	2.20561800	-0.38113000
	H	-4.57694900	2.46182700	-0.88179900
	H	-2.97016900	3.06376800	-0.45830300
	C	-5.15666900	-0.23640400	-1.73316400
	H	-5.83757200	0.54031100	-1.37573000
	O	-0.83681400	-0.20818200	-2.13396400
	C	0.47465300	-0.07488200	-0.05279200
	C	1.66170300	-0.68028500	-0.84247000
	C	2.45626400	-1.69767100	-0.30460400
	C	3.58889800	-2.15913800	-0.97644000
	C	3.95109400	-1.60956100	-2.20178500
	C	3.17430900	-0.58599300	-2.74298500
	C	2.04904500	-0.12331600	-2.06861900
	C	-0.09589400	-1.04950600	1.00972300
	C	-0.43714100	-2.35127200	0.61435600
	C	-1.02144400	-3.24378100	1.50832800
	C	-1.28637700	-2.85077200	2.81953500
	C	-0.97189600	-1.55450300	3.21719600
	C	-0.38921800	-0.66007100	2.31876700
	C	1.00982600	1.25146500	0.53808100
	C	2.01045400	1.20441500	1.51946100
	C	2.58455200	2.36765200	2.02112000
	C	2.18357200	3.61374900	1.54018400
	C	1.20712500	3.67562200	0.55185100
	C	0.62686000	2.50661100	0.05726900
	H	2.19674000	-2.14296900	0.64704200
	H	4.18464200	-2.95092800	-0.53551200
	H	4.82836700	-1.96943800	-2.72762100
	H	3.44517300	-0.14265900	-3.69489800
	H	1.46801400	0.67628200	-2.50727700
	H	-0.24182800	-2.67028200	-0.40308900
	H	-1.26996800	-4.24654000	1.17851100
	H	-1.73818000	-3.54500700	3.51919700
	H	-1.18400900	-1.22944900	4.22991900
	H	-0.17175100	0.34780100	2.64575300
	H	2.35377200	0.24613400	1.88989800
	H	3.35371100	2.29920400	2.78252800
	H	2.63316700	4.52162000	1.92611300
	H	0.89055300	4.63519700	0.15800900
	H	-0.13198900	2.59161200	-0.70895000
	H	-3.85062500	2.02692800	0.67675000
	H	-5.68866800	-1.18906200	-1.67479300
	H	-4.94618400	-0.04071900	-2.78914400
	H	-4.09103600	-0.47497100	0.14412400
39	O	1.80152600	-0.27735800	-0.74804000
	C	0.56842500	-0.07658300	-1.24194400

	C	2.92991600	-0.25320800	-1.68336100
	H	2.68096800	0.47432900	-2.45839900
	C	4.13567300	0.24842800	-0.88708600
	H	4.95669500	0.38998900	-1.59962600
	C	3.10405700	-1.62574600	-2.32324600
	H	3.97414900	-1.61533500	-2.98603700
	H	2.22849000	-1.88304800	-2.92115700
	C	4.59259300	-0.65335100	0.26377000
	H	3.78135800	-0.82527800	0.97441100
	O	0.33072100	0.04716600	-2.41797900
	C	-0.47498000	0.08067200	-0.09302700
	C	-0.51636500	-1.15042800	0.84326200
	C	-1.34066200	-1.10728400	1.97755700
	C	-1.47694400	-2.20986000	2.81384300
	C	-0.80327300	-3.39707300	2.52712000
	C	0.00160800	-3.46164500	1.39526300
	C	0.14462800	-2.34946600	0.56347700
	C	-1.90286900	0.20643900	-0.67880400
	C	-2.35343700	-0.72634700	-1.62252100
	C	-3.66077900	-0.69482000	-2.09518700
	C	-4.55782700	0.26436300	-1.62557800
	C	-4.12938800	1.18521100	-0.67578700
	C	-2.81474800	1.15505500	-0.20740400
	C	0.00100300	1.37983100	0.60888900
	C	0.58066200	1.39032000	1.87962000
	C	1.05864600	2.57688600	2.43763000
	C	0.97832300	3.77255700	1.73001800
	C	0.42257000	3.77107700	0.45110300
	C	-0.05666600	2.58774800	-0.10259000
	H	-1.89220200	-0.20283900	2.20462400
	H	-2.11886400	-2.14405300	3.68540400
	H	-0.91216100	-4.26007200	3.17418700
	H	0.52528300	-4.37937100	1.15086200
	H	0.78504200	-2.43267700	-0.30340100
	H	-1.67916100	-1.48542500	-1.99633100
	H	-3.97972500	-1.42350800	-2.83227500
	H	-5.57736100	0.28870400	-1.99380000
	H	-4.81456400	1.93369400	-0.29292700
	H	-2.50740600	1.88354100	0.53149800
	H	0.67109100	0.47041200	2.44126400
	H	1.49867600	2.55965000	3.42874600
	H	1.34904300	4.69374000	2.16545100
	H	0.36142400	4.69202700	-0.11824200
	H	-0.48038900	2.60208600	-1.10018800
	H	3.25903700	-2.40163800	-1.57052700
	H	5.41901600	-0.18582300	0.80525200
	H	4.94442500	-1.62526500	-0.09187000
	H	3.88816300	1.24104300	-0.49885200
40	O	-1.91209600	-0.02851800	-0.10410800
	C	-0.83077200	-0.14074600	-0.89408600
	C	-3.23312400	-0.00811600	-0.73636200
	H	-3.10620600	0.42016300	-1.73306200

	C	-3.74871800	-1.44355000	-0.86222900
	H	-2.99260700	-2.02893100	-1.39286400
	C	-4.08669900	0.90051000	0.13460800
	H	-3.62065500	1.88311500	0.22843500
	H	-4.20745800	0.47865100	1.13609100
	C	-5.08093400	-1.55162300	-1.61219700
	H	-5.01130200	-1.10551900	-2.60905600
	O	-0.88687900	-0.29176600	-2.08943400
	C	0.48030000	0.05788000	-0.07147100
	C	0.34813800	1.52758500	0.40658500
	C	0.38231500	2.55263900	-0.55051700
	C	0.19203400	3.88212400	-0.18543000
	C	-0.04394200	4.21686300	1.14753000
	C	-0.09976800	3.20615000	2.10277500
	C	0.08703300	1.87321600	1.73447400
	C	1.72144300	-0.17095300	-0.96827600
	C	2.83459500	0.67317700	-0.91788300
	C	3.98583300	0.39165900	-1.65530400
	C	4.04619800	-0.74274700	-2.45749300
	C	2.94692900	-1.59930100	-2.50689200
	C	1.80312500	-1.31953400	-1.76706500
	C	0.61033300	-0.96131300	1.08550800
	C	1.68301400	-0.83587900	1.98062000
	C	1.89184900	-1.76687800	2.99240600
	C	1.03981900	-2.86296900	3.12616500
	C	-0.01624600	-3.01153900	2.23409600
	C	-0.22880100	-2.06985400	1.22565500
	H	0.55893300	2.30720500	-1.59145700
	H	0.22808500	4.65624500	-0.94403800
	H	-0.18865800	5.25223200	1.43509500
	H	-0.29528100	3.44929400	3.14146200
	H	0.01835000	1.10284600	2.49043900
	H	2.81451200	1.56269100	-0.30168900
	H	4.83363900	1.06557300	-1.59784100
	H	4.93790800	-0.96078800	-3.03452500
	H	2.97898400	-2.49061500	-3.12380300
	H	0.96638700	-2.00327700	-1.81777500
	H	2.37062200	-0.00472800	1.88044100
	H	2.72808500	-1.64041200	3.67117500
	H	1.20373400	-3.59303700	3.91069800
	H	-0.68365700	-3.86236900	2.31662200
	H	-1.06457600	-2.21340500	0.55524200
	H	-5.07651400	1.03296600	-0.30767700
	H	-5.89498100	-1.05607000	-1.07747000
	H	-5.36346300	-2.59948500	-1.73962400
	H	-3.84582100	-1.87089300	0.14221000
41	O	1.80305700	-0.48936600	-0.39647000
	C	0.66613500	-0.23038100	-1.06482500
	C	3.04059200	-0.63241900	-1.16714400
	H	2.95688800	0.02831600	-2.03320300
	C	4.15345500	-0.15094100	-0.23808600
	H	3.86736900	0.83358700	0.14279000

	C	3.18385700	-2.07535100	-1.63455100
	H	3.24862200	-2.75427600	-0.77930900
	H	4.09114300	-2.19163100	-2.23190900
	C	5.52276900	-0.06153800	-0.91993200
	H	5.88876000	-1.04035300	-1.24021600
	O	0.59636400	-0.17421600	-2.26738900
	C	-0.50414400	0.06815100	-0.07511700
	C	-1.79535800	0.39076900	-0.86745700
	C	-2.24665600	-0.50099300	-1.85054800
	C	-3.44398800	-0.28237000	-2.52239400
	C	-4.23078400	0.82887600	-2.21883400
	C	-3.80486600	1.71155000	-1.23290600
	C	-2.59864400	1.49333700	-0.56404700
	C	0.01537400	1.28402500	0.73482400
	C	0.29800100	2.47509500	0.04890300
	C	0.82091600	3.58014000	0.71294000
	C	1.07892700	3.51800400	2.08239800
	C	0.81770700	2.33712400	2.76991400
	C	0.29640200	1.22805600	2.10107200
	C	-0.84002300	-1.15639300	0.81163000
	C	-0.20609400	-2.39581100	0.69325400
	C	-0.59962200	-3.48355800	1.47592800
	C	-1.63220000	-3.35300400	2.39738000
	C	-2.27860600	-2.12317200	2.52218200
	C	-1.89217700	-1.04528100	1.73360800
	H	-1.65848600	-1.37502900	-2.09690700
	H	-3.76424100	-0.98390700	-3.28483300
	H	-5.16490600	0.99884400	-2.74250300
	H	-4.40727400	2.57626800	-0.97668200
	H	-2.29240200	2.19563400	0.19993900
	H	0.10421600	2.53921300	-1.01625300
	H	1.02622800	4.49015800	0.15991100
	H	1.48351900	4.37852100	2.60338300
	H	1.02374100	2.26929600	3.83254600
	H	0.11907400	0.31558400	2.65401300
	H	0.60761400	-2.53166700	-0.00472200
	H	-0.09105200	-4.43419800	1.35840400
	H	-1.93628200	-4.19723500	3.00579600
	H	-3.09377000	-2.00473900	3.22751900
	H	-2.42397200	-0.10610900	1.82783500
	H	2.33728600	-2.36480000	-2.25922700
	H	6.26344400	0.35484100	-0.23292500
	H	5.48810600	0.58765400	-1.80046600
	H	4.20518600	-0.81881100	0.62873300
42	O	-1.90427600	0.06014300	-0.48328000
	C	-0.71483900	0.05471100	-1.10902500
	C	-3.10578800	0.28856800	-1.29369300
	H	-2.78879300	0.85089200	-2.17325500
	C	-3.68943000	-1.04848700	-1.76119400
	H	-4.49176700	-0.81800200	-2.47203300
	C	-4.03459400	1.13395800	-0.43520500
	H	-3.56307300	2.08722200	-0.18946400

	H	-4.28803900	0.62814900	0.49837200
	C	-4.22723400	-1.96257600	-0.65523000
	H	-5.09734700	-1.52932200	-0.15580400
	O	-0.58526700	0.11942400	-2.30671900
	C	0.45769100	0.08350900	-0.08174700
	C	0.34800100	-1.03585900	0.97781200
	C	-0.40748500	-2.19195800	0.76186500
	C	-0.39978200	-3.24089300	1.68104700
	C	0.36817900	-3.15663100	2.83817300
	C	1.13878200	-2.01621100	3.05977100
	C	1.13318700	-0.97472700	2.13689900
	C	0.32047500	1.51899300	0.49217400
	C	-0.26600500	1.78020800	1.73417300
	C	-0.45231900	3.08998300	2.17573800
	C	-0.07246000	4.16489300	1.37635600
	C	0.48564700	3.91788700	0.12322100
	C	0.67519800	2.60986300	-0.31449400
	C	1.81794100	-0.15513600	-0.78070200
	C	1.95630500	-1.15936700	-1.74719700
	C	3.19817500	-1.45623700	-2.30059200
	C	4.33689700	-0.76382400	-1.89261700
	C	4.21695400	0.22461300	-0.92060700
	C	2.97054700	0.52345000	-0.37090100
	H	-1.01281300	-2.28960900	-0.12960500
	H	-0.99705800	-4.12498200	1.48668400
	H	0.37357100	-3.97042400	3.55440500
	H	1.75386700	-1.93864400	3.94954200
	H	1.75615500	-0.10766000	2.31892800
	H	-0.58964400	0.96099300	2.36193300
	H	-0.90174300	3.26601300	3.14695200
	H	-0.21645000	5.18270900	1.72127500
	H	0.77381900	4.74350400	-0.51808200
	H	1.10028200	2.43483600	-1.29547000
	H	1.08994300	-1.71363200	-2.08140500
	H	3.27403400	-2.23231500	-3.05425100
	H	5.30405900	-0.99494600	-2.32477400
	H	5.09267400	0.76930700	-0.58496200
	H	2.90481700	1.29768300	0.38285400
	H	-4.96048100	1.33739000	-0.98069300
	H	-3.46937200	-2.15804900	0.10705500
	H	-4.53705600	-2.92344600	-1.07437100
	H	-2.91748300	-1.56658300	-2.33798900
43	O	-1.97909500	0.02678900	-0.31349800
	C	-0.82924600	0.10232700	-1.00951600
	C	-3.24967600	0.26288800	-1.00908800
	H	-3.01062700	0.49208500	-2.04846900
	C	-4.08671100	-1.01164900	-0.92587900
	H	-4.20043600	-1.28549400	0.12886200
	C	-3.90702700	1.46025500	-0.33690300
	H	-4.12067700	1.24881200	0.71436100
	H	-3.26124900	2.33894700	-0.39127200
	C	-3.52623700	-2.19059600	-1.72462900

	H	-4.17937800	-3.06274800	-1.63790800
	O	-0.76839600	0.27264700	-2.21632800
	C	0.41585700	0.07973400	-0.07246100
	C	0.64549300	1.60473600	0.12010000
	C	-0.19691800	2.34637800	0.96121200
	C	-0.05743200	3.72693900	1.07908800
	C	0.91724100	4.40042900	0.34492000
	C	1.74032600	3.67886900	-0.51498300
	C	1.60180300	2.29625100	-0.63071300
	C	1.63599800	-0.62169900	-0.72069500
	C	1.56973700	-1.39767900	-1.88133100
	C	2.69714600	-2.07258800	-2.35545200
	C	3.91176800	-1.98369400	-1.68503500
	C	3.99051400	-1.21449600	-0.52421300
	C	2.86582700	-0.54987400	-0.04899000
	C	0.15524900	-0.68989700	1.24416600
	C	-0.41398800	-1.96895800	1.18810500
	C	-0.57866200	-2.73721100	2.33480500
	C	-0.16195600	-2.24908500	3.57340700
	C	0.42520500	-0.99034400	3.64222700
	C	0.58358400	-0.22028800	2.48850400
	H	-0.96984000	1.84225700	1.52699000
	H	-0.71469200	4.27588500	1.74462900
	H	1.02717000	5.47520600	0.43642300
	H	2.49268200	4.18975500	-1.10559500
	H	2.24190700	1.75684100	-1.31579600
	H	0.64865700	-1.46686800	-2.44036500
	H	2.61690000	-2.66580400	-3.25984400
	H	4.78546100	-2.50665800	-2.05750800
	H	4.92724200	-1.13612000	0.01660900
	H	2.94541300	0.03002400	0.86269600
	H	-0.72653900	-2.37726400	0.23424100
	H	-1.02722100	-3.72183200	2.26057200
	H	-0.28787900	-2.84681900	4.46909800
	H	0.76641600	-0.59929500	4.59456900
	H	1.04887300	0.75327400	2.57096000
	H	-4.84874400	1.69781800	-0.83899400
	H	-2.53704200	-2.48297000	-1.36520500
	H	-3.43477300	-1.94333100	-2.78601600
	H	-5.08906900	-0.76567500	-1.29429700
44	O	-1.90261100	-0.06855400	-0.56074700
	C	-0.71103400	0.18077500	-1.12870600
	C	-3.10528000	0.17643500	-1.36469300
	H	-2.88428900	-0.15781100	-2.38133600
	C	-4.21572600	-0.67243100	-0.75474000
	H	-4.35681200	-0.36833000	0.28839600
	C	-3.42050500	1.66712200	-1.36618600
	H	-2.59864600	2.24066000	-1.79609600
	H	-3.60804800	2.02425400	-0.35029100
	C	-3.97719700	-2.18174100	-0.83668700
	H	-3.84203600	-2.50471600	-1.87399100
	O	-0.57839000	0.52545500	-2.27793100

	C	0.44454500	0.08971400	-0.08896500
	C	1.82277200	-0.18105800	-0.74161200
	C	2.97739600	0.04903300	0.02053300
	C	4.24108200	-0.27059500	-0.46468000
	C	4.38302000	-0.84044600	-1.72915700
	C	3.24585400	-1.08562000	-2.49129200
	C	1.97850100	-0.76151000	-2.00438400
	C	0.23915100	-1.08135900	0.90003700
	C	-0.18183400	-2.32579200	0.41484700
	C	-0.26879500	-3.43478200	1.25009700
	C	0.07489400	-3.32772200	2.59701600
	C	0.51358300	-2.10234300	3.08911100
	C	0.59771200	-0.99239300	2.24814100
	C	0.35946600	1.50191900	0.55269500
	C	-0.57328000	1.78093300	1.56083300
	C	-0.70997300	3.06857200	2.07382000
	C	0.06937800	4.11101000	1.57587000
	C	0.97576300	3.85342200	0.55071500
	C	1.11503200	2.56367600	0.04090700
	H	2.89110200	0.48221000	1.00973100
	H	5.11445800	-0.07727200	0.14847400
	H	5.36618200	-1.09128300	-2.11130300
	H	3.33659400	-1.52901800	-3.47689500
	H	1.11825100	-0.94788600	-2.62932300
	H	-0.43650500	-2.43834900	-0.63286700
	H	-0.60105200	-4.38497500	0.84650400
	H	0.00816200	-4.19022300	3.25043800
	H	0.79635100	-2.00377800	4.13148800
	H	0.94918000	-0.05350700	2.65598100
	H	-1.20312400	0.98871600	1.94302800
	H	-1.43092500	3.25601600	2.86208600
	H	-0.03613400	5.11335500	1.97566000
	H	1.57707400	4.65689200	0.13999200
	H	1.81622200	2.38577800	-0.76371000
	H	-4.31460600	1.85324700	-1.96753500
	H	-4.82919800	-2.73096900	-0.42748700
	H	-3.08974100	-2.47162400	-0.27129100
	H	-5.14158800	-0.41550300	-1.28134500
45	O	-1.85031800	0.43077900	-0.68650500
	C	-0.61941300	0.24674500	-1.20000000
	C	-2.94375100	0.72311100	-1.62196600
	H	-2.78321800	0.09824600	-2.50364200
	C	-4.23571300	0.32748500	-0.91385200
	H	-4.32063500	0.90907200	0.01099000
	C	-2.90528700	2.19422600	-2.01843200
	H	-3.05238800	2.83698600	-1.14617900
	H	-3.70602100	2.40228100	-2.73331700
	C	-4.35932800	-1.16706500	-0.61046600
	H	-4.29959900	-1.76143800	-1.52793300
	O	-0.39249000	0.27655600	-2.38348600
	C	0.44935800	-0.01873500	-0.08148200
	C	-0.13457800	-1.07599800	0.88481700

	C	-0.06552500	-0.94559600	2.27250200
	C	-0.55744900	-1.94848200	3.11134200
	C	-1.12583800	-3.09933500	2.57587600
	C	-1.19786300	-3.24270500	1.18956900
	C	-0.70707900	-2.24232800	0.35772800
	C	0.75227700	1.31572800	0.66705900
	C	-0.26821200	2.19377900	1.06075400
	C	0.01564000	3.35602300	1.77505100
	C	1.32599200	3.66749500	2.12879000
	C	2.34759200	2.79642200	1.76302300
	C	2.06373500	1.63742400	1.04143600
	C	1.74218800	-0.54696900	-0.75475300
	C	2.37569700	-1.71646100	-0.32600000
	C	3.56817700	-2.14889500	-0.91163900
	C	4.15223300	-1.41667100	-1.93838000
	C	3.53771200	-0.24007300	-2.36785700
	C	2.35284200	0.19025800	-1.78145300
	H	0.37406600	-0.05987200	2.71180700
	H	-0.49231800	-1.82295400	4.18656900
	H	-1.50816100	-3.87713700	3.22733300
	H	-1.63586200	-4.13496800	0.75586700
	H	-0.75909500	-2.37731500	-0.71775900
	H	-1.29555200	1.96342600	0.82249300
	H	-0.79638000	4.01656100	2.05929500
	H	1.54601300	4.57202400	2.68466500
	H	3.37436700	3.01362400	2.03621500
	H	2.87884100	0.97987000	0.77319700
	H	1.94949600	-2.30291800	0.47622800
	H	4.03513900	-3.06097600	-0.55647300
	H	5.07566300	-1.75233300	-2.39715500
	H	3.98240300	0.34696600	-3.16394000
	H	1.90105600	1.10986400	-2.12562000
	H	-1.95635100	2.44875000	-2.49142500
	H	-5.31889900	-1.38715400	-0.13525600
	H	-3.56645900	-1.50071800	0.06093400
	H	-5.06408300	0.64515400	-1.55695300
49	O	-1.85036800	0.43124000	-0.68624600
	C	-0.61955800	0.24703200	-1.19986000
	C	-2.94386200	0.72378200	-1.62163700
	H	-2.78334100	0.09907900	-2.50342900
	C	-4.23580200	0.32798800	-0.91349500
	H	-4.32043200	0.90918200	0.01162400
	C	-2.90548000	2.19493100	-2.01791300
	H	-3.05282000	2.83759900	-1.14564000
	H	-3.70608000	2.40295200	-2.73296900
	C	-4.35948900	-1.16669400	-0.61072000
	H	-3.56636700	-1.50072300	0.06019400
	O	-0.39280100	0.27695800	-2.38339300
	C	0.44931900	-0.01880300	-0.08147300
	C	1.74189400	-0.54733300	-0.75492900
	C	2.35254000	0.18974400	-1.78177600
	C	3.53733600	-0.24076000	-2.36820900

	C	4.15179400	-1.41737100	-1.93863800
	C	3.56777400	-2.14942700	-0.91176700
	C	2.37538900	-1.71681100	-0.32605100
	C	-0.13480000	-1.07599400	0.88482500
	C	-0.70723700	-2.24236900	0.35777800
	C	-1.19833300	-3.24256600	1.18962700
	C	-1.12662100	-3.09904000	2.57593900
	C	-0.55822200	-1.94819400	3.11139700
	C	-0.06604300	-0.94544500	2.27253300
	C	0.75279600	1.31556100	0.66702000
	C	-0.26731000	2.19411900	1.06060700
	C	0.01707800	3.35629900	1.77481400
	C	1.32754300	3.66712200	2.12865400
	C	2.34875900	2.79554600	1.76298700
	C	2.06440700	1.63667100	1.04141600
	H	1.90077500	1.10931800	-2.12604000
	H	3.98201800	0.34612700	-3.16441000
	H	5.07515400	-1.75317100	-2.39745100
	H	4.03468400	-3.06151100	-0.55654100
	H	1.94919900	-2.30313300	0.47628000
	H	-0.75894900	-2.37750800	-0.71770800
	H	-1.63631300	-4.13484400	0.75593500
	H	-1.50917200	-3.87673200	3.22739400
	H	-0.49331200	-1.82254200	4.18662400
	H	0.37352000	-0.05972000	2.71184400
	H	-1.29473500	1.96427800	0.82222400
	H	-0.79464100	4.01725000	2.05894800
	H	1.54798400	4.57152500	2.68457000
	H	3.37561500	3.01229000	2.03624600
	H	2.87920100	0.97870500	0.77323100
	H	-1.95647700	2.44960900	-2.49069100
	H	-4.30025500	-1.76066000	-1.52847900
	H	-5.31886100	-1.38689500	-0.13516500
	H	-5.06427600	0.64602700	-1.55626600
50	O	-1.80439400	0.07288800	-0.84688600
	C	-0.53098400	0.12042500	-1.27784900
	C	-2.87184100	0.13723300	-1.85218600
	H	-2.50832700	-0.39779500	-2.73150700
	C	-4.06478300	-0.61465600	-1.25941100
	H	-4.82889500	-0.67405000	-2.04316400
	C	-3.14460400	1.58872200	-2.22906500
	H	-2.26100800	2.03797800	-2.68395900
	H	-3.43256700	2.18316200	-1.35966900
	C	-4.66829200	-0.00958500	0.01191400
	H	-3.92185800	0.06425400	0.80578000
	O	-0.23424700	0.24042000	-2.44000500
	C	0.48823000	-0.01854300	-0.09293700
	C	1.88289200	-0.34065000	-0.68734900
	C	2.63852200	-1.43680900	-0.26368600
	C	3.91268800	-1.67932400	-0.78349900
	C	4.45652400	-0.82671000	-1.73677200
	C	3.71817900	0.27924400	-2.15922900

	C	2.45231600	0.52074700	-1.63819300
	C	-0.00517500	-1.16735800	0.81817900
	C	-0.38179300	-2.38815200	0.23969900
	C	-0.79558400	-3.45919900	1.02346700
	C	-0.84052300	-3.33468700	2.41291900
	C	-0.46597500	-2.13077900	2.99934900
	C	-0.05243300	-1.05609300	2.20825500
	C	0.55272500	1.32689900	0.69041500
	C	-0.59256700	2.08926600	0.95860800
	C	-0.51769200	3.26627000	1.70165500
	C	0.70159800	3.70529100	2.21045300
	C	1.84483100	2.94866400	1.96923600
	C	1.77071600	1.77684900	1.21779000
	H	2.24520700	-2.11276900	0.48305900
	H	4.47483000	-2.53852300	-0.43437000
	H	5.44385900	-1.01510300	-2.14364600
	H	4.12941200	0.95830000	-2.89807500
	H	1.90299700	1.38884200	-1.97474400
	H	-0.34145700	-2.50800800	-0.83814600
	H	-1.08140100	-4.39229000	0.55035100
	H	-1.16324500	-4.16784000	3.02713900
	H	-0.49360300	-2.01857200	4.07767500
	H	0.23204200	-0.12848800	2.68743700
	H	-1.55539400	1.75809300	0.59928700
	H	-1.42146900	3.83720300	1.88500500
	H	0.75927100	4.62050500	2.78889300
	H	2.80363700	3.26769700	2.36295700
	H	2.67548400	1.21044500	1.04388800
	H	-3.96015800	1.63144700	-2.95673400
	H	-5.48302400	-0.63851700	0.37975500
	H	-5.08047300	0.98739100	-0.16412400
	H	-3.74659100	-1.64221900	-1.05853400
52	O	-1.86702600	0.22462600	-0.43442800
	C	-0.68743600	0.14626900	-1.07725800
	C	-3.08267700	0.34952600	-1.24569500
	H	-2.91744900	-0.22634400	-2.15919300
	C	-4.19336300	-0.28658200	-0.41229300
	H	-3.85147200	-1.27704800	-0.09844200
	C	-3.31010200	1.81404700	-1.59759600
	H	-2.45529600	2.21713500	-2.14218300
	H	-3.47135200	2.41038700	-0.69494900
	C	-5.52188600	-0.41487900	-1.16515900
	H	-5.40072700	-0.98411400	-2.09214600
	O	-0.58838300	0.19648600	-2.27755700
	C	0.50868500	-0.02800400	-0.07725300
	C	0.09005900	-1.06786200	0.98899500
	C	-0.48104500	-2.28098400	0.57798100
	C	-0.83697500	-3.25841300	1.50031700
	C	-0.62762000	-3.04502000	2.86369900
	C	-0.05856000	-1.84799900	3.28414900
	C	0.29726100	-0.86802200	2.35428500
	C	0.82355300	1.34871100	0.58154200

	C	2.14873900	1.71401100	0.85339700
	C	2.45109900	2.91465200	1.49404600
	C	1.43434400	3.78475100	1.87710500
	C	0.11181900	3.43099000	1.62325400
	C	-0.19017900	2.22602500	0.99105100
	C	1.74375500	-0.51768500	-0.87598700
	C	2.20993700	0.22955000	-1.96914800
	C	3.33936100	-0.16273600	-2.67828900
	C	4.04234400	-1.30986600	-2.30881200
	C	3.60244600	-2.05098100	-1.21856600
	C	2.46483500	-1.65706000	-0.50925500
	H	-0.64008600	-2.46984900	-0.47876300
	H	-1.27656200	-4.18797200	1.15548100
	H	-0.90547900	-3.80457500	3.58579600
	H	0.11163000	-1.66718100	4.33984100
	H	0.73689200	0.05599100	2.70605400
	H	2.95874000	1.05781800	0.56565400
	H	3.48765600	3.16656700	1.68944500
	H	1.66851900	4.72222500	2.36889500
	H	-0.69506400	4.09142800	1.92184700
	H	-1.22546000	1.96257700	0.83306500
	H	1.68777100	1.12704900	-2.26942600
	H	3.67196700	0.43094700	-3.52269600
	H	4.92320100	-1.61592000	-2.86226100
	H	4.14055700	-2.94016700	-0.90885800
	H	2.15175300	-2.24933000	0.33953100
	H	-4.19075700	1.91633600	-2.23597300
	H	-5.94476900	0.55919500	-1.42380700
	H	-6.25875600	-0.93801500	-0.55096000
	H	-4.33255700	0.29932400	0.50287400
53	O	1.79630700	-0.57752700	-0.80167600
	C	0.63801400	-0.03205900	-1.19378500
	C	2.90299100	-0.61202400	-1.76461500
	H	2.45779300	-0.69906600	-2.75701700
	C	3.69233300	0.69964100	-1.70169700
	H	4.45931100	0.64863600	-2.48348700
	C	3.70177300	-1.86381600	-1.43463200
	H	4.55478800	-1.94734400	-2.11364800
	H	3.08155000	-2.75472600	-1.55114500
	C	4.34369700	1.01725500	-0.35210900
	H	3.59865700	1.06708700	0.44533400
	O	0.46203500	0.43477900	-2.29434000
	C	-0.44930200	-0.00704700	-0.06767900
	C	-1.83623100	-0.09311800	-0.75129300
	C	-2.05214000	-0.98084100	-1.81432000
	C	-3.30856700	-1.11556400	-2.39635000
	C	-4.38528000	-0.36425000	-1.92652800
	C	-4.18667200	0.51776800	-0.86956600
	C	-2.92416400	0.65162700	-0.28957900
	C	-0.30038000	1.37385800	0.64008600
	C	-0.53143000	1.51426900	2.01364100
	C	-0.46918300	2.76407400	2.62961500

	C -0.17238700 3.90250800 1.88559400<br>C 0.04653400 3.77851200 0.51548100<br>C -0.02449700 2.53112000 -0.10118100<br>C -0.25397500 -1.17594700 0.93551700<br>C -1.19960700 -2.19630100 1.07544200<br>C -1.01323000 -3.23787000 1.98808400<br>C 0.12214300 -3.27908400 2.78749200<br>C 1.07027500 -2.26215600 2.66901300<br>C 0.88279100 -1.22845500 1.75916900<br>H -1.23281100 -1.57514400 -2.19967100<br>H -3.44439400 -1.80544900 -3.22194900<br>H -5.36403300 -0.46455800 -2.38218400<br>H -5.01201300 1.11148700 -0.49226900<br>H -2.79486300 1.34960700 0.52700600<br>H -0.76777200 0.64577000 2.61382100<br>H -0.65504200 2.84201000 3.69526400<br>H -0.11828200 4.87344800 2.36508500<br>H 0.26707700 4.65593100 -0.08255000<br>H 0.11961400 2.46390200 -1.17172900<br>H -2.10020600 -2.18792600 0.47778600<br>H -1.76769800 -4.01259900 2.06993200<br>H 0.26713500 -4.08562000 3.49760000<br>H 1.95951300 -2.27192900 3.29001400<br>H 1.62952300 -0.44966700 1.69203500<br>H 4.07667900 -1.84350300 -0.40974800<br>H 4.84734600 1.98628600 -0.39398900<br>H 5.09152400 0.27135600 -0.07102100<br>H 3.01884600 1.51139600 -1.99013200

1 (optimized at the M06-2X/6-311++G(d,p) level)

Conformer no	
1	O -1.69643000 0.76038800 -0.65441700<br>C -0.54324600 0.36375200 -1.19990600<br>C -2.80097800 1.05438300 -1.53942300<br>H -2.73399900 0.36516200 -2.38591200<br>C -4.05982700 0.77907900 -0.73388900<br>H -4.08826600 1.47111600 0.11436100<br>C -2.68941600 2.48944900 -2.02269700<br>H -3.52678000 2.72835700 -2.68160200<br>H -1.76212600 2.63671300 -2.57870800<br>C -4.13993300 -0.66370500 -0.24174400<br>H -3.31255400 -0.88965900 0.43418700<br>O -0.34602100 0.31128800 -2.38263600<br>C 0.44322000 -0.07007500 -0.09103300<br>C 1.74816400 -0.60847600 -0.69935200<br>C 2.42965100 0.15334200 -1.65298400<br>C 3.65610000 -0.26387600 -2.15080200<br>C 4.23643600 -1.44416200 -1.69361700<br>C 3.57924200 -2.19602500 -0.73032400<br>C 2.34428200 -1.78033800 -0.23664100<br>C -0.34149500 -1.15409200 0.67099300

	C	-0.68213600	-2.32026000	-0.02360300
	C	-1.45124100	-3.30839000	0.57475800
	C	-1.90089600	-3.14551700	1.88297800
	C	-1.58327700	-1.98328600	2.57297500
	C	-0.81378000	-0.98906000	1.96952800
	C	0.83858000	1.12912300	0.78393100
	C	0.46189700	2.43863000	0.49659300
	C	0.90773900	3.49655200	1.28755600
	C	1.73275100	3.25951200	2.37733500
	C	2.12470800	1.95416100	2.66618200
	C	1.68927100	0.90435700	1.87119700
	H	1.99832300	1.08099000	-2.00817900
	H	4.16280900	0.33721800	-2.89660600
	H	5.19474000	-1.76882600	-2.08164000
	H	4.02252900	-3.11109500	-0.35510700
	H	1.85111300	-2.38007800	0.51825900
	H	-0.33521300	-2.45072900	-1.04435400
	H	-1.70174300	-4.20465000	0.01933500
	H	-2.49968700	-3.91550000	2.35482600
	H	-1.94044300	-1.83878500	3.58597900
	H	-0.59786800	-0.07946300	2.51527300
	H	-0.18528200	2.65385900	-0.34494900
	H	0.60438400	4.50817000	1.04424800
	H	2.07566700	4.08238800	2.99314200
	H	2.77983400	1.75496600	3.50614000
	H	2.01715600	-0.10693100	2.08844000
	H	-2.71358000	3.17431800	-1.17117500
	H	-4.09053900	-1.36492300	-1.07956700
	H	-5.07515500	-0.84310600	0.29160700
	H	-4.91861000	1.01661500	-1.36964600
17	O	-1.54946100	0.96747000	-0.83991400
	C	-0.52463700	0.21691200	-1.25635200
	C	-2.69967700	1.06938100	-1.71544100
	H	-2.33079500	1.15590800	-2.73953600
	C	-3.53037900	-0.20534500	-1.58463700
	H	-4.33892600	-0.15405500	-2.32102200
	C	-3.42225400	2.33515800	-1.29836100
	H	-4.34745300	2.44009500	-1.86924200
	H	-2.79740200	3.21047900	-1.48248900
	C	-4.09456000	-0.42889100	-0.18262900
	H	-4.86912800	0.30190600	0.06080000
	O	-0.43743700	-0.24589100	-2.36056100
	C	0.45433100	-0.05408400	-0.09268300
	C	-0.42145600	-0.88920800	0.86154900
	C	-0.88325100	-0.41728500	2.08743700
	C	-1.73826000	-1.19482500	2.86726200
	C	-2.15654500	-2.44282900	2.42460300
	C	-1.72171300	-2.91034400	1.18711100
	C	-0.86402000	-2.13930800	0.41401000
	C	0.99065000	1.24333000	0.52701700
	C	0.82279600	2.49290900	-0.06547500
	C	1.41377700	3.62737900	0.48781100

	C	2.17861900	3.52928800	1.64121600
	C	2.36216200	2.28270100	2.23521500
	C	1.78121600	1.15347800	1.67697500
	C	1.68643500	-0.82619900	-0.58802400
	C	2.21655700	-1.89793200	0.12751700
	C	3.40004300	-2.50971200	-0.28019800
	C	4.06818800	-2.05757600	-1.40961100
	C	3.55076800	-0.98042200	-2.12429000
	C	2.37614600	-0.36724400	-1.71292200
	H	-0.59143200	0.56481500	2.43655600
	H	-2.08362500	-0.81319800	3.82114800
	H	-2.82308100	-3.04352400	3.03217900
	H	-2.05168300	-3.87616700	0.82252900
	H	-0.52812900	-2.50673800	-0.55104700
	H	0.22796000	2.59816700	-0.96417400
	H	1.27167000	4.58956500	0.00959200
	H	2.63525100	4.41244600	2.07177000
	H	2.96745300	2.18979100	3.12930900
	H	1.94723600	0.18247400	2.13194800
	H	1.70844100	-2.26384600	1.01178800
	H	3.79516600	-3.34194300	0.29061300
	H	4.98624600	-2.53560400	-1.73039000
	H	4.06554000	-0.61446800	-3.00488000
	H	1.99064200	0.47704700	-2.27133500
	H	-3.66742600	2.30605700	-0.23567900
	H	-3.30483800	-0.35572500	0.56925400
	H	-4.53840600	-1.42272700	-0.10180800
	H	-2.89771000	-1.04938400	-1.87468900
33	O	1.68476200	0.39573200	-0.88551700
	C	0.47636200	-0.00657200	-1.28588000
	C	2.76056500	0.38108200	-1.85258000
	H	2.37595000	0.80370200	-2.78451800
	C	3.83867400	1.28644500	-1.27205400
	H	4.62255600	1.40241500	-2.02701600
	C	3.20543800	-1.05533200	-2.07847100
	H	4.10806200	-1.07326600	-2.69396700
	H	2.42456500	-1.61948200	-2.58895000
	C	4.43173400	0.77203700	0.03882900
	H	3.64018900	0.55388800	0.75970900
	O	0.21146200	-0.29818800	-2.41960500
	C	-0.48939800	-0.06586800	-0.07714100
	C	-0.80773600	1.35429500	0.41698200
	C	-0.29911200	2.50537200	-0.17948400
	C	-0.67138100	3.76839900	0.27960300
	C	-1.55154700	3.89756500	1.34379000
	C	-2.07181200	2.75212100	1.94263000
	C	-1.71021500	1.49697600	1.47651500
	C	0.26870000	-0.91297500	0.95845600
	C	0.70809200	-0.42006800	2.18266600
	C	1.46284500	-1.22293300	3.03790800
	C	1.79745000	-2.51992000	2.67459900
	C	1.37379900	-3.01662100	1.44363700

	C	0.61805400	-2.21917500	0.59680500
	C	-1.82696600	-0.69564000	-0.49735300
	C	-2.52121100	-0.13868700	-1.57598600
	C	-3.76763700	-0.62093900	-1.94483500
	C	-4.35590700	-1.66296900	-1.23191400
	C	-3.68593700	-2.20792700	-0.14624100
	C	-2.42907000	-1.72712300	0.21989200
	H	0.39268100	2.43665500	-1.00997400
	H	-0.26765600	4.65070200	-0.20299100
	H	-1.83952200	4.87936000	1.70008700
	H	-2.77054400	2.83787100	2.76649500
	H	-2.13888300	0.60987600	1.93138200
	H	0.48094200	0.59784600	2.47290200
	H	1.79649500	-0.82245600	3.98827600
	H	2.38817000	-3.13936200	3.33915700
	H	1.63425600	-4.02507500	1.14388800
	H	0.29117500	-2.60983800	-0.36302500
	H	-2.08053300	0.68259400	-2.12742500
	H	-4.28398400	-0.17884400	-2.78877100
	H	-5.33100200	-2.03870500	-1.51862800
	H	-4.13694300	-3.01064100	0.42557000
	H	-1.92705400	-2.16206800	1.07531100
	H	3.42128300	-1.53690600	-1.12254800
	H	5.09847400	1.51690000	0.47642500
	H	5.01162400	-0.14020400	-0.11534500
	H	3.39720900	2.27543100	-1.11729500
38	O	-1.55040200	0.97240600	-0.63489500
	C	-0.62585400	0.12720300	-1.10031500
	C	-2.83761800	0.98164200	-1.29565800
	H	-2.66488100	0.93539200	-2.37451500
	C	-3.61147900	-0.25085600	-0.84075400
	H	-3.02145300	-1.13606000	-1.09323500
	C	-3.48528000	2.29649500	-0.91029200
	H	-4.43419400	2.42183000	-1.43351200
	H	-2.83460500	3.13177000	-1.17345200
	C	-4.99165100	-0.35176600	-1.48642500
	H	-5.65291000	0.45259500	-1.15789000
	O	-0.72184600	-0.46890900	-2.13794700
	C	0.49636200	-0.07595900	-0.05935900
	C	1.68953700	-0.82306700	-0.66898100
	C	2.39846400	-1.77835100	0.05834700
	C	3.54987800	-2.36091800	-0.46465700
	C	4.01082600	-1.99382500	-1.72220300
	C	3.31676900	-1.03162200	-2.44949000
	C	2.17122300	-0.44887100	-1.92485000
	C	-0.26151000	-0.90140800	0.99946000
	C	-0.57946700	-2.23155700	0.70853000
	C	-1.36056700	-2.98234500	1.57841600
	C	-1.84697000	-2.41101500	2.75036000
	C	-1.55709500	-1.08160100	3.03443200
	C	-0.77764700	-0.32841300	2.16093100
	C	1.06188900	1.24789700	0.46863300

	C	1.85640500	1.23209300	1.61813300
	C	2.47360100	2.38800000	2.07552400
	C	2.32055700	3.58407600	1.37970700
	C	1.55102500	3.60635600	0.22421400
	C	0.92628700	2.44683700	-0.22793800
	H	2.05327300	-2.07809200	1.04101100
	H	4.08442000	-3.10355000	0.11609000
	H	4.90457900	-2.44910500	-2.13204200
	H	3.66845500	-0.73161800	-3.42954300
	H	1.64622200	0.30314200	-2.50078400
	H	-0.21602800	-2.67523500	-0.21255800
	H	-1.59218900	-4.01340100	1.33758900
	H	-2.45406400	-2.99576100	3.43146000
	H	-1.94425100	-0.62211100	3.93646500
	H	-0.58284200	0.71379700	2.38121500
	H	1.99645400	0.30039400	2.15591500
	H	3.08198100	2.35394700	2.97178900
	H	2.80342700	4.48753100	1.73258200
	H	1.43231000	4.52891900	-0.33183400
	H	0.32868300	2.48952500	-1.13069400
	H	-3.67025700	2.32547600	0.16620900
	H	-5.46916200	-1.29761000	-1.22681100
	H	-4.91663400	-0.30542400	-2.57645700
	H	-3.69445900	-0.21847400	0.25114400
39	O	1.76880400	-0.16329600	-0.76428100
	C	0.53326100	-0.04927300	-1.25638100
	C	2.87348500	-0.18451400	-1.69728100
	H	2.64591800	0.53177800	-2.49033700
	C	4.08454500	0.28785000	-0.90329900
	H	4.92146500	0.39729000	-1.60007200
	C	3.01222300	-1.57790300	-2.28879700
	H	3.89146100	-1.62009400	-2.93606200
	H	2.13316700	-1.82702400	-2.88457400
	C	4.46770100	-0.63968100	0.24850300
	H	3.60461900	-0.83080100	0.89044000
	O	0.28106600	0.00561200	-2.42884700
	C	-0.48369100	0.08960100	-0.10079600
	C	-0.42296200	-1.12244400	0.83954300
	C	-1.14602800	-1.08022400	2.03541900
	C	-1.18270200	-2.17533200	2.88652400
	C	-0.51033900	-3.34730100	2.54824900
	C	0.18812200	-3.40967700	1.35053200
	C	0.23147300	-2.30512700	0.50230200
	C	-1.92436300	0.13841800	-0.63091100
	C	-2.34971100	-0.80455300	-1.56999600
	C	-3.67304400	-0.84536600	-1.98661200
	C	-4.60361500	0.04678000	-1.46082100
	C	-4.19654100	0.97247300	-0.51010200
	C	-2.86702500	1.01687600	-0.09770400
	C	-0.04569600	1.41105800	0.55892000
	C	0.63024100	1.46319800	1.77536900
	C	1.08390700	2.67985600	2.28011400

	C	0.88259600	3.85601000	1.56949000
	C	0.23109800	3.80926500	0.34005800
	C	-0.22453900	2.59685300	-0.16059500
	H	-1.68864500	-0.17776400	2.29693700
	H	-1.74478600	-2.11797000	3.81129900
	H	-0.53970500	-4.20505300	3.20957000
	H	0.70509400	-4.31943500	1.06848300
	H	0.78504500	-2.38031300	-0.42599700
	H	-1.64108100	-1.51337100	-1.98018500
	H	-3.97986600	-1.57889400	-2.72296900
	H	-5.63655900	0.01443600	-1.78682000
	H	-4.91127500	1.66708500	-0.08409000
	H	-2.56938800	1.74623400	0.64617200
	H	0.82039400	0.55325700	2.33023800
	H	1.60413400	2.70191800	3.23063800
	H	1.23715800	4.80100900	1.96409300
	H	0.07796200	4.71764100	-0.23070900
	H	-0.72711300	2.56417900	-1.12211500
	H	3.13059700	-2.32141800	-1.49798300
	H	5.25180300	-0.18839400	0.85860800
	H	4.84368200	-1.59881900	-0.11390300
	H	3.85658600	1.28493200	-0.51602000
40	O	-1.88251000	0.07261900	-0.14328000
	C	-0.80257200	-0.13477700	-0.90277200
	C	-3.17565900	0.03836400	-0.78886300
	H	-3.05805400	0.46489600	-1.78875700
	C	-3.63500100	-1.41034700	-0.90398300
	H	-2.86614400	-1.96664700	-1.44713000
	C	-4.07568700	0.91056400	0.06441500
	H	-3.62510300	1.89607200	0.19238800
	H	-4.21549000	0.46149400	1.05085300
	C	-4.97268700	-1.54832500	-1.62775600
	H	-4.93624900	-1.06278600	-2.60669300
	O	-0.84807300	-0.37906400	-2.07727200
	C	0.48445700	0.06549800	-0.06825500
	C	0.36010600	1.52528300	0.40398000
	C	0.40835400	2.53396000	-0.56413800
	C	0.21903200	3.86442800	-0.21660600
	C	-0.02957500	4.21097000	1.10904400
	C	-0.09925000	3.21374700	2.07285000
	C	0.08686100	1.87799000	1.72246400
	C	1.73356700	-0.17858900	-0.92981700
	C	2.83832000	0.67012300	-0.89082900
	C	3.99576100	0.36551100	-1.60524900
	C	4.06547900	-0.79351000	-2.36532800
	C	2.97277400	-1.65584900	-2.39618200
	C	1.82332100	-1.35433900	-1.68056300
	C	0.55911900	-0.95041000	1.08045800
	C	1.60670100	-0.84069900	2.00028900
	C	1.76346400	-1.77031000	3.01805200
	C	0.88378700	-2.84507100	3.12552400
	C	-0.14446500	-2.97743100	2.20352400

	C -0.30626300 -2.03614900 1.18839100<br>H 0.59821600 2.26847400 -1.59965100<br>H 0.26592200 4.63109700 -0.98121100<br>H -0.17503900 5.24894900 1.38391800<br>H -0.30769900 3.46923400 3.10540100<br>H 0.00130000 1.11171600 2.48228600<br>H 2.80783100 1.57780900 -0.30024100<br>H 4.84229800 1.04087800 -1.56217700<br>H 4.96347900 -1.02894900 -2.92428900<br>H 3.01602300 -2.56859600 -2.97876800<br>H 0.98579000 -2.04032800 -1.70535000<br>H 2.30896100 -0.01843900 1.90976100<br>H 2.57922300 -1.66207200 3.72311100<br>H 1.00766000 -3.57587200 3.91580600<br>H -0.82859500 -3.81550200 2.26726700<br>H -1.11920500 -2.16476400 0.48426300<br>H -5.05122700 1.03274400 -0.40855200<br>H -5.78914100 -1.10113500 -1.05755000<br>H -5.21770600 -2.59932500 -1.78743700<br>H -3.70206600 -1.83118400 0.10525700
41	O 1.79187100 -0.38918800 -0.40058100<br>C 0.64567800 -0.22634300 -1.06792300<br>C 3.00397600 -0.55548400 -1.17004100<br>H 2.92694500 0.08517900 -2.05313200<br>C 4.12153600 -0.06837300 -0.26057100<br>H 3.87354500 0.94862200 0.05585100<br>C 3.13527500 -2.00808000 -1.59558700<br>H 3.23496900 -2.65046700 -0.71605700<br>H 4.01624500 -2.14232200 -2.22549100<br>C 5.48889700 -0.09138800 -0.93892300<br>H 5.81247000 -1.10969400 -1.16330500<br>O 0.56036300 -0.25856300 -2.26481900<br>C -0.50835400 0.06685000 -0.08036900<br>C -1.80922500 0.36706400 -0.84293100<br>C -2.25918500 -0.53006800 -1.81633500<br>C -3.47123000 -0.32925000 -2.46136700<br>C -4.26933400 0.76448800 -2.13484800<br>C -3.84378200 1.64731400 -1.15298000<br>C -2.62251400 1.44957800 -0.51164400<br>C -0.00206800 1.28423500 0.71464500<br>C 0.24279600 2.46685000 0.00785900<br>C 0.75856600 3.58535000 0.64630500<br>C 1.04475600 3.54011200 2.00920100<br>C 0.82164900 2.36552700 2.71378800<br>C 0.30728500 1.24069400 2.07016900<br>C -0.79383200 -1.15917400 0.80113800<br>C -0.14808000 -2.38300100 0.64467800<br>C -0.49538300 -3.47644600 1.43759000<br>C -1.48844400 -3.36012600 2.39912700<br>C -2.14784200 -2.14283200 2.55608700<br>C -1.80950000 -1.06018400 1.75819200<br>H -1.65613700 -1.39270400 -2.07149600

	H	-3.79667300	-1.03176800	-3.21965700
	H	-5.21661500	0.91957300	-2.63769800
	H	-4.45867900	2.49657100	-0.87838800
	H	-2.31184400	2.14938200	0.25417100
	H	0.01987400	2.50622200	-1.05448500
	H	0.93793400	4.49289000	0.08176500
	H	1.44469000	4.41263500	2.51204100
	H	1.05327000	2.31455200	3.77135100
	H	0.16271900	0.32695200	2.63195400
	H	0.63502800	-2.50355200	-0.09356800
	H	0.01721800	-4.42084900	1.29664400
	H	-1.75505900	-4.21004800	3.01609200
	H	-2.93412800	-2.03975500	3.29472000
	H	-2.34159900	-0.12141000	1.87111300
	H	2.26121000	-2.31425600	-2.17252700
	H	6.24445200	0.35906300	-0.29362500
	H	5.47245900	0.47171200	-1.87611800
	H	4.12997700	-0.68840600	0.64148600
44	O	1.79366000	0.30543700	-0.68455500
	C	0.61262100	-0.07072000	-1.18440000
	C	2.97477900	-0.00554600	-1.46037900
	H	2.77584900	0.27308000	-2.49937300
	C	4.09027900	0.85084300	-0.88721900
	H	4.26804800	0.54315900	0.14849200
	C	3.24327900	-1.49719600	-1.34795600
	H	2.43529000	-2.07150300	-1.80343000
	H	3.32414600	-1.77834900	-0.29424500
	C	3.78716800	2.34493000	-0.95116100
	H	3.55567200	2.65130400	-1.97538100
	O	0.45062900	-0.44990000	-2.31164900
	C	-0.46076400	-0.08051900	-0.07482300
	C	-1.86584900	-0.28708800	-0.65384200
	C	-2.81044000	-1.07308100	0.00525100
	C	-4.11714700	-1.16153100	-0.46663000
	C	-4.49897900	-0.46207700	-1.60414400
	C	-3.56659500	0.33594400	-2.25985400
	C	-2.26474400	0.42690200	-1.78546100
	C	-0.51392500	1.24454700	0.69368800
	C	-0.00790600	2.43028000	0.16462400
	C	-0.16521800	3.63608900	0.84362900
	C	-0.83494000	3.67388400	2.05938200
	C	-1.35997800	2.49743400	2.58742200
	C	-1.20587200	1.29792100	1.90605300
	C	0.02734100	-1.27760800	0.76604600
	C	0.75282900	-1.11508900	1.94501700
	C	1.28342500	-2.21922400	2.60626700
	C	1.11296100	-3.49807200	2.08917700
	C	0.41316900	-3.66677800	0.89832400
	C	-0.12055300	-2.56515600	0.24103400
	H	-2.52964900	-1.62684500	0.89367300
	H	-4.83466300	-1.78046800	0.05922000
	H	-5.51429400	-0.53408400	-1.97564600

	H	-3.85276700	0.89196600	-3.14496300
	H	-1.55255200	1.05648300	-2.30411600
	H	0.51743600	2.42556600	-0.78341500
	H	0.23827800	4.54664700	0.41591900
	H	-0.95526900	4.61198200	2.58790700
	H	-1.89781300	2.51512700	3.52802200
	H	-1.63194700	0.38877400	2.31697100
	H	0.91850200	-0.12256500	2.34476600
	H	1.83809100	-2.07410200	3.52599600
	H	1.52877700	-4.35584400	2.60447600
	H	0.28541200	-4.65675900	0.47612800
	H	-0.65043500	-2.69942300	-0.69624600
	H	4.17645000	-1.74983900	-1.85563000
	H	4.64103600	2.93299400	-0.61040000
	H	2.93172300	2.59111300	-0.31956700
	H	5.00072000	0.62277400	-1.44979700
45	O	-1.81050800	0.42395100	-0.74661500
	C	-0.57895200	0.25018000	-1.23939400
	C	-2.88495000	0.66570900	-1.68606200
	H	-2.74073500	-0.01885800	-2.52707800
	C	-4.16788800	0.33014400	-0.94312200
	H	-4.28879100	1.04192000	-0.11936200
	C	-2.83342200	2.10686700	-2.16321300
	H	-2.93604700	2.78684900	-1.31369200
	H	-3.65521400	2.29584200	-2.85715000
	C	-4.19644200	-1.09958800	-0.41127400
	H	-4.03969000	-1.81990900	-1.21950900
	O	-0.33409800	0.27152300	-2.41386700
	C	0.44805600	-0.00690200	-0.10204800
	C	-0.17986000	-1.04816200	0.83780200
	C	-0.16348300	-0.92227200	2.22242600
	C	-0.69169800	-1.93079000	3.02927300
	C	-1.23883200	-3.07251100	2.46113200
	C	-1.25620800	-3.20685100	1.07369500
	C	-0.73161700	-2.20296600	0.27405900
	C	0.72818200	1.32021400	0.64462400
	C	-0.31367300	2.16226600	1.05155200
	C	-0.05634100	3.31737900	1.78095500
	C	1.24551400	3.65350900	2.13537300
	C	2.28541000	2.81549700	1.75604700
	C	2.03017500	1.66244400	1.01866500
	C	1.74961800	-0.55202800	-0.71299900
	C	2.37309000	-1.69054400	-0.20618100
	C	3.59478200	-2.12580600	-0.71871100
	C	4.21209400	-1.42710600	-1.74521900
	C	3.60371100	-0.28062800	-2.25139000
	C	2.39033600	0.15458800	-1.73802300
	H	0.26409600	-0.03999300	2.68299100
	H	-0.67044700	-1.81670500	4.10680900
	H	-1.64802600	-3.85391000	3.09037700
	H	-1.68079200	-4.09323500	0.61692900
	H	-0.73783600	-2.31954200	-0.80664300

	H	-1.33685000	1.90452500	0.81719700
	H	-0.88279200	3.95225400	2.07902300
	H	1.44547800	4.55363300	2.70452600
	H	3.30621900	3.05380500	2.03159300
	H	2.86038300	1.02597400	0.74187700
	H	1.91807200	-2.24748300	0.60270000
	H	4.05872400	-3.01388900	-0.30598000
	H	5.16018400	-1.76607300	-2.14623500
	H	4.07823500	0.27956500	-3.04868400
	H	1.93964400	1.05658700	-2.13051500
	H	-1.89391400	2.31352300	-2.67543900
	H	-5.15775400	-1.32238300	0.05510700
	H	-3.41120300	-1.25188300	0.33098800
	H	-4.99973400	0.49973900	-1.63377400
50	O	-1.75276800	0.01494400	-0.92543300
	C	-0.48473500	0.21191600	-1.29786900
	C	-2.79439000	0.25202800	-1.90148500
	H	-2.42131700	-0.08833600	-2.86974000
	C	-3.96804400	-0.61532000	-1.46329300
	H	-4.76918200	-0.48951900	-2.19835100
	C	-3.09142700	1.74109500	-1.96620000
	H	-2.20253300	2.28982300	-2.28078800
	H	-3.41289800	2.11860500	-0.99366600
	C	-4.47795700	-0.30866800	-0.05632300
	H	-3.67051900	-0.40718000	0.67285100
	O	-0.16923900	0.55165600	-2.40460100
	C	0.49209900	-0.01391800	-0.10877500
	C	1.87121500	-0.42399800	-0.65488900
	C	2.54246800	-1.55075700	-0.18158600
	C	3.81568100	-1.87455800	-0.64920400
	C	4.44011600	-1.07477600	-1.59438500
	C	3.78580100	0.06226500	-2.06208700
	C	2.52063800	0.38741400	-1.59421300
	C	-0.07660600	-1.10470200	0.80891500
	C	-0.52383200	-2.30273300	0.24166700
	C	-0.98987900	-3.33896700	1.03543900
	C	-1.01278400	-3.19931900	2.42274800
	C	-0.56312000	-2.01913600	2.99591600
	C	-0.09714700	-0.97671700	2.19361800
	C	0.58386500	1.33520000	0.64221800
	C	-0.57792100	2.04830900	0.95689600
	C	-0.51404200	3.23523900	1.67676900
	C	0.71168900	3.72765700	2.11253400
	C	1.86894900	3.01562200	1.82591400
	C	1.80686100	1.83056100	1.09664200
	H	2.08491500	-2.18733100	0.56442200
	H	4.31414000	-2.75659800	-0.26458600
	H	5.42859400	-1.32733000	-1.95974300
	H	4.26424100	0.70361500	-2.79305500
	H	2.03435900	1.28336500	-1.95475800
	H	-0.49685000	-2.42546300	-0.83710900
	H	-1.33399100	-4.25788800	0.57513000

	H	-1.37625700	-4.00713900	3.04673500
	H	-0.57109000	-1.89952700	4.07300600
	H	0.25187700	-0.06316700	2.65963800
	H	-1.54371600	1.66169800	0.65753200
	H	-1.42788300	3.77205200	1.90361000
	H	0.76177500	4.65238200	2.67509500
	H	2.83079600	3.37878000	2.16887400
	H	2.72185200	1.29120600	0.88762500
	H	-3.88791600	1.92784100	-2.69007000
	H	-5.27021700	-1.00546000	0.22228000
	H	-4.88860600	0.70064400	0.01568000
	H	-3.64849800	-1.66029400	-1.51413800
52	O	-1.86596600	0.10987300	-0.42720500
	C	-0.68901300	0.18401700	-1.05925800
	C	-3.05872500	0.32643800	-1.21863400
	H	-2.90152200	-0.14881600	-2.19062700
	C	-4.17578200	-0.37383400	-0.46134900
	H	-3.87795900	-1.41486700	-0.30894900
	C	-3.27536500	1.81914400	-1.40450500
	H	-2.38943700	2.28047900	-1.84267800
	H	-3.49240500	2.29316000	-0.44305700
	C	-5.51312300	-0.30856500	-1.19504300
	H	-5.42139400	-0.69361600	-2.21437800
	O	-0.58876400	0.41032500	-2.23348500
	C	0.50029400	-0.03341400	-0.08378500
	C	0.09456400	-1.08009300	0.96254100
	C	-0.47602700	-2.28080300	0.52655800
	C	-0.81466800	-3.27929400	1.42608100
	C	-0.58765800	-3.09580500	2.78962400
	C	-0.02156700	-1.90957400	3.23193100
	C	0.31757300	-0.90571600	2.32395800
	C	0.81535500	1.32187700	0.58865300
	C	2.13198200	1.64252700	0.92996600
	C	2.42826600	2.82233900	1.60401100
	C	1.41493300	3.71071300	1.94489600
	C	0.10160500	3.39988500	1.61440200
	C	-0.19675600	2.21406200	0.95013800
	C	1.72242800	-0.50168800	-0.89469900
	C	2.20209900	0.30870100	-1.93099000
	C	3.33105700	-0.04991800	-2.65159000
	C	4.01686800	-1.22360100	-2.34591400
	C	3.56238100	-2.02450500	-1.30942700
	C	2.42362500	-1.66584800	-0.58697500
	H	-0.64620200	-2.43597800	-0.53523400
	H	-1.25419200	-4.20231700	1.06605200
	H	-0.85244300	-3.87341700	3.49622900
	H	0.15852600	-1.75390000	4.28924600
	H	0.75431000	0.01519100	2.69060600
	H	2.93614500	0.96454700	0.67179700
	H	3.45776000	3.04560600	1.85923000
	H	1.64620400	4.63319100	2.46401400
	H	-0.70113000	4.07891600	1.87733200

	H	-1.23042300	1.98032600	0.73278200
	H	1.68919400	1.23131900	-2.16788600
	H	3.67972100	0.59138600	-3.45250000
	H	4.90031700	-1.50315600	-2.90800500
	H	4.09169700	-2.93402900	-1.05047000
	H	2.09895500	-2.30178500	0.22649500
	H	-4.11641000	1.99897000	-2.07617100
	H	-5.89147900	0.71378600	-1.25446900
	H	-6.26440800	-0.90782600	-0.67894700
	H	-4.26166000	0.07896600	0.53157200
53	O	-1.56878600	0.73592800	-1.06952000
	C	-0.47430100	0.01663400	-1.31920700
	C	-2.70279400	0.57448600	-1.95507700
	H	-2.32160000	0.48971600	-2.97438400
	C	-3.43405800	-0.71195400	-1.57683300
	H	-4.25160900	-0.85331700	-2.29092300
	C	-3.52820400	1.83608000	-1.79582900
	H	-4.43681500	1.76574700	-2.39776800
	H	-2.95814000	2.70711300	-2.12166500
	C	-3.96704700	-0.72455700	-0.14446900
	H	-3.17160000	-0.49900100	0.57132600
	O	-0.31375900	-0.61761100	-2.32634300
	C	0.48907200	-0.00004800	-0.09987900
	C	1.82931500	-0.61192400	-0.52183900
	C	2.43639300	-0.22449200	-1.71979000
	C	3.69437600	-0.69797800	-2.06626300
	C	4.37490200	-1.56760100	-1.21815200
	C	3.78387200	-1.95404400	-0.02316200
	C	2.51950100	-1.48024000	0.32128200
	C	-0.22696600	-0.94000200	0.89771500
	C	-0.36285000	-0.64791600	2.25274700
	C	-1.00224700	-1.54293000	3.10952900
	C	-1.51090800	-2.74086500	2.62574500
	C	-1.35545600	-3.05299900	1.27791500
	C	-0.71314600	-2.16495400	0.42605700
	C	0.69415000	1.42079700	0.47363300
	C	1.95914400	2.00839800	0.51896300
	C	2.14102100	3.28937900	1.03923900
	C	1.06337400	4.00682100	1.53420600
	C	-0.20177200	3.42583400	1.51506700
	C	-0.38309700	2.15169000	0.99640300
	H	1.92482500	0.45854400	-2.38626400
	H	4.14365900	-0.38725500	-3.00225700
	H	5.35620300	-1.93863000	-1.48940600
	H	4.30256400	-2.62815100	0.64840000
	H	2.07517700	-1.79218000	1.25898500
	H	0.03328200	0.27703300	2.65203900
	H	-1.09827000	-1.29658900	4.16059900
	H	-2.01382000	-3.43075500	3.29298400
	H	-1.73088000	-3.99257100	0.88895900
	H	-0.57495000	-2.42921300	-0.61731100
	H	2.82451100	1.47269700	0.15315200

	H	3.13653400	3.71715700	1.05667200
	H	1.20370900	5.00231800	1.93885600
	H	-1.05473600	3.96531400	1.91037600
	H	-1.37171200	1.71406000	1.00537500
	H	-3.81242900	1.98314200	-0.75239900
	H	-4.36194500	-1.71100000	0.10539000
	H	-4.77259900	-0.00011700	-0.00569700
	H	-2.74263800	-1.54628900	-1.72613900

4 (optimized at the B3LYP/6-311++G(d,p) level)

Conformer no				
1	O	1.62566600	-0.50851900	-0.68420100
	C	0.42884000	-0.19034600	-1.20761900
	C	2.75305800	-0.68522100	-1.60483800
	H	2.59688900	0.01034300	-2.43193600
	C	4.02769800	-0.28267000	-0.84573500
	H	4.84636300	-0.47386300	-1.55250200
	C	4.02873600	1.21786800	-0.52210100
	H	3.22455100	1.47125400	0.17248300
	C	2.75151000	-2.10985400	-2.14794700
	H	3.60993400	-2.25283300	-2.81019700
	H	1.84635000	-2.29615600	-2.72835200
	C	4.28278900	-1.12077500	0.41573800
	H	4.37295300	-2.18689000	0.19374500
	O	0.22936000	-0.06629700	-2.39056900
	C	-0.62386700	0.07845100	-0.08692700
	C	-2.00651200	0.38361200	-0.71622600
	C	-2.80725300	1.43980700	-0.27259800
	C	-4.09082900	1.63545500	-0.78588000
	C	-4.59926100	0.77563000	-1.75307800
	C	-3.81505600	-0.28988200	-2.19469200
	C	-2.53935200	-0.48603500	-1.67774400
	C	-0.03064500	1.29879200	0.66358000
	C	0.45970800	1.22573000	1.96912700
	C	1.03400700	2.34115500	2.58123400
	C	1.14117100	3.54625200	1.89385300
	C	0.67659000	3.62524400	0.58120700
	C	0.10101500	2.51342000	-0.02604300
	C	-0.84304400	-1.15632900	0.82144500
	C	-0.28374600	-2.41094600	0.56513900
	C	-0.58430500	-3.51153300	1.37050600
	C	-1.44819900	-3.37963600	2.45161700
	C	-2.02100400	-2.13528600	2.71446300
	C	-1.72779200	-1.04387400	1.90457400
	H	-2.43798700	2.12360400	0.48047600
	H	-4.68879200	2.46451700	-0.42342600
	H	-5.59424300	0.92849400	-2.15592900
	H	-4.19796700	-0.97312400	-2.94475500
	H	-1.95413800	-1.32448400	-2.03117000
	H	0.40292800	0.29520500	2.51730600
	H	1.40206600	2.25986200	3.59814900

	H	1.58680500	4.41218600	2.37040700
	H	0.76242500	4.55358600	0.02731200
	H	-0.25302600	2.58989400	-1.04795300
	H	0.39823100	-2.54851300	-0.26199900
	H	-0.13671600	-4.47326800	1.14499300
	H	-1.67959100	-4.23378400	3.07790800
	H	-2.70588900	-2.01522900	3.54665000
	H	-2.20301700	-0.09259800	2.11122600
	H	3.89615900	1.82003000	-1.42597800
	H	4.97628500	1.51121400	-0.06175800
	H	2.81429000	-2.84709200	-1.34491400
	H	3.47436400	-0.99166700	1.13926900
	H	5.21501900	-0.80634800	0.89276100
20	O	-1.68501800	-0.07794300	-0.66203700
	C	-0.46168000	0.09395500	-1.19122400
	C	-2.84381100	0.10188000	-1.54322800
	H	-2.58766200	-0.35145600	-2.50381400
	C	-4.01905500	-0.67096500	-0.92266700
	H	-4.86438700	-0.47325400	-1.59511300
	C	-3.76762500	-2.18536000	-0.92713700
	H	-2.96263100	-2.45038700	-0.23775200
	C	-3.09909600	1.59154400	-1.74529800
	H	-3.23933200	2.10457900	-0.79241700
	H	-3.99847600	1.73192500	-2.35153500
	C	-4.40407700	-0.18334500	0.48172900
	H	-3.57750300	-0.32468400	1.18203900
	O	-0.26290500	0.30475200	-2.36224200
	C	0.63387900	0.08188000	-0.08035000
	C	0.28493000	1.36121300	0.72427500
	C	0.51723000	2.61220900	0.13451300
	C	0.14666000	3.78973200	0.77782900
	C	-0.47321500	3.74240500	2.02556300
	C	-0.72982200	2.50553300	2.61083500
	C	-0.36256200	1.32644300	1.96222300
	C	2.05603100	0.13457500	-0.69174100
	C	3.07609800	0.87588400	-0.08564000
	C	4.38380800	0.83273700	-0.56863500
	C	4.69988300	0.04278200	-1.66942800
	C	3.69560800	-0.71095200	-2.27444900
	C	2.39247500	-0.67063200	-1.78763000
	C	0.60265500	-1.22276000	0.74783300
	C	1.32025300	-1.30171900	1.94910400
	C	1.40176100	-2.49357600	2.66254400
	C	0.77927700	-3.64508200	2.18258700
	C	0.08165100	-3.58731400	0.98033900
	C	-0.00395100	-2.38866900	0.27187200
	H	0.99282800	2.66527800	-0.83779300
	H	0.34256800	4.74421200	0.30181500
	H	-0.75852200	4.65820200	2.53105000
	H	-1.22358900	2.45167200	3.57495200
	H	-0.59211500	0.37677600	2.42608300
	H	2.85761300	1.49943500	0.77170300

	H	5.15284600	1.42041900	-0.07927800
	H	5.71484500	0.01063600	-2.04946900
	H	3.92521200	-1.33575400	-3.13059000
	H	1.63442600	-1.26837200	-2.27427300
	H	1.83166500	-0.42574900	2.32874100
	H	1.96136200	-2.52364300	3.59098700
	H	0.84533400	-4.57546200	2.73513700
	H	-0.39905600	-4.47571500	0.58571100
	H	-0.54965700	-2.37748000	-0.66270400
	H	-3.49662900	-2.54403200	-1.92511400
	H	-4.66564000	-2.72411300	-0.61227700
	H	-2.26277300	2.05442600	-2.27004900
	H	-5.26206700	-0.74994000	0.85392600
	H	-4.67909700	0.87363700	0.49133900
22	O	-1.55134100	0.82434000	-0.33138600
	C	-0.55008000	0.23528900	-1.00731800
	C	-2.79373300	1.11815200	-1.05325900
	H	-2.51842000	1.32747500	-2.08886700
	C	-3.70197900	-0.12736600	-1.03980000
	H	-3.08212100	-0.94477200	-1.42297600
	C	-4.18699700	-0.51417300	0.36353100
	H	-3.35505300	-0.60539400	1.06476000
	C	-3.36446100	2.36640000	-0.39579300
	H	-4.30674900	2.64795700	-0.87091700
	H	-2.67067200	3.20295100	-0.50105000
	C	-4.87642700	0.04524700	-2.01510500
	H	-4.53006900	0.29731200	-3.02186200
	O	-0.61089700	-0.05739600	-2.17586900
	C	0.65253400	-0.06672700	-0.05983800
	C	0.03681700	-1.03607500	0.98137000
	C	-0.37116600	-2.30896800	0.55581600
	C	-0.99041000	-3.19563900	1.43176700
	C	-1.22411600	-2.82549200	2.75558600
	C	-0.84378400	-1.55695900	3.18352200
	C	-0.22526400	-0.66817000	2.30301600
	C	1.22910600	1.22895500	0.56040500
	C	0.86654400	2.50748100	0.12841400
	C	1.47958700	3.64641200	0.65331800
	C	2.46848100	3.53008600	1.62395700
	C	2.84880300	2.25994700	2.05680500
	C	2.24235000	1.12710100	1.52494100
	C	1.82431300	-0.70520400	-0.84715600
	C	2.55290400	-1.78231800	-0.33304400
	C	3.67164000	-2.28119600	-1.00178500
	C	4.08576700	-1.70966600	-2.20012200
	C	3.37626900	-0.62595300	-2.71640600
	C	2.26532800	-0.12619500	-2.04492600
	H	-0.19952200	-2.61094300	-0.47114900
	H	-1.28996500	-4.17618600	1.07843300
	H	-1.70315600	-3.51529700	3.44131300
	H	-1.03090300	-1.24901800	4.20646400
	H	0.04449600	0.31866300	2.65428600

	H	0.09907800	2.63522400	-0.62302900
	H	1.17860500	4.62534200	0.29666600
	H	2.94362100	4.41445300	2.03313600
	H	3.62730000	2.14915400	2.80359500
	H	2.57049000	0.14954900	1.85710900
	H	2.25250400	-2.24569800	0.59747100
	H	4.21551600	-3.11906500	-0.57938800
	H	4.95192600	-2.09820900	-2.72399800
	H	3.68920200	-0.16420800	-3.64641500
	H	1.73738500	0.71992400	-2.46272600
	H	-4.70239400	-1.47797300	0.33058900
	H	-4.89383600	0.21980700	0.76239700
	H	-3.54609100	2.20777100	0.66863900
	H	-5.56593600	0.82904000	-1.68679800
	H	-5.44948300	-0.88323200	-2.08501100
42	O	1.61517400	-0.38424100	-0.52217800
	C	0.44922500	-0.11164800	-1.13165500
	C	2.83119300	-0.41819900	-1.33992400
	H	2.67981800	0.28833200	-2.15844200
	C	3.96270400	0.09900600	-0.43475100
	H	3.60065200	1.05633300	-0.04269200
	C	4.25705000	-0.81507200	0.76217500
	H	4.70188900	-1.76353400	0.44591800
	C	3.01316600	-1.81878500	-1.91336800
	H	3.07487600	-2.56903000	-1.12225400
	H	3.92922300	-1.87024400	-2.50677400
	C	5.23193700	0.38084000	-1.25254300
	H	5.02896400	1.04466300	-2.09842000
	O	0.33462200	0.03341400	-2.32351200
	C	-0.69181800	0.08423800	-0.08530900
	C	-2.03874300	0.35120700	-0.80136300
	C	-2.46411100	-0.50071300	-1.82982700
	C	-3.70980900	-0.33891700	-2.42626400
	C	-4.57068500	0.67218900	-1.99999500
	C	-4.16927900	1.51326800	-0.96803200
	C	-2.91559300	1.35309200	-0.37533200
	C	-0.19863900	1.30767100	0.73081100
	C	0.21858200	1.21915200	2.06050400
	C	0.71802000	2.33764200	2.72964800
	C	0.82267100	3.56223800	2.07716200
	C	0.43023200	3.65813300	0.74231500
	C	-0.07161400	2.54302700	0.07816800
	C	-0.91921400	-1.18237400	0.77496700
	C	-0.30294000	-2.40898300	0.51342200
	C	-0.60786100	-3.54350300	1.26819300
	C	-1.53433000	-3.47436900	2.30249200
	C	-2.16512400	-2.25906300	2.56840700
	C	-1.86653600	-1.13353500	1.80851400
	H	-1.81823100	-1.29743700	-2.17368900
	H	-4.00903400	-1.00667800	-3.22662900
	H	-5.54206600	0.79777800	-2.46509000
	H	-4.82803000	2.30017400	-0.61718700

	H	-2.62854100	2.02204600	0.42551700
	H	0.16503400	0.27385900	2.58331000
	H	1.03026400	2.24364500	3.76401900
	H	1.20996300	4.43054100	2.59836000
	H	0.51353500	4.60252400	0.21575900
	H	-0.36973300	2.63364500	-0.96029300
	H	0.42623200	-2.49869900	-0.27960800
	H	-0.11504900	-4.48219000	1.03988400
	H	-1.76955000	-4.35495400	2.88950500
	H	-2.89935300	-2.18855000	3.36336200
	H	-2.38635000	-0.20582400	2.01472200
	H	3.34917600	-1.03346900	1.32806300
	H	4.96736300	-0.33234400	1.43922500
	H	2.17848600	-2.06725800	-2.57105800
	H	5.67491200	-0.53944700	-1.64490600
	H	5.98629800	0.86348200	-0.62557300
44	O	-1.72403600	0.27458500	-0.09982900
	C	-0.65022600	0.22047200	-0.90583300
	C	-3.01655600	0.62755700	-0.69787800
	H	-2.80108500	1.17041000	-1.61932200
	C	-3.77284300	-0.66433300	-1.06309000
	H	-3.07106900	-1.25083500	-1.66722800
	C	-4.17188300	-1.50346800	0.15838300
	H	-4.94849400	-1.00783500	0.74864600
	C	-3.70124900	1.54501400	0.30510700
	H	-3.11118200	2.45237900	0.44772400
	H	-3.82158200	1.05816800	1.27484700
	C	-4.98643600	-0.35458400	-1.95228000
	H	-4.70099600	0.22653600	-2.83391000
	O	-0.70048400	0.38013200	-2.10145700
	C	0.66533600	0.06577000	-0.08743900
	C	0.46117100	-0.82144400	1.16317800
	C	-0.21170900	-2.04289100	1.02928300
	C	-0.32702800	-2.92466000	2.09812400
	C	0.24153400	-2.60964600	3.33221300
	C	0.92855000	-1.40852000	3.47494400
	C	1.03888900	-0.52485600	2.40018500
	C	0.98316200	1.55474100	0.22409300
	C	0.28667900	2.23916500	1.23048900
	C	0.50442600	3.59589300	1.45686800
	C	1.41154100	4.30402400	0.67056500
	C	2.08752900	3.64227800	-0.35063000
	C	1.87122900	2.28332800	-0.57492300
	C	1.80264600	-0.62063900	-0.88540100
	C	1.60887200	-1.33393100	-2.07216800
	C	2.66943000	-2.00721100	-2.68274800
	C	3.94241700	-1.98172700	-2.12464400
	C	4.14811500	-1.27770700	-0.93843400
	C	3.09052100	-0.61316300	-0.32821800
	H	-0.64409600	-2.31697900	0.07351200
	H	-0.85683900	-3.86160500	1.96567100
	H	0.15429400	-3.29535200	4.16739600

	H	1.38549800	-1.15206000	4.42453100
	H	1.58462600	0.39960400	2.53747200
	H	-0.43587900	1.70994300	1.83774400
	H	-0.03956200	4.09959700	2.24849200
	H	1.58204600	5.36015500	0.84698700
	H	2.78444200	4.18191800	-0.98223900
	H	2.39643800	1.79128000	-1.38253800
	H	0.63869700	-1.35391900	-2.54461100
	H	2.49041900	-2.54980700	-3.60462000
	H	4.76364100	-2.50373200	-2.60289900
	H	5.13209800	-1.24974400	-0.48340200
	H	3.27048800	-0.08283800	0.59916000
	H	-3.31790400	-1.69272000	0.81252800
	H	-4.57325300	-2.46935400	-0.16135600
	H	-4.68805200	1.83883900	-0.06024100
	H	-5.75360900	0.20593600	-1.40958600
	H	-5.44762400	-1.28249600	-2.30093200
46	O	1.71171400	-0.32454800	-0.68589300
	C	0.47734100	-0.12062300	-1.16442600
	C	2.80175100	-0.50113600	-1.65254000
	H	2.51867600	0.04650700	-2.55319600
	C	4.05987000	0.14427400	-1.05080500
	H	4.84350600	-0.02034900	-1.80258500
	C	3.87916100	1.66042200	-0.88879800
	H	3.11720800	1.88461400	-0.13781100
	C	2.92563600	-1.98246400	-1.98598400
	H	2.01926800	-2.33850500	-2.47894300
	H	3.09229800	-2.58247000	-1.08932400
	C	4.51863200	-0.49967100	0.26558100
	H	4.74125600	-1.56310900	0.14890200
	O	0.22423800	-0.05476400	-2.34442600
	C	-0.61670800	0.01860500	-0.05558500
	C	-1.50429700	1.22376300	-0.45971100
	C	-2.88885700	1.21135700	-0.27754500
	C	-3.66442500	2.32913100	-0.58945100
	C	-3.06928600	3.48148800	-1.09247600
	C	-1.68787900	3.50722700	-1.27982100
	C	-0.91808900	2.39155300	-0.96606700
	C	0.01982000	0.23857100	1.34121300
	C	0.77090200	-0.78629700	1.93942800
	C	1.34386400	-0.62403900	3.19469200
	C	1.17982200	0.57267400	3.89470100
	C	0.43359800	1.59391300	3.32043100
	C	-0.14264600	1.42657000	2.05779300
	C	-1.46504000	-1.29098700	-0.06469200
	C	-1.74315700	-1.98161700	-1.25189500
	C	-2.55333500	-3.11605700	-1.24889400
	C	-3.11987000	-3.57962000	-0.06457000
	C	-2.86993200	-2.89030000	1.11888300
	C	-2.05191900	-1.76158000	1.11782900
	H	-3.37651400	0.32488700	0.10556400
	H	-4.73806500	2.29041900	-0.44168100

	H -3.67214300 4.34790500 -1.34035000
	H -1.20742800 4.39557900 -1.67472700
	H 0.15391200 2.43764000 -1.11995400
	H 0.90489800 -1.72523100 1.41791300
	H 1.91766100 -1.43504000 3.62997400
	H 1.62412100 0.69995800 4.87560600
	H 0.28733500 2.52818600 3.85135800
	H -0.72756500 2.23592500 1.64325400
	H -1.33817500 -1.62702400 -2.18938100
	H -2.74452400 -3.63325200 -2.18281400
	H -3.75067000 -4.46151600 -0.06446300
	H -3.30976300 -3.22785000 2.05100500
	H -1.87454100 -1.24481900 2.05162500
	H 3.57955800 2.13026700 -1.83063700
	H 4.81327200 2.12756000 -0.56541300
	H 3.76549500 -2.14046400 -2.66846800
	H 3.75401900 -0.39353300 1.03890700
	H 5.42955200 -0.01272100 0.62444600

4 (optimized at the M06-2X/6-311++G(d,p) level)

Conformer no	
1	O -1.56743500 0.49260400 -0.73644500
	C -0.36478800 0.20821400 -1.24186100
	C -2.67234100 0.65940400 -1.65371700
	H -2.50747200 -0.02562300 -2.48969900
	C -3.92413500 0.22785400 -0.88868800
	H -4.75488100 0.32416600 -1.59853700
	C -3.80505200 -1.23750100 -0.46631700
	H -3.01729000 -1.35630300 0.28250400
	C -2.68862600 2.09104800 -2.16124900
	H -3.56530100 2.25459500 -2.79230200
	H -1.79513600 2.29059600 -2.75532700
	C -4.20288200 1.11635800 0.32494700
	H -4.41947200 2.14847200 0.04224400
	O -0.13068300 0.14460000 -2.41726400
	C 0.63222900 -0.08151600 -0.09560600
	C 2.00560100 -0.48528000 -0.65447300
	C 2.72223900 -1.56600600 -0.14341600
	C 4.01035000 -1.84518900 -0.59585100
	C 4.60088600 -1.04600300 -1.56426800
	C 3.89868800 0.04530100 -2.06903200
	C 2.61893600 0.32655700 -1.61313700
	C -0.05055900 -1.21960000 0.68521900
	C -0.59538400 -1.05050600 1.95508800
	C -1.27403400 -2.09686500 2.57850200
	C -1.42893400 -3.31792400 1.93572300
	C -0.90765100 -3.48765800 0.65528400
	C -0.22855800 -2.44651500 0.03700800
	C 0.86911500 1.17908700 0.75118600
	C 0.35113800 2.42919500 0.42267700
	C 0.66140400 3.55138200 1.19053000

	C	1.48926200	3.43755000	2.29762100
	C	2.01814400	2.19227200	2.63011300
	C	1.71716000	1.07963300	1.85898000
	H	2.28369300	-2.19926300	0.61793400
	H	4.54810700	-2.69096800	-0.18343900
	H	5.60118500	-1.26436800	-1.91911900
	H	4.35114600	0.68382700	-2.81863300
	H	2.09123300	1.18745900	-2.00399300
	H	-0.50573100	-0.09810400	2.46207100
	H	-1.68922000	-1.94790700	3.56861400
	H	-1.95794600	-4.12883800	2.42242100
	H	-1.03329300	-4.43083700	0.13656800
	H	0.17269900	-2.58241800	-0.96294600
	H	-0.30183800	2.54714100	-0.43342200
	H	0.25111500	4.51562500	0.91414800
	H	1.72957400	4.31002700	2.89359800
	H	2.67588100	2.09100600	3.48537600
	H	2.15003400	0.11719600	2.11111500
	H	-3.56159000	-1.87966900	-1.31741300
	H	-4.74307900	-1.59038600	-0.03179200
	H	-2.72557100	2.79534800	-1.32790400
	H	-3.34058600	1.11489200	0.99750000
	H	-5.06456900	0.73694200	0.87852200
20	O	1.58004000	0.00321300	-0.83449900
	C	0.32262500	-0.07778600	-1.26995600
	C	2.65713500	-0.00687200	-1.79677400
	H	2.40034200	0.70320700	-2.59015700
	C	3.88347300	0.49398500	-1.03071000
	H	4.69908100	0.53502700	-1.76254600
	C	3.64091400	1.90436500	-0.49150600
	H	2.87620200	1.88517700	0.28961500
	C	2.81040600	-1.40356800	-2.37730900
	H	2.88488100	-2.13817200	-1.57300100
	H	3.71929200	-1.45457900	-2.98158300
	C	4.27872700	-0.45654500	0.10271700
	H	3.42677800	-0.63515500	0.76638600
	O	0.01906800	-0.11470000	-2.43036200
	C	-0.65579400	-0.06854300	-0.06617200
	C	0.02007100	-0.89039200	1.04237200
	C	0.36020900	-2.21864500	0.75827800
	C	1.03304800	-2.99906600	1.68509900
	C	1.38438000	-2.46272800	2.92337200
	C	1.05658400	-1.14657100	3.21257400
	C	0.38085200	-0.36195500	2.27646600
	C	-2.01643300	-0.64641700	-0.49166300
	C	-2.64574300	-1.68217700	0.19631300
	C	-3.91080600	-2.12663900	-0.18811000
	C	-4.56424800	-1.53986600	-1.26181400
	C	-3.95150400	-0.49028000	-1.94229600
	C	-2.69672600	-0.04399000	-1.55621200
	C	-0.88051300	1.39456200	0.35583700
	C	-1.90820700	1.67297300	1.26295600

	C	-2.14482400	2.96948400	1.69613100
	C	-1.36510900	4.02061000	1.21884700
	C	-0.35272500	3.75750900	0.30730500
	C	-0.11016400	2.45279200	-0.12024900
	H	0.09395600	-2.63944500	-0.20773400
	H	1.28733000	-4.02465500	1.44323600
	H	1.91256400	-3.06787200	3.65056700
	H	1.33118100	-0.71572600	4.16841100
	H	0.15271400	0.66898700	2.51648800
	H	-2.16246200	-2.14743400	1.04593100
	H	-4.38166400	-2.93327400	0.36183100
	H	-5.54570900	-1.88724300	-1.56225400
	H	-4.45580500	-0.01283600	-2.77427600
	H	-2.23775400	0.78371200	-2.08131900
	H	-2.52964300	0.86125600	1.62617000
	H	-2.94495600	3.16122100	2.40141800
	H	-1.55283200	5.03489200	1.55045000
	H	0.25575100	4.56624100	-0.08029500
	H	0.68825400	2.27993400	-0.83173400
	H	3.30728900	2.58260300	-1.28276100
	H	4.55708100	2.31373700	-0.06069500
	H	1.95748200	-1.65382400	-3.00749400
	H	5.08587100	-0.01938600	0.69465500
	H	4.62813200	-1.41992900	-0.27340200
22	O	-1.42402400	0.96372500	-0.56072000
	C	-0.47334800	0.18531000	-1.08593800
	C	-2.67192200	1.07534900	-1.28643300
	H	-2.43469900	1.11675600	-2.35259800
	C	-3.50877000	-0.18157000	-1.01572900
	H	-2.87014000	-1.02854800	-1.29129200
	C	-3.88625800	-0.32503500	0.45851800
	H	-3.01185700	-0.22401800	1.10626300
	C	-3.29078500	2.38156400	-0.82773300
	H	-4.25751600	2.53702100	-1.30967100
	H	-2.63918700	3.21678400	-1.08919500
	C	-4.74302300	-0.21476700	-1.91693700
	H	-4.47329000	-0.10409400	-2.97043100
	O	-0.53800500	-0.30817300	-2.17831400
	C	0.65080500	-0.06609300	-0.05510500
	C	-0.09800600	-0.84351200	1.04342000
	C	-0.62044000	-2.10000800	0.71727400
	C	-1.38301600	-2.81368800	1.63246700
	C	-1.64117200	-2.28045600	2.89260700
	C	-1.14148800	-1.02617700	3.21776300
	C	-0.38163900	-0.30711600	2.29684900
	C	1.28809200	1.24033000	0.43820800
	C	1.04898700	2.47720100	-0.15585700
	C	1.73045500	3.61504700	0.27281200
	C	2.65697900	3.53337300	1.30238900
	C	2.91131800	2.29903200	1.89580200
	C	2.24055300	1.16606200	1.45963800
	C	1.78824600	-0.87685000	-0.69314100

	C	2.38739900	-1.95642000	-0.04728600
	C	3.48943500	-2.59819100	-0.61001700
	C	4.00584300	-2.16882300	-1.82443900
	C	3.41987400	-1.08319400	-2.47063400
	C	2.32744300	-0.44184000	-1.90682700
	H	-0.42486600	-2.51714100	-0.26607000
	H	-1.77691200	-3.78594500	1.36021400
	H	-2.23447000	-2.83541500	3.60962000
	H	-1.34802000	-0.59352300	4.18986100
	H	-0.02431500	0.68065700	2.55780100
	H	0.32737900	2.57140300	-0.95763200
	H	1.53157400	4.56704200	-0.20546000
	H	3.18417400	4.41945600	1.63540900
	H	3.64215800	2.21671100	2.69172800
	H	2.46120600	0.20378500	1.90996900
	H	2.00119800	-2.30541000	0.90321400
	H	3.94162700	-3.43588700	-0.09185300
	H	4.86028000	-2.66978500	-2.26384800
	H	3.81695300	-0.73344600	-3.41639100
	H	1.88734800	0.40798500	-2.41435900
	H	-4.32480500	-1.30927300	0.63932200
	H	-4.62606200	0.42553300	0.75229800
	H	-3.43081100	2.38273100	0.25418100
	H	-5.44661500	0.58076600	-1.65652500
	H	-5.26747700	-1.16571200	-1.80230800
42	O	1.59316400	-0.27006900	-0.54421900
	C	0.41365600	-0.12026700	-1.15147400
	C	2.77809600	-0.37670200	-1.36468700
	H	2.64059700	0.28091900	-2.22731600
	C	3.91996000	0.15796300	-0.49752500
	H	3.61604900	1.17115200	-0.21049900
	C	4.11610400	-0.65724500	0.78093300
	H	4.51289500	-1.65196700	0.55951900
	C	2.92892900	-1.81305900	-1.83889700
	H	3.00484300	-2.49574600	-0.98994200
	H	3.82450800	-1.91908500	-2.45379200
	C	5.21662200	0.25076100	-1.30128300
	H	5.07311500	0.79565900	-2.23821100
	O	0.27111200	-0.10564100	-2.34314500
	C	-0.69716500	0.08940400	-0.09563400
	C	-2.05594400	0.33373100	-0.77079300
	C	-2.50082200	-0.54830700	-1.75984900
	C	-3.76171500	-0.40789300	-2.32194500
	C	-4.61272300	0.60828700	-1.89531600
	C	-4.19050300	1.47438500	-0.89684100
	C	-2.92111200	1.33773300	-0.33863400
	C	-0.20102800	1.31093200	0.69964600
	C	0.25323600	1.23133800	2.01276000
	C	0.76808600	2.35749200	2.65249700
	C	0.84696900	3.57205800	1.98487600
	C	0.41446300	3.65536700	0.66336200
	C	-0.10107700	2.53411800	0.02830200

	C	-0.86875200	-1.17024700	0.76788300
	C	-0.21294400	-2.37051600	0.50545500
	C	-0.46284100	-3.50201600	1.28097900
	C	-1.36834600	-3.44797200	2.33051100
	C	-2.03699100	-2.25445400	2.59459700
	C	-1.79496500	-1.13328900	1.81509400
	H	-1.85589300	-1.35192600	-2.09246000
	H	-4.08299300	-1.09696400	-3.09417000
	H	-5.59773300	0.71690000	-2.33398500
	H	-4.84613400	2.26233800	-0.54503100
	H	-2.61307200	2.02181200	0.44263700
	H	0.22224000	0.28785500	2.54251800
	H	1.11432600	2.27683400	3.67634500
	H	1.24719500	4.44635300	2.48437800
	H	0.47886200	4.59476100	0.12676000
	H	-0.43603900	2.60364100	-1.00227900
	H	0.50035000	-2.44297200	-0.30635700
	H	0.05613300	-4.42655800	1.05650500
	H	-1.56131400	-4.32729500	2.93343600
	H	-2.75568900	-2.20014300	3.40387100
	H	-2.33432000	-0.21300000	2.01336300
	H	3.17490900	-0.77101000	1.32251000
	H	4.83183400	-0.15859900	1.43846300
	H	2.06778700	-2.09452000	-2.44729600
	H	5.60461100	-0.74315200	-1.54052300
	H	5.98356100	0.77078500	-0.72362700
44	O	-1.65985000	0.41785100	-0.22911300
	C	-0.59152800	0.09371800	-0.96454600
	C	-2.92855900	0.56776300	-0.90778700
	H	-2.72050900	0.91745500	-1.92169600
	C	-3.61173100	-0.80246200	-0.99015700
	H	-2.86978900	-1.47659400	-1.43444100
	C	-4.00551200	-1.33811400	0.38725400
	H	-4.86614900	-0.79385800	0.78587700
	C	-3.68288000	1.62615300	-0.12499300
	H	-3.12897500	2.56577400	-0.14653900
	H	-3.80314600	1.32443800	0.91666900
	C	-4.81425600	-0.75662600	-1.93256100
	H	-4.52986100	-0.39805400	-2.92473500
	O	-0.62421300	-0.09439200	-2.14965700
	C	0.67277900	0.06956200	-0.07402900
	C	0.52453100	-0.99123700	1.02575800
	C	-0.42249300	-2.00930600	0.93795400
	C	-0.48011800	-3.01659700	1.89727900
	C	0.41512000	-3.02312800	2.95822300
	C	1.38043200	-2.02295200	3.04339200
	C	1.43896300	-1.02329500	2.08164100
	C	0.74196700	1.51829200	0.44242400
	C	0.37490900	1.88188400	1.73546000
	C	0.36137800	3.22173000	2.11658600
	C	0.69690700	4.21598400	1.20662600
	C	1.03715500	3.86332100	-0.09645100

	C	1.05528300	2.52730800	-0.47404300
	C	1.92084900	-0.33581200	-0.87347800
	C	1.87268500	-1.43813800	-1.73105700
	C	3.01557200	-1.88288100	-2.38140000
	C	4.23496500	-1.24389600	-2.17683700
	C	4.29899100	-0.16311000	-1.30789300
	C	3.15100400	0.28615900	-0.66035600
	H	-1.12815800	-2.03273900	0.11512600
	H	-1.22849300	-3.79572900	1.80846800
	H	0.36943300	-3.80413200	3.70778900
	H	2.09522200	-2.02554600	3.85792300
	H	2.20634000	-0.25889600	2.14565800
	H	0.08011800	1.12262800	2.44848000
	H	0.07809300	3.48505600	3.12908500
	H	0.68508000	5.25770100	1.50528700
	H	1.28580800	4.62949000	-0.82149400
	H	1.31394400	2.25978300	-1.49371300
	H	0.93421800	-1.95185600	-1.89533000
	H	2.95349300	-2.73388400	-3.04947000
	H	5.12668500	-1.59094100	-2.68534900
	H	5.24350400	0.33743300	-1.12911000
	H	3.22287000	1.13006200	0.01509200
	H	-3.18445800	-1.24797700	1.10266900
	H	-4.28725200	-2.39178300	0.31955200
	H	-4.66960100	1.79445200	-0.56063500
	H	-5.59840000	-0.10225900	-1.54202000
	H	-5.24599100	-1.75361300	-2.04504200
46	O	-1.63740000	0.23510000	-0.75050000
	C	-0.38700000	0.18140000	-1.20440000
	C	-2.69660000	0.46310000	-1.71040000
	H	-2.41060000	-0.04160000	-2.63680000
	C	-3.94600000	-0.21010000	-1.14180000
	H	-4.72280000	-0.07810000	-1.90510000
	C	-3.70140000	-1.70760000	-0.94680000
	H	-2.95070000	-1.87300000	-0.16870000
	C	-2.82000000	1.95650000	-1.95730000
	H	-1.92940000	2.32670000	-2.46690000
	H	-2.93280000	2.49370000	-1.01410000
	C	-4.42660000	0.43040000	0.16090000
	H	-4.69220000	1.48130000	0.03200000
	O	-0.09410000	0.33210000	-2.36030000
	C	0.64030000	-0.01910000	-0.05870000
	C	1.95940000	-0.52410000	-0.66220000
	C	3.19250000	-0.06180000	-0.20780000
	C	4.38170000	-0.58870000	-0.70900000
	C	4.35630000	-1.58410000	-1.67500000
	C	3.12930000	-2.05240000	-2.13820000
	C	1.94670000	-1.52700000	-1.63720000
	C	0.07970000	-1.01770000	0.97900000
	C	-0.99700000	-0.64450000	1.79590000
	C	-1.54130000	-1.52790000	2.71540000
	C	-1.01660000	-2.81220000	2.85100000

	C	0.06140000	-3.18770000	2.06480000
	C	0.60780000	-2.29710000	1.13950000
	C	0.84860000	1.37860000	0.56260000
	C	0.83670000	2.51700000	-0.24630000
	C	1.09360000	3.77400000	0.29070000
	C	1.38160000	3.91380000	1.64340000
	C	1.41640000	2.78370000	2.45270000
	C	1.15050000	1.52770000	1.91720000
	H	3.23970000	0.72010000	0.53970000
	H	5.32830000	-0.21040000	-0.34080000
	H	5.28080000	-1.99020000	-2.06850000
	H	3.09160000	-2.82650000	-2.89580000
	H	1.00170000	-1.90400000	-2.00990000
	H	-1.40660000	0.35390000	1.70950000
	H	-2.37660000	-1.21260000	3.33050000
	H	-1.43970000	-3.50440000	3.56950000
	H	0.49280000	-4.17660000	2.16760000
	H	1.45810000	-2.61520000	0.55140000
	H	0.64490000	2.42420000	-1.30950000
	H	1.07470000	4.64460000	-0.35450000
	H	1.58160000	4.89290000	2.06230000
	H	1.65120000	2.87580000	3.50670000
	H	1.18620000	0.65590000	2.55990000
	H	-3.34780000	-2.17630000	-1.86920000
	H	-4.62170000	-2.21100000	-0.64300000
	H	-3.68970000	2.16250000	-2.58580000
	H	-3.64850000	0.35970000	0.92530000
	H	-5.30980000	-0.09370000	0.53270000

6 (optimized at the B3LYP/6-311++G(d,p) level)

Conformer no				
1	C	-4.68569900	2.13362200	0.99457900
	C	-4.43744900	2.22194600	-0.51687600
	C	-3.18608400	1.43472900	-0.93018600
	C	-3.26356200	-0.03216800	-0.46781800
	C	-3.50757200	-0.11879400	1.04993000
	C	-4.75779500	0.67338900	1.46130200
	C	-2.02738500	-0.82141200	-0.93604500
	C	-2.15638400	-2.32136800	-0.66887600
	O	-0.86978300	-0.30383200	-0.26068300
	C	0.33297000	-0.60597400	-0.80352000
	O	0.44889900	-1.29032900	-1.78719500
	C	1.48596900	0.13475800	-0.07068400
	C	1.52232300	-0.16490700	1.44776200
	C	2.44686900	0.52757500	2.24398100
	C	2.58185800	0.24887000	3.59949300
	C	1.80439400	-0.74657100	4.19135000
	C	0.89928500	-1.45438900	3.40824700
	C	0.75689700	-1.16871800	2.04825600
	C	2.85326200	-0.33625100	-0.62400600
	C	3.12514500	-1.70690700	-0.72960500

	C	4.37670700	-2.15938200	-1.13280700
	C	5.39379400	-1.25200600	-1.42848700
	C	5.14244100	0.11067300	-1.31084200
	C	3.88358100	0.56300900	-0.91188200
	C	1.18305500	1.62015300	-0.40687400
	C	0.73380900	2.53826100	0.54548700
	C	0.41494100	3.84743000	0.18162800
	C	0.52420500	4.25857400	-1.14351500
	C	0.94742300	3.34436400	-2.10755700
	C	1.27001700	2.04059200	-1.74253700
	O	-1.58165800	-2.93722200	0.19273600
	O	-3.03381400	-2.86531000	-1.52915800
	C	-3.26103600	-4.28320700	-1.39325500
	H	-3.87017700	2.64225300	1.52446800
	H	-5.60771500	2.66171100	1.25903700
	H	-5.31094700	1.82483800	-1.05090300
	H	-4.33473400	3.26735800	-0.82515400
	H	-3.05955000	1.47360400	-2.01788600
	H	-2.29820200	1.90241200	-0.49182200
	H	-4.11341400	-0.50194900	-0.97975000
	H	-3.61002000	-1.16354600	1.36137600
	H	-2.63172400	0.27687800	1.57503900
	H	-5.64761400	0.19957600	1.02584900
	H	-4.88273900	0.62711800	2.54785000
	H	-1.89672900	-0.69361600	-2.01278200
	H	3.07777600	1.28705000	1.79807600
	H	3.30278400	0.80270900	4.19086000
	H	1.91141600	-0.97073800	5.24682500
	H	0.29474900	-2.23988400	3.84827600
	H	0.04123500	-1.74233800	1.47477600
	H	2.35501100	-2.43045300	-0.49646400
	H	4.55808400	-3.22545100	-1.21397200
	H	6.36970300	-1.60527100	-1.74232700
	H	5.92343200	0.83095700	-1.52883700
	H	3.71401300	1.62866000	-0.82883600
	H	0.62559900	2.23641100	1.57837600
	H	0.07678300	4.54365300	0.94139200
	H	0.27722000	5.27629700	-1.42438900
	H	1.02807800	3.64508400	-3.14634000
	H	1.59425400	1.34092300	-2.50432200
	H	-3.66437600	-4.51115300	-0.40590100
	H	-3.97909300	-4.53624900	-2.16971000
	H	-2.32811800	-4.82864000	-1.53827000
4	C	-5.69721500	1.25179000	0.87114300
	C	-4.40559600	1.28690800	1.69917000
	C	-3.38185600	0.25924500	1.19607100
	C	-3.08874300	0.45639000	-0.30172400
	C	-4.38431300	0.39753600	-1.13104000
	C	-5.40665900	1.42700700	-0.62598300
	C	-1.98957000	-0.48188100	-0.84144400
	C	-2.23041800	-1.96782700	-0.56518900
	O	-0.76289600	-0.07430500	-0.20846100

	C	0.38712200	-0.51771000	-0.76380000
	O	0.40929200	-1.25172500	-1.71824100
	C	1.62284200	0.13835000	-0.08749000
	C	2.92790900	-0.49303500	-0.62877000
	C	4.04448800	0.28413800	-0.94987300
	C	5.24854900	-0.30962200	-1.33134700
	C	5.35845200	-1.69450400	-1.39756300
	C	4.25464100	-2.48071500	-1.06856900
	C	3.05735700	-1.88719000	-0.68322000
	C	1.45630600	1.62703700	-0.49642900
	C	1.59296400	1.97759400	-1.84783800
	C	1.37525900	3.28424600	-2.27561500
	C	1.00812500	4.27017800	-1.36072200
	C	0.84976900	3.92961100	-0.02038600
	C	1.06436500	2.61876100	0.40646700
	C	1.65430600	-0.08573400	1.44383700
	C	2.63666400	0.57344500	2.19748500
	C	2.76597800	0.35176200	3.56413200
	C	1.92341500	-0.55246800	4.21062400
	C	0.95901000	-1.22775900	3.47061900
	C	0.82267000	-0.99851300	2.09951800
	O	-1.75365200	-2.59366800	0.34798000
	O	-3.07036300	-2.48539900	-1.47625400
	C	-3.38044100	-3.88662000	-1.32851200
	H	-6.20228900	0.28997600	1.03036800
	H	-6.38996600	2.02706900	1.21438400
	H	-3.96628900	2.29129400	1.64313300
	H	-4.62605900	1.10403900	2.75565000
	H	-2.45451100	0.33044500	1.76964500
	H	-3.77618900	-0.75146800	1.36222800
	H	-2.66173900	1.45975800	-0.42913300
	H	-4.16129400	0.57618400	-2.18850500
	H	-4.81889800	-0.60602600	-1.07156200
	H	-5.01859400	2.43840700	-0.80316600
	H	-6.33209100	1.34390700	-1.20490000
	H	-1.89066000	-0.35675100	-1.92144700
	H	3.98614100	1.36404100	-0.90550200
	H	6.09871200	0.31796400	-1.57555700
	H	6.29197500	-2.15766000	-1.69701900
	H	4.32521100	-3.56209100	-1.10925500
	H	2.21832600	-2.51896400	-0.42304100
	H	1.87184300	1.22098200	-2.57203400
	H	1.49301500	3.53070500	-3.32510500
	H	0.84240600	5.28951700	-1.69110300
	H	0.55391300	4.68313800	0.70134200
	H	0.91906700	2.37284500	1.44962100
	H	3.31640200	1.26197900	1.70991900
	H	3.53239300	0.87871200	4.12191400
	H	2.02525600	-0.73197100	5.27510300
	H	0.30222400	-1.94278300	3.95381300
	H	0.05663600	-1.54165100	1.56202200
	H	-3.85535200	-4.07255500	-0.36446000

	H	-4.06084500	-4.11900200	-2.14434400
	H	-2.47007100	-4.48178600	-1.40451700
7	C	4.83304100	-2.21544700	0.57159800
	C	4.52761300	-2.19225000	-0.93237100
	C	3.23782100	-1.41514000	-1.23431000
	C	3.29125100	0.01275000	-0.66005100
	C	3.59107100	-0.01603400	0.85063700
	C	4.87964000	-0.79549900	1.15023100
	C	2.02405300	0.81109800	-1.01720900
	C	2.12343700	2.27175300	-0.57043000
	O	0.89394800	0.19025000	-0.37974600
	C	-0.32738000	0.62606100	-0.76993500
	O	-0.46584500	1.44069000	-1.64497500
	C	-1.48942800	-0.09359200	-0.01213400
	C	-2.67203200	0.90563200	0.13553000
	C	-3.25185700	1.18737500	1.37598900
	C	-4.34055200	2.05573500	1.48345300
	C	-4.87397900	2.65832900	0.35038800
	C	-4.31413900	2.37541000	-0.89529300
	C	-3.23320300	1.50686000	-1.00257900
	C	-1.92267600	-1.29028300	-0.90576100
	C	-0.98686700	-2.02385100	-1.64479800
	C	-1.37516000	-3.12187700	-2.41033600
	C	-2.70893300	-3.52071400	-2.44205500
	C	-3.64779500	-2.80931700	-1.69909100
	C	-3.25911100	-1.70496800	-0.94272200
	C	-1.00305000	-0.58753700	1.37001400
	C	-0.32283300	0.29481700	2.22347900
	C	0.07981700	-0.10511500	3.49288200
	C	-0.18902300	-1.39872600	3.94304900
	C	-0.86128600	-2.28232100	3.10629900
	C	-1.26403600	-1.87935900	1.83056800
	O	1.59684300	2.74735600	0.40206800
	O	2.92377100	2.95184700	-1.40948000
	C	3.12114500	4.34746600	-1.10300600
	H	4.05503000	-2.79076100	1.08937600
	H	5.78012100	-2.73272800	0.75675100
	H	5.36593600	-1.72478800	-1.46534200
	H	4.44404300	-3.21272300	-1.31989800
	H	3.06720900	-1.37489500	-2.31627100
	H	2.38577100	-1.94512900	-0.79454500
	H	4.11020000	0.54389200	-1.16283300
	H	3.67409000	1.00243000	1.24268000
	H	2.74856100	-0.48070300	1.37338000
	H	5.73736000	-0.25929600	0.72274900
	H	5.04513900	-0.83201500	2.23164300
	H	1.87332600	0.80602900	-2.09894100
	H	-2.86391300	0.72978900	2.27499100
	H	-4.76677000	2.25451300	2.46067100
	H	-5.71762700	3.33467900	0.43250400
	H	-4.72204200	2.83040700	-1.79130900
	H	-2.82128100	1.29730400	-1.97887700

	H	0.05977600	-1.74956600	-1.62075600
	H	-0.62996800	-3.66581700	-2.98034000
	H	-3.01257700	-4.37413600	-3.03783100
	H	-4.69021900	-3.10816800	-1.70872900
	H	-4.00621100	-1.16237800	-0.37839500
	H	-0.09405200	1.30106000	1.89192200
	H	0.60567200	0.59632700	4.13132200
	H	0.12416600	-1.71079200	4.93325500
	H	-1.07757600	-3.29161800	3.43919700
	H	-1.78467300	-2.58660800	1.19878800
	H	3.59276600	4.45937700	-0.12590300
	H	3.77007300	4.72691600	-1.88878800
	H	2.16464000	4.87074100	-1.10562000
8	C	-5.62909600	1.75820400	0.21413600
	C	-5.68131000	0.25903600	0.53329100
	C	-4.57072100	-0.52102300	-0.18645000
	C	-3.17861100	0.05710300	0.13249300
	C	-3.13169200	1.56086300	-0.20022500
	C	-4.24233800	2.33857400	0.51894600
	C	-2.04495300	-0.69071500	-0.59266400
	C	-2.09846000	-2.20272500	-0.36511600
	O	-0.80964200	-0.17067300	-0.05738700
	C	0.33153900	-0.56984500	-0.66034300
	O	0.34064900	-1.34169700	-1.58515800
	C	1.56670500	0.16323700	-0.06587700
	C	1.27792400	1.64717700	-0.41696200
	C	0.95786200	2.60678200	0.54620500
	C	0.64136000	3.91434800	0.17455100
	C	0.62379600	4.28262100	-1.16739500
	C	0.91799800	3.32735300	-2.13978100
	C	1.23840800	2.02514400	-1.76745900
	C	1.72616600	-0.09404400	1.45304600
	C	0.95862700	-1.02500000	2.15883300
	C	1.20625100	-1.27315100	3.51109700
	C	2.21895800	-0.59764100	4.18264800
	C	2.99753500	0.32631400	3.48538100
	C	2.75823800	0.56590800	2.13683200
	C	2.86456000	-0.36681300	-0.72343200
	C	3.87861000	0.49118900	-1.15755800
	C	5.08303200	-0.01129700	-1.65332400
	C	5.29520400	-1.38374200	-1.72342300
	C	4.29518200	-2.24999600	-1.28186100
	C	3.09887600	-1.74739900	-0.78235200
	O	-1.67394800	-2.76544300	0.61424100
	O	-2.71828100	-2.81816500	-1.38190400
	C	-2.84703000	-4.25005300	-1.27212100
	H	-5.85988700	1.91054600	-0.84833000
	H	-6.39925700	2.29242200	0.77998200
	H	-5.57589400	0.11638100	1.61641100
	H	-6.65702700	-0.15514600	0.25968700
	H	-4.62494200	-1.57551000	0.09931300
	H	-4.74092600	-0.48337800	-1.27007600

	H	-2.99298400	-0.06963400	1.20682800
	H	-2.15336400	1.97004200	0.05889500
	H	-3.24951500	1.68373500	-1.28564800
	H	-4.06445800	2.30436300	1.60156400
	H	-4.19830700	3.39395100	0.23147200
	H	-2.07458900	-0.50367800	-1.66798600
	H	0.94750100	2.33879900	1.59391300
	H	0.40397600	4.64301900	0.94198500
	H	0.37841000	5.29901300	-1.45434000
	H	0.89917200	3.59470700	-3.19059100
	H	1.46251300	1.29366200	-2.53562000
	H	0.15726000	-1.57011600	1.67802300
	H	0.59718400	-2.00296000	4.03300900
	H	2.40723300	-0.79166700	5.23268200
	H	3.80003800	0.85440900	3.98866600
	H	3.39096100	1.26820800	1.60722200
	H	3.73895200	1.56338000	-1.11341600
	H	5.85252900	0.67789400	-1.98367400
	H	6.22827800	-1.77620900	-2.11205300
	H	4.44739100	-3.32290200	-1.32338400
	H	2.34267300	-2.43897200	-0.43522300
	H	-3.41855900	-4.51508300	-0.38157400
	H	-3.36995500	-4.56283700	-2.17275800
	H	-1.86034000	-4.71107700	-1.22000500
10	C	5.75599300	-1.42697600	0.41707500
	C	4.46654700	-1.68552500	1.20806200
	C	3.41081200	-0.60235400	0.94332500
	C	3.12134400	-0.46730500	-0.56198800
	C	4.41397100	-0.18721700	-1.35044200
	C	5.46840400	-1.27187600	-1.08282600
	C	1.99912200	0.53945100	-0.88731500
	C	2.22836500	1.94507400	-0.32296900
	O	0.78770500	-0.00391900	-0.32534800
	C	-0.36935800	0.59468200	-0.69210200
	O	-0.39522300	1.51656600	-1.46550000
	C	-1.61701300	-0.08569600	-0.04168000
	C	-2.75496200	0.96805900	0.03795800
	C	-3.39212600	1.28299400	1.24125200
	C	-4.43560300	2.21086600	1.28366100
	C	-4.86406700	2.84074800	0.12129700
	C	-4.24496600	2.52677000	-1.08871100
	C	-3.20911400	1.60014900	-1.13055400
	C	-2.04232200	-1.25326000	-0.97915100
	C	-1.09698900	-2.04783300	-1.64004700
	C	-1.49033500	-3.11818100	-2.44161000
	C	-2.83934800	-3.42964700	-2.58938800
	C	-3.78917700	-2.65904500	-1.92382300
	C	-3.39486400	-1.58248000	-1.13129500
	C	-1.24889300	-0.61705800	1.36352200
	C	-0.55214700	0.20991200	2.25707500
	C	-0.25108500	-0.22074400	3.54462300
	C	-0.64228200	-1.49023500	3.97212700

	C	-1.33347000	-2.31916700	3.09521700
	C	-1.63313100	-1.88599100	1.80155700
	O	1.80953800	2.35076300	0.73082400
	O	2.98908900	2.66688600	-1.16263600
	C	3.27690200	4.01874400	-0.74970900
	H	6.23007300	-0.50978600	0.79023100
	H	6.47337400	-2.23746900	0.58211000
	H	4.05858700	-2.66481300	0.92578400
	H	4.68163900	-1.73809000	2.27997800
	H	2.48779200	-0.82810600	1.48234100
	H	3.77282400	0.35757400	1.33212100
	H	2.72780900	-1.43112900	-0.91238800
	H	4.19596900	-0.12896100	-2.42237600
	H	4.81828100	0.78954100	-1.06384300
	H	5.11258200	-2.22984400	-1.48377900
	H	6.38942400	-1.03280500	-1.62419600
	H	1.88162300	0.63196700	-1.96876000
	H	-3.08436200	0.80599100	2.16122200
	H	-4.90927600	2.43454900	2.23322400
	H	-5.67234700	3.56296700	0.15296800
	H	-4.57039300	3.00382400	-2.00669800
	H	-2.75016100	1.36764800	-2.08079700
	H	-0.04127000	-1.84504600	-1.52159200
	H	-0.73598800	-3.71067800	-2.94766200
	H	-3.14585900	-4.26205300	-3.21277100
	H	-4.84425300	-2.89014500	-2.02105300
	H	-4.15098700	-0.99463700	-0.62798900
	H	-0.22577700	1.19514200	1.94503200
	H	0.29262700	0.43731400	4.21342200
	H	-0.40731200	-1.82722800	4.97570500
	H	-1.64255500	-3.30982200	3.41036400
	H	-2.16861900	-2.55132200	1.13724700
	H	3.82384600	4.02126300	0.19397800
	H	3.88340400	4.44133500	-1.54730500
	H	2.34919300	4.57928900	-0.63207800
14	C	-4.85736700	1.66609700	1.12226500
	C	-4.53589700	2.08129400	-0.31966500
	C	-3.24013100	1.42786100	-0.82167100
	C	-3.28984200	-0.10415600	-0.68371700
	C	-3.61086600	-0.51858000	0.76418200
	C	-4.90676100	0.13897900	1.26167400
	C	-2.00604700	-0.75356100	-1.22975600
	C	-2.15600300	-2.27083900	-1.33919600
	O	-0.89850900	-0.34242100	-0.40385400
	C	0.33881100	-0.46934700	-0.93413000
	O	0.53850400	-0.97500100	-2.00843600
	C	1.41590800	0.22239200	-0.04881100
	C	1.42562000	-0.28423800	1.41398700
	C	2.31522700	0.31248100	2.32027000
	C	2.42097200	-0.13828800	3.63126200
	C	1.64821700	-1.21428400	4.06819000
	C	0.77624800	-1.82670400	3.17542000

	C	0.66378200	-1.36745800	1.86080800
	C	2.82721700	-0.08127600	-0.60939400
	C	3.19281500	-1.40578400	-0.88435600
	C	4.48186600	-1.72245500	-1.29788000
	C	5.44401100	-0.72196900	-1.43499900
	C	5.09908400	0.59457100	-1.14991900
	C	3.80223700	0.91083600	-0.74103500
	C	1.03059300	1.72125300	-0.17770600
	C	0.51647000	2.46669600	0.88600200
	C	0.12695200	3.79475400	0.70562200
	C	0.22986600	4.39676500	-0.54484600
	C	0.71924500	3.65544800	-1.61983700
	C	1.11187700	2.33276300	-1.43789100
	O	-2.91035600	-2.78045200	-2.13003900
	O	-1.40315800	-2.95755000	-0.46550300
	C	-1.49041600	-4.39530400	-0.55776000
	H	-4.08661900	2.06567500	1.79395700
	H	-5.80809300	2.10626800	1.44040300
	H	-5.36796400	1.78898100	-0.97354600
	H	-4.45208600	3.17040900	-0.39243100
	H	-3.06493200	1.69924700	-1.86860400
	H	-2.38969800	1.81321300	-0.24944500
	H	-4.09049000	-0.47980700	-1.33334400
	H	-3.69923300	-1.60801300	0.83615200
	H	-2.77833000	-0.22666100	1.41354600
	H	-5.75501400	-0.24916300	0.68292700
	H	-5.08732500	-0.14240900	2.30415200
	H	-1.82664400	-0.40684500	-2.24995100
	H	2.94148100	1.13443000	1.99496800
	H	3.11514900	0.34595600	4.30924500
	H	1.73210100	-1.57149800	5.08841800
	H	0.17298300	-2.66918500	3.49605300
	H	-0.02905400	-1.86391900	1.19684200
	H	2.46552600	-2.20004300	-0.77702200
	H	4.73596100	-2.75455300	-1.51283700
	H	6.44942600	-0.96886900	-1.75710900
	H	5.83597600	1.38467100	-1.24430100
	H	3.55922200	1.94326100	-0.52563200
	H	0.41220000	2.01503600	1.86318100
	H	-0.26172600	4.35483800	1.54907800
	H	-0.07302800	5.42850600	-0.68377500
	H	0.79608200	4.10613700	-2.60316900
	H	1.48606800	1.76838500	-2.28432800
	H	-2.51461600	-4.72559800	-0.38203700
	H	-1.16707900	-4.72727600	-1.54463100
	H	-0.82422400	-4.77546900	0.21321700
17	C	-4.82892600	2.14782700	0.81213400
	C	-4.48842400	2.46443500	-0.65031500
	C	-3.20315300	1.75205000	-1.09634700
	C	-3.29099900	0.23273500	-0.86652900
	C	-3.63208100	-0.08351400	0.60129800
	C	-4.91491600	0.63402900	1.04693200

	C	-2.02423100	-0.48531900	-1.36469700
	C	-2.21872400	-2.00265400	-1.37301000
	O	-0.90556500	-0.05671900	-0.56025800
	C	0.32785900	-0.35010000	-1.02609700
	O	0.51165600	-0.94877700	-2.05535400
	C	1.42905000	0.09303100	-0.02277400
	C	1.33126100	-1.02776000	1.04773000
	C	2.01524700	-2.23475800	0.85594900
	C	1.87845500	-3.28720800	1.75852000
	C	1.04369500	-3.15929400	2.86664800
	C	0.33278500	-1.97536400	3.05003700
	C	0.46845100	-0.92522700	2.14463000
	C	2.83335000	0.16137700	-0.67441600
	C	3.02854100	0.44104900	-2.03134200
	C	4.31249400	0.61053500	-2.54926200
	C	5.42881900	0.51206600	-1.72456700
	C	5.24784600	0.24716900	-0.36860300
	C	3.96623000	0.07821900	0.14672500
	C	1.17355600	1.52343600	0.50782400
	C	1.60276400	1.92188700	1.77808900
	C	1.48211800	3.24637300	2.19548000
	C	0.93840800	4.20617100	1.34600300
	C	0.52431500	3.82710500	0.07078500
	C	0.64453700	2.50264700	-0.34107300
	O	-2.95860000	-2.54258400	-2.15930300
	O	-1.52975700	-2.63384600	-0.41840500
	C	-1.64762600	-4.07074300	-0.39514900
	H	-4.05354000	2.57166300	1.46329000
	H	-5.77110200	2.62917400	1.09388500
	H	-5.32090000	2.14844600	-1.29236600
	H	-4.38103400	3.54493100	-0.79045500
	H	-3.00876200	1.95487400	-2.15591500
	H	-2.35231500	2.15025000	-0.53331200
	H	-4.09810900	-0.16008300	-1.49791200
	H	-3.74414500	-1.16354200	0.74037600
	H	-2.79692900	0.22913400	1.23722000
	H	-5.76883800	0.22972500	0.48793600
	H	-5.10848400	0.42370700	2.10376200
	H	-1.83327100	-0.21092800	-2.40466100
	H	2.65991900	-2.35596700	-0.00524800
	H	2.42797100	-4.20738100	1.59261500
	H	0.94341300	-3.97432600	3.57492900
	H	-0.33434300	-1.86614800	3.89803300
	H	-0.10932300	-0.02311900	2.29351700
	H	2.18538800	0.51094700	-2.70235700
	H	4.43399800	0.81758900	-3.60682600
	H	6.42565400	0.64203500	-2.13070800
	H	6.10417300	0.17273700	0.29262300
	H	3.85143500	-0.12384300	1.20453900
	H	2.04087100	1.19836400	2.45335600
	H	1.82019600	3.52567800	3.18733800
	H	0.84544600	5.23667700	1.66950100

	H	0.10968300	4.56328400	-0.60902300
	H	0.32744600	2.23882500	-1.34347300
	H	-1.30340900	-4.48966000	-1.34127900
	H	-1.01252600	-4.39548400	0.42509900
	H	-2.68463500	-4.36334900	-0.22553700
18	C	5.67507500	-1.83756300	-0.16876400
	C	5.74281500	-0.36247400	0.24422400
	C	4.59908800	0.45863300	-0.36969200
	C	3.22165600	-0.14108300	-0.02567700
	C	3.15931400	-1.62170800	-0.44665600
	C	4.30492400	-2.43979900	0.16587400
	C	2.06641500	0.65879200	-0.65362400
	C	2.10979300	2.13872800	-0.26169900
	O	0.84381200	0.07200800	-0.15437500
	C	-0.31316700	0.59131000	-0.62083000
	O	-0.33427100	1.47458300	-1.43899300
	C	-1.56706300	-0.11485000	-0.01143700
	C	-1.24201500	-0.60604300	1.41870500
	C	-0.64024900	0.27346300	2.33154500
	C	-0.37131100	-0.12209300	3.63692200
	C	-0.69936300	-1.40977300	4.06373100
	C	-1.29451500	-2.29109800	3.16821400
	C	-1.56280200	-1.89225900	1.85643500
	C	-2.74077000	0.90336400	-0.00727500
	C	-3.16777800	1.48231800	-1.21288900
	C	-4.23337600	2.37499500	-1.24174200
	C	-4.91052900	2.70571800	-0.06778300
	C	-4.50943100	2.12690800	1.13036300
	C	-3.43577400	1.23360500	1.15932400
	C	-1.91966300	-1.30817900	-0.94363400
	C	-3.24998300	-1.72460900	-1.08011300
	C	-3.58248000	-2.82303400	-1.87008000
	C	-2.59182100	-3.52783600	-2.55002500
	C	-1.26502200	-3.12726000	-2.42054900
	C	-0.93314700	-2.03451100	-1.62058800
	O	1.73097600	2.57821800	0.79497400
	O	2.67022000	2.87428200	-1.23296900
	C	2.77319700	4.28882900	-0.97299900
	H	5.85346000	-1.92027200	-1.24892400
	H	6.47122700	-2.40575000	0.32298700
	H	5.68984400	-0.29043400	1.33810600
	H	6.70400700	0.07171800	-0.04887900
	H	4.66647000	1.49268000	-0.02006700
	H	4.71829600	0.49200900	-1.46041600
	H	3.08140100	-0.08321600	1.06116200
	H	2.19817500	-2.04916000	-0.15382400
	H	3.21869500	-1.68089400	-1.54263700
	H	4.17926700	-2.47420700	1.25547900
	H	4.24646600	-3.47517400	-0.18487700
	H	2.07965800	0.58808600	-1.74352200
	H	-0.36382200	1.27437600	2.02025800
	H	0.09885100	0.57663800	4.32004900

	H	-0.48920700	-1.71990200	5.08136900
	H	-1.55294500	-3.29639000	3.48264600
	H	-2.02275900	-2.59871000	1.17825400
	H	-2.66318600	1.23549000	-2.13608400
	H	-4.53697600	2.81182600	-2.18684200
	H	-5.74239700	3.40095100	-0.09138700
	H	-5.02846500	2.36363100	2.05267700
	H	-3.15063700	0.79507500	2.10536700
	H	-4.03632800	-1.18598500	-0.56733900
	H	-4.62114200	-3.12278500	-1.95580800
	H	-2.85079500	-4.37714700	-3.17215600
	H	-0.48000100	-3.66557100	-2.94020900
	H	0.10868700	-1.76087800	-1.52188700
	H	3.38387600	4.47137900	-0.08768100
	H	3.24204500	4.71179000	-1.85830900
	H	1.78021400	4.71314300	-0.82227300
20	C	4.99333800	-1.76162700	0.77396400
	C	4.64079500	-2.08409300	-0.68430600
	C	3.30807800	-1.44160900	-1.09689200
	C	3.31462600	0.07828700	-0.85315300
	C	3.66759000	0.39943900	0.61078400
	C	4.99846800	-0.24754000	1.02202600
	C	1.99810700	0.72736800	-1.31658100
	C	2.09644300	2.25334800	-1.29521300
	O	0.92441200	0.21814100	-0.49864500
	C	-0.33283000	0.45707400	-0.93081900
	O	-0.55750800	1.04780500	-1.95581300
	C	-1.42036000	-0.17965100	-0.00361400
	C	-1.86927000	-1.48578400	-0.71629300
	C	-3.19598000	-1.92372800	-0.64905600
	C	-3.58574600	-3.12299800	-1.24417700
	C	-2.65775000	-3.90693500	-1.92454700
	C	-1.33240800	-3.48378000	-1.99539100
	C	-0.94344200	-2.28983100	-1.39237100
	C	-0.83003900	-0.48861300	1.39163100
	C	-0.95491900	-1.74149800	1.99420800
	C	-0.44951400	-1.97689900	3.27577600
	C	0.18926000	-0.96147900	3.97746700
	C	0.32030300	0.29658100	3.38628300
	C	-0.18410000	0.52775100	2.11220900
	C	-2.62505500	0.80051300	0.10987700
	C	-3.18201500	1.13455500	1.34922800
	C	-4.29460900	1.97347400	1.43972900
	C	-4.87759500	2.49489100	0.29052200
	C	-4.34432400	2.15597000	-0.95234300
	C	-3.23981900	1.31444100	-1.04340900
	O	2.78778400	2.85576600	-2.07871100
	O	1.38157600	2.82839500	-0.31799000
	C	1.40742200	4.27095000	-0.28919600
	H	4.25755300	-2.23435500	1.43712300
	H	5.96718400	-2.19102200	1.03078300
	H	5.43968500	-1.71474600	-1.34026600

	H	4.59167100	-3.16749200	-0.83374900
	H	3.10226800	-1.64635500	-2.15383800
	H	2.49515500	-1.89332400	-0.51745500
	H	4.08421400	0.52123100	-1.49811600
	H	3.72364000	1.48315500	0.75723600
	H	2.86533600	0.03485700	1.26086700
	H	5.81666100	0.20961800	0.45032900
	H	5.20122900	-0.03656200	2.07691000
	H	1.80247700	0.46379200	-2.35864700
	H	-3.93510000	-1.32668400	-0.13111000
	H	-4.62102100	-3.43884400	-1.17756600
	H	-2.96296900	-4.83492300	-2.39468900
	H	-0.59615800	-4.08258900	-2.52002400
	H	0.09698400	-1.99381200	-1.44578500
	H	-1.44868100	-2.54915500	1.47047100
	H	-0.56106500	-2.96004800	3.71973400
	H	0.58144900	-1.14334200	4.97189900
	H	0.81769400	1.09939300	3.91961100
	H	-0.06758000	1.50929100	1.66850500
	H	-2.75725500	0.73958700	2.26122700
	H	-4.70131400	2.21265500	2.41624400
	H	-5.73969900	3.14910000	0.35891000
	H	-4.79250700	2.54369700	-1.86067200
	H	-2.85039000	1.05942300	-2.01751800
	H	0.77718300	4.55506100	0.55019400
	H	2.42732800	4.62994400	-0.14758600
	H	1.00798600	4.67013500	-1.22180400
21	C	-5.83770200	0.86021500	0.82372700
	C	-4.61836800	0.61777000	1.72379200
	C	-3.54339700	-0.21388800	1.00880800
	C	-3.13045700	0.43484500	-0.32428200
	C	-4.35389600	0.66023200	-1.23128100
	C	-5.42654200	1.49264400	-0.51297100
	C	-1.99582200	-0.31626800	-1.05013200
	C	-2.30290500	-1.79342900	-1.29182900
	O	-0.79886200	-0.10804600	-0.27097200
	C	0.38027700	-0.32119400	-0.89550100
	O	0.45646800	-0.77509900	-2.00824900
	C	1.57259000	0.20970700	-0.04879700
	C	2.91185300	-0.17362500	-0.72348400
	C	3.96629100	0.73567900	-0.84464900
	C	5.20108800	0.33995800	-1.36156900
	C	5.40419900	-0.97471600	-1.76674200
	C	4.36278700	-1.89384300	-1.64106700
	C	3.13551500	-1.49865500	-1.12038400
	C	1.31223900	1.74040000	-0.05339400
	C	1.38804600	2.43544700	-1.26959800
	C	1.09196400	3.79370600	-1.33903400
	C	0.70658500	4.48727300	-0.19242300
	C	0.60973000	3.80365200	1.01602500
	C	0.90247700	2.44094400	1.08360200
	C	1.62489500	-0.40017200	1.37258900

	C	2.60933100	0.05802000	2.26092100
	C	2.75371800	-0.49759600	3.52726700
	C	1.92405800	-1.54240000	3.93429700
	C	0.95560300	-2.01762900	3.05735100
	C	0.80515800	-1.45307100	1.78848600
	O	-3.07775700	-2.16246100	-2.13900900
	O	-1.65617200	-2.62044000	-0.45345200
	C	-1.89222800	-4.02901700	-0.65964400
	H	-6.33995500	-0.09661800	0.63087800
	H	-6.56630400	1.49711500	1.33583700
	H	-4.18887500	1.58308500	2.02145300
	H	-4.92094400	0.11561900	2.64835900
	H	-2.66752700	-0.33828600	1.65123000
	H	-3.94216800	-1.21840800	0.81536800
	H	-2.69675600	1.41781400	-0.09972700
	H	-4.04474100	1.16289900	-2.15411600
	H	-4.77470700	-0.30477300	-1.53174100
	H	-5.03917900	2.50338100	-0.32985400
	H	-6.30004400	1.60777000	-1.16252500
	H	-1.84421100	0.11661800	-2.04097600
	H	3.83469200	1.76515100	-0.53755700
	H	6.00157200	1.06688000	-1.44535300
	H	6.36125100	-1.28256800	-2.17275800
	H	4.50585200	-2.92361700	-1.94935000
	H	2.34515400	-2.23167500	-1.02544800
	H	1.68122900	1.90888600	-2.17052000
	H	1.16247000	4.30918800	-2.29048000
	H	0.47877800	5.54608700	-0.24337300
	H	0.30035600	4.32706100	1.91409600
	H	0.80303600	1.92600200	2.02948400
	H	3.27905100	0.85346400	1.95667200
	H	3.52185500	-0.11971100	4.19300900
	H	2.03770400	-1.98145300	4.91908100
	H	0.30604300	-2.83363800	3.35512500
	H	0.03533000	-1.84246100	1.13705200
	H	-1.29146400	-4.53639200	0.09168900
	H	-2.94982100	-4.26055800	-0.52930900
	H	-1.57960900	-4.31844300	-1.66329600
28	C	4.99823200	-1.78193200	0.76737200
	C	4.64375600	-2.07971200	-0.69579200
	C	3.31628900	-1.42121600	-1.09950700
	C	3.33309800	0.09416000	-0.83011600
	C	3.68578800	0.38906200	0.63909100
	C	5.01298900	-0.27191800	1.03980400
	C	2.02096500	0.75665000	-1.28409100
	C	2.12205400	2.28187200	-1.23908600
	O	0.94271600	0.24751100	-0.47271300
	C	-0.30692400	0.40262000	-0.95572200
	O	-0.52426700	0.90547300	-2.02997300
	C	-1.40980500	-0.19754100	-0.02667600
	C	-2.02477700	-1.36593000	-0.84410000
	C	-3.40185800	-1.59120400	-0.89530200

	C	-3.92345800	-2.67413400	-1.60514100
	C	-3.07768700	-3.54827300	-2.28033200
	C	-1.70062000	-3.33350300	-2.23721600
	C	-1.18320700	-2.25637100	-1.52465600
	C	-0.80356900	-0.70155600	1.30564700
	C	-0.93555500	-2.02165500	1.73889100
	C	-0.41600600	-2.43039700	2.97097900
	C	0.24265000	-1.52357600	3.79217800
	C	0.37670200	-0.19814800	3.37258300
	C	-0.14185000	0.20502400	2.14860200
	C	-2.49488000	0.88355800	0.26984600
	C	-2.91513500	1.80856800	-0.69640500
	C	-3.93236000	2.72087600	-0.42066800
	C	-4.56946200	2.72302700	0.81766700
	C	-4.17796000	1.79542900	1.77840200
	C	-3.15386300	0.88894600	1.50710500
	O	2.86632200	2.89231000	-1.96625300
	O	1.33308000	2.84702800	-0.31523500
	C	1.36142200	4.28871400	-0.26225500
	H	4.25958100	-2.26050900	1.42303500
	H	5.96944000	-2.22133200	1.01726500
	H	5.44565700	-1.70642000	-1.34583800
	H	4.58695500	-3.16038400	-0.86146000
	H	3.11083800	-1.60627800	-2.16011600
	H	2.49823100	-1.87637500	-0.52981800
	H	4.10556700	0.54416000	-1.46638000
	H	3.74732900	1.47022400	0.80321100
	H	2.88157100	0.01642700	1.28207700
	H	5.83367500	0.18954500	0.47523300
	H	5.21747200	-0.07943100	2.09790400
	H	1.82291000	0.50722900	-2.32904000
	H	-4.07969200	-0.92048600	-0.38452700
	H	-4.99686000	-2.82608400	-1.63093500
	H	-3.48406100	-4.38528700	-2.83680700
	H	-1.02744900	-4.00422300	-2.75955500
	H	-0.10839200	-2.11547300	-1.49456400
	H	-1.45127300	-2.74630500	1.12331700
	H	-0.53592800	-3.46211100	3.28295900
	H	0.64425000	-1.83899900	4.74873000
	H	0.88491300	0.52329500	4.00315600
	H	-0.03061200	1.23795400	1.84171300
	H	-2.45078400	1.81443200	-1.67111100
	H	-4.22951900	3.42885800	-1.18673500
	H	-5.36200600	3.43242100	1.02841100
	H	-4.66655100	1.77136500	2.74635700
	H	-2.87350400	0.17904800	2.27325400
	H	0.65902900	4.56279500	0.52120400
	H	2.36589900	4.63976000	-0.02331400
	H	1.05067900	4.70496800	-1.22083700
46	C	-5.75298200	1.64981400	0.00519100
	C	-5.80753600	0.11754200	0.03794100
	C	-4.62421400	-0.51657700	-0.70804300

	C	-3.27507000	-0.01192400	-0.15968500	
	C	-3.22455600	1.52859700	-0.20075300	
	C	-4.41040600	2.16188200	0.54002900	
	C	-2.07279700	-0.61311000	-0.91485200	
	C	-2.11686800	-2.14080400	-0.97426100	
	O	-0.88525700	-0.12601000	-0.24596500	
	C	0.30246700	-0.45289000	-0.79783100	
	O	0.38220800	-1.11548400	-1.80020500	
	C	1.51314200	0.16891000	-0.02695400	
	C	1.13943500	0.41946800	1.45280400	
	C	0.53345500	-0.60073400	2.20120500	
	C	0.23338300	-0.42336400	3.54689200	
	C	0.53515400	0.78208400	4.18243200	
	C	1.13812600	1.80052800	3.45321000	
	C	1.43686400	1.61988400	2.10042400	
	C	2.71936400	-0.80503000	-0.13965400	
	C	3.18759700	-1.20511000	-1.40170600	
	C	4.28528500	-2.05039000	-1.52430100	
	C	4.95521200	-2.50976400	-0.39052200	
	C	4.51513400	-2.10594200	0.86451200	
	C	3.40952300	-1.26119900	0.98770900	
	C	1.84525200	1.50892800	-0.74379800	
	C	3.16735300	1.95712600	-0.84398500	
	C	3.46629200	3.18626300	-1.42994500	
	C	2.44948600	3.99221900	-1.93530100	
	C	1.12939600	3.55912000	-1.83997400	
	C	0.83153900	2.33466700	-1.24509500	
	O	-2.65999100	-2.74763800	-1.86180800	
	O	-1.54488900	-2.71738000	0.09697200	
	C	-1.52592000	-4.15999800	0.09312100	
	H	-5.88773000	1.99495000	-1.02820700	
	H	-6.58014200	2.07189700	0.58510200	
	H	-5.79898800	-0.22154200	1.08209200	
	H	-6.74658400	-0.24013600	-0.39609500	
	H	-4.68740600	-1.60514500	-0.64109400	
	H	-4.69274000	-0.27308400	-1.77596300	
	H	-3.18270700	-0.33067700	0.88665300	
	H	-2.28579600	1.88137200	0.23074800	
	H	-3.24004300	1.85191900	-1.25114100	
	H	-4.33225700	1.92839500	1.60952600	
	H	-4.35571500	3.25214600	0.45708500	
	H	-2.05956900	-0.27668400	-1.95417300	
	H	0.28328100	-1.54158500	1.72569900	
	H	-0.23896500	-1.22729700	4.10090800	
	H	0.30043300	0.92212500	5.23172200	
	H	1.37991500	2.74379200	3.93055400	
	H	1.90620900	2.42815700	1.55571300	
	H	2.69017800	-0.85593300	-2.29451400	
	H	4.61991200	-2.34764400	-2.51212300	
	H	5.81218500	-3.16706000	-0.48771200	
	H	5.02924600	-2.44192200	1.75836900	
	H	3.09706400	-0.95803000	1.97713300	

	H 3.97393000 1.34485800 -0.46261200<br>H 4.49969700 3.50892800 -1.49276700<br>H 2.68247900 4.94466800 -2.39781900<br>H 0.32462000 4.17446800 -2.22679900<br>H -0.20483200 2.03392900 -1.16421200<br>H -1.01889600 -4.44589600 1.01185200<br>H -2.54259000 -4.55393100 0.07451600<br>H -0.97942200 -4.52411600 -0.77725900

6 (optimized at the M06-2X/6-311++G(d,p) level)

Conformer no	
1	C -4.22105000 2.26854900 1.02424900<br>C -3.96308000 2.42340200 -0.47511500<br>C -2.83427500 1.49833800 -0.92996400<br>C -3.12885300 0.03582600 -0.57577800<br>C -3.40876400 -0.11912800 0.92350000<br>C -4.53556800 0.81270500 1.37396000<br>C -1.96576000 -0.85120000 -1.02753800<br>C -2.21257200 -2.32599900 -0.75272000<br>O -0.81471700 -0.43004700 -0.31239800<br>C 0.38659400 -0.66205600 -0.86825600<br>O 0.53165200 -1.29123200 -1.87641200<br>C 1.47967000 0.09009200 -0.07772700<br>C 1.59928900 -0.44371900 1.35814500<br>C 2.53237000 0.16027600 2.20763300<br>C 2.71725600 -0.29929500 3.50310400<br>C 1.98353300 -1.38876800 3.96890300<br>C 1.07178700 -2.00612400 3.12544100<br>C 0.87540700 -1.53765300 1.82603800<br>C 2.84855300 -0.11353300 -0.74212700<br>C 3.27916400 -1.41375900 -1.02115600<br>C 4.54356400 -1.64589000 -1.54215800<br>C 5.41052900 -0.58254100 -1.78175600<br>C 5.00063300 0.70999900 -1.48720600<br>C 3.72742700 0.94317400 -0.97022100<br>C 0.99208400 1.55155700 -0.14026800<br>C 0.51711900 2.24283400 0.97104000<br>C -0.00272800 3.52984700 0.83454900<br>C -0.06728700 4.13530300 -0.41362200<br>C 0.38893900 3.44339300 -1.53300700<br>C 0.91068000 2.16387300 -1.39560000<br>O -1.57546400 -3.00932000 -0.00026500<br>O -3.25963900 -2.76308900 -1.45805200<br>C -3.58744000 -4.14442100 -1.27756900<br>H -3.32592100 2.58484300 1.57424400<br>H -5.04000600 2.92027400 1.34053500<br>H -4.88075900 2.18442100 -1.02686300<br>H -3.70799900 3.45976200 -0.71348600<br>H -2.67007500 1.59416200 -2.00859800<br>H -1.90468800 1.79997100 -0.43477500<br>H -4.01058400 -0.29640100 -1.13746100

	H	-3.66310000	-1.15911800	1.15661300
	H	-2.49244100	0.11677400	1.47709300
	H	-5.46919300	0.51839600	0.87839100
	H	-4.69979200	0.70442700	2.44925900
	H	-1.79358000	-0.73626300	-2.10189500
	H	3.12169200	0.99620100	1.84477600
	H	3.44188700	0.18549300	4.14685300
	H	2.13071400	-1.75485300	4.97814600
	H	0.50172500	-2.86142900	3.46912500
	H	0.14933300	-2.03875200	1.19698400
	H	2.61867800	-2.25006700	-0.82623200
	H	4.85464300	-2.66093500	-1.75951600
	H	6.39842000	-0.76333400	-2.18893600
	H	5.66915100	1.54579500	-1.65770700
	H	3.42789400	1.95904800	-0.74264600
	H	0.53576800	1.77710800	1.94820900
	H	-0.36476900	4.05324800	1.71206500
	H	-0.47491800	5.13405500	-0.51735300
	H	0.33456600	3.89880300	-2.51500500
	H	1.25978900	1.62686600	-2.27269500
	H	-3.84103100	-4.33728700	-0.23488800
	H	-4.44130600	-4.33184700	-1.92267200
	H	-2.74208000	-4.77034900	-1.56317200
7	C	4.55955900	-2.25366000	0.65842500
	C	4.32468800	-2.28821800	-0.85269200
	C	3.08613900	-1.47323600	-1.23004200
	C	3.20551400	-0.02649400	-0.73854100
	C	3.44445000	0.00937500	0.77640500
	C	4.67743000	-0.81284000	1.15742400
	C	1.96686200	0.77904900	-1.13994700
	C	2.09109900	2.23482100	-0.71136600
	O	0.85472700	0.19179400	-0.48166300
	C	-0.36462000	0.64935400	-0.81865300
	O	-0.53096100	1.46232200	-1.68199200
	C	-1.47134200	-0.06581100	-0.00517500
	C	-2.68137100	0.87843800	0.12485200
	C	-3.22764600	1.22193200	1.35943100
	C	-4.35593600	2.03822600	1.43688500
	C	-4.95425900	2.51899600	0.28192100
	C	-4.42458800	2.16742700	-0.95746600
	C	-3.30596700	1.35111600	-1.03549300
	C	-1.88315900	-1.31808300	-0.80684300
	C	-0.98107900	-1.99476500	-1.62864300
	C	-1.35765700	-3.15677900	-2.29580600
	C	-2.64007700	-3.66852500	-2.14350600
	C	-3.54456400	-3.00586000	-1.32032400
	C	-3.17129400	-1.83971300	-0.66244300
	C	-0.89920500	-0.45026600	1.36742500
	C	-0.25263700	0.53860600	2.11886100
	C	0.26930900	0.25177500	3.37020000
	C	0.15575100	-1.03431300	3.89838500
	C	-0.48087000	-2.02021100	3.15949600

	C	-1.00572500	-1.73103400	1.89839900
	O	1.52616100	2.73514000	0.21992100
	O	2.96194000	2.87460300	-1.49652600
	C	3.17648800	4.25236800	-1.17440800
	H	3.71645000	-2.73996500	1.16373700
	H	5.45796300	-2.82076200	0.91637200
	H	5.20196600	-1.87434400	-1.36508400
	H	4.21216000	-3.31890500	-1.19897200
	H	2.93218900	-1.48770600	-2.31456700
	H	2.20294900	-1.92741400	-0.76589900
	H	4.05662300	0.44604700	-1.24524800
	H	3.55949800	1.04242400	1.12028600
	H	2.55769700	-0.39286500	1.28134900
	H	5.57083200	-0.35230300	0.71732200
	H	4.81357200	-0.79536100	2.24174200
	H	1.81808500	0.75299900	-2.22357100
	H	-2.78570400	0.85109800	2.27491900
	H	-4.76282700	2.29170100	2.40880500
	H	-5.82947400	3.15542200	0.34112700
	H	-4.88747100	2.52748800	-1.86885300
	H	-2.91362400	1.07199800	-2.00456900
	H	0.03003200	-1.62605000	-1.75136800
	H	-0.64234600	-3.66016700	-2.93568000
	H	-2.93341400	-4.57268500	-2.66355700
	H	-4.54846700	-3.39354600	-1.19228600
	H	-3.88817500	-1.32888600	-0.03021700
	H	-0.13951700	1.53735300	1.70761500
	H	0.77193700	1.03100400	3.93143500
	H	0.56411300	-1.26097200	4.87636100
	H	-0.57343400	-3.02414200	3.55734300
	H	-1.49264200	-2.51765600	1.33513700
	H	3.58410600	4.34453000	-0.16743600
	H	3.88450100	4.62122700	-1.91137900
	H	2.23573700	4.79952000	-1.23333200
11	C	-4.82999900	1.91439900	-0.62839500
	C	-5.05544600	0.39996500	-0.66716100
	C	-4.54580700	-0.26298300	0.61614900
	C	-3.06919400	0.05615700	0.89067200
	C	-2.85475900	1.57926700	0.91603600
	C	-3.36244200	2.25876900	-0.35872600
	C	-2.12440000	-0.59578000	-0.12742300
	C	-2.28370800	-2.10633200	-0.18137600
	O	-0.80716200	-0.27971000	0.30909800
	C	0.18450200	-0.52623200	-0.56233700
	O	0.00983100	-1.11348000	-1.59248400
	C	1.49744400	0.11639600	-0.06789200
	C	2.04128600	-0.64027500	1.15512800
	C	3.28941200	-0.25220700	1.65406000
	C	3.85312900	-0.89735900	2.74400300
	C	3.18477000	-1.96139800	3.34673500
	C	1.95499400	-2.36415800	2.84705500
	C	1.37927700	-1.70766900	1.75848300

	C	2.56525400	0.03219900	-1.16902700
	C	2.88140300	-1.21931300	-1.70656100
	C	3.89183000	-1.35266700	-2.64696800
	C	4.61974700	-0.23843800	-3.05887600
	C	4.32743100	1.00424700	-2.51639200
	C	3.30552400	1.13948700	-1.57721100
	C	1.09305800	1.56688500	0.25582600
	C	1.16105200	2.11512900	1.53202100
	C	0.69544500	3.40860400	1.77052300
	C	0.14659700	4.16172900	0.74212200
	C	0.06456700	3.61532200	-0.53763600
	C	0.53418800	2.33194800	-0.77494700
	O	-1.72740400	-2.87619600	0.55286200
	O	-3.14967900	-2.46441300	-1.13152500
	C	-3.38711900	-3.87036900	-1.24738200
	H	-5.16351500	2.37125900	-1.56402300
	H	-5.44644400	2.34323100	0.17163400
	H	-6.11905400	0.18184500	-0.79518500
	H	-4.54498300	-0.03224200	-1.53496400
	H	-5.12403900	0.11824700	1.46532400
	H	-4.71437100	-1.34261200	0.58343900
	H	-2.78680100	-0.35871300	1.86431800
	H	-3.41031200	1.97643400	1.77359300
	H	-1.79985200	1.81047100	1.08312600
	H	-3.23620300	3.34116000	-0.26514700
	H	-2.74674500	1.95799300	-1.21523700
	H	-2.26876300	-0.20480400	-1.13615100
	H	3.82371000	0.56156100	1.17463400
	H	4.81917800	-0.57763900	3.11678500
	H	3.62639200	-2.47359200	4.19341600
	H	1.42892500	-3.19627300	3.30024000
	H	0.41297400	-2.04523500	1.40128900
	H	2.33284200	-2.09496900	-1.38109700
	H	4.11588400	-2.33057700	-3.05661700
	H	5.41138300	-0.34403400	-3.79134400
	H	4.89397600	1.87806900	-2.81684800
	H	3.09975800	2.11761800	-1.16006300
	H	1.56023300	1.53304200	2.35268100
	H	0.75676900	3.81920700	2.77169400
	H	-0.21959700	5.16369600	0.93254600
	H	-0.36945100	4.18829800	-1.34897800
	H	0.46741800	1.90949100	-1.77417900
	H	-3.78862500	-4.26389800	-0.31296800
	H	-4.10685600	-3.98261100	-2.05349700
	H	-2.45673600	-4.38598600	-1.48505500
14	C	-4.49037700	1.76522400	1.10481800
	C	-4.20661600	2.16872100	-0.34285600
	C	-2.99302900	1.41663400	-0.88983100
	C	-3.17277300	-0.10076100	-0.77035500
	C	-3.48114800	-0.50574100	0.67611500
	C	-4.69472700	0.25340200	1.21637000
	C	-1.92824500	-0.81656400	-1.29865600

	C	-2.13582400	-2.32220400	-1.33780400
	O	-0.84316600	-0.43020100	-0.46289800
	C	0.39908400	-0.55257700	-0.95780400
	O	0.63830100	-1.10597100	-1.99241300
	C	1.40292700	0.20123000	-0.05415000
	C	1.52042100	-0.45930700	1.32995300
	C	2.46366900	0.05987800	2.22404200
	C	2.62971100	-0.49140900	3.48591600
	C	1.86426200	-1.58857200	3.87614000
	C	0.93782800	-2.11929500	2.99093700
	C	0.76197500	-1.55890700	1.72518500
	C	2.80002400	0.17139800	-0.69011900
	C	3.35514200	-1.06313600	-1.03935700
	C	4.64376400	-1.14471000	-1.54529300
	C	5.41156800	0.00717100	-1.69946600
	C	4.87764000	1.23488500	-1.33629100
	C	3.57961500	1.31668300	-0.83415600
	C	0.80703500	1.62062400	0.02034400
	C	0.30107800	2.16946900	1.19515100
	C	-0.31305000	3.42147900	1.18383100
	C	-0.43869000	4.13402800	-0.00125900
	C	0.05379800	3.58609500	-1.18380100
	C	0.66788300	2.34143300	-1.17115800
	O	-2.95070600	-2.83909200	-2.05124100
	O	-1.35208700	-2.99039700	-0.48839600
	C	-1.50434300	-4.41327400	-0.50707200
	H	-3.63848900	2.06058300	1.73010900
	H	-5.36764200	2.29629000	1.48397600
	H	-5.08647800	1.94498100	-0.95893600
	H	-4.03373700	3.24628800	-0.41226800
	H	-2.81226200	1.68737200	-1.93558900
	H	-2.10224200	1.71184000	-0.32371300
	H	-4.00398000	-0.41777100	-1.41224400
	H	-3.65534200	-1.58587500	0.73573900
	H	-2.60496100	-0.28412700	1.29722500
	H	-5.58541900	-0.03223900	0.64333000
	H	-4.88041300	-0.03193800	2.25516900
	H	-1.71946700	-0.51066500	-2.32818400
	H	3.07493700	0.90419200	1.92278600
	H	3.36324500	-0.07047600	4.16363700
	H	1.99624600	-2.02523200	4.85914100
	H	0.34001600	-2.97659000	3.27905500
	H	0.02417700	-1.98980900	1.06109400
	H	2.77179000	-1.96710100	-0.91201300
	H	5.05198300	-2.11080200	-1.81775900
	H	6.41905500	-0.05705100	-2.09318900
	H	5.46718500	2.13850900	-1.43842800
	H	3.18466600	2.28446900	-0.55079900
	H	0.36680200	1.61828300	2.12462500
	H	-0.70117700	3.83226500	2.10890700
	H	-0.92026100	5.10469900	-0.00826800
	H	-0.04447600	4.12664200	-2.11798900

	H	1.04570300	1.91689200	-2.09702800
	H	-2.52512900	-4.68563700	-0.23801600
	H	-1.27861200	-4.79937500	-1.50095500
	H	-0.79607600	-4.79471200	0.22392200
17	C	4.65800000	-2.08632500	0.83871000
	C	4.35877300	-2.47359800	-0.61049700
	C	3.09661500	-1.77082700	-1.11416500
	C	3.21453100	-0.24938000	-0.97433800
	C	3.53129600	0.14330400	0.47382200
	C	4.78774400	-0.56900200	0.97855300
	C	1.94602900	0.43637300	-1.48442500
	C	2.09257800	1.95051100	-1.41288100
	O	0.85882800	-0.03378900	-0.69275000
	C	-0.37512900	0.31682500	-1.08945800
	O	-0.58935500	0.93831100	-2.09186600
	C	-1.41219400	-0.09238000	-0.02828700
	C	-1.18007300	1.00699800	1.02886800
	C	-1.82994500	2.23669600	0.90766500
	C	-1.55619500	3.27356200	1.79325000
	C	-0.61111400	3.10411600	2.79970900
	C	0.07017400	1.89574000	2.90136000
	C	-0.20859900	0.85803000	2.01943200
	C	-2.84586400	-0.10642300	-0.57569400
	C	-3.12795100	-0.42749800	-1.90314200
	C	-4.44444600	-0.55472700	-2.33640300
	C	-5.49935900	-0.37237500	-1.45141200
	C	-5.22772500	-0.06830800	-0.12151400
	C	-3.91359500	0.05942500	0.30995400
	C	-1.15317700	-1.52062400	0.47276600
	C	-1.52684500	-1.91310900	1.75760700
	C	-1.39092100	-3.23630700	2.16399600
	C	-0.88824800	-4.19187000	1.28808300
	C	-0.53208900	-3.81422000	-0.00227200
	C	-0.66884100	-2.49135600	-0.40473500
	O	2.82226200	2.55847100	-2.14787100
	O	1.38047300	2.49453800	-0.42982200
	C	1.47558700	3.91701500	-0.31440000
	H	3.84104700	-2.43909500	1.48010000
	H	5.57099500	-2.57923000	1.18324200
	H	5.21016100	-2.19162500	-1.24196400
	H	4.24226800	-3.55696600	-0.69998500
	H	2.90269300	-2.03258200	-2.16001000
	H	2.23394500	-2.11013800	-0.52890900
	H	4.02733000	0.10606300	-1.62028300
	H	3.65798900	1.22808900	0.55196800
	H	2.67471800	-0.12365400	1.10368000
	H	5.65317100	-0.22806300	0.39714600
	H	4.97840000	-0.29601400	2.01980700
	H	1.76993300	0.18912600	-2.53482600
	H	-2.55561800	2.38416200	0.11630300
	H	-2.08110500	4.21650100	1.69162500
	H	-0.40110600	3.90982800	3.49371600

	H	0.82354100	1.75806600	3.66841700
	H	0.34104200	-0.07221700	2.09542700
	H	-2.32502400	-0.56990400	-2.61309800
	H	-4.64157000	-0.79586200	-3.37447000
	H	-6.52306900	-0.46955500	-1.79276100
	H	-6.03853200	0.06850000	0.58430900
	H	-3.71666200	0.29346400	1.35023200
	H	-1.92542800	-1.18064900	2.45045800
	H	-1.68298600	-3.51985600	3.16838000
	H	-0.78162200	-5.22215500	1.60566000
	H	-0.14812600	-4.54961000	-0.69987400
	H	-0.39249400	-2.21702700	-1.41805200
	H	1.12046500	4.38782800	-1.23150000
	H	0.84308000	4.18032500	0.52862800
	H	2.51078700	4.21041600	-0.13580700
28	C	4.95274300	-1.68393600	0.68382600
	C	4.63931800	-2.03840400	-0.77067300
	C	3.29809900	-1.44097200	-1.20163200
	C	3.28111400	0.07830500	-1.00053000
	C	3.60549300	0.43573300	0.45446400
	C	4.94252400	-0.16822900	0.88747300
	C	1.94341800	0.66888300	-1.44865200
	C	1.94272100	2.18325300	-1.28369500
	O	0.92036200	0.06109100	-0.66501400
	C	-0.34227700	0.29616800	-1.03842500
	O	-0.61999800	0.89258800	-2.04300600
	C	-1.38163100	-0.24051400	-0.02555300
	C	-2.59546700	-0.75432200	-0.81478500
	C	-3.88851200	-0.62542600	-0.31233200
	C	-4.97319000	-1.17780100	-0.98920200
	C	-4.78095700	-1.86420200	-2.18076300
	C	-3.49306300	-1.99728700	-2.69155300
	C	-2.41281300	-1.44820900	-2.01349000
	C	-0.75999000	-1.36274800	0.82861000
	C	-1.16942700	-2.68944000	0.72687200
	C	-0.55517900	-3.68670900	1.48717800
	C	0.47217000	-3.37131700	2.36205000
	C	0.87856800	-2.04278200	2.48559800
	C	0.26668500	-1.05461800	1.73146200
	C	-1.80326700	0.96944900	0.84544500
	C	-2.01277900	2.22300700	0.26317900
	C	-2.47323300	3.29161000	1.02415100
	C	-2.74458800	3.12912700	2.37848400
	C	-2.56086300	1.88131400	2.96135200
	C	-2.09562900	0.81172200	2.20141000
	O	2.62029400	2.90716300	-1.96018900
	O	1.15563000	2.59283900	-0.28927700
	C	1.09531300	4.00700200	-0.08692400
	H	4.19483400	-2.13919200	1.33353600
	H	5.91989200	-2.10053100	0.97767000
	H	5.43558600	-1.64877500	-1.41669700
	H	4.62313300	-3.12303800	-0.90474400

	H	3.09116000	-1.67914500	-2.25020500
	H	2.49396900	-1.88412300	-0.60160700
	H	4.03931900	0.53293900	-1.65085700
	H	3.62503900	1.52296700	0.58354100
	H	2.80545900	0.04596800	1.09385500
	H	5.74983200	0.28105200	0.29623900
	H	5.14145100	0.07833700	1.93384100
	H	1.76580000	0.46574000	-2.50777400
	H	-4.05961200	-0.08961700	0.61321600
	H	-5.97060000	-1.06358000	-0.58096200
	H	-5.62540200	-2.28915700	-2.71028300
	H	-3.32807000	-2.52578900	-3.62302600
	H	-1.41687800	-1.56448900	-2.42605700
	H	-1.97403200	-2.96317800	0.05676900
	H	-0.89177200	-4.71200300	1.38817000
	H	0.94917600	-4.14664300	2.95004300
	H	1.67431000	-1.77732800	3.17266800
	H	0.58683600	-0.02440000	1.83560400
	H	-1.82722100	2.36554100	-0.79399200
	H	-2.62775500	4.25457000	0.55016700
	H	-3.10335600	3.96291900	2.97040500
	H	-2.78192700	1.73147000	4.01171600
	H	-1.97012700	-0.15507000	2.67305800
	H	0.37858300	4.15432600	0.71695200
	H	2.07911600	4.38856200	0.18886000
	H	0.76111900	4.50027600	-1.00029300
30	C	-5.46389600	1.80433400	0.06818700
	C	-5.60549800	0.31350400	0.37742800
	C	-4.49271000	-0.50469500	-0.28172000
	C	-3.11009200	0.01021600	0.13228300
	C	-2.97041500	1.50753300	-0.17331400
	C	-4.08574200	2.31645500	0.48937600
	C	-1.98297100	-0.75960600	-0.55597100
	C	-2.06360200	-2.25869000	-0.31049100
	O	-0.76919700	-0.26194400	-0.00356500
	C	0.36295600	-0.63288800	-0.62338700
	O	0.37833900	-1.43337500	-1.51538400
	C	1.56136600	0.16192100	-0.06390300
	C	2.80923400	-0.10444700	-0.92007200
	C	3.60519100	0.92306700	-1.42079900
	C	4.77866200	0.63770200	-2.11799400
	C	5.16900300	-0.67742400	-2.32333500
	C	4.38565800	-1.71046900	-1.81393900
	C	3.22340900	-1.42613800	-1.11300100
	C	1.09242600	1.62724400	-0.13117200
	C	0.74165600	2.14581400	-1.38291200
	C	0.22845100	3.42915500	-1.50591900
	C	0.05304300	4.22116600	-0.37307000
	C	0.39046900	3.71175800	0.87306500
	C	0.90253500	2.41977200	0.99582300
	C	1.88870700	-0.29908400	1.36601800
	C	3.00746500	0.26321700	1.99036300

	C	3.37935800	-0.12397900	3.26868400
	C	2.64735400	-1.09966000	3.94271300
	C	1.54746100	-1.67489400	3.32362200
	C	1.16305100	-1.27676000	2.04251300
	O	-1.62087000	-2.81068800	0.65999400
	O	-2.72213000	-2.87540300	-1.29130300
	C	-2.87538900	-4.28873500	-1.13220200
	H	-5.59292800	1.96253800	-1.00965200
	H	-6.25309900	2.37092800	0.56964700
	H	-5.56224400	0.16598700	1.46337800
	H	-6.58016200	-0.05469800	0.04665900
	H	-4.60431600	-1.56073500	-0.01973100
	H	-4.58657600	-0.43965200	-1.37370900
	H	-2.98409300	-0.14402800	1.21245200
	H	-1.98878800	1.86549000	0.14800800
	H	-3.02106900	1.64588000	-1.26244600
	H	-3.98814400	2.23728600	1.57909500
	H	-3.97452600	3.37469000	0.23850600
	H	-1.99196100	-0.58416900	-1.63586600
	H	3.32334400	1.95704500	-1.26518800
	H	5.38498300	1.45211100	-2.49691100
	H	6.07890500	-0.89990200	-2.86816900
	H	4.68495900	-2.74190600	-1.95804700
	H	2.63199700	-2.23642600	-0.70446600
	H	0.87387300	1.53042700	-2.26867500
	H	-0.03764200	3.81044200	-2.48486600
	H	-0.34805700	5.22365200	-0.46475400
	H	0.24914600	4.31417900	1.76291600
	H	1.13537600	2.03178200	1.97913700
	H	3.59429500	1.00691000	1.46111200
	H	4.24715100	0.32710900	3.73543200
	H	2.94055400	-1.41115900	4.93827400
	H	0.97520800	-2.44222000	3.83174900
	H	0.29252800	-1.74308800	1.59523300
	H	-1.89651700	-4.76488200	-1.07638900
	H	-3.43473700	-4.50817800	-0.22199800
	H	-3.41942800	-4.62646900	-2.00991000
32	C	2.80631700	-2.63501300	0.97475800
	C	2.11794800	-2.69432600	-0.38988600
	C	2.90574500	-1.92128500	-1.45792500
	C	3.31101800	-0.49041800	-1.05384900
	C	3.92008400	-0.47116200	0.35823600
	C	3.05612300	-1.18724000	1.39836300
	C	2.16583900	0.51279500	-1.26075700
	C	2.54121600	1.89181100	-0.73476500
	O	1.00513500	0.06485200	-0.57743200
	C	-0.13854800	0.72746100	-0.83172000
	O	-0.20770200	1.62134700	-1.62523400
	C	-1.29892800	0.12590300	-0.00866800
	C	-1.80733300	-1.13455600	-0.73328900
	C	-2.99838700	-1.71166400	-0.27987400
	C	-3.50569300	-2.85926900	-0.86986100

	C	-2.83851500	-3.44817400	-1.94199100
	C	-1.66527000	-2.87559600	-2.41095800
	C	-1.15052100	-1.72707000	-1.80942800
	C	-0.73030100	-0.19710600	1.38253200
	C	-0.71641300	-1.47740100	1.92665000
	C	-0.16248600	-1.70477900	3.18776200
	C	0.38852500	-0.65828200	3.91233400
	C	0.39795700	0.62353000	3.36410700
	C	-0.15199600	0.84884400	2.11151200
	C	-2.46782100	1.12651400	0.04953000
	C	-3.05726700	1.52189700	1.24824100
	C	-4.16250200	2.37235500	1.25082900
	C	-4.69356300	2.83611500	0.05638700
	C	-4.12090100	2.43223000	-1.14736200
	C	-3.02684900	1.58001900	-1.15040700
	O	2.09141000	2.40051700	0.25261700
	O	3.48397000	2.44252800	-1.50475400
	C	3.94034800	3.73160800	-1.08271600
	H	2.19172100	-3.14464500	1.72205700
	H	3.76500500	-3.16866800	0.92753200
	H	2.01205600	-3.73344500	-0.71583400
	H	1.10999700	-2.28655500	-0.29237100
	H	3.83148900	-2.47073400	-1.66543700
	H	2.34535400	-1.89511200	-2.39962300
	H	4.08142900	-0.14855700	-1.75296900
	H	4.89096500	-0.97822900	0.30048200
	H	4.12793000	0.55707500	0.67140400
	H	3.55554900	-1.15678600	2.37100000
	H	2.10117600	-0.66769900	1.51357900
	H	1.94593900	0.60607000	-2.32893400
	H	-3.53012800	-1.24839500	0.54442700
	H	-4.42804200	-3.29030300	-0.49842500
	H	-3.23659800	-4.34085000	-2.40945800
	H	-1.14113100	-3.31829600	-3.24996600
	H	-0.22762800	-1.30839900	-2.19473900
	H	-1.12790700	-2.31226900	1.37321600
	H	-0.16213200	-2.70931900	3.59501700
	H	0.82025000	-0.83667800	4.89032100
	H	0.84683500	1.44693700	3.90705700
	H	-0.11404100	1.84486300	1.68096600
	H	-2.66812100	1.16457800	2.19293600
	H	-4.60590600	2.66566300	2.19524400
	H	-5.55178200	3.49778100	0.05880000
	H	-4.53239100	2.77766800	-2.08860800
	H	-2.60193800	1.25739000	-2.09254400
	H	3.10606400	4.43230100	-1.05420700
	H	4.38840900	3.66462800	-0.09077500
	H	4.67811500	4.03956900	-1.81843000

10 (optimized at the B3LYP/6-311++G(d,p) level)

Conformer no	

1	O	0.93507500	-0.22417200	0.00732300
	C	-0.12408900	-0.38253400	-0.80487100
	C	2.27068900	-0.45161400	-0.55032200
	H	2.23322900	-0.17105400	-1.60460500
	C	4.04176000	-2.24886100	-0.89348200
	H	4.11286300	-1.98987900	-1.95946900
	C	3.25224300	0.44210200	0.21949400
	H	3.18060600	0.14195200	1.27449700
	C	4.68571600	0.12589600	-0.25689300
	H	4.80001300	0.42815100	-1.30462500
	H	5.40091900	0.72260700	0.31563300
	C	2.60965700	-1.93845700	-0.42871800
	H	1.89044900	-2.52060300	-1.01219200
	H	2.50078100	-2.23546600	0.62228900
	C	5.03527300	-1.36189000	-0.12986000
	H	5.03257400	-1.65216400	0.92957000
	H	6.05206300	-1.53820600	-0.49786100
	C	2.88403300	1.94974300	0.16246400
	H	1.82578900	2.01246400	0.43217000
	C	4.36460200	-3.73909100	-0.74735900
	H	4.30568400	-4.05133000	0.30091700
	H	5.37481500	-3.96063400	-1.10411200
	H	3.66526000	-4.35619400	-1.31917900
	C	3.04231200	2.58632300	-1.22759000
	H	4.09140300	2.64298600	-1.53346700
	H	2.65139500	3.60747400	-1.21621500
	H	2.49513400	2.04101700	-2.00122000
	C	3.66032300	2.75766500	1.21394400
	H	3.30181400	3.79056700	1.24344000
	H	4.73232300	2.79238900	0.99606500
	H	3.53427600	2.33422100	2.21519100
	O	-0.03586900	-0.69507700	-1.96672100
	C	-1.45441000	-0.01822800	-0.07452400
	C	-1.67852600	-0.86307300	1.20281300
	C	-2.77302300	-0.56184400	2.02689000
	C	-3.07254100	-1.33604400	3.14243800
	C	-2.29205500	-2.44857700	3.45586300
	C	-1.21607100	-2.77218700	2.63680400
	C	-0.91266200	-1.98717600	1.52278800
	C	-2.67047600	-0.32135800	-0.98549700
	C	-2.77263900	-1.56400100	-1.62542600
	C	-3.89967700	-1.89871500	-2.36823800
	C	-4.96291800	-1.00333500	-2.48013200
	C	-4.88338700	0.22612900	-1.83515700
	C	-3.74871200	0.56195300	-1.09496800
	C	-1.28360100	1.49719000	0.20593400
	C	-1.06721800	2.01377900	1.48593400
	C	-0.86141100	3.38034400	1.68029700
	C	-0.85355800	4.25412000	0.59704100
	C	-1.04009100	3.74696700	-0.68839600
	C	-1.24862400	2.38436500	-0.88035400
	H	-3.40763400	0.28293200	1.78810100

	H -3.92333300 -1.07489200 3.76212700<br>H -2.52595100 -3.05700300 4.32216100<br>H -0.60376600 -3.63936700 2.85878500<br>H -0.06531800 -2.26555000 0.91190400<br>H -1.96534200 -2.27980900 -1.54860500<br>H -3.94702700 -2.86393600 -2.86032100<br>H -5.84161900 -1.26443900 -3.05921700<br>H -5.70339500 0.93267100 -1.90338700<br>H -3.71499300 1.52551000 -0.60338100<br>H -1.05050200 1.35189000 2.34114200<br>H -0.70243400 3.75723600 2.68473400<br>H -0.69696800 5.31610500 0.74990100<br>H -1.02400000 4.41182500 -1.54496400<br>H -1.38826200 2.00496100 -1.88610800

10 (optimized at the M06-2X/6-311++G(d,p) level)

Conformer no	
1	O 0.90263700 -0.34292700 0.01234900<br>C -0.14528400 -0.45409400 -0.80855500<br>C 2.22381600 -0.52055500 -0.53963900<br>H 2.18477600 -0.25678900 -1.59990500<br>C 4.06749000 -2.19078800 -0.90994200<br>H 4.08878100 -1.92788200 -1.97658600<br>C 3.15182000 0.43001500 0.21099900<br>H 3.12520800 0.12744700 1.26824400<br>C 4.58306200 0.21632600 -0.29990500<br>H 4.64736900 0.51582400 -1.35284000<br>H 5.27086700 0.85925800 0.25469500<br>C 2.63860200 -1.97791300 -0.40391300<br>H 1.93712600 -2.60601700 -0.96277100<br>H 2.57909500 -2.26434600 0.65459000<br>C 5.01947900 -1.24508400 -0.17366400<br>H 5.04586000 -1.52879400 0.88691500<br>H 6.03597500 -1.36921700 -0.55982700<br>C 2.66121100 1.89255800 0.14944500<br>H 1.65265000 1.89671500 0.57451300<br>C 4.49436000 -3.64791800 -0.76192400<br>H 4.48734200 -3.94290800 0.29204700<br>H 5.50536300 -3.80212400 -1.14689300<br>H 3.81968700 -4.31534900 -1.30359600<br>C 2.56569600 2.44869900 -1.27346700<br>H 3.54035500 2.45088500 -1.77042800<br>H 2.20901600 3.48118200 -1.24113300<br>H 1.86412700 1.88459700 -1.89423900<br>C 3.52396800 2.80947400 1.01813500<br>H 3.04542700 3.78656500 1.12377000<br>H 4.51188000 2.97201500 0.57731300<br>H 3.66310300 2.39312600 2.02005200<br>O -0.06239700 -0.77322800 -1.96269700<br>C -1.44074300 -0.03737500 -0.07313200<br>C -1.75158300 -0.99035200 1.09149700

	C	-2.87535200	-0.71343600	1.87709000
	C	-3.25035500	-1.55667200	2.91150300
	C	-2.51566800	-2.71142800	3.17230100
	C	-1.41027000	-3.00612800	2.38749800
	C	-1.02789900	-2.15061600	1.35425600
	C	-2.64853800	-0.11349700	-1.02002300
	C	-2.87901100	-1.29314100	-1.73400800
	C	-4.01396200	-1.43965600	-2.51768200
	C	-4.95234900	-0.41338700	-2.59416300
	C	-4.74283400	0.75439000	-1.87482200
	C	-3.59916000	0.90281600	-1.09238500
	C	-1.12935300	1.39827700	0.38976500
	C	-0.91426400	1.73503400	1.72388700
	C	-0.53447100	3.02915400	2.07503400
	C	-0.35754400	4.00094400	1.09790800
	C	-0.55721900	3.66899200	-0.23945900
	C	-0.93394200	2.37881900	-0.58875900
	H	-3.46348300	0.17359800	1.66603800
	H	-4.12288600	-1.31866700	3.50863000
	H	-2.80939100	-3.37625100	3.97576800
	H	-0.83584500	-3.90600600	2.57420600
	H	-0.15529400	-2.40231800	0.76340800
	H	-2.16497600	-2.10510400	-1.67186700
	H	-4.16946100	-2.36078100	-3.06688100
	H	-5.84029000	-0.52833600	-3.20454100
	H	-5.46878900	1.55807700	-1.91602300
	H	-3.45926400	1.82008900	-0.53388500
	H	-1.02279800	0.98491700	2.49671800
	H	-0.36903500	3.27073600	3.11859100
	H	-0.06168000	5.00638500	1.37371000
	H	-0.41364900	4.41407800	-1.01362600
	H	-1.07731500	2.12387000	-1.63464400

11 (optimized at the B3LYP/6-311++G(d,p) level)

Conformer no				
1	O	1.02377600	-0.22135000	-0.46333300
	C	-0.15565400	-0.03160000	-1.07899500
	C	2.23841600	-0.16240600	-1.29341500
	H	1.99081900	-0.61841300	-2.25050600
	C	3.07848300	2.03031000	-0.25291300
	H	2.21558800	2.08408700	0.42068800
	C	3.33674600	-0.96026000	-0.57088100
	H	4.19331700	-0.93733000	-1.26272600
	C	3.76298500	-0.22924400	0.71823300
	H	2.93209200	-0.25345300	1.43265300
	H	4.59643100	-0.75596200	1.18788600
	C	2.63439500	1.29809400	-1.52796100
	H	1.80509900	1.82259200	-2.01014100
	H	3.46030000	1.29586800	-2.25206700
	C	4.17696100	1.22501600	0.45442300
	H	5.08503500	1.23734800	-0.16534000

	H	4.44333900	1.71104900	1.39964100
	C	2.98206800	-2.45364700	-0.34523000
	H	2.14905600	-2.49021100	0.36555400
	C	3.52753600	3.46119400	-0.56402400
	H	4.38943800	3.46537200	-1.24083800
	H	3.81878300	3.99044000	0.34831600
	H	2.72653500	4.03417900	-1.04035000
	C	4.15699100	-3.23455400	0.26804900
	H	5.04593800	-3.17189500	-0.37023500
	H	3.89917200	-4.29269300	0.36848700
	H	4.43033700	-2.87263500	1.26065400
	C	2.53813400	-3.15408300	-1.64064600
	H	1.62633000	-2.72942400	-2.06648600
	H	2.34531400	-4.21431500	-1.45339600
	H	3.31987500	-3.09469000	-2.40664000
	O	-0.27296100	0.10687000	-2.27192300
	C	-1.32396100	0.08951100	-0.05017500
	C	-0.98487700	1.42446200	0.66223300
	C	-0.43824600	1.48003400	1.94740400
	C	-0.08341200	2.70094800	2.52183200
	C	-0.25145700	3.88880600	1.81548000
	C	-0.76692900	3.84383700	0.52111800
	C	-1.12467400	2.62518500	-0.04907500
	C	-1.40991700	-1.12858400	0.89702800
	C	-0.80692500	-2.35241600	0.59501000
	C	-0.99762800	-3.47067200	1.40732100
	C	-1.79829100	-3.38811200	2.54177000
	C	-2.41850900	-2.17738100	2.84791300
	C	-2.23198100	-1.06683400	2.03095300
	C	-2.69427600	0.13137000	-0.77249800
	C	-3.72445600	0.96758000	-0.32972100
	C	-4.99197600	0.91980000	-0.91044900
	C	-5.25629300	0.03021200	-1.94681600
	C	-4.24229600	-0.81848000	-2.38813400
	C	-2.98036100	-0.77261700	-1.80380900
	H	-0.27969200	0.56855900	2.50777500
	H	0.33015500	2.71783600	3.52429300
	H	0.02266400	4.83718400	2.26377400
	H	-0.89181400	4.75817000	-0.04844200
	H	-1.51867000	2.60628000	-1.05841000
	H	-0.18073000	-2.45038000	-0.28151000
	H	-0.51713900	-4.40720800	1.14631900
	H	-1.94555400	-4.25582600	3.17475800
	H	-3.05779400	-2.09783600	3.72027900
	H	-2.74304600	-0.14349000	2.27487600
	H	-3.54560400	1.66968600	0.47431800
	H	-5.77020400	1.58242400	-0.54779900
	H	-6.23932300	-0.00568500	-2.40286300
	H	-4.43234800	-1.52132100	-3.19183400
	H	-2.21380500	-1.44527200	-2.16277800
22	O	1.01933700	0.21221800	-0.48621300
	C	-0.16301400	0.01614300	-1.09431000

	C	2.23336500	0.24855700	-1.31639100
	H	1.98115300	-0.21490300	-2.26717000
	C	3.06305500	2.44444300	-0.28087100
	H	2.20004100	2.48628700	0.39427300
	C	3.33254800	-0.54705700	-0.59035600
	H	4.19083000	-0.52769100	-1.28058000
	C	3.75801500	0.18942700	0.69545100
	H	2.92946900	0.16450300	1.41243300
	H	4.59505800	-0.33198700	1.16450200
	C	2.62540600	1.70810300	-1.55614300
	H	1.79453000	2.22918200	-2.04378200
	H	3.45270300	1.71088100	-2.27820100
	C	4.16635200	1.64455600	0.42586800
	H	5.07244700	1.65896000	-0.19657700
	H	4.43291500	2.13528400	1.36853400
	C	2.96729500	-2.03859800	-0.36744600
	H	2.09654700	-2.07339100	0.29637400
	C	3.50070000	3.87982200	-0.58782100
	H	3.78560300	4.40998600	0.32588900
	H	2.69806200	4.44901600	-1.06732500
	H	4.36407800	3.89243900	-1.26228500
	C	4.10801400	-2.81305300	0.31393600
	H	3.84659500	-3.87132500	0.40100200
	H	4.32129300	-2.44872200	1.32049600
	H	5.03311200	-2.74943600	-0.27109600
	C	2.59263800	-2.74694400	-1.68057500
	H	1.70278500	-2.32647600	-2.15263500
	H	2.38902200	-3.80506900	-1.49452300
	H	3.41533200	-2.69310900	-2.40336700
	O	-0.29155000	-0.13057800	-2.28488200
	C	-1.32062700	-0.07567800	-0.04930900
	C	-1.43811300	1.21833300	0.79005000
	C	-0.83641500	2.41894800	0.40407700
	C	-1.05032600	3.59689900	1.12097800
	C	-1.87398700	3.59840700	2.24156600
	C	-2.49292700	2.41085500	2.63057100
	C	-2.28302400	1.24035800	1.90873500
	C	-0.94033900	-1.33117100	0.77680100
	C	-0.42303700	-1.25971200	2.07304600
	C	-0.03489600	-2.41424200	2.75350400
	C	-0.14201200	-3.66167800	2.14515300
	C	-0.62897700	-3.74355700	0.84138700
	C	-1.01871000	-2.59092700	0.16525400
	C	-2.69314000	-0.22206500	-0.75507300
	C	-3.02886200	0.61149700	-1.83039900
	C	-4.29390900	0.56417900	-2.40736200
	C	-5.26324400	-0.30833800	-1.91475100
	C	-4.95096200	-1.12660700	-0.83429300
	C	-3.67966300	-1.08180100	-0.26101200
	H	-0.19418400	2.45372900	-0.46576100
	H	-0.57018600	4.51337000	0.79599400
	H	-2.04015600	4.51260300	2.80005400

	H	-3.14954400	2.39613400	3.49350500
	H	-2.79347100	0.33525400	2.21459700
	H	-0.31227000	-0.30000200	2.55912300
	H	0.35647400	-2.33225900	3.76164000
	H	0.15734400	-4.55845500	2.67597700
	H	-0.70598900	-4.70578800	0.34728700
	H	-1.39148600	-2.67138500	-0.84922000
	H	-2.29848800	1.30172400	-2.22897600
	H	-4.52173200	1.21352300	-3.24551500
	H	-6.24883500	-0.34485600	-2.36520000
	H	-5.69410300	-1.80557200	-0.43055200
	H	-3.46447200	-1.73014600	0.57825200
27	O	1.07405000	-0.04957400	-0.46271400
	C	-0.13244800	0.00942700	-1.04003200
	C	2.25372300	0.03250900	-1.33816800
	H	1.97382800	-0.41668900	-2.28893600
	C	3.09112600	2.22117500	-0.29916600
	H	2.25395500	2.23646800	0.41021200
	C	3.38043300	-0.76614400	-0.66211200
	H	4.20622100	-0.75522300	-1.39059200
	C	3.86898300	-0.03713200	0.60628600
	H	3.08344700	-0.08768300	1.36872800
	H	4.73827100	-0.55327500	1.01937100
	C	2.61472900	1.50108500	-1.56864700
	H	1.75737200	2.01515800	-2.01226700
	H	3.41425400	1.52615100	-2.32115600
	C	4.23937400	1.42899400	0.34145000
	H	5.11287100	1.46986200	-0.32462500
	H	4.54507200	1.90760300	1.27853200
	C	3.01133100	-2.25327600	-0.41316000
	H	2.19013700	-2.26994500	0.31231900
	C	3.48649000	3.67089300	-0.59522000
	H	4.31815700	3.71343500	-1.30730800
	H	3.80231700	4.18831100	0.31567700
	H	2.65073000	4.23131300	-1.02473800
	C	4.18698200	-3.04365900	0.18549100
	H	5.06533200	-2.99893100	-0.46899000
	H	3.91614800	-4.09698400	0.30009600
	H	4.48263000	-2.67705600	1.17005600
	C	2.52989400	-2.95971500	-1.69177400
	H	3.29493100	-2.91956100	-2.47593300
	H	1.61356900	-2.52671900	-2.09843800
	H	2.32569600	-4.01448000	-1.48759900
	O	-0.29350300	0.11097900	-2.23360400
	C	-1.31924100	-0.00455400	-0.01810800
	C	-1.63949200	1.49181700	0.28192600
	C	-2.05746800	1.90686700	1.55177600
	C	-2.40914400	3.23440200	1.79529700
	C	-2.35077900	4.17777400	0.77349300
	C	-1.95100500	3.77474200	-0.49866300
	C	-1.60750600	2.44695900	-0.74365300
	C	-0.94441500	-0.77118600	1.27902300

	C	-1.58535700	-1.95802100	1.64731400
	C	-1.24155400	-2.63449000	2.82069300
	C	-0.25248800	-2.13408800	3.65813000
	C	0.38895500	-0.94428500	3.31163600
	C	0.04651400	-0.27527000	2.14226800
	C	-2.53498700	-0.66466900	-0.71403100
	C	-2.36982000	-1.81124800	-1.50301800
	C	-3.46205500	-2.45500000	-2.07605700
	C	-4.75232900	-1.96737700	-1.87189600
	C	-4.93183100	-0.83223300	-1.08842700
	C	-3.83339000	-0.18791000	-0.51724700
	H	-2.11706100	1.19249000	2.36179500
	H	-2.73063200	3.52588700	2.78924200
	H	-2.61944900	5.21085500	0.96357000
	H	-1.91202500	4.49283200	-1.31044100
	H	-1.32885700	2.15059600	-1.74655500
	H	-2.36904200	-2.36766900	1.02575000
	H	-1.76002200	-3.55239800	3.07516200
	H	0.01266900	-2.65539800	4.57119400
	H	1.15739200	-0.53244600	3.95699800
	H	0.55268700	0.64876500	1.89986600
	H	-1.37887000	-2.21097900	-1.67848400
	H	-3.30304700	-3.33715400	-2.68639700
	H	-5.60411900	-2.46556100	-2.32121800
	H	-5.92779500	-0.43745600	-0.92033800
	H	-4.00047100	0.69632600	0.08356700
52	O	1.13439500	-0.15836900	-0.43080000
	C	-0.06062100	-0.05472200	-1.02824400
	C	2.32311300	-0.14329500	-1.29988100
	H	2.02776200	-0.59069900	-2.24746900
	C	3.24895400	2.01435800	-0.26057000
	H	2.40836200	2.07200000	0.44222200
	C	3.44655200	-0.95240000	-0.63529600
	H	4.27386000	-0.86101000	-1.35551400
	C	3.92046700	-0.26840600	0.65922900
	H	3.11676000	-0.28205300	1.40141000
	H	4.75218100	-0.83968000	1.08613200
	C	2.74322400	1.31253600	-1.53204700
	H	1.90640700	1.86393200	-1.97034000
	H	3.53994400	1.30914500	-2.28721000
	C	4.35699500	1.18120900	0.40440300
	H	5.24439600	1.18702700	-0.24411000
	H	4.65967900	1.65347700	1.34562300
	C	3.22328200	-2.48299600	-0.48642500
	H	4.20681700	-2.87189300	-0.18819000
	C	3.70991300	3.44364100	-0.56235400
	H	4.04297300	3.95202400	0.34748500
	H	2.90269200	4.03814800	-1.00047200
	H	4.54682100	3.44488700	-1.26947400
	C	2.87320500	-3.14858100	-1.82639300
	H	1.86864400	-2.87919600	-2.16671600
	H	2.90103300	-4.23786000	-1.73128100

	H	3.57916100	-2.86704700	-2.61451400
	C	2.23330900	-2.91018000	0.60757700
	H	2.49488100	-2.49668900	1.58360300
	H	2.23447200	-4.00054600	0.70199500
	H	1.21351600	-2.59492300	0.38372800
	O	-0.20283700	-0.01853400	-2.22819400
	C	-1.26638300	0.05412100	-0.03559000
	C	-1.64706400	1.56621200	0.03726100
	C	-1.59922700	2.38303400	-1.10101100
	C	-1.99908700	3.71688300	-1.04451800
	C	-2.47268900	4.26417000	0.14533400
	C	-2.54814100	3.45768400	1.27735600
	C	-2.13997700	2.12549600	1.22327600
	C	-0.90200800	-0.48890200	1.37055300
	C	-1.51914500	-1.61867800	1.91443900
	C	-1.18484400	-2.08340700	3.18942400
	C	-0.22900100	-1.42318800	3.95116500
	C	0.38996600	-0.28764500	3.42612600
	C	0.05646600	0.17018600	2.15730600
	C	-2.43984600	-0.74905100	-0.65109100
	C	-3.75936600	-0.29928600	-0.56408200
	C	-4.81457700	-1.06408800	-1.06384000
	C	-4.56947900	-2.29520500	-1.66327300
	C	-3.25707700	-2.75674000	-1.75629100
	C	-2.20824900	-1.99188500	-1.25554900
	H	-1.26302500	1.97383100	-2.04404500
	H	-1.94501100	4.32567300	-1.94044100
	H	-2.78431900	5.30180700	0.18800800
	H	-2.92619300	3.86077500	2.21057600
	H	-2.21345900	1.52027700	2.11686600
	H	-2.27502900	-2.14816000	1.35147300
	H	-1.68380600	-2.96284200	3.58142500
	H	0.02856600	-1.78031200	4.94207000
	H	1.13262900	0.24602100	4.00945200
	H	0.54291900	1.05742000	1.77495900
	H	-3.97641000	0.65782500	-0.10844900
	H	-5.82877300	-0.68817400	-0.98540600
	H	-5.38793500	-2.88734600	-2.05703900
	H	-3.04701300	-3.71228400	-2.22402700
	H	-1.19795100	-2.37357800	-1.34234500
56	O	1.06815000	-0.02175300	-0.50648600
	C	-0.14352500	0.03387900	-1.07693800
	C	2.23557100	0.13220700	-1.38904100
	H	1.95104200	-0.27261700	-2.36090400
	C	3.07047000	2.25559100	-0.21685200
	H	2.25248700	2.21594000	0.51360100
	C	3.41435700	-0.66229500	-0.81010800
	H	4.21151600	-0.48524500	-1.54703200
	C	3.89592900	-0.04718900	0.51597400
	H	3.11728700	-0.15852100	1.27655400
	H	4.77161400	-0.59270700	0.88156100
	C	2.56196700	1.62209500	-1.52277600

	H	1.68097800	2.15417000	-1.89303600	
	H	3.33281500	1.72140100	-2.29822400	
	C	4.24349600	1.43962100	0.35160600	
	H	5.10652700	1.54086500	-0.32140600	
	H	4.55282100	1.86019200	1.31494800	
	C	3.19948200	-2.20317700	-0.78726100	
	H	2.56947200	-2.44157400	-1.65484500	
	C	3.44431600	3.72666300	-0.42379000	
	H	3.77176400	4.18685400	0.51331700	
	H	2.59471400	4.30411800	-0.80031500	
	H	4.26199400	3.82597400	-1.14627100	
	C	2.48685200	-2.73849900	0.46463000	
	H	3.11351100	-2.62885800	1.35476700	
	H	2.27766600	-3.80613700	0.34686300	
	H	1.54364500	-2.22822200	0.65773500	
	C	4.53482700	-2.93972900	-0.98646600	
	H	5.23482700	-2.72120000	-0.17324600	
	H	5.01493700	-2.65447700	-1.92747700	
	H	4.38321200	-4.02292200	-1.00349800	
	O	-0.31273800	0.14373900	-2.26864400	
	C	-1.33348800	-0.02214100	-0.06114800	
	C	-1.85236000	1.44072200	0.10677700	
	C	-2.37650800	1.87795100	1.33019900	
	C	-2.90750400	3.15945800	1.47141500	
	C	-2.92561400	4.03706200	0.39111500	
	C	-2.42124100	3.61060300	-0.83491200	
	C	-1.89826700	2.32667100	-0.97851400	
	C	-0.89397600	-0.60469200	1.30694700	
	C	-1.39868400	-1.81139100	1.79930500	
	C	-0.99996000	-2.30867500	3.04314900	
	C	-0.09163500	-1.60554300	3.82435400	
	C	0.41469700	-0.39397100	3.35097000	
	C	0.01722800	0.09652100	2.11324700	
	C	-2.43798600	-0.89676200	-0.70601200	
	C	-3.79190800	-0.57710100	-0.58199700	
	C	-4.77861100	-1.40971700	-1.11234400	
	C	-4.42921900	-2.57879600	-1.78030400	
	C	-3.08113200	-2.91022000	-1.91109900	
	C	-2.10073400	-2.07865800	-1.37918500	
	H	-2.37806000	1.21552500	2.18525700	
	H	-3.30666000	3.46702900	2.43179000	
	H	-3.33181800	5.03630600	0.50130200	
	H	-2.43804500	4.27568100	-1.69143700	
	H	-1.53927400	2.01003400	-1.94823500	
	H	-2.11556600	-2.37667300	1.22016900	
	H	-1.41108600	-3.24822900	3.39570300	
	H	0.21617800	-1.98909400	4.79072200	
	H	1.11925800	0.17265600	3.95012700	
	H	0.41548000	1.04213400	1.77038500	
	H	-4.08996800	0.33004300	-0.07317100	
	H	-5.82205100	-1.13464600	-1.00447200	
	H	-5.19498800	-3.22255200	-2.19831200	

	H	-2.79023300	-3.81535100	-2.43270500
	H	-1.06032000	-2.35872100	-1.49483100
62	O	-1.04659400	-0.02902600	-0.51966700
	C	0.16710400	-0.09249500	-1.08780100
	C	-2.21180700	-0.13685100	-1.41166000
	H	-1.93480300	0.33794900	-2.35374800
	C	-2.99181100	-2.34430400	-0.37196600
	H	-2.16713200	-2.32191800	0.35084200
	C	-3.40703400	0.59317600	-0.78291800
	H	-4.20281700	0.43381000	-1.52534200
	C	-3.86538900	-0.11259700	0.50645300
	H	-3.08377200	-0.03651200	1.26811200
	H	-4.74977300	0.38833300	0.91217700
	C	-2.51644300	-1.62043700	-1.64328200
	H	-1.63280000	-2.11000700	-2.06251900
	H	-3.29748100	-1.68157700	-2.41246100
	C	-4.17949700	-1.59441600	0.25320300
	H	-5.04644700	-1.67473400	-0.41766500
	H	-4.46853400	-2.08019400	1.19164000
	C	-3.24753500	2.13698700	-0.67863200
	H	-2.66888200	2.44858000	-1.55850900
	C	-3.33099200	-3.80969500	-0.66170600
	H	-4.15251100	-3.88884300	-1.38251100
	H	-3.63750500	-4.33132100	0.24991700
	H	-2.47091000	-4.34314000	-1.07770600
	C	-2.49568600	2.63318600	0.56536000
	H	-3.03840200	2.38785400	1.48333400
	H	-2.39286200	3.72167000	0.53058600
	H	-1.49369200	2.21090500	0.63534800
	C	-4.61752600	2.83038700	-0.77446000
	H	-5.26446500	2.55831400	0.06592800
	H	-5.13941500	2.56366900	-1.69850700
	H	-4.50347900	3.91811900	-0.75791700
	O	0.33667300	-0.15691700	-2.28183700
	C	1.34106500	0.00227600	-0.05299900
	C	1.51109600	1.51822100	0.25096900
	C	1.86984000	1.97859000	1.52221500
	C	2.09095800	3.33575800	1.75788200
	C	1.95765700	4.26106300	0.72663900
	C	1.61294800	3.81348500	-0.54718000
	C	1.39895300	2.45777400	-0.78309400
	C	2.62824900	-0.53508000	-0.72755500
	C	2.60230200	-1.70952000	-1.49340200
	C	3.76517100	-2.23427400	-2.04813100
	C	4.98988100	-1.59772900	-1.84972100
	C	5.03252500	-0.43520900	-1.08797200
	C	3.86325700	0.08919400	-0.53430700
	C	1.01732500	-0.80832800	1.23393800
	C	1.75806900	-1.93731200	1.60043400
	C	1.45945300	-2.65745700	2.75965600
	C	0.41630700	-2.26060800	3.58700800
	C	-0.32338200	-1.12861500	3.24479200

	C -0.02586300 -0.41536500 2.08868200<br>H 1.98607700 1.27778300 2.33831100<br>H 2.36975700 3.66478600 2.75297100<br>H 2.12526500 5.31627100 0.91100600<br>H 1.51482900 4.51923100 -1.36470200<br>H 1.15752700 2.12956300 -1.78682900<br>H 1.66627000 -2.22398200 -1.66677800<br>H 3.71180400 -3.14065200 -2.64118300<br>H 5.89596800 -2.00311000 -2.28601700<br>H 5.97521100 0.07473800 -0.92257600<br>H 3.92560800 0.99766500 0.04995500<br>H 2.58511700 -2.26725900 0.98809800<br>H 2.05534500 -3.52805300 3.01084400<br>H 0.18568100 -2.81651200 4.48898200<br>H -1.13421400 -0.79450800 3.88315300<br>H -0.61036800 0.46180900 1.85315200

11 (optimized at the M06-2X/6-311++G(d,p) level)

Conformer no	
1	O 0.97753200 -0.29277400 -0.57265200<br>C -0.20746700 -0.05419400 -1.13902700<br>C 2.16475600 -0.16155400 -1.39322800<br>H 1.95888500 -0.61924700 -2.36146900<br>C 2.80555500 2.02388600 -0.27297700<br>H 1.92282200 1.96180800 0.37318700<br>C 3.26510900 -0.91928100 -0.64763500<br>H 4.14490100 -0.89819100 -1.30883500<br>C 3.61686000 -0.18535900 0.65530100<br>H 2.75976800 -0.26102900 1.33615400<br>H 4.46097500 -0.67635600 1.14319500<br>C 2.47750300 1.31816100 -1.58848000<br>H 1.63232400 1.79991800 -2.09011100<br>H 3.33650000 1.39101800 -2.26840500<br>C 3.94916800 1.28976500 0.42586200<br>H 4.85650600 1.37167500 -0.18932200<br>H 4.17096800 1.77421700 1.38225700<br>C 2.89116300 -2.39566400 -0.40101200<br>H 2.08268500 -2.40566500 0.33998500<br>C 3.12800500 3.49562900 -0.50340300<br>H 4.02191700 3.60418600 -1.12694100<br>H 3.31226300 4.00768500 0.44496800<br>H 2.30081500 4.00433600 -1.00594600<br>C 4.07457100 -3.18416000 0.16704400<br>H 4.92823300 -3.14099400 -0.51787000<br>H 3.80630100 -4.23536200 0.29630000<br>H 4.39998900 -2.80873700 1.13775000<br>C 2.38724200 -3.08863600 -1.66997200<br>H 1.45837700 -2.65590300 -2.04908100<br>H 2.20137100 -4.14803100 -1.47631700<br>H 3.13408900 -3.02339100 -2.46899800<br>O -0.36794300 0.11280000 -2.31684600

	C	-1.29832800	0.06855300	-0.04873300
	C	-0.81425800	1.30417300	0.73460700
	C	-0.22125000	1.21874900	1.99236200
	C	0.29678100	2.35579600	2.60990000
	C	0.24187900	3.58966400	1.97373600
	C	-0.32737200	3.67981300	0.70623400
	C	-0.84542900	2.54671400	0.09188000
	C	-1.38859400	-1.20736500	0.79889500
	C	-0.79775000	-2.40667800	0.40907500
	C	-0.97139100	-3.56544500	1.16236200
	C	-1.74160000	-3.54113700	2.31682200
	C	-2.35281100	-2.35124500	2.70530800
	C	-2.18394300	-1.20035200	1.94876900
	C	-2.68689200	0.26425400	-0.67277600
	C	-3.60242600	1.17632300	-0.14930400
	C	-4.89393000	1.26383400	-0.66360600
	C	-5.28878000	0.43867200	-1.70767700
	C	-4.38640000	-0.48647300	-2.22550000
	C	-3.10185200	-0.57714500	-1.70801700
	H	-0.14373600	0.26054600	2.49031000
	H	0.75218700	2.26845400	3.58969300
	H	0.64773200	4.47216000	2.45403300
	H	-0.36410200	4.63308100	0.19175200
	H	-1.27983700	2.62200400	-0.90021600
	H	-0.19162400	-2.45274200	-0.48746600
	H	-0.50019300	-4.48669500	0.83911800
	H	-1.87496600	-4.44144600	2.90464100
	H	-2.96983300	-2.32266100	3.59586500
	H	-2.67848500	-0.28192600	2.24787200
	H	-3.31350000	1.82848700	0.66622600
	H	-5.58898500	1.98164000	-0.24351600
	H	-6.29128100	0.51051800	-2.11263200
	H	-4.68429000	-1.14128100	-3.03586500
	H	-2.41420700	-1.30806600	-2.11519000
27	O	1.00372300	-0.23232200	-0.57916900
	C	-0.20757800	-0.09814400	-1.11752100
	C	2.15353300	-0.01357900	-1.43324600
	H	1.92669300	-0.42744700	-2.41567700
	C	2.73559000	2.12773300	-0.20591600
	H	1.89232700	1.96691600	0.47999800
	C	3.31635900	-0.76504300	-0.78284200
	H	4.14991500	-0.69582900	-1.49865400
	C	3.74519700	-0.08032600	0.52415300
	H	2.97102800	-0.25770900	1.28065300
	H	4.66140500	-0.54138500	0.89829900
	C	2.38189500	1.48902600	-1.54927700
	H	1.48820100	1.95109500	-1.98057700
	H	3.20204100	1.65594700	-2.25932400
	C	3.96585000	1.42488400	0.36708300
	H	4.81595400	1.60213600	-0.30636900
	H	4.23326100	1.86388100	1.33343100
	C	2.99120800	-2.25912300	-0.57504900

	H	2.21518400	-2.31816700	0.19747100
	C	2.95600700	3.62871700	-0.35618200
	H	3.79283500	3.82972300	-1.03322200
	H	3.18202700	4.09192900	0.60763400
	H	2.06614400	4.11506700	-0.76395500
	C	4.21906300	-3.03294300	-0.08717800
	H	5.04333100	-2.93705400	-0.80240600
	H	3.98564800	-4.09571100	0.00963800
	H	4.57216600	-2.68591700	0.88482600
	C	2.44142000	-2.91555200	-1.84402100
	H	3.13122800	-2.77780600	-2.68413700
	H	1.46864400	-2.51184200	-2.13273700
	H	2.31628400	-3.98960600	-1.68980600
	O	-0.39817200	0.05123400	-2.29425500
	C	-1.30991100	-0.04030500	-0.02411800
	C	-1.12465700	1.36361600	0.59712200
	C	-1.17018000	1.59432100	1.97014200
	C	-1.01382000	2.88289900	2.47831700
	C	-0.81614000	3.95876700	1.62295700
	C	-0.79999500	3.74175500	0.24790700
	C	-0.95899400	2.45836300	-0.25835600
	C	-1.10707100	-1.17135600	1.00905200
	C	-2.05118200	-2.18399500	1.18253100
	C	-1.84979100	-3.20338600	2.11308600
	C	-0.70449900	-3.22834300	2.89385900
	C	0.23782100	-2.21319800	2.74620800
	C	0.03622900	-1.19960600	1.82115300
	C	-2.69091200	-0.14194200	-0.68121200
	C	-2.92968800	-1.07974100	-1.69060900
	C	-4.19978400	-1.23869000	-2.22822300
	C	-5.26010500	-0.46415100	-1.76543400
	C	-5.03568000	0.46518300	-0.75900000
	C	-3.75993300	0.62480100	-0.22193300
	H	-1.33177400	0.77166600	2.65491300
	H	-1.04957000	3.03993500	3.55008700
	H	-0.68817200	4.95830900	2.02136500
	H	-0.66891000	4.57368600	-0.43438200
	H	-0.96875800	2.30424400	-1.33263000
	H	-2.96136800	-2.18841500	0.59794200
	H	-2.60182700	-3.97595600	2.22346400
	H	-0.54913500	-4.02030700	3.61700000
	H	1.13359200	-2.20685900	3.35718400
	H	0.77053000	-0.41091500	1.73119800
	H	-2.11790700	-1.69475800	-2.05818300
	H	-4.36029600	-1.96893400	-3.01274000
	H	-6.25064300	-0.58560600	-2.18804800
	H	-5.85139300	1.07428000	-0.38759300
	H	-3.60459000	1.35624000	0.56201800
31	O	-0.94500600	0.27902300	-0.67872800
	C	0.23852600	-0.06442400	-1.19546400
	C	-2.11904200	0.11420300	-1.51057700
	H	-1.90272400	0.57229800	-2.48023000

	C	-2.78945500	-2.04526600	-0.35849700	
	H	-1.93404300	-1.94647500	0.31988000	
	C	-3.25959300	0.85826200	-0.81742300	
	H	-4.10879300	0.75121500	-1.50724700	
	C	-3.64635100	0.16306600	0.49483200	
	H	-2.81189500	0.23616700	1.20046500	
	H	-4.49661300	0.68181800	0.94867600	
	C	-2.42093000	-1.37277000	-1.68295800	
	H	-1.56452600	-1.86851400	-2.14717400	
	H	-3.26053900	-1.46305500	-2.38428000	
	C	-3.97139600	-1.31482000	0.27982700	
	H	-4.85189800	-1.41572600	-0.37003000	
	H	-4.22429700	-1.78581400	1.23528200	
	C	-2.98597300	2.37442400	-0.66146400	
	H	-2.30251700	2.66491400	-1.47038100	
	C	-3.07207300	-3.52982700	-0.55618200	
	H	-3.29129200	-4.01543400	0.39862000	
	H	-2.21236200	-4.03649500	-1.00313800	
	H	-3.93263800	-3.67765300	-1.21721500	
	C	-2.32815000	2.75685200	0.66850900	
	H	-1.43836900	2.16154900	0.87577700	
	H	-3.03150200	2.62933000	1.49658500	
	H	-2.03180100	3.80905800	0.64949900	
	C	-4.28336800	3.16829500	-0.83640000	
	H	-4.71708000	3.01569000	-1.82786800	
	H	-4.11027900	4.23892600	-0.70163400	
	H	-5.02442500	2.85732500	-0.09266600	
	O	0.40795800	-0.36344600	-2.34523600	
	C	1.30789100	-0.08309300	-0.07933500	
	C	0.74629800	-1.15395300	0.87502800	
	C	0.03511200	-0.83167400	2.02992800	
	C	-0.56457300	-1.82972600	2.79417600	
	C	-0.47395700	-3.16242300	2.40849300	
	C	0.21108500	-3.49095200	1.24259400	
	C	0.81009000	-2.49437500	0.48112500	
	C	1.45011000	1.31266000	0.54241700	
	C	1.03614200	2.45915700	-0.13253300	
	C	1.27542400	3.72357400	0.39886400	
	C	1.93238200	3.85842300	1.61442200	
	C	2.36206400	2.71912900	2.29023100	
	C	2.13040500	1.46007200	1.75342300	
	C	2.70245400	-0.43443900	-0.61818600	
	C	3.57874300	-1.22565700	0.12573200	
	C	4.88371400	-1.44589300	-0.30503000	
	C	5.33598800	-0.87350000	-1.48667600	
	C	4.47574600	-0.06734000	-2.22552400	
	C	3.17540100	0.15681900	-1.79214800	
	H	-0.07115900	0.20438500	2.32783900	
	H	-1.11262400	-1.55915100	3.68940400	
	H	-0.94286700	-3.93710800	3.00372800	
	H	0.27415500	-4.52359200	0.91960800	
	H	1.33301000	-2.75598200	-0.43303700	

	H	0.51346300	2.37838900	-1.08019000
	H	0.94222200	4.60212500	-0.14142900
	H	2.11468900	4.84181500	2.03130100
	H	2.88644100	2.81198000	3.23405500
	H	2.48625300	0.57829600	2.27573600
	H	3.24657000	-1.67779100	1.05267500
	H	5.54426100	-2.06656500	0.28926800
	H	6.35004000	-1.04716000	-1.82676400
	H	4.81832600	0.39287200	-3.14494500
	H	2.52490100	0.79492900	-2.37568200
33	O	-0.98589000	0.42870000	-0.62277400
	C	0.15556300	-0.00539300	-1.16545400
	C	-2.18358900	0.40237100	-1.44082400
	H	-1.94865500	0.89873600	-2.38488200
	C	-2.97408100	-1.79289200	-0.42944600
	H	-2.09572600	-1.80116300	0.22448100
	C	-3.26860900	1.16116400	-0.67240500
	H	-4.13400200	1.14199000	-1.35077300
	C	-3.65919200	0.39767000	0.59942300
	H	-2.79798200	0.37265300	1.27596500
	H	-4.45818200	0.94103000	1.11490500
	C	-2.59849400	-1.04492100	-1.70966800
	H	-1.79549500	-1.56176600	-2.24039100
	H	-3.46350800	-1.02084100	-2.38490900
	C	-4.08431500	-1.03960500	0.30302900
	H	-4.99334700	-1.04437800	-0.31445300
	H	-4.32920500	-1.55865400	1.23554400
	C	-2.96895300	2.65423100	-0.39092500
	H	-3.94919800	3.11836500	-0.22309000
	C	-3.35952600	-3.23717400	-0.72667100
	H	-4.24462000	-3.28006500	-1.37026900
	H	-3.58647900	-3.77693300	0.19674700
	H	-2.54633900	-3.76346000	-1.23399600
	C	-2.33662200	3.35084500	-1.59678700
	H	-1.30695600	3.01288400	-1.74696800
	H	-2.30929000	4.43179900	-1.44212700
	H	-2.89459800	3.15523100	-2.51722700
	C	-2.13597500	2.90467800	0.87194100
	H	-2.67922700	2.61644900	1.77410500
	H	-1.89933500	3.96889900	0.95657600
	H	-1.19584200	2.35185600	0.84735000
	O	0.28628800	-0.27094500	-2.32878000
	C	1.22658200	-0.17289300	-0.06025200
	C	0.57522400	-1.23575500	0.84589600
	C	-0.16238600	-0.90239800	1.98204200
	C	-0.84138000	-1.88334400	2.70001200
	C	-0.80917600	-3.20981500	2.28357000
	C	-0.10253700	-3.54707600	1.13356900
	C	0.57907100	-2.56748300	0.41991700
	C	1.48960700	1.17958800	0.61472100
	C	1.29894500	2.36999100	-0.08529700
	C	1.62868400	3.59505200	0.48454400

	C	2.16331300	3.64613000	1.76573600
	C	2.38183300	2.46175900	2.46327400
	C	2.05609200	1.23952800	1.88849700
	C	2.58924100	-0.62222400	-0.60936000
	C	3.09509300	-0.10906900	-1.80579200
	C	4.37539000	-0.44090000	-2.23406300
	C	5.18175200	-1.27616100	-1.46904900
	C	4.69878000	-1.76862800	-0.26267300
	C	3.41605000	-1.44154300	0.16266600
	H	-0.23059300	0.13166200	2.29810300
	H	-1.40743500	-1.60371400	3.58127000
	H	-1.34007100	-3.97196500	2.84184800
	H	-0.08572500	-4.57318900	0.78510100
	H	1.11955900	-2.83603400	-0.48165100
	H	0.88718400	2.34992400	-1.08933200
	H	1.46137000	4.50829700	-0.07457600
	H	2.41608100	4.59886200	2.21540300
	H	2.81310200	2.48851700	3.45710800
	H	2.24699500	0.32234100	2.43463600
	H	2.48781800	0.54651200	-2.41461200
	H	4.74290100	-0.04159000	-3.17225400
	H	6.17854300	-1.53473600	-1.80604600
	H	5.31928900	-2.40956200	0.35293700
	H	3.05748400	-1.83439700	1.10689500
50	O	-1.05115800	0.11938200	-0.56288600
	C	0.14664000	-0.09532100	-1.10850200
	C	-2.20519500	0.12200000	-1.43823500
	H	-1.92928400	0.65618300	-2.34874800
	C	-3.01102400	-2.12143300	-0.54991100
	H	-2.16037800	-2.16184200	0.14360700
	C	-3.33147400	0.84285100	-0.69709500
	H	-4.16658100	0.83409300	-1.41303200
	C	-3.77161500	0.03260800	0.52787600
	H	-2.94932500	-0.00599400	1.24888500
	H	-4.60447600	0.54512600	1.02077400
	C	-2.57965800	-1.31880700	-1.78008800
	H	-1.73729100	-1.79882600	-2.28556000
	H	-3.40701500	-1.28762200	-2.50044300
	C	-4.15995900	-1.39883100	0.15745300
	H	-5.03529500	-1.38728900	-0.50656900
	H	-4.44984300	-1.95492000	1.05464400
	C	-3.06875200	2.33387800	-0.37148400
	H	-4.06184300	2.76124600	-0.18168600
	C	-3.39529500	-3.54580600	-0.93742300
	H	-2.56632000	-4.05955400	-1.43068300
	H	-4.24520900	-3.53959800	-1.62754800
	H	-3.68066300	-4.12915000	-0.05847700
	C	-2.46546400	3.07859900	-1.56354800
	H	-1.42563800	2.77620200	-1.72363000
	H	-2.46753300	4.15579500	-1.38158700
	H	-3.02285600	2.88912700	-2.48566900
	C	-2.22737500	2.59407800	0.88309500

	H	-1.21000000	2.21626400	0.76907100
	H	-2.66963200	2.14344200	1.77444400
	H	-2.15873800	3.67093100	1.06032700
	O	0.32549600	-0.21327400	-2.29036800
	C	1.26449800	-0.08318200	-0.02718700
	C	2.60041600	-0.50164200	-0.65501300
	C	2.67024100	-1.58752900	-1.53428000
	C	3.88864300	-2.01345900	-2.04572500
	C	5.06728200	-1.36579800	-1.68607400
	C	5.01171300	-0.29303200	-0.80750500
	C	3.78784100	0.13537600	-0.29790000
	C	0.86115800	-1.03832700	1.12042800
	C	1.54996400	-2.22397300	1.37451200
	C	1.14249800	-3.08723100	2.39232700
	C	0.04410100	-2.77905500	3.18012800
	C	-0.63936500	-1.58657100	2.95275700
	C	-0.23327100	-0.72924500	1.94144800
	C	1.34273600	1.38610300	0.43523700
	C	1.28542200	2.39724800	-0.52793200
	C	1.35792100	3.73517500	-0.16267500
	C	1.50840900	4.08680000	1.17540000
	C	1.60282800	3.08754200	2.13584900
	C	1.52351000	1.74594600	1.76872700
	H	1.76602000	-2.10779200	-1.82281300
	H	3.91602700	-2.85353600	-2.72975700
	H	6.01759400	-1.69648300	-2.08855600
	H	5.91987900	0.22174200	-0.51663900
	H	3.76723400	0.97921000	0.38066700
	H	2.41792900	-2.48879700	0.78552100
	H	1.69599600	-4.00302500	2.56370600
	H	-0.27268200	-3.45120400	3.96882600
	H	-1.49186000	-1.31956200	3.56745000
	H	-0.76813500	0.19847700	1.78866100
	H	1.18661600	2.13480300	-1.57691500
	H	1.29426600	4.50311300	-0.92494500
	H	1.55863600	5.12985400	1.46481400
	H	1.73816200	3.34665400	3.17943500
	H	1.60328800	0.98030700	2.53050800
52	O	1.06595200	-0.29598100	-0.55921200
	C	-0.13294800	-0.10640400	-1.10617300
	C	2.22309800	-0.19019400	-1.42477200
	H	1.96522500	-0.63285300	-2.38732400
	C	2.91904000	1.96567200	-0.28174900
	H	2.04214000	1.91201900	0.37787400
	C	3.36257900	-0.95112600	-0.75012800
	H	4.21337200	-0.82070500	-1.43512700
	C	3.73112400	-0.27705400	0.57636400
	H	2.88624500	-0.36648900	1.26736300
	H	4.57342900	-0.80760300	1.03254500
	C	2.55036600	1.28974700	-1.60443100
	H	1.69957000	1.79237700	-2.07464200
	H	3.39308800	1.36959300	-2.30257400

	C	4.06861000	1.20309800	0.38394500	
	H	4.96430700	1.29761600	-0.24561500	
	H	4.30966000	1.66441600	1.34683700	
	C	3.15293400	-2.47890100	-0.62083500	
	H	4.15378900	-2.88685400	-0.42842800	
	C	3.26297000	3.43543100	-0.49898500	
	H	3.50445600	3.92768300	0.44628600	
	H	2.42537300	3.97018900	-0.95464400	
	H	4.12900500	3.53413300	-1.16152700	
	C	2.65082800	-3.09514500	-1.92824000	
	H	1.60568900	-2.82666500	-2.11323400	
	H	2.70443100	-4.18516100	-1.88118000	
	H	3.24217800	-2.76505600	-2.78753800	
	C	2.26219600	-2.92269000	0.54449100	
	H	2.64420200	-2.57766100	1.50696800	
	H	2.22411100	-4.01493000	0.57795100	
	H	1.24017000	-2.55174600	0.44224800	
	O	-0.31506800	0.00872900	-2.28888700	
	C	-1.24940200	0.03850500	-0.04000800	
	C	-1.27703100	1.54071700	0.32666500	
	C	-0.98349900	2.52119800	-0.62372800	
	C	-1.05392000	3.87263700	-0.29776500	
	C	-1.43751200	4.27002600	0.97704300	
	C	-1.75919000	3.30225800	1.92247300	
	C	-1.67665100	1.95217900	1.60091500	
	C	-0.91397200	-0.82264300	1.19619800	
	C	-1.60689100	-1.99226100	1.49989000	
	C	-1.24603600	-2.77211500	2.60040300	
	C	-0.19669100	-2.38853700	3.42062400	
	C	0.49013500	-1.20860000	3.13962600	
	C	0.13469300	-0.43948400	2.04351900	
	C	-2.58394900	-0.38063900	-0.67142700	
	C	-3.76945800	0.29412000	-0.39024200	
	C	-4.98344200	-0.14985500	-0.91123700	
	C	-5.02954600	-1.27397100	-1.72378700	
	C	-3.85012900	-1.95520700	-2.01288800	
	C	-2.64259800	-1.51150300	-1.49237900	
	H	-0.71646600	2.23627500	-1.63441300	
	H	-0.81549400	4.61494400	-1.05077900	
	H	-1.49272900	5.32227500	1.22974200	
	H	-2.07417800	3.59565500	2.91707800	
	H	-1.92852000	1.21138100	2.35059100	
	H	-2.44055400	-2.30855800	0.88671100	
	H	-1.79906600	-3.67977700	2.81195000	
	H	0.08177000	-2.99418400	4.27512200	
	H	1.30920000	-0.89000000	3.77470700	
	H	0.67947700	0.47331600	1.83476600	
	H	-3.75670600	1.17840900	0.23461300	
	H	-5.89241700	0.39332900	-0.68066700	
	H	-5.97287600	-1.61658900	-2.13255800	
	H	-3.86874700	-2.83221400	-2.64930300	
	H	-1.73446100	-2.05410700	-1.72929200	

56	O	1.00021500	-0.19442700	-0.65815800
	C	-0.22351500	-0.08272600	-1.17145600
	C	2.12488400	0.11857600	-1.51655200
	H	1.90914700	-0.27611100	-2.51176600
	C	2.55811900	2.21421400	-0.16401800
	H	1.69347500	1.99037900	0.47508700
	C	3.35836900	-0.55977800	-0.92349400
	H	4.18019200	-0.24345300	-1.58205900
	C	3.64698300	0.00702100	0.47204600
	H	2.82625000	-0.26629900	1.14289300
	H	4.55562400	-0.44610200	0.87928200
	C	2.27077500	1.63769200	-1.55349400
	H	1.36283800	2.07972000	-1.97518800
	H	3.09553500	1.88783800	-2.23213600
	C	3.79087300	1.53147800	0.43679400
	H	4.66971700	1.80240000	-0.16459800
	H	3.96977500	1.91662500	1.44566500
	C	3.30971700	-2.10351400	-0.99116500
	H	2.81020600	-2.36171000	-1.93409900
	C	2.72111800	3.72924800	-0.22630700
	H	2.90184400	4.14680100	0.76740500
	H	1.82237200	4.20130200	-0.63201400
	H	3.56741000	4.00114000	-0.86549500
	C	2.53025400	-2.76633600	0.14924300
	H	3.02854700	-2.60347300	1.10973900
	H	2.48349700	-3.84605300	-0.01447600
	H	1.51189200	-2.38721600	0.23524100
	C	4.72733600	-2.68008200	-1.04973600
	H	5.28790600	-2.43437800	-0.14271700
	H	5.28351100	-2.29105100	-1.90643300
	H	4.69884700	-3.76935500	-1.13115900
	O	-0.44407000	0.08438300	-2.34091200
	C	-1.31545400	-0.08785700	-0.06949300
	C	-1.40683900	1.38340200	0.40214500
	C	-1.71610900	1.70041200	1.72665100
	C	-1.84688900	3.02497000	2.13169200
	C	-1.66938200	4.05989100	1.22056100
	C	-1.38449100	3.75524500	-0.10521400
	C	-1.26449600	2.43053100	-0.51260100
	C	-0.89488600	-1.02604700	1.08198500
	C	-1.50792400	-2.25936900	1.29363700
	C	-1.07389200	-3.10998100	2.31165000
	C	-0.02787700	-2.73757900	3.14160800
	C	0.58126400	-1.49842700	2.95264200
	C	0.15195200	-0.65770000	1.93816200
	C	-2.65053700	-0.52459700	-0.68653700
	C	-3.85472800	0.04634700	-0.28007300
	C	-5.06911100	-0.41501000	-0.78349700
	C	-5.09727400	-1.45307500	-1.70449900
	C	-3.90006700	-2.02998200	-2.11846400
	C	-2.69109300	-1.56969900	-1.61426100
	H	-1.86017700	0.90818600	2.45108300

	H -2.08788900 3.24462700 3.16543400<br>H -1.76205700 5.09144000 1.53880900<br>H -1.26144300 4.54898400 -0.83311500<br>H -1.07720600 2.21479900 -1.55785600<br>H -2.33569100 -2.57120300 0.67043600<br>H -1.56649200 -4.06522000 2.45097100<br>H 0.30827400 -3.39856000 3.93171700<br>H 1.39784500 -1.18813100 3.59488600<br>H 0.63922500 0.29987400 1.80174900<br>H -3.85527900 0.86380800 0.43023700<br>H -5.99270000 0.04658900 -0.45427800<br>H -6.04117400 -1.80916200 -2.10036200<br>H -3.90585100 -2.83929500 -2.83911700<br>H -1.76961400 -2.03100200 -1.94893200

14 (optimized at the B3LYP/6-311++G(d,p) level)

Conformer no	
1	O -1.31685100 -0.21812100 -0.22959000<br>C -0.18101200 -0.13744300 -0.97783000<br>C -2.55672200 -0.34359900 -0.91588000<br>H -2.39951300 -0.14181100 -1.98050100<br>C -3.67884900 0.54601500 -0.34801200<br>C -4.88834200 -0.28503300 -0.83072900<br>C -3.12392000 -1.76061300 -0.79246400<br>O -0.18730100 -0.16174300 -2.17884400<br>C 1.05943200 0.08510100 -0.06810600<br>C 0.73318700 1.42727700 0.63967600<br>C 0.46163000 1.51918900 2.00643100<br>C 0.11114600 2.74041400 2.58496800<br>C 0.01283400 3.88899500 1.80525800<br>C 0.26152900 3.80586000 0.43533400<br>C 0.61502700 2.58839300 -0.13908500<br>C 2.34022000 0.16286700 -0.93615000<br>C 2.60884900 -0.85298700 -1.86402900<br>C 3.79178200 -0.86175900 -2.59420300<br>C 4.74526000 0.13858300 -2.40516700<br>C 4.50013600 1.14143500 -1.47393100<br>C 3.30836700 1.15260100 -0.74649400<br>C 1.27861500 -1.09372600 0.91225900<br>C 2.34184300 -1.01193300 1.82424200<br>C 2.63224800 -2.06288400 2.68673200<br>C 1.87414200 -3.23346500 2.64701100<br>C 0.83037700 -3.33404600 1.73444600<br>C 0.53260600 -2.27452900 0.87390400<br>H 0.51615000 0.63619800 2.62842200<br>H -0.08769500 2.78705700 3.65008100<br>H -0.25718800 4.83715800 2.25649100<br>H 0.18429800 4.69015100 -0.18774100<br>H 0.80608600 2.54036200 -1.20532300<br>H 1.89094100 -1.64738900 -2.02008800<br>H 3.97025600 -1.65556200 -3.31102800<br>H 5.66851400 0.13056900 -2.97359800

	H	5.23404200	1.92202200	-1.30581400
	H	3.14553000	1.94427000	-0.02689200
	H	2.95858900	-0.12152700	1.85331400
	H	3.45854500	-1.97089200	3.38307000
	H	2.10212100	-4.05691800	3.31447000
	H	0.23454800	-4.23830500	1.68103900
	H	-0.29265500	-2.39746000	0.18546900
	C	-3.63480300	0.59043500	1.18909700
	C	-3.68361300	1.96064700	-0.93353400
	O	-2.52337800	-2.79364900	-0.72300800
	H	-5.16046200	-0.05144600	-1.86438300
	H	-5.76700000	-0.18259900	-0.19432700
	H	-2.71932700	1.07252400	1.53414400
	H	-3.67378400	-0.40972300	1.62912200
	H	-4.48852900	1.15981500	1.56697100
	H	-3.69413300	1.94184300	-2.02701700
	H	-2.79819600	2.51353800	-0.61047200
	H	-4.56274800	2.51733700	-0.59485000
	O	-4.47499500	-1.67573500	-0.77643500
6	O	1.32265100	0.25432800	-0.28356900
	C	0.19764100	-0.02000300	-0.99856200
	C	2.55366700	0.44023600	-1.01395400
	H	2.32822200	0.55401500	-2.07203600
	C	3.61094600	-0.64045000	-0.71663100
	C	4.25749600	-0.05856100	0.55924000
	C	3.17590200	1.72058200	-0.42940100
	O	0.21036900	-0.22742700	-2.18217200
	C	-1.04125700	-0.09789700	-0.06370200
	C	-2.33332700	-0.29646300	-0.89569800
	C	-3.34081900	-1.17573300	-0.48709700
	C	-4.54750500	-1.26129400	-1.18263100
	C	-4.77110600	-0.46598500	-2.30138200
	C	-3.77998400	0.42527500	-2.71044900
	C	-2.58039100	0.51367200	-2.01206400
	C	-1.25552000	1.21765000	0.72647600
	C	-0.57813600	2.40474000	0.43508600
	C	-0.87339000	3.58473400	1.12050300
	C	-1.85237700	3.60155000	2.10748800
	C	-2.54504900	2.42627700	2.39858200
	C	-2.25403500	1.25341500	1.71077800
	C	-0.71104600	-1.31735000	0.83801500
	C	-0.31663500	-1.18235600	2.17190100
	C	0.03499700	-2.30001000	2.92960200
	C	0.01195500	-3.57209700	2.36411900
	C	-0.35491500	-3.71663100	1.02689800
	C	-0.70639200	-2.60068700	0.27235300
	H	-3.19523900	-1.80408600	0.38172900
	H	-5.31044300	-1.95323200	-0.84308100
	H	-5.70631000	-0.53405200	-2.84568000
	H	-3.94063800	1.05837700	-3.57597900
	H	-1.83321900	1.22181300	-2.34396500
	H	0.19626400	2.43888700	-0.31894200

	H	-0.32581900	4.48764800	0.87550800
	H	-2.07922300	4.51805100	2.64033300
	H	-3.31999000	2.42317900	3.15717800
	H	-2.81855300	0.35691500	1.93692500
	H	-0.27928200	-0.20218600	2.62769000
	H	0.32879700	-2.16987900	3.96541400
	H	0.28069700	-4.44016900	2.95558000
	H	-0.36819300	-4.69900900	0.56791500
	H	-0.98449100	-2.72980700	-0.76721500
	C	3.05228800	-2.04621400	-0.49451700
	C	4.63280800	-0.63687400	-1.87090000
	O	2.86313900	2.85239400	-0.66483600
	H	5.31502700	-0.30279200	0.66142100
	H	3.72335900	-0.35876900	1.46409000
	H	2.58935200	-2.42832100	-1.40764300
	H	2.30556400	-2.06499900	0.29967700
	H	3.86010400	-2.73173900	-0.22044300
	H	4.16245200	-0.96275800	-2.80207200
	H	5.45578000	-1.32167400	-1.64953100
	H	5.06188900	0.35541100	-2.03847000
	O	4.15871600	1.38220800	0.43648700
8	O	-1.35989100	0.08138000	-0.25676000
	C	-0.20460800	-0.05105500	-0.97102600
	C	-2.58546300	-0.04144000	-0.97198200
	H	-2.41341300	0.18670200	-2.02891100
	C	-3.73025000	0.81535000	-0.40169200
	C	-4.91821800	-0.01922100	-0.93227200
	C	-3.13492800	-1.47122800	-0.89052600
	O	-0.19791400	-0.14678900	-2.16762000
	C	1.05762000	0.00121200	-0.05173400
	C	2.19412400	-0.80044600	-0.74637700
	C	2.86050300	-1.84913700	-0.10640800
	C	3.90601500	-2.52889700	-0.73571800
	C	4.30728700	-2.17102400	-2.01712800
	C	3.65991000	-1.11711800	-2.66187600
	C	2.62251700	-0.43757900	-2.03315300
	C	0.71752600	-0.59287900	1.33518400
	C	0.07252300	-1.83637600	1.41342800
	C	-0.21157400	-2.41970800	2.64302500
	C	0.14613600	-1.77424200	3.82765200
	C	0.78584400	-0.54139600	3.76321800
	C	1.06772200	0.04461800	2.52659700
	C	1.47895500	1.49363800	0.07025700
	C	0.53807500	2.52949900	0.09003500
	C	0.93168000	3.85802700	0.24427300
	C	2.27742700	4.18005600	0.39621100
	C	3.22360400	3.15785500	0.39303700
	C	2.82875400	1.83181000	0.22831100
	H	2.57597600	-2.14693300	0.89295500
	H	4.40250000	-3.33955100	-0.21385400
	H	5.11625600	-2.70071900	-2.50784800
	H	3.96403300	-0.81992100	-3.65947700

	H	2.14262400	0.38126100	-2.54974200
	H	-0.22391500	-2.35398400	0.50835000
	H	-0.71573000	-3.37918200	2.67392000
	H	-0.07459200	-2.22863600	4.78715800
	H	1.06911600	-0.02527400	4.67401800
	H	1.56344700	1.00597700	2.50417500
	H	-0.51595800	2.30559900	-0.00316200
	H	0.18108400	4.64084300	0.24711000
	H	2.58486000	5.21282100	0.51528300
	H	4.27612600	3.38899700	0.51411400
	H	3.58110100	1.05399600	0.22146900
	C	-3.72285300	0.81686700	1.13685300
	C	-3.75443300	2.24485300	-0.95006400
	O	-2.52278700	-2.49578400	-0.82381400
	H	-5.17077200	0.23869100	-1.96519100
	H	-5.81117200	0.05832900	-0.31235500
	H	-2.82295500	1.29782700	1.52215400
	H	-3.75731100	-0.19557400	1.54697200
	H	-4.59284600	1.36404900	1.51022900
	H	-3.73505200	2.25649800	-2.04338400
	H	-2.89407000	2.81475800	-0.59057900
	H	-4.65545900	2.77100300	-0.62038000
	O	-4.48906500	-1.40378500	-0.90327800
39	O	1.36960100	0.12676700	-0.26369200
	C	0.22530300	-0.11956500	-0.94500200
	C	2.60018000	0.18904700	-1.01499300
	H	2.37364800	0.35294100	-2.06603200
	C	3.52219000	-1.01869600	-0.75136900
	C	4.23671800	-0.54878100	0.53460900
	C	3.37914100	1.37201400	-0.41301000
	O	0.20759600	-0.33836200	-2.12840400
	C	-1.04275200	-0.01842600	-0.04157200
	C	-1.44175800	1.48672300	-0.03421700
	C	-2.07182400	2.05621600	1.07874700
	C	-2.49359200	3.38456600	1.06212100
	C	-2.28957200	4.17398200	-0.06672300
	C	-1.67234900	3.61615900	-1.18298900
	C	-1.26067100	2.28501300	-1.16993200
	C	-0.73259000	-0.52206700	1.39749100
	C	0.12253500	0.20971900	2.23870000
	C	0.41517900	-0.22682200	3.52570400
	C	-0.14414900	-1.40798800	4.01547600
	C	-1.00216100	-2.13651300	3.20102800
	C	-1.29504200	-1.69591700	1.90758400
	C	-2.16554900	-0.85846300	-0.69937200
	C	-1.89376500	-2.12961400	-1.22489700
	C	-2.90515200	-2.91895500	-1.76296800
	C	-4.22006500	-2.45581200	-1.78882600
	C	-4.50511000	-1.19754000	-1.26993100
	C	-3.48745300	-0.40766700	-0.73270400
	H	-2.24010800	1.46252400	1.96767900
	H	-2.97984000	3.80006900	1.93786900

	H -2.60789100 5.21025500 -0.07628000<br>H -1.50744400 4.21511700 -2.07147000<br>H -0.80412400 1.87140600 -2.06013800<br>H 0.56011300 1.13377200 1.88668700<br>H 1.07726000 0.36268600 4.15058000<br>H 0.08098600 -1.74648100 5.02069300<br>H -1.45773500 -3.05051500 3.56581400<br>H -1.97754200 -2.27809200 1.30463100<br>H -0.88162700 -2.51425000 -1.22103300<br>H -2.66335100 -3.89586900 -2.16689900<br>H -5.00868200 -3.06784600 -2.21189100<br>H -5.52105100 -0.81871300 -1.28451000<br>H -3.73641900 0.57070700 -0.34444700<br>C 2.79936200 -2.35251200 -0.55419100<br>C 4.53077800 -1.11188300 -1.91273400<br>O 3.21962600 2.53687500 -0.63157100<br>H 5.25681300 -0.92266700 0.62597900<br>H 3.67164000 -0.80451600 1.43444400<br>H 2.28997700 -2.65789900 -1.47145600<br>H 2.06328900 -2.29690200 0.24967000<br>H 3.52030500 -3.13499800 -0.29862300<br>H 5.07362600 -0.17377800 -2.06053800<br>H 4.02000000 -1.35761800 -2.84717800<br>H 5.26767600 -1.89433100 -1.71292600<br>O 4.31361400 0.89443200 0.44531600

14 (optimized at the M06-2X/6-311++G(d,p) level)

Conformer no	
1	O -1.25239700 -0.25287900 -0.28574500<br>C -0.11629900 -0.16355100 -1.01287000<br>C -2.47728300 -0.30731100 -0.97280000<br>H -2.35172100 0.04746700 -2.00202400<br>C -3.58501300 0.46218400 -0.25513300<br>C -4.78724200 -0.27032100 -0.87124000<br>C -3.03212100 -1.72571300 -1.02469600<br>O -0.09547500 -0.19462700 -2.20730000<br>C 1.07303500 0.09634800 -0.06957700<br>C 0.67676700 1.44538500 0.56347900<br>C 0.20137600 1.54957000 1.86870100<br>C -0.26433100 2.76879600 2.35666400<br>C -0.27149800 3.89600200 1.54388700<br>C 0.18508800 3.79658400 0.23238900<br>C 0.64951900 2.58065600 -0.25364100<br>C 2.38517300 0.15816700 -0.86254100<br>C 2.67921100 -0.86557800 -1.76711900<br>C 3.89614000 -0.89675500 -2.43223800<br>C 4.85076100 0.08874500 -2.19561800<br>C 4.57684500 1.09774900 -1.28263400<br>C 3.35210400 1.13162400 -0.61894500<br>C 1.23400500 -1.03823700 0.95299100<br>C 2.19056000 -0.88104000 1.96057400<br>C 2.43128400 -1.89346200 2.87734500

	C	1.72896800	-3.09374200	2.79169500
	C	0.79328900	-3.26604900	1.78158300
	C	0.54337500	-2.24511500	0.86507700
	H	0.17856100	0.67421900	2.50610000
	H	-0.62745100	2.83161800	3.37601700
	H	-0.63363900	4.84328400	1.92600100
	H	0.17736900	4.66586300	-0.41486800
	H	0.99296300	2.50638000	-1.28062400
	H	1.95028500	-1.64566000	-1.95141000
	H	4.10058500	-1.69507400	-3.13578000
	H	5.80115400	0.06443000	-2.71543100
	H	5.31516400	1.86515100	-1.08096800
	H	3.15968100	1.92499000	0.09350100
	H	2.75444100	0.04430600	2.01946700
	H	3.17434300	-1.75034900	3.65321000
	H	1.91756300	-3.88838800	3.50378000
	H	0.24661900	-4.19778800	1.69659500
	H	-0.20031500	-2.41350000	0.09384100
	C	-3.52123500	0.21019800	1.25425000
	C	-3.55082000	1.95379600	-0.55639900
	O	-2.42099700	-2.74762300	-1.05326000
	H	-5.03509200	0.11869200	-1.86347100
	H	-5.67462100	-0.25454200	-0.23924200
	H	-2.60127200	0.62770500	1.66695600
	H	-3.54495500	-0.85758600	1.48980000
	H	-4.37332200	0.68841500	1.74319300
	H	-3.60421800	2.14663500	-1.63098800
	H	-2.61879400	2.38301000	-0.17427800
	H	-4.38327700	2.46912700	-0.07015400
	O	-4.37738000	-1.64637000	-1.00973800
6	O	1.24577100	0.42814700	-0.37181600
	C	0.16036400	-0.05106800	-1.01333600
	C	2.47073000	0.49133800	-1.10252400
	H	2.26977200	0.58442100	-2.16892800
	C	3.42758000	-0.64453800	-0.73253800
	C	4.02126600	-0.07528600	0.56654400
	C	3.17412400	1.73509100	-0.54424400
	O	0.18848400	-0.45745000	-2.13878200
	C	-1.04369900	-0.09640100	-0.05440700
	C	-2.35327200	-0.34679200	-0.81375400
	C	-3.37054100	-1.11463300	-0.24699300
	C	-4.60374900	-1.24120800	-0.87829900
	C	-4.83994200	-0.59735900	-2.08655600
	C	-3.83716900	0.18416100	-2.65067900
	C	-2.60777400	0.31359800	-2.01701500
	C	-1.23788600	1.22854400	0.69621800
	C	-0.72388800	2.43477500	0.22393200
	C	-1.00263500	3.62989000	0.88151200
	C	-1.80386500	3.63667500	2.01543300
	C	-2.33602500	2.43865500	2.48447700
	C	-2.05940300	1.24833900	1.82612600
	C	-0.63332600	-1.27310400	0.85495300

	C	0.00438300	-1.06618300	2.07822700
	C	0.48972800	-2.14460800	2.81272400
	C	0.35632000	-3.44173300	2.32927000
	C	-0.25963300	-3.65448000	1.09984400
	C	-0.74415000	-2.57786200	0.36630000
	H	-3.20447500	-1.62271700	0.69575300
	H	-5.37906700	-1.84476100	-0.42117700
	H	-5.79839100	-0.69877200	-2.58176100
	H	-4.01127500	0.69838100	-3.58843700
	H	-1.84303000	0.93411700	-2.46713800
	H	-0.08983600	2.46281900	-0.65451100
	H	-0.58261800	4.55410800	0.50285300
	H	-2.01599600	4.56667100	2.52915900
	H	-2.97074700	2.43111600	3.36289800
	H	-2.48600000	0.32118900	2.19430900
	H	0.13442800	-0.05892900	2.45485800
	H	0.97643300	-1.96679300	3.76473600
	H	0.73372500	-4.28004100	2.90286500
	H	-0.35790900	-4.65895400	0.70528300
	H	-1.20234700	-2.74624500	-0.60236800
	C	2.75353500	-1.99276300	-0.51649400
	C	4.50945700	-0.72637200	-1.81382100
	O	2.97198200	2.87053000	-0.84145800
	H	5.03661900	-0.41906800	0.76424200
	H	3.37962100	-0.29060600	1.42569900
	H	2.26753300	-2.33270400	-1.43407300
	H	2.00308300	-1.94079900	0.27479200
	H	3.49865900	-2.73963300	-0.22835400
	H	4.07461800	-1.04672300	-2.76334700
	H	5.27428400	-1.45090300	-1.52512000
	H	5.00101000	0.23854300	-1.96947400
	O	4.07235500	1.34955100	0.38178600
8	O	-1.32775600	0.12719700	-0.33447100
	C	-0.17529300	-0.12918600	-1.00146500
	C	-2.53384000	-0.04609800	-1.04216600
	H	-2.39357100	0.18504900	-2.10394300
	C	-3.68858800	0.75355800	-0.44059300
	C	-4.85058800	-0.11001400	-0.95983800
	C	-3.03073100	-1.48754300	-0.92643200
	O	-0.15509500	-0.37861200	-2.16924100
	C	1.05232600	0.00002000	-0.06386400
	C	2.20652900	-0.82492600	-0.66074700
	C	2.88120900	-1.79459100	0.07785900
	C	3.96119800	-2.48525000	-0.47192200
	C	4.38270500	-2.21421600	-1.76478100
	C	3.72171500	-1.23792000	-2.50703600
	C	2.65037100	-0.54861600	-1.95976900
	C	0.66743000	-0.51204500	1.33163600
	C	-0.01641900	-1.72783000	1.43848000
	C	-0.34258800	-2.25020600	2.68092700
	C	0.01726800	-1.56928900	3.84312800
	C	0.70306100	-0.36709000	3.74532200

	C	1.02598500	0.16108000	2.49496100
	C	1.43842800	1.49719100	-0.00746000
	C	0.46260000	2.48781400	0.14067100
	C	0.81498900	3.82743000	0.25769200
	C	2.15285200	4.20558400	0.24431400
	C	3.13174500	3.22808900	0.11909900
	C	2.77886900	1.88756600	-0.00714600
	H	2.57732100	-2.01898300	1.09212900
	H	4.46971700	-3.23602700	0.12138400
	H	5.22027800	-2.75233900	-2.19287500
	H	4.04394500	-1.00982700	-3.51624000
	H	2.15776500	0.21864100	-2.54232300
	H	-0.30461900	-2.26838200	0.54163100
	H	-0.87903600	-3.18993700	2.74125700
	H	-0.23681700	-1.97607700	4.81490600
	H	0.98999300	0.17086400	4.64139100
	H	1.55980500	1.10197600	2.43753400
	H	-0.58266000	2.21125400	0.18752400
	H	0.03917300	4.57663800	0.36699500
	H	2.42845500	5.24938400	0.33599400
	H	4.17992200	3.50393900	0.11740800
	H	3.55930100	1.14328700	-0.10211600
	C	-3.62865200	0.70611400	1.08920300
	C	-3.76208400	2.18769000	-0.94760600
	O	-2.37850700	-2.47725400	-0.82326300
	H	-5.11280800	0.14345300	-1.99154000
	H	-5.73974600	-0.05422700	-0.33226500
	H	-2.73562500	1.21597500	1.45464100
	H	-3.59416100	-0.32233200	1.45908400
	H	-4.51011500	1.19549300	1.50974700
	H	-3.79336900	2.22319900	-2.03941700
	H	-2.89058300	2.75571000	-0.61286200
	H	-4.65411100	2.68769500	-0.56089000
	O	-4.37882200	-1.46862100	-0.92826900
27	O	1.26239200	-0.40417300	0.72414300
	C	0.48400800	-0.33562300	-0.36821300
	C	2.61435000	-0.81360600	0.55386600
	H	2.92338100	-1.08286900	1.56416900
	C	3.58895900	0.19668800	-0.08288000
	C	3.86359300	-0.44452500	-1.45920700
	C	2.72655900	-2.07010500	-0.32592000
	O	0.90376500	-0.53177700	-1.47561700
	C	-0.92928700	0.13834200	-0.00086700
	C	-0.65620000	1.62572000	0.30835300
	C	-0.42134100	2.07370500	1.60809500
	C	-0.05596700	3.39557600	1.84404000
	C	0.09664000	4.28294000	0.78456200
	C	-0.11077500	3.83766600	-0.51693300
	C	-0.47922000	2.51832800	-0.75222100
	C	-1.50926500	-0.65461000	1.17828000
	C	-1.07745500	-1.94621100	1.47801900
	C	-1.70853300	-2.68560900	2.47520300

	C	-2.77705900	-2.14799000	3.18117100
	C	-3.22193400	-0.86489700	2.87682700
	C	-2.59613700	-0.12937800	1.87922300
	C	-1.91296900	-0.06543500	-1.16158500
	C	-1.87473200	-1.22717900	-1.93446800
	C	-2.84436500	-1.46446900	-2.90202400
	C	-3.87552600	-0.55348200	-3.10453600
	C	-3.93301900	0.59534900	-2.32515800
	C	-2.96035500	0.83472100	-1.36056500
	H	-0.51263900	1.38532300	2.43914300
	H	0.11407900	3.72907700	2.86112500
	H	0.37923800	5.31236800	0.97108900
	H	0.01675900	4.51636100	-1.35201600
	H	-0.62474000	2.17250900	-1.76997000
	H	-0.24635000	-2.39463500	0.94276200
	H	-1.35817700	-3.68710500	2.69520000
	H	-3.26526300	-2.72475000	3.95776000
	H	-4.06217000	-0.43727500	3.41118700
	H	-2.95951100	0.86392100	1.63888900
	H	-1.08664900	-1.95502000	-1.78561600
	H	-2.79329100	-2.36877200	-3.49693500
	H	-4.63002300	-0.74004800	-3.85962700
	H	-4.73567100	1.31008500	-2.46400300
	H	-3.02179800	1.73542700	-0.76096200
	C	3.02792700	1.61012200	-0.20028200
	C	4.88011200	0.19227700	0.74447100
	O	2.16485100	-3.10581100	-0.13908800
	H	4.90322400	-0.34333200	-1.77082600
	H	3.19518600	-0.05577000	-2.22834300
	H	2.71709100	1.99252600	0.77551000
	H	2.16568900	1.64832800	-0.86685900
	H	3.79316800	2.28006700	-0.60108900
	H	5.29028700	-0.81856800	0.83148200
	H	4.69397700	0.57601700	1.75074700
	H	5.63585200	0.82340100	0.27130600
	O	3.59902800	-1.84913500	-1.31664100
49	O	-1.33872900	-0.16864400	-0.74304900
	C	-0.55487200	-0.24267700	0.34581400
	C	-2.72729600	-0.44004200	-0.57665600
	H	-3.07447100	-0.60035000	-1.59762300
	C	-3.57436900	0.62532700	0.14691100
	C	-3.89546200	-0.06909700	1.48622900
	C	-2.97927000	-1.73498600	0.21659300
	O	-1.00139800	-0.50565200	1.42769100
	C	0.92854200	0.04127600	0.01246100
	C	1.76277200	-0.24056500	1.27253000
	C	2.87447000	-1.07969800	1.24608100
	C	3.63286600	-1.29588400	2.39632800
	C	3.29157600	-0.67749100	3.58924600
	C	2.18311600	0.16562600	3.62751300
	C	1.42974000	0.37906000	2.48359400
	C	1.04144400	1.52176600	-0.43805100

	C	0.37766300	1.94540100	-1.59750300
	C	0.48278500	3.25489300	-2.04448200
	C	1.26697900	4.17450800	-1.35297300
	C	1.95330100	3.75976200	-0.22180000
	C	1.84515800	2.44484300	0.23033600
	C	1.34721300	-0.89699700	-1.12972800
	C	2.34800900	-0.51983400	-2.02345600
	C	2.80503100	-1.41070300	-2.98982600
	C	2.26212300	-2.68754200	-3.07665800
	C	1.26222000	-3.06970800	-2.18830100
	C	0.81118400	-2.18268100	-1.21725100
	H	3.15971900	-1.57980300	0.32957500
	H	4.49114000	-1.95583300	2.35079800
	H	3.87814500	-0.85009700	4.48388700
	H	1.90211100	0.65500100	4.55258300
	H	0.57292000	1.03989900	2.53043200
	H	-0.20809400	1.23605100	-2.16631200
	H	-0.04098600	3.55399200	-2.94514300
	H	1.35168700	5.19619900	-1.70374600
	H	2.58669500	4.45426500	0.31749600
	H	2.41040400	2.14787600	1.10301600
	H	2.78090200	0.47204800	-1.96057300
	H	3.58649400	-1.10263100	-3.67450900
	H	2.61429600	-3.37985900	-3.83228100
	H	0.82793000	-4.06060000	-2.24765200
	H	0.03262500	-2.50650300	-0.53219500
	C	-2.87115900	1.96503900	0.33433700
	C	-4.87535900	0.80872000	-0.64481600
	O	-2.56485800	-2.81790400	-0.05974000
	H	-4.91127300	0.13126600	1.82775500
	H	-3.17587600	0.18980800	2.26339200
	H	-2.60839900	2.40505600	-0.63048300
	H	-1.95803200	1.87070600	0.92489900
	H	-3.53676500	2.65938300	0.85424400
	H	-5.39533400	-0.14539300	-0.77434900
	H	-4.67160100	1.22575400	-1.63417500
	H	-5.54712200	1.48962500	-0.11702500
	O	-3.79970800	-1.47977100	1.24499200

15 (optimized at the B3LYP/6-311++G(d,p) level)

Conformer no				
1	O	1.67283900	0.38268200	-0.64553200
	C	0.49155800	-0.01470800	-1.15977200
	C	2.81734000	0.39610100	-1.54376500
	H	2.50496100	0.83939200	-2.48793900
	C	3.40412500	-1.00603800	-1.71332200
	O	4.23009300	-1.19593200	-0.57121700
	C	3.94332400	1.15725000	-0.82407800
	C	4.91260900	0.04245800	-0.35658000
	H	3.52832700	1.72510100	0.00830400
	O	0.36262900	-0.35072500	-2.30999400

	C	-0.64251600	0.02362700	-0.08348600
	C	-0.51138000	-1.23003700	0.83208300
	C	-1.66244700	-1.78767100	1.40634600
	C	-1.58284100	-2.87662700	2.27135300
	C	-0.34804000	-3.44362000	2.57792400
	C	0.80360700	-2.89792200	2.01909000
	C	0.72539300	-1.79866500	1.16367800
	C	-2.00952500	-0.01814000	-0.81388800
	C	-2.30553500	-1.08683000	-1.67410600
	C	-3.53712000	-1.17425800	-2.31276500
	C	-4.51310400	-0.19980700	-2.10145100
	C	-4.24020100	0.85545100	-1.23934700
	C	-3.00010300	0.94394000	-0.60160600
	C	-0.47723200	1.32912100	0.73011100
	C	-0.33333400	2.54692100	0.04909600
	C	-0.20828100	3.74745900	0.73870900
	C	-0.22543000	3.75854600	2.13438800
	C	-0.36760000	2.55811500	2.82152000
	C	-0.49123500	1.35334700	2.12492200
	H	-2.63412100	-1.37047200	1.17690500
	H	-2.49203300	-3.28333400	2.70043800
	H	-0.28538700	-4.29760100	3.24305100
	H	1.77550500	-3.32151400	2.24673000
	H	1.64436100	-1.38571800	0.77365400
	H	-1.56884400	-1.85941500	-1.84565700
	H	-3.73548100	-2.00827100	-2.97693400
	H	-5.47394000	-0.26872000	-2.59930200
	H	-4.98943000	1.61710200	-1.05312200
	H	-2.81963900	1.77204300	0.06990100
	H	-0.32866700	2.55897600	-1.03618100
	H	-0.09911400	4.67505200	0.18748100
	H	-0.12789200	4.69296400	2.67562200
	H	-0.38095600	2.54973700	3.90587600
	H	-0.59489800	0.43134400	2.68135500
	H	4.43360200	1.85747800	-1.50256900
	H	2.65900300	-1.79853100	-1.74511400
	H	3.99959600	-1.04066500	-2.63900300
	H	5.16652000	0.09960900	0.70202500
	H	5.84186200	0.05917400	-0.94188900
17	O	-1.56519600	-0.63145000	-0.68464500
	C	-0.48743700	0.02815700	-1.14855300
	C	-2.74492000	-0.64327100	-1.53866600
	H	-2.41717800	-0.82636100	-2.55991400
	C	-3.54674900	0.66973000	-1.39891700
	O	-4.76098500	0.34781900	-0.71097200
	C	-3.69282800	-1.70933700	-0.98623300
	C	-4.57758000	-0.89801200	-0.03797500
	H	-3.14931800	-2.52453000	-0.50749800
	O	-0.46279100	0.57766300	-2.22254300
	C	0.64530700	0.09368800	-0.08682100
	C	0.73887300	-1.19963800	0.75577700
	C	1.09710500	-1.17580700	2.10703100

	C	1.29590700	-2.35827300	2.81958700
	C	1.14693700	-3.59167300	2.19275900
	C	0.80671100	-3.62990600	0.84138300
	C	0.61086800	-2.44800600	0.13397700
	C	0.20136400	1.35209500	0.70933500
	C	-0.76316100	1.26141800	1.72192300
	C	-1.23334400	2.40315300	2.36706900
	C	-0.76316300	3.66226400	1.99998800
	C	0.17141300	3.76777900	0.97285300
	C	0.64353500	2.62518300	0.32988200
	C	2.04512900	0.25645900	-0.72692500
	C	2.33872300	-0.14188900	-2.03480600
	C	3.64394600	-0.08220100	-2.52485200
	C	4.68270400	0.37457700	-1.72030100
	C	4.40459400	0.76775400	-0.41221800
	C	3.10353900	0.70411100	0.07593600
	H	1.22731900	-0.23042600	2.61759000
	H	1.57145400	-2.30942200	3.86735100
	H	1.30008600	-4.51125300	2.74610700
	H	0.69773000	-4.58208000	0.33385800
	H	0.36147300	-2.50497400	-0.91926100
	H	-1.15475000	0.29369300	2.00673100
	H	-1.97337900	2.30595000	3.15370300
	H	-1.12901200	4.55099700	2.50174500
	H	0.53389000	4.74180500	0.66363200
	H	1.35701100	2.72816100	-0.47708200
	H	1.55269200	-0.48365500	-2.69199500
	H	3.84161800	-0.39218800	-3.54510600
	H	5.69544000	0.42238000	-2.10440300
	H	5.20140500	1.12157000	0.23270600
	H	2.91243500	1.00673600	1.09833400
	H	-4.29597100	-2.11571200	-1.80212700
	H	-2.96770400	1.40772900	-0.83408000
	H	-3.81236000	1.09754500	-2.36715200
	H	-4.08247300	-0.74681100	0.93119500
	H	-5.56347900	-1.33153200	0.13355600
18	O	1.67388900	0.42796600	-0.56440900
	C	0.51242200	0.01441700	-1.11121000
	C	2.80458600	0.58369300	-1.47026800
	H	2.45762800	1.12462000	-2.34918900
	C	3.43724400	-0.77942300	-1.82944600
	O	4.71041400	-0.83638900	-1.17823300
	C	3.90918300	1.29932600	-0.68961000
	C	4.71747900	0.12985100	-0.12567200
	H	3.50315400	1.96905900	0.06915900
	O	0.40626400	-0.25117200	-2.28178500
	C	-0.64054600	-0.02980500	-0.05475800
	C	-0.04397000	-0.50147400	1.29175200
	C	0.69198300	-1.69523900	1.33236600
	C	1.24072700	-2.16461700	2.51984800
	C	1.06484500	-1.44717600	3.70421700
	C	0.33788500	-0.26246000	3.67840200

	C	-0.21061800	0.20684700	2.48199900
	C	-1.74531500	-0.99496100	-0.56180500
	C	-2.22422800	-2.05513400	0.21296600
	C	-3.25406000	-2.87838400	-0.24899300
	C	-3.82713400	-2.65484200	-1.49523600
	C	-3.36912100	-1.59068200	-2.27222900
	C	-2.34780100	-0.76866100	-1.80921200
	C	-1.23994700	1.40099100	0.06336600
	C	-0.48591100	2.55731700	-0.16678400
	C	-1.04927900	3.82516800	-0.02142100
	C	-2.37622400	3.96633900	0.37266600
	C	-3.13499700	2.82404400	0.61883800
	C	-2.57457100	1.55897400	0.46130700
	H	0.83310000	-2.27141600	0.42383000
	H	1.80578700	-3.09018900	2.52134700
	H	1.49160200	-1.80990100	4.63255400
	H	0.19347200	0.30770800	4.58952400
	H	-0.76683600	1.13479300	2.48856900
	H	-1.80535800	-2.24866100	1.19071100
	H	-3.60445200	-3.69270000	0.37567900
	H	-4.62458300	-3.29464200	-1.85649100
	H	-3.81059800	-1.39633600	-3.24348800
	H	-2.01641100	0.05562600	-2.42459100
	H	0.55428700	2.48250100	-0.45137500
	H	-0.44262300	4.70260200	-0.21675700
	H	-2.81417100	4.95167900	0.48522100
	H	-4.17022200	2.91336300	0.92912100
	H	-3.18598200	0.68526900	0.64704000
	H	4.52467600	1.87910700	-1.38223700
	H	2.79394500	-1.59759400	-1.49079700
	H	3.60145600	-0.88770100	-2.90291900
	H	4.24429700	-0.27670800	0.77813700
	H	5.75751000	0.37164300	0.09586400
20	O	-1.58015700	0.59163900	-0.66533000
	C	-0.44654100	0.07686300	-1.18286700
	C	-2.74535400	0.65560400	-1.53655700
	H	-2.40299900	0.89050000	-2.54221300
	C	-3.70421400	1.70514400	-0.94317800
	O	-4.84626200	1.00385500	-0.44266500
	C	-3.54862100	-0.64915600	-1.46367700
	C	-4.49595400	-0.37538600	-0.29580900
	H	-2.90758500	-1.52044900	-1.32844200
	O	-0.34524300	-0.28657300	-2.32745300
	C	0.64945100	-0.06689300	-0.08635400
	C	1.99474300	-0.50601900	-0.71439000
	C	2.81264600	-1.45952700	-0.10026200
	C	4.07259400	-1.76621500	-0.61544100
	C	4.53999900	-1.12306400	-1.75680100
	C	3.73867200	-0.16198600	-2.37173900
	C	2.48552000	0.14657800	-1.85278800
	C	0.03475900	-1.15256900	0.83638200
	C	-0.09440100	-2.46011000	0.34491800

	C	-0.70898700	-3.45395200	1.10149500
	C	-1.21716100	-3.15927500	2.36592800
	C	-1.11415500	-1.85994800	2.85485400
	C	-0.50115700	-0.86383900	2.09402700
	C	0.94585300	1.27073700	0.63153300
	C	1.78182900	1.26658100	1.75725100
	C	2.14917100	2.45059900	2.38774100
	C	1.70175300	3.67666300	1.89658200
	C	0.88941600	3.69819500	0.76807400
	C	0.51559900	2.50757600	0.14314200
	H	2.47190900	-1.97584900	0.78787400
	H	4.68487800	-2.51114400	-0.11911300
	H	5.51683700	-1.36312500	-2.16138000
	H	4.08982000	0.35225900	-3.25951500
	H	1.88649500	0.90098200	-2.34477300
	H	0.29124700	-2.70290900	-0.63841700
	H	-0.78941600	-4.45875200	0.70159000
	H	-1.69231100	-3.93226700	2.95942700
	H	-1.51544300	-1.61274200	3.83154700
	H	-0.44939600	0.14270000	2.48665900
	H	2.15999700	0.32695400	2.14151200
	H	2.79354100	2.41484100	3.25919600
	H	1.98973300	4.60123800	2.38383800
	H	0.54037600	4.64289400	0.36591500
	H	-0.11951800	2.56149800	-0.73088600
	H	-4.11663200	-0.77469700	-2.38941900
	H	-3.20192100	2.25298500	-0.13888300
	H	-4.05378000	2.41591000	-1.69548900
	H	-3.99605500	-0.54677400	0.66654300
	H	-5.41924300	-0.95502100	-0.32438500
34	O	1.59584000	0.62300400	-0.52751100
	C	0.49793800	0.09625200	-1.10403500
	C	2.77689000	0.75716300	-1.36321700
	H	2.45886800	1.14139400	-2.33138400
	C	3.54027800	-0.57199600	-1.50814500
	O	4.59094100	-0.53060000	-0.54902500
	C	3.77127500	1.67319700	-0.62423700
	C	5.04753900	0.81869700	-0.53073000
	H	3.37479300	1.88845100	0.36889200
	O	0.46496500	-0.25854500	-2.25626200
	C	-0.65360300	-0.06321500	-0.06908600
	C	-1.94497600	-0.56061500	-0.76440900
	C	-2.75707100	-1.54202300	-0.18791900
	C	-3.97360300	-1.90207600	-0.76911100
	C	-4.40226100	-1.28601600	-1.94012200
	C	-3.60669900	-0.29770000	-2.51857400
	C	-2.39761700	0.06419700	-1.93402600
	C	-1.03282400	1.28308200	0.59355500
	C	-1.96091000	1.28216300	1.64491000
	C	-2.40047100	2.46917600	2.22076600
	C	-1.93458500	3.69536700	1.74697500
	C	-1.02907500	3.71329100	0.69190700

	C	-0.58239300	2.51945700	0.12278300
	C	-0.06677600	-1.10384800	0.92050000
	C	0.35324000	-0.77074500	2.21034100
	C	0.94356500	-1.72624300	3.03789900
	C	1.13890200	-3.02776200	2.58594900
	C	0.74641600	-3.36619000	1.29170400
	C	0.15382100	-2.41324500	0.46837600
	H	-2.44595200	-2.03786700	0.72235700
	H	-4.58235300	-2.66759600	-0.30050100
	H	-5.34496600	-1.56770500	-2.39566900
	H	-3.92835600	0.19620700	-3.42882400
	H	-1.80278700	0.83933300	-2.39802500
	H	-2.35397000	0.34196300	2.01207300
	H	-3.11546600	2.43574200	3.03538400
	H	-2.27979600	4.62226800	2.19075800
	H	-0.66322800	4.65750900	0.30370700
	H	0.12512600	2.57180400	-0.69369200
	H	0.23048000	0.23968300	2.57597600
	H	1.25738700	-1.44461600	4.03702100
	H	1.60004700	-3.76826600	3.22965100
	H	0.90288400	-4.37275600	0.91994000
	H	-0.14326400	-2.69015700	-0.53673500
	H	3.93271400	2.61871900	-1.14452900
	H	2.92326700	-1.44717400	-1.31223800
	H	3.95377100	-0.64523800	-2.52479500
	H	5.61269600	0.97079500	0.38878500
	H	5.71212400	1.00445100	-1.38808600
47	O	-1.72450400	0.10735100	-0.43909300
	C	-0.54228200	0.03871800	-1.08428300
	C	-2.91570900	0.30849800	-1.25152000
	H	-2.65557900	0.99397100	-2.05560200
	C	-4.01775800	0.83976500	-0.31364100
	O	-5.03073900	-0.16867100	-0.23505200
	C	-3.48841600	-1.01960500	-1.76430200
	C	-4.45417800	-1.40925500	-0.64494400
	H	-2.71074800	-1.75377400	-1.97469500
	O	-0.44407200	0.04673600	-2.28541600
	C	0.64671500	0.06715100	-0.07849400
	C	0.57642000	-1.07483800	0.96283900
	C	-0.31215400	-2.14857800	0.86340300
	C	-0.27429500	-3.20263200	1.77792700
	C	0.65345800	-3.20380200	2.81351000
	C	1.55385600	-2.14390300	2.91930000
	C	1.51929100	-1.10021300	2.00104600
	C	0.51150800	1.48399500	0.54081300
	C	0.09182000	1.70066600	1.85549200
	C	-0.08420600	2.99553900	2.34492000
	C	0.14139100	4.09697100	1.52465200
	C	0.53746300	3.89302200	0.20334900
	C	0.71668600	2.60116100	-0.28248300
	C	1.98698800	-0.13380500	-0.82737800
	C	3.11595100	0.63770500	-0.53736800

	C	4.34330800	0.37489100	-1.14766900
	C	4.46481900	-0.66751100	-2.06013900
	C	3.34866300	-1.45223700	-2.34863600
	C	2.12830600	-1.19211500	-1.73459200
	H	-1.05060700	-2.17720100	0.07433100
	H	-0.97617800	-4.02282300	1.67457000
	H	0.68148700	-4.02097500	3.52528300
	H	2.29198900	-2.13305500	3.71361400
	H	2.24292300	-0.29869200	2.08721600
	H	-0.10771700	0.86005900	2.50607200
	H	-0.40472100	3.13777500	3.37113300
	H	0.00456800	5.10242000	1.90655600
	H	0.70787400	4.73999100	-0.45204900
	H	1.02072400	2.45862700	-1.31308900
	H	3.04823100	1.45553500	0.16814400
	H	5.20220100	0.99094100	-0.90483000
	H	5.41635200	-0.87066600	-2.53852200
	H	3.42757400	-2.27235200	-3.05368900
	H	1.27935300	-1.82065000	-1.96891400
	H	-4.04279500	-0.83642200	-2.68856800
	H	-3.59175800	1.04342400	0.67411400
	H	-4.48478600	1.74982200	-0.69528700
	H	-3.92338000	-1.88302700	0.19282900
	H	-5.26636200	-2.06378000	-0.96294200
54	O	-1.66055400	0.37054700	-0.62840400
	C	-0.48930100	-0.04695900	-1.14691400
	C	-2.81138800	0.38106000	-1.51619100
	H	-2.50206800	0.82915400	-2.45941100
	C	-3.91607900	1.16282400	-0.80528000
	O	-4.66319700	0.19465400	-0.07949200
	C	-3.42318700	-1.02777400	-1.68951900
	C	-4.77328500	-0.93604900	-0.94513000
	H	-2.76628000	-1.77280000	-1.24068100
	O	-0.37768800	-0.39749700	-2.29458900
	C	0.65838300	-0.00639800	-0.08114100
	C	2.01976000	-0.13084700	-0.81043700
	C	3.07110000	0.75502600	-0.55670400
	C	4.30963600	0.60701400	-1.18497100
	C	4.52282100	-0.43378200	-2.08112400
	C	3.48720100	-1.33295300	-2.33497500
	C	2.25566700	-1.18637000	-1.70555100
	C	0.45138400	-1.20187200	0.90031700
	C	1.51827900	-2.03456600	1.25944700
	C	1.34775100	-3.07347400	2.17504600
	C	0.10537400	-3.30541900	2.75586300
	C	-0.96258500	-2.47600900	2.42018400
	C	-0.79153600	-1.43532200	1.51039200
	C	0.56608000	1.34604700	0.66477400
	C	0.70792400	1.44742300	2.04921100
	C	0.67520500	2.69193100	2.68265400
	C	0.49914600	3.85549400	1.94139800
	C	0.36209500	3.76733800	0.55542000

	C	0.39903600	2.52696700	-0.07189600
	H	2.93759000	1.57264700	0.13818900
	H	5.10400500	1.31231500	-0.96685900
	H	5.48224700	-0.54829400	-2.57327600
	H	3.63712700	-2.15474300	-3.02656600
	H	1.47315900	-1.90165700	-1.91441500
	H	2.49750100	-1.87679300	0.82989100
	H	2.19550500	-3.69959300	2.43079200
	H	-0.02841400	-4.11554300	3.46397700
	H	-1.93625600	-2.63063700	2.87215000
	H	-1.63028600	-0.79121100	1.28968100
	H	0.84483000	0.55526800	2.64607700
	H	0.78631400	2.74443300	3.76010000
	H	0.46952200	4.82084300	2.43403600
	H	0.22941400	4.66574500	-0.03732100
	H	0.30873800	2.47965700	-1.15239600
	H	-3.54391300	-1.28338300	-2.74290600
	H	-3.53148400	1.90652500	-0.10865700
	H	-4.55462900	1.65782500	-1.55303700
	H	-4.99542500	-1.80648500	-0.32775000
	H	-5.60256800	-0.78489200	-1.65097800
65	O	-1.68789000	0.28344600	-0.59345700
	C	-0.49430800	-0.02670200	-1.14327600
	C	-2.83685400	0.34026500	-1.48808200
	H	-2.50482800	0.78443700	-2.42396500
	C	-3.93126400	1.15323800	-0.76661000
	O	-5.03035300	0.26783800	-0.53205600
	C	-3.47766200	-1.04118300	-1.68322400
	C	-4.53510000	-1.07035500	-0.57933700
	H	-2.74580500	-1.84739600	-1.63341700
	O	-0.36623000	-0.28755800	-2.31204600
	C	0.65926100	0.00999700	-0.08292800
	C	2.01200500	0.08407000	-0.83631100
	C	2.96554300	1.06563300	-0.55310800
	C	4.19767700	1.08603100	-1.21140100
	C	4.50028600	0.12215400	-2.16587400
	C	3.56268500	-0.87119400	-2.44940100
	C	2.33861200	-0.89251900	-1.79051700
	C	0.59751300	-1.29530400	0.76638900
	C	-0.61803200	-1.82094200	1.22668200
	C	-0.65383100	-2.96094700	2.02737800
	C	0.52597700	-3.59829600	2.40262800
	C	1.74165400	-3.07664400	1.97012200
	C	1.77612600	-1.94110200	1.16178300
	C	0.45159100	1.25876400	0.80600900
	C	0.54956400	1.21281900	2.19704500
	C	0.39580400	2.36997000	2.96460500
	C	0.14098100	3.59243800	2.35307600
	C	0.04434800	3.65214200	0.96194400
	C	0.20003600	2.49886200	0.20142100
	H	2.76108800	1.82394300	0.19003900
	H	4.91710000	1.86057000	-0.96949900

	H 5.45471200 0.13847800 -2.68027700<br>H 3.78484600 -1.63487200 -3.18660200<br>H 1.63262300 -1.67784600 -2.02072300<br>H -1.54634200 -1.32885500 0.97645000<br>H -1.61079900 -3.34553500 2.36299300<br>H 0.49804600 -4.48462800 3.02637900<br>H 2.67283000 -3.55166600 2.25865800<br>H 2.73520900 -1.55812600 0.84107300<br>H 0.74452700 0.27249600 2.69564800<br>H 0.47451000 2.30720200 4.04434800<br>H 0.01838000 4.48970300 2.94914600<br>H -0.15085500 4.59835400 0.46933800<br>H 0.13506400 2.56785100 -0.87973100<br>H -3.96010300 -1.07637400 -2.66338200<br>H -3.53521100 1.54322300 0.17713300<br>H -4.30004000 1.98512400 -1.36960000<br>H -4.09596900 -1.35354800 0.38815300<br>H -5.37887500 -1.72952200 -0.78576400
70	O 1.67620500 -0.35128300 -0.71074800<br>C 0.48230700 0.07867700 -1.15165900<br>C 2.80598100 -0.24699600 -1.62556100<br>H 2.44064500 -0.43154700 -2.63336300<br>C 3.86799300 -1.25344600 -1.15310400<br>O 4.93332300 -0.50299300 -0.55683300<br>C 3.50416100 1.10859900 -1.47192100<br>C 4.48181900 0.83451200 -0.32932700<br>H 2.79991000 1.91760200 -1.27724600<br>O 0.31579400 0.53305000 -2.25760600<br>C -0.64272000 -0.01162000 -0.07014600<br>C -0.38654600 -1.18764500 0.91205700<br>C -1.24192200 -2.29150900 0.98018600<br>C -1.00046100 -3.34223200 1.86896800<br>C 0.10054200 -3.30985200 2.71550100<br>C 0.95786100 -2.21001900 2.66887500<br>C 0.71531300 -1.16643600 1.78337700<br>C -1.99457200 -0.18943800 -0.80405000<br>C -2.10405600 -1.05573500 -1.90044000<br>C -3.32755600 -1.27433000 -2.52585100<br>C -4.47664300 -0.63150500 -2.06716500<br>C -4.38370400 0.22730100 -0.97694300<br>C -3.15441900 0.44558500 -0.35331700<br>C -0.62592100 1.36053600 0.66951000<br>C -0.90909900 1.45319900 2.03718400<br>C -0.96253400 2.69087900 2.67789000<br>C -0.73234400 3.86423400 1.96516100<br>C -0.46364800 3.78687300 0.60051300<br>C -0.41870000 2.55099900 -0.04119900<br>H -2.11366500 -2.34385600 0.34335200<br>H -1.68479800 -4.18309600 1.89439900<br>H 0.28851200 -4.12387800 3.40664000<br>H 1.81893000 -2.16219000 3.32654600<br>H 1.39156100 -0.32302400 1.77337100

	H -1.22769400 -1.56748700 -2.27818600<br>H -3.38082700 -1.94520300 -3.37616700<br>H -5.42984400 -0.79741700 -2.55648600<br>H -5.26672000 0.73663300 -0.60705900<br>H -3.10880400 1.12221000 0.48982900<br>H -1.09560400 0.55629200 2.61261400<br>H -1.18660100 2.73189900 3.73819000<br>H -0.76844300 4.82598900 2.46434700<br>H -0.29535400 4.69132800 0.02630900<br>H -0.23880200 2.51906500 -1.10818000<br>H 4.05091100 1.33684900 -2.39092200<br>H 3.43118300 -1.94825500 -0.42934700<br>H 4.28611200 -1.82307100 -1.98673200<br>H 3.98227100 0.91275100 0.64590000<br>H 5.35776100 1.48443600 -0.32753300

15 (optimized at the M06-2X/6-311++G(d,p) level)

Conformer no	
1	O 1.59571500 0.49102600 -0.88052700<br>C 0.42094900 -0.04105000 -1.24925700<br>C 2.69384400 0.33812500 -1.79728100<br>H 2.38262100 0.63618400 -2.79742200<br>C 3.23795400 -1.08102500 -1.72349000<br>O 3.99594600 -1.12994500 -0.52153000<br>C 3.84119400 1.13648200 -1.20063100<br>C 4.32945300 0.19486100 -0.09452100<br>H 3.51417000 2.10549600 -0.82544200<br>O 0.24587200 -0.53802600 -2.32679600<br>C -0.61811400 0.02966200 -0.10216300<br>C -0.39490200 -1.21563200 0.78751300<br>C -1.48283000 -1.80887100 1.43341500<br>C -1.30109200 -2.88635100 2.29337400<br>C -0.02833100 -3.39909900 2.51797500<br>C 1.06074100 -2.81385100 1.88425000<br>C 0.88250300 -1.72602900 1.03423200<br>C -2.03084800 -0.01245100 -0.71225800<br>C -2.39810400 -1.10692300 -1.50543300<br>C -3.67497500 -1.20345300 -2.03744000<br>C -4.62102100 -0.21363000 -1.78008600<br>C -4.27479600 0.86428800 -0.97943400<br>C -2.98894400 0.96366000 -0.44706500<br>C -0.39046100 1.31945700 0.69715400<br>C -0.29222700 2.53191200 0.00609500<br>C -0.12957500 3.72972100 0.68423400<br>C -0.06212000 3.73871100 2.07708200<br>C -0.15928400 2.54215500 2.77150800<br>C -0.32192200 1.33762600 2.08586700<br>H -2.48235200 -1.42541300 1.26755000<br>H -2.16093600 -3.32733600 2.78403400<br>H 0.11234000 -4.24404500 3.18157400<br>H 2.06149100 -3.19669800 2.04723300

	H	1.76162400	-1.28051400	0.58573200
	H	-1.67805400	-1.89142600	-1.69795400
	H	-3.93496500	-2.05789100	-2.65135500
	H	-5.61942600	-0.29064000	-2.19447400
	H	-5.00287600	1.63621200	-0.75896300
	H	-2.74945400	1.80860300	0.18532300
	H	-0.35554900	2.53371200	-1.07840500
	H	-0.05717000	4.65794700	0.12944000
	H	0.06594300	4.67312600	2.61049900
	H	-0.10753300	2.53584700	3.85395800
	H	-0.39055800	0.41280300	2.64540100
	H	4.60964200	1.28461600	-1.96231000
	H	2.45196700	-1.83632600	-1.68148000
	H	3.87718300	-1.27935600	-2.59202100
	H	3.81233300	0.39647600	0.84836200
	H	5.40673100	0.25442800	0.06971300
17	O	1.41923100	0.80644300	-0.82415400
	C	0.39862100	0.02437500	-1.19562700
	C	2.59843800	0.73842800	-1.64915000
	H	2.31652200	0.96515300	-2.67643300
	C	3.27992900	-0.64172700	-1.49981700
	O	4.52682000	-0.40853100	-0.86310500
	C	3.61111900	1.70517600	-1.04288100
	C	4.38433100	0.78050500	-0.10607400
	H	3.11783200	2.54292800	-0.55072600
	O	0.38621600	-0.59535600	-2.22388700
	C	-0.64983700	-0.07959800	-0.07007700
	C	-1.05035900	1.29183200	0.48639600
	C	-1.68960100	1.36485900	1.72583600
	C	-2.16089700	2.57609800	2.21430200
	C	-2.01373600	3.73825400	1.46280900
	C	-1.39805700	3.67304300	0.21968000
	C	-0.92099000	2.45816700	-0.26488600
	C	0.12356700	-0.96153300	0.93114600
	C	0.84538700	-0.40943800	1.98930000
	C	1.64589100	-1.21323300	2.79532800
	C	1.75041400	-2.57667800	2.54510200
	C	1.05579100	-3.13078900	1.47501400
	C	0.25384700	-2.32841500	0.67111900
	C	-1.95013900	-0.72046000	-0.57118900
	C	-2.47574600	-0.35684900	-1.81169000
	C	-3.71220600	-0.83428700	-2.22698100
	C	-4.45214600	-1.67659700	-1.40369900
	C	-3.94575800	-2.03068400	-0.15959900
	C	-2.70489900	-1.55468700	0.25371500
	H	-1.82162700	0.46204500	2.31235900
	H	-2.65038800	2.61144900	3.18046900
	H	-2.38300200	4.68413300	1.84108900
	H	-1.28716200	4.56876100	-0.38014900
	H	-0.44395500	2.43120400	-1.23777000
	H	0.79612200	0.65598900	2.17736500
	H	2.19524800	-0.76663000	3.61604700

	H	2.37686900	-3.20063300	3.17160800
	H	1.14227900	-4.18902600	1.25877000
	H	-0.26714200	-2.76372900	-0.17465900
	H	-1.91570400	0.30133000	-2.46385400
	H	-4.09769400	-0.54544700	-3.19761200
	H	-5.41639100	-2.04923700	-1.72842500
	H	-4.51534100	-2.67922400	0.49581400
	H	-2.32500500	-1.84181400	1.22742700
	H	4.27788100	2.07931900	-1.82183600
	H	2.65061400	-1.29330600	-0.88031700
	H	3.46640200	-1.12927300	-2.45589600
	H	3.80847700	0.58516700	0.80903900
	H	5.37925200	1.13583800	0.15839900
18	O	1.57599300	0.57737000	-0.87419300
	C	0.42137600	0.00473900	-1.24658600
	C	2.67416100	0.51035400	-1.80094800
	H	2.36814300	0.90393900	-2.76979600
	C	3.21942000	-0.91048900	-1.86331600
	O	3.99320200	-1.07683700	-0.68303300
	C	3.82136600	1.24039000	-1.11909700
	C	4.27060200	0.19638200	-0.09327200
	H	3.50505300	2.17980200	-0.66701300
	O	0.23763600	-0.42094000	-2.35225700
	C	-0.60738500	-0.00897100	-0.08486200
	C	0.14733900	-0.18266200	1.24195400
	C	1.14627300	-1.15824900	1.33559400
	C	1.82107400	-1.37768200	2.52668400
	C	1.50246600	-0.62651700	3.65753000
	C	0.51035600	0.33975700	3.57657000
	C	-0.16225700	0.56302000	2.37470800
	C	-1.58747200	-1.17161100	-0.32257000
	C	-1.79074500	-2.17556900	0.62188600
	C	-2.71711500	-3.19308800	0.39271700
	C	-3.45463000	-3.21844400	-0.78106400
	C	-3.26848200	-2.21127100	-1.72588700
	C	-2.34923700	-1.19827700	-1.49743300
	C	-1.36265400	1.33766600	-0.11118300
	C	-0.69967300	2.53277600	-0.40022700
	C	-1.37164800	3.74970200	-0.35694300
	C	-2.71630500	3.79811500	-0.00903900
	C	-3.38118700	2.61645700	0.29702800
	C	-2.71220100	1.39789100	0.24344000
	H	1.40977800	-1.75151100	0.46455000
	H	2.59914100	-2.13099100	2.56948700
	H	2.02703800	-0.79535600	4.59054700
	H	0.25384900	0.93093000	4.44806600
	H	-0.92738700	1.32833800	2.32901100
	H	-1.23612200	-2.17291500	1.55104100
	H	-2.85765400	-3.96276500	1.14267300
	H	-4.17402200	-4.00911400	-0.95918600
	H	-3.84433400	-2.21358300	-2.64393400
	H	-2.22313000	-0.41533600	-2.23388200

	H	0.35484100	2.52254500	-0.64440300
	H	-0.83726300	4.66256300	-0.59276700
	H	-3.23986500	4.74606000	0.02597600
	H	-4.42791300	2.63724600	0.57758400
	H	-3.24711100	0.48745800	0.48484800
	H	4.60658300	1.43846500	-1.85204200
	H	2.43402400	-1.66625400	-1.89404700
	H	3.84976500	-1.02161700	-2.75382200
	H	3.69519600	0.28575600	0.83350900
	H	5.33608000	0.25307500	0.13535200
20	O	-1.44369200	0.78232200	-0.89466300
	C	-0.36221700	0.08810900	-1.27419800
	C	-2.60422400	0.69662300	-1.74545400
	H	-2.31751500	0.92354800	-2.77153600
	C	-3.64114300	1.64835900	-1.15717400
	O	-4.44646700	0.88391200	-0.26736200
	C	-3.26509400	-0.66606900	-1.55905400
	C	-3.96560900	-0.45785000	-0.22088600
	H	-2.54534500	-1.48426300	-1.56177300
	O	-0.25046700	-0.42469400	-2.35285400
	C	0.62783800	-0.04824200	-0.09748600
	C	1.90511400	-0.77646000	-0.53979900
	C	2.49628700	-1.75798300	0.25379200
	C	3.71445400	-2.32772500	-0.10850600
	C	4.35739700	-1.92376400	-1.27042100
	C	3.77920100	-0.93603000	-2.06324200
	C	2.56912700	-0.36345600	-1.69731900
	C	-0.20516400	-0.86998700	0.90670900
	C	-0.55800500	-2.17530700	0.54647400
	C	-1.38469200	-2.94027600	1.35918900
	C	-1.87526200	-2.41083600	2.55019200
	C	-1.54343500	-1.11004400	2.90693400
	C	-0.72238800	-0.33900800	2.08581300
	C	1.07828800	1.31694500	0.43723600
	C	1.85725200	1.35205600	1.59809800
	C	2.35707300	2.55037500	2.08642400
	C	2.10304800	3.74117600	1.41003800
	C	1.35065200	3.71493000	0.24438100
	C	0.84033000	2.51117000	-0.23855700
	H	2.00849400	-2.08612300	1.16387400
	H	4.15645200	-3.08923200	0.52334500
	H	5.30272100	-2.36969000	-1.55616500
	H	4.27393600	-0.60812000	-2.96988600
	H	2.13549300	0.41098400	-2.31826400
	H	-0.17628100	-2.59109700	-0.38146100
	H	-1.64405700	-3.95025500	1.06365100
	H	-2.51708300	-3.00631500	3.18875400
	H	-1.93390100	-0.68177800	3.82268400
	H	-0.50136200	0.68352600	2.36405000
	H	2.07871700	0.42609200	2.11841300
	H	2.95476500	2.55414900	2.99039600
	H	2.49593900	4.67818600	1.78657700

	H	1.15385900	4.63275200	-0.29710900
	H	0.25130200	2.51980500	-1.14754000
	H	-3.99379100	-0.82543900	-2.35763900
	H	-3.15329600	2.46565900	-0.62045100
	H	-4.27914500	2.06127400	-1.94385400
	H	-3.25814500	-0.57954500	0.60782700
	H	-4.81973000	-1.11667300	-0.06398000
34	O	1.45204100	0.67902400	-0.93178700
	C	0.38528700	-0.06513600	-1.25218200
	C	2.63623200	0.47813200	-1.72388700
	H	2.36825900	0.48556900	-2.77900600
	C	3.32400900	-0.80969800	-1.27943600
	O	4.00894800	-0.44635400	-0.09746300
	C	3.63535600	1.56113300	-1.30721300
	C	4.59959000	0.81896300	-0.35302700
	H	3.10482600	2.37542000	-0.81460700
	O	0.32728700	-0.73715900	-2.24628300
	C	-0.65588600	-0.06988200	-0.11675900
	C	-2.10082500	-0.19867400	-0.61770400
	C	-3.10068900	-0.35780600	0.34913000
	C	-4.43892500	-0.42249000	-0.00636900
	C	-4.81053700	-0.31442200	-1.34467200
	C	-3.82920100	-0.14274300	-2.30951100
	C	-2.48162400	-0.08707600	-1.95276800
	C	-0.60266100	1.22794500	0.70252700
	C	-0.61621900	1.23930600	2.09413200
	C	-0.66026000	2.44481200	2.79292000
	C	-0.69275100	3.65197300	2.10937900
	C	-0.69416400	3.64891000	0.71598000
	C	-0.65687200	2.44803200	0.02392000
	C	-0.18204600	-1.31849800	0.66156200
	C	1.03214100	-1.27339800	1.35615300
	C	1.53784700	-2.40999700	1.97442200
	C	0.84155400	-3.61306700	1.90466800
	C	-0.35375800	-3.67094100	1.19917000
	C	-0.85872100	-2.53461700	0.57322100
	H	-2.82086400	-0.42971300	1.39496800
	H	-5.19334800	-0.55036500	0.76112000
	H	-5.85553300	-0.36043800	-1.62776600
	H	-4.10399600	-0.05499600	-3.35419500
	H	-1.73760500	0.02547500	-2.72828000
	H	-0.59161200	0.30689600	2.64527600
	H	-0.66903500	2.43296100	3.87653100
	H	-0.72351100	4.58833000	2.65369800
	H	-0.73073400	4.58395200	0.16929600
	H	-0.67543200	2.45499200	-1.06162400
	H	1.59802700	-0.34956500	1.39482500
	H	2.48425900	-2.35264300	2.49893200
	H	1.23503900	-4.50053900	2.38612900
	H	-0.89618200	-4.60608100	1.12353600
	H	-1.77895200	-2.60259200	0.00732200
	H	4.15960000	1.96242300	-2.17450000

	H	2.63078900	-1.61987600	-1.05195200
	H	4.03037200	-1.13766600	-2.05576000
	H	4.73818200	1.32101700	0.60347400
	H	5.57999400	0.67878300	-0.82508100
47	O	-1.67304600	0.24435300	-0.57022100
	C	-0.48279100	0.03435100	-1.15015000
	C	-2.81864100	0.33547500	-1.43803600
	H	-2.54556700	0.91924100	-2.31560600
	C	-3.95029600	0.94724000	-0.60883300
	O	-4.79174400	-0.12486900	-0.19503300
	C	-3.34642900	-1.05935900	-1.75997100
	C	-4.15309200	-1.35685800	-0.49968300
	H	-2.54539700	-1.76645700	-1.97525500
	O	-0.34245000	-0.11078300	-2.33251100
	C	0.64040600	0.07080800	-0.08860100
	C	0.48770400	-1.09830000	0.89597900
	C	-0.43359500	-2.12647200	0.71507900
	C	-0.48933400	-3.19953100	1.60265000
	C	0.37835100	-3.25979900	2.68332200
	C	1.31602200	-2.24522600	2.86394000
	C	1.37431400	-1.18259700	1.97420300
	C	0.46411500	1.45367400	0.56548400
	C	0.00857100	1.62987400	1.86849200
	C	-0.20027600	2.91095100	2.37661200
	C	0.02878200	4.02853300	1.58585400
	C	0.46189600	3.86010900	0.27276300
	C	0.67387700	2.58439400	-0.23087700
	C	2.01939900	-0.09999700	-0.74667300
	C	3.10525200	0.70168900	-0.39781700
	C	4.36828200	0.46353400	-0.93566900
	C	4.56348000	-0.58066700	-1.82842900
	C	3.48855800	-1.39776000	-2.16864800
	C	2.23273000	-1.16451000	-1.62682100
	H	-1.12196600	-2.10758600	-0.12084800
	H	-1.21593200	-3.98763700	1.44241100
	H	0.33352900	-4.09209200	3.37537400
	H	2.00915600	-2.28702300	3.69583700
	H	2.12015200	-0.40634600	2.11084400
	H	-0.20205000	0.77081100	2.49233900
	H	-0.55248600	3.02866300	3.39472300
	H	-0.13409900	5.02304000	1.98412700
	H	0.63562800	4.72291100	-0.35958500
	H	1.01282500	2.45856100	-1.25480000
	H	2.97607500	1.51947300	0.30035300
	H	5.19798400	1.10019700	-0.65140900
	H	5.54418200	-0.76229800	-2.25219700
	H	3.62911000	-2.22187100	-2.85800800
	H	1.40935100	-1.81625000	-1.89066400
	H	-4.00997800	-1.00536300	-2.62615000
	H	-3.53557000	1.47083100	0.25688400
	H	-4.54974600	1.64182200	-1.20131000
	H	-3.49402400	-1.65762200	0.32503000

	H	-4.92696000	-2.11194000	-0.63497500
58	O	-1.50527700	0.59506400	-0.97346500
	C	-0.37215700	-0.04256500	-1.29336600
	C	-2.63915100	0.37563200	-1.82831300
	H	-2.37236400	0.62477400	-2.85480200
	C	-3.75793100	1.21545900	-1.23859500
	O	-4.21435600	0.46082200	-0.13401700
	C	-3.17704800	-1.05070300	-1.65601100
	C	-4.23823600	-0.90322000	-0.54190600
	H	-2.37697300	-1.74098000	-1.38932100
	O	-0.21886000	-0.60126300	-2.34386500
	C	0.63609100	-0.00581100	-0.11647800
	C	2.04482200	-0.34270100	-0.63203900
	C	2.26322100	-1.49875800	-1.39218600
	C	3.53737400	-1.83757800	-1.82533100
	C	4.62821400	-1.03396300	-1.50283000
	C	4.42739900	0.10551000	-0.73871100
	C	3.14699900	0.44803600	-0.30653000
	C	0.13380000	-1.06577200	0.90024500
	C	0.97543700	-2.06410200	1.39203500
	C	0.52310500	-2.97760600	2.34258400
	C	-0.77698700	-2.90919700	2.82226500
	C	-1.61979600	-1.90560800	2.35376600
	C	-1.17158400	-0.99106700	1.40897900
	C	0.62006600	1.39412900	0.51061200
	C	0.63842500	2.51542400	-0.32287300
	C	0.71741100	3.79536100	0.20694600
	C	0.78624800	3.97643200	1.58664600
	C	0.77684400	2.86785500	2.42208700
	C	0.69377300	1.58329700	1.88696800
	H	1.43074100	-2.14249300	-1.64104000
	H	3.67850600	-2.73567300	-2.41524400
	H	5.62263900	-1.29942800	-1.84184700
	H	5.26569800	0.73831000	-0.47168900
	H	3.02144900	1.34130400	0.29143500
	H	1.99853600	-2.13269400	1.04735600
	H	1.20010700	-3.74078000	2.70836900
	H	-1.12852200	-3.61988200	3.56098400
	H	-2.63077400	-1.81998000	2.73615800
	H	-1.83734900	-0.19812600	1.09038100
	H	0.60405500	2.38319300	-1.40036200
	H	0.72839900	4.65278700	-0.45576400
	H	0.84793000	4.97484400	2.00315400
	H	0.83351200	2.99626700	3.49668000
	H	0.69282100	0.72743200	2.55115900
	H	-3.61878800	-1.39761600	-2.59084100
	H	-3.41834500	2.18704900	-0.88235100
	H	-4.55555200	1.34679900	-1.98339900
	H	-4.01837100	-1.51254700	0.33512900
	H	-5.23742900	-1.16044900	-0.91115100
65	O	-1.60725900	0.47330500	-0.79099000

	C	-0.42734400	0.01866800	-1.23193000
	C	-2.69836100	0.48940800	-1.72819800
	H	-2.36900900	0.94841300	-2.65988400
	C	-3.83898300	1.21545100	-1.03697500
	O	-4.49848200	0.24491700	-0.23422700
	C	-3.27117100	-0.91493800	-1.88594100
	C	-4.05035500	-1.06783200	-0.57998300
	H	-2.49685400	-1.66758400	-2.02900900
	O	-0.23930700	-0.28973700	-2.37578200
	C	0.62107800	-0.01238400	-0.08755800
	C	1.98429000	-0.42186500	-0.66563300
	C	3.14937600	0.27504200	-0.34956400
	C	4.38759600	-0.14330700	-0.83506200
	C	4.48067800	-1.26471100	-1.64566000
	C	3.32403100	-1.97244600	-1.96406400
	C	2.09271100	-1.55678400	-1.47834300
	C	0.13842100	-1.01527800	0.99148500
	C	-1.05824800	-0.77930500	1.68391900
	C	-1.49418800	-1.64556800	2.67677000
	C	-0.74099300	-2.76701100	3.01427700
	C	0.45601200	-2.99937700	2.35372800
	C	0.89413400	-2.13063900	1.35474600
	C	0.70107900	1.41171000	0.48663900
	C	0.91774500	1.64460900	1.84252900
	C	1.08432300	2.94293900	2.31921800
	C	1.03401100	4.02320000	1.44759400
	C	0.82393700	3.79829900	0.08985300
	C	0.66387900	2.50278100	-0.38420900
	H	3.10588000	1.15443500	0.28028900
	H	5.27761700	0.41771800	-0.57498100
	H	5.44219700	-1.58835000	-2.02658000
	H	3.37916500	-2.85200500	-2.59482700
	H	1.20636800	-2.12476300	-1.72949400
	H	-1.64779300	0.09895500	1.45620900
	H	-2.42409300	-1.43631200	3.19298700
	H	-1.08077600	-3.44265100	3.79033000
	H	1.06473300	-3.85752300	2.61400500
	H	1.84129800	-2.32991800	0.87222100
	H	0.96626900	0.81151600	2.53356700
	H	1.25397100	3.10572300	3.37714300
	H	1.15873300	5.03263500	1.82103100
	H	0.78891000	4.63214000	-0.60133900
	H	0.52548000	2.33742400	-1.44905800
	H	-3.94048500	-0.92984900	-2.74940200
	H	-3.47576900	2.02640100	-0.40335700
	H	-4.53386800	1.61855400	-1.78206200
	H	-3.40310000	-1.44737300	0.21715700
	H	-4.92042400	-1.71946100	-0.67279100
70	O	-1.49426200	0.53333900	-1.03414700
	C	-0.34317000	-0.09047300	-1.29585100
	C	-2.60732700	0.27827000	-1.90945300
	H	-2.29280900	0.38468900	-2.94660800

	C	-3.70218600	1.23689200	-1.47424600
	O	-4.36138000	0.61396300	-0.37779100
	C	-3.21803300	-1.07396700	-1.55808200
	C	-3.91699500	-0.73792500	-0.24216900
	H	-2.47091300	-1.86206800	-1.47044100
	O	-0.15581200	-0.72294900	-2.29876000
	C	0.64129900	0.00884400	-0.10070700
	C	0.70749600	1.45236900	0.44926600
	C	1.90187700	2.17369400	0.44999700
	C	1.95770400	3.47332600	0.95286300
	C	0.82260600	4.07543200	1.47275200
	C	-0.37217300	3.36022300	1.49759200
	C	-0.42840200	2.06731500	0.99743700
	C	2.02666000	-0.46888100	-0.55053300
	C	2.55708800	-0.04273100	-1.77157800
	C	3.84440700	-0.39699400	-2.15129500
	C	4.63161500	-1.18253400	-1.31332400
	C	4.11787300	-1.60418200	-0.09476300
	C	2.82423000	-1.25023400	0.28326800
	C	0.05421000	-0.98170300	0.93229700
	C	-0.04647500	-0.68468100	2.28973000
	C	-0.55144800	-1.62588100	3.18519800
	C	-0.96072800	-2.87537600	2.73860400
	C	-0.84051700	-3.18984400	1.38791100
	C	-0.33008600	-2.25464100	0.49683600
	H	2.80906100	1.73021400	0.06240100
	H	2.90025800	4.00777200	0.93632000
	H	0.86586300	5.08549800	1.86278000
	H	-1.26740800	3.80780000	1.91345800
	H	-1.36407900	1.52636300	1.04256700
	H	1.96128800	0.57532100	-2.43188100
	H	4.23268000	-0.05919300	-3.10502000
	H	5.63542900	-1.46244700	-1.61031900
	H	4.72017900	-2.21395800	0.56854100
	H	2.44088800	-1.58950200	1.23822400
	H	0.27547000	0.27988200	2.66061600
	H	-0.62136800	-1.37479000	4.23714600
	H	-1.35973300	-3.60205800	3.43644200
	H	-1.13712100	-4.16802400	1.02766000
	H	-0.20844500	-2.52211400	-0.54798700
	H	-3.94037400	-1.35220900	-2.32931900
	H	-3.29083700	2.19721100	-1.15734000
	H	-4.41354600	1.39720900	-2.29154100
	H	-3.21596100	-0.81118200	0.59759100
	H	-4.78404200	-1.36714500	-0.03821700
75	O	-1.58157200	0.50866900	-0.85724200
	C	-0.45037200	-0.11539400	-1.18642200
	C	-2.71506800	0.28878600	-1.71296000
	H	-2.41052500	0.44589200	-2.74689400
	C	-3.80029200	1.24242000	-1.23349400
	O	-4.44122400	0.55733300	-0.17875500
	C	-3.32870700	-1.09817400	-1.45197300

	C	-4.59626100	-0.77887200	-0.63111500
	H	-2.62290200	-1.70972300	-0.88857700
	O	-0.33273600	-0.76257800	-2.19251000
	C	0.63615800	0.00454900	-0.08762200
	C	0.58926700	1.38625900	0.60017300
	C	1.66521800	2.27111100	0.53863400
	C	1.61286700	3.51098000	1.17628400
	C	0.48709300	3.88520100	1.89281900
	C	-0.58894600	3.00381300	1.97616200
	C	-0.53742200	1.77189000	1.34215300
	C	2.00892300	-0.24087800	-0.72802200
	C	2.32414800	0.34397300	-1.95740400
	C	3.59311300	0.21305600	-2.50478200
	C	4.57556200	-0.50718700	-1.83063100
	C	4.27369900	-1.09054000	-0.60786100
	C	2.99861100	-0.95923800	-0.06109100
	C	0.31413500	-1.13858000	0.90479700
	C	-0.01024100	-2.40617900	0.41096200
	C	-0.23916200	-3.47013500	1.27537500
	C	-0.13148000	-3.29224100	2.65079600
	C	0.21596900	-2.04328600	3.14917500
	C	0.43676300	-0.97432600	2.28436000
	H	2.56320800	2.00313000	-0.00217200
	H	2.46435800	4.17804500	1.10982700
	H	0.44624400	4.84758000	2.38935300
	H	-1.47280400	3.27466300	2.54213400
	H	-1.37972500	1.09774300	1.42788100
	H	1.57311400	0.91214300	-2.49317600
	H	3.81345600	0.67226700	-3.46129700
	H	5.56551200	-0.61376000	-2.25848200
	H	5.02861600	-1.65435500	-0.07249600
	H	2.78200900	-1.42244800	0.89397600
	H	-0.06053600	-2.56891800	-0.65988600
	H	-0.49226000	-4.44295800	0.87019700
	H	-0.30864100	-4.12077100	3.32621900
	H	0.31863900	-1.89241100	4.21752800
	H	0.71673400	-0.01225000	2.69407900
	H	-3.55632100	-1.61557600	-2.38349800
	H	-3.40047500	2.18341900	-0.85808300
	H	-4.50792400	1.43628000	-2.05257300
	H	-4.72286400	-1.41591300	0.24280000
	H	-5.49410500	-0.85151800	-1.25867600

18 (optimized at the B3LYP/6-311G(d,p) level)

Conformer no				
1	O	-1.42887100	-1.00169500	-0.90951200
	C	-2.45972000	-0.25217300	-1.34135800
	C	-0.56676000	-1.60231500	-1.92016500
	H	-1.19503400	-1.84907100	-2.78023100
	C	0.46529300	-0.60063200	-2.42060600
	H	1.04094600	-1.04667100	-3.23198500

	C	0.02190400	-2.85490500	-1.29509900
	H	0.68259400	-3.35221500	-2.00901500
	H	-0.77712800	-3.54398100	-1.01742300
	O	-2.63901600	0.02544800	-2.50146000
	C	-3.43897000	0.08341200	-0.18248200
	C	-4.17493100	-1.27648700	-0.03654200
	C	-3.76585200	-2.23959600	0.89218600
	C	-4.37961300	-3.48952700	0.94138300
	C	-5.40078200	-3.80844300	0.05037600
	C	-5.79390100	-2.86995900	-0.90056300
	C	-5.18253000	-1.61993900	-0.94761000
	C	-4.38873000	1.24423700	-0.56212100
	C	-3.94157200	2.33287700	-1.31912700
	C	-4.76818000	3.42740700	-1.55728300
	C	-6.05875500	3.46488700	-1.03651100
	C	-6.51127900	2.39579800	-0.26920000
	C	-5.68356000	1.30072100	-0.03390100
	C	-2.71582100	0.55607200	1.09911800
	C	-3.36394300	0.48473900	2.33849700
	C	-2.78355600	1.01713000	3.48597400
	C	-1.54402400	1.64881100	3.41802900
	C	-0.89542400	1.74248900	2.18993900
	C	-1.47566200	1.19978800	1.04467400
	H	-2.95944300	-2.01615700	1.57742400
	H	-4.05217500	-4.21621500	1.67672000
	H	-5.87884600	-4.78075400	0.09046900
	H	-6.57575500	-3.10949300	-1.61260400
	H	-5.48650000	-0.90565500	-1.70225200
	H	-2.94564700	2.33002700	-1.73866600
	H	-4.39834100	4.25356900	-2.15447300
	H	-6.70200400	4.31720100	-1.22425700
	H	-7.51123200	2.40942800	0.15024300
	H	-6.05772900	0.48032700	0.56535800
	H	-4.33621100	0.01380300	2.41175900
	H	-3.30754500	0.94419100	4.43267300
	H	-1.09291000	2.06911400	4.30985900
	H	0.06480300	2.24005900	2.11334800
	H	-0.94041600	1.28270600	0.10805600
	H	0.59648600	-2.61091900	-0.40102500
	O	1.36837500	-0.18881100	-1.36929600
	C	2.67209800	-0.52464800	-1.48539800
	C	3.48489900	-0.05953500	-0.24629900
	C	3.10808100	1.36804100	0.21234400
	C	3.31865700	1.77160200	1.53579500
	O	3.11452200	-1.17623400	-2.39616800
	C	2.69482300	2.33732300	-0.70831800
	C	2.47503200	3.65577600	-0.31778000
	C	2.67156000	4.03877800	1.00617900
	C	3.09922700	3.08910000	1.93040500
	C	5.00200500	0.00460200	-0.54871300
	C	5.49150900	0.35936100	-1.81045700
	C	6.85704500	0.53051100	-2.02597300

	C	7.76448600	0.35848600	-0.98516200
	C	7.29004100	0.01674400	0.27821400
	C	5.92527900	-0.15447700	0.49187400
	C	3.12374900	-1.17721300	0.77019200
	C	3.79690100	-2.40498400	0.71901700
	C	3.43241500	-3.45549100	1.55683600
	C	2.37785200	-3.30496400	2.45444800
	C	1.68040300	-2.10048700	2.49084200
	C	2.04293200	-1.05050700	1.64949200
	H	3.66090800	1.05404800	2.27049500
	H	2.54266000	2.07146000	-1.74703400
	H	2.15163400	4.38395800	-1.05320500
	H	2.50069900	5.06464900	1.31193400
	H	3.26999700	3.37236000	2.96321600
	H	4.81309800	0.48952200	-2.64114400
	H	7.20901700	0.79799800	-3.01615500
	H	8.82704600	0.49019700	-1.15554600
	H	7.98094200	-0.11705600	1.10325600
	H	5.57976700	-0.41969100	1.48312600
	H	4.60931200	-2.54122000	0.01668500
	H	3.97361600	-4.39355300	1.50322200
	H	2.09653400	-4.12107700	3.11050800
	H	0.84361300	-1.97197700	3.16809700
	H	1.46751000	-0.13556400	1.67367900
	H	-0.03010900	0.30254300	-2.77450600
5	O	-1.61035200	-1.54932400	-0.98500900
	C	-1.88905300	-0.23052600	-0.95883700
	C	-0.51365300	-2.02188300	-1.82389700
	H	-0.16957500	-1.19093300	-2.43598900
	C	0.60809300	-2.50298500	-0.91300400
	H	0.19590300	-3.07716500	-0.08346200
	C	-1.05272900	-3.15086600	-2.69147800
	H	-0.27241700	-3.50385900	-3.36945900
	H	-1.89248400	-2.79843200	-3.29241800
	O	-1.26042000	0.58737200	-1.58045100
	C	-3.13637200	0.07253500	-0.05788700
	C	-3.11616800	1.57012200	0.34384900
	C	-3.28750700	1.97083000	1.67248200
	C	-3.30826100	3.32157300	2.02381200
	C	-3.15894200	4.30327800	1.05163400
	C	-3.00118600	3.92013800	-0.27960200
	C	-2.98651300	2.57421000	-0.62977800
	C	-3.08049400	-0.82332300	1.20119500
	C	-1.87610600	-0.95426200	1.90694400
	C	-1.81041200	-1.70019100	3.07831200
	C	-2.95116100	-2.32955100	3.57638500
	C	-4.15335900	-2.19892000	2.89066900
	C	-4.21682900	-1.45164900	1.71328800
	C	-4.40146800	-0.25052200	-0.91222000
	C	-4.53361200	-1.49047600	-1.55576800
	C	-5.67395200	-1.80337800	-2.28939700
	C	-6.72087800	-0.89010400	-2.39051100

	C	-6.61335400	0.33395300	-1.74042300
	C	-5.46772300	0.64986200	-1.01000600
	H	-3.40573900	1.23043200	2.45088300
	H	-3.44161000	3.59787100	3.06391700
	H	-3.17009000	5.35303400	1.32304700
	H	-2.89070200	4.67199700	-1.05320600
	H	-2.86824700	2.30362900	-1.66824600
	H	-0.97824300	-0.46438000	1.54715800
	H	-0.86604200	-1.78563600	3.60429200
	H	-2.90063100	-2.91226100	4.48937400
	H	-5.05060300	-2.67782800	3.26701500
	H	-5.16351200	-1.36082200	1.19716600
	H	-3.74614200	-2.22450900	-1.46629100
	H	-5.74455000	-2.76902000	-2.77811100
	H	-7.60923900	-1.13326800	-2.96243400
	H	-7.42201600	1.05427200	-1.79628100
	H	-5.41486700	1.60710400	-0.51116900
	H	-1.39241500	-3.99225800	-2.08121800
	O	1.34266600	-1.42640200	-0.29195600
	C	2.34478200	-0.89771100	-1.03249500
	C	3.29203400	-0.01217700	-0.18220300
	C	4.22579300	-1.11234100	0.39664200
	C	5.37836500	-1.50143700	-0.29592600
	O	2.52968800	-1.19845900	-2.18467500
	C	3.86284900	-1.83941900	1.53785100
	C	4.65678900	-2.88512500	2.00181400
	C	5.81976400	-3.24012000	1.32258000
	C	6.17132500	-2.54896500	0.16666300
	C	2.54379700	0.82036200	0.88245000
	C	1.33458300	1.44338000	0.55197800
	C	0.70034000	2.29929600	1.44772200
	C	1.26864200	2.55818500	2.69352200
	C	2.47935500	1.95949800	3.02722500
	C	3.11235300	1.10214300	2.12853800
	C	4.05385200	1.02353100	-1.04288500
	C	5.21973700	1.60441300	-0.52572400
	C	5.88568000	2.61700900	-1.20748200
	C	5.39414200	3.08316300	-2.42488800
	C	4.23085600	2.52489600	-2.94313900
	C	3.56556700	1.50713700	-2.26041900
	H	5.65749500	-0.98526000	-1.20488300
	H	2.95141800	-1.58905200	2.06416100
	H	4.36084700	-3.42503200	2.89455300
	H	6.43911700	-4.05288300	1.68506300
	H	7.06355400	-2.82578200	-0.38382300
	H	0.87354000	1.27040900	-0.41294400
	H	-0.23884000	2.76179800	1.16793000
	H	0.77418000	3.22408000	3.39171700
	H	2.94051000	2.15946700	3.98835700
	H	4.05860000	0.65638500	2.40719000
	H	5.61464900	1.26419000	0.42381200
	H	6.78713100	3.04478000	-0.78266100

	H	5.91008500	3.87318600	-2.95892200
	H	3.83117700	2.87797300	-3.88736400
	H	2.67140000	1.08752600	-2.69695700
	H	1.30191300	-3.11675500	-1.48858600
9	O	1.44896600	0.63132400	-1.27106200
	C	2.77666700	0.85691700	-1.30940100
	C	0.61492600	1.43866000	-2.15333800
	H	1.17516500	1.59646400	-3.07836500
	C	-0.60410500	0.60512600	-2.49148300
	H	-1.17992500	1.09215500	-3.28226600
	C	0.30380300	2.77738300	-1.49879400
	H	-0.33957400	3.37108100	-2.15385300
	H	1.22662200	3.33259900	-1.32897500
	O	3.29418500	1.64487100	-2.06043600
	C	3.51489900	0.06723500	-0.19313900
	C	3.02973300	-1.39627700	-0.08356800
	C	3.16890400	-2.10767500	1.11356900
	C	2.84697700	-3.46057700	1.18873500
	C	2.38384300	-4.13776300	0.06336100
	C	2.25683300	-3.44708600	-1.13869600
	C	2.58034900	-2.09436400	-1.20958100
	C	3.18335100	0.94696700	1.04294700
	C	3.93413000	2.10192100	1.29776900
	C	3.59920100	2.95563000	2.34569500
	C	2.49829000	2.67884800	3.15253900
	C	1.72522500	1.55143100	2.88789300
	C	2.05780000	0.69985500	1.83676300
	C	5.03471000	-0.03368800	-0.46786000
	C	5.92714100	-0.17208900	0.60231600
	C	7.28557700	-0.38578900	0.38646000
	C	7.78430400	-0.47233700	-0.91038100
	C	6.90694800	-0.34765200	-1.98330500
	C	5.54763000	-0.13483400	-1.76578700
	H	3.53543600	-1.60535800	1.99972300
	H	2.96593800	-3.98613400	2.12992200
	H	2.13320100	-5.19087400	0.12034200
	H	1.90849000	-3.96082700	-2.02782400
	H	2.48281700	-1.58573100	-2.16060600
	H	4.78459500	2.33678800	0.67050100
	H	4.20013100	3.83985100	2.52672600
	H	2.23980400	3.34066000	3.97161400
	H	0.85396400	1.33049200	3.49430200
	H	1.42534700	-0.15271400	1.63056100
	H	5.56217000	-0.10821400	1.61966900
	H	7.95243300	-0.48553600	1.23577900
	H	8.84216200	-0.63630800	-1.08213000
	H	7.27811300	-0.41379100	-3.00008700
	H	4.89332800	-0.03173300	-2.61913800
	H	-0.21185600	2.62992200	-0.55047200
	O	-1.43267100	0.47187900	-1.31934500
	C	-2.61751100	-0.14592200	-1.50280800
	C	-3.48315600	-0.09444800	-0.21336400

	C	-3.78752700	1.42292200	-0.09412400
	C	-4.61074200	2.02198500	-1.05884500
	O	-2.96014100	-0.59532000	-2.56591400
	C	-3.19164100	2.24280100	0.86702000
	C	-3.44022600	3.61516600	0.88755500
	C	-4.27503600	4.19536500	-0.06227100
	C	-4.85492200	3.39156000	-1.04192200
	C	-2.75050300	-0.68648300	1.01357100
	C	-1.58235800	-1.44597500	0.90767900
	C	-1.01332200	-2.05030500	2.02961700
	C	-1.59882500	-1.90217400	3.28214700
	C	-2.76841400	-1.15302700	3.40172300
	C	-3.33942800	-0.56245400	2.28014300
	C	-4.76373500	-0.94678000	-0.38553900
	C	-6.00032700	-0.51135300	0.10020700
	C	-7.12710600	-1.33071500	0.03824400
	C	-7.03843700	-2.60461400	-0.51142400
	C	-5.80917800	-3.05431700	-0.98962100
	C	-4.68533900	-2.23842200	-0.92182700
	H	-5.06321700	1.40913500	-1.82963700
	H	-2.52366300	1.81544600	1.60234000
	H	-2.97129900	4.22980700	1.64798800
	H	-4.46712000	5.26224000	-0.04475000
	H	-5.49886800	3.82971800	-1.79636800
	H	-1.09070200	-1.57249100	-0.04696700
	H	-0.10879200	-2.63614000	1.91191400
	H	-1.15615100	-2.37060900	4.15399500
	H	-3.24544400	-1.03659400	4.36863500
	H	-4.26192100	-0.00561000	2.39002900
	H	-6.09533900	0.47751900	0.52973800
	H	-8.07371300	-0.96575700	0.42141000
	H	-7.91393400	-3.24193600	-0.56483800
	H	-5.72229100	-4.04698100	-1.41737900
	H	-3.74258600	-2.61211200	-1.29721100
	H	-0.31248700	-0.38636700	-2.84352000
13	O	1.42783400	0.87079600	-1.11343400
	C	2.50072200	0.11376300	-1.42442700
	C	0.53305300	1.23630100	-2.20814300
	H	1.14277800	1.33638300	-3.10922600
	C	-0.45802000	0.11373100	-2.48984900
	H	-1.02856100	0.35726100	-3.38671800
	C	-0.10373300	2.56277100	-1.83017700
	H	-0.82266700	2.86121000	-2.59599400
	H	0.66017500	3.33811600	-1.74899300
	O	2.69032500	-0.31455700	-2.53452100
	C	3.44400500	-0.10283100	-0.19291500
	C	2.57682000	-0.40269900	1.05108400
	C	2.85333500	0.15257900	2.30155300
	C	2.09841500	-0.19749000	3.42246900
	C	1.05306100	-1.10796500	3.31042200
	C	0.76799200	-1.67229400	2.06670500
	C	1.52468400	-1.32277200	0.95406600

	C	4.26622100	1.20791000	0.00772500
	C	5.65062800	1.17283000	0.20966500
	C	6.37619400	2.34171000	0.43908100
	C	5.73520200	3.57493800	0.47293500
	C	4.35551700	3.62431400	0.28908900
	C	3.63040100	2.45743400	0.06588500
	C	4.38715300	-1.29633600	-0.49308600
	C	5.15231900	-1.32376300	-1.67026300
	C	6.03153700	-2.36856000	-1.93100400
	C	6.17946400	-3.41265300	-1.01912400
	C	5.44092500	-3.39191500	0.15717000
	C	4.55583900	-2.34416700	0.41690100
	H	3.66458700	0.86025300	2.41070900
	H	2.33532200	0.24498100	4.38391100
	H	0.46489100	-1.37838100	4.18024300
	H	-0.04646400	-2.37983200	1.96108400
	H	1.29590800	-1.78337800	-0.00051100
	H	6.17634400	0.22863900	0.19342100
	H	7.44804900	2.27925100	0.59140900
	H	6.29983000	4.48416200	0.64611000
	H	3.83512900	4.57524500	0.32448500
	H	2.55767600	2.51678900	-0.04540900
	H	5.05949300	-0.52456400	-2.39061700
	H	6.60414200	-2.36461100	-2.85197600
	H	6.86476900	-4.22760400	-1.22420900
	H	5.54668800	-4.19049700	0.88319900
	H	3.99821400	-2.35426400	1.34293000
	H	-0.62603100	2.48757000	-0.87605100
	O	-1.37176500	-0.10671200	-1.39260700
	C	-2.65317200	0.29136900	-1.54769100
	C	-3.47884300	0.03201300	-0.25832900
	C	-3.16471200	-1.34344200	0.37451100
	C	-3.31623200	-1.55652200	1.74860400
	O	-3.06247300	0.85108300	-2.53268400
	C	-2.86806900	-2.44896700	-0.43037800
	C	-2.70383500	-3.71733100	0.11919100
	C	-2.84269200	-3.91227000	1.49135400
	C	-3.15458600	-2.82453400	2.30242700
	C	-5.00151400	0.00635600	-0.53784200
	C	-5.53267200	-0.41562400	-1.76121500
	C	-6.90935200	-0.54075100	-1.93829100
	C	-7.78602700	-0.25383300	-0.89712000
	C	-7.27017000	0.15641100	0.32959300
	C	-5.89551900	0.28071700	0.50517100
	C	-3.04714600	1.24850700	0.60593900
	C	-3.70055100	2.47906900	0.46261000
	C	-3.26474000	3.60434300	1.15809100
	C	-2.15860100	3.52529200	2.00062100
	C	-1.48189800	2.31456900	2.12626900
	C	-1.91598500	1.18969200	1.42835700
	H	-3.56368100	-0.72744700	2.39909600
	H	-2.76691200	-2.32929000	-1.50225700

	H -2.47097900 -4.55474100 -0.52914400<br>H -2.71731600 -4.89989100 1.92047900<br>H -3.27866500 -2.95967700 3.37128000<br>H -4.87917000 -0.63284700 -2.59314800<br>H -7.29333100 -0.86275600 -2.89998100<br>H -8.85671600 -0.34972400 -1.03808800<br>H -7.93689000 0.37957600 1.15521100<br>H -5.51712700 0.59897000 1.46849900<br>H -4.55276000 2.56044100 -0.19960500<br>H -3.79127000 4.54432800 1.03554600<br>H -1.82136000 4.40041800 2.54483800<br>H -0.60543500 2.23840100 2.75972200<br>H -1.35555700 0.26901500 1.51464600<br>H 0.07301100 -0.82577500 -2.63472100
15	O -1.58670800 -1.41332100 -1.30963900<br>C -1.76658700 -0.11586000 -0.98669000<br>C -0.53597700 -1.76056100 -2.25924300<br>H -0.15579400 -0.84272300 -2.70261000<br>C 0.57403500 -2.47235100 -1.49841100<br>H 0.15086500 -3.23070600 -0.83992500<br>C -1.15311200 -2.66189400 -3.31972900<br>H -1.97854900 -2.14958500 -3.81640000<br>H -0.40639300 -2.91488200 -4.07593500<br>O -1.08823200 0.77257400 -1.43467100<br>C -2.98239300 0.07212600 -0.01641000<br>C -4.26241800 0.08438800 -0.90883400<br>C -5.24181100 1.07367100 -0.77183600<br>C -6.40348900 1.04929600 -1.54367400<br>C -6.61367800 0.03564900 -2.47144100<br>C -5.65412400 -0.96476000 -2.60874700<br>C -4.49824800 -0.94369100 -1.83417000<br>C -2.82793600 1.41858200 0.73852700<br>C -2.60473900 2.61561800 0.03859500<br>C -2.50926500 3.83128400 0.70706400<br>C -2.64560700 3.88956100 2.09301700<br>C -2.88312000 2.71598400 2.79774400<br>C -2.97339200 1.49526200 2.12715100<br>C -3.02526300 -1.10394700 0.98616300<br>C -1.84857500 -1.50993600 1.63123300<br>C -1.86885100 -2.52323100 2.58297600<br>C -3.06844500 -3.15048500 2.91894100<br>C -4.24361000 -2.74732400 2.29543500<br>C -4.22117300 -1.73271200 1.33700000<br>H -5.10775900 1.87242200 -0.05629200<br>H -7.14367200 1.83091900 -1.41261300<br>H -7.51442000 0.01993400 -3.07464200<br>H -5.80621400 -1.77176800 -3.31719700<br>H -3.78117500 -1.74567300 -1.93524200<br>H -2.50329100 2.59833200 -1.03602900<br>H -2.33047800 4.73772200 0.13917800<br>H -2.57235700 4.83856300 2.61260000<br>H -3.00042400 2.73955700 3.87558100

	H	-3.16086900	0.60006300	2.70282300
	H	-0.90444400	-1.02891600	1.40044500
	H	-0.94329600	-2.81820800	3.06388600
	H	-3.08406200	-3.94160400	3.66034700
	H	-5.18604800	-3.21988900	2.54953600
	H	-5.14773700	-1.43286600	0.86560200
	H	-1.53394400	-3.58776700	-2.88017900
	O	1.31089300	-1.59283000	-0.62165700
	C	2.28934200	-0.86468100	-1.20013200
	C	3.14415500	-0.03125100	-0.19820200
	C	3.26983700	1.39038400	-0.80507900
	C	2.14489300	2.01442000	-1.36131200
	O	2.48648600	-0.88457000	-2.38992100
	C	4.46977700	2.10335100	-0.76474800
	C	4.54727100	3.40287100	-1.26600200
	C	3.42624300	4.01110400	-1.81993600
	C	2.22308600	3.30915600	-1.86425700
	C	4.55512300	-0.69735200	-0.11799000
	C	5.16731700	-1.26758300	-1.24195200
	C	6.45383600	-1.79692900	-1.16575500
	C	7.16862500	-1.75442000	0.02758700
	C	6.58234800	-1.16830900	1.14514200
	C	5.29205800	-0.64731100	1.07265300
	C	2.49280600	0.00940700	1.20777800
	C	2.37445100	-1.17118700	1.95943700
	C	1.83112900	-1.16024500	3.23741800
	C	1.38334700	0.03516300	3.80225500
	C	1.49263300	1.21045700	3.07129100
	C	2.04687700	1.19770500	1.78882800
	H	1.19083400	1.50154000	-1.39759600
	H	5.35707700	1.64858600	-0.34534600
	H	5.49203900	3.93381000	-1.22542800
	H	3.48776900	5.01914800	-2.21480600
	H	1.33772900	3.76708700	-2.29089300
	H	4.64471800	-1.28420900	-2.18766500
	H	6.89832000	-2.23800500	-2.05123900
	H	8.17025000	-2.16572300	0.08404700
	H	7.12631800	-1.11285400	2.08169400
	H	4.86138300	-0.19322600	1.95460200
	H	2.72067000	-2.10773100	1.54153200
	H	1.76203200	-2.08670500	3.79742400
	H	0.95655100	0.04457800	4.79884200
	H	1.14802000	2.14879100	3.49051200
	H	2.12796300	2.12936400	1.24697900
	H	1.26462500	-2.92873000	-2.20859100
17	O	1.43567500	-0.87503800	1.20079500
	C	2.52266000	-0.12408600	1.47018500
	C	0.53700400	-1.17788800	2.30958900
	H	1.14507100	-1.23792700	3.21554700
	C	-0.44383200	-0.03329000	2.53443100
	H	-1.03089600	-0.23370600	3.43100800
	C	-0.10430200	-2.51759600	1.99164000

	H	-0.80983200	-2.79034700	2.77929900	
	H	0.66247500	-3.29057600	1.92049700	
	O	2.74235600	0.33670800	2.56153300	
	C	3.41486700	0.08225000	0.20070500	
	C	3.37895000	-1.20654000	-0.65213300	
	C	3.14869500	-1.18843500	-2.02783000	
	C	3.16603900	-2.36947700	-2.77339800	
	C	3.41144300	-3.58956700	-2.15472700	
	C	3.64536000	-3.62133300	-0.77956000	
	C	3.63051300	-2.44406200	-0.04199400	
	C	4.86058600	0.40920200	0.66094400	
	C	5.09438900	1.49681400	1.51732600	
	C	6.38380400	1.83651300	1.90896200	
	C	7.47862300	1.10646900	1.44785200	
	C	7.26427800	0.03883800	0.58553900	
	C	5.96827300	-0.30442200	0.19515800	
	C	2.83530500	1.29086600	-0.59004100	
	C	3.69124800	2.13541700	-1.30855000	
	C	3.18968700	3.19297500	-2.06385100	
	C	1.82005900	3.43701700	-2.11116600	
	C	0.95685500	2.60391300	-1.40570100	
	C	1.45838400	1.53773700	-0.66055400	
	H	2.95361900	-0.25034900	-2.52992100	
	H	2.98720600	-2.32703600	-3.84229300	
	H	3.42322200	-4.50657200	-2.73309000	
	H	3.84108000	-4.56472500	-0.28186100	
	H	3.82738000	-2.48619800	1.02400600	
	H	4.26181800	2.08051400	1.88146700	
	H	6.53306300	2.67829600	2.57617600	
	H	8.48417700	1.37302500	1.75382800	
	H	8.10286400	-0.53503500	0.20671400	
	H	5.83399600	-1.13438100	-0.48428600	
	H	4.76002000	1.97006700	-1.27859300	
	H	3.87705200	3.82927100	-2.61052300	
	H	1.42973900	4.26633700	-2.69044000	
	H	-0.11266600	2.77804600	-1.42818200	
	H	0.75934300	0.89431500	-0.14497500	
	H	-0.63937400	-2.47929500	1.04257400	
	O	-1.33926800	0.15542400	1.41423300	
	C	-2.62357200	-0.24275800	1.55743100	
	C	-3.42545400	-0.02996900	0.24425900	
	C	-4.95500100	-0.04475600	0.48724300	
	C	-5.81424800	-0.38286700	-0.56611800	
	O	-3.04806100	-0.76878000	2.55390900	
	C	-5.52868200	0.40192400	1.68238000	
	C	-6.91237800	0.48851500	1.82296200	
	C	-7.75413800	0.13868800	0.77216300	
	C	-7.19603100	-0.29605800	-0.42716600	
	C	-2.94137500	-1.24808400	-0.58882300	
	C	-3.54688800	-2.49857900	-0.40927400	
	C	-3.06479000	-3.62755200	-1.06663900	
	C	-1.95660300	-3.53293500	-1.90466700	

	C	-1.32758800 -2.30154500 -2.06766300
	C	-1.81138900 -1.17212100 -1.41116500
	C	-3.14042000 1.34671300 -0.39834900
	C	-2.89722500 2.46938400 0.40103200
	C	-2.77657700 3.73895800 -0.15701500
	C	-2.90242600 3.91673700 -1.53269900
	C	-3.15505500 2.81050800 -2.33943600
	C	-3.27661600 1.54187400 -1.77700900
	H	-5.40291400 -0.72219000 -1.50850000
	H	-4.90320500 0.66960600 2.52099700
	H	-7.32948600 0.83067900 2.76364600
	H	-8.83038900 0.20522200 0.88472400
	H	-7.83510300 -0.56807600 -1.25995700
	H	-4.39807500 -2.59168100 0.25279600
	H	-3.55500900 -4.58289400 -0.91573500
	H	-1.57882600 -4.41174200 -2.41491100
	H	-0.44671000 -2.21684200 -2.69340300
	H	-1.29307000 -0.23084900 -1.53238900
	H	-2.80705500 2.36271400 1.47536300
	H	-2.58543000 4.58990100 0.48714100
	H	-2.81072800 4.90511000 -1.96849800
	H	-3.26435700 2.93135200 -3.41148900
	H	-3.48487400 0.69909900 -2.42370400
	H	0.09565700 0.90598700 2.64756900
18	O	-1.39787800 -1.37818800 -0.59015100
	C	-2.34404900 -0.64784100 -1.22000400
	C	-0.67875100 -2.35030300 -1.40317100
	H	-1.36401200 -2.69139200 -2.18333000
	C	0.49373200 -1.69921200 -2.12545700
	H	0.90305300 -2.41224400 -2.84520700
	C	-0.29266300 -3.49481200 -0.48109300
	H	-1.18669100 -3.92628200 -0.02831100
	H	0.37847800 -3.15391800 0.30710500
	O	-2.50369400 -0.67059800 -2.41453500
	C	-3.27935000 0.07928400 -0.21611600
	C	-3.96411700 1.31612400 -0.84505100
	C	-5.12059400 1.82924500 -0.24249700
	C	-5.71617200 2.99858600 -0.70298300
	C	-5.16111800 3.69207100 -1.77632400
	C	-4.00533800 3.20172000 -2.37415400
	C	-3.41057700 2.02767100 -1.91378600
	C	-2.52583400 0.62183900 1.01858100
	C	-1.28422900 1.24650600 0.85043800
	C	-0.63732800 1.85783900 1.92052200
	C	-1.22393500 1.86435500 3.18476700
	C	-2.46622800 1.26339900 3.36122500
	C	-3.11262700 0.65276400 2.28728700
	C	-4.28269000 -1.06899200 0.08703700
	C	-4.00111900 -2.04053600 1.05647900
	C	-4.85765200 -3.12042700 1.25652200
	C	-6.00322700 -3.26396300 0.47748200
	C	-6.27530300 -2.32391000 -0.51246300

	C	-5.42002700	-1.24254200	-0.71044400	
	H	-5.56260300	1.31030100	0.59930500	
	H	-6.61262000	3.36928400	-0.21818800	
	H	-5.62265700	4.60409700	-2.13792000	
	H	-3.55697700	3.73086300	-3.20778900	
	H	-2.52031500	1.66743000	-2.40711100	
	H	-0.80949000	1.26984300	-0.12289400	
	H	0.32465700	2.33124700	1.76086800	
	H	-0.72069800	2.34039500	4.01881300	
	H	-2.94279700	1.27126800	4.33549800	
	H	-4.08402700	0.20289200	2.44754600	
	H	-3.10524000	-1.95454700	1.65646600	
	H	-4.62415500	-3.85243300	2.02189400	
	H	-6.67061400	-4.10390600	0.63442400	
	H	-7.15336600	-2.43109600	-1.13953500	
	H	-5.63694000	-0.52970100	-1.49482600	
	H	0.21637100	-4.27574100	-1.05253800	
	O	1.58127800	-1.38883200	-1.22283300	
	C	1.87714400	-0.08921500	-1.00089900	
	C	3.17447700	0.06152800	-0.13739200	
	C	4.39114500	-0.13039500	-1.09510000	
	C	5.50819000	0.70942100	-1.01526600	
	O	1.22543300	0.81665300	-1.45142900	
	C	4.43595100	-1.18995300	-2.01321800	
	C	5.54273100	-1.38384600	-2.83507700	
	C	6.64122400	-0.53233000	-2.75282100	
	C	6.61989100	0.51029800	-1.83280300	
	C	3.16824700	-1.01840800	0.96919900	
	C	2.01881000	-1.19335800	1.75308500	
	C	1.99496000	-2.11480300	2.79395400	
	C	3.12470700	-2.88214500	3.07873600	
	C	4.27247100	-2.71172100	2.31335400	
	C	4.29393400	-1.78717100	1.26733900	
	C	3.20332600	1.48146300	0.48544900	
	C	3.05404500	2.61909100	-0.32419500	
	C	3.11606500	3.89841500	0.21710200	
	C	3.34348900	4.07955000	1.58062600	
	C	3.51503100	2.96378100	2.39095600	
	C	3.44632500	1.67940700	1.84805400	
	H	5.51917400	1.52692900	-0.30816800	
	H	3.60915700	-1.88278900	-2.07328500	
	H	5.54490200	-2.20896700	-3.53884700	
	H	7.50275300	-0.68305200	-3.39345700	
	H	7.46938800	1.17860100	-1.74566000	
	H	1.13326200	-0.59792200	1.55909200	
	H	1.09303800	-2.22966500	3.38479600	
	H	3.10795500	-3.60117300	3.89008400	
	H	5.16017500	-3.29770400	2.52456000	
	H	5.19871000	-1.67014600	0.68568500	
	H	2.88569900	2.50545400	-1.38470700	
	H	2.98802600	4.75734100	-0.43227900	
	H	3.39175300	5.07779400	2.00120300	

	H	3.70457200	3.08261800	3.45206200
	H	3.58676000	0.83138000	2.50343400
	H	0.17640200	-0.79816300	-2.64447200
26	O	-1.39639000	-0.27517400	-1.39620400
	C	-2.72771400	-0.45157200	-1.50328800
	C	-0.61008500	-0.42398100	-2.61506900
	H	-1.22792800	-0.06902100	-3.44374800
	C	0.57400200	0.51259600	-2.48759300
	H	1.09462300	0.58804200	-3.44525200
	C	-0.23896400	-1.88455000	-2.83420700
	H	0.37089600	-1.98134400	-3.73697400
	H	-1.14099300	-2.48173800	-2.97020800
	O	-3.27053600	-0.80369600	-2.52007100
	C	-3.46577600	-0.01121200	-0.20882100
	C	-2.76689500	-0.49059100	1.08316800
	C	-3.00439800	0.16473000	2.29757200
	C	-2.48301200	-0.32254900	3.49296300
	C	-1.71429400	-1.48424300	3.50331500
	C	-1.48157500	-2.15379300	2.30573800
	C	-2.00595000	-1.66376900	1.11157500
	C	-4.89200200	-0.60665300	-0.14236100
	C	-5.16798000	-1.89411400	-0.61591400
	C	-6.42601900	-2.46589600	-0.44563500
	C	-7.43649300	-1.76791000	0.20952200
	C	-7.17216800	-0.49150200	0.69714000
	C	-5.91371500	0.07927900	0.52452600
	C	-3.45793900	1.53025500	-0.40088800
	C	-4.40028200	2.13111300	-1.24577300
	C	-4.35327500	3.49693900	-1.51222100
	C	-3.35343500	4.28820800	-0.95228900
	C	-2.39190600	3.69656300	-0.13722700
	C	-2.43819200	2.32948000	0.12816500
	H	-3.60701200	1.06415800	2.31495100
	H	-2.68493700	0.20554300	4.41848400
	H	-1.30703000	-1.86458600	4.43319000
	H	-0.88812600	-3.06099400	2.29372600
	H	-1.81023200	-2.20921200	0.19764300
	H	-4.40603900	-2.45760000	-1.13541700
	H	-6.61391100	-3.46232200	-0.83002900
	H	-8.41620400	-2.21357400	0.33985600
	H	-7.94495600	0.06605200	1.21476500
	H	-5.73283800	1.07414600	0.91134300
	H	-5.17190500	1.52540100	-1.70360500
	H	-5.09815800	3.93957800	-2.16410300
	H	-3.31733000	5.35211300	-1.15791200
	H	-1.59503400	4.29475400	0.29029100
	H	-1.66731700	1.88470000	0.74295100
	H	0.33420900	-2.26796000	-1.98959600
	O	1.48286900	-0.00403100	-1.49309000
	C	2.63831800	0.67440200	-1.34213700
	C	3.46218200	0.14375400	-0.13928800
	C	2.89081000	1.02374200	1.00803400

	C	1.61836400	0.76284900	1.53570200
	O	2.92238300	1.63222900	-2.01586400
	C	3.56976600	2.16415700	1.44945700
	C	3.01605500	2.99297700	2.42352200
	C	1.76811600	2.70400500	2.96690300
	C	1.06817900	1.58748700	2.51371800
	C	4.98890800	0.33628000	-0.32077100
	C	5.59989600	0.69324300	-1.52594400
	C	6.99070200	0.76261200	-1.62796000
	C	7.79788900	0.48096200	-0.53284200
	C	7.20070300	0.12103800	0.67450600
	C	5.81706100	0.04496400	0.77390100
	C	3.26238800	-1.37700000	0.06330400
	C	3.08415300	-1.95476400	1.32196400
	C	3.01175900	-3.34045400	1.47162000
	C	3.12028000	-4.17535100	0.36535400
	C	3.31380400	-3.61154100	-0.89536200
	C	3.38926000	-2.23139200	-1.03985500
	H	1.04756700	-0.08396800	1.17837000
	H	4.53611200	2.41070800	1.03107400
	H	3.56529100	3.86790200	2.75317500
	H	1.34096400	3.34527600	3.72990100
	H	0.09062200	1.34938700	2.91733800
	H	5.00027100	0.94402500	-2.38721200
	H	7.43744600	1.04466600	-2.57506100
	H	8.87741700	0.53827100	-0.61555500
	H	7.81297500	-0.10735200	1.53991500
	H	5.37341200	-0.25009700	1.71708300
	H	2.99899100	-1.32717900	2.19916400
	H	2.87375100	-3.76244800	2.46108900
	H	3.06375400	-5.25175500	0.48146500
	H	3.41279100	-4.24843800	-1.76743300
	H	3.56104100	-1.81425400	-2.02590100
	H	0.24806300	1.51170600	-2.19329700
28	O	-1.58840400	-1.38368800	-1.13120800
	C	-1.95207800	-0.08939600	-1.01955500
	C	-0.49925900	-1.73506500	-2.03607300
	H	-0.13093100	-0.82165700	-2.49832500
	C	0.59632100	-2.38627000	-1.20285200
	H	0.15273500	-3.03846600	-0.45083200
	C	-1.05028900	-2.68724700	-3.08900300
	H	-0.27135500	-2.92781000	-3.81611200
	H	-1.88187000	-2.22196500	-3.62055100
	O	-1.40583300	0.80106200	-1.61808000
	C	-3.18191600	0.07763900	-0.06119200
	C	-3.22866500	1.53838800	0.45887700
	C	-3.40127700	1.82030200	1.81776600
	C	-3.48382000	3.13465800	2.28002000
	C	-3.39643600	4.19935900	1.39116100
	C	-3.23938700	3.93475800	0.03146400
	C	-3.16349900	2.62447000	-0.42962000
	C	-3.04505300	-0.90762100	1.12280600

	C	-1.81814700	-1.02069800	1.79185400
	C	-1.68131300	-1.84711300	2.90145100
	C	-2.77238400	-2.57679700	3.37361100
	C	-3.99680700	-2.46484100	2.72489800
	C	-4.13140400	-1.63679700	1.60904200
	C	-4.45069400	-0.24607800	-0.90939300
	C	-4.53854900	-1.44587400	-1.63182700
	C	-5.67826000	-1.76375000	-2.36415100
	C	-6.76796600	-0.89623400	-2.38551000
	C	-6.70328900	0.28637600	-1.65775300
	C	-5.55841900	0.60758900	-0.92827500
	H	-3.47301500	1.01355200	2.53335400
	H	-3.61703600	3.31726300	3.34065000
	H	-3.45583100	5.22140300	1.74853600
	H	-3.17825600	4.75202000	-0.67876500
	H	-3.04646100	2.44708900	-1.48786000
	H	-0.95905900	-0.45348700	1.45206300
	H	-0.72060900	-1.91603400	3.39945200
	H	-2.66681900	-3.22160800	4.23894000
	H	-4.85629900	-3.02107500	3.08260800
	H	-5.09450500	-1.56218500	1.12153900
	H	-3.71577300	-2.14574700	-1.60558200
	H	-5.71447300	-2.69731500	-2.91497800
	H	-7.65601700	-1.14316400	-2.95629300
	H	-7.54522000	0.96977000	-1.65135200
	H	-5.53899400	1.53157200	-0.36808300
	H	-1.40517300	-3.61731100	-2.63621200
	O	1.37980500	-1.43005000	-0.45305800
	C	2.41096600	-0.86217200	-1.12738300
	C	3.33142800	-0.00702400	-0.19805600
	C	2.46995200	0.74498500	0.84050200
	C	2.83853500	0.83145000	2.18419600
	O	2.58896900	-1.06290000	-2.30064800
	C	1.32665800	1.43799400	0.41985800
	C	0.57013900	2.18508100	1.31649400
	C	0.94742600	2.26125800	2.65743600
	C	2.08366100	1.58409400	3.08567000
	C	4.13142500	0.99743600	-1.06968200
	C	4.87612000	0.55394200	-2.17476000
	C	5.62684900	1.44179500	-2.93711800
	C	5.66519100	2.79672900	-2.61219600
	C	4.94720100	3.24777500	-1.51184400
	C	4.19018000	2.35708000	-0.74915100
	C	4.29560900	-1.01135500	0.50782100
	C	5.66962900	-0.76223600	0.59418000
	C	6.51921900	-1.63845900	1.26906400
	C	6.01546100	-2.78529500	1.87266700
	C	4.64777500	-3.03970200	1.80614500
	C	3.79881000	-2.16192000	1.13850700
	H	3.72091400	0.31462400	2.53779500
	H	1.01516700	1.40047800	-0.61787300
	H	-0.31614400	2.70167800	0.96757500

	H	0.35692900	2.84201600	3.35688500
	H	2.39109700	1.63632500	4.12445800
	H	4.86665400	-0.49135400	-2.44462100
	H	6.18578300	1.07016800	-3.78897100
	H	6.25050400	3.48797400	-3.20839500
	H	4.96842100	4.29665700	-1.23739500
	H	3.64469000	2.73771200	0.10266400
	H	6.08770200	0.12339800	0.13702600
	H	7.57957100	-1.41673300	1.31859500
	H	6.67700100	-3.46912900	2.39247700
	H	4.23447700	-3.92323300	2.28028900
	H	2.73703800	-2.36367500	1.12447100
	H	1.26099400	-2.95443300	-1.85379900
32	O	-1.38123200	-1.44605100	-0.97605600
	C	-2.27759900	-0.52244000	-1.37399400
	C	-0.65570300	-2.15362800	-2.02205400
	H	-1.33772800	-2.28444800	-2.86625800
	C	0.50545400	-1.31203500	-2.53563000
	H	0.96978500	-1.82855000	-3.37914200
	C	-0.24719300	-3.49571600	-1.43782200
	H	0.26615600	-4.08990100	-2.19858300
	H	-1.13017300	-4.04724000	-1.11031600
	O	-2.43093400	-0.22311300	-2.53261100
	C	-3.12294500	0.08283200	-0.21049400
	C	-4.53394900	-0.58206600	-0.28286700
	C	-5.27423500	-0.81169200	0.88423600
	C	-6.56310900	-1.33836700	0.82852200
	C	-7.14441100	-1.64932200	-0.39680200
	C	-6.42654100	-1.41110200	-1.56511700
	C	-5.14147300	-0.87617500	-1.51090900
	C	-3.25064800	1.60288600	-0.48640900
	C	-4.44293700	2.29135900	-0.25162800
	C	-4.52548700	3.66978300	-0.44877000
	C	-3.41643700	4.38482700	-0.88710800
	C	-2.22084000	3.70879300	-1.12292500
	C	-2.13793700	2.33433600	-0.92397000
	C	-2.46015800	-0.18688700	1.16547200
	C	-1.94161700	0.84116700	1.95578200
	C	-1.36287800	0.57526000	3.19913800
	C	-1.30131400	-0.72477600	3.68323600
	C	-1.81962400	-1.76302400	2.90765800
	C	-2.38553900	-1.49644500	1.66739800
	H	-4.84737100	-0.57496900	1.84935100
	H	-7.11035100	-1.50366900	1.75013000
	H	-8.14503700	-2.06454200	-0.44084200
	H	-6.86740200	-1.63482000	-2.53036700
	H	-4.61582100	-0.67115400	-2.43295700
	H	-5.32180300	1.75585100	0.08160700
	H	-5.46462100	4.17920300	-0.26258500
	H	-3.48159000	5.45550200	-1.04618400
	H	-1.34518800	4.24949400	-1.46430400
	H	-1.19120200	1.84023800	-1.10580000

	H	-1.98334000	1.86410700	1.60981900
	H	-0.96270300	1.39581200	3.78359100
	H	-0.85822900	-0.93118100	4.65106500
	H	-1.78728700	-2.78433000	3.27186300
	H	-2.78359700	-2.31576000	1.08355700
	H	0.42518000	-3.36595200	-0.58959600
	O	1.54517400	-1.16936100	-1.54089200
	C	1.75432200	0.05843200	-1.01361200
	C	3.02326900	0.08435400	-0.09880700
	C	2.93779000	1.31737400	0.83821200
	C	3.11436800	1.20346600	2.21998300
	O	1.05932400	1.00655700	-1.26926200
	C	2.74319400	2.60302100	0.30775300
	C	2.70296600	3.72142400	1.13237000
	C	2.86722400	3.59015700	2.51062600
	C	3.07834800	2.32674100	3.04821800
	C	4.26069400	0.20373800	-1.04236800
	C	4.39734000	-0.62278600	-2.16752700
	C	5.52169800	-0.54654500	-2.98452100
	C	6.54742200	0.34867500	-2.69238500
	C	6.43603300	1.15918600	-1.56799200
	C	5.30659100	1.08765500	-0.75339900
	C	3.08556100	-1.22382300	0.72210400
	C	4.27002400	-1.94495700	0.88067700
	C	4.31001900	-3.09036500	1.67742100
	C	3.16517400	-3.53349600	2.32953200
	C	1.97727400	-2.81701500	2.18418600
	C	1.93947700	-1.67507700	1.39172300
	H	3.28523900	0.23445800	2.66723400
	H	2.62195100	2.73144900	-0.75766400
	H	2.54471200	4.70051100	0.69374400
	H	2.83591800	4.46340900	3.15273100
	H	3.21822000	2.20354400	4.11653700
	H	3.62920900	-1.34732200	-2.39685600
	H	5.59534500	-1.19509200	-3.85062100
	H	7.42268500	0.40883900	-3.32933100
	H	7.22873400	1.85496000	-1.31613900
	H	5.24769000	1.72745400	0.11577100
	H	5.17348600	-1.61786100	0.38341600
	H	5.24273500	-3.63333200	1.78362000
	H	3.19533000	-4.42468800	2.94647100
	H	1.07518300	-3.14177500	2.68978900
	H	1.00706500	-1.12788900	1.30689600
	H	0.16016000	-0.33097700	-2.85190700
34	O	-1.55591200	1.64017800	-0.84698700
	C	-1.73275900	0.83091800	0.21510000
	C	-0.54946200	2.68894700	-0.76949700
	H	-0.19246100	2.74646000	0.25697200
	C	0.59994700	2.32615200	-1.69818400
	H	0.21671300	2.04300600	-2.67861100
	C	-1.22143500	3.98831100	-1.19083600
	H	-0.52310800	4.82301600	-1.09334800

	H	-2.08532900	4.18442100	-0.55446500	
	O	-1.03824800	0.85033000	1.19968600	
	C	-3.02659900	-0.01698000	0.05434100	
	C	-3.14644300	-0.67230200	-1.33991700	
	C	-4.39150200	-1.12340400	-1.79616700	
	C	-4.51153200	-1.81126700	-2.99982800	
	C	-3.38309300	-2.07891700	-3.77123400	
	C	-2.13699900	-1.65878600	-3.31732900	
	C	-2.01919000	-0.96607400	-2.11379200	
	C	-4.10503700	1.06141600	0.34974000	
	C	-4.31919900	1.47128400	1.67302900	
	C	-5.21114100	2.49788800	1.96953600	
	C	-5.90072700	3.14598000	0.94723100	
	C	-5.67487700	2.76725500	-0.37309500	
	C	-4.77828800	1.74212300	-0.66976200	
	C	-3.05901700	-1.18983000	1.06152100	
	C	-4.26902100	-1.62702600	1.61137200	
	C	-4.32273600	-2.75366300	2.42930500	
	C	-3.16454300	-3.47099300	2.71171300	
	C	-1.95642800	-3.05364300	2.15886200	
	C	-1.90439900	-1.93070100	1.33858000	
	H	-5.27998200	-0.94200900	-1.20419200	
	H	-5.48939800	-2.14425800	-3.32988500	
	H	-3.47421200	-2.61608800	-4.70858000	
	H	-1.24492600	-1.87136100	-3.89562200	
	H	-1.03280400	-0.66469500	-1.78587800	
	H	-3.77917500	0.98558200	2.47615200	
	H	-5.36316300	2.79220600	3.00199400	
	H	-6.59943200	3.94255200	1.17705400	
	H	-6.19289100	3.27273000	-1.18072100	
	H	-4.60074900	1.47612900	-1.70283300	
	H	-5.18346300	-1.08466000	1.40700600	
	H	-5.27436800	-3.06745600	2.84412000	
	H	-3.20270900	-4.34555800	3.35148300	
	H	-1.04456300	-3.60335700	2.36371100	
	H	-0.95272700	-1.63743400	0.91784200	
	H	-1.56553600	3.93590600	-2.22716300	
	O	1.34585000	1.17401000	-1.24831100	
	C	2.28328500	1.40638900	-0.30594700	
	C	3.16381600	0.16847900	0.04535500	
	C	3.34457000	0.17615500	1.58435100	
	C	2.24428800	0.42409100	2.41663000	
	O	2.44184900	2.49510000	0.18749400	
	C	4.57099800	-0.12605200	2.17923600	
	C	4.69936500	-0.17724900	3.56747400	
	C	3.60278900	0.07533000	4.38434300	
	C	2.37299000	0.37575500	3.80097100	
	C	4.54413200	0.39125400	-0.65268000	
	C	5.14543000	1.65740400	-0.68050000	
	C	6.40434500	1.84044200	-1.24656900	
	C	7.10251500	0.76250500	-1.78435200	
	C	6.52776300	-0.50339700	-1.74199300	

	C	5.26358500	-0.68651800	-1.18333900
	C	2.52367700	-1.15997200	-0.43326400
	C	2.20731300	-2.18983800	0.45636800
	C	1.67175300	-3.39808300	0.00309100
	C	1.44501600	-3.60414400	-1.35125600
	C	1.76598200	-2.58961300	-2.25349800
	C	2.29866600	-1.38895100	-1.80084100
	H	1.27265400	0.64974900	1.99247900
	H	5.43856500	-0.32140400	1.56304100
	H	5.66359500	-0.41093700	4.00537800
	H	3.70338600	0.04123500	5.46349300
	H	1.50676700	0.57384100	4.42227700
	H	4.63372600	2.50397000	-0.24440700
	H	6.84120300	2.83297000	-1.25904100
	H	8.08328500	0.90653300	-2.22361900
	H	7.06010600	-1.35866400	-2.14349700
	H	4.84306800	-1.68239900	-1.15876800
	H	2.38161300	-2.06159900	1.51534600
	H	1.43892200	-4.17800500	0.71953500
	H	1.02770600	-4.54050100	-1.70362200
	H	1.60756100	-2.73699100	-3.31628900
	H	2.55123200	-0.62131900	-2.51978200
	H	1.27578200	3.17739700	-1.78819800
35	O	-1.54594500	-0.49632600	-1.48893600
	C	-2.61083300	0.32877700	-1.46147500
	C	-0.59741200	-0.32668000	-2.58247600
	H	-1.15384400	0.04885000	-3.44439900
	C	0.42297100	0.75063400	-2.23725400
	H	1.03048600	0.96394300	-3.11747900
	C	-0.01761300	-1.70046100	-2.87088000
	H	0.73369400	-1.63286700	-3.65965400
	H	-0.80769400	-2.38277800	-3.18862100
	O	-2.80034800	1.19023300	-2.28476200
	C	-3.47961000	0.11288800	-0.19288300
	C	-2.83214200	1.14790100	0.76915700
	C	-1.59607500	0.87783700	1.37211600
	C	-0.96968900	1.82898000	2.17383600
	C	-1.55543600	3.07767800	2.37084000
	C	-2.76543800	3.36927000	1.74779200
	C	-3.39442200	2.41632800	0.94930500
	C	-3.42350300	-1.34812500	0.31206700
	C	-3.60504000	-2.39477000	-0.60120900
	C	-3.66232100	-3.71754700	-0.17985100
	C	-3.55145900	-4.02805800	1.17497400
	C	-3.39067100	-2.99838900	2.09517400
	C	-3.32878700	-1.67144100	1.66757200
	C	-4.98112000	0.40209600	-0.44332400
	C	-5.55249300	0.53906900	-1.71142900
	C	-6.93014100	0.71001100	-1.85911000
	C	-7.76343900	0.74937900	-0.74800000
	C	-7.20639900	0.61039900	0.52236100
	C	-5.83605200	0.43346600	0.66860400

	H -1.11121100 -0.07612600 1.21330300<br>H -0.02187500 1.58471700 2.63957300<br>H -1.06960600 3.81775400 2.99708000<br>H -3.22522400 4.34275700 1.87751700<br>H -4.32816100 2.66489200 0.46341400<br>H -3.71262100 -2.17477500 -1.65708700<br>H -3.79938800 -4.50758400 -0.90993700<br>H -3.59793100 -5.05916600 1.50667000<br>H -3.31518300 -3.22075200 3.15397900<br>H -3.20548700 -0.88796600 2.40370500<br>H -4.92977500 0.54006600 -2.59299900<br>H -7.34584100 0.81694000 -2.85505400<br>H -8.83261300 0.88451800 -0.86673000<br>H -7.84004400 0.63342600 1.40209400<br>H -5.42487500 0.31479000 1.66375200<br>H 0.45223000 -2.11733300 -1.97899200<br>O 1.29281000 0.38327300 -1.14151900<br>C 2.55885300 0.02507200 -1.44431300<br>C 3.46794800 0.01166700 -0.18596100<br>C 4.64063500 -0.99150400 -0.31168000<br>C 5.69027100 -0.90459700 0.61405800<br>O 2.94927500 -0.14764100 -2.57114300<br>C 4.66059000 -2.04879200 -1.22570200<br>C 5.70060300 -2.97919900 -1.21934000<br>C 6.74030200 -2.87394000 -0.30304300<br>C 6.72944800 -1.82744300 0.61743400<br>C 2.71278000 -0.40245200 1.09743800<br>C 1.84131700 -1.49776900 1.06985900<br>C 1.23074100 -1.96403900 2.22901500<br>C 1.49107900 -1.35182600 3.45386700<br>C 2.37029500 -0.27480600 3.50076000<br>C 2.97436400 0.19426700 2.33428600<br>C 3.94241600 1.49253600 -0.19018300<br>C 3.09508300 2.50849800 0.27398800<br>C 3.47112900 3.84604300 0.18973400<br>C 4.69486500 4.19805000 -0.37526800<br>C 5.53035400 3.19992700 -0.86720200<br>C 5.15443200 1.86075000 -0.78340300<br>H 5.69541700 -0.10537500 1.34504000<br>H 3.88004500 -2.14478600 -1.96509600<br>H 5.69231400 -3.78720100 -1.94258700<br>H 7.54821500 -3.59690600 -0.30269400<br>H 7.52884300 -1.73092300 1.34377700<br>H 1.63678400 -2.00187400 0.13316100<br>H 0.55090100 -2.80666200 2.17371600<br>H 1.01790300 -1.71437900 4.35929600<br>H 2.59330800 0.20593000 4.44703600<br>H 3.65483800 1.03359400 2.39732300<br>H 2.13115500 2.25325100 0.69466600<br>H 2.80108900 4.61322900 0.56157700<br>H 4.98816200 5.23976300 -0.44073700<br>H 6.47785000 3.45954000 -1.32602600

	H	5.80908900	1.10016100	-1.18689900
	H	-0.08957900	1.65490000	-1.91233200
36	O	-1.47866400	-1.44413700	-0.43927300
	C	-2.29728200	-0.56505800	-1.05260200
	C	-0.61007300	-2.24584300	-1.28799100
	H	-0.32210000	-1.63570500	-2.14467300
	C	0.61327300	-2.59445200	-0.46627500
	H	0.32338300	-2.96997400	0.51817100
	C	-1.36148500	-3.49099300	-1.74550800
	H	-1.65665800	-4.10581200	-0.89166200
	H	-0.73361500	-4.09003000	-2.41003800
	O	-2.28167300	-0.36077900	-2.23955300
	C	-3.33614900	0.03047300	-0.06082200
	C	-2.71160500	0.53446800	1.25979900
	C	-3.54870000	0.81472100	2.34857600
	C	-3.04653400	1.37119300	3.51984300
	C	-1.69116600	1.67690300	3.62563100
	C	-0.85211800	1.42110000	2.54639400
	C	-1.35604300	0.85548100	1.37496300
	C	-4.28903900	-1.18308300	0.11668100
	C	-5.15188000	-1.53601600	-0.93045500
	C	-5.95421000	-2.67027100	-0.84596700
	C	-5.90272000	-3.48385100	0.28389100
	C	-5.03100800	-3.15761400	1.31872000
	C	-4.22526900	-2.02348400	1.23219200
	C	-4.03473700	1.26365200	-0.68038400
	C	-5.39095400	1.51846000	-0.45401900
	C	-5.99076700	2.68850700	-0.91708100
	C	-5.24320500	3.63099900	-1.61490000
	C	-3.88842100	3.39397600	-1.83685100
	C	-3.29027600	2.22776700	-1.37096800
	H	-4.60862400	0.60321900	2.27908500
	H	-3.71830700	1.57392100	4.34669900
	H	-1.29701400	2.11572900	4.53540700
	H	0.20335900	1.65941300	2.60654200
	H	-0.67200500	0.66571000	0.55888200
	H	-5.19153100	-0.91942300	-1.82000800
	H	-6.61816600	-2.91800500	-1.66669100
	H	-6.52980300	-4.36560900	0.35310900
	H	-4.97036100	-3.78860200	2.19864900
	H	-3.54111100	-1.79647300	2.03851500
	H	-5.99398200	0.79880900	0.08483000
	H	-7.04527900	2.85819600	-0.72917400
	H	-5.70816400	4.54011300	-1.97941800
	H	-3.29086200	4.12082900	-2.37599200
	H	-2.23592000	2.07042100	-1.55364200
	H	-2.26010900	-3.20443800	-2.29236600
	O	1.41990200	-1.41056200	-0.27290300
	C	2.76231300	-1.54834100	-0.39523400
	C	3.50793600	-0.19795100	-0.13832600
	C	3.67848700	-0.04867700	1.40301400
	C	4.84204300	0.51879300	1.93660500

	O	3.28815300	-2.59961700	-0.64747700
	C	2.65781800	-0.40846700	2.29335900
	C	2.80545000	-0.22663200	3.66684400
	C	3.97121000	0.33147700	4.18360200
	C	4.98597400	0.70902800	3.30961200
	C	2.66384800	0.96082200	-0.71556300
	C	2.16111500	0.86369000	-2.02100200
	C	1.42788400	1.89806900	-2.58960200
	C	1.18642400	3.06555400	-1.86453400
	C	1.68897100	3.18044700	-0.57369200
	C	2.42080400	2.13613200	-0.00414600
	C	4.90040900	-0.26142000	-0.82048100
	C	5.34327900	0.74293400	-1.68597300
	C	6.61782600	0.69588400	-2.25381300
	C	7.47711800	-0.35653000	-1.96456400
	C	7.05414900	-1.35790500	-1.09148000
	C	5.78688500	-1.30870200	-0.52264700
	H	5.64578400	0.81767000	1.27773100
	H	1.73009900	-0.81711400	1.91810100
	H	1.99976600	-0.52031300	4.33042100
	H	4.08604200	0.47204100	5.25246900
	H	5.89887800	1.15192300	3.69215700
	H	2.35227400	-0.02993800	-2.60556800
	H	1.04244700	1.79230400	-3.59733000
	H	0.61418000	3.87427300	-2.30460500
	H	1.51049200	4.08177800	0.00185200
	H	2.80156100	2.24677300	1.00260200
	H	4.69950800	1.57840600	-1.92250000
	H	6.93144500	1.48998600	-2.92237500
	H	8.46627000	-0.39624600	-2.40702400
	H	7.71490400	-2.18268300	-0.84829400
	H	5.48393900	-2.09179200	0.15648800
	H	1.21007800	-3.35002300	-0.97577000
42	O	-1.43177600	-1.31192400	-0.82108600
	C	-2.41746100	-0.54196600	-1.34103800
	C	-0.65259900	-2.09820800	-1.77173200
	H	-1.30847000	-2.33476400	-2.61251200
	C	0.49267800	-1.27339000	-2.34293800
	H	0.92268400	-1.80733500	-3.19475200
	C	-0.21607000	-3.36288600	-1.05026700
	H	-1.08882800	-3.93186400	-0.72535800
	H	0.40108800	-3.12642000	-0.18356500
	O	-2.56985900	-0.41281200	-2.52821800
	C	-3.32740100	0.08782100	-0.23395000
	C	-4.30268400	-1.03520400	0.23977100
	C	-5.68236200	-0.81914400	0.32415500
	C	-6.54122100	-1.81437500	0.79110600
	C	-6.04172000	-3.05016800	1.18555100
	C	-4.66853700	-3.27513100	1.12191900
	C	-3.81122900	-2.28047500	0.66145300
	C	-4.11648700	1.27626800	-0.84483800
	C	-4.85292400	1.11903600	-2.03037100

	C	-5.59639000	2.16872300	-2.55838600
	C	-5.63475500	3.40359800	-1.91317200
	C	-4.92477000	3.56984400	-0.73071300
	C	-4.17563800	2.51722900	-0.20304900
	C	-2.44483900	0.57877800	0.93514500
	C	-1.28904800	1.32279300	0.66225100
	C	-0.51406000	1.84752600	1.69113800
	C	-0.88362700	1.64293500	3.02073300
	C	-2.03189000	0.91157100	3.30427900
	C	-2.80607500	0.38374400	2.26951500
	H	-6.09983600	0.13315600	0.02973100
	H	-7.60560000	-1.61378800	0.84423600
	H	-6.70993400	-3.82504400	1.54409800
	H	-4.25770000	-4.22780800	1.43826300
	H	-2.74718300	-2.46649000	0.64796700
	H	-4.84392300	0.17152300	-2.54752100
	H	-6.14918700	2.01807400	-3.47918500
	H	-6.21386400	4.22162200	-2.32711300
	H	-4.94617700	4.52026900	-0.20902500
	H	-3.63607700	2.67649800	0.71968900
	H	-0.98296000	1.50294500	-0.36197700
	H	0.37996800	2.41125900	1.45176600
	H	-0.27932400	2.05076000	3.82311000
	H	-2.33439300	0.74810700	4.33292800
	H	-3.69859800	-0.17797700	2.51161600
	H	0.36833400	-3.99208900	-1.72691300
	O	1.56145500	-1.12212500	-1.38115100
	C	1.95011800	0.12828800	-1.04322000
	C	3.22454600	0.10195900	-0.13430000
	C	3.11143100	-1.06835200	0.87003100
	C	4.18848300	-1.90967000	1.15330500
	O	1.39072800	1.11732200	-1.43757300
	C	1.91926900	-1.25379500	1.58426900
	C	1.80690600	-2.25505400	2.54261800
	C	2.88847000	-3.09361800	2.81284600
	C	4.07811600	-2.91429000	2.11633400
	C	3.33444200	1.45465900	0.61638300
	C	3.29317000	2.66876300	-0.08843400
	C	3.42215300	3.88606600	0.57136300
	C	3.60987100	3.92605700	1.95235700
	C	3.67371700	2.73213700	2.66048500
	C	3.53811100	1.51058500	1.99846200
	C	4.45041000	-0.09714000	-1.07918300
	C	5.61319200	0.66643200	-0.92626800
	C	6.72760600	0.45287100	-1.73689300
	C	6.70615100	-0.52862100	-2.72161700
	C	5.56082200	-1.30525600	-2.87722300
	C	4.45114200	-1.09713200	-2.06318800
	H	5.12468900	-1.78617400	0.62515200
	H	1.06975400	-0.60544000	1.40129600
	H	0.87319200	-2.37583000	3.08046400
	H	2.80233200	-3.87514000	3.55948800

	H	4.92932400	-3.55541600	2.31739600
	H	3.15454700	2.66363300	-1.15921400
	H	3.37836500	4.80728300	0.00091000
	H	3.71114300	4.87609800	2.46514900
	H	3.83064900	2.74057600	3.73347800
	H	3.59402100	0.59814900	2.57510700
	H	5.65914500	1.43464300	-0.16729800
	H	7.61331800	1.06160000	-1.59254200
	H	7.57030300	-0.69038400	-3.35598900
	H	5.52865100	-2.08271400	-3.63265200
	H	3.58523100	-1.73255400	-2.18120400
	H	0.14683300	-0.29547800	-2.66894200
43	O	-1.56267300	1.18378800	-1.22144900
	C	-1.86126300	0.80430000	0.03660400
	C	-0.59719100	2.25399100	-1.42720500
	H	-0.28338000	2.61781000	-0.44882900
	C	0.60096300	1.69269600	-2.17044700
	H	0.27495200	1.09280000	-3.02374600
	C	-1.29762100	3.34562000	-2.22612200
	H	-0.63209100	4.20155300	-2.36254800
	H	-2.19056200	3.68162800	-1.69745500
	O	-1.29301200	1.20967500	1.01840100
	C	-3.12602300	-0.10109000	0.05729500
	C	-4.23258100	0.97418800	-0.12435000
	C	-4.79926900	1.25347800	-1.37231600
	C	-5.72223100	2.28694700	-1.52298900
	C	-6.08120800	3.07534600	-0.43342900
	C	-5.49804600	2.82896900	0.80750900
	C	-4.57950600	1.79411800	0.95839200
	C	-3.24033400	-0.86668200	1.39547900
	C	-2.10849200	-1.42986300	1.99612800
	C	-2.22305400	-2.21398900	3.13987800
	C	-3.47143400	-2.46247700	3.70512800
	C	-4.60595200	-1.92260800	3.10771800
	C	-4.48998000	-1.13558600	1.96373700
	C	-3.11782900	-1.19115700	-1.03767700
	C	-4.31648100	-1.80882000	-1.41676400
	C	-4.32850300	-2.86251700	-2.32554200
	C	-3.13594000	-3.33525200	-2.86792600
	C	-1.93593300	-2.74799300	-2.47991700
	C	-1.92599900	-1.69048700	-1.57233600
	H	-4.51751100	0.66521600	-2.23497900
	H	-6.15578600	2.47653900	-2.49874900
	H	-6.80017000	3.87834200	-0.55104000
	H	-5.75431200	3.44481100	1.66239700
	H	-4.12132000	1.62417700	1.92456200
	H	-1.12607800	-1.26144000	1.57664100
	H	-1.32867200	-2.63208100	3.58810000
	H	-3.55847300	-3.07237600	4.59735300
	H	-5.58734100	-2.11179500	3.52865000
	H	-5.38693500	-0.72649400	1.51625700
	H	-5.25421200	-1.46848300	-0.99534200

	H	-5.27262500	-3.31768200	-2.60379200
	H	-3.14238500	-4.15609800	-3.57614000
	H	-0.99665700	-3.11145000	-2.88174400
	H	-0.97420400	-1.26406300	-1.28445900
	H	-1.60144500	2.97801600	-3.20954600
	O	1.35978200	0.84132900	-1.28520300
	C	2.70641700	0.96861700	-1.31082500
	C	3.37557800	0.20709700	-0.13763400
	C	2.61060400	-1.08282300	0.23576500
	C	2.41407900	-1.47918800	1.56048900
	O	3.28609400	1.67691500	-2.09363100
	C	2.22069500	-1.96551300	-0.77979900
	C	1.63379000	-3.19011700	-0.48282800
	C	1.43092900	-3.56883400	0.84357700
	C	1.82898100	-2.70966000	1.86172300
	C	3.34554800	1.31061700	0.95822600
	C	4.46229800	2.11829900	1.19865500
	C	4.40349300	3.16101000	2.12124000
	C	3.22288800	3.42730700	2.80844600
	C	2.09482300	2.65104900	2.55244100
	C	2.15191900	1.60867900	1.63103300
	C	4.81497400	-0.25813100	-0.47087900
	C	5.60738000	-0.74771200	0.57806600
	C	6.87777300	-1.26070300	0.34585300
	C	7.38792200	-1.30895100	-0.95052600
	C	6.60957600	-0.83796400	-2.00088500
	C	5.33607300	-0.31698100	-1.76649300
	H	2.71670500	-0.82956600	2.37117000
	H	2.38887300	-1.70000600	-1.81766500
	H	1.33852400	-3.85375700	-1.28777000
	H	0.97140200	-4.52232900	1.07668700
	H	1.69049700	-2.99331000	2.89926400
	H	5.38377600	1.93718000	0.66204500
	H	5.28483600	3.76830300	2.29527700
	H	3.17852700	4.23799200	3.52719300
	H	1.15976900	2.85438900	3.06202400
	H	1.25343200	1.04051600	1.43105100
	H	5.22509100	-0.73040100	1.59148000
	H	7.46708200	-1.62804400	1.17873800
	H	8.37787100	-1.71019600	-1.13626600
	H	6.98989600	-0.86803200	-3.01606400
	H	4.76703600	0.06277300	-2.60123100
	H	1.24336800	2.49854100	-2.52542200
45	O	-1.53489600	-0.58319600	-1.38747700
	C	-2.56528200	0.28961000	-1.38524200
	C	-0.59396000	-0.50109800	-2.49818400
	H	-1.15876200	-0.18930900	-3.38024000
	C	0.44126500	0.59057700	-2.25891800
	H	1.03725200	0.71885200	-3.16292900
	C	-0.02885000	-1.89823500	-2.68608400
	H	0.70919000	-1.89909600	-3.49029700
	H	-0.82974600	-2.59635400	-2.93370400

	O	-2.69130500	1.14372800	-2.22541600
	C	-3.52454200	0.05450700	-0.17119700
	C	-2.90058300	0.77114400	1.06354100
	C	-3.70852200	1.47218700	1.96702300
	C	-3.16593000	2.06779400	3.10443200
	C	-1.80237600	1.97659600	3.36520600
	C	-0.98855000	1.27346300	2.48107300
	C	-1.53099600	0.67465600	1.34623200
	C	-3.64747900	-1.46698400	0.07468700
	C	-3.93633000	-2.31585300	-1.00342700
	C	-4.09943300	-3.68327800	-0.81925600
	C	-3.98225200	-4.23727400	0.45599900
	C	-3.70428300	-3.40598400	1.53450500
	C	-3.53759600	-2.03226500	1.34497500
	C	-4.91013800	0.67038000	-0.50012300
	C	-6.09429000	-0.05329700	-0.33014400
	C	-7.33888600	0.52814300	-0.57843900
	C	-7.42576000	1.84895400	-0.99987200
	C	-6.25435600	2.58785900	-1.15930900
	C	-5.01534200	2.00997100	-0.90724700
	H	-4.77156800	1.55584400	1.78838900
	H	-3.81765000	2.60396700	3.78555700
	H	-1.37992200	2.44187800	4.24888200
	H	0.07292900	1.17059800	2.67479800
	H	-0.87719600	0.11492600	0.69304100
	H	-4.04840300	-1.89976100	-1.99920200
	H	-4.32099900	-4.31760600	-1.67051600
	H	-4.10867100	-5.30400500	0.60291800
	H	-3.61452200	-3.82047000	2.53261000
	H	-3.31950500	-1.40376900	2.19809900
	H	-6.05958400	-1.08052700	0.00465500
	H	-8.23824900	-0.06124500	-0.43767400
	H	-8.39137100	2.30137400	-1.19665300
	H	-6.30294200	3.62281400	-1.47960700
	H	-4.12323200	2.60556000	-1.03046400
	H	0.45375800	-2.24757500	-1.77223100
	O	1.32708900	0.30924900	-1.14888700
	C	2.59890000	-0.04227300	-1.44303200
	C	3.52839300	0.07946900	-0.20527900
	C	3.84941400	1.59901900	-0.26988200
	C	2.99527900	2.54274400	0.31492200
	O	2.97519200	-0.29948400	-2.55785700
	C	4.91766100	2.06412500	-1.04612200
	C	5.15460400	3.42897100	-1.19077000
	C	4.31904100	4.35705200	-0.57550200
	C	3.23350600	3.90727400	0.17186200
	C	4.78923800	-0.80794900	-0.33771700
	C	4.80040800	-1.99319200	-1.07983300
	C	5.91749200	-2.82687100	-1.07887300
	C	7.04526200	-2.49831700	-0.33411200
	C	7.04370500	-1.32560600	0.41727200
	C	5.92741100	-0.49562700	0.41694200

	C	2.85674300	-0.37197600	1.11041800
	C	3.20873300	0.19937400	2.33817000
	C	2.70251100	-0.30218300	3.53581300
	C	1.83328600	-1.38927600	3.53236500
	C	1.48329100	-1.97493200	2.31838200
	C	1.99546000	-1.47449600	1.12522000
	H	2.13267100	2.21213800	0.87781100
	H	5.56613400	1.35607100	-1.54513500
	H	5.99256600	3.76396400	-1.79178200
	H	4.50480800	5.41943800	-0.68627400
	H	2.56270400	4.61804600	0.64150000
	H	3.94627100	-2.26937100	-1.68058000
	H	5.90116500	-3.73661400	-1.66885700
	H	7.91371100	-3.14730200	-0.33703000
	H	7.91126800	-1.05487500	1.00884400
	H	5.94590900	0.40864100	1.01244600
	H	3.88673600	1.04266600	2.36745400
	H	2.99352600	0.15980800	4.47283900
	H	1.43515900	-1.77695800	4.46304700
	H	0.80778400	-2.82248900	2.29579300
	H	1.72079100	-1.95907900	0.19663800
	H	-0.05586000	1.52605200	-2.00789100
46	O	-1.44827700	-0.98385000	-0.93722100
	C	-2.48598000	-0.22107400	-1.33568600
	C	-0.56901700	-1.50604000	-1.97749700
	H	-1.18160700	-1.66947600	-2.86791700
	C	0.47725300	-0.47221200	-2.37274700
	H	1.05579100	-0.85290600	-3.21473100
	C	-0.01132900	-2.81992200	-1.45782400
	H	-0.82672300	-3.51613000	-1.25582600
	H	0.55319900	-2.67294100	-0.53611900
	O	-2.64327900	0.13751800	-2.47608400
	C	-3.50096900	0.00975700	-0.18240000
	C	-2.82956100	0.43342700	1.14297900
	C	-3.53773400	0.32279000	2.34672500
	C	-3.00901500	0.80353500	3.54031500
	C	-1.76056900	1.42169700	3.55724600
	C	-1.05297200	1.55424800	2.36680600
	C	-1.58272300	1.06547600	1.17338100
	C	-4.19166900	-1.38065700	-0.13069600
	C	-5.12246900	-1.72304100	-1.12129300
	C	-5.69388800	-2.99184100	-1.15866000
	C	-5.33711400	-3.95055300	-0.21329100
	C	-4.39235500	-3.63130600	0.75795200
	C	-3.81789600	-2.36199800	0.79303000
	C	-4.48236100	1.15576000	-0.52498100
	C	-5.80872100	1.11740400	-0.08168300
	C	-6.66767200	2.19536400	-0.28513000
	C	-6.21571100	3.33968500	-0.93456700
	C	-4.89415600	3.39560800	-1.37000800
	C	-4.03694500	2.31908400	-1.16317500
	H	-4.51774200	-0.13778100	2.35419500

	H	-3.57934900	0.70072800	4.45694000
	H	-1.34796900	1.79956200	4.48584800
	H	-0.08243600	2.03673000	2.35599900
	H	-1.00214200	1.18152200	0.26755600
	H	-5.39787000	-0.99198400	-1.87121100
	H	-6.41622000	-3.23012000	-1.93140000
	H	-5.78416800	-4.93797000	-0.23884300
	H	-4.09379000	-4.37228700	1.49128000
	H	-3.07160700	-2.13833400	1.54310200
	H	-6.18354900	0.23756300	0.42561300
	H	-7.69173500	2.13573700	0.06668800
	H	-6.88312700	4.17841200	-1.09745600
	H	-4.52446700	4.28131600	-1.87471100
	H	-3.01640000	2.38937900	-1.51268600
	H	0.65484000	-3.26080000	-2.20232000
	O	1.37963700	-0.13908900	-1.29195500
	C	2.65247900	-0.59397600	-1.37307400
	C	3.54855500	-0.01930700	-0.22425500
	C	5.03551500	-0.20691600	-0.62148700
	C	5.94823700	0.85039300	-0.57048800
	O	3.02084800	-1.34223200	-2.23966500
	C	5.52088300	-1.47336600	-0.98487900
	C	6.86035900	-1.66422500	-1.30317400
	C	7.75655400	-0.59707100	-1.25938300
	C	7.29444200	0.65874200	-0.88674000
	C	3.22730800	-0.80541400	1.08309100
	C	1.90280000	-0.98670500	1.50852400
	C	1.61471700	-1.63863200	2.70461400
	C	2.64244600	-2.11834000	3.51267000
	C	3.96063800	-1.93080100	3.11185500
	C	4.24986900	-1.28192500	1.91171800
	C	3.22140700	1.48271500	-0.05389900
	C	3.11174700	2.08097100	1.20225500
	C	2.87856300	3.45221900	1.32443800
	C	2.74990700	4.24859500	0.19193300
	C	2.86589500	3.66414600	-1.06895800
	C	3.10208300	2.29928700	-1.18692600
	H	5.61909200	1.83825500	-0.27997100
	H	4.84776500	-2.31729700	-1.02098000
	H	7.20444700	-2.65288200	-1.58626600
	H	8.80099200	-0.74704000	-1.50920600
	H	7.97660300	1.50034400	-0.83879700
	H	1.08680900	-0.60165500	0.91456600
	H	0.58015500	-1.75873800	3.00512200
	H	2.41793900	-2.62548000	4.44433200
	H	4.77579300	-2.28813900	3.73142000
	H	5.28394700	-1.14615500	1.62826200
	H	3.21009800	1.47964500	2.09641600
	H	2.79918400	3.89362700	2.31185800
	H	2.56405800	5.31237500	0.28723700
	H	2.77367100	4.27208800	-1.96208600
	H	3.20893700	1.86677400	-2.17621700

	H	-0.00885000	0.46179700	-2.65008400
48	O	-1.40291800	-0.43221900	-1.37723000
	C	-2.74280200	-0.56881300	-1.47722400
	C	-0.61501200	-0.84986100	-2.53258800
	H	-1.21704100	-0.65763100	-3.42351500
	C	0.60134200	0.04976300	-2.60296100
	H	1.12551200	-0.11309300	-3.54758600
	C	-0.29621400	-2.33558800	-2.43262700
	H	-1.21841800	-2.91695900	-2.46316800
	H	0.24433400	-2.55225000	-1.51096200
	O	-3.27458700	-1.02574100	-2.45588600
	C	-3.48575100	-0.05125400	-0.19774000
	C	-4.94102900	-0.58613400	-0.21831900
	C	-5.50327800	-1.21763400	0.89511700
	C	-6.82654100	-1.66164300	0.88390300
	C	-7.61837700	-1.48203700	-0.24320100
	C	-7.07554400	-0.84544400	-1.35823900
	C	-5.75840200	-0.40079400	-1.34526000
	C	-3.46015500	1.50832000	-0.24431500
	C	-2.24145800	2.19680600	-0.35178800
	C	-2.19807800	3.58746100	-0.35878000
	C	-3.37159900	4.33055900	-0.24906200
	C	-4.58384900	3.66288800	-0.12056700
	C	-4.62759200	2.26818900	-0.11599200
	C	-2.74554700	-0.57308900	1.05512300
	C	-2.58841200	0.21186600	2.19932500
	C	-1.98235000	-0.30556900	3.34520700
	C	-1.52028700	-1.61735800	3.36539600
	C	-1.68030600	-2.41419100	2.23261800
	C	-2.29069300	-1.89711400	1.09531200
	H	-4.91332900	-1.37023800	1.78794500
	H	-7.23072900	-2.14951100	1.76400700
	H	-8.64545100	-1.82955700	-0.25530400
	H	-7.67947700	-0.69292400	-2.24596500
	H	-5.36340000	0.09171000	-2.22129800
	H	-1.31685700	1.64149600	-0.41172400
	H	-1.23976100	4.08780400	-0.44283400
	H	-3.33838700	5.41425600	-0.25609100
	H	-5.50736700	4.22241000	-0.02032100
	H	-5.58398900	1.77752800	-0.00553000
	H	-2.94420000	1.23391800	2.20406500
	H	-1.87452600	0.32312600	4.22227900
	H	-1.04331600	-2.01743800	4.25289000
	H	-1.32768800	-3.43929700	2.23304900
	H	-2.42233600	-2.53873600	0.23121300
	H	0.32436100	-2.64095800	-3.27985600
	O	1.49055800	-0.25271100	-1.50828800
	C	2.67787900	0.38809400	-1.52284600
	C	3.48192400	0.13401100	-0.22080400
	C	5.01376600	0.22836600	-0.42783900
	C	5.82847000	0.20295300	0.71426900
	O	3.00086800	1.13647200	-2.41021300

	C	5.63988400	0.24195100	-1.67737700
	C	7.03249100	0.23825300	-1.77764300
	C	7.82653900	0.22331800	-0.63763800
	C	7.21411500	0.20621100	0.61474900
	C	3.23173300	-1.28729300	0.33635200
	C	3.32668400	-2.38291200	-0.53160200
	C	3.20832400	-3.68586700	-0.06295200
	C	3.00232100	-3.92676300	1.29505300
	C	2.92461300	-2.84963300	2.17041700
	C	3.04049800	-1.54256000	1.69564700
	C	2.93606700	1.28186400	0.67435600
	C	1.65546000	1.19255300	1.23871900
	C	1.12311400	2.25018000	1.97108000
	C	1.85179300	3.42559800	2.14091500
	C	3.11079400	3.53683600	1.55874300
	C	3.64468700	2.47836400	0.82595500
	H	5.37222000	0.17613100	1.69644000
	H	5.05184600	0.28055100	-2.58143000
	H	7.49138100	0.25201000	-2.76017400
	H	8.90760600	0.22299300	-0.72003600
	H	7.81580600	0.18918500	1.51677800
	H	3.50773300	-2.21786000	-1.58793600
	H	3.28324300	-4.51485700	-0.75802000
	H	2.91213400	-4.94226800	1.66382100
	H	2.77511000	-3.01913900	3.23100000
	H	2.97751400	-0.72202900	2.39820400
	H	1.06511900	0.29631500	1.09981000
	H	0.13351900	2.15105400	2.40161500
	H	1.43763800	4.24847300	2.71279000
	H	3.68303000	4.45150300	1.66709200
	H	4.61833800	2.58915100	0.36828900
	H	0.31312100	1.10161600	-2.55196700
58	O	-1.42794400	-0.36753600	-1.31003900
	C	-2.76221300	-0.51323300	-1.44395200
	C	-0.61517900	-0.63822600	-2.49176900
	H	-1.21762800	-0.37014000	-3.36293100
	C	0.56475200	0.31094600	-2.44032400
	H	1.08903900	0.30444200	-3.39931400
	C	-0.24278500	-2.11312800	-2.55512400
	H	0.37474000	-2.30271900	-3.43736000
	H	-1.14430800	-2.72136000	-2.63616300
	O	-3.28346000	-0.93858800	-2.44343500
	C	-3.52596400	0.05821900	-0.21652600
	C	-2.96750300	-0.43717600	1.13637600
	C	-2.20809900	-1.60541000	1.24999200
	C	-1.82748400	-2.09750600	2.49787200
	C	-2.20131100	-1.43314400	3.66096100
	C	-2.96561200	-0.27168600	3.56380400
	C	-3.34682700	0.21480000	2.31760400
	C	-5.00707800	-0.39125200	-0.23235100
	C	-5.35156300	-1.70798200	-0.55983700
	C	-6.66797800	-2.14829200	-0.46713900

	C	-7.67209400	-1.28558700	-0.03387700
	C	-7.34158600	0.02127900	0.30889400
	C	-6.02283600	0.46154300	0.21175800
	C	-3.35550000	1.58349900	-0.45406700
	C	-4.08698900	2.20187400	-1.47779200
	C	-3.89612400	3.54659200	-1.78195300
	C	-2.95971500	4.30001100	-1.07717000
	C	-2.20890600	3.69035800	-0.07646700
	C	-2.39922900	2.34310100	0.22664900
	H	-1.89959900	-2.14532200	0.36499200
	H	-1.23400600	-3.00301700	2.55409800
	H	-1.90478100	-1.81553000	4.63098100
	H	-3.27499400	0.25415700	4.46035800
	H	-3.95726300	1.10766100	2.26529400
	H	-4.59222800	-2.39847900	-0.89992200
	H	-6.90770700	-3.17117900	-0.73566900
	H	-8.69786700	-1.62933000	0.03718700
	H	-8.10873100	0.70592600	0.65353300
	H	-5.79107900	1.48384700	0.48197800
	H	-4.80785600	1.62401700	-2.04303700
	H	-4.47872600	4.00314600	-2.57432000
	H	-2.81120700	5.34795500	-1.31193300
	H	-1.46469200	4.25877600	0.47002800
	H	-1.79090600	1.88544400	0.99459200
	H	0.32521600	-2.40873600	-1.67287600
	O	1.47625500	-0.11579600	-1.40612400
	C	2.59509600	0.62874900	-1.26518500
	C	3.51159100	0.08641900	-0.12054700
	C	3.53511000	-1.45701400	-0.22092400
	C	3.79625400	-2.05759900	-1.46083700
	O	2.81636700	1.58598800	-1.95922500
	C	3.35117800	-2.28543100	0.88607300
	C	3.42071800	-3.67458700	0.75913500
	C	3.67320600	-4.25764500	-0.47708700
	C	3.86296600	-3.43937600	-1.59119300
	C	2.91675200	0.55003700	1.24334000
	C	3.76035200	0.97209000	2.27840700
	C	3.24841100	1.33208000	3.52387800
	C	1.87957100	1.27727400	3.76491600
	C	1.02939400	0.84867600	2.74911000
	C	1.54057200	0.48659700	1.50468000
	C	4.93525800	0.67157500	-0.30393200
	C	6.07282300	-0.14044100	-0.28659900
	C	7.35228200	0.40620000	-0.40156900
	C	7.51983800	1.77887900	-0.53366900
	C	6.39499700	2.60261700	-0.53754500
	C	5.12157000	2.05827500	-0.41850200
	H	3.96348200	-1.43490000	-2.33369300
	H	3.15096700	-1.85259400	1.85708800
	H	3.27539100	-4.29725200	1.63504300
	H	3.72411200	-5.33616700	-0.57534000
	H	4.06576600	-3.87860800	-2.56173900

	H	4.82842900	1.02037100	2.11809700
	H	3.92840100	1.65521300	4.30446800
	H	1.48017000	1.55869400	4.73293000
	H	-0.03809400	0.77768800	2.92306300
	H	0.85796500	0.13576200	0.74413900
	H	5.97317500	-1.21152400	-0.17803500
	H	8.21473200	-0.25103300	-0.38598800
	H	8.51242700	2.20531400	-0.62719400
	H	6.50775200	3.67705900	-0.63190700
	H	4.26601800	2.71748800	-0.41700000
	H	0.23344700	1.33053700	-2.23794600
59	O	-1.60880500	-1.65782700	0.84821100
	C	-1.74427800	-0.76922300	-0.15395800
	C	-0.66644700	-2.75559000	0.69422500
	H	-0.42552800	-2.85374900	-0.36485100
	C	0.61720000	-2.47695100	1.47591600
	H	0.40371100	-1.87049200	2.35647000
	C	-1.38325800	-3.99465400	1.21701300
	H	-0.77026200	-4.88466200	1.05530100
	H	-2.33108900	-4.12449200	0.69341200
	O	-1.03252000	-0.74510300	-1.12653900
	C	-3.01846900	0.10260600	0.02456700
	C	-2.91512100	1.40840700	-0.79591000
	C	-4.06136200	2.00180300	-1.33592400
	C	-3.99691500	3.23479900	-1.98070500
	C	-2.78188500	3.90422400	-2.09326500
	C	-1.63685200	3.33201800	-1.54505400
	C	-1.70315300	2.10053400	-0.89905100
	C	-3.26603900	0.55345600	1.48171600
	C	-2.20441500	0.77396000	2.36585400
	C	-2.43273900	1.29273000	3.63880700
	C	-3.72283000	1.60999000	4.05344700
	C	-4.78500800	1.41417200	3.17463400
	C	-4.55653800	0.89767200	1.90223500
	C	-4.08470000	-0.88585300	-0.52518600
	C	-4.78949400	-1.74940100	0.32142500
	C	-5.66563100	-2.70152600	-0.19624400
	C	-5.84032500	-2.82321100	-1.57217300
	C	-5.12009600	-1.99160800	-2.42621500
	C	-4.24669000	-1.03891100	-1.90862700
	H	-5.01759600	1.49967100	-1.25875900
	H	-4.90071200	3.66983000	-2.39322200
	H	-2.72867600	4.86250100	-2.59795600
	H	-0.68161400	3.83968400	-1.61558700
	H	-0.79795000	1.68702700	-0.47599400
	H	-1.18741600	0.55222000	2.07079600
	H	-1.59160700	1.45190200	4.30463400
	H	-3.89822200	2.01261600	5.04481600
	H	-5.79567900	1.66875300	3.47455400
	H	-5.39619500	0.76742900	1.23086200
	H	-4.65291800	-1.68065800	1.39218200
	H	-6.20915700	-3.35068900	0.48149900

	H	-6.52363400	-3.56256800	-1.97472300
	H	-5.23233000	-2.08489000	-3.50069900
	H	-3.67900600	-0.41318800	-2.58515800
	H	-1.59306600	-3.90307900	2.28608700
	O	1.64340300	-1.84503100	0.67712400
	C	1.79851500	-0.50848700	0.76637400
	C	3.06063900	-0.01628900	-0.01606500
	C	4.26150700	-0.17204800	0.96794300
	C	5.22160500	0.83539400	1.11029800
	O	1.06923600	0.18817000	1.42532700
	C	4.45090600	-1.36254000	1.68463400
	C	5.54358600	-1.52683600	2.53075100
	C	6.48488600	-0.51020700	2.67236400
	C	6.32099100	0.66783300	1.95169000
	C	2.85961500	1.47338100	-0.40152000
	C	2.55290600	2.43099300	0.57951800
	C	2.40509500	3.77404000	0.25086100
	C	2.57040000	4.20196100	-1.06581600
	C	2.88796800	3.26837400	-2.04479000
	C	3.03161900	1.91964500	-1.71518900
	C	3.27913600	-0.88400000	-1.27724400
	C	2.19762400	-1.17176100	-2.12220900
	C	2.38288100	-1.89650100	-3.29456700
	C	3.65376100	-2.34438200	-3.65436900
	C	4.73420600	-2.05668400	-2.82834300
	C	4.54767900	-1.33304100	-1.64943800
	H	5.11910800	1.75976800	0.55927500
	H	3.75104100	-2.17788500	1.56472300
	H	5.66047300	-2.45695500	3.07608000
	H	7.33645500	-0.63785600	3.33108300
	H	7.04892200	1.46678100	2.03957000
	H	2.42699900	2.12505600	1.60736400
	H	2.16195600	4.48849600	1.02954900
	H	2.45877300	5.25015900	-1.32045000
	H	3.02670400	3.58093500	-3.07380700
	H	3.28116700	1.21813200	-2.49836900
	H	1.20008400	-0.83192700	-1.87005800
	H	1.52913400	-2.10902500	-3.92856600
	H	3.79726700	-2.90981300	-4.56845800
	H	5.72978800	-2.39455600	-3.09445100
	H	5.40341600	-1.12127200	-1.02205500
	H	1.06425600	-3.42462700	1.77746700
60	O	-1.41836600	-1.57421300	-0.72142900
	C	-2.40666700	-0.78561500	-1.17641800
	C	-0.62994300	-2.30818800	-1.70070700
	H	-0.46760800	-1.65996000	-2.56305700
	C	0.69362400	-2.63432800	-1.03028400
	H	0.53360300	-2.97801500	-0.00840500
	C	-1.37664900	-3.57181300	-2.11548000
	H	-0.79352800	-4.13134600	-2.85134200
	H	-2.33121600	-3.30910100	-2.57105600
	O	-2.62048800	-0.61794200	-2.35191200

	C	-3.20534300	-0.07261400	-0.03702900
	C	-4.63647500	0.19393900	-0.56643900
	C	-5.30584600	1.38739600	-0.28690600
	C	-6.62176500	1.58948600	-0.70383800
	C	-7.29497300	0.60022600	-1.41128000
	C	-6.64041600	-0.59729200	-1.69502700
	C	-5.32966400	-0.79603800	-1.27605700
	C	-2.48208300	1.28620300	0.20229200
	C	-2.03301200	2.04825300	-0.88569000
	C	-1.45164900	3.29856400	-0.69275200
	C	-1.31776400	3.82431700	0.59000100
	C	-1.77804200	3.08627200	1.67573300
	C	-2.35239500	1.82959900	1.48451400
	C	-3.24947600	-0.92772300	1.26012100
	C	-2.06922800	-1.24198400	1.95528000
	C	-2.10199800	-1.98786300	3.12780400
	C	-3.31529700	-2.43908000	3.64649600
	C	-4.49176200	-2.12750100	2.97754000
	C	-4.45854300	-1.37759800	1.79999700
	H	-4.80262800	2.17354600	0.25999600
	H	-7.11499800	2.52755600	-0.47434700
	H	-8.31575800	0.75822600	-1.74079500
	H	-7.14992200	-1.37917300	-2.24732500
	H	-4.84550600	-1.73655100	-1.50671700
	H	-2.15615800	1.66944900	-1.89252500
	H	-1.10692800	3.86519900	-1.55033300
	H	-0.86322300	4.79690500	0.73936600
	H	-1.69562700	3.48537400	2.68079800
	H	-2.70647600	1.27661300	2.34361900
	H	-1.11039100	-0.91088100	1.58402900
	H	-1.17037000	-2.21292500	3.63493900
	H	-3.33961500	-3.02053300	4.56155000
	H	-5.44742300	-2.46097600	3.36699100
	H	-5.39286100	-1.14320700	1.31029600
	H	-1.56103500	-4.21496300	-1.25129100
	O	1.58461100	-1.49997000	-1.02838300
	C	1.92462700	-0.94364400	0.15483700
	C	3.15986300	-0.01497300	-0.00631300
	C	3.10343000	0.86672600	-1.27354400
	C	4.28136200	1.37285500	-1.83617700
	O	1.41073400	-1.23161500	1.20441700
	C	1.88491800	1.30178300	-1.80391400
	C	1.84471800	2.18720000	-2.87827300
	C	3.02362200	2.66670800	-3.44117800
	C	4.24405300	2.25810100	-2.90977900
	C	4.28841200	-1.08421000	-0.02746300
	C	4.74921400	-1.63509900	1.17542000
	C	5.69308700	-2.65865700	1.17822500
	C	6.18476300	-3.16448000	-0.02246000
	C	5.70902500	-2.64863000	-1.22492100
	C	4.76261500	-1.62613700	-1.22765300
	C	3.28357700	0.97274600	1.17606500

	C	4.54122600	1.42942900	1.58733400
	C	4.66641500	2.40456400	2.57346800
	C	3.53217500	2.95097000	3.16697700
	C	2.27529800	2.51686900	2.75534700
	C	2.15142200	1.54259000	1.76823000
	H	5.24177300	1.07686300	-1.43325500
	H	0.95021500	0.95809600	-1.38046100
	H	0.88482600	2.49990400	-3.27349700
	H	2.99223000	3.35388800	-4.27911000
	H	5.17286300	2.63070300	-3.32748800
	H	4.36304100	-1.26393600	2.11606300
	H	6.03958100	-3.06279800	2.12284300
	H	6.92281400	-3.95871200	-0.02096600
	H	6.07009800	-3.04389900	-2.16801100
	H	4.39012300	-1.25018900	-2.17108400
	H	5.43747200	1.01998500	1.13857400
	H	5.65400300	2.73627600	2.87464100
	H	3.62666700	3.70799100	3.93744400
	H	1.37997500	2.93578200	3.20056300
	H	1.16072000	1.23109700	1.46848500
	H	1.20861600	-3.40434800	-1.60727600
61	O	1.66995600	-1.85309500	0.54664700
	C	1.76876200	-0.51972900	0.71157900
	C	0.65753800	-2.58659400	1.29953400
	H	0.41412100	-2.01304100	2.19452900
	C	-0.60829200	-2.77350000	0.46114900
	H	-0.35946100	-2.80328900	-0.59989000
	C	1.29286100	-3.92554100	1.65298000
	H	0.60580800	-4.51974200	2.25987800
	H	2.20878200	-3.77365600	2.22551000
	O	1.02069800	0.10838800	1.41775500
	C	2.99472400	0.07555600	-0.06181200
	C	2.71089800	1.56771500	-0.38330500
	C	2.86554200	2.07986700	-1.67486400
	C	2.65876500	3.43342400	-1.94565500
	C	2.29406400	4.30642100	-0.92792300
	C	2.14567800	3.81269300	0.36764500
	C	2.35664600	2.46490900	0.63782800
	C	3.24146200	-0.72765100	-1.36010700
	C	4.52060700	-1.13020500	-1.74788300
	C	4.72565500	-1.80145800	-2.95476100
	C	3.65369500	-2.08124400	-3.79402800
	C	2.37188100	-1.67812700	-3.41933600
	C	2.16806700	-1.00689500	-2.21881800
	C	4.21650200	-0.04615700	0.89976600
	C	5.13067800	1.00207700	1.05040900
	C	6.24950500	0.86886800	1.87192300
	C	6.47964300	-0.31506300	2.56446100
	C	5.58558700	-1.37198700	2.41323600
	C	4.47341800	-1.24092700	1.58665500
	H	3.15316800	1.42646800	-2.48594900
	H	2.78592200	3.79741900	-2.95913400

	H	2.13370900	5.35854400	-1.13621400
	H	1.86746900	4.47973300	1.17614900
	H	2.24421100	2.10782800	1.65048600
	H	5.37053700	-0.92317700	-1.11123600
	H	5.72943200	-2.10416000	-3.23225500
	H	3.81186000	-2.60529700	-4.73009800
	H	1.52448700	-1.88417200	-4.06396800
	H	1.16279100	-0.69979100	-1.95492700
	H	4.97643700	1.93225700	0.52164500
	H	6.94020800	1.69940400	1.96704700
	H	7.34603900	-0.41615100	3.20827500
	H	5.75507800	-2.30731900	2.93546500
	H	3.81214600	-2.08578300	1.45600500
	H	1.54093700	-4.48996000	0.75054700
	O	-1.60169200	-1.75835700	0.70650200
	C	-1.77764000	-0.79190200	-0.21725700
	C	-3.08578700	0.00237300	0.04063600
	C	-3.04774700	1.38646500	-0.64634600
	C	-4.23440100	1.99230600	-1.07523200
	O	-1.07023200	-0.65756400	-1.18306900
	C	-1.86247300	2.12505600	-0.73001600
	C	-1.86170100	3.41652100	-1.25145200
	C	-3.04595200	4.00143400	-1.69099600
	C	-4.23507700	3.28383000	-1.59535800
	C	-3.34559100	0.29452200	1.53567500
	C	-2.28619900	0.51183400	2.42368500
	C	-2.52775100	0.89026600	3.74235000
	C	-3.83020900	1.06777500	4.20053200
	C	-4.89129200	0.87495600	3.32010400
	C	-4.64966500	0.49839200	2.00150900
	C	-4.10938900	-0.97506900	-0.60369600
	C	-4.73289900	-1.97798300	0.14815900
	C	-5.57184100	-2.90967400	-0.45924500
	C	-5.78899600	-2.87046500	-1.83400500
	C	-5.14645100	-1.89941900	-2.59768200
	C	-4.30952000	-0.96697500	-1.99012400
	H	-5.17131000	1.45352200	-1.00766700
	H	-0.92720400	1.70168300	-0.39105300
	H	-0.92580800	3.96022600	-1.31046500
	H	-3.04354600	5.00631900	-2.09851400
	H	-5.16873800	3.72733000	-1.92378400
	H	-1.26137300	0.39445000	2.09616700
	H	-1.68844900	1.04995000	4.41035000
	H	-4.01619000	1.36119600	5.22766500
	H	-5.91220900	1.02214700	3.65518900
	H	-5.48994100	0.36625000	1.33142900
	H	-4.56090800	-2.03433400	1.21465500
	H	-6.05255300	-3.66978400	0.14676800
	H	-6.44401900	-3.59407500	-2.30620100
	H	-5.29102600	-1.86672400	-3.67185700
	H	-3.80296300	-0.22942300	-2.59891000
	H	-1.09710700	-3.70400500	0.75218800

69	O	-1.40330700	-0.87316700	-1.40937800	
	C	-2.10912900	0.24159700	-1.15383000	
	C	-0.46115700	-0.83363200	-2.52127300	
	H	-0.07804000	0.18302900	-2.60355200	
	C	0.65851400	-1.79635000	-2.16423800	
	H	0.24880100	-2.73500300	-1.79193600	
	C	-1.17205400	-1.23691900	-3.80680400	
	H	-2.00729100	-0.56336600	-4.00052600	
	H	-1.55583700	-2.25842800	-3.74051200	
	O	-1.96843500	1.26519100	-1.77573300	
	C	-3.17986300	0.05239800	-0.03123900	
	C	-4.48935900	-0.35741700	-0.77442400	
	C	-5.37449700	-1.29987000	-0.24001800	
	C	-6.57542800	-1.60200400	-0.88151000	
	C	-6.91803500	-0.96818500	-2.07123900	
	C	-6.05312700	-0.01611400	-2.60534800	
	C	-4.85785100	0.29137700	-1.96151000	
	C	-3.39283300	1.41843300	0.66472900	
	C	-2.30613300	2.25419400	0.95458400	
	C	-2.48416500	3.44411200	1.65308000	
	C	-3.75343800	3.82750200	2.08133900	
	C	-4.84000100	3.00585000	1.80218500	
	C	-4.66017000	1.81458900	1.10009000	
	C	-2.74733500	-1.02640600	0.99809500	
	C	-2.50694100	-0.70885000	2.33806600	
	C	-2.13345800	-1.68939100	3.25994900	
	C	-1.99728400	-3.01345300	2.86336300	
	C	-2.23989900	-3.34925600	1.53132200	
	C	-2.60996900	-2.37166000	0.61611800	
	H	-5.13559200	-1.80098500	0.68798400	
	H	-7.24242200	-2.33582900	-0.44251700	
	H	-7.84945700	-1.20675300	-2.57221100	
	H	-6.31002900	0.49858600	-3.52462200	
	H	-4.21415000	1.05573900	-2.37698100	
	H	-1.30773300	1.98606500	0.63359700	
	H	-1.62438100	4.07139300	1.85896200	
	H	-3.89235200	4.75664300	2.62274700	
	H	-5.83560300	3.28889700	2.12570200	
	H	-5.52150100	1.19374100	0.89183300	
	H	-2.61483600	0.31003300	2.68144400	
	H	-1.95685900	-1.40679000	4.29189000	
	H	-1.71406900	-3.77729800	3.57900500	
	H	-2.14417900	-4.37862800	1.20357700	
	H	-2.80031100	-2.65712500	-0.40874400	
	H	-0.48207200	-1.17548700	-4.65190000	
	O	1.48424100	-1.29808900	-1.09187000	
	C	2.54132600	-0.54167400	-1.44201200	
	C	3.35555700	-0.01977900	-0.21697800	
	C	3.35314000	-1.04863100	0.94779700	
	C	4.53496300	-1.63789600	1.40775300	
	O	2.78956200	-0.26063700	-2.58853200	
	C	2.15686700	-1.39738800	1.59702700	

	C	2.14561600	-2.30963600	2.64527500
	C	3.33175400	-2.89780600	3.08320500
	C	4.52482500	-2.55456200	2.46093300
	C	2.67009100	1.31568500	0.19879100
	C	2.22869900	2.21657000	-0.77994800
	C	1.67666700	3.44475000	-0.42676300
	C	1.57064300	3.80919600	0.91346100
	C	2.02054600	2.93046000	1.89430800
	C	2.56248400	1.69524500	1.54082200
	C	4.80007300	0.26779900	-0.69432200
	C	5.50572500	1.38504200	-0.24214400
	C	6.83242100	1.59872200	-0.61674800
	C	7.47986700	0.69849100	-1.45509000
	C	6.78867000	-0.42200700	-1.91265000
	C	5.46718700	-0.63367800	-1.53521600
	H	5.48119100	-1.38464600	0.95144800
	H	1.22272000	-0.95675500	1.28023600
	H	1.20204700	-2.55773600	3.11789100
	H	3.32271400	-3.60940200	3.90146200
	H	5.45999800	-2.99368600	2.79065900
	H	2.33228500	1.96657600	-1.82813100
	H	1.33279000	4.11761000	-1.20397500
	H	1.14894300	4.76960600	1.18802200
	H	1.95349700	3.20156900	2.94215400
	H	2.90840600	1.02929600	2.31965800
	H	5.02253300	2.10239300	0.40761100
	H	7.35411900	2.47661600	-0.25201800
	H	8.50893900	0.86666300	-1.75222600
	H	7.27762400	-1.13340000	-2.56898200
	H	4.95341300	-1.51195600	-1.90462500
	H	1.27914100	-1.97426000	-3.04224100
70	O	-1.43564500	-0.23557400	-1.38714000
	C	-2.76065100	-0.47679900	-1.48745300
	C	-0.61762200	-0.58348600	-2.54449700
	H	-1.19827900	-0.32944800	-3.43508900
	C	0.59628300	0.32207700	-2.51962600
	H	1.13310300	0.24702300	-3.46684800
	C	-0.29415200	-2.07206000	-2.55093400
	H	0.33530100	-2.30824300	-3.41374700
	H	0.24087800	-2.35761100	-1.64505200
	O	-3.25726200	-0.96062500	-2.47164300
	C	-3.53595500	-0.04442900	-0.19820300
	C	-3.03839900	1.35914900	0.21795600
	C	-2.80421800	1.70001800	1.55055500
	C	-2.42443700	2.99719700	1.90080600
	C	-2.26656400	3.97370500	0.92355000
	C	-2.50005300	3.64595400	-0.41186000
	C	-2.88483700	2.35549200	-0.75633100
	C	-3.25987500	-1.08666200	0.92811400
	C	-1.96888700	-1.57193100	1.18202500
	C	-1.72746700	-2.46228400	2.22563000
	C	-2.76648100	-2.88002500	3.05259200

	C	-4.04901900	-2.39191900	2.82534100
	C	-4.29263200	-1.50782700	1.77555300
	C	-5.04865500	-0.00991600	-0.53524300
	C	-5.67801400	-1.14924400	-1.06125500
	C	-7.04010100	-1.15427500	-1.33537100
	C	-7.81539000	-0.02248100	-1.08449600
	C	-7.20946800	1.10788100	-0.55146300
	C	-5.83981200	1.11264800	-0.27898900
	H	-2.92104200	0.95489100	2.32630300
	H	-2.25561600	3.23890000	2.94460400
	H	-1.96772300	4.97971800	1.19534000
	H	-2.38532700	4.39723000	-1.18526100
	H	-3.08407700	2.12447200	-1.79759800
	H	-1.13966000	-1.24636000	0.57176000
	H	-0.71766800	-2.82227200	2.38720900
	H	-2.57831000	-3.57295100	3.86512600
	H	-4.87090700	-2.69785300	3.46332600
	H	-5.29931100	-1.14486300	1.62216800
	H	-5.09894800	-2.03992100	-1.25876100
	H	-7.49759000	-2.04702600	-1.74732900
	H	-8.87812300	-0.02708700	-1.29980800
	H	-7.79621700	1.99554900	-0.34232600
	H	-5.39699500	2.00512500	0.14070700
	H	-1.21224700	-2.65356200	-2.63166900
	O	1.47859300	-0.06596000	-1.44355300
	C	2.73691700	0.42041200	-1.51093500
	C	3.54959400	0.11813900	-0.22417300
	C	3.27628800	1.40909200	0.59883800
	C	1.99900000	1.64506700	1.12838400
	O	3.12590600	1.09095600	-2.43327600
	C	4.24294800	2.41011800	0.73194700
	C	3.95641100	3.59317600	1.41136700
	C	2.69436800	3.80418900	1.95696500
	C	1.71334500	2.82638800	1.80596500
	C	5.06177500	-0.08831000	-0.49946100
	C	5.63779200	-0.14415700	-1.77154100
	C	7.00153300	-0.40272700	-1.92657200
	C	7.81684700	-0.60485400	-0.82029800
	C	7.25448600	-0.55144000	0.45443400
	C	5.89641200	-0.30336600	0.60854300
	C	3.07261900	-1.18254300	0.46469100
	C	3.00194100	-2.35952700	-0.29307000
	C	2.67535700	-3.57568000	0.29359700
	C	2.42347600	-3.64843400	1.66406700
	C	2.50643300	-2.49184300	2.43084200
	C	2.82811800	-1.27003000	1.83606400
	H	1.21865900	0.90540600	1.00360900
	H	5.22544400	2.27158400	0.30227300
	H	4.72526100	4.35198800	1.50571600
	H	2.47272800	4.72511300	2.48470700
	H	0.71731200	2.98202100	2.20374600
	H	5.03748700	0.03704100	-2.64939900

	H	7.42131200	-0.43965900	-2.92586100
	H	8.87539600	-0.80311800	-0.94550300
	H	7.87233300	-0.71069100	1.33126000
	H	5.47656900	-0.28228400	1.60689400
	H	3.22094100	-2.32761200	-1.35461700
	H	2.62608900	-4.47044700	-0.31699900
	H	2.17513100	-4.59753800	2.12544200
	H	2.32567700	-2.53188200	3.49943600
	H	2.89105500	-0.38488900	2.45505100
	H	0.29911500	1.36215600	-2.37024900
71	O	1.72166100	0.36506500	0.35623100
	C	2.62547600	0.19644400	-0.62978800
	C	0.36924600	0.74592300	-0.02458000
	H	0.12003700	0.22853800	-0.95171800
	C	-0.48409500	0.20007600	1.11971100
	H	-0.29570800	-0.86509200	1.24861800
	C	0.26846100	2.25145500	-0.20511900
	H	-0.76896700	2.53021800	-0.40135200
	H	0.87902600	2.57335900	-1.04930700
	O	2.37094000	0.34719200	-1.79669200
	C	4.03301500	-0.10286500	-0.04074900
	C	5.00372500	-0.64726500	-1.11574900
	C	6.37958800	-0.41531300	-0.99872000
	C	7.28652300	-0.99323100	-1.88316000
	C	6.83657500	-1.82321500	-2.90579400
	C	5.47236800	-2.07133600	-3.02662600
	C	4.56640300	-1.49485100	-2.13994500
	C	3.98377000	-1.19770300	1.04995300
	C	3.01464300	-2.20631500	1.01758200
	C	3.03906100	-3.25811700	1.93004600
	C	4.03892600	-3.32757800	2.89586100
	C	5.02029200	-2.34034500	2.92897800
	C	4.99531200	-1.29246700	2.01240500
	C	4.43784900	1.30456700	0.47426200
	C	4.21390500	1.70378800	1.79634000
	C	4.49725000	3.00468400	2.20713800
	C	4.99066200	3.93851700	1.30014300
	C	5.18729600	3.56227000	-0.02620000
	C	4.90849100	2.26098300	-0.43518300
	H	6.75317700	0.22753500	-0.21160400
	H	8.34608400	-0.79240300	-1.76892000
	H	7.53981700	-2.27241200	-3.59803200
	H	5.10468200	-2.71724900	-3.81626700
	H	3.51308900	-1.69991300	-2.26421600
	H	2.22993100	-2.18490700	0.27150900
	H	2.27365600	-4.02464600	1.88104400
	H	4.05808300	-4.14454600	3.60818600
	H	5.81412400	-2.38569200	3.66637400
	H	5.77660300	-0.54385500	2.04784600
	H	3.80950800	0.99890000	2.51011300
	H	4.32509600	3.28741200	3.23987100
	H	5.21148100	4.95028800	1.62122000

	H	5.55566900	4.28199900	-0.74867800
	H	5.05324500	1.98620200	-1.47241000
	H	0.60845400	2.76850300	0.69609600
	O	-1.88624800	0.42341700	0.86602000
	C	-2.53536800	-0.51250200	0.13627300
	C	-4.01378700	-0.09453500	-0.10102400
	C	-3.85803200	1.18812900	-0.96032900
	C	-3.36861400	1.06911900	-2.26931100
	O	-1.99600000	-1.49328300	-0.30530600
	C	-4.07375500	2.47007900	-0.44936200
	C	-3.84691500	3.59924000	-1.23585400
	C	-3.38353900	3.46812500	-2.54138900
	C	-3.13780600	2.19541500	-3.05324600
	C	-4.76957100	0.13396000	1.22974000
	C	-4.26975100	-0.27684800	2.46834400
	C	-5.02625900	-0.13648000	3.63196800
	C	-6.29890200	0.41998300	3.58145900
	C	-6.81471100	0.82481700	2.35143100
	C	-6.06227600	0.67521900	1.19212400
	C	-4.78881100	-1.21606600	-0.83461300
	C	-5.70750400	-0.92520700	-1.84763900
	C	-6.47975900	-1.93012700	-2.42948600
	C	-6.34968800	-3.24815200	-2.00694700
	C	-5.44709600	-3.54926900	-0.98890200
	C	-4.68205300	-2.54534800	-0.40557600
	H	-3.16745600	0.08587700	-2.67829000
	H	-4.41324800	2.59440200	0.56976700
	H	-4.02901800	4.58376900	-0.81931400
	H	-3.20723100	4.34676700	-3.15149000
	H	-2.76231000	2.07659900	-4.06336600
	H	-3.28111500	-0.70904300	2.54579800
	H	-4.61196200	-0.46498100	4.57867600
	H	-6.88593600	0.53155200	4.48594100
	H	-7.81029000	1.25034000	2.29147000
	H	-6.49008900	0.97552300	0.24350700
	H	-5.82560200	0.09271500	-2.19541000
	H	-7.18214500	-1.67524500	-3.21541800
	H	-6.94540100	-4.03190000	-2.46120200
	H	-5.33726200	-4.57145000	-0.64403900
	H	-3.99176700	-2.80466400	0.38506500
	H	-0.27233500	0.73240300	2.04710900
73	O	1.52243800	1.54488400	0.23271100
	C	2.33453700	0.98625500	-0.68739900
	C	0.69072700	2.66019000	-0.19198100
	H	0.48298800	2.53942000	-1.25532400
	C	-0.59333100	2.57156600	0.60516800
	H	-0.39157900	2.39229900	1.66229400
	C	1.43738200	3.96425600	0.06225500
	H	1.64986900	4.09325600	1.12623900
	H	0.84485800	4.81516400	-0.28377700
	O	2.34689700	1.30822600	-1.84803100
	C	3.33350600	-0.00576900	-0.02938900

	C	2.67927000	-0.92791200	1.02414800	
	C	3.47065900	-1.54627700	2.00041100	
	C	2.92835500	-2.46812500	2.89078100	
	C	1.57928600	-2.80515100	2.81625600	
	C	0.78587200	-2.21447700	1.83758000	
	C	1.32957700	-1.28766500	0.94983600	
	C	4.36450600	0.99796800	0.55691800	
	C	5.30846600	1.59462000	-0.28978600	
	C	6.18334400	2.56744100	0.18628300	
	C	6.12431000	2.97482200	1.51697000	
	C	5.17060100	2.41134400	2.36013300	
	C	4.29266100	1.44033800	1.88228700	
	C	3.94982800	-0.95725500	-1.08104700	
	C	5.25275600	-1.44255200	-0.92339400	
	C	5.78155300	-2.38615100	-1.80101700	
	C	5.01385500	-2.86968100	-2.85606800	
	C	3.71132600	-2.40453100	-3.01604500	
	C	3.18304300	-1.46371300	-2.13647100	
	H	4.52522100	-1.30996700	2.06813500	
	H	3.56526300	-2.92710500	3.63894100	
	H	1.15443600	-3.52486900	3.50694300	
	H	-0.26283400	-2.47520300	1.75302600	
	H	0.68293500	-0.85027700	0.20044100	
	H	5.35499100	1.29927600	-1.33033600	
	H	6.90966100	3.00848500	-0.48731200	
	H	6.80831000	3.72887900	1.88993600	
	H	5.10252400	2.72913100	3.39474300	
	H	3.54582600	1.02755300	2.54687200	
	H	5.86918500	-1.07961400	-0.11072300	
	H	6.79590200	-2.74127400	-1.65605700	
	H	5.42390100	-3.60122200	-3.54329200	
	H	3.09874800	-2.77400000	-3.83114900	
	H	2.16860600	-1.12120800	-2.28487800	
	H	2.38292900	3.96442500	-0.48107200	
	O	-1.38245600	1.48570800	0.07084300	
	C	-2.53408400	1.22635000	0.71372900	
	C	-3.41504100	0.13872900	0.02424800	
	C	-4.48372100	0.91533200	-0.80808500	
	C	-4.91906500	0.44405100	-2.05199400	
	O	-2.86557100	1.82297800	1.70788700	
	C	-5.10604300	2.05664700	-0.28310300	
	C	-6.10627900	2.71658500	-0.99172600	
	C	-6.52069300	2.24548700	-2.23485300	
	C	-5.92640200	1.10148300	-2.75718800	
	C	-4.11742700	-0.66677000	1.14451000	
	C	-3.42361900	-1.02498900	2.30789800	
	C	-4.02066500	-1.81235400	3.28717300	
	C	-5.32875800	-2.26428100	3.12440600	
	C	-6.02799500	-1.91806600	1.97338700	
	C	-5.42804800	-1.12580100	0.99478100	
	C	-2.57248500	-0.79658300	-0.88226600	
	C	-2.47396900	-2.16927100	-0.63700200	

	C	-1.72406900	-3.00197200	-1.47070900
	C	-1.06098400	-2.48027300	-2.57365900
	C	-1.15885600	-1.11427600	-2.84037800
	C	-1.90499600	-0.28799200	-2.00893400
	H	-4.47750200	-0.44607300	-2.47878300
	H	-4.81758700	2.42513600	0.69216800
	H	-6.56622200	3.60019600	-0.56315100
	H	-7.30016800	2.75990600	-2.78554400
	H	-6.24311300	0.71200000	-3.71843500
	H	-2.40664600	-0.68533700	2.46156300
	H	-3.46254600	-2.06787400	4.18107300
	H	-5.79681000	-2.87429300	3.88877300
	H	-7.04803100	-2.25787900	1.83223900
	H	-5.99386000	-0.86431800	0.11036500
	H	-2.98862100	-2.60681000	0.20683700
	H	-1.66564400	-4.06177300	-1.24959200
	H	-0.47842500	-3.12576800	-3.22108200
	H	-0.65382300	-0.68859200	-3.70037300
	H	-1.97276000	0.76564500	-2.24107600
	H	-1.16379300	3.49883000	0.51406800
76	O	-1.75874300	0.46644100	-0.28068900
	C	-2.67238000	0.36306600	0.70616800
	C	-0.42576500	0.92133000	0.08181600
	H	-0.17502000	0.49217200	1.05325200
	C	0.47131000	0.31367100	-0.99477200
	H	0.29169400	-0.75735800	-1.07115500
	C	-0.38010900	2.44025000	0.13392000
	H	0.64871800	2.77415100	0.28510900
	H	-0.99352700	2.81333800	0.95466700
	O	-2.43598100	0.62389300	1.85735300
	C	-4.06084700	-0.03175100	0.12943000
	C	-3.98171300	-1.29065400	-0.76505600
	C	-5.04550200	-1.60295700	-1.62118700
	C	-5.05304600	-2.78118000	-2.36079500
	C	-4.00065400	-3.68733300	-2.25013600
	C	-2.94802600	-3.40151900	-1.38717200
	C	-2.93993100	-2.21682400	-0.65268800
	C	-4.45545500	1.26548000	-0.62561300
	C	-4.84533200	2.39101100	0.11360100
	C	-5.12025200	3.60154600	-0.51581500
	C	-5.00176900	3.71587200	-1.89915600
	C	-4.59158300	2.61191000	-2.64086900
	C	-4.31146800	1.40129200	-2.00942600
	C	-5.05536200	-0.39419600	1.25807900
	C	-6.41393800	-0.07979500	1.14464300
	C	-7.33428200	-0.50570500	2.10045800
	C	-6.91475000	-1.26022900	3.19109200
	C	-5.56679300	-1.59008400	3.30968400
	C	-4.64961800	-1.16765000	2.35250800
	H	-5.88318400	-0.92206600	-1.70778800
	H	-5.88837900	-2.99358000	-3.01885500
	H	-4.00595300	-4.60730100	-2.82356100

	H	-2.12547900	-4.09968800	-1.27930700
	H	-2.10712000	-2.02940500	0.01302800
	H	-4.93200200	2.31889300	1.19101100
	H	-5.42508700	4.45596200	0.07814800
	H	-5.21991300	4.65674200	-2.39177900
	H	-4.48203600	2.68958800	-3.71700100
	H	-3.97281400	0.56145500	-2.60068900
	H	-6.76503100	0.50769100	0.30596300
	H	-8.38064400	-0.24408000	1.98744200
	H	-7.62819700	-1.58974900	3.93802400
	H	-5.22314700	-2.18097600	4.15149000
	H	-3.60970300	-1.43768200	2.46991300
	H	-0.74902000	2.86303100	-0.80414200
	O	1.86072300	0.55454400	-0.68609300
	C	2.50551700	-0.40393800	0.01771300
	C	4.03226100	-0.13040100	0.06731200
	C	4.47537400	-0.78588900	-1.27141300
	C	4.25977600	-0.13250600	-2.49308200
	O	1.95568600	-1.38546100	0.44735500
	C	4.96926200	-2.09432200	-1.30047500
	C	5.28186600	-2.71401300	-2.50882700
	C	5.09418300	-2.04322400	-3.71328900
	C	4.57465100	-0.75078600	-3.69970600
	C	4.72575400	-0.77408300	1.29321100
	C	4.04666500	-1.24780700	2.41921000
	C	4.74876200	-1.72869600	3.52588200
	C	6.13803000	-1.74703100	3.53198200
	C	6.82721800	-1.27212500	2.41710000
	C	6.12837900	-0.78839500	1.31797900
	C	4.33587000	1.38239400	0.17484100
	C	5.36675500	1.99769700	-0.53856800
	C	5.67726100	3.34251500	-0.33366600
	C	4.96633000	4.09655500	0.59303900
	C	3.94655300	3.48953200	1.32468200
	C	3.64202500	2.14893600	1.11992900
	H	3.83681900	0.86327900	-2.50077700
	H	5.10933600	-2.63648500	-0.37513100
	H	5.66833500	-3.72705100	-2.50347200
	H	5.34003300	-2.52474600	-4.65304000
	H	4.40803700	-0.22064800	-4.63089500
	H	2.96797600	-1.26941300	2.43721800
	H	4.19580400	-2.09334800	4.38452000
	H	6.67942700	-2.12328300	4.39265300
	H	7.91154000	-1.27241700	2.40430500
	H	6.68296100	-0.41321000	0.46641800
	H	5.93871400	1.43144100	-1.26202000
	H	6.48105100	3.79605900	-0.90307800
	H	5.20653200	5.14178000	0.75089000
	H	3.39090300	4.05896400	2.06143000
	H	2.86203300	1.69075300	1.71796600
	H	0.30151400	0.79207200	-1.95958400
77	O	-1.42395100	-1.13008000	-1.00885700

	C	-2.23832200	-0.07538300	-1.18791000
	C	-0.46857800	-1.42049900	-2.07433900
	H	-0.10845300	-0.47408800	-2.47455000
	C	0.66969200	-2.19801200	-1.43353300
	H	0.27826100	-2.97196100	-0.77393200
	C	-1.14772100	-2.23285000	-3.17057200
	H	-1.99771400	-1.68374800	-3.57493100
	H	-1.50083300	-3.19401700	-2.78717500
	O	-2.16514900	0.65211400	-2.14693900
	C	-3.33716100	0.06414200	-0.08445600
	C	-4.56073500	-0.74023900	-0.62460600
	C	-5.35814500	-1.52284800	0.21697000
	C	-6.48779000	-2.17939100	-0.27130100
	C	-6.84417200	-2.06777600	-1.61096500
	C	-6.06739100	-1.27854000	-2.45595500
	C	-4.94443200	-0.61688400	-1.96779000
	C	-3.71624300	1.56089100	0.02461400
	C	-2.73130800	2.55601800	-0.02469900
	C	-3.05685000	3.89718700	0.15040200
	C	-4.37739100	4.27665700	0.38317900
	C	-5.36432100	3.29852900	0.43960300
	C	-5.03571600	1.95490200	0.26083500
	C	-2.85816700	-0.48085500	1.28747200
	C	-2.77108800	0.34334600	2.41410500
	C	-2.35399800	-0.15772100	3.64926100
	C	-2.01952000	-1.49832000	3.78862500
	C	-2.11437300	-2.33757700	2.67922400
	C	-2.53012600	-1.83712600	1.45115300
	H	-5.10729500	-1.62005300	1.26433700
	H	-7.08830000	-2.77799200	0.40482100
	H	-7.71944800	-2.58242400	-1.99107000
	H	-6.33793600	-1.16855500	-3.50028300
	H	-4.37072700	0.01572700	-2.63272600
	H	-1.69787300	2.29120400	-0.20623100
	H	-2.27425200	4.64599500	0.09962300
	H	-4.63220700	5.32230100	0.51477000
	H	-6.39740700	3.57543500	0.61849000
	H	-5.82133300	1.21214800	0.30610200
	H	-3.03731600	1.38842600	2.34190200
	H	-2.29802500	0.51049100	4.50151400
	H	-1.69479700	-1.88861800	4.74653900
	H	-1.86559900	-3.38926000	2.76988200
	H	-2.60618600	-2.50941900	0.60889200
	H	-0.44406200	-2.41712700	-3.98585700
	O	1.52041500	-1.39316700	-0.59118100
	C	2.49516700	-0.69541000	-1.21797500
	C	3.46241100	-0.00159500	-0.20332600
	C	3.90267700	-1.08851100	0.80849100
	C	3.95332500	-0.85747200	2.18315400
	O	2.59615400	-0.67185000	-2.41739200
	C	4.31774200	-2.33962800	0.33091000
	C	4.75901100	-3.33037000	1.19995200

	C	4.80190400	-3.08874700	2.57292000
	C	4.40108600	-1.84914200	3.05786700
	C	2.72951800	1.16864800	0.51889900
	C	3.37979000	2.39163300	0.72739900
	C	2.76589100	3.42644000	1.43147000
	C	1.48564600	3.26344700	1.94870900
	C	0.83086100	2.04889000	1.76082000
	C	1.44415300	1.01369200	1.05908900
	C	4.68226300	0.53925900	-0.98980800
	C	4.49174300	1.38445500	-2.09394800
	C	5.57379800	1.91999900	-2.78197400
	C	6.87809300	1.63267000	-2.38064700
	C	7.08208900	0.80902700	-1.28079700
	C	5.99400600	0.26874900	-0.59261400
	H	3.64182800	0.09855300	2.58235900
	H	4.31104800	-2.53663400	-0.73632800
	H	5.07211500	-4.29065000	0.80519600
	H	5.14504700	-3.85959700	3.25372700
	H	4.43084400	-1.64561200	4.12258900
	H	4.37827200	2.54512600	0.34319000
	H	3.29843200	4.36043600	1.57335700
	H	1.00613700	4.06747000	2.49582300
	H	-0.16194100	1.89302100	2.16641000
	H	0.92035300	0.07583700	0.94978600
	H	3.49017800	1.62700900	-2.41998200
	H	5.39645600	2.56489700	-3.63554800
	H	7.72173100	2.05024400	-2.91887300
	H	8.08863800	0.58010400	-0.94848700
	H	6.18152600	-0.36474200	0.26335800
	H	1.27717500	-2.64817400	-2.21919000
93	O	1.81843400	0.67956000	0.30696100
	C	2.72478500	0.53886800	-0.68400100
	C	0.46780700	1.07065000	-0.06597700
	H	0.26629000	0.67552100	-1.06273800
	C	-0.41541100	0.36163800	0.95876000
	H	-0.22041200	-0.70908800	0.94403000
	C	0.33204200	2.58526300	-0.05222300
	H	0.95470300	3.03124400	-0.82942300
	H	0.63058100	2.98703300	0.92004900
	O	2.48903100	0.79516800	-1.83593500
	C	4.04356000	-0.08436900	-0.14247100
	C	4.66020100	0.74592300	1.00817700
	C	5.76465200	0.22862200	1.69994000
	C	6.40754700	0.96642500	2.68719100
	C	5.97201100	2.25349400	2.99846400
	C	4.89129700	2.78787700	2.30701400
	C	4.24208900	2.04148700	1.32310500
	C	5.12097200	-0.12187900	-1.25322500
	C	5.36686000	1.01556100	-2.03311900
	C	6.39531700	1.03405800	-2.96840100
	C	7.21397300	-0.08074100	-3.13951900
	C	6.99247600	-1.21022200	-2.35991900

	C	5.95719500	-1.22883900	-1.42538400
	C	3.57544100	-1.49929600	0.29010400
	C	3.17125800	-2.40749300	-0.69908600
	C	2.66514900	-3.65805900	-0.35944100
	C	2.54608900	-4.02657000	0.97908700
	C	2.92620500	-3.12632200	1.96940800
	C	3.42779400	-1.87059400	1.62855900
	H	6.13291400	-0.76043400	1.45680400
	H	7.25666600	0.53757600	3.20769900
	H	6.47478400	2.83273800	3.76457300
	H	4.54493500	3.79133900	2.52864000
	H	3.40231500	2.48673700	0.80776700
	H	4.74731000	1.89422800	-1.91728900
	H	6.55733900	1.92473100	-3.56535900
	H	8.01556100	-0.06556100	-3.86941100
	H	7.62362100	-2.08472200	-2.47372400
	H	5.80453000	-2.12049800	-0.83137800
	H	3.24935500	-2.13020600	-1.74382300
	H	2.35852500	-4.34203200	-1.14273900
	H	2.15284500	-5.00105000	1.24551900
	H	2.82682200	-3.39415300	3.01555200
	H	3.69651100	-1.17774800	2.41420900
	H	-0.70700100	2.86699500	-0.23406300
	O	-1.80799400	0.61219500	0.67018600
	C	-2.47662500	-0.33572700	-0.02719900
	C	-4.00004500	-0.03759100	-0.05143400
	C	-4.72692600	-0.68536900	-1.25587500
	C	-6.12979500	-0.68871100	-1.24894800
	O	-1.95013000	-1.32302600	-0.46978700
	C	-4.07702900	-1.17107200	-2.39401300
	C	-4.80762700	-1.65380700	-3.48116000
	C	-6.19683900	-1.66157700	-3.45572000
	C	-6.85701400	-1.17400000	-2.32888100
	C	-4.28036800	1.47815900	-0.17635900
	C	-3.58744500	2.21944800	-1.14216800
	C	-3.87397300	3.56143100	-1.36391900
	C	-4.87403200	4.19578000	-0.62828400
	C	-5.58394600	3.46709200	0.31929600
	C	-5.29189300	2.12079500	0.54075500
	C	-4.42943500	-0.66795000	1.30332700
	C	-4.19398400	0.00156200	2.51246700
	C	-4.49776300	-0.59690300	3.73193100
	C	-5.02532300	-1.88553600	3.77128800
	C	-5.23209200	-2.57276800	2.57929500
	C	-4.93100100	-1.97271500	1.35823800
	H	-6.66224000	-0.30404400	-0.38753600
	H	-2.99923900	-1.20069900	-2.43597700
	H	-4.27700300	-2.02839500	-4.34956100
	H	-6.76041100	-2.03949300	-4.30131500
	H	-7.94075500	-1.16584200	-2.29162200
	H	-2.82326300	1.73982400	-1.74398000
	H	-3.32001600	4.11034900	-2.11738100

	H -5.10040800 5.24199900 -0.79940900<br>H -6.37306700 3.94173600 0.89203800<br>H -5.86364700 1.57505700 1.28000900<br>H -3.76379300 0.99416300 2.50073000<br>H -4.31589100 -0.05422700 4.65301200<br>H -5.26240400 -2.35148400 4.72109700<br>H -5.62457700 -3.58340300 2.59358700<br>H -5.08612300 -2.52749500 0.44278600<br>H -0.24701500 0.76198900 1.95915300

18 (optimized at the B3LYP-GD3BJ/6-311G(d,p) level)

Conformer no	
13	O 1.29751500 0.86228200 -1.34559100<br>C 2.41771100 0.15187300 -1.55496800<br>C 0.47984700 1.19589300 -2.49850800<br>H 1.14091200 1.30313300 -3.36133800<br>C -0.45512100 0.03329000 -2.79878200<br>H -1.07736400 0.26922700 -3.66231700<br>C -0.20730000 2.50692400 -2.16413300<br>H -0.89384300 2.78729100 -2.96434100<br>H 0.53843300 3.29397100 -2.04126100<br>O 2.74559300 -0.24961100 -2.64236400<br>C 3.18272600 -0.09016200 -0.22057000<br>C 2.19168000 -0.73930800 0.76334600<br>C 2.20971200 -0.43684600 2.12412600<br>C 1.36539400 -1.10406400 3.01100800<br>C 0.49059900 -2.08044300 2.54699900<br>C 0.46581900 -2.39043600 1.18847600<br>C 1.31296300 -1.72785200 0.30942100<br>C 3.68754000 1.26836600 0.33581000<br>C 5.02316700 1.44958700 0.70298600<br>C 5.45742800 2.64949500 1.26612400<br>C 4.56409600 3.69201700 1.47928900<br>C 3.22603000 3.51784800 1.13204100<br>C 2.79222900 2.32183900 0.57284400<br>C 4.36004700 -1.04429800 -0.49011300<br>C 5.27112100 -0.76589000 -1.51800600<br>C 6.37108400 -1.58460700 -1.73808000<br>C 6.59025900 -2.70138100 -0.93269200<br>C 5.69797100 -2.98372900 0.09376000<br>C 4.59259000 -2.16126500 0.31259100<br>H 2.88895800 0.31792800 2.49715200<br>H 1.39347800 -0.85422900 4.06551000<br>H -0.17800600 -2.59012400 3.22919600<br>H -0.22592200 -3.13366000 0.81476100<br>H 1.29467000 -1.99118900 -0.74158200<br>H 5.73586000 0.65081700 0.56024900<br>H 6.50039000 2.75978100 1.54018700<br>H 4.90200200 4.62489800 1.91557300<br>H 2.50932900 4.31381800 1.30106200<br>H 1.74841900 2.19973300 0.33353300

	H	5.11950000	0.09794000	-2.14876200
	H	7.05916400	-1.34988300	-2.54213100
	H	7.44815900	-3.34091900	-1.10563600
	H	5.85452400	-3.84617000	0.73148800
	H	3.91280100	-2.39823300	1.11868700
	H	-0.77307400	2.42470500	-1.23728600
	O	-1.29153500	-0.26704700	-1.66587100
	C	-2.53876000	0.24341900	-1.65235000
	C	-3.18920000	0.04009000	-0.26573200
	C	-2.96592700	-1.37434900	0.29336600
	C	-3.03468100	-1.60439900	1.66896000
	O	-3.02176600	0.85777500	-2.56820000
	C	-2.85485400	-2.47661400	-0.55691600
	C	-2.79940400	-3.77063900	-0.04771700
	C	-2.86027200	-3.98757400	1.32635000
	C	-2.98114600	-2.89604200	2.18200500
	C	-4.71753300	0.20770200	-0.31526200
	C	-5.46159700	-0.20329400	-1.42333800
	C	-6.85261200	-0.16042700	-1.40128600
	C	-7.52688200	0.28481600	-0.26834100
	C	-6.79492900	0.68282900	0.84678200
	C	-5.40483800	0.64082100	0.82230900
	C	-2.48403100	1.15721300	0.53684800
	C	-2.95685000	2.47161700	0.45360900
	C	-2.27226400	3.51301900	1.07268600
	C	-1.09678700	3.25754200	1.77558400
	C	-0.60571400	1.95664900	1.83951700
	C	-1.28615700	0.91585100	1.21364000
	H	-3.13217000	-0.76766100	2.34786600
	H	-2.81470200	-2.33294300	-1.62896500
	H	-2.71096400	-4.60985600	-0.72802500
	H	-2.81849700	-4.99466500	1.72417100
	H	-3.03766200	-3.04797900	3.25378900
	H	-4.95938400	-0.54670100	-2.31574000
	H	-7.40907000	-0.47667500	-2.27610400
	H	-8.61002200	0.31872700	-0.25336200
	H	-7.30442600	1.02599400	1.73990000
	H	-4.85022600	0.95155000	1.69805800
	H	-3.85832400	2.67974000	-0.10749000
	H	-2.65613200	4.52393200	0.99925100
	H	-0.56377900	4.06640700	2.26217200
	H	0.31647300	1.74228200	2.36361500
	H	-0.86906900	-0.07917000	1.24054000
	H	0.12218600	-0.86947800	-2.98832000
15	O	-1.54044500	-1.58042300	-1.34097600
	C	-1.65211500	-0.25278400	-1.16215000
	C	-0.60803600	-2.06275100	-2.34394200
	H	-0.24294600	-1.21165100	-2.91384000
	C	0.54041700	-2.71901700	-1.59591200
	H	0.16419400	-3.49789000	-0.93351200
	C	-1.34994500	-3.04565400	-3.23171300
	H	-2.17893600	-2.54381800	-3.73309400

	H	-0.68020600	-3.44562300	-3.99647400
	O	-1.08846000	0.55249500	-1.86090300
	C	-2.66773800	0.08642900	-0.03864200
	C	-3.98286200	0.36559100	-0.80782600
	C	-4.69708700	1.55176300	-0.64487100
	C	-5.88512300	1.76756200	-1.34285200
	C	-6.37403400	0.80427100	-2.21730300
	C	-5.66671000	-0.38448300	-2.38765600
	C	-4.48482600	-0.60108000	-1.68961700
	C	-2.24335200	1.32926500	0.78025500
	C	-1.52966000	2.40260000	0.23702600
	C	-1.23193200	3.52485500	1.00662700
	C	-1.66644000	3.61673000	2.32481400
	C	-2.39832800	2.56522400	2.86934100
	C	-2.67473100	1.43394600	2.10827300
	C	-2.80474200	-1.10928000	0.91994800
	C	-1.64900200	-1.61190900	1.52910900
	C	-1.72691700	-2.65069700	2.44545500
	C	-2.96416000	-3.20173300	2.78184100
	C	-4.11748400	-2.69698200	2.19454600
	C	-4.03741900	-1.65549600	1.26791300
	H	-4.33133600	2.31351600	0.02870600
	H	-6.42472600	2.69665300	-1.19951000
	H	-7.29555600	0.97521600	-2.76146200
	H	-6.03704500	-1.14663600	-3.06376800
	H	-3.95260100	-1.53485800	-1.81545600
	H	-1.20516400	2.36384700	-0.78889300
	H	-0.65800700	4.33020700	0.56270100
	H	-1.43949000	4.49447400	2.91905800
	H	-2.74956500	2.61664700	3.89367000
	H	-3.23302000	0.62429700	2.55654500
	H	-0.68216000	-1.18777400	1.29033800
	H	-0.81843400	-3.02470000	2.90125000
	H	-3.02461100	-4.01265500	3.49867900
	H	-5.08669900	-3.10783100	2.45348900
	H	-4.94461900	-1.26824600	0.82455800
	H	-1.75009800	-3.87542100	-2.64390600
	O	1.20898300	-1.79271400	-0.71978700
	C	2.11310800	-0.96819400	-1.28548100
	C	2.85550200	-0.07958900	-0.25271900
	C	2.90096500	1.36124900	-0.80237100
	C	1.82389200	1.88034400	-1.52501000
	O	2.32482700	-0.95687100	-2.47199900
	C	3.96977000	2.20661100	-0.49940200
	C	3.95836200	3.54049700	-0.90018200
	C	2.87912200	4.05069600	-1.61517700
	C	1.81242100	3.21199800	-1.92770600
	C	4.30796100	-0.61764100	-0.13420000
	C	5.01191400	-1.04854600	-1.26362100
	C	6.34261400	-1.44467300	-1.16564000
	C	7.00482900	-1.40244000	0.05764000
	C	6.32152600	-0.95257100	1.18261300

	C 4.98746300 -0.56457100 1.08738700<br>C 2.13779600 -0.11776200 1.11241000<br>C 2.15439400 -1.29193700 1.88029300<br>C 1.53893000 -1.34381800 3.12241700<br>C 0.87197400 -0.22492200 3.62324100<br>C 0.83629200 0.93791600 2.86746800<br>C 1.46967300 0.99233100 1.62460400<br>H 0.97712000 1.25780000 -1.77310200<br>H 4.81948200 1.82713200 0.05140700<br>H 4.79973100 4.17838300 -0.65448700<br>H 2.87230900 5.08767600 -1.93086200<br>H 0.96506800 3.58893900 -2.48910800<br>H 4.52209700 -1.05896900 -2.22646300<br>H 6.86356900 -1.78000900 -2.05520100<br>H 8.04169900 -1.70929200 0.13168700<br>H 6.82394400 -0.89958200 2.14175100<br>H 4.47750800 -0.21005600 1.97195800<br>H 2.66203600 -2.16870800 1.50069700<br>H 1.57646600 -2.26023500 3.70062100<br>H 0.37773400 -0.26854300 4.58656200<br>H 0.30676000 1.80996500 3.22686200<br>H 1.43198100 1.91183600 1.06012900<br>H 1.25659000 -3.13205300 -2.30758500
17	O 1.32156100 -0.92029100 1.34968300<br>C 2.43449800 -0.19400800 1.55357100<br>C 0.47237100 -1.18549300 2.50128900<br>H 1.11732100 -1.27529700 3.37813300<br>C -0.43703000 0.01075300 2.74767900<br>H -1.05602700 -0.16949300 3.62648100<br>C -0.24447000 -2.48842300 2.20342100<br>H -0.95158400 -2.72042000 3.00133500<br>H 0.48011500 -3.29984800 2.12083300<br>O 2.75506800 0.22477700 2.63655700<br>C 3.19297300 0.08179200 0.22374500<br>C 2.98740600 -1.06464000 -0.78105500<br>C 2.83927700 -0.81168500 -2.14465300<br>C 2.76776900 -1.85889400 -3.06332200<br>C 2.83091100 -3.17873500 -2.63034200<br>C 2.97402700 -3.44305700 -1.26884600<br>C 3.05917600 -2.39671600 -0.35893000<br>C 4.69287500 0.26028700 0.53846600<br>C 5.10396300 1.25150000 1.44092500<br>C 6.45082800 1.45337500 1.71278600<br>C 7.42232600 0.67620300 1.08352000<br>C 7.02783700 -0.29983100 0.17783400<br>C 5.67447000 -0.50467500 -0.09308400<br>C 2.59488600 1.39207200 -0.34676600<br>C 3.40884600 2.33139900 -0.98516300<br>C 2.85628500 3.46460100 -1.57856100<br>C 1.48215300 3.68062400 -1.53848700<br>C 0.66322200 2.75085700 -0.90405400<br>C 1.21452300 1.61548700 -0.31882600

	H	2.78469400	0.20863000	-2.49843100
	H	2.65691200	-1.63621100	-4.11839100
	H	2.76915400	-3.99339600	-3.34244700
	H	3.02490500	-4.46640800	-0.91542100
	H	3.19065900	-2.61981200	0.69237000
	H	4.36418100	1.86664700	1.93029600
	H	6.74263300	2.22387300	2.41725700
	H	8.47331600	0.83609400	1.29522900
	H	7.76935800	-0.90822900	-0.32721400
	H	5.39425300	-1.26458800	-0.80821900
	H	4.47818600	2.17647600	-1.02620200
	H	3.50693100	4.17782800	-2.07183900
	H	1.05137300	4.56341200	-1.99684400
	H	-0.40655000	2.90132000	-0.86438400
	H	0.55365300	0.90005300	0.14662500
	H	-0.78882600	-2.42466200	1.26314700
	O	-1.28201200	0.27147200	1.60895500
	C	-2.54556100	-0.20251700	1.63598400
	C	-3.20730200	-0.04537200	0.24776200
	C	-4.73844200	-0.18111000	0.30872700
	C	-5.43212400	-0.61330400	-0.82570500
	O	-3.03113100	-0.75391400	2.58903200
	C	-5.47559800	0.25071100	1.41313600
	C	-6.86756400	0.22939900	1.39001000
	C	-7.54826400	-0.21440400	0.26071300
	C	-6.82225600	-0.63371100	-0.85086200
	C	-2.53334800	-1.21211500	-0.50860600
	C	-3.03513300	-2.50929900	-0.35585600
	C	-2.36380500	-3.59936100	-0.90177500
	C	-1.16855900	-3.41144000	-1.59147200
	C	-0.65001600	-2.12740900	-1.72911700
	C	-1.32668000	-1.03755100	-1.19063600
	C	-2.96073800	1.34520400	-0.35844700
	C	-2.89842000	2.47407600	0.46261600
	C	-2.83596200	3.75302100	-0.08150200
	C	-2.83437400	3.92799500	-1.46302900
	C	-2.89720200	2.81037800	-2.29068800
	C	-2.96557300	1.53286800	-1.74240200
	H	-4.88093600	-0.94079500	-1.69768400
	H	-4.96880600	0.59067400	2.30384300
	H	-7.41932800	0.56122800	2.26202700
	H	-8.63180000	-0.23136500	0.24547400
	H	-7.33677100	-0.97666000	-1.74116300
	H	-3.94658100	-2.66569600	0.20583600
	H	-2.77005800	-4.59601900	-0.77332800
	H	-0.63464000	-4.25866200	-2.00579200
	H	0.29268800	-1.97269400	-2.23545600
	H	-0.89580400	-0.05164000	-1.28478700
	H	-2.90593900	2.36202900	1.53973900
	H	-2.78526900	4.61257400	0.57655400
	H	-2.78376800	4.92320300	-1.88859100
	H	-2.89713900	2.92996800	-3.36781600

	H	-3.02880700	0.67644800	-2.40044000
	H	0.15719400	0.91195900	2.88547500
26	O	-1.36439800	0.02227600	-1.69369000
	C	-2.68015500	-0.23500200	-1.65305700
	C	-0.68024200	-0.04102900	-2.96519900
	H	-1.31329400	0.43057300	-3.72130900
	C	0.56596500	0.79548000	-2.77393300
	H	1.17563100	0.80668300	-3.67860100
	C	-0.39934100	-1.48969300	-3.33928200
	H	0.17522700	-1.53195200	-4.26827900
	H	-1.33650900	-2.02427700	-3.49211900
	O	-3.34373700	-0.49888200	-2.62285300
	C	-3.19963300	-0.02844900	-0.21148400
	C	-2.28007400	-0.68892600	0.83382900
	C	-2.33740300	-0.27147400	2.16629500
	C	-1.58378000	-0.90581400	3.14852000
	C	-0.75381100	-1.97383200	2.81566700
	C	-0.70300600	-2.41060200	1.49592500
	C	-1.46574100	-1.77840500	0.51883600
	C	-4.58307000	-0.66079700	0.00178100
	C	-4.92718200	-1.86929700	-0.60893100
	C	-6.13196400	-2.49921600	-0.31244100
	C	-7.01290600	-1.93942400	0.60821900
	C	-6.67344700	-0.74360500	1.23390500
	C	-5.46931500	-0.11404400	0.93431600
	C	-3.20544600	1.51481400	-0.13040500
	C	-4.28020100	2.24290600	-0.64854400
	C	-4.24486000	3.63472500	-0.67651100
	C	-3.12782100	4.31864900	-0.20382900
	C	-2.03838100	3.59855200	0.28110400
	C	-2.07450000	2.20845200	0.30782900
	H	-2.97275200	0.56142300	2.43874100
	H	-1.64364700	-0.56152800	4.17484900
	H	-0.15221800	-2.45942100	3.57485400
	H	-0.05383500	-3.23061800	1.21781900
	H	-1.40218300	-2.13789900	-0.49832900
	H	-4.26016800	-2.32078300	-1.32861500
	H	-6.38079300	-3.43219000	-0.80505600
	H	-7.95147100	-2.43057000	0.83727000
	H	-7.34484000	-0.29693500	1.95834000
	H	-5.22110600	0.81511500	1.42997700
	H	-5.14467100	1.71910600	-1.03512700
	H	-5.09099500	4.18279100	-1.07469200
	H	-3.10215100	5.40208700	-0.22481100
	H	-1.14999700	4.11068800	0.63241900
	H	-1.20996000	1.66328400	0.65520600
	H	0.17275900	-1.97726700	-2.54945000
	O	1.30994800	0.21765200	-1.68527900
	C	2.58889400	0.60696200	-1.54559300
	C	3.15374500	0.14178300	-0.18425400
	C	2.47532000	1.15092600	0.77096400
	C	1.36844700	0.81486300	1.55137200

	O	3.15988200	1.31028100	-2.33828400
	C	2.91762500	2.47955100	0.76643300
	C	2.29830800	3.43852000	1.56023600
	C	1.22175700	3.08496100	2.37133600
	C	0.75909300	1.77297600	2.35943000
	C	4.68874100	0.24103500	-0.15756000
	C	5.44457000	-0.16753800	-1.25947900
	C	6.83336700	-0.19377100	-1.19918900
	C	7.49448300	0.17763000	-0.03098000
	C	6.75083700	0.57296800	1.07607400
	C	5.36019600	0.60289900	1.01199100
	C	2.84191500	-1.32987800	0.13500500
	C	2.99556600	-1.79448700	1.44510200
	C	2.84378400	-3.14146500	1.75152200
	C	2.54357000	-4.06070000	0.74864500
	C	2.40397300	-3.61399300	-0.56121000
	C	2.55619100	-2.26243300	-0.86346300
	H	0.96763800	-0.18805000	1.53020700
	H	3.75073200	2.76000300	0.13441500
	H	2.65938000	4.46037900	1.54569000
	H	0.74303700	3.82697700	2.99997300
	H	-0.08921500	1.48498700	2.96665600
	H	4.94995000	-0.45993800	-2.17527600
	H	7.40003900	-0.50573300	-2.06895400
	H	8.57708300	0.15729700	0.01514300
	H	7.25019900	0.86064300	1.99417700
	H	4.79615100	0.91667500	1.88037300
	H	3.24462300	-1.09691400	2.23377400
	H	2.96659900	-3.47389700	2.77583300
	H	2.42798900	-5.11199300	0.98497700
	H	2.18230400	-4.31683500	-1.35616600
	H	2.45620900	-1.94269100	-1.89130100
	H	0.30627700	1.82149400	-2.50569000
32	O	-1.20090100	-1.58910500	-1.10106900
	C	-2.06290400	-0.62888800	-1.48114800
	C	-0.57517400	-2.38293300	-2.14383000
	H	-1.30594100	-2.52674300	-2.94344900
	C	0.60359100	-1.62155800	-2.73374900
	H	1.11997600	-2.24955800	-3.46125300
	C	-0.18938800	-3.69892300	-1.49365000
	H	0.24511200	-4.36841400	-2.24020700
	H	-1.07117600	-4.18077300	-1.06850200
	O	-2.26341300	-0.35325200	-2.63761600
	C	-2.78951600	0.03935600	-0.28247800
	C	-4.25265200	-0.48700800	-0.28672500
	C	-4.93487800	-0.72335400	0.91106200
	C	-6.27514700	-1.10283600	0.90942900
	C	-6.96206400	-1.25321900	-0.29052000
	C	-6.29742600	-1.00263100	-1.48763900
	C	-4.96116800	-0.61480300	-1.48647000
	C	-2.81840500	1.56553100	-0.50429100
	C	-3.83723100	2.33659700	0.06022400

	C	-3.81298800	3.72555700	-0.02794900
	C	-2.76973700	4.36953900	-0.68732200
	C	-1.75464500	3.60832000	-1.25945400
	C	-1.77893700	2.21928600	-1.16934700
	C	-2.07646900	-0.29839800	1.04314500
	C	-1.38652400	0.66831900	1.77207600
	C	-0.76032200	0.34802700	2.97794800
	C	-0.82490100	-0.94369600	3.48028500
	C	-1.51031300	-1.92242800	2.75919900
	C	-2.11734700	-1.60503800	1.55274100
	H	-4.42395900	-0.60288200	1.85569400
	H	-6.77934800	-1.27829700	1.85296000
	H	-8.00349800	-1.55346900	-0.29283200
	H	-6.82118000	-1.10017500	-2.43174200
	H	-4.46756600	-0.39606400	-2.42232500
	H	-4.65661900	1.85314600	0.57461400
	H	-4.61496800	4.30285400	0.41784300
	H	-2.75224200	5.45096900	-0.75976500
	H	-0.93683100	4.09118200	-1.78235000
	H	-0.97367600	1.65672300	-1.61634700
	H	-1.32549700	1.68239800	1.40698100
	H	-0.21568200	1.11731900	3.50890600
	H	-0.33912000	-1.19380700	4.41608700
	H	-1.56923900	-2.93707400	3.13699200
	H	-2.63911500	-2.37549300	1.00133200
	H	0.54158700	-3.53619000	-0.70175400
	O	1.55834400	-1.33537600	-1.69174900
	C	1.65390500	-0.05717000	-1.27315000
	C	2.69247100	0.09349700	-0.13134500
	C	2.27973400	1.17412300	0.89829300
	C	2.74449800	1.05536600	2.21398200
	O	1.06019900	0.84735900	-1.80293200
	C	1.53656800	2.31126600	0.56681300
	C	1.23638000	3.27402000	1.52778400
	C	1.69952500	3.14192800	2.83250600
	C	2.46551800	2.02875900	3.16775700
	C	3.98833800	0.50988100	-0.87018100
	C	4.47838200	-0.29143900	-1.91024400
	C	5.64265500	0.05206600	-2.58653300
	C	6.34284200	1.20523700	-2.23681200
	C	5.86351900	2.00669500	-1.20740100
	C	4.69351000	1.66380500	-0.53048300
	C	2.85161800	-1.24648500	0.60771300
	C	4.08702500	-1.85597000	0.80993100
	C	4.18395200	-3.03641300	1.54908700
	C	3.04608000	-3.61696100	2.09539600
	C	1.80677500	-3.00415200	1.90619800
	C	1.71226800	-1.82933600	1.17392100
	H	3.32997200	0.19324900	2.50098600
	H	1.18648100	2.44509700	-0.44218700
	H	0.63493700	4.13017700	1.24428600
	H	1.46899900	3.89520700	3.57713700

	H	2.84153900	1.90808300	4.17736800
	H	3.95009400	-1.19650300	-2.18129500
	H	6.00404000	-0.58267800	-3.38749800
	H	7.25064900	1.47489300	-2.76380800
	H	6.39621700	2.90765600	-0.92560400
	H	4.33254100	2.30147800	0.26376500
	H	4.98321600	-1.41094600	0.39994800
	H	5.15491200	-3.49543700	1.69655500
	H	3.12069600	-4.53389600	2.66866800
	H	0.91002800	-3.43521500	2.33363200
	H	0.74573700	-1.35907100	1.04702600
	H	0.27897100	-0.69596700	-3.20151700
35	O	-1.38964300	-0.35074000	-1.74880100
	C	-2.60945100	0.21330100	-1.68071900
	C	-0.54473800	-0.00535400	-2.87771200
	H	-1.17951500	0.40946900	-3.66258300
	C	0.39791400	1.09398500	-2.41282200
	H	1.05848500	1.39958700	-3.22450900
	C	0.13141200	-1.28040700	-3.34498500
	H	0.83359300	-1.06157300	-4.15074800
	H	-0.61610400	-1.98864200	-3.70645400
	O	-3.11669200	0.83187600	-2.58143400
	C	-3.19217000	0.09319100	-0.25620500
	C	-2.44812600	1.26299700	0.43093400
	C	-1.30494600	1.06125500	1.20489700
	C	-0.62985900	2.14595900	1.76089900
	C	-1.05926300	3.44611100	1.51490200
	C	-2.17054500	3.65803800	0.70055200
	C	-2.85800000	2.57531400	0.16362200
	C	-2.95479900	-1.27544000	0.40180400
	C	-2.78480200	-2.43840400	-0.35181300
	C	-2.70634000	-3.68547700	0.26318700
	C	-2.80149000	-3.79432300	1.64710200
	C	-2.98474200	-2.64219700	2.40789300
	C	-3.06538900	-1.39977700	1.78984400
	C	-4.71828500	0.28021400	-0.25978300
	C	-5.50267100	-0.27049800	-1.27575700
	C	-6.89080600	-0.20431900	-1.21814800
	C	-7.52209800	0.40642500	-0.13791500
	C	-6.74997800	0.94774000	0.88528300
	C	-5.36096500	0.88263900	0.82417900
	H	-0.92944700	0.06391100	1.37468700
	H	0.23706800	1.96072800	2.38164800
	H	-0.53067200	4.28762100	1.94819500
	H	-2.50623700	4.66592400	0.48552200
	H	-3.71663600	2.74431700	-0.47324800
	H	-2.70909000	-2.38125900	-1.42864800
	H	-2.57159100	-4.57279500	-0.34468400
	H	-2.73955900	-4.76420800	2.12652500
	H	-3.07140600	-2.70958900	3.48625500
	H	-3.22027600	-0.51673800	2.39552400
	H	-5.03200500	-0.74589100	-2.12505500

	H	-7.47962400	-0.63128400	-2.02182900
	H	-8.60372300	0.45855400	-0.09409300
	H	-7.22646000	1.42283400	1.73516000
	H	-4.77447800	1.31101900	1.62650500
	H	0.68081500	-1.74960200	-2.52880000
	O	1.18055700	0.65583800	-1.28773700
	C	2.45330100	0.27782400	-1.50709100
	C	3.17399700	0.00722100	-0.16716100
	C	4.28774200	-1.05070900	-0.27687800
	C	5.08264500	-1.27040200	0.85617300
	O	2.96622600	0.25801000	-2.59704000
	C	4.49456300	-1.85966600	-1.39442500
	C	5.47631900	-2.85147700	-1.38104200
	C	6.26680100	-3.05131700	-0.25667400
	C	6.06433300	-2.25156000	0.86722200
	C	2.20248200	-0.52970000	0.90052400
	C	1.35104100	-1.58955000	0.57350200
	C	0.52266400	-2.16606900	1.52575800
	C	0.54372600	-1.70287700	2.84063600
	C	1.40238300	-0.66534500	3.18486400
	C	2.22536900	-0.08265100	2.22061000
	C	3.72486200	1.41731400	0.15117600
	C	2.83812600	2.43380900	0.52855100
	C	3.29456900	3.72673200	0.75481900
	C	4.64459000	4.03242100	0.59263200
	C	5.52635100	3.03558500	0.18847800
	C	5.06921400	1.73859900	-0.03845500
	H	4.92582000	-0.66813000	1.74196600
	H	3.91195600	-1.70781000	-2.28910600
	H	5.61954000	-3.46579700	-2.26269100
	H	7.02962500	-3.82117200	-0.25110600
	H	6.66691600	-2.39691400	1.75644200
	H	1.33297500	-1.97089000	-0.43938700
	H	-0.15203000	-2.96328800	1.24245300
	H	-0.11054800	-2.14596100	3.58127700
	H	1.43407200	-0.29957100	4.20494700
	H	2.88451600	0.72718600	2.50398700
	H	1.78537200	2.21173700	0.63167100
	H	2.59115100	4.49684700	1.05048600
	H	5.00228100	5.04028800	0.76859600
	H	6.57559900	3.26411400	0.04132600
	H	5.76252500	0.97645100	-0.36570300
	H	-0.17613400	1.94572800	-2.04918600
36	O	-1.26537300	-1.43675300	-0.45649400
	C	-1.99408000	-0.47555800	-1.05062300
	C	-0.46689600	-2.29567100	-1.30842900
	H	-0.02723700	-1.68640200	-2.09719300
	C	0.61541000	-2.86124700	-0.41224700
	H	0.17653200	-3.30546700	0.48027000
	C	-1.35513000	-3.37996800	-1.89995900
	H	-1.83266800	-3.96117000	-1.10776700
	H	-0.76698000	-4.04933700	-2.53200300

	O	-1.89131800	-0.17774500	-2.21279500	
	C	-3.05940000	0.06230100	-0.06495200	
	C	-2.45457900	0.46968700	1.28748600	
	C	-3.29100500	0.61500400	2.39909700	
	C	-2.79588100	1.08575700	3.60954200	
	C	-1.45288800	1.43787800	3.72629100	
	C	-0.61842700	1.31908100	2.62011900	
	C	-1.11420900	0.83801700	1.41000100	
	C	-4.00463000	-1.15759200	0.00496800	
	C	-4.85230100	-1.42241300	-1.07625900	
	C	-5.62583200	-2.57841300	-1.10910400	
	C	-5.55643900	-3.49696000	-0.06459400	
	C	-4.69809100	-3.25298000	1.00395000	
	C	-3.92126400	-2.09789000	1.03363800	
	C	-3.74972100	1.32567200	-0.60084000	
	C	-5.10072200	1.56691000	-0.34132000	
	C	-5.69816300	2.76631400	-0.72068400	
	C	-4.95154900	3.74840000	-1.36364400	
	C	-3.60034900	3.52176300	-1.61388600	
	C	-3.00475700	2.32537500	-1.23070500	
	H	-4.34034800	0.36201100	2.31589900	
	H	-3.46200800	1.18482400	4.45897100	
	H	-1.06401400	1.80959700	4.66723000	
	H	0.42406200	1.60079900	2.68978400	
	H	-0.44295400	0.75818100	0.56627800	
	H	-4.89785300	-0.72139100	-1.89999200	
	H	-6.27912200	-2.76179800	-1.95427700	
	H	-6.15992000	-4.39693000	-0.08694600	
	H	-4.62542700	-3.96656200	1.81680700	
	H	-3.23859100	-1.93356700	1.85563800	
	H	-5.69616000	0.81331300	0.15731100	
	H	-6.74902700	2.92999500	-0.51096200	
	H	-5.41553700	4.68098100	-1.66306600	
	H	-3.00498800	4.28005800	-2.11013500	
	H	-1.95484500	2.16970600	-1.42236000	
	H	-2.13527800	-2.92771600	-2.51235900	
	O	1.47597800	-1.81497400	0.07274000	
	C	2.50270000	-1.45812300	-0.71907800	
	C	3.24323800	-0.20400600	-0.17658800	
	C	3.54931000	-0.33607900	1.33635200	
	C	4.85497700	-0.25261100	1.82430200	
	O	2.75020500	-2.01388100	-1.75857000	
	C	2.50749600	-0.46372600	2.26742900	
	C	2.76762200	-0.52451600	3.63033100	
	C	4.07713400	-0.45573600	4.10336600	
	C	5.11700800	-0.31697700	3.19343700	
	C	2.28590100	0.98171400	-0.43648300	
	C	1.52760100	1.02352400	-1.61034500	
	C	0.70325100	2.10813700	-1.89045200	
	C	0.63542900	3.18181200	-1.00597400	
	C	1.40267900	3.15983000	0.15428100	
	C	2.22063900	2.06838100	0.43752900	

	C	4.53734900	-0.01732300	-0.98586700
	C	4.92083600	1.23330500	-1.46816300
	C	6.13359900	1.40114700	-2.13643300
	C	6.98133600	0.31846000	-2.33457800
	C	6.60785600	-0.93686800	-1.85663100
	C	5.40067300	-1.10073500	-1.18975000
	H	5.68120000	-0.12691200	1.14030900
	H	1.48607100	-0.50863900	1.92588100
	H	1.93909100	-0.61989200	4.32305300
	H	4.27996900	-0.50307900	5.16707500
	H	6.14169800	-0.25100800	3.54083100
	H	1.59067400	0.21124700	-2.32338100
	H	0.10956200	2.10261600	-2.79636200
	H	-0.01368100	4.02372200	-1.21467400
	H	1.36001100	3.98969000	0.84980700
	H	2.81228900	2.06459200	1.34292800
	H	4.27442000	2.08822600	-1.32620900
	H	6.40842600	2.38336100	-2.50338100
	H	7.92186600	0.44685400	-2.85769100
	H	7.25732600	-1.79177800	-2.00559300
	H	5.12870000	-2.08131300	-0.82326400
	H	1.20442800	-3.59845300	-0.95669900
46	O	-1.35443300	-0.92774400	-1.27755600
	C	-2.53393900	-0.34851800	-1.56973800
	C	-0.52075100	-1.37562500	-2.37664000
	H	-1.16310700	-1.53751400	-3.24458300
	C	0.43827000	-0.24882300	-2.72972900
	H	1.07212800	-0.54134600	-3.56647900
	C	0.13331600	-2.67003700	-1.93001300
	H	-0.63484300	-3.42110700	-1.73906200
	H	0.70378100	-2.52383200	-1.01246800
	O	-2.94642300	-0.21094700	-2.69351300
	C	-3.27854800	0.00338000	-0.26177800
	C	-2.29255100	0.62190100	0.74584600
	C	-2.34926500	0.35418100	2.11273600
	C	-1.49651900	1.01020900	2.99980500
	C	-0.57696000	1.94176500	2.53214200
	C	-0.52558800	2.22988300	1.17001100
	C	-1.38175700	1.58091500	0.29203300
	C	-3.84179800	-1.37600300	0.15096600
	C	-5.14889200	-1.73901600	-0.18159300
	C	-5.63028700	-3.01267200	0.11258200
	C	-4.80892400	-3.94851100	0.73259800
	C	-3.49402900	-3.60586900	1.04116700
	C	-3.01325500	-2.33550900	0.74437600
	C	-4.38966400	1.05591600	-0.43212400
	C	-5.25375800	1.25994100	0.65211300
	C	-6.22947300	2.24656300	0.61963900
	C	-6.35841100	3.06568300	-0.50092900
	C	-5.49903900	2.88147000	-1.57648800
	C	-4.52073800	1.88686700	-1.54522800
	H	-3.05577600	-0.37215500	2.49239800

	H	-1.54902100	0.78101400	4.05802200
	H	0.10049600	2.43701000	3.21677900
	H	0.19125100	2.94591500	0.79084900
	H	-1.33665200	1.82531000	-0.76290100
	H	-5.79443600	-1.02357400	-0.67229600
	H	-6.64961800	-3.27126900	-0.14975400
	H	-5.18475000	-4.93837500	0.96400100
	H	-2.83640600	-4.33010600	1.50819300
	H	-1.98291200	-2.09124200	0.95704700
	H	-5.15880700	0.63849600	1.53346300
	H	-6.88599700	2.37953000	1.47185900
	H	-7.11785700	3.83835800	-0.53036100
	H	-5.58445500	3.51150500	-2.45455300
	H	-3.87909500	1.75176600	-2.40092400
	H	0.80861400	-3.03670700	-2.70458000
	O	1.26262200	0.11430700	-1.60339700
	C	2.50885600	-0.40190200	-1.55580600
	C	3.24186900	-0.00675500	-0.24186800
	C	4.75591600	-0.22236700	-0.44406200
	C	5.67988200	0.79017700	-0.18120200
	O	2.95820600	-1.10255400	-2.42424700
	C	5.24491700	-1.46788700	-0.86489300
	C	6.60765700	-1.68439500	-1.02303300
	C	7.51948700	-0.66280600	-0.76213800
	C	7.04894700	0.57319300	-0.33899100
	C	2.67537500	-0.92579800	0.87647300
	C	1.29845100	-0.93827100	1.13849400
	C	0.78178300	-1.70222900	2.17922000
	C	1.62478900	-2.46503100	2.98348300
	C	2.99352700	-2.44574200	2.74182000
	C	3.51446500	-1.68033100	1.69949800
	C	2.96614100	1.46309000	0.11417900
	C	2.93271700	1.87308100	1.44734700
	C	2.81187700	3.22095800	1.77661700
	C	2.70767300	4.18170200	0.77561000
	C	2.73751200	3.78233500	-0.55941300
	C	2.87405400	2.43663300	-0.88385000
	H	5.34097700	1.76052200	0.15107100
	H	4.55527700	-2.27257900	-1.07059600
	H	6.95801300	-2.65600000	-1.35210200
	H	8.58277100	-0.83261700	-0.88667000
	H	7.74258100	1.37914200	-0.12853600
	H	0.62786000	-0.33558100	0.54555200
	H	-0.28294400	-1.67766500	2.37133600
	H	1.21951300	-3.05768100	3.79559900
	H	3.66705000	-3.02151700	3.36628700
	H	4.58262300	-1.66690600	1.54003400
	H	3.00535800	1.13581500	2.23534500
	H	2.79387200	3.51719200	2.81914700
	H	2.60460600	5.22994200	1.03042800
	H	2.66150700	4.51998400	-1.34990400
	H	2.92062600	2.14657800	-1.92639300

	H	-0.12201200	0.65069300	-2.98095500
60	O	-1.45669600	-2.07700700	-0.22247600
	C	-1.85413700	-0.97764200	-0.88200800
	C	-0.68184400	-3.07831600	-0.92771100
	H	-0.56287400	-2.75167700	-1.96055400
	C	0.67512100	-3.17943600	-0.25392200
	H	0.55888800	-3.26749700	0.82598300
	C	-1.45570100	-4.38499900	-0.84549900
	H	-0.91513600	-5.18435200	-1.35796300
	H	-2.43129800	-4.27171100	-1.32031000
	O	-1.58291300	-0.76481800	-2.03818900
	C	-2.72678900	-0.02659700	-0.01270600
	C	-3.98739000	0.26847500	-0.85079700
	C	-4.55955700	1.53853300	-0.89001500
	C	-5.74325000	1.76362200	-1.59168000
	C	-6.37000500	0.72225400	-2.26681300
	C	-5.80597100	-0.55185600	-2.23244000
	C	-4.62856100	-0.77325300	-1.52887900
	C	-1.93646400	1.28888200	0.21530900
	C	-1.23387000	1.89250700	-0.83307300
	C	-0.60025100	3.11809100	-0.65573200
	C	-0.67554300	3.77969700	0.56614900
	C	-1.39165100	3.20066600	1.60857900
	C	-2.01233600	1.96502300	1.43711500
	C	-3.10960800	-0.66738600	1.33603100
	C	-2.10482600	-1.02474200	2.24707300
	C	-2.42623600	-1.56514200	3.48436800
	C	-3.75998800	-1.75625200	3.84734500
	C	-4.76292400	-1.39484900	2.95798100
	C	-4.43935700	-0.85186900	1.71287900
	H	-4.08268400	2.35921000	-0.37192800
	H	-6.17053500	2.75963700	-1.61077800
	H	-7.28700700	0.89903400	-2.81678100
	H	-6.28318600	-1.37350800	-2.75411500
	H	-4.21025900	-1.77277400	-1.49869300
	H	-1.18818000	1.40626100	-1.79453800
	H	-0.04128700	3.54599100	-1.47775800
	H	-0.17668200	4.73149500	0.70557800
	H	-1.46996000	3.70579300	2.56466400
	H	-2.56531300	1.53498200	2.25950500
	H	-1.06466200	-0.88185600	1.99144600
	H	-1.62971500	-1.83360600	4.16906900
	H	-4.00981500	-2.17624500	4.81506500
	H	-5.80497400	-1.52720600	3.22608400
	H	-5.23761200	-0.56836700	1.04197800
	H	-1.61367700	-4.67428400	0.19583500
	O	1.50013900	-2.03990000	-0.55922800
	C	1.66845100	-1.09945100	0.39646600
	C	2.74623300	-0.05905700	-0.01611300
	C	2.32375900	0.79068300	-1.23752600
	C	2.86892800	2.07371100	-1.37759700
	O	1.10582700	-1.13498500	1.45864200

	C	1.48209300	0.32234700	-2.24731100
	C	1.18113900	1.11442800	-3.35351900
	C	1.73367300	2.38276800	-3.48578800
	C	2.58523300	2.85729600	-2.49043800
	C	4.00731500	-0.88950600	-0.33304800
	C	4.43403600	-1.84842900	0.59451000
	C	5.57098400	-2.61105300	0.36090900
	C	6.30752000	-2.42722500	-0.80905000
	C	5.89074800	-1.47836900	-1.73507200
	C	4.74608300	-0.71581300	-1.50048400
	C	2.97239700	0.88077300	1.18238200
	C	4.23459900	1.10314400	1.72947700
	C	4.40740100	2.01194800	2.77497900
	C	3.31868500	2.70875000	3.28372600
	C	2.05452100	2.50037400	2.73218000
	C	1.88438600	1.60060500	1.68971500
	H	3.52102900	2.46446100	-0.60871200
	H	1.04491800	-0.65893800	-2.18325800
	H	0.50255600	0.72909500	-4.10530700
	H	1.50188300	2.99637100	-4.34874700
	H	3.02545900	3.84453500	-2.57322800
	H	3.87591000	-1.98593000	1.51423500
	H	5.88452100	-3.34677800	1.09267600
	H	7.19561700	-3.02022300	-0.99409600
	H	6.45299100	-1.32736200	-2.64947600
	H	4.43140400	0.01340600	-2.23386700
	H	5.09450200	0.57642100	1.33956600
	H	5.39818200	2.17111800	3.18514700
	H	3.45078300	3.41174000	4.09831700
	H	1.19543400	3.04051800	3.11079100
	H	0.90088600	1.46568700	1.26299800
	H	1.21676700	-4.03976100	-0.64855400
63	O	1.24626300	-1.76990700	-0.71342500
	C	1.98290900	-0.78886700	-1.26259700
	C	0.59389800	-2.75784600	-1.55973600
	H	0.15744000	-3.42440100	-0.81469300
	C	-0.54723700	-2.13346900	-2.34939400
	H	-0.20569900	-1.32003800	-2.98390300
	C	1.57426300	-3.52971200	-2.43343200
	H	1.08289300	-4.42501100	-2.82271300
	H	2.43061300	-3.84646500	-1.83509300
	O	2.09375700	-0.62323400	-2.45118500
	C	2.72223300	0.06039500	-0.19225300
	C	4.17142700	-0.49440000	-0.12368100
	C	4.87321800	-0.52527000	1.08536400
	C	6.20273300	-0.93748800	1.13147200
	C	6.85831700	-1.32833800	-0.03151600
	C	6.17407800	-1.28572700	-1.24279400
	C	4.84818000	-0.86527500	-1.29067200
	C	2.01184000	-0.04501400	1.17455200
	C	2.05563200	-1.24183400	1.90593000
	C	1.44243500	-1.34686800	3.14617200

	C	0.75115700	-0.26038400	3.68298100	
	C	0.68851300	0.92454000	2.96406800	
	C	1.31726500	1.03241000	1.72258100	
	C	2.77613200	1.52891800	-0.65848400	
	C	1.72293500	2.09452200	-1.38179100	
	C	1.72547300	3.44984500	-1.69775100	
	C	2.77968300	4.26612300	-1.29709300	
	C	3.83466600	3.71003000	-0.58001300	
	C	3.83290500	2.35337900	-0.26630500	
	H	4.38371400	-0.21751700	1.99860600	
	H	6.72366200	-0.94923100	2.08208300	
	H	7.89139600	-1.65415300	0.00407500	
	H	6.67428900	-1.57260200	-2.16075600	
	H	4.34252600	-0.80614900	-2.24418900	
	H	2.58204100	-2.09441000	1.49972000	
	H	1.50269000	-2.27995200	3.69516100	
	H	0.26152600	-0.34491000	4.64598800	
	H	0.14376500	1.77415900	3.35303800	
	H	1.25689500	1.96839600	1.18800000	
	H	0.89120400	1.48596500	-1.70149100	
	H	0.89798500	3.86394400	-2.26290800	
	H	2.78228400	5.32112900	-1.54621100	
	H	4.66649200	4.32971300	-0.26496300	
	H	4.66329800	1.93905300	0.28910800	
	H	1.93380000	-2.92712500	-3.26519800	
	O	-1.54446200	-1.65893500	-1.42216600	
	C	-1.64688900	-0.32899600	-1.23168400	
	C	-2.70726000	0.00630800	-0.14860900	
	C	-4.00589800	0.25679200	-0.95535100	
	C	-4.75659600	1.42155200	-0.79864800	
	O	-1.03622400	0.47222100	-1.89200700	
	C	-4.46058500	-0.71470900	-1.85692100	
	C	-5.63151500	-0.52318700	-2.58052700	
	C	-6.37515300	0.64390100	-2.41643500	
	C	-5.93333400	1.61192300	-1.52223900	
	C	-2.32643500	1.26409000	0.66857700	
	C	-1.65434700	2.36018300	0.11703200	
	C	-1.38856800	3.49386500	0.88077000	
	C	-1.81938900	3.57597200	2.20095200	
	C	-2.51587400	2.50430900	2.75180600	
	C	-2.75738200	1.36067200	1.99696500	
	C	-2.85067800	-1.18353700	0.81620000	
	C	-1.70330500	-1.66082900	1.46078900	
	C	-1.78833500	-2.69338300	2.38360100	
	C	-3.02488000	-3.26320600	2.69019400	
	C	-4.17011200	-2.78368700	2.06693100	
	C	-4.08275600	-1.74844200	1.13397400	
	H	-4.42777300	2.18608200	-0.10946900	
	H	-3.89982300	-1.63201700	-1.97981300	
	H	-5.96428400	-1.28825200	-3.27264500	
	H	-7.28795200	0.79474500	-2.98087200	
	H	-6.50128000	2.52461700	-1.38318100	

	H	-1.33348900	2.32762800	-0.90996100
	H	-0.84146800	4.31484000	0.43181600
	H	-1.61757600	4.46248600	2.79122900
	H	-2.86537400	2.54868600	3.77708600
	H	-3.28655000	0.53537700	2.45164600
	H	-0.73765600	-1.22032700	1.24577400
	H	-0.88668800	-3.04705800	2.86821300
	H	-3.09121400	-4.06903700	3.41222300
	H	-5.13871200	-3.20971900	2.30256400
	H	-4.98347000	-1.38068200	0.66170100
	H	-1.03437300	-2.90448500	-2.94763000
77	O	-1.54479000	-1.77605400	-1.05627600
	C	-1.47578700	-0.44089800	-0.89052800
	C	-0.59588400	-2.39934400	-1.96332200
	H	-0.23433600	-1.63761200	-2.65069600
	C	0.57048100	-2.94700500	-1.15522500
	H	0.21841900	-3.66269400	-0.41214400
	C	-1.34767900	-3.49756500	-2.69342600
	H	-2.20528000	-3.07440500	-3.21768500
	H	-1.71426100	-4.25343600	-1.99442600
	O	-0.63562200	0.24467500	-1.41135700
	C	-2.66100600	0.08289200	-0.02946100
	C	-3.94171400	-0.45899200	-0.69415800
	C	-4.97960600	-1.03141400	0.03641900
	C	-6.13806600	-1.47309900	-0.60412700
	C	-6.27057100	-1.35174300	-1.98222600
	C	-5.23613800	-0.77813100	-2.72164700
	C	-4.08640300	-0.33470500	-2.08130400
	C	-2.62940200	1.62068600	-0.06352900
	C	-1.46280100	2.28633700	0.33011300
	C	-1.41374500	3.67261600	0.36429200
	C	-2.53454800	4.42531100	0.01414700
	C	-3.70214800	3.77361200	-0.36358900
	C	-3.74924000	2.37900200	-0.40143600
	C	-2.55670900	-0.40592100	1.43692800
	C	-2.96004300	0.44080400	2.47452500
	C	-2.96134600	0.00796800	3.79785800
	C	-2.55343200	-1.28324100	4.11654000
	C	-2.15239300	-2.13669700	3.09260000
	C	-2.16092400	-1.70607700	1.76899900
	H	-4.88979900	-1.13601300	1.10892100
	H	-6.93564500	-1.91517800	-0.01806800
	H	-7.16941100	-1.69838500	-2.47866000
	H	-5.32780700	-0.67264600	-3.79658300
	H	-3.29533300	0.12818600	-2.66136600
	H	-0.59147800	1.71954200	0.61737900
	H	-0.49545700	4.16167000	0.66656100
	H	-2.49621500	5.50840400	0.03989300
	H	-4.58440900	4.34436900	-0.63011300
	H	-4.66866000	1.88927600	-0.69081000
	H	-3.27685300	1.44921800	2.25045700
	H	-3.28117900	0.68774100	4.57926600

	H -2.54888100 -1.62032200 5.14660800<br>H -1.83402700 -3.14767900 3.32042400<br>H -1.87156500 -2.39102000 0.98867900<br>H -0.69565900 -3.98061900 -3.42473300<br>O 1.23571500 -1.92727400 -0.39143000<br>C 2.04265000 -1.09785300 -1.08534700<br>C 2.81566500 -0.09019900 -0.19322500<br>C 3.99115600 -0.91787200 0.38896400<br>C 4.38150400 -0.80469000 1.72360600<br>O 2.21773000 -1.21730800 -2.27022900<br>C 4.72948000 -1.75816000 -0.45384300<br>C 5.81705300 -2.47598900 0.03035100<br>C 6.19542400 -2.36183000 1.36646700<br>C 5.47601700 -1.52029400 2.20744100<br>C 1.98803100 0.54119300 0.95248300<br>C 2.10569200 1.90977000 1.22336000<br>C 1.46267900 2.48949700 2.31553500<br>C 0.67751900 1.71566800 3.16129000<br>C 0.55844700 0.35222700 2.91093700<br>C 1.21381600 -0.22885600 1.83088600<br>C 3.33655700 1.01781000 -1.12544500<br>C 2.42353300 1.69856600 -1.94032700<br>C 2.84062400 2.75807700 -2.73455000<br>C 4.17666500 3.16048500 -2.72775500<br>C 5.08646400 2.49246600 -1.91776800<br>C 4.66745800 1.42628300 -1.12010000<br>H 3.83653400 -0.15308300 2.39164200<br>H 4.46075600 -1.83530400 -1.49971300<br>H 6.37281200 -3.12140500 -0.64024300<br>H 7.04367400 -2.92034800 1.74495800<br>H 5.76263500 -1.41499600 3.24759300<br>H 2.70806700 2.53594200 0.58263700<br>H 1.57633500 3.55254900 2.49493800<br>H 0.15871900 2.16596200 3.99924100<br>H -0.05293100 -0.26769000 3.55312500<br>H 1.12140900 -1.29072300 1.67533800<br>H 1.38200700 1.40396300 -1.94084200<br>H 2.12012300 3.27294100 -3.35976700<br>H 4.50145600 3.98737000 -3.34901900<br>H 6.12709700 2.79588800 -1.90039700<br>H 5.38599000 0.92014200 -0.48985400<br>H 1.28491000 -3.42610000 -1.82663200

18 (optimized at the M06-2X/6-311G(d,p) level)

Conformer no	
1	O -1.30405700 -0.93445900 -1.20937400<br>C -2.44325200 -0.30756600 -1.52265700<br>C -0.49802800 -1.44801300 -2.28355500<br>H -1.15210300 -1.69563500 -3.12303300<br>C 0.43519900 -0.33980300 -2.74180700<br>H 1.05621100 -0.68131200 -3.57061100

	C	0.19717300	-2.68123800	-1.74168600	
	H	0.90384400	-3.07271200	-2.47499900	
	H	-0.54579900	-3.44517900	-1.50834400	
	O	-2.82686700	-0.15050300	-2.64896400	
	C	-3.19586900	0.07215800	-0.23285400	
	C	-3.77198400	-1.29356400	0.19565000	
	C	-2.94929300	-2.23712800	0.81863100	
	C	-3.42825500	-3.50267200	1.12801600	
	C	-4.73436200	-3.85645700	0.80323800	
	C	-5.54716000	-2.93864200	0.15158500	
	C	-5.06836000	-1.66888900	-0.15721800	
	C	-4.27928600	1.14026300	-0.44163200	
	C	-4.28404000	2.02874000	-1.51563200	
	C	-5.22666600	3.05175500	-1.58395000	
	C	-6.17407000	3.20909200	-0.58337300	
	C	-6.16888900	2.33636100	0.50065300	
	C	-5.22794400	1.31980700	0.57111000	
	C	-2.21097300	0.69276900	0.77154600	
	C	-2.30649800	0.47161200	2.14271500	
	C	-1.47563300	1.15428100	3.02907900	
	C	-0.54804700	2.07349100	2.55597700	
	C	-0.45292700	2.30894800	1.18659400	
	C	-1.27599800	1.62157200	0.30755300	
	H	-1.92004000	-1.98217200	1.03932300	
	H	-2.77408500	-4.21652800	1.61522000	
	H	-5.10941000	-4.84374500	1.04508600	
	H	-6.55902700	-3.20868000	-0.12639400	
	H	-5.70858400	-0.96860300	-0.67902700	
	H	-3.56662300	1.92398700	-2.31541300	
	H	-5.21442400	3.72640000	-2.43181000	
	H	-6.90682000	4.00496900	-0.64231500	
	H	-6.89523600	2.45115200	1.29655200	
	H	-5.22666900	0.65415300	1.42750600	
	H	-3.03276600	-0.23513200	2.52780900	
	H	-1.56228700	0.96677300	4.09314800	
	H	0.10145000	2.60321800	3.24282300	
	H	0.27512400	3.01408900	0.80313100	
	H	-1.19104800	1.81712900	-0.75749400	
	H	0.73859000	-2.44113100	-0.82494300	
	O	1.26265500	0.09936100	-1.65818700	
	C	2.49475600	-0.42334400	-1.56979900	
	C	3.15886500	-0.06052400	-0.22759000	
	C	2.89706900	1.38725800	0.20724000	
	C	2.97702200	1.73927600	1.55510300	
	O	2.96609400	-1.16261600	-2.38788000	
	C	2.75716000	2.40476800	-0.73705900	
	C	2.68923400	3.73716600	-0.34613700	
	C	2.76643100	4.07680400	1.00006700	
	C	2.91123500	3.07047500	1.94888600	
	C	4.68747500	-0.18270500	-0.31023000	
	C	5.37668500	0.15061200	-1.47587800	
	C	6.76628600	0.15383100	-1.50188900	

	C	7.49164900	-0.16800600	-0.36144400
	C	6.81414700	-0.48639100	0.80955200
	C	5.42497300	-0.48953000	0.83435500
	C	2.51256400	-1.12267900	0.68620400
	C	3.03477700	-2.41862000	0.71165100
	C	2.39710700	-3.42473200	1.42738700
	C	1.22482800	-3.15199700	2.12535200
	C	0.68687200	-1.87100800	2.08517400
	C	1.31425900	-0.86623400	1.35431200
	H	3.09771900	0.96582400	2.30518000
	H	2.70987800	2.16219500	-1.79238500
	H	2.58073100	4.51073300	-1.09724500
	H	2.71797700	5.11493800	1.30632000
	H	2.98089600	3.32064600	3.00122800
	H	4.83194000	0.39999200	-2.37680000
	H	7.28195800	0.40780100	-2.42032100
	H	8.57470400	-0.16772800	-0.38387600
	H	7.36616200	-0.73229000	1.70895800
	H	4.90963600	-0.73828600	1.75501400
	H	3.93686500	-2.64273300	0.15452900
	H	2.81673100	-4.42358500	1.43565800
	H	0.73287900	-3.93438700	2.69149800
	H	-0.22774900	-1.63943700	2.61940600
	H	0.85454800	0.11246700	1.29692600
	H	-0.14717900	0.53075600	-3.04448400
5	O	-1.57855600	-1.45185300	-1.27859000
	C	-1.81734400	-0.14077700	-1.14312900
	C	-0.60970100	-1.89444600	-2.24632800
	H	-0.28732100	-1.03987400	-2.84191400
	C	0.56032600	-2.44945900	-1.46245000
	H	0.19832700	-3.15542600	-0.71323500
	C	-1.27325700	-2.95484400	-3.10434100
	H	-0.59036500	-3.29204400	-3.88591000
	H	-2.16888700	-2.54626900	-3.57338200
	O	-1.26115100	0.70164300	-1.78898400
	C	-2.90842500	0.10728200	-0.06453700
	C	-2.96584900	1.61223800	0.24111700
	C	-2.93443900	2.09406500	1.54756000
	C	-2.99844900	3.46229000	1.80751400
	C	-3.09123500	4.37179400	0.76533100
	C	-3.14322700	3.90129500	-0.54499700
	C	-3.08778300	2.53946100	-0.80259000
	C	-2.51992200	-0.67090800	1.20299200
	C	-1.18374300	-0.70403700	1.61179800
	C	-0.83038700	-1.28555600	2.82261500
	C	-1.80424500	-1.84749800	3.64305700
	C	-3.13341200	-1.81925900	3.24211800
	C	-3.48950600	-1.23247800	2.03036400
	C	-4.25722500	-0.40600600	-0.63108400
	C	-4.40686000	-1.75862100	-0.96676300
	C	-5.61472300	-2.24800600	-1.44310700
	C	-6.71118400	-1.40288400	-1.58486100

	C	-6.58424500	-0.06907400	-1.22973500
	C	-5.37059300	0.42455900	-0.75418600
	H	-2.84304300	1.40812700	2.37965900
	H	-2.96916600	3.81024400	2.83373600
	H	-3.13111700	5.43573600	0.96640700
	H	-3.22637600	4.59822200	-1.37052600
	H	-3.13330900	2.19212500	-1.82589600
	H	-0.40811700	-0.25986900	0.99491100
	H	0.20961400	-1.28147000	3.13005400
	H	-1.52818100	-2.29972300	4.58825300
	H	-3.90180900	-2.24899900	3.87388200
	H	-4.53235900	-1.20383000	1.73714500
	H	-3.57494800	-2.43598300	-0.83310300
	H	-5.70103500	-3.29845900	-1.69543400
	H	-7.65457100	-1.78547400	-1.95605900
	H	-7.43137400	0.60129500	-1.31397200
	H	-5.30895200	1.46553000	-0.46838500
	H	-1.56387700	-3.81449800	-2.49531800
	O	1.21314100	-1.41281500	-0.72795500
	C	2.29947700	-0.85993300	-1.30096100
	C	3.08868900	-0.04209000	-0.26395900
	C	3.81528900	-1.17492300	0.49578300
	C	5.12550200	-1.52612700	0.17004700
	O	2.63344200	-1.09442500	-2.42771700
	C	3.12718500	-1.95981700	1.42699800
	C	3.75192100	-3.02927200	2.05488000
	C	5.07048400	-3.34948400	1.74793100
	C	5.74956300	-2.60091500	0.79621400
	C	2.13005500	0.79839200	0.59392900
	C	1.16616500	1.56666300	-0.06426900
	C	0.33485300	2.42510200	0.64069800
	C	0.46457900	2.53704300	2.02287900
	C	1.43010400	1.79088800	2.68362000
	C	2.26339800	0.92824000	1.97377800
	C	4.06873800	0.96668000	-0.88050200
	C	5.00724200	1.55062900	-0.02248500
	C	5.87447200	2.53447200	-0.47044100
	C	5.81251900	2.96886800	-1.79183700
	C	4.87311700	2.41075300	-2.64524200
	C	4.00398100	1.41712000	-2.19697800
	H	5.66423200	-0.95835900	-0.57795400
	H	2.09023200	-1.73760200	1.64823400
	H	3.20313800	-3.61715200	2.78118500
	H	5.55883400	-4.18293500	2.23876900
	H	6.77008000	-2.85193500	0.53223800
	H	1.05212500	1.48950100	-1.14123500
	H	-0.42134700	2.99663100	0.11358600
	H	-0.18844500	3.20221400	2.57469600
	H	1.54379400	1.87723300	3.75814200
	H	3.02240400	0.36573700	2.50415600
	H	5.05403600	1.22785000	1.01220500
	H	6.59451300	2.96719400	0.21407700

	H	6.48692000	3.73869400	-2.14731800
	H	4.80799800	2.74487200	-3.67403100
	H	3.29013500	0.99256800	-2.88709600
	H	1.27178200	-2.93279500	-2.13321700
13	O	1.29128400	0.88407700	-1.34892000
	C	2.38428200	0.13959700	-1.54883400
	C	0.47248400	1.19306400	-2.49243500
	H	1.12505200	1.31911600	-3.36034000
	C	-0.43778100	0.01056100	-2.78336300
	H	-1.05888400	0.22492200	-3.65402400
	C	-0.24688700	2.48353700	-2.15199400
	H	-0.95482700	2.74419200	-2.93958900
	H	0.48111200	3.28801500	-2.03717300
	O	2.68215900	-0.30512600	-2.62230700
	C	3.15305800	-0.08992300	-0.22164500
	C	2.15164200	-0.71110100	0.76584100
	C	2.16797700	-0.39751100	2.12208400
	C	1.32425400	-1.06119800	3.01132400
	C	0.45638500	-2.04366800	2.55413400
	C	0.42873500	-2.36004400	1.19764400
	C	1.27170100	-1.69980000	0.31528400
	C	3.68328000	1.26185700	0.31900900
	C	5.01860300	1.41908400	0.69207000
	C	5.47196400	2.61342400	1.24893200
	C	4.60028000	3.67297100	1.44935200
	C	3.26253900	3.52264000	1.09775100
	C	2.80790800	2.33295600	0.54565600
	C	4.30928600	-1.06520400	-0.48750100
	C	5.21619300	-0.79952700	-1.52038300
	C	6.30641800	-1.62670600	-1.74058800
	C	6.51866900	-2.73955700	-0.93045800
	C	5.63057100	-3.00906200	0.09917500
	C	4.53337200	-2.17765500	0.31961100
	H	2.84934700	0.35872000	2.49415300
	H	1.35230000	-0.80656400	4.06456100
	H	-0.20539900	-2.55673200	3.24134700
	H	-0.26077700	-3.11065300	0.82912300
	H	1.25582300	-1.96853700	-0.73721500
	H	5.72077600	0.60686400	0.56175100
	H	6.51496800	2.70500700	1.52803100
	H	4.95478500	4.60199600	1.87990200
	H	2.56071700	4.33359800	1.25684800
	H	1.75919600	2.23049600	0.30495400
	H	5.06768700	0.06609000	-2.15292600
	H	6.99294500	-1.40271800	-2.54844000
	H	7.36978700	-3.38731800	-1.10396100
	H	5.78356300	-3.86894600	0.74046900
	H	3.85571100	-2.40738800	1.13192800
	H	-0.79165700	2.38024600	-1.21160200
	O	-1.26454600	-0.28766800	-1.65402600
	C	-2.49446000	0.24810400	-1.62871800
	C	-3.15308400	0.04390700	-0.25160800

	C	-2.92012600	-1.36332100	0.31304500
	C	-2.95320300	-1.58475400	1.68908400
	O	-2.96138200	0.88883300	-2.52826300
	C	-2.85400300	-2.46829900	-0.53671900
	C	-2.81612300	-3.75999400	-0.02526300
	C	-2.84823500	-3.97020000	1.34950800
	C	-2.91867500	-2.87580800	2.20363700
	C	-4.67940500	0.19917900	-0.31748200
	C	-5.40707300	-0.18688900	-1.44255000
	C	-6.79721500	-0.15447100	-1.43240400
	C	-7.48379900	0.25546000	-0.29688200
	C	-6.76751900	0.62751400	0.83513400
	C	-5.37922800	0.59576100	0.82398100
	C	-2.46281300	1.17218400	0.54331800
	C	-2.96780900	2.47339400	0.47571900
	C	-2.28803600	3.52873700	1.07272700
	C	-1.08859500	3.30018700	1.73919500
	C	-0.56702100	2.01232400	1.78917300
	C	-1.24044400	0.95621500	1.18270300
	H	-3.01226200	-0.74113900	2.36738000
	H	-2.84351800	-2.32528000	-1.61150400
	H	-2.76764800	-4.60356200	-0.70373100
	H	-2.82451200	-4.97689600	1.74915300
	H	-2.95364800	-3.02478000	3.27674500
	H	-4.89343100	-0.50318300	-2.34011200
	H	-7.34336100	-0.45028100	-2.32033400
	H	-8.56678100	0.28152300	-0.29196800
	H	-7.28926600	0.94120200	1.73152800
	H	-4.83279900	0.88551200	1.71442300
	H	-3.89068100	2.66271400	-0.05960700
	H	-2.69641600	4.53046500	1.01104700
	H	-0.56206400	4.12083800	2.21284700
	H	0.37402900	1.81898100	2.29113300
	H	-0.79905200	-0.03303500	1.19626600
	H	0.16064500	-0.88220100	-2.96053400
15	O	-1.54637100	-1.59234100	-1.28986800
	C	-1.45279000	-0.30708600	-0.92401400
	C	-0.57346700	-2.11372500	-2.21503400
	H	-0.17377300	-1.29293500	-2.80943700
	C	0.54362000	-2.76740900	-1.41902800
	H	0.12530100	-3.50660800	-0.73516200
	C	-1.29660000	-3.12578000	-3.08206800
	H	-2.12438100	-2.64259800	-3.60225100
	H	-0.61472900	-3.54252300	-3.82517900
	O	-0.57379800	0.41605300	-1.30256300
	C	-2.64083800	0.08865600	-0.00938700
	C	-3.86788700	0.22065800	-0.94615100
	C	-4.54396300	1.42821600	-1.11355600
	C	-5.64438000	1.51818300	-1.96407400
	C	-6.09144900	0.40418600	-2.65744300
	C	-5.43428000	-0.81091800	-2.48599400
	C	-4.33897000	-0.90274200	-1.63954200

	C	-2.37210900	1.41313100	0.73006900
	C	-1.77224800	2.50980700	0.09934500
	C	-1.61968400	3.72046800	0.76537400
	C	-2.08693500	3.87695100	2.06615800
	C	-2.70098400	2.80260500	2.69584400
	C	-2.83403800	1.58367400	2.03743900
	C	-2.81803000	-1.03812100	1.01768800
	C	-1.67598600	-1.53711500	1.64739100
	C	-1.78129800	-2.49520800	2.64490400
	C	-3.03351100	-2.96505000	3.03401500
	C	-4.17321800	-2.46389600	2.41996900
	C	-4.06713300	-1.50415300	1.41488600
	H	-4.22479000	2.31113300	-0.57639900
	H	-6.15033000	2.46976600	-2.07651000
	H	-6.94635900	0.47543300	-3.31917900
	H	-5.77971100	-1.69522600	-3.00882000
	H	-3.85177400	-1.85865800	-1.49812000
	H	-1.42060800	2.42399300	-0.91757900
	H	-1.13881100	4.54750100	0.25570100
	H	-1.97414300	4.82486500	2.57910600
	H	-3.07636800	2.90411800	3.70742000
	H	-3.30594200	0.76071400	2.55824000
	H	-0.69328800	-1.16700600	1.36256300
	H	-0.88172800	-2.87011400	3.11886300
	H	-3.11895400	-3.71404600	3.81244700
	H	-5.15283600	-2.81583300	2.72121900
	H	-4.96444200	-1.11277200	0.95056000
	H	-1.69552500	-3.93839900	-2.47051200
	O	1.22675400	-1.83558500	-0.57890100
	C	2.07048900	-0.99560800	-1.20007200
	C	2.82783700	-0.06797600	-0.22549600
	C	3.04639600	1.31252200	-0.87857600
	C	2.16985800	1.82611200	-1.83576900
	O	2.24746800	-1.02648000	-2.38740600
	C	4.09091600	2.12494300	-0.43371300
	C	4.25482900	3.41426300	-0.92594400
	C	3.37709800	3.91710500	-1.87864100
	C	2.33583100	3.11611500	-2.32939700
	C	4.21924500	-0.71913100	0.00187900
	C	4.90842600	-1.31085900	-1.05955100
	C	6.18957000	-1.82128100	-0.88133600
	C	6.81826200	-1.73508300	0.35407700
	C	6.15446000	-1.12215300	1.40922800
	C	4.86941500	-0.62027200	1.23514900
	C	2.03305000	0.06924100	1.08744200
	C	2.00865300	-0.98431500	2.01184000
	C	1.31764300	-0.86989400	3.20814300
	C	0.60182100	0.29130800	3.49601700
	C	0.59094500	1.32656100	2.57487500
	C	1.30721300	1.21949100	1.38238900
	H	1.34398200	1.22943300	-2.19267000
	H	4.79066600	1.75246200	0.30397400

	H	5.07542700	4.02315400	-0.56528100
	H	3.50583700	4.92020300	-2.26782000
	H	1.64321100	3.49155600	-3.07384900
	H	4.45162000	-1.35792800	-2.03937100
	H	6.69861400	-2.28154200	-1.72001200
	H	7.81692400	-2.13251900	0.49057900
	H	6.63457900	-1.02898800	2.37638800
	H	4.37703600	-0.13729900	2.06963100
	H	2.54689300	-1.89823500	1.79104100
	H	1.33335100	-1.69094800	3.91601000
	H	0.05023700	0.37817300	4.42488000
	H	0.02747200	2.23048000	2.77220300
	H	1.28717200	2.04705800	0.68459100
	H	1.25308300	-3.23756100	-2.10337800
17	O	-1.29613100	0.93948300	1.34732900
	C	-2.39694800	0.20086200	1.54056500
	C	-0.44948800	1.16962900	2.49415000
	H	-1.08854500	1.27247500	3.37472300
	C	0.42799700	-0.05256000	2.72497800
	H	1.03638700	0.09106500	3.61883700
	C	0.30889400	2.44963000	2.20782700
	H	1.03178800	2.64628600	3.00081900
	H	-0.38953100	3.28456900	2.13792800
	O	-2.70781100	-0.23576600	2.61357300
	C	-3.15715600	-0.08241100	0.21976000
	C	-2.94667000	1.03514300	-0.81239300
	C	-2.87759700	0.74607900	-2.17446400
	C	-2.83339800	1.76794800	-3.12073200
	C	-2.84232700	3.09692600	-2.71923000
	C	-2.90118400	3.39711200	-1.36045800
	C	-2.96059400	2.37708100	-0.42080200
	C	-4.65493200	-0.23764400	0.54555000
	C	-5.06719400	-1.24028400	1.43279800
	C	-6.41000400	-1.42188000	1.72566500
	C	-7.37587000	-0.61183700	1.13360900
	C	-6.98070500	0.37474300	0.24442100
	C	-5.63040500	0.55989500	-0.04961100
	C	-2.57016200	-1.40752900	-0.31895400
	C	-3.38020000	-2.34852400	-0.95587300
	C	-2.82403600	-3.48753700	-1.53098100
	C	-1.45302600	-3.70742700	-1.47447900
	C	-0.63910900	-2.77809000	-0.83707500
	C	-1.19364200	-1.63825900	-0.26725100
	H	-2.86562400	-0.28393700	-2.50852600
	H	-2.78644900	1.51755900	-4.17405300
	H	-2.80274800	3.89240100	-3.45391900
	H	-2.90878300	4.42938900	-1.03051100
	H	-3.02895400	2.62913800	0.63114900
	H	-4.32768300	-1.88329400	1.89028300
	H	-6.70440000	-2.20319600	2.41630500
	H	-8.42530100	-0.75651300	1.36133600
	H	-7.71968200	1.00847000	-0.23136900

	H	-5.35247400	1.33213600	-0.75475000
	H	-4.45012800	-2.18957900	-1.01344000
	H	-3.47094800	-4.20314400	-2.02487500
	H	-1.02074600	-4.59488800	-1.92165500
	H	0.43256600	-2.92872300	-0.78202300
	H	-0.53117500	-0.92347200	0.20440400
	H	0.84016400	2.37354500	1.25836800
	O	1.27370200	-0.30453500	1.59637000
	C	2.52068400	0.19637600	1.61781400
	C	3.18277300	0.05280200	0.23281500
	C	4.70859400	0.20539000	0.29542800
	C	5.39530300	0.63535700	-0.84207100
	O	2.99257300	0.76318100	2.56218200
	C	5.44813900	-0.20748300	1.40284200
	C	6.83828300	-0.16785900	1.37949300
	C	7.51184800	0.27563700	0.24899600
	C	6.78297200	0.67428700	-0.86598000
	C	2.49023200	1.21572100	-0.50697200
	C	2.99099800	2.51340100	-0.37391700
	C	2.28521900	3.59944600	-0.87994100
	C	1.05861700	3.40672400	-1.50643500
	C	0.54386900	2.12092800	-1.62679800
	C	1.25549300	1.03425000	-1.13261100
	C	2.94281400	-1.33434800	-0.37720300
	C	2.94176100	-2.46628200	0.43999900
	C	2.90040500	-3.74219500	-0.10876700
	C	2.85521800	-3.90876700	-1.48908600
	C	2.85199400	-2.78801100	-2.31156400
	C	2.90137700	-1.51222200	-1.75966700
	H	4.83788700	0.94592200	-1.71868600
	H	4.94476500	-0.54874100	2.29677100
	H	7.39449000	-0.48417400	2.25403900
	H	8.59461200	0.30743900	0.23458600
	H	7.29425100	1.01483800	-1.75857200
	H	3.92713300	2.67586300	0.14691900
	H	2.69082300	4.59805900	-0.76762700
	H	0.49810600	4.25200700	-1.88892200
	H	-0.42088700	1.96001800	-2.09155600
	H	0.82943900	0.04199100	-1.21689900
	H	2.98664900	-2.35472200	1.51840100
	H	2.90248600	-4.60691200	0.54410900
	H	2.82312700	-4.90295300	-1.91836200
	H	2.81739300	-2.90359700	-3.38829300
	H	2.91343500	-0.64787200	-2.41357300
	H	-0.19312600	-0.94167700	2.83263300
18	O	-1.19201300	-1.24693600	-0.94878400
	C	-2.27846500	-0.61824900	-1.43124800
	C	-0.57203100	-2.22576800	-1.80555800
	H	-1.30716700	-2.54681700	-2.54668100
	C	0.57751200	-1.56697000	-2.54400600
	H	0.99985200	-2.26209000	-3.27226100
	C	-0.14280500	-3.37873600	-0.91806900

	H	-1.01531600	-3.80512000	-0.42084800	
	H	0.56849900	-3.03705700	-0.16494500	
	O	-2.63111100	-0.70310300	-2.57407800	
	C	-3.05548300	0.06679400	-0.29132700	
	C	-4.02762200	1.15599100	-0.76791300	
	C	-4.94002900	1.65337500	0.17033400	
	C	-5.79705700	2.69576500	-0.14513000	
	C	-5.75151900	3.27800200	-1.40900000	
	C	-4.83849200	2.80606500	-2.33939700	
	C	-3.98010100	1.75365600	-2.02519700	
	C	-2.08929800	0.77980100	0.66681100	
	C	-1.12793700	1.63250400	0.11620800	
	C	-0.29593300	2.38809100	0.92983000	
	C	-0.42116300	2.30984800	2.31511000	
	C	-1.38041200	1.47546300	2.87094000	
	C	-2.21460300	0.71598900	2.05186900	
	C	-3.78746100	-1.14934500	0.31959100	
	C	-3.08511500	-2.06954900	1.10577400	
	C	-3.70550600	-3.21457600	1.58718200	
	C	-5.03808900	-3.47115300	1.27927400	
	C	-5.73461500	-2.58019200	0.47449900	
	C	-5.11311400	-1.43210800	-0.00875300	
	H	-4.97322000	1.21716900	1.16322800	
	H	-6.49570000	3.06044500	0.59867100	
	H	-6.41685200	4.09555100	-1.65939100	
	H	-4.78478300	3.25432400	-3.32452200	
	H	-3.28813500	1.39928500	-2.77402500	
	H	-1.01823300	1.70147500	-0.96177300	
	H	0.45895400	3.02757600	0.48522300	
	H	0.23001300	2.89820500	2.95005100	
	H	-1.49003800	1.41301200	3.94754000	
	H	-2.96717100	0.08017100	2.50269200	
	H	-2.03981700	-1.88939100	1.32629000	
	H	-3.14298900	-3.90955400	2.19990700	
	H	-5.52366400	-4.36337600	1.65609000	
	H	-6.76734500	-2.77712200	0.21220300	
	H	-5.66652700	-0.75615100	-0.64804000	
	H	0.32964800	-4.15600100	-1.52297200	
	O	1.61957400	-1.27039100	-1.61065200	
	C	1.85532300	0.01062100	-1.28234900	
	C	2.94404200	0.09346900	-0.17777500	
	C	4.32909400	-0.25842500	-0.77568300	
	C	5.46287500	0.45842400	-0.38274500	
	O	1.28305500	0.93503100	-1.78364400	
	C	4.50971900	-1.34590400	-1.63717500	
	C	5.77310600	-1.68222000	-2.10906100	
	C	6.89019800	-0.95270600	-1.72068600	
	C	6.72782000	0.11466000	-0.84865400	
	C	2.54048600	-0.90915500	0.91734700	
	C	1.23229600	-0.86408400	1.41334100	
	C	0.83748700	-1.70050600	2.44732200	
	C	1.73998500	-2.60977700	2.99579600	

	C	3.03575800	-2.66518200	2.50300600
	C	3.43747300	-1.81615500	1.47190500
	C	2.97236600	1.52574800	0.37592100
	C	3.12709900	2.60158400	-0.50620800
	C	3.20124200	3.90353500	-0.03494400
	C	3.14419200	4.15938900	1.33396000
	C	3.02525800	3.09734700	2.21775000
	C	2.93749400	1.78941100	1.74241200
	H	5.36767700	1.29311100	0.29890200
	H	3.66272000	-1.94989800	-1.92784600
	H	5.88083200	-2.52515800	-2.78154200
	H	7.87415700	-1.21701100	-2.08892700
	H	7.58703000	0.69078900	-0.52584000
	H	0.51469000	-0.16379700	0.99579400
	H	-0.17514000	-1.63030500	2.82916700
	H	1.43387100	-3.26466900	3.80291600
	H	3.74729100	-3.36742700	2.92157000
	H	4.45579200	-1.86757200	1.10676800
	H	3.18849200	2.41628200	-1.57020500
	H	3.30869400	4.72120600	-0.73772000
	H	3.20379800	5.17626100	1.70336900
	H	2.99712100	3.27790500	3.28621300
	H	2.83539700	0.97793300	2.45162800
	H	0.24767300	-0.65763100	-3.04523700
28	O	-1.26821100	-1.01212600	-1.38260700
	C	-1.93147800	0.14158800	-1.23413700
	C	-0.42701500	-1.14522700	-2.54416300
	H	0.01350000	-0.17253300	-2.76800600
	C	0.65419100	-2.12556600	-2.14172600
	H	0.20817300	-3.02189800	-1.71071800
	C	-1.25187800	-1.63759300	-3.71894400
	H	-0.63351100	-1.69074200	-4.61671500
	H	-2.07476100	-0.94658200	-3.90618500
	O	-1.82138600	1.05545300	-2.00449700
	C	-2.91160200	0.08626900	-0.03113700
	C	-3.06214800	1.47644800	0.62075000
	C	-3.09958600	1.61027200	2.01051700
	C	-3.29524400	2.85275900	2.60853400
	C	-3.44894600	3.99085700	1.82994900
	C	-3.43039300	3.86868500	0.44390700
	C	-3.25575200	2.62667600	-0.15440700
	C	-2.41864100	-0.91729800	1.01932900
	C	-1.09586700	-0.83307100	1.46373700
	C	-0.65242700	-1.61347200	2.52122400
	C	-1.52166600	-2.50752300	3.14425800
	C	-2.83208100	-2.60676200	2.69999400
	C	-3.28069800	-1.81091500	1.64617100
	C	-4.24749400	-0.37930000	-0.65758000
	C	-4.27393100	-1.56598800	-1.39979100
	C	-5.45704100	-2.03386800	-1.95348200
	C	-6.64315300	-1.32969800	-1.76872200
	C	-6.62972400	-0.16086500	-1.02158700

	C	-5.44126600	0.31301700	-0.46933900
	H	-2.97357800	0.74440100	2.64675600
	H	-3.32027000	2.92297500	3.68973400
	H	-3.59252500	4.95987900	2.29360200
	H	-3.56580600	4.74388900	-0.18084200
	H	-3.26914400	2.54991700	-1.23165500
	H	-0.41244400	-0.13689100	0.98868300
	H	0.37146000	-1.51315400	2.86508000
	H	-1.17786400	-3.11757600	3.97130700
	H	-3.51832900	-3.29714500	3.17625000
	H	-4.31352700	-1.88322900	1.32758000
	H	-3.36110500	-2.13660400	-1.52247900
	H	-5.45366900	-2.95403700	-2.52589800
	H	-7.56847800	-1.69418700	-2.19861300
	H	-7.54770700	0.39179100	-0.86040800
	H	-5.45584100	1.22345400	0.11537500
	H	-1.66275500	-2.62937700	-3.51359300
	O	1.46542300	-1.56823100	-1.10618100
	C	2.45863800	-0.75357100	-1.49510400
	C	3.14040200	-0.06094300	-0.28712100
	C	2.04954900	0.83294300	0.33461800
	C	1.94012300	1.02426900	1.71054700
	O	2.71055400	-0.54969200	-2.64926900
	C	1.19799700	1.54885200	-0.51165100
	C	0.24686600	2.42020800	0.00266900
	C	0.13884500	2.60226700	1.37843800
	C	0.99074000	1.90611300	2.22670600
	C	4.29287800	0.80679900	-0.81075400
	C	5.23908800	0.25754800	-1.68314000
	C	6.33218600	0.99878700	-2.10499100
	C	6.50658100	2.30717400	-1.66064300
	C	5.57681000	2.86024200	-0.79352800
	C	4.47705000	2.11521300	-0.37181600
	C	3.65504800	-1.09877300	0.73769600
	C	4.97990100	-1.09205300	1.17521900
	C	5.41888100	-1.98974400	2.14695700
	C	4.54220100	-2.90623800	2.70672100
	C	3.21354200	-2.91124500	2.29205900
	C	2.77473700	-2.01742700	1.32567500
	H	2.59852200	0.48967900	2.38507800
	H	1.29363900	1.44567600	-1.58863100
	H	-0.41759300	2.94627100	-0.67163900
	H	-0.61279200	3.27390600	1.77690500
	H	0.91598000	2.03884800	3.29980800
	H	5.11983300	-0.76034400	-2.03252600
	H	7.05019800	0.55517000	-2.78464500
	H	7.36042700	2.88710500	-1.99049100
	H	5.69860800	3.87663500	-0.43856200
	H	3.76351200	2.56659800	0.30608200
	H	5.68470900	-0.37872700	0.76964800
	H	6.45438200	-1.96094700	2.46515200
	H	4.88547100	-3.60395900	3.46129800

	H	2.50797100	-3.61211000	2.72437700
	H	1.73472200	-2.02655600	1.03196100
	H	1.27022800	-2.38189400	-3.00409300
32	O	-1.26228200	-1.61539400	-1.01283900
	C	-2.05790500	-0.61218000	-1.41211500
	C	-0.61413100	-2.37654200	-2.04776100
	H	-1.34242400	-2.56614500	-2.84146200
	C	0.52558500	-1.57461700	-2.65971100
	H	1.01948900	-2.17767100	-3.42285500
	C	-0.15216600	-3.66536000	-1.39830700
	H	0.28895300	-4.32378700	-2.14935300
	H	-1.00019300	-4.17592300	-0.94094300
	O	-2.21761800	-0.33366200	-2.56902500
	C	-2.80203800	0.06367500	-0.23922300
	C	-4.17965200	-0.64427500	-0.14515700
	C	-4.81997800	-0.82766400	1.08341500
	C	-6.09716800	-1.37376900	1.14931100
	C	-6.76298100	-1.74738600	-0.01126800
	C	-6.14413300	-1.55115300	-1.23926600
	C	-4.87067100	-0.99748500	-1.30688700
	C	-3.04321300	1.55090000	-0.56585300
	C	-4.14019600	2.21595900	-0.01754900
	C	-4.32346700	3.57991700	-0.21751500
	C	-3.41301600	4.30531800	-0.97436200
	C	-2.31727900	3.65206300	-1.52612900
	C	-2.13196300	2.28896300	-1.32494200
	C	-1.98098000	-0.08806700	1.05777700
	C	-1.24103100	0.97073100	1.57646400
	C	-0.50205600	0.82190100	2.74995400
	C	-0.50773200	-0.38270800	3.43524400
	C	-1.23874100	-1.45332800	2.92316400
	C	-1.94719000	-1.31299200	1.73942200
	H	-4.32641700	-0.52975300	1.99975100
	H	-6.57088600	-1.50254400	2.11558800
	H	-7.75642800	-2.17647800	0.04131100
	H	-6.65592900	-1.82003700	-2.15572400
	H	-4.42230500	-0.82018200	-2.27603700
	H	-4.86780100	1.67042200	0.56955200
	H	-5.18534900	4.07115200	0.21840200
	H	-3.55766200	5.36699400	-1.13640200
	H	-1.59864700	4.20281800	-2.12193300
	H	-1.26611100	1.80535200	-1.75343500
	H	-1.22717700	1.92729400	1.07007300
	H	0.07710000	1.66055800	3.11719700
	H	0.05913000	-0.49468200	4.35217400
	H	-1.25175400	-2.40358100	3.44513500
	H	-2.49451300	-2.15887700	1.34140300
	H	0.59515500	-3.45800300	-0.63103300
	O	1.53113800	-1.29476300	-1.67735200
	C	1.48307000	-0.10073800	-1.06636300
	C	2.68985600	0.08411500	-0.11249100
	C	2.45066500	1.23511000	0.88447300

	C	2.92761700	1.12836900	2.19320300
	O	0.62267500	0.70529800	-1.28501100
	C	1.86542700	2.44666000	0.49796200
	C	1.74114800	3.49836700	1.39867200
	C	2.22301700	3.37913900	2.69804200
	C	2.82341000	2.18949300	3.08762200
	C	3.90973900	0.38677100	-1.01752200
	C	4.34350600	-0.56770900	-1.94735000
	C	5.43941000	-0.32228800	-2.76200100
	C	6.13177800	0.88130800	-2.66656900
	C	5.71967800	1.82719100	-1.74060200
	C	4.62010100	1.58239600	-0.92065500
	C	2.86166800	-1.22728300	0.66560300
	C	4.10380200	-1.79907900	0.92117300
	C	4.20326700	-2.93759500	1.71898800
	C	3.06448000	-3.51182500	2.26748600
	C	1.81981100	-2.93742000	2.02078400
	C	1.72116200	-1.80338400	1.22812800
	H	3.38935000	0.20918000	2.52966900
	H	1.50636900	2.57766400	-0.51142000
	H	1.27215700	4.41988200	1.07361800
	H	2.13295100	4.20389800	3.39511100
	H	3.21007400	2.07625700	4.09370200
	H	3.82646500	-1.51577100	-2.01730400
	H	5.75713800	-1.07776900	-3.47104300
	H	6.98659700	1.07380100	-3.30385300
	H	6.25302500	2.76553600	-1.64519600
	H	4.32806200	2.33384500	-0.19975200
	H	5.00176500	-1.35426300	0.50938400
	H	5.17759300	-3.37206600	1.90974600
	H	3.14275200	-4.39847200	2.88541200
	H	0.92066400	-3.36883300	2.44517300
	H	0.74521400	-1.35608100	1.04960100
	H	0.15921700	-0.64656600	-3.09416500
36	O	1.15955900	1.36661200	-0.36091800
	C	1.93324900	0.48553000	-1.00797400
	C	0.43048800	2.31597900	-1.15770900
	H	-0.04177400	1.79224800	-1.99097900
	C	-0.62420400	2.88112700	-0.23076400
	H	-0.15880100	3.27123800	0.67416600
	C	1.38811400	3.38466400	-1.65714100
	H	1.91185100	3.84388500	-0.81417800
	H	0.84663000	4.15415100	-2.21049700
	O	1.86059000	0.27213300	-2.18594700
	C	3.01237100	-0.06579200	-0.05288700
	C	2.41556600	-0.60062100	1.25389100
	C	3.26515000	-0.83219600	2.33862200
	C	2.78834400	-1.40864800	3.50706100
	C	1.45104900	-1.78156900	3.60582200
	C	0.60420500	-1.57680900	2.52494200
	C	1.08099800	-0.98907700	1.35486100
	C	3.87950500	1.19908600	0.11634700

	C	4.67391900	1.60918900	-0.95816800
	C	5.37157300	2.80915800	-0.90949500
	C	5.27860200	3.62534700	0.21311500
	C	4.47459500	3.23498800	1.27751000
	C	3.77181600	2.03483700	1.22676900
	C	3.77916800	-1.23501500	-0.68026100
	C	5.14836500	-1.39841600	-0.47326500
	C	5.80986200	-2.52451300	-0.95357400
	C	5.11120100	-3.50483400	-1.64560800
	C	3.74232100	-3.35642100	-1.84291300
	C	3.08255300	-2.23546000	-1.35859000
	H	4.31268700	-0.56051000	2.26210600
	H	3.46406900	-1.57466600	4.33768600
	H	1.07607100	-2.23588200	4.51525700
	H	-0.43763000	-1.87236000	2.58159500
	H	0.39422700	-0.83846000	0.52737700
	H	4.73320000	0.98377100	-1.84260400
	H	5.98410500	3.10754900	-1.75207200
	H	5.82405700	4.56057100	0.25479600
	H	4.38688600	3.86878300	2.15222800
	H	3.12729900	1.75411500	2.05027500
	H	5.70875900	-0.64140700	0.06303700
	H	6.87476200	-2.63168200	-0.78404000
	H	5.62662900	-4.37919000	-2.02455200
	H	3.18519900	-4.11843600	-2.37584600
	H	2.01514600	-2.13503400	-1.50331800
	H	2.12915100	2.93881400	-2.32157400
	O	-1.50203400	1.84338300	0.21079900
	C	-2.48761000	1.49769600	-0.62610600
	C	-3.21069300	0.20623800	-0.17314000
	C	-3.51516900	0.22580200	1.34271500
	C	-4.81644400	0.12393700	1.83179100
	O	-2.71058300	2.08531000	-1.64781700
	C	-2.46369400	0.25699800	2.26944000
	C	-2.71076100	0.20932400	3.63295500
	C	-4.01670800	0.12437700	4.10966400
	C	-5.06430000	0.07972900	3.20367500
	C	-2.23404500	-0.94179700	-0.50672800
	C	-1.43253100	-0.87781200	-1.64873100
	C	-0.59130700	-1.93145700	-1.98875000
	C	-0.55123700	-3.07658600	-1.19980100
	C	-1.36171000	-3.15903900	-0.07405700
	C	-2.19464500	-2.09982600	0.27191500
	C	-4.49677500	0.06413500	-0.99662900
	C	-4.87442600	-1.14611600	-1.57036600
	C	-6.08944700	-1.26562900	-2.24348100
	C	-6.94064300	-0.17685100	-2.35281900
	C	-6.56997600	1.03980700	-1.78543100
	C	-5.36145600	1.15692900	-1.11650100
	H	-5.65348100	0.06508700	1.14929700
	H	-1.44126500	0.30708000	1.91997300
	H	-1.87475000	0.23163400	4.32291200

	H	-4.21020300	0.08575800	5.17504100
	H	-6.08626000	0.00120900	3.55515100
	H	-1.47885400	-0.01034000	-2.29810400
	H	0.03670200	-1.84429800	-2.86802200
	H	0.10836900	-3.89707900	-1.45752900
	H	-1.34254000	-4.04786400	0.54572900
	H	-2.82192000	-2.17741900	1.15222600
	H	-4.22270500	-2.00813600	-1.50129300
	H	-6.36266800	-2.21692400	-2.68437300
	H	-7.88381300	-0.26959500	-2.87794300
	H	-7.22330400	1.90039700	-1.86653600
	H	-5.08599500	2.10882600	-0.67826800
	H	-1.19149200	3.66230700	-0.73730400
42	O	-1.42196000	-1.44259300	-1.15381200
	C	-2.43380800	-0.64785900	-1.52915700
	C	-0.62824000	-2.04753300	-2.19111300
	H	-1.27208800	-2.24823100	-3.04959500
	C	0.43685300	-1.05323600	-2.61571100
	H	0.98093100	-1.42060800	-3.49103900
	C	-0.06342000	-3.32517800	-1.59943000
	H	-0.87571300	-4.00516100	-1.34097600
	H	0.51336500	-3.09778400	-0.70059000
	O	-2.70811600	-0.44595800	-2.67873100
	C	-3.11352100	0.02127300	-0.30561800
	C	-3.64213000	-1.03943100	0.68913900
	C	-4.95775900	-1.00042600	1.15313200
	C	-5.41262300	-1.91555300	2.10064200
	C	-4.56161100	-2.88480400	2.60920300
	C	-3.24249400	-2.92427900	2.16785200
	C	-2.78708600	-2.01213800	1.22591200
	C	-4.25824600	0.91126900	-0.80804200
	C	-5.21313000	0.38809500	-1.68694400
	C	-6.30056600	1.14803800	-2.08976400
	C	-6.46137500	2.44873700	-1.61843800
	C	-5.52393200	2.97535300	-0.74308300
	C	-4.42947300	2.21180700	-0.34081900
	C	-2.01675800	0.88783300	0.34175300
	C	-1.17806600	1.63792300	-0.48821400
	C	-0.21753000	2.48598600	0.04550800
	C	-0.08390900	2.60695500	1.42609500
	C	-0.92156900	1.87543600	2.25858500
	C	-1.88291500	1.01842200	1.72201000
	H	-5.64282200	-0.24844200	0.78544400
	H	-6.44015000	-1.85957800	2.44008900
	H	-4.91732300	-3.59663200	3.34464300
	H	-2.55629900	-3.66622500	2.56110300
	H	-1.75350600	-2.04984100	0.91280700
	H	-5.10525300	-0.62392300	-2.05604400
	H	-7.02513300	0.72463600	-2.77536300
	H	-7.31104000	3.04296100	-1.93335100
	H	-5.63583100	3.98499100	-0.36615600
	H	-3.71048100	2.64203900	0.34516100

	H	-1.29160700	1.57726200	-1.56700200
	H	0.43628300	3.03869500	-0.61779000
	H	0.67679400	3.25887300	1.83978400
	H	-0.82602900	1.96046100	3.33496000
	H	-2.53009500	0.45535500	2.38421400
	H	0.58980700	-3.81724600	-2.32340100
	O	1.35025700	-0.93471000	-1.52502400
	C	1.96562600	0.23738900	-1.32159900
	C	2.95976400	0.12825200	-0.13371300
	C	2.46299000	-0.93539100	0.85661200
	C	3.30232000	-1.90592600	1.39221500
	O	1.79853800	1.19237900	-2.02556800
	C	1.14631400	-0.85203200	1.32140900
	C	0.68761300	-1.70845300	2.31081300
	C	1.53358500	-2.68248000	2.84033600
	C	2.83642900	-2.78016800	2.37516700
	C	3.10491800	1.48092400	0.58898500
	C	3.28674100	2.67017100	-0.12722700
	C	3.46384300	3.87903100	0.53457600
	C	3.49679800	3.92705600	1.92514100
	C	3.35499900	2.74896300	2.64467400
	C	3.15682000	1.53967400	1.98322500
	C	4.30082700	-0.29652600	-0.77707500
	C	5.49508400	0.36602600	-0.50083000
	C	6.69470200	-0.07079100	-1.05873200
	C	6.71985300	-1.17273300	-1.90104400
	C	5.53395400	-1.84714200	-2.17531400
	C	4.33921100	-1.41691300	-1.61590900
	H	4.32946100	-1.98235200	1.05714600
	H	0.47874300	-0.10058500	0.91214200
	H	-0.32914100	-1.60713800	2.67463600
	H	1.17672700	-3.35401500	3.61236000
	H	3.50502200	-3.53070400	2.78019600
	H	3.29000400	2.65050100	-1.20722300
	H	3.59040200	4.78729800	-0.04301400
	H	3.64309400	4.87071300	2.43738600
	H	3.39211700	2.76219800	3.72763100
	H	3.04206900	0.63784900	2.56999800
	H	5.49987700	1.22584600	0.15616200
	H	7.61119600	0.45943300	-0.82851600
	H	7.65344600	-1.50869500	-2.33622000
	H	5.53927300	-2.71652000	-2.82223600
	H	3.42810600	-1.96990900	-1.81155500
	H	0.00154500	-0.08218000	-2.85194600
73	O	1.36545200	1.55337200	0.22839400
	C	2.12359400	0.95678400	-0.70188500
	C	0.71957700	2.78689100	-0.12408100
	H	0.53045100	2.78772600	-1.19875700
	C	-0.58988700	2.78220400	0.62756700
	H	-0.43243200	2.58131200	1.68890400
	C	1.63243800	3.93256300	0.27522100
	H	1.82190300	3.90960900	1.35056100

	H	1.18696800	4.89298400	0.00856800
	O	2.13360800	1.26685900	-1.85991300
	C	3.08264800	-0.04705600	-0.03167200
	C	2.36853100	-0.93875100	0.99061200
	C	3.10533500	-1.56499400	1.99720500
	C	2.50201400	-2.46496600	2.86597300
	C	1.15175900	-2.76758700	2.72957900
	C	0.41615400	-2.16856900	1.71437300
	C	1.01802500	-1.25901800	0.84973200
	C	4.09437100	0.94504400	0.58019300
	C	5.05558200	1.52844900	-0.24905700
	C	5.90137700	2.52042900	0.23254100
	C	5.79123400	2.95415600	1.54915500
	C	4.81983900	2.39826000	2.37320900
	C	3.97051200	1.40783400	1.89047500
	C	3.71915000	-1.00010300	-1.04869000
	C	5.00641100	-1.49866000	-0.84588100
	C	5.54287500	-2.45257600	-1.70365500
	C	4.79805000	-2.92616000	-2.77666600
	C	3.50947800	-2.44495400	-2.97668400
	C	2.97291800	-1.49449600	-2.11716600
	H	4.16245700	-1.34711000	2.10232900
	H	3.09023800	-2.93578500	3.64481600
	H	0.67958800	-3.47292300	3.40319600
	H	-0.63209400	-2.41021700	1.58452300
	H	0.42194200	-0.80274500	0.06393000
	H	5.13215500	1.20688500	-1.28156700
	H	6.64373300	2.95629800	-0.42546200
	H	6.45239700	3.72469800	1.92736000
	H	4.71490300	2.73996400	3.39623500
	H	3.19858200	1.00202900	2.53241200
	H	5.60023600	-1.13741700	-0.01397900
	H	6.54567600	-2.82483800	-1.53037100
	H	5.21644800	-3.66553900	-3.44912900
	H	2.91703300	-2.80824000	-3.80869400
	H	1.96681700	-1.13358600	-2.27969900
	H	2.58992100	3.83738000	-0.24139900
	O	-1.37112500	1.73616300	0.04841800
	C	-2.41175000	1.28647300	0.75065900
	C	-3.19427500	0.16737600	0.01607100
	C	-4.26958100	0.89994000	-0.82046300
	C	-4.59248300	0.50563600	-2.11834500
	O	-2.72790600	1.74873800	1.81151800
	C	-5.01479000	1.92335700	-0.22720000
	C	-6.03515100	2.55484000	-0.92587900
	C	-6.34147400	2.16572200	-2.22560500
	C	-5.62166200	1.13532400	-2.81449500
	C	-3.87079800	-0.71063500	1.07746100
	C	-3.17284000	-1.07178200	2.23320100
	C	-3.71834400	-1.96614800	3.14347800
	C	-4.97616500	-2.51697200	2.91535400
	C	-5.67922200	-2.16046200	1.77289200

	C -5.13078000 -1.26184500 0.86041600<br>C -2.26556700 -0.68746600 -0.87205100<br>C -2.12242800 -2.05984500 -0.67262800<br>C -1.26809900 -2.81855200 -1.47353600<br>C -0.55292400 -2.22168800 -2.49813900<br>C -0.70956700 -0.85599600 -2.73147600<br>C -1.55963700 -0.10380700 -1.93573900<br>H -4.04965900 -0.30246900 -2.59196900<br>H -4.80425500 2.21418900 0.79522500<br>H -6.59642900 3.34912700 -0.44807800<br>H -7.13854300 2.65777900 -2.77021600<br>H -5.85761800 0.81281100 -3.82174000<br>H -2.19226000 -0.65255600 2.42730100<br>H -3.16039700 -2.22911600 4.03459400<br>H -5.40399800 -3.21403700 3.62609900<br>H -6.66111300 -2.57903100 1.58594300<br>H -5.69217200 -0.99543100 -0.02718800<br>H -2.67344500 -2.55744800 0.11506600<br>H -1.16402000 -3.87976900 -1.28179500<br>H 0.11844300 -2.81083100 -3.11218600<br>H -0.16349100 -0.36920200 -3.53172800<br>H -1.68074600 0.95041100 -2.14335900<br>H -1.11415500 3.73485800 0.51666600

20 (optimized at the B3LYP/6-311G(d,p) level)

Conformer no	
1	O -1.38412400 0.01295600 -1.37159700<br>C -2.69773500 -0.25877500 -1.49227900<br>C -0.59530100 0.03647500 -2.59764200<br>H -1.21864800 0.48297300 -3.37643500<br>C 0.58845900 0.96954400 -2.36101900<br>H 1.15822000 1.02505000 -3.29249800<br>C -0.20219300 -1.37700000 -3.00597300<br>H -1.09376000 -1.96378400 -3.22751000<br>H 0.37143900 -1.86231500 -2.21611600<br>C 0.19037600 2.36998200 -1.91727000<br>H 1.07559900 2.99762800 -1.81734500<br>O -3.22697200 -0.51870000 -2.54341600<br>C -3.45405400 -0.07016000 -0.14561800<br>C -2.76045900 -0.74424900 1.06104100<br>C -3.21213400 -0.44857300 2.35532200<br>C -2.68636100 -1.09397000 3.46911800<br>C -1.70041700 -2.06744200 3.31652400<br>C -1.25651600 -2.38661100 2.03810900<br>C -1.78227500 -1.73300100 0.92319300<br>C -4.85923900 -0.71457100 -0.22469000<br>C -5.02075400 -1.99200700 -0.77482100<br>C -6.25617500 -2.62996900 -0.75499700<br>C -7.36067300 -2.01028700 -0.17385600<br>C -7.21153100 -0.74737500 0.38860700<br>C -5.97287700 -0.10782600 0.36368700

	C	-3.50895100	1.47923600	-0.04948700
	C	-4.33106100	2.18765100	-0.93770400
	C	-4.35576800	3.57894100	-0.93574900
	C	-3.54999700	4.29472000	-0.05230500
	C	-2.71115200	3.60302300	0.81577200
	C	-2.68494700	2.20872200	0.81168700
	H	-3.99169100	0.28998300	2.49580000
	H	-3.05543700	-0.84135400	4.45706500
	H	-1.29045700	-2.57386500	4.18303500
	H	-0.49341100	-3.14351300	1.89689700
	H	-1.40773300	-2.00109600	-0.05412900
	H	-4.17853300	-2.49571300	-1.22993200
	H	-6.35471100	-3.61590200	-1.19558200
	H	-8.32381800	-2.50786400	-0.15907600
	H	-8.05876200	-0.25164600	0.84960900
	H	-5.88275600	0.87634500	0.80511200
	H	-4.95320400	1.64384200	-1.63814100
	H	-5.00499300	4.10277600	-1.62849000
	H	-3.57024100	5.37867600	-0.04782400
	H	-2.06422000	4.14484600	1.49669200
	H	-2.01121400	1.69221000	1.48144900
	H	0.41301600	-1.34227400	-3.90968000
	H	-0.34448800	2.33826900	-0.96904800
	H	-0.46463700	2.82097200	-2.66785600
	O	1.44595000	0.33956300	-1.36330200
	C	2.73955200	0.71291700	-1.34085200
	C	3.47805700	0.09993400	-0.11724000
	C	5.01965500	0.14136500	-0.26368100
	C	5.80808300	0.03319500	0.89023400
	O	3.21697200	1.49229500	-2.12710500
	C	5.67055000	0.14561700	-1.50179700
	C	7.06042700	0.06240300	-1.57848100
	C	7.83042800	-0.03252300	-0.42454900
	C	7.19422700	-0.04871000	0.81459100
	C	3.13953000	-1.39814900	0.06030000
	C	3.01522800	-2.22146000	-1.06493200
	C	2.82862600	-3.59458700	-0.93861900
	C	2.77174500	-4.18135800	0.32367400
	C	2.91293200	-3.37797700	1.45126300
	C	3.09818900	-2.00271300	1.32014600
	C	2.96816900	1.02129200	1.02424900
	C	1.76797900	0.75739000	1.69577300
	C	1.28463500	1.63389000	2.66445900
	C	1.97573100	2.80588500	2.96262400
	C	3.14791400	3.09969400	2.27157300
	C	3.63597800	2.21891800	1.30919300
	H	5.33480200	0.01461700	1.86406400
	H	5.10328800	0.23663400	-2.41546700
	H	7.53853700	0.07559300	-2.55179500
	H	8.91097400	-0.09479500	-0.48805600
	H	7.77589000	-0.12785200	1.72639300
	H	3.07661200	-1.79169000	-2.05816300

	H	2.73384400	-4.20646100	-1.82885600
	H	2.62941800	-5.25121100	0.42549600
	H	2.88545800	-3.81967300	2.44135400
	H	3.21258300	-1.40190800	2.21281700
	H	1.19718300	-0.13010200	1.45872200
	H	0.36306400	1.39425300	3.18310600
	H	1.60034700	3.48639500	3.71878900
	H	3.68771500	4.01726000	2.47753100
	H	4.54471000	2.46676900	0.77595900
11	O	-0.37515800	1.41559700	-1.27918900
	C	-0.04574600	2.70422700	-1.49641900
	C	-0.54152300	0.53965800	-2.43396800
	H	-0.38245900	1.14614700	-3.32733000
	C	0.54152300	-0.53965800	-2.43396800
	H	0.38245900	-1.14614700	-3.32733000
	C	-1.96179600	-0.00447900	-2.40343900
	H	-2.16826900	-0.50501900	-1.45746300
	H	-2.10615700	-0.72025300	-3.21619500
	C	1.96179600	0.00447900	-2.40343900
	H	2.68021600	-0.80782900	-2.53080500
	O	0.17468800	3.16664000	-2.58749800
	C	0.11772100	3.47560600	-0.15522900
	C	1.52739500	2.99702100	0.28484500
	C	1.70016400	1.85657300	1.07749100
	C	2.97461000	1.38540400	1.38548700
	C	4.10237200	2.03543700	0.89039600
	C	3.94319700	3.15090200	0.07192500
	C	2.66893900	3.62242900	-0.23302800
	C	-1.01193100	3.15411300	0.85030800
	C	-2.31286600	2.89948300	0.40356700
	C	-3.36434000	2.73439900	1.30055700
	C	-3.13914700	2.82580600	2.67182500
	C	-1.85214800	3.09251900	3.13116600
	C	-0.80354200	3.25984700	2.22986000
	C	0.04574600	5.00954500	-0.35530900
	C	0.66792200	5.85510800	0.57196000
	C	0.52890900	7.23771300	0.49598000
	C	-0.24463900	7.81041900	-0.51016400
	C	-0.87768000	6.98202500	-1.43128500
	C	-0.73791300	5.59784000	-1.35388700
	H	0.83601300	1.32271900	1.44817300
	H	3.07933800	0.49923000	2.00086500
	H	5.09452300	1.67000900	1.13087400
	H	4.81116400	3.65681200	-0.33612800
	H	2.56105400	4.48185000	-0.88214700
	H	-2.51925000	2.83290100	-0.65737700
	H	-4.36167300	2.53696100	0.92355400
	H	-3.95705500	2.69996600	3.37229400
	H	-1.66003100	3.17756200	4.19503200
	H	0.18580800	3.47782400	2.61096800
	H	1.27422000	5.43296600	1.36367700
	H	1.02649100	7.86576000	1.22672800

	H	-0.35320100	8.88727100	-0.57385400
	H	-1.48504300	7.41043300	-2.22096400
	H	-1.23153900	4.98338400	-2.09223100
	H	-2.68021600	0.80782900	-2.53080500
	H	2.16826900	0.50501900	-1.45746300
	H	2.10615700	0.72025300	-3.21619500
	O	0.37515800	-1.41559700	-1.27918900
	C	0.04574600	-2.70422700	-1.49641900
	C	-0.11772100	-3.47560600	-0.15522900
	C	-1.52739500	-2.99702100	0.28484500
	C	-1.70016400	-1.85657300	1.07749100
	O	-0.17468800	-3.16664000	-2.58749800
	C	-2.66893900	-3.62242900	-0.23302800
	C	-3.94319700	-3.15090200	0.07192500
	C	-4.10237200	-2.03543700	0.89039600
	C	-2.97461000	-1.38540400	1.38548700
	C	1.01193100	-3.15411300	0.85030800
	C	2.31286600	-2.89948300	0.40356700
	C	3.36434000	-2.73439900	1.30055700
	C	3.13914700	-2.82580600	2.67182500
	C	1.85214800	-3.09251900	3.13116600
	C	0.80354200	-3.25984700	2.22986000
	C	-0.04574600	-5.00954500	-0.35530900
	C	0.73791300	-5.59784000	-1.35388700
	C	0.87768000	-6.98202500	-1.43128500
	C	0.24463900	-7.81041900	-0.51016400
	C	-0.52890900	-7.23771300	0.49598000
	C	-0.66792200	-5.85510800	0.57196000
	H	-0.83601300	-1.32271900	1.44817300
	H	-2.56105400	-4.48185000	-0.88214700
	H	-4.81116400	-3.65681200	-0.33612800
	H	-5.09452300	-1.67000900	1.13087400
	H	-3.07933800	-0.49923000	2.00086500
	H	2.51925000	-2.83290100	-0.65737700
	H	4.36167300	-2.53696100	0.92355400
	H	3.95705500	-2.69996600	3.37229400
	H	1.66003100	-3.17756200	4.19503200
	H	-0.18580800	-3.47782400	2.61096800
	H	1.23153900	-4.98338400	-2.09223100
	H	1.48504300	-7.41043300	-2.22096400
	H	0.35320100	-8.88727100	-0.57385400
	H	-1.02649100	-7.86576000	1.22672800
	H	-1.27422000	-5.43296600	1.36367700
14	O	1.36749800	0.64157800	-1.07900700
	C	2.60265600	0.20676800	-1.39317200
	C	0.54885000	1.22454500	-2.13699100
	H	1.19043100	1.36161600	-3.00965500
	C	-0.56508500	0.26177600	-2.55231700
	H	-1.15660800	0.77079100	-3.31557700
	C	0.04439000	2.56389600	-1.62212400
	H	0.88628700	3.22882400	-1.42249400
	H	-0.52607300	2.43822500	-0.70158500

	C	-0.07809400	-1.08180400	-3.07473700
	H	0.64345400	-0.93529700	-3.88079100
	O	3.02368800	0.13988000	-2.52110400
	C	3.45911900	-0.06132000	-0.12316400
	C	4.51983600	-1.16550200	-0.34983800
	C	5.54582800	-1.30780000	0.59550700
	C	6.47798300	-2.33465400	0.50212200
	C	6.40163800	-3.25831500	-0.53873800
	C	5.38380600	-3.13612800	-1.47718300
	C	4.45187500	-2.10167100	-1.38573000
	C	2.61739700	-0.54322900	1.08077900
	C	1.62842400	-1.51616000	0.89492700
	C	0.92423000	-2.04623700	1.97044100
	C	1.20519900	-1.62335700	3.26868900
	C	2.20013800	-0.67268900	3.47246800
	C	2.89932900	-0.14002700	2.38953100
	C	4.09379100	1.34428600	0.07036000
	C	3.36077300	2.38103300	0.66442200
	C	3.88855200	3.66610400	0.75906400
	C	5.15275400	3.94670400	0.24573100
	C	5.87600200	2.93270300	-0.37501400
	C	5.34865900	1.64648400	-0.46901300
	H	5.61718500	-0.60853400	1.41973300
	H	7.26148300	-2.41539900	1.24750000
	H	7.12570000	-4.06177300	-0.61387900
	H	5.30896200	-3.84534100	-2.29420900
	H	3.68897800	-2.02173400	-2.14539400
	H	1.40172300	-1.87295700	-0.10191200
	H	0.15358000	-2.78748000	1.79210300
	H	0.65817700	-2.03539700	4.10913100
	H	2.44088900	-0.34186900	4.47694400
	H	3.67014900	0.59663500	2.57571600
	H	2.36939200	2.18366500	1.05073800
	H	3.30612200	4.44910000	1.23202500
	H	5.56464000	4.94682300	0.32069800
	H	6.85403800	3.13965800	-0.79490600
	H	5.91799400	0.87502500	-0.96968700
	H	-0.59857300	3.03419500	-2.37012900
	H	-0.91751300	-1.66364500	-3.46077400
	H	0.40172900	-1.65745200	-2.28217900
	O	-1.44046300	0.00632800	-1.41667400
	C	-2.72967100	0.39312400	-1.49691400
	C	-3.48693500	0.07487300	-0.17609200
	C	-3.19804700	-1.35557000	0.33493100
	C	-3.34371000	-1.67571400	1.68929700
	O	-3.19619200	0.98108000	-2.43946900
	C	-2.93004100	-2.39888600	-0.55754100
	C	-2.78777800	-3.71064300	-0.11283300
	C	-2.91828700	-4.01166800	1.24030000
	C	-3.20177200	-2.98605200	2.13858700
	C	-5.02312600	0.13012300	-0.36290000
	C	-5.63969200	-0.25563300	-1.55830100

	C	-7.02763900	-0.31181000	-1.66228200
	C	-7.83248200	0.00719800	-0.57293700
	C	-7.23223000	0.37955300	0.62661400
	C	-5.84516800	0.43683200	0.72865400
	C	-2.95446900	1.20428700	0.74693600
	C	-1.82858200	1.02227000	1.55771500
	C	-1.31548800	2.07603000	2.31177900
	C	-1.90406100	3.33679100	2.25340400
	C	-3.00255800	3.53883400	1.42122300
	C	-3.51843900	2.48476700	0.67166200
	H	-3.57214600	-0.89708000	2.40560500
	H	-2.83427000	-2.19684000	-1.61699100
	H	-2.57792000	-4.49790200	-0.82844300
	H	-2.80961400	-5.03257700	1.58848800
	H	-3.31973800	-3.20380400	3.19437200
	H	-5.04289700	-0.49844300	-2.42505800
	H	-7.47793200	-0.60555100	-2.60405300
	H	-8.91259900	-0.03533200	-0.65662600
	H	-7.84199900	0.62640800	1.48875100
	H	-5.40173400	0.72906200	1.67219000
	H	-1.33469600	0.06106000	1.59410200
	H	-0.44802300	1.90392700	2.93896500
	H	-1.50561000	4.15545800	2.84230800
	H	-3.46108200	4.51892800	1.35072000
	H	-4.36538000	2.65941900	0.02033700
16	O	0.07408100	1.46221100	1.35017800
	C	0.28333200	2.78638800	1.49501100
	C	0.44537800	0.61808600	2.48190000
	H	0.21580600	1.17838800	3.39118700
	C	-0.44537800	-0.61808600	2.48190000
	H	-0.21580600	-1.17838800	3.39118700
	C	1.93650900	0.31603000	2.43020600
	H	2.20867900	-0.34963700	3.25429600
	H	2.50931700	1.23785000	2.53488400
	C	-1.93650900	-0.31603000	2.43020600
	H	-2.20867900	0.34963700	3.25429600
	O	0.67413600	3.28534600	2.52091000
	C	0.09107100	3.55281600	0.15752200
	C	-1.06156700	2.97016200	-0.69185600
	C	-0.98451100	2.85454500	-2.08223000
	C	-2.09032400	2.45261200	-2.83145700
	C	-3.29792400	2.16280800	-2.20525600
	C	-3.39140700	2.28383000	-0.81927300
	C	-2.28738300	2.68677200	-0.07665000
	C	1.50457100	3.38088000	-0.46423800
	C	1.86952000	2.18168800	-1.09086300
	C	3.16179100	1.99105900	-1.57262700
	C	4.12276000	2.98788500	-1.42023100
	C	3.78219700	4.16875400	-0.76697300
	C	2.48844000	4.36011400	-0.28680900
	C	-0.28333200	5.04126400	0.36725000
	C	-0.80378600	5.55222700	1.55993100

	C	-1.21721300	6.88226200	1.64667800
	C	-1.12128400	7.72842600	0.54839300
	C	-0.61037800	7.22966900	-0.64859600
	C	-0.20341000	5.90370600	-0.73593300
	H	-0.05928100	3.08289300	-2.59486800
	H	-2.00295900	2.37349400	-3.90949000
	H	-4.15963700	1.85757200	-2.78822900
	H	-4.32764900	2.07077300	-0.31554200
	H	-2.38896100	2.79859000	0.99678800
	H	1.14487300	1.38502600	-1.19210700
	H	3.41323100	1.05485200	-2.05764400
	H	5.12921900	2.84055600	-1.79585600
	H	4.52442000	4.94598800	-0.62275600
	H	2.24571200	5.27774900	0.23189600
	H	-0.86897700	4.92816800	2.43803800
	H	-1.61247200	7.25287400	2.58612800
	H	-1.44114400	8.76184300	0.62054200
	H	-0.53239100	7.87187400	-1.51891600
	H	0.18073400	5.53388300	-1.67887700
	H	2.19856600	-0.16535100	1.48843800
	H	-2.50931700	-1.23785000	2.53488400
	H	-2.19856600	0.16535100	1.48843800
	O	-0.07408100	-1.46221100	1.35017800
	C	-0.28333200	-2.78638800	1.49501100
	C	-0.09107100	-3.55281600	0.15752200
	C	0.28333200	-5.04126400	0.36725000
	C	0.20341000	-5.90370600	-0.73593300
	O	-0.67413600	-3.28534600	2.52091000
	C	0.80378600	-5.55222700	1.55993100
	C	1.21721300	-6.88226200	1.64667800
	C	1.12128400	-7.72842600	0.54839300
	C	0.61037800	-7.22966900	-0.64859600
	C	1.06156700	-2.97016200	-0.69185600
	C	2.28738300	-2.68677200	-0.07665000
	C	3.39140700	-2.28383000	-0.81927300
	C	3.29792400	-2.16280800	-2.20525600
	C	2.09032400	-2.45261200	-2.83145700
	C	0.98451100	-2.85454500	-2.08223000
	C	-1.50457100	-3.38088000	-0.46423800
	C	-1.86952000	-2.18168800	-1.09086300
	C	-3.16179100	-1.99105900	-1.57262700
	C	-4.12276000	-2.98788500	-1.42023100
	C	-3.78219700	-4.16875400	-0.76697300
	C	-2.48844000	-4.36011400	-0.28680900
	H	-0.18073400	-5.53388300	-1.67887700
	H	0.86897700	-4.92816800	2.43803800
	H	1.61247200	-7.25287400	2.58612800
	H	1.44114400	-8.76184300	0.62054200
	H	0.53239100	-7.87187400	-1.51891600
	H	2.38896100	-2.79859000	0.99678800
	H	4.32764900	-2.07077300	-0.31554200
	H	4.15963700	-1.85757200	-2.78822900

	H	2.00295900	-2.37349400	-3.90949000
	H	0.05928100	-3.08289300	-2.59486800
	H	-1.14487300	-1.38502600	-1.19210700
	H	-3.41323100	-1.05485200	-2.05764400
	H	-5.12921900	-2.84055600	-1.79585600
	H	-4.52442000	-4.94598800	-0.62275600
	H	-2.24571200	-5.27774900	0.23189600
17	O	1.41002400	-0.27131900	1.42051500
	C	2.69100800	0.13423100	1.54760900
	C	0.54418800	-0.20977800	2.59338300
	H	1.15831500	0.11397200	3.43557300
	C	-0.53099700	0.86059200	2.39386700
	H	-1.16641100	0.83721400	3.28097300
	C	0.00318700	-1.61008800	2.84196600
	H	-0.52017800	-1.99467300	1.96594700
	H	0.82198500	-2.29207000	3.07986300
	C	0.01914400	2.26014400	2.16446300
	H	-0.79876200	2.98128500	2.12096200
	O	3.15379600	0.57709900	2.56821100
	C	3.44307400	0.05966200	0.18852500
	C	3.12432800	-1.23885800	-0.58734600
	C	3.14695400	-1.27834000	-1.98521200
	C	2.99923000	-2.48122100	-2.67298500
	C	2.83272100	-3.67465200	-1.97633800
	C	2.81964600	-3.65164500	-0.58331500
	C	2.96694500	-2.44818000	0.09941100
	C	4.98152200	0.04146200	0.36974200
	C	5.60462300	-0.47844600	1.50900600
	C	6.99287300	-0.58452400	1.57942700
	C	7.78941700	-0.18030700	0.51347400
	C	7.18138400	0.32870800	-0.63152500
	C	5.79610800	0.43332400	-0.70104000
	C	2.94059600	1.36254000	-0.49084500
	C	3.59000900	2.57765300	-0.23882100
	C	3.09778900	3.77260700	-0.75777500
	C	1.93582700	3.77982300	-1.52515200
	C	1.26245800	2.58295000	-1.75584100
	C	1.75621000	1.38806100	-1.23719600
	H	3.28595700	-0.36503000	-2.54903800
	H	3.01969200	-2.48147300	-3.75716600
	H	2.72131100	-4.61144400	-2.51052900
	H	2.69769300	-4.57262300	-0.02418700
	H	2.96979000	-2.45993700	1.18271700
	H	5.01593100	-0.78674800	2.35994200
	H	7.44878600	-0.98455400	2.47838500
	H	8.86895400	-0.26182200	0.57190600
	H	7.78440700	0.64406000	-1.47586800
	H	5.34544300	0.82793900	-1.60318600
	H	4.48424300	2.59093600	0.37064700
	H	3.62306800	4.69909900	-0.55396600
	H	1.55147900	4.70999300	-1.92842600
	H	0.33946100	2.57493200	-2.32404800

	H	1.20384800	0.47389300	-1.40708600
	H	-0.69303700	-1.59804600	3.68338700
	H	0.57474400	2.31726200	1.22840600
	H	0.68512100	2.53904400	2.98445900
	O	-1.37271800	0.53340400	1.24724800
	C	-2.63882500	0.12323100	1.47357700
	C	-3.43665800	-0.05705800	0.13543400
	C	-3.19154100	1.19656000	-0.73734100
	C	-2.95251200	1.11401700	-2.10964600
	O	-3.08319100	-0.06020500	2.57725300
	C	-3.27886000	2.46958000	-0.15728600
	C	-3.12285900	3.62080600	-0.92051800
	C	-2.88266100	3.52530800	-2.29122800
	C	-2.80139600	2.26842400	-2.88071800
	C	-4.94140000	-0.19229600	0.48017600
	C	-5.37248100	-1.16740100	1.39345000
	C	-6.72176400	-1.33575500	1.68022100
	C	-7.68159000	-0.53959300	1.05609900
	C	-7.27164600	0.42004000	0.13947400
	C	-5.91534800	0.59026900	-0.14549300
	C	-2.93336400	-1.34134700	-0.59135400
	C	-3.83950400	-2.23050600	-1.18265900
	C	-3.39684400	-3.34882300	-1.88823700
	C	-2.03665900	-3.60748800	-2.01838300
	C	-1.12208200	-2.72718400	-1.44575900
	C	-1.56444800	-1.60515500	-0.74887400
	H	-2.88608900	0.14618600	-2.58859500
	H	-3.48662100	2.56382900	0.90344800
	H	-3.19303500	4.59320600	-0.44582600
	H	-2.76486400	4.42152300	-2.88996400
	H	-2.62211400	2.17715100	-3.94648700
	H	-4.64860400	-1.80094100	1.88552000
	H	-7.02402200	-2.09347000	2.39473600
	H	-8.73411400	-0.67118500	1.28119700
	H	-8.00273800	1.04418600	-0.36247400
	H	-5.62727000	1.34185800	-0.86737200
	H	-4.90271300	-2.05412400	-1.09995700
	H	-4.12550100	-4.01687900	-2.33429000
	H	-1.69220300	-4.48028100	-2.56184400
	H	-0.05684300	-2.90489300	-1.53957500
	H	-0.83320000	-0.92727800	-0.33450900
20	O	1.36732800	0.55696600	-1.15499100
	C	2.60401100	0.08250200	-1.41615200
	C	0.53479900	0.96374000	-2.28510900
	H	1.17511500	0.99020400	-3.16856200
	C	-0.55528900	-0.07358700	-2.56434900
	H	-1.14649900	0.31222800	-3.39715600
	C	-0.00502600	2.35117300	-1.97113700
	H	0.81459400	3.06950700	-1.91150200
	H	-0.54512900	2.35763300	-1.02413000
	C	-0.04000600	-1.46816800	-2.88734200
	H	0.43050200	-1.92188000	-2.01466400

	O	3.00330700	-0.12325100	-2.53367000
	C	3.46288300	-0.08540600	-0.11378200
	C	2.56279000	-0.43047700	1.09351700
	C	2.73846900	0.17195800	2.34111400
	C	1.96237800	-0.20795600	3.43758900
	C	0.99395400	-1.19702400	3.30499000
	C	0.81414700	-1.81431900	2.06699200
	C	1.59486100	-1.43690900	0.98065600
	C	4.14925200	1.29934300	0.09644900
	C	3.37353800	2.46802000	0.13083300
	C	3.95745700	3.71221100	0.34646800
	C	5.33244700	3.81989500	0.54496600
	C	6.10993600	2.66746400	0.53302800
	C	5.52514900	1.42028000	0.31093000
	C	4.51780700	-1.20406800	-0.33250400
	C	5.35616700	-1.20211200	-1.45994400
	C	6.32719500	-2.18261400	-1.63483000
	C	6.49893000	-3.18806300	-0.68553600
	C	5.68986000	-3.19367900	0.44376200
	C	4.71300700	-2.21258800	0.61783400
	H	3.48752600	0.94222100	2.46818200
	H	2.12225300	0.27396800	4.39598000
	H	0.38571100	-1.48827000	4.15390900
	H	0.06024800	-2.58362900	1.94541900
	H	1.45242300	-1.94257300	0.03331200
	H	2.30129600	2.40049100	0.00864100
	H	3.33300300	4.59874000	0.36491800
	H	5.78885000	4.78894200	0.71261800
	H	7.18006500	2.73015200	0.69687100
	H	6.15197800	0.53982500	0.31056300
	H	5.24618700	-0.43337700	-2.20904300
	H	6.95378900	-2.15634200	-2.51965000
	H	7.25623300	-3.95169400	-0.82421000
	H	5.81130200	-3.96161200	1.19979600
	H	4.10379600	-2.24393000	1.50995600
	H	-0.68705200	2.67363200	-2.76119400
	H	0.69670300	-1.42380700	-3.69158600
	H	-0.86482500	-2.10991900	-3.20349700
	O	-1.44135600	-0.18733800	-1.41350200
	C	-2.72017100	0.22049200	-1.54180900
	C	-3.49219300	0.05920400	-0.20194800
	C	-2.97582900	1.29672000	0.58151000
	C	-1.81849300	1.23171400	1.36630300
	O	-3.16707000	0.72128400	-2.54275200
	C	-3.58410900	2.54502300	0.39803400
	C	-3.08107200	3.68409800	1.02195000
	C	-1.95096500	3.60037000	1.83145700
	C	-1.31751200	2.37063100	1.99356900
	C	-3.20022600	-1.29603500	0.48279600
	C	-3.29856500	-1.43977600	1.87068100
	C	-3.15943500	-2.68618800	2.47689700
	C	-2.92434500	-3.82177800	1.70642100

	C	-2.84010400	-3.69596200	0.32193900
	C	-2.98147100	-2.44857500	-0.27957400
	C	-5.02703100	0.07925700	-0.40660700
	C	-5.85705800	0.44725900	0.66036000
	C	-7.24246500	0.36787100	0.55907600
	C	-7.83460200	-0.09002100	-0.61511600
	C	-7.02257200	-0.46996200	-1.67867200
	C	-5.63503600	-0.39018000	-1.57585300
	H	-1.29048000	0.29472000	1.47879300
	H	-4.45586200	2.62837700	-0.23815500
	H	-3.57443400	4.63771300	0.87013900
	H	-1.56204900	4.48589300	2.32176900
	H	-0.42336800	2.28933200	2.60112300
	H	-3.48769600	-0.57319700	2.49106400
	H	-3.24112000	-2.76683300	3.55528700
	H	-2.81743500	-4.79268700	2.17678800
	H	-2.66876600	-4.57092900	-0.29527200
	H	-2.92664500	-2.38280800	-1.35931300
	H	-5.41947400	0.80403700	1.58428100
	H	-7.85779900	0.66355900	1.40166400
	H	-8.91386300	-0.15110400	-0.69833900
	H	-7.46610500	-0.83042100	-2.60027700
	H	-5.03342800	-0.68011700	-2.42435300
21	O	-1.44668000	0.11121700	1.41097700
	C	-2.75885300	0.40558200	1.50265700
	C	-0.60943000	0.46649600	2.55090800
	H	-1.17858200	0.23457500	3.45524500
	C	0.60858200	-0.45040800	2.54079600
	H	1.20550100	-0.20704800	3.42254000
	C	-0.27632500	1.95207500	2.53484000
	H	0.36669700	2.19200300	3.38628400
	H	-1.18907400	2.54104700	2.62228000
	C	0.26569500	-1.93399800	2.54712900
	H	-0.38552500	-2.16041100	3.39629400
	O	-3.25439100	0.91457800	2.47648000
	C	-3.49864900	0.09803700	0.16855700
	C	-5.03922400	0.14505100	0.32744700
	C	-5.83914100	0.45831700	-0.77904500
	C	-7.22774900	0.39399400	-0.70849300
	C	-7.85273500	0.00747300	0.47378000
	C	-7.07033600	-0.31939300	1.57692800
	C	-5.68051400	-0.25694100	1.50458400
	C	-3.19833500	-1.33192500	-0.33545300
	C	-2.96123600	-2.37094700	0.57028400
	C	-2.81490900	-3.68643500	0.13767000
	C	-2.90845600	-3.99378000	-1.21675900
	C	-3.16054300	-2.97220200	-2.12841700
	C	-3.30957100	-1.65867300	-1.69142600
	C	-2.96168700	1.23265200	-0.74366800
	C	-1.83641800	1.05875000	-1.55701300
	C	-1.32847700	2.11761600	-2.30688100
	C	-1.92061300	3.37670300	-2.24073100

	C	-3.01608400	3.57176400	-1.40321100
	C	-3.52687000	2.51179500	-0.65854300
	H	-5.37705900	0.76096100	-1.71023800
	H	-7.81914600	0.64622800	-1.58180300
	H	-8.93423000	-0.04063200	0.53319400
	H	-7.53976000	-0.62521200	2.50543400
	H	-5.10219700	-0.50700900	2.38116800
	H	-2.89851100	-2.16228200	1.63150400
	H	-2.63114300	-4.47083700	0.86350100
	H	-2.79511300	-5.01701200	-1.55633600
	H	-3.24800500	-3.19553500	-3.18592500
	H	-3.51806900	-0.88390300	-2.41791400
	H	-1.34442900	0.09642900	-1.60096200
	H	-0.46134600	1.95472800	-2.93670300
	H	-1.52750300	4.19820100	-2.82928400
	H	-3.47697400	4.55019000	-1.32585200
	H	-4.37451400	2.67990400	-0.00620300
	H	0.24753000	2.22892700	1.62137400
	H	1.17395700	-2.52771100	2.65405600
	H	-0.24790500	-2.21306300	1.62729400
	O	1.41865600	-0.13620500	1.36748200
	C	2.74311200	-0.37248900	1.47008600
	C	3.51132700	-0.07197000	0.13484200
	C	2.84235300	-0.90203300	-0.98795800
	C	2.73222700	-0.41950400	-2.29457800
	O	3.24858300	-0.79095300	2.48008700
	C	2.41330800	-2.20901700	-0.72887100
	C	1.86915600	-3.00050800	-1.73522400
	C	1.75471300	-2.50421500	-3.03232200
	C	2.19479300	-1.21345900	-3.30796200
	C	4.98710700	-0.51153200	0.30661300
	C	5.73549200	-0.09899000	1.42068000
	C	7.07286400	-0.45226000	1.55982100
	C	7.70416400	-1.22563100	0.58687000
	C	6.97976500	-1.63504000	-0.52545300
	C	5.63663800	-1.28122200	-0.66261100
	C	3.41161300	1.45361900	-0.17127600
	C	2.16022800	2.04891000	-0.39712000
	C	2.04886600	3.40275300	-0.69610300
	C	3.18754500	4.20134600	-0.78876400
	C	4.43464400	3.62259700	-0.58958200
	C	4.54569500	2.26417200	-0.28777700
	H	3.07209600	0.58057500	-2.52957300
	H	2.51558300	-2.62278500	0.26806500
	H	1.53386100	-4.00532200	-1.50465400
	H	1.32953200	-3.11855900	-3.81779400
	H	2.12305500	-0.81693100	-4.31483900
	H	5.27102100	0.50020300	2.19042400
	H	7.62225500	-0.12250100	2.43472500
	H	8.74648000	-1.50327600	0.69754600
	H	7.45256300	-2.23509800	-1.29510000
	H	5.09904900	-1.61644700	-1.53860300

	H	1.26558800	1.44519900	-0.34882200
	H	1.06558400	3.82842900	-0.86255500
	H	3.10125600	5.25687300	-1.02139900
	H	5.33460800	4.22215300	-0.67087100
	H	5.53114700	1.84305000	-0.15121700
22	O	-1.42073500	-0.35226700	-1.26455900
	C	-2.72939400	-0.66481500	-1.29818500
	C	-0.58825900	-0.83685200	-2.35793300
	H	-1.18875600	-0.78979100	-3.26985700
	C	0.57217900	0.13992600	-2.51881100
	H	1.17985700	-0.20422200	-3.35884300
	C	-0.15977500	-2.27342600	-2.09628000
	H	-1.03600400	-2.92068300	-2.04974900
	H	0.39837200	-2.34858900	-1.16351800
	C	0.14404300	1.58213100	-2.75292700
	H	1.02107000	2.21110000	-2.91097300
	O	-3.22569400	-1.29326000	-2.19855700
	C	-3.50954800	-0.05457400	-0.08462800
	C	-2.87984400	-0.52459500	1.26187600
	C	-1.53495500	-0.25972400	1.57054100
	C	-0.98707900	-0.63993800	2.79174200
	C	-1.76406900	-1.29859200	3.74267300
	C	-3.09792800	-1.56134000	3.45670500
	C	-3.64925400	-1.17411800	2.23473600
	C	-4.99188600	-0.49172700	-0.17118500
	C	-5.33562400	-1.82968900	-0.41572800
	C	-6.66441600	-2.23918000	-0.42765500
	C	-7.68763200	-1.32346400	-0.18777600
	C	-7.36220200	0.00315400	0.06795200
	C	-6.02839100	0.41267700	0.07587900
	C	-3.40792100	1.48414500	-0.26154100
	C	-3.15906300	2.35115400	0.80389600
	C	-3.13593700	3.73328600	0.60896800
	C	-3.35891400	4.27190800	-0.65379200
	C	-3.61631500	3.41589600	-1.72379400
	C	-3.64516400	2.03951100	-1.52661500
	H	-0.90569700	0.24900300	0.85688600
	H	0.05026000	-0.40286500	2.99965900
	H	-1.33587900	-1.59397700	4.69416600
	H	-3.72468300	-2.06510400	4.18437700
	H	-4.69336700	-1.38177500	2.05162900
	H	-4.56195700	-2.56093500	-0.60205100
	H	-6.89978500	-3.27880700	-0.62711000
	H	-8.72373700	-1.64295800	-0.20016900
	H	-8.14356900	0.72982600	0.26133100
	H	-5.80320400	1.45093000	0.27895200
	H	-2.98614200	1.95370100	1.79493500
	H	-2.94526100	4.38661900	1.45333500
	H	-3.33795500	5.34541400	-0.80398900
	H	-3.80080100	3.81986200	-2.71307200
	H	-3.87144700	1.38760700	-2.36350000
	H	0.47902000	-2.62431400	-2.91134400

	H	-0.43362900	1.96121000	-1.90954200
	H	-0.47934700	1.64280300	-3.64911100
	O	1.39999800	0.04959000	-1.32114600
	C	2.72200200	0.26828000	-1.47248500
	C	3.50252500	-0.02083600	-0.15754100
	C	2.92265800	0.72879400	1.06405900
	C	3.37128600	0.39400400	2.34947100
	O	3.22752700	0.56387900	-2.52539100
	C	2.06598600	1.82609000	0.93783800
	C	1.65326500	2.54991700	2.05641500
	C	2.09124200	2.19364600	3.32695200
	C	2.95713700	1.11038000	3.46735500
	C	4.96784400	0.46594000	-0.27619800
	C	5.25470700	1.72805200	-0.81114000
	C	6.55445700	2.22221800	-0.82320000
	C	7.60023400	1.47158800	-0.28992000
	C	7.32774000	0.22284700	0.25742400
	C	6.02463700	-0.27318400	0.26415900
	C	3.39559500	-1.56663600	-0.06544100
	C	4.08754200	-2.34623100	-1.00408500
	C	3.96941300	-3.73252700	-1.01076000
	C	3.14913500	-4.37153600	-0.08279100
	C	2.43915600	-3.60718700	0.83771400
	C	2.55473400	-2.21707200	0.84110600
	H	4.06247200	-0.42965300	2.47859000
	H	1.70366900	2.12909500	-0.03481400
	H	0.98421000	3.39301100	1.92616800
	H	1.76875000	2.75479100	4.19656200
	H	3.32047600	0.82648300	4.44886200
	H	4.46161100	2.33216800	-1.23005600
	H	6.74935800	3.19930400	-1.25121700
	H	8.61327100	1.85773600	-0.30007000
	H	8.12773000	-0.37380300	0.68175300
	H	5.83892900	-1.24937500	0.69311400
	H	4.72270900	-1.86131000	-1.73576000
	H	4.51909300	-4.31268900	-1.74358800
	H	3.05890500	-5.45192900	-0.08376400
	H	1.78309500	-4.08754400	1.55504700
	H	1.97590400	-1.64366800	1.55164800
23	O	-1.42835100	1.46867800	-0.15176100
	C	-2.28481300	0.90026400	0.72125500
	C	-0.68526000	2.63151700	0.31781800
	H	-0.47835600	2.49019600	1.37933200
	C	0.62641500	2.64900200	-0.46020200
	H	0.43467700	2.45973800	-1.51745300
	C	-1.55046000	3.86952000	0.10657700
	H	-2.50862200	3.73440300	0.60916800
	H	-1.73943600	4.04135100	-0.95592500
	C	1.42726200	3.93458600	-0.28397800
	H	2.39247300	3.83643400	-0.77972800
	O	-2.35048200	1.20816000	1.88424300
	C	-3.27023800	-0.06943700	0.01032100

	C	-3.93460100	-1.03132700	1.02305100
	C	-5.23330900	-1.50495200	0.80578000
	C	-5.80285900	-2.45799800	1.64683400
	C	-5.08104600	-2.96347600	2.72386800
	C	-3.78303400	-2.51006600	2.94289000
	C	-3.21412000	-1.55912900	2.10021200
	C	-2.59641500	-0.98420200	-1.03524600
	C	-1.26292600	-1.38434200	-0.90511700
	C	-0.70786900	-2.31725200	-1.77922600
	C	-1.47259500	-2.86987400	-2.80206900
	C	-2.80515800	-2.48871600	-2.93533500
	C	-3.36004500	-1.56315800	-2.05677300
	C	-4.26714100	0.95841300	-0.59263700
	C	-4.13593800	1.43160200	-1.90294600
	C	-4.98485700	2.42188800	-2.39343500
	C	-5.96825900	2.97443300	-1.57744400
	C	-6.08624000	2.53644100	-0.26047800
	C	-5.24030800	1.54422900	0.22800000
	H	-5.81423300	-1.12617800	-0.02570100
	H	-6.81278000	-2.80387500	1.45578500
	H	-5.52289700	-3.70305700	3.38216800
	H	-3.20536100	-2.89677900	3.77526200
	H	-2.20448800	-1.22565000	2.29456700
	H	-0.64110100	-0.97601700	-0.11868900
	H	0.32612200	-2.61417100	-1.65017600
	H	-1.03837000	-3.59457500	-3.48160300
	H	-3.41997200	-2.91711500	-3.71925400
	H	-4.40302100	-1.29445400	-2.16815500
	H	-3.36542700	1.02797200	-2.54583600
	H	-4.87089600	2.76317400	-3.41646900
	H	-6.62971400	3.74339400	-1.96051600
	H	-6.83630800	2.96826400	0.39276000
	H	-5.33257100	1.22468600	1.25822000
	H	-1.07306300	4.75513800	0.52942600
	H	1.59692100	4.14957200	0.77430100
	H	0.90469800	4.77985900	-0.73483400
	O	1.40746200	1.52996700	0.05992500
	C	2.36560000	1.02336300	-0.73348800
	C	3.26440600	-0.03426300	-0.01228400
	C	4.38797900	0.80073600	0.67651400
	C	4.85662100	0.50074500	1.95934900
	O	2.55400500	1.40737000	-1.86131000
	C	5.02318900	1.83013000	-0.03293100
	C	6.06830700	2.55288000	0.53435200
	C	6.51520600	2.25496000	1.81978400
	C	5.90908300	1.22073400	2.52486500
	C	3.90473900	-0.95533100	-1.07902500
	C	3.18749100	-1.37531200	-2.20695700
	C	3.74212300	-2.26981500	-3.11796000
	C	5.02710900	-2.77072000	-2.92182300
	C	5.74904000	-2.36520800	-1.80434500
	C	5.19312300	-1.46606100	-0.89541800

	C	2.45087100	-0.86982400	1.01324600
	C	2.28933600	-2.25145200	0.86477600
	C	1.56156700	-3.00095500	1.79089800
	C	0.98367000	-2.38688200	2.89444700
	C	1.14407400	-1.01205200	3.06530400
	C	1.86715300	-0.26732600	2.14055000
	H	4.40876100	-0.30446300	2.52538000
	H	4.70944500	2.05604300	-1.04420200
	H	6.53835400	3.34655100	-0.03579500
	H	7.33005400	2.81719000	2.26171400
	H	6.25294300	0.96511600	3.52108100
	H	2.19106100	-0.99766000	-2.39105500
	H	3.16678700	-2.57021000	-3.98671900
	H	5.46011800	-3.46428600	-3.63389700
	H	6.75158100	-2.74251200	-1.63566300
	H	5.77585900	-1.16256600	-0.03558900
	H	2.73738800	-2.76104400	0.02361600
	H	1.45310700	-4.06925100	1.64108900
	H	0.42000000	-2.96811500	3.61539100
	H	0.70392200	-0.51369800	3.92183900
	H	1.98003800	0.79530400	2.29793400
25	O	1.43947800	1.62497400	0.26857200
	C	2.19284800	0.99372200	-0.65853700
	C	0.64966600	2.76758500	-0.17990100
	H	0.44903200	2.63631700	-1.24316400
	C	-0.66647400	2.72180900	0.59206900
	H	-0.47651600	2.50839300	1.64534700
	C	1.46720700	4.03447800	0.04594900
	H	0.95964800	4.90380100	-0.37534200
	H	2.43354900	3.93852500	-0.45075500
	C	-1.50444800	3.98769300	0.44534900
	H	-2.46629100	3.84776500	0.93681600
	O	2.16214800	1.28544600	-1.82670900
	C	3.15463700	-0.06630200	-0.01994200
	C	3.45885400	-1.19586700	-1.04337600
	C	3.45384000	-2.53893900	-0.64922800
	C	3.77896200	-3.55907500	-1.54337500
	C	4.11989500	-3.26090200	-2.85731900
	C	4.14330300	-1.92803500	-3.26257900
	C	3.82554800	-0.90941300	-2.36889600
	C	2.52923200	-0.66936800	1.25777900
	C	1.21518500	-1.15542500	1.22225500
	C	0.65408400	-1.78190700	2.32922700
	C	1.39451800	-1.93905800	3.50080700
	C	2.70179800	-1.46939300	3.54559800
	C	3.26454400	-0.84315100	2.43210200
	C	4.44832100	0.73575500	0.32196000
	C	4.36984000	1.87660000	1.13539100
	C	5.50947400	2.59814400	1.47580200
	C	6.76178700	2.19276100	1.01861300
	C	6.85611500	1.05465900	0.22609300
	C	5.71227600	0.33375900	-0.11896700

	H	3.19349400	-2.80451300	0.36523000
	H	3.76288500	-4.58846400	-1.20248800
	H	4.37056800	-4.05259100	-3.55476400
	H	4.41645400	-1.67337300	-4.28089200
	H	3.85715300	0.11446500	-2.70649800
	H	0.62532900	-1.05953100	0.31840300
	H	-0.36193200	-2.15477000	2.27116800
	H	0.95608300	-2.42591400	4.36477400
	H	3.29457500	-1.58975200	4.44572000
	H	4.28775100	-0.49588100	2.48648000
	H	3.41006200	2.18905200	1.52330800
	H	5.41736900	3.47584100	2.10618500
	H	7.65102800	2.75382700	1.28299500
	H	7.82317700	0.71729400	-0.13021500
	H	5.81582800	-0.54992400	-0.73252200
	H	1.63964900	4.21291100	1.11073800
	H	-1.67893500	4.22300500	-0.60772700
	H	-1.00796900	4.83645600	0.91875800
	O	-1.40174400	1.59353400	0.03189100
	C	-2.37376600	1.04646300	0.78209900
	C	-3.18947900	-0.04008100	0.00400500
	C	-4.14693200	0.77400200	-0.91620200
	C	-4.39471500	0.41970100	-2.24478900
	O	-2.62615000	1.42220900	1.89960900
	C	-4.85664000	1.85641100	-0.37723300
	C	-5.76174000	2.57693600	-1.14986700
	C	-5.98959500	2.22311800	-2.47836300
	C	-5.30770200	1.13781000	-3.01804000
	C	-4.03529700	-0.86240200	1.00623100
	C	-3.52942600	-1.24462400	2.25615000
	C	-4.27055100	-2.05305500	3.11337300
	C	-5.53435300	-2.50570800	2.74105800
	C	-6.04594800	-2.14043300	1.50063000
	C	-5.30395700	-1.32711600	0.64544600
	C	-2.24605500	-0.97336700	-0.80630000
	C	-2.17355400	-2.34536600	-0.54022500
	C	-1.31993700	-3.18013100	-1.26404500
	C	-0.51918000	-2.66448900	-2.27504800
	C	-0.58975800	-1.30258900	-2.56452800
	C	-1.44358800	-0.47312300	-1.84597500
	H	-3.88194200	-0.42505500	-2.68401100
	H	-4.71329500	2.12574500	0.66240300
	H	-6.29500400	3.41189100	-0.70893700
	H	-6.69531200	2.78286600	-3.08172500
	H	-5.48311900	0.84067300	-4.04612400
	H	-2.55621000	-0.90011100	2.57576600
	H	-3.85755300	-2.32477900	4.07861700
	H	-6.11224300	-3.13204000	3.41143500
	H	-7.02767100	-2.48271200	1.19241500
	H	-5.72468600	-1.05492100	-0.31357400
	H	-2.78606600	-2.78027500	0.23650100
	H	-1.28555000	-4.23742500	-1.02565800

	H	0.15524600	-3.30762900	-2.82810900
	H	0.03055000	-0.87770300	-3.34494900
	H	-1.48202600	0.57670900	-2.09402400
43	O	-1.39102600	-1.60909100	-0.43239000
	C	-2.35860200	-0.88439200	-1.01756200
	C	-0.61755200	-2.52098000	-1.26722100
	H	-0.43347900	-2.03370300	-2.22642400
	C	0.70644300	-2.72497200	-0.52900700
	H	0.50540800	-2.82624100	0.53803800
	C	-1.42471400	-3.79933700	-1.46784000
	H	-0.90324800	-4.48791800	-2.13458400
	H	-2.38210500	-3.55510200	-1.92836500
	C	1.53764500	-3.89275600	-1.04569700
	H	1.71135800	-3.80875900	-2.12161900
	O	-2.54116000	-0.87727800	-2.21036000
	C	-3.18731700	-0.02278800	-0.00772600
	C	-3.24602900	-0.68109200	1.39852500
	C	-2.07484900	-0.87718700	2.15018000
	C	-2.12007100	-1.45071200	3.41540700
	C	-3.33717700	-1.84105500	3.97304500
	C	-4.50516300	-1.64298400	3.24839100
	C	-4.45953700	-1.06635700	1.97735500
	C	-4.61221500	0.14107100	-0.59344900
	C	-5.27572400	-0.94732700	-1.17564700
	C	-6.58199500	-0.83141900	-1.63828900
	C	-7.26210300	0.37988800	-1.52622100
	C	-6.61890900	1.46684200	-0.94529600
	C	-5.30740000	1.34778600	-0.48519900
	C	-2.48982900	1.36936500	0.04017500
	C	-2.36793200	2.08950100	1.23288000
	C	-1.82323000	3.37371900	1.24175900
	C	-1.38520400	3.96265300	0.06001300
	C	-1.50978800	3.25866600	-1.13539400
	C	-2.06198300	1.98097500	-1.14682100
	H	-1.11316000	-0.58932300	1.75114200
	H	-1.19515400	-1.58906200	3.96420200
	H	-3.37103400	-2.28751100	4.96076000
	H	-5.46366300	-1.93133000	3.66584500
	H	-5.38756700	-0.91732700	1.44417700
	H	-4.77237300	-1.90112300	-1.27089100
	H	-7.06766700	-1.68957000	-2.08956200
	H	-8.27931200	0.47311100	-1.88972700
	H	-7.13260300	2.41709400	-0.84993600
	H	-4.82837900	2.20951800	-0.03975600
	H	-2.70652100	1.65470600	2.16313700
	H	-1.74734400	3.91192500	2.18026600
	H	-0.95439500	4.95726700	0.06828700
	H	-1.17920600	3.70570200	-2.06597500
	H	-2.17927600	1.46055300	-2.08891300
	H	-1.60891500	-4.29989000	-0.51389900
	H	1.04333400	-4.84392400	-0.84119700
	H	2.50656900	-3.89814600	-0.54495900

	O	1.52002700	-1.52948100	-0.72090400
	C	1.86643600	-0.79042700	0.35216000
	C	3.07322500	0.13784100	0.02891400
	C	2.97240400	0.84874200	-1.33896000
	C	4.12236200	1.40729900	-1.91295300
	O	1.38490400	-0.92438600	1.44803800
	C	1.74874600	1.07585800	-1.97473500
	C	1.68058700	1.80601100	-3.16029200
	C	2.83326600	2.33492900	-3.73102300
	C	4.05716500	2.13638000	-3.09571300
	C	4.23033500	-0.89732400	0.11173300
	C	4.65827000	-1.35400100	1.36613000
	C	5.62927900	-2.34538600	1.47406700
	C	6.18542300	-2.91173600	0.32903700
	C	5.74718300	-2.48751500	-0.92216500
	C	4.77168200	-1.49752800	-1.02975500
	C	3.19641300	1.26765900	1.07641500
	C	4.44894100	1.73787900	1.48490300
	C	4.56295800	2.82891800	2.34412500
	C	3.42314500	3.47597600	2.81050600
	C	2.17082100	3.02589100	2.39952000
	C	2.05853900	1.93846100	1.53881900
	H	5.08254800	1.27526500	-1.42986800
	H	0.83225200	0.68715600	-1.55314500
	H	0.71783200	1.95664700	-3.63556200
	H	2.77942100	2.90046400	-4.65442200
	H	4.96503600	2.55298800	-3.51784400
	H	4.22185100	-0.93418100	2.26361500
	H	5.94835400	-2.67609500	2.45619300
	H	6.94590200	-3.68009400	0.41191300
	H	6.15994500	-2.92926300	-1.82244900
	H	4.43054700	-1.19446800	-2.01027200
	H	5.35012000	1.24953000	1.13658500
	H	5.54697300	3.16987900	2.64674700
	H	3.50880400	4.32266000	3.48238900
	H	1.27141600	3.52145700	2.74692900
	H	1.07209400	1.61878100	1.23152400
46	O	1.37395600	-1.57836200	0.53496700
	C	2.16564600	-1.19153000	-0.48118700
	C	0.61354500	-2.82562800	0.43212000
	H	0.16403600	-2.88740300	1.42521100
	C	-0.53431500	-2.68918400	-0.57610800
	H	-0.16685700	-2.26620600	-1.50784200
	C	1.52935900	-4.02993900	0.22324200
	H	0.99546700	-4.94721900	0.47860100
	H	2.38874500	-3.94863800	0.89215400
	C	-1.28471700	-3.99133100	-0.82447500
	H	-2.15509100	-3.79426700	-1.45198500
	O	2.21314700	-1.76514300	-1.54110000
	C	3.06686100	0.03935900	-0.13581400
	C	4.48092100	-0.54044900	0.19020100
	C	5.27653600	0.00366000	1.20533100

	C	6.56617300	-0.47126300	1.44201400
	C	7.09088600	-1.50105300	0.66839000
	C	6.31686900	-2.03912200	-0.35662800
	C	5.03227700	-1.56036500	-0.59869900
	C	2.50273600	0.84492000	1.06297000
	C	2.43018500	0.26849200	2.34196600
	C	1.95101800	0.98514500	3.43161600
	C	1.52443200	2.30449000	3.27666700
	C	1.59161100	2.89074500	2.01986300
	C	2.07951700	2.16977000	0.92715000
	C	3.15724600	0.92650400	-1.40268200
	C	2.03268300	1.13118100	-2.21301800
	C	2.08911200	1.99021300	-3.30695100
	C	3.26848700	2.66541900	-3.61433600
	C	4.39030200	2.47121100	-2.81508800
	C	4.33443400	1.60909700	-1.72067800
	H	4.89722900	0.81106900	1.81613700
	H	7.15838300	-0.02803600	2.23509300
	H	8.09168700	-1.87388400	0.85542100
	H	6.71431000	-2.83240700	-0.98020200
	H	4.45902600	-1.97090300	-1.41846900
	H	2.75966100	-0.75112600	2.48795600
	H	1.91587600	0.51234100	4.40731000
	H	1.14825200	2.86375200	4.12569800
	H	1.26796800	3.91578800	1.87831700
	H	2.13097000	2.65626200	-0.03680300
	H	1.10023900	0.62232200	-2.00318200
	H	1.20450900	2.12726500	-3.91874200
	H	3.31249600	3.33127100	-4.46908900
	H	5.31765200	2.98590900	-3.04160200
	H	5.22093500	1.46997200	-1.11586100
	H	1.89229100	-4.09886200	-0.80062900
	H	-1.63427000	-4.43340600	0.11244900
	H	-0.64777300	-4.71252200	-1.33974400
	O	-1.50311900	-1.76064400	0.00132800
	C	-1.73711500	-0.58981800	-0.61865300
	C	-3.01930800	0.09174100	-0.06036800
	C	-4.11059200	-0.76867900	-0.75493200
	C	-4.38934200	-0.56308000	-2.11312500
	O	-1.10017200	-0.18449300	-1.55818700
	C	-4.73090500	-1.84314400	-0.10883200
	C	-5.64104300	-2.65415400	-0.78431200
	C	-5.93283800	-2.42081500	-2.12533900
	C	-5.29487100	-1.37564200	-2.78954100
	C	-3.07979800	1.58357700	-0.46353600
	C	-4.30551500	2.20574800	-0.72375000
	C	-4.37963200	3.57431500	-0.97484400
	C	-3.22622000	4.35227500	-0.96690800
	C	-2.00165400	3.74724300	-0.69527500
	C	-1.92975900	2.38086800	-0.44065100
	C	-3.09255000	0.08298700	1.48276100
	C	-4.32401800	0.26811600	2.12411900

	C	-4.40629000	0.37410800	3.50906800
	C	-3.25292200	0.31136700	4.28738100
	C	-2.02079100	0.15146100	3.66163900
	C	-1.94067900	0.04090400	2.27468400
	H	-3.88920600	0.23583300	-2.64593500
	H	-4.50108900	-2.05299300	0.92694800
	H	-6.11788000	-3.47363700	-0.25785100
	H	-6.64242300	-3.05105700	-2.64957700
	H	-5.49782500	-1.19132200	-3.83860600
	H	-5.21653300	1.62094800	-0.73655600
	H	-5.34343300	4.02876600	-1.17648800
	H	-3.28037700	5.41660600	-1.16651700
	H	-1.09296500	4.33874700	-0.68098000
	H	-0.96597900	1.94140900	-0.22470500
	H	-5.23163300	0.33663500	1.53741100
	H	-5.37421000	0.51307800	3.97801700
	H	-3.31438500	0.39492200	5.36664700
	H	-1.10943600	0.11677300	4.24735700
	H	-0.96460300	-0.07497200	1.82185900
47	O	-1.40921200	1.67012900	-0.14310000
	C	-2.22306800	1.01984400	0.70625500
	C	-0.66449400	2.81105700	0.37453300
	H	-0.48953000	2.64187000	1.43748400
	C	0.66449300	2.81106000	-0.37452600
	H	0.48953000	2.64187300	-1.43747700
	C	-1.50215600	4.06710100	0.16535400
	H	-1.01612600	4.93749700	0.60984700
	H	-2.47263900	3.94383200	0.64728700
	C	1.50214900	4.06710800	-0.16534600
	H	2.47263300	3.94384400	-0.64727800
	O	-2.30463100	1.29207100	1.87797200
	C	-3.12068300	-0.05000200	0.00400000
	C	-3.48709300	-1.12659300	1.05323800
	C	-4.76456400	-1.69014300	1.09717500
	C	-5.06892400	-2.71369700	1.99453400
	C	-4.09923300	-3.19243500	2.86836400
	C	-2.82011300	-2.64008800	2.83282900
	C	-2.51747300	-1.62111100	1.93589800
	C	-2.41455300	-0.69217300	-1.22090200
	C	-2.06771900	0.08079900	-2.34208600
	C	-1.46176600	-0.48990400	-3.45475600
	C	-1.19025500	-1.85748400	-3.48641600
	C	-1.53267200	-2.63850700	-2.38996400
	C	-2.13880000	-2.06207300	-1.27191700
	C	-4.40950600	0.71945100	-0.42228700
	C	-5.06168300	0.44936400	-1.63006000
	C	-6.25075800	1.09690100	-1.96460400
	C	-6.81351400	2.02917800	-1.09928600
	C	-6.18253300	2.29578500	0.11342300
	C	-5.00012000	1.64362000	0.45145700
	H	-5.53654000	-1.33201700	0.42900100
	H	-6.06989200	-3.13071900	2.00817000

	H	-4.33543900	-3.98413200	3.57065200
	H	-2.05086100	-2.99992000	3.50673200
	H	-1.51417700	-1.21440800	1.93059300
	H	-2.27934600	1.14012800	-2.34756600
	H	-1.20253100	0.13637100	-4.30106200
	H	-0.72453100	-2.30378500	-4.35802500
	H	-1.33524400	-3.70473200	-2.39522800
	H	-2.40134700	-2.69850700	-0.43921300
	H	-4.64812700	-0.27583000	-2.31743500
	H	-6.73463100	0.86596100	-2.90725400
	H	-7.73499000	2.53638300	-1.36217200
	H	-6.61422500	3.00896900	0.80701700
	H	-4.54188200	1.84240100	1.41166600
	H	-1.66581700	4.25998100	-0.89812600
	H	1.66580900	4.25998800	0.89813400
	H	1.01611600	4.93750200	-0.60983900
	O	1.40921800	1.67013600	0.14310600
	C	2.22307400	1.01985300	-0.70625000
	C	3.12068300	-0.05000200	-0.00399900
	C	4.40950800	0.71944400	0.42229300
	C	5.00011800	1.64362500	-0.45144100
	O	2.30462600	1.29207200	-1.87796800
	C	5.06169100	0.44934200	1.63005900
	C	6.25076700	1.09687600	1.96460800
	C	6.81351800	2.02916500	1.09929900
	C	6.18253200	2.29578700	-0.11340300
	C	3.48709100	-1.12658800	-1.05324300
	C	4.76456400	-1.69013200	-1.09718900
	C	5.06892400	-2.71368300	-1.99455200
	C	4.09922900	-3.19242400	-2.86837600
	C	2.82010600	-2.64008300	-2.83283200
	C	2.51746700	-1.62110900	-1.93589700
	C	2.41455100	-0.69218000	1.22089900
	C	2.13876100	-2.06207300	1.27188700
	C	1.53263400	-2.63851600	2.38992900
	C	1.19025600	-1.85750900	3.48640600
	C	1.46180600	-0.48993600	3.45477300
	C	2.06775700	0.08077500	2.34210600
	H	4.54187600	1.84241900	-1.41164600
	H	4.64813800	-0.27586000	2.31742700
	H	6.73464400	0.86592400	2.90725300
	H	7.73499500	2.53636700	1.36218900
	H	6.61422000	3.00898000	-0.80699000
	H	5.53654300	-1.33200500	-0.42901800
	H	6.06989300	-3.13070100	-2.00819500
	H	4.33543400	-3.98411800	-3.57066800
	H	2.05085200	-2.99991800	-3.50673000
	H	1.51417000	-1.21441200	-1.93058500
	H	2.40127800	-2.69849500	0.43916400
	H	1.33517700	-3.70473500	2.39517100
	H	0.72453400	-2.30381700	4.35801100
	H	1.20260200	0.13632600	4.30109800

	H 2.27941500 1.14009800 2.34760900

20 (optimized at the B3LYP-GD3BJ/6-311G(d,p) level)

Conformer no	
1	O -1.36096300 0.18834000 -1.60886900<br>C -2.66826200 -0.10858300 -1.60610400<br>C -0.66812500 0.27630100 -2.87536300<br>H -1.31178600 0.80697600 -3.58123600<br>C 0.56507600 1.12447500 -2.59847500<br>H 1.18442300 1.15688100 -3.49767700<br>C -0.35325200 -1.11798800 -3.39592600<br>H -1.27693800 -1.65279300 -3.61595900<br>H 0.22061200 -1.67363800 -2.65451600<br>C 0.24268100 2.53151600 -2.12261600<br>H 1.16353100 3.08485600 -1.93860100<br>O -3.32118200 -0.28541700 -2.60262700<br>C -3.20153200 -0.07609100 -0.15447000<br>C -2.29218000 -0.85646200 0.81351400<br>C -2.40481500 -0.63668400 2.18956000<br>C -1.65364500 -1.37571500 3.09710500<br>C -0.77678400 -2.35977500 2.64509500<br>C -0.67566500 -2.60585000 1.27993000<br>C -1.43140100 -1.86465400 0.37608300<br>C -4.58600500 -0.73092100 -0.03826300<br>C -4.88648300 -1.89796700 -0.74565000<br>C -6.09382300 -2.55923100 -0.54741800<br>C -7.02122200 -2.07346600 0.37056700<br>C -6.72584500 -0.92066400 1.09112500<br>C -5.51846200 -0.25816200 0.88850100<br>C -3.22386600 1.44530400 0.11183300<br>C -4.24779400 2.22629800 -0.43422900<br>C -4.23247200 3.61119000 -0.29788900<br>C -3.18617600 4.23935700 0.37395200<br>C -2.14813900 3.47094000 0.89327900<br>C -2.16339800 2.08623000 0.75388500<br>H -3.08357800 0.12287200 2.55546100<br>H -1.75558800 -1.18249300 4.15904900<br>H -0.17973700 -2.92862900 3.34807400<br>H 0.00670600 -3.36040100 0.91079200<br>H -1.32548200 -2.07420100 -0.67839300<br>H -4.18024600 -2.29175000 -1.46249500<br>H -6.30892400 -3.45829400 -1.11348300<br>H -7.96192800 -2.58945800 0.52369800<br>H -7.43425400 -0.53248300 1.81402300<br>H -5.30529500 0.63785500 1.45645100<br>H -5.05570800 1.74695200 -0.97225000<br>H -5.03776200 4.19923100 -0.72282400<br>H -3.17552200 5.31794500 0.48047200<br>H -1.31088900 3.94187500 1.39516000<br>H -1.33256600 1.50849200 1.12925600<br>H 0.23205100 -1.05045200 -4.31652700

	H	-0.34806300	2.49806600	-1.20857100
	H	-0.32925000	3.06050500	-2.88934500
	O	1.30485100	0.41885400	-1.56576000
	C	2.61409200	0.68857400	-1.44489200
	C	3.16159300	0.09320800	-0.12463300
	C	4.69502500	0.20026200	-0.05395600
	C	5.33746600	0.48364300	1.15307400
	O	3.23121400	1.37541700	-2.21836400
	C	5.47937200	-0.12497800	-1.16445100
	C	6.86646000	-0.14294400	-1.07464000
	C	7.49843500	0.15116000	0.13134200
	C	6.72696600	0.46061900	1.24622700
	C	2.85697600	-1.40752800	0.01182200
	C	2.55411400	-2.20361800	-1.09338100
	C	2.40149900	-3.58271600	-0.96479200
	C	2.55723500	-4.19295400	0.27535700
	C	2.87587300	-3.41013900	1.38335600
	C	3.02991000	-2.03560500	1.24961500
	C	2.46026100	0.98066600	0.92706300
	C	1.38578500	0.53429800	1.69567600
	C	0.75978700	1.39137500	2.59909000
	C	1.17060200	2.71546200	2.71664800
	C	2.21359600	3.18168700	1.91841000
	C	2.85238700	2.32073600	1.03323000
	H	4.75242700	0.72911000	2.02940700
	H	5.00749500	-0.35779000	-2.10839900
	H	7.45507500	-0.38869800	-1.95106300
	H	8.57995300	0.13660500	0.20020300
	H	7.20299300	0.68622100	2.19364000
	H	2.44143200	-1.75468800	-2.07065300
	H	2.16636000	-4.17784400	-1.83976300
	H	2.44082900	-5.26555800	0.37712400
	H	3.01251500	-3.87154100	2.35457100
	H	3.29760000	-1.44467300	2.11578300
	H	1.02195100	-0.47681900	1.58990200
	H	-0.05952300	1.01523700	3.19880000
	H	0.68024700	3.37942900	3.41930900
	H	2.53503800	4.21448900	1.98779400
	H	3.66536000	2.68742300	0.41860700
11	O	-0.47444700	1.34155300	-1.41485500
	C	-0.02371100	2.59933800	-1.54707800
	C	-0.57734800	0.50073700	-2.59291600
	H	-0.45970900	1.13800300	-3.47114200
	C	0.57734800	-0.50073700	-2.59291600
	H	0.45970900	-1.13800300	-3.47114200
	C	-1.95457700	-0.13760700	-2.55455100
	H	-2.11705400	-0.63961500	-1.60203900
	H	-2.05655200	-0.86945700	-3.35781300
	C	1.95457700	0.13760700	-2.55455100
	H	2.72509200	-0.62616900	-2.67348300
	O	0.29651200	3.09994900	-2.59489400
	C	0.12290200	3.24906400	-0.14892900

	C	1.38587800	2.52469100	0.36602000
	C	1.28773800	1.33585600	1.09037400
	C	2.42079500	0.59845400	1.41531200
	C	3.67794400	1.04126700	1.01348200
	C	3.78870200	2.21997700	0.27972400
	C	2.65076400	2.95165200	-0.05029400
	C	-1.13007600	3.02586600	0.71623300
	C	-2.39410900	2.90562000	0.13552700
	C	-3.53926100	2.82312200	0.92162100
	C	-3.44219400	2.86292300	2.30937000
	C	-2.18760600	2.99402700	2.90018300
	C	-1.04546000	3.08025900	2.10996300
	C	0.29349800	4.77441900	-0.22407800
	C	1.00232400	5.44130600	0.77955000
	C	1.06994700	6.83048400	0.80720100
	C	0.42225200	7.58199800	-0.16898100
	C	-0.29651200	6.92835500	-1.16542400
	C	-0.36505200	5.53841700	-1.19056200
	H	0.31301600	0.97370200	1.37632600
	H	2.32051500	-0.33104400	1.96086700
	H	4.56052600	0.46278900	1.25990200
	H	4.76089600	2.56730600	-0.05065100
	H	2.74263600	3.85048600	-0.64612300
	H	-2.49572700	2.87371100	-0.94136700
	H	-4.50776700	2.72358600	0.44554700
	H	-4.33248300	2.79592800	2.92356400
	H	-2.09556400	3.03399200	3.97944900
	H	-0.07897900	3.19315300	2.58364900
	H	1.51077900	4.87149400	1.54639200
	H	1.62900600	7.32370500	1.59412800
	H	0.47499400	8.66438600	-0.15193200
	H	-0.80844000	7.50055100	-1.93065100
	H	-0.92263700	5.05204700	-1.97710300
	H	-2.72509200	0.62616900	-2.67348300
	H	2.11705400	0.63961500	-1.60203900
	H	2.05655200	0.86945700	-3.35781300
	O	0.47444700	-1.34155300	-1.41485500
	C	0.02371100	-2.59933800	-1.54707800
	C	-0.12290200	-3.24906400	-0.14892900
	C	-1.38587800	-2.52469100	0.36602000
	C	-1.28773800	-1.33585600	1.09037400
	O	-0.29651200	-3.09994900	-2.59489400
	C	-2.65076400	-2.95165200	-0.05029400
	C	-3.78870200	-2.21997700	0.27972400
	C	-3.67794400	-1.04126700	1.01348200
	C	-2.42079500	-0.59845400	1.41531200
	C	1.13007600	-3.02586600	0.71623300
	C	2.39410900	-2.90562000	0.13552700
	C	3.53926100	-2.82312200	0.92162100
	C	3.44219400	-2.86292300	2.30937000
	C	2.18760600	-2.99402700	2.90018300
	C	1.04546000	-3.08025900	2.10996300

	C	-0.29349800	-4.77441900	-0.22407800
	C	0.36505200	-5.53841700	-1.19056200
	C	0.29651200	-6.92835500	-1.16542400
	C	-0.42225200	-7.58199800	-0.16898100
	C	-1.06994700	-6.83048400	0.80720100
	C	-1.00232400	-5.44130600	0.77955000
	H	-0.31301600	-0.97370200	1.37632600
	H	-2.74263600	-3.85048600	-0.64612300
	H	-4.76089600	-2.56730600	-0.05065100
	H	-4.56052600	-0.46278900	1.25990200
	H	-2.32051500	0.33104400	1.96086700
	H	2.49572700	-2.87371100	-0.94136700
	H	4.50776700	-2.72358600	0.44554700
	H	4.33248300	-2.79592800	2.92356400
	H	2.09556400	-3.03399200	3.97944900
	H	0.07897900	-3.19315300	2.58364900
	H	0.92263700	-5.05204700	-1.97710300
	H	0.80844000	-7.50055100	-1.93065100
	H	-0.47499400	-8.66438600	-0.15193200
	H	-1.62900600	-7.32370500	1.59412800
	H	-1.51077900	-4.87149400	1.54639200
14	O	1.26615500	0.62739500	-1.28369900
	C	2.53981700	0.25489400	-1.48601500
	C	0.51760800	1.20902800	-2.38190300
	H	1.20255500	1.35310500	-3.21924200
	C	-0.55511600	0.21223500	-2.81284600
	H	-1.19857300	0.70022300	-3.54641300
	C	-0.03049100	2.53615700	-1.88670500
	H	0.79237100	3.21179800	-1.64858000
	H	-0.63039200	2.39687900	-0.98839700
	C	-0.00474900	-1.09322900	-3.35873900
	H	0.66383500	-0.90072200	-4.19979500
	O	3.08736700	0.27133600	-2.55939800
	C	3.22235300	-0.05960600	-0.13393800
	C	4.37778100	-1.06772800	-0.25487400
	C	5.20923800	-1.24563600	0.85823000
	C	6.21840300	-2.19913800	0.85674000
	C	6.41259500	-3.01016000	-0.26020700
	C	5.58561700	-2.85139000	-1.36524500
	C	4.57562100	-1.88912200	-1.36521300
	C	2.23855500	-0.68399700	0.87169100
	C	1.38965000	-1.71251000	0.45273600
	C	0.55086700	-2.36216900	1.34760200
	C	0.55946200	-2.00675000	2.69528600
	C	1.41731000	-1.00282400	3.13057700
	C	2.24995700	-0.34647400	2.22476700
	C	3.70177200	1.35551300	0.26371300
	C	2.77599800	2.29642000	0.73105300
	C	3.16746000	3.60071000	1.01039400
	C	4.48987100	3.99336300	0.81256700
	C	5.40915300	3.07294300	0.31955000
	C	5.01683400	1.76564800	0.03888400

	H	5.06160700	-0.63143400	1.73747600
	H	6.84970100	-2.31320000	1.73047900
	H	7.19773200	-3.75725400	-0.26482900
	H	5.72265700	-3.47534200	-2.24107700
	H	3.96189200	-1.76996700	-2.24428700
	H	1.38047300	-2.01070800	-0.58768500
	H	-0.12187200	-3.13219600	0.99348200
	H	-0.10282800	-2.50613700	3.39162800
	H	1.44000500	-0.72113700	4.17724200
	H	2.90722000	0.43636300	2.57958000
	H	1.74276300	2.00874800	0.86019500
	H	2.43413100	4.31072300	1.37574400
	H	4.79719100	5.00965100	1.03037800
	H	6.43643900	3.37070400	0.14405400
	H	5.73666200	1.06425600	-0.35990200
	H	-0.65428000	2.99699800	-2.65547400
	H	-0.82046600	-1.73393900	-3.69845000
	H	0.55267800	-1.62636200	-2.58866000
	O	-1.36146000	-0.11283900	-1.65207200
	C	-2.61700600	0.35999400	-1.58014800
	C	-3.21518300	0.07445200	-0.18268000
	C	-2.94798000	-1.36014400	0.30396300
	C	-3.03459400	-1.65897100	1.66634100
	O	-3.15128000	0.99932900	-2.44978200
	C	-2.77342800	-2.41585600	-0.59285100
	C	-2.67315000	-3.72939300	-0.14248100
	C	-2.75026400	-4.01332100	1.21793700
	C	-2.93386000	-2.96898100	2.12066800
	C	-4.74702800	0.21419900	-0.18132600
	C	-5.51088200	-0.21853700	-1.26788600
	C	-6.90079000	-0.20477600	-1.21076500
	C	-7.55485700	0.23223900	-0.06223500
	C	-6.80322800	0.65166500	1.03115300
	C	-5.41297300	0.63966300	0.97092100
	C	-2.51006800	1.16369200	0.65639300
	C	-1.33550400	0.89760600	1.36277500
	C	-0.67717000	1.91695100	2.04632100
	C	-1.15904800	3.22182100	2.00198000
	C	-2.30552100	3.50374300	1.26198700
	C	-2.97349800	2.48292600	0.59340800
	H	-3.18456600	-0.86019800	2.38046200
	H	-2.71809400	-2.22137600	-1.65538600
	H	-2.53682900	-4.53118800	-0.85910600
	H	-2.67271900	-5.03530300	1.56986200
	H	-3.00388600	-3.17329100	3.18287200
	H	-5.02354700	-0.55817300	-2.17044600
	H	-7.47309200	-0.53718900	-2.06921200
	H	-8.63773100	0.24341900	-0.01945700
	H	-7.29671500	0.98922000	1.93534000
	H	-4.84299600	0.96875400	1.82979800
	H	-0.92034500	-0.09877600	1.37378000
	H	0.21771300	1.67913700	2.60676000

	H	-0.64323700	4.01328100	2.53362500
	H	-2.68173000	4.51842600	1.20269500
	H	-3.85717800	2.70955000	0.01107700
17	O	1.34912800	-0.34044900	1.53751400
	C	2.62109800	0.09642700	1.59717200
	C	0.51847400	-0.24284700	2.72513200
	H	1.16474000	0.02377400	3.56282000
	C	-0.47856000	0.90021100	2.53532900
	H	-1.13965000	0.90512700	3.40339300
	C	-0.11245000	-1.60717000	2.94310900
	H	-0.65437500	-1.93505600	2.05598400
	H	0.66315500	-2.34385100	3.16057600
	C	0.16785000	2.25756600	2.32479300
	H	-0.59840900	3.03358400	2.29122100
	O	3.13928000	0.53015400	2.59365900
	C	3.25757900	0.06547600	0.18642600
	C	3.01113300	-1.27454000	-0.52563400
	C	3.05492600	-1.35457800	-1.91982600
	C	2.97053900	-2.58272900	-2.56838900
	C	2.85289400	-3.75928400	-1.83267000
	C	2.82013700	-3.69254500	-0.44258800
	C	2.89930800	-2.46167200	0.20179300
	C	4.78724200	0.21643100	0.23082400
	C	5.55043700	-0.34526000	1.25694400
	C	6.94092700	-0.30188500	1.21329400
	C	7.59537000	0.29374000	0.13929300
	C	6.84411400	0.84377400	-0.89529600
	C	5.45446400	0.80203000	-0.84904400
	C	2.55643800	1.27945200	-0.46096500
	C	3.00919200	2.56897200	-0.16007500
	C	2.30906100	3.68840500	-0.60027500
	C	1.13420200	3.53619400	-1.33267900
	C	0.66753400	2.25765200	-1.62182900
	C	1.37305100	1.13980900	-1.18826700
	H	3.16123700	-0.45110200	-2.50560600
	H	3.00088400	-2.61855700	-3.65114300
	H	2.78850500	-4.71641500	-2.33657400
	H	2.72891300	-4.59890800	0.14444500
	H	2.87623900	-2.43407300	1.28356600
	H	5.06430500	-0.80598600	2.10400000
	H	7.51282100	-0.73625900	2.02523100
	H	8.67818800	0.32717700	0.10783700
	H	7.33784600	1.30654500	-1.74211500
	H	4.88424600	1.23521600	-1.66052800
	H	3.90441400	2.69441700	0.43507800
	H	2.67669900	4.67846000	-0.35627700
	H	0.57663200	4.40414600	-1.66450600
	H	-0.25822300	2.12586500	-2.16435200
	H	0.98308200	0.15593700	-1.40215600
	H	-0.81001500	-1.57021000	3.78136700
	H	0.71617600	2.28309100	1.38482800
	H	0.85904500	2.47873100	3.14033100

	O	-1.29179600	0.64755000	1.35771300
	C	-2.53224100	0.15434600	1.51883200
	C	-3.23014600	-0.05627400	0.14278700
	C	-2.97307700	1.16100700	-0.76213000
	C	-2.85239200	1.01479600	-2.14417900
	O	-3.01242900	-0.09532500	2.59379600
	C	-2.97438900	2.45263900	-0.22760000
	C	-2.84930700	3.56737600	-1.04850700
	C	-2.73681200	3.41156700	-2.42879800
	C	-2.74263200	2.13081200	-2.97258600
	C	-4.74121400	-0.22798300	0.38781900
	C	-5.19913100	-1.24089000	1.24203800
	C	-6.55733800	-1.43722000	1.45262600
	C	-7.49418000	-0.62746300	0.81141300
	C	-7.05321700	0.37526100	-0.04193700
	C	-5.68777900	0.57235000	-0.25200900
	C	-2.62359600	-1.32721100	-0.51045100
	C	-3.43968700	-2.28278600	-1.12198200
	C	-2.88467700	-3.37731700	-1.78381500
	C	-1.50467400	-3.53795300	-1.84594900
	C	-0.68134900	-2.58767900	-1.24765300
	C	-1.23522900	-1.49242900	-0.59394000
	H	-2.85140300	0.02470800	-2.57965000
	H	-3.08725500	2.59287500	0.84072800
	H	-2.84559400	4.55859300	-0.61028200
	H	-2.64448500	4.27921500	-3.07159700
	H	-2.65607200	1.99398500	-4.04441300
	H	-4.48723500	-1.88045200	1.74300400
	H	-6.88556100	-2.22564600	2.12022500
	H	-8.55443900	-0.78108500	0.97629400
	H	-7.76734400	1.01182200	-0.55168300
	H	-5.36990400	1.35720300	-0.92338300
	H	-4.51433500	-2.17351600	-1.09251000
	H	-3.54052300	-4.10298200	-2.25141600
	H	-1.07285400	-4.39086600	-2.35690900
	H	0.39444700	-2.69279400	-1.28679800
	H	-0.57872400	-0.75962000	-0.15382300
20	O	1.24172500	0.53878000	-1.34661400
	C	2.50645300	0.10981800	-1.50572500
	C	0.49486100	0.96461900	-2.51818700
	H	1.18759100	1.00955300	-3.35971500
	C	-0.56488900	-0.08680800	-2.84025200
	H	-1.21033800	0.31728200	-3.62175200
	C	-0.07478400	2.33787600	-2.20436000
	H	0.73537900	3.05394500	-2.05837100
	H	-0.67988400	2.31351900	-1.29919700
	C	-0.00588800	-1.43684300	-3.25100800
	H	0.54983800	-1.89082600	-2.43161000
	O	3.01929300	-0.05314300	-2.58237100
	C	3.20363000	-0.08242600	-0.12467700
	C	2.20948700	-0.67881400	0.88540100
	C	2.19244500	-0.26886700	2.21786900

	C	1.34474300	-0.88066700	3.14116900
	C	0.49878500	-1.90941100	2.74241000
	C	0.51352600	-2.33291800	1.41451300
	C	1.36788700	-1.72742300	0.50280500
	C	3.63989400	1.33540400	0.33419000
	C	2.68843500	2.36011400	0.43166000
	C	3.04202200	3.62283000	0.89158300
	C	4.35453900	3.89062000	1.27514000
	C	5.30199900	2.87632700	1.20069600
	C	4.94837500	1.60977700	0.73554100
	C	4.41833800	-1.01682000	-0.28816000
	C	5.39189300	-0.75950400	-1.26428300
	C	6.51126900	-1.57339700	-1.38775500
	C	6.69161600	-2.66102700	-0.53519800
	C	5.74052200	-2.91936300	0.44361000
	C	4.61582100	-2.10387700	0.56586300
	H	2.84547100	0.53036600	2.54194200
	H	1.34569300	-0.54430000	4.17169200
	H	-0.17578800	-2.37401600	3.45055200
	H	-0.15371000	-3.12021400	1.08904500
	H	1.38308400	-2.08043700	-0.51992500
	H	1.66080600	2.16069100	0.17145200
	H	2.28391200	4.39535900	0.95513000
	H	4.63179700	4.87505800	1.63391200
	H	6.32488800	3.06257800	1.50731900
	H	5.69923800	0.83400700	0.69625900
	H	5.27178400	0.08087100	-1.93042900
	H	7.24656600	-1.35439800	-2.15361000
	H	7.56535000	-3.29501900	-0.63325200
	H	5.86606200	-3.75728000	1.11975900
	H	3.89326400	-2.32350400	1.33843800
	H	-0.70115600	2.68182600	-3.02978800
	H	0.66784500	-1.32553400	-4.10225800
	H	-0.81842100	-2.11013500	-3.53018200
	O	-1.37344600	-0.29685100	-1.65472400
	C	-2.62193000	0.20017900	-1.62520300
	C	-3.21625800	0.06031800	-0.20456300
	C	-2.47982600	1.20890200	0.52088800
	C	-1.28982200	0.98916700	1.21807600
	O	-3.14980900	0.76121100	-2.55103900
	C	-2.92585400	2.52426800	0.34754300
	C	-2.22353900	3.59147000	0.89847600
	C	-1.05816500	3.35984600	1.62614800
	C	-0.59402200	2.05675600	1.77969900
	C	-2.97325900	-1.32746100	0.41119600
	C	-3.04905100	-1.49489200	1.79612600
	C	-2.97212600	-2.75880100	2.37045200
	C	-2.82242400	-3.88717100	1.56827400
	C	-2.75617300	-3.73387900	0.18645400
	C	-2.83345500	-2.46639300	-0.38426500
	C	-4.74467300	0.23221500	-0.20532700
	C	-5.39067900	0.76887600	0.91163600

	C	-6.77960100	0.81736400	0.98094000
	C	-7.55046300	0.32302400	-0.06692400
	C	-6.91682500	-0.22482400	-1.17857200
	C	-5.52798000	-0.27442400	-1.24497700
	H	-0.89002900	-0.00926200	1.31214600
	H	-3.82160600	2.71084900	-0.23035200
	H	-2.58647600	4.60258700	0.75478700
	H	-0.51202600	4.18811200	2.06307400
	H	0.32032500	1.86102000	2.32392100
	H	-3.17302100	-0.62973300	2.43397000
	H	-3.03464800	-2.86102600	3.44778500
	H	-2.76349900	-4.87324200	2.01382600
	H	-2.64625500	-4.60209400	-0.45317500
	H	-2.78646600	-2.37418100	-1.46105800
	H	-4.80589600	1.15736400	1.73514700
	H	-7.25710900	1.24180600	1.85665000
	H	-8.63239500	0.36197100	-0.01704500
	H	-7.50412600	-0.61644900	-2.00116700
	H	-5.05727900	-0.69965100	-2.11946700
21	O	-1.34202400	0.20709600	1.62767500
	C	-2.66122300	0.45238500	1.57371000
	C	-0.61158200	0.69361900	2.78234100
	H	-1.21453300	0.49549600	3.67174500
	C	0.65144800	-0.15027300	2.85967600
	H	1.28204200	0.23250000	3.66503700
	C	-0.35942900	2.18699100	2.64960100
	H	0.19539500	2.54595700	3.52020600
	H	-1.30750300	2.72208400	2.60217600
	C	0.37522600	-1.63278600	3.06290300
	H	-0.22056400	-1.77961500	3.96754000
	O	-3.28501800	0.95807600	2.47127900
	C	-3.20429200	0.09577000	0.16911200
	C	-4.74019400	0.16974900	0.11380700
	C	-5.38746100	0.60368400	-1.04564900
	C	-6.77498100	0.55726200	-1.15092400
	C	-7.54112100	0.07125400	-0.09652700
	C	-6.90457000	-0.37541200	1.05866100
	C	-5.51828000	-0.33308200	1.16024100
	C	-2.86150700	-1.34813300	-0.23199800
	C	-2.59132200	-2.32933600	0.72286800
	C	-2.42352800	-3.66204900	0.35410500
	C	-2.53041000	-4.03836500	-0.98072400
	C	-2.80844800	-3.06842000	-1.94124100
	C	-2.97511600	-1.73989600	-1.56896900
	C	-2.52212500	1.17494100	-0.69855400
	C	-1.37246200	0.91416800	-1.44609300
	C	-0.74008600	1.93933400	-2.14497900
	C	-1.22790500	3.24126200	-2.08547000
	C	-2.35149800	3.51678300	-1.30959500
	C	-2.99006000	2.49172500	-0.61963300
	H	-4.80628900	0.98591500	-1.87426700
	H	-7.25407300	0.90263400	-2.05992600

	H	-8.62162700	0.03772200	-0.17416400	
	H	-7.48846600	-0.76070200	1.88665700	
	H	-5.04386800	-0.68545100	2.06452200	
	H	-2.52063500	-2.06358000	1.76932900	
	H	-2.21574400	-4.40515700	1.11543500	
	H	-2.40567100	-5.07545000	-1.26957100	
	H	-2.90412800	-3.34753900	-2.98427100	
	H	-3.20675200	-1.00088100	-2.32474800	
	H	-0.95317600	-0.08143900	-1.47559200	
	H	0.14884700	1.71565900	-2.71970900	
	H	-0.73016300	4.03519400	-2.63015000	
	H	-2.73303800	4.52894300	-1.23988300	
	H	-3.85941500	2.71194100	-0.01269600	
	H	0.22119800	2.40433300	1.75506700	
	H	1.31193300	-2.17721800	3.17963200	
	H	-0.17421800	-2.02983900	2.20957600	
	O	1.35184800	0.04849400	1.60395500	
	C	2.66436000	-0.23821700	1.57349700	
	C	3.23650900	-0.09613600	0.12918400	
	C	2.27563800	-0.78062700	-0.85950900	
	C	2.12534700	-0.30221800	-2.16135800	
	O	3.28523700	-0.55960800	2.55345200	
	C	1.60577400	-1.95286700	-0.50145800	
	C	0.77427300	-2.60675700	-1.40222700	
	C	0.61815900	-2.11204500	-2.69484800	
	C	1.30768600	-0.96518700	-3.07483900	
	C	4.62227500	-0.76503000	0.06791700	
	C	5.61249800	-0.44667100	1.00864300	
	C	6.87662000	-1.01899800	0.93745600	
	C	7.18633900	-1.92189000	-0.07797800	
	C	6.21648100	-2.24127100	-1.01951300	
	C	4.94729900	-1.66801800	-0.94627000	
	C	3.31416200	1.42332500	-0.17860700	
	C	2.13119300	2.17320000	-0.22618000	
	C	2.15385300	3.52913700	-0.52611200	
	C	3.36223000	4.16877800	-0.79702700	
	C	4.53923000	3.43029500	-0.77669400	
	C	4.51596000	2.06860900	-0.47298800	
	H	2.65022500	0.59404500	-2.46493400	
	H	1.72897300	-2.36241600	0.49308100	
	H	0.23605900	-3.49275100	-1.09198800	
	H	-0.03997700	-2.61472500	-3.39282000	
	H	1.20323500	-0.57517900	-4.08091900	
	H	5.39196700	0.24928300	1.80368900	
	H	7.62183000	-0.75829200	1.68027900	
	H	8.17239500	-2.36886900	-0.13152600	
	H	6.43898100	-2.94054000	-1.81735600	
	H	4.21026400	-1.93448300	-1.68995300	
	H	1.18519400	1.68308800	-0.05464400	
	H	1.22027500	4.07817700	-0.55974100	
	H	3.38247100	5.22680200	-1.03164900	
	H	5.48624600	3.90723500	-1.00202000	

	H	5.44222600	1.51312300	-0.47888900
22	O	-1.35040500	-0.50033100	-1.39655300
	C	-2.62534000	-0.88801900	-1.24653500
	C	-0.56472600	-1.08297700	-2.46734800
	H	-1.19402700	-1.12140900	-3.36014700
	C	0.56811400	-0.09240300	-2.70242200
	H	1.22357000	-0.48244300	-3.48447800
	C	-0.09006200	-2.47699100	-2.09007200
	H	-0.94575000	-3.13766000	-1.95574400
	H	0.49644000	-2.44693900	-1.17315900
	C	0.10194900	1.31152100	-3.05249000
	H	0.96338100	1.95493800	-3.23129500
	O	-3.15288900	-1.70640600	-1.95412500
	C	-3.29682000	-0.08873200	-0.08951000
	C	-2.66111100	-0.48616700	1.26930600
	C	-1.27250500	-0.44852700	1.46254000
	C	-0.72041100	-0.69889100	2.71400000
	C	-1.53480700	-1.00563300	3.80044000
	C	-2.91234300	-1.04814200	3.62145900
	C	-3.46895800	-0.78369100	2.37138800
	C	-4.80607300	-0.38031300	-0.08037900
	C	-5.26910300	-1.70232500	-0.08986300
	C	-6.62743000	-1.98226200	-0.01086700
	C	-7.55586000	-0.94721500	0.08898400
	C	-7.10746400	0.36751500	0.11407600
	C	-5.74354000	0.64710700	0.03082700
	C	-3.03740400	1.39953000	-0.40261900
	C	-2.56862600	2.30147300	0.54941300
	C	-2.37051100	3.64277500	0.21785600
	C	-2.63756300	4.09883300	-1.06796600
	C	-3.11789300	3.20426700	-2.02395300
	C	-3.31813100	1.87112300	-1.69041200
	H	-0.61785500	-0.19328400	0.64481300
	H	0.35085400	-0.61921000	2.84490100
	H	-1.10004900	-1.19757200	4.77469100
	H	-3.56576500	-1.27710500	4.45561100
	H	-4.54344700	-0.80176100	2.26435800
	H	-4.56302000	-2.51681400	-0.15645900
	H	-6.96175700	-3.01328800	-0.02626600
	H	-8.61562700	-1.16660200	0.14956500
	H	-7.81500000	1.18433800	0.19910600
	H	-5.41451300	1.67649000	0.05916300
	H	-2.35058300	1.96214400	1.55224200
	H	-2.00918500	4.33115600	0.97359100
	H	-2.47859900	5.13968700	-1.32430300
	H	-3.33515500	3.54577900	-3.02941200
	H	-3.70348200	1.18469200	-2.43662900
	H	0.53769700	-2.88216700	-2.88779400
	H	-0.50817500	1.72430300	-2.24966300
	H	-0.50280600	1.28625300	-3.96267800
	O	1.32072900	-0.07496500	-1.46178800
	C	2.62148400	0.25213400	-1.51268000

	C	3.28352100	-0.00500800	-0.13701900
	C	2.58286600	0.74918500	1.00898500
	C	3.03978000	0.53715200	2.31643700
	O	3.18526600	0.60262200	-2.51677800
	C	1.58484700	1.70343700	0.80798500
	C	1.04519800	2.41073900	1.88165900
	C	1.49229500	2.17735200	3.17540900
	C	2.49757600	1.23491900	3.38822000
	C	4.74027100	0.48662900	-0.14190100
	C	5.02841900	1.78172200	-0.58401500
	C	6.31989400	2.28724000	-0.51090800
	C	7.34999600	1.51182600	0.01910400
	C	7.07116500	0.22926400	0.47588700
	C	5.77517000	-0.27881700	0.39577200
	C	3.17411900	-1.54153000	-0.02076800
	C	3.85759700	-2.32836600	-0.95673200
	C	3.72400600	-3.71189700	-0.95699200
	C	2.89930000	-4.33691600	-0.02288200
	C	2.20625100	-3.56237900	0.90083300
	C	2.33876500	-2.17464000	0.89695200
	H	3.83642200	-0.17465600	2.49262200
	H	1.20162200	1.89935700	-0.18222400
	H	0.26336700	3.13597800	1.69311400
	H	1.06543000	2.72049100	4.01012300
	H	2.86573600	1.04732300	4.39033600
	H	4.23837700	2.39860400	-0.99034400
	H	6.52272400	3.29082100	-0.86666600
	H	8.35754500	1.90699200	0.07683300
	H	7.86006400	-0.38372600	0.89654400
	H	5.57577100	-1.27965000	0.75469400
	H	4.49314500	-1.84822900	-1.69115100
	H	4.26273400	-4.30213000	-1.68926500
	H	2.79339600	-5.41541100	-0.02234500
	H	1.54705100	-4.03090900	1.62233900
	H	1.77245500	-1.58915600	1.60411400
25	O	1.41292900	1.73284600	0.31164600
	C	2.04611800	1.03641700	-0.65189600
	C	0.66194800	2.91242200	-0.08024200
	H	0.47654800	2.85290700	-1.15248600
	C	-0.65955700	2.83450700	0.66969700
	H	-0.47791800	2.61384800	1.72262700
	C	1.50102700	4.13684000	0.25046800
	H	1.01740200	5.04708900	-0.10712600
	H	2.47287600	4.05472900	-0.23805200
	C	-1.52943300	4.07339300	0.51798200
	H	-2.49236300	3.90167100	0.99742300
	O	1.96366500	1.31070600	-1.82151900
	C	2.95154300	-0.07077800	-0.03314000
	C	3.12694600	-1.25901200	-1.00444700
	C	3.13327400	-2.56857200	-0.51569900
	C	3.35139000	-3.65345000	-1.36165400
	C	3.56528000	-3.45279400	-2.72045500

	C	3.57615800	-2.15198000	-3.21834200	
	C	3.37199400	-1.06756500	-2.37088500	
	C	2.33671000	-0.57498000	1.28232400	
	C	1.00208100	-0.99263400	1.27989300	
	C	0.42951100	-1.55184800	2.41325100	
	C	1.18207900	-1.70110900	3.57842500	
	C	2.51051700	-1.29299300	3.58900300	
	C	3.08641800	-0.73752900	2.44505200	
	C	4.30761900	0.63979500	0.21528700	
	C	4.35191000	1.79323000	1.01060500	
	C	5.55511600	2.44101100	1.26388100	
	C	6.74484400	1.94606400	0.73336700	
	C	6.71384000	0.79495700	-0.04472300	
	C	5.50596700	0.14732000	-0.30230000	
	H	2.96136500	-2.75311200	0.53442100	
	H	3.34679400	-4.65677100	-0.95135300	
	H	3.72841200	-4.29563600	-3.38233400	
	H	3.75250000	-1.97482700	-4.27330000	
	H	3.39454600	-0.06764800	-2.77294300	
	H	0.41298600	-0.89448600	0.37954400	
	H	-0.60070700	-1.88301700	2.38050700	
	H	0.73574000	-2.13607500	4.46526600	
	H	3.10955700	-1.41001500	4.48489400	
	H	4.12625200	-0.44072600	2.46426100	
	H	3.43919700	2.16978000	1.44966600	
	H	5.56317300	3.33086400	1.88308200	
	H	7.68391800	2.44998800	0.93028800	
	H	7.63147400	0.39142700	-0.45715300	
	H	5.50481700	-0.74823700	-0.90643400	
	H	1.66058000	4.22407100	1.32832200	
	H	-1.69869700	4.30116700	-0.53717600	
	H	-1.06307200	4.93533900	0.99764600	
	O	-1.34745000	1.69833900	0.08552800	
	C	-2.27487100	1.07852400	0.83085500	
	C	-3.01023600	-0.02584500	0.01589300	
	C	-3.91193900	0.75272200	-0.97161300	
	C	-4.07249700	0.36460600	-2.30140800	
	O	-2.53978400	1.41119200	1.95824300	
	C	-4.65920900	1.83591400	-0.49602500	
	C	-5.52107800	2.53165800	-1.33582300	
	C	-5.66408200	2.14661400	-2.66735600	
	C	-4.94281500	1.05676100	-3.14264800	
	C	-3.89833800	-0.86785200	0.94816400	
	C	-3.47346500	-1.23884400	2.22881000	
	C	-4.24178600	-2.08827600	3.01807900	
	C	-5.45192200	-2.58872500	2.54419500	
	C	-5.88405300	-2.22761000	1.27280800	
	C	-5.11425900	-1.37491300	0.48496200	
	C	-1.98568500	-0.92691100	-0.71425600	
	C	-1.86308400	-2.28421800	-0.40798400	
	C	-0.91191600	-3.08165700	-1.04581800	
	C	-0.07088400	-2.54238100	-2.00989900	

	C	-0.19890300	-1.19556400	-2.34299100
	C	-1.14460600	-0.40158200	-1.70802700
	H	-3.52305400	-0.48384600	-2.68484600
	H	-4.57844600	2.12330000	0.54535600
	H	-6.08650800	3.37085300	-0.94726700
	H	-6.33594000	2.68692400	-3.32400100
	H	-5.05423600	0.73768200	-4.17260400
	H	-2.54457500	-0.85427100	2.62093000
	H	-3.89241100	-2.35550400	4.00889700
	H	-6.05121000	-3.24865500	3.16073900
	H	-6.82427400	-2.60583600	0.88815600
	H	-5.46771100	-1.10583500	-0.50117600
	H	-2.50387600	-2.73099900	0.33795100
	H	-0.82839800	-4.12728200	-0.77291600
	H	0.68589000	-3.15104900	-2.48723800
	H	0.45848200	-0.75146300	-3.07864900
	H	-1.22003100	0.64018500	-1.97633200
43	O	-1.45794200	-2.02887300	-0.06199500
	C	-1.85789800	-0.98273100	-0.80027800
	C	-0.67410200	-3.07716900	-0.68687500
	H	-0.57528600	-2.84447600	-1.74762000
	C	0.70196000	-3.05996700	-0.02913800
	H	0.57578100	-3.03089600	1.05349800
	C	-1.43634000	-4.37519700	-0.47254900
	H	-0.93965700	-5.20717700	-0.97405100
	H	-2.44227000	-4.28065400	-0.88412900
	C	1.60946000	-4.20169000	-0.45968400
	H	1.67287800	-4.25945300	-1.54909000
	O	-1.58131800	-0.84992600	-1.96716300
	C	-2.74872600	0.01736300	-0.00582200
	C	-3.11524600	-0.52540100	1.38962700
	C	-2.10071000	-0.78446100	2.32259900
	C	-2.40598500	-1.23794600	3.59814500
	C	-3.73387700	-1.43732200	3.97800100
	C	-4.74682800	-1.17218200	3.06632400
	C	-4.43920900	-0.71708100	1.78251300
	C	-4.01620600	0.21881100	-0.86131800
	C	-4.63406800	-0.88517000	-1.45768700
	C	-5.81443900	-0.74328300	-2.17663400
	C	-6.40553400	0.51203300	-2.30842500
	C	-5.80246700	1.61508600	-1.71456400
	C	-4.61546600	1.46962300	-0.99783400
	C	-1.98868500	1.36373900	0.11828100
	C	-2.08833500	2.13555900	1.28060200
	C	-1.49408400	3.39363800	1.35492900
	C	-0.77763000	3.89905800	0.27475700
	C	-0.67795000	3.14148500	-0.88816300
	C	-1.28981100	1.89483400	-0.97060500
	H	-1.06468100	-0.63381900	2.05459900
	H	-1.60157000	-1.43208100	4.29849800
	H	-3.97162400	-1.78935100	4.97543000
	H	-5.78466000	-1.31243700	3.34657000

	H	-5.24505900	-0.50804800	1.09344200	
	H	-4.19476200	-1.87034100	-1.35159500	
	H	-6.27306600	-1.61241100	-2.63407800	
	H	-7.32504200	0.62663100	-2.87053800	
	H	-6.25080200	2.59741600	-1.80962800	
	H	-4.15668900	2.33756600	-0.54464900	
	H	-2.63831400	1.76081700	2.13179000	
	H	-1.59109200	3.97332200	2.26591900	
	H	-0.29567800	4.86731500	0.34094300	
	H	-0.11461300	3.50841400	-1.73665100	
	H	-1.22808900	1.33378600	-1.88914600	
	H	-1.52321900	-4.60137000	0.59271400	
	H	1.24707500	-5.15624900	-0.07477800	
	H	2.61372600	-4.02841400	-0.07082500	
	O	1.37852200	-1.83960500	-0.43299200	
	C	1.60955700	-0.88743400	0.49168400	
	C	2.67692600	0.13260000	0.00466100	
	C	2.28360800	0.89326900	-1.28306700	
	C	2.85962600	2.15019600	-1.51146600	
	O	1.12725600	-0.90410500	1.59428400	
	C	1.43847000	0.37254700	-2.26472400	
	C	1.16573500	1.08964800	-3.42815000	
	C	1.74655200	2.33339500	-3.64579200	
	C	2.60061000	2.85924200	-2.67898400	
	C	3.93039400	-0.73943200	-0.22876800	
	C	4.35740800	-1.59281600	0.79719100	
	C	5.46804300	-2.41033600	0.62896600	
	C	6.17725700	-2.38785300	-0.57187000	
	C	5.76125500	-1.54225600	-1.59361000	
	C	4.64295600	-0.72517400	-1.42504200	
	C	2.89347800	1.15767900	1.13070100	
	C	4.15393500	1.44363800	1.65139700	
	C	4.31528500	2.43666700	2.61918500	
	C	3.21675100	3.15454800	3.07527300	
	C	1.95363500	2.87938100	2.55123600	
	C	1.79510100	1.89449600	1.58692000	
	H	3.51520500	2.58202700	-0.76800600	
	H	0.97069500	-0.58712000	-2.13101500	
	H	0.48568300	0.66567300	-4.15769800	
	H	1.53501600	2.88847400	-4.55250500	
	H	3.06289400	3.82853600	-2.82746400	
	H	3.81747800	-1.60677600	1.73739500	
	H	5.78270100	-3.06217800	1.43597500	
	H	7.04450500	-3.02378000	-0.70614400	
	H	6.30346100	-1.51564300	-2.53188400	
	H	4.32579700	-0.07932500	-2.23184100	
	H	5.02091600	0.90123400	1.29994700	
	H	5.30470400	2.64529700	3.01002600	
	H	3.34044100	3.92433100	3.82847000	
	H	1.08663000	3.43359100	2.88957900	
	H	0.81199500	1.70582800	1.17982600	

20 (optimized at the M06-2X/6-311G(d,p) level)

Conformer no	
1	O -1.36986100 0.23909800 -1.60990300<br>C -2.66410300 -0.09487500 -1.60723800<br>C -0.67612900 0.27552800 -2.86553300<br>H -1.29759800 0.80614500 -3.59283200<br>C 0.58321800 1.08482500 -2.60463200<br>H 1.18284700 1.11062400 -3.51879900<br>C -0.38562500 -1.13910700 -3.33961000<br>H -1.31756500 -1.66498300 -3.54681600<br>H 0.17535800 -1.67362600 -2.57078400<br>C 0.30937300 2.49017300 -2.09901400<br>H 1.24681800 3.03505600 -1.98423600<br>O -3.30336300 -0.30043200 -2.60091900<br>C -3.19801000 -0.07262900 -0.16163700<br>C -2.24421600 -0.82130000 0.78422600<br>C -2.26232500 -0.55636100 2.15372500<br>C -1.47896500 -1.29543800 3.03364000<br>C -0.66429500 -2.31726900 2.55742200<br>C -0.65352700 -2.60245100 1.19672000<br>C -1.44121400 -1.86434000 0.32205700<br>C -4.55457700 -0.77481200 -0.02338200<br>C -4.86803700 -1.91154300 -0.76912800<br>C -6.04937500 -2.60700000 -0.53726000<br>C -6.93280800 -2.18732900 0.44937500<br>C -6.62156800 -1.06624300 1.20928000<br>C -5.44249400 -0.37027200 0.97549200<br>C -3.27417000 1.44445500 0.10215400<br>C -4.37438700 2.17793800 -0.34615700<br>C -4.40725300 3.56037200 -0.20097100<br>C -3.33578900 4.23135400 0.37851000<br>C -2.22161500 3.51106800 0.79393600<br>C -2.18915400 2.12925500 0.64671900<br>H -2.89570400 0.23630700 2.53699100<br>H -1.50824200 -1.07140800 4.09394600<br>H -0.04294800 -2.88811600 3.23763000<br>H -0.02016400 -3.39134700 0.80980200<br>H -1.41840400 -2.11051700 -0.73344600<br>H -4.19487400 -2.25605700 -1.54245000<br>H -6.27788000 -3.48280400 -1.13311700<br>H -7.85335800 -2.73014700 0.62716900<br>H -7.29603900 -0.73015600 1.98787600<br>H -5.21441800 0.50214300 1.57690200<br>H -5.20658700 1.66366800 -0.81303300<br>H -5.27236900 4.11346900 -0.54712900<br>H -3.36470900 5.30842900 0.49301400<br>H -1.36331500 4.01755100 1.22169400<br>H -1.30154700 1.58095600 0.93515200<br>H 0.20690300 -1.11270800 -4.25690800<br>H -0.20301500 2.44346000 -1.13715400<br>H -0.32355100 3.02883900 -2.80769900<br>O 1.31300500 0.35233200 -1.59993500

	C	2.60850100	0.64919800	-1.45274900
	C	3.14192000	0.08812600	-0.11996300
	C	4.67378300	0.17302300	-0.04881300
	C	5.31626900	0.44689000	1.15857500
	O	3.22254200	1.34565500	-2.21218500
	C	5.45324700	-0.16084100	-1.15752700
	C	6.83837500	-0.19575300	-1.06545200
	C	7.47058000	0.09033300	0.13943900
	C	6.70323000	0.40717800	1.25267000
	C	2.80848400	-1.39741500	0.06501600
	C	2.55244600	-2.22805300	-1.02477500
	C	2.41682300	-3.60346900	-0.85916500
	C	2.54294700	-4.17254700	0.40182300
	C	2.80083100	-3.35272100	1.49655500
	C	2.93468200	-1.98111900	1.32795100
	C	2.45333300	1.03326100	0.88517800
	C	1.32875800	0.66280700	1.62002300
	C	0.72105000	1.57783700	2.47762800
	C	1.19656100	2.87951100	2.57499900
	C	2.28657100	3.26998100	1.80235100
	C	2.90949400	2.35312400	0.96684400
	H	4.73291100	0.69983800	2.03620800
	H	4.97803200	-0.38686000	-2.10331400
	H	7.42514500	-0.44877700	-1.94061300
	H	8.55138300	0.06344100	0.20962000
	H	7.18152100	0.62522800	2.20021500
	H	2.47234400	-1.80869100	-2.02113300
	H	2.22286900	-4.22915600	-1.72267600
	H	2.44663300	-5.24407300	0.53129300
	H	2.91136800	-3.78374200	2.48494600
	H	3.15954700	-1.35571800	2.18504100
	H	0.91453200	-0.33470200	1.52507300
	H	-0.13907200	1.26496300	3.05878700
	H	0.71975600	3.58610900	3.24456000
	H	2.65796700	4.28646700	1.85536300
	H	3.76179000	2.65758200	0.36857600
11	O	-0.48657700	1.33623500	-1.39097000
	C	-0.01585500	2.58077300	-1.52883500
	C	-0.58599900	0.48920900	-2.55064600
	H	-0.49776200	1.11267600	-3.44342000
	C	0.58599900	-0.48920900	-2.55064600
	H	0.49776200	-1.11267600	-3.44342000
	C	-1.94253200	-0.18596000	-2.47515400
	H	-2.07439100	-0.64694900	-1.49444300
	H	-2.03140900	-0.95609700	-3.24347600
	C	1.94253200	0.18596000	-2.47515400
	H	2.73583600	-0.55010600	-2.61450600
	O	0.33017700	3.06275600	-2.57106400
	C	0.11996600	3.24729400	-0.14207500
	C	1.36051400	2.50524200	0.39366500
	C	1.21569700	1.31872400	1.11067200
	C	2.31812500	0.53698300	1.43278400

	C	3.59110700	0.94085300	1.04378000
	C	3.74816700	2.12224100	0.32657200
	C	2.63980300	2.89424900	-0.00857200
	C	-1.14022000	3.04462100	0.71277500
	C	-2.39653000	2.87863200	0.13140000
	C	-3.54216100	2.80684200	0.91734000
	C	-3.45083900	2.90221500	2.30043900
	C	-2.20301800	3.08043000	2.89046700
	C	-1.06142300	3.15632100	2.10226400
	C	0.31178900	4.76430000	-0.24788400
	C	1.05839900	5.44502700	0.71477300
	C	1.14022000	6.83255000	0.70410300
	C	0.47009000	7.56473900	-0.26803800
	C	-0.28792700	6.89648600	-1.22212200
	C	-0.37085400	5.50959600	-1.20979200
	H	0.22262700	0.99602400	1.38943200
	H	2.18346500	-0.39574100	1.97151200
	H	4.45120800	0.32935800	1.29161500
	H	4.73432400	2.43965800	0.00899000
	H	2.76584200	3.79348300	-0.60080500
	H	-2.49304900	2.80217800	-0.94557300
	H	-4.50761900	2.67489200	0.44279600
	H	-4.34258800	2.84675600	2.91348100
	H	-2.11717500	3.16609300	3.96734100
	H	-0.09503600	3.30232300	2.57225600
	H	1.58612800	4.88812600	1.48045600
	H	1.72812500	7.33977400	1.45988200
	H	0.53481700	8.64612200	-0.27999700
	H	-0.81940000	7.45505700	-1.98345500
	H	-0.96307000	5.00772600	-1.96330800
	H	-2.73583600	0.55010600	-2.61450600
	H	2.07439100	0.64694900	-1.49444300
	H	2.03140900	0.95609700	-3.24347600
	O	0.48657700	-1.33623500	-1.39097000
	C	0.01585500	-2.58077300	-1.52883500
	C	-0.11996600	-3.24729400	-0.14207500
	C	-1.36051400	-2.50524200	0.39366500
	C	-1.21569700	-1.31872400	1.11067200
	O	-0.33017700	-3.06275600	-2.57106400
	C	-2.63980300	-2.89424900	-0.00857200
	C	-3.74816700	-2.12224100	0.32657200
	C	-3.59110700	-0.94085300	1.04378000
	C	-2.31812500	-0.53698300	1.43278400
	C	1.14022000	-3.04462100	0.71277500
	C	2.39653000	-2.87863200	0.13140000
	C	3.54216100	-2.80684200	0.91734000
	C	3.45083900	-2.90221500	2.30043900
	C	2.20301800	-3.08043000	2.89046700
	C	1.06142300	-3.15632100	2.10226400
	C	-0.31178900	-4.76430000	-0.24788400
	C	0.37085400	-5.50959600	-1.20979200
	C	0.28792700	-6.89648600	-1.22212200

	C	-0.47009000	-7.56473900	-0.26803800
	C	-1.14022000	-6.83255000	0.70410300
	C	-1.05839900	-5.44502700	0.71477300
	H	-0.22262700	-0.99602400	1.38943200
	H	-2.76584200	-3.79348300	-0.60080500
	H	-4.73432400	-2.43965800	0.00899000
	H	-4.45120800	-0.32935800	1.29161500
	H	-2.18346500	0.39574100	1.97151200
	H	2.49304900	-2.80217800	-0.94557300
	H	4.50761900	-2.67489200	0.44279600
	H	4.34258800	-2.84675600	2.91348100
	H	2.11717500	-3.16609300	3.96734100
	H	0.09503600	-3.30232300	2.57225600
	H	0.96307000	-5.00772600	-1.96330800
	H	0.81940000	-7.45505700	-1.98345500
	H	-0.53481700	-8.64612200	-0.27999700
	H	-1.72812500	-7.33977400	1.45988200
	H	-1.58612800	-4.88812600	1.48045600
14	O	1.25148100	0.66635800	-1.26031300
	C	2.49500600	0.22537400	-1.47959200
	C	0.51105200	1.25980400	-2.34163600
	H	1.19592700	1.44952100	-3.17154000
	C	-0.53990800	0.25763000	-2.80660700
	H	-1.18214700	0.74816700	-3.54084400
	C	-0.07625600	2.55123000	-1.80256700
	H	0.72757900	3.24205600	-1.54295200
	H	-0.66263200	2.35355100	-0.90289300
	C	0.04820000	-1.02151900	-3.36988000
	H	0.71408400	-0.79594500	-4.20411800
	O	3.01202900	0.17551300	-2.56090900
	C	3.19022500	-0.06756700	-0.13492500
	C	4.33560700	-1.08280400	-0.24562300
	C	5.19850200	-1.20622300	0.84858600
	C	6.20396200	-2.15991700	0.86545500
	C	6.36185700	-3.02522200	-0.21358700
	C	5.50163000	-2.92273700	-1.29641500
	C	4.49338500	-1.96057600	-1.31574700
	C	2.20384400	-0.66910800	0.87867100
	C	1.34857300	-1.69267600	0.46315400
	C	0.51693200	-2.34293800	1.36149700
	C	0.53558300	-1.98720600	2.70829800
	C	1.39313200	-0.98336100	3.13735700
	C	2.22249400	-0.32814400	2.22842700
	C	3.67998800	1.34977200	0.23197800
	C	2.76425000	2.29801900	0.69969000
	C	3.16056200	3.60524900	0.94826300
	C	4.47700600	3.99323400	0.71848700
	C	5.38460400	3.06644900	0.22290300
	C	4.98851000	1.75568200	-0.02695400
	H	5.07809700	-0.54250600	1.69795700
	H	6.86178300	-2.23106500	1.72354800
	H	7.14612200	-3.77266800	-0.20498900

	H	5.60977900	-3.59313400	-2.14086700
	H	3.84723400	-1.89264200	-2.17889800
	H	1.33623200	-1.99167000	-0.57945100
	H	-0.15693800	-3.11596600	1.01103000
	H	-0.11844700	-2.48964300	3.41105300
	H	1.42175600	-0.70229900	4.18402500
	H	2.88667300	0.45286900	2.58084200
	H	1.73038200	2.01102800	0.84952600
	H	2.43446800	4.32177800	1.31559300
	H	4.78831900	5.01265300	0.91303900
	H	6.40737500	3.36109300	0.01994000
	H	5.70263100	1.04911500	-0.43103800
	H	-0.72271600	3.01647400	-2.54886900
	H	-0.74859900	-1.68039400	-3.71787800
	H	0.61994800	-1.54606200	-2.60358900
	O	-1.33947400	-0.10745700	-1.66568200
	C	-2.56670200	0.41314800	-1.54607700
	C	-3.17203400	0.07577000	-0.16857100
	C	-2.89312400	-1.36578300	0.27972800
	C	-2.96037400	-1.69929000	1.63350200
	O	-3.07724100	1.13059400	-2.36048600
	C	-2.74902600	-2.39696700	-0.64879900
	C	-2.66609700	-3.72266300	-0.23754000
	C	-2.73318700	-4.04297500	1.11355200
	C	-2.88098300	-3.02318200	2.04752800
	C	-4.70244900	0.20310600	-0.18156400
	C	-5.44817000	-0.16189600	-1.30221000
	C	-6.83742000	-0.15953300	-1.26063500
	C	-7.50570500	0.19870200	-0.09633100
	C	-6.77139200	0.55044100	1.03002000
	C	-5.38271400	0.54986100	0.98668400
	C	-2.48887400	1.15270600	0.69940000
	C	-1.29427000	0.89730500	1.37310500
	C	-0.65303500	1.91504700	2.07496800
	C	-1.16910100	3.20532900	2.07435300
	C	-2.33489600	3.47649900	1.36454900
	C	-2.98896700	2.45811200	0.68264700
	H	-3.08469500	-0.91581900	2.37253500
	H	-2.71139800	-2.17214100	-1.70807000
	H	-2.55637500	-4.50626700	-0.97806600
	H	-2.67539100	-5.07598500	1.43524300
	H	-2.94328700	-3.25741800	3.10401100
	H	-4.94698900	-0.44000200	-2.22001400
	H	-7.39798300	-0.43817700	-2.14513500
	H	-8.58859600	0.20162700	-0.06628900
	H	-7.27864900	0.82543000	1.94714300
	H	-4.82310000	0.82697300	1.87277900
	H	-0.85004200	-0.09034800	1.34360000
	H	0.25675500	1.68591400	2.61753100
	H	-0.66562600	3.99545600	2.61951200
	H	-2.73784400	4.48193700	1.33999600
	H	-3.88937000	2.67733300	0.12047500

17	O	1.33658400	-0.37923500	1.52771200
	C	2.58338800	0.11428600	1.56982200
	C	0.51302500	-0.28478600	2.70780400
	H	1.15722900	-0.04852000	3.55750700
	C	-0.46142300	0.87819500	2.53478700
	H	-1.11350500	0.89641000	3.41083100
	C	-0.15186200	-1.63552000	2.89503500
	H	-0.71871300	-1.91895800	2.00653400
	H	0.60794900	-2.39844300	3.07183000
	C	0.22459900	2.21392600	2.32036100
	H	-0.51100800	3.01933600	2.33318900
	O	3.07790300	0.60684500	2.54458200
	C	3.22786200	0.07001800	0.16845400
	C	3.00024100	-1.27784000	-0.52756000
	C	3.05150600	-1.37186700	-1.91902400
	C	2.99522100	-2.60827100	-2.55176500
	C	2.89847700	-3.77606000	-1.80239100
	C	2.85716800	-3.69443100	-0.41528300
	C	2.90735400	-2.45600100	0.21417100
	C	4.75101600	0.24533200	0.22908900
	C	5.50594600	-0.29417200	1.27009300
	C	6.89422700	-0.22836500	1.24559600
	C	7.55218600	0.36800200	0.17701500
	C	6.80889200	0.89497200	-0.87219400
	C	5.42128600	0.83109600	-0.84614500
	C	2.51227900	1.26731300	-0.48780200
	C	2.94553000	2.56307000	-0.19286300
	C	2.21666400	3.66701400	-0.61999400
	C	1.03276000	3.49199500	-1.32917700
	C	0.58907800	2.20681900	-1.61705500
	C	1.32577400	1.10310400	-1.20142000
	H	3.14065000	-0.46989200	-2.51400300
	H	3.03302600	-2.65737400	-3.63347100
	H	2.85893000	-4.74024100	-2.29489200
	H	2.78596800	-4.59604000	0.18137100
	H	2.88006000	-2.41392300	1.29727100
	H	5.01364300	-0.75843500	2.11407500
	H	7.46168900	-0.64646900	2.06853300
	H	8.63419800	0.41962600	0.16097000
	H	7.30748600	1.35854400	-1.71521100
	H	4.85380100	1.24832800	-1.67024300
	H	3.84662800	2.70460800	0.39292500
	H	2.56808000	4.66377200	-0.38096300
	H	0.45120500	4.34916400	-1.64888600
	H	-0.34098000	2.05694300	-2.15129800
	H	0.95849300	0.10893000	-1.42216800
	H	-0.83261500	-1.60464100	3.74681300
	H	0.72131000	2.22556800	1.34910400
	H	0.96819300	2.39199800	3.09915200
	O	-1.27336400	0.65593500	1.36493900
	C	-2.49443900	0.12456600	1.51252500
	C	-3.19721400	-0.06082700	0.14027900

	C	-2.94559900 1.15975700 -0.75676700
	C	-2.92358100 1.02854900 -2.14474500
	O	-2.95613000 -0.18071900 2.57526900
	C	-2.87417900 2.44189200 -0.20864100
	C	-2.77218000 3.56216400 -1.02331200
	C	-2.76015800 3.42204400 -2.40790100
	C	-2.84109100 2.15129100 -2.96464800
	C	-4.70375400 -0.23169000 0.39703500
	C	-5.14923400 -1.27908600 1.21330600
	C	-6.50141100 -1.47473100 1.44573800
	C	-7.44412600 -0.62901100 0.86530800
	C	-7.01571600 0.40715200 0.05133700
	C	-5.65513600 0.60470900 -0.18229200
	C	-2.59124300 -1.32224800 -0.52474500
	C	-3.39827900 -2.26999300 -1.15590100
	C	-2.83239600 -3.34847000 -1.83213100
	C	-1.45336000 -3.50068300 -1.88933800
	C	-0.63923300 -2.55823500 -1.27040000
	C	-1.20304700 -1.47893600 -0.60191800
	H	-2.98413800 0.04377600 -2.59285300
	H	-2.91073100 2.57218900 0.86736100
	H	-2.71053200 4.54698600 -0.57519200
	H	-2.69175800 4.29561200 -3.04552200
	H	-2.83500100 2.02685700 -4.04115700
	H	-4.42779100 -1.94842800 1.66370300
	H	-6.82114000 -2.29119700 2.08230800
	H	-8.50119700 -0.78237400 1.04749700
	H	-7.73618100 1.07253100 -0.40950800
	H	-5.34906700 1.42052900 -0.82419200
	H	-4.47564100 -2.16759400 -1.13369300
	H	-3.48112100 -4.06946200 -2.31573400
	H	-1.01413200 -4.34233300 -2.41206200
	H	0.44061200 -2.65375500 -1.30368400
	H	-0.54876200 -0.75249800 -0.14033700
20	O	1.22650600 0.59352500 -1.32439800
	C	2.45652300 0.09004900 -1.49271100
	C	0.49112300 1.04454600 -2.47836500
	H	1.18483000 1.13983000 -3.31684700
	C	-0.55020900 -0.00833900 -2.84634700
	H	-1.19508700 0.41235200 -3.62063900
	C	-0.11147800 2.38892400 -2.10977000
	H	0.68110700 3.12307900 -1.95561300
	H	-0.69136000 2.31189100 -1.18789600
	C	0.03791000 -1.33032700 -3.29819100
	H	0.59660200 -1.79886400 -2.48851200
	O	2.92873500 -0.14867800 -2.56803400
	C	3.16806900 -0.08386100 -0.12289200
	C	2.16244000 -0.64284000 0.89379000
	C	2.14106000 -0.21878700 2.21897100
	C	1.29464000 -0.82879300 3.14487300
	C	0.45922400 -1.86594800 2.75470700
	C	0.47505700 -2.29972800 1.43036400

	C	1.32364200	-1.69482900	0.51597600	
	C	3.63900300	1.32958900	0.30245600	
	C	2.70006700	2.36162300	0.43423800	
	C	3.08270000	3.62472600	0.86319500	
	C	4.41244500	3.88680900	1.18016800	
	C	5.34658500	2.86741500	1.07182300	
	C	4.96491500	1.59864500	0.63895000	
	C	4.35420800	-1.05175900	-0.27226000	
	C	5.31203000	-0.85993700	-1.27686800	
	C	6.40896800	-1.70302500	-1.38415800	
	C	6.58263700	-2.75325700	-0.48727600	
	C	5.64961400	-2.94422800	0.52004200	
	C	4.54635600	-2.10072300	0.62671200	
	H	2.79320200	0.58481700	2.54084600	
	H	1.29255400	-0.48514000	4.17284400	
	H	-0.20908100	-2.33279300	3.46843400	
	H	-0.18401500	-3.09873800	1.11174200	
	H	1.34959300	-2.05855700	-0.50519300	
	H	1.65663000	2.16737500	0.22367700	
	H	2.33346100	4.40317300	0.95575100	
	H	4.71326800	4.87231700	1.51497100	
	H	6.38378800	3.05041100	1.32685300	
	H	5.71211800	0.81905900	0.57527800	
	H	5.19971600	-0.04380500	-1.97673900	
	H	7.13330900	-1.53657900	-2.17283600	
	H	7.43953300	-3.41084100	-0.57321700	
	H	5.77184600	-3.75155700	1.23224300	
	H	3.83587800	-2.27275500	1.42437100	
	H	-0.77046400	2.73881400	-2.90627000	
	H	0.71746800	-1.17607700	-4.13717400	
	H	-0.76075600	-2.00766100	-3.60442500	
	O	-1.35017100	-0.27423300	-1.67871700	
	C	-2.57435600	0.26217100	-1.60318100	
	C	-3.17037800	0.06426700	-0.19567100	
	C	-2.46162300	1.20986500	0.55674500	
	C	-1.24419800	1.00843900	1.20903700	
	O	-3.08588000	0.90202600	-2.47935200	
	C	-2.95883600	2.51137700	0.44148900	
	C	-2.27938600	3.58289200	1.00764100	
	C	-1.08825900	3.36964900	1.69436000	
	C	-0.57364500	2.08195700	1.78972500	
	C	-2.90360100	-1.33105100	0.38494800	
	C	-2.94802100	-1.52767900	1.76539400	
	C	-2.88736400	-2.80580200	2.30743600	
	C	-2.77818500	-3.91551100	1.47690700	
	C	-2.73089300	-3.73126200	0.09958500	
	C	-2.79501400	-2.45115200	-0.43981900	
	C	-4.69931700	0.20750700	-0.20461600	
	C	-5.36188300	0.66105500	0.93708600	
	C	-6.74970000	0.67959300	0.99651000	
	C	-7.50123900	0.23847300	-0.08612300	
	C	-6.85072800	-0.22595300	-1.22245200	

	C	-5.46203300	-0.24547100	-1.28028900
	H	-0.80353900	0.01924900	1.25373400
	H	-3.87778000	2.68485400	-0.10617100
	H	-2.68250000	4.58388700	0.90958500
	H	-0.56302400	4.20168900	2.14918800
	H	0.36144300	1.90235800	2.30701200
	H	-3.03969900	-0.67301200	2.42613600
	H	-2.93369300	-2.93355500	3.38290000
	H	-2.73486900	-4.91323100	1.89712800
	H	-2.65158000	-4.58621600	-0.56162100
	H	-2.77115600	-2.33193200	-1.51673400
	H	-4.78829600	1.00673700	1.78950600
	H	-7.24226100	1.03876300	1.89232500
	H	-8.58363000	0.25425100	-0.04355100
	H	-7.42463300	-0.57439100	-2.07295800
	H	-4.97567200	-0.60616300	-2.17694100
21	O	-1.34710800	0.18730500	1.63837100
	C	-2.64997500	0.47800100	1.55696000
	C	-0.61571100	0.73227100	2.75119000
	H	-1.19863500	0.56640500	3.66208400
	C	0.65654100	-0.09240200	2.84120300
	H	1.27025300	0.28883500	3.66201800
	C	-0.38206000	2.21760900	2.53839100
	H	0.24775200	2.61413200	3.33763100
	H	-1.33394500	2.74827600	2.54837400
	C	0.37564400	-1.57567400	3.02411500
	H	-0.24421700	-1.72826400	3.91086400
	O	-3.26036400	1.04439900	2.42037200
	C	-3.19055800	0.09759200	0.16529600
	C	-4.72399300	0.15723800	0.11230000
	C	-5.37469400	0.51969700	-1.06777400
	C	-6.75983600	0.45730200	-1.16676500
	C	-7.51894000	0.02644500	-0.08615800
	C	-6.87900000	-0.35016000	1.08869800
	C	-5.49422500	-0.29238100	1.18529500
	C	-2.83164100	-1.34317800	-0.22048100
	C	-2.60373200	-2.32010700	0.74790500
	C	-2.45576400	-3.65736800	0.39202700
	C	-2.54215900	-4.04026000	-0.94068000
	C	-2.76552600	-3.07223000	-1.91496600
	C	-2.90707600	-1.73800300	-1.55710800
	C	-2.51266600	1.17670000	-0.70246800
	C	-1.30821100	0.93946900	-1.36633900
	C	-0.67474600	1.97006900	-2.05534200
	C	-1.21526800	3.25055300	-2.06492000
	C	-2.39382000	3.50220500	-1.37073700
	C	-3.03430200	2.47322900	-0.69142100
	H	-4.79694900	0.85914500	-1.91953000
	H	-7.24372000	0.74626100	-2.09222700
	H	-8.59897300	-0.01869400	-0.15945600
	H	-7.45885800	-0.69191500	1.93787900
	H	-5.01472600	-0.59115700	2.10788500

	H	-2.55827200	-2.04648900	1.79656000
	H	-2.28711200	-4.40051200	1.16282200
	H	-2.44010400	-5.08275500	-1.21798100
	H	-2.84099900	-3.35778900	-2.95792000
	H	-3.09438900	-0.99426400	-2.32392400
	H	-0.84773100	-0.04300000	-1.33515200
	H	0.25750800	1.76812200	-2.56928100
	H	-0.71546100	4.04810700	-2.60279100
	H	-2.81664400	4.49968200	-1.35462400
	H	-3.94692100	2.67600100	-0.14206400
	H	0.11534300	2.38966700	1.58378100
	H	1.30716300	-2.12498200	3.15708900
	H	-0.15716500	-1.95536600	2.15005900
	O	1.36122400	0.12662800	1.60451700
	C	2.64778700	-0.24273400	1.55956600
	C	3.22762100	-0.09578400	0.12470300
	C	2.24133600	-0.74144000	-0.86084000
	C	2.11994300	-0.27298800	-2.16746700
	O	3.24184600	-0.64102200	2.52131900
	C	1.52661900	-1.88214700	-0.49249600
	C	0.68313300	-2.51843300	-1.39254200
	C	0.55962500	-2.03635900	-2.69255000
	C	1.28946800	-0.91947100	-3.07987100
	C	4.58663600	-0.81035800	0.05785400
	C	5.57942400	-0.53429700	1.00691800
	C	6.82312100	-1.14431700	0.93640600
	C	7.10853000	-2.04485700	-0.08627700
	C	6.13632500	-2.32357500	-1.03371200
	C	4.88619600	-1.71247100	-0.96201800
	C	3.35173400	1.41953600	-0.16768800
	C	2.18356500	2.18695100	-0.25072800
	C	2.23614700	3.54410800	-0.53151700
	C	3.46248700	4.16737600	-0.74784300
	C	4.62425300	3.41167700	-0.69526500
	C	4.57132900	2.04737200	-0.41202700
	H	2.68383500	0.59959300	-2.47839000
	H	1.63361500	-2.28627600	0.50885400
	H	0.11614500	-3.38618100	-1.07840400
	H	-0.10479600	-2.52851200	-3.39287300
	H	1.20880700	-0.53988900	-4.09202100
	H	5.37640900	0.16403400	1.80748900
	H	7.57204400	-0.91692900	1.68584300
	H	8.07971900	-2.52236100	-0.14008900
	H	6.34051900	-3.02216400	-1.83641300
	H	4.14511600	-1.95309000	-1.71290700
	H	1.22308700	1.70486400	-0.12109300
	H	1.31131000	4.10781400	-0.58935300
	H	3.50771100	5.22759600	-0.96670200
	H	5.58534700	3.87708900	-0.87990900
	H	5.49178600	1.48005400	-0.39773300
22	O	1.34948100	0.48049800	-1.36272400
	C	2.61188700	0.88527800	-1.21397700

	C	0.57627900	0.99336100	-2.45842400
	H	1.20895400	0.99558400	-3.35153300
	C	-0.53943000	-0.02544900	-2.64044900
	H	-1.18642800	0.29609900	-3.46126600
	C	0.07024600	2.39110900	-2.14887900
	H	0.90986900	3.08070300	-2.06817200
	H	-0.49237200	2.38625900	-1.21351600
	C	-0.04339100	-1.44286200	-2.86792600
	H	-0.89099200	-2.11023000	-3.02731400
	O	3.13064900	1.69544900	-1.92907800
	C	3.29114400	0.09007900	-0.06672900
	C	2.59931600	0.38563400	1.28707400
	C	1.21694100	0.21266400	1.44877700
	C	0.62062900	0.36570600	2.69401000
	C	1.38115200	0.71118200	3.80632800
	C	2.74845200	0.88924200	3.65866100
	C	3.35265300	0.71912600	2.41497100
	C	4.77727800	0.46017500	-0.00831300
	C	5.16086000	1.80522700	0.02278200
	C	6.49499000	2.16049800	0.15342200
	C	7.47628000	1.17886200	0.26475200
	C	7.10594400	-0.15744300	0.24920100
	C	5.76577700	-0.51403100	0.11385900
	C	3.11685500	-1.38909000	-0.46616500
	C	2.66548100	-2.36689800	0.41464000
	C	2.53797100	-3.69078500	-0.00595500
	C	2.85952700	-4.05212500	-1.30670700
	C	3.32525000	-3.08080800	-2.19000700
	C	3.45486300	-1.76542800	-1.77042600
	H	0.59784300	-0.07264400	0.60857200
	H	-0.44303200	0.18088300	2.79602100
	H	0.91267800	0.82831600	4.77659000
	H	3.36104200	1.15089200	4.51356000
	H	4.42488500	0.83984800	2.33839700
	H	4.40846600	2.57922400	-0.05275700
	H	6.76994600	3.20862800	0.16864500
	H	8.51855700	1.45714600	0.36558500
	H	7.85715800	-0.93271300	0.34357400
	H	5.49708300	-1.56313300	0.11012500
	H	2.41000900	-2.10512000	1.43399500
	H	2.18734200	-4.44028300	0.69440200
	H	2.75501100	-5.08029200	-1.63146600
	H	3.58766300	-3.34941600	-3.20665400
	H	3.83028800	-1.01407500	-2.46011100
	H	-0.58949700	2.73387700	-2.94954000
	H	0.53905400	-1.78070000	-2.00759100
	H	0.59926100	-1.47941000	-3.75074700
	O	-1.30074400	0.03439400	-1.42004100
	C	-2.59318700	-0.30707000	-1.47277800
	C	-3.27806400	0.02434100	-0.12956300
	C	-2.66594100	-0.78465800	1.02611500
	C	-3.18754500	-0.59177600	2.31084300

	O	-3.12669200	-0.73581700	-2.45648100
	C	-1.69210000	-1.76668000	0.84971300
	C	-1.24521700	-2.52828300	1.92871400
	C	-1.76316100	-2.32159300	3.19819900
	C	-2.73934700	-1.34503800	3.38575400
	C	-4.76663900	-0.34822000	-0.18740100
	C	-5.13104900	-1.63490100	-0.59296500
	C	-6.45859400	-2.03415400	-0.57637600
	C	-7.44973900	-1.15884000	-0.14047900
	C	-7.09638100	0.11421000	0.28145100
	C	-5.76302600	0.51756200	0.25774400
	C	-3.05384900	1.54397400	-0.00730800
	C	-3.59449100	2.36422200	-1.00401800
	C	-3.35839200	3.73144700	-1.00556800
	C	-2.57204100	4.30608100	-0.00970300
	C	-2.02468600	3.49858800	0.97684100
	C	-2.26000000	2.12462700	0.97564800
	H	-3.96549600	0.14948400	2.46099400
	H	-1.25984000	-1.94966400	-0.12637900
	H	-0.48401500	-3.28207400	1.76430600
	H	-1.41470000	-2.91368000	4.03578900
	H	-3.16074400	-1.17804600	4.37012300
	H	-4.36801800	-2.32762800	-0.92608700
	H	-6.72136800	-3.03348300	-0.90217700
	H	-8.48696700	-1.47198100	-0.12629400
	H	-7.85565200	0.80308400	0.63250600
	H	-5.50539500	1.51507700	0.59198500
	H	-4.20508700	1.91844500	-1.78374200
	H	-3.78620400	4.34941200	-1.78612900
	H	-2.38424400	5.37299100	-0.01015900
	H	-1.39724100	3.92822500	1.74878100
	H	-1.80038100	1.51182300	1.73797400
23	O	1.26795200	1.41461800	0.13380000
	C	2.09536900	0.83726100	-0.74923300
	C	0.66399800	2.66268000	-0.24993800
	H	0.45047800	2.63343000	-1.32090300
	C	-0.64038500	2.72685700	0.52136300
	H	-0.45491400	2.50778300	1.57547400
	C	1.63630100	3.78080100	0.08561700
	H	2.59484500	3.58995300	-0.40175100
	H	1.80663700	3.82746700	1.16414600
	C	-1.39164900	4.03548200	0.36097100
	H	-2.37186600	3.94663400	0.83060300
	O	2.14370500	1.12848200	-1.91119300
	C	3.08261800	-0.09970200	-0.02372200
	C	3.84743000	-0.98380100	-1.01342400
	C	5.18776200	-1.30621200	-0.80623700
	C	5.83925300	-2.20803600	-1.64148200
	C	5.15741200	-2.80369900	-2.69438900
	C	3.81584000	-2.49764500	-2.89910900
	C	3.16568600	-1.60005900	-2.06346300
	C	2.39230800	-1.05893900	0.95158000

	C	1.05439300	-1.42055900	0.80598800
	C	0.49153500	-2.38541000	1.63851600
	C	1.25417700	-2.99731600	2.62422400
	C	2.59472800	-2.65244600	2.76475500
	C	3.15822600	-1.69895800	1.92892700
	C	3.97210400	0.95930700	0.66115800
	C	3.77232700	1.34025800	1.98756600
	C	4.49788300	2.39151200	2.53828300
	C	5.41888900	3.09017900	1.76699300
	C	5.60584700	2.73422200	0.43550000
	C	4.88474600	1.68084100	-0.11357800
	H	5.73244800	-0.84951000	0.01211100
	H	6.88210800	-2.44337500	-1.46438400
	H	5.66425000	-3.50404400	-3.34740700
	H	3.27223000	-2.96218100	-3.71391700
	H	2.11823300	-1.37752400	-2.22385000
	H	0.43737200	-0.95563000	0.04323400
	H	-0.54823500	-2.65926600	1.50495000
	H	0.81184000	-3.74472000	3.27213900
	H	3.20535300	-3.13281500	3.52009500
	H	4.20864100	-1.44911700	2.03289200
	H	3.03619600	0.82319400	2.59001000
	H	4.33402000	2.66795300	3.57327000
	H	5.98204600	3.90938800	2.19798200
	H	6.31073300	3.27973100	-0.18058900
	H	5.01782200	1.42001300	-1.15785400
	H	1.26014500	4.74326700	-0.26420700
	H	-1.52769400	4.27117400	-0.69720100
	H	-0.85411100	4.85286600	0.84362200
	O	-1.42792800	1.65846200	-0.03248000
	C	-2.33298600	1.07422900	0.75110400
	C	-3.12475700	-0.02861200	-0.00044900
	C	-4.18295100	0.74008800	-0.82178400
	C	-4.51509400	0.38333300	-2.12714600
	O	-2.55104600	1.42511900	1.87789600
	C	-4.89791800	1.77194000	-0.20596000
	C	-5.89939500	2.44822400	-0.88949700
	C	-6.21607100	2.09500400	-2.19731200
	C	-5.52610900	1.05720000	-2.80877500
	C	-3.81531200	-0.92281800	1.03674100
	C	-3.11619100	-1.34391900	2.17197600
	C	-3.68210600	-2.24561600	3.06179500
	C	-4.96172000	-2.74492800	2.83475900
	C	-5.66501300	-2.33056800	1.71273200
	C	-5.09583400	-1.42447700	0.81999800
	C	-2.19418600	-0.86957400	-0.90321800
	C	-2.05966400	-2.24688600	-0.72774700
	C	-1.19636500	-2.99479400	-1.52846000
	C	-0.46074900	-2.38287200	-2.52993400
	C	-0.61180000	-1.01395000	-2.74375000
	C	-1.47368300	-0.27192000	-1.94982500
	H	-3.99269500	-0.42858100	-2.61746000

	H	-4.68027600	2.03193800	0.82443800
	H	-6.43820100	3.24792400	-0.39501400
	H	-6.99873300	2.62073900	-2.73115900
	H	-5.77157800	0.76413300	-3.82276500
	H	-2.12057000	-0.96349500	2.36515800
	H	-3.12287900	-2.55394200	3.93750500
	H	-5.40511900	-3.44715200	3.53076600
	H	-6.66307500	-2.70872000	1.52561200
	H	-5.65891700	-1.11368400	-0.05187700
	H	-2.62399000	-2.75616200	0.04257800
	H	-1.10063200	-4.05981700	-1.35391100
	H	0.21806900	-2.96358700	-3.14394500
	H	-0.05362200	-0.51595300	-3.52842900
	H	-1.59120100	0.78403500	-2.14684700
25	O	1.37529200	1.69381400	0.34470500
	C	2.03798800	1.04619200	-0.62457700
	C	0.63082800	2.87345700	-0.01184400
	H	0.41268000	2.83687100	-1.08080200
	C	-0.67290700	2.78227300	0.76282900
	H	-0.46812800	2.53630100	1.80799700
	C	1.47536200	4.08991300	0.32310600
	H	0.99389800	5.00508800	-0.02343500
	H	2.44346800	4.00394500	-0.17376600
	C	-1.53510000	4.02751700	0.65093600
	H	-2.49779800	3.84580100	1.12820700
	O	1.98415200	1.36680100	-1.77920300
	C	2.94659500	-0.06711200	-0.03300700
	C	3.12273100	-1.23567500	-1.02477700
	C	3.14138300	-2.55345800	-0.56105400
	C	3.37857900	-3.61799200	-1.42606200
	C	3.59370000	-3.38971900	-2.77806900
	C	3.58628300	-2.08150000	-3.25166200
	C	3.36793700	-1.01595000	-2.38607200
	C	2.34011700	-0.59648100	1.27262900
	C	1.00716300	-1.01588900	1.25469900
	C	0.43912900	-1.62492200	2.36197900
	C	1.19415100	-1.81550800	3.51831000
	C	2.51657400	-1.39826200	3.54577300
	C	3.09087300	-0.79643700	2.42539200
	C	4.29554700	0.64687400	0.22154200
	C	4.32517300	1.78173600	1.04197000
	C	5.51783800	2.44090200	1.30396600
	C	6.71081200	1.97427700	0.75942100
	C	6.69438600	0.84034100	-0.03926500
	C	5.49685400	0.18051100	-0.30791600
	H	2.96703600	-2.76483100	0.48556100
	H	3.38907700	-4.62828300	-1.03420400
	H	3.77289700	-4.21700200	-3.45471400
	H	3.76524900	-1.88267500	-4.30202800
	H	3.39068800	-0.00715400	-2.77055200
	H	0.41840700	-0.87968800	0.35410000
	H	-0.58868000	-1.96681700	2.31260400

	H	0.75293100	-2.29019900	4.38666100	
	H	3.11492000	-1.54677100	4.43698500	
	H	4.13181600	-0.49800300	2.45646700	
	H	3.40660200	2.13295100	1.49638600	
	H	5.51605900	3.31756300	1.94129800	
	H	7.64301500	2.48721300	0.96341900	
	H	7.61692600	0.45822400	-0.46031400	
	H	5.50979100	-0.70486300	-0.92967500	
	H	1.64006000	4.16193800	1.40148100	
	H	-1.70290500	4.28225500	-0.39846800	
	H	-1.06054900	4.87156600	1.15316000	
	O	-1.35644200	1.67028700	0.15984700	
	C	-2.31512900	1.06233200	0.86163400	
	C	-3.02534500	-0.02613800	0.01393500	
	C	-3.91756900	0.76919200	-0.96473200	
	C	-4.07430600	0.39933600	-2.29888600	
	O	-2.62288800	1.39429200	1.97318700	
	C	-4.66623600	1.84338800	-0.47596200	
	C	-5.52359000	2.55024400	-1.30850300	
	C	-5.66116300	2.18518800	-2.64366600	
	C	-4.94100200	1.10296300	-3.13123400	
	C	-3.91128600	-0.89821900	0.91406100	
	C	-3.49276600	-1.28474400	2.19055400	
	C	-4.24712800	-2.17062800	2.94923400	
	C	-5.43626100	-2.69002000	2.44849600	
	C	-5.86316400	-2.31045300	1.18346000	
	C	-5.10767000	-1.42170000	0.42465300	
	C	-1.97513100	-0.88893500	-0.72179800	
	C	-1.80301800	-2.24194600	-0.42980900	
	C	-0.81056300	-2.99174100	-1.06384900	
	C	0.01927400	-2.40768200	-2.00775300	
	C	-0.16192800	-1.06488700	-2.33152500	
	C	-1.14782200	-0.31971300	-1.70319900	
	H	-3.52624500	-0.44670700	-2.69410200	
	H	-4.59239700	2.11420700	0.57215100	
	H	-6.09124900	3.38275600	-0.90993400	
	H	-6.32969100	2.73587800	-3.29447300	
	H	-5.04980600	0.79817100	-4.16534600	
	H	-2.57747200	-0.88312500	2.60349900	
	H	-3.90469900	-2.45186500	3.93812700	
	H	-6.02543000	-3.37880800	3.04212100	
	H	-6.78948300	-2.70259200	0.78035900	
	H	-5.45895600	-1.13757500	-0.55970800	
	H	-2.43603700	-2.72781100	0.30137700	
	H	-0.68826400	-4.03643600	-0.80306800	
	H	0.80738200	-2.97849600	-2.48524900	
	H	0.48340500	-0.58886500	-3.06024600	
	H	-1.27055100	0.72072100	-1.97031900	

21 (optimized at the B3LYP/6-311G(d,p) level)

Conformer no	
1	O -1.75074800 -1.68170200 -0.23110800<br>C -2.08266200 -0.46972200 -0.72088900<br>C -0.70077500 -2.43132300 -0.93092500<br>H -0.02219700 -1.70628600 -1.37730100<br>C -1.33451300 -3.28087100 -2.02408100<br>H -0.55560100 -3.81260100 -2.57669200<br>H -1.87975900 -2.65287900 -2.73026300<br>O -1.50974200 0.03195700 -1.65514100<br>C -3.28657900 0.17440800 0.05127300<br>C -3.07986100 -0.04760800 1.56763800<br>C -1.83330500 0.23914800 2.14031000<br>C -1.62174000 0.10189200 3.50762500<br>C -2.66085000 -0.31694100 4.33854700<br>C -3.90601600 -0.59253400 3.78431800<br>C -4.11359300 -0.45976700 2.41013400<br>C -4.59358300 -0.52216700 -0.43874200<br>C -4.69551100 -1.92053200 -0.48935700<br>C -5.87614600 -2.54329200 -0.88442600<br>C -6.99361500 -1.78643800 -1.22743800<br>C -6.91504800 -0.39986500 -1.16072200<br>C -5.73025600 0.22402900 -0.77118100<br>C -3.32835600 1.68977800 -0.27662200<br>C -3.41127300 2.65715200 0.72869400<br>C -3.48899400 4.01651100 0.42010600<br>C -3.48868700 4.43763300 -0.90362600<br>C -3.42163300 3.48367500 -1.91864500<br>C -3.34764000 2.13003400 -1.61018100<br>H -1.01211500 0.58612500 1.52368200<br>H -0.64219500 0.32411900 3.91497700<br>H -2.49980400 -0.42347900 5.40556200<br>H -4.72555600 -0.91367100 4.41791800<br>H -5.09107100 -0.67774200 2.00065300<br>H -3.85081600 -2.52802200 -0.20002800<br>H -5.92110900 -3.62645400 -0.91729300<br>H -7.91307700 -2.27143100 -1.53543500<br>H -7.77711400 0.20832200 -1.41161900<br>H -5.70057500 1.30339700 -0.72476100<br>H -3.41620100 2.36005700 1.76791700<br>H -3.54797200 4.74117900 1.22455300<br>H -3.54574300 5.49321400 -1.14530600<br>H -3.42936900 3.79295900 -2.95814700<br>H -3.30311600 1.41106400 -2.41479900<br>H -2.02353500 -4.01778600 -1.60284700<br>C 0.02536100 -3.25281800 0.12833900<br>C 0.79370300 -2.41919000 1.14856000<br>H 0.14127000 -1.67273400 1.59768600<br>C 1.45783800 -3.25264200 2.23505700<br>H 2.02191900 -2.61379800 2.91678600<br>H 2.13800700 -3.99057800 1.80153100<br>H 0.69784500 -3.78058900 2.81703000

	O	1.83125200	-1.71119600	0.39420700
	C	2.15075300	-0.46464700	0.77145300
	C	3.29409400	0.17841700	-0.07882500
	C	2.85685900	1.63781100	-0.36538500
	C	1.54590000	1.90553400	-0.78392700
	O	1.61376700	0.10592600	1.69121000
	C	3.74898400	2.70755200	-0.27235500
	C	3.34454200	4.00621500	-0.58406700
	C	2.03912600	4.25890300	-0.99020800
	C	1.13843900	3.19949500	-1.09003800
	C	3.53298300	-0.60668500	-1.39507700
	C	4.01683500	-1.92479000	-1.35052600
	C	4.25399200	-2.64655100	-2.51298500
	C	4.01867800	-2.06865800	-3.76134400
	C	3.54983100	-0.76334400	-3.82342900
	C	3.31267800	-0.03940300	-2.65213900
	C	4.58203300	0.17229900	0.80058600
	C	4.52692000	0.39386300	2.18329200
	C	5.69036100	0.44924400	2.94745500
	C	6.93834300	0.30233500	2.34903300
	C	7.00892600	0.10520000	0.97336300
	C	5.84518400	0.04021600	0.20950600
	H	0.82640600	1.10189000	-0.89084900
	H	4.76745200	2.53808800	0.05033200
	H	4.05686100	4.81972100	-0.50025000
	H	1.72312900	5.26934900	-1.22451200
	H	0.11560300	3.37389000	-1.40365000
	H	4.21382400	-2.38875900	-0.39317100
	H	4.62794600	-3.66247600	-2.44514800
	H	4.20578400	-2.63034800	-4.66982900
	H	3.36730000	-0.29340800	-4.78346500
	H	2.95525500	0.97736900	-2.73218600
	H	3.57066600	0.54230000	2.66499300
	H	5.61531600	0.61569400	4.01649900
	H	7.84299500	0.34704800	2.94501900
	H	7.97178900	0.00058200	0.48541700
	H	5.92761600	-0.11050700	-0.85835600
	H	0.72241600	-3.92661800	-0.38043700
	H	-0.69904200	-3.87542500	0.66356800
6	O	-1.87456300	1.50646100	0.57167300
	C	-2.38416700	0.31259000	0.92037700
	C	-0.79077700	2.05169400	1.39707700
	H	-0.16303700	1.21944700	1.71241900
	C	-1.38781500	2.74299700	2.61653000
	H	-1.95541300	2.03381500	3.22007100
	H	-2.04545300	3.56319400	2.31620300
	O	-1.96786300	-0.34325300	1.84495500
	C	-3.48648300	-0.17248400	-0.06569000
	C	-4.21148700	1.00379600	-0.76145900
	C	-4.52154200	0.99874100	-2.12346700
	C	-5.27352900	2.02719300	-2.69348400
	C	-5.73502200	3.07875200	-1.91070100

	C	-5.44702300	3.08744900	-0.54623600
	C	-4.70062600	2.06055500	0.01815100
	C	-4.60402900	-0.97759300	0.64702200
	C	-4.75966200	-1.05274400	2.03373700
	C	-5.84176100	-1.73317300	2.59564500
	C	-6.78630600	-2.35388400	1.78780200
	C	-6.64377600	-2.28464600	0.40267500
	C	-5.57124400	-1.60080600	-0.15604900
	C	-2.64710200	-1.06202600	-1.02344800
	C	-2.60363100	-2.45177400	-0.87277800
	C	-1.77756200	-3.23150600	-1.68032900
	C	-0.96490200	-2.63640900	-2.64074500
	C	-0.97482700	-1.25019000	-2.78039200
	C	-1.80485900	-0.47357900	-1.97697700
	H	-4.17999900	0.18888600	-2.75456400
	H	-5.49795100	1.99763700	-3.75405900
	H	-6.31808300	3.87853800	-2.35309300
	H	-5.80984500	3.89395300	0.08144200
	H	-4.50545600	2.07731300	1.08398900
	H	-4.02843900	-0.60618500	2.68992600
	H	-5.93591600	-1.77667000	3.67518000
	H	-7.62391200	-2.88349200	2.22757400
	H	-7.37250500	-2.75865000	-0.24556200
	H	-5.48671400	-1.54709700	-1.23461800
	H	-3.21415600	-2.93079800	-0.11972500
	H	-1.76700600	-4.30775900	-1.54858500
	H	-0.32207300	-3.24327300	-3.26838500
	H	-0.32273500	-0.76821700	-3.49860800
	H	-1.79619900	0.60264800	-2.09166100
	H	-0.58842200	3.15095100	3.24031700
	C	-0.00987300	3.00924900	0.50425800
	C	0.76908700	2.36410200	-0.63751100
	H	0.12036000	1.72298800	-1.23489600
	C	1.45331200	3.39351800	-1.52877100
	H	0.70370400	4.04842100	-1.98113100
	H	2.00508400	2.90287700	-2.33019800
	H	2.14557100	4.00741100	-0.94687800
	O	1.76963100	1.50426400	-0.00616300
	C	2.33625800	0.54148500	-0.75836500
	C	3.48405700	-0.16817300	0.01837900
	C	4.56434400	0.94632500	0.02966600
	C	4.75167500	1.79635900	1.12419500
	O	2.04025900	0.33394200	-1.90816800
	C	5.28582900	1.21045300	-1.14277200
	C	6.19645700	2.26152700	-1.20344800
	C	6.39810000	3.08044100	-0.09487700
	C	5.66669100	2.84592100	1.06602200
	C	3.95419300	-1.45028100	-0.70889600
	C	3.03270500	-2.32075000	-1.30331900
	C	3.44497400	-3.53112600	-1.85269800
	C	4.78535300	-3.90698600	-1.81318500
	C	5.70865200	-3.05909300	-1.21001300

	C	5.29543900	-1.84655100	-0.66197000
	C	3.03116000	-0.64142100	1.41779300
	C	3.97600900	-0.92251600	2.41233500
	C	3.58854600	-1.44645700	3.64247900
	C	2.24614300	-1.71321200	3.90336000
	C	1.29825100	-1.46264000	2.91582900
	C	1.69147900	-0.93889300	1.68590800
	H	4.17654200	1.64484900	2.02729200
	H	5.13103100	0.58906400	-2.01616000
	H	6.74637500	2.44002400	-2.12081700
	H	7.11052200	3.89658300	-0.13868800
	H	5.80152200	3.48418600	1.93240100
	H	1.98511500	-2.05836900	-1.34565100
	H	2.71030000	-4.18421100	-2.31080400
	H	5.10427000	-4.85031200	-2.24219100
	H	6.75562500	-3.33771300	-1.16123100
	H	6.03271200	-1.20502300	-0.19627400
	H	5.02668200	-0.73701500	2.22628000
	H	4.34111500	-1.65075900	4.39624500
	H	1.94457700	-2.11905800	4.86244300
	H	0.24742700	-1.66392700	3.08787300
	H	0.93069300	-0.77344000	0.93396600
	H	0.69464900	3.56238000	1.13396900
	H	-0.70453100	3.74007400	0.07785100
12	O	1.96913200	-1.48031900	1.06850000
	C	1.96798700	-0.19424900	0.67544500
	C	0.84036400	-1.99738900	1.84783500
	H	0.19709100	-1.15743500	2.10284400
	C	1.42103900	-2.63224900	3.10331000
	H	0.61826400	-3.05321300	3.71364100
	H	1.95095400	-1.89223200	3.70621800
	O	1.07570200	0.58120900	0.91526600
	C	3.23873900	0.12239500	-0.17168500
	C	4.52914000	-0.05870100	0.66465700
	C	5.77312400	0.06483600	0.02944200
	C	6.95996200	-0.01265600	0.74877700
	C	6.93458000	-0.19854600	2.13032000
	C	5.70877700	-0.30142400	2.77689400
	C	4.51942300	-0.23334700	2.05054600
	C	3.24319100	1.60431900	-0.62398600
	C	2.98650500	2.62537900	0.30121200
	C	3.08242900	3.96368100	-0.06350400
	C	3.45430600	4.31811300	-1.35905800
	C	3.73347800	3.31610600	-2.28108800
	C	3.62986100	1.97394500	-1.91588600
	C	3.12798200	-0.86535500	-1.36181300
	C	2.09611500	-0.68813800	-2.29505300
	C	1.91463600	-1.58779100	-3.34171200
	C	2.75792000	-2.68936000	-3.47471600
	C	3.77008700	-2.88789200	-2.54057400
	C	3.94831200	-1.98873900	-1.48973100
	H	5.81368200	0.23348600	-1.03969300

	H	7.90696100	0.08250300	0.22917800
	H	7.85920500	-0.25375900	2.69350800
	H	5.66920600	-0.43530200	3.85228200
	H	3.58319600	-0.31708400	2.58562800
	H	2.70389600	2.37786700	1.31465400
	H	2.86827900	4.73241300	0.67076300
	H	3.52977200	5.36204400	-1.64205800
	H	4.03296300	3.57138800	-3.29160500
	H	3.85189700	1.21423200	-2.65348300
	H	1.42018800	0.15366800	-2.20341100
	H	1.10748900	-1.42569600	-4.04692200
	H	2.62188600	-3.38773600	-4.29292300
	H	4.42533300	-3.74824600	-2.62222800
	H	4.72854900	-2.17355700	-0.76426000
	H	2.12057300	-3.43184600	2.84704000
	C	0.07663400	-2.99767300	0.98197800
	C	-0.62048000	-2.40270500	-0.23595500
	H	0.07433800	-1.80595700	-0.82452900
	C	-1.26400000	-3.45842200	-1.12545400
	H	-0.49556700	-4.12494900	-1.52546700
	H	-1.77601100	-2.98884900	-1.96636900
	H	-1.98764300	-4.05472000	-0.56376000
	O	-1.65633300	-1.50344800	0.27306200
	C	-2.01168800	-0.46829600	-0.51117800
	C	-3.27805400	0.25685600	0.02775900
	C	-3.31580400	0.35632800	1.56935400
	C	-4.52417700	0.33314100	2.27317700
	O	-1.48021900	-0.21227700	-1.56248900
	C	-2.14390900	0.63563300	2.28332700
	C	-2.17789500	0.84957300	3.65841200
	C	-3.38499600	0.79967900	4.35218400
	C	-4.55907300	0.54632000	3.65021400
	C	-4.38090000	-0.64933600	-0.58525000
	C	-4.81956500	-1.80411000	0.07514500
	C	-5.73688300	-2.66578900	-0.52143400
	C	-6.21940500	-2.40340500	-1.80103200
	C	-5.76278500	-1.27878500	-2.48300400
	C	-4.84786600	-0.41570000	-1.88464700
	C	-3.35094400	1.72025200	-0.46698700
	C	-2.20139600	2.47559700	-0.72206400
	C	-2.29464900	3.82641500	-1.05163000
	C	-3.53475400	4.45290900	-1.12729600
	C	-4.68544500	3.71557100	-0.85964800
	C	-4.59154400	2.36703700	-0.52912900
	H	-5.45317500	0.15097200	1.74840800
	H	-1.19374300	0.70474400	1.76804400
	H	-1.25502900	1.06569500	4.18578000
	H	-3.41080200	0.96712300	5.42313900
	H	-5.51044500	0.51932900	4.17026300
	H	-4.43802200	-2.03587100	1.06043200
	H	-6.06978800	-3.54681000	0.01629500
	H	-6.93517400	-3.07284900	-2.26489700

	H	-6.11417600	-1.07046000	-3.48747900
	H	-4.48982400	0.44431300	-2.43494400
	H	-1.22339700	2.01933300	-0.66869400
	H	-1.38716800	4.38630900	-1.24876700
	H	-3.60444000	5.50351200	-1.38641900
	H	-5.66020800	4.18899000	-0.90370300
	H	-5.49848900	1.81428800	-0.31649500
	H	-0.67094400	-3.49332900	1.61059800
	H	0.77379300	-3.76777900	0.63580700
15	O	1.93377900	-1.69332700	0.60517900
	C	1.90141500	-0.36151900	0.43153100
	C	0.84706100	-2.33435500	1.35316300
	H	0.21319600	-1.54683900	1.75517500
	C	1.49779500	-3.12808600	2.47608600
	H	0.72951000	-3.60611400	3.08921300
	H	2.08894300	-2.47136000	3.11674800
	O	0.97859200	0.34128500	0.76554300
	C	3.24673100	0.19061200	-0.11886700
	C	3.03237300	1.50723300	-0.90211600
	C	4.07675100	2.43353200	-1.00610700
	C	3.95268700	3.57351200	-1.79531100
	C	2.77977100	3.80817700	-2.50818100
	C	1.74028200	2.88617500	-2.42696300
	C	1.86481300	1.74582200	-1.63557800
	C	3.95521500	-0.75758100	-1.11202100
	C	3.22006400	-1.52214200	-2.02459600
	C	3.85277100	-2.28423400	-3.00211400
	C	5.24192000	-2.29331900	-3.09717800
	C	5.98645700	-1.52135300	-2.21050100
	C	5.34920700	-0.75952500	-1.23368000
	C	4.00834200	0.38841300	1.22113400
	C	4.75178300	-0.65260900	1.79157700
	C	5.34837600	-0.50374900	3.04169400
	C	5.19730500	0.68160800	3.75651300
	C	4.43109300	1.71055600	3.21533400
	C	3.83605300	1.56228700	1.96510800
	H	4.99991100	2.26769200	-0.46462000
	H	4.77698800	4.27582100	-1.85341700
	H	2.67963000	4.69546000	-3.12322200
	H	0.82112200	3.04701200	-2.97901000
	H	1.03425300	1.05427500	-1.59721800
	H	2.13864000	-1.51960500	-1.98554600
	H	3.25565100	-2.86772500	-3.69419500
	H	5.73627300	-2.88694600	-3.85774700
	H	7.06864800	-1.50467300	-2.27827600
	H	5.95034100	-0.15908300	-0.56291900
	H	4.86375900	-1.58733900	1.25832200
	H	5.92901400	-1.32023600	3.45665200
	H	5.66371700	0.79847300	4.72829000
	H	4.28855500	2.63258500	3.76781000
	H	3.22705700	2.36337000	1.56774900
	H	2.15546700	-3.90399600	2.07568800

	C	0.04287500	-3.20853300	0.39361900
	C	-0.70269000	-2.45491400	-0.70236200
	H	-0.02954400	-1.78735500	-1.23914000
	C	-1.40200300	-3.37806900	-1.69224200
	H	-2.11668800	-4.02657400	-1.17956800
	H	-0.66735200	-4.00546900	-2.20439200
	H	-1.93692900	-2.79626200	-2.44420500
	O	-1.70460400	-1.62206100	-0.04273300
	C	-2.02979200	-0.45882700	-0.64079000
	C	-3.28791400	0.19247100	0.00159800
	C	-3.33491500	0.05547500	1.53998400
	C	-4.55189300	-0.02149400	2.22549200
	O	-1.48140400	-0.04075100	-1.62983400
	C	-2.16093100	0.17154600	2.29378000
	C	-2.20143900	0.17219700	3.68535400
	C	-3.41742000	0.06866700	4.35682300
	C	-4.59342800	-0.02088100	3.61845900
	C	-4.39808000	-0.59256700	-0.74974100
	C	-4.81930400	-0.17552200	-2.01887900
	C	-5.73837800	-0.92311000	-2.75072900
	C	-6.24551600	-2.11364400	-2.23667600
	C	-5.80924900	-2.55703700	-0.99097900
	C	-4.88767300	-1.81011800	-0.26063900
	C	-3.33047900	1.71516300	-0.26423900
	C	-4.55878700	2.38565500	-0.29855500
	C	-4.61946000	3.77094400	-0.42423000
	C	-3.44843900	4.51920600	-0.50842700
	C	-2.22041000	3.86597600	-0.45515100
	C	-2.16040900	2.47982400	-0.33006500
	H	-5.48197900	-0.07712400	1.67436800
	H	-1.20403300	0.28155900	1.79895500
	H	-1.27635700	0.26475900	4.24394100
	H	-3.44852500	0.07002300	5.44063000
	H	-5.55099200	-0.08489300	4.12368700
	H	-4.42026200	0.73715600	-2.44150200
	H	-6.05289900	-0.57334200	-3.72774700
	H	-6.96436500	-2.69389100	-2.80426300
	H	-6.18159900	-3.49073600	-0.58386400
	H	-4.54416700	-2.18177100	0.69547300
	H	-5.48234000	1.82442800	-0.22747500
	H	-5.58536900	4.26334200	-0.45256700
	H	-3.49283300	5.59807200	-0.60752600
	H	-1.29733000	4.43240300	-0.50909300
	H	-1.19118200	2.00376000	-0.28178100
	H	-0.68068600	-3.78386000	0.98091500
	H	0.71864800	-3.92732600	-0.08156400
31	O	2.24549100	-1.21490000	-0.60107600
	C	2.95557800	-0.83574700	0.47666000
	C	1.18124700	-2.19705200	-0.40335500
	H	1.48794800	-2.83107800	0.42977600
	C	1.11038900	-2.99349900	-1.69711500
	H	0.38826200	-3.80867700	-1.60855500

	H	2.08568700	-3.42596400	-1.92660600
	O	2.71807900	-1.20826600	1.59845700
	C	4.18082000	0.02917000	0.06268900
	C	4.80049500	0.74823800	1.28411800
	C	6.17775700	0.98768100	1.34652800
	C	6.73323600	1.73826000	2.38043500
	C	5.91994100	2.27033800	3.37594000
	C	4.54629000	2.04873700	3.32001900
	C	3.99268600	1.30149900	2.28457400
	C	3.81015800	1.15668500	-0.92793500
	C	2.51836500	1.68780800	-0.99175100
	C	2.22854300	2.77873000	-1.80882200
	C	3.22708600	3.36770100	-2.57767200
	C	4.52244000	2.85974800	-2.51183900
	C	4.80927400	1.77240100	-1.69246900
	C	5.11331200	-1.06004100	-0.53311200
	C	5.18295600	-1.30768100	-1.90786600
	C	5.95734700	-2.35345900	-2.40686100
	C	6.66131500	-3.18480800	-1.54016900
	C	6.57413800	-2.96816900	-0.16702700
	C	5.80394100	-1.92094100	0.33081400
	H	6.83102400	0.58241900	0.58433400
	H	7.80458200	1.90485200	2.40352500
	H	6.34966600	2.85171800	4.18393800
	H	3.89785100	2.45828600	4.08674800
	H	2.92368200	1.14287200	2.26837400
	H	1.71815000	1.25656800	-0.40500400
	H	1.21483200	3.16174000	-1.84056300
	H	3.00168300	4.21448600	-3.21605300
	H	5.31614600	3.31300100	-3.09544700
	H	5.82617600	1.40321600	-1.64625600
	H	4.62525100	-0.68623400	-2.59520700
	H	6.00329000	-2.51888900	-3.47763300
	H	7.26443600	-3.99742900	-1.92933200
	H	7.10369800	-3.61653200	0.52216200
	H	5.73410400	-1.77286100	1.40127400
	H	0.80618200	-2.35536000	-2.53004900
	C	-0.10495200	-1.44288900	-0.04810100
	C	-1.31750200	-2.33968600	0.22541800
	H	-1.57071200	-2.91392900	-0.66691800
	C	-1.17343500	-3.24835800	1.43602700
	H	-2.10429100	-3.78874400	1.61770700
	H	-0.92869400	-2.66981800	2.32973000
	H	-0.38065600	-3.98269400	1.27553900
	O	-2.45988800	-1.47364900	0.51366400
	C	-3.15874900	-0.99149400	-0.52962200
	C	-4.21869700	0.04508000	-0.05880700
	C	-4.99131400	-0.43162400	1.19210300
	C	-5.56990700	0.48812300	2.07355100
	O	-2.91925100	-1.26724900	-1.67870200
	C	-5.25976800	-1.78959400	1.39491900
	C	-6.05684800	-2.21634600	2.45334100

	C	-6.61092100	-1.29089400	3.33350900
	C	-6.36602100	0.06489200	3.13493700
	C	-5.30398900	0.29542400	-1.13239400
	C	-5.73290400	-0.71089500	-2.00466100
	C	-6.79389600	-0.49372700	-2.88108300
	C	-7.45582000	0.73001100	-2.90182900
	C	-7.04738000	1.73610100	-2.03049800
	C	-5.98696800	1.51804000	-1.15590200
	C	-3.31244900	1.28383300	0.17849400
	C	-2.94710100	2.09682800	-0.90198600
	C	-2.05768900	3.15520000	-0.73245500
	C	-1.50146600	3.41323800	0.51814200
	C	-1.83386000	2.59292800	1.59350400
	C	-2.72293200	1.53411600	1.42338800
	H	-5.40279300	1.54847000	1.93291100
	H	-4.84930800	-2.53102100	0.72047200
	H	-6.24498100	-3.27584900	2.58740600
	H	-7.23012000	-1.62174700	4.15951200
	H	-6.79819500	0.80045600	3.80433500
	H	-5.23111000	-1.66719500	-2.02088100
	H	-7.09973600	-1.28912000	-3.55174600
	H	-8.28015300	0.89733800	-3.58587300
	H	-7.55436000	2.69473100	-2.02722500
	H	-5.68809200	2.31495500	-0.48646600
	H	-3.35782000	1.89967800	-1.88385700
	H	-1.80091100	3.77744300	-1.58265000
	H	-0.81176400	4.23895200	0.65171000
	H	-1.39812700	2.77240300	2.57009800
	H	-2.95226100	0.89436000	2.26481600
	H	-0.34771300	-0.76940900	-0.87412800
	H	0.07718700	-0.83630900	0.84267000
54	O	2.43878200	-1.44864700	-0.61056900
	C	3.10239100	-0.98369700	0.46373900
	C	1.31169300	-2.34913300	-0.37977700
	H	1.57650500	-2.98019400	0.47047200
	C	1.18203400	-3.17390800	-1.65108700
	H	2.12566600	-3.67423700	-1.87651100
	H	0.91522000	-2.53921100	-2.49907400
	O	2.82935300	-1.28697500	1.59810300
	C	4.18498800	0.05037800	0.03587300
	C	3.31072800	1.21758600	-0.49308600
	C	2.58757100	1.99361000	0.42359300
	C	1.71774000	2.98968500	-0.01009600
	C	1.54638600	3.22686300	-1.37219000
	C	2.24285000	2.44852600	-2.29169900
	C	3.10903300	1.44660300	-1.85673000
	C	5.16061900	-0.53174800	-1.01322700
	C	5.30916600	-1.90655300	-1.21655300
	C	6.27531000	-2.40548600	-2.08944500
	C	7.11501600	-1.53827200	-2.77947600
	C	6.98639300	-0.16539000	-2.57824800
	C	6.02688700	0.32847600	-1.70105400

	C	5.07183200	0.46030200	1.23626600
	C	5.52777200	1.77475800	1.38155000
	C	6.41800000	2.12064300	2.39698100
	C	6.87416200	1.15613600	3.28899800
	C	6.43702300	-0.15902600	3.14831300
	C	5.55266300	-0.50365900	2.13137700
	H	2.70492600	1.81322500	1.48555700
	H	1.16753000	3.57529700	0.71746900
	H	0.86886900	4.00224900	-1.71105500
	H	2.10776700	2.61116300	-3.35531200
	H	3.62067000	0.83534900	-2.58753900
	H	4.67184800	-2.60827000	-0.69454500
	H	6.36731600	-3.47727100	-2.22651200
	H	7.86467000	-1.92527700	-3.46023200
	H	7.63996900	0.52526500	-3.09951100
	H	5.95696900	1.39777000	-1.54450400
	H	5.18589400	2.54411600	0.70130500
	H	6.75206700	3.14853400	2.48618200
	H	7.56343700	1.42351600	4.08206900
	H	6.78566400	-0.92438000	3.83290700
	H	5.22891100	-1.53122000	2.04513400
	H	0.41072100	-3.93947400	-1.53913200
	C	0.08026500	-1.49950100	-0.04384300
	C	-1.18801100	-2.31082100	0.24338300
	H	-1.47983100	-2.87609300	-0.64308100
	C	-1.09902200	-3.21790500	1.46117600
	H	-0.78849300	-2.65637000	2.34542500
	H	-0.37482900	-4.01887900	1.29389100
	H	-2.07003100	-3.67329100	1.66293400
	O	-2.28280600	-1.38244500	0.52771100
	C	-2.95748400	-0.87059400	-0.52044200
	C	-4.14996300	0.03538500	-0.05426500
	C	-3.58533300	1.37586600	0.50719800
	C	-4.17053100	2.60074400	0.16306500
	O	-2.67042700	-1.11665400	-1.66381300
	C	-2.53246900	1.39565700	1.43406200
	C	-2.07090500	2.59310400	1.97381000
	C	-2.65576400	3.80424300	1.61251900
	C	-3.71158300	3.79946400	0.70765100
	C	-4.92152200	-0.74021800	1.04035000
	C	-5.24239800	-2.08846000	0.82803600
	C	-5.97214000	-2.80998400	1.76524500
	C	-6.40523200	-2.19502200	2.94008300
	C	-6.10244800	-0.85603000	3.15769600
	C	-5.36636900	-0.13474400	2.21574000
	C	-5.06378800	0.30806900	-1.27485000
	C	-6.44595200	0.10985900	-1.21888900
	C	-7.26297600	0.40654500	-2.31152700
	C	-6.71280100	0.91042600	-3.48329800
	C	-5.33651700	1.12457200	-3.54987900
	C	-4.52459500	0.83191600	-2.46051300
	H	-4.99686700	2.62779200	-0.53290300

	H	-2.07925200	0.46781500	1.74882000
	H	-1.25366300	2.57322300	2.68639400
	H	-2.29796000	4.73613600	2.03596600
	H	-4.18864800	4.72994300	0.42016500
	H	-4.93014200	-2.57671700	-0.08940700
	H	-6.20539600	-3.85235300	1.57782500
	H	-6.97311500	-2.75568900	3.67395600
	H	-6.43536000	-0.36300700	4.06436400
	H	-5.14047800	0.90610900	2.40585900
	H	-6.90255400	-0.27699800	-0.31836200
	H	-8.33197400	0.24014500	-2.23695600
	H	-7.34514300	1.13778700	-4.33435500
	H	-4.89065300	1.52173400	-4.45516500
	H	-3.46171700	1.01131100	-2.53466000
	H	-0.11209400	-0.82687400	-0.88283900
	H	0.30807700	-0.89049300	0.83431000

21 (optimized at the B3LYP-GD3BJ/6-311G(d,p) level)

Conformer no				
1	O	-1.60974500	-1.72530100	-0.20882400
	C	-2.12580700	-0.70336500	-0.91331200
	C	-0.69419200	-2.62749900	-0.89928000
	H	0.00431100	-2.00838900	-1.46087500
	C	-1.45953500	-3.55071300	-1.83546700
	H	-0.76375900	-4.23905000	-2.32186700
	H	-1.97237700	-2.97774700	-2.60610900
	O	-1.87887800	-0.52563300	-2.07907800
	C	-3.09538100	0.14655400	-0.03934600
	C	-2.54324000	0.21577900	1.39444000
	C	-1.21029400	0.59771800	1.58623200
	C	-0.68526900	0.73948400	2.86388000
	C	-1.49180100	0.50650800	3.97803800
	C	-2.82016000	0.13873400	3.79810300
	C	-3.34382400	-0.00532200	2.51236600
	C	-4.46242300	-0.58567400	-0.07924400
	C	-4.54454200	-1.95240200	0.21928800
	C	-5.76931800	-2.61027700	0.22706400
	C	-6.94366000	-1.91600500	-0.05442700
	C	-6.87595800	-0.55663300	-0.33626500
	C	-5.64741400	0.10199000	-0.34897800
	C	-3.23289400	1.57167800	-0.61378400
	C	-3.21619700	2.68304400	0.23222100
	C	-3.40257000	3.97112200	-0.26615000
	C	-3.60606000	4.17479200	-1.62593500
	C	-3.64069300	3.07299400	-2.47855200
	C	-3.46901700	1.78687700	-1.97818800
	H	-0.57993900	0.79951100	0.72919500
	H	0.35578300	1.00809300	2.98290800
	H	-1.08335400	0.61167500	4.97652600
	H	-3.45789000	-0.03907300	4.65668200
	H	-4.38060900	-0.28674700	2.38760600

	H	-3.64501900	-2.49734000	0.46533200
	H	-5.80520600	-3.66854400	0.45975700
	H	-7.89856800	-2.42856700	-0.04842700
	H	-7.78045800	0.00192500	-0.54817600
	H	-5.61914100	1.15986500	-0.56621400
	H	-3.05421100	2.55044500	1.29180200
	H	-3.38237600	4.81345900	0.41581200
	H	-3.74414400	5.17605200	-2.01765100
	H	-3.81097200	3.21218400	-3.54011200
	H	-3.51339000	0.94573400	-2.65167400
	H	-2.19410300	-4.13944000	-1.27978000
	C	0.03625800	-3.38744500	0.19700600
	C	0.79060200	-2.52553100	1.19927100
	H	0.11498200	-1.82690000	1.69264500
	C	1.53268400	-3.37039800	2.22493600
	H	2.04502800	-2.73940600	2.94839300
	H	2.25987300	-4.01994700	1.72974600
	H	0.81998600	-4.00124000	2.76209400
	O	1.72894500	-1.73404500	0.40453700
	C	2.33626000	-0.69903300	1.00169400
	C	3.15219800	0.14922400	-0.00053000
	C	2.07827800	1.17015300	-0.44092900
	C	1.19184400	0.88111900	-1.48278300
	O	2.18426600	-0.41008200	2.16312900
	C	1.88390700	2.34491200	0.29290800
	C	0.86490600	3.23157100	-0.04345100
	C	0.01715200	2.95672500	-1.11304200
	C	0.18072000	1.77384900	-1.82684400
	C	3.74820600	-0.68100700	-1.14989100
	C	4.28279800	-1.94768700	-0.89671200
	C	4.93128400	-2.66462900	-1.89541100
	C	5.06902400	-2.12331000	-3.17154300
	C	4.56194500	-0.85420400	-3.42946700
	C	3.91288100	-0.13893900	-2.42607400
	C	4.36245000	0.82138600	0.67231300
	C	4.98070100	0.29288100	1.80639100
	C	6.14359800	0.86713600	2.31563900
	C	6.71073900	1.97702300	1.69954200
	C	6.10673800	2.50642300	0.56128900
	C	4.94872500	1.93026300	0.05301300
	H	1.28966800	-0.04436600	-2.03288400
	H	2.53128100	2.56225900	1.13152300
	H	0.72838300	4.13423000	0.54067800
	H	-0.78333000	3.63788900	-1.37038700
	H	-0.50188400	1.52232000	-2.62615400
	H	4.19728100	-2.38052700	0.09154700
	H	5.33281300	-3.64710000	-1.67467500
	H	5.57286800	-2.68163900	-3.95184100
	H	4.67222500	-0.41287200	-4.41336900
	H	3.53274500	0.84991300	-2.64430200
	H	4.55197200	-0.55869800	2.31353200
	H	6.60260200	0.44278500	3.20129400

	H	7.61341900	2.42451000	2.09893600
	H	6.53852300	3.36811400	0.06526200
	H	4.49284500	2.34868400	-0.83519000
	H	0.74024000	-4.07501500	-0.28236200
	H	-0.68469600	-3.99575300	0.75249200
6	O	-1.74761800	1.74055800	0.35475500
	C	-2.24157000	0.64187800	0.94285600
	C	-0.80086300	2.55123900	1.11493700
	H	-0.12688500	1.87321300	1.63703900
	C	-1.55215800	3.41316800	2.11726200
	H	-2.07611100	2.78939400	2.84083700
	H	-2.27412300	4.05346300	1.60400000
	O	-1.92546500	0.29015900	2.05476100
	C	-3.17214300	-0.16307800	0.00360600
	C	-3.65022600	0.69083600	-1.18650800
	C	-3.54863300	0.27306700	-2.51167500
	C	-4.07097600	1.05020400	-3.54726300
	C	-4.70764200	2.25311300	-3.27111000
	C	-4.83252500	2.67034600	-1.94541300
	C	-4.31620900	1.89317200	-0.91899500
	C	-4.44725000	-0.65272200	0.72392000
	C	-4.71225500	-0.47140900	2.08131900
	C	-5.90689700	-0.93040400	2.63927800
	C	-6.84886500	-1.58548600	1.85696200
	C	-6.59070800	-1.77633600	0.50012100
	C	-5.40839200	-1.30937100	-0.05715300
	C	-2.26616100	-1.34640100	-0.40754100
	C	-2.48081700	-2.63796200	0.07505000
	C	-1.60802000	-3.67180400	-0.25626100
	C	-0.50412300	-3.43049800	-1.06663500
	C	-0.27262600	-2.14270400	-1.53855300
	C	-1.14132900	-1.11041600	-1.20488300
	H	-3.06529600	-0.66473800	-2.74737000
	H	-3.97798100	0.70495900	-4.57058900
	H	-5.11173500	2.85733400	-4.07500800
	H	-5.34058200	3.59938500	-1.71295300
	H	-4.43810400	2.21755800	0.10757400
	H	-3.98337000	0.00558500	2.71740400
	H	-6.09098800	-0.77451600	3.69622700
	H	-7.77385500	-1.94280200	2.29446500
	H	-7.31471400	-2.28365400	-0.12712000
	H	-5.22692600	-1.45070400	-1.11493800
	H	-3.32670600	-2.83899500	0.71719800
	H	-1.78890800	-4.66658200	0.13438200
	H	0.18377500	-4.23141200	-1.30871900
	H	0.59123100	-1.92138300	-2.15002900
	H	-0.93380900	-0.11431800	-1.57067300
	H	-0.84835400	4.04931400	2.65953000
	C	-0.04195800	3.37234700	0.08378900
	C	0.67753500	2.55469300	-0.97764900
	H	-0.02363600	1.89560100	-1.48745200
	C	1.41282600	3.42321300	-1.98686400

	H	0.70152600	4.07009200	-2.50614400
	H	1.91614200	2.80434300	-2.72893600
	H	2.15007900	4.05256600	-1.48195300
	O	1.63412500	1.71921800	-0.25816500
	C	2.13139400	0.64893500	-0.90151500
	C	3.16400400	-0.12586100	-0.02794700
	C	3.79528300	0.83485900	0.99374700
	C	3.86717800	0.53476300	2.35121700
	O	1.77278400	0.32917000	-2.00737400
	C	4.37234500	2.02943300	0.54375100
	C	4.99381300	2.90157700	1.42593400
	C	5.05431300	2.59486600	2.78625500
	C	4.49152500	1.40981600	3.24233700
	C	4.25267300	-0.74156900	-0.93558500
	C	3.89984100	-1.53837800	-2.03367500
	C	4.87200300	-2.14952100	-2.81601100
	C	6.22329000	-1.99450600	-2.51338100
	C	6.58577700	-1.22664300	-1.41402800
	C	5.60981900	-0.60879500	-0.63240100
	C	2.37504600	-1.24807100	0.68878100
	C	2.90601300	-2.53124000	0.82893500
	C	2.21228200	-3.51241000	1.53521900
	C	0.97841000	-3.22735900	2.10972200
	C	0.44118000	-1.94996100	1.98030600
	C	1.13781500	-0.97409800	1.27884800
	H	3.43585900	-0.38465000	2.72191800
	H	4.34196900	2.27158000	-0.51225300
	H	5.43377700	3.82019800	1.05460500
	H	5.53802700	3.27432500	3.47844200
	H	4.53421100	1.15666500	4.29549000
	H	2.85914500	-1.68110200	-2.27511800
	H	4.56996200	-2.75525200	-3.66288700
	H	6.98074500	-2.47340300	-3.12317600
	H	7.63097100	-1.10431800	-1.15375200
	H	5.91846200	-0.02677000	0.22372200
	H	3.86515600	-2.76998700	0.39122800
	H	2.64323800	-4.50262300	1.63163400
	H	0.43448400	-3.99515500	2.64745400
	H	-0.52466700	-1.70348300	2.40046800
	H	0.71071800	0.01340700	1.19649000
	H	0.68678200	3.99426400	0.61245100
	H	-0.74223100	4.04264800	-0.42393900
16	O	-1.26014200	-1.31678500	0.32206900
	C	-2.40103900	-0.96947400	0.92943600
	C	-0.58991800	-2.57637600	0.65903500
	H	0.26873800	-2.53714900	-0.00705800
	C	-1.46527800	-3.76577900	0.29789100
	H	-0.88372500	-4.68465800	0.40825600
	H	-1.79308600	-3.69269800	-0.74008800
	O	-2.86355300	-1.54080200	1.88724200
	C	-3.10671800	0.16794200	0.14747300
	C	-2.08907000	1.10406400	-0.53143300

	C	-2.17876400	1.48581500	-1.86938100
	C	-1.28844300	2.41845900	-2.40484700
	C	-0.30253800	2.98936800	-1.60830700
	C	-0.21368300	2.62389900	-0.26553000
	C	-1.10126500	1.69593600	0.26246600
	C	-3.97691000	1.08139500	1.02925900
	C	-3.99976700	1.04532600	2.42318600
	C	-4.76572300	1.96323900	3.14370400
	C	-5.52141900	2.92609700	2.48737300
	C	-5.50164000	2.97274600	1.09384500
	C	-4.73282300	2.06585500	0.37814300
	C	-3.95875400	-0.66685900	-0.83719200
	C	-5.32744200	-0.85305000	-0.63563000
	C	-6.06568700	-1.67654200	-1.48314200
	C	-5.44608500	-2.33871600	-2.53769300
	C	-4.07516000	-2.18154600	-2.73097500
	C	-3.33898400	-1.36090900	-1.88410800
	H	-2.94646000	1.06374400	-2.50349800
	H	-1.37333000	2.69596100	-3.44954400
	H	0.40052800	3.70083400	-2.02271400
	H	0.56158400	3.03803000	0.36444900
	H	-1.01984700	1.43267400	1.30964000
	H	-3.44203000	0.29033800	2.95388700
	H	-4.76801700	1.91563200	4.22677900
	H	-6.11632400	3.63558400	3.05080000
	H	-6.07878300	3.72223400	0.56447700
	H	-4.71039000	2.12414800	-0.70298700
	H	-5.82033700	-0.35591200	0.18795000
	H	-7.12833100	-1.80254600	-1.31065900
	H	-6.02180600	-2.97812500	-3.19673900
	H	-3.57498100	-2.70344700	-3.53900700
	H	-2.27165400	-1.26585200	-2.02167800
	H	-2.34263400	-3.82570400	0.94161900
	C	-0.08528400	-2.60100900	2.10140300
	C	0.65853000	-1.33734800	2.50230700
	H	-0.00456900	-0.47438700	2.45691400
	C	1.30275000	-1.41747600	3.87593700
	H	1.80700000	-0.48002500	4.11482100
	H	2.02704600	-2.23518800	3.91374900
	H	0.53827400	-1.59836200	4.63548500
	O	1.68552400	-1.14300400	1.48599700
	C	2.04134800	0.11534000	1.18666100
	C	3.01393300	0.12073800	-0.02582000
	C	3.19392700	1.55409400	-0.56356500
	C	3.14292000	1.84455400	-1.92742900
	O	1.64305200	1.07781200	1.79140200
	C	3.52816400	2.58751300	0.32043000
	C	3.74618000	3.87912100	-0.14226300
	C	3.66340500	4.16418500	-1.50436900
	C	3.37437200	3.13750700	-2.39482800
	C	2.38550100	-0.83094000	-1.06412400
	C	2.92605400	-2.08524200	-1.34818500

	C 2.29596600 -2.94441200 -2.24745700<br>C 1.11813500 -2.55917700 -2.88072900<br>C 0.56658000 -1.31237500 -2.59376200<br>C 1.18584300 -0.46328200 -1.68445500<br>C 4.40864600 -0.35813200 0.42858900<br>C 5.45926700 -0.30482800 -0.49566400<br>C 6.73965100 -0.71301300 -0.14680400<br>C 7.00329500 -1.17027100 1.14395600<br>C 5.97144100 -1.21513900 2.07292600<br>C 4.68280400 -0.81623000 1.71665400<br>H 2.91617600 1.06361000 -2.63884600<br>H 3.60846700 2.38292300 1.37762100<br>H 3.98871900 4.66489600 0.56392900<br>H 3.83517800 5.17191100 -1.86462800<br>H 3.32393400 3.33547500 -3.45946500<br>H 3.83778500 -2.40105600 -0.86047200<br>H 2.73222400 -3.91534300 -2.45277000<br>H 0.63366300 -3.22326200 -3.58727400<br>H -0.34957700 -0.99236600 -3.07464400<br>H 0.72891600 0.48887100 -1.45635400<br>H 5.26869600 0.06623900 -1.49468500<br>H 7.53564500 -0.66596400 -0.88101500<br>H 8.00364600 -1.48219200 1.42036000<br>H 6.16126400 -1.56236100 3.08209100<br>H 3.89750000 -0.87587600 2.45640500<br>H 0.58971500 -3.45907700 2.19137300<br>H -0.91577600 -2.74973000 2.79193800

21 (optimized at the M06-2X/6-311G(d,p) level)

Conformer no	
1	O -1.64975900 -1.72188000 -0.13033200<br>C -2.07249400 -0.63366000 -0.78654900<br>C -0.73517800 -2.60221100 -0.83010500<br>H -0.02674400 -1.98257300 -1.38308800<br>C -1.50898100 -3.48946800 -1.78896100<br>H -0.81646700 -4.12679900 -2.34210200<br>H -2.06398400 -2.88257800 -2.50489900<br>O -1.70806700 -0.36232100 -1.89798400<br>C -3.09635600 0.19328900 0.03948400<br>C -2.72614800 0.12324300 1.52705100<br>C -1.40235000 0.36134400 1.91192900<br>C -1.03054600 0.33670800 3.24822300<br>C -1.98523200 0.08110600 4.23031400<br>C -3.30230600 -0.14772700 3.86055400<br>C -3.67140800 -0.13033800 2.51639500<br>C -4.49263500 -0.41949700 -0.20375600<br>C -4.67341800 -1.79766700 -0.33242200<br>C -5.94584300 -2.33816700 -0.48673900<br>C -7.06320200 -1.51346500 -0.49817900<br>C -6.89637300 -0.14164700 -0.35066300<br>C -5.62412100 0.40026400 -0.20863300

	C	-3.05455900	1.63771300	-0.49102600
	C	-2.69981800	2.72315700	0.30506200
	C	-2.65027000	4.01071100	-0.22922200
	C	-2.96079400	4.23041700	-1.56205900
	C	-3.34352000	3.15398900	-2.36086400
	C	-3.39615000	1.87525700	-1.82880400
	H	-0.64659700	0.57955900	1.15991100
	H	0.00735300	0.50381500	3.50715900
	H	-1.69926500	0.06040100	5.27545500
	H	-4.05348300	-0.34698800	4.61595400
	H	-4.70237900	-0.32422600	2.24847700
	H	-3.82074000	-2.46196200	-0.29141600
	H	-6.06015700	-3.41063800	-0.59299700
	H	-8.05407500	-1.93526300	-0.61726500
	H	-7.75863300	0.51463900	-0.34880400
	H	-5.51097900	1.47162600	-0.09597400
	H	-2.45450000	2.57907600	1.34854200
	H	-2.36192500	4.83873600	0.40745500
	H	-2.91745200	5.23071400	-1.97704800
	H	-3.60167800	3.31257000	-3.40121000
	H	-3.69584500	1.04646500	-2.45694800
	H	-2.20520200	-4.12957200	-1.24083400
	C	-0.01054400	-3.38567600	0.25052300
	C	0.74280100	-2.50415300	1.23223700
	H	0.05882700	-1.80146000	1.71080700
	C	1.50310000	-3.30280000	2.27550400
	H	2.04442900	-2.63500500	2.94563800
	H	2.21127700	-3.97876900	1.78991600
	H	0.80529700	-3.89576200	2.86994900
	O	1.67965300	-1.74538600	0.42849700
	C	2.17522200	-0.62292100	0.95298300
	C	3.14012100	0.11722400	-0.01111500
	C	2.33514600	1.35251900	-0.46560000
	C	1.05596700	1.15930800	-0.99981800
	O	1.84259700	-0.20857400	2.03227300
	C	2.84270500	2.64594400	-0.39830100
	C	2.08095400	3.72410600	-0.84848400
	C	0.81122200	3.52289600	-1.36921600
	C	0.29746800	2.23032500	-1.45022100
	C	3.50540600	-0.78910800	-1.19717900
	C	4.13572400	-2.01069300	-0.93175200
	C	4.53892900	-2.84581200	-1.95945700
	C	4.32636300	-2.47241400	-3.28634000
	C	3.71522600	-1.25998500	-3.56146900
	C	3.30843600	-0.42016900	-2.52267900
	C	4.43063000	0.55102800	0.72056600
	C	4.41726800	1.03224900	2.03392400
	C	5.58392100	1.49256000	2.63427600
	C	6.78473000	1.50356200	1.93551600
	C	6.80524700	1.05138000	0.62303800
	C	5.64203200	0.58053800	0.02315600
	H	0.65916200	0.15343400	-1.09848400

	H	3.83127400	2.82422000	0.00547100
	H	2.49095800	4.72537100	-0.78473900
	H	0.21575600	4.36105300	-1.71174100
	H	-0.68637900	2.05212400	-1.86914700
	H	4.31808600	-2.29992200	0.09827400
	H	5.02511000	-3.78677500	-1.73016900
	H	4.64190000	-3.12230100	-4.09383700
	H	3.55174600	-0.95275700	-4.58759900
	H	2.84187200	0.52723100	-2.76033900
	H	3.49052500	1.05770100	2.58693500
	H	5.54615300	1.85257000	3.65581300
	H	7.69113800	1.86606900	2.40568500
	H	7.72868700	1.06087400	0.05609300
	H	5.68599100	0.23778100	-1.00251800
	H	0.69230000	-4.07157800	-0.23345800
	H	-0.73499700	-3.98537000	0.81071100
12	O	1.98664500	-1.45284800	1.23923100
	C	1.81954000	-0.23784400	0.71541600
	C	0.87937200	-2.08412500	1.92948900
	H	0.17079900	-1.31018400	2.22710100
	C	1.47278400	-2.77496300	3.14153200
	H	0.68557000	-3.27623300	3.70740700
	H	1.96285300	-2.05095000	3.79406300
	O	0.82471000	0.42450500	0.85126900
	C	3.00677900	0.14039000	-0.19404800
	C	4.36082200	-0.18615800	0.44529000
	C	5.48480900	-0.36461400	-0.36192400
	C	6.74129600	-0.55436000	0.19918800
	C	6.89738800	-0.55815600	1.58084400
	C	5.78752300	-0.36308800	2.39292000
	C	4.53050400	-0.17639600	1.82877600
	C	3.03375200	1.64422100	-0.49526800
	C	2.69313200	2.58078600	0.48093100
	C	2.82196400	3.94246800	0.23362200
	C	3.30376200	4.39203600	-0.99023700
	C	3.66555300	3.46652600	-1.96122100
	C	3.53412700	2.10536000	-1.71364500
	C	2.66692600	-0.72312000	-1.42658300
	C	1.69792900	-0.27719100	-2.32609100
	C	1.23787000	-1.10762800	-3.34222100
	C	1.73533800	-2.39816700	-3.46805000
	C	2.68739300	-2.85898700	-2.56226300
	C	3.14361100	-2.03144900	-1.54345200
	H	5.37518600	-0.35625400	-1.44070700
	H	7.60002800	-0.69512400	-0.44636900
	H	7.87670200	-0.70578300	2.02014800
	H	5.89701600	-0.35518800	3.47089800
	H	3.67790900	-0.02393500	2.48079500
	H	2.31649100	2.25161600	1.44041100
	H	2.54319400	4.65241100	1.00333100
	H	3.40124600	5.45364100	-1.18321100
	H	4.05013800	3.80215100	-2.91703000

	H	3.81681600	1.39565800	-2.48229400
	H	1.26174400	0.70737100	-2.20621700
	H	0.46601100	-0.74739000	-4.01085500
	H	1.37445900	-3.04813200	-4.25657600
	H	3.07211100	-3.86898500	-2.64364100
	H	3.85861100	-2.40937700	-0.82284200
	H	2.21159200	-3.51690300	2.83065300
	C	0.20914800	-3.05775300	0.96754100
	C	-0.43438000	-2.38663200	-0.23347800
	H	0.29424700	-1.77830600	-0.77082800
	C	-1.07838600	-3.36654000	-1.19740600
	H	-0.31872700	-4.03057900	-1.61547900
	H	-1.55208400	-2.82474400	-2.01854700
	H	-1.83598800	-3.96395100	-0.68396300
	O	-1.45593000	-1.50991000	0.29445300
	C	-1.76874400	-0.44439900	-0.45088600
	C	-3.02218700	0.28737500	0.07175500
	C	-3.20290300	0.18898500	1.59238300
	C	-4.46264000	0.06845700	2.17576900
	O	-1.22320200	-0.16504700	-1.48232000
	C	-2.09629300	0.37212500	2.42374100
	C	-2.24134100	0.38757600	3.80411700
	C	-3.49952300	0.23827700	4.38049900
	C	-4.60926100	0.08867500	3.56017900
	C	-4.09836700	-0.46827100	-0.73416100
	C	-4.53345900	-1.72445900	-0.30135900
	C	-5.41029400	-2.47750800	-1.07218600
	C	-5.85380300	-1.99580600	-2.29894000
	C	-5.39962200	-0.76362500	-2.75288300
	C	-4.52078700	-0.00980400	-1.98212500
	C	-2.95066500	1.78784300	-0.23646100
	C	-1.73049900	2.45624100	-0.33967200
	C	-1.69744100	3.83769900	-0.50428100
	C	-2.87622600	4.57021000	-0.56079900
	C	-4.09572800	3.91268000	-0.43739100
	C	-4.12998300	2.53452000	-0.26960900
	H	-5.34093900	-0.04173600	1.55066300
	H	-1.11614800	0.51534200	1.98228100
	H	-1.36883700	0.52725400	4.43237100
	H	-3.61374100	0.25100700	5.45789200
	H	-5.59752100	-0.01086600	3.99387300
	H	-4.17562200	-2.11457500	0.64439400
	H	-5.74322800	-3.44474500	-0.71438000
	H	-6.53898500	-2.58133400	-2.90035800
	H	-5.72278900	-0.38672600	-3.71606600
	H	-4.15229600	0.93628500	-2.35844900
	H	-0.79685700	1.90938900	-0.28840000
	H	-0.73868700	4.33633400	-0.58895400
	H	-2.84717100	5.64529800	-0.69307200
	H	-5.02284600	4.47305600	-0.46566800
	H	-5.08503800	2.03111200	-0.16182200
	H	-0.55478300	-3.61784500	1.51722300

	H	0.95903100	-3.77015000	0.60703500
15	O	1.64875600	-1.71209600	0.14603700
	C	1.70646800	-0.41435300	0.46269200
	C	0.82456400	-2.58573900	0.95197800
	H	0.15797200	-1.96913900	1.55349900
	C	1.75441400	-3.38846500	1.84250900
	H	1.17624200	-4.06365300	2.47715500
	H	2.33798800	-2.72076300	2.48079800
	O	0.94071800	0.13527600	1.20839000
	C	2.98389800	0.23285800	-0.11043600
	C	2.86772400	1.76021400	-0.14720800
	C	3.97710900	2.57134200	0.08659100
	C	3.89604200	3.95027500	-0.07931300
	C	2.70726200	4.53598000	-0.49500400
	C	1.60245200	3.73052700	-0.75256300
	C	1.68085100	2.35408000	-0.58183500
	C	3.26785500	-0.18867000	-1.55605800
	C	2.21380400	-0.42288300	-2.43881000
	C	2.46084200	-0.69593800	-3.77902600
	C	3.76462800	-0.72718000	-4.26030700
	C	4.81838200	-0.46207200	-3.39357500
	C	4.57066900	-0.18753600	-2.05348900
	C	3.99931000	-0.30075700	0.92200000
	C	4.68420900	-1.49923200	0.71231700
	C	5.49071500	-2.03915300	1.70794300
	C	5.60910400	-1.40210400	2.93734200
	C	4.90261200	-0.22680400	3.16870700
	C	4.09829200	0.31436400	2.17267700
	H	4.91550300	2.12794600	0.39928100
	H	4.76844800	4.56372100	0.11313000
	H	2.64470600	5.60950300	-0.62848300
	H	0.67172700	4.16792300	-1.09525300
	H	0.81271700	1.74425200	-0.80903700
	H	1.18933500	-0.36262100	-2.08725000
	H	1.62848100	-0.87518200	-4.44947700
	H	3.95754400	-0.94113600	-5.30481400
	H	5.83796000	-0.46090000	-3.76053600
	H	5.39927100	0.03346000	-1.39001900
	H	4.57410500	-2.02240100	-0.22922000
	H	6.01987400	-2.96630300	1.52171800
	H	6.23850100	-1.82280300	3.71242600
	H	4.97098800	0.26933600	4.12958600
	H	3.52934900	1.21577000	2.36889400
	H	2.44682600	-3.97707700	1.23619600
	C	-0.00011000	-3.43518500	-0.00071700
	C	-0.82477900	-2.58544800	-0.95316600
	H	-0.15821600	-1.96858000	-1.55445800
	C	-1.75448800	-3.38796900	-1.84403800
	H	-2.44692900	-3.97680400	-1.23797900
	H	-1.17621200	-4.06292100	-2.47884300
	H	-2.33802900	-2.72011100	-2.48219300
	O	-1.64913400	-1.71215700	-0.14707600

	C	-1.70648900	-0.41417900	-0.46288100
	C	-2.98387800	0.23283900	0.11052800
	C	-3.26794800	-0.18959000	1.55586800
	C	-4.57075500	-0.18888400	2.05327400
	O	-0.94057300	0.13577700	-1.20815600
	C	-2.21389200	-0.42433300	2.43850200
	C	-2.46092100	-0.69832700	3.77851500
	C	-3.76472500	-0.72999600	4.25975200
	C	-4.81846300	-0.46435900	3.39318200
	C	-3.99929100	-0.29996700	-0.92231200
	C	-4.09815400	0.31600100	-2.17256100
	C	-4.90253400	-0.22438300	-3.16898700
	C	-5.60920100	-1.39971600	-2.93842900
	C	-5.49091600	-2.03762900	-1.70944600
	C	-4.68434400	-1.49851300	-0.71345300
	C	-2.86753400	1.76016100	0.14826300
	C	-3.97686800	2.57150700	-0.08515800
	C	-3.89574500	3.95030700	0.08160900
	C	-2.70694900	4.53570500	0.49777000
	C	-1.60222800	3.73004600	0.75492500
	C	-1.68068500	2.35368000	0.58334200
	H	-5.39938100	0.03250300	1.38996400
	H	-1.18941000	-0.36371300	2.08703500
	H	-1.62855200	-0.87794600	4.44885500
	H	-3.95766500	-0.94468300	5.30410500
	H	-5.83805300	-0.46351500	3.76011700
	H	-3.52909900	1.21747100	-2.36815900
	H	-4.97079200	0.27243500	-4.12952300
	H	-6.23867400	-1.81981800	-3.71377400
	H	-6.02021200	-2.96483100	-1.52387600
	H	-4.57428100	-2.02235000	0.22771700
	H	-4.91523600	2.12833300	-0.39824200
	H	-4.76808800	4.56393800	-0.11053300
	H	-2.64437500	5.60914400	0.63191400
	H	-0.67148900	4.16715100	1.09795200
	H	-0.81259100	1.74370900	0.81031400
	H	-0.66126800	-4.07853000	0.58893400
	H	0.66104100	-4.07834500	-0.59057100
16	O	1.31187900	-1.33617300	-0.25996400
	C	2.36819200	-0.88565600	-0.94380700
	C	0.73770400	-2.62578100	-0.60286700
	H	-0.08350500	-2.69270200	0.11195300
	C	1.74728300	-3.73087000	-0.34118700
	H	1.26494000	-4.70141700	-0.47664300
	H	2.11919000	-3.66724300	0.68310200
	O	2.73965200	-1.34325800	-1.99097400
	C	3.12377900	0.18741900	-0.13319800
	C	2.15363000	1.12244200	0.60002600
	C	2.40294800	1.60563800	1.88215600
	C	1.54571500	2.53599000	2.46608700
	C	0.43828900	3.00406900	1.77254000
	C	0.19448100	2.54751000	0.47970700

	C	1.05032500	1.61993200	-0.09810000
	C	3.99709800	1.10019700	-1.00205400
	C	3.74431200	1.34025800	-2.35101200
	C	4.50467300	2.26948700	-3.05656300
	C	5.52191800	2.97327700	-2.42784200
	C	5.77284700	2.74875700	-1.07755600
	C	5.01390600	1.82519500	-0.37423400
	C	3.96200800	-0.74001900	0.77134100
	C	5.20847400	-1.19454400	0.33581200
	C	5.91928800	-2.13352400	1.07423000
	C	5.39212300	-2.64317400	2.25475500
	C	4.13954500	-2.21790500	2.68314000
	C	3.42598600	-1.28434100	1.94062600
	H	3.27102800	1.26361500	2.43328100
	H	1.75310900	2.89587100	3.46717200
	H	-0.23336800	3.72329200	2.22524200
	H	-0.66460800	2.90393000	-0.07797200
	H	0.85000300	1.28559500	-1.11156600
	H	2.96411000	0.79734000	-2.86573300
	H	4.29507200	2.43954800	-4.10601300
	H	6.11180900	3.69381000	-2.98149400
	H	6.55781300	3.29657900	-0.56960300
	H	5.21035200	1.66265200	0.68022100
	H	5.62014400	-0.81813900	-0.59293200
	H	6.88592000	-2.47097900	0.71969200
	H	5.94821000	-3.37263600	2.83133000
	H	3.71055600	-2.61947000	3.59387900
	H	2.43794000	-0.98076500	2.26214700
	H	2.59120400	-3.66128300	-1.02901700
	C	0.15203100	-2.66150600	-2.01353800
	C	-0.60764200	-1.39827700	-2.37285200
	H	0.07093400	-0.54354900	-2.39197900
	C	-1.34820800	-1.49876000	-3.69401900
	H	-1.88053600	-0.57063400	-3.90342200
	H	-2.06409900	-2.32365700	-3.66566500
	H	-0.63733900	-1.68214300	-4.50209200
	O	-1.55366900	-1.18333800	-1.29900400
	C	-2.00081700	0.06406600	-1.11758800
	C	-3.05764200	0.13535200	0.02011400
	C	-3.16694200	1.59513200	0.48778000
	C	-3.01781600	1.95611800	1.82449400
	O	-1.62977500	0.99158300	-1.78356800
	C	-3.45774100	2.60143600	-0.44316300
	C	-3.55659100	3.92764100	-0.05158700
	C	-3.38469600	4.28007700	1.28566600
	C	-3.12765500	3.28868600	2.21989400
	C	-2.60645500	-0.77142800	1.17337000
	C	-3.50315300	-1.57137900	1.87520000
	C	-3.07536400	-2.32181100	2.96906400
	C	-1.74847200	-2.27950200	3.37319500
	C	-0.84677800	-1.47691200	2.67813300
	C	-1.27251500	-0.72741500	1.59074200

	C	-4.40183900	-0.35870000	-0.56906200
	C	-5.58957500	0.34213000	-0.35685500
	C	-6.80274800	-0.14669400	-0.83496000
	C	-6.85300300	-1.34517200	-1.53254800
	C	-5.67836700	-2.06148000	-1.73420200
	C	-4.46877800	-1.57850000	-1.25231300
	H	-2.80856300	1.20350800	2.57349300
	H	-3.60260600	2.34220700	-1.48321700
	H	-3.76905800	4.68939800	-0.79240300
	H	-3.46118600	5.31654600	1.59250500
	H	-3.00415600	3.54330000	3.26616700
	H	-4.54271600	-1.61417400	1.57314000
	H	-3.78722000	-2.93996100	3.50318400
	H	-1.41807400	-2.86520900	4.22295100
	H	0.19198400	-1.42903900	2.98403200
	H	-0.55490300	-0.10511900	1.06357900
	H	-5.57922800	1.27367300	0.19320500
	H	-7.71026500	0.41717400	-0.65425500
	H	-7.79680600	-1.72239900	-1.90806300
	H	-5.70185900	-3.00646800	-2.26415500
	H	-3.57035200	-2.16518100	-1.38654600
	H	-0.53026300	-3.51767900	-2.05796200
	H	0.93916200	-2.81344200	-2.75325500
25	O	1.73683100	-1.66296900	-0.23261000
	C	2.30395300	-0.91181800	0.71656800
	C	0.53564700	-2.40310000	0.10303800
	H	-0.07339200	-1.76400700	0.74958300
	C	0.88481600	-3.69007900	0.82649300
	H	-0.03173300	-4.23588000	1.06048900
	H	1.40519400	-3.47099500	1.75867100
	O	2.03268700	-0.98894900	1.88196000
	C	3.26716000	0.12038800	0.08048200
	C	4.45093900	-0.56349200	-0.61859400
	C	5.42783200	0.25146400	-1.20116100
	C	6.54177800	-0.29729400	-1.81740200
	C	6.71471200	-1.67889200	-1.84664600
	C	5.76327600	-2.49535800	-1.25444200
	C	4.63680000	-1.94336800	-0.64677900
	C	3.85339500	1.02881900	1.17057400
	C	4.49715800	0.44453000	2.26542700
	C	5.10799700	1.22934200	3.23097200
	C	5.10043400	2.61726300	3.11569500
	C	4.47939100	3.20622800	2.02461600
	C	3.85868100	2.41706300	1.05798100
	C	2.34693000	0.88530200	-0.88931400
	C	1.21885600	1.51120800	-0.34518800
	C	0.28401600	2.13639600	-1.15637900
	C	0.46578900	2.15004500	-2.53753900
	C	1.58322100	1.53567500	-3.08739500
	C	2.51783000	0.89870200	-2.26990200
	H	5.31146700	1.32943900	-1.16083100
	H	7.28260600	0.35468000	-2.26484400

	H	7.58833200	-2.11088000	-2.31962900
	H	5.89050700	-3.57143800	-1.26076900
	H	3.90434800	-2.60577500	-0.20389000
	H	4.51766100	-0.63440600	2.35924400
	H	5.59569700	0.75662200	4.07527800
	H	5.58045300	3.23024600	3.86917200
	H	4.47352700	4.28442100	1.91651200
	H	3.38112700	2.89530400	0.21161900
	H	1.07660900	1.49302700	0.73159200
	H	-0.59455400	2.60178800	-0.72115500
	H	-0.26623200	2.63411500	-3.17358000
	H	1.73075400	1.53697100	-4.16122200
	H	3.36552800	0.39766700	-2.71937500
	H	1.51547800	-4.32259300	0.19575800
	C	-0.15549700	-2.64194600	-1.23206900
	C	-0.50841700	-1.34827800	-1.95958400
	H	0.37356900	-0.70843900	-1.98035800
	C	-1.00399100	-1.54909300	-3.38244200
	H	-1.28859300	-0.58482400	-3.81051200
	H	-1.85385300	-2.22890300	-3.42458800
	H	-0.18936200	-1.95497500	-3.98616700
	O	-1.43175700	-0.55399300	-1.17380000
	C	-2.65816800	-1.00618800	-0.91419300
	C	-3.34400900	-0.07673800	0.11190200
	C	-4.83588000	-0.39221000	0.26821900
	C	-5.46239900	-0.25595600	1.50740300
	O	-3.12027500	-2.02962800	-1.34101500
	C	-5.62200600	-0.68390700	-0.84760000
	C	-6.99350900	-0.86047100	-0.72102200
	C	-7.60751700	-0.73629500	0.52039200
	C	-6.83657000	-0.42811600	1.63378400
	C	-3.26712800	1.38907400	-0.32969300
	C	-3.15401300	1.73624200	-1.67580600
	C	-3.17458200	3.06916900	-2.07246300
	C	-3.30388600	4.07970900	-1.12730300
	C	-3.44125200	3.74346100	0.21556600
	C	-3.43619000	2.41040700	0.60670100
	C	-2.52543800	-0.41377800	1.37358700
	C	-1.46706200	0.38099800	1.81357300
	C	-0.65319600	-0.03645000	2.86079800
	C	-0.87537200	-1.26290600	3.47564700
	C	-1.91780500	-2.07014700	3.03482400
	C	-2.73286300	-1.65198900	1.98792800
	H	-4.87346800	-0.01776100	2.38581300
	H	-5.16127200	-0.78660200	-1.82129800
	H	-7.58410500	-1.09744800	-1.59813400
	H	-8.67756900	-0.87586500	0.61759900
	H	-7.30173700	-0.31979400	2.60657100
	H	-3.03986000	0.96518800	-2.42969200
	H	-3.08758600	3.31575700	-3.12437400
	H	-3.31054900	5.11860500	-1.43461000
	H	-3.56234500	4.51998200	0.96155100

	H	-3.56210700	2.15952000	1.65420100
	H	-1.25834000	1.32091700	1.31960700
	H	0.18218700	0.57884100	3.17263400
	H	-0.22495700	-1.59301100	4.27615400
	H	-2.09390900	-3.03419600	3.49794500
	H	-3.53163400	-2.29458000	1.63319100
	H	-1.05357200	-3.24361400	-1.07591300
	H	0.52261200	-3.21448100	-1.87403700
35	O	-1.64355700	-1.36600500	-1.24667300
	C	-2.08492100	-0.10525400	-1.24198200
	C	-0.65989100	-1.73491500	-2.23818700
	H	0.02540900	-0.89176200	-2.34815400
	C	-1.33214200	-2.03407000	-3.56593500
	H	-2.06020500	-2.84035600	-3.44514100
	H	-1.83451500	-1.14513300	-3.94793300
	O	-1.81100500	0.68709800	-2.10057000
	C	-2.99791300	0.13850100	-0.01287300
	C	-2.35067000	-0.61976100	1.15882500
	C	-2.93236800	-1.73057400	1.76265200
	C	-2.27255000	-2.39850600	2.79386200
	C	-1.03031600	-1.96060800	3.23580400
	C	-0.43863200	-0.85502000	2.62877500
	C	-1.08889100	-0.20192400	1.59202100
	C	-4.40585400	-0.39561400	-0.34024600
	C	-4.67925200	-1.19021300	-1.45135100
	C	-5.97062000	-1.65443000	-1.69307500
	C	-7.00376800	-1.34065400	-0.82281100
	C	-6.74018000	-0.54946100	0.29199500
	C	-5.45730300	-0.07722000	0.52524700
	C	-3.12831800	1.64490200	0.28466600
	C	-3.47005900	2.53402700	-0.73978200
	C	-3.65667600	3.88476000	-0.47629000
	C	-3.54146800	4.37388400	0.82190300
	C	-3.24133000	3.49381900	1.85191000
	C	-3.03301200	2.14302600	1.58452100
	H	-3.89880600	-2.08764700	1.42901400
	H	-2.73925300	-3.26191300	3.25341300
	H	-0.52668100	-2.47528100	4.04619300
	H	0.53342200	-0.49841900	2.95168300
	H	-0.60838800	0.64448600	1.11245100
	H	-3.89029000	-1.46356500	-2.14059800
	H	-6.16277200	-2.26407900	-2.56807700
	H	-8.00710100	-1.70341600	-1.01085900
	H	-7.53872900	-0.29195100	0.97753700
	H	-5.26477800	0.55137700	1.38745800
	H	-3.59024800	2.16595000	-1.74924700
	H	-3.90962400	4.55525100	-1.28918000
	H	-3.69767600	5.42656100	1.02636900
	H	-3.16421600	3.85243700	2.87170400
	H	-2.79810700	1.47849000	2.40550500
	H	-0.58393000	-2.35120900	-4.29514300
	C	0.06600400	-2.93514300	-1.65643700

	C	0.54595700	-2.71267500	-0.22400100
	H	-0.32869900	-2.58270100	0.41505200
	C	1.39081700	-3.86388900	0.29819800
	H	1.63370300	-3.71662900	1.35179500
	H	2.31321300	-3.97080800	-0.27310000
	H	0.81791600	-4.78988200	0.21360700
	O	1.22584200	-1.43878800	-0.04412500
	C	2.36648600	-1.19472000	-0.69798400
	C	3.06281500	0.09257200	-0.16717700
	C	4.06181600	0.59895200	-1.22448300
	C	4.07350400	1.92389600	-1.65679500
	O	2.79843100	-1.90515800	-1.56378400
	C	5.06147500	-0.26025000	-1.69789700
	C	6.01652800	0.18610000	-2.59854400
	C	6.00628900	1.50701500	-3.03915500
	C	5.03653700	2.37330400	-2.55894000
	C	3.82806200	-0.27798600	1.12480200
	C	3.29206000	-1.15505500	2.07176000
	C	3.96194100	-1.41985500	3.26164100
	C	5.17454400	-0.80156200	3.53909000
	C	5.70842200	0.08608200	2.61328600
	C	5.04453000	0.34295700	1.41860400
	C	1.98990400	1.15190600	0.12529700
	C	1.91589800	1.84137800	1.33238000
	C	0.96587900	2.84878200	1.51474200
	C	0.08756000	3.18338500	0.49373600
	C	0.15364400	2.49706300	-0.71711700
	C	1.09093700	1.48933500	-0.89168600
	H	3.33838800	2.62588600	-1.28619800
	H	5.09345300	-1.28414000	-1.35193800
	H	6.77550300	-0.50030400	-2.95504900
	H	6.75340200	1.85598900	-3.74222700
	H	5.02186800	3.40837300	-2.87951200
	H	2.33252100	-1.61947400	1.89379000
	H	3.52757900	-2.11130200	3.97481600
	H	5.69571400	-1.00671400	4.46640600
	H	6.64939600	0.58379400	2.81576400
	H	5.47940600	1.03861200	0.71215600
	H	2.59944600	1.60373000	2.13848700
	H	0.91879100	3.36962500	2.46412100
	H	-0.65837500	3.95685900	0.63565200
	H	-0.54130200	2.72702300	-1.51422100
	H	1.14734100	0.97686200	-1.84773600
	H	0.90190000	-3.19476600	-2.30749900
	H	-0.62520600	-3.78473400	-1.62776900
46	O	-0.83947400	1.33780400	1.05527700
	C	-0.27824300	2.51154700	1.33719000
	C	-1.20387800	0.41863400	2.11591400
	H	-1.55737300	-0.44015100	1.54380800
	C	-2.35672700	0.96440200	2.94089100
	H	-2.71618600	0.18266300	3.61339100
	H	-3.18041900	1.24853200	2.28228900

	O	-0.05795300	2.92110300	2.44505300
	C	0.15815800	3.20933100	0.02686000
	C	1.38613300	2.35157400	-0.34046800
	C	1.25531900	1.22165700	-1.14619900
	C	2.31236400	0.33539200	-1.31629700
	C	3.52320400	0.56868400	-0.67416000
	C	3.66677400	1.69278200	0.13408000
	C	2.60460600	2.57471800	0.30689700
	C	0.49500000	4.68937200	0.23728900
	C	-0.23569600	5.47706900	1.12746700
	C	0.00000000	6.84317100	1.22218200
	C	0.96202000	7.44881200	0.42318200
	C	1.68288400	6.67610900	-0.47909000
	C	1.44810900	5.30928300	-0.57203400
	C	-0.96802700	3.17823300	-1.01517200
	C	-0.67382300	3.26661500	-2.37589300
	C	-1.68953700	3.31068300	-3.32369200
	C	-3.02152900	3.29001400	-2.92449500
	C	-3.32562600	3.23810100	-1.56879600
	C	-2.30710000	3.18237800	-0.62429800
	H	0.30450100	1.02212000	-1.62023500
	H	2.18361900	-0.54971500	-1.93200000
	H	4.34500600	-0.12674800	-0.80019300
	H	4.60450700	1.88016700	0.64405300
	H	2.71131300	3.42880700	0.96604500
	H	-0.98640700	5.02430800	1.76067600
	H	-0.57324400	7.43525800	1.92566100
	H	1.14571000	8.51373800	0.50017500
	H	2.43055100	7.13502400	-1.11511200
	H	2.01799500	4.71818800	-1.27992000
	H	0.36098500	3.29302700	-2.69929400
	H	-1.43794300	3.36752800	-4.37611300
	H	-3.81430000	3.32601800	-3.66192700
	H	-4.35906900	3.23933100	-1.24189800
	H	-2.56560400	3.13457500	0.42805600
	H	-2.04904300	1.82603700	3.53353700
	C	0.00000000	0.00000000	2.95491500
	C	1.20387800	-0.41863400	2.11591400
	H	1.55737300	0.44015100	1.54380800
	C	2.35672700	-0.96440200	2.94089100
	H	3.18041900	-1.24853200	2.28228900
	H	2.04904300	-1.82603700	3.53353700
	H	2.71618600	-0.18266300	3.61339100
	O	0.83947400	-1.33780400	1.05527700
	C	0.27824300	-2.51154700	1.33719000
	C	-0.15815800	-3.20933100	0.02686000
	C	-0.49500000	-4.68937200	0.23728900
	C	-1.44810900	-5.30928300	-0.57203400
	O	0.05795300	-2.92110300	2.44505300
	C	0.23569600	-5.47706900	1.12746700
	C	0.00000000	-6.84317100	1.22218200
	C	-0.96202000	-7.44881200	0.42318200

	C	-1.68288400	-6.67610900	-0.47909000
	C	0.96802700	-3.17823300	-1.01517200
	C	2.30710000	-3.18237800	-0.62429800
	C	3.32562600	-3.23810100	-1.56879600
	C	3.02152900	-3.29001400	-2.92449500
	C	1.68953700	-3.31068300	-3.32369200
	C	0.67382300	-3.26661500	-2.37589300
	C	-1.38613300	-2.35157400	-0.34046800
	C	-1.25531900	-1.22165700	-1.14619900
	C	-2.31236400	-0.33539200	-1.31629700
	C	-3.52320400	-0.56868400	-0.67416000
	C	-3.66677400	-1.69278200	0.13408000
	C	-2.60460600	-2.57471800	0.30689700
	H	-2.01799500	-4.71818800	-1.27992000
	H	0.98640700	-5.02430800	1.76067600
	H	0.57324400	-7.43525800	1.92566100
	H	-1.14571000	-8.51373800	0.50017500
	H	-2.43055100	-7.13502400	-1.11511200
	H	2.56560400	-3.13457500	0.42805600
	H	4.35906900	-3.23933100	-1.24189800
	H	3.81430000	-3.32601800	-3.66192700
	H	1.43794300	-3.36752800	-4.37611300
	H	-0.36098500	-3.29302700	-2.69929400
	H	-0.30450100	-1.02212000	-1.62023500
	H	-2.18361900	0.54971500	-1.93200000
	H	-4.34500600	0.12674800	-0.80019300
	H	-4.60450700	-1.88016700	0.64405300
	H	-2.71131300	-3.42880700	0.96604500
	H	-0.31510300	-0.82769500	3.59347900
	H	0.31510300	0.82769500	3.59347900
61	O	-1.34368300	0.58022700	-1.42179800
	C	-2.53143900	-0.02742400	-1.46966600
	C	-0.48031700	0.58895100	-2.58936600
	H	0.39782600	1.11466100	-2.20771300
	C	-1.08281300	1.40335900	-3.72207300
	H	-0.32865300	1.54082200	-4.49973800
	H	-1.37756700	2.38992200	-3.35891200
	O	-2.98485700	-0.55984700	-2.44591600
	C	-3.17275400	-0.06658600	-0.06330700
	C	-4.68111800	-0.34187600	-0.11823800
	C	-5.30109400	-0.96352100	0.96731300
	C	-6.68022200	-1.12436600	1.00484400
	C	-7.46580800	-0.65911700	-0.04320800
	C	-6.85909000	-0.02710300	-1.12137900
	C	-5.47860900	0.13539300	-1.15763000
	C	-3.03086600	1.28140700	0.65445500
	C	-3.11025700	2.46820000	-0.07723600
	C	-3.11730100	3.70244600	0.55947100
	C	-3.04170600	3.77278100	1.94669400
	C	-2.97912200	2.59769400	2.68602600
	C	-2.98560800	1.36285000	2.04566600
	C	-2.38255500	-1.23799100	0.55448800

	C	-1.21098400	-1.03894200	1.28344300
	C	-0.41549200	-2.11713900	1.66076800
	C	-0.78115300	-3.41101500	1.31347600
	C	-1.94817800	-3.62227300	0.58385100
	C	-2.73824500	-2.54519300	0.20169700
	H	-4.69834600	-1.32971700	1.79086700
	H	-7.14018200	-1.61262000	1.85581000
	H	-8.54141000	-0.78650600	-0.01877600
	H	-7.46097200	0.34195000	-1.94331300
	H	-5.02505800	0.62352800	-2.00963500
	H	-3.17090800	2.43226200	-1.15982700
	H	-3.17633200	4.60986700	-0.02983100
	H	-3.04049900	4.73382800	2.44666400
	H	-2.93232000	2.63788200	3.76772800
	H	-2.95847200	0.45510000	2.63726000
	H	-0.89447100	-0.03700300	1.54396500
	H	0.50157800	-1.93630800	2.20794300
	H	-0.15637200	-4.24755400	1.60518700
	H	-2.24079100	-4.62637000	0.29984800
	H	-3.62779300	-2.71348100	-0.39448400
	H	-1.94837600	0.90290600	-4.15432900
	C	-0.06762600	-0.81783600	-3.00746300
	C	0.35963300	-1.70491400	-1.83970100
	H	-0.51748900	-1.94093200	-1.23570700
	C	1.01530100	-3.00157000	-2.28203200
	H	1.25064000	-3.61276300	-1.40826100
	H	1.92592600	-2.80568400	-2.85003000
	H	0.32078800	-3.56026600	-2.91286400
	O	1.20017100	-1.02215500	-0.87177400
	C	2.36360900	-0.48828100	-1.24500800
	C	3.13146600	0.09387600	-0.02211100
	C	2.50376100	1.45408600	0.36182200
	C	3.30599600	2.51436600	0.78993900
	O	2.75390000	-0.46100000	-2.38039200
	C	1.11613500	1.61743900	0.40444600
	C	0.54528900	2.80345900	0.85178700
	C	1.35395300	3.85226600	1.27424000
	C	2.73476700	3.70085700	1.24170200
	C	4.60562400	0.27543000	-0.41810900
	C	4.92608700	1.09856600	-1.50471400
	C	6.24587700	1.32051400	-1.86406600
	C	7.28009300	0.73029300	-1.14109800
	C	6.97557700	-0.07667300	-0.05643700
	C	5.64721800	-0.30153200	0.30408500
	C	3.00274900	-0.89378300	1.14633900
	C	3.15114500	-2.26215500	0.90117900
	C	3.11606300	-3.18210600	1.93860300
	C	2.94086100	-2.74718900	3.25125700
	C	2.80118000	-1.38978300	3.50612000
	C	2.82631800	-0.46799500	2.45970000
	H	4.38425800	2.41612400	0.78257300
	H	0.46266200	0.81290100	0.10154400

	H -0.53535900 2.89423600 0.87296300<br>H 0.91176800 4.77624100 1.62840300<br>H 3.37914900 4.50748300 1.57138400<br>H 4.13186200 1.57213700 -2.06674700<br>H 6.47006100 1.95825000 -2.71098800<br>H 8.31206700 0.90470900 -1.42169500<br>H 7.76861000 -0.53724200 0.52077900<br>H 5.43589600 -0.92805100 1.16096400<br>H 3.30944300 -2.60849300 -0.11591200<br>H 3.23171400 -4.23842500 1.72540700<br>H 2.91477800 -3.46207900 4.06491300<br>H 2.66596400 -1.03849300 4.52223100<br>H 2.70870200 0.58712200 2.67506900<br>H 0.74899000 -0.71794100 -3.72448600<br>H -0.90364500 -1.31703300 -3.50261900

22 (optimized at the B3LYP/6-311G(d,p) level)

Conformer no	
1	O -0.79709600 2.13039800 -1.41739200<br>C -0.50222100 3.44166700 -1.38789600<br>C -1.20244700 1.55010900 -2.70065900<br>H -0.67288600 2.10269900 -3.48009600<br>C -2.70514000 1.72362400 -2.88261100<br>H -3.01287900 1.30969700 -3.84655300<br>H -2.97482700 2.78094900 -2.86950300<br>O -0.58411600 4.16744900 -2.34679400<br>C 0.05194000 3.85809900 0.00785800<br>C -0.90364200 3.47754900 1.16330000<br>C -0.45467400 3.59047100 2.48662900<br>C -1.30415400 3.34882500 3.56041000<br>C -2.63612600 3.00344800 3.33911700<br>C -3.10161800 2.90721400 2.03231600<br>C -2.24377000 3.13988900 0.95683700<br>C 0.21250700 5.39501000 0.09876500<br>C -0.80605800 6.24249500 -0.35546700<br>C -0.72246900 7.61986200 -0.18296400<br>C 0.37634600 8.18587600 0.46003500<br>C 1.38711700 7.35550600 0.93124700<br>C 1.30415400 5.97508400 0.75265900<br>C 1.41064600 3.11050800 0.04743800<br>C 2.44470400 3.53961900 -0.79721800<br>C 3.65598700 2.85664600 -0.84974600<br>C 3.85679200 1.72218900 -0.06512100<br>C 2.82811100 1.27071600 0.75660500<br>C 1.61354000 1.95444200 0.80507700<br>H 0.57080500 3.87899300 2.68190700<br>H -0.92568000 3.43984300 4.57248600<br>H -3.30210700 2.82077100 4.17477300<br>H -4.13835500 2.65273900 1.84063000<br>H -2.63711300 3.05065200 -0.04632900<br>H -1.67067900 5.82938000 -0.85591700

	H	-1.52161700	8.25264300	-0.55296400
	H	0.44058700	9.25991000	0.59369600
	H	2.24706400	7.77665300	1.44038300
	H	2.10488800	5.35074700	1.12770700
	H	2.29848600	4.41555900	-1.41796500
	H	4.44270900	3.21268500	-1.50567800
	H	4.80297600	1.19363500	-0.09985300
	H	2.95927800	0.37816100	1.35788900
	H	0.82003000	1.57510000	1.43388000
	H	-3.25325600	1.20026300	-2.09527600
	C	-0.76055900	0.09118800	-2.67363500
	H	-1.18687000	-0.39687500	-3.55824400
	H	-1.19957000	-0.39676100	-1.79933800
	C	0.76055900	-0.09118800	-2.67363500
	C	1.20244700	-1.55010900	-2.70065900
	H	0.67288600	-2.10269900	-3.48009600
	C	2.70514000	-1.72362400	-2.88261100
	H	3.01287900	-1.30969700	-3.84655300
	H	2.97482700	-2.78094900	-2.86950300
	H	3.25325600	-1.20026300	-2.09527600
	O	0.79709600	-2.13039800	-1.41739200
	C	0.50222100	-3.44166700	-1.38789600
	C	-0.05194000	-3.85809900	0.00785800
	C	0.90364200	-3.47754900	1.16330000
	C	0.45467400	-3.59047100	2.48662900
	O	0.58411600	-4.16744900	-2.34679400
	C	2.24377000	-3.13988900	0.95683700
	C	3.10161800	-2.90721400	2.03231600
	C	2.63612600	-3.00344800	3.33911700
	C	1.30415400	-3.34882500	3.56041000
	C	-0.21250700	-5.39501000	0.09876500
	C	0.80605800	-6.24249500	-0.35546700
	C	0.72246900	-7.61986200	-0.18296400
	C	-0.37634600	-8.18587600	0.46003500
	C	-1.38711700	-7.35550600	0.93124700
	C	-1.30415400	-5.97508400	0.75265900
	C	-1.41064600	-3.11050800	0.04743800
	C	-2.44470400	-3.53961900	-0.79721800
	C	-3.65598700	-2.85664600	-0.84974600
	C	-3.85679200	-1.72218900	-0.06512100
	C	-2.82811100	-1.27071600	0.75660500
	C	-1.61354000	-1.95444200	0.80507700
	H	-0.57080500	-3.87899300	2.68190700
	H	2.63711300	-3.05065200	-0.04632900
	H	4.13835500	-2.65273900	1.84063000
	H	3.30210700	-2.82077100	4.17477300
	H	0.92568000	-3.43984300	4.57248600
	H	1.67067900	-5.82938000	-0.85591700
	H	1.52161700	-8.25264300	-0.55296400
	H	-0.44058700	-9.25991000	0.59369600
	H	-2.24706400	-7.77665300	1.44038300
	H	-2.10488800	-5.35074700	1.12770700

	H	-2.29848600	-4.41555900	-1.41796500
	H	-4.44270900	-3.21268500	-1.50567800
	H	-4.80297600	-1.19363500	-0.09985300
	H	-2.95927800	-0.37816100	1.35788900
	H	-0.82003000	-1.57510000	1.43388000
	H	1.19957000	0.39676100	-1.79933800
	H	1.18687000	0.39687500	-3.55824400
3	O	-2.12706600	-0.80610600	-1.41612900
	C	-3.43910200	-0.51451100	-1.38781900
	C	-1.54445200	-1.20992700	-2.69883500
	H	-2.09922700	-0.68364600	-3.47893600
	C	-1.71108600	-2.71354900	-2.87933600
	H	-1.29523100	-3.02041300	-3.84273100
	H	-2.76722800	-2.98791900	-2.86655900
	O	-4.16391200	-0.59856500	-2.34725700
	C	-3.85796400	0.03891800	0.00754000
	C	-3.47652700	-0.91605600	1.16319500
	C	-3.59121600	-0.46752700	2.48651600
	C	-3.34880700	-1.31676800	3.56031800
	C	-3.00083800	-2.64806800	3.33904000
	C	-2.90279500	-3.11315200	2.03222800
	C	-3.13626900	-2.25553300	0.95674500
	C	-5.39528300	0.19646600	0.09715000
	C	-6.24050500	-0.82354000	-0.35810500
	C	-7.61816900	-0.74252500	-0.18675900
	C	-8.18677000	0.35505500	0.45607700
	C	-7.35867400	1.36721000	0.92830100
	C	-5.97794800	1.28684200	0.75086500
	C	-3.11322700	1.39914400	0.04793500
	C	-3.54378900	2.43242400	-0.79693700
	C	-2.86340100	3.64520300	-0.84858700
	C	-1.73013900	3.84831900	-0.06282800
	C	-1.27716700	2.82044800	0.75909200
	C	-1.95828000	1.60437700	0.80665600
	H	-3.88175100	0.55739100	2.68176800
	H	-3.44125400	-0.93865000	4.57239900
	H	-2.81755300	-3.31385600	4.17471900
	H	-2.64625300	-4.14937900	1.84053900
	H	-3.04562600	-2.64854600	-0.04643200
	H	-5.82537300	-1.68723100	-0.85847900
	H	-8.24916800	-1.54271900	-0.55754400
	H	-9.26103800	0.41727100	0.58882400
	H	-7.78182600	2.22624000	1.43732500
	H	-5.35543000	2.08863800	1.12667200
	H	-4.41885000	2.28443700	-1.41850900
	H	-3.22052500	4.43128400	-1.50470000
	H	-1.20362400	4.79566700	-0.09683000
	H	-0.38543100	2.95349000	1.36118200
	H	-1.57780600	0.81151200	1.43558600
	H	-1.18575500	-3.25850300	-2.09112700
	C	-0.08757200	-0.76124600	-2.67174800
	H	0.40260600	-1.18543000	-3.55621100

	H	0.40231000	-1.19777500	-1.79730300
	C	0.08760500	0.76066800	-2.67193200
	C	1.54448800	1.20933800	-2.69912000
	H	2.09926100	0.68287000	-3.47909800
	C	1.71112800	2.71291600	-2.87997600
	H	1.29527700	3.01955100	-3.84344500
	H	2.76727100	2.98728500	-2.86725900
	H	1.18579500	3.25805900	-2.09189900
	O	2.12709400	0.80580800	-1.41632100
	C	3.43912300	0.51418500	-1.38794500
	C	3.85796900	-0.03892900	0.00754600
	C	3.11312500	-1.39908600	0.04830700
	C	1.95821900	-1.60405500	0.80715900
	O	4.16392700	0.59798000	-2.34741000
	C	3.54353400	-2.43258500	-0.79637500
	C	2.86304100	-3.64531900	-0.84770800
	C	1.72982300	-3.84816800	-0.06181700
	C	1.27700000	-2.82008000	0.75991300
	C	3.47662300	0.91637200	1.16295900
	C	3.13638600	2.25580100	0.95616400
	C	2.90299900	3.11371900	2.03142600
	C	3.00110700	2.64899000	3.33835900
	C	3.34905700	1.31774200	3.55998000
	C	3.59138300	0.46820200	2.48639600
	C	5.39528000	-0.19656900	0.09717800
	C	6.24056800	0.82329200	-0.35827800
	C	7.61822800	0.74222000	-0.18691800
	C	8.18675800	-0.35527100	0.45613300
	C	7.35859600	-1.36727400	0.92856300
	C	5.97787400	-1.28685000	0.75111500
	H	1.57785800	-0.81101800	1.43594100
	H	4.41856000	-2.28480800	-1.41804700
	H	3.22004800	-4.43157300	-1.50367800
	H	1.20322400	-4.79547900	-0.09557200
	H	0.38529700	-2.95291400	1.36209700
	H	3.04569300	2.64854100	-0.04711600
	H	2.64647400	4.14990100	1.83946900
	H	2.81789000	3.31501200	4.17386700
	H	3.44155600	0.93989900	4.57216000
	H	3.88190900	-0.55666800	2.68191100
	H	5.82549200	1.68691400	-0.85881700
	H	8.24927900	1.54230000	-0.55786200
	H	9.26102200	-0.41752900	0.58889100
	H	7.78169200	-2.22623000	1.43776100
	H	5.35530500	-2.08852700	1.12709000
	H	-0.40228000	1.19741000	-1.79759400
	H	-0.40256600	1.18463800	-3.55650100
5	O	2.09110100	-0.92849000	1.28897200
	C	3.43181200	-0.86265700	1.21911200
	C	1.49539600	-1.67460900	2.40079100
	H	2.05998700	-1.41475900	3.30095800
	C	1.60643500	-3.17149100	2.13801000

	H	1.14919900	-3.72513400	2.96250000
	H	2.65067300	-3.47436700	2.06244800
	O	4.16524700	-1.36655000	2.03167100
	C	3.87491800	-0.06857600	-0.05149900
	C	5.41026300	-0.15515600	-0.25453500
	C	5.97201400	-0.36508200	-1.51828400
	C	7.35397800	-0.34071700	-1.70682200
	C	8.20449900	-0.10176800	-0.63391800
	C	7.65743400	0.12645600	0.62726300
	C	6.27968700	0.10909400	0.81331900
	C	3.56399800	1.43673900	0.13456400
	C	3.13511800	1.97541100	1.34990500
	C	2.94343800	3.34959700	1.50147700
	C	3.17584400	4.21301600	0.43756800
	C	3.61527300	3.69053900	-0.77820000
	C	3.81463100	2.32251600	-0.92305000
	C	3.08636800	-0.72574100	-1.21076700
	C	2.03264400	-0.09476000	-1.87386500
	C	1.31797800	-0.75597300	-2.87357100
	C	1.63429700	-2.06636700	-3.21783500
	C	2.66808000	-2.71704200	-2.54503200
	C	3.38142000	-2.05379500	-1.55230800
	H	5.33331900	-0.55058400	-2.37144100
	H	7.75912200	-0.50926000	-2.69859600
	H	9.27917700	-0.08649700	-0.77709900
	H	8.30603900	0.32359900	1.47371000
	H	5.88076800	0.29527700	1.79956000
	H	2.95258600	1.33211500	2.20093600
	H	2.61537800	3.73920500	2.45881800
	H	3.02748600	5.28056300	0.55403600
	H	3.81414700	4.35122000	-1.61473000
	H	4.18102600	1.93822100	-1.86712600
	H	1.75171600	0.91504800	-1.60865500
	H	0.50792000	-0.23964500	-3.37674200
	H	1.08105900	-2.57729300	-3.99801900
	H	2.92190700	-3.74154900	-2.79403500
	H	4.18310500	-2.57219600	-1.03820500
	H	1.08555600	-3.43609700	1.21497600
	C	0.04940500	-1.19944600	2.50354200
	H	-0.43815300	-1.80021200	3.28018500
	H	-0.46050700	-1.42389700	1.56366100
	C	-0.10199600	0.28786400	2.83155200
	C	-1.55153500	0.75467400	2.91859900
	H	-2.12911900	0.11664300	3.59203500
	C	-1.68576200	2.20997400	3.35034400
	H	-2.73417500	2.50887400	3.37913300
	H	-1.14115600	2.86702500	2.66698400
	H	-1.27054500	2.33976500	4.35344100
	O	-2.11554300	0.58188100	1.57801200
	C	-3.44604500	0.41614000	1.47579000
	C	-3.86728200	0.06384500	0.01882400
	C	-3.37512400	-1.40490700	-0.08905100

	C	-2.20581200	-1.75595300	-0.76953900
	O	-4.19616500	0.44484800	2.41912600
	C	-4.05505700	-2.40529800	0.62058700
	C	-3.60319700	-3.72149200	0.61246000
	C	-2.45431500	-4.06524000	-0.09760700
	C	-1.75479300	-3.07557700	-0.78201400
	C	-3.25460400	1.00566900	-1.04365400
	C	-2.73877800	2.26639900	-0.73023000
	C	-2.29086700	3.13006200	-1.72938000
	C	-2.35059000	2.75298500	-3.06626400
	C	-2.87650200	1.50504000	-3.39579800
	C	-3.32794100	0.64870300	-2.39732100
	C	-5.39980000	0.19666800	-0.15187700
	C	-6.08011600	1.29937100	0.37899400
	C	-7.43756400	1.48666200	0.14093900
	C	-8.14784900	0.58079500	-0.64388900
	C	-7.48172700	-0.51093000	-1.19008200
	C	-6.12188100	-0.69879800	-0.94706800
	H	-1.63218400	-0.99986800	-1.28767000
	H	-4.94285400	-2.14906300	1.18579100
	H	-4.15049800	-4.47790400	1.16400100
	H	-2.10473700	-5.09156200	-0.10958500
	H	-0.84791400	-3.31926200	-1.32405900
	H	-2.67397300	2.58986100	0.29921800
	H	-1.89337600	4.10076100	-1.45451200
	H	-2.00106700	3.42404600	-3.84267500
	H	-2.94587600	1.19936500	-4.43399500
	H	-3.74974700	-0.30868200	-2.67712600
	H	-5.55228900	2.01711700	0.99189900
	H	-7.94123500	2.34490200	0.57183400
	H	-9.20629200	0.72669300	-0.82810700
	H	-8.01678100	-1.22352900	-1.80814900
	H	-5.62614100	-1.55732700	-1.38155400
	H	0.41306400	0.88775000	2.07687600
	H	0.37349200	0.50605200	3.79572100
7	O	-2.22569600	0.11332800	1.70464200
	C	-3.52727500	-0.18713500	1.54375700
	C	-1.63972900	-0.08117200	3.03162400
	H	-2.14969600	-0.93379100	3.48586000
	C	-1.87513300	1.16413300	3.87769900
	H	-1.44583900	1.02519700	4.87342800
	H	-2.94316500	1.35327000	3.99617300
	O	-4.23497300	-0.58545300	2.43430300
	C	-3.95026300	-0.05200800	0.05137500
	C	-3.60028500	1.33483600	-0.53725800
	C	-3.67260200	1.53363700	-1.92253100
	C	-3.46152000	2.78878500	-2.48324000
	C	-3.18704900	3.88606600	-1.66884100
	C	-3.13382100	3.70890200	-0.29041300
	C	-3.33897900	2.44728100	0.26769700
	C	-5.48481000	-0.18460600	-0.10519600
	C	-6.35254400	0.46958100	0.77828500

	C	-7.72898600	0.44660600	0.57916700
	C	-8.27370600	-0.22138700	-0.51522800
	C	-7.42306200	-0.86221300	-1.40941200
	C	-6.04414600	-0.84202900	-1.20592000
	C	-3.17881900	-1.22863900	-0.60279300
	C	-3.59685100	-2.54144900	-0.34044500
	C	-2.89394500	-3.63128400	-0.84506900
	C	-1.75109200	-3.43218400	-1.61763300
	C	-1.31097900	-2.13430500	-1.86075300
	C	-2.01258300	-1.04272100	-1.34974000
	H	-3.90462300	0.69933900	-2.57279400
	H	-3.52245100	2.91045400	-3.55909700
	H	-3.02728800	4.86633800	-2.10328600
	H	-2.93448600	4.55334100	0.36017600
	H	-3.29081800	2.34406700	1.34322300
	H	-5.95743900	0.99496100	1.63641700
	H	-8.37761000	0.95439600	1.28442400
	H	-9.34681300	-0.23897200	-0.66893600
	H	-7.82744200	-1.38221200	-2.27090400
	H	-5.40391300	-1.35033700	-1.91538500
	H	-4.48002000	-2.71031100	0.26399900
	H	-3.24168800	-4.63643600	-0.63384800
	H	-1.20727000	-4.28000100	-2.01920500
	H	-0.41385500	-1.95979200	-2.44388000
	H	-1.63455900	-0.04607400	-1.53057300
	H	-1.40444700	2.03870000	3.42079900
	C	-0.16289900	-0.38926600	2.80967400
	H	0.32773400	-0.37815700	3.78993600
	H	0.27792500	0.42203100	2.22343200
	C	0.09655900	-1.73385800	2.12358200
	C	1.57748100	-2.06693200	1.97242600
	H	2.11011000	-1.89231500	2.90860000
	C	1.83325900	-3.48732400	1.48734400
	H	1.31955400	-3.67276900	0.54085200
	H	1.46473700	-4.20459500	2.22571500
	H	2.90172000	-3.66179400	1.34770800
	O	2.13653200	-1.14011300	0.98110800
	C	3.34359400	-0.60134100	1.22837000
	C	3.91905900	0.13959600	-0.01433200
	C	2.81426200	0.69508700	-0.94293800
	C	1.77284900	1.45105400	-0.38993200
	O	3.95277800	-0.75861600	2.25752400
	C	2.87642000	0.58871600	-2.33476900
	C	1.91494100	1.19390700	-3.14554500
	C	0.87339900	1.92059000	-2.57952400
	C	0.80873100	2.04943900	-1.19211200
	C	4.76274000	-1.00223700	-0.64554200
	C	6.14169400	-1.08980400	-0.43103000
	C	6.87575900	-2.16525800	-0.92776300
	C	6.24387300	-3.18283400	-1.63575300
	C	4.86561400	-3.12308600	-1.83025000
	C	4.13234800	-2.04937500	-1.33299600

	C	4.78375500	1.36490800	0.37365700
	C	4.81364300	1.92849300	1.65225900
	C	5.54815500	3.08854800	1.90460900
	C	6.26868500	3.70689100	0.89011900
	C	6.24541900	3.15602200	-0.39035000
	C	5.50797100	2.00569600	-0.64269300
	H	1.71815500	1.58239900	0.68433300
	H	3.67934200	0.03278300	-2.80086900
	H	1.98890700	1.09564100	-4.22316900
	H	0.12224300	2.38829300	-3.20555800
	H	0.00429600	2.61592500	-0.73702700
	H	6.64760700	-0.31687200	0.13116500
	H	7.94505800	-2.20632600	-0.75267800
	H	6.81602300	-4.01806000	-2.02373900
	H	4.35611700	-3.91592100	-2.36683100
	H	3.05973000	-2.02609200	-1.47492500
	H	4.28676300	1.45683800	2.46770600
	H	5.55522500	3.50226700	2.90709800
	H	6.84025000	4.60617800	1.09043200
	H	6.79701900	3.62605700	-1.19708500
	H	5.49041400	1.60244600	-1.64797800
	H	-0.37931900	-1.75573300	1.13975600
	H	-0.36011900	-2.53549300	2.71608700
8	O	2.41159400	-1.86362800	-0.37874100
	C	2.40877400	-0.74023600	0.35733000
	C	1.45451500	-2.91341700	-0.02774800
	H	1.29844200	-2.85799500	1.05186400
	C	2.12546000	-4.22288500	-0.41015600
	H	3.08242800	-4.32368500	0.10478700
	H	2.30622000	-4.26517200	-1.48743300
	O	1.62780900	-0.52939600	1.25348700
	C	3.61067300	0.17629100	-0.00935500
	C	3.80533000	0.32825800	-1.53517000
	C	5.04385500	0.73108300	-2.04968600
	C	5.21189400	0.98562300	-3.40766200
	C	4.13870200	0.85535800	-4.28550700
	C	2.89708100	0.47449000	-3.78616400
	C	2.73354500	0.21653800	-2.42686600
	C	4.76436900	-0.57539200	0.70891000
	C	5.56665400	-1.50795600	0.04249400
	C	6.53143800	-2.24073900	0.73086800
	C	6.69976400	-2.07079800	2.10240300
	C	5.88499700	-1.16911700	2.78255600
	C	4.92364600	-0.43435100	2.09392600
	C	3.41130400	1.61656900	0.51945100
	C	2.15216900	2.22726000	0.51804600
	C	2.00187200	3.56137300	0.88712600
	C	3.10913900	4.32031500	1.25593400
	C	4.36973100	3.73054300	1.24698200
	C	4.51708300	2.39489700	0.88088500
	H	5.88926200	0.85370800	-1.38428700
	H	6.18424600	1.29228400	-3.77709700

	H	4.26739100	1.05455800	-5.34333700
	H	2.04750100	0.37651200	-4.45282200
	H	1.75435300	-0.07520900	-2.07035700
	H	5.43566900	-1.66921300	-1.01891100
	H	7.14862500	-2.95004400	0.19044400
	H	7.45167100	-2.64066900	2.63668100
	H	5.99265500	-1.03705700	3.85339600
	H	4.28704200	0.25246500	2.63728400
	H	1.27213500	1.66579000	0.23939300
	H	1.01138600	4.00224100	0.88556500
	H	2.99092100	5.35856900	1.54510300
	H	5.24476700	4.30766800	1.52518500
	H	5.50755900	1.95753000	0.88262000
	H	1.49601300	-5.07055500	-0.12974700
	C	0.13224200	-2.63514600	-0.74487800
	H	0.29274500	-2.68626600	-1.82728300
	H	-0.16087100	-1.61033400	-0.51144200
	C	-0.99974200	-3.58393200	-0.33255900
	C	-2.37269200	-3.16346800	-0.85841000
	H	-2.31506100	-2.91649100	-1.91949300
	C	-3.45438100	-4.20479100	-0.60841700
	H	-4.42344000	-3.84519500	-0.95892600
	H	-3.53495800	-4.43318600	0.45751800
	H	-3.21661900	-5.12811700	-1.14343400
	O	-2.78071700	-1.94943500	-0.15052600
	C	-2.91521100	-0.80480900	-0.84611200
	C	-3.59597200	0.30124100	0.01049300
	C	-3.22584100	1.72880400	-0.46052600
	C	-4.00674800	2.80273300	-0.01027100
	O	-2.63328300	-0.69031700	-2.01308400
	C	-2.08415400	2.02211400	-1.21226000
	C	-1.74263800	3.34182400	-1.51096000
	C	-2.53268700	4.39586800	-1.06583300
	C	-3.67074300	4.11777400	-0.31110900
	C	-3.16983600	0.24403400	1.49624400
	C	-1.82109200	0.05653000	1.82063600
	C	-1.37838400	0.11390100	3.13724400
	C	-2.28108400	0.37139200	4.16736600
	C	-3.62211400	0.57780500	3.86003700
	C	-4.06114200	0.51892800	2.53754300
	C	-5.08972700	-0.03866000	-0.24724200
	C	-5.72857000	-1.05922300	0.46986200
	C	-7.03897200	-1.42766500	0.17607600
	C	-7.73301500	-0.79718700	-0.85415300
	C	-7.09774600	0.19501800	-1.59495600
	C	-5.78767000	0.56514800	-1.29940400
	H	-4.88907200	2.61031600	0.58793600
	H	-1.46464400	1.22536700	-1.59490500
	H	-0.85283300	3.53820400	-2.09881400
	H	-2.26690700	5.42010500	-1.30209900
	H	-4.29725200	4.92586100	0.05016500
	H	-1.08970100	-0.12800800	1.04427900

	H	-0.32503100	-0.03581200	3.34306100
	H	-1.94071300	0.41812800	5.19577400
	H	-4.33559400	0.79044300	4.64881100
	H	-5.10828100	0.69211400	2.32538500
	H	-5.19597900	-1.57115000	1.26012400
	H	-7.51535800	-2.21221900	0.75371900
	H	-8.75398000	-1.08254400	-1.08169400
	H	-7.61834700	0.68342400	-2.41118000
	H	-5.30495000	1.32805700	-1.89542300
	H	-1.05908100	-3.64864600	0.76006500
	H	-0.80003700	-4.59519800	-0.70160100
9	O	-1.93372400	1.54483500	-0.36479500
	C	-2.78853300	1.67446000	0.66434200
	C	-0.49642500	1.55633400	-0.06759600
	H	-0.37051400	1.08110900	0.90707300
	C	-0.00316900	2.99542600	0.00401000
	H	1.06613300	3.00723200	0.22837400
	H	-0.52261000	3.54287800	0.79210400
	O	-2.44056600	1.81193400	1.81018100
	C	-4.26534600	1.51224900	0.19384200
	C	-4.61286300	2.42083800	-1.00820500
	C	-5.79397700	2.19139900	-1.72698600
	C	-6.19349300	3.04395000	-2.75051900
	C	-5.42727500	4.16209600	-3.07282700
	C	-4.26424900	4.41540100	-2.35405900
	C	-3.86254300	3.55447300	-1.33349100
	C	-5.25215500	1.92328900	1.31348200
	C	-5.02307900	3.07232100	2.08042000
	C	-5.95952300	3.51110300	3.01071800
	C	-7.15483200	2.81885100	3.19029500
	C	-7.40274000	1.68487600	2.42427500
	C	-6.46129800	1.24409900	1.49562400
	C	-4.32306700	-0.00520400	-0.12472800
	C	-4.31320200	-0.92400000	0.93436700
	C	-4.29214200	-2.29497400	0.69355000
	C	-4.26861800	-2.77829900	-0.61353500
	C	-4.24746700	-1.87509800	-1.67243700
	C	-4.26534600	-0.50211500	-1.43014700
	H	-6.41568100	1.33945400	-1.48096000
	H	-7.11056800	2.83724600	-3.29105800
	H	-5.73814000	4.82998300	-3.86814000
	H	-3.66084600	5.28673900	-2.58362100
	H	-2.95258200	3.78135300	-0.79477600
	H	-4.10350500	3.62801900	1.96223800
	H	-5.75169800	4.39901400	3.59752900
	H	-7.88366000	3.16138800	3.91623100
	H	-8.33027800	1.13615100	2.54528300
	H	-6.67762700	0.35819000	0.91232300
	H	-4.32221100	-0.56192300	1.95524000
	H	-4.29511400	-2.98460200	1.53037300
	H	-4.26057700	-3.84580900	-0.80298700
	H	-4.21378300	-2.23527500	-2.69476000

	H	-4.22971700	0.18294100	-2.26619400
	H	-0.15867200	3.50549700	-0.95074300
	C	0.17165300	0.74813900	-1.17581500
	H	1.25405900	0.87643000	-1.08892000
	H	-0.12055200	1.18725100	-2.13517500
	C	-0.17165300	-0.74813900	-1.17581500
	C	0.49642500	-1.55633400	-0.06759600
	H	0.37051400	-1.08110900	0.90707300
	C	0.00316900	-2.99542600	0.00401000
	H	0.52261000	-3.54287800	0.79210400
	H	0.15867200	-3.50549700	-0.95074300
	H	-1.06613300	-3.00723200	0.22837400
	O	1.93372400	-1.54483500	-0.36479500
	C	2.78853300	-1.67446000	0.66434200
	C	4.26534600	-1.51224900	0.19384200
	C	4.61286300	-2.42083800	-1.00820500
	C	5.79397700	-2.19139900	-1.72698600
	O	2.44056600	-1.81193400	1.81018100
	C	3.86254300	-3.55447300	-1.33349100
	C	4.26424900	-4.41540100	-2.35405900
	C	5.42727500	-4.16209600	-3.07282700
	C	6.19349300	-3.04395000	-2.75051900
	C	5.25215500	-1.92328900	1.31348200
	C	5.02307900	-3.07232100	2.08042000
	C	5.95952300	-3.51110300	3.01071800
	C	7.15483200	-2.81885100	3.19029500
	C	7.40274000	-1.68487600	2.42427500
	C	6.46129800	-1.24409900	1.49562400
	C	4.32306700	0.00520400	-0.12472800
	C	4.31320200	0.92400000	0.93436700
	C	4.29214200	2.29497400	0.69355000
	C	4.26861800	2.77829900	-0.61353500
	C	4.24746700	1.87509800	-1.67243700
	C	4.26534600	0.50211500	-1.43014700
	H	6.41568100	-1.33945400	-1.48096000
	H	2.95258200	-3.78135300	-0.79477600
	H	3.66084600	-5.28673900	-2.58362100
	H	5.73814000	-4.82998300	-3.86814000
	H	7.11056800	-2.83724600	-3.29105800
	H	4.10350500	-3.62801900	1.96223800
	H	5.75169800	-4.39901400	3.59752900
	H	7.88366000	-3.16138800	3.91623100
	H	8.33027800	-1.13615100	2.54528300
	H	6.67762700	-0.35819000	0.91232300
	H	4.32221100	0.56192300	1.95524000
	H	4.29511400	2.98460200	1.53037300
	H	4.26057700	3.84580900	-0.80298700
	H	4.21378300	2.23527500	-2.69476000
	H	4.22971700	-0.18294100	-2.26619400
	H	0.12055200	-1.18725100	-2.13517500
	H	-1.25405900	-0.87643000	-1.08892000
10	O	0.05561100	2.33170500	-1.91257200

	C	0.74781400	2.47441300	-0.76935500
	C	0.76293700	1.82934200	-3.09369500
	H	1.80804300	2.12454100	-2.98331700
	C	0.13591700	2.54846900	-4.27925000
	H	0.62017500	2.23053300	-5.20654500
	H	0.26154700	3.62763300	-4.17572300
	O	1.86925200	2.05774700	-0.61045200
	C	-0.03296700	3.35829500	0.24871400
	C	0.56608000	3.26546100	1.67163100
	C	0.50588400	4.36391700	2.53729400
	C	0.92960000	4.26328900	3.86028600
	C	1.41990300	3.05655500	4.35034600
	C	1.47392300	1.95250200	3.50389600
	C	1.04776300	2.05497600	2.18245100
	C	-1.50748900	2.92236200	0.40199800
	C	-1.89830000	1.59681900	0.19165500
	C	-3.20201300	1.17428600	0.44224800
	C	-4.14769500	2.07771200	0.91835200
	C	-3.77078200	3.39851000	1.15068900
	C	-2.46509700	3.81304500	0.90183300
	C	0.15747100	4.75979900	-0.39248100
	C	-0.80163200	5.32397200	-1.24048100
	C	-0.56608000	6.54178700	-1.87561600
	C	0.64169200	7.20980500	-1.69272400
	C	1.61798800	6.64155200	-0.87809400
	C	1.38052000	5.42684200	-0.24042000
	H	0.12788600	5.31335600	2.17971400
	H	0.87331000	5.13255800	4.50634000
	H	1.75262700	2.97617800	5.37912400
	H	1.84766800	1.00206800	3.86780600
	H	1.10383700	1.17992400	1.55169300
	H	-1.18560900	0.86958000	-0.17312900
	H	-3.46239300	0.13740900	0.26322700
	H	-5.16415200	1.75504500	1.11338800
	H	-4.49252800	4.11154600	1.53390000
	H	-2.19193000	4.84088600	1.10568600
	H	-1.73672500	4.80859400	-1.41246200
	H	-1.33012800	6.96422600	-2.51893700
	H	0.82367600	8.15807300	-2.18594200
	H	2.57030500	7.14097700	-0.73863500
	H	2.15387900	4.99110000	0.37965200
	H	-0.93247300	2.33694700	-4.35537200
	C	0.70649300	0.29887100	-3.15950500
	H	1.27809300	-0.10088700	-2.32008600
	H	1.24814000	0.00274700	-4.06482200
	C	-0.70649300	-0.29887100	-3.15950500
	C	-0.76293700	-1.82934200	-3.09369500
	H	-1.80804300	-2.12454100	-2.98331700
	C	-0.13591700	-2.54846900	-4.27925000
	H	-0.26154700	-3.62763300	-4.17572300
	H	0.93247300	-2.33694700	-4.35537200
	H	-0.62017500	-2.23053300	-5.20654500

	O	-0.05561100	-2.33170500	-1.91257200
	C	-0.74781400	-2.47441300	-0.76935500
	C	0.03296700	-3.35829500	0.24871400
	C	-0.56608000	-3.26546100	1.67163100
	C	-0.50588400	-4.36391700	2.53729400
	O	-1.86925200	-2.05774700	-0.61045200
	C	-1.04776300	-2.05497600	2.18245100
	C	-1.47392300	-1.95250200	3.50389600
	C	-1.41990300	-3.05655500	4.35034600
	C	-0.92960000	-4.26328900	3.86028600
	C	1.50748900	-2.92236200	0.40199800
	C	1.89830000	-1.59681900	0.19165500
	C	3.20201300	-1.17428600	0.44224800
	C	4.14769500	-2.07771200	0.91835200
	C	3.77078200	-3.39851000	1.15068900
	C	2.46509700	-3.81304500	0.90183300
	C	-0.15747100	-4.75979900	-0.39248100
	C	0.80163200	-5.32397200	-1.24048100
	C	0.56608000	-6.54178700	-1.87561600
	C	-0.64169200	-7.20980500	-1.69272400
	C	-1.61798800	-6.64155200	-0.87809400
	C	-1.38052000	-5.42684200	-0.24042000
	H	-0.12788600	-5.31335600	2.17971400
	H	-1.10383700	-1.17992400	1.55169300
	H	-1.84766800	-1.00206800	3.86780600
	H	-1.75262700	-2.97617800	5.37912400
	H	-0.87331000	-5.13255800	4.50634000
	H	1.18560900	-0.86958000	-0.17312900
	H	3.46239300	-0.13740900	0.26322700
	H	5.16415200	-1.75504500	1.11338800
	H	4.49252800	-4.11154600	1.53390000
	H	2.19193000	-4.84088600	1.10568600
	H	1.73672500	-4.80859400	-1.41246200
	H	1.33012800	-6.96422600	-2.51893700
	H	-0.82367600	-8.15807300	-2.18594200
	H	-2.57030500	-7.14097700	-0.73863500
	H	-2.15387900	-4.99110000	0.37965200
	H	-1.24814000	-0.00274700	-4.06482200
	H	-1.27809300	0.10088700	-2.32008600
11	O	2.05452300	-0.88762700	1.35757300
	C	3.37036200	-0.61737000	1.31993900
	C	1.53239600	-1.57642300	2.54323200
	H	2.09819700	-1.20749400	3.40166700
	C	1.74797900	-3.07655100	2.39025300
	H	1.22147100	-3.45213300	1.50994800
	H	1.36389900	-3.59684300	3.27182700
	O	4.12504000	-0.91117700	2.21252900
	C	3.79116400	0.06422400	-0.03103200
	C	3.01340600	1.40272600	-0.21143800
	C	1.61013100	1.41806800	-0.27818500
	C	0.90770000	2.60407600	-0.46703900
	C	1.58872600	3.81272000	-0.60294800

	C	2.97703600	3.81390800	-0.55431300
	C	3.68026000	2.62326500	-0.36439900
	C	3.44329500	-0.94876200	-1.15241200
	C	3.74984400	-2.30381300	-0.97128800
	C	3.50407900	-3.23717900	-1.97268300
	C	2.95406000	-2.83272300	-3.18840300
	C	2.66103900	-1.48719900	-3.38736500
	C	2.90299000	-0.55383400	-2.37815200
	C	5.31848800	0.31753100	-0.01319700
	C	5.93991800	0.90761000	1.09827700
	C	7.30085200	1.19285500	1.09299700
	C	8.07977100	0.90227300	-0.02582400
	C	7.47702500	0.32797400	-1.13828500
	C	6.11179400	0.03971400	-1.13018000
	H	1.06094100	0.49309300	-0.18620000
	H	-0.17515600	2.57862500	-0.51350300
	H	1.04148500	4.73673900	-0.75291600
	H	3.52756400	4.74132800	-0.66824500
	H	4.75970200	2.65779100	-0.34331700
	H	4.20129300	-2.63217900	-0.04160700
	H	3.74814800	-4.28032200	-1.80480500
	H	2.76378600	-3.55743900	-3.97204900
	H	2.24440500	-1.15455000	-4.33177200
	H	2.67143600	0.48803000	-2.55519900
	H	5.36052400	1.14354200	1.97913000
	H	7.75379300	1.64283000	1.96961300
	H	9.14152300	1.12239600	-0.02746700
	H	8.06436300	0.09758500	-2.02030700
	H	5.66982600	-0.40719000	-2.01024800
	H	2.81014100	-3.30649500	2.29959700
	C	0.06080000	-1.19163200	2.65154000
	H	-0.39024500	-1.82761200	3.42207700
	H	-0.43652700	-1.43632700	1.70937700
	C	-0.17526700	0.27981700	3.00598700
	C	-1.65056100	0.66337800	3.06008200
	H	-2.21398700	-0.04233700	3.67447300
	C	-1.88990100	2.08264700	3.55880800
	H	-2.95640700	2.31437100	3.56287900
	H	-1.36681200	2.80739800	2.92971300
	H	-1.51949500	2.18636200	4.58216300
	O	-2.16056000	0.53991100	1.69166400
	C	-3.45039900	0.19079400	1.53050900
	C	-3.78313500	-0.03118400	0.02386500
	C	-3.44244500	1.20871400	-0.83472200
	C	-3.43574500	1.10178900	-2.23212100
	O	-4.21067300	0.00447900	2.44674700
	C	-3.26935900	2.47909700	-0.27763100
	C	-3.07619700	3.60013100	-1.08497900
	C	-3.05011200	3.47408400	-2.46964000
	C	-3.23371000	2.21585700	-3.03969200
	C	-5.30000800	-0.26408600	-0.18222000
	C	-6.24133500	0.53965500	0.47322600

	C	-7.60111600	0.40836500	0.21173000
	C	-8.05484000	-0.52093400	-0.72180100
	C	-7.13022700	-1.31362100	-1.39277800
	C	-5.76801900	-1.18429700	-1.12596000
	C	-2.93745100	-1.28817800	-0.30992600
	C	-3.32247700	-2.52282400	0.23258900
	C	-2.55364300	-3.66504800	0.03049200
	C	-1.37471500	-3.59570200	-0.70983100
	C	-0.96760900	-2.37116600	-1.23167900
	C	-1.73974500	-1.22758700	-1.02746100
	H	-3.59948000	0.13758100	-2.69724100
	H	-3.28391100	2.61234500	0.79583200
	H	-2.94838300	4.57312300	-0.62338400
	H	-2.89743800	4.34416700	-3.09788800
	H	-3.22866900	2.10041200	-4.11784100
	H	-5.91663800	1.26861300	1.20224800
	H	-8.30835600	1.03716200	0.74122700
	H	-9.11512700	-0.62240400	-0.92430100
	H	-7.46326900	-2.03808200	-2.12784300
	H	-5.06903000	-1.81482400	-1.66021000
	H	-4.23176500	-2.58877900	0.81817000
	H	-2.87698100	-4.60913700	0.45489300
	H	-0.77587800	-4.48487400	-0.87190100
	H	-0.04107600	-2.29663300	-1.78969700
	H	-1.39616800	-0.28325100	-1.42689800
	H	0.33188300	0.92869800	2.28688400
	H	0.25920700	0.49507000	3.98935500
12	O	-2.68586600	1.98294100	0.02549000
	C	-2.73398700	0.82482800	0.70402400
	C	-1.68236600	2.97313000	0.41883900
	H	-1.51466200	2.85913900	1.49178800
	C	-2.29827600	4.32826800	0.10767100
	H	-1.62735700	5.13352500	0.41457100
	H	-3.24169200	4.45324800	0.64259800
	O	-2.04526600	0.58231200	1.66494600
	C	-3.69843000	-0.18585800	0.01947000
	C	-2.86195600	-0.58068300	-1.22784600
	C	-1.74805900	-1.41688500	-1.06651300
	C	-0.90876600	-1.71021700	-2.13796700
	C	-1.16439000	-1.16622100	-3.39500400
	C	-2.25492600	-0.31805600	-3.56330900
	C	-3.08946800	-0.01853800	-2.48754600
	C	-5.07729600	0.42945900	-0.31138300
	C	-5.57396000	1.54927600	0.36219300
	C	-6.86645900	2.01590100	0.12856000
	C	-7.69361800	1.36991000	-0.78400800
	C	-7.21791700	0.24355900	-1.45185400
	C	-5.92957400	-0.22301900	-1.21173900
	C	-4.00045300	-1.38547500	0.94788800
	C	-4.16608100	-2.67310300	0.42714100
	C	-4.55188000	-3.73545700	1.24241500
	C	-4.78417700	-3.53077100	2.59853500

	C	-4.63417200	-2.25099100	3.12757900
	C	-4.25238900	-1.19087100	2.31137100
	H	-1.53206600	-1.83928200	-0.09276000
	H	-0.05069200	-2.35536300	-1.98760000
	H	-0.51148300	-1.39360900	-4.22994500
	H	-2.45699800	0.12344300	-4.53309200
	H	-3.91647300	0.66317100	-2.63255900
	H	-4.95521400	2.07320300	1.07858900
	H	-7.22346400	2.88810300	0.66504200
	H	-8.69804500	1.73376500	-0.96830200
	H	-7.85296600	-0.27991800	-2.15797200
	H	-5.58618000	-1.11054200	-1.72859800
	H	-3.98886400	-2.85678900	-0.62478700
	H	-4.66942800	-4.72368200	0.81154600
	H	-5.08084800	-4.35639700	3.23559600
	H	-4.81556100	-2.07346300	4.18189500
	H	-4.14375300	-0.20745800	2.74733500
	H	-2.49516300	4.42765200	-0.96289200
	C	-0.38831000	2.66627500	-0.33795700
	H	-0.58508300	2.73710700	-1.41354700
	H	-0.11303800	1.63134400	-0.12799500
	C	0.78223700	3.57899900	0.04691500
	C	2.10022900	3.18972700	-0.61796800
	H	1.95835200	3.01465400	-1.68636200
	C	3.20703800	4.21504600	-0.40686300
	H	4.13279400	3.88248100	-0.87825200
	H	3.38597000	4.37622000	0.65964200
	H	2.92285700	5.16985500	-0.85721100
	O	2.50638400	1.91634400	-0.01926200
	C	3.25982300	1.09155300	-0.76554800
	C	3.70232400	-0.20948300	-0.01204300
	C	3.29841200	-1.46084400	-0.84991500
	C	2.90787700	-2.64237100	-0.20543800
	O	3.60508800	1.36100700	-1.89026700
	C	3.40189900	-1.48495000	-2.24837800
	C	3.09934000	-2.63755500	-2.96987300
	C	2.70153600	-3.80141200	-2.31659700
	C	2.61322000	-3.79776600	-0.92805200
	C	3.07019200	-0.29126100	1.39797700
	C	1.67357500	-0.31607500	1.52919400
	C	1.06254800	-0.41872800	2.77219300
	C	1.84286300	-0.51615900	3.92569800
	C	3.22718800	-0.51123900	3.81331600
	C	3.83565800	-0.39967200	2.56025900
	C	5.24881100	-0.10219800	0.07508600
	C	5.83579300	1.10249500	0.48614400
	C	7.21534100	1.22614900	0.60863200
	C	8.04323200	0.14037400	0.32779500
	C	7.47303800	-1.06251400	-0.07350600
	C	6.08854400	-1.18245200	-0.20104800
	H	2.83307800	-2.66976800	0.87275500
	H	3.72511100	-0.59896800	-2.77369200

	H	3.18514700	-2.62259700	-4.05104500
	H	2.47099200	-4.69856500	-2.88043400
	H	2.31514500	-4.69502000	-0.39676900
	H	1.05152700	-0.26024700	0.64457500
	H	-0.01930500	-0.41453700	2.83109400
	H	1.37114700	-0.59848900	4.89867800
	H	3.84748000	-0.59492800	4.69912300
	H	4.91545800	-0.40282400	2.50164700
	H	5.20732400	1.95217400	0.72787900
	H	7.64330000	2.17120000	0.92436200
	H	9.11940700	0.23379900	0.42029900
	H	8.10358800	-1.91643100	-0.29536000
	H	5.66829700	-2.12622400	-0.52158700
	H	0.92782700	3.57179200	1.13311500
	H	0.56795800	4.61364000	-0.23985500
13	O	-2.48677300	1.86591400	0.02652400
	C	-2.79711300	0.80698400	0.79183500
	C	-1.49090100	2.82528000	0.51024600
	H	-1.31409600	2.60919600	1.56484400
	C	-2.11169700	4.20394500	0.33888600
	H	-3.05142600	4.26917100	0.89085600
	H	-2.31705100	4.40959100	-0.71491600
	O	-2.34419400	0.61657500	1.89495500
	C	-3.70619700	-0.20017200	0.03003300
	C	-4.60154900	0.49710600	-1.02064300
	C	-4.80104600	-0.02460100	-2.30078000
	C	-5.69710800	0.57281000	-3.18827500
	C	-6.41385000	1.70231100	-2.81081100
	C	-6.23451100	2.22468300	-1.53037300
	C	-5.34467900	1.62461700	-0.64795700
	C	-4.68195700	-0.95335500	0.96707300
	C	-4.94573800	-0.57855300	2.28750700
	C	-5.90642100	-1.25323200	3.04315500
	C	-6.61990900	-2.31440500	2.49950500
	C	-6.36899800	-2.69574700	1.18224400
	C	-5.41800800	-2.01914600	0.42822500
	C	-2.62347900	-1.13417900	-0.57838100
	C	-2.30560600	-2.36184200	0.01098400
	C	-1.25387800	-3.13594300	-0.47697400
	C	-0.48665500	-2.69033400	-1.54926000
	C	-0.77986800	-1.45860600	-2.13137500
	C	-1.83445400	-0.68907400	-1.64920400
	H	-4.25661200	-0.90451000	-2.61797500
	H	-5.83105700	0.14745100	-4.17685500
	H	-7.10827000	2.16846200	-3.50065700
	H	-6.79287800	3.09975300	-1.21636200
	H	-5.23360600	2.03620300	0.34867200
	H	-4.39039500	0.22521700	2.74644200
	H	-6.08877200	-0.94222800	4.06604200
	H	-7.36333400	-2.83760400	3.09038800
	H	-6.91908800	-3.51769400	0.73749500
	H	-5.24743900	-2.32195700	-0.59772900

	H	-2.87399800	-2.71465000	0.86079900
	H	-1.02761200	-4.08531900	-0.00429600
	H	0.34470600	-3.28199100	-1.91354900
	H	-0.17243400	-1.08424100	-2.94678900
	H	-2.03776200	0.27468000	-2.09759800
	H	-1.44330100	4.97809000	0.72215500
	C	-0.21229500	2.58629200	-0.29656600
	H	-0.44347400	2.69833500	-1.36152800
	H	0.09037500	1.54803100	-0.14599700
	C	0.94203900	3.52103800	0.08413000
	C	2.25256200	3.22727000	-0.65110600
	H	2.05993600	3.03354900	-1.70779800
	C	3.28077500	4.33821400	-0.48229400
	H	4.21350300	4.08818800	-0.99114600
	H	3.50202100	4.50252000	0.57521000
	H	2.90026500	5.27055800	-0.90725400
	O	2.85209000	2.02018600	-0.08573700
	C	2.84642300	0.87640200	-0.79656300
	C	3.57401100	-0.25123200	0.00281300
	C	2.90292800	-0.22586500	1.39902400
	C	1.54222600	-0.55422200	1.49180500
	O	2.33660800	0.76515600	-1.88268100
	C	3.55830900	0.20493900	2.55328100
	C	2.88151000	0.28067000	3.77120900
	C	1.53354500	-0.05229100	3.85116100
	C	0.86073600	-0.46500100	2.70148900
	C	5.09722600	0.03141200	0.04559400
	C	5.69880000	1.07362900	-0.66322600
	C	7.08307100	1.24968000	-0.65112700
	C	7.89513200	0.38604500	0.07372900
	C	7.30991000	-0.66567300	0.77774300
	C	5.93166100	-0.84397400	0.75550500
	C	3.42516500	-1.62305700	-0.70271100
	C	3.15709200	-2.78967400	0.02027700
	C	3.14131400	-4.03752300	-0.60338400
	C	3.39188800	-4.14450200	-1.96680200
	C	3.66965700	-2.99027200	-2.69769600
	C	3.69597300	-1.74808600	-2.07280900
	H	1.00529700	-0.88339600	0.60866900
	H	4.59807800	0.49846300	2.50867400
	H	3.41393200	0.61353700	4.65567300
	H	1.00641100	0.01656000	4.79615500
	H	-0.19693900	-0.69682900	2.73676900
	H	5.09736900	1.76166100	-1.24207200
	H	7.52104200	2.06654300	-1.21406200
	H	8.97048300	0.52274300	0.08517300
	H	7.92912100	-1.35662000	1.33904000
	H	5.49792900	-1.68022600	1.28993700
	H	2.95654600	-2.73399500	1.08182600
	H	2.93328800	-4.92429000	-0.01464200
	H	3.37996600	-5.11319200	-2.45405000
	H	3.87692600	-3.05632400	-3.76019200

	H	3.92492200	-0.86967500	-2.65864900
	H	1.13824700	3.46926000	1.16104700
	H	0.67111100	4.55773400	-0.14106700
15	O	-2.27917200	0.45301300	-0.41898900
	C	-3.12867600	0.45555700	0.62252100
	C	-0.96390200	1.07773400	-0.22406600
	H	-0.62554700	0.79986900	0.77600900
	C	-1.10131400	2.59196700	-0.31255300
	H	-0.12299500	3.05679800	-0.16861600
	H	-1.77641600	2.96558800	0.45771400
	O	-2.83972800	0.87189500	1.71705100
	C	-4.54839900	-0.02312500	0.19955700
	C	-5.37397800	-0.59420800	1.37944300
	C	-6.74560500	-0.80904100	1.17865700
	C	-7.53563900	-1.40311900	2.15544600
	C	-6.96945800	-1.81008100	3.36240200
	C	-5.60981800	-1.61290700	3.57131100
	C	-4.81753800	-1.01163800	2.59203700
	C	-4.48013900	-1.17145100	-0.83361100
	C	-3.60976300	-2.24389600	-0.60050100
	C	-3.58523500	-3.35034300	-1.44151300
	C	-4.44440700	-3.41750400	-2.53734500
	C	-5.32905600	-2.37052200	-2.76917100
	C	-5.34915800	-1.26080900	-1.92370600
	C	-5.14329000	1.30465200	-0.34420300
	C	-4.81306400	1.76934600	-1.62488700
	C	-5.27397300	3.00160700	-2.07995900
	C	-6.05788600	3.80729100	-1.25725000
	C	-6.36471100	3.37207400	0.02828500
	C	-5.90694700	2.13675700	0.48213400
	H	-7.20341200	-0.50970100	0.24358800
	H	-8.59368600	-1.55262200	1.97051500
	H	-7.58170800	-2.27554200	4.12661700
	H	-5.15211300	-1.92257400	4.50444300
	H	-3.77023300	-0.84927800	2.79449300
	H	-2.95187400	-2.22309100	0.26132500
	H	-2.89969400	-4.16530200	-1.23675000
	H	-4.42834400	-4.27927300	-3.19482100
	H	-6.01313900	-2.41183800	-3.60965700
	H	-6.05316200	-0.46360800	-2.12356100
	H	-4.18486000	1.16756400	-2.26782800
	H	-5.01364600	3.33342400	-3.07906900
	H	-6.41677600	4.76706000	-1.61162500
	H	-6.95897400	3.99495600	0.68750400
	H	-6.14474900	1.82102900	1.48901200
	H	-1.48458400	2.88665000	-1.29282300
	C	-0.05357300	0.50217600	-1.30462500
	H	0.90172400	1.03255800	-1.25911800
	H	-0.49606000	0.73635000	-2.27842600
	C	0.19331500	-1.00990100	-1.21854700
	C	1.09044500	-1.45409000	-0.06716600
	H	0.77070200	-1.02270400	0.88349600

	C	1.17784700	-2.96933100	0.06976200
	H	1.85650500	-3.24616200	0.87765000
	H	1.52296200	-3.42377700	-0.86294400
	H	0.19126500	-3.37794800	0.30359800
	O	2.42049100	-0.90909700	-0.35629000
	C	3.24572500	-0.68433300	0.68194000
	C	4.55698400	0.01795900	0.21763100
	C	5.25770500	-0.72738200	-0.94233200
	C	6.30937100	-0.09924800	-1.62412900
	O	2.95720500	-0.92324400	1.82743100
	C	4.97475200	-2.05746200	-1.26483000
	C	5.70055700	-2.73061300	-2.24717000
	C	6.72649500	-2.08712200	-2.92979000
	C	7.02925200	-0.76497700	-2.61004300
	C	5.59470300	0.05613900	1.36591900
	C	5.82714100	-1.08038500	2.15049500
	C	6.83002000	-1.09312000	3.11393500
	C	7.63496300	0.02699600	3.31035500
	C	7.42624100	1.15691700	2.52731700
	C	6.41779800	1.16912800	1.56455200
	C	4.03764200	1.42908500	-0.16517200
	C	3.61709700	2.29654200	0.85327300
	C	3.08247600	3.54646400	0.55467700
	C	2.94587100	3.95289900	-0.77166000
	C	3.33247700	3.08824900	-1.79152500
	C	3.86541100	1.83503200	-1.49147600
	H	6.57598200	0.92083600	-1.37645700
	H	4.18091900	-2.58604100	-0.75563500
	H	5.45736900	-3.76247400	-2.47542000
	H	7.28905400	-2.60902800	-3.69554300
	H	7.83456000	-0.25097800	-3.12309500
	H	5.21659800	-1.96287800	2.01900700
	H	6.98149800	-1.98376200	3.71367600
	H	8.41576000	0.01640000	4.06248500
	H	8.04687300	2.03614400	2.66079000
	H	6.27539300	2.06137500	0.96855200
	H	3.71017600	1.98951900	1.88804200
	H	2.77318400	4.20221600	1.36094600
	H	2.53696500	4.92928000	-1.00609900
	H	3.21859300	3.38432300	-2.82852900
	H	4.14388600	1.17175800	-2.29900000
	H	0.65300700	-1.35209700	-2.15148800
	H	-0.76394100	-1.53132100	-1.13307800
17	O	2.28125200	-1.83573000	-0.10294300
	C	2.50823100	-0.79644400	0.71894200
	C	1.77413600	-3.08338800	0.47394400
	H	2.03797800	-3.07165600	1.53267400
	C	2.52235900	-4.19953300	-0.24148200
	H	3.59606000	-4.10337200	-0.07131300
	H	2.34770100	-4.17156200	-1.31875400
	O	2.15482100	-0.76455600	1.87233100
	C	3.39594300	0.28008600	0.02425300

	C	3.45082000	1.58560800	0.85206200
	C	4.62390200	2.34456900	0.92384500
	C	4.64576800	3.57773900	1.57303000
	C	3.49073000	4.08171200	2.16166400
	C	2.31221400	3.34327500	2.08422900
	C	2.29194800	2.11423800	1.43280800
	C	2.84925100	0.69680200	-1.35929700
	C	1.50067600	0.54793800	-1.69281400
	C	0.99152100	1.03113600	-2.89675800
	C	1.83043900	1.68109900	-3.79607700
	C	3.17496700	1.85342100	-3.47223200
	C	3.67535800	1.37422900	-2.26543400
	C	4.76061200	-0.45894300	-0.02181200
	C	5.22989200	-1.09424800	-1.17539000
	C	6.41678300	-1.82495800	-1.15596200
	C	7.14755400	-1.95193400	0.02195500
	C	6.67391300	-1.35001400	1.18567700
	C	5.49129100	-0.61630400	1.16438600
	H	5.53570200	1.97474200	0.47273800
	H	5.57080000	4.14232600	1.61462100
	H	3.50619300	5.03933000	2.66991100
	H	1.40000300	3.72366800	2.53017600
	H	1.36103300	1.56754900	1.38240900
	H	0.82086500	0.05225100	-1.01332600
	H	-0.06138300	0.89409500	-3.11336900
	H	1.44054000	2.05815400	-4.73483700
	H	3.83822800	2.37072900	-4.15690700
	H	4.71869700	1.53776200	-2.02499700
	H	4.66475000	-1.02507300	-2.09470100
	H	6.76505700	-2.29996000	-2.06656100
	H	8.07121600	-2.51963400	0.03569400
	H	7.22339100	-1.45184300	2.11496900
	H	5.12842900	-0.16362400	2.07906900
	H	2.19645500	-5.17142400	0.13925600
	C	0.24681500	-3.15201700	0.36721100
	H	-0.17904400	-2.37255300	1.00193700
	H	-0.05486100	-4.10647700	0.81296700
	C	-0.31229800	-3.02489900	-1.05578100
	C	-1.84135500	-3.06200900	-1.15835600
	H	-2.11449700	-2.86623700	-2.19671800
	C	-2.48141200	-4.35782900	-0.68039100
	H	-3.55534000	-4.35164500	-0.87690700
	H	-2.33187100	-4.50631700	0.39066100
	H	-2.04432400	-5.20639900	-1.21316900
	O	-2.45007200	-1.99786900	-0.35099000
	C	-2.57747800	-0.77970600	-0.91046600
	C	-3.39339400	0.20245300	0.00209100
	C	-4.90504400	-0.14363700	-0.16636500
	C	-5.86332100	0.86362900	-0.32956600
	O	-2.12753800	-0.51200400	-1.99685900
	C	-5.36055900	-1.46756800	-0.08126200
	C	-6.71501900	-1.77178100	-0.18443500

	C	-7.65481600	-0.75981500	-0.36210200
	C	-7.22088800	0.55964500	-0.42599500
	C	-3.12073200	1.65667200	-0.46135800
	C	-3.31597800	2.02575700	-1.80217800
	C	-3.11450000	3.33551000	-2.22244200
	C	-2.72244200	4.31759300	-1.31389200
	C	-2.54426000	3.97202200	0.01954700
	C	-2.74309700	2.65592800	0.44022600
	C	-2.94582800	0.00549700	1.46892200
	C	-1.57804000	-0.03084100	1.77102000
	C	-1.12514600	-0.15939400	3.07909500
	C	-2.04438300	-0.24130400	4.12549400
	C	-3.40502400	-0.19330900	3.84366000
	C	-3.85272400	-0.07309700	2.52638100
	H	-5.55560300	1.89848800	-0.37896100
	H	-4.65302800	-2.26551700	0.08846500
	H	-7.03472700	-2.80609800	-0.11840300
	H	-8.70974600	-0.99676200	-0.44244600
	H	-7.93701300	1.36425200	-0.55147700
	H	-3.62780200	1.28456100	-2.52340800
	H	-3.26655500	3.58922100	-3.26583200
	H	-2.56299300	5.33804900	-1.64383800
	H	-2.24836600	4.72280500	0.74378100
	H	-2.60331300	2.41809000	1.48539300
	H	-0.85100500	0.05242900	0.97084900
	H	-0.05889300	-0.20138400	3.26970100
	H	-1.69930500	-0.34136400	5.14845000
	H	-4.12999200	-0.25209200	4.64814700
	H	-4.91644700	-0.04056800	2.33138800
	H	0.06026400	-3.83822300	-1.68855900
	H	0.04072500	-2.09896500	-1.51263800
26	O	-2.40627900	-1.74068000	-1.01873700
	C	-2.93039100	-0.54541500	-1.35107200
	C	-1.99189200	-2.61032100	-2.12299300
	H	-1.58057600	-1.96545100	-2.90200600
	C	-3.21635300	-3.34597100	-2.65632200
	H	-3.97078100	-2.63720900	-2.99895900
	H	-3.65391400	-3.98273300	-1.88265800
	O	-2.99464500	-0.15428000	-2.49025800
	C	-3.46892000	0.27864300	-0.13770600
	C	-5.02841100	0.24626400	-0.21112600
	C	-5.79708200	0.25550200	0.95993700
	C	-7.18908100	0.30346000	0.90949900
	C	-7.84809200	0.34268600	-0.31512100
	C	-7.09636900	0.35022300	-1.48664700
	C	-5.70485000	0.31152500	-1.43701600
	C	-2.97029200	1.73383000	-0.33344900
	C	-3.77779400	2.83293000	-0.03196800
	C	-3.29016500	4.13415600	-0.15467200
	C	-1.98675200	4.35932200	-0.58468900
	C	-1.17166700	3.27016000	-0.88770400
	C	-1.65808500	1.97269200	-0.76202300

	C	-2.97870700	-0.30620800	1.21110500
	C	-2.13703000	0.40599800	2.06799100
	C	-1.73007500	-0.13044300	3.29244800
	C	-2.16439200	-1.38857500	3.68827200
	C	-3.00882600	-2.11215400	2.84467500
	C	-3.40680800	-1.57838700	1.62586800
	H	-5.31013400	0.23131500	1.92521600
	H	-7.75479300	0.31041500	1.83478100
	H	-8.93114200	0.37403400	-0.35603400
	H	-7.59122600	0.39428600	-2.45070000
	H	-5.14093400	0.34996800	-2.35830500
	H	-4.79750900	2.68303300	0.29690200
	H	-3.93878300	4.97051900	0.08213200
	H	-1.61004900	5.37111500	-0.68681200
	H	-0.15336300	3.42165300	-1.22734300
	H	-0.99213600	1.15083800	-0.99359300
	H	-1.79135600	1.39153500	1.78969000
	H	-1.07150000	0.44663900	3.93157300
	H	-1.85455000	-1.80331500	4.64108200
	H	-3.36178000	-3.09457300	3.13959300
	H	-4.06692900	-2.15278900	0.98919700
	H	-2.93473300	-3.97678600	-3.50359300
	C	-0.91862000	-3.55879800	-1.59386200
	H	-0.77090500	-4.31361000	-2.37328100
	H	-1.31243100	-4.09664900	-0.72584000
	C	0.45302300	-2.93111600	-1.28500600
	C	0.58291200	-2.22303100	0.06784500
	H	-0.14723000	-1.42059300	0.15264200
	C	0.50767400	-3.14937900	1.26993400
	H	0.67563600	-2.58581700	2.18859000
	H	1.26168500	-3.93812800	1.19842600
	H	-0.48039900	-3.60733100	1.33750700
	O	1.91898300	-1.61542400	0.13551400
	C	2.08045100	-0.38421700	-0.36398100
	C	3.48237600	0.19106300	-0.00588700
	C	3.26454900	0.56886700	1.48441600
	C	2.58138800	1.74911900	1.80584800
	O	1.21158700	0.23114100	-0.93462900
	C	3.60645500	-0.30076900	2.52587000
	C	3.32151900	0.02405300	3.85053800
	C	2.66992900	1.21498500	4.15981100
	C	2.29246500	2.07234500	3.12902500
	C	3.84143500	1.41155400	-0.88666600
	C	3.47546900	1.47352800	-2.23609800
	C	3.90693600	2.51911100	-3.04841200
	C	4.72251500	3.52325000	-2.53417100
	C	5.10559100	3.46691600	-1.19742800
	C	4.67119900	2.42177200	-0.38611100
	C	4.62287800	-0.82519400	-0.24349600
	C	4.52373800	-1.81779700	-1.22393800
	C	5.60070200	-2.65097100	-1.51723800
	C	6.80669100	-2.50786000	-0.83793300

	C	6.92571600	-1.51387900	0.12994000
	C	5.84862300	-0.68038600	0.41748300
	H	2.27067500	2.42027400	1.01527700
	H	4.09374200	-1.24064100	2.30457400
	H	3.60735200	-0.66150200	4.64068500
	H	2.45242200	1.46881900	5.19129100
	H	1.76964600	2.99604700	3.35069500
	H	2.83877800	0.71130700	-2.66173700
	H	3.60076900	2.54566400	-4.08834800
	H	5.05677700	4.33731300	-3.16744000
	H	5.74539600	4.23666500	-0.78003300
	H	4.98241800	2.39987500	0.65059000
	H	3.60052700	-1.95117200	-1.77282200
	H	5.49296200	-3.41348700	-2.28080400
	H	7.64463700	-3.15747400	-1.06393500
	H	7.86137100	-1.37993500	0.66150300
	H	5.96947500	0.09434200	1.16405400
	H	0.69808100	-2.21463500	-2.07460500
	H	1.21613800	-3.71640000	-1.31889600
31	O	-2.24458300	1.41059900	0.80254200
	C	-3.44542200	0.89802500	1.12980800
	C	-1.72815900	2.49653000	1.63923300
	H	-1.97015800	2.24536000	2.67392100
	C	-2.42736500	3.80076700	1.26803700
	H	-2.32673600	4.01841700	0.20327800
	H	-1.99325000	4.62793800	1.83679600
	O	-4.06519100	1.22920800	2.10908000
	C	-3.95905000	-0.08081700	0.03296000
	C	-4.20923500	0.88607600	-1.15425500
	C	-3.33103700	0.99144300	-2.23641500
	C	-3.54332600	1.93655400	-3.23911800
	C	-4.62801000	2.80575500	-3.17105500
	C	-5.49568900	2.72866100	-2.08358600
	C	-5.28510500	1.78254100	-1.08479200
	C	-5.24564100	-0.80524500	0.49919700
	C	-5.33213300	-1.34063800	1.79056300
	C	-6.43633600	-2.08973300	2.18280000
	C	-7.47787800	-2.33377700	1.29022600
	C	-7.39800500	-1.82190400	-0.00016900
	C	-6.29252500	-1.06732900	-0.39035600
	C	-2.94934000	-1.20900400	-0.27684400
	C	-3.15355800	-2.02228600	-1.39963100
	C	-2.33279500	-3.11488600	-1.65697200
	C	-1.29203300	-3.43221500	-0.78614500
	C	-1.08713200	-2.64456400	0.34172000
	C	-1.90710100	-1.54420300	0.59197500
	H	-2.47015400	0.33973100	-2.30048600
	H	-2.85139700	1.99294400	-4.07217000
	H	-4.79261800	3.53913500	-3.95246800
	H	-6.33881900	3.40646400	-2.00899900
	H	-5.96241500	1.74039600	-0.24007900
	H	-4.53769300	-1.16671700	2.50266800

	H	-6.48081100	-2.48472900	3.19174100
	H	-8.33805700	-2.91801500	1.59730400
	H	-8.19548200	-2.00694300	-0.71143400
	H	-6.25447500	-0.67904100	-1.39986500
	H	-3.97081000	-1.80553600	-2.07625300
	H	-2.51370600	-3.72424300	-2.53553700
	H	-0.65240900	-4.28546800	-0.97974800
	H	-0.28517900	-2.88261900	1.03097400
	H	-1.72392300	-0.94923000	1.47723400
	H	-3.48864000	3.74303500	1.51050500
	C	-0.20800600	2.51173200	1.47001300
	H	0.19667300	1.56805800	1.84550400
	H	0.16784800	3.29378700	2.13816500
	C	0.28590200	2.74996100	0.03717200
	C	1.80262800	2.89614700	-0.12099900
	H	2.01686900	3.02278100	-1.18316700
	C	2.43177200	4.02852500	0.67866400
	H	2.33488800	3.86385400	1.75359500
	H	1.94699300	4.97604300	0.42781600
	H	3.49410500	4.11365800	0.44373100
	O	2.48987800	1.67354300	0.30403900
	C	2.77833100	0.76036500	-0.64076400
	C	3.80474200	-0.28757500	-0.11922700
	C	3.79573600	-1.57892600	-0.97028500
	C	4.94453100	-2.37805000	-1.03027900
	O	2.38098600	0.82185900	-1.77693000
	C	2.63237200	-2.05587700	-1.58334400
	C	2.62640700	-3.27939900	-2.25029300
	C	3.77834400	-4.05738400	-2.31340800
	C	4.93949600	-3.60056000	-1.69545300
	C	3.50945600	-0.75014100	1.32459900
	C	2.19246600	-0.86391500	1.78302200
	C	1.91070100	-1.38710500	3.04235700
	C	2.94224800	-1.81854500	3.87230100
	C	4.25591300	-1.73050300	3.42137300
	C	4.53408800	-1.20687900	2.16083700
	C	5.11967100	0.52475600	-0.27441900
	C	5.60697300	1.33393400	0.75892900
	C	6.73332900	2.13206400	0.57134200
	C	7.38336400	2.15438900	-0.65995900
	C	6.88771400	1.38031700	-1.70581700
	C	5.76426300	0.57959400	-1.51678400
	H	5.85905000	-2.04311200	-0.55677300
	H	1.72372800	-1.47243400	-1.56092000
	H	1.71230000	-3.61799300	-2.72507000
	H	3.77216900	-5.00743300	-2.83589200
	H	5.84586900	-4.19508000	-1.72856300
	H	1.37046400	-0.54997900	1.15208900
	H	0.88064700	-1.45738800	3.37471500
	H	2.72409600	-2.22355900	4.85387400
	H	5.07139600	-2.07300700	4.04876000
	H	5.56347500	-1.15722400	1.82946700

	H	5.10132500	1.34714000	1.71507900
	H	7.09820700	2.74118100	1.39104700
	H	8.26064300	2.77473000	-0.80515800
	H	7.37166100	1.40024600	-2.67591700
	H	5.38087300	-0.00332600	-2.34417600
	H	-0.15491300	3.66803000	-0.36613500
	H	-0.05374300	1.93841600	-0.60984100
36	O	-2.17474000	0.72281000	1.31719800
	C	-3.51515200	0.62590800	1.35232200
	C	-1.51921700	1.44027300	2.41462500
	H	-2.01684400	1.13307900	3.33814000
	C	-1.68185800	2.94226200	2.21471000
	H	-1.19487200	3.47787200	3.03421300
	H	-2.73568000	3.22060800	2.20664400
	O	-4.18912600	1.03435000	2.26522100
	C	-4.06883700	0.03674700	0.02211000
	C	-3.25538000	-1.19357900	-0.44047700
	C	-3.09734600	-1.50760500	-1.79363900
	C	-2.47607500	-2.69125900	-2.18880900
	C	-2.00468300	-3.59130700	-1.23761100
	C	-2.16853900	-3.29776700	0.11460500
	C	-2.78978200	-2.11550600	0.50439900
	C	-3.96199700	1.27691300	-0.90534000
	C	-5.00187000	2.21396700	-0.94229700
	C	-4.88499600	3.38494100	-1.68749600
	C	-3.71997100	3.64958800	-2.40211400
	C	-2.66675400	2.73994400	-2.34870400
	C	-2.78227400	1.57144700	-1.59983400
	C	-5.52271700	-0.47908100	0.15442000
	C	-6.29962100	-0.61637600	-1.00418700
	C	-7.57008500	-1.18073300	-0.96016200
	C	-8.09517400	-1.63116000	0.24876200
	C	-7.33139300	-1.51109200	1.40465600
	C	-6.05823000	-0.94447900	1.35990800
	H	-3.46509500	-0.82957500	-2.55284900
	H	-2.36842600	-2.90945300	-3.24561600
	H	-1.52585500	-4.51441300	-1.54388700
	H	-1.81883500	-3.99359000	0.86916000
	H	-2.92449100	-1.91952400	1.56186300
	H	-5.90901200	2.02953900	-0.38161300
	H	-5.70736400	4.09143600	-1.70377900
	H	-3.62969600	4.55872800	-2.98598000
	H	-1.74500400	2.94010200	-2.88353700
	H	-1.94207400	0.89258000	-1.54635400
	H	-5.91004400	-0.27640100	-1.95583900
	H	-8.14780400	-1.26980100	-1.87356800
	H	-9.08568100	-2.07038600	0.28736300
	H	-7.72427800	-1.85663700	2.35456000
	H	-5.49728200	-0.84945200	2.27714400
	H	-1.22228500	3.25609300	1.27464000
	C	-0.06041300	0.99706000	2.41113700
	H	0.45320100	1.57340300	3.18994400

	H	0.39606600	1.27992700	1.45910200
	C	0.14448900	-0.49921400	2.66331000
	C	1.60899600	-0.91146900	2.77359500
	H	2.12408800	-0.31214400	3.52807500
	C	1.79532700	-2.39296400	3.07591400
	H	2.85484200	-2.64065300	3.14791600
	H	1.33967100	-3.00451200	2.29322500
	H	1.32289100	-2.64096600	4.03026500
	O	2.22758200	-0.59842500	1.48314100
	C	3.55528000	-0.38932700	1.46054800
	C	4.07205400	-0.06910300	0.01470300
	C	3.36065900	1.23860500	-0.41814700
	C	2.89218300	1.44107500	-1.71785200
	O	4.23724700	-0.40879700	2.45403900
	C	3.25453000	2.29526400	0.49593600
	C	2.68132700	3.50827200	0.12851900
	C	2.20935800	3.69605900	-1.16990100
	C	2.32244100	2.65936300	-2.09063300
	C	3.72722400	-1.25168800	-0.94106900
	C	4.72345500	-1.93803700	-1.64461300
	C	4.40713000	-2.97928900	-2.51833000
	C	3.08482500	-3.35628700	-2.71624600
	C	2.07855900	-2.67338400	-2.03551800
	C	2.39426600	-1.63584800	-1.16467600
	C	5.60179000	0.16513600	0.06589100
	C	6.44607000	-0.71892000	0.75510100
	C	7.82578000	-0.54529400	0.75095600
	C	8.40275400	0.51384200	0.05223100
	C	7.58083900	1.39160100	-0.64398800
	C	6.19650300	1.21748600	-0.63624500
	H	2.97494100	0.64914000	-2.45034500
	H	3.64096600	2.17542000	1.50235800
	H	2.60960100	4.30948200	0.85566200
	H	1.76448900	4.64136600	-1.45937000
	H	1.97128100	2.79372300	-3.10788900
	H	5.76087500	-1.66266600	-1.52261300
	H	5.20595500	-3.48996600	-3.04478600
	H	2.83871000	-4.16556100	-3.39477300
	H	1.03801000	-2.94126900	-2.18148400
	H	1.59451700	-1.11677200	-0.65877900
	H	6.02618600	-1.54862200	1.30529400
	H	8.45151100	-1.24122800	1.29886300
	H	9.47848100	0.65005700	0.05228300
	H	8.00983300	2.22007600	-1.19678300
	H	5.58194600	1.91614000	-1.18740600
	H	-0.33164800	-1.07931800	1.86832500
	H	-0.34434400	-0.78525400	3.60260100
71	O	1.94095700	-1.40558700	0.12699100
	C	2.22236100	-0.09213600	0.16883000
	C	0.53800800	-1.81400800	0.27480600
	H	-0.07007600	-1.00342600	-0.12142200
	C	0.22344900	-2.01649500	1.74889400

	H	-0.83103100	-2.26588100	1.87920200
	H	0.41359700	-1.09993200	2.30883100
	O	1.38447700	0.75603600	0.34894200
	C	3.74966800	0.17993400	-0.06204400
	C	4.20853200	-0.70730700	-1.24334900
	C	3.45735100	-0.71057100	-2.42787700
	C	3.85118600	-1.46387200	-3.52716700
	C	5.01253100	-2.23483700	-3.46814400
	C	5.76756600	-2.23723900	-2.30140600
	C	5.36872500	-1.47994200	-1.19793500
	C	4.53320200	-0.17087000	1.23783500
	C	4.22588100	-1.30008100	2.00942600
	C	4.96994600	-1.62211200	3.14228100
	C	6.05015900	-0.83352700	3.52684900
	C	6.37936900	0.27981000	2.76027100
	C	5.62946800	0.60728600	1.63246800
	C	3.93596700	1.68268600	-0.39223300
	C	4.63951800	2.11247800	-1.52022300
	C	4.83735100	3.47152600	-1.77325700
	C	4.33696500	4.42838500	-0.89964600
	C	3.64484700	4.01375100	0.23812300
	C	3.45166300	2.66076000	0.49067100
	H	2.55922200	-0.10522900	-2.49630400
	H	3.25268000	-1.44750300	-4.43137500
	H	5.32211200	-2.82459900	-4.32362100
	H	6.67325400	-2.83059500	-2.24035300
	H	5.97059200	-1.49934900	-0.29908700
	H	3.41082800	-1.94476500	1.71663600
	H	4.70158500	-2.49836100	3.72233300
	H	6.62850800	-1.08471200	4.40885700
	H	7.22197000	0.90365900	3.03749000
	H	5.90627900	1.47989700	1.05699900
	H	5.04581800	1.38962700	-2.21385100
	H	5.38640400	3.77323700	-2.65839300
	H	4.48659100	5.48422400	-1.09615900
	H	3.25462100	4.74714700	0.93524800
	H	2.91829700	2.36261000	1.38186100
	H	0.83089500	-2.82319100	2.16943600
	C	0.40785000	-3.06651000	-0.59031800
	H	1.03503900	-3.85282600	-0.15633200
	H	0.83525900	-2.84060800	-1.57244500
	C	-1.01708600	-3.61120900	-0.77807200
	C	-1.98146700	-2.70437000	-1.54599300
	H	-1.45385500	-2.12789700	-2.30820800
	C	-3.13700100	-3.46591500	-2.18479900
	H	-3.67983500	-4.04741700	-1.43479300
	H	-2.75729800	-4.15275400	-2.94577800
	H	-3.83304600	-2.77824500	-2.66750600
	O	-2.53757800	-1.74820900	-0.58306200
	C	-2.92472700	-0.54658000	-1.04984000
	C	-3.69440500	0.32500700	-0.00446400
	C	-3.18112700	1.77782400	-0.16910100

	C -4.04942400 2.87152600 -0.16145300<br>O -2.72037100 -0.18684000 -2.18296700<br>C -1.80613700 2.02702300 -0.27127600<br>C -1.31544400 3.32462300 -0.36767900<br>C -2.19319700 4.40744600 -0.36330000<br>C -3.56003600 4.17463500 -0.25734800<br>C -3.47958700 -0.18849600 1.44301400<br>C -3.94859600 -1.45815300 1.81968700<br>C -3.79205600 -1.93179300 3.11616500<br>C -3.16303200 -1.14525900 4.08227500<br>C -2.70217200 0.11614500 3.72928000<br>C -2.86119800 0.58963500 2.42440600<br>C -5.20599300 0.25232300 -0.38959900<br>C -6.19784500 0.28390700 0.59870000<br>C -7.55064800 0.29193800 0.26282700<br>C -7.94496900 0.26790700 -1.07106200<br>C -6.96954700 0.25309800 -2.06414600<br>C -5.61750200 0.25413200 -1.72961100<br>H -5.11727500 2.71585500 -0.08569400<br>H -1.09263400 1.21249100 -0.26242400<br>H -0.24543500 3.47989000 -0.44370500<br>H -1.81395700 5.42024400 -0.44299200<br>H -4.25636900 5.00612200 -0.25353000<br>H -4.45041800 -2.07906900 1.08998600<br>H -4.16788300 -2.91580100 3.37511700<br>H -3.04276600 -1.51202300 5.09558200<br>H -2.21788800 0.74679100 4.46660600<br>H -2.49933400 1.57865300 2.18206500<br>H -5.91852700 0.30798700 1.64318100<br>H -8.29420800 0.31745600 1.05186400<br>H -8.99700900 0.26796100 -1.33341500<br>H -7.25716700 0.24803300 -3.10981300<br>H -4.87780400 0.27364800 -2.51784500<br>H -1.47481300 -3.87228200 0.18125500<br>H -0.93502200 -4.54725300 -1.33951100

22 (optimized at the B3LYP-GD3BJ/6-311G(d,p) level)

Conformer no	
1	O -0.89732700 1.97856600 -1.52191500<br>C -0.40953000 3.22365300 -1.43556000<br>C -1.28514800 1.46287200 -2.83020700<br>H -0.79643900 2.08686300 -3.58050300<br>C -2.79532800 1.57285600 -2.95855400<br>H -3.11886800 1.18116500 -3.92613500<br>H -3.11027800 2.61645400 -2.89275000<br>O -0.30265400 3.97972200 -2.36726200<br>C 0.07321400 3.49071800 0.01306500<br>C -1.05232000 3.23560500 1.03030100<br>C -0.74316800 3.13046100 2.39028500<br>C -1.74317500 2.96920100 3.34266200<br>C -3.08147600 2.93433900 2.95653200

	C	-3.40345300	3.07240300	1.61048600
	C	-2.39771600	3.22025900	0.65800300
	C	0.48485900	4.95782300	0.21782200
	C	-0.30265400	5.99431600	-0.29227400
	C	0.00210300	7.32176300	-0.01344000
	C	1.09258000	7.64073000	0.79248600
	C	1.87224800	6.61672400	1.31910300
	C	1.56893800	5.28736900	1.03447600
	C	1.26743400	2.51865000	0.13289600
	C	2.43045200	2.79280400	-0.59714700
	C	3.49758600	1.90026000	-0.59477200
	C	3.41604600	0.71186900	0.12930900
	C	2.25555300	0.41952200	0.83757400
	C	1.19010900	1.31570300	0.83259700
	H	0.29068800	3.17679500	2.70762800
	H	-1.47581600	2.87872900	4.38911000
	H	-3.86244400	2.81016100	3.69731000
	H	-4.44020100	3.06082100	1.29419100
	H	-2.67703100	3.31319800	-0.38223500
	H	-1.15295500	5.76641100	-0.91858500
	H	-0.61654400	8.10964400	-0.42758000
	H	1.32883400	8.67612400	1.00896000
	H	2.72040600	6.84750800	1.95353500
	H	2.18661800	4.50294200	1.45139000
	H	2.49510800	3.70946200	-1.17048100
	H	4.39098800	2.13292600	-1.16286300
	H	4.24497500	0.01367100	0.13581300
	H	2.17402000	-0.51302600	1.38017200
	H	0.28729000	1.06932000	1.36823000
	H	-3.28475700	0.99939700	-2.16926800
	C	-0.76365300	0.03519900	-2.88146800
	H	-1.15935700	-0.44418200	-3.78376600
	H	-1.16327700	-0.51003200	-2.02381100
	C	0.76365300	-0.03519900	-2.88146800
	C	1.28514800	-1.46287200	-2.83020700
	H	0.79643900	-2.08686300	-3.58050300
	C	2.79532800	-1.57285600	-2.95855400
	H	3.11886800	-1.18116500	-3.92613500
	H	3.11027800	-2.61645400	-2.89275000
	H	3.28475700	-0.99939700	-2.16926800
	O	0.89732700	-1.97856600	-1.52191500
	C	0.40953000	-3.22365300	-1.43556000
	C	-0.07321400	-3.49071800	0.01306500
	C	1.05232000	-3.23560500	1.03030100
	C	0.74316800	-3.13046100	2.39028500
	O	0.30265400	-3.97972200	-2.36726200
	C	2.39771600	-3.22025900	0.65800300
	C	3.40345300	-3.07240300	1.61048600
	C	3.08147600	-2.93433900	2.95653200
	C	1.74317500	-2.96920100	3.34266200
	C	-0.48485900	-4.95782300	0.21782200
	C	0.30265400	-5.99431600	-0.29227400

	C	-0.00210300	-7.32176300	-0.01344000
	C	-1.09258000	-7.64073000	0.79248600
	C	-1.87224800	-6.61672400	1.31910300
	C	-1.56893800	-5.28736900	1.03447600
	C	-1.26743400	-2.51865000	0.13289600
	C	-2.43045200	-2.79280400	-0.59714700
	C	-3.49758600	-1.90026000	-0.59477200
	C	-3.41604600	-0.71186900	0.12930900
	C	-2.25555300	-0.41952200	0.83757400
	C	-1.19010900	-1.31570300	0.83259700
	H	-0.29068800	-3.17679500	2.70762800
	H	2.67703100	-3.31319800	-0.38223500
	H	4.44020100	-3.06082100	1.29419100
	H	3.86244400	-2.81016100	3.69731000
	H	1.47581600	-2.87872900	4.38911000
	H	1.15295500	-5.76641100	-0.91858500
	H	0.61654400	-8.10964400	-0.42758000
	H	-1.32883400	-8.67612400	1.00896000
	H	-2.72040600	-6.84750800	1.95353500
	H	-2.18661800	-4.50294200	1.45139000
	H	-2.49510800	-3.70946200	-1.17048100
	H	-4.39098800	-2.13292600	-1.16286300
	H	-4.24497500	-0.01367100	0.13581300
	H	-2.17402000	0.51302600	1.38017200
	H	-0.28729000	-1.06932000	1.36823000
	H	1.16327700	0.51003200	-2.02381100
	H	1.15935700	0.44418200	-3.78376600
3	O	-1.97673400	-0.90243500	-1.52176600
	C	-3.22277300	-0.41704000	-1.43556200
	C	-1.45990300	-1.28914300	-2.82995900
	H	-2.08487200	-0.80196200	-3.58042800
	C	-1.56643700	-2.79960000	-2.95814500
	H	-1.17378100	-3.12236000	-3.92559700
	H	-2.60933600	-3.11694500	-2.89254200
	O	-3.97896900	-0.31163400	-2.36731700
	C	-3.49097600	0.06527700	0.01302400
	C	-3.23309200	-1.05934800	1.03052500
	C	-3.12814200	-0.74962600	2.39041000
	C	-2.96469300	-1.74904400	3.34301000
	C	-2.92733300	-3.08739100	2.95723100
	C	-3.06516000	-3.40998900	1.61131500
	C	-3.21526600	-2.40479100	0.65860600
	C	-4.95908600	0.47334800	0.21772200
	C	-5.99365600	-0.31658800	-0.29256400
	C	-7.32184200	-0.01504400	-0.01381000
	C	-7.64347500	1.07459400	0.79220700
	C	-6.62138500	1.85663700	1.31899900
	C	-5.29128100	1.55654000	1.03444400
	C	-2.52183800	1.26191600	0.13245100
	C	-2.79909900	2.42420700	-0.59757300
	C	-1.90922100	3.49357800	-0.59548800
	C	-0.72044900	3.41503100	0.12827900

	C	-0.42496000	2.25525500	0.83645500
	C	-1.31847400	1.18758000	0.83178600
	H	-3.17647800	0.28422000	2.70748700
	H	-2.87444300	-1.48125000	4.38936800
	H	-2.80136400	-3.86790000	3.69819500
	H	-3.05171700	-4.44679300	1.29526700
	H	-3.30815200	-2.68458200	-0.38153200
	H	-5.76364900	-1.16627900	-0.91894100
	H	-8.10822900	-0.63551000	-0.42807400
	H	-8.67945100	1.30836600	1.00860400
	H	-6.85424800	2.70417600	1.95350100
	H	-4.50835900	2.17608400	1.45144000
	H	-3.71602600	2.48653000	-1.17071600
	H	-2.14428600	4.38636600	-1.16355400
	H	-0.02435600	4.24573800	0.13465300
	H	0.50793400	2.17614200	1.37882700
	H	-1.06969800	0.28537700	1.36739300
	H	-0.99206700	-3.28765000	-2.16866900
	C	-0.03342500	-0.76441300	-2.88098500
	H	0.44709100	-1.15917500	-3.78309300
	H	0.51250300	-1.16266900	-2.02313800
	C	0.03354000	0.76304000	-2.88128100
	C	1.46003300	1.28775600	-2.83048800
	H	2.08500300	0.80018100	-3.58070300
	C	1.56663900	2.79814000	-2.95944000
	H	1.17408100	3.12040400	-3.92709800
	H	2.60954500	3.11547300	-2.89391300
	H	0.99221800	3.28663500	-2.17027600
	O	1.97680300	0.90165500	-1.52209300
	C	3.22289000	0.41643800	-1.43559200
	C	3.49101000	-0.06526400	0.01321300
	C	2.52199400	-1.26196400	0.13301000
	C	1.31845700	-1.18739200	0.83201900
	O	3.97916400	0.31072000	-2.36724900
	C	2.79953700	-2.42458800	-0.59637800
	C	1.90976500	-3.49404600	-0.59394800
	C	0.72082800	-3.41526200	0.12952600
	C	0.42505100	-2.25515500	0.83703900
	C	3.23287200	1.05977100	1.03020200
	C	3.21520000	2.40505300	0.65767600
	C	3.06485300	3.41067900	1.60988900
	C	2.92663400	3.08867900	2.95591200
	C	2.96383800	1.75050500	3.34229700
	C	3.12752000	0.75065300	2.39018700
	C	4.95913900	-0.47308400	0.21827400
	C	5.99372200	0.31662000	-0.29234600
	C	7.32189400	0.01530600	-0.01326600
	C	7.64350000	-1.07384900	0.79341200
	C	6.62139400	-1.85564000	1.32054900
	C	5.29130800	-1.55577900	1.03567000
	H	1.06945000	-0.28492700	1.36706600
	H	3.71659800	-2.48710600	-1.16928200

	H	2.14504000	-4.38709500	-1.16151600
	H	0.02484100	-4.24605800	0.13617400
	H	-0.50797400	-2.17585300	1.37915900
	H	3.30840700	2.68436600	-0.38256300
	H	3.05153900	4.44734300	1.29338200
	H	2.80047000	3.86952000	3.69649400
	H	2.87327100	1.48318100	4.38874700
	H	3.17571500	-0.28305400	2.70773600
	H	5.76374600	1.16593400	-0.91924000
	H	8.10829500	0.63557600	-0.42779800
	H	8.67946500	-1.30744100	1.01005400
	H	6.85423100	-2.70279700	1.95557100
	H	4.50837200	-2.17512500	1.45293300
	H	-0.51234400	1.16163300	-2.02356400
	H	-0.44701300	1.15746300	-3.78351700
5	O	1.67542600	-1.53502800	0.90071800
	C	2.99282200	-1.29031600	0.81907200
	C	1.25838800	-2.77010800	1.57107700
	H	1.91785800	-2.90781200	2.43106300
	C	1.41543400	-3.93045900	0.60154100
	H	1.09534200	-4.85969100	1.07953200
	H	2.45821600	-4.04155500	0.30308300
	O	3.83343500	-1.98272600	1.33369800
	C	3.26781800	-0.03341000	-0.05555800
	C	4.78468900	0.11622600	-0.29625200
	C	5.31681800	0.32723800	-1.56850000
	C	6.68219500	0.55464800	-1.74335700
	C	7.53534400	0.57578000	-0.64740400
	C	7.01072400	0.37917700	0.62942000
	C	5.65081300	0.15998900	0.80290000
	C	2.82699200	1.25279700	0.66937000
	C	2.23769700	1.25522800	1.93178600
	C	1.92311000	2.45142100	2.57679700
	C	2.19817500	3.66980100	1.96836500
	C	2.79469100	3.68112800	0.70745700
	C	3.10877900	2.48791000	0.07079500
	C	2.50461600	-0.27816800	-1.36992800
	C	1.51660400	0.57405200	-1.85473700
	C	0.88356100	0.30789000	-3.06968800
	C	1.21519400	-0.82386400	-3.80483200
	C	2.18260900	-1.70005000	-3.31175700
	C	2.81767400	-1.42817700	-2.10714200
	H	4.66910500	0.32078400	-2.43401600
	H	7.07189100	0.71649100	-2.74192200
	H	8.59675700	0.74930900	-0.78204800
	H	7.66336400	0.40062300	1.49463200
	H	5.25733400	0.02051900	1.79972700
	H	2.02823200	0.32278000	2.43325000
	H	1.47239100	2.42265500	3.56235600
	H	1.95974800	4.60031000	2.47017300
	H	3.02521600	4.62287500	0.22290300
	H	3.59769700	2.51097600	-0.89476300

	H	1.21807200	1.44529100	-1.29071200
	H	0.11964400	0.98892200	-3.42347400
	H	0.72019500	-1.02980000	-4.74672600
	H	2.44507500	-2.59271400	-3.86812000
	H	3.57844300	-2.10684000	-1.73790900
	H	0.80077600	-3.76453300	-0.28407000
	C	-0.18026400	-2.53428100	2.00744000
	H	-0.59844200	-3.48608800	2.34984200
	H	-0.75330100	-2.22810900	1.13130800
	C	-0.30103200	-1.47570200	3.10651100
	C	-1.69509300	-0.87977600	3.23873200
	H	-2.45088900	-1.65330000	3.39986100
	C	-1.79343300	0.18179300	4.32316300
	H	-2.79838100	0.60071800	4.35898300
	H	-1.07566700	0.98463000	4.13324100
	H	-1.56611700	-0.25878200	5.29732900
	O	-1.95506000	-0.29328200	1.93136900
	C	-3.22649900	-0.08348400	1.55846300
	C	-3.30845800	0.17971700	0.03274800
	C	-2.85417000	-1.17815000	-0.54960700
	C	-1.69024000	-1.31403500	-1.30136700
	O	-4.16913000	-0.20325000	2.29822600
	C	-3.58991400	-2.32845300	-0.23437300
	C	-3.18092700	-3.57823800	-0.68458500
	C	-2.02639300	-3.70108900	-1.45717900
	C	-1.28330000	-2.56571300	-1.76096500
	C	-2.41812600	1.34091000	-0.45928000
	C	-1.49399000	2.02459400	0.33191000
	C	-0.73525300	3.07017500	-0.19478800
	C	-0.87961300	3.44874600	-1.52320300
	C	-1.80054300	2.77476000	-2.32446000
	C	-2.56125600	1.74027700	-1.79559200
	C	-4.75689700	0.53031700	-0.34413000
	C	-5.39526900	1.58220300	0.32103500
	C	-6.67215300	1.98597900	-0.04451400
	C	-7.33642000	1.35147000	-1.09379100
	C	-6.70603000	0.31480500	-1.77058300
	C	-5.42484400	-0.09240900	-1.39768900
	H	-1.08243300	-0.45093800	-1.52131500
	H	-4.48612200	-2.23905500	0.36808700
	H	-3.76458700	-4.45628200	-0.43265900
	H	-1.70741500	-4.67504600	-1.81023300
	H	-0.37538800	-2.63805000	-2.34642500
	H	-1.33284200	1.73609600	1.35778100
	H	-0.01845300	3.57166200	0.44195100
	H	-0.28401200	4.25708300	-1.93136300
	H	-1.93410600	3.06039800	-3.36186400
	H	-3.28249800	1.23691100	-2.42624300
	H	-4.88746800	2.08964600	1.13099700
	H	-7.15070700	2.79883800	0.48952800
	H	-8.33329400	1.66647300	-1.37968200
	H	-7.20625800	-0.18367400	-2.59309200

	H	-4.94789100	-0.89900400	-1.93813900
	H	0.37546800	-0.64936000	2.88205200
	H	0.00028300	-1.88796000	4.07507400
7	O	-2.12198800	0.74389200	1.74631800
	C	-3.33219400	0.19965800	1.54627200
	C	-1.64628000	0.90303200	3.11487500
	H	-2.26176300	0.26328400	3.74946200
	C	-1.82264800	2.35965700	3.51136000
	H	-1.45405200	2.52269000	4.52708100
	H	-2.87758600	2.63980800	3.48327700
	O	-4.08939600	-0.11207500	2.42969000
	C	-3.54822800	-0.06073900	0.03548600
	C	-3.32450300	1.20717200	-0.80781300
	C	-3.19669200	1.09324200	-2.19593100
	C	-3.12131800	2.22151400	-3.00371300
	C	-3.18686400	3.49455800	-2.44002200
	C	-3.32298500	3.62047400	-1.06156300
	C	-3.38927400	2.48656100	-0.25446500
	C	-4.99554300	-0.48806500	-0.26464700
	C	-6.07014300	0.16694600	0.34379700
	C	-7.38017900	-0.13477100	-0.01082000
	C	-7.64346800	-1.08849700	-0.99110800
	C	-6.58122600	-1.73349600	-1.61552300
	C	-5.26969600	-1.43331200	-1.25581200
	C	-2.52494600	-1.18284600	-0.23766100
	C	-2.78738000	-2.47369800	0.23683000
	C	-1.85395000	-3.49274200	0.07467300
	C	-0.63876100	-3.23428100	-0.55745400
	C	-0.35681500	-1.94524300	-0.99762800
	C	-1.28744500	-0.92482700	-0.82609900
	H	-3.16535500	0.11146800	-2.65010000
	H	-3.02172000	2.10576300	-4.07682900
	H	-3.13597000	4.37566600	-3.06893100
	H	-3.37973300	4.60309100	-0.60735100
	H	-3.49628500	2.61244900	0.81425600
	H	-5.88627900	0.91117000	1.10509800
	H	-8.19802400	0.37927800	0.48100300
	H	-8.66519300	-1.32279900	-1.26658900
	H	-6.76850800	-2.47304400	-2.38567300
	H	-4.45503800	-1.94492600	-1.75077100
	H	-3.72769900	-2.67889600	0.73318100
	H	-2.07803000	-4.48763800	0.44202700
	H	0.08543400	-4.02897700	-0.69703800
	H	0.59463200	-1.72065400	-1.45969000
	H	-1.03256000	0.07628400	-1.13772000
	H	-1.26703500	3.00984400	2.83120800
	C	-0.19786400	0.43872200	3.10878100
	H	0.24425100	0.67471700	4.08330000
	H	0.34202400	1.01579900	2.35612800
	C	-0.04251600	-1.05337900	2.81432400
	C	1.41154900	-1.47692600	2.67328900
	H	2.01888000	-1.07726500	3.48752800

	C	1.60298400	-2.98216400	2.57842800
	H	1.00714300	-3.39158000	1.76009000
	H	1.27940300	-3.45495300	3.50903100
	H	2.65449800	-3.22718100	2.42129400
	O	1.88960800	-0.85363400	1.43808000
	C	3.19016400	-0.52593100	1.36513400
	C	3.54823100	0.06375500	-0.03378800
	C	2.35831000	0.88558100	-0.55719800
	C	1.77463400	1.84473600	0.27897400
	O	3.97046400	-0.70494700	2.26444800
	C	1.86390100	0.75011800	-1.85236100
	C	0.80396000	1.54290900	-2.29665600
	C	0.21899800	2.47723100	-1.45113300
	C	0.71355000	2.62641800	-0.15629900
	C	3.84816800	-1.12344300	-0.97947000
	C	4.74939400	-0.95695500	-2.03618000
	C	4.98324400	-1.98426500	-2.94526600
	C	4.32822000	-3.20559200	-2.81132800
	C	3.43248600	-3.38354900	-1.76221200
	C	3.19040900	-2.35061600	-0.85952100
	C	4.81193900	0.93873200	0.09877900
	C	5.99137900	0.37871100	0.60845900
	C	7.15740000	1.12811400	0.69119400
	C	7.17778800	2.45246200	0.25538700
	C	6.01954700	3.01254300	-0.26767300
	C	4.84673200	2.26049700	-0.34656500
	H	2.16356100	1.98186300	1.28158900
	H	2.29975900	0.02213900	-2.52293100
	H	0.42504500	1.41352500	-3.30395100
	H	-0.62150400	3.06949300	-1.78664800
	H	0.26436100	3.34979300	0.51402600
	H	5.27072600	-0.01605200	-2.14938500
	H	5.68339000	-1.82815600	-3.75789600
	H	4.51567000	-4.00817400	-3.51498000
	H	2.91496900	-4.32856400	-1.64176600
	H	2.47087000	-2.50202000	-0.07014600
	H	5.99306300	-0.64829700	0.94179700
	H	8.05551500	0.67454400	1.09465400
	H	8.08935800	3.03557300	0.31854800
	H	6.01950900	4.03720200	-0.62151900
	H	3.96059000	2.71288500	-0.76802800
	H	-0.56889400	-1.31152900	1.89329600
	H	-0.49333400	-1.64156100	3.62097800
17	O	2.07352600	-1.98408200	-0.12499300
	C	2.25026900	-0.99046000	0.75960800
	C	1.76371800	-3.31559000	0.39173500
	H	2.15172600	-3.36202500	1.40978300
	C	2.51045000	-4.28468700	-0.50946800
	H	3.58657800	-4.12174500	-0.42987700
	H	2.22018100	-4.15386400	-1.55339600
	O	2.04883200	-1.11566000	1.94384400
	C	2.87764200	0.25275600	0.08007800

	C	2.52181100	1.59328100	0.75778900	
	C	3.03205100	2.75934400	0.16953700	
	C	2.78631700	4.01083100	0.71750200	
	C	2.00355900	4.13221100	1.86540900	
	C	1.47599900	2.98598300	2.44597800	
	C	1.73713800	1.72708500	1.90282600	
	C	2.42824900	0.36649100	-1.38697200	
	C	1.06497500	0.28779500	-1.68213900	
	C	0.59827200	0.44879200	-2.97930800	
	C	1.49590200	0.71944600	-4.01081500	
	C	2.85263900	0.82871800	-3.72742800	
	C	3.31620800	0.65131200	-2.42408100	
	C	4.38495300	-0.06826700	0.24168700	
	C	4.96589000	-1.09749700	-0.51086100	
	C	6.30066800	-1.44245400	-0.33174200	
	C	7.07749100	-0.77496000	0.61352100	
	C	6.50283800	0.23270700	1.38013800	
	C	5.16529700	0.58004700	1.19973100	
	H	3.62810300	2.68514400	-0.73032500	
	H	3.20042000	4.89319100	0.24313500	
	H	1.80467200	5.10785700	2.29374700	
	H	0.85609600	3.05761900	3.33245100	
	H	1.34130500	0.85506300	2.39224500	
	H	0.35154700	0.11309800	-0.88837100	
	H	-0.46174300	0.35962200	-3.16962900	
	H	1.13670500	0.84865200	-5.02531500	
	H	3.55968500	1.04970600	-4.51919000	
	H	4.37550700	0.73634500	-2.22192600	
	H	4.36647200	-1.62449700	-1.24036700	
	H	6.73419500	-2.23427400	-0.93200000	
	H	8.11840400	-1.04280400	0.75296900	
	H	7.09246400	0.75266900	2.12634100	
	H	4.72929600	1.35919700	1.80896000	
	H	2.29354500	-5.31404600	-0.21288200	
	C	0.24945100	-3.51560200	0.43653900	
	H	-0.17351500	-2.79040900	1.13346600	
	H	0.06880000	-4.50349500	0.87296600	
	C	-0.44032500	-3.39604300	-0.92593000	
	C	-1.96779700	-3.35141800	-0.85899900	
	H	-2.35616900	-3.28151500	-1.87567200	
	C	-2.61190700	-4.50775900	-0.11507200	
	H	-3.69986400	-4.44545000	-0.17851800	
	H	-2.33176600	-4.50216500	0.93947800	
	H	-2.29578900	-5.45667200	-0.55575000	
	O	-2.39605800	-2.14254600	-0.15338700	
	C	-2.45053600	-1.00744100	-0.86882400	
	C	-2.93992300	0.21054000	-0.02554300	
	C	-4.47563700	0.26786700	-0.19295300	
	C	-5.14092300	1.49415800	-0.26557400	
	O	-2.18905100	-0.95981100	-2.04469500	
	C	-5.23955500	-0.90288300	-0.17657900	
	C	-6.62790900	-0.85085100	-0.25545700	

	C	-7.28098000	0.37568500	-0.33716900
	C	-6.53074100	1.54771400	-0.33553300
	C	-2.26906500	1.46864900	-0.61746800
	C	-2.55133800	1.84339700	-1.93918900
	C	-1.92750800	2.94041900	-2.51777800
	C	-1.00421300	3.68944000	-1.78990900
	C	-0.73171500	3.34088700	-0.47471600
	C	-1.36657500	2.24448100	0.10887100
	C	-2.58450400	0.02951000	1.45905200
	C	-1.30130000	-0.38870800	1.82482400
	C	-0.93563800	-0.52757500	3.15704700
	C	-1.85381000	-0.22946900	4.16310200
	C	-3.12792200	0.20425700	3.81608100
	C	-3.49131700	0.32934200	2.47506100
	H	-4.57049100	2.41288600	-0.26393500
	H	-4.74890200	-1.86151000	-0.07736100
	H	-7.19892500	-1.77226800	-0.24709300
	H	-8.36226000	0.41754800	-0.39752000
	H	-7.02519000	2.51085500	-0.38952500
	H	-3.25613500	1.26419600	-2.51786900
	H	-2.15480600	3.20355600	-3.54460200
	H	-0.50233300	4.53412300	-2.24741200
	H	-0.01559000	3.90547200	0.10710200
	H	-1.13500400	1.99874900	1.13408000
	H	-0.57207600	-0.60791400	1.05767200
	H	0.06443500	-0.86690800	3.39647300
	H	-1.57506000	-0.33541000	5.20514300
	H	-3.85116000	0.44290900	4.58756000
	H	-4.49060000	0.65876500	2.22649000
	H	-0.17161400	-4.24355500	-1.56512900
	H	-0.09301900	-2.49880300	-1.44220500
26	O	-2.13032300	-1.84993400	-1.09310800
	C	-2.35069200	-0.55702100	-1.37174400
	C	-1.79856400	-2.73532500	-2.19721700
	H	-1.23347800	-2.14843000	-2.92353200
	C	-3.09284000	-3.23400700	-2.82333200
	H	-3.68681300	-2.39315800	-3.18299100
	H	-3.68092400	-3.79431100	-2.09192100
	O	-2.30097500	-0.10776300	-2.49086200
	C	-2.75029100	0.25859400	-0.11227700
	C	-4.28760100	0.48430500	-0.19383400
	C	-5.08972500	0.44606400	0.95045600
	C	-6.44958900	0.74041400	0.88265500
	C	-7.03573000	1.08041700	-0.33166900
	C	-6.24265900	1.13706100	-1.47455600
	C	-4.88302500	0.85074800	-1.40608900
	C	-2.09538600	1.66267000	-0.11463900
	C	-2.49358800	2.56419700	0.88123800
	C	-1.89527700	3.81195800	1.00429300
	C	-0.88711500	4.19520400	0.12023600
	C	-0.49686000	3.31675400	-0.88343500
	C	-1.09629000	2.06248500	-1.00226300

	C	-2.34233400	-0.52776600	1.14898400
	C	-1.24114000	-0.14648500	1.91146200
	C	-0.83110300	-0.89266200	3.01634700
	C	-1.52447000	-2.04045800	3.37852100
	C	-2.62461600	-2.44091100	2.61957000
	C	-3.02506500	-1.69494300	1.51888200
	H	-4.65506100	0.18728400	1.90573800
	H	-7.04775400	0.70304500	1.78609900
	H	-8.09447400	1.30593300	-0.38603700
	H	-6.68005200	1.41351700	-2.42719500
	H	-4.27473100	0.92270400	-2.29649500
	H	-3.27224100	2.28232700	1.57817800
	H	-2.22054600	4.48634100	1.78851000
	H	-0.41556000	5.16700000	0.21302000
	H	0.29089700	3.58940300	-1.57548500
	H	-0.75976700	1.40010900	-1.77902800
	H	-0.68477000	0.73610700	1.64395700
	H	0.03471100	-0.56700200	3.57844300
	H	-1.21271800	-2.62249400	4.23841400
	H	-3.17194200	-3.33835000	2.88553300
	H	-3.87064000	-2.02781900	0.93254500
	H	-2.87669000	-3.88863100	-3.67133300
	C	-0.93139600	-3.85716400	-1.63130500
	H	-0.84529600	-4.61512400	-2.41584700
	H	-1.46320800	-4.33323500	-0.80027400
	C	0.48163000	-3.42633000	-1.20452800
	C	0.55425000	-2.82770300	0.20734400
	H	-0.33287600	-2.23489700	0.40724800
	C	0.76361800	-3.86597800	1.29001800
	H	0.79468900	-3.39238400	2.27080800
	H	1.69619300	-4.41152300	1.12428700
	H	-0.06555900	-4.57738400	1.28278700
	O	1.70420800	-1.92074000	0.30078500
	C	1.54582400	-0.72734200	-0.27835600
	C	2.75094800	0.21146200	-0.03438900
	C	2.51173200	0.65407400	1.42616000
	C	1.65979200	1.72703200	1.70565700
	O	0.54006000	-0.40336200	-0.86147500
	C	3.03498100	-0.07963400	2.49466300
	C	2.76539500	0.28997000	3.80917200
	C	1.94748500	1.38534500	4.07811800
	C	1.38549100	2.09407800	3.02007800
	C	2.75079300	1.39337600	-1.02370300
	C	2.29941900	1.25090800	-2.33840400
	C	2.38749300	2.30564000	-3.24200500
	C	2.94142800	3.52247600	-2.85482400
	C	3.42114000	3.66655800	-1.55633500
	C	3.33102800	2.61035900	-0.65530400
	C	4.09682600	-0.49901600	-0.25375800
	C	4.21950000	-1.54373600	-1.17209200
	C	5.46103300	-2.10488800	-1.45556200
	C	6.60726900	-1.62617500	-0.82827100

	C 6.49896800 -0.57450800 0.07775500<br>C 5.25660400 -0.01455200 0.35678400<br>H 1.19712600 2.27681200 0.89776300<br>H 3.65544100 -0.94291400 2.29688600<br>H 3.19169300 -0.28428500 4.62376700<br>H 1.73860800 1.67334800 5.10193500<br>H 0.72004200 2.92839700 3.20765700<br>H 1.85677900 0.32150400 -2.66279700<br>H 2.01586400 2.17317600 -4.25150400<br>H 3.00365300 4.34515600 -3.55757800<br>H 3.86708800 4.60246200 -1.23988800<br>H 3.70836900 2.74301100 0.34975300<br>H 3.34285700 -1.93041200 -1.67475300<br>H 5.52991800 -2.91845200 -2.16858200<br>H 7.57433700 -2.06389800 -1.04630800<br>H 7.38382700 -0.18418600 0.56730800<br>H 5.19005200 0.80768200 1.05728600<br>H 0.84538000 -2.69150400 -1.92495700<br>H 1.16010100 -4.28379900 -1.25517000

22 (optimized at the M06-2X/6-311G(d,p) level)

Conformer no	
1	O -0.83205600 1.99166700 -1.52716800<br>C -0.42839400 3.25875200 -1.41545400<br>C -1.22702700 1.50140100 -2.82693400<br>H -0.70581900 2.09928900 -3.57926100<br>C -2.72836500 1.66857000 -2.97205000<br>H -3.06010300 1.28569500 -3.93921100<br>H -2.99950800 2.72474900 -2.91141300<br>O -0.42137800 4.04776000 -2.31855800<br>C 0.07637200 3.50463300 0.02395700<br>C -1.06064100 3.25761600 1.02624400<br>C -0.76130000 3.17538300 2.38826600<br>C -1.76606500 3.00700300 3.33110300<br>C -3.09811400 2.94486500 2.93162000<br>C -3.41041300 3.06233500 1.58384000<br>C -2.39897900 3.21396100 0.63840800<br>C 0.51841000 4.95893800 0.23402100<br>C -0.28637400 6.00632900 -0.22062700<br>C 0.04642400 7.32578400 0.05178600<br>C 1.18230900 7.62551200 0.79715900<br>C 1.97677600 6.59167200 1.27219900<br>C 1.64626700 5.26841300 0.99415200<br>C 1.24212300 2.50238300 0.13859500<br>C 2.39858000 2.74123500 -0.61070400<br>C 3.43842200 1.81982300 -0.62429600<br>C 3.33507400 0.63674500 0.10354700<br>C 2.17998000 0.37924600 0.83070900<br>C 1.14148400 1.30602600 0.84294400<br>H 0.27252200 3.24238300 2.71018900<br>H -1.50917700 2.93378000 4.38114400

	H	-3.88343800	2.81709000	3.66679400
	H	-4.44422400	3.03356700	1.25944600
	H	-2.67023900	3.29086500	-0.40819200
	H	-1.17624100	5.78897300	-0.79658700
	H	-0.58673000	8.12326600	-0.31839900
	H	1.44050800	8.65629100	1.00922900
	H	2.85943500	6.80964600	1.86191300
	H	2.27852500	4.47502200	1.37402300
	H	2.47956000	3.65788700	-1.18619300
	H	4.32935900	2.02724800	-1.20559000
	H	4.14578100	-0.08330000	0.10128000
	H	2.08404700	-0.54862800	1.38503000
	H	0.23980000	1.08680500	1.39715100
	H	-3.23944600	1.11533600	-2.18055100
	C	-0.76130000	0.05697200	-2.86747800
	H	-1.17352300	-0.41765300	-3.76486400
	H	-1.17610600	-0.46236400	-1.99616500
	C	0.76130000	-0.05697200	-2.86747800
	C	1.22702700	-1.50140100	-2.82693400
	H	0.70581900	-2.09928900	-3.57926100
	C	2.72836500	-1.66857000	-2.97205000
	H	3.06010300	-1.28569500	-3.93921100
	H	2.99950800	-2.72474900	-2.91141300
	H	3.23944600	-1.11533600	-2.18055100
	O	0.83205600	-1.99166700	-1.52716800
	C	0.42839400	-3.25875200	-1.41545400
	C	-0.07637200	-3.50463300	0.02395700
	C	1.06064100	-3.25761600	1.02624400
	C	0.76130000	-3.17538300	2.38826600
	O	0.42137800	-4.04776000	-2.31855800
	C	2.39897900	-3.21396100	0.63840800
	C	3.41041300	-3.06233500	1.58384000
	C	3.09811400	-2.94486500	2.93162000
	C	1.76606500	-3.00700300	3.33110300
	C	-0.51841000	-4.95893800	0.23402100
	C	0.28637400	-6.00632900	-0.22062700
	C	-0.04642400	-7.32578400	0.05178600
	C	-1.18230900	-7.62551200	0.79715900
	C	-1.97677600	-6.59167200	1.27219900
	C	-1.64626700	-5.26841300	0.99415200
	C	-1.24212300	-2.50238300	0.13859500
	C	-2.39858000	-2.74123500	-0.61070400
	C	-3.43842200	-1.81982300	-0.62429600
	C	-3.33507400	-0.63674500	0.10354700
	C	-2.17998000	-0.37924600	0.83070900
	C	-1.14148400	-1.30602600	0.84294400
	H	-0.27252200	-3.24238300	2.71018900
	H	2.67023900	-3.29086500	-0.40819200
	H	4.44422400	-3.03356700	1.25944600
	H	3.88343800	-2.81709000	3.66679400
	H	1.50917700	-2.93378000	4.38114400
	H	1.17624100	-5.78897300	-0.79658700

	H	0.58673000	-8.12326600	-0.31839900
	H	-1.44050800	-8.65629100	1.00922900
	H	-2.85943500	-6.80964600	1.86191300
	H	-2.27852500	-4.47502200	1.37402300
	H	-2.47956000	-3.65788700	-1.18619300
	H	-4.32935900	-2.02724800	-1.20559000
	H	-4.14578100	0.08330000	0.10128000
	H	-2.08404700	0.54862800	1.38503000
	H	-0.23980000	-1.08680500	1.39715100
	H	1.17610600	0.46236400	-1.99616500
	H	1.17352300	0.41765300	-3.76486400
3	O	-1.98946600	-0.83843000	-1.52632500
	C	-3.25745400	-0.43743700	-1.41507900
	C	-1.49776400	-1.23246400	-2.82585900
	H	-2.09728400	-0.71357100	-3.57849100
	C	-1.66002800	-2.73439900	-2.97022800
	H	-1.27560100	-3.06536800	-3.93703600
	H	-2.71534300	-3.00897400	-2.90993400
	O	-4.04616100	-0.43206200	-2.31845500
	C	-3.50491300	0.06692800	0.02422800
	C	-3.25522200	-1.06920600	1.02682100
	C	-3.17400600	-0.76930500	2.38880400
	C	-3.00296900	-1.77336000	3.33190800
	C	-2.93704500	-3.10534300	2.93277200
	C	-3.05344000	-3.41828200	1.58505800
	C	-3.20774400	-2.40749200	0.63934100
	C	-4.96030300	0.50553100	0.23387500
	C	-6.00575600	-0.30155900	-0.22116700
	C	-7.32604000	0.02832700	0.05078500
	C	-7.62854700	1.16355900	0.79607600
	C	-6.59663800	1.96029200	1.27148300
	C	-5.27254400	1.63269900	0.99389600
	C	-2.50555400	1.23516600	0.13885100
	C	-2.74711900	2.39090200	-0.61074000
	C	-1.82829300	3.43302000	-0.62432600
	C	-0.64514700	3.33278000	0.10386600
	C	-0.38495000	2.17848000	0.83133400
	C	-1.30913800	1.13766600	0.84353200
	H	-3.24392000	0.26439000	2.71049100
	H	-2.93061800	-1.51599900	4.38189600
	H	-2.80722300	-3.89012300	3.66816900
	H	-3.02177400	-4.45207400	1.26088300
	H	-3.28364300	-2.67925600	-0.40720600
	H	-5.78624700	-1.19090900	-0.79711800
	H	-8.12198500	-0.60656500	-0.31973000
	H	-8.65997600	1.41949900	1.00773600
	H	-6.81674500	2.84247700	1.86112300
	H	-4.48070400	2.26676800	1.37398700
	H	-3.66377600	2.46944700	-1.18654900
	H	-2.03776600	4.32328700	-1.20591800
	H	0.07288700	4.14526400	0.10160500
	H	0.54301200	2.08502300	1.38595100

	H	-1.08779000	0.23662800	1.39794900
	H	-1.10551400	-3.24329300	-2.17821900
	C	-0.05481500	-0.76202000	-2.86643800
	H	0.42133400	-1.17290700	-3.76362700
	H	0.46574400	-1.17487500	-1.99493700
	C	0.05412400	0.76091200	-2.86673600
	C	1.49707800	1.23138400	-2.82667700
	H	2.09643000	0.71220600	-3.57924500
	C	1.65927800	2.73326400	-2.97168400
	H	1.27462900	3.06383700	-3.93853900
	H	2.71460100	3.00788500	-2.91174100
	H	1.10493000	3.24246900	-2.17975800
	O	1.98907800	0.83787100	-1.52709700
	C	3.25715300	0.43712300	-1.41593600
	C	3.50491400	-0.06685600	0.02346000
	C	2.50571600	-1.23518900	0.13854200
	C	1.30930000	-1.13759000	0.84320700
	O	4.04570900	0.43161300	-2.31944300
	C	2.74741500	-2.39116300	-0.61063900
	C	1.82873600	-3.43341600	-0.62381700
	C	0.64559300	-3.33306900	0.10436600
	C	0.38525300	-2.17853400	0.83140700
	C	3.25525700	1.06947500	1.02584600
	C	3.20751300	2.40767500	0.63810200
	C	3.05327200	3.41863600	1.58364800
	C	2.93722600	3.10595800	2.93145100
	C	3.00344500	1.77406900	3.33084800
	C	3.17441500	0.76984800	2.38791200
	C	4.96040100	-0.50522200	0.23296900
	C	6.00565900	0.30190900	-0.22244500
	C	7.32604000	-0.02771400	0.04936400
	C	7.62884100	-1.16271000	0.79489300
	C	6.59712800	-1.95945800	1.27070100
	C	5.27293900	-1.63212900	0.99326100
	H	1.08783600	-0.23637600	1.39729100
	H	3.66407400	-2.46979300	-1.18643600
	H	2.03831600	-4.32387000	-1.20508400
	H	-0.07233800	-4.14564500	0.10242200
	H	-0.54270800	-2.08500700	1.38601400
	H	3.28313000	2.67923600	-0.40851700
	H	3.02138300	4.45235700	1.25926700
	H	2.80745900	3.89087200	3.66671500
	H	2.93138000	1.51691200	4.38090700
	H	3.24457200	-0.26376900	2.70979700
	H	5.78592400	1.19108000	-0.79858500
	H	8.12182500	0.60720100	-0.32145300
	H	8.66034200	-1.41844100	1.00645100
	H	6.81746600	-2.84144500	1.86055100
	H	4.48125800	-2.26619400	1.37368500
	H	-0.46621700	1.17409200	-1.99525900
	H	-0.42224800	1.17146200	-3.76396000
11	O	1.92270000	-0.98888200	1.41268200

	C	3.21900300	-0.69834800	1.29757800
	C	1.47666700	-1.70593000	2.58697900
	H	2.08161500	-1.36595900	3.43222200
	C	1.68000300	-3.19217000	2.35947700
	H	1.12308800	-3.50592800	1.47397300
	H	1.32006800	-3.75667800	3.22208400
	O	4.04185300	-1.02381000	2.10733000
	C	3.50626000	0.04768900	-0.03562500
	C	2.65338900	1.33748400	-0.11858400
	C	1.25513000	1.24732600	-0.11144200
	C	0.45780700	2.37290600	-0.25452900
	C	1.04187800	3.62681700	-0.41734100
	C	2.42463100	3.73070100	-0.43960200
	C	3.22514500	2.59693400	-0.29687000
	C	3.11010400	-0.91926000	-1.16598500
	C	3.34172500	-2.28840300	-1.02005400
	C	3.05913500	-3.17351500	-2.05343900
	C	2.55231500	-2.70030000	-3.26011200
	C	2.33312900	-1.33716300	-3.42026200
	C	2.60497200	-0.45293800	-2.37956700
	C	5.00492100	0.36650300	-0.11238600
	C	5.63894700	0.99463100	0.96623700
	C	6.97531000	1.35684200	0.89390600
	C	7.71120000	1.10099300	-0.26036000
	C	7.09362500	0.48132600	-1.33564800
	C	5.75042800	0.11623700	-1.26156400
	H	0.78499100	0.28102700	0.00163200
	H	-0.62124400	2.26368700	-0.24283700
	H	0.41928400	4.50644000	-0.53476500
	H	2.89682600	4.69656900	-0.57707900
	H	4.30032400	2.70876900	-0.33967100
	H	3.76325200	-2.66746400	-0.09404600
	H	3.24232500	-4.23288700	-1.91710100
	H	2.33209100	-3.38822800	-4.06747000
	H	1.94375500	-0.95546400	-4.35680100
	H	2.42629300	0.60694000	-2.51689300
	H	5.07994100	1.20450300	1.86909300
	H	7.44527100	1.83918100	1.74282800
	H	8.75609500	1.38255600	-0.31564800
	H	7.65207600	0.27380400	-2.24071400
	H	5.29099700	-0.36757000	-2.11401100
	H	2.73968000	-3.41235700	2.22125100
	C	0.02084400	-1.30608100	2.76329900
	H	-0.42399400	-1.93698100	3.54013000
	H	-0.50788700	-1.51797700	1.82655400
	C	-0.13007100	0.16847300	3.13383600
	C	-1.57567100	0.63400200	3.12094900
	H	-2.20099700	-0.02401600	3.72954300
	C	-1.75315500	2.07812000	3.55269800
	H	-2.80801200	2.35733600	3.51590500
	H	-1.18116600	2.73525900	2.89250700
	H	-1.39795200	2.21379000	4.57609800

	O	-2.01564700	0.50905700	1.74942600
	C	-3.29196000	0.17784400	1.52482800
	C	-3.50867500	-0.03235000	0.01027200
	C	-3.17866200	1.26406200	-0.74061700
	C	-2.95608100	1.23565600	-2.11843300
	O	-4.10722000	0.02172500	2.39040800
	C	-3.20831400	2.50215700	-0.09918300
	C	-2.97600300	3.67807000	-0.80639500
	C	-2.70940500	3.63314400	-2.16874400
	C	-2.70901100	2.40586000	-2.82392700
	C	-4.97103500	-0.35302500	-0.32902300
	C	-6.01158900	0.36202400	0.26795100
	C	-7.32849500	0.14886200	-0.11638100
	C	-7.63118200	-0.76960400	-1.11571900
	C	-6.60195000	-1.46952500	-1.73060800
	C	-5.28286100	-1.26159900	-1.34059800
	C	-2.56159900	-1.21285700	-0.28254300
	C	-2.90815100	-2.46788900	0.22935800
	C	-2.04843600	-3.55130600	0.10186600
	C	-0.81822500	-3.39571000	-0.53106400
	C	-0.45154400	-2.14721400	-1.01731800
	C	-1.31885400	-1.06388500	-0.89416400
	H	-2.97343100	0.28682500	-2.64336000
	H	-3.40403900	2.56234000	0.96613400
	H	-2.99934000	4.62810700	-0.28551500
	H	-2.51534000	4.54618400	-2.71843900
	H	-2.52251400	2.35924200	-3.89010400
	H	-5.79574700	1.08420800	1.04376200
	H	-8.12199700	0.70569400	0.36790100
	H	-8.65961300	-0.93390300	-1.41426300
	H	-6.81987700	-2.18194200	-2.51749800
	H	-4.49159600	-1.81761700	-1.82930000
	H	-3.86215700	-2.59143700	0.73176500
	H	-2.34108700	-4.51629500	0.49873400
	H	-0.14442200	-4.23822200	-0.63879400
	H	0.51554700	-2.00797200	-1.48665400
	H	-1.00626000	-0.09610800	-1.26529600
	H	0.43851800	0.78966800	2.43400400
	H	0.27933400	0.34461100	4.13450200
17	O	2.10903800	-1.92269200	-0.20782200
	C	2.22204600	-0.94530700	0.69556900
	C	1.81669400	-3.25571300	0.27510400
	H	2.24474500	-3.33902000	1.27632600
	C	2.52699200	-4.19014000	-0.68640000
	H	3.60371800	-4.01999400	-0.64405600
	H	2.19394600	-4.01316100	-1.71046300
	O	1.91897300	-1.07090000	1.85209100
	C	2.92604100	0.28580400	0.08682700
	C	2.82639400	1.51114900	1.00395600
	C	3.86384100	2.44185300	1.07042100
	C	3.71710800	3.62142100	1.79246200
	C	2.53051400	3.89031900	2.46397300

	C	1.48991900	2.97004500	2.40119800
	C	1.63664300	1.79672700	1.67189700
	C	2.31562600	0.71050200	-1.25557900
	C	1.02847100	0.34398900	-1.64220900
	C	0.48476200	0.79504900	-2.84117400
	C	1.21784300	1.64235600	-3.66092000
	C	2.49187200	2.04294800	-3.27021600
	C	3.03270100	1.58248500	-2.07790800
	C	4.36703400	-0.25359000	-0.03471000
	C	4.86337500	-0.76906200	-1.23161000
	C	6.13143700	-1.33865900	-1.28219300
	C	6.91306100	-1.41853600	-0.13582700
	C	6.41264900	-0.93545500	1.06853000
	C	5.14766200	-0.36330800	1.11916300
	H	4.79800400	2.24736500	0.55654500
	H	4.53605900	4.33005500	1.82957800
	H	2.41868400	4.80701000	3.03062600
	H	0.55645900	3.16043000	2.92007600
	H	0.81430000	1.09759900	1.62791100
	H	0.42857500	-0.30670900	-1.01643400
	H	-0.51542900	0.48101300	-3.11157900
	H	0.79560400	1.99807000	-4.59366200
	H	3.06913000	2.71465000	-3.89493200
	H	4.02738900	1.89919100	-1.78398800
	H	4.25425800	-0.73365000	-2.12609100
	H	6.50559500	-1.72521800	-2.22306400
	H	7.90159900	-1.86001200	-0.17901600
	H	7.00570500	-1.00406500	1.97278000
	H	4.75641500	0.00172600	2.06267300
	H	2.32119400	-5.22994300	-0.42246500
	C	0.30925600	-3.47261900	0.37019300
	H	-0.10378600	-2.74904500	1.07918800
	H	0.15554000	-4.46318600	0.81150100
	C	-0.41253200	-3.37283500	-0.97458700
	C	-1.93485300	-3.34594900	-0.85741400
	H	-2.36213100	-3.30194400	-1.86151800
	C	-2.51604800	-4.50726500	-0.07351200
	H	-3.60601700	-4.48955700	-0.11356700
	H	-2.21232600	-4.45344300	0.97308800
	H	-2.16757700	-5.45313100	-0.49409900
	O	-2.36306600	-2.15286400	-0.15317000
	C	-2.42259600	-1.01774100	-0.85800000
	C	-2.97565800	0.17505800	-0.02963100
	C	-4.50789300	0.17282100	-0.21296000
	C	-5.21803900	1.37473800	-0.23309800
	O	-2.12095400	-0.95332900	-2.01906700
	C	-5.22542000	-1.02352300	-0.25999800
	C	-6.61329900	-1.01853600	-0.34716000
	C	-7.31027300	0.18272200	-0.37292800
	C	-6.60625900	1.37931800	-0.30957600
	C	-2.35101800	1.45536100	-0.61949000
	C	-2.67564500	1.83086500	-1.92959300

	C	-2.10331100	2.95172300	-2.51054500
	C	-1.17908300	3.71735000	-1.80277900
	C	-0.86044300	3.36523300	-0.50142800
	C	-1.45663500	2.25181400	0.09074800
	C	-2.62381900	0.00785500	1.45411500
	C	-1.32658000	-0.36649100	1.81400400
	C	-0.93755300	-0.45714600	3.14192100
	C	-1.85782200	-0.17250000	4.14881000
	C	-3.15002000	0.19931400	3.80781500
	C	-3.53238500	0.28980300	2.47003500
	H	-4.68238100	2.31471600	-0.18232600
	H	-4.70273500	-1.97038000	-0.20606400
	H	-7.14917800	-1.95942800	-0.38919700
	H	-8.39160300	0.18674800	-0.43895300
	H	-7.13672900	2.32407200	-0.32098600
	H	-3.37773200	1.23175300	-2.49595300
	H	-2.36810900	3.22044400	-3.52690500
	H	-0.71288500	4.57831200	-2.26675600
	H	-0.13996900	3.94295500	0.06610700
	H	-1.21305900	2.01791300	1.11837600
	H	-0.59790000	-0.57892600	1.03742500
	H	0.08311200	-0.73929500	3.37394500
	H	-1.56472900	-0.24148700	5.18962500
	H	-3.87438500	0.42025600	4.58303700
	H	-4.54756800	0.57818100	2.22769600
	H	-0.15522200	-4.22922900	-1.60661600
	H	-0.08904500	-2.47882900	-1.51583800
26	O	-2.12040600	-1.84344600	-1.01001700
	C	-2.29829700	-0.55568000	-1.31600000
	C	-1.76823400	-2.74244100	-2.07812400
	H	-1.15972900	-2.18331400	-2.79356100
	C	-3.04590300	-3.22575500	-2.74340100
	H	-3.61862600	-2.37426300	-3.11381700
	H	-3.65793600	-3.77960000	-2.02761600
	O	-2.20896500	-0.12921200	-2.43579700
	C	-2.72795300	0.28441700	-0.08838100
	C	-4.27445200	0.38692500	-0.16326600
	C	-5.07145300	0.32794400	0.98184700
	C	-6.44911100	0.50752800	0.90310900
	C	-7.05750600	0.74856700	-0.32132600
	C	-6.27126000	0.82724600	-1.46482000
	C	-4.89468400	0.65686200	-1.38671500
	C	-2.15820700	1.71714500	-0.15898000
	C	-2.66706600	2.66112800	0.73924000
	C	-2.11307100	3.92966800	0.83992300
	C	-1.03646700	4.28895500	0.03353100
	C	-0.53531200	3.36777700	-0.87669600
	C	-1.09184000	2.09337300	-0.97584200
	C	-2.24079900	-0.41763300	1.19393700
	C	-1.14462000	0.06645800	1.90122900
	C	-0.63067600	-0.62170600	2.99946000
	C	-1.22837000	-1.79934000	3.42400100

	C	-2.33301200	-2.29278900	2.73214100
	C	-2.82811400	-1.61282700	1.62865000
	H	-4.61802500	0.14984700	1.94888000
	H	-7.04426100	0.46001700	1.80764800
	H	-8.13076700	0.88366600	-0.38278700
	H	-6.72821100	1.03031900	-2.42627400
	H	-4.29329800	0.75044500	-2.28204100
	H	-3.49886300	2.39576500	1.38161800
	H	-2.52468500	4.63859300	1.54912200
	H	-0.59949000	5.27780000	0.11187400
	H	0.30656900	3.62295500	-1.51282400
	H	-0.66652000	1.39848900	-1.68131900
	H	-0.66790600	0.98538300	1.59117500
	H	0.23654800	-0.22348900	3.51348600
	H	-0.83971200	-2.33290600	4.28397600
	H	-2.80891400	-3.21314100	3.05114800
	H	-3.67179300	-2.02019600	1.08520100
	H	-2.81258600	-3.87919600	-3.58638900
	C	-0.94808000	-3.86740000	-1.45773700
	H	-0.87095200	-4.66101800	-2.20642300
	H	-1.50654100	-4.28518700	-0.61157800
	C	0.46365000	-3.44407400	-1.02959400
	C	0.53859000	-2.81909000	0.37080400
	H	-0.36889400	-2.25431900	0.58571700
	C	0.80207500	-3.84439300	1.45274500
	H	0.86994200	-3.35956900	2.42646700
	H	1.73472300	-4.37651700	1.25098000
	H	-0.01806800	-4.56589100	1.48185900
	O	1.65610300	-1.89076400	0.42656000
	C	1.47235300	-0.73077800	-0.19819500
	C	2.68882800	0.20747200	-0.05357000
	C	2.56398700	0.68538700	1.40748700
	C	1.76351600	1.78594700	1.72221200
	O	0.44861000	-0.43947500	-0.75953300
	C	3.15361200	-0.03235400	2.45016800
	C	3.00638300	0.38385400	3.76877200
	C	2.24625400	1.50984400	4.06896100
	C	1.61015700	2.19834900	3.04190000
	C	2.63879400	1.36643100	-1.06598800
	C	2.10728500	1.20591200	-2.34736700
	C	2.17297700	2.23542900	-3.28019100
	C	2.78692300	3.44017300	-2.95971700
	C	3.34592200	3.60104500	-1.69721000
	C	3.27551000	2.57294900	-0.76527800
	C	4.00427600	-0.52970300	-0.34944700
	C	4.04172500	-1.59258900	-1.25053800
	C	5.24857100	-2.18136800	-1.60920700
	C	6.44193400	-1.71233800	-1.07437400
	C	6.41743200	-0.64307300	-0.18621900
	C	5.21002200	-0.05452300	0.16791800
	H	1.25237000	2.32740000	0.93284900
	H	3.73407200	-0.91914300	2.22635400

	H	3.48726800	-0.17556300	4.56267400	
	H	2.13677600	1.83750800	5.09597600	
	H	0.98617600	3.05726200	3.26053700	
	H	1.62351800	0.27988400	-2.62599700	
	H	1.73924300	2.08971200	-4.26253800	
	H	2.83637200	4.24134800	-3.68742300	
	H	3.84228000	4.52751000	-1.43388600	
	H	3.71997600	2.71765600	0.21245700	
	H	3.12202400	-1.97212300	-1.68133800	
	H	5.25341400	-3.00994800	-2.30734100	
	H	7.38286300	-2.17255300	-1.35065800	
	H	7.34176700	-0.26060300	0.23049300	
	H	5.20547900	0.78313100	0.85589400	
	H	0.83789100	-2.72725400	-1.76474900	
	H	1.13604700	-4.30728800	-1.05665000	

References

[1] SCIGRESS 2.5, Fujitsu Ltd

[2] M. J. Frisch, G. W. Trucks, H. B. Schlegel, G. E. Scuseria, M. A. Robb, J. R. Cheeseman, G. Scalmani, V. Barone, B. Mennucci, G. A. Petersson, H. Nakatsuji, M. Caricato, X. Li, H. P. Hratchian, A. F. Izmaylov, J. Bloino, G. Zheng, J. L. Sonnenberg, M. Hada, M. Ehara, K. Toyota, R. Fukuda, J. Hasegawa, M. Ishida, T. Nakajima, Y. Honda, O. Kitao, H. Nakai, T. Vreven, J. A. Montgomery Jr., J. E. Peralta, F. Ogliaro, M. Bearpark, J. J. Heyd, E. Brothers, K. N. Kudin, V. N. Staroverov, R. Kobayashi, J. Normand, K. Raghavachari, A. Rendell, J. C. Burant, S. S. Iyengar, J. Tomasi, M. Cossi, N. Rega, N. J. Millam, M. Klene, J. E. Knox, J. B. Cross, V. Bakken, C. Adamo, J. Jaramillo, R. Gomperts, R. E. Stratmann, O. Yazyev, A. J. Austin, R. Cammi, C. Pomelli, J. W. Ochterski, R. L. Martin, K. Morokuma, V. G. Zakrzewski, G. A. Voth, P. Salvador, J. J. Dannenberg, S. Dapprich, A. D. Daniels, Ö. Farkas, J. B. Foresman, J. V. Ortiz, J. Cioslowski, D. J. Fox, Gaussian 09, revision A.02, Gaussian, Inc., Wallingford CT, 2009.

[3] a) Becke, A. D. Density-functional thermochemistry. III. The role of exact exchange. *J. Chem. Phys.* **1993**, *98*, 5648-5652; b) Lee, C.; Yang, W.; Parr, R. G. Development of the Colle-Salvetti correlation-energy formula into a functional of the electron density. *Phys. Rev. B* **1988**, *37*, 785-789; c) Becke, A. D. Density-functional exchange-energy approximation with correct asymptotic behavior. *Phys. Rev. A*, **1988**, *38*, 3098-3100; d) Perdew, J. P. Density-functional approximation for the correlation energy of the inhomogeneous electron gas. *Phys. Rev. B* **1986**, *33*, 8822-8824.

[4] a) Zhao, Y.; Truhlar, D. G. The M06 suite of density functionals for main group thermochemistry, thermochemical kinetics, noncovalent interactions, excited states, and transition elements: two new functionals and systematic testing of four M06-class functionals and 12 other functionals. *Theor. Chem. Acc*. **2008**, 120, 215-241; b) Jacquemin, D.; Perpète, E. A.; Ciofini, I.; Adamo, C.; Valero, R.; Zhao, Y.; Truhlar, D. G. On the Performances of the M06 Family of Density Functionals for Electronic Excitation Energies. *J. Chem. Theor*. **2010**, *6*, 2071-2085.

[5] a) Grimme, S.; Ehrlich, S.; Goerigk, L. Effect of the damping function in dispersion corrected density functional theory *J. Comp. Chem*. **2011**, *32*, 1456-1465; b) Antony, J.; Sure, R.; Grimme, S. Using dispersion-corrected density functional theory to understand supramolecular binding thermodynamics. *Chem. Commun*. **2015**, *51*, 1764-1774.

[6] Kwit, M.; Rozwadowska, M. D.; Gawroński, J.; Grajewska A. Density Functional Theory Calculations of the Optical Rotation and Electronic Circular Dichroism: The Absolute Configuration of

the Highly Flexible *trans*-Isocytoxazone Revised. *J. Org. Chem*. **2009**, *74*, 8051-8063 and references therein.

[7] Yanai, T.; Tew, D.; Handy, N. A new hybrid exchange-correlation functional using the Coulomb-attenuating method (CAM-B3LYP). *Chem. Phys. Lett*. **2004**, *393*, 51-57.

[8] Harada, N.; Stephens, P. ECD cotton effect approximated by the Gaussian curve and other methods. *Chirality* **2010**, *22*, 229-233.

[9] CrysAlisPro 1.171.40.57a, Rigaku Oxford Diffraction, **2019**.

[10] Sheldrick, G. M. SHELXT – Integrated space-group and crystal-structure determination. *Acta Crystallogr.* **2015**, *A71*, 3-8.

[11] Sheldrick, G. M. Crystal structure refinement with SHELXL. *Acta Crystallogr.* **2015**, *C71*, 3-8.

[12] Parsons, S.; Flack, H. D.; Wagner, T. Use of intensity quotients and differences in absolute structure refinement. *Acta Crystallogr*. **2013**, *B69*, 249-259.

[13] Macrae, C. F.; Sovago, I.; Cottrell, S. J.; Galek, P. T. A.; McCabe, P.; Pidcock, E.; Platings, M.; Shields, G. P.; Stevens, J. S.; Towler, M.; Wood, P. A., Mercury 4.0: from visualization to analysis, design and prediction. *J. Appl. Cryst.* **2020***, 45,* 226-235.

[14] Dolomanov, O. V.; Bourhis, L. J.; Gildea, R. J.; Howard, J. A. K.; Puschmann, H. OLEX2: A Complete Structure Solution, Refinement and Analysis Program. *J. Appl. Cryst*. **2009**, *42*, 339-341.

[15] Spackman, M. A.; Jayatilaka, D. Hirshfeld surface analysis. *CrystEngComm* **2009**, *11* ,19-32.
